# The differential disease regulome

**DOI:** 10.1186/1471-2164-12-353

**Published:** 2011-07-07

**Authors:** Geir K Sandve, Sveinung Gundersen, Halfdan Rydbeck, Ingrid K Glad, Lars Holden, Marit Holden, Knut Liestøl, Trevor Clancy, Finn Drabløs, Egil Ferkingstad, Morten Johansen, Vegard Nygaard, Eivind Tøstesen, Arnoldo Frigessi, Eivind Hovig

**Affiliations:** 1 Department of Informatics, University of Oslo, Blindern, 0316 Oslo, Norway; 2 Department of Tumor Biology, The Norwegian Radium Hospital, Oslo University Hospital, Montebello, 0310 Oslo, Norway; 3 Statistics For Innovation, Norwegian Computing Center, 0314 Oslo, Norway; 4 Centre for Cancer Biomedicine, The Norwegian Radium Hospital, Oslo University Hospital, Montebello, 0310 Oslo, Norway; 5 Department of Mathematics, University of Oslo, Blindern, 0316 Oslo, Norway; 6 Department of Cancer Research and Molecular Medicine, Norwegian University of Science and Technology (NTNU), 7491 Trondheim, Norway; 7 Institute for Medical Informatics, The Norwegian Radium Hospital, Oslo University Hospital, Montebello, 0310 Oslo, Norway; 8 Department of Biostatistics, Institute of Basic Medical Sciences, University of Oslo, Blindern, 0317 Oslo, Norway

## Abstract

**Background:**

Transcription factors in disease-relevant pathways represent potential drug targets, by impacting a distinct set of pathways that may be modulated through gene regulation. The influence of transcription factors is typically studied on a per disease basis, and no current resources provide a global overview of the relations between transcription factors and disease. Furthermore, existing pipelines for related large-scale analysis are tailored for particular sources of input data, and there is a need for generic methodology for integrating complementary sources of genomic information.

**Results:**

We here present a large-scale analysis of multiple diseases versus multiple transcription factors, with a global map of over-and under-representation of 446 transcription factors in 1010 diseases. This map, referred to as the differential disease regulome, provides a first global statistical overview of the complex interrelationships between diseases, genes and controlling elements. The map is visualized using the Google map engine, due to its very large size, and provides a range of detailed information in a dynamic presentation format.

The analysis is achieved through a novel methodology that performs a pairwise, genome-wide comparison on the cartesian product of two distinct sets of annotation tracks, e.g. all combinations of one disease and one TF.

The methodology was also used to extend with maps using alternative data sets related to transcription and disease, as well as data sets related to Gene Ontology classification and histone modifications. We provide a web-based interface that allows users to generate other custom maps, which could be based on precisely specified subsets of transcription factors and diseases, or, in general, on any categorical genome annotation tracks as they are improved or become available.

**Conclusion:**

We have created a first resource that provides a global overview of the complex relations between transcription factors and disease. As the accuracy of the disease regulome depends mainly on the quality of the input data, forthcoming ChIP-seq based binding data for many TFs will provide improved maps. We further believe our approach to genome analysis could allow an advance from the current typical situation of one-time integrative efforts to reproducible and upgradable integrative analysis. The differential disease regulome and its associated methodology is available at http://hyperbrowser.uio.no.

## Background

Knowledge of the molecular biology of the cell is rapidly being gained, providing increasing detail of the cellular signalling systems, as well as better mapping of the various parts of cell regulation. Among the elements that provide dynamics to a signalling system are the transcription factors that bind to sequence specific transcription factor binding sites (TFBSs) along the DNA to regulate gene transcription. Transcription factors represent a potential as drug targets, as ablation of activity of a certain transcription factor may impact a distinct set of genes under its control. One option is therefore to target a transcription factor of a disease-relevant pathway.

However, the challenges associated with the development of drugs for transcription factors have to some extent limited their use, partly due to the structural requirements of inhibition. A recent example of a successful strategy involves inhibition of NOTCH1 in leukemia [1], hinting towards a more rapid development of opportunities for transcription factor inhibition. Other examples targeting transcription factors using small molecule drugs include Stat3 [2] and NFKappaB [3].

The development of a global map of transcription factor over-and under-representation in disease could reveal information relevant for drug target prioritization, as well as serving as a novel knowledge resource.

The relation between a single transcription factor (TF) and a single disease can be probed by evaluating the frequency of binding sites for the TF in regulatory regions of genes assumed to have a role in the disease. One useful strategy in this direction has been to identify differentially expressed genes in a disease state, followed by motif discovery [4]. Binding motif profiles are available for a large number of TFs in motif libraries like Transfac [5] and JASPAR [6], facilitating investigations of multiple TFs. With the advent of technology such as ChIP-chip [7] and ChIP-seq [8], it is now becoming possible to map the binding sites for each TF in unprecedented detail, although such experimental data is still sparse. Therefore, genome-wide predictions of binding sites, albeit noisy, remain valuable sources, and predictions for a large number of TFs are available [9,10], as well as predictions of the target genes for a large number of TFs [11,12] A number of in-depth studies have addressed the functional characterization of TF binding motifs [13-15]. Also, several sources provide information regarding gene-associations for a range of diseases. Phenopedia [16] is a recently developed disease-centered view of the manually curated Human Genome Epidemiology Network (HuGENet) database of genetic associations [17], covering all multifactorial diseases. Another approach is represented by the IntOGen tool, which facilitates integration of data sources relevant for cancer development [18]. Combining such resources with TF binding predictions now permit the development of a global visualization of statistical overrepresentation of regulatory elements across all diseases.

## Results

### Pairwise analysis of cartesian products

In order to advance from the current typical situation of one-time integrative efforts, we have created a generic methodology for integrating complementary sources of genomic information. This is based on an abstract representation of genomic information in the form of genome annotation tracks, allowing very different information types to be treated in a similar manner. Each input source is a set of related genome annotation tracks, e.g. a set of disease tracks or a set of TF tracks. The methodology performs a pairwise, genome-wide comparison on the cartesian product of two distinct sets of annotation tracks, e.g. all combinations of one disease and one TF. The results are provided in the form of tables and interactive heatmaps with the underlying data easily available.

**Figure 1:**
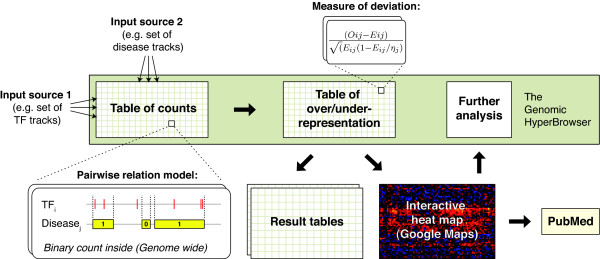
**Schematic model of regulome construction**. Two input sources are selected, e.g a set of TF tracks and a set of disease tracks. For each combination, the pairwise relation model, in this case the number or genes containing TF binding locations, is evaluated and subsequently differentiated against the full matrix of counts. The main output is an interactive heat map of over-/under-representation that for each entry also includes detailed information and links to follow-up analysis. The regulome construction is performed by a web-based system, the Genomic HyperBrowser [33], that allows input data, a pairwise relation model and a measure of deviation to be selected.

The pairwise comparison of annotations is based on a principled mathematical approach to genomic analysis, where the test statistic in principle can be selected from a range of relations between annotation tracks generically represented as tracks of points, segments or functions. Based on the selected test-statistic, normalized values of over-/under-representation are computed and visualized (see Figure 1 for a schematic representation of the strategy and Methods for details).

### Differential disease regulome

**Figure 2:**
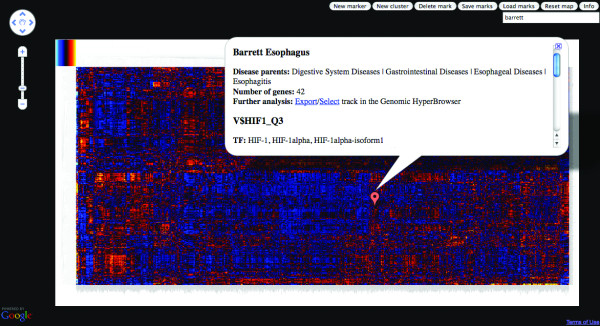
**A screenshot of the differential disease regulome, using Google Maps API http://code.google.com/apis/maps/index.html for visualization and user interface**. Detailed information about each disease-TF combination is available. The selected cell contains information about the overrepresentation of HIF-1alpha in the regulation of the genes associated to Barret Esophagus, a relation previously reported [35].

In order to obtain a global view of diseases and transcription factors, we have used our integrative methodology to perform a large-scale analysis on the combination of the Phenopedia disease-gene catalogue [16] and a recent resource of predicted target genes of TFs [12] (where the datasets of 1010 diseases and 446 TF motifs are constructed as detailed in Methods). The resulting map is referred to as the differential disease transcriptional regulome. For each combination of disease and TF (i.e. the Cartesian product), we find the intersection of TF target genes and disease-associated genes across the genome. Under our main scheme, we investigate deviations from the average across the set of all selected diseases and therefore refer to the regulome as differential. We have developed two different views of the resulting data: a dynamic list of TFs for each disease, and a clustered heat map representation. While the dynamic lists provide direct access to z-score values of over-/under-representation, the heat map representation allows a broader overview of results, providing powerful visual clues of the most deviant associations, and also providing a broad impression of similarities and differences between specific TFs/diseases of interest. As both rows and columns are clustered, diseases with similar profiles of association to TFs will be adjacent (and similarly for TFs against diseases), allowing larger patterns of associations between sets of diseases and TFs to be spotted visually (as well as specific deviances within such clusters). The clustered heat map was visualized using the Google Maps engine, due to the very large number of elements (see Figure 2).

The information to be gained from this approach will obviously depend on the signal-to-noise ratio in the underlying data. To demonstrate the presence of useful signals in the disease regulome, we analyzed a prominent set of TF motifs that were overrepresented in a set of 116 immune related conditions, including immunologic deficiency syndromes, graft versus host disease, asthma, allergies, a number of autoimmune and infectious diseases. The cluster was defined by six NF-κB/Rel-related TF motifs. A smaller cluster based on a subset of 81 of the immune related conditions was also identified, based on six TF motifs, four related to the IRF family, one to Stat1 and to one Cux1 (See Supplemental Results in Additional file 1). There is currently intense ongoing activity in mapping of the regulation of immune diseases, and both experimental data and computational methods are applied. In essence, all of the TFs identified here have previously been implicated in important immune related settings [19-26]. For each cluster, the underlying genes were ranked according to the number of TF bindings found. The highest scoring gene was TNF, regulated by the NF-κB/Rel TFs, according to the clustering. When ranking diseases according to effect size, i.e. the over-/under-representation of TF binding as compared with the expected value for that disease, a set of autoimmune diseases, including arteritis and spondylitis scored highest.

**Figure 3:**
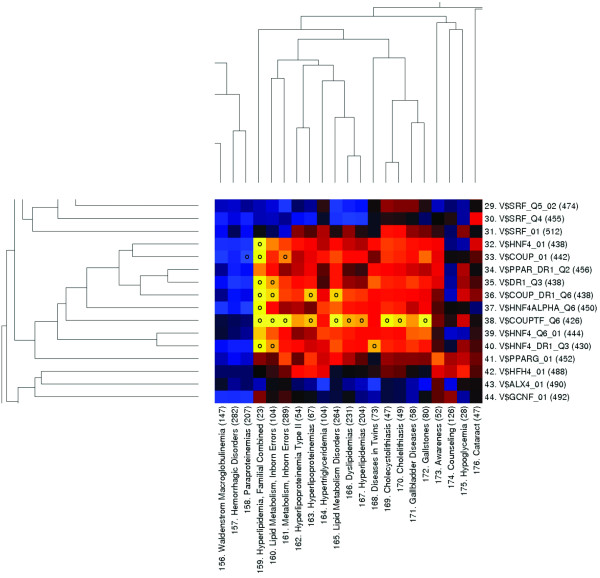
**A small part of the differential disease regulome, showing a cluster of TFs associated with a range of lipid metabolism disorders in addition to gallstone, including dendrograms of the hierarchical clustering of the TFs and diseases**. The disease category "Diseases in Twins" seems to have joined the cluster because of twin studies on lipid metabolism and gallstone. The color of each square indicates the difference between the observed and expected number of gene regions with TF bindings, as denoted by z-scores calculated under the specific null hypothesis. Black denotes no difference, blue to cyan (lowest) denotes under-representation, while red to yellow (highest) represent over-representation of TF binding. Small circles denote significant under-or over-representation, as calculated by the appropriate hypothesis test (see Additional file 1). Different parts of the regulome have been joined together in the figure and dendrograms have been shortened for illustrative purposes.

In addition to the immune-related example, the disease regulome contains a vast number of small and large clusters not described further here, that may represent interesting hypotheses for further investigation (see Figure 3 and Additional file 2 and 3 for listings). One example is that of the ETS1 transcription factor (V$ETS1_B), indicated to be statistically significantly overrepresented in glioblastoma and astrocytoma (indexes: (48, 141 and 142)). Using a decoy strategy to functionally deactivate ETS1, Sahin et al. could demonstrate reduced tumorigenesis of rat C6 glioma cells in an *in vivo *model, underlining the concept value [27].

An additional example investigation of a small cluster is provided in Additional file 1.

### A flexible approach to integrative genomics

There are several reasons why the process of generating a resource like the disease regulome should be as highly automated and as flexible in scope as possible.

First, there is an obvious need to update maps such as the disease regulome, as the underlying data quality constantly improves. Second, there are also presently several alternative sources available both for disease associations and regulation, each having different characteristics, and thus having the potential to provide complementary information. We have generated various alternate versions of the disease regulome based on combinations of different sources of regulation data. For diseases, we have used both literature-mined [28] and experimentally-based cancer disease associations [18], and for TFs, we have used predicted binding sites from UCSC (see Methods). Third, the disease regulome represents only one instance of a whole class of similar maps that may be generated. We have compiled a large collection of resources similar to the disease regulome, addressing other regulatory aspects of genes, including microRNAs, histone modifications and repeat structures in DNA instead of TFs, and with gene regions associated to Gene Ontology terms or even simply chromosome arms or cytobands instead of disease gene regions (Additional file 1). A total of 17 different regulomes can be browsed interactively on our web server http://hyperbrowser.uio.no.

### Analyzing the immune component in alternative regulomes

**Figure 4:**
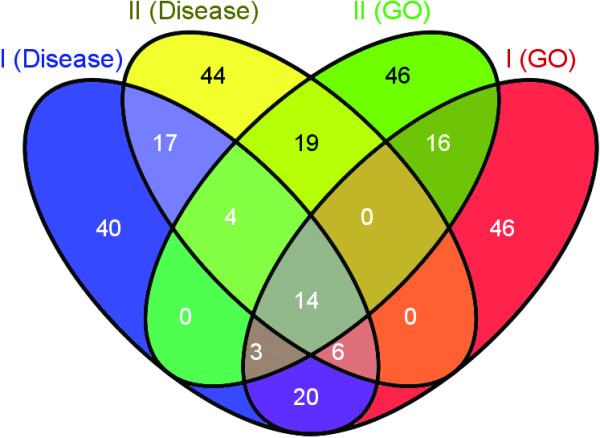
**Venn-diagram of the number of contributing genes that are unique for different combinations of the four immune-related clusters analyzed, one pair of which were based on NF-κB/Rel-related TF motifs (I) and the other pair on IRF/Stat1/Cutl1-related TF motifs (II)**. Note that each cluster pair is comprised of one cluster found in the disease regulome and one found in the TF vs Gene Ontology regulome. Only the 100 genes with highest hit rate were considered (including all genes with the exact same hit rate as the 100th gene). The gene symbols for a select set of combinations are presented in Table 1. The figure was created with the online tool VENNY, by Oliveros, J.C. http://bioinfogp.cnb.csic.es/tools/venny/index.html.

To further characterize the immune component that we observed in the disease regulome, we examined a map of all gene ontology terms versus TFs for potential immune related clusters, and could indeed identify two clusters containing 83 and 79 immune related GO terms, respectively. This cluster was intriguingly defined essentially by the same TF motifs as for the disease list, indicating that this is a strong signal of functional importance (Additional file 1). Based of the top 100 ranked genes from each cluster, we identified the genes with most influence on the differences and similarities across the clusters, thus most central for regulation. Of these 275 unique genes, 14 were found in all the clusters, while an additional 85 genes were present in more than one cluster (see Figure 4 and Table 1). As it is known that there is a level of transcriptional regulation by histone modifications [29], we further analyzed a heatmap of co-localization of TF binding sites and various histone modifications in T-cells, to examine whether we could identify a cluster of TFs similar to those we had identified with the disease and GO regulomes. We identified a cluster that contained 7 of the 9 TF motifs previously found that were part of the dataset used (Additional file 1), with the histone modifications H3K4me3, H3K36me3 and H2AZ being enriched. Interestingly, the H3K4me3 pattern has been identified as important for the binding of the transcription factor and autoimmune inhibitor Aire, which is not present in the TF data set used. It has previously been speculated that IRF family members may form a higher order transcriptional complex with Aire [30]. The H3K36me3 modification has been linked to a number of autoimmune diseases through SNP associations in GWAS studies [31].

**Table 1:** Lists of unique genes contributing to the immune clusters

All clusters		Both NF-κB/Rel clusters		Both IRF/Stat1/Cutl1 clusters		Both disease clusters		Both GO clusters		NF-κB/Rel cluster (GO)	
*Gene*	*Hit rate*	*Gene*	*Hit rate*	*Gene*	*Hit rate*	*Gene*	*Hit rate*	*Gene*	*Hit rate*	*Gene*	*Hit rate*
HLA-A	22.1%	TNF	99.5%	TLR4	12.1%	HLA-B	30.1%	CSF1	19.3%	ICAM1	74.7%
NOD2	20.1%	LTA	58.3%	IL6	9.5%	CARD15	22.3%	TNFSF13	13.3%	IRF6	44.0%
STAT1	12.2%	TGFB1	36.9%	CTLA4	9.4%	VDR	17.5%	FLT3LG	8.0%	CD69	28.9%
CD40	11.6%	CD4	24.8%	CCR5	8.5%	VEGF	4.1%	RELB	6.9%	REL	27.7%
TAP1	10.7%	AKT1	12.1%	TLR3	7.7%	NFKBIZ	3.3%	CD27	6.0%	TNFRSF4	21.1%
NFKBIA	10.7%	LTB	11.6%	HLA-DRB1	7.2%	COL6A1	2.4%	PER1	5.9%	RELA	18.7%
IRF1	10.1%	IL1RN	10.2%	IL2	5.9%	PSORS1	2.1%	PTPN6	5.6%	EDC4	16.9%
CXCL10	10.0%	IL2RA	10.2%	IFNB1	4.2%	CYBA	1.7%	LCK	5.3%	CD58	14.5%
IRF5	7.3%	TNFRSF1B	5.3%	CCL2	4.1%	DDAH2	1.5%	MYD88	5.0%	TNFRSF18	14.1%
PSMB8	6.7%	CD86	5.3%	TNFSF13B	3.5%	CYP27B1	1.4%	PTMA	4.8%	CD83	14.1%
PSMB9	6.2%	ITGAM	5.1%	MX1	3.3%	PLAU	1.4%	IL27	4.7%	TNFRSF9	12.9%
FAS	5.5%	TRADD	5.0%	STAT5A	3.0%	RUNX1	1.3%	B2M	4.3%	IL17C	12.0%
IL7R	2.6%	MIF	3.9%	NOD1	3.0%	RUNX3	1.2%	IRF2	3.1%	CD5	10.8%
IFIH1	1.9%	NFKBIB	3.4%	TLR1	2.9%	PAX2	1.1%	STAT3	3.0%	CD70	9.6%
		CXCL5	3.2%	HLA-DMA	2.3%	CCND1	1.0%	TAPBP	2.9%	DPP4	9.6%
		PTGS1	3.0%	CASP1	2.1%	RXRB	1.0%	BIRC3	2.5%	NFATC2	9.0%
				SOCS1	1.7%	HIF1A	0.8%			TRAF2	9.0%
				TRIM21	1.3%					CREB1	8.0%
				CCL21	1.1%					...	

Lists of genes that are unique for selected combinations of the four immune-related clusters analyzed (see Figure 4). For each gene, the hit rate (proportion of cluster where the gene is relevant to disease regulation) is presented. The NF-κB/Rel cluster in the Gene Ontology regulome is included because of the large hit rates (only the top of the list is shown). Note that TNF is relevant for regulation for nearly all disease-TF pairs in both NF-κB/Rel-related clusters. The full gene listing is included in Additional file 4.

## Discussion

We have here introduced a generic methodology for large-scale integration of genomic information. Based on this methodology we have generated a collection of novel genomic resources in the form of interactive maps that show the relation between various genomic elements. This collection of genomic resources includes the disease regulome, which shows the relation between TFs and diseases in the form of ≈450000 values of over-/under-representation for specific combinations of a TF and a disease.

A common approach to automating large-scale analysis is the construction of a dedicated pipeline for the purpose. Examples of this include a pipeline of for integrating binding site scanning of TF motifs with sets of gene promoters [14], and the GREAT tool for finding enriched annotations in an input set of genomic regions [15]. Although such pipelines may allow e.g. thousands of TF vs gene set combinations to be explored efficiently, the construction of the pipeline itself is a labour-intensive task. The resulting pipeline may be quite ad hoc and limited to certain investigations. Our methodology is based on a generic representation of genomic information in the form of annotation tracks, making it possible to exchange both input sources for a pairwise comparison. The methodology presented here can thus be used in a much wider range of settings than what can be achieved with a typical pipeline. We are able to treat the relation between for example chromosomes and histone modifications in the same manner as the TF-disease relation. This allows us to generate the large number of maps presented in the article, and also allows our methodology to be easily applied to future investigations.

In light of the many possible variations of input data and parameterizations, the question of the robustness of the methodology logically arises. Consider again the immune-related clusters discussed previously. Looking at the hit rate of a gene (the percentage of disease-TF pairs in the clusters where the gene is relevant to disease regulation) we find that only 24 genes have a hit rate exceeding 25%. Still, the combined hit rate of these 24 genes only comprise about a third of the total hit rate, leaving the rest of the contributions scattered over many genes. These clusters therefore seem to be quite robust as regards to noise of single genes. Other clusters may, however, be chiefly caused by one or a small number of genes. Inspecting the gene lists is therefore important when assessing the robustness of a finding.

The results of the clustering algorithm seem to be quite dependent on variations in parameters and input data. When parameters are varied, large and distinct clusters will usually stay robust, but smaller, less well-defined clusters will typically move around, or be split and merged together. Thus, there is no "final" version of the disease regulome. We provide a set of disease regulomes with varying data sources and under two specific null hypotheses (Additional file 1), in addition to a range of other maps based on other combination of data sources. Note that clustering of diseases in the disease regulome heat map should not be thought of in terms of phenotypic similarities, and neither in terms of general similarity at a molecular level. The clustering is exclusively focused on transcriptional regulation of genes connected to the diseases, where high similarity between two diseases could mean that these diseases share several associated genes, or they could be associated to different genes that are still targeted by many of the same TFs.

As genome-wide dataset typically contain a substantial amount of noise, a main consideration when generating regulomes is to provide sufficient signal-to-noise ratio for the global maps to be meaningful. Predicted TF binding sites are currently of very limited accuracy. The predicted TF-gene bindings used in the main disease regulome are more accurate [12], but could still be largely improved by either more precise prediction or by substituting predictions with forthcoming ChIP-seq based results for many of the TFs. For instance, a very interesting regulome would be that of combining TF binding sites actually used in T cells and T cell histone modifications, as opposed to the presently applied less precise prediction scheme. The Phenopedia disease-association database [16] is constantly growing, and could also be complemented by experimentally-based evaluations of disease associations. The TF-relevance for a given disease could use more sophisticated strategies than the generic model of track intersection used here, as could other model assumptions for the expected values be improved from the two used here.

A large-scale, automated effort like the disease regulome clearly represents an inferior handling of a specific TF-disease relation compared to what can be achieved by a separate, manual investigation of the same relation. We don't claim that each value in the disease regulome represents a satisfactory conclusion regarding the relation between a specific TF and disease. What we claim is merely that our approach is able to capture a part of the underlying reality, and that multiplied with the large number of combinations studied, a map like the disease regulome constitutes a substantial resource of genomic information. We consider the disease regulome as mainly a hypothesis-generating tool to be used as the starting point for a number of future investigations.

## Conclusion

We believe the disease regulome may prove immensely useful in early phases of research projects, as a resource for obtaining an initial overview of the regulation of disease and for supporting the formation of hypotheses to be studied further by computational or experimental methods. Moreover, we believe that the disease regulome will provide important pathway information for diseases, thereby serving as an aid to target identification and drug development.

## Methods

### Data set of transcription factor binding sites

For the main disease regulome, we used binding predictions for a set of 446 transcription factor motifs (PWMs), each with a list of the top 1000 predicted target genes [12]. These predictions are based on machine learning from 29 relevant features, including conservation, CpG island content, DNase I hypersensitivity and histone modifications, in addition to the PWM score. Other regulomes were calculated on the basis of a track of transcription factor binding site (TFBS) predictions for 258 PWMs from the UCSC genome browser called "TFBS conserved". The track was generated by Matt Weirauch and Brian Raney at UCSC and last updated July 17, 2007 http://genome.ucsc.edu/cgi-bin/hgTrackUi?hgsid=153908909&c=chrX&g=tfbsConsSites. Each PWM represent the binding specificity of one or a small set of closely related transcription factors. For simplicity, we mostly refer to the regulatory categories as TFs, instead of TF motifs, or PWM.

### Data set of disease gene lists

Disease-associated genes were mapped to genomic coordinates, resulting in a set of genome regions for each disease. For the main regulome we used the complete list of all disease-gene association from Phenopedia [16], which is based on years of manually curation of reported associations in literature, collected the Human Genome Epidemiology Network (HuGENet) database. Only diseases with more than 20 gene associations were included.

An alternative data source for disease-associated genes sets was also used, based on citations and co-citations of disease and gene terms in the literature, as collected by PubGene [28]. Let N be the number of documents in this collection and let m be the number of documents that mention disease term d and n the number of documents that mention gene term g. Under the null model that there is no association between the disease term d and gene term g, the number of documents that mention both d and g follows a hypergeometric distribution with parameters N, m and n. We then define the gene list for a given disease to be all genes for which we obtain a Bonferroni-corrected p-value less than 0.02. Only diseases with more than 300 citations in literature were included. Both sources of gene lists use the set of diseases as defined by MeSH (Medical Subject Headings).

### Intersecting transcription factors and diseases

For each combination of TF and disease, we counted, over the whole genome, the number of segments (disease genes) with at least one point (TF binding prediction) falling inside them. For the main TF data set, the points refer to the TSS of the genes with predicted associations. As this dataset used gene coordinates according to Refseq, while the disease datasets were encoded using Ensembl, we extended the gene regions by 150 bp upstream to provide support for TSS inconsistencies between the two standards. To reduce noise, only diseases with a gene list of at least 20 genes were included. For the UCSC data set, each point refers to a predicted TFBS. As TFBSs acting on a gene are often close to but outside a gene, we extended the gene regions to include flanks, set to 5 kb in each direction. In this case we only included diseases with a gene list of at least 10 genes. The choice of 5 kb in each direction is somewhat arbitrary, simply assuming that a substantial amount of relevant binding sites would be included. Other schemes are easily incorporated in the approach.

To conclude whether a TF is associated to a disease, we used hypothesis testing to investigate whether the number of disease-associated gene regions containing locations of binding (TSS or TFBS) of the given TF, were greater or less than expected by chance. Two different tests, providing complementary information, have been implemented. In both tests we assume under the null hypothesis that gene regions are fixed, that the number of binding locations for each TF is fixed, and that their positions are randomly selected among the positions containing binding locations for any TF.

We have calculated z-scores based on the deviation from expected values under two specific null hypotheses. Several different model assumptions may be reasonable. In our main scheme, the null-hypothesis is that the proportion of binding sites associated to a given TF is the same within the regions of a given disease as it is across all diseases.

The first test further assumes in the null hypothesis that binding locations of a given TF falls uniformly among the set of positions containing binding locations for any TF.

Conversely, the second test modifies this by assuming in the null hypothesis that binding locations of the given TF falls inside gene regions of the given disease proportionally to how often the binding locations of this TF on average falls inside gene region sets across all diseases. The first test is based on the hypergeometric distribution, while the second test is based on the binomial distribution. Details and formulas for both tests are provided in Additional file 1.

### Clustering

Groups of similar disease/TF tracks are found by separately clustering rows and columns of a matrix of z-values (see above). Hierarchical clustering has been used, as this provides information on several levels, both closely related diseases and large groups of diseases with a certain amount of similarity. As similarity measure between individual objects (diseases/TFs) we used the Euclidean distance, and for distance between clusters we used the average (Euclidean) distance between all pairs of objects. Further details on the clustering are given in Additional file 1.

### Data sets of complementary regulomes

A range of regulomes have been generated based on different combinations of input data. These regulomes make use of gene lists associated with GO terms, as well as histone modification data. The gene lists for GO terms are generated based on literature co-occurrence in the same way as the gene lists for diseases. The histone modification dataset is based on raw tag hit data from ChIP-seq experiments on human T-Cells [29]. These were preprocessed using the NPS (Nucleosome Positioning from Sequencing) software [32], using peak detection, leading to nucleosome positioning information as short segments, treated as points. When looking at the regulatory effects of histone modifications, we counted the number of points (defined as the middle of the DNA strand eclipsing modified nucleosomes) in the 2 kb up-and downstream region surrounding the transcription start site of each gene. A complete overview of complementary regulomes is given in Additional file 1.

### Software

The methodology is implemented within a software system that supports interactive, real time, large-scale genomic analyses [33] (further details given in Additional file 1). The software system allows large and fully customizable analyses to be performed interactively. The system is open source, runs integrated with the Galaxy web server [34], and is available on the web at http://hyperbrowser.uio.no.

## List of abbreviations used

bp: base pair; GO: Gene Ontology; kb: kilobases; PWM: position weight matrix; TF: transcription factor; TFBS: transcription factor binding sites; TSS: Transcription start site.

## Authors' contributions

GKS and SG contributed equally to this work. GKS, SG and EH conceived the regulome approach. GKS, AF and EH conceived the general HyperBrowser (HB) approach. HR inspired the clustered heatmap presentation. SG and MJ implemented the regulome-specific functionality. GKS, SG and MJ implemented the general HB system. LH and MH developed the regulome-specific statistics. EF, IKG, LH, MH, KL and AF developed the general statistical HB approach. TC, VN and ET participated in the general HB development. FD and EH contributed to disease regulome development and analysis. GKS and EH wrote the main parts of the manuscript. EH supervised the project. All authors read and approved the final manuscript.

## Supplementary Material

Additional file 1 **Supplemental material for "The differential disease regulome"**. Miscellaneous supplemental material: details on the Genomic HyperBrowser; overview of generated regulomes; details on immunology example; additional example from a disease regulome variant; statistics overview and supplemental figures. Click here for file

Additional file 2 **TF-disease clusters**. A listing of 57 manually indentified TF-disease clusters in the differential disease regulome. Click here for file

Additional file 3 **TF-GO clusters**. A listing of 105 manually indentified TF-GO clusters in the Gene Ontology regulome. Click here for file

Additional file 4 **Gene listings for immunology example**. Listings of all the genes of the immunology clusters in the differential disease regulome and the Gene Ontology regulome, sorted on their hit rate. The genes indicated in the different sections of the Venn diagram in Figure 4 are also detailed here. Click here for file
