# Supplementary material for: The differential disease regulome
Source: BMC Genomics. 2011 Jul 7;12:353. doi: 10.1186/1471-2164-12-353 (PMC3160420; doi:10.1186/1471-2164-12-353)
Supplement: Additional file 1 — Supplemental material for "The differential disease regulome". Miscellaneous supplemental material: details on the Genomic HyperBrowser; overview of generated regulomes; details on immunology example; additional example from a disease regulome variant; statistics overview and supplemental figures. [file 1471-2164-12-353-S1.PDF]

Supplemental material for "The differential disease regulome"

Geir Kjetil Sandve et al.

April 15, 2011

Supplemental Results

The Genomic HyperBrowser

The disease regulome is studied in a general system for statistical analysis of sequence-level genomic information (1). The system considers a collections of generic biological questions that precisely capture a multitude of investigation needs, and query pairwise relations between tracks, abstractly represented as mathematical objects, along the genome. Significance testing with biologically motivated construction of null hypotheses is used to answer questions at the appropriate scale. As an implementation of our approach, we provide The Genomic HyperBrowser, a web-based software system with the power to handle many types of biological inquiries on the fly. The software is open source and widely extensible. We have constructed an abstract representation of generic genomic elements by means of mathematical objects. Hypotheses of interest are translated into mathematical relations, combining formal significance testing with geometric and analytical tools. The biologist can explore advanced and problem-specific null hypotheses, hierarchically preserving various degrees of track information, strengthening power and reducing false discoveries. Comparison or analysis of (sets of) pair-wise tracks are considered and confounding tracks can be incorporated when necessary. We offer an open-ended system, foreseeing a large body of solutions to be added by the community.

Overview of generated regulomes

The Genomic HyperBrowser allows large-scale regulatory analyses to be performed with ease. We have generated several regulomes, where the main

result is the Differential disease regulome. The regulome shows how a range of TFs with TF-gene predictions (2) are over/under-represented in a range of diseases with gene associations retrieved from Phenopedia (3). We have also generated regulomes for the same TF predictions, but with diseases-gene associations calculated from the PubGene database of literature co-citations (4). Using the same disease-gene associations, we have created regulomes on a subset of diseases defined as neoplasms. Continuing in the similar vein, we have created regulomes that makes use of experimentally-based cancer gene associations, retrieved from the IntOGen database (5). We provide two variants. First, for genes identified as having altered copy number variation in cancer cells, and second, for genes transcriptionally altered. In addition to the disease regulomes in different versions, we have generated maps showing combinations of TFs and gene lists associated with Gene ontology terms, also based on the PubGene database of literature co-citations.

We have also generated a similar set of regulomes based on transcription factor binding site predictions (the "TFBS Conserved" track from UCSC Genome Browser). First we have created a regulome based on the Phenopedia disease-gene associations (3). Second, a regulome based on diseases-gene associations calculated from the PubGene database (4).

Furthermore, we have generated maps that are not based on gene lists at all, but that instead query the relation between TF binding sites and DNA regions wound around nucleosomes with particular histone modifications in T-cells. We also present a map of the relation between histone modifications in T-Cells and the Gene ontology terms of the genes where such modifitacion are over/under-expressed around the TSS. Looking at other regulatory elements apart from TFs and histone modifications, we present a map of MiRNAs versus diseases and a map of repeating elements versus disases (both based on PubGene data (4)). These two maps are in need of updates. Finally, we have generated maps of how different TFs (according to the TFBS predictions from UCSC) preferentially regulate genes of particular chromosome arms, as well as maps of how different histone modifications (in T-cells) distribute across chromosome arms.

All regulomes are available for browsing from the front page of the Genomic HyperBrowser. All maps is normalized differentially in both directions, and some exist in a version normalized differentially for only rows (usually TFs).

Details on immunology example

The regulatory input data for the main disease regulome are tracks of predicted target genes for 446 Transfac position weight matrices (PWMs). The regulomes comparing PWMs to Gene ontologies and histone modifications use a TF data set with 258 PWMs. Each PWM represent the binding specificity of one or a small set of closely related transcription factors. Comparing the associated clusters in the three maps, we find that of the 13 PWMs present in the clusters that also is a part of both datasets, 6 is found in the clusters of all maps. Several of the other PWMs are also related to Nf-KabbaB and IRFs, without being the exact same ones in all clusters (see figure S1).

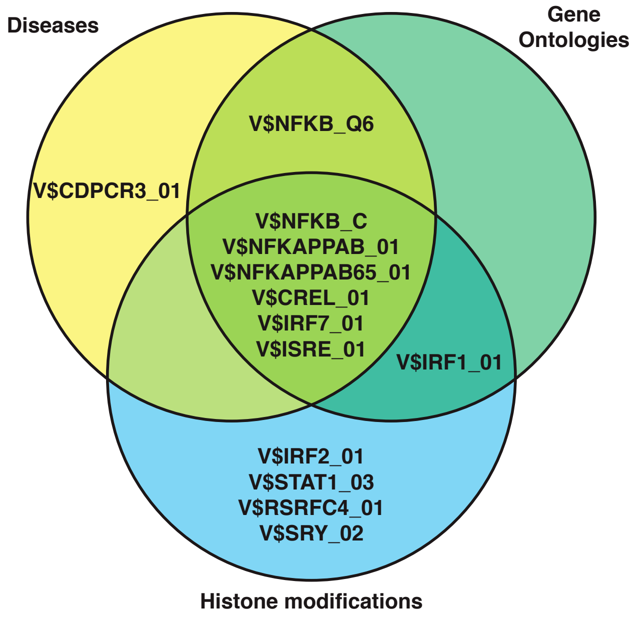

Figure S1: Venn diagram of the PWMs of the immune-related clusters analyzed. The clusters were located in the main disease regulome, the TF vs Gene Ontology regulome and the TF vs histone modifications regulome.

Additional example from a disease regulome variant

There are in the regulomes a range of small clusters that may provide biological insight. From the regulome based on disease-gene accosiations from PubGene (4) and TFBS predictions from UCSC, we e.g. identified a set of TFs associated with genomic and chromosomal instability and DNA repair syndromes, in particular Cockayne syndrome, xeroderma pigmentosum, ataxia telangiectasia and Bloom syndrome, but also Fanconi anemia. These diseases were significantly associated with the TFs Nrf-2, c-Ets-1 and Elk-1. As a control we used a curated set of genes for DNA repair (6, 7), and queried whether the top ranking TFBSs identified from the disease analysis were overrepresented also in the curated repair gene list. The most overrepresented transcription factor was NRF2, which is well known as a key modulator of oxidative stress response, and a range of other features related to maintenance of genomic integrity (8). Apparently, a part of this role may be related to regulating DNA repair responses, as a number of the repair genes are significantly enriched in the NRF2 motif.

Supplemental Methods

Statistics overview

The inputs to the disease regulome are a set of genes for each disease and a set of binding sites for each transcription factor (TF). In the Genomic HyperBrowser, such information is represented as genome annotation tracks.

A genome annotation track is a collection of objects of a specific genomic feature, such as genes, with base pair (bp) specific locations from the start of chromosome 1 to the end of chromosome Y. A track representing genes contains positional information that can be reduced to segments (intervals of bps) along the genome. A track of binding sites can be reduced to points (single bps) on the genome. Five genomic types are defined in the Genomic HyperBrowser: Unmarked points (UP), marked points (MP), unmarked segments (US), marked segments (MS) and functions (F). The difference between the marked and unmarked track types is that the first has an associated value of interest, while the latter only provides positional information. Inputs to the disease regulome are thus represented as tracks of type UP (TF binding sites) and type US (disease gene regions).

For each disease j, we define a track denoted US_j consisting of a list of disjoint segments, corresponding to regions for genes associated with disease j. The binding sites for each transcription factor i are represented as a track

UP_i of unmarked point locations along the genome. The set of all binding sites regardless of TF is called UP (without an index), and similarly gene regions across diseases is called US. For each combination of TF and disease, we count, over the whole genome, the number of segments (disease genes) with at least one point (TFBS) falling inside them.

Let O_{ij} be the number of points of track UP_i inside the segments of track US_j, $i = 1, \ldots, n$, $j = 1, \ldots, m$. We can make a matrix O of the O_{ij} values as shown in the following table:

Track	US_1	US_2	$\cdots$	US_m	Sum		Track UP
UP_1	O_{11}	O_{12}	$\cdots$	O_{1m}	M_1		N_{UP_1}
UP_2	O_{21}	O_{22}	$\cdots$	O_{2m}	M_2		N_{UP_2}
$\vdots$	$\vdots$	$\vdots$	$\ddots$	$\vdots$	$\vdots$		$\vdots$
UP_n	O_{n1}	O_{n2}	$\cdots$	O_{nm}	M_n		N_{UP_n}
Sum	N_1	N_2	$\cdots$	N_m	N		N_{UP}

where $M_i = \sum_{j=1}^{m} O_{ij}$, $N_j = \sum_{i=1}^{n} O_{ij}$, and $N = \sum_{j=1}^{m} N_j = \sum_{i=1}^{n} M_i = \sum_{j=1}^{m} \sum_{i=1}^{n} O_{ij}$, $i = 1, \ldots, n$, $j = 1, \ldots, m$. The last column contains counts for the track UP, where N_{UP_i} is the number of points in UP_i, $i = 1, \ldots, n$, and $N_{UP} \leq \sum_{i=1}^{n} N_{UP_i}$ is the number of points in UP. Note that $N_{UP} = \sum_{i=1}^{n} N_{UP_i}$ only if there is no common point in the UP_i tracks. Also, note that if we assume that all UP points are in segments of some US_j, then $N \geq N_{UP}$, since points that are inside segments of several US_j are counted several times in N.

For concluding whether a TF represented by the track UP_i is associated with a disease represented by the track US_j, we might check whether there are more points of track UP_i inside the segments of track US_j than expected by chance. Note that "more" can be substituted with "less" or "more or less". Below, we only consider the case "more". The two other cases are similar.

Whether there is an association between a TF and a disease is investigated using hypothesis testing. Two different tests, providing complementary information, have been implemented in the Genomic HyperBrowser. We have analysed the disease regulome according to either, though we consider results using hypothesis test 2 as the main result. In both tests we assume under the null hypothesis that segments are fixed, that the number of points in UP_i is fixed, $i = 1, \ldots, n$, and that their positions are randomly selected with equal probabilities among the positions in UP. Under these assumptions define $E_{ij} = E(O_{ij})$. Also define $\eta_j = \sum_{i=1}^{n} E_{ij}$, $\mu_i = \sum_{j=1}^{m} E_{ij}$ and $\nu = \sum_{i=1}^{n} \mu_i$. Note that $\nu = \sum_{i=1}^{n} \mu_i = \sum_{j=1}^{m} \eta_j$.

Hypothesis test 1

We test

$$\mathrm{H}_{01} : \frac{E_{ij}}{\eta_j} = \frac{N_{UP_i}}{N_{UP}}$$

against the alternative

$$\mathrm{H}_{A1} : \frac{E_{ij}}{\eta_j} > \frac{N_{UP_i}}{N_{UP}}.$$

Note that the result of the test is independent of the other US tracks, i.e. the results for a specific disease are independent of other diseases. What is tested is whether a particular UP_i track has a higher proportion of points inside the US_j segments than the UP track. No UP points are counted several times.

Under the null hypothesis we assume that the expected proportion of points in the US_j segments that are UP_i points is the same as the proportion of points in UP that are UP_i points, i.e. $\frac{E_{ij}}{\eta_j} = \frac{N_{UP_i}}{N_{UP}}$. We also assume that the positions of the N_{UP_i} points in UP_i are randomly selected with equal probabilities from the N_{UP} positions in UP. Assuming that the points are selected without replacement, the statistic we use is $\frac{O_{ij}}{N_j}$. Under the null hypothesis this leads for O_{ij} to the hypergeometric distribution with parameters N_{UP}, η_j and N_{UP_i} as follows: i) We have an urn with N_{UP} red and white balls, where the η_j red balls represent positions in UP within segments of US_j, while the $N_{UP} - \eta_j$ white balls represent positions outside segments of US_j; ii) We draw N_{UP_i} balls/positions without replacement from the urn; iii) O_{ij}, the number of these N_{UP_i} positions that are within segments of US_j, i.e. the number of red balls drawn, follows a hypergeometric distribution. We approximate η_j by N_j.

Hypothesis test 2

We test

$$\mathrm{H}_{02} : \frac{E_{ij}}{\eta_j} = \frac{\mu_i}{\nu}$$

against the alternative

$$\mathrm{H}_{A2} : \frac{E_{ij}}{\eta_j} > \frac{\mu_i}{\nu}.$$

Note that the result of the test depends on all $n + m$ tracks. This means that the results for a specific disease are dependent on the set of diseases used in the analysis, leading to results that are specific for a given set of diseases, chosen in the analysis. If e.g. only cancer diseases are used, then

results will show how a specific cancer differs from general cancers. Define the expected number of points and UP_i points in the average US track to be $\frac{1}{m}\sum_{j=1}^{m}\eta_j = \frac{\nu}{m}$ and $\frac{1}{m}\sum_{j=1}^{m}E_{ij} = \frac{\mu_i}{m}$, respectively. What is tested in hypothesis test 2 is whether there is a higher expected proportion of UP_i points inside the US_j segments than inside the segments of the average US track since $\frac{E_{ij}}{\eta_j} = \frac{\mu_i/m}{\nu/m}$. If the same segment is part of several of the US tracks then the points in these segments are counted several times. Therefore, the tests for the US_j track depend strongly on segments with many points that are part of many of the other US_k tracks, $k = 1, \ldots, m$.

Under the null hypothesis we assume that the expected proportion of points in the US_j segments that are UP_i points is the same as the proportion of points in the average US track that are UP_i points, i.e. $\frac{E_{ij}}{\eta_j} = \frac{\mu_i}{\nu}$. We also assume that the positions of the N_{UP_i} points in UP_i are randomly selected with equal probabilities from the N_{UP} positions in UP. In this case we cannot choose a hypergeomtric distribution as positions might have been counted several times in ν and μ_i, not only once as in N_{UP} and N_{UP_i}. Instead of assuming that the points are selected without replacement, we assume that the points are selected with replacement. Then O_{ij} follows a Binomial distribution with parameters η_j and $\frac{\mu_i}{\nu}$. We approximate η_j by N_j and μ_i by M_i. Then $\nu = \sum_{i=1}^{n}\mu_i$ will be approximated by N.

Clustering

It is interesting to find groups of US tracks or groups of UP tracks that are similar. This is achieved by clustering a matrix S that for each pair of tracks (UP_i, US_j) expresses how different the observed value O_{ij} is from the expected value E_{ij}. A natural score is the statistics used in the hypothesis tests, but these are not necessarily comparable, and needs to be normalized before clustering the matrix. One possibility is to use the z-score $\frac{(Oij-Eij)}{\sqrt{(E_{ij}(1-E_{ij}/\eta_j)}}$, which is approximately standard normally distributed, as a score in the matrix to be clustered. For the hypothesis test based on the Binomial distribution, the reasoning behind this is that $O_{ij} \sim Binomial(\eta_j, \frac{\mu_i}{\nu})$ is approximated by $O_{ij} \sim Normal(E_{ij}, E_{ij}(1-\frac{E_{ij}}{\eta_j}))$, where $E_{ij} = \eta_j \cdot \frac{\mu_i}{\nu}$ (this is a good approximation if $5 < E_{ij} < \eta_j - 5$). This means that $\frac{O_{ij}-E_{ij}}{\sqrt{(E_{ij}(1-\frac{E_{ij}}{\eta_j}))}} \sim Normal(0,1)$. For the hypothesis test based on the hypergeometric distribution we first approximate the hypergeometric distribution with a Binomial distribution: $O_{ij} \sim Binomial(\eta_j, \frac{N_{UP_i}}{N_{UP}})$. Otherwise the reasoning is as for the case where the hypothesis test is based on the Bino-

mial distribution, except that $E_{ij} = \eta_j \cdot \frac{N_{UP_i}}{N_{UP}}$. The approximation of the Binomial to the Normal becomes better if O_{ij} is substituted by $O_{ij} - 0.5$ (continuity correction). As before we approximate η_j by N_j, μ_i by M_i, and ν by N.

Z-scores according to a Normal approximation to the Binomial distribution were used both in the results table and in the clustered heatmap, regardless of how good the Normal approximation was. Additionally, significance was marked by a small "o" in the heatmap, computed either from the Normal approximation or directly from the Binomial distribution as appropriate.

References

1. Sandve, G.K., Gundersen, S., Rydbeck, H., Glad, I., Holden, L., Holden, M., Liestol, K., Clancy, T., Ferkingstad, E., Johansen, M. et al. (2010) The Genomic HyperBrowser: inferential genomics at the sequence level. *Genome Biology*, **11**, R121.

2. Ernst J, Plasterer H, Simon I, Bar-Joseph Z (2010) Integrating multiple evidence sources to predict transcription factor binding in the human genome. *Genome Res.*, **20**, 526.

3. Yu W, Clyne M, Khoury M, Gwinn M (2010) Phenopedia and Genopedia: disease-centered and gene-centered views of the evolving knowledge of human genetic associations. *Bioinformatics* **26**, 145.

4. Jenssen T, Lægreid A, Komorowski J, Hovig E (2001) A literature network of human genes for high-throughput analysis of gene expression. *Nat. Genet.* **28**, 21–28.

5. Gundem G, *et al.* (2010) IntOGen: integration and data mining of multidimensional oncogenomic data. *Nat. Methods* **7**, 92–93.

6. Nakken S, Alseth I, Rognes T (2007) Computational prediction of the effects of non-synonymous single nucleotide polymorphisms in human DNA repair genes. *Neuroscience* **145**, 1273–1279.

7. Wood R, Mitchell M, Lindahl T (2005) Human DNA repair genes, 2005. *Mutat. Res., Fundam. Mol. Mech. Mutagen.* **577**, 275–283.

8. Kensler T, Wakabayashi N, Biswal S (2007) Cell survival responses to environmental stresses via the Keap1-Nrf2-ARE pathway. *Annu. Rev. Pharmacol. Toxicol.*, **47**, 89–116
