# Supplementary material for: The differential disease regulome
Source: BMC Genomics. 2011 Jul 7;12:353. doi: 10.1186/1471-2164-12-353 (PMC3160420; doi:10.1186/1471-2164-12-353)
Supplement: Additional file 2 — TF-disease clusters. A listing of 57 manually indentified TF-disease clusters in the differential disease regulome. [file 1471-2164-12-353-S2.PDF]

Cluster for columns 11 to 14, rows 47 to 50

Phenopedia diseases

Connective Tissue Diseases | Lupus Erythematosus, Systemic | Autoimmune Diseases | Immune System Diseases

TFs

V$TEL2_Q6 | V$ETS1_B | V$PAX_Q6 | V$GATA1_03

Information

All related TFs:
(List of all TFs that are related to any of the PWMs)
GATA-1, GATA-1A, Pax-1, Pax-2, Pax-2a, Pax-3, Pax-4a, Pax-4c, Pax-5, Pax-6, Pax-8, Pax6-1, Tel-2a, Tel-2b, Tel-2c, c-Ets-1

Ranked gene list:
(All genes of the selected Phenopedia diseases with hits of any of the selected TFs, ranked according to the total number of TF hits)
SYNGAP1, SH2B3, PTPN6, ZFP36, TNIP1, RING1, PBX2, NFKBIE, IRF1, HSD17B8, CUTA, COL11A2, CDKN1A, C6orf47, BAK1, AGER, PSTPIP1, PRF1, NLRP3, IMPDH2, IL11RA, IKBKE, TRADD, TNFRSF6B, TNFRSF1B, TLR9, TCF7, SLC39A7, SLC23A1, RXRB, RNF5, PSMB9, PSMB8, NOTCH4, NCR3, MSH5, LYN, LY6G5B, LTB, LST1, KIAA1949, IL4R, HMHA1, GPSM3, FOXJ1, DDAH2, CYBA, CTLA4, CSNK2B, CLIC1, CD19, CCR7, C6orf26, BRD2, BAT4, AGPAT1, UCP3, SLC9A3R1, SELPLG, PLCG1, IL2RB, APOM, VPS52, VDR, TUBB, TRIM39, TRIM26, TREX1, TNFSF13B, TNFSF13, TNFSF12, TNFRSF4, TNFRSF1A, TGFBR2, TAPBP, TAP1, SOCS1, S100A5, RPS18, RGL2, PRRT1, PRDM1, PPP1R11, PPP1R10, PHRF1, PHF1, PFDN6, NRM, NFKB1, NDUFS2, MRPS18B, MMP9, MDC1, MAN2A1, LY9, LY6G5C, LAG3, KIAA1542, ITPR3, ITGB2, ITGA2B, IRF7, IL16, IL10RA, IKZF1, IER3, IDDM12, HSPA1L, HCLS1, GPR132, GIMAP5, FYN, FLOT1, FLI1, FKBPL, FEN1, FAS, EIF2B4, EHMT2, DNMT1, DHX16, DBP, CD4, C6orf48, C1QC, C1QB, C1QA, BTLA, BAT3, B3GALT4, ATF6B, ZAP70, VIPR1, UNC84B, TRAF6, TRAF5, TNFRSF14, TANK, SMAD3, SH2D2A, SELS, S100A4, RHOG, RBPJ, RAC2, PTPN11, PLCL2, PALB2, NOS1, MTHFD1, MSX2, MEFV, MAP3K7, MAP3K1, LIF, LCK, IRAK4, IL7R, IL6R, ICAM3, HSPD1, HAVCR2, FANK1, DUSP2, DUSP1, DCTN5, CDK6, CD79A, CD226, CD14, CARD8, BRAF, ARRB2, ANTXR2, AKT1, ADORA2A, WFS1, TXN, TNFRSF25, TG, TFRC, TBP, TAC3, SOCS3, SMAD4, SHMT2, S100A6, S100A3, RTEL1, RPL5, RHOC, RASGRP1, PTPRA, PTGER4, PTGER3, PSMB1, PRKD2, PRKCB1, PRKCA, PRDX5, POLG, PLD5, PLCD1, PLCB3, PIK3R1, PCDH12, PAX5, NTRK1, NRP1, NR1H3, NBN, MIA3, MBP, LTC4S, KNG1, KIF1B, KCNQ1, IRF9, IL27, ICOSLG, HSD11B2, HDAC7, GIPR, GFI1, GCA, GBA, EGR1, ECE2, DCLRE1C, CYP27B1, CYFIP2, CXXC5, CXCR4, CRHR1, COL17A1, CNDP2, CHRNE, CFLAR, CDK4, CD79B, CD72, CD55, CASP8, C1RL, C10orf2, BSCL2, BCL2L1, BAX, ATP1A1, ARRB1, AKR1B1, XRCC5, WNT2B, TUBGCP6, TSLP, TRAF3, TNPO3, TNFRSF18, TNFRSF13C, TNFRSF10D, TAL1, SYK, STIP1, SLC3A2, SIGIRR, SFTPC, SCNN1A, RUNX3, RIPK3, PTPN1, PRMT6, PLCB2, PLA2G4B, PIM1, PIK3CG, NPAS2, NOTCH1, NOD1, NCF4, MYC, MUC4, MSH2, MMP14, MFNG, MAP3K5, MAN1B1, LY96, LSP1, JUND, JAK3, ITGB7, ITGB1, IL17C, HSPA14, HSP90AB1, HSD17B1, H2AFX, GSTO1, GPX4, GATA3, G6PD, FOS, FGR, ERCC6, ERCC4, ERCC1, DUOX2, CTTN, CSF3R, CREBBP, COL7A1, COL2A1, CLC, CEBPE, CDKN1B, CD27, CCL27, CASP8AP2, CASP6, CARD4, C3orf18, BGLAP, BCL7C, BCL6, BCL2A1, ATG9B, APOA2, AKT2, ADAM8

Corresponding total number of TF hits:
(For each gene listed above, the total number of TF hits for any of the selected TFs, multiplied by the

number of selected Phenopedia diseases containing that gene)
16, 16, 16, 12, 12, 12, 12, 12, 12, 12, 12, 12, 12, 12, 12, 12, 9, 9, 9, 9, 9, 9, 8, 8, 8, 8, 8, 8, 8, 8, 8, 8, 8,
8, 8, 8, 8, 8, 8, 8, 8, 8, 8, 8, 8, 8, 8, 8, 8, 8, 8, 8, 8, 8, 6, 6, 6, 6, 6, 6, 4, 4, 4, 4, 4, 4, 4, 4, 4, 4, 4, 4, 4,
4, 4, 4, 4, 4, 4, 4, 4, 4, 4, 4, 4, 4, 4, 4, 4, 4, 4, 4, 4, 4, 4, 4, 4, 4, 4, 4, 4, 4, 4, 4, 4, 4, 4, 4, 4, 4, 4, 4, 4,
4, 4, 4, 4, 4, 4, 4, 4, 4, 4, 4, 4, 4, 3, 3, 3, 3, 3, 3, 3, 3, 3, 3, 3, 3, 3, 3, 3, 3, 3, 3, 3, 3, 3, 3, 3, 3, 3, 3, 3,
3, 3, 3, 3, 3, 3, 3, 3, 3, 3, 3, 3, 3, 3, 3, 3, 2, 2, 2, 2, 2, 2, 2, 2, 2, 2, 2, 2, 2, 2, 2, 2, 2, 2, 2, 2, 2, 2, 2, 2,
2, 2, 2, 2, 2, 2, 2, 2, 2, 2, 2, 2, 2, 2, 2, 2, 2, 2, 2, 2, 2, 2, 2, 2, 2, 2, 2, 2, 2, 2, 2, 2, 2, 2, 2, 2, 2, 2, 2, 2,
2, 2, 2, 2, 2, 2, 2, 1, 1, 1, 1, 1, 1, 1, 1, 1, 1, 1, 1, 1, 1, 1, 1, 1, 1, 1, 1, 1, 1, 1, 1, 1, 1, 1, 1, 1, 1, 1, 1, 1,
1, 1, 1, 1, 1, 1, 1, 1, 1, 1, 1, 1, 1, 1, 1, 1, 1, 1, 1, 1, 1, 1, 1, 1, 1, 1, 1, 1, 1, 1, 1, 1, 1, 1, 1, 1, 1, 1, 1, 1, 1

Corresponding number of selected Phenopedia diseases each gene is involved with:
(In the same order as above)
4, 4, 4, 3, 4, 4, 4, 3, 3, 4, 4, 4, 4, 4, 4, 4, 3, 3, 3, 3, 3, 3, 4, 4, 4, 4, 2, 4, 2, 4, 4, 4, 4, 4, 4, 4, 4, 4, 4, 4, 4, 4,
4, 4, 4, 4, 4, 4, 4, 4, 4, 4, 4, 4, 4, 4, 2, 2, 2, 2, 3, 3, 4, 4, 4, 4, 4, 4, 4, 4, 4, 4, 4, 4, 4, 4, 2, 1, 4, 4, 4, 4, 4, 4,
4, 4, 4, 4, 4, 2, 4, 4, 4, 4, 4, 4, 2, 4, 4, 2, 4, 4, 4, 4, 4, 4, 2, 4, 4, 4, 4, 4, 4, 4, 4, 4, 4, 2, 4, 4, 4, 2, 4, 4, 4, 4,
4, 4, 4, 4, 4, 1, 3, 1, 3, 3, 3, 3, 3, 3, 3, 1, 1, 3, 1, 3, 3, 3, 3, 3, 3, 3, 3, 3, 1, 3, 3, 3, 3, 3, 3, 1, 1, 3, 3, 3, 1,
3, 3, 3, 3, 1, 3, 1, 3, 2, 2, 2, 2, 2, 2, 2, 2, 1, 1, 1, 1, 2, 1, 2, 2, 2, 2, 2, 1, 2, 2, 1, 2, 2, 1, 1, 1, 1, 1, 2, 2, 1,
2, 2, 2, 1, 2, 2, 2, 2, 2, 2, 2, 1, 2, 1, 2, 1, 1, 2, 1, 2, 1, 1, 2, 2, 2, 2, 2, 2, 1, 1, 2, 2, 2, 2, 2, 2, 2, 2, 2, 1, 2, 1,
1, 1, 1, 1, 1, 1, 1, 1, 1, 1, 1, 1, 1, 1, 1, 1, 1, 1, 1, 1, 1, 1, 1, 1, 1, 1, 1, 1, 1, 1, 1, 1, 1, 1, 1, 1, 1, 1, 1, 1, 1, 1,
1, 1, 1, 1, 1, 1, 1, 1, 1, 1, 1, 1, 1, 1, 1, 1, 1, 1, 1, 1, 1, 1, 1, 1, 1, 1, 1, 1, 1, 1, 1, 1, 1, 1, 1

Corresponding number of selected TFs each gene is involved with:
(In the same order as above)
4, 4, 4, 4, 3, 3, 3, 4, 4, 3, 3, 3, 3, 3, 3, 3, 3, 3, 3, 3, 3, 3, 2, 2, 2, 2, 4, 2, 4, 2, 2, 2, 2, 2, 2, 2, 2, 2, 2, 2, 2, 2,
2, 2, 2, 2, 2, 2, 2, 2, 2, 2, 2, 2, 2, 3, 3, 3, 3, 2, 2, 1, 1, 1, 1, 1, 1, 1, 1, 1, 1, 1, 1, 1, 2, 4, 1, 1, 1, 1, 1, 1,
1, 1, 1, 1, 1, 2, 1, 1, 1, 1, 1, 1, 2, 1, 1, 2, 1, 1, 1, 1, 1, 1, 2, 1, 1, 1, 1, 1, 1, 1, 1, 1, 2, 1, 1, 1, 2, 1, 1, 1, 1,
1, 1, 1, 1, 1, 3, 1, 3, 1, 1, 1, 1, 1, 1, 1, 3, 3, 1, 3, 1, 1, 1, 1, 1, 1, 1, 1, 1, 3, 1, 1, 1, 1, 1, 1, 3, 3, 1, 1, 1, 3,
1, 1, 1, 1, 3, 1, 3, 1, 1, 1, 1, 1, 1, 1, 1, 1, 2, 2, 2, 2, 1, 2, 1, 1, 1, 1, 1, 2, 1, 1, 2, 1, 1, 2, 2, 2, 2, 2, 1, 1, 2,
1, 1, 1, 2, 1, 1, 1, 1, 1, 1, 1, 2, 1, 2, 1, 2, 2, 1, 2, 1, 2, 2, 1, 1, 1, 1, 1, 1, 2, 2, 1, 1, 1, 1, 1, 1, 1, 1, 2, 1, 1,
1, 1, 1, 1, 1, 1, 1, 1, 1, 1, 1, 1, 1, 1, 1, 1, 1, 1, 1, 1, 1, 1, 1, 1, 1, 1, 1, 1, 1, 1, 1, 1, 1, 1, 1, 1, 1, 1, 1, 1, 1, 1,
1, 1, 1, 1, 1, 1, 1, 1, 1, 1, 1, 1, 1, 1, 1, 1, 1, 1, 1, 1, 1, 1, 1, 1, 1, 1, 1, 1, 1, 1, 1, 1, 1, 1, 1

Phenopedia diseases ranked according to mean of effect sizes in cluster:
Connective Tissue Diseases | Immune System Diseases | Autoimmune Diseases | Lupus Erythematosus, Systemic

Corresponding mean of effect sizes of each Phenopedia disease:
(In the same order as above)
2.53, 2.48, 2.24, 2.12

Corresponding total number of genes of each Phenopedia disease:
(In the same order as above)
732, 1.8K, 1.1K, 416

Corresponding number of genes of each Phenopedia disease with at least one TF hit (of selected TFs):
(In the same order as above)
161, 320, 214, 106

Corresponding total number of TF hits (of selected TFs) for each Phenopedia disease (in all genes):
(In the same order as above)
248, 473, 317, 167

Corresponding number of selected TFs each Phenopedia disease is involved with:
(In the same order as above)
4, 4, 4, 4

TFs ranked according to mean of effect sizes in cluster:
V$TEL2_Q6, V$ETS1_B, V$GATA1_03, V$PAX_Q6

Corresponding mean of effect sizes of each TF:
(In the same order as above)
2.86, 2.68, 2.68, 1.14

Corresponding total number of TF hits for each TF (genome-wide):
(In the same order as above)
457, 460, 416, 461

Corresponding total number of TF hits for each TF (in all genes in selected Phenopedia diseases):
(In the same order as above)
320, 315, 290, 280

Corresponding number of genes (of selected Phenopedia diseases) each TF is involved with:
(In the same order as above)
129, 126, 108, 120

Corresponding number of selected Phenopedia diseases each TF is involved with:
(In the same order as above)
4, 4, 4, 4

Disease groups of selected Phenopedia diseases ranked according to sum of effect sizes
Immune System Diseases | Connective Tissue Diseases | Autoimmune Diseases | Thoracic Diseases | Respiratory Tract Diseases | Myocarditis | Mediastinitis | Mediastinal Diseases | Lupus Erythematosus, Systemic | Endocarditis | Collagen Diseases | Cardiovascular Diseases | Cardiomyopathies

Corresponding sum of effect sizes for each disease group of selected Phenopedia diseases
(In the same order as above)
18.87, 18.57, 8.94, 8.46, 8.46, 8.46, 8.46, 8.46, 8.46, 8.46, 8.46, 8.46, 8.46

Cluster for columns 16 to 20, rows 50 to 60

Phenopedia diseases

Arthritis, Juvenile Rheumatoid | Arthritis | Arthritis, Rheumatoid | Joint Diseases | Rheumatic Diseases

TFs

V$GATA1_03 | V$AP1_Q6 | V$AP1_01 | V$AP1_Q6_01 | V$AP1_Q4_01 | V$AP1_C | V$AP1_Q4 | V$AP1_Q2 | V$AP1FJ_Q2 | V$AP1_Q2_01 | V$PEBP_Q6

Information

All related TFs:
(List of all TFs that are related to any of the PWMs)
AML1, AML1a, AML1b, AML1c, AML2, AML3, AML3-isoform1, AML3-isoform2, AP-1, FosB, Fra-1, Fra-2, GATA-1, GATA-1A, JunB, JunB:Fra-1, JunB:Fra-2, JunD, JunD:Fra-2, JunD:deltaFosB, PEBP2, PEBP2alpha, PEBP2alphaA1, PEBP2alphaA2, PEBP2alphaB1, PEBP2alphaB2, PEBP2beta, PEBP2beta1, PEBP2beta2, PEBP2beta3, RUNX2-isoform2, RUNX3, Runx3, YAP1, c-Fos, c-Jun, c-Jun:FosB, c-Jun:JunD, c-Jun:c-Fos, deltaFosB

Ranked gene list:
(All genes of the selected Phenopedia diseases with hits of any of the selected TFs, ranked according to the total number of TF hits)
TNF, IRF1, VDR, TNFRSF1A, PSTPIP1, PSMB9, PSMB8, NLRP3, LTA, DDR1, ZFP36, LTB, MAP3K14, IL23A, IL11RA, RUNX1, NFKBIE, CD44, ADORA2A, TNFAIP3, MTHFR, IKBKE, PRDM1, NRM, NKIRAS2, NFKB2, MAL, DUSP1, ABCC1, TNFRSF1B, IL4R, RELA, CD247, CCR7, BAK1, ANP32A, STAT6, SH2D2A, IL7R, HMOX1, HLA-C, HLA-B, RBPJ, NOS2A, NOS2, NFKBIA, IMPDH2, IL10RA, COL6A1, UNC13D, MIF, VEGFA, VEGF, TLR9, TBX21, MSH5, IL2RB, GIMAP5, FAM167A, ERAP2, CLEC16A, C6orf47, SH2B3, RNPEPL1, PTPN6, IL6R, FLI1, TAPBP, PSORS1, PRF1, IL2RA, GSTP1, CDKN1A, SQSTM1, TNFRSF4, TNFRSF18, PRRT1, LIF, IRAK1, GPR132, ESR2, CD3G, LAG3, HCLS1, SOCS1, CSNK1D, CALM1, TAP2, PTPN22, PSMD2, PRKCQ, MEFV, IL10, HLA-DQB1, FOXP3, CLIC1, CD226, CD14, ADRB2, TRADD, TNFRSF14, TNFAIP1, TGFBR2, STAT3, STAT1, SLC19A1, SCGB1A1, RAMP2, PLCL2, PLAU, PDCD1, PADI4, MYD88, MSX2, MMP9, MAP3K7IP1, MAP3K1, IL3, ICAM3, HSPD1, HSPA1L, HMHA1, HLA-E, CSNK2B, COL11A2, CD4, CD2, CD19, CD109, CCR2, CASP7, C6orf15, BAT4, BAT1, APOM, SLC9A3R1, PSORS1C1, P2RX7, COL9A3, CALCA, PTGER4, PHTF1, NQO1, ERBB3, EGFL8, ADRA1A, TNFRSF9, TNFRSF25, TGFB2, SELPLG, RGS2, PHRF1, NCR3, LY6G5B, IL21R, GREM2, FADD, EGR2, ECE2, CD3D, C6orf27, BCL2, AKT3

Corresponding total number of TF hits:
(For each gene listed above, the total number of TF hits for any of the selected TFs, multiplied by the number of selected Phenopedia diseases containing that gene)
50, 50, 45, 45, 45, 45, 45, 45, 45, 45, 44, 44, 40, 40, 40, 36, 36, 36, 36, 35, 35, 32, 28, 28, 28, 28, 28, 28, 28, 25, 25, 24, 24, 24, 24, 24, 20, 20, 20, 20, 20, 20, 16, 16, 16, 16, 16, 16, 16, 15, 15, 12, 12, 12, 12, 12, 12, 12, 12, 12, 12, 12, 11, 11, 11, 11, 11, 10, 10, 10, 10, 10, 10, 9, 8, 8, 8, 8, 8, 8, 8, 8, 7, 7, 6, 6, 6, 5, 5, 5, 5, 5, 5, 5, 5, 5, 5, 5, 5, 4, 4, 4, 4, 4, 4, 4, 4, 4, 4, 4, 4, 4, 4, 4, 4, 4, 4, 4, 4, 4, 4, 4, 4, 4, 4, 4, 4, 4, 4, 4, 4, 4, 4, 3, 3, 3, 3, 3, 2, 2, 2, 2, 2, 2, 1, 1, 1, 1, 1, 1, 1, 1, 1, 1, 1, 1, 1, 1, 1, 1

Corresponding number of selected Phenopedia diseases each gene is involved with:
(In the same order as above)
5, 5, 5, 5, 5, 5, 5, 5, 5, 5, 4, 4, 4, 4, 5, 4, 4, 4, 4, 5, 5, 4, 4, 4, 4, 4, 4, 4, 4, 5, 5, 4, 4, 4, 4, 3, 4, 5, 5, 4, 4, 4, 4, 4, 4, 4, 4, 4, 4, 5, 5, 4, 4, 4, 4, 4, 2, 4, 4, 4, 4, 4, 1, 1, 1, 1, 1, 5, 2, 5, 5, 5, 1, 1, 1, 1, 4, 4, 4, 1, 4, 4, 1, 1, 1, 3, 3, 5, 5, 1, 5, 5, 5, 5, 5, 1, 5, 5, 5, 4, 4, 4, 1, 1, 2, 4, 4, 4, 4, 4, 4, 4, 4, 4, 4, 4, 4, 4, 4, 4, 4, 4, 4, 4, 4, 4, 4, 4, 4, 4, 4, 4, 4, 4, 4, 1, 3, 1, 3, 3, 1, 1, 1, 1, 1, 2, 1, 1, 1, 1, 1, 1, 1, 1, 1, 1, 1, 1, 1, 1, 1, 1

Corresponding number of selected TFs each gene is involved with:
(In the same order as above)
10, 10, 9, 9, 9, 9, 9, 9, 9, 9, 11, 11, 10, 10, 8, 9, 9, 9, 9, 7, 7, 8, 7, 7, 7, 7, 7, 7, 7, 5, 5, 6, 6, 6, 6, 8, 5, 4, 4, 5, 5, 5, 4, 4, 4, 4, 4, 4, 4, 3, 3, 3, 3, 3, 3, 3, 6, 3, 3, 3, 3, 3, 11, 11, 11, 11, 11, 2, 5, 2, 2, 2, 10, 9, 8, 8, 2, 2, 2, 8, 2, 2, 7, 7, 6, 2, 2, 1, 1, 5, 1, 1, 1, 1, 1, 5, 1, 1, 1, 1, 1, 1, 4, 4, 2, 1, 1, 1, 1, 1, 1, 1, 1, 1, 1, 1, 1, 1, 1, 1, 1, 1, 1, 1, 1, 1, 1, 1, 1, 1, 1, 1, 1, 1, 1, 3, 1, 3, 1, 1, 2, 2, 2, 2, 2, 1, 1, 1, 1, 1, 1, 1, 1, 1, 1, 1, 1, 1, 1, 1, 1, 1

Phenopedia diseases ranked according to mean of effect sizes in cluster:
Arthritis, Juvenile Rheumatoid | Arthritis, Rheumatoid | Arthritis | Rheumatic Diseases | Joint Diseases

Corresponding mean of effect sizes of each Phenopedia disease:
(In the same order as above)
2.02, 1.52, 1.49, 1.37, 1.24

Corresponding total number of genes of each Phenopedia disease:
(In the same order as above)
91, 425, 695, 531, 511

Corresponding number of genes of each Phenopedia disease with at least one TF hit (of selected TFs):

(In the same order as above)
33, 112, 161, 122, 120

Corresponding total number of TF hits (of selected TFs) for each Phenopedia disease (in all genes):
(In the same order as above)
156, 436, 624, 468, 458

Corresponding number of selected TFs each Phenopedia disease is involved with:
(In the same order as above)
11, 11, 11, 11, 11

TFs ranked according to mean of effect sizes in cluster:
V$AP1_Q6_01, V$AP1_01, V$AP1_Q4_01, V$AP1_Q2_01, V$AP1FJ_Q2, V$AP1_C, V$AP1_Q6, V$GATA1_03, V$AP1_Q2, V$AP1_Q4, V$PEBP_Q6

Corresponding mean of effect sizes of each TF:
(In the same order as above)
2.38, 1.68, 1.67, 1.66, 1.66, 1.51, 1.49, 1.39, 1.23, 1.08, 1.02

Corresponding total number of TF hits for each TF (genome-wide):
(In the same order as above)
470, 459, 472, 444, 436, 469, 453, 416, 444, 446, 464

Corresponding total number of TF hits for each TF (in all genes in selected Phenopedia diseases):
(In the same order as above)
244, 206, 222, 181, 183, 217, 190, 146, 192, 192, 169

Corresponding number of genes (of selected Phenopedia diseases) each TF is involved with:
(In the same order as above)
70, 63, 67, 50, 54, 63, 58, 43, 56, 59, 51

Corresponding number of selected Phenopedia diseases each TF is involved with:
(In the same order as above)
5, 5, 5, 5, 5, 5, 5, 5, 5, 5, 5

Disease groups of selected Phenopedia diseases ranked according to sum of effect sizes
Immune System Diseases | Autoimmune Diseases | Arthritis, Rheumatoid | Musculoskeletal Diseases | Arthritis, Juvenile Rheumatoid | Arthritis | Rheumatic Diseases | Joint Diseases

Corresponding sum of effect sizes for each disease group of selected Phenopedia diseases
(In the same order as above)
38.87, 38.87, 38.87, 31.47, 22.19, 16.37, 15.10, 13.60

Cluster for columns 104 to 110, rows 43 to 49

Phenopedia diseases

Bloom Syndrome | Hepatitis | Hepatitis B | Hepatitis C | Hepatitis E | Hepatitis A | Hepatitis D

TFs

V$ALX4_01 | V$GCNF_01 | V$STAT5A_02 | V$HSF_Q6 | V$TEL2_Q6 | V$ETS1_B | V$PAX_Q6

Information

All related TFs:
(List of all TFs that are related to any of the PWMs)
Alx-4, GCNF, GCNF-1, GCNF-2, HSF, HSF1, Pax-1, Pax-2, Pax-2a, Pax-3, Pax-4a, Pax-4c, Pax-5, Pax-6, Pax-8, Pax6-1, STAT5A, Tel-2a, Tel-2b, Tel-2c, c-Ets-1

Ranked gene list:
(All genes of the selected Phenopedia diseases with hits of any of the selected TFs, ranked according to the total number of TF hits)
TSPAN31, SH2B3, RAX2, NLRP3, NFATC1, IRF1, HAGHL, CD82, CD79A, ULBP1, TNFRSF6B, TLR9, SFTPC, RASSF5, PIK3R1, NDUFS2, LDLRAP1, ITGB2, GRN, GPIHBP1, GIMAP5, FAIM3, F11R, CCDC78, SOCS1, MAPKAPK3, IRF8, XCR1, TSPAN4, TNFRSF1B, TCOF1, SPAG4L, SOCS3, SH3BP2, RASSF1, PTMS, PRF1, POLR2H, PNKD, PIK3CG, PDE4C, OPRD1, NUDT22, NR1H3, NFE2L1, MS4A14, MADD, MACF1, LZTFL1, LIMS2, LAG3, KLF6, JAG2, IL7R, IL4R, IL2RB, IL16, IL15, IFI6, HSH2D, HLA-DPB1, HLA-DPA1, HAGH, GATA3, FCER1A, FASLG, FAHD1, ESR1, CYP1A1, CTLA4, CREM, CNTFR, CD97, CD79B, CD63, CD4, CD3G, CD3D, CD27, CCR7, CCR4, CASP8AP2, BPIL1, BMP1, BCL6, BCL2L13, ARHGAP4, ALDH16A1, AHRR, AAMP, TPH2, SIGIRR, PSMB9, PSMB8, NOTCH4, IRF9, ELF2, EIF2S1, DHX58, BTLA, ZBP1, VDR, VDAC1, VAV1, USP35, UMOD, ULBP3, TRAF6, TRAF5, TRAF3, TRAF1, TP53, TNFSF14, TNFSF13B, TNFSF13, TNFRSF18, TNFRSF14, TNFRSF13C, TNFRSF10D, THPO, TGFBR2, TFRC, TANK, STAT1, STARD5, SPHK1, SPARCL1, SOCS2, SNAPC4, SMG7, SLITRK2, SLAMF7, SIGLEC9, SIGLEC12, SIGLEC1, SAMHD1, RPAIN, RAG2, PTGIR, PTGER4, PTGER3, PSRC1, PRDX2, PRDX1, PKN1, PIM1, PGLYRP4, PGLYRP2, OSM, NUP214, NOS3, NGFR, NFKBIA, NFKB1, NFATC3, NFATC2, NCR1, NCK2, NCK1, NAB2, MYO7B, MX2, MX1, MR1, MMP9, MIF, MFNG, MAGED2, LY96, LY9, LY6E, LTBR, LSP1, LRRC32, LRMP, LIPA, LIF, LAT, ITGB1, IRF3, IMPACT, ILF2, IL8RB, IL6R, IL5RA, IL2RG, IL28RA, IL21R, IL21, IL20, IL1F5, IL1F10, IL17RA, IL17C, IL10RA, IFNGR2, IFNAR1, IFNA7, ICAM4, HLA-DRA, HLA-C, HLA-B, GSTO1, GSTA5, GSTA4, GSTA2, GSTA1, GRIN1, GPSM1, GPR68, GNLY, GML, GIMAP6, GDF10, GCA, GALT, GAB2, FYCO1, FPR2, FOXP4, FOXP1, FOXJ2, FGFR1OP, FCN1, FCER1G, FBXW5, FAS, FAM20B, ESR2, EMR2, EDN1, DUS4L, DNAJC4, DLGAP1, CXCR2, CX3CL1, CTBP1, CSF3, COPE, CLEC4G, CLC, CHUK, CES2, CDH1, CD9, CD86, CD83, CD72, CD58, CD55, CD53, CD22, CD19, CASP14, CARD11, CADM3, C9, C3, C1QC, C1QB, C1QA, BPHL, BIRC3, ATP2C1, APOL3, APOE, APOA2, APC2, ALDH3B1, ALDH2, AIF1L, ADRB2, TNFRSF4, TGFBRAP1, TGFA, TBX21, TAP2, TAP1, STAT5A, SPARC, SP110, SLC26A9, RB1, POLG2, OAS3, OAS2, MT1X, MMP14, MMP11, MBP, LTBP4, LTBP3, LTBP2, LTBP1, LILRA5, IRF7, IL28B, IL23A, IL18BP, IFNA17, IFNA14, IFNA10, IFITM1, ICAM3, HAVCR2, FURIN, EIF2AK2, DDX5, CXCR4, CPT1A, CLDN1, CD34, CABIN1, BMP8B, AIRE, ADA, UCP3, TNIP1, SLC35B2, SLC23A1, SIPA1, GPX4, TAPBPL, NFKBIE, BAK1, AKT1, TSPO, TRADD, TEP1, TDP1, SRA1, RECQL4, RDH5, PSD, POLR2E, POLR2A, PLCD1, NUBP2, NHEJ1, MPG, METTL1, LMNA, JAK3, HSP90AB1, HDAC10, GYS1, GBF1, GBA, FOXC1, DCLRE1C, CYBA, COASY, CDKN1A, CDK5, CDC25B, CCND3, CCDC97, CASP8, BCL2A1, ARHGDIB, AP2M1, AGER, ZNF575, XRCC4, WRAP53, WDR79, WDR66, UCP2, TRPM5, TP53I3, TNFSF12, TMEM151B, THOC6, TCTE1, STX1A, SMPD1, SLC6A18, SLC30A1, SLC19A1, SHFM1, SF3B14, SEC14L2, SCNN1A, RXRB, RP5-1077B9.4, RFFL, RFC4, RFC2, RELA, RB1CC1, RAD23B, RAB15, PTPN1, PTGDS, PPARD, POLRMT, POLR2K, POLR2J, POLR2C, POLL, POLK, POLG, PMS1, PLA2G6, PCTP, PARP4, PARP1, NUBP1, NPPA, NINJ1, NCOA3, N4BP2L1, MYO5A, MYNN, MYC, MTHFD2, MSH5, MRPL4, MRE11A, MPDU1, MGMT, MDN1, MATR3, MAP3K2, LST1, LRP5, LEPREL2, LCAT, KRAS, KDSR, IRGQ, HSPA8, HSD17B1, HMOX1, GTF2H1, GPR162, GNB5, GCLC, FBXW7, FAM82A2, FADD, ERCC6, ERCC4, ERCC3, ERBB2, ENG, EFNB3, DRD1, DIO1, CYP27B1, COL18A1, CHEK2, CFLAR, CETP, CDKN1C, CDK4, CBS, CASP6, CAPN10, C11orf74, BZRPL1, BHMT, BCL7C, BCL2L2, BCL2L11, BCL2L1, BAX, BARD1, APAF1, ANKRD49, ALOX12, ABCA5

Corresponding total number of TF hits:
(For each gene listed above, the total number of TF hits for any of the selected TFs, multiplied by the number of selected Phenopedia diseases containing that gene)

28, 28, 28, 28, 28, 28, 28, 28, 28, 21, 21, 21, 21, 21, 21, 21, 21, 21, 21, 21, 21, 21, 21, 21, 18, 18, 18, 14, 14, 14, 14, 14, 14, 14, 14, 14, 14, 14, 14, 14, 14, 14, 14, 14, 14, 14, 14, 14, 14, 14, 14, 14, 14, 14, 14, 14, 14, 14, 14, 14, 14, 14, 14, 14, 14, 14, 14, 14, 14, 14, 14, 14, 14, 14, 14, 14, 14, 14, 14, 14, 14, 14, 14, 14, 14, 14, 14, 14, 14, 12, 12, 12, 12, 12, 12, 12, 12, 12, 12, 7, 6, 6, 6, 6, 6, 6, 6, 6, 6, 6, 6, 6, 6, 6, 6, 6, 6, 6, 6, 6, 6, 6, 6, 6, 6, 6, 6, 6, 6, 6, 6, 6, 6, 6, 6, 6, 6, 6, 6, 6, 6, 6, 6, 6, 4, 4, 4, 4, 4, 4, 3, 3, 3, 3, 2, 2, 2, 2, 2, 2, 2, 2, 2, 2, 2, 2, 2, 2, 2, 2, 2, 2, 2, 2, 2, 2, 2, 2, 2, 2, 2, 2, 2, 2, 2, 2, 2, 2, 2, 2, 1

Corresponding number of selected Phenopedia diseases each gene is involved with:
(In the same order as above)
7, 7, 7, 7, 7, 7, 7, 7, 7, 7, 7, 7, 7, 7, 7, 7, 7, 7, 7, 7, 7, 7, 7, 7, 6, 6, 6, 7, 7, 7, 7, 7, 7, 7, 7, 7, 7, 7, 7, 7, 7, 7, 7, 7, 7, 7, 7, 7, 7, 7, 7, 7, 7, 7, 7, 7, 7, 7, 7, 7, 7, 7, 7, 7, 7, 7, 7, 7, 7, 7, 7, 7, 7, 7, 7, 7, 7, 7, 7, 7, 7, 7, 7, 7, 7, 7, 7, 7, 7, 7, 6, 6, 6, 6, 6, 6, 6, 6, 6, 6, 7, 6, 6, 6, 6, 6, 6, 6, 6, 6, 6, 6, 6, 6, 6, 6, 6, 6, 6, 6, 6, 6, 6, 6, 6, 6, 6, 6, 6, 6, 6, 6, 6, 6, 6, 6, 6, 6, 6, 6, 6, 6, 6, 6, 1, 1, 1, 1, 1, 2, 1, 1, 1, 1, 1, 1, 1, 1, 1, 1, 1, 1, 1, 1, 1, 1, 1, 1, 1, 1, 1, 1, 1, 1, 2, 2, 1

Corresponding number of selected TFs each gene is involved with:
(In the same order as above)
4, 4, 4, 4, 4, 4, 4, 4, 4, 3, 3, 3, 3, 3, 3, 3, 3, 3, 3, 3, 3, 3, 3, 3, 3, 3, 2, 2, 2, 2, 2, 2, 2, 2, 2, 2, 2, 2, 2, 2, 2, 2, 2, 2, 2, 2, 2, 2, 2, 2, 2, 2, 2, 2, 2, 2, 2, 2, 2, 2, 2, 2, 2, 2, 2, 2, 2, 2, 2, 2, 2, 2, 2, 2, 2, 2, 2, 2, 2, 2, 2, 2, 2, 2, 2, 2, 2, 2, 2, 2, 2, 2, 2, 2, 2, 2, 2, 2, 2, 1, 4, 4, 4, 4, 4, 2, 3, 3, 3, 3, 2, 2, 2, 2, 2, 2, 2, 2, 2, 2, 2, 2, 2, 2, 1, 1, 2, 2, 2, 2, 2, 2, 2, 2, 2, 2, 2, 2, 2, 2, 2, 2, 1

Phenopedia diseases ranked according to mean of effect sizes in cluster:
Hepatitis E | Hepatitis D | Hepatitis A | Hepatitis C | Hepatitis B | Hepatitis | Bloom Syndrome

Corresponding mean of effect sizes of each Phenopedia disease:
(In the same order as above)
1.89, 1.89, 1.89, 1.89, 1.85, 1.73, 1.65

Corresponding total number of genes of each Phenopedia disease:
(In the same order as above)
1.1K, 1.1K, 1.1K, 1.1K, 1.1K, 1.1K, 1.4K

Corresponding number of genes of each Phenopedia disease with at least one TF hit (of selected

TFs):
(In the same order as above)
316, 316, 316, 316, 317, 320, 413

Corresponding total number of TF hits (of selected TFs) for each Phenopedia disease (in all genes):
(In the same order as above)
452, 452, 452, 452, 453, 458, 590

Corresponding number of selected TFs each Phenopedia disease is involved with:
(In the same order as above)
7, 7, 7, 7, 7, 7, 7

TFs ranked according to mean of effect sizes in cluster:
VPAX_Q6, VGCNF_01, V$ETS1_B, V$ALX4_01, V$TEL2_Q6, V$STAT5A_02, V$HSF_Q6

Corresponding mean of effect sizes of each TF:
(In the same order as above)
2.91, 2.91, 2.55, 1.84, 1.43, 0.71, 0.43

Corresponding total number of TF hits for each TF (genome-wide):
(In the same order as above)
461, 492, 460, 490, 457, 471, 448

Corresponding total number of TF hits for each TF (in all genes in selected Phenopedia diseases):
(In the same order as above)
544, 532, 494, 463, 428, 429, 419

Corresponding number of genes (of selected Phenopedia diseases) each TF is involved with:
(In the same order as above)
114, 101, 100, 86, 92, 89, 84

Corresponding number of selected Phenopedia diseases each TF is involved with:
(In the same order as above)
7, 7, 7, 7, 7, 7, 7

Disease groups of selected Phenopedia diseases ranked according to sum of effect sizes
Signs and Symptoms | Virus Diseases | Communicable Diseases | Liver Diseases | Hepatitis | Digestive System Diseases | Hepatitis, Viral, Human | RNA Virus Infections | Picornaviridae Infections | Hepatitis E | Hepatitis D | Hepatitis A | Enterovirus Infections | Hepatitis C | Flaviviridae Infections | Arbovirus Infections | Hepatitis B | Hepadnaviridae Infections | DNA Virus Infections | DNA Repair-Deficiency Disorders | Congenital, Hereditary, and Neonatal Diseases and Abnormalities | Bloom Syndrome | Abnormalities, Multiple

Corresponding sum of effect sizes for each disease group of selected Phenopedia diseases
(In the same order as above)
77.45, 65.89, 65.89, 64.74, 64.74, 64.74, 52.64, 26.50, 13.25, 13.25, 13.25, 13.25, 13.25, 13.20, 13.20, 13.20, 12.94, 12.94, 12.94, 11.55, 11.55, 11.55, 11.55

Cluster for columns 139 to 155, rows 47 to 50

Phenopedia diseases

Meningioma | Glioma | Astrocytoma | Glioblastoma | Leukemia | Neoplasms, Nerve Tissue | DNA Damage | Lymphoma | Occupational Diseases | Hematologic Diseases | Stress | Lymphoma, Non-Hodgkin | Lymphatic Diseases | Lymphoproliferative Disorders | Leukemia, B-Cell, Chronic | Leukemia,

Lymphocytic, Chronic | Hodgkin Disease

TFs

V$TEL2_Q6 | V$ETS1_B | V$PAX_Q6 | V$GATA1_03

Information

All related TFs:
(List of all TFs that are related to any of the PWMs)
GATA-1, GATA-1A, Pax-1, Pax-2, Pax-2a, Pax-3, Pax-4a, Pax-4c, Pax-5, Pax-6, Pax-8, Pax6-1, Tel-2a, Tel-2b, Tel-2c, c-Ets-1

Ranked gene list:
(All genes of the selected Phenopedia diseases with hits of any of the selected TFs, ranked according to the total number of TF hits)
NFKBIE, SELPLG, RAC2, CDKN1A, IL4R, CYBA, TRADD, SLC23A1, CD79A, BAK1, AKT1, TNFRSF1B, TLR9, ITGB2, CD79B, IL11RA, SLC35B2, CTLA4, CDK4, TWF2, TAPBPL, NFKB1, NBN, JAK3, ERCC1, CCR7, TNIP1, PRF1, CASP8, TNFRSF18, NDUFS2, DCLRE1C, CD14, NOS1, MSH2, ERCC6, UNC84B, TRAF5, SOCS1, RAVER1, PTMS, PIM1, NCF4, MYC, MPG, MMP9, IL6R, IL10RA, GPX4, FAS, ERCC4, DUSP2, CFLAR, CASP8AP2, CASP6, BCL7C, BCL6, BCL2L1, BCL2A1, BAX, XRCC5, TRAF6, TNFRSF14, TNFRSF13C, TNFRSF10D, TANK, SOCS3, NOD1, CD4, ATG9B, APOA2, VDR, TNFSF13B, SIPA1, SHMT2, SCNN1A, RFFL, PRKD2, NR1H3, LTBR, LST1, LAG3, ICAM5, ICAM4, CD55, C1RL, C1QC, C1QB, C1QA, TNFSF13, TNFRSF1A, TFRC, MEFV, LTC4S, IRAK4, IL16, GBA, CXCR4, NOTCH1, FOXJ2, BRAF, TWF1, TSLP, TMCO6, TDP1, SFRS15, RTEL1, PTPN11, PAX5, NOTCH4, NLRP3, NAB2, MTHFD1, MBP, LY96, LEPREL2, IL17C, ICAM3, HES7, GPR108, GFI1, G6PD, DHX33, DEF6, CXXC5, COL11A2, CNTNAP1, CDKN1B, C8G, ALOXE3, AGER, ZNF575, TNFSF12, TNFRSF25, TMEM151B, TGFBR2, TCTE1, SLC3A2, RAB15, PTPN1, PSD, POLR2E, PLEKHG5, MDN1, IRGQ, IL7R, HSPA1L, HSP90AB1, HSD17B1, H2AFX, GSTO1, GPR162, GBF1, CRHR1, CDK6, CD27, CCDC109B, ULK3, SYK, RIPK3, PTPN6, PNKP, NTRK1, NPAS2, NHEJ1, MSH5, MDC1, MAPKAPK3, MAP3K5, LSP1, HMHA1, GATA2, ERBB2, EME1, CSF3R, CDC25C, BCL3, APAF1, UCP3, TRAF3, TAL1, RECQL4, RCAN3, RAP1GAP, PTCH1, PER1, NDUFS8, MMP14, JUNB, IKZF1, IER3, GNAI2, GMNN, GADD45A, COL2A1, CEBPE, CCL27, CARD8, C12orf57, BMP4, BID, BGLAP, BAT3, VEGFB, TXNRD1, TXN, TNFRSF6B, TINF2, TAF8, SHFM1, RPS16, RCSD1, RAD23A, PRDX5, PRDX2, PPP1R1B, POLG, PER2, NDUFV1, LMNA, KCNQ1, ITGA2B, IL2RB, IL27, HSPD1, HSPB6, GTF2H1, GGT1, FEN1, EMILIN1, E2F4, DUOX2, DNMT1, DLL1, CYP27B1, CDC25B, CDC20, CD226, CCND3, CARD4, CAMKK1, CAMK2G, BRD2, B4GALT1, ATF3, ARF1, AQP1, WFS1, VPS13B, VKORC1, VIM, TSPAN9, TNIK, TNFRSF4, TMEM173, THPO, TAP1, STX11, SPRY2, SPRED3, SOX18, SMAD4, SMAD3, SIGMAR1, SHC1, SELS, SCYL3, RPL5, RPL35A, RORC, PTGER3, PRDM1, POLG2, PLA2G6, PITX3, PHF1, PC, PARP4, PALB2, NME7, NDUFB7, NDUFB10, NDUFA7, NDRG1, MME, MGRN1, MAN2A1, MAN1B1, LIF, KNG1, ITGB1, IRF9, IRF7, ING1, HSPA14, HSD11B2, HINT1, HAVCR2, GLI1, GFRA2, FOSB, EPB41L2, EFNA3, ECE1, DNAJB2, CYB561, CTSZ, CTSD, CNTFR, CLPTM1L, CD72, CCND2, C6orf129, BLZF1, BECN1, ATF4, AKR1B1, AHRR, ADRA2C, ACHE

Corresponding total number of TF hits:
(For each gene listed above, the total number of TF hits for any of the selected TFs, multiplied by the number of selected Phenopedia diseases containing that gene)
44, 36, 36, 33, 32, 26, 24, 24, 24, 24, 24, 22, 22, 22, 22, 21, 20, 20, 20, 18, 18, 17, 17, 17, 16, 16, 15, 15, 15, 14, 14, 14, 14, 13, 13, 13, 12, 12, 12, 12, 12, 12, 12, 12, 12, 12, 12, 12, 12, 12, 12, 12, 12, 12, 12, 12, 12, 12, 12, 11, 11, 11, 11, 11, 11, 11, 11, 11, 11, 11, 10, 10, 10, 10, 10, 10, 10, 10, 10, 10, 10, 10, 10, 10, 10, 10, 10, 10, 9, 9, 8, 8, 8, 8, 8, 8, 8, 8, 7, 7, 7, 6, 6, 6, 6, 6, 6, 6, 6, 6, 6, 6, 6, 6, 6, 6, 6, 6, 6, 6, 6, 6, 6, 6, 6, 6, 6, 6, 6, 6, 6, 5, 5, 5, 5, 5, 5, 5, 5, 5, 5, 5, 5, 5, 5, 5, 5, 5, 5, 5, 5, 5, 5, 5, 5, 5, 5, 4, 4, 4, 4, 4, 4, 4, 4, 4, 4, 4, 4, 4, 4, 4, 4, 4, 4, 4, 4, 4, 3, 3, 3, 3, 3, 3, 3, 3, 3, 3, 3, 3, 3, 3, 3, 3, 3, 3, 3, 3, 3, 3, 3, 3, 3, 2, 2, 2, 2, 2,

2, 2, 2, 2, 2, 2, 2, 2, 2, 2, 2, 2, 2, 2, 2, 2, 2, 2, 2, 2, 2, 2, 2, 2, 2, 2, 2, 2, 2, 2, 2, 2, 2, 2, 2, 2, 1, 1, 1, 1, 1, 1, 1, 1, 1, 1, 1, 1, 1, 1, 1, 1, 1, 1, 1, 1, 1, 1, 1, 1, 1, 1, 1, 1, 1, 1, 1, 1, 1, 1, 1, 1, 1, 1, 1, 1, 1, 1, 1, 1, 1, 1, 1, 1, 1, 1, 1, 1, 1, 1, 1, 1, 1, 1, 1, 1, 1, 1, 1, 1, 1, 1, 1, 1, 1, 1, 1

Corresponding number of selected Phenopedia diseases each gene is involved with:
(In the same order as above)
11, 12, 12, 11, 16, 13, 12, 6, 8, 8, 8, 11, 11, 11, 11, 7, 5, 10, 10, 6, 6, 17, 17, 17, 16, 8, 5, 5, 15, 14, 7, 7, 14, 13, 13, 13, 4, 12, 6, 6, 6, 12, 12, 12, 4, 12, 12, 12, 12, 12, 12, 4, 12, 12, 12, 12, 12, 12, 12, 12, 11, 11, 11, 11, 11, 11, 11, 11, 11, 11, 11, 10, 10, 5, 5, 10, 5, 5, 5, 10, 5, 5, 10, 10, 10, 10, 10, 10, 10, 9, 9, 8, 8, 4, 8, 8, 4, 8, 7, 7, 7, 6, 6, 6, 3, 6, 3, 6, 3, 3, 2, 6, 6, 6, 6, 6, 6, 6, 6, 3, 6, 6, 6, 3, 2, 6, 6, 6, 6, 2, 5, 5, 5, 5, 5, 5, 5, 5, 5, 5, 5, 5, 5, 5, 5, 5, 5, 5, 5, 5, 5, 5, 5, 5, 5, 2, 4, 4, 1, 2, 4, 4, 2, 2, 4, 1, 4, 4, 2, 2, 4, 4, 4, 4, 2, 4, 1, 3, 3, 3, 3, 3, 3, 1, 1, 3, 1, 3, 3, 1, 3, 3, 3, 3, 3, 3, 1, 3, 3, 3, 3, 2, 2, 2, 1, 1, 1, 2, 1, 1, 2, 1, 2, 1, 2, 2, 1, 1, 2, 2, 1, 2, 2, 2, 2, 2, 2, 1, 1, 2, 2, 1, 2, 1, 1, 2, 2, 2, 1, 1, 1, 1, 1, 2, 1, 1, 1, 1, 1, 1, 1, 1, 1, 1, 1, 1, 1, 1, 1, 1, 1, 1, 1, 1, 1, 1, 1, 1, 1, 1, 1, 1, 1, 1, 1, 1, 1, 1, 1, 1, 1, 1, 1, 1, 1, 1, 1, 1, 1, 1, 1, 1, 1, 1, 1, 1, 1, 1, 1, 1, 1, 1, 1, 1, 1, 1, 1, 1, 1, 1, 1, 1, 1, 1, 1, 1

Corresponding number of selected TFs each gene is involved with:
(In the same order as above)
4, 3, 3, 3, 2, 2, 2, 4, 3, 3, 3, 2, 2, 2, 2, 3, 4, 2, 2, 3, 3, 1, 1, 1, 1, 2, 3, 3, 1, 1, 2, 2, 1, 1, 1, 1, 3, 1, 2, 2, 2, 1, 1, 1, 3, 1, 1, 1, 1, 1, 1, 3, 1, 1, 1, 1, 1, 1, 1, 1, 1, 1, 1, 1, 1, 1, 1, 1, 1, 1, 1, 1, 1, 1, 2, 2, 1, 2, 2, 2, 1, 2, 2, 1, 1, 1, 1, 1, 1, 1, 1, 1, 1, 2, 1, 1, 2, 1, 1, 1, 1, 1, 1, 2, 1, 2, 1, 2, 2, 3, 1, 1, 1, 1, 1, 1, 1, 1, 1, 2, 1, 1, 1, 2, 3, 1, 1, 1, 1, 3, 1, 1, 1, 1, 1, 1, 1, 1, 1, 1, 1, 1, 1, 1, 1, 1, 1, 1, 1, 1, 1, 1, 1, 1, 1, 1, 1, 1, 2, 1, 1, 4, 2, 1, 1, 2, 2, 1, 4, 1, 1, 2, 2, 1, 1, 1, 1, 2, 1, 3, 1, 1, 1, 1, 1, 1, 3, 3, 1, 3, 1, 1, 3, 1, 1, 1, 1, 1, 1, 3, 1, 1, 1, 1, 1, 1, 1, 2, 2, 2, 1, 2, 2, 1, 2, 1, 2, 1, 1, 2, 2, 1, 1, 2, 1, 1, 1, 1, 1, 1, 2, 2, 1, 1, 2, 1, 2, 2, 1, 1, 1, 2, 2, 2, 2, 2, 1, 2, 1, 1, 1, 1, 1, 1, 1, 1, 1, 1, 1, 1, 1, 1, 1, 1, 1, 1, 1, 1, 1, 1, 1, 1, 1, 1, 1, 1, 1, 1, 1, 1, 1, 1, 1, 1, 1, 1, 1, 1, 1, 1, 1, 1, 1, 1, 1, 1, 1, 1, 1, 1, 1, 1, 1, 1, 1, 1, 1, 1, 1, 1, 1, 1, 1, 1, 1, 1, 1

Phenopedia diseases ranked according to mean of effect sizes in cluster:
Lymphoma | Occupational Diseases | Meningioma | Leukemia | Astrocytoma | Neoplasms, Nerve Tissue | Glioma | Hematologic Diseases | Glioblastoma | DNA Damage | Lymphoma, Non-Hodgkin | Lymphoproliferative Disorders | Lymphatic Diseases | Leukemia, B-Cell, Chronic | Leukemia, Lymphocytic, Chronic | Hodgkin Disease | Stress

Corresponding mean of effect sizes of each Phenopedia disease:
(In the same order as above)
1.97, 1.82, 1.77, 1.76, 1.74, 1.57, 1.46, 1.45, 1.43, 1.36, 1.23, 1.22, 1.14, 0.94, 0.89, 0.65, 0.43

Corresponding total number of genes of each Phenopedia disease:
(In the same order as above)
585, 358, 320, 869, 255, 593, 336, 733, 275, 455, 374, 606, 654, 307, 312, 172, 775

Corresponding number of genes of each Phenopedia disease with at least one TF hit (of selected TFs):
(In the same order as above)
118, 79, 67, 173, 55, 114, 67, 122, 56, 95, 67, 111, 119, 64, 64, 38, 123

Corresponding total number of TF hits (of selected TFs) for each Phenopedia disease (in all genes):
(In the same order as above)
166, 108, 90, 231, 73, 162, 88, 163, 74, 125, 93, 152, 161, 83, 83, 48, 169

Corresponding number of selected TFs each Phenopedia disease is involved with:
(In the same order as above)
4, 4, 4, 4, 4, 4, 4, 4, 4, 4, 4, 4, 4, 4, 4, 4, 4

TFs ranked according to mean of effect sizes in cluster:
V$ETS1_B, V$PAX_Q6, V$TEL2_Q6, V$GATA1_03

Corresponding mean of effect sizes of each TF:
(In the same order as above)
1.98, 1.61, 0.89, 0.89

Corresponding total number of TF hits for each TF (genome-wide):
(In the same order as above)
460, 461, 457, 416

Corresponding total number of TF hits for each TF (in all genes in selected Phenopedia diseases):
(In the same order as above)
583, 572, 464, 450

Corresponding number of genes (of selected Phenopedia diseases) each TF is involved with:
(In the same order as above)
124, 124, 104, 87

Corresponding number of selected Phenopedia diseases each TF is involved with:
(In the same order as above)
17, 17, 17, 17

Disease groups of selected Phenopedia diseases ranked according to sum of effect sizes
Neoplasms | Hematologic Neoplasms | Neoplasms, Neuroepithelial | Glioma | Lymphoma, T-Cell | Lymphoma, Non-Hodgkin | Lymphoma | Nervous System Neoplasms | Nervous System Diseases | Head and Neck Neoplasms | Central Nervous System Neoplasms | Central Nervous System Diseases | Brain Neoplasms | Brain Diseases | Occupational Diseases | Spinal Diseases | Spinal Cord Neoplasms | Spinal Cord Diseases | Musculoskeletal Diseases | Meningioma | Meningeal Neoplasms | Bone Diseases | Leukemia | Astrocytoma | Neoplasms, Nerve Tissue | Hematologic Diseases | Glioblastoma | DNA Damage | Signs and Symptoms | Sex Chromosome Disorders | Lymphoproliferative Disorders | Genetic Diseases, X-Linked | Genetic Diseases, Inborn | Lymphatic Diseases | Leukemia, B-Cell, Chronic | Leukemia, Lymphocytic, Chronic | Hodgkin Disease | Stress

Corresponding sum of effect sizes for each disease group of selected Phenopedia diseases
(In the same order as above)
42.76, 22.44, 18.51, 18.51, 15.38, 15.38, 15.38, 14.05, 14.05, 14.05, 14.05, 14.05, 14.05, 14.05, 7.29, 7.07, 7.07, 7.07, 7.07, 7.07, 7.07, 7.07, 7.06, 6.98, 6.27, 5.78, 5.70, 5.43, 4.88, 4.88, 4.88, 4.88, 4.88, 4.56, 3.77, 3.56, 2.59, 1.72

Cluster for columns 159 to 172, rows 32 to 41

Phenopedia diseases

Hyperlipidemia, Familial Combined | Lipid Metabolism, Inborn Errors | Metabolism, Inborn Errors | Hyperlipoproteinemia Type II | Hyperlipoproteinemias | Hypertriglyceridemia | Lipid Metabolism Disorders | Dyslipidemias | Hyperlipidemias | Diseases in Twins | Cholecystolithiasis | Cholelithiasis | Gallbladder Diseases | Gallstones

TFs

V$HNF4_01 | V$COUP_01 | V$PPAR_DR1_Q2 | V$DR1_Q3 | V$COUP_DR1_Q6 | V$HNF4ALPHA_Q6 | V$COUPTF_Q6 | V$HNF4_Q6_01 | V$HNF4_DR1_Q3 | V$PPARG_01

Information

All related TFs:
(List of all TFs that are related to any of the PWMs)
COUP, COUP-TF1, COUP-TF2, HNF-4, HNF-4alpha, HNF-4alpha1, HNF-4alpha2, HNF-4alpha3, HNF-4alpha4, HNF-4alpha7, HNF-4gamma, PPAR-alpha, PPAR-alpha:RXR-alpha, PPAR-beta, PPAR-gamma, PPAR-gamma1, PPAR-gamma2, PPAR-gamma2:RXR-alpha, PPAR-gamma:RXR-alpha, RAR-alpha:RXR-alpha

Ranked gene list:
(All genes of the selected Phenopedia diseases with hits of any of the selected TFs, ranked according to the total number of TF hits)
APOC3, APOA4, APOA1, RXRB, NR1H3, CRABP2, LCAT, PPARA, CYP1A1, LRP1, COMT, NOS3, CILP2, VEGFA, CAPN10, FADS3, TNF, OSBPL2, ARSA, GAL, ACAT2, TPP1, LPIN1, GCKR, ESR1, RXRA, NR1H4, MC1R, APOA5, SILV, PNPLA2, VEGF, TCF1, SOD2, MYL3, INSR, HNF1A, GALK1, FABP2, ETFDH, CFB, PPARD, NPC1L1, ITPA, CAPG, PAFAH2, MTRR, LTA, ITGB2, IL1B, TNFRSF1B, SLC11A2, SF4, GALNT2, F5, BAZ1B, SREBF1, PSMB9, PSMB8, LMNA, ETFA, ACADS, UTS2, UROD, UBL5, TAP1, SLC2A9, PLXNA2, NPC1, ITIH4, HEXA, CCL11, UCP2, TP53, SLC1A5, MYH7, HLA-B, GALT, TNFRSF1A, SLC6A9, SLC2A6, SLC25A13, OTC, MGMT, LRRC16A, IDUA, HPS1, HLA-C, HAMP, HADHA, GUSB, CYP11A1, CRHR1, CBS, C1QA, AMT, ALDH2

Corresponding total number of TF hits:
(For each gene listed above, the total number of TF hits for any of the selected TFs, multiplied by the number of selected Phenopedia diseases containing that gene)
126, 126, 108, 70, 70, 56, 54, 49, 45, 40, 35, 27, 25, 24, 24, 20, 18, 18, 18, 16, 16, 15, 12, 12, 12, 10, 10, 10, 10, 9, 9, 8, 8, 8, 8, 8, 8, 8, 8, 8, 8, 8, 7, 7, 7, 7, 6, 6, 6, 6, 6, 5, 5, 5, 5, 5, 5, 4, 4, 4, 4, 4, 4, 3, 3, 3, 3, 3, 3, 3, 3, 3, 3, 2, 2, 2, 2, 2, 2, 1, 1, 1, 1, 1, 1, 1, 1, 1, 1, 1, 1, 1, 1, 1, 1, 1, 1

Corresponding number of selected Phenopedia diseases each gene is involved with:
(In the same order as above)
14, 14, 12, 7, 10, 7, 9, 7, 5, 8, 5, 9, 5, 3, 4, 2, 9, 6, 3, 4, 4, 3, 2, 6, 12, 10, 10, 1, 10, 1, 1, 1, 4, 2, 2, 4, 4, 1, 8, 1, 1, 7, 7, 1, 7, 3, 2, 3, 3, 6, 1, 1, 5, 5, 5, 5, 4, 1, 1, 4, 1, 2, 3, 1, 3, 1, 3, 1, 3, 3, 3, 3, 2, 2, 1, 2, 2, 1, 1, 1, 1, 1, 1, 1, 1, 1, 1, 1, 1, 1, 1, 1, 1, 1, 1, 1, 1

Corresponding number of selected TFs each gene is involved with:
(In the same order as above)
9, 9, 9, 10, 7, 8, 6, 7, 9, 5, 7, 3, 5, 8, 6, 10, 2, 3, 6, 4, 4, 5, 6, 2, 1, 1, 1, 10, 1, 9, 9, 8, 2, 4, 4, 2, 2, 8, 1, 8, 8, 1, 1, 7, 1, 2, 3, 2, 2, 1, 5, 5, 1, 1, 1, 1, 1, 4, 4, 1, 4, 2, 1, 3, 1, 3, 1, 3, 1, 1, 1, 1, 1, 1, 2, 1, 1, 2, 1, 1, 1, 1, 1, 1, 1, 1, 1, 1, 1, 1, 1, 1, 1, 1, 1, 1, 1

Phenopedia diseases ranked according to mean of effect sizes in cluster:
Hyperlipidemia, Familial Combined | Lipid Metabolism, Inborn Errors | Lipid Metabolism Disorders | Metabolism, Inborn Errors | Cholecystolithiasis | Gallstones | Cholelithiasis | Gallbladder Diseases | Hyperlipoproteinemias | Dyslipidemias | Diseases in Twins | Hyperlipoproteinemia Type II | Hyperlipidemias | Hypertriglyceridemia

Corresponding mean of effect sizes of each Phenopedia disease:
(In the same order as above)
3.88, 2.94, 2.76, 2.50, 2.43, 2.36, 2.30, 2.21, 2.14, 2.01, 1.93, 1.87, 1.84, 1.83

Corresponding total number of genes of each Phenopedia disease:
(In the same order as above)
23, 104, 264, 289, 47, 80, 49, 58, 67, 231, 73, 54, 204, 104

Corresponding number of genes of each Phenopedia disease with at least one TF hit (of selected

TFs):
(In the same order as above)
10, 29, 56, 61, 8, 17, 8, 12, 16, 45, 19, 11, 42, 24

Corresponding total number of TF hits (of selected TFs) for each Phenopedia disease (in all genes):
(In the same order as above)
48, 107, 199, 205, 47, 77, 47, 60, 55, 148, 92, 45, 136, 81

Corresponding number of selected TFs each Phenopedia disease is involved with:
(In the same order as above)
10, 10, 10, 10, 10, 10, 10, 10, 10, 10, 10, 10, 10, 10

TFs ranked according to mean of effect sizes in cluster:
V$COUPTF_Q6, V$COUP_DR1_Q6, V$HNF4_DR1_Q3, V$COUP_01, V$HNF4_Q6_01, V$DR1_Q3, V$HNF4_01, V$HNF4ALPHA_Q6, V$PPAR_DR1_Q2, V$PPARG_01

Corresponding mean of effect sizes of each TF:
(In the same order as above)
3.83, 2.71, 2.51, 2.39, 2.37, 2.33, 2.31, 2.15, 1.66, 1.33

Corresponding total number of TF hits for each TF (genome-wide):
(In the same order as above)
426, 438, 430, 442, 444, 438, 438, 450, 456, 452

Corresponding total number of TF hits for each TF (in all genes in selected Phenopedia diseases):
(In the same order as above)
160, 132, 145, 141, 153, 138, 128, 136, 113, 101

Corresponding number of genes (of selected Phenopedia diseases) each TF is involved with:
(In the same order as above)
35, 31, 32, 35, 36, 35, 29, 32, 28, 29

Corresponding number of selected Phenopedia diseases each TF is involved with:
(In the same order as above)
14, 14, 14, 14, 14, 14, 14, 14, 14, 14

Disease groups of selected Phenopedia diseases ranked according to sum of effect sizes
Signs and Symptoms | Metabolic Diseases | Lipid Metabolism Disorders | Dyslipidemias | Hyperlipidemias | Digestive System Diseases | Biliary Tract Diseases | Metabolism, Inborn Errors | Genetic Diseases, Inborn | Congenital, Hereditary, and Neonatal Diseases and Abnormalities | Pathological Conditions, Anatomical | Gallstones | Calculi | Lipid Metabolism, Inborn Errors | Gallbladder Diseases | Hyperlipoproteinemias | Hyperlipidemia, Familial Combined | Cholecystolithiasis | Cholelithiasis | Diseases in Twins | Disease Attributes | Hyperlipoproteinemia Type II | Hypertriglyceridemia

Corresponding sum of effect sizes for each disease group of selected Phenopedia diseases
(In the same order as above)
217.75, 217.75, 192.70, 135.70, 115.62, 93.00, 93.00, 73.23, 73.23, 73.23, 70.88, 70.88, 70.88, 48.19, 46.45, 40.16, 38.80, 24.34, 22.97, 19.35, 19.35, 18.75, 18.27

Cluster for columns 228 to 230, rows 44 to 45

Phenopedia diseases

Respiratory Hypersensitivity | Hypersensitivity | Hypersensitivity, Immediate

TFs

V$GCNF_01 | V$STAT5A_02

Information

All related TFs:
(List of all TFs that are related to any of the PWMs)
GCNF, GCNF-1, GCNF-2, STAT5A

Ranked gene list:
(All genes of the selected Phenopedia diseases with hits of any of the selected TFs, ranked according to the total number of TF hits)
RUNX1, MEFV, HDAC7, FCER1A, ELAC2, CYP27A1, CREM, XPNPEP2, XCR1, TNFRSF1B, TLR9, TBX21, STAT5A, SOCS3, SOCS2, SNW1, SH2B3, SFTPC, SFTPA1B, RTEL1, RASGRP4, PTGIR, PIP, PGDS, PARP1, NR0B2, NPPA, NOS3, NOS1, NGFR, NDFIP1, MIF, KCNS3, JUND, IRF9, IRF5, IRF3, IRF1, IL8RB, IL7R, IL2RG, IL28RA, IL21, IL20, IL16, IL15, IFNAR1, IFNA14, HSPA8, HRH3, HLA-DPB1, HLA-DPA1, HDAC3, HDAC2, GZMB, GPR123, FYN, FKBP4, FANK1, ESR1, EHF, EGR1, EDNRB, CYP1A1, CX3CL1, CSF3, CREBL2, COL18A1, CDH1, CD86, CD4, CCR1, BDKRB1, ARRB2, ALOX12, ALDH2, ADCYAP1R1, ADCYAP1, ADCY9, ADAM8, S100A6, S100A5, S100A4, S100A3, S100A2, NLRP3, CCR4, BCL2A1, FANCM, CCKBR

Corresponding total number of TF hits:
(For each gene listed above, the total number of TF hits for any of the selected TFs, multiplied by the number of selected Phenopedia diseases containing that gene)
6, 6, 6, 6, 6, 6, 6, 4, 3, 3, 3, 3, 3, 3, 3, 3, 3, 3, 3, 3, 3, 3, 3, 3, 3, 3, 3, 3, 3, 3, 3, 3, 3, 3, 3, 3, 3, 3, 3, 3, 3, 3, 3, 3, 3, 3, 3, 3, 3, 3, 3, 3, 3, 3, 3, 3, 3, 3, 3, 3, 3, 3, 3, 3, 3, 3, 3, 3, 3, 3, 3, 3, 3, 3, 3, 3, 3, 3, 3, 3, 2, 2, 2, 2, 2, 2, 2, 2, 1, 1

Corresponding number of selected Phenopedia diseases each gene is involved with:
(In the same order as above)
3, 3, 3, 3, 3, 3, 3, 2, 3, 3, 3, 3, 3, 3, 3, 3, 3, 3, 3, 3, 3, 3, 3, 3, 3, 3, 3, 3, 3, 3, 3, 3, 3, 3, 3, 3, 3, 3, 3, 3, 3, 3, 3, 3, 3, 3, 3, 3, 3, 3, 3, 3, 3, 3, 3, 3, 3, 3, 3, 3, 3, 3, 3, 3, 3, 3, 3, 3, 3, 3, 3, 3, 3, 3, 3, 3, 3, 3, 3, 3, 2, 2, 2, 2, 2, 2, 2, 2, 1, 1

Corresponding number of selected TFs each gene is involved with:
(In the same order as above)
2, 2, 2, 2, 2, 2, 2, 2, 1, 1, 1, 1, 1, 1, 1, 1, 1, 1, 1, 1, 1, 1, 1, 1, 1, 1, 1, 1, 1, 1, 1, 1, 1, 1, 1, 1, 1, 1, 1, 1, 1, 1, 1, 1, 1, 1, 1, 1, 1, 1, 1, 1, 1, 1, 1, 1, 1, 1, 1, 1, 1, 1, 1, 1, 1, 1, 1, 1, 1, 1, 1, 1, 1, 1, 1, 1, 1, 1, 1, 1, 1, 1, 1, 1, 1, 1, 1, 1, 1, 1

Phenopedia diseases ranked according to mean of effect sizes in cluster:
Hypersensitivity, Immediate | Hypersensitivity | Respiratory Hypersensitivity

Corresponding mean of effect sizes of each Phenopedia disease:
(In the same order as above)
3.42, 3.29, 3.14

Corresponding total number of genes of each Phenopedia disease:
(In the same order as above)
606, 654, 554

Corresponding number of genes of each Phenopedia disease with at least one TF hit (of selected TFs):
(In the same order as above)

88, 90, 79

Corresponding total number of TF hits (of selected TFs) for each Phenopedia disease (in all genes):
(In the same order as above)
96, 98, 86

Corresponding number of selected TFs each Phenopedia disease is involved with:
(In the same order as above)
2, 2, 2

TFs ranked according to mean of effect sizes in cluster:
V$STAT5A_02, V$GCNF_01

Corresponding mean of effect sizes of each TF:
(In the same order as above)
3.29, 3.27

Corresponding total number of TF hits for each TF (genome-wide):
(In the same order as above)
471, 492

Corresponding total number of TF hits for each TF (in all genes in selected Phenopedia diseases):
(In the same order as above)
139, 141

Corresponding number of genes (of selected Phenopedia diseases) each TF is involved with:
(In the same order as above)
47, 51

Corresponding number of selected Phenopedia diseases each TF is involved with:
(In the same order as above)
3, 3

Disease groups of selected Phenopedia diseases ranked according to sum of effect sizes
Immune System Diseases | Hypersensitivity | Hypersensitivity, Immediate | Respiratory Tract Diseases | Respiratory Hypersensitivity

Corresponding sum of effect sizes for each disease group of selected Phenopedia diseases
(In the same order as above)
19.70, 19.70, 13.12, 6.27, 6.27

Cluster for columns 267 to 277, rows 31 to 41

Phenopedia diseases

Intracranial Thrombosis | Brain Infarction | Cerebral Infarction | Brain Ischemia | Hypoxia-Ischemia, Brain | Coronary Arteriosclerosis | Heart Diseases | Coronary Disease | Ischemia | Myocardial Ischemia | Carotid Artery Diseases

TFs

V$SRF_01 | V$HNF4_01 | V$COUP_01 | V$PPAR_DR1_Q2 | V$DR1_Q3 | V$COUP_DR1_Q6 | V$HNF4ALPHA_Q6 | V$COUPTF_Q6 | V$HNF4_Q6_01 | V$HNF4_DR1_Q3 | V$PPARG_01

Information

All related TFs:
(List of all TFs that are related to any of the PWMs)
COUP, COUP-TF1, COUP-TF2, HNF-4, HNF-4alpha, HNF-4alpha1, HNF-4alpha2, HNF-4alpha3, HNF-4alpha4, HNF-4alpha7, HNF-4gamma, PPAR-alpha, PPAR-alpha:RXR-alpha, PPAR-beta, PPAR-gamma, PPAR-gamma1, PPAR-gamma2, PPAR-gamma2:RXR-alpha, PPAR-gamma:RXR-alpha, RAR-alpha:RXR-alpha, SRF, SRF-L

Ranked gene list:
(All genes of the selected Phenopedia diseases with hits of any of the selected TFs, ranked according to the total number of TF hits)
APOA1, APOC3, APOA4, CYP1A1, SLC39A7, VEGFA, PPARA, COMT, LY6G5B, AGER, LCAT, VEGF, SHBG, RHOA, FADS3, SKIV2L, PRDX5, KIF6, CAPN10, NR1H3, CFB, NOS3, LTC4S, APOM, GCK, TCF7, CTSD, TNFRSF1B, LRP1, VAMP8, LIPE, GCLM, CALM1, TNF, SOD2, PSMB9, PSMB8, LTA, ACAT2, ITGB2, TAP2, HSPD1, ARHGEF2, OAZ1, MTRR, CLEC16A, ADM, GCKR, TNFRSF1A, PLEKHG2, MAPT, LTB4R, ARHGEF1, TP53, TNNC1, F5, CBS, TNFSF4, NDUFAB1, LDLR, INSR, GSTM1, FGB, FABP2, F13A1, ESR1, APOA5, ALOX5AP, TNFRSF4, SPP1, LGALS2, KCNH2, IL1B, CDKN1A, BRAP, UCP3, STK11, SLC2A9, PROZ, MS, MMP26, ITGA3, HNF1A, F12, EPHX2, CYP27B1, CSNK1D, CSF1R, CRNKL1, CHRNA4, CCR5, CCL11, ADRB3, TLR9, THBS1, ALDH2, TIMP2, SH2B3, PSMA6, PON3, PLA2G7, PARP12, MTP18, MMP2, LLGL2, LGALS14, KALRN, IL4R, HSPB6, HPS1, HLA-B, FSTL4, CPT1B, BAT2, AXIN1, AR, ALOX12B, ADRBK1, UCP2, TNXB, THBS2, TCAP, TAF1, SREBF1, SELPLG, RXRA, PROS1, PPARD, NR1H4, MTR, MACROD2, LTB4R2, LTB, LMNA, KCNN4, GSTO1, GALNT2, EDNRB, CD40, CASP1, ALOX12, SCN1B, RHOC, PTPRA, NPC1L1, MYO9B, MYO9A, MYL3, MYD88, KCNJ5, INSIG1, IL18R1, IL18BP, HSPA4, HLA-C, GBE1, GATA4, F2R, CHN2, CAMTA1, BDKRB1, ARHGAP1, ANG, UTS2, TRPM7, SEMA3F, SCGB1A1, PTGER1, P2RY11, MAP2K2, KIAA0391, KCNQ1, KCNIP4, ITIH3, DNAI1, CALM3, CALM2, ADORA3, TRADD, TPM1, SORBS1, SLCO3A1, LDB3, HSD11B1, BAT3, ZP1, WNT11, VCL, TBX1, SRR, SMG6, SCN3B, PTPN22, PLN, PCK1, NOVA1, NNMT, NFATC1, MYLK, MYH7, MYH11, LOXL2, KCNJ2, KCNE4, IL2RA, GRID1, GLI1, FBLN1, ERBB2, DNMT3B, DMPK, DMD, DM1, CYP11A1, CTGF, CRHR1, CPT2, CPT1A, CERKL, CAPG, C6orf204, ADRA1D

Corresponding total number of TF hits:
(For each gene listed above, the total number of TF hits for any of the selected TFs, multiplied by the number of selected Phenopedia diseases containing that gene)
90, 72, 72, 63, 60, 56, 49, 49, 48, 45, 42, 40, 40, 40, 40, 36, 36, 36, 36, 35, 32, 30, 30, 30, 28, 27, 27, 25, 25, 24, 24, 24, 21, 20, 20, 20, 20, 20, 20, 18, 16, 16, 16, 15, 15, 15, 15, 14, 12, 12, 12, 12, 12, 11, 11, 11, 11, 10, 10, 10, 10, 10, 10, 10, 10, 10, 10, 10, 9, 9, 9, 9, 9, 9, 9, 8, 8, 8, 8, 8, 8, 8, 8, 8, 8, 8, 8, 8, 8, 8, 8, 8, 8, 7, 7, 7, 6, 6, 6, 6, 6, 6, 6, 6, 6, 6, 6, 6, 6, 6, 6, 6, 6, 6, 6, 6, 5, 5, 5, 5, 5, 5, 5, 5, 5, 5, 5, 5, 5, 5, 5, 5, 5, 5, 5, 5, 5, 5, 5, 4, 4, 4, 4, 4, 4, 4, 4, 4, 4, 4, 4, 4, 4, 4, 4, 4, 4, 4, 4, 4, 3, 3, 3, 3, 3, 3, 3, 3, 3, 3, 3, 3, 3, 3, 3, 2, 2, 2, 2, 2, 2, 2, 1, 1, 1, 1, 1, 1, 1, 1, 1, 1, 1, 1, 1, 1, 1, 1, 1, 1, 1, 1, 1, 1, 1, 1, 1, 1, 1, 1, 1, 1, 1, 1, 1, 1, 1

Corresponding number of selected Phenopedia diseases each gene is involved with:
(In the same order as above)
10, 8, 8, 7, 6, 7, 7, 7, 6, 5, 7, 5, 5, 4, 4, 4, 4, 6, 6, 5, 4, 10, 6, 6, 4, 3, 3, 5, 5, 6, 4, 8, 7, 10, 5, 4, 4, 10, 5, 9, 4, 4, 4, 5, 5, 3, 3, 7, 6, 4, 6, 3, 4, 11, 1, 11, 11, 10, 5, 10, 5, 10, 10, 10, 10, 10, 10, 10, 3, 9, 9, 1, 9, 3, 3, 8, 1, 4, 4, 8, 4, 8, 4, 8, 8, 1, 4, 1, 1, 1, 8, 8, 8, 1, 7, 7, 6, 3, 6, 6, 6, 3, 2, 6, 3, 6, 3, 6, 2, 6, 6, 6, 1, 6, 1, 6, 6, 2, 5, 5, 5, 1, 5, 5, 5, 5, 5, 5, 5, 5, 5, 1, 5, 5, 5, 5, 5, 5, 5, 5, 5, 1, 4, 4, 4, 4, 4, 1, 4, 1, 4, 4, 4, 4, 4, 4, 4, 4, 4, 4, 4, 4, 1, 3, 3, 3, 3, 1, 3, 1, 3, 3, 3, 3, 1, 1, 1, 3, 1, 1, 2, 1, 1, 1, 1, 1, 1, 1, 1, 1, 1, 1, 1, 1, 1, 1, 1, 1, 1, 1, 1, 1, 1, 1, 1, 1, 1, 1, 1, 1, 1, 1, 1, 1, 1, 1, 1, 1, 1, 1, 1

Corresponding number of selected TFs each gene is involved with:
(In the same order as above)
9, 9, 9, 9, 10, 8, 7, 7, 8, 9, 6, 8, 8, 10, 10, 9, 9, 6, 6, 7, 8, 3, 5, 5, 7, 9, 9, 5, 5, 4, 6, 3, 3, 2, 4, 5, 5, 2, 4, 2, 4, 4, 4, 3, 3, 5, 5, 2, 2, 3, 2, 4, 3, 1, 11, 1, 1, 1, 2, 1, 2, 1, 1, 1, 1, 1, 1, 1, 3, 1, 1, 9, 1, 3, 3, 1, 8, 2, 2, 1, 2, 1, 2,

1, 1, 8, 2, 8, 8, 8, 1, 1, 1, 7, 1, 1, 1, 2, 1, 1, 1, 2, 3, 1, 2, 1, 2, 1, 3, 1, 1, 1, 6, 1, 6, 1, 1, 3, 1, 1, 1, 5, 1, 1, 1, 1, 1, 1, 1, 1, 1, 5, 1, 1, 1, 1, 1, 1, 1, 1, 1, 4, 1, 1, 1, 1, 1, 4, 1, 4, 1, 1, 1, 1, 1, 1, 1, 1, 1, 1, 1, 1, 4, 1, 1, 1, 1, 3, 1, 3, 1, 1, 1, 1, 3, 3, 3, 1, 2, 2, 1, 2, 2, 2, 2, 1, 1, 1, 1, 1, 1, 1, 1, 1, 1, 1, 1, 1, 1, 1, 1, 1, 1, 1, 1, 1, 1, 1, 1, 1, 1, 1, 1, 1, 1, 1, 1, 1, 1, 1

Phenopedia diseases ranked according to mean of effect sizes in cluster:
Coronary Arteriosclerosis | Coronary Disease | Brain Infarction | Myocardial Ischemia | Brain Ischemia | Hypoxia-Ischemia, Brain | Ischemia | Cerebral Infarction | Heart Diseases | Carotid Artery Diseases | Intracranial Thrombosis

Corresponding mean of effect sizes of each Phenopedia disease:
(In the same order as above)
1.94, 1.66, 1.47, 1.34, 1.24, 1.23, 1.20, 1.15, 1.14, 1.04, 0.57

Corresponding total number of genes of each Phenopedia disease:
(In the same order as above)
221, 506, 105, 604, 274, 275, 684, 109, 896, 153, 28

Corresponding number of genes of each Phenopedia disease with at least one TF hit (of selected TFs):
(In the same order as above)
63, 120, 37, 141, 63, 63, 158, 33, 204, 41, 10

Corresponding total number of TF hits (of selected TFs) for each Phenopedia disease (in all genes):
(In the same order as above)
162, 337, 84, 384, 165, 165, 430, 83, 552, 85, 18

Corresponding number of selected TFs each Phenopedia disease is involved with:
(In the same order as above)
11, 11, 11, 11, 11, 11, 11, 11, 11, 11, 11

TFs ranked according to mean of effect sizes in cluster:
VSRF_01, VHNF4_DR1_Q3, V$PPARG_01, V$HNF4_Q6_01, V$HNF4ALPHA_Q6, V$HNF4_01, V$COUP_01, V$DR1_Q3, V$COUP_DR1_Q6, V$COUPTF_Q6, V$PPAR_DR1_Q2

Corresponding mean of effect sizes of each TF:
(In the same order as above)
2.39, 1.63, 1.63, 1.59, 1.58, 1.14, 1.09, 1.00, 0.98, 0.62, 0.32

Corresponding total number of TF hits for each TF (genome-wide):
(In the same order as above)
512, 430, 452, 444, 450, 438, 442, 438, 438, 426, 456

Corresponding total number of TF hits for each TF (in all genes in selected Phenopedia diseases):
(In the same order as above)
298, 250, 209, 263, 244, 213, 220, 221, 190, 168, 189

Corresponding number of genes (of selected Phenopedia diseases) each TF is involved with:
(In the same order as above)
69, 61, 50, 61, 62, 50, 55, 53, 48, 40, 52

Corresponding number of selected Phenopedia diseases each TF is involved with:
(In the same order as above)
11, 11, 11, 11, 11, 11, 11, 11, 11, 11, 11

Disease groups of selected Phenopedia diseases ranked according to sum of effect sizes

Cardiovascular Diseases | Respiratory Tract Diseases | Vascular Diseases | Ischemia | Nervous System Diseases | Cerebrovascular Disorders | Central Nervous System Diseases | Brain Diseases | Thoracic Diseases | Myocardial Ischemia | Mediastinal Diseases | Cardiomyopathies | Necrosis | Infarction | Cerebral Infarction | Brain Infarction | Brain Ischemia | Coronary Arteriosclerosis | Coronary Disease | Signs and Symptoms | Respiration Disorders | Hypoxia-Ischemia, Brain | Hypoxia, Brain | Death | Asphyxia | Anoxia | Heart Diseases | Carotid Artery Diseases | Arteriosclerosis | Arterial Occlusive Diseases | Thrombosis | Thromboembolism | Intracranial Thrombosis | Intracranial Embolism and Thrombosis | Embolism

Corresponding sum of effect sizes for each disease group of selected Phenopedia diseases
(In the same order as above)
77.85, 46.45, 44.93, 40.37, 33.44, 33.44, 33.44, 33.44, 32.92, 32.92, 32.92, 32.92, 28.78, 28.78, 28.78, 28.78, 27.17, 21.34, 18.23, 13.53, 13.53, 13.53, 13.53, 13.53, 13.53, 13.53, 12.59, 11.49, 11.49, 11.49, 6.27, 6.27, 6.27, 6.27, 6.27

Cluster for columns 176 to 179, rows 3 to 5

Phenopedia diseases

Cataract | Nervous System Malformations | Neural Tube Defects | Spinal Dysraphism

TFs

V$LXR_DR4_Q3 | V$DR4_Q2 | V$LXR_Q3

Information

All related TFs:
(List of all TFs that are related to any of the PWMs)
CAR, CAR2:RXR-alpha, CAR:RXR-alpha, COUP, COUP-TF1, COUP-TF2, LXR-alpha, LXR-alpha:RXR-alpha, LXR-beta, LXR-beta:RXR-alpha, PXR-1, PXR-1:RXR-alpha, PXR-1A, PXR-1A:RXR-alpha, PXR-1A:RXR-beta, PXR-2, PXR-2:RXR-alpha, RAR-alpha, RAR-alpha1, RAR-alpha:RXR-alpha, RAR-alpha:RXR-gamma, RAR-beta, RAR-gamma, RXR-alpha, RXR-beta, SXR:RXR-alpha

Ranked gene list:
(All genes of the selected Phenopedia diseases with hits of any of the selected TFs, ranked according to the total number of TF hits)
ERCC2, APOE, CYP26B1, MTHFR, XRCC3, SLC19A1, SHMT2, HLA-DRB5, GAMT, AMT, TSC2, RFC1, PRMT1, NOS3, FPGS, ENG, DNMT1, VSX2, MPZ, LOXL1, LAMB2, HLA-A, EPHB4, CRYAA, BSCL2, AKR1B1, SPAST, SIX6, NDRG1, L1CAM, HLA-B, HESX1

Corresponding total number of TF hits:
(For each gene listed above, the total number of TF hits for any of the selected TFs, multiplied by the number of selected Phenopedia diseases containing that gene)
12, 12, 9, 8, 6, 6, 6, 6, 6, 6, 3, 3, 3, 3, 3, 3, 3, 2, 2, 2, 2, 2, 2, 2, 2, 2, 1, 1, 1, 1, 1, 1

Corresponding number of selected Phenopedia diseases each gene is involved with:
(In the same order as above)
4, 4, 3, 4, 3, 3, 2, 3, 3, 3, 1, 3, 3, 3, 3, 1, 3, 1, 1, 1, 1, 1, 1, 1, 1, 1, 1, 1, 1, 1, 1, 1

Corresponding number of selected TFs each gene is involved with:
(In the same order as above)

3, 3, 3, 2, 2, 2, 3, 2, 2, 2, 3, 1, 1, 1, 1, 3, 1, 2, 2, 2, 2, 2, 2, 2, 2, 2, 1, 1, 1, 1, 1, 1

Phenopedia diseases ranked according to mean of effect sizes in cluster:
Nervous System Malformations | Cataract | Spinal Dysraphism | Neural Tube Defects

Corresponding mean of effect sizes of each Phenopedia disease:
(In the same order as above)
3.18, 3.13, 2.47, 2.15

Corresponding total number of genes of each Phenopedia disease:
(In the same order as above)
146, 47, 77, 97

Corresponding number of genes of each Phenopedia disease with at least one TF hit (of selected TFs):
(In the same order as above)
26, 9, 14, 15

Corresponding total number of TF hits (of selected TFs) for each Phenopedia disease (in all genes):
(In the same order as above)
48, 19, 26, 29

Corresponding number of selected TFs each Phenopedia disease is involved with:
(In the same order as above)
3, 3, 3, 3

TFs ranked according to mean of effect sizes in cluster:
VLXR_DR4_Q3, VLXR_Q3, V$DR4_Q2

Corresponding mean of effect sizes of each TF:
(In the same order as above)
4.43, 1.95, 1.82

Corresponding total number of TF hits for each TF (genome-wide):
(In the same order as above)
436, 439, 451

Corresponding total number of TF hits for each TF (in all genes in selected Phenopedia diseases):
(In the same order as above)
53, 36, 33

Corresponding number of genes (of selected Phenopedia diseases) each TF is involved with:
(In the same order as above)
25, 19, 15

Corresponding number of selected Phenopedia diseases each TF is involved with:
(In the same order as above)
4, 4, 4

Disease groups of selected Phenopedia diseases ranked according to sum of effect sizes
Signs and Symptoms | Nervous System Malformations | Congenital, Hereditary, and Neonatal Diseases and Abnormalities | Spinal Dysraphism | Spinal Diseases | Spinal Cord Diseases | Neural Tube Defects | Nervous System Diseases | Musculoskeletal Diseases | Central Nervous System Diseases | Bone Diseases | Lens Diseases | Eye Diseases | Cataract

Corresponding sum of effect sizes for each disease group of selected Phenopedia diseases

(In the same order as above)
23.41, 23.41, 23.41, 13.85, 13.85, 13.85, 13.85, 13.85, 13.85, 13.85, 13.85, 9.40, 9.40, 9.40

Cluster for columns 454 to 463, rows 6 to 9

Phenopedia diseases

Stomatitis | Epidermal Necrolysis, Toxic | Stevens-Johnson Syndrome | Erythema | Erythema Nodosum | Drug Eruptions | Drug Hypersensitivity | Encephalitis | Severe Acute Respiratory Syndrome | Meningitis

TFs

V$PBX1_02 | V$HSF1_Q6 | V$NFY_C | V$NFKAPPAB50_01

Information

All related TFs:
(List of all TFs that are related to any of the PWMs)
CBF(2), CBF-A, CBF-B, HSF1, HSF1-L, HSF1-S, HSF1long, HSF1short, NF-Y, NF-YA, NF-YB, Pbx1a, p50

Ranked gene list:
(All genes of the selected Phenopedia diseases with hits of any of the selected TFs, ranked according to the total number of TF hits)
HLA-B, HLA-A, HLA-C, HSPA1L, FASLG, HLA-DQB1, IL10, NFKBIZ, CYP2B6, TNFAIP1, SELE, IFNG, CTLA4, BDNF, GSTM1, FCGR2A, CXCL9, CD40, TNFRSF1B, TLR1, OAS1, MVK, TBXA2R, PTGS2, LACTB, IRF1, HMOX1, HLA-DRA, F5, CXCL10, ALOXE3, ALOX12B, ADORA2A, ADD1

Corresponding total number of TF hits:
(For each gene listed above, the total number of TF hits for any of the selected TFs, multiplied by the number of selected Phenopedia diseases containing that gene)
40, 30, 28, 12, 12, 9, 8, 6, 6, 4, 4, 4, 4, 4, 3, 3, 3, 3, 2, 2, 2, 2, 1, 1, 1, 1, 1, 1, 1, 1, 1, 1, 1, 1

Corresponding number of selected Phenopedia diseases each gene is involved with:
(In the same order as above)
10, 10, 7, 6, 6, 9, 8, 6, 6, 2, 4, 4, 4, 2, 3, 3, 1, 3, 2, 2, 2, 1, 1, 1, 1, 1, 1, 1, 1, 1, 1, 1, 1, 1

Corresponding number of selected TFs each gene is involved with:
(In the same order as above)
4, 3, 4, 2, 2, 1, 1, 1, 1, 2, 1, 1, 1, 2, 1, 1, 3, 1, 1, 1, 1, 2, 1, 1, 1, 1, 1, 1, 1, 1, 1, 1, 1, 1

Phenopedia diseases ranked according to mean of effect sizes in cluster:
Severe Acute Respiratory Syndrome | Stevens-Johnson Syndrome | Epidermal Necrolysis, Toxic | Stomatitis | Meningitis | Drug Eruptions | Encephalitis | Erythema | Drug Hypersensitivity | Erythema Nodosum

Corresponding mean of effect sizes of each Phenopedia disease:
(In the same order as above)
2.67, 2.53, 2.53, 1.78, 1.76, 1.58, 1.17, 1.04, 1.00, 0.75

Corresponding total number of genes of each Phenopedia disease:
(In the same order as above)
37, 21, 20, 36, 37, 52, 42, 67, 84, 38

Corresponding number of genes of each Phenopedia disease with at least one TF hit (of selected TFs):
(In the same order as above)
10, 8, 8, 10, 8, 13, 9, 18, 19, 10

Corresponding total number of TF hits (of selected TFs) for each Phenopedia disease (in all genes):
(In the same order as above)
17, 18, 18, 21, 14, 23, 15, 29, 29, 19

Corresponding number of selected TFs each Phenopedia disease is involved with:
(In the same order as above)
4, 4, 4, 4, 4, 4, 4, 4, 4, 4

TFs ranked according to mean of effect sizes in cluster:
V$PBX1_02, V$HSF1_Q6, VNFY_C, VNFKAPPAB50_01

Corresponding mean of effect sizes of each TF:
(In the same order as above)
2.20, 1.89, 1.44, 1.18

Corresponding total number of TF hits for each TF (genome-wide):
(In the same order as above)
507, 499, 440, 468

Corresponding total number of TF hits for each TF (in all genes in selected Phenopedia diseases):
(In the same order as above)
64, 56, 42, 41

Corresponding number of genes (of selected Phenopedia diseases) each TF is involved with:
(In the same order as above)
16, 12, 8, 13

Corresponding number of selected Phenopedia diseases each TF is involved with:
(In the same order as above)
10, 10, 10, 10

Disease groups of selected Phenopedia diseases ranked according to sum of effect sizes
Skin Diseases | Exanthema | Erythema | Signs and Symptoms | Nervous System Diseases | Central Nervous System Diseases | Virus Diseases | Severe Acute Respiratory Syndrome | Coronavirus Infections | Coronaviridae Infections | Communicable Diseases | Poisoning | Drug Toxicity | Stevens-Johnson Syndrome | Erythema Multiforme | Epidermal Necrolysis, Toxic | Stomatitis | Mucositis | Inflammation | Meningitis | Central Nervous System Infections | Drug Eruptions | Dermatitis | Encephalitis | Brain Diseases | Immune System Diseases | Hypersensitivity | Drug Hypersensitivity | Erythema Nodosum

Corresponding sum of effect sizes for each disease group of selected Phenopedia diseases
(In the same order as above)
33.73, 33.73, 27.42, 20.97, 11.69, 11.69, 10.66, 10.66, 10.66, 10.66, 10.66, 10.31, 10.31, 10.13, 10.13, 10.13, 7.11, 7.11, 7.11, 7.02, 7.02, 6.31, 6.31, 4.67, 4.67, 4.00, 4.00, 4.00, 2.99

Cluster for columns 412 to 451, rows 110 to 126

Phenopedia diseases

Arthralgia | Stomach Ulcer | Enterocolitis | Idiopathic Pulmonary Fibrosis | Pulmonary Fibrosis |

Lipodystrophy | Shock | Viremia | Sepsis | Systemic Inflammatory Response Syndrome | Rubella | Measles | Mumps | Tuberculosis | Mycobacterium Infections | Tuberculosis, Pulmonary | Bacterial Infections | Gram-Negative Bacterial Infections | Malaria | Parasitic Diseases | Bronchiectasis | Helminthiasis | Brucellosis | Coronary Aneurysm | Salmonella Infections | Shock, Septic | Myelitis | Deltaretrovirus Infections | HTLV-I Infections | Spinal Diseases | Spondylitis | Ankylosis | Spondylitis, Ankylosing | Spondylarthritis | Spondylarthropathies | Vasculitis | Uveitis | Behcet Syndrome | Panuveitis | Uveitis, Anterior

TFs

V$PR_Q2 | V$GR_Q6_01 | V$CEBP_01 | V$AP3_Q6 | V$PU1_Q6 | V$PEA3_Q6 | V$ETS_Q6 | V$TEF1_Q6 | V$STAT3_02 | V$STAT1_02 | V$GATA2_01 | V$TFIII_Q6 | V$MZF1_01 | V$STAT6_02 | V$GATA3_01 | V$GATA4_Q3 | V$AML1_Q6

Information

All related TFs:
(List of all TFs that are related to any of the PWMs)
AML1, AP-3, C/EBP, C/EBPalpha, ELF-1, ELFR, Elf-1, Elk-1, Elk-1-isoform1, Erg-1, Erg-2, Ets-1, Fli-1, GABP-alpha, GABP-alpha:GABP-beta, GABP-beta1, GABP-beta2, GATA-2, GATA-3, GATA-4, GR, GR-alpha, GR-beta, MZF1B-C, NERF-1a, NERF-1b, NERF-2, Net, PEA3, PR, PR-alpha, PR-beta, PU.1, PU.1-xbb1, SAP-1a, SAP-1b, STAT1, STAT1alpha, STAT3, STAT6, Spi-B, TCF, TEF-1, TEL1, TFII-I, Tel-2a, Tel-2b, Tel-2c, Tel-2d, Tel-2e, Tel-2f, c-Ets-1, c-Ets-1A, c-Ets-1B, c-Ets-2, c-Ets-2A, c-Ets-2B, p38erg, p49erg, p55, p55erg

Ranked gene list:
(All genes of the selected Phenopedia diseases with hits of any of the selected TFs, ranked according to the total number of TF hits)
TNF, LTA, TNFRSF1A, VEGFA, TAP1, TAP2, TGFB1, IRF1, VEGF, TLR9, PSMB9, CISH, PSMB8, LST1, TNFRSF1B, TRADD, NOS3, DDAH2, PSTPIP1, LTB, CD4, NOTCH4, VIPR1, BAT3, IL10, TCF7, COL11A2, STAT5A, CXCR4, SP110, PGC, GNAS, NR3C1, TPI1, STAT1, PDCD1, HLA-DPB1, IL6R, COL6A1, NLRP3, CTLA4, VDR, LTC4S, SH2B3, RXRB, RARG, RARA, NFKBIE, RUNX3, IL4R, CDK6, AKT1, AGER, CYP27B1, SOCS1, NFKBIA, MMP9, STAT6, RUNX1, NCF4, LMNA, CLEC16A, MMP11, ITGB2, IL11RA, TRAF1, MYD88, LPIN1, IL7R, HSPA1L, KLF12, SIGIRR, MUC1, IL2RB, IL10RA, TNFAIP3, PNMT, CYBA, BAT1, HLA-DPA1, DDR1, NR3C2, MBP, IL2RA, IGF1R, HRAS, UCP2, BAT2, WISP1, TNFSF13B, SFTPC, SDHC, PTPN1, PRKCQ, PRF1, PAX2, NFKB2, ITPR3, EGR2, CSF2, CRABP2, COL1A1, CHST11, CEACAM1, BCL2, ATP6V1G2, AKT2, AIF1, ZFHX3, TRIM5, TRIM22, TOP2B, TBX6, SNAPC4, SERPINH1, ITPKC, HSPA8, FOXP3, COL9A3, CES2, CD226, CASP3, BAT5, AANAT

Corresponding total number of TF hits:
(For each gene listed above, the total number of TF hits for any of the selected TFs, multiplied by the number of selected Phenopedia diseases containing that gene)
680, 510, 448, 299, 288, 264, 232, 204, 195, 180, 135, 119, 105, 96, 81, 72, 70, 68, 56, 51, 45, 44, 42, 40, 38, 34, 32, 30, 30, 28, 27, 26, 25, 24, 24, 24, 23, 21, 21, 20, 20, 19, 18, 17, 17, 17, 17, 17, 16, 16, 16, 16, 16, 15, 14, 14, 14, 12, 12, 12, 12, 12, 11, 11, 11, 10, 10, 10, 10, 10, 9, 8, 8, 8, 8, 6, 6, 6, 6, 5, 5, 4, 4, 4, 4, 4, 3, 3, 2, 2, 2, 2, 2, 2, 2, 2, 2, 2, 2, 2, 2, 2, 2, 2, 2, 2, 2, 2, 1, 1, 1, 1, 1, 1, 1, 1, 1, 1, 1, 1, 1, 1, 1, 1

Corresponding number of selected Phenopedia diseases each gene is involved with:
(In the same order as above)
40, 30, 28, 23, 24, 24, 29, 12, 15, 15, 9, 7, 7, 6, 27, 6, 14, 4, 8, 3, 3, 4, 6, 5, 38, 2, 2, 3, 3, 7, 3, 2, 5, 2, 8, 8, 23, 7, 7, 10, 20, 19, 3, 1, 1, 1, 1, 1, 2, 16, 2, 1, 1, 5, 1, 7, 14, 2, 3, 4, 2, 1, 1, 1, 1, 5, 2, 1, 2, 10, 1, 1, 2, 8, 8, 3, 1, 6, 2, 5, 1, 1, 4, 4, 4, 2, 3, 3, 2, 2, 2, 2, 2, 1, 2, 1, 1, 1, 1, 2, 1, 2, 1, 2, 2, 2, 1, 2, 1, 1, 1, 1, 1, 1, 1, 1, 1, 1, 1, 1, 1, 1, 1, 1

Corresponding number of selected TFs each gene is involved with:
(In the same order as above)
17, 17, 16, 13, 12, 11, 8, 17, 13, 12, 15, 17, 15, 16, 3, 12, 5, 17, 7, 17, 15, 11, 7, 8, 1, 17, 16, 10, 10, 4, 9, 13, 5, 12, 3, 3, 1, 3, 3, 2, 1, 1, 6, 17, 17, 17, 17, 17, 8, 1, 8, 16, 16, 3, 14, 2, 1, 6, 4, 3, 6, 12, 11, 11, 11, 2, 5, 10, 5, 1, 9, 8, 4, 1, 1, 2, 6, 1, 3, 1, 5, 4, 1, 1, 1, 2, 1, 1, 1, 1, 1, 1, 1, 2, 1, 2, 2, 2, 2, 1, 2, 1, 2, 1, 1, 1, 2, 1, 1, 1, 1, 1, 1, 1, 1, 1, 1, 1, 1, 1, 1, 1, 1

Phenopedia diseases ranked according to mean of effect sizes in cluster:
Helminthiasis | Arthralgia | Myelitis | Shock, Septic | Behcet Syndrome | Parasitic Diseases | Uveitis, Anterior | Panuveitis | Stomach Ulcer | Uveitis | Measles | Enterocolitis | Vasculitis | HTLV-I Infections | Mumps | Rubella | Spondylitis, Ankylosing | Mycobacterium Infections | Ankylosis | Brucellosis | Malaria | Deltaretrovirus Infections | Systemic Inflammatory Response Syndrome | Lipodystrophy | Bronchiectasis | Salmonella Infections | Tuberculosis, Pulmonary | Spondylarthropathies | Shock | Tuberculosis | Spondylarthritis | Idiopathic Pulmonary Fibrosis | Sepsis | Viremia | Spondylitis | Coronary Aneurysm | Pulmonary Fibrosis | Bacterial Infections | Gram-Negative Bacterial Infections | Spinal Diseases

Corresponding mean of effect sizes of each Phenopedia disease:
(In the same order as above)
1.86, 1.74, 1.63, 1.63, 1.58, 1.57, 1.57, 1.54, 1.54, 1.47, 1.34, 1.32, 1.30, 1.27, 1.25, 1.23, 1.21, 1.20, 1.20, 1.19, 1.17, 1.16, 1.15, 1.15, 1.10, 1.09, 1.06, 1.05, 1.04, 1.03, 1.02, 1.01, 0.90, 0.90, 0.87, 0.78, 0.72, 0.68, 0.64, 0.53

Corresponding total number of genes of each Phenopedia disease:
(In the same order as above)
34, 21, 22, 26, 95, 144, 99, 102, 32, 110, 46, 19, 210, 29, 32, 48, 72, 142, 73, 21, 139, 30, 149, 23, 36, 25, 93, 106, 112, 159, 111, 36, 185, 58, 99, 21, 61, 300, 197, 176

Corresponding number of genes of each Phenopedia disease with at least one TF hit (of selected TFs):
(In the same order as above)
10, 6, 10, 6, 18, 28, 19, 19, 7, 22, 15, 7, 48, 11, 12, 18, 18, 21, 18, 7, 23, 12, 25, 8, 5, 6, 18, 25, 20, 26, 25, 10, 27, 9, 26, 5, 15, 47, 31, 34

Corresponding total number of TF hits (of selected TFs) for each Phenopedia disease (in all genes):
(In the same order as above)
87, 72, 83, 71, 160, 231, 161, 161, 66, 168, 89, 64, 306, 84, 66, 104, 152, 150, 152, 79, 195, 85, 193, 60, 58, 55, 114, 187, 155, 179, 187, 73, 199, 73, 183, 31, 93, 298, 194, 212

Corresponding number of selected TFs each Phenopedia disease is involved with:
(In the same order as above)
17, 17, 17, 17, 17, 17, 17, 17, 17, 17, 17, 17, 17, 17, 17, 17, 17, 17, 17, 17, 17, 17, 17, 17, 17, 17, 17, 17, 17, 17, 17, 17, 17, 17, 17, 17, 17, 17, 17, 17

TFs ranked according to mean of effect sizes in cluster:
V$GATA4_Q3, V$STAT6_02, VPR_Q2, VPU1_Q6, VGR_Q6_01, VTEF1_Q6, V$AML1_Q6, V$AP3_Q6, V$PEA3_Q6, V$STAT1_02, V$GATA3_01, V$TFIII_Q6, V$CEBP_01, V$STAT3_02, V$GATA2_01, V$ETS_Q6, V$MZF1_01

Corresponding mean of effect sizes of each TF:
(In the same order as above)
1.57, 1.56, 1.51, 1.42, 1.40, 1.40, 1.28, 1.16, 1.13, 1.12, 1.11, 1.05, 1.04, 0.95, 0.90, 0.88, 0.79

Corresponding total number of TF hits for each TF (genome-wide):
(In the same order as above)
525, 468, 457, 460, 484, 463, 471, 479, 438, 422, 457, 458, 508, 434, 423, 466, 435

Corresponding total number of TF hits for each TF (in all genes in selected Phenopedia diseases):
(In the same order as above)
378, 376, 350, 324, 357, 343, 341, 326, 277, 289, 311, 281, 323, 286, 255, 263, 250

Corresponding number of genes (of selected Phenopedia diseases) each TF is involved with:
(In the same order as above)
51, 50, 48, 46, 50, 45, 50, 49, 38, 43, 42, 44, 51, 39, 34, 42, 36

Corresponding number of selected Phenopedia diseases each TF is involved with:
(In the same order as above)
40, 40, 40, 40, 40, 40, 40, 40, 40, 40, 40, 40, 40, 40, 40, 40, 40

Disease groups of selected Phenopedia diseases ranked according to sum of effect sizes
Signs and Symptoms | Communicable Diseases | Musculoskeletal Diseases | Bacterial Infections | Virus Diseases | Vascular Diseases | Cardiovascular Diseases | Spinal Diseases | Bone Diseases | Uveitis | Uveal Diseases | Eye Diseases | Endophthalmitis | Gram-Positive Bacterial Infections | Thoracic Diseases | Respiratory Tract Diseases | Infection | Gram-Negative Bacterial Infections | Spondylarthropathies | Parasitic Diseases | Mycobacterium Infections | Actinomycetales Infections | Uveitis, Anterior | Spondylarthritis | Arthritis | Systemic Inflammatory Response Syndrome | Sepsis | Vasculitis | Stomach Diseases | Gastrointestinal Diseases | Digestive System Diseases | Stomatognathic Diseases | Mouth Diseases | Jaw Diseases | Pneumonia | Lung Diseases | Skin Diseases | Shock | Paramyxoviridae Infections | Retroviridae Infections | Deltaretrovirus Infections | Tuberculosis | Spondylitis | Osteitis | Helminthiasis | Neurologic Manifestations | Arthralgia | Pulmonary Fibrosis | Lung Diseases, Interstitial | Connective Tissue Diseases | Spinal Cord Diseases | Nervous System Diseases | Myelitis | Central Nervous System Diseases | Staphylococcal Infections | Soft Tissue Infections | Shock, Septic | Endotoxemia | Cardiovascular Infections | Skin Diseases, Vascular | Peripheral Vascular Diseases | Behcet Syndrome | Panuveitis | Stomach Ulcer | Morbillivirus Infections | Measles | Intestinal Diseases | Gastroenteritis | Gastritis | Enterocolitis | Enteritis | Colonic Diseases | Colitis | HTLV-I Infections | Sialadenitis | Salivary Gland Diseases | Rubulavirus Infections | Parotitis | Mumps | Togaviridae Infections | Rubella | Spondylitis, Ankylosing | Ankylosis | Brucellosis | Protozoan Infections | Malaria | Coccidiosis | Skin Diseases, Metabolic | Metabolic Diseases | Lipodystrophy | Lipid Metabolism Disorders | Bronchiectasis | Bronchial Diseases | Salmonella Infections | Enterobacteriaceae Infections | Tuberculosis, Pulmonary | Idiopathic Pulmonary Fibrosis | Viremia | Mediastinal Diseases | Coronary Artery Disease | Coronary Aneurysm | Arteriosclerosis | Arterial Occlusive Diseases

Corresponding sum of effect sizes for each disease group of selected Phenopedia diseases
(In the same order as above)
393.77, 344.60, 205.51, 144.76, 121.64, 107.78, 107.78, 107.37, 107.37, 104.66, 104.66, 104.66, 104.66, 83.56, 79.27, 79.27, 77.80, 77.42, 70.57, 58.35, 55.83, 55.83, 53.47, 52.67, 52.67, 50.08, 50.08, 48.99, 48.56, 48.56, 48.56, 48.13, 48.13, 48.13, 47.29, 47.29, 46.32, 45.45, 44.12, 41.37, 41.37, 35.46, 35.33, 35.33, 31.61, 29.66, 29.66, 29.30, 29.30, 29.30, 27.75, 27.75, 27.75, 27.75, 27.72, 27.72, 27.72, 27.72, 27.72, 26.81, 26.81, 26.81, 26.22, 26.17, 22.80, 22.80, 22.39, 22.39, 22.39, 22.39, 22.39, 22.39, 22.39, 21.67, 21.32, 21.32, 21.32, 21.32, 21.32, 20.91, 20.91, 20.55, 20.34, 20.20, 19.85, 19.85, 19.85, 19.51, 19.51, 19.51, 19.51, 18.64, 18.64, 18.53, 18.53, 17.99, 17.09, 15.24, 13.34, 13.34, 13.34, 13.34, 13.34

Cluster for columns 227 to 252, rows 212 to 216

Phenopedia diseases

Asthma | Respiratory Hypersensitivity | Hypersensitivity | Hypersensitivity, Immediate | Infant, Newborn, Diseases | Infant, Premature, Diseases | Respiratory Tract Infections | Airway Remodeling | Paramyxoviridae Infections | Respiratory Syncytial Virus Infections | Bronchitis | Bronchiolitis | Bronchiolitis, Viral | Dengue | Dengue Hemorrhagic Fever | Fever | Q Fever | Cystic Fibrosis | Fibrosis | Infection | Hepatitis, Chronic | Hepatitis C, Chronic | Liver Diseases | Flaviviridae Infections | Hepatitis,

Viral, Human | Necrosis

TFs

V$IRF7_01 | V$CDPCR3_01 | V$IRF_Q6_01 | V$IRF_Q6 | V$ICSBP_Q6

Information

All related TFs:
(List of all TFs that are related to any of the PWMs)
CUTL1, IRF-1, IRF-10, IRF-2, IRF-3, IRF-4, IRF-5, IRF-6, IRF-7, IRF-7A, IRF-7H, IRF-8, IRF-9, ISGF-3

Ranked gene list:
(All genes of the selected Phenopedia diseases with hits of any of the selected TFs, ranked according to the total number of TF hits)
TAP2, TAP1, STAT1, PSMB8, TLR3, IRF5, NR3C1, HLA-A, VDR, CTLA4, TLR4, CCR5, IRF1, IL15RA, IL27, PSMB9, HLA-B, NOD2, SOCS1, NFKBIA, MX1, IL18R1, STAT5A, IL12RB1, HLA-C, CASP1, IL15, IFI44, EIF2AK2, OAS3, IL7, GATA3, IL6, IL2, IL1R1, IFNAR1, CCR2, PON1, FAS, CCL2, NOD1, NLRP3, IL7R, HSPA8, CARD4, ALOX5AP, IFNB1, HLA-DRB1, EDN1, IL19, IFNGR1, TLR8, TBX21, IKBKB, CXCL10, STAT3, IL2RB, CD40, STAT6, RNASEL, RARRES3, ITGB7, GNAS, FYN, FOXP3, FANK1, DHX58, CARD15, APOA5, TNFSF13B, TLR1, SP110, LY86, IL6R, EGR1, PTGER4, OAS2, NFKB1, ITGB2, IL5RA, IL3, IL13RA2, GZMA, FCER2, DDX58, CDKN2A, ZEB2, VEGFA, SOD2, MAP3K5, LCAT, ISG15, GRK5, CXCR4, CIITA, PTPN22, MYD88, MEFV, IRF7, TRAF5, OASL, LAG3, IGF1, ADAR, VEGF, TBX1, CCND1, BIRC3, TNFAIP3, TBK1, SIGIRR, SH2B3, RUNX1, RAPGEF3, PIK3CG, PIK3CA, PDGFA, PCAF, ORMDL3, NDFIP1, MX2, ITPR2, IRS1, IKBKE, ICOS, ICAM5, ICAM4, HAT1, GSDML, GSDMB, DDR1, CYP2J2, CFLAR, CD4, TFRC, SERPINA6, IL28B, DDX5, TNFSF15, TNFRSF18, STX11, PTCH1, IRS2, IL1RAP, GHRL, FOXJ1, CLOCK, CDKN1B, BAT1, ATM, ACP1, TNFSF10, TIMP3, TIMD4, SCNN1A, OGG1, NPPA, LMAN1, GPX4, FKBP5, CYP27B1, CX3CL1, CD27, CASP5, ZPBP2, TRAF1, TLE4, TAPBP, SAP30L, S100A10, RAC2, PYCARD, PPP1R3F, PDCD1, PCNA, NRP1, MYB, MT2A, LRP8, ITPR1, HSP90AB1, HLA-DMA, HDAC7, GSTO2, GRAP2, FGL2, F13A1, EZR, ERAP1, EHF, DCLRE1C, CYFIP2, CTSS, CSF2RB, CREM, CREB1, CKM, BCL6, B2M, ARRB2, APEX1, ADORA3, ADAM23, ZNRD1, USF1, UCP2, TBX5, SERPINB3, RNF39, PNPLA3, NFKBIZ, NFKBIE, MAP3K7IP1, LMNA, IFIH1, HSD11B2, HIF1A, GART, ERAP2, EIF2S1, CCR4, CARD8, CAPN10, ALOXE3, ABCA1, UNC13D, TNFSF11, TNFRSF7, TIMP4, TICAM2, SOD1, S100A6, S100A5, S100A2, RUNX3, PSORS1, P2RX7, OPTN, MT1X, MMP17, MCL1, MAP3K14, MAP3K1, LOR, IL22RA1, IL21R, HRAS, GBP2, GBA, FRMD7, DUSP1, DDAH2, CES2, CD274, CCL21, CALCA, BCL2A1, BAT5, BAT2, ATP7B, APOBEC3B, YPEL2, XRCC5, TRIM5, TRIM22, TNFRSF13C, TCF7, TBPL1, TAC3, STCH, SMAD6, SLC23A1, RXRB, RIPK1, REV3L, RELB, REL, PROX1, PRKCQ, PPP1R14C, PDCD1LG2, PAX2, P2RY12, NR1H2, NEUROD1, MAL, JMJD1C, IL11RA, HMBS, HIST1H2BJ, GHSR, GCK, FZD5, FLJ78302, FECH, DLEC1, CXADR, CSNK1D, CEACAM6, CCL3, CASP9, CAMK2D, BAT4, ANXA6, AIF1, AGFG1, ADIPOR1, ACP5

Corresponding total number of TF hits:
(For each gene listed above, the total number of TF hits for any of the selected TFs, multiplied by the number of selected Phenopedia diseases containing that gene)
130, 130, 100, 95, 92, 76, 68, 68, 50, 50, 48, 48, 46, 45, 42, 40, 40, 38, 36, 36, 36, 36, 34, 34, 34, 34, 32, 32, 30, 28, 28, 28, 26, 24, 24, 24, 23, 22, 22, 22, 21, 21, 21, 21, 21, 21, 20, 20, 20, 19, 19, 18, 18, 18, 18, 17, 17, 17, 16, 16, 16, 16, 16, 16, 16, 16, 16, 16, 15, 15, 15, 15, 15, 15, 14, 14, 14, 14, 14, 14, 14, 14, 14, 14, 12, 12, 12, 12, 12, 12, 12, 12, 12, 11, 11, 11, 11, 10, 10, 10, 10, 10, 9, 9, 9, 9, 8, 8, 8, 8, 8, 8, 8, 8, 8, 8, 8, 8, 8, 8, 8, 8, 8, 8, 8, 8, 8, 8, 8, 8, 8, 8, 7, 7, 7, 7, 6, 6, 6, 6, 6, 6, 6, 6, 6, 6, 6, 6, 5, 5, 5, 5, 5, 5, 5, 5, 5, 5, 5, 5, 5, 4, 4, 4, 4, 4, 4, 4, 4, 4, 4, 4, 4, 4, 4, 4, 4, 4, 4, 4, 4, 4, 4, 4, 4, 4, 4, 4, 4, 4, 4, 4, 4, 4, 4, 4, 4, 4, 3, 3, 3, 3, 3, 3, 3, 3, 3, 3, 3, 3, 3, 3, 3, 3, 3, 3, 3, 3, 3, 3, 2, 2, 2, 2, 2, 2, 2, 2, 2, 2, 2, 2, 2, 2, 2, 2,

2, 2, 2, 2, 2, 2, 2, 2, 2, 2, 2, 2, 2, 2, 2, 2, 2, 2, 2, 2, 1, 1, 1, 1, 1, 1, 1, 1, 1, 1, 1, 1, 1, 1, 1, 1, 1, 1, 1, 1, 1, 1, 1, 1, 1, 1, 1, 1, 1, 1, 1, 1, 1, 1, 1, 1, 1, 1, 1, 1, 1, 1, 1, 1, 1

Corresponding number of selected Phenopedia diseases each gene is involved with:
(In the same order as above)
26, 26, 25, 19, 23, 19, 17, 17, 25, 25, 24, 24, 23, 15, 14, 8, 20, 19, 12, 18, 12, 18, 17, 17, 17, 17, 16, 8, 10, 7, 14, 14, 26, 24, 24, 24, 23, 11, 11, 22, 7, 7, 7, 7, 7, 7, 10, 20, 20, 19, 19, 18, 18, 18, 6, 17, 17, 17, 16, 8, 4, 4, 4, 4, 16, 4, 4, 8, 8, 5, 15, 5, 5, 15, 5, 7, 7, 14, 14, 14, 14, 14, 14, 14, 7, 7, 3, 12, 12, 4, 3, 3, 4, 4, 6, 11, 11, 11, 11, 5, 10, 5, 5, 10, 9, 3, 3, 3, 4, 8, 8, 4, 4, 4, 4, 4, 4, 4, 4, 4, 4, 4, 2, 4, 8, 4, 4, 4, 4, 4, 2, 4, 4, 8, 7, 7, 7, 7, 2, 6, 2, 2, 6, 3, 6, 3, 3, 2, 6, 3, 6, 1, 5, 5, 5, 5, 5, 5, 5, 5, 5, 5, 5, 5, 4, 4, 4, 1, 4, 2, 4, 2, 2, 4, 1, 4, 4, 1, 4, 4, 4, 2, 4, 2, 4, 2, 4, 4, 1, 4, 2, 4, 4, 4, 4, 4, 4, 1, 1, 4, 2, 4, 4, 1, 1, 1, 3, 3, 1, 3, 1, 3, 1, 1, 1, 3, 3, 3, 1, 3, 3, 3, 3, 1, 3, 2, 2, 2, 2, 1, 2, 2, 2, 2, 1, 1, 2, 1, 2, 2, 2, 1, 1, 2, 1, 2, 2, 2, 2, 1, 1, 1, 2, 1, 1, 1, 2, 1, 2, 1, 2, 1, 1, 1, 1, 1, 1, 1, 1, 1, 1, 1, 1, 1, 1, 1, 1, 1, 1, 1, 1, 1, 1, 1, 1, 1, 1, 1, 1, 1, 1, 1, 1, 1, 1, 1, 1, 1, 1, 1, 1, 1, 1, 1, 1, 1, 1, 1, 1

Corresponding number of selected TFs each gene is involved with:
(In the same order as above)
5, 5, 4, 5, 4, 4, 4, 4, 2, 2, 2, 2, 2, 3, 3, 5, 2, 2, 3, 2, 3, 2, 2, 2, 2, 2, 2, 4, 3, 4, 2, 2, 1, 1, 1, 1, 1, 2, 2, 1, 3, 3, 3, 3, 3, 3, 2, 1, 1, 1, 1, 1, 1, 1, 3, 1, 1, 1, 1, 2, 4, 4, 4, 4, 1, 4, 4, 2, 2, 3, 1, 3, 3, 1, 3, 2, 2, 1, 1, 1, 1, 1, 1, 1, 2, 2, 4, 1, 1, 3, 4, 4, 3, 3, 2, 1, 1, 1, 1, 2, 1, 2, 2, 1, 1, 3, 3, 3, 2, 1, 1, 2, 2, 2, 2, 2, 2, 2, 2, 2, 2, 2, 4, 2, 1, 2, 2, 2, 2, 2, 4, 2, 2, 1, 1, 1, 1, 1, 3, 1, 3, 3, 1, 2, 1, 2, 2, 3, 1, 2, 1, 5, 1, 1, 1, 1, 1, 1, 1, 1, 1, 1, 1, 1, 1, 1, 1, 4, 1, 2, 1, 2, 2, 1, 4, 1, 1, 4, 1, 1, 1, 2, 1, 2, 1, 2, 1, 1, 4, 1, 2, 1, 1, 1, 1, 1, 1, 4, 4, 1, 2, 1, 1, 3, 3, 3, 1, 1, 3, 1, 3, 1, 3, 3, 3, 1, 1, 1, 3, 1, 1, 1, 1, 3, 1, 1, 1, 1, 1, 2, 1, 1, 1, 1, 2, 2, 1, 2, 1, 1, 1, 2, 2, 1, 2, 1, 1, 1, 1, 2, 2, 2, 1, 2, 2, 2, 1, 2, 1, 2, 1, 1, 1, 1, 1, 1, 1, 1, 1, 1, 1, 1, 1, 1, 1, 1, 1, 1, 1, 1, 1, 1, 1, 1, 1, 1, 1, 1, 1, 1, 1, 1, 1, 1, 1, 1, 1, 1, 1, 1, 1, 1, 1, 1, 1, 1, 1

Phenopedia diseases ranked according to mean of effect sizes in cluster:
Q Fever | Fever | Dengue Hemorrhagic Fever | Dengue | Paramyxoviridae Infections | Respiratory Syncytial Virus Infections | Hepatitis, Viral, Human | Infection | Respiratory Tract Infections | Flaviviridae Infections | Hepatitis, Chronic | Hepatitis C, Chronic | Airway Remodeling | Liver Diseases | Bronchiolitis, Viral | Bronchiolitis | Bronchitis | Necrosis | Infant, Premature, Diseases | Respiratory Hypersensitivity | Hypersensitivity | Hypersensitivity, Immediate | Asthma | Fibrosis | Infant, Newborn, Diseases | Cystic Fibrosis

Corresponding mean of effect sizes of each Phenopedia disease:
(In the same order as above)
2.82, 2.65, 2.64, 2.63, 2.17, 1.83, 1.82, 1.76, 1.65, 1.60, 1.50, 1.46, 1.41, 1.41, 1.40, 1.39, 1.32, 1.31, 1.30, 1.26, 1.24, 1.17, 1.09, 1.06, 1.06, 0.76

Corresponding total number of genes of each Phenopedia disease:
(In the same order as above)
199, 202, 107, 108, 224, 219, 308, 833, 320, 272, 184, 141, 212, 498, 211, 214, 226, 519, 341, 554, 654, 606, 603, 291, 420, 101

Corresponding number of genes of each Phenopedia disease with at least one TF hit (of selected TFs):
(In the same order as above)
53, 53, 35, 35, 53, 50, 77, 174, 68, 67, 44, 35, 47, 105, 47, 47, 47, 109, 69, 123, 142, 134, 132, 59, 79, 19

Corresponding total number of TF hits (of selected TFs) for each Phenopedia disease (in all genes):
(In the same order as above)
109, 109, 76, 76, 105, 98, 149, 314, 128, 129, 94, 76, 89, 192, 89, 89, 89, 198, 129, 222, 252, 241, 236, 108, 145, 34

Corresponding number of selected TFs each Phenopedia disease is involved with:
(In the same order as above)

5, 5, 5, 5, 5, 5, 5, 5, 5, 5, 5, 5, 5, 5, 5, 5, 5, 5, 5, 5, 5, 5, 5, 5, 5, 5

TFs ranked according to mean of effect sizes in cluster:
V$IRF7_01, V$IRF_Q6, VIRF_Q6_01, VICSBP_Q6, V$CDPCR3_01

Corresponding mean of effect sizes of each TF:
(In the same order as above)
1.99, 1.71, 1.65, 1.36, 1.31

Corresponding total number of TF hits for each TF (genome-wide):
(In the same order as above)
478, 542, 548, 522, 490

Corresponding total number of TF hits for each TF (in all genes in selected Phenopedia diseases):
(In the same order as above)
654, 817, 753, 713, 639

Corresponding number of genes (of selected Phenopedia diseases) each TF is involved with:
(In the same order as above)
98, 126, 114, 121, 90

Corresponding number of selected Phenopedia diseases each TF is involved with:
(In the same order as above)
26, 26, 26, 26, 26

Disease groups of selected Phenopedia diseases ranked according to sum of effect sizes
Signs and Symptoms | Communicable Diseases | Virus Diseases | Respiratory Tract Diseases | Flaviviridae Infections | Arbovirus Infections | Liver Diseases | Digestive System Diseases | Gram-Negative Bacterial Infections | Bacterial Infections | Hemorrhagic Fevers, Viral | Flavivirus Infections | Dengue | Thoracic Diseases | Hepatitis | Infection | Bronchitis | Bronchial Diseases | Paramyxoviridae Infections | Immune System Diseases | Hypersensitivity | Hepatitis, Viral, Human | Typhus, Epidemic Louse-Borne | Rickettsia Infections | Q Fever | Bronchiolitis | Respiration Disorders | Fever of Unknown Origin | Fever | Vascular Diseases | Staphylococcal Infections | Soft Tissue Infections | Shock, Septic | Shock | Gram-Positive Bacterial Infections | Endotoxemia | Dengue Hemorrhagic Fever | Cardiovascular Infections | Cardiovascular Diseases | Hypersensitivity, Immediate | Infant, Newborn, Diseases | Congenital, Hereditary, and Neonatal Diseases and Abnormalities | Respiratory Syncytial Virus Infections | Pneumovirus Infections | Respiratory Tract Infections | Hepatitis, Chronic | Hepatitis C, Chronic | Hepatitis C | Airway Remodeling | Bronchiolitis, Viral | Necrosis | Infant, Premature, Diseases | Respiratory Hypersensitivity | Asthma | Fibrosis | Lung Diseases | Cystic Fibrosis

Corresponding sum of effect sizes for each disease group of selected Phenopedia diseases
(In the same order as above)
103.69, 91.89, 77.80, 44.36, 41.69, 41.69, 30.94, 30.94, 27.30, 27.30, 26.38, 26.38, 26.38, 24.34, 23.90, 22.00, 20.57, 20.57, 19.99, 18.37, 18.37, 16.41, 14.08, 14.08, 14.08, 13.97, 13.69, 13.23, 13.23, 13.22, 13.22, 13.22, 13.22, 13.22, 13.22, 13.22, 13.22, 13.22, 13.22, 12.19, 11.80, 11.80, 9.15, 9.15, 8.23, 7.49, 7.29, 7.29, 7.05, 7.00, 6.57, 6.50, 6.32, 5.46, 5.32, 3.78, 3.78

Cluster for columns 227 to 239, rows 191 to 201

Phenopedia diseases

Asthma | Respiratory Hypersensitivity | Hypersensitivity | Hypersensitivity, Immediate | Infant, Newborn, Diseases | Infant, Premature, Diseases | Respiratory Tract Infections | Airway Remodeling | Paramyxoviridae Infections | Respiratory Syncytial Virus Infections | Bronchitis | Bronchiolitis | Bronchiolitis, Viral

TFs

V$STAT5B_01 | V$STAT5A_01 | V$STAT_01 | V$STAT3_01 | V$STAT1_01 | V$NFKB_C | V$NFKAPPAB_01 | V$NFKAPPAB65_01 | V$CREL_01 | V$NFKB_Q6_01 | V$NFKB_Q6

Information

All related TFs:
(List of all TFs that are related to any of the PWMs)
NF-TNF, NF-kappaB, NF-kappaB(-like), NF-kappaB2, RelA-p65, STAT1alpha, STAT1beta, STAT2, STAT3, STAT4, STAT5A, STAT5B, STAT6, c-Rel, p100, p105, p50, p52

Ranked gene list:
(All genes of the selected Phenopedia diseases with hits of any of the selected TFs, ranked according to the total number of TF hits)
IRF1, PSMB8, TNF, SOCS2, NOD2, NFKB2, LTA, IRF5, STAT6, SOCS3, NFKBIA, IL2RA, TGFB1, TAP1, MUC1, IRF9, STAT1, NGFR, JUN, IL19, IL11, CCL2, VDR, TNFRSF1A, STAT3, NR3C1, IL4R, IL27, IL10, IFNAR2, GATA3, CD40, RTEL1, GSTP1, CARD15, ALOX5AP, HDAC7, VIP, TNFRSF1B, TAP2, STAT5A, NOS1, JAK1, IRF3, IL16, CXCL6, CXCL5, CXCL16, CXCL1, CHUK, CD14, CCL25, ADA, ZPBP2, RGS16, PTGS1, PTGER4, LTB, EGR1, ADORA2A, NOS3, HLA-B, TNFAIP1, RAC2, PHLDB1, CREB1, NFKB1, SH2B3, PSMB9, JUND, IFNGR1, CTSS, CD4, ADAM8, TNFAIP3, LIG4, IL7R, HLA-C, CYP27B1, SERPINA1, TLR5, TACR3, SFTPB, MUC4, IL2RB, IL27RA, IL1RN, IL17RA, IL12B, IL10RB, IKBKB, IFNGR2, IFNAR1, HDC, FOS, CYSLTR1, CXCL3, CXCL2, CD86, CD40LG, CCL1, ARG1, ADRB2, TRAF1, RUNX1, RASGRP4, PTGFR, PTGER1, PLAU, PARP1, MYB, IL28RA, FYN, FKBP4, FAS, CRHR1, CFLAR, CCR1, BDNF, ARRB2, TRAF2, TRADD, MEFV, HLA-A, CYP1B1, CYBA, TNFRSF6B, TBXA2R, SOD2, SOCS1, SFRP5, SFRP2, NOD1, ITPR1, HSP90AA1, GPX4, DNAJB1, CYP1A1, CREM, CREBL2, CREBBP, COL18A1, CD55, CD27, CARD4, NFKBIE, VEGFA, VEGF, SLC19A1, SERPINF1, MMP12, MIF, ITGB3, IGF1R, HSD11B2, GART, FADS2, DUOX2, CXCL10, BIRC3, ALDH1B1, TNFRSF4, TNFRSF18, PLAUR, PDCD1, NFKBIB, LTC4S, IL22RA1, FANCM, ACHE, TNFRSF9, TLE4, SMARCE1, SLC6A4, SHMT1, S100A9, S100A5, S100A1, RXRB, RNASE2, PTGES3, PTGES, PTGDR, PLCB4, PLCB2, P11, ORMDL3, NRIP2, MMP2, LTA4H, LAG3, IGF1, GSDML, GSDMB, GNB1, FOLR1, F2RL1, DEFA6, DEFA4, DCLRE1C, CYP17A1, CX3CL1, CDKN2A, BHR1, BCL2A1, ARRB1, APOA4, ADORA1, ADAMTSL1, RAG2, NFKBIZ, IL21R, IFRD1, FOXJ1, FADS1, CTSZ, WAS, TOLLIP, TNFRSF7, TNFRSF14, SCNN1B, S100A6, S100A10, PTGES2, MYD88, LDLR, HSPA1L, HIF1A, EDN2, ATM, ABCA3, UNG, TNFRSF25, TNFRSF11B, PAX2, JAK3, ICAM3, HFE, GSTO1, GJB6, FRMD7, FCN3, ESD, ERCC2, CLCN2, ALOXE3

Corresponding total number of TF hits:
(For each gene listed above, the total number of TF hits for any of the selected TFs, multiplied by the number of selected Phenopedia diseases containing that gene)
130, 104, 91, 91, 91, 91, 91, 91, 78, 78, 78, 78, 65, 65, 65, 65, 52, 52, 52, 52, 52, 52, 39, 39, 39, 39, 39, 39, 39, 39, 39, 39, 36, 36, 35, 30, 28, 26, 26, 26, 26, 26, 26, 26, 26, 26, 26, 26, 26, 26, 26, 25, 24, 24, 24, 24, 24, 24, 24, 21, 21, 20, 20, 20, 20, 18, 16, 16, 16, 16, 16, 16, 16, 15, 15, 15, 15, 15, 14, 13, 13, 13, 13, 13, 13, 13, 13, 13, 13, 13, 13, 13, 13, 13, 13, 13, 13, 13, 13, 13, 13, 13, 13, 13, 13, 13, 13, 12, 12, 12, 12, 12, 12, 12, 12, 12, 12, 12, 12, 12, 12, 12, 12, 12, 10, 10, 10, 10, 10, 10, 9, 8, 8, 8, 8, 8, 8, 8, 8, 8, 8, 8, 8, 8, 8, 8, 8, 8, 8, 7, 6, 6, 6, 6, 6, 6, 6, 6, 6, 6, 6, 6, 6, 6, 6, 5, 5, 5, 5, 5, 5, 5, 5, 5, 5, 4, 4, 4, 4, 4, 4, 4, 4, 4, 4, 4, 4, 4, 4, 4, 4, 4, 4, 4, 4, 4, 4, 4, 4, 4, 4, 4, 4, 4, 4, 4, 4, 4, 4, 4, 4, 4, 4, 4, 4, 3, 3, 3, 3, 3, 3, 3, 2, 2, 2, 2, 2, 2, 2, 2, 2, 2, 2, 2, 2, 2, 2, 1, 1, 1, 1, 1, 1, 1, 1, 1, 1, 1, 1, 1, 1, 1

Corresponding number of selected Phenopedia diseases each gene is involved with:
(In the same order as above)
13, 13, 13, 13, 13, 13, 13, 13, 13, 13, 13, 13, 13, 13, 13, 13, 13, 13, 13, 13, 13, 13, 13, 13, 13, 13, 13, 13, 13, 13, 13, 13, 4, 9, 5, 5, 4, 13, 13, 13, 13, 13, 13, 13, 13, 13, 13, 13, 13, 13, 13, 13, 5, 4, 4, 6, 6, 4, 4, 4, 7, 7, 4, 4, 4, 4, 3, 4, 2, 4, 8, 4, 4, 4, 3, 5, 5, 5, 5, 7, 13, 13, 13, 13, 13, 13, 13, 13, 13, 13, 13, 13, 13, 13, 13,

13, 13, 13, 13, 13, 13, 13, 13, 2, 4, 4, 6, 6, 6, 4, 4, 3, 4, 4, 3, 6, 4, 3, 4, 4, 2, 2, 5, 5, 5, 10, 1, 4, 4, 4, 4, 4, 4, 4, 4, 4, 4, 8, 4, 4, 4, 4, 4, 4, 4, 1, 6, 6, 2, 2, 6, 6, 6, 2, 2, 2, 3, 2, 1, 2, 2, 1, 1, 5, 1, 1, 5, 1, 1, 1, 1, 4, 4, 4, 2, 2, 2, 2, 1, 2, 4, 2, 4, 4, 4, 4, 4, 4, 4, 4, 1, 2, 4, 4, 4, 2, 4, 2, 2, 1, 2, 4, 4, 4, 2, 4, 2, 4, 4, 1, 1, 1, 1, 3, 3, 1, 1, 2, 1, 1, 1, 2, 2, 2, 2, 2, 2, 2, 2, 1, 2, 1, 1, 1, 1, 1, 1, 1, 1, 1, 1, 1, 1, 1, 1, 1

Corresponding number of selected TFs each gene is involved with:
(In the same order as above)
10, 8, 7, 7, 7, 7, 7, 7, 6, 6, 6, 6, 5, 5, 5, 5, 4, 4, 4, 4, 4, 4, 3, 3, 3, 3, 3, 3, 3, 3, 3, 3, 9, 4, 7, 6, 7, 2, 2, 2, 2, 2, 2, 2, 2, 2, 2, 2, 2, 2, 2, 2, 5, 6, 6, 4, 4, 6, 6, 6, 3, 3, 5, 5, 5, 5, 6, 4, 8, 4, 2, 4, 4, 4, 5, 3, 3, 3, 3, 2, 1, 1, 1, 1, 1, 1, 1, 1, 1, 1, 1, 1, 1, 1, 1, 1, 1, 1, 1, 1, 1, 1, 6, 3, 3, 2, 2, 2, 3, 3, 4, 3, 3, 4, 2, 3, 4, 3, 3, 5, 5, 2, 2, 2, 1, 9, 2, 2, 2, 2, 2, 2, 2, 2, 2, 2, 1, 2, 2, 2, 2, 2, 2, 2, 7, 1, 1, 3, 3, 1, 1, 1, 3, 3, 3, 2, 3, 6, 3, 3, 5, 5, 1, 5, 5, 1, 5, 5, 5, 4, 1, 1, 1, 2, 2, 2, 2, 4, 2, 1, 2, 1, 1, 1, 1, 1, 1, 1, 1, 4, 2, 1, 1, 1, 2, 1, 2, 2, 4, 2, 1, 1, 1, 2, 1, 2, 1, 1, 3, 3, 3, 3, 1, 1, 3, 2, 1, 2, 2, 2, 1, 1, 1, 1, 1, 1, 1, 1, 2, 1, 1, 1, 1, 1, 1, 1, 1, 1, 1, 1, 1, 1, 1, 1

Phenopedia diseases ranked according to mean of effect sizes in cluster:
Respiratory Tract Infections | Bronchitis | Airway Remodeling | Bronchiolitis, Viral | Bronchiolitis | Respiratory Syncytial Virus Infections | Paramyxoviridae Infections | Infant, Premature, Diseases | Infant, Newborn, Diseases | Hypersensitivity | Respiratory Hypersensitivity | Asthma | Hypersensitivity, Immediate

Corresponding mean of effect sizes of each Phenopedia disease:
(In the same order as above)
2.26, 2.23, 2.22, 2.11, 2.10, 2.07, 2.01, 1.98, 1.83, 1.26, 1.26, 1.12, 1.10

Corresponding total number of genes of each Phenopedia disease:
(In the same order as above)
320, 226, 212, 211, 214, 219, 224, 341, 420, 654, 554, 603, 606

Corresponding number of genes of each Phenopedia disease with at least one TF hit (of selected TFs):
(In the same order as above)
101, 75, 72, 71, 71, 72, 72, 106, 126, 191, 166, 178, 180

Corresponding total number of TF hits (of selected TFs) for each Phenopedia disease (in all genes):
(In the same order as above)
298, 220, 216, 212, 212, 214, 214, 302, 343, 527, 463, 505, 497

Corresponding number of selected TFs each Phenopedia disease is involved with:
(In the same order as above)
11, 11, 11, 11, 11, 11, 11, 11, 11, 11, 11, 11, 11

TFs ranked according to mean of effect sizes in cluster:
V$NFKB_Q6_01, V$NFKAPPAB65_01, V$NFKB_Q6, V$STAT_01, V$NFKB_C, V$STAT1_01, V$STAT5B_01, V$STAT3_01, V$STAT5A_01, V$NFKAPPAB_01, V$CREL_01

Corresponding mean of effect sizes of each TF:
(In the same order as above)
2.74, 2.37, 2.20, 2.17, 1.70, 1.61, 1.58, 1.47, 1.45, 1.37, 1.27

Corresponding total number of TF hits for each TF (genome-wide):
(In the same order as above)
467, 488, 465, 454, 472, 453, 495, 453, 493, 477, 456

Corresponding total number of TF hits for each TF (in all genes in selected Phenopedia diseases):
(In the same order as above)
415, 443, 467, 382, 408, 289, 397, 298, 367, 386, 371

Corresponding number of genes (of selected Phenopedia diseases) each TF is involved with:
(In the same order as above)
64, 70, 73, 53, 63, 51, 60, 55, 62, 68, 60

Corresponding number of selected Phenopedia diseases each TF is involved with:
(In the same order as above)
13, 13, 13, 13, 13, 13, 13, 13, 13, 13, 13

Disease groups of selected Phenopedia diseases ranked according to sum of effect sizes
Respiratory Tract Diseases | Signs and Symptoms | Thoracic Diseases | Bronchitis | Bronchial Diseases | Virus Diseases | Communicable Diseases | Bronchiolitis | Paramyxoviridae Infections | Infant, Newborn, Diseases | Congenital, Hereditary, and Neonatal Diseases and Abnormalities | Immune System Diseases | Hypersensitivity | Respiration Disorders | Hypersensitivity, Immediate | Respiratory Tract Infections | Airway Remodeling | Bronchiolitis, Viral | Respiratory Syncytial Virus Infections | Pneumovirus Infections | Infant, Premature, Diseases | Respiratory Hypersensitivity | Asthma

Corresponding sum of effect sizes for each disease group of selected Phenopedia diseases
(In the same order as above)
121.86, 109.99, 70.83, 70.83, 70.83, 68.09, 68.09, 46.29, 44.91, 41.90, 41.90, 39.79, 39.79, 37.18, 25.92, 24.86, 24.47, 23.19, 22.76, 22.76, 21.73, 13.85, 12.32

Cluster for columns 252 to 259, rows 196 to 201

Phenopedia diseases

Necrosis | Intestinal Fistula | Rectal Fistula | Crohn Disease | Gastroenteritis | Inflammatory Bowel Diseases | Colitis | Colitis, Ulcerative

TFs

V$NFKB_C | V$NFKAPPAB_01 | V$NFKAPPAB65_01 | V$CREL_01 | V$NFKB_Q6_01 | V$NFKB_Q6

Information

All related TFs:
(List of all TFs that are related to any of the PWMs)
NF-TNF, NF-kappaB, NF-kappaB(-like), NF-kappaB2, RelA-p65, c-Rel, p100, p105, p50, p52

Ranked gene list:
(All genes of the selected Phenopedia diseases with hits of any of the selected TFs, ranked according to the total number of TF hits)
TNF, NOD2, CARD15, STAT6, NFKB1, LTA, TPMT, TNFRSF6B, TNFAIP3, TGFB1, PDCD1, NFKBIA, AGER, IRF5, STX8, RUNX3, PTGS1, PBX2, IRF1, GSTP1, VDR, NOTCH4, CCND1, SBNO2, IL27, IL11, GPSM3, BSN, NAT9, HLA-C, HLA-B, HLA-A, IL2RA, GRM8, CLIC1, TRAF1, TNFRSF1B, STAT1, NR3C1, IMPDH2, IL12B, CXCL16, ADA, STAT3, ZPBP2, REL, OPTN, NFKBIE, NFKB2, MIF, MAP3K1, LTB, LST1, IL23A, IL1RN, HSPA1L, FAS, CD40, BCL3, B2M, TRAF2, TRADD, TNFRSF4, TNFRSF18, RUNX1, ORMDL3, NFKBIB, ITPA, IL4R, ICOSLG, GSDMB, FAM167A, CARD8, XRCC1, VEGFA, VEGF, TNFRSF9, TNFRSF14, RNPEPL1, RELB, PTGER4, LAG3, IL17REL, CYBA, CXCL5, CLEC16A, ATXN2L, ZGPAT, SLC19A1, RELA, MAL, ITGB3, IL7R, HSD11B2, CD58, BIRC3, AFF3, TNFRSF7, TNFAIP1, TAP1, PTGER1, PSORS1, PLAU, PGC, MOCOS, IL21R, IL16, GGT1, ERCC2, ERAP1, EIF4H, EGR1, CRHR1, CD27, CCL21, BID, APPL1,

APPL, APOA4, APBB1, TSHR, TNFRSF25, TNFRSF11B, TAP2, SREBF2, SREBF1, SOCS3, SERPINA1, PTGES, OS9, MYD88, MUC1, MMD2, MAP3K14, LPIN2, KLRK1, ITGAM, IKBKB, IFNGR2, HIF1A, HFE, DLEC1, CREBBP, CLOCK, CDKN2A, CDK6, ARPC1A, ALOX5AP

Corresponding total number of TF hits:
(For each gene listed above, the total number of TF hits for any of the selected TFs, multiplied by the number of selected Phenopedia diseases containing that gene)
48, 48, 42, 36, 36, 36, 30, 30, 30, 30, 30, 30, 25, 24, 21, 20, 20, 20, 20, 20, 18, 18, 18, 15, 15, 15, 15, 14, 12, 12, 12, 12, 10, 10, 10, 8, 8, 8, 8, 8, 8, 8, 8, 7, 6, 6, 6, 6, 6, 6, 6, 6, 6, 6, 6, 6, 6, 6, 6, 5, 5, 5, 5, 5, 5, 5, 5, 5, 5, 5, 5, 5, 4, 4, 4, 4, 4, 4, 4, 4, 4, 4, 4, 4, 4, 4, 3, 3, 3, 3, 3, 3, 3, 3, 3, 3, 2, 2, 2, 2, 2, 2, 2, 2, 2, 2, 2, 2, 2, 2, 2, 2, 2, 2, 2, 2, 2, 2, 1, 1, 1, 1, 1, 1, 1, 1, 1, 1, 1, 1, 1, 1, 1, 1, 1, 1, 1, 1, 1, 1, 1, 1, 1, 1, 1

Corresponding number of selected Phenopedia diseases each gene is involved with:
(In the same order as above)
8, 8, 7, 6, 6, 6, 5, 5, 6, 6, 6, 5, 5, 4, 7, 4, 5, 5, 4, 5, 6, 6, 6, 5, 5, 5, 5, 7, 3, 6, 6, 6, 5, 5, 5, 2, 8, 2, 4, 2, 8, 4, 4, 7, 1, 1, 1, 1, 1, 6, 6, 1, 1, 3, 6, 6, 6, 2, 1, 1, 1, 1, 1, 1, 5, 5, 1, 5, 5, 5, 5, 1, 5, 2, 4, 4, 1, 2, 1, 1, 4, 1, 4, 4, 2, 4, 1, 3, 1, 1, 1, 3, 1, 1, 1, 1, 1, 1, 1, 1, 1, 1, 1, 1, 2, 1, 1, 1, 2, 2, 1, 1, 1, 1, 2, 1, 1, 1, 1, 1, 1, 1, 1, 1, 1, 1, 1, 1, 1, 1, 1, 1, 1, 1, 1, 1, 1, 1, 1, 1, 1, 1, 1, 1

Corresponding number of selected TFs each gene is involved with:
(In the same order as above)
6, 6, 6, 6, 6, 6, 6, 6, 5, 5, 5, 6, 5, 6, 3, 5, 4, 4, 5, 4, 3, 3, 3, 3, 3, 3, 3, 2, 4, 2, 2, 2, 2, 2, 2, 4, 1, 4, 2, 4, 1, 2, 2, 1, 6, 6, 6, 6, 6, 1, 1, 6, 6, 2, 1, 1, 1, 3, 6, 6, 5, 5, 5, 5, 1, 1, 5, 1, 1, 1, 1, 5, 1, 2, 1, 1, 4, 2, 4, 4, 1, 4, 1, 1, 2, 1, 4, 1, 3, 3, 3, 1, 3, 3, 3, 3, 3, 2, 2, 2, 2, 2, 2, 2, 1, 2, 2, 2, 1, 1, 2, 2, 2, 2, 1, 2, 2, 2, 2, 2, 1, 1, 1, 1, 1, 1, 1, 1, 1, 1, 1, 1, 1, 1, 1, 1, 1, 1, 1, 1, 1, 1, 1, 1, 1

Phenopedia diseases ranked according to mean of effect sizes in cluster:
Colitis | Crohn Disease | Gastroenteritis | Colitis, Ulcerative | Necrosis | Inflammatory Bowel Diseases | Rectal Fistula | Intestinal Fistula

Corresponding mean of effect sizes of each Phenopedia disease:
(In the same order as above)
2.12, 2.11, 1.95, 1.86, 1.78, 1.75, 1.31, 1.07

Corresponding total number of genes of each Phenopedia disease:
(In the same order as above)
261, 363, 351, 214, 519, 313, 25, 29

Corresponding number of genes of each Phenopedia disease with at least one TF hit (of selected TFs):
(In the same order as above)
54, 77, 69, 45, 100, 64, 7, 8

Corresponding total number of TF hits (of selected TFs) for each Phenopedia disease (in all genes):
(In the same order as above)
161, 216, 195, 129, 267, 180, 20, 26

Corresponding number of selected TFs each Phenopedia disease is involved with:
(In the same order as above)
6, 6, 6, 6, 6, 6, 6, 6

TFs ranked according to mean of effect sizes in cluster:
V$NFKAPPAB65_01, V$NFKAPPAB_01, V$NFKB_Q6, V$NFKB_Q6_01, V$CREL_01, V$NFKB_C

Corresponding mean of effect sizes of each TF:
(In the same order as above)

2.37, 2.00, 1.82, 1.55, 1.39, 1.32

Corresponding total number of TF hits for each TF (genome-wide):
(In the same order as above)
488, 477, 465, 467, 456, 472

Corresponding total number of TF hits for each TF (in all genes in selected Phenopedia diseases):
(In the same order as above)
223, 217, 204, 172, 189, 189

Corresponding number of genes (of selected Phenopedia diseases) each TF is involved with:
(In the same order as above)
73, 73, 63, 59, 60, 66

Corresponding number of selected Phenopedia diseases each TF is involved with:
(In the same order as above)
8, 8, 8, 8, 8, 8

Disease groups of selected Phenopedia diseases ranked according to sum of effect sizes
Intestinal Diseases | Gastrointestinal Diseases | Digestive System Diseases | Colonic Diseases | Colitis | Inflammatory Bowel Diseases | Stomach Diseases | Gastroenteritis | Gastritis | Ileitis | Enteritis | Crohn Disease | Colitis, Ulcerative | Necrosis | Rectal Fistula | Rectal Diseases | Signs and Symptoms | Intestinal Fistula | Fistula | Cutaneous Fistula

Corresponding sum of effect sizes for each disease group of selected Phenopedia diseases
(In the same order as above)
72.98, 72.98, 72.98, 36.54, 36.54, 34.27, 24.34, 24.34, 24.34, 12.64, 12.64, 12.64, 11.16, 10.70, 7.83, 7.83, 6.43, 6.43, 6.43, 6.43

Cluster for columns 270 to 271, rows 202 to 203

Phenopedia diseases

Brain Ischemia | Hypoxia-Ischemia, Brain

TFs

V$PR_02 | V$GR_01

Information

All related TFs:
(List of all TFs that are related to any of the PWMs)
GR, PR

Ranked gene list:
(All genes of the selected Phenopedia diseases with hits of any of the selected TFs, ranked according to the total number of TF hits)
VDR, VAMP8, UCP3, SREBF2, SLC39A7, SCNN1A, RUVBL2, MMP9, MLF1, LLGL2, LIMK1, LIF, HSPA4, F3, CAPN10, CALM1, SPATA13, PRKG1, PRKCH, NOTCH3, NOS2A, NOS2, MGAT5, MBL2, LTC4S, LTB4R, LGALS2, KL, IL4R, IL10, HSPA1L, GJA5, ESR2, BDNF, ARHGEF10, AR, ABO

Corresponding total number of TF hits:
(For each gene listed above, the total number of TF hits for any of the selected TFs, multiplied by the number of selected Phenopedia diseases containing that gene)
4, 4, 4, 4, 4, 4, 4, 4, 4, 4, 4, 4, 4, 4, 4, 4, 2, 2, 2, 2, 2, 2, 2, 2, 2, 2, 2, 2, 2, 2, 2, 2, 2, 2, 2, 2, 2

Corresponding number of selected Phenopedia diseases each gene is involved with:
(In the same order as above)
2, 2, 2, 2, 2, 2, 2, 2, 2, 2, 2, 2, 2, 2, 2, 2, 2, 2, 2, 2, 2, 2, 2, 2, 2, 2, 2, 2, 2, 2, 2, 2, 2, 2, 2, 2, 2

Corresponding number of selected TFs each gene is involved with:
(In the same order as above)
2, 2, 2, 2, 2, 2, 2, 2, 2, 2, 2, 2, 2, 2, 2, 2, 1, 1, 1, 1, 1, 1, 1, 1, 1, 1, 1, 1, 1, 1, 1, 1, 1, 1, 1, 1, 1

Phenopedia diseases ranked according to mean of effect sizes in cluster:
Brain Ischemia | Hypoxia-Ischemia, Brain

Corresponding mean of effect sizes of each Phenopedia disease:
(In the same order as above)
4.30, 4.29

Corresponding total number of genes of each Phenopedia disease:
(In the same order as above)
274, 275

Corresponding number of genes of each Phenopedia disease with at least one TF hit (of selected TFs):
(In the same order as above)
37, 37

Corresponding total number of TF hits (of selected TFs) for each Phenopedia disease (in all genes):
(In the same order as above)
53, 53

Corresponding number of selected TFs each Phenopedia disease is involved with:
(In the same order as above)
2, 2

TFs ranked according to mean of effect sizes in cluster:
VGR_01, VPR_02

Corresponding mean of effect sizes of each TF:
(In the same order as above)
4.69, 3.89

Corresponding total number of TF hits for each TF (genome-wide):
(In the same order as above)
476, 487

Corresponding total number of TF hits for each TF (in all genes in selected Phenopedia diseases):
(In the same order as above)
56, 50

Corresponding number of genes (of selected Phenopedia diseases) each TF is involved with:
(In the same order as above)
28, 25

Corresponding number of selected Phenopedia diseases each TF is involved with:
(In the same order as above)
2, 2

Disease groups of selected Phenopedia diseases ranked according to sum of effect sizes
Vascular Diseases | Nervous System Diseases | Ischemia | Cerebrovascular Disorders | Central Nervous System Diseases | Cardiovascular Diseases | Brain Ischemia | Brain Diseases | Signs and Symptoms | Respiratory Tract Diseases | Respiration Disorders | Hypoxia-Ischemia, Brain | Hypoxia, Brain | Death | Asphyxia | Anoxia

Corresponding sum of effect sizes for each disease group of selected Phenopedia diseases
(In the same order as above)
17.18, 17.18, 17.18, 17.18, 17.18, 17.18, 17.18, 17.18, 8.57, 8.57, 8.57, 8.57, 8.57, 8.57, 8.57, 8.57

Cluster for columns 452 to 453, rows 51 to 58

Phenopedia diseases

Arthritis, Psoriatic | Psoriasis

TFs

V$AP1_Q6 | V$AP1_01 | V$AP1_Q6_01 | V$AP1_Q4_01 | V$AP1_C | V$AP1_Q4 | V$AP1_Q2 | V$AP1FJ_Q2

Information

All related TFs:
(List of all TFs that are related to any of the PWMs)
AP-1, FosB, Fra-1, Fra-2, JunB, JunB:Fra-1, JunB:Fra-2, JunD, JunD:Fra-2, JunD:deltaFosB, YAP1, c-Fos, c-Jun, c-Jun:FosB, c-Jun:JunD, c-Jun:c-Fos, deltaFosB

Ranked gene list:
(All genes of the selected Phenopedia diseases with hits of any of the selected TFs, ranked according to the total number of TF hits)
VDR, TNF, RUNX1, PSTPIP1, NLRP3, TNFRSF1A, MTHFR, LTA, PSORS1, HLA-C, HLA-B, TNIP1, PSMB9, PSMB8, IRF1, IL6R, IL4R, IL23A, S100A2, RXRB, VEGFA, VEGF, TNFRSF1B, TNFAIP3, ABCC1, LAG3, IRAK1, SLC9A3R1, MIF, MC1R, PSORS1C1, NQO1, IL10, CSNK1D, TAP2, PADI4, IL19, CDSN, C6orf15

Corresponding total number of TF hits:
(For each gene listed above, the total number of TF hits for any of the selected TFs, multiplied by the number of selected Phenopedia diseases containing that gene)
16, 16, 16, 16, 16, 14, 14, 14, 10, 10, 10, 8, 8, 8, 8, 8, 8, 8, 7, 7, 6, 6, 6, 6, 6, 5, 4, 3, 3, 3, 2, 2, 2, 2, 1, 1, 1, 1, 1

Corresponding number of selected Phenopedia diseases each gene is involved with:
(In the same order as above)
2, 2, 2, 2, 2, 2, 2, 2, 2, 2, 2, 1, 1, 1, 1, 1, 2, 1, 1, 1, 2, 2, 2, 1, 1, 1, 2, 1, 1, 1, 2, 1, 2, 1, 1, 1, 1, 1, 1

Corresponding number of selected TFs each gene is involved with:
(In the same order as above)
8, 8, 8, 8, 8, 7, 7, 7, 5, 5, 5, 8, 8, 8, 8, 8, 4, 8, 7, 7, 3, 3, 3, 6, 6, 5, 2, 3, 3, 3, 1, 2, 1, 2, 1, 1, 1, 1, 1

Phenopedia diseases ranked according to mean of effect sizes in cluster:
Psoriasis | Arthritis, Psoriatic

Corresponding mean of effect sizes of each Phenopedia disease:
(In the same order as above)
3.21, 3.11

Corresponding total number of genes of each Phenopedia disease:
(In the same order as above)
155, 55

Corresponding number of genes of each Phenopedia disease with at least one TF hit (of selected TFs):
(In the same order as above)
39, 18

Corresponding total number of TF hits (of selected TFs) for each Phenopedia disease (in all genes):
(In the same order as above)
190, 93

Corresponding number of selected TFs each Phenopedia disease is involved with:
(In the same order as above)
8, 8

TFs ranked according to mean of effect sizes in cluster:
V$AP1_Q4, V$AP1_Q4_01, V$AP1_Q6_01, V$AP1_01, V$AP1_C, V$AP1_Q2, V$AP1_Q6, V$AP1FJ_Q2

Corresponding mean of effect sizes of each TF:
(In the same order as above)
3.99, 3.31, 3.26, 3.11, 3.09, 2.98, 2.90, 2.64

Corresponding total number of TF hits for each TF (genome-wide):
(In the same order as above)
446, 472, 470, 459, 469, 444, 453, 436

Corresponding total number of TF hits for each TF (in all genes in selected Phenopedia diseases):
(In the same order as above)
43, 38, 38, 32, 37, 36, 30, 29

Corresponding number of genes (of selected Phenopedia diseases) each TF is involved with:
(In the same order as above)
29, 25, 25, 23, 24, 23, 21, 20

Corresponding number of selected Phenopedia diseases each TF is involved with:
(In the same order as above)
2, 2, 2, 2, 2, 2, 2, 2

Disease groups of selected Phenopedia diseases ranked according to sum of effect sizes
Skin Diseases, Papulosquamous | Skin Diseases | Psoriasis | Exanthema | Arthritis, Psoriatic

Corresponding sum of effect sizes for each disease group of selected Phenopedia diseases
(In the same order as above)
50.56, 50.56, 50.56, 24.85, 24.85

Cluster for columns 773 to 781, rows 27 to 31

Phenopedia diseases

Cardiomyopathies | Cardiomyopathy, Dilated | Heart Failure, Congestive | Hypertrophy | Cardiomegaly | Hypertrophy, Left Ventricular | Cardiomyopathy, Hypertrophic | Aortic Valve Stenosis | Heart Valve Diseases

TFs

V$SRF_Q6 | V$SRF_C | V$SRF_Q5_02 | V$SRF_Q4 | V$SRF_01

Information

All related TFs:
(List of all TFs that are related to any of the PWMs)
SRF, SRF-I, SRF-L, SRF-M, SRF-S

Ranked gene list:
(All genes of the selected Phenopedia diseases with hits of any of the selected TFs, ranked according to the total number of TF hits)
MYL3, TNNC1, TPM1, ACTC1, LDB3, VCL, MYH7, CCR5, ACTC, MMP2, PLN, ADRA1D, PPARGC1A, NPPA, ESR1, AR, KCNH2, HLA-DRB1, CTGF, ADRB3, TLR9, MTHFR, ENO3, DMD, SLC6A2, PLA2G7, KCNE1, JPH2, IL2, EDNRB, DSC2, TIMP2, SFRP2, CPT2, NEURL2, MYOZ3, LCAT, KRAS, ELN, SOS1, NR3C2, HSPG2, GSTM1, COMT, ANKH

Corresponding total number of TF hits:
(For each gene listed above, the total number of TF hits for any of the selected TFs, multiplied by the number of selected Phenopedia diseases containing that gene)
36, 35, 32, 32, 21, 20, 18, 18, 16, 12, 8, 8, 7, 7, 7, 7, 6, 6, 6, 6, 5, 5, 5, 5, 4, 4, 4, 4, 4, 4, 4, 3, 3, 3, 2, 2, 2, 2, 2, 1, 1, 1, 1, 1, 1

Corresponding number of selected Phenopedia diseases each gene is involved with:
(In the same order as above)
9, 7, 8, 8, 7, 4, 9, 6, 4, 4, 8, 4, 7, 7, 7, 7, 2, 6, 2, 6, 1, 5, 1, 5, 4, 4, 4, 4, 4, 4, 4, 3, 3, 3, 1, 1, 1, 1, 2, 1, 1, 1, 1, 1, 1

Corresponding number of selected TFs each gene is involved with:
(In the same order as above)
4, 5, 4, 4, 3, 5, 2, 3, 4, 3, 1, 2, 1, 1, 1, 1, 3, 1, 3, 1, 5, 1, 5, 1, 1, 1, 1, 1, 1, 1, 1, 1, 1, 1, 2, 2, 2, 2, 1, 1, 1, 1, 1, 1, 1

Phenopedia diseases ranked according to mean of effect sizes in cluster:
Aortic Valve Stenosis | Cardiomyopathy, Hypertrophic | Cardiomyopathies | Heart Valve Diseases | Cardiomyopathy, Dilated | Cardiomegaly | Heart Failure, Congestive | Hypertrophy | Hypertrophy, Left Ventricular

Corresponding mean of effect sizes of each Phenopedia disease:
(In the same order as above)
4.56, 4.06, 3.19, 3.14, 2.24, 1.82, 0.94, 0.63, 0.54

Corresponding total number of genes of each Phenopedia disease:
(In the same order as above)

63, 56, 146, 91, 75, 149, 112, 163, 89

Corresponding number of genes of each Phenopedia disease with at least one TF hit (of selected TFs):
(In the same order as above)
19, 17, 31, 24, 15, 25, 18, 21, 13

Corresponding total number of TF hits (of selected TFs) for each Phenopedia disease (in all genes):
(In the same order as above)
46, 42, 69, 52, 32, 46, 37, 36, 21

Corresponding number of selected TFs each Phenopedia disease is involved with:
(In the same order as above)
5, 5, 5, 5, 5, 5, 5, 5, 5

TFs ranked according to mean of effect sizes in cluster:
VSRF_Q6, VSRF_C, VSRF_01, VSRF_Q5_02, V$SRF_Q4

Corresponding mean of effect sizes of each TF:
(In the same order as above)
3.42, 2.97, 2.08, 1.72, 1.53

Corresponding total number of TF hits for each TF (genome-wide):
(In the same order as above)
491, 467, 512, 474, 455

Corresponding total number of TF hits for each TF (in all genes in selected Phenopedia diseases):
(In the same order as above)
91, 84, 81, 65, 60

Corresponding number of genes (of selected Phenopedia diseases) each TF is involved with:
(In the same order as above)
22, 17, 20, 16, 14

Corresponding number of selected Phenopedia diseases each TF is involved with:
(In the same order as above)
9, 9, 9, 9, 9

Disease groups of selected Phenopedia diseases ranked according to sum of effect sizes
Thoracic Diseases | Respiratory Tract Diseases | Mediastinal Diseases | Cardiovascular Diseases | Cardiomyopathies | Heart Valve Diseases | Pathological Conditions, Anatomical | Hypertrophy | Cardiomegaly | Hypertrophy, Left Ventricular | Ventricular Outflow Obstruction | Aortic Valve Stenosis | Cardiomyopathy, Hypertrophic | Cardiomyopathy, Dilated | Heart Failure, Congestive

Corresponding sum of effect sizes for each disease group of selected Phenopedia diseases
(In the same order as above)
97.71, 97.71, 97.71, 97.71, 47.45, 38.46, 35.23, 35.23, 32.10, 23.02, 22.78, 22.78, 20.30, 11.21, 4.72

Cluster for columns 754 to 763, rows 1 to 9

Phenopedia diseases

Aortic Diseases | Aortic Aneurysm | Aortic Aneurysm, Abdominal | Aneurysm | Intracranial Aneurysm | Anthropology | Anthropology | Abruptio Placentae | Polycythemia | Hematuria

TFs

V$TCF11MAFG_01 | V$AR_01 | V$LXR_DR4_Q3 | V$DR4_Q2 | V$LXR_Q3 | V$PBX1_02 | V$HSF1_Q6 | V$NFY_C | V$NFKAPPAB50_01

Information

All related TFs:
(List of all TFs that are related to any of the PWMs)
AR, CAR, CAR2:RXR-alpha, CAR:RXR-alpha, CBF(2), CBF-A, CBF-B, COUP, COUP-TF1, COUP-TF2, HSF1, HSF1-L, HSF1-S, HSF1long, HSF1short, LCR-F1, LXR-alpha, LXR-alpha:RXR-alpha, LXR-beta, LXR-beta:RXR-alpha, MafG, NF-Y, NF-YA, NF-YB, PXR-1, PXR-1:RXR-alpha, PXR-1A, PXR-1A:RXR-alpha, PXR-1A:RXR-beta, PXR-2, PXR-2:RXR-alpha, Pbx1a, RAR-alpha, RAR-alpha1, RAR-alpha:RXR-alpha, RAR-alpha:RXR-gamma, RAR-beta, RAR-gamma, RXR-alpha, RXR-beta, SXR:RXR-alpha, p50

Ranked gene list:
(All genes of the selected Phenopedia diseases with hits of any of the selected TFs, ranked according to the total number of TF hits)
HLA-B, HLA-A, APOE, MTHFR, NOS3, HLA-C, F5, LTBP4, HMOX1, HLA-DQB1, SLC19A1, LTBP3, MTHFD1, HPN, HLA-DRB5, SERPINE1, IL6, ESR2, ENG, CAT, IL10, RFC1, ERCC2, APOC1, VKORC1, VCAN, TGFBR1, MTR, HSPG2, CBS, VEGFA, TYMS, PTGS2, NQO1, LTBP1, LOXL1, LIPA, LIMK1, KLK1, GSTO1, GABBR1, DRD4, DRD1, CYP46A1, ATP2B3, APEX1, AKT1, ADRA2C, ADD1, PLAT, HSPA8, HLA-G, HLA-E, HLA-DPB1, GSTM1, EPHX1, CTLA4, XRCC3, WNK4, VDR, TNFRSF1B, TNFAIP1, SMYD3, PLXNA2, OLR1, OCA2, NPHS1, MPL, MICB, MGMT, LPL, JDP2, IQSEC1, HSPB1, GTF2H1, GSTP1, GAB2, FCGR2A, CRHR1, COL4A1, CHI3L1, CHEK2, C3, BAIAP2, AR, APOC2, AHR, ADAMTS20, ADAM17, ADA, ACVR1, THBD, SCGB1A1, JAK2, HIF1A, EPOR, COL4A3, BAT3, APOA5, AGTR2

Corresponding total number of TF hits:
(For each gene listed above, the total number of TF hits for any of the selected TFs, multiplied by the number of selected Phenopedia diseases containing that gene)
48, 40, 21, 20, 18, 18, 16, 15, 14, 14, 12, 12, 10, 10, 10, 8, 8, 8, 8, 8, 7, 6, 6, 6, 5, 5, 5, 5, 5, 5, 4, 4, 4, 4, 4, 4, 4, 4, 4, 4, 4, 4, 4, 4, 4, 4, 4, 3, 3, 3, 3, 3, 3, 3, 3, 2, 2, 2, 2, 2, 2, 2, 2, 2, 2, 2, 2, 2, 2, 2, 2, 2, 2, 2, 2, 2, 2, 2, 2, 2, 2, 2, 2, 2, 2, 2, 2, 2, 2, 2, 2, 1, 1, 1, 1, 1, 1, 1, 1, 1

Corresponding number of selected Phenopedia diseases each gene is involved with:
(In the same order as above)
7, 7, 6, 9, 8, 2, 7, 5, 6, 6, 4, 4, 5, 5, 5, 7, 7, 4, 2, 1, 6, 3, 1, 1, 4, 5, 5, 5, 5, 5, 1, 4, 4, 2, 4, 2, 1, 2, 2, 2, 1, 1, 1, 1, 1, 1, 1, 1, 1, 2, 3, 2, 1, 2, 2, 2, 2, 1, 1, 1, 1, 1, 1, 1, 1, 1, 1, 1, 1, 1, 1, 2, 2, 1, 1, 1, 1, 1, 1, 2, 1, 1, 1, 1, 1, 1, 1, 1, 1, 1, 2, 1, 1, 1, 1, 1, 1, 1, 1, 1

Corresponding number of selected TFs each gene is involved with:
(In the same order as above)
6, 5, 3, 2, 2, 6, 2, 3, 2, 2, 3, 3, 2, 2, 2, 1, 1, 2, 4, 4, 1, 2, 3, 3, 1, 1, 1, 1, 1, 1, 2, 1, 1, 2, 1, 2, 2, 2, 2, 2, 2, 2, 2, 2, 2, 2, 2, 2, 2, 2, 1, 1, 1, 3, 1, 1, 1, 1, 2, 1, 1, 1, 2, 1, 1, 1, 1, 2, 2, 1, 1, 1, 1, 1, 1, 1, 1, 1, 1, 1, 1, 1, 1, 1, 1, 1, 1, 1, 1, 1, 1, 1, 1, 1, 1, 1, 1, 1, 1, 1

Phenopedia diseases ranked according to mean of effect sizes in cluster:
Hematuria | Abruptio Placentae | Aneurysm | Aortic Aneurysm, Abdominal | Aortic Aneurysm | Aortic Diseases | Anthropology | Anthropology | Intracranial Aneurysm | Polycythemia

Corresponding mean of effect sizes of each Phenopedia disease:
(In the same order as above)
2.72, 2.52, 1.60, 1.49, 1.38, 1.17, 1.02, 1.02, 0.95, 0.43

Corresponding total number of genes of each Phenopedia disease:
(In the same order as above)
26, 19, 136, 70, 79, 84, 181, 181, 70, 22

Corresponding number of genes of each Phenopedia disease with at least one TF hit (of selected TFs):
(In the same order as above)
13, 12, 45, 25, 28, 28, 59, 59, 25, 8

Corresponding total number of TF hits (of selected TFs) for each Phenopedia disease (in all genes):
(In the same order as above)
27, 28, 87, 50, 54, 53, 98, 98, 43, 12

Corresponding number of selected TFs each Phenopedia disease is involved with:
(In the same order as above)
9, 9, 9, 9, 9, 9, 9, 9, 9, 6

TFs ranked according to mean of effect sizes in cluster:
VLXR_DR4_Q3, VHSF1_Q6, VLXR_Q3, VTCF11MAFG_01, V$DR4_Q2, V$NFY_C, VAR_01, VPBX1_02, V$NFKAPPAB50_01

Corresponding mean of effect sizes of each TF:
(In the same order as above)
2.39, 2.27, 1.75, 1.47, 1.46, 1.38, 0.81, 0.74, 0.59

Corresponding total number of TF hits for each TF (genome-wide):
(In the same order as above)
436, 499, 439, 499, 451, 440, 471, 507, 468

Corresponding total number of TF hits for each TF (in all genes in selected Phenopedia diseases):
(In the same order as above)
70, 85, 69, 71, 62, 56, 47, 50, 40

Corresponding number of genes (of selected Phenopedia diseases) each TF is involved with:
(In the same order as above)
22, 27, 21, 20, 19, 16, 12, 15, 12

Corresponding number of selected Phenopedia diseases each TF is involved with:
(In the same order as above)
9, 8, 9, 8, 9, 9, 9, 9, 8

Disease groups of selected Phenopedia diseases ranked according to sum of effect sizes
Cardiovascular Diseases | Female Urogenital Diseases | Vascular Diseases | Aortic Diseases | Aortic Aneurysm | Urologic Diseases | Male Urogenital Diseases | Hematuria | Aneurysm | Uterine Diseases | Placenta Diseases | Genital Diseases, Female | Abruptio Placentae | Anthropology | Aortic Aneurysm, Abdominal | Nervous System Diseases | Intracranial Aneurysm | Cerebrovascular Disorders | Central Nervous System Diseases | Brain Diseases | Polycythemia

Corresponding sum of effect sizes for each disease group of selected Phenopedia diseases
(In the same order as above)
59.19, 47.17, 44.78, 36.25, 25.77, 24.48, 24.48, 24.48, 22.94, 22.68, 22.68, 22.68, 22.68, 18.39, 13.37, 8.52, 8.52, 8.52, 8.52, 8.52, 3.87

Cluster for columns 821 to 832, rows 32 to 41

Phenopedia diseases

Social Perception | Flushing | Hot Flashes | Vision Disorders | Geographic Atrophy | Choroid Diseases | Choroidal Neovascularization | Frontotemporal Dementia | Frontotemporal Lobar Degeneration | Spinal Cord Diseases | Amyotrophic Lateral Sclerosis | Motor Neuron Disease

TFs

V$HNF4_01 | V$COUP_01 | V$PPAR_DR1_Q2 | V$DR1_Q3 | V$COUP_DR1_Q6 | V$HNF4ALPHA_Q6 | V$COUPTF_Q6 | V$HNF4_Q6_01 | V$HNF4_DR1_Q3 | V$PPARG_01

Information

All related TFs:
(List of all TFs that are related to any of the PWMs)
COUP, COUP-TF1, COUP-TF2, HNF-4, HNF-4alpha, HNF-4alpha1, HNF-4alpha2, HNF-4alpha3, HNF-4alpha4, HNF-4alpha7, HNF-4gamma, PPAR-alpha, PPAR-alpha:RXR-alpha, PPAR-beta, PPAR-gamma, PPAR-gamma1, PPAR-gamma2, PPAR-gamma2:RXR-alpha, PPAR-gamma:RXR-alpha, RAR-alpha:RXR-alpha

Ranked gene list:
(All genes of the selected Phenopedia diseases with hits of any of the selected TFs, ranked according to the total number of TF hits)
VEGFA, CYP1A1, CFB, TYMP, PDGFB, APOC3, APOA4, VEGF, COMT, DCTN1, NOS3, CHRNA4, HUS1, ANG, TNF, MAPT, SKIV2L, RDBP, SHBG, VPS54, PNMT, MTRR, LTA, FGF14, TNFRSF1B, SCA13, PAX6, KCNC3, FUS, F5, SOD2, PRKCG, IL1B, HLA-B, ATN1, TSPO, TRPM7, ROBO4, PON3, PGF, NTRK3, IL18BP, ESR1, EPO, C13orf18, ABAT, LCA5, HOXA1, HLA-C, DMPK, DCUN1D1, WFS1, VGF, TNFRSF1A, SLC25A13, SCP2, PIN1, NTRK1, NINJ1, NGFR, MYH7, HSD11B1, CYP11A1, APP, ALOX12, ALDH2, AGRN

Corresponding total number of TF hits:
(For each gene listed above, the total number of TF hits for any of the selected TFs, multiplied by the number of selected Phenopedia diseases containing that gene)
96, 45, 32, 30, 27, 27, 27, 24, 21, 20, 18, 16, 12, 12, 10, 10, 9, 9, 8, 6, 6, 6, 6, 6, 5, 5, 5, 5, 5, 5, 4, 4, 4, 4, 4, 3, 3, 3, 3, 3, 3, 3, 3, 3, 3, 3, 2, 2, 2, 2, 2, 1, 1, 1, 1, 1, 1, 1, 1, 1, 1, 1, 1, 1, 1, 1, 1

Corresponding number of selected Phenopedia diseases each gene is involved with:
(In the same order as above)
12, 5, 4, 3, 3, 3, 3, 3, 3, 5, 6, 2, 3, 3, 5, 5, 1, 1, 1, 3, 1, 2, 3, 1, 1, 1, 1, 1, 5, 5, 1, 1, 4, 4, 2, 1, 3, 3, 3, 3, 1, 3, 3, 3, 3, 1, 1, 1, 2, 2, 2, 1, 1, 1, 1, 1, 1, 1, 1, 1, 1, 1, 1, 1, 1, 1, 1

Corresponding number of selected TFs each gene is involved with:
(In the same order as above)
8, 9, 8, 10, 9, 9, 9, 8, 7, 4, 3, 8, 4, 4, 2, 2, 9, 9, 8, 2, 6, 3, 2, 6, 5, 5, 5, 5, 1, 1, 4, 4, 1, 1, 2, 3, 1, 1, 1, 1, 3, 1, 1, 1, 1, 3, 2, 2, 1, 1, 1, 1, 1, 1, 1, 1, 1, 1, 1, 1, 1, 1, 1, 1, 1, 1, 1

Phenopedia diseases ranked according to mean of effect sizes in cluster:
Hot Flashes | Social Perception | Choroid Diseases | Flushing | Amyotrophic Lateral Sclerosis | Geographic Atrophy | Motor Neuron Disease | Spinal Cord Diseases | Choroidal Neovascularization | Frontotemporal Lobar Degeneration | Frontotemporal Dementia | Vision Disorders

Corresponding mean of effect sizes of each Phenopedia disease:
(In the same order as above)
2.45, 1.94, 1.91, 1.82, 1.57, 1.31, 1.18, 1.17, 1.07, 0.63, 0.61, 0.42

Corresponding total number of genes of each Phenopedia disease:
(In the same order as above)
20, 67, 35, 20, 133, 21, 155, 177, 37, 27, 23, 50

Corresponding number of genes of each Phenopedia disease with at least one TF hit (of selected TFs):
(In the same order as above)
5, 18, 11, 6, 25, 2, 28, 37, 6, 8, 7, 5

Corresponding total number of TF hits (of selected TFs) for each Phenopedia disease (in all genes):
(In the same order as above)
28, 53, 47, 26, 102, 16, 106, 128, 22, 22, 18, 24

Corresponding number of selected TFs each Phenopedia disease is involved with:
(In the same order as above)
10, 10, 10, 10, 10, 10, 10, 10, 10, 10, 9, 10

TFs ranked according to mean of effect sizes in cluster:
V$HNF4_DR1_Q3, V$HNF4_Q6_01, V$COUP_01, V$COUP_DR1_Q6, V$PPAR_DR1_Q2, V$HNF4_01, V$DR1_Q3, V$COUPTF_Q6, V$PPARG_01, V$HNF4ALPHA_Q6

Corresponding mean of effect sizes of each TF:
(In the same order as above)
1.91, 1.64, 1.62, 1.48, 1.45, 1.24, 1.23, 1.22, 0.96, 0.65

Corresponding total number of TF hits for each TF (genome-wide):
(In the same order as above)
430, 444, 442, 438, 456, 438, 438, 426, 452, 450

Corresponding total number of TF hits for each TF (in all genes in selected Phenopedia diseases):
(In the same order as above)
75, 74, 64, 57, 60, 56, 60, 53, 48, 45

Corresponding number of genes (of selected Phenopedia diseases) each TF is involved with:
(In the same order as above)
27, 27, 23, 20, 22, 22, 23, 19, 21, 19

Corresponding number of selected Phenopedia diseases each TF is involved with:
(In the same order as above)
12, 12, 12, 12, 12, 12, 12, 12, 12, 11

Disease groups of selected Phenopedia diseases ranked according to sum of effect sizes
Nervous System Diseases | Central Nervous System Diseases | Uveal Diseases | Eye Diseases | Choroid Diseases | Motor Neuron Disease | Signs and Symptoms | Hot Flashes | Social Perception | Vascular Diseases | Skin Diseases, Vascular | Skin Diseases | Peripheral Vascular Diseases | Flushing | Cardiovascular Diseases | Amyotrophic Lateral Sclerosis | Geographic Atrophy | Spinal Diseases | Spinal Cord Diseases | Musculoskeletal Diseases | Bone Diseases | Neovascularization, Pathologic | Metaplasia | Choroidal Neovascularization | Frontotemporal Lobar Degeneration | Frontotemporal Dementia | Vision, Low | Vision Disorders

Corresponding sum of effect sizes for each disease group of selected Phenopedia diseases
(In the same order as above)
39.18, 39.18, 29.79, 29.79, 29.79, 27.46, 24.50, 24.50, 19.36, 18.15, 18.15, 18.15, 18.15, 18.15, 18.15, 15.66, 13.11, 11.72, 11.72, 11.72, 11.72, 10.68, 10.68, 10.68, 6.35, 6.10, 4.16, 4.16

Cluster for columns 943 to 950, rows 206 to 211

Phenopedia diseases

Conduct Disorder | Appetite | Hyperphagia | Thinness | Substance Withdrawal Syndrome | Delirium | Alcohol-Induced Disorders, Nervous System | Alcohol Withdrawal Delirium

TFs

V$MYOGNF1_01 | V$TAL1BETAITF2_01 | V$TAL1BETAE47_01 | V$TAL1ALPHAE47_01 | V$RFX1_01 | V$PAX6_01

Information

All related TFs:
(List of all TFs that are related to any of the PWMs)
E47, ITF-2, NF-1, NF-1/L, NF-1/Red1, NF-1A1, NF-1B1, NF-1B2, NF-1C2, NF-1X, Pax-6, Pax6-1, RFX1, Tal-1alpha, Tal-1beta

Ranked gene list:
(All genes of the selected Phenopedia diseases with hits of any of the selected TFs, ranked according to the total number of TF hits)
COMT, GRM7, HTR2A, DBH, SLC6A4, SLC6A3, NPY, LEPR, HTR1B, GABRB1, CNR1, CCK, GRM8, GRIK3, ESR2, DRD4, DRD3, ADRB3, IL6, HAAO, AGRP, TNF, SOAT1, SLC27A1, PRLHR, MTHFR, MAOA, INS, GPR55, CYP2E1, CHN1, ADORA2A, ACLY, UCP3, SREBF2, SLC1A2, SIM1, PUS10, PNMT, PAX6, NMB, MGAT5, INSIG1, HSD17B1, GABBR1, ESR1, DNMT3B, CARTPT, ANKK1, AHSG, ADM

Corresponding total number of TF hits:
(For each gene listed above, the total number of TF hits for any of the selected TFs, multiplied by the number of selected Phenopedia diseases containing that gene)
24, 12, 10, 10, 8, 7, 6, 6, 6, 6, 6, 5, 4, 4, 4, 4, 4, 4, 3, 3, 3, 2, 2, 2, 2, 2, 2, 2, 2, 2, 2, 2, 2, 1, 1, 1, 1, 1, 1, 1, 1, 1, 1, 1, 1, 1, 1, 1, 1, 1, 1

Corresponding number of selected Phenopedia diseases each gene is involved with:
(In the same order as above)
8, 4, 5, 5, 8, 7, 6, 3, 3, 2, 6, 5, 4, 4, 2, 4, 4, 1, 3, 1, 3, 2, 1, 1, 1, 2, 2, 2, 1, 1, 1, 1, 1, 1, 1, 1, 1, 1, 1, 1, 1, 1, 1, 1, 1, 1, 1, 1, 1, 1, 1

Corresponding number of selected TFs each gene is involved with:
(In the same order as above)
3, 3, 2, 2, 1, 1, 1, 2, 2, 3, 1, 1, 1, 1, 2, 1, 1, 4, 1, 3, 1, 1, 2, 2, 2, 1, 1, 1, 2, 2, 2, 2, 2, 1, 1, 1, 1, 1, 1, 1, 1, 1, 1, 1, 1, 1, 1, 1, 1, 1, 1

Phenopedia diseases ranked according to mean of effect sizes in cluster:
Alcohol Withdrawal Delirium | Hyperphagia | Alcohol-Induced Disorders, Nervous System | Thinness | Delirium | Substance Withdrawal Syndrome | Appetite | Conduct Disorder

Corresponding mean of effect sizes of each Phenopedia disease:
(In the same order as above)
3.26, 2.76, 2.73, 2.59, 2.13, 2.08, 1.87, 1.26

Corresponding total number of genes of each Phenopedia disease:

(In the same order as above)
21, 26, 31, 31, 27, 50, 74, 88

Corresponding number of genes of each Phenopedia disease with at least one TF hit (of selected TFs):
(In the same order as above)
10, 11, 13, 10, 14, 17, 26, 21

Corresponding total number of TF hits (of selected TFs) for each Phenopedia disease (in all genes):
(In the same order as above)
15, 17, 19, 17, 20, 25, 36, 32

Corresponding number of selected TFs each Phenopedia disease is involved with:
(In the same order as above)
6, 6, 6, 6, 6, 6, 6, 6

TFs ranked according to mean of effect sizes in cluster:
V$TAL1BETAE47_01, V$PAX6_01, V$TAL1BETAITF2_01, V$MYOGNF1_01, V$RFX1_01, V$TAL1ALPHAE47_01

Corresponding mean of effect sizes of each TF:
(In the same order as above)
3.17, 2.74, 2.61, 2.15, 1.72, 1.61

Corresponding total number of TF hits for each TF (genome-wide):
(In the same order as above)
463, 493, 494, 412, 423, 475

Corresponding total number of TF hits for each TF (in all genes in selected Phenopedia diseases):
(In the same order as above)
39, 34, 34, 28, 23, 23

Corresponding number of genes (of selected Phenopedia diseases) each TF is involved with:
(In the same order as above)
17, 14, 11, 14, 9, 10

Corresponding number of selected Phenopedia diseases each TF is involved with:
(In the same order as above)
8, 8, 8, 8, 8, 8

Disease groups of selected Phenopedia diseases ranked according to sum of effect sizes
Signs and Symptoms | Nervous System Diseases | Central Nervous System Diseases | Brain Diseases | Substance-Related Disorders | Alcohol-Related Disorders | Psychoses, Alcoholic | Hallucinations | Alcohol Withdrawal Delirium | Hyperphagia | Poisoning | Neurotoxicity Syndromes | Encephalitis | Alcohol-Induced Disorders, Nervous System | Alcohol-Induced Disorders | Thinness | Delirium | Confusion | Substance Withdrawal Syndrome | Appetite | Conduct Disorder

Corresponding sum of effect sizes for each disease group of selected Phenopedia diseases
(In the same order as above)
93.33, 48.75, 48.75, 48.75, 48.42, 35.95, 19.54, 19.54, 19.54, 16.55, 16.41, 16.41, 16.41, 16.41, 16.41, 15.56, 12.80, 12.80, 12.47, 11.19, 7.54

Cluster for columns 962 to 985, rows 202 to 211

Phenopedia diseases

Neurotic Disorders | Genetics, Behavioral | Reinforcement (C0035007) | Pain Threshold | Borderline Personality Disorder | Personality Assessment | Tourette Syndrome | Tic Disorders | Tics | Postmortem Changes | Psychopathology | Heroin Dependence | Opioid-Related Disorders | Antisocial Personality Disorder | Personality Disorders | Bulimia | Bulimia Nervosa | Stress Disorders, Traumatic | Reaction Time | Cues | Memory, Short-Term | Mental Health | Thinking | Hallucinations

TFs

V$PR_02 | V$GR_01 | V$PR_01 | V$SREBP1_02 | V$MYOGNF1_01 | V$TAL1BETAITF2_01 | V$TAL1BETAE47_01 | V$TAL1ALPHAE47_01 | V$RFX1_01 | V$PAX6_01

Information

All related TFs:
(List of all TFs that are related to any of the PWMs)
E47, GR, ITF-2, NF-1, NF-1/L, NF-1/Red1, NF-1A1, NF-1B1, NF-1B2, NF-1C2, NF-1X, PR, Pax-6, Pax6-1, RFX1, SREBP-1, SREBP-1a, SREBP-1b, SREBP-1c, Tal-1alpha, Tal-1beta

Ranked gene list:
(All genes of the selected Phenopedia diseases with hits of any of the selected TFs, ranked according to the total number of TF hits)
COMT, HTR2A, BDNF, HTR1B, DBH, NR3C1, SLC6A4, SLC6A3, DRD4, MAOA, NR4A2, AKT1, DRD3, EIF3D, CNR1, DISC1, DAOA, CRHR2, LBXCOR1, IL10RA, GBA, ANKK1, PSEN2, NOS1, CNTF, ESR1, SNAP25, OLIG1, LEPR, ESR2, AR, ADRA2C, NPY, ZDHHC8, VEGFA, UCP3, UCP2, TNF, TH, SREBF2, PER3, PDE1A, MYH9, MDGA1, IL6R, GNB1L, CYP2E1, ACP1, WNT2B, VDR, SLC1A2, ROBO4, RGS4, NOS3, MAP2K5, LIF, LEP, KCNN3, IL10, IGF2, HTT, HD, HCN1, GRID1, GDNF, GABBR1, WNT7A, TFAP2B, TCF4, SPATA17, RELN, PLXNA2, PLEKHB1, PDE4B, PARK2, OPRL1, NTF3, NR4A1, NOS1AP, NGFR, MDGA2, INSIG1, IL5, HTR3C, GSK3B, GRN, GRM8, GRIK3, EN2, DAO, CSNK1E, CCK, TTC12, TRPM2, SYNGR1, SREBF1, PRKG1, PNMT, OPRS1, NRGN, NOTCH4, NCOA3, MTHFR, MC1R, MBL2, KCNH2, INPP1, IMMP2L, IL6, IL4, HTR1E, GNPAT, GATA4, FYN, F7, EPM2A, DNMT3B, CXCL12, CHRNB1, CCR2, CBS, CARTPT, BCL2, ATP7B, ATF4, AHI1

Corresponding total number of TF hits:
(For each gene listed above, the total number of TF hits for any of the selected TFs, multiplied by the number of selected Phenopedia diseases containing that gene)
144, 76, 44, 28, 26, 24, 23, 22, 22, 18, 16, 16, 15, 14, 12, 10, 10, 10, 9, 9, 9, 9, 8, 8, 8, 7, 6, 6, 6, 6, 6, 6, 5, 4, 4, 4, 4, 4, 4, 4, 4, 4, 4, 4, 4, 4, 4, 4, 3, 3, 3, 3, 3, 3, 3, 3, 3, 3, 3, 3, 3, 3, 3, 3, 3, 2, 2, 2, 2, 2, 2, 2, 2, 2, 2, 2, 2, 2, 2, 2, 2, 2, 2, 2, 2, 2, 2, 2, 2, 2, 1, 1, 1, 1, 1, 1, 1, 1, 1, 1, 1, 1, 1, 1, 1, 1, 1, 1, 1, 1, 1, 1, 1, 1, 1, 1, 1, 1, 1, 1, 1, 1

Corresponding number of selected Phenopedia diseases each gene is involved with:
(In the same order as above)
24, 19, 22, 14, 13, 8, 23, 22, 22, 18, 4, 2, 15, 2, 12, 5, 5, 2, 3, 3, 3, 9, 4, 4, 2, 7, 3, 3, 3, 2, 6, 2, 5, 1, 1, 1, 1, 2, 4, 1, 4, 1, 2, 1, 1, 1, 2, 4, 1, 1, 3, 3, 3, 3, 3, 1, 3, 1, 1, 1, 3, 3, 3, 1, 3, 1, 1, 2, 1, 2, 2, 2, 1, 2, 2, 1, 2, 1, 1, 2, 2, 1, 1, 2, 2, 1, 2, 2, 1, 2, 2, 2, 1, 1, 1, 1, 1, 1, 1, 1, 1, 1, 1, 1, 1, 1, 1, 1, 1, 1, 1, 1, 1, 1, 1, 1, 1, 1, 1, 1, 1, 1, 1, 1, 1, 1

Corresponding number of selected TFs each gene is involved with:
(In the same order as above)
6, 4, 2, 2, 2, 3, 1, 1, 1, 1, 4, 8, 1, 7, 1, 2, 2, 5, 3, 3, 3, 1, 2, 2, 4, 1, 2, 2, 2, 3, 1, 3, 1, 4, 4, 4, 4, 2, 1, 4, 1, 4, 2, 4, 4, 4, 2, 1, 3, 3, 1, 1, 1, 1, 1, 3, 1, 3, 3, 3, 1, 1, 1, 3, 1, 3, 2, 1, 2, 1, 1, 1, 2, 1, 1, 2, 1, 2, 2, 1, 1, 2, 2, 1,

1, 2, 1, 1, 2, 1, 1, 1, 1, 1, 1, 1, 1, 1, 1, 1, 1, 1, 1, 1, 1, 1, 1, 1, 1, 1, 1, 1, 1, 1, 1, 1, 1, 1, 1, 1, 1, 1, 1, 1, 1, 1

Phenopedia diseases ranked according to mean of effect sizes in cluster:
Pain Threshold | Neurotic Disorders | Personality Assessment | Borderline Personality Disorder | Tics | Tic Disorders | Memory, Short-Term | Tourette Syndrome | Antisocial Personality Disorder | Reaction Time | Genetics, Behavioral | Personality Disorders | Bulimia | Stress Disorders, Traumatic | Cues | Mental Health | Psychopathology | Reinforcement (C0035007) | Hallucinations | Bulimia Nervosa | Thinking | Heroin Dependence | Postmortem Changes | Opioid-Related Disorders

Corresponding mean of effect sizes of each Phenopedia disease:
(In the same order as above)
2.36, 2.33, 2.25, 2.17, 2.16, 2.15, 2.06, 1.94, 1.84, 1.84, 1.79, 1.70, 1.69, 1.60, 1.56, 1.47, 1.46, 1.44, 1.40, 1.34, 1.32, 1.26, 1.26, 0.95

Corresponding total number of genes of each Phenopedia disease:
(In the same order as above)
22, 26, 25, 20, 42, 42, 26, 47, 36, 40, 20, 52, 37, 21, 30, 286, 73, 24, 50, 34, 45, 36, 28, 48

Corresponding number of genes of each Phenopedia disease with at least one TF hit (of selected TFs):
(In the same order as above)
11, 12, 11, 10, 20, 20, 14, 21, 17, 16, 11, 24, 16, 11, 14, 85, 28, 10, 15, 14, 14, 13, 9, 16

Corresponding total number of TF hits (of selected TFs) for each Phenopedia disease (in all genes):
(In the same order as above)
22, 26, 21, 20, 37, 37, 29, 38, 31, 30, 21, 40, 36, 23, 24, 172, 51, 17, 30, 29, 28, 26, 20, 30

Corresponding number of selected TFs each Phenopedia disease is involved with:
(In the same order as above)
10, 10, 9, 9, 10, 10, 10, 10, 10, 10, 10, 10, 10, 10, 10, 10, 10, 9, 10, 10, 10, 10, 10, 10

TFs ranked according to mean of effect sizes in cluster:
V$TAL1BETAITF2_01, V$TAL1BETAE47_01, VPR_02, VMYOGNF1_01, V$RFX1_01, V$TAL1ALPHAE47_01, V$SREBP1_02, V$PR_01, V$PAX6_01, V$GR_01

Corresponding mean of effect sizes of each TF:
(In the same order as above)
3.10, 2.71, 2.00, 1.89, 1.71, 1.38, 1.36, 1.30, 1.23, 0.57

Corresponding total number of TF hits for each TF (genome-wide):
(In the same order as above)
494, 463, 487, 412, 423, 475, 468, 490, 493, 476

Corresponding total number of TF hits for each TF (in all genes in selected Phenopedia diseases):
(In the same order as above)
120, 116, 95, 80, 76, 70, 73, 80, 75, 53

Corresponding number of genes (of selected Phenopedia diseases) each TF is involved with:
(In the same order as above)
23, 27, 28, 24, 19, 19, 21, 26, 28, 20

Corresponding number of selected Phenopedia diseases each TF is involved with:
(In the same order as above)
24, 24, 24, 24, 24, 23, 24, 24, 22, 24

Disease groups of selected Phenopedia diseases ranked according to sum of effect sizes

Signs and Symptoms | Nervous System Diseases | Central Nervous System Diseases | Brain Diseases | Tic Disorders | Stomatognathic Diseases | Neurologic Manifestations | Musculoskeletal Diseases | Movement Disorders | Mouth Diseases | Jaw Diseases | Basal Ganglia Diseases | Pain Threshold | Neurotic Disorders | Personality Assessment | Substance-Related Disorders | Opioid-Related Disorders | Borderline Personality Disorder | Tics | Memory, Short-Term | Tourette Syndrome | Antisocial Personality Disorder | Reaction Time | Genetics, Behavioral | Personality Disorders | Hyperphagia | Bulimia | Stress Disorders, Traumatic | Cues | Mental Health | Psychopathology | Reinforcement (C0035007) | Hallucinations | Bulimia Nervosa | Thinking | Heroin Dependence | Postmortem Changes | Death

Corresponding sum of effect sizes for each disease group of selected Phenopedia diseases
(In the same order as above)
115.56, 76.51, 76.51, 76.51, 62.52, 62.52, 62.52, 62.52, 62.52, 62.52, 62.52, 62.52, 23.60, 23.30, 22.54, 22.16, 22.16, 21.68, 21.60, 20.57, 19.40, 18.36, 18.35, 17.90, 17.03, 16.88, 16.88, 16.05, 15.58, 14.70, 14.56, 14.37, 14.00, 13.37, 13.25, 12.62, 12.56, 12.56

Cluster for columns 601 to 604, rows 106 to 107

Phenopedia diseases

Hyperpigmentation | Melanosis | Developmental Disabilities | Facies

TFs

V$P53_DECAMER_Q2 | V$P53_02

Information

All related TFs:
(List of all TFs that are related to any of the PWMs)
DeltaNp63alpha, p53, p53-isoform-1, p63alpha, p63gamma, p73alpha, p73beta

Ranked gene list:
(All genes of the selected Phenopedia diseases with hits of any of the selected TFs, ranked according to the total number of TF hits)
SOS1, TNF, STK11, RAF1, OCA2, LOXL1, ADRB2, RARA, HRAS, DHCR7, VEGFA, VEGF, IL4R

Corresponding total number of TF hits:
(For each gene listed above, the total number of TF hits for any of the selected TFs, multiplied by the number of selected Phenopedia diseases containing that gene)
8, 4, 4, 4, 4, 4, 3, 2, 2, 2, 1, 1, 1

Corresponding number of selected Phenopedia diseases each gene is involved with:
(In the same order as above)
4, 2, 2, 4, 2, 2, 3, 1, 1, 1, 1, 1, 1

Corresponding number of selected TFs each gene is involved with:
(In the same order as above)
2, 2, 2, 1, 2, 2, 1, 2, 2, 2, 1, 1, 1

Phenopedia diseases ranked according to mean of effect sizes in cluster:
Hyperpigmentation | Melanosis | Facies | Developmental Disabilities

Corresponding mean of effect sizes of each Phenopedia disease:

(In the same order as above)
3.56, 2.82, 1.57, 1.23

Corresponding total number of genes of each Phenopedia disease:
(In the same order as above)
24, 20, 57, 41

Corresponding number of genes of each Phenopedia disease with at least one TF hit (of selected TFs):
(In the same order as above)
6, 5, 7, 7

Corresponding total number of TF hits (of selected TFs) for each Phenopedia disease (in all genes):
(In the same order as above)
10, 8, 12, 10

Corresponding number of selected TFs each Phenopedia disease is involved with:
(In the same order as above)
2, 2, 2, 2

TFs ranked according to mean of effect sizes in cluster:
V$P53_02, V$P53_DECAMER_Q2

Corresponding mean of effect sizes of each TF:
(In the same order as above)
2.93, 1.66

Corresponding total number of TF hits for each TF (genome-wide):
(In the same order as above)
469, 440

Corresponding total number of TF hits for each TF (in all genes in selected Phenopedia diseases):
(In the same order as above)
23, 17

Corresponding number of genes (of selected Phenopedia diseases) each TF is involved with:
(In the same order as above)
11, 10

Corresponding number of selected Phenopedia diseases each TF is involved with:
(In the same order as above)
4, 4

Disease groups of selected Phenopedia diseases ranked according to sum of effect sizes
Vascular Diseases | Skin Diseases, Vascular | Skin Diseases | Signs and Symptoms | Pigmentation Disorders | Peripheral Vascular Diseases | Cardiovascular Diseases | Hyperpigmentation | Melanosis | Facies | Developmental Disabilities

Corresponding sum of effect sizes for each disease group of selected Phenopedia diseases
(In the same order as above)
12.76, 12.76, 12.76, 12.76, 12.76, 12.76, 12.76, 7.13, 5.64, 3.14, 2.45

Cluster for columns 592 to 595, rows 145 to 174

Phenopedia diseases

Graves Disease | Orbital Diseases | Goiter | Hyperthyroidism

TFs

V$GATA_Q6 | V$GATA6_01 | B$CRP_C | V$PAX2_02 | V$AREB6_04 | V$BRCA_01 | V$STAT5A_04 | V$STAT5A_03 | V$STAT4_01 | V$STAT6_01 | V$STAT1_03 | V$HMGIY_Q6 | V$TST1_01 | V$HOXA4_Q2 | V$TCF4_Q5 | V$LEF1_Q2 | V$SOX10_Q6 | V$DBP_Q6 | V$CEBP_Q3 | V$HSF2_01 | V$HSF1_01 | V$NKX25_Q5 | V$ELF1_Q6 | V$CEBP_Q2 | V$CEBPB_02 | V$CEBP_Q2_01 | V$CEBPB_01 | V$CEBPA_01 | V$NFAT_Q6 | V$NFAT_Q4_01

Information

All related TFs:
(List of all TFs that are related to any of the PWMs)
ANF-2, BRCA1, BRCA1:USF2, C/EBP, C/EBPalpha, C/EBPalpha(p20), C/EBPalpha(p30), C/EBPbeta, C/EBPbeta(LAP), C/EBPbeta(p20), C/EBPbeta(p34), C/EBPbeta(p35), C/EBPdelta, C/EBPepsilon, C/EBPgamma, DBP, ELF-1, Elf-1, GATA-1, GATA-1A, GATA-2, GATA-3, GATA-4, GATA-5, GATA-5A, GATA-5B, GATA-6, GATA-6A, GATA-6B, HMG, HMG-Y, HMGI-C, HOXA4, HSF1, HSF1-L, HSF1-S, HSF1long, HSF1short, HSF2, HSF2A, LEF-1, LEF-1S, NF-AT, NF-AT1, NF-AT1C, NF-AT2, NF-AT3, NF-AT4, Nkx2-5, Nkx2.5, POU3F1, Pax-2, Pax-2.1, Pax-2.2, Pax-2a, Pax-2b, STAT1, STAT1alpha, STAT4, STAT5A, STAT6, Sox10, TCF-1, TCF-1(P), TCF-3, TCF-4, ZEB

Ranked gene list:
(All genes of the selected Phenopedia diseases with hits of any of the selected TFs, ranked according to the total number of TF hits)
IRF1, TAP1, TNF, LTA, ATP1A1, IL16, CTLA4, TAP2, GNAS, NR3C1, VDR, TG, DBP, ADRB2, ATP1B1, STAT6, CYP27B1, CBLB, HSPA1L, RUNX1, IL2RA, APEX1, NFKBIA, LAG3, SLC9A3R1, FAS, ESR1, TNFRSF1A, PTPN22, IL6, HLA-B, PDCD1, NFKB1, IFIH1, HLA-DPB1, MYC, HLA-C, IL1RAP, TP53, SELL, PTPN12, MAP3K7IP2, ITPR3, IL2, IGF1, HDLBP, CD40, BRCA2, TNFRSF1B, SLC22A4, PSMA6, NOD2, LTC4S, CARD15, BRCA1, BRAF

Corresponding total number of TF hits:
(For each gene listed above, the total number of TF hits for any of the selected TFs, multiplied by the number of selected Phenopedia diseases containing that gene)
116, 104, 100, 96, 84, 72, 68, 64, 58, 56, 52, 52, 48, 48, 40, 36, 36, 36, 32, 24, 20, 20, 19, 19, 16, 16, 16, 14, 12, 12, 12, 8, 8, 8, 8, 6, 6, 5, 4, 4, 4, 4, 4, 4, 4, 4, 4, 3, 2, 1, 1, 1, 1, 1, 1, 1

Corresponding number of selected Phenopedia diseases each gene is involved with:
(In the same order as above)
4, 4, 4, 4, 4, 4, 4, 4, 2, 4, 4, 4, 4, 4, 4, 4, 4, 4, 4, 1, 4, 4, 1, 1, 1, 4, 4, 1, 4, 4, 4, 4, 4, 4, 4, 1, 2, 1, 4, 4, 4, 4, 4, 4, 1, 1, 4, 1, 1, 1, 1, 1, 1, 1, 1, 1

Corresponding number of selected TFs each gene is involved with:
(In the same order as above)
29, 26, 25, 24, 21, 18, 17, 16, 29, 14, 13, 13, 12, 12, 10, 9, 9, 9, 8, 24, 5, 5, 19, 19, 16, 4, 4, 14, 3, 3, 3, 2, 2, 2, 2, 6, 3, 5, 1, 1, 1, 1, 1, 1, 4, 4, 1, 3, 2, 1, 1, 1, 1, 1, 1, 1

Phenopedia diseases ranked according to mean of effect sizes in cluster:
Graves Disease | Goiter | Hyperthyroidism | Orbital Diseases

Corresponding mean of effect sizes of each Phenopedia disease:
(In the same order as above)

1.67, 1.40, 1.37, 1.36

Corresponding total number of genes of each Phenopedia disease:
(In the same order as above)
125, 103, 103, 92

Corresponding number of genes of each Phenopedia disease with at least one TF hit (of selected TFs):
(In the same order as above)
48, 40, 41, 37

Corresponding total number of TF hits (of selected TFs) for each Phenopedia disease (in all genes):
(In the same order as above)
434, 366, 367, 328

Corresponding number of selected TFs each Phenopedia disease is involved with:
(In the same order as above)
30, 30, 30, 30

TFs ranked according to mean of effect sizes in cluster:
V$NFAT_Q6, V$STAT6_01, V$CEBP_Q3, V$NFAT_Q4_01, V$CEBPA_01, V$GATA6_01, V$CEBP_Q2, V$STAT5A_03, V$STAT4_01, V$STAT5A_04, V$HMGIY_Q6, V$CEBP_Q2_01, V$PAX2_02, V$GATA_Q6, V$ELF1_Q6, V$SOX10_Q6, V$HOXA4_Q2, V$TCF4_Q5, V$CEBPB_02, V$DBP_Q6, V$CEBPB_01, V$AREB6_04, V$STAT1_03, V$LEF1_Q2, V$HSF1_01, V$TST1_01, BCRP_C, VBRCA_01, V$HSF2_01, V$NKX25_Q5

Corresponding mean of effect sizes of each TF:
(In the same order as above)
3.06, 2.76, 2.60, 2.38, 2.33, 2.23, 1.95, 1.90, 1.88, 1.73, 1.62, 1.60, 1.59, 1.59, 1.44, 1.42, 1.40, 1.17, 1.14, 1.08, 1.04, 0.90, 0.89, 0.87, 0.84, 0.76, 0.73, 0.71, 0.23, -0.33

Corresponding total number of TF hits for each TF (genome-wide):
(In the same order as above)
540, 520, 500, 536, 528, 512, 537, 527, 523, 509, 564, 533, 529, 520, 542, 511, 526, 531, 486, 519, 500, 480, 502, 556, 469, 548, 497, 474, 442, 455

Corresponding total number of TF hits for each TF (in all genes in selected Phenopedia diseases):
(In the same order as above)
80, 64, 68, 67, 64, 59, 54, 55, 55, 51, 55, 53, 51, 50, 49, 49, 48, 44, 46, 46, 46, 41, 43, 44, 43, 42, 40, 37, 28, 23

Corresponding number of genes (of selected Phenopedia diseases) each TF is involved with:
(In the same order as above)
27, 18, 22, 20, 21, 16, 17, 18, 18, 17, 18, 19, 17, 13, 18, 15, 14, 13, 18, 15, 18, 13, 15, 13, 14, 14, 12, 12, 9, 8

Corresponding number of selected Phenopedia diseases each TF is involved with:
(In the same order as above)
4, 4, 4, 4, 4, 4, 4, 4, 4, 4, 4, 4, 4, 4, 4, 4, 4, 4, 4, 4, 4, 4, 4, 4, 4, 4, 4, 4, 4, 4

Disease groups of selected Phenopedia diseases ranked according to sum of effect sizes
Thyroid Diseases | Endocrine System Diseases | Goiter | Hyperthyroidism | Orbital Diseases | Eye Diseases | Immune System Diseases | Graves Disease | Exophthalmos | Autoimmune Diseases

Corresponding sum of effect sizes for each disease group of selected Phenopedia diseases
(In the same order as above)

133.22, 133.22, 92.13, 91.29, 90.95, 90.95, 50.20, 50.20, 50.20, 50.20

Cluster for columns 409 to 524, rows 196 to 201

Phenopedia diseases

Dyspepsia | Vesico-Ureteral Reflux | Urinary Tract Infections | Arthralgia | Stomach Ulcer | Enterocolitis | Idiopathic Pulmonary Fibrosis | Pulmonary Fibrosis | Lipodystrophy | Shock | Viremia | Sepsis | Systemic Inflammatory Response Syndrome | Rubella | Measles | Mumps | Tuberculosis | Mycobacterium Infections | Tuberculosis, Pulmonary | Bacterial Infections | Gram-Negative Bacterial Infections | Malaria | Parasitic Diseases | Bronchiectasis | Helminthiasis | Brucellosis | Coronary Aneurysm | Salmonella Infections | Shock, Septic | Myelitis | Deltaretrovirus Infections | HTLV-I Infections | Spinal Diseases | Spondylitis | Ankylosis | Spondylitis, Ankylosing | Spondylarthritis | Spondylarthropathies | Vasculitis | Uveitis | Behcet Syndrome | Panuveitis | Uveitis, Anterior | Arthritis, Psoriatic | Psoriasis | Stomatitis | Epidermal Necrolysis, Toxic | Stevens-Johnson Syndrome | Erythema | Erythema Nodosum | Drug Eruptions | Drug Hypersensitivity | Encephalitis | Severe Acute Respiratory Syndrome | Meningitis | Nasal Polyps | Scleroderma, Systemic | Salivary Gland Diseases | Sjogren's Syndrome | Polymyalgia Rheumatica | Arteritis | Temporal Arteritis | Graft vs Host Disease | Purpura, Schoenlein-Henoch | Immune Complex Diseases | Vasculitis, Hypersensitivity | Streptococcal Infections | Rheumatic Fever | Rheumatic Heart Disease | Hematologic Neoplasms | Leprosy | Pneumoconiosis | Silicosis | Endometriosis | Leukemia, T-Cell, Acute | Sarcoidosis | Mycobacterium Infections, Atypical | Sarcoidosis, Pulmonary | Pregnancy Complications, Infectious | Vaginal Diseases | Vaginosis, Bacterial | Malaria, Cerebral | Malaria, Falciparum | Mycoses | Chagas Cardiomyopathy | Chagas Disease | Parasitemia | Aspergillosis | Lung Diseases, Fungal | Cytomegalovirus Infections | Opportunistic Infections | Hepatitis, Autoimmune | Urticaria | Respiratory Sounds | Paranasal Sinus Diseases | Sinusitis | Albuminuria | Conjunctival Diseases | Corneal Diseases | Conjunctivitis | Chlamydia Infections | Eye Infections, Bacterial | Trachoma | Exanthema | Liver Failure | Food Hypersensitivity | Leptospirosis | Picornaviridae Infections | Gingival Diseases | Sarcoma, Kaposi | Pemphigus | Skin Diseases, Vesiculobullous | Thymoma | Oral Submucous Fibrosis | Purpura, Thrombocytopenic | Purpura, Thrombocytopenic, Idiopathic

TFs

V$NFKB_C | V$NFKAPPAB_01 | V$NFKAPPAB65_01 | V$CREL_01 | V$NFKB_Q6_01 | V$NFKB_Q6

Information

All related TFs:
(List of all TFs that are related to any of the PWMs)
NF-TNF, NF-kappaB, NF-kappaB(-like), NF-kappaB2, RelA-p65, c-Rel, p100, p105, p50, p52

Ranked gene list:
(All genes of the selected Phenopedia diseases with hits of any of the selected TFs, ranked according to the total number of TF hits)
TNF, LTA, TGFB1, NOD2, CARD15, HLA-B, HLA-A, NFKB1, HLA-C, VDR, IL1RN, IRF1, IRF5, GSTP1, TNFAIP3, TAP1, TRAF1, PSMB9, NFKBIA, IL4R, TNFRSF1B, IL12B, TPMT, VEGFA, PSMB8, PDCD1, LST1, CXCL10, STAT1, TRADD, TAP2, STAT6, VEGF, MIF, HSPA1L, FAM167A, CD40, AKT1, NFKBIZ, DUOX2, COL6A1, FAS, LTB, HFE, PTGS1, CXCL5, CD4, BDNF, TNFAIP1, PROZ, CISH, SERPINA1, CYBA, XRCC1, PLAU, NR3C1, NOTCH4, NFKBIE, IL7R, DDAH2, CYP27B1, RUNX3, PSORS1, NFKBIB, IL2RA, IFIH1, AGER, ADA, TCF7, PARP1, IFNGR2, HMHA1, AIRE, RXRB, ITGB3, FGF1, ERAP1, CD86, TNIP1, TNFRSF6B, PGC, NFKB2, LMNA, CRHR1, CCND1, BRAF, RUNX1, PAX2, HIF1A, ERCC2, TPI1, TNFRSF11B, SH2B3, RARG, NRAS, NAT9, LAG3, ITGAM, IL23A, IGF1R, EGR2, CYSLTR1, COL11A2, ADORA2A, MYH9, MYD88,

MUC1, MAP3K8, ITPKC, IL19, CD58, CARD8, ALOX5AP, ALDH1B1, TNFRSF14, TBXA2R, SNAPC4, SERPINH1, NOTCH1, JAK1, IL21R, IL17RE, IL16, GATA2, ENG, DNMT3B, CXCL2, CHST11, CDKN2A, CDK6, CCL21, ZNF148, TRIM5, STAT3, SIM2, RET, RARA, PLAUR, MMEL1, MATN1, MAN1B1, LPIN1, JAK3, GFRA1, GDNF, FZD5, CX3CL1, CLOCK, CLEC16A, CHST12, CCL1, ALOXE3, AHR

Corresponding total number of TF hits:
(For each gene listed above, the total number of TF hits for any of the selected TFs, multiplied by the number of selected Phenopedia diseases containing that gene)
696, 438, 380, 234, 210, 188, 168, 156, 126, 123, 83, 80, 78, 76, 65, 56, 52, 50, 48, 47, 44, 43, 42, 40, 40, 40, 36, 36, 32, 30, 30, 30, 27, 26, 25, 25, 24, 24, 21, 21, 21, 19, 18, 17, 16, 16, 16, 15, 14, 14, 14, 13, 13, 12, 12, 12, 12, 12, 12, 12, 12, 10, 10, 10, 10, 10, 10, 9, 9, 9, 9, 9, 8, 8, 8, 7, 7, 6, 6, 6, 6, 6, 6, 6, 6, 5, 5, 5, 5, 4, 4, 4, 4, 4, 4, 4, 4, 4, 4, 4, 4, 4, 4, 3, 3, 3, 3, 3, 3, 3, 3, 3, 3, 2, 2, 2, 2, 2, 2, 2, 2, 2, 2, 2, 2, 2, 2, 2, 2, 1, 1, 1, 1, 1, 1, 1, 1, 1, 1, 1, 1, 1, 1, 1, 1, 1, 1, 1, 1

Corresponding number of selected Phenopedia diseases each gene is involved with:
(In the same order as above)
116, 73, 76, 39, 35, 94, 84, 26, 63, 41, 83, 16, 13, 19, 13, 28, 13, 10, 8, 47, 44, 43, 7, 40, 8, 8, 6, 9, 8, 6, 30, 5, 27, 26, 25, 5, 8, 4, 7, 7, 7, 19, 3, 17, 4, 8, 4, 5, 7, 7, 7, 13, 13, 6, 6, 6, 4, 2, 4, 4, 6, 2, 5, 2, 5, 2, 2, 5, 9, 3, 9, 3, 3, 2, 8, 8, 7, 7, 1, 1, 3, 1, 2, 3, 2, 2, 5, 5, 5, 5, 2, 4, 1, 1, 2, 1, 1, 4, 2, 4, 1, 4, 2, 1, 1, 3, 3, 1, 1, 1, 1, 3, 3, 1, 1, 1, 1, 1, 1, 1, 1, 1, 1, 1, 2, 2, 1, 2, 2, 2, 1, 1, 1, 1, 1, 1, 1, 1, 1, 1, 1, 1, 1, 1, 1, 1, 1, 1, 1, 1

Corresponding number of selected TFs each gene is involved with:
(In the same order as above)
6, 6, 5, 6, 6, 2, 2, 6, 2, 3, 1, 5, 6, 4, 5, 2, 4, 5, 6, 1, 1, 1, 6, 1, 5, 5, 6, 4, 4, 5, 1, 6, 1, 1, 1, 5, 3, 6, 3, 3, 3, 1, 6, 1, 4, 2, 4, 3, 2, 2, 2, 1, 1, 2, 2, 2, 3, 6, 3, 3, 2, 5, 2, 5, 2, 5, 5, 2, 1, 3, 1, 3, 3, 4, 1, 1, 1, 1, 6, 6, 2, 6, 3, 2, 3, 3, 1, 1, 1, 1, 2, 1, 4, 4, 2, 4, 4, 1, 2, 1, 4, 1, 2, 4, 3, 1, 1, 3, 3, 3, 3, 1, 1, 3, 2, 2, 2, 2, 2, 2, 2, 2, 2, 2, 2, 1, 1, 2, 1, 1, 1, 1, 1, 1, 1, 1, 1, 1, 1, 1, 1, 1, 1, 1, 1, 1, 1, 1, 1, 1

Phenopedia diseases ranked according to mean of effect sizes in cluster:
Arteritis | Spondylitis | Drug Hypersensitivity | Sarcoidosis, Pulmonary | Temporal Arteritis | Hepatitis, Autoimmune | Pemphigus | Drug Eruptions | Skin Diseases, Vesiculobullous | Sarcoidosis | Erythema Nodosum | Dyspepsia | Polymyalgia Rheumatica | Food Hypersensitivity | Leprosy | Streptococcal Infections | Uveitis | Purpura, Thrombocytopenic, Idiopathic | Erythema | Silicosis | Hematologic Neoplasms | Spondylitis, Ankylosing | Ankylosis | Pneumoconiosis | Rheumatic Heart Disease | Leptospirosis | Encephalitis | Spondylarthropathies | Vasculitis | Rheumatic Fever | Liver Failure | Spondylarthritis | Nasal Polyps | Gingival Diseases | Arthralgia | Behcet Syndrome | Idiopathic Pulmonary Fibrosis | Uveitis, Anterior | Purpura, Thrombocytopenic | Stomach Ulcer | Panuveitis | Psoriasis | Sinusitis | Oral Submucous Fibrosis | Tuberculosis, Pulmonary | Bacterial Infections | Stevens-Johnson Syndrome | Epidermal Necrolysis, Toxic | Respiratory Sounds | Graft vs Host Disease | Malaria, Falciparum | Mycoses | Scleroderma, Systemic | Paranasal Sinus Diseases | Pregnancy Complications, Infectious | Urinary Tract Infections | Pulmonary Fibrosis | Arthritis, Psoriatic | Vesico-Ureteral Reflux | Severe Acute Respiratory Syndrome | Exanthema | Enterocolitis | Vaginosis, Bacterial | Spinal Diseases | Aspergillosis | Mycobacterium Infections | Viremia | Mycobacterium Infections, Atypical | Brucellosis | Chagas Disease | Helminthiasis | Gram-Negative Bacterial Infections | Leukemia, T-Cell, Acute | Chagas Cardiomyopathy | Sepsis | Vaginal Diseases | Urticaria | Salmonella Infections | Malaria | Measles | Tuberculosis | Lipodystrophy | Salivary Gland Diseases | Myelitis | Bronchiectasis | Conjunctivitis | Thymoma | Endometriosis | Sarcoma, Kaposi | Picornaviridae Infections | Conjunctival Diseases | Purpura, Schoenlein-Henoch | Vasculitis, Hypersensitivity | Immune Complex Diseases | Coronary Aneurysm | Parasitemia | Sjogren's Syndrome | Systemic Inflammatory Response Syndrome | Lung Diseases, Fungal | HTLV-I Infections | Parasitic Diseases | Meningitis | Cytomegalovirus Infections | Stomatitis | Deltaretrovirus Infections | Opportunistic Infections | Shock | Mumps | Shock, Septic | Eye Infections, Bacterial | Trachoma | Malaria, Cerebral | Chlamydia Infections | Rubella | Corneal Diseases | Albuminuria

Corresponding mean of effect sizes of each Phenopedia disease:

(In the same order as above)
2.81, 2.72, 2.65, 2.61, 2.57, 2.45, 2.43, 2.40, 2.25, 2.22, 2.19, 2.11, 2.09, 2.08, 1.99, 1.86, 1.86, 1.79, 1.75, 1.73, 1.72, 1.71, 1.69, 1.68, 1.66, 1.63, 1.61, 1.60, 1.60, 1.59, 1.59, 1.56, 1.56, 1.51, 1.49, 1.48, 1.47, 1.45, 1.44, 1.43, 1.42, 1.37, 1.28, 1.27, 1.26, 1.26, 1.25, 1.25, 1.25, 1.24, 1.24, 1.24, 1.23, 1.21, 1.21, 1.21, 1.20, 1.14, 1.12, 1.12, 1.11, 1.09, 1.09, 1.08, 1.08, 1.07, 1.06, 1.06, 1.03, 1.03, 1.03, 1.00, 0.99, 0.99, 0.98, 0.94, 0.94, 0.92, 0.90, 0.89, 0.89, 0.86, 0.82, 0.81, 0.79, 0.77, 0.76, 0.75, 0.75, 0.75, 0.72, 0.71, 0.69, 0.69, 0.69, 0.65, 0.62, 0.59, 0.58, 0.56, 0.52, 0.50, 0.50, 0.44, 0.43, 0.39, 0.28, 0.23, 0.22, 0.20, 0.19, 0.18, 0.18, 0.11, 0.09, -0.29

Corresponding total number of genes of each Phenopedia disease:
(In the same order as above)
47, 99, 84, 46, 38, 22, 34, 52, 53, 100, 38, 41, 27, 24, 50, 49, 110, 24, 67, 20, 57, 72, 73, 50, 27, 25, 42, 106, 210, 30, 23, 111, 25, 32, 21, 95, 36, 99, 31, 32, 102, 155, 27, 24, 93, 300, 21, 20, 31, 81, 68, 46, 84, 28, 44, 32, 61, 55, 21, 37, 20, 19, 30, 176, 22, 142, 58, 22, 21, 36, 34, 197, 25, 22, 185, 32, 38, 25, 139, 46, 159, 23, 61, 22, 36, 47, 25, 105, 18, 20, 51, 33, 34, 35, 21, 29, 45, 149, 23, 29, 144, 37, 34, 36, 30, 38, 112, 32, 26, 39, 41, 39, 48, 48, 51, 111

Corresponding number of genes of each Phenopedia disease with at least one TF hit (of selected TFs):
(In the same order as above)
17, 36, 24, 14, 14, 11, 13, 15, 14, 29, 16, 10, 8, 7, 9, 12, 25, 8, 23, 8, 15, 21, 21, 13, 8, 8, 13, 31, 44, 8, 9, 31, 8, 7, 8, 20, 12, 20, 8, 8, 20, 39, 8, 7, 20, 55, 7, 7, 6, 19, 11, 10, 20, 8, 8, 7, 15, 19, 8, 6, 6, 7, 6, 38, 6, 24, 10, 7, 8, 10, 7, 38, 9, 7, 33, 6, 9, 6, 26, 15, 27, 9, 14, 9, 8, 11, 7, 23, 3, 5, 13, 9, 9, 9, 6, 6, 8, 27, 5, 10, 26, 5, 6, 9, 10, 5, 21, 10, 4, 7, 7, 5, 8, 15, 8, 13

Corresponding total number of TF hits (of selected TFs) for each Phenopedia disease (in all genes):
(In the same order as above)
49, 106, 73, 49, 41, 37, 40, 48, 43, 73, 49, 35, 27, 26, 33, 45, 79, 25, 60, 25, 48, 69, 69, 36, 27, 20, 36, 88, 138, 27, 26, 88, 23, 24, 27, 68, 41, 68, 25, 26, 68, 114, 26, 24, 56, 148, 17, 17, 23, 59, 35, 27, 52, 26, 27, 23, 51, 50, 17, 17, 18, 26, 22, 101, 19, 68, 34, 18, 27, 27, 27, 97, 33, 20, 93, 22, 23, 21, 75, 33, 78, 24, 37, 24, 21, 40, 25, 63, 10, 13, 42, 20, 20, 20, 14, 16, 27, 78, 15, 25, 74, 14, 14, 19, 25, 17, 52, 19, 16, 24, 24, 12, 25, 30, 25, 35

Corresponding number of selected TFs each Phenopedia disease is involved with:
(In the same order as above)
6, 6

TFs ranked according to mean of effect sizes in cluster:
V$NFKB_C, V$NFKAPPAB_01, V$NFKB_Q6, V$CREL_01, V$NFKAPPAB65_01, V$NFKB_Q6_01

Corresponding mean of effect sizes of each TF:
(In the same order as above)
1.74, 1.65, 1.12, 1.06, 0.92, 0.74

Corresponding total number of TF hits for each TF (genome-wide):
(In the same order as above)
472, 477, 465, 456, 488, 467

Corresponding total number of TF hits for each TF (in all genes in selected Phenopedia diseases):
(In the same order as above)
913, 906, 818, 726, 707, 585

Corresponding number of genes (of selected Phenopedia diseases) each TF is involved with:
(In the same order as above)

69, 70, 62, 68, 71, 55

Corresponding number of selected Phenopedia diseases each TF is involved with:
(In the same order as above)
116, 116, 116, 116, 116, 116

Disease groups of selected Phenopedia diseases ranked according to sum of effect sizes
Signs and Symptoms | Communicable Diseases | Musculoskeletal Diseases | Skin Diseases | Respiratory Tract Diseases | Bacterial Infections | Thoracic Diseases | Exanthema | Immune System Diseases | Gram-Positive Bacterial Infections | Cardiovascular Diseases | Lung Diseases | Connective Tissue Diseases | Pneumonia | Spinal Diseases | Bone Diseases | Arthritis | Hypersensitivity | Eye Diseases | Vascular Diseases | Uveitis | Uveal Diseases | Endophthalmitis | Spondylarthropathies | Vasculitis | Digestive System Diseases | Erythema | Mycobacterium Infections | Actinomycetales Infections | Pulmonary Fibrosis | Lung Diseases, Interstitial | Spondylarthritis | Virus Diseases | Hypersensitivity, Immediate | Streptococcal Infections | Poisoning | Drug Toxicity | Sex Chromosome Disorders | Sarcoidosis | Lymphoproliferative Disorders | Genetic Diseases, X-Linked | Genetic Diseases, Inborn | Female Urogenital Diseases | Infection | Skin Diseases, Vesiculobullous | Spondylitis | Osteitis | Protozoan Infections | Parasitic Diseases | Mediastinal Diseases | Otorhinolaryngologic Diseases | Nose Diseases | Liver Diseases | Collagen Diseases | Purpura | Hemorrhage | Blood Coagulation Disorders | Stomatognathic Diseases | Mouth Diseases | Jaw Diseases | Gram-Negative Bacterial Infections | Respiratory Hypersensitivity | Pneumoconiosis | Alveolitis, Extrinsic Allergic | Systemic Inflammatory Response Syndrome | Sepsis | Rheumatic Fever | Rheumatic Diseases | Thrombocytopenia | Purpura, Thrombocytopenic | Blood Platelet Disorders | Neoplasms | Autoimmune Diseases | Polyps | Uveitis, Anterior | Nervous System Diseases | Central Nervous System Diseases | Mycoses | Arteritis | Genital Diseases, Female | Drug Hypersensitivity | Cardiomyopathies | Sarcoidosis, Pulmonary | Temporal Arteritis | Stomach Diseases | Gastrointestinal Diseases | Skin Diseases, Papulosquamous | Psoriasis | Paranasal Sinus Diseases | Hematologic Neoplasms | Hepatitis, Autoimmune | Hepatitis | Pemphigus | Drug Eruptions | Dermatitis | Malaria | Coccidiosis | Peripheral Vascular Diseases | Erythema Nodosum | Tuberculosis | Dyspepsia | Polymyalgia Rheumatica | Food Hypersensitivity | Urologic Diseases | Male Urogenital Diseases | Vaginal Diseases | Trypanosomiasis | Sarcomastigophora Infections | Mastigophora Infections | Chagas Disease | Leprosy | Purpura, Thrombocytopenic, Idiopathic | Silicosis | Spondylitis, Ankylosing | Ankylosis | Conjunctival Diseases | Rheumatic Heart Disease | Myocarditis | Mediastinitis | Spirochaetales Infections | Leptospirosis | Encephalitis | Brain Diseases | Liver Failure | Nasal Polyps | Gingivitis | Gingival Overgrowth | Gingival Diseases | Neurologic Manifestations | Arthralgia | Skin Diseases, Vascular | Behcet Syndrome | Idiopathic Pulmonary Fibrosis | Stomach Ulcer | Panuveitis | Vasculitis, Hypersensitivity | Mucositis | Inflammation | Sinusitis | Oral Submucous Fibrosis | Tuberculosis, Pulmonary | Stevens-Johnson Syndrome | Erythema Multiforme | Epidermal Necrolysis, Toxic | Respiratory Sounds | Graft vs Host Disease | Malaria, Falciparum | Scleroderma, Systemic | Scleroderma, Localized | Pregnancy Complications, Infectious | Pregnancy Complications | Urinary Tract Infections | Arthritis, Psoriatic | Vesico-Ureteral Reflux | Urinary Bladder Diseases | Severe Acute Respiratory Syndrome | Coronavirus Infections | Coronaviridae Infections | Paramyxoviridae Infections | Intestinal Diseases | Gastroenteritis | Gastritis | Enterocolitis | Enteritis | Colonic Diseases | Colitis | Vaginosis, Bacterial | Vaginitis | Aspergillosis | Viremia | Mycobacterium Infections, Atypical | Salivary Gland Diseases | Brucellosis | Helminthiasis | Leukemia, T-Cell, Acute | Chagas Cardiomyopathy | Retroviridae Infections | Deltaretrovirus Infections | Conjunctivitis | Urticaria | Edema | Salmonella Infections | Enterobacteriaceae Infections | Morbillivirus Infections | Measles | Skin Diseases, Metabolic | Metabolic Diseases | Lipodystrophy | Lipid Metabolism Disorders | Spinal Cord Diseases | Myelitis | Bronchiectasis | Bronchial Diseases | Thymus Neoplasms | Thymoma | Thoracic Neoplasms | Neoplasms, Complex and Mixed | Mediastinal Neoplasms | Uterine Diseases | Ovarian Diseases | Ovarian Cysts | Endometriosis | Cysts | Sarcoma, Kaposi | Picornaviridae Infections | Purpura, Schoenlein-Henoch | Hemostatic Disorders | Immune Complex Diseases | Coronary Artery Disease | Coronary Aneurysm | Arteriosclerosis | Arterial Occlusive Diseases | Parasitemia | Sjogren's Syndrome | Lacrimal Apparatus Diseases | Dacryocystitis | Respiratory Tract Infections | Respiration Disorders | Lung Diseases, Fungal | HTLV-I Infections | Meningitis | Central Nervous System Infections | Shock | Herpesviridae Infections | Cytomegalovirus Infections | Stomatitis | Eye Infections, Bacterial | Eye Infections | Opportunistic Infections | Chlamydia Infections | Sialadenitis | Rubulavirus Infections | Parotitis | Mumps |

Staphylococcal Infections | Soft Tissue Infections | Shock, Septic | Endotoxemia | Cardiovascular Infections | Trachoma | Conjunctivitis, Inclusion | Conjunctivitis, Bacterial | Malaria, Cerebral | Togaviridae Infections | Rubella | Corneal Diseases | Urination Disorders | Proteinuria | Albuminuria

Corresponding sum of effect sizes for each disease group of selected Phenopedia diseases
(In the same order as above)
343.99, 207.38, 118.31, 116.96, 116.81, 114.59, 92.47, 81.20, 79.18, 69.61, 66.81, 63.21, 60.13, 59.72, 56.91, 56.91, 55.44, 52.99, 52.74, 50.92, 46.69, 46.69, 46.69, 45.58, 43.79, 39.31, 38.71, 37.62, 37.62, 36.46, 36.46, 35.96, 33.89, 32.92, 30.66, 30.32, 30.32, 29.01, 29.01, 29.01, 29.01, 29.01, 28.95, 28.31, 28.09, 26.58, 26.58, 26.01, 25.30, 24.54, 24.34, 24.34, 24.18, 23.68, 23.66, 23.66, 23.66, 22.80, 22.80, 22.80, 21.26, 20.46, 20.46, 20.46, 19.72, 19.72, 19.48, 19.48, 19.39, 19.39, 19.39, 19.35, 18.73, 18.42, 17.56, 17.52, 17.52, 17.39, 16.84, 16.71, 15.93, 15.89, 15.68, 15.45, 15.12, 15.12, 15.06, 15.06, 14.97, 14.87, 14.67, 14.67, 14.60, 14.39, 14.39, 13.89, 13.89, 13.16, 13.16, 12.91, 12.66, 12.57, 12.46, 12.24, 12.24, 12.18, 12.12, 12.12, 12.12, 12.12, 11.94, 10.75, 10.35, 10.25, 10.16, 10.05, 9.95, 9.95, 9.95, 9.75, 9.75, 9.64, 9.64, 9.51, 9.37, 9.05, 9.05, 9.05, 8.95, 8.95, 8.89, 8.89, 8.80, 8.59, 8.51, 8.44, 8.41, 8.41, 7.69, 7.62, 7.59, 7.52, 7.52, 7.52, 7.48, 7.46, 7.44, 7.41, 7.41, 7.27, 7.27, 7.25, 6.86, 6.74, 6.74, 6.71, 6.71, 6.71, 6.71, 6.53, 6.53, 6.53, 6.53, 6.53, 6.53, 6.53, 6.53, 6.53, 6.46, 6.37, 6.34, 6.29, 6.21, 6.17, 5.95, 5.94, 5.93, 5.93, 5.75, 5.63, 5.63, 5.52, 5.52, 5.34, 5.34, 5.18, 5.18, 5.18, 5.18, 4.86, 4.86, 4.72, 4.72, 4.53, 4.53, 4.53, 4.53, 4.53, 4.53, 4.53, 4.53, 4.53, 4.53, 4.48, 4.48, 4.27, 4.27, 4.15, 4.12, 4.12, 4.12, 4.12, 3.92, 3.70, 3.70, 3.70, 3.50, 3.50, 3.50, 3.38, 3.03, 3.03, 3.01, 3.00, 3.00, 2.66, 2.37, 2.37, 2.32, 2.21, 1.38, 1.38, 1.38, 1.38, 1.32, 1.32, 1.32, 1.32, 1.32, 1.14, 1.14, 1.14, 1.07, 0.68, 0.68, 0.52, -1.75, -1.75, -1.75

Cluster for columns 534 to 539, rows 202 to 207

Phenopedia diseases

Sensation | Encephalomyelitis | Fatigue Syndrome, Chronic | Neuralgia | Intervertebral Disk Displacement | Sciatica

TFs

V$PR_02 | V$GR_01 | V$PR_01 | V$SREBP1_02 | V$MYOGNF1_01 | V$TAL1BETAITF2_01

Information

All related TFs:
(List of all TFs that are related to any of the PWMs)
GR, ITF-2, NF-1, NF-1/L, NF-1/Red1, NF-1A1, NF-1B1, NF-1B2, NF-1C2, NF-1X, PR, SREBP-1, SREBP-1a, SREBP-1b, SREBP-1c, Tal-1beta

Ranked gene list:
(All genes of the selected Phenopedia diseases with hits of any of the selected TFs, ranked according to the total number of TF hits)
COMT, IL10, HTR2A, TNF, IL4R, VDR, ADRA2C, COL11A2, BDNF, SLC6A4, IL4, TAS1R3, IL1A, DISC1, SERPINA6, NR3C1, NOS2A, NOS2, MMP9, LIF, GABRB1, NOS3, MMP2, MBL2, LBXCOR1, IL6R, IGF2, HTR1E, CCL5, CAPN10, TNFRSF1A, TAS2R16, SLC6A3, OTOF, NFKBIA, MYB, MC1R, LTA, LEP, IL5, IL13, IL12RB1, IL10RA, IGF2AS, IGF1, IFNAR1, HLA-DPA1, ELAVL4, DRD4, COL5A1, CNTF, CD40, CBLB

Corresponding total number of TF hits:
(For each gene listed above, the total number of TF hits for any of the selected TFs, multiplied by the number of selected Phenopedia diseases containing that gene)
24, 18, 15, 12, 10, 9, 9, 8, 8, 6, 6, 4, 4, 4, 3, 3, 3, 3, 3, 3, 3, 2, 2, 2, 2, 2, 2, 2, 2, 2, 2, 1, 1, 1, 1, 1, 1, 1, 1, 1, 1,

1, 1, 1, 1, 1, 1, 1, 1, 1, 1, 1, 1, 1

Corresponding number of selected Phenopedia diseases each gene is involved with:
(In the same order as above)
6, 6, 5, 6, 5, 3, 3, 2, 4, 6, 6, 1, 4, 2, 3, 3, 1, 1, 1, 1, 3, 2, 1, 2, 1, 1, 1, 2, 1, 1, 1, 1, 1, 1, 1, 1, 1, 1, 1, 1, 1, 1, 1, 1, 1, 1, 1, 1, 1, 1, 1, 1, 1

Corresponding number of selected TFs each gene is involved with:
(In the same order as above)
4, 3, 3, 2, 2, 3, 3, 4, 2, 1, 1, 4, 1, 2, 1, 1, 3, 3, 3, 3, 1, 1, 2, 1, 2, 2, 2, 1, 2, 2, 1, 1, 1, 1, 1, 1, 1, 1, 1, 1, 1, 1, 1, 1, 1, 1, 1, 1, 1, 1, 1, 1, 1

Phenopedia diseases ranked according to mean of effect sizes in cluster:
Sciatica | Neuralgia | Intervertebral Disk Displacement | Fatigue Syndrome, Chronic | Encephalomyelitis | Sensation

Corresponding mean of effect sizes of each Phenopedia disease:
(In the same order as above)
2.41, 2.24, 1.89, 1.69, 1.51, 1.29

Corresponding total number of genes of each Phenopedia disease:
(In the same order as above)
31, 32, 43, 43, 73, 143

Corresponding number of genes of each Phenopedia disease with at least one TF hit (of selected TFs):
(In the same order as above)
12, 12, 13, 12, 19, 39

Corresponding total number of TF hits (of selected TFs) for each Phenopedia disease (in all genes):
(In the same order as above)
27, 24, 29, 23, 35, 61

Corresponding number of selected TFs each Phenopedia disease is involved with:
(In the same order as above)
6, 6, 6, 6, 6, 6

TFs ranked according to mean of effect sizes in cluster:
VPR_01, VPR_02, V$MYOGNF1_01, V$TAL1BETAITF2_01, VGR_01, VSREBP1_02

Corresponding mean of effect sizes of each TF:
(In the same order as above)
3.29, 1.98, 1.82, 1.53, 1.30, 1.11

Corresponding total number of TF hits for each TF (genome-wide):
(In the same order as above)
490, 487, 412, 494, 476, 468

Corresponding total number of TF hits for each TF (in all genes in selected Phenopedia diseases):
(In the same order as above)
48, 35, 31, 31, 28, 26

Corresponding number of genes (of selected Phenopedia diseases) each TF is involved with:
(In the same order as above)
19, 17, 14, 13, 15, 10

Corresponding number of selected Phenopedia diseases each TF is involved with:
(In the same order as above)
6, 6, 6, 6, 6, 6

Disease groups of selected Phenopedia diseases ranked according to sum of effect sizes
Nervous System Diseases | Peripheral Nervous System Diseases | Signs and Symptoms | Spinal Diseases | Musculoskeletal Diseases | Bone Diseases | Sciatica | Sciatic Neuropathy | Neuritis | Mononeuropathies | Sensation Disorders | Neurologic Manifestations | Neuralgia | Myokymia | Facial Nerve Diseases | Cranial Nerve Diseases | Intervertebral Disk Displacement | Fatigue Syndrome, Chronic | Spinal Cord Diseases | Myelitis | Encephalomyelitis | Encephalitis | Central Nervous System Diseases | Brain Diseases | Sensation

Corresponding sum of effect sizes for each disease group of selected Phenopedia diseases
(In the same order as above)
37.00, 27.93, 23.58, 20.43, 20.43, 20.43, 14.48, 14.48, 14.48, 14.48, 13.45, 13.45, 13.45, 13.45, 13.45, 13.45, 11.36, 10.13, 9.06, 9.06, 9.06, 9.06, 9.06, 9.06, 7.74

Cluster for columns 419 to 479, rows 212 to 217

Phenopedia diseases

Viremia | Sepsis | Systemic Inflammatory Response Syndrome | Rubella | Measles | Mumps | Tuberculosis | Mycobacterium Infections | Tuberculosis, Pulmonary | Bacterial Infections | Gram-Negative Bacterial Infections | Malaria | Parasitic Diseases | Bronchiectasis | Helminthiasis | Brucellosis | Coronary Aneurysm | Salmonella Infections | Shock, Septic | Myelitis | Deltaretrovirus Infections | HTLV-I Infections | Spinal Diseases | Spondylitis | Ankylosis | Spondylitis, Ankylosing | Spondylarthritis | Spondylarthropathies | Vasculitis | Uveitis | Behcet Syndrome | Panuveitis | Uveitis, Anterior | Arthritis, Psoriatic | Psoriasis | Stomatitis | Epidermal Necrolysis, Toxic | Stevens-Johnson Syndrome | Erythema | Erythema Nodosum | Drug Eruptions | Drug Hypersensitivity | Encephalitis | Severe Acute Respiratory Syndrome | Meningitis | Nasal Polyps | Scleroderma, Systemic | Salivary Gland Diseases | Sjogren's Syndrome | Polymyalgia Rheumatica | Arteritis | Temporal Arteritis | Graft vs Host Disease | Purpura, Schoenlein-Henoch | Immune Complex Diseases | Vasculitis, Hypersensitivity | Streptococcal Infections | Rheumatic Fever | Rheumatic Heart Disease | Hematologic Neoplasms | Leprosy

TFs

V$IRF7_01 | V$CDPCR3_01 | V$IRF_Q6_01 | V$IRF_Q6 | V$ICSBP_Q6 | V$ISRE_01

Information

All related TFs:
(List of all TFs that are related to any of the PWMs)
CUTL1, IRF-1, IRF-10, IRF-2, IRF-3, IRF-4, IRF-5, IRF-6, IRF-7, IRF-7A, IRF-7H, IRF-8, IRF-9, ISGF-3

Ranked gene list:
(All genes of the selected Phenopedia diseases with hits of any of the selected TFs, ranked according to the total number of TF hits)
HLA-A, HLA-B, TAP2, TAP1, HLA-C, VDR, TLR4, CTLA4, CCR5, HLA-DRB1, NOD2, NLRP3, IL6, CARD15, PSMB9, IRF5, IL12RB1, PSMB8, FAS, TLR3, STAT1, IRF1, VEGFA, PTPN22, ERAP1, NR3C1, NFKBIA, MX1, ERAP2, CCR2, VEGF, TNFAIP3, IL2, SP110, NFKB1, MEFV, IL1R1, CCL2, IFNGR1, SOD2, NFKBIZ, APOE, UCP2, TNFSF13B, PDCD1, IL18R1, IFNB1, HLA-DMA, PSORS1, NOD1, CXCL10, PON1, TLR1, PRR3, GNL1, TRAF1, RUNX1, TNFSF15, STAT5A, IL7R, IL2RB,

TLR8, PAX2, IL6R, HLA-G, GNAS, BAK1, P2RX7, LMAN1, CYBA, COL6A1, CISH, TRIM21, MSX2, IL1RAP, IFIH1, DDAH2, CDK6, CCND1, CASP1, CARD4, CALCA, BANK1, ANKH, IFNAR1, CYP27B1, CD40, SOCS1, RXRB, RUNX3, PLAU, MCL1, IL5RA, HIF1A, FLJ78302, DDR1, CLEC16A, CEACAM6, CDKAL1, CCL21, APEX1, ALOXE3, ALOX5AP, ACP1, STAT6, SMAD6, SH2B3, LAG3, IL22RA1, GRAP2, F13A1, EDN1, DCLRE1C, CXCR4, CDKN2A, CD4, CCL3, CARD8, BAT2, AIF1, TNFSF11, TIMD4, TCF7, TBX21, S100A2, RNASEL, PRKCQ, OGG1, NFKBIE, MYD88, IRS2, IL3, IL15, IGF1, HPSE, HBE1, FOXP3, FGL2, DDX58, COL11A2, CIITA, CHST11, CARD11, CALM1, BAT5, BAT1, ZNF148, WNT5A, WAS, TRIM5, TRIM22, TNPO3, TNIP1, TNFSF4, TIMP4, STAT3, SOD1, SIGIRR, SEPT8, PPP1R14C, PPARGC1A, PFKFB1, OASL, NR1H2, MTNR1B, MGST2, KIAA1217, ITGA4, IL21R, IL20RA, IL19, IL13RA2, ICOS, GHSR, GHRL, GATA2, FZD5, FCER2, ERBB3, CX3CL1, CSNK1D, CES2, CAMK2D, BCL2A1, AKT1, ADORA3, ABCA1

Corresponding total number of TF hits:
(For each gene listed above, the total number of TF hits for any of the selected TFs, multiplied by the number of selected Phenopedia diseases containing that gene)
265, 168, 135, 135, 135, 84, 78, 68, 64, 60, 56, 56, 56, 54, 50, 48, 48, 40, 33, 32, 32, 30, 28, 28, 28, 25, 24, 24, 24, 23, 22, 22, 22, 21, 21, 21, 21, 21, 19, 18, 18, 17, 16, 16, 16, 16, 16, 16, 15, 15, 15, 14, 13, 12, 12, 11, 10, 9, 9, 9, 9, 8, 8, 8, 8, 8, 8, 7, 7, 7, 7, 7, 6, 6, 6, 6, 6, 6, 6, 6, 6, 6, 6, 6, 5, 5, 5, 4, 4, 4, 4, 4, 4, 4, 4, 4, 4, 4, 4, 4, 4, 4, 4, 4, 3, 3, 3, 3, 3, 3, 3, 3, 3, 3, 3, 3, 3, 3, 3, 3, 2, 2, 2, 2, 2, 2, 2, 2, 2, 2, 2, 2, 2, 2, 2, 2, 2, 2, 2, 2, 2, 2, 2, 2, 2, 1, 1, 1, 1, 1, 1, 1, 1, 1, 1, 1, 1, 1, 1, 1, 1, 1, 1, 1, 1, 1, 1, 1, 1, 1, 1, 1, 1, 1, 1, 1, 1, 1, 1, 1, 1, 1, 1, 1

Corresponding number of selected Phenopedia diseases each gene is involved with:
(In the same order as above)
53, 56, 27, 27, 45, 28, 39, 34, 32, 60, 28, 14, 56, 27, 10, 12, 16, 8, 11, 8, 8, 15, 28, 28, 7, 5, 8, 6, 6, 23, 22, 11, 22, 7, 21, 21, 21, 21, 19, 18, 6, 17, 4, 4, 8, 8, 8, 8, 5, 5, 5, 7, 13, 6, 6, 11, 5, 3, 3, 3, 9, 4, 4, 8, 8, 2, 2, 7, 7, 7, 7, 7, 2, 6, 2, 2, 3, 2, 2, 3, 2, 3, 3, 6, 5, 5, 5, 1, 2, 2, 4, 4, 2, 4, 4, 1, 1, 2, 1, 2, 2, 1, 1, 4, 3, 3, 1, 1, 1, 3, 3, 3, 1, 1, 1, 3, 3, 3, 3, 3, 2, 2, 2, 2, 1, 1, 1, 2, 2, 2, 2, 2, 1, 1, 2, 2, 2, 1, 1, 2, 1, 1, 2, 1, 1, 2, 1, 1, 1, 1, 1, 1, 1, 1, 1, 1, 1, 1, 1, 1, 1, 1, 1, 1, 1, 1, 1, 1, 1, 1, 1, 1, 1, 1, 1, 1, 1, 1, 1, 1, 1, 1, 1, 1

Corresponding number of selected TFs each gene is involved with:
(In the same order as above)
5, 3, 5, 5, 3, 3, 2, 2, 2, 2, 1, 2, 4, 1, 2, 5, 4, 3, 5, 3, 4, 4, 2, 1, 1, 4, 5, 3, 4, 4, 1, 1, 2, 1, 3, 1, 1, 1, 1, 1, 1, 3, 1, 4, 4, 2, 2, 2, 2, 3, 3, 3, 2, 1, 2, 2, 1, 2, 3, 3, 3, 1, 2, 2, 1, 1, 4, 4, 1, 1, 1, 1, 1, 3, 1, 3, 3, 2, 3, 3, 2, 3, 2, 2, 1, 1, 1, 1, 4, 2, 2, 1, 1, 2, 1, 1, 4, 4, 2, 4, 2, 2, 4, 4, 1, 1, 1, 3, 3, 3, 1, 1, 1, 3, 3, 3, 1, 1, 1, 1, 1, 1, 1, 1, 1, 2, 2, 2, 1, 1, 1, 1, 1, 2, 2, 1, 1, 1, 2, 2, 1, 2, 2, 1, 2, 2, 1, 1, 1, 1, 1, 1, 1, 1, 1, 1, 1, 1, 1, 1, 1, 1, 1, 1, 1, 1, 1, 1, 1, 1, 1, 1, 1, 1, 1, 1, 1, 1, 1, 1, 1, 1, 1, 1, 1, 1, 1, 1

Phenopedia diseases ranked according to mean of effect sizes in cluster:
Measles | Severe Acute Respiratory Syndrome | Encephalitis | Leprosy | Sjogren's Syndrome | Psoriasis | Coronary Aneurysm | Spondylitis | Spondylitis, Ankylosing | Ankylosis | Mycobacterium Infections | Spondylarthropathies | Rubella | Spondylarthritis | Stevens-Johnson Syndrome | Epidermal Necrolysis, Toxic | Erythema Nodosum | Erythema | Salivary Gland Diseases | Mumps | Tuberculosis, Pulmonary | Drug Eruptions | Viremia | Uveitis | Vasculitis, Hypersensitivity | Immune Complex Diseases | Brucellosis | Bronchiectasis | Arteritis | Uveitis, Anterior | Panuveitis | Purpura, Schoenlein-Henoch | Behcet Syndrome | Spinal Diseases | Drug Hypersensitivity | Vasculitis | Tuberculosis | Meningitis | Hematologic Neoplasms | Rheumatic Fever | Nasal Polyps | Rheumatic Heart Disease | Arthritis, Psoriatic | Sepsis | Systemic Inflammatory Response Syndrome | Polymyalgia Rheumatica | Bacterial Infections | Parasitic Diseases | Deltaretrovirus Infections | Stomatitis | Scleroderma, Systemic | HTLV-I Infections | Temporal Arteritis | Helminthiasis | Gram-Negative Bacterial Infections | Graft vs Host Disease | Salmonella Infections | Streptococcal Infections | Malaria | Myelitis | Shock, Septic

Corresponding mean of effect sizes of each Phenopedia disease:
(In the same order as above)
2.54, 2.43, 2.28, 1.95, 1.78, 1.68, 1.67, 1.59, 1.56, 1.54, 1.54, 1.53, 1.50, 1.49, 1.47, 1.47, 1.45, 1.44, 1.43,

1.39, 1.33, 1.28, 1.26, 1.20, 1.14, 1.14, 1.13, 1.11, 1.11, 1.07, 1.04, 1.04, 1.03, 0.99, 0.94, 0.91, 0.83, 0.83, 0.82, 0.81, 0.80, 0.74, 0.70, 0.70, 0.70, 0.66, 0.65, 0.64, 0.64, 0.63, 0.60, 0.57, 0.47, 0.41, 0.31, 0.30, 0.30, 0.18, 0.11, 0.11, -0.49

Corresponding total number of genes of each Phenopedia disease:
(In the same order as above)
46, 37, 42, 50, 45, 155, 21, 99, 72, 73, 142, 106, 48, 111, 21, 20, 38, 67, 61, 32, 93, 52, 58, 110, 34, 35, 21, 36, 47, 99, 102, 33, 95, 176, 84, 210, 159, 37, 57, 30, 25, 27, 55, 185, 149, 27, 300, 144, 30, 36, 84, 29, 38, 34, 197, 81, 25, 49, 139, 22, 26

Corresponding number of genes of each Phenopedia disease with at least one TF hit (of selected TFs):
(In the same order as above)
20, 11, 21, 15, 16, 53, 8, 38, 28, 28, 34, 36, 20, 36, 6, 6, 15, 21, 19, 13, 25, 14, 17, 30, 13, 13, 10, 7, 17, 27, 27, 12, 26, 45, 21, 61, 38, 8, 15, 10, 7, 9, 21, 46, 41, 9, 64, 40, 13, 8, 24, 12, 13, 8, 39, 23, 8, 13, 30, 8, 6

Corresponding total number of TF hits (of selected TFs) for each Phenopedia disease (in all genes):
(In the same order as above)
51, 26, 37, 33, 39, 117, 21, 87, 67, 67, 70, 87, 48, 87, 19, 19, 37, 51, 44, 30, 51, 33, 36, 63, 23, 23, 30, 23, 30, 57, 57, 22, 56, 99, 46, 114, 70, 16, 34, 21, 18, 20, 41, 81, 74, 15, 129, 78, 27, 21, 41, 25, 20, 20, 80, 41, 15, 26, 62, 18, 9

Corresponding number of selected TFs each Phenopedia disease is involved with:
(In the same order as above)
6, 6, 6, 6, 6, 6, 6, 6, 6, 6, 6, 6, 6, 6, 6, 6, 6, 6, 6, 6, 6, 6, 6, 6, 6, 6, 6, 6, 6, 6, 6, 6, 6, 6, 6, 6, 6, 6, 6, 6, 6, 6, 6, 5, 6, 6, 6, 6, 6, 6, 6, 6, 6, 6, 6, 6, 6, 6, 6, 6, 4

TFs ranked according to mean of effect sizes in cluster:
VIRF_Q6, VCDPCR3_01, V$IRF7_01, V$IRF_Q6_01, V$ICSBP_Q6, V$ISRE_01

Corresponding mean of effect sizes of each TF:
(In the same order as above)
1.74, 1.25, 1.14, 1.01, 0.86, 0.34

Corresponding total number of TF hits for each TF (genome-wide):
(In the same order as above)
542, 490, 478, 548, 522, 507

Corresponding total number of TF hits for each TF (in all genes in selected Phenopedia diseases):
(In the same order as above)
637, 462, 404, 471, 459, 369

Corresponding number of genes (of selected Phenopedia diseases) each TF is involved with:
(In the same order as above)
75, 53, 43, 65, 65, 51

Corresponding number of selected Phenopedia diseases each TF is involved with:
(In the same order as above)
61, 61, 61, 61, 60, 59

Disease groups of selected Phenopedia diseases ranked according to sum of effect sizes
Signs and Symptoms | Communicable Diseases | Musculoskeletal Diseases | Skin Diseases | Virus Diseases | Bacterial Infections | Exanthema | Spinal Diseases | Bone Diseases | Cardiovascular Diseases | Gram-Positive Bacterial Infections | Vascular Diseases | Arthritis | Spondylarthropathies | Uveitis | Uveal Diseases | Eye Diseases | Endophthalmitis | Erythema | Mycobacterium Infections | Actinomycetales

Infections | Respiratory Tract Diseases | Vasculitis | Immune System Diseases | Thoracic Diseases | Spondylarthritis | Paramyxoviridae Infections | Stomatognathic Diseases | Mouth Diseases | Jaw Diseases | Nervous System Diseases | Central Nervous System Diseases | Spondylitis | Osteitis | Connective Tissue Diseases | Collagen Diseases | Autoimmune Diseases | Salivary Gland Diseases | Systemic Inflammatory Response Syndrome | Sepsis | Morbillivirus Infections | Measles | Severe Acute Respiratory Syndrome | Coronavirus Infections | Coronaviridae Infections | Mediastinal Diseases | Skin Diseases, Papulosquamous | Psoriasis | Encephalitis | Brain Diseases | Poisoning | Drug Toxicity | Vasculitis, Hypersensitivity | Infection | Tuberculosis | Uveitis, Anterior | Hypersensitivity | Peripheral Vascular Diseases | Leprosy | Sjogren's Syndrome | Lacrimal Apparatus Diseases | Dacryocystitis | Streptococcal Infections | Coronary Artery Disease | Coronary Aneurysm | Arteriosclerosis | Arterial Occlusive Diseases | Spondylitis, Ankylosing | Rheumatic Fever | Rheumatic Diseases | Ankylosis | Togaviridae Infections | Rubella | Stevens-Johnson Syndrome | Erythema Multiforme | Epidermal Necrolysis, Toxic | Erythema Nodosum | Sialadenitis | Rubulavirus Infections | Parotitis | Mumps | Tuberculosis, Pulmonary | Pneumonia | Lung Diseases | Drug Eruptions | Dermatitis | Viremia | Gram-Negative Bacterial Infections | Retroviridae Infections | Deltaretrovirus Infections | Immune Complex Diseases | Brucellosis | Bronchiectasis | Bronchial Diseases | Arteritis | Parasitic Diseases | Panuveitis | Purpura, Schoenlein-Henoch | Purpura | Hemostatic Disorders | Hemorrhage | Blood Coagulation Disorders | Skin Diseases, Vascular | Behcet Syndrome | Drug Hypersensitivity | Meningitis | Central Nervous System Infections | Neoplasms | Hematologic Neoplasms | Polyps | Otorhinolaryngologic Diseases | Nose Diseases | Nasal Polyps | Rheumatic Heart Disease | Myocarditis | Mediastinitis | Cardiomyopathies | Arthritis, Psoriatic | Polymyalgia Rheumatica | Stomatitis | Mucositis | Inflammation | Scleroderma, Systemic | Scleroderma, Localized | HTLV-I Infections | Temporal Arteritis | Helminthiasis | Graft vs Host Disease | Salmonella Infections | Enterobacteriaceae Infections | Protozoan Infections | Malaria | Coccidiosis | Spinal Cord Diseases | Myelitis | Staphylococcal Infections | Soft Tissue Infections | Shock, Septic | Shock | Endotoxemia | Cardiovascular Infections

Corresponding sum of effect sizes for each disease group of selected Phenopedia diseases
(In the same order as above)
149.00, 124.67, 85.27, 63.14, 61.96, 55.71, 46.89, 43.63, 43.63, 42.86, 41.36, 38.45, 37.13, 37.01, 36.71, 36.71, 36.71, 36.71, 34.99, 33.92, 33.92, 33.86, 31.33, 31.23, 29.08, 27.84, 23.55, 23.09, 23.09, 23.09, 19.32, 19.32, 18.91, 18.91, 18.30, 18.30, 16.92, 16.92, 15.95, 15.95, 15.21, 15.21, 14.57, 14.57, 14.57, 14.47, 14.32, 14.32, 13.68, 13.68, 13.33, 13.33, 13.07, 13.02, 12.98, 12.57, 12.49, 12.39, 11.72, 10.70, 10.70, 10.70, 10.37, 10.05, 10.05, 10.05, 10.05, 9.35, 9.28, 9.28, 9.27, 9.03, 9.03, 8.82, 8.82, 8.82, 8.72, 8.34, 8.34, 8.34, 8.34, 7.97, 7.97, 7.97, 7.67, 7.67, 7.56, 7.52, 7.26, 7.26, 6.83, 6.78, 6.64, 6.64, 6.64, 6.32, 6.25, 6.22, 6.22, 6.22, 6.22, 6.22, 6.17, 6.17, 5.66, 4.98, 4.98, 4.91, 4.91, 4.78, 4.78, 4.78, 4.78, 4.42, 4.42, 4.42, 4.42, 4.23, 3.99, 3.76, 3.76, 3.76, 3.61, 3.61, 3.43, 2.82, 2.47, 1.82, 1.81, 1.81, 0.67, 0.67, 0.67, 0.66, 0.66, -2.93, -2.93, -2.93, -2.93, -2.93, -2.93

Cluster for columns 590 to 599, rows 196 to 201

Phenopedia diseases

Hashimoto Disease | Thyroiditis, Autoimmune | Graves Disease | Orbital Diseases | Goiter | Hyperthyroidism | Graves Ophthalmopathy | Myasthenia Gravis | Multiple Sclerosis, Relapsing-Remitting | Lymphopenia

TFs

V$NFKB_C | V$NFKAPPAB_01 | V$NFKAPPAB65_01 | V$CREL_01 | V$NFKB_Q6_01 | V$NFKB_Q6

Information

All related TFs:
(List of all TFs that are related to any of the PWMs)
NF-TNF, NF-kappaB, NF-kappaB(-like), NF-kappaB2, RelA-p65, c-Rel, p100, p105, p50, p52

Ranked gene list:
(All genes of the selected Phenopedia diseases with hits of any of the selected TFs, ranked according to the total number of TF hits)
TNF, LTA, TGFB1, IFIH1, NFKB1, STAT6, GSTP1, CD40, PDCD1, IRF1, CXCL10, VDR, HLA-B, AIRE, HLA-A, NR3C1, CYP27B1, IL1RN, BRAF, XRCC1, TAP1, PDCD1LG2, IL2RA, IL16, HLA-C, TSHR, IL12B, TPMT, NOD2, NFKBIA, CARD15, IL4R, HSPA1L, FAS, TAP2, LAG3, DBP, CNTF, SLC19A1, OAS3, LGALS3, IL7R, CD58, BDNF, TNFRSF1B, RET, PLAU, ADA, STAT3, RUNX1, JAK3, HFE, CTSS, CD86

Corresponding total number of TF hits:
(For each gene listed above, the total number of TF hits for any of the selected TFs, multiplied by the number of selected Phenopedia diseases containing that gene)
54, 36, 35, 35, 30, 24, 24, 21, 20, 20, 20, 18, 16, 15, 14, 12, 12, 9, 9, 8, 8, 8, 8, 8, 8, 7, 7, 6, 6, 6, 6, 5, 5, 5, 4, 4, 4, 4, 3, 3, 3, 3, 3, 3, 2, 2, 2, 2, 1, 1, 1, 1, 1, 1

Corresponding number of selected Phenopedia diseases each gene is involved with:
(In the same order as above)
9, 6, 7, 7, 5, 4, 6, 7, 4, 4, 5, 6, 8, 5, 7, 6, 6, 9, 3, 4, 4, 4, 4, 4, 4, 7, 7, 1, 1, 1, 1, 5, 5, 5, 4, 1, 4, 1, 1, 1, 1, 1, 1, 1, 2, 2, 1, 1, 1, 1, 1, 1, 1, 1

Corresponding number of selected TFs each gene is involved with:
(In the same order as above)
6, 6, 5, 5, 6, 6, 4, 3, 5, 5, 4, 3, 2, 3, 2, 2, 2, 1, 3, 2, 2, 2, 2, 2, 2, 1, 1, 6, 6, 6, 6, 1, 1, 1, 1, 4, 1, 4, 3, 3, 3, 3, 3, 3, 1, 1, 2, 2, 1, 1, 1, 1, 1, 1

Phenopedia diseases ranked according to mean of effect sizes in cluster:
Orbital Diseases | Graves Disease | Goiter | Thyroiditis, Autoimmune | Lymphopenia | Hyperthyroidism | Multiple Sclerosis, Relapsing-Remitting | Hashimoto Disease | Graves Ophthalmopathy | Myasthenia Gravis

Corresponding mean of effect sizes of each Phenopedia disease:
(In the same order as above)
2.31, 2.28, 2.22, 2.07, 1.92, 1.89, 1.85, 1.76, 1.60, 0.71

Corresponding total number of genes of each Phenopedia disease:
(In the same order as above)
92, 125, 103, 44, 27, 103, 56, 29, 35, 28

Corresponding number of genes of each Phenopedia disease with at least one TF hit (of selected TFs):
(In the same order as above)
30, 36, 33, 17, 8, 32, 17, 10, 8, 8

Corresponding total number of TF hits (of selected TFs) for each Phenopedia disease (in all genes):
(In the same order as above)
87, 111, 95, 46, 22, 90, 42, 30, 26, 24

Corresponding number of selected TFs each Phenopedia disease is involved with:
(In the same order as above)
6, 6, 6, 6, 6, 6, 6, 6, 6, 6

TFs ranked according to mean of effect sizes in cluster:
V$NFKAPPAB_01, V$NFKB_Q6, V$NFKB_C, V$NFKAPPAB65_01, V$CREL_01, V$NFKB_Q6_01

Corresponding mean of effect sizes of each TF:
(In the same order as above)
2.30, 2.26, 2.13, 1.70, 1.59, 1.18

Corresponding total number of TF hits for each TF (genome-wide):
(In the same order as above)
477, 465, 472, 488, 456, 467

Corresponding total number of TF hits for each TF (in all genes in selected Phenopedia diseases):
(In the same order as above)
108, 106, 101, 92, 93, 73

Corresponding number of genes (of selected Phenopedia diseases) each TF is involved with:
(In the same order as above)
27, 28, 29, 26, 23, 21

Corresponding number of selected Phenopedia diseases each TF is involved with:
(In the same order as above)
10, 10, 10, 10, 10, 10

Disease groups of selected Phenopedia diseases ranked according to sum of effect sizes
Thyroid Diseases | Endocrine System Diseases | Immune System Diseases | Autoimmune Diseases | Orbital Diseases | Eye Diseases | Goiter | Hyperthyroidism | Graves Disease | Exophthalmos | Thyroiditis, Autoimmune | Thyroiditis | Lymphopenia | Leukopenia | Leukocyte Disorders | Immunologic Deficiency Syndromes | Nervous System Diseases | Multiple Sclerosis, Relapsing-Remitting | Multiple Sclerosis | Demyelinating Diseases | Demyelinating Autoimmune Diseases, CNS | Autoimmune Diseases of the Nervous System | Hashimoto Disease | Signs and Symptoms | Graves Ophthalmopathy | Genetic Diseases, Inborn | Eye Diseases, Hereditary | Myasthenia Gravis

Corresponding sum of effect sizes for each disease group of selected Phenopedia diseases
(In the same order as above)
70.90, 70.90, 50.12, 38.59, 37.12, 37.12, 36.57, 34.57, 23.24, 23.24, 23.00, 23.00, 11.54, 11.54, 11.54, 11.54, 11.11, 11.11, 11.11, 11.11, 11.11, 11.11, 10.56, 9.57, 9.57, 9.57, 9.57, 4.24

Cluster for columns 546 to 561, rows 197 to 201

Phenopedia diseases

Postoperative Complications | Pain, Postoperative | Autonomic Nervous System Diseases | Prosthesis Failure | Decision Making | Metal Metabolism, Inborn Errors | Iron Metabolism Disorders | Iron Overload | Pancreatitis | Pancreatitis, Alcoholic | Pancreatitis, Chronic | Alcohol-Induced Disorders | Liver Cirrhosis, Alcoholic | Liver Diseases, Alcoholic | Hepatitis B, Chronic | Liver Cirrhosis

TFs

V$NFKAPPAB_01 | V$NFKAPPAB65_01 | V$CREL_01 | V$NFKB_Q6_01 | V$NFKB_Q6

Information

All related TFs:
(List of all TFs that are related to any of the PWMs)
NF-TNF, NF-kappaB, NF-kappaB(-like), NF-kappaB2, RelA-p65, c-Rel, p100, p105, p50, p52

Ranked gene list:
(All genes of the selected Phenopedia diseases with hits of any of the selected TFs, ranked according to the total number of TF hits)
TNF, TGFB1, GSTP1, NFKBIA, LTA, NFKB1, NOD2, XRCC1, VDR, TPMT, HLA-B, HLA-A, HFE, IL12B, PTGS1, IL19, TNFRSF6B, RUNX3, PDCD1, IL4R, CREB1, CARD15, STAT1, JAK1, HLA-C, FURIN, VEGFA, TYMP, MYB, KLF6, ITGB3, HAMP, CXCL10, VEGF, OAS3, GRM8, GCH1, DDAH2, BDNF, ADRA2B, TNFRSF1B, PRKCH, MIF, ITPA, IRF3, IFNGR2, HSPA1L, HIF1A, ERCC2, DRD4, DMPK, DM1, CLOCK, ALOX5AP

Corresponding total number of TF hits:
(For each gene listed above, the total number of TF hits for any of the selected TFs, multiplied by the number of selected Phenopedia diseases containing that gene)
80, 75, 44, 25, 25, 20, 15, 12, 12, 10, 8, 8, 8, 7, 6, 6, 5, 5, 5, 5, 5, 5, 4, 4, 4, 4, 3, 3, 3, 3, 3, 3, 3, 2, 2, 2, 2, 2, 2, 2, 1, 1, 1, 1, 1, 1, 1, 1, 1, 1, 1, 1, 1, 1

Corresponding number of selected Phenopedia diseases each gene is involved with:
(In the same order as above)
16, 15, 11, 5, 5, 4, 3, 6, 6, 2, 8, 8, 8, 7, 2, 2, 1, 1, 1, 5, 1, 1, 1, 2, 4, 2, 3, 1, 1, 1, 3, 3, 1, 2, 1, 1, 2, 1, 1, 1, 1, 1, 1, 1, 1, 1, 1, 1, 1, 1, 1, 1, 1, 1

Corresponding number of selected TFs each gene is involved with:
(In the same order as above)
5, 5, 4, 5, 5, 5, 5, 2, 2, 5, 1, 1, 1, 1, 3, 3, 5, 5, 5, 1, 5, 5, 4, 2, 1, 2, 1, 3, 3, 3, 1, 1, 3, 1, 2, 2, 1, 2, 2, 2, 1, 1, 1, 1, 1, 1, 1, 1, 1, 1, 1, 1, 1, 1

Phenopedia diseases ranked according to mean of effect sizes in cluster:
Hepatitis B, Chronic | Liver Cirrhosis, Alcoholic | Liver Diseases, Alcoholic | Liver Cirrhosis | Alcohol-Induced Disorders | Pain, Postoperative | Iron Overload | Decision Making | Pancreatitis, Alcoholic | Pancreatitis | Iron Metabolism Disorders | Postoperative Complications | Pancreatitis, Chronic | Prosthesis Failure | Metal Metabolism, Inborn Errors | Autonomic Nervous System Diseases

Corresponding mean of effect sizes of each Phenopedia disease:
(In the same order as above)
2.20, 2.01, 1.90, 1.57, 1.32, 1.22, 1.17, 1.12, 1.05, 0.87, 0.62, 0.56, 0.54, 0.33, 0.23, 0.17

Corresponding total number of genes of each Phenopedia disease:
(In the same order as above)
84, 46, 56, 185, 94, 26, 49, 93, 39, 97, 50, 127, 32, 27, 49, 22

Corresponding number of genes of each Phenopedia disease with at least one TF hit (of selected TFs):
(In the same order as above)
19, 8, 8, 25, 9, 3, 8, 15, 3, 15, 9, 21, 3, 5, 8, 4

Corresponding total number of TF hits (of selected TFs) for each Phenopedia disease (in all genes):
(In the same order as above)
47, 28, 28, 75, 30, 13, 23, 35, 14, 36, 21, 50, 11, 13, 20, 12

Corresponding number of selected TFs each Phenopedia disease is involved with:
(In the same order as above)

5, 5, 5, 5, 5, 5, 5, 5, 5, 5, 5, 5, 5, 5, 5, 5

TFs ranked according to mean of effect sizes in cluster:
V$NFKAPPAB65_01, V$NFKAPPAB_01, V$NFKB_Q6, V$NFKB_Q6_01, V$CREL_01

Corresponding mean of effect sizes of each TF:
(In the same order as above)
1.35, 1.17, 0.99, 0.92, 0.84

Corresponding total number of TF hits for each TF (genome-wide):
(In the same order as above)
488, 477, 465, 467, 456

Corresponding total number of TF hits for each TF (in all genes in selected Phenopedia diseases):
(In the same order as above)
102, 97, 94, 78, 85

Corresponding number of genes (of selected Phenopedia diseases) each TF is involved with:
(In the same order as above)
29, 25, 28, 22, 25

Corresponding number of selected Phenopedia diseases each TF is involved with:
(In the same order as above)
16, 16, 16, 16, 16

Disease groups of selected Phenopedia diseases ranked according to sum of effect sizes
Signs and Symptoms | Digestive System Diseases | Liver Diseases | Substance-Related Disorders | Alcohol-Related Disorders | Alcohol-Induced Disorders | Liver Diseases, Alcoholic | Liver Cirrhosis | Pancreatitis | Pancreatic Diseases | Virus Diseases | Hepatitis, Viral, Human | Hepatitis B, Chronic | Hepatitis B | Hepatitis | Hepadnaviridae Infections | DNA Virus Infections | Communicable Diseases | Metabolic Diseases | Liver Cirrhosis, Alcoholic | Iron Metabolism Disorders | Pain, Postoperative | Iron Overload | Decision Making | Pancreatitis, Alcoholic | Postoperative Complications | Pancreatitis, Chronic | Prosthesis Failure | Metal Metabolism, Inborn Errors | Metabolism, Inborn Errors | Genetic Diseases, Inborn | Congenital, Hereditary, and Neonatal Diseases and Abnormalities | Peripheral Nervous System Diseases | Nervous System Diseases | Autonomic Nervous System Diseases

Corresponding sum of effect sizes for each disease group of selected Phenopedia diseases
(In the same order as above)
52.44, 50.60, 38.35, 31.37, 31.37, 31.37, 19.54, 17.88, 12.25, 12.25, 10.98, 10.98, 10.98, 10.98, 10.98, 10.98, 10.98, 10.98, 10.09, 10.05, 8.92, 6.12, 5.85, 5.60, 5.23, 2.82, 2.68, 1.65, 1.16, 1.16, 1.16, 1.16, 0.84, 0.84, 0.84

Cluster for columns 568 to 598, rows 212 to 217

Phenopedia diseases

Hemophilia A | Hemophilia B | Blood Coagulation Disorders | Blood Coagulation Disorders, Inherited | Coagulation Protein Disorders | Anemia, Aplastic | Common Variable Immunodeficiency | Uveomeningoencephalitic Syndrome | Liver Cirrhosis, Biliary | Cholestasis | Cholestasis, Intrahepatic | Cholangitis, Sclerosing | Granuloma | Periodontal Diseases | Chronic Periodontitis | Periodontitis | Bronchial Hyperreactivity | Airway Obstruction | Multiple Sclerosis, Chronic Progressive | Addison Disease | Adrenal Insufficiency | Hypothyroidism | Hashimoto Disease | Thyroiditis, Autoimmune | Graves Disease | Orbital Diseases | Goiter | Hyperthyroidism | Graves Ophthalmopathy | Myasthenia Gravis | Multiple Sclerosis, Relapsing-Remitting

TFs

V$IRF7_01 | V$CDPCR3_01 | V$IRF_Q6_01 | V$IRF_Q6 | V$ICSBP_Q6 | V$ISRE_01

Information

All related TFs:
(List of all TFs that are related to any of the PWMs)
CUTL1, IRF-1, IRF-10, IRF-2, IRF-3, IRF-4, IRF-5, IRF-6, IRF-7, IRF-7A, IRF-7H, IRF-8, IRF-9, ISGF-3

Ranked gene list:
(All genes of the selected Phenopedia diseases with hits of any of the selected TFs, ranked according to the total number of TF hits)
HLA-A, HLA-B, CTLA4, VDR, HLA-C, NR3C1, CCR5, TAP1, HLA-DRB1, IFIH1, TAP2, PTPN22, NOD2, PDCD1, CXCL10, IL2, NOD1, IL6, FAS, CCND1, TLR4, CDKN2A, CD274, CARD15, IL1R1, APOE, IRF1, IGF1, ICOS, FOXP3, PIK3R1, F12, CCR2, UCP2, PON1, IRF5, GNAS, CYP27B1, CLEC16A, CBLB, APEX1, CD40, SH2B3, IL3, IL12RB1, HLA-G, FGL2, CIITA, VWF, STAT6, OAS3, NFKB1, F13A1, EDN1, PSMA6, PDCD1LG2, NR3C2, MX1, LMAN1, IFNGR1, CFLAR, ATP1B1, ALOX5AP, ABCD1, TNFSF11, PLAU, NFKBIA, LAG3, IL7R, IL6R, IL1RAP, GATA3, CYBA, CKAP2L, CCL2, CARD4, TET2, SYN3, SOD2, SLC25A13, RUNX1, RPS16, RPL35A, PAX2, P2RX7, NR1H3, IL5RA, IL15, FKBP5, ERBB3, CNTF, BACH1, WNK4, VEGFA, VEGF, TTF2, TSLP, TNFRSF13C, TBX21, STAT3, SLC22A3, RPS14, RPL11, OGG1, NOLA2, MST1, MEFV, MBD4, LGALS3BP, KCNE3, IL2RB, FOXE1, CYFIP2, CTSS, CSF2RB, CD27, CCL3, APOC1, ADAR, ADAM23

Corresponding total number of TF hits:
(For each gene listed above, the total number of TF hits for any of the selected TFs, multiplied by the number of selected Phenopedia diseases containing that gene)
120, 69, 60, 45, 45, 40, 36, 30, 30, 27, 20, 20, 20, 18, 18, 16, 15, 15, 15, 15, 14, 12, 12, 12, 11, 11, 10, 10, 10, 10, 9, 9, 9, 8, 8, 8, 8, 8, 8, 8, 8, 7, 6, 6, 6, 6, 6, 6, 5, 5, 5, 5, 5, 5, 4, 4, 4, 4, 4, 4, 4, 4, 4, 4, 3, 3, 3, 3, 3, 3, 3, 3, 3, 3, 3, 3, 2, 2, 2, 2, 2, 2, 2, 2, 2, 2, 2, 2, 2, 2, 2, 1, 1, 1, 1, 1, 1, 1, 1, 1, 1, 1, 1, 1, 1, 1, 1, 1, 1, 1, 1, 1, 1, 1, 1, 1, 1, 1

Corresponding number of selected Phenopedia diseases each gene is involved with:
(In the same order as above)
24, 23, 30, 15, 15, 8, 18, 6, 30, 9, 4, 20, 10, 9, 6, 16, 5, 15, 5, 5, 7, 4, 6, 6, 11, 11, 5, 5, 10, 10, 3, 3, 9, 2, 4, 2, 2, 8, 2, 4, 4, 7, 2, 6, 2, 6, 3, 3, 5, 5, 1, 5, 5, 5, 1, 4, 1, 1, 4, 4, 2, 4, 1, 2, 3, 3, 1, 1, 1, 3, 1, 1, 3, 3, 3, 1, 1, 2, 2, 2, 1, 1, 1, 1, 2, 1, 1, 1, 2, 2, 1, 1, 1, 1, 1, 1, 1, 1, 1, 1, 1, 1, 1, 1, 1, 1, 1, 1, 1, 1, 1, 1, 1, 1, 1, 1, 1, 1, 1

Corresponding number of selected TFs each gene is involved with:
(In the same order as above)
5, 3, 2, 3, 3, 5, 2, 5, 1, 3, 5, 1, 2, 2, 3, 1, 3, 1, 3, 3, 2, 3, 2, 2, 1, 1, 2, 2, 1, 1, 3, 3, 1, 4, 2, 4, 4, 1, 4, 2, 2, 1, 3, 1, 3, 1, 2, 2, 1, 1, 5, 1, 1, 1, 4, 1, 4, 4, 1, 1, 2, 1, 4, 2, 1, 1, 3, 3, 3, 1, 3, 3, 1, 1, 1, 3, 2, 1, 1, 1, 2, 2, 2, 2, 1, 2, 2, 2, 1, 1, 2, 2, 1, 1, 1, 1, 1, 1, 1, 1, 1, 1, 1, 1, 1, 1, 1, 1, 1, 1, 1, 1, 1, 1, 1, 1, 1, 1, 1

Phenopedia diseases ranked according to mean of effect sizes in cluster:
Uveomeningoencephalitic Syndrome | Cholangitis, Sclerosing | Multiple Sclerosis, Relapsing-Remitting | Hemophilia B | Hemophilia A | Addison Disease | Common Variable Immunodeficiency | Adrenal Insufficiency | Granuloma | Cholestasis | Cholestasis, Intrahepatic | Liver Cirrhosis, Biliary | Coagulation Protein Disorders | Graves Disease | Hyperthyroidism | Orbital Diseases | Goiter | Anemia, Aplastic | Multiple Sclerosis, Chronic Progressive | Periodontitis | Myasthenia Gravis | Blood Coagulation Disorders | Graves Ophthalmopathy | Blood Coagulation Disorders, Inherited | Airway Obstruction | Periodontal Diseases | Bronchial Hyperreactivity | Chronic Periodontitis | Thyroiditis, Autoimmune | Hypothyroidism | Hashimoto Disease

Corresponding mean of effect sizes of each Phenopedia disease:
(In the same order as above)
2.41, 2.32, 1.98, 1.64, 1.58, 1.46, 1.46, 1.44, 1.35, 1.17, 1.12, 1.09, 0.98, 0.96, 0.87, 0.86, 0.86, 0.81, 0.80, 0.73, 0.71, 0.69, 0.66, 0.65, 0.62, 0.60, 0.46, 0.43, 0.34, 0.29, 0.21

Corresponding total number of genes of each Phenopedia disease:
(In the same order as above)
19, 35, 56, 35, 39, 45, 25, 48, 21, 67, 59, 49, 39, 125, 103, 92, 103, 46, 23, 130, 28, 104, 35, 59, 40, 130, 124, 118, 44, 49, 29

Corresponding number of genes of each Phenopedia disease with at least one TF hit (of selected TFs):
(In the same order as above)
8, 16, 21, 13, 14, 19, 9, 20, 9, 17, 15, 13, 12, 35, 32, 28, 32, 13, 9, 28, 10, 24, 10, 13, 10, 27, 29, 25, 13, 10, 10

Corresponding total number of TF hits (of selected TFs) for each Phenopedia disease (in all genes):
(In the same order as above)
18, 38, 43, 28, 29, 38, 16, 41, 21, 31, 28, 26, 26, 79, 70, 60, 69, 25, 13, 53, 24, 42, 17, 27, 20, 49, 51, 47, 25, 17, 15

Corresponding number of selected TFs each Phenopedia disease is involved with:
(In the same order as above)
6, 6, 6, 6, 6, 6, 6, 6, 6, 6, 6, 6, 6, 6, 6, 6, 6, 6, 5, 6, 6, 6, 6, 6, 6, 6, 6, 6, 6, 6, 6

TFs ranked according to mean of effect sizes in cluster:
V$CDPCR3_01, V$IRF_Q6_01, V$IRF7_01, V$ISRE_01, VIRF_Q6, VICSBP_Q6

Corresponding mean of effect sizes of each TF:
(In the same order as above)
1.69, 1.05, 0.99, 0.82, 0.82, 0.73

Corresponding total number of TF hits for each TF (genome-wide):
(In the same order as above)
490, 548, 478, 507, 542, 522

Corresponding total number of TF hits for each TF (in all genes in selected Phenopedia diseases):
(In the same order as above)
195, 192, 151, 169, 203, 176

Corresponding number of genes (of selected Phenopedia diseases) each TF is involved with:
(In the same order as above)
31, 45, 33, 37, 43, 39

Corresponding number of selected Phenopedia diseases each TF is involved with:
(In the same order as above)
31, 31, 31, 31, 31, 30

Disease groups of selected Phenopedia diseases ranked according to sum of effect sizes
Immune System Diseases | Signs and Symptoms | Endocrine System Diseases | Autoimmune Diseases | Genetic Diseases, Inborn | Digestive System Diseases | Biliary Tract Diseases | Eye Diseases | Sex Chromosome Disorders | Genetic Diseases, X-Linked | Thyroid Diseases | Cholestasis | Bile Duct Diseases | Adrenal Insufficiency | Adrenal Gland Diseases | Nervous System Diseases | Multiple Sclerosis | Demyelinating Diseases | Demyelinating Autoimmune Diseases, CNS | Autoimmune Diseases of the Nervous System | Hyperthyroidism | Orbital Diseases | Goiter | Uveomeningoencephalitic Syndrome |

Uveitis, Posterior | Uveitis | Uveal Diseases | Panuveitis | Endophthalmitis | Cholangitis, Sclerosing | Cholangitis | Blood Coagulation Disorders | Multiple Sclerosis, Relapsing-Remitting | Hemophilia B | Graves Disease | Exophthalmos | Hemophilia A | Addison Disease | Immunologic Deficiency Syndromes | Common Variable Immunodeficiency | Lymphoproliferative Disorders | Granuloma | Cholestasis, Intrahepatic | Liver Diseases | Liver Cirrhosis, Biliary | Liver Cirrhosis | Cholestasis, Extrahepatic | Periodontal Diseases | Chronic Periodontitis | Coagulation Protein Disorders | Musculoskeletal Diseases | Bone Marrow Diseases | Bone Diseases | Anemia, Aplastic | Multiple Sclerosis, Chronic Progressive | Periodontitis | Myasthenia Gravis | Graves Ophthalmopathy | Eye Diseases, Hereditary | Blood Coagulation Disorders, Inherited | Airway Obstruction | Thyroiditis, Autoimmune | Thyroiditis | Thoracic Diseases | Respiratory Tract Diseases | Bronchial Hyperreactivity | Bronchial Diseases | Hypothyroidism | Hashimoto Disease

Corresponding sum of effect sizes for each disease group of selected Phenopedia diseases
(In the same order as above)
48.23, 45.24, 42.57, 39.48, 35.24, 34.24, 34.24, 29.36, 27.41, 27.41, 25.16, 20.29, 20.29, 17.41, 17.41, 16.70, 16.70, 16.70, 16.70, 16.70, 14.97, 14.91, 14.87, 14.45, 14.45, 14.45, 14.45, 14.45, 14.45, 13.95, 13.95, 13.89, 11.87, 9.87, 9.73, 9.73, 9.45, 8.76, 8.75, 8.75, 8.09, 8.09, 6.72, 6.56, 6.56, 6.56, 6.56, 6.17, 6.17, 5.88, 4.85, 4.85, 4.85, 4.85, 4.83, 4.38, 4.28, 3.94, 3.94, 3.89, 3.74, 3.32, 3.32, 2.74, 2.74, 2.74, 2.74, 1.74, 1.26

Cluster for columns 605 to 609, rows 218 to 232

Phenopedia diseases

Carcinoma, Pancreatic Ductal | Micronuclei, Chromosome-Defective | Chromosome Aberrations | Chromosome Deletion | Chromosome Breakage

TFs

V$E2F_Q6_01 | V$E2F_Q4_01 | V$E2F_Q3_01 | V$E2F1_Q4_01 | V$E2F_03 | V$E2F1_Q6_01 | V$E2F_Q6 | V$E2F_Q4 | V$E2F_Q3 | V$E2F4DP2_01 | V$E2F1DP2_01 | V$E2F1DP1_01 | V$E2F4DP1_01 | V$E2F1DP1RB_01 | V$E2F_02

Information

All related TFs:
(List of all TFs that are related to any of the PWMs)
DP-1, E2F, E2F+E4, E2F-1, E2F-1:DP-1, E2F-1:DP-2, E2F-2, E2F-3a, E2F-4, E2F-4:DP-1, E2F-4:DP-2, E2F-5, E2F-7, pRb:E2F-1:DP-1

Ranked gene list:
(All genes of the selected Phenopedia diseases with hits of any of the selected TFs, ranked according to the total number of TF hits)
MTHFR, MDM2, SGCE, TP53, RFC1, CDKN1B, TP73, KPNB1, GFI1, CCND1, SPOCK2, FANCC, PIM1, MATR3, CDKN1A, MTR, DCK, XPC, IKZF1, XRCC6, RB1, NBN, IGF1R, GCH1, PI4KA, MSH2, MLH1, MAPT, BRAF, ZWILCH, RRM1, PRKDC, NF2, APOE, RAD54L, NPM1, HFE, GNB1L, XRCC5, RAD51, MYC, CTNNB1, CHEK1

Corresponding total number of TF hits:
(For each gene listed above, the total number of TF hits for any of the selected TFs, multiplied by the number of selected Phenopedia diseases containing that gene)
35, 30, 20, 16, 15, 15, 14, 14, 14, 13, 12, 11, 10, 10, 10, 9, 9, 8, 8, 6, 6, 6, 6, 6, 4, 4, 4, 4, 4, 3, 3, 3, 3, 3, 2, 2, 2, 2, 1, 1, 1, 1, 1

Corresponding number of selected Phenopedia diseases each gene is involved with:
(In the same order as above)
5, 3, 2, 4, 3, 1, 1, 2, 1, 1, 2, 1, 1, 2, 1, 3, 1, 2, 1, 2, 2, 3, 1, 2, 2, 1, 1, 1, 1, 1, 1, 1, 1, 1, 1, 1, 1, 2, 1, 1, 1, 1, 1

Corresponding number of selected TFs each gene is involved with:
(In the same order as above)
7, 10, 10, 4, 5, 15, 14, 7, 14, 13, 6, 11, 10, 5, 10, 3, 9, 4, 8, 3, 3, 2, 6, 3, 2, 4, 4, 4, 4, 3, 3, 3, 3, 3, 2, 2, 2, 1, 1, 1, 1, 1, 1

Phenopedia diseases ranked according to mean of effect sizes in cluster:
Carcinoma, Pancreatic Ductal | Chromosome Aberrations | Chromosome Breakage | Micronuclei, Chromosome-Defective | Chromosome Deletion

Corresponding mean of effect sizes of each Phenopedia disease:
(In the same order as above)
2.54, 1.64, 0.85, 0.70, 0.69

Corresponding total number of genes of each Phenopedia disease:
(In the same order as above)
37, 219, 23, 28, 86

Corresponding number of genes of each Phenopedia disease with at least one TF hit (of selected TFs):
(In the same order as above)
9, 37, 5, 5, 12

Corresponding total number of TF hits (of selected TFs) for each Phenopedia disease (in all genes):
(In the same order as above)
61, 187, 21, 22, 60

Corresponding number of selected TFs each Phenopedia disease is involved with:
(In the same order as above)
15, 15, 14, 15, 15

TFs ranked according to mean of effect sizes in cluster:
V$E2F1DP1RB_01, V$E2F4DP1_01, V$E2F_02, V$E2F_Q4_01, V$E2F_Q6_01, V$E2F_03, V$E2F_Q4, V$E2F1_Q4_01, V$E2F_Q3, V$E2F4DP2_01, V$E2F1DP2_01, V$E2F1DP1_01, V$E2F_Q3_01, V$E2F_Q6, V$E2F1_Q6_01

Corresponding mean of effect sizes of each TF:
(In the same order as above)
2.17, 2.06, 1.76, 1.71, 1.61, 1.50, 1.34, 1.30, 1.19, 1.08, 0.96, 0.95, 0.94, 0.83, -0.13

Corresponding total number of TF hits for each TF (genome-wide):
(In the same order as above)
448, 442, 442, 432, 452, 450, 438, 437, 442, 452, 454, 450, 431, 430, 447

Corresponding total number of TF hits for each TF (in all genes in selected Phenopedia diseases):
(In the same order as above)
33, 31, 28, 24, 26, 22, 23, 22, 23, 24, 22, 22, 18, 20, 13

Corresponding number of genes (of selected Phenopedia diseases) each TF is involved with:
(In the same order as above)
21, 19, 18, 14, 16, 13, 12, 15, 14, 17, 16, 17, 12, 12, 11

Corresponding number of selected Phenopedia diseases each TF is involved with:
(In the same order as above)
5, 5, 5, 5, 5, 5, 5, 5, 5, 5, 5, 5, 5, 5, 4

Disease groups of selected Phenopedia diseases ranked according to sum of effect sizes
Pancreatic Neoplasms | Pancreatic Diseases | Neoplasms, Glandular and Epithelial | Neoplasms, Ductal, Lobular, and Medullary | Neoplasms | Gastrointestinal Diseases | Endocrine System Diseases | Endocrine Gland Neoplasms | Digestive System Neoplasms | Digestive System Diseases | Carcinoma, Pancreatic Ductal | Carcinoma, Ductal | Adenoma, Oxyphilic | Adenocarcinoma | Chromosome Aberrations | DNA Damage | Chromosome Breakage | Micronuclei, Chromosome-Defective | Chromosome Deletion

Corresponding sum of effect sizes for each disease group of selected Phenopedia diseases
(In the same order as above)
38.06, 38.06, 38.06, 38.06, 38.06, 38.06, 38.06, 38.06, 38.06, 38.06, 38.06, 38.06, 38.06, 38.06, 37.34, 12.74, 12.74, 10.49, 10.39

Cluster for columns 634 to 641, rows 193 to 201

Phenopedia diseases

Blood Platelet Disorders | Thrombocytopenia | Neutropenia | Agranulocytosis | Leukopenia | Mucositis | Cystitis | Prenatal Exposure Delayed Effects

TFs

V$STAT_01 | V$STAT3_01 | V$STAT1_01 | V$NFKB_C | V$NFKAPPAB_01 | V$NFKAPPAB65_01 | V$CREL_01 | V$NFKB_Q6_01 | V$NFKB_Q6

Information

All related TFs:
(List of all TFs that are related to any of the PWMs)
NF-TNF, NF-kappaB, NF-kappaB(-like), NF-kappaB2, RelA-p65, STAT1alpha, STAT1beta, STAT2, STAT3, STAT4, STAT5A, STAT5B, STAT6, c-Rel, p100, p105, p50, p52

Ranked gene list:
(All genes of the selected Phenopedia diseases with hits of any of the selected TFs, ranked according to the total number of TF hits)
TNF, TGFB1, GSTP1, TPMT, STAT1, IRF9, IRF7, LTA, VDR, DPYD, ALDH3A1, JAK1, IRF3, IFNAR2, PARP1, XRCC1, HLA-B, HLA-A, FAS, NFKB1, MYH9, IL1RN, GP1BB, CD46, CCL2, IMPDH2, IFNAR1, NRAS, ITPA, IL10, HPS3, ERCC2, DNMT3B, TOP1, SLC19A1, OCA2, MIF, IL7R, DCLRE1C, BDNF, ADA, VWF, SOD2, SOCS1, PLAU, ITGB3, IL4R, IFNGR1, HLA-C, GBA, CYP1B1, CYP17A1, CYBA, COMT, ADRB2, VEGFA, TLR5, THPO, SLC6A4, RET, RAG2, MYD88, LIG4, JAK3, ITGAM, IFNGR2, HFE, DRD4, CDC45L

Corresponding total number of TF hits:
(For each gene listed above, the total number of TF hits for any of the selected TFs, multiplied by the number of selected Phenopedia diseases containing that gene)
35, 35, 32, 30, 20, 15, 15, 14, 12, 12, 12, 10, 10, 10, 9, 8, 8, 8, 8, 6, 6, 6, 6, 6, 6, 5, 5, 4, 4, 4, 4, 4, 4, 3, 3, 3, 3, 3, 3, 3, 3, 2, 2, 2, 2, 2, 2, 2, 2, 2, 2, 2, 2, 2, 2, 1, 1, 1, 1, 1, 1, 1, 1, 1, 1, 1, 1, 1, 1

Corresponding number of selected Phenopedia diseases each gene is involved with:
(In the same order as above)

5, 7, 8, 5, 5, 5, 5, 2, 4, 6, 3, 5, 5, 5, 3, 4, 4, 4, 2, 1, 2, 6, 2, 2, 3, 1, 5, 2, 4, 4, 1, 4, 2, 3, 1, 1, 3, 1, 1, 1, 1, 2, 1, 1, 1, 2, 2, 1, 1, 1, 1, 1, 2, 1, 2, 1, 1, 1, 1, 1, 1, 1, 1, 1, 1, 1, 1, 1, 1

Corresponding number of selected TFs each gene is involved with:
(In the same order as above)
7, 5, 4, 6, 4, 3, 3, 7, 3, 2, 4, 2, 2, 2, 3, 2, 2, 2, 4, 6, 3, 1, 3, 3, 2, 5, 1, 2, 1, 1, 4, 1, 2, 1, 3, 3, 1, 3, 3, 3, 3, 1, 2, 2, 2, 1, 1, 2, 2, 2, 2, 2, 1, 2, 1, 1, 1, 1, 1, 1, 1, 1, 1, 1, 1, 1, 1, 1, 1

Phenopedia diseases ranked according to mean of effect sizes in cluster:
Blood Platelet Disorders | Thrombocytopenia | Leukopenia | Neutropenia | Agranulocytosis | Cystitis | Prenatal Exposure Delayed Effects | Mucositis

Corresponding mean of effect sizes of each Phenopedia disease:
(In the same order as above)
2.37, 1.75, 1.48, 1.37, 1.32, 0.67, 0.59, 0.56

Corresponding total number of genes of each Phenopedia disease:
(In the same order as above)
103, 121, 107, 114, 81, 27, 52, 20

Corresponding number of genes of each Phenopedia disease with at least one TF hit (of selected TFs):
(In the same order as above)
31, 35, 31, 29, 21, 7, 12, 5

Corresponding total number of TF hits (of selected TFs) for each Phenopedia disease (in all genes):
(In the same order as above)
86, 89, 75, 78, 55, 15, 26, 15

Corresponding number of selected TFs each Phenopedia disease is involved with:
(In the same order as above)
9, 9, 9, 9, 9, 8, 9, 8

TFs ranked according to mean of effect sizes in cluster:
V$NFKAPPAB65_01, V$CREL_01, V$STAT3_01, V$NFKB_Q6, V$NFKAPPAB_01, V$NFKB_C, V$NFKB_Q6_01, V$STAT1_01, V$STAT_01

Corresponding mean of effect sizes of each TF:
(In the same order as above)
1.72, 1.66, 1.52, 1.33, 1.25, 1.24, 1.05, 0.81, 0.80

Corresponding total number of TF hits for each TF (genome-wide):
(In the same order as above)
488, 456, 453, 465, 477, 472, 467, 453, 454

Corresponding total number of TF hits for each TF (in all genes in selected Phenopedia diseases):
(In the same order as above)
59, 57, 46, 55, 52, 52, 43, 34, 41

Corresponding number of genes (of selected Phenopedia diseases) each TF is involved with:
(In the same order as above)
19, 19, 20, 20, 15, 20, 14, 17, 15

Corresponding number of selected Phenopedia diseases each TF is involved with:
(In the same order as above)
8, 8, 7, 8, 8, 8, 8, 8, 7

Disease groups of selected Phenopedia diseases ranked according to sum of effect sizes
Leukopenia | Leukocyte Disorders | Signs and Symptoms | Blood Platelet Disorders | Blood Coagulation Disorders | Neutropenia | Thrombocytopenia | Agranulocytosis | Urologic Diseases | Urinary Bladder Diseases | Male Urogenital Diseases | Female Urogenital Diseases | Cystitis | Prenatal Injuries | Prenatal Exposure Delayed Effects | Pregnancy Complications | Mucositis | Inflammation

Corresponding sum of effect sizes for each disease group of selected Phenopedia diseases
(In the same order as above)
37.62, 37.62, 37.02, 37.02, 37.02, 24.28, 15.74, 11.91, 6.00, 6.00, 6.00, 6.00, 6.00, 5.29, 5.29, 5.29, 5.07, 5.07

Cluster for columns 690 to 727, rows 218 to 232

Phenopedia diseases

Leukemia, Lymphocytic | Leukemia, Lymphocytic, Acute | Lymphoma, Lymphoblastic | Translocation, Genetic | Multiple Myeloma | Plasmacytoma | Lymphoma, Follicular | Lymphoma, B-Cell | Lymphoma, Large-Cell | Lymphoma, Large-Cell, Diffuse | Carcinoma in Situ | DNA Virus Infections | Uterine Diseases | Uterine Neoplasms | Cervical Intraepithelial Neoplasia | Uterine Cervical Diseases | Uterine Cervical Neoplasms | Papillomavirus Infections | Tumor Virus Infections | Carcinoma, Non-Small-Cell Lung | Nasopharyngeal Neoplasms | Mouth Neoplasms | Laryngeal Diseases | Laryngeal Neoplasms | Otorhinolaryngologic Neoplasms | Pharyngeal Diseases | Pharyngeal Neoplasms | Eye Neoplasms | Retinoblastoma | Neoplasms, Second Primary | Leukemia, Lymphocytic, Acute, L1 | Neoplasms, Multiple Primary | Skin Neoplasms | Carcinoma, Basal Cell | Neoplasms, Basal Cell | Bone Neoplasms | Osteosarcoma | Sarcoma

TFs

V$E2F_Q6_01 | V$E2F_Q4_01 | V$E2F_Q3_01 | V$E2F1_Q4_01 | V$E2F_03 | V$E2F1_Q6_01 | V$E2F_Q6 | V$E2F_Q4 | V$E2F_Q3 | V$E2F4DP2_01 | V$E2F1DP2_01 | V$E2F1DP1_01 | V$E2F4DP1_01 | V$E2F1DP1RB_01 | V$E2F_02

Information

All related TFs:
(List of all TFs that are related to any of the PWMs)
DP-1, E2F, E2F+E4, E2F-1, E2F-1:DP-1, E2F-1:DP-2, E2F-2, E2F-3a, E2F-4, E2F-4:DP-1, E2F-4:DP-2, E2F-5, E2F-7, pRb:E2F-1:DP-1

Ranked gene list:
(All genes of the selected Phenopedia diseases with hits of any of the selected TFs, ranked according to the total number of TF hits)
CCND1, MDM2, MTHFR, CDKN1A, PCNA, TP73, TP53, GNAS, EXO1, XPC, PIK3CA, CDKN1B, MTR, LIG4, COL1A1, AKT1, BRAF, IRF4, IRF1, IRS2, BCL6, MLH1, BCL2L11, BAT3, RFC1, PIM1, MTHFD1, SMAD7, MSH2, IRS1, VDR, NBN, HFE, TAPBP, IKZF1, PAX5, IGF1R, MS, REV1, PTCH1, IFNAR1, CDKN2C, STAT1, CD3EAP, WRAP53, RCC2, PTCH, MSH3, HIF1A, CDK6, RASSF1, DAXX, ABCB1, PSMB9, PSMB8, GFI1, GEMIN4, CBS, SLC3A2, SIPA1, PPP1R13L, NR3C1, MAPT, DOK1, CASP7, BCL7C, FUS, CTNNB1, XRCC6, RXRB, DPYD, CYP11A1, APOE, TOPBP1, POLI, PMS1, PCGF2, ACP1, LIG1, XRCC5, RB1, NF2, MYC, MGRN1, LRCH1, KEAP1, GBA, CXCR4, TPMT, NOTCH1, RAD51, PROX1, MX1, HMGA2, DGCR8, COL18A1, BCL2L1, AHRR, SUMO1, RRM1, NME1, NHEJ1, LRP5, HRAS, GLI1, CDKN2D, CDKN1C, NPM1, ITGB3, IGF2, GSK3B, ELF2, UBE2I, STX17, RECQL4, MBD1, MAP3K12, DICER1, CHEK1

Corresponding total number of TF hits:
(For each gene listed above, the total number of TF hits for any of the selected TFs, multiplied by the number of selected Phenopedia diseases containing that gene)
364, 280, 252, 230, 225, 196, 136, 135, 117, 92, 90, 90, 78, 77, 77, 72, 68, 65, 64, 60, 60, 56, 52, 52, 50, 50, 49, 45, 44, 40, 39, 36, 36, 35, 32, 30, 30, 27, 24, 24, 24, 24, 22, 20, 18, 18, 18, 18, 18, 18, 15, 15, 15, 14, 14, 14, 14, 14, 12, 12, 12, 12, 12, 12, 12, 12, 11, 10, 9, 9, 9, 9, 9, 8, 8, 8, 8, 8, 7, 6, 6, 6, 6, 6, 6, 6, 6, 6, 5, 5, 4, 4, 4, 4, 4, 4, 4, 4, 3, 3, 3, 3, 3, 3, 3, 3, 3, 2, 2, 2, 2, 2, 1, 1, 1, 1, 1, 1, 1

Corresponding number of selected Phenopedia diseases each gene is involved with:
(In the same order as above)
28, 28, 36, 23, 15, 14, 34, 9, 9, 23, 15, 6, 26, 11, 7, 8, 17, 5, 8, 4, 4, 14, 4, 4, 10, 5, 7, 5, 11, 4, 13, 18, 18, 7, 4, 3, 5, 9, 6, 4, 2, 3, 11, 5, 6, 3, 3, 3, 6, 2, 1, 3, 15, 7, 7, 1, 1, 7, 2, 4, 3, 4, 3, 1, 3, 4, 1, 10, 3, 1, 3, 3, 3, 1, 8, 8, 1, 2, 1, 6, 2, 2, 6, 1, 1, 2, 2, 3, 5, 5, 4, 1, 1, 2, 1, 2, 4, 1, 1, 1, 1, 1, 3, 3, 3, 1, 1, 1, 1, 2, 2, 1, 1, 1, 1, 1, 1, 1, 1

Corresponding number of selected TFs each gene is involved with:
(In the same order as above)
13, 10, 7, 10, 15, 14, 4, 15, 13, 4, 6, 15, 3, 7, 11, 9, 4, 13, 8, 15, 15, 4, 13, 13, 5, 10, 7, 9, 4, 10, 3, 2, 2, 5, 8, 10, 6, 3, 4, 6, 12, 8, 2, 4, 3, 6, 6, 6, 3, 9, 15, 5, 1, 2, 2, 14, 14, 2, 6, 3, 4, 3, 4, 12, 4, 3, 11, 1, 3, 9, 3, 3, 3, 3, 8, 1, 1, 8, 4, 7, 1, 3, 3, 1, 6, 6, 3, 3, 2, 1, 1, 1, 4, 4, 2, 4, 2, 1, 4, 3, 3, 3, 3, 1, 1, 1, 3, 3, 2, 2, 1, 1, 2, 1, 1, 1, 1, 1, 1, 1

Phenopedia diseases ranked according to mean of effect sizes in cluster:
Bone Neoplasms | Sarcoma | Uterine Diseases | Lymphoma, Lymphoblastic | Leukemia, Lymphocytic, Acute, L1 | Lymphoma, Follicular | Neoplasms, Basal Cell | Carcinoma, Basal Cell | Leukemia, Lymphocytic | Carcinoma, Non-Small-Cell Lung | Uterine Neoplasms | Uterine Cervical Diseases | Otorhinolaryngologic Neoplasms | Leukemia, Lymphocytic, Acute | Osteosarcoma | Mouth Neoplasms | Laryngeal Neoplasms | Laryngeal Diseases | Uterine Cervical Neoplasms | Pharyngeal Diseases | Papillomavirus Infections | Lymphoma, Large-Cell, Diffuse | Lymphoma, Large-Cell | Neoplasms, Multiple Primary | Pharyngeal Neoplasms | Multiple Myeloma | Neoplasms, Second Primary | Translocation, Genetic | Cervical Intraepithelial Neoplasia | Tumor Virus Infections | Skin Neoplasms | DNA Virus Infections | Retinoblastoma | Eye Neoplasms | Lymphoma, B-Cell | Plasmacytoma | Nasopharyngeal Neoplasms | Carcinoma in Situ

Corresponding mean of effect sizes of each Phenopedia disease:
(In the same order as above)
1.76, 1.75, 1.74, 1.65, 1.51, 1.47, 1.46, 1.46, 1.46, 1.45, 1.44, 1.39, 1.36, 1.30, 1.27, 1.27, 1.24, 1.24, 1.12, 1.11, 1.00, 0.97, 0.95, 0.93, 0.86, 0.85, 0.84, 0.83, 0.83, 0.79, 0.76, 0.76, 0.75, 0.68, 0.66, 0.61, 0.34, 0.23

Corresponding total number of genes of each Phenopedia disease:
(In the same order as above)
40, 64, 213, 111, 39, 84, 71, 71, 170, 223, 210, 143, 161, 138, 34, 159, 90, 90, 142, 140, 101, 116, 117, 50, 132, 85, 62, 34, 89, 110, 154, 199, 25, 19, 134, 80, 73, 54

Corresponding number of genes of each Phenopedia disease with at least one TF hit (of selected TFs):
(In the same order as above)
11, 16, 38, 25, 11, 18, 16, 16, 33, 38, 36, 25, 23, 29, 9, 28, 13, 13, 24, 19, 17, 19, 19, 14, 14, 19, 15, 5, 12, 18, 26, 28, 7, 7, 20, 18, 8, 9

Corresponding total number of TF hits (of selected TFs) for each Phenopedia disease (in all genes):
(In the same order as above)
69, 92, 219, 157, 67, 99, 89, 89, 216, 210, 204, 154, 142, 172, 48, 163, 94, 94, 143, 128, 113, 108, 108, 74, 107, 94, 79, 35, 83, 115, 145, 160, 46, 37, 111, 81, 52, 44

Corresponding number of selected TFs each Phenopedia disease is involved with:
(In the same order as above)
15, 15, 15, 15, 15, 15, 15, 15, 15, 15, 15, 15, 15, 15, 15, 15, 15, 15, 15, 15, 15, 15, 15, 15, 15, 15, 15, 15, 15, 15, 15, 15, 15, 15, 15, 15, 15, 15

TFs ranked according to mean of effect sizes in cluster:
V$E2F_Q6_01, V$E2F_Q4_01, V$E2F_Q4, V$E2F1DP1RB_01, V$E2F4DP1_01, V$E2F_Q3, V$E2F_Q6, V$E2F_02, V$E2F_03, V$E2F1_Q4_01, V$E2F1_Q6_01, V$E2F1DP1_01, V$E2F_Q3_01, V$E2F1DP2_01, V$E2F4DP2_01

Corresponding mean of effect sizes of each TF:
(In the same order as above)
1.48, 1.41, 1.24, 1.24, 1.22, 1.18, 1.17, 1.13, 1.09, 1.03, 1.01, 0.97, 0.96, 0.80, 0.69

Corresponding total number of TF hits for each TF (genome-wide):
(In the same order as above)
452, 432, 438, 448, 442, 442, 430, 442, 450, 437, 447, 450, 431, 454, 452

Corresponding total number of TF hits for each TF (in all genes in selected Phenopedia diseases):
(In the same order as above)
318, 286, 290, 306, 302, 291, 290, 282, 291, 263, 274, 277, 246, 270, 255

Corresponding number of genes (of selected Phenopedia diseases) each TF is involved with:
(In the same order as above)
45, 41, 43, 40, 38, 43, 43, 41, 36, 41, 47, 49, 38, 46, 47

Corresponding number of selected Phenopedia diseases each TF is involved with:
(In the same order as above)
38, 38, 38, 38, 38, 38, 38, 38, 38, 38, 38, 38, 38, 38, 38

Disease groups of selected Phenopedia diseases ranked according to sum of effect sizes
Neoplasms | Musculoskeletal Diseases | Uterine Diseases | Genital Diseases, Female | Female Urogenital Diseases | Head and Neck Neoplasms | Bone Diseases | Otorhinolaryngologic Diseases | Stomatognathic Diseases | Mouth Diseases | Jaw Diseases | Signs and Symptoms | Uterine Neoplasms | Pelvic Neoplasms | Genital Neoplasms, Female | Abdominal Neoplasms | Uterine Cervical Diseases | Neoplasms, Glandular and Epithelial | Soft Tissue Neoplasms | Sarcoma | Neoplasms, Basal Cell | Lymphoma, T-Cell | Lymphoma, Non-Hodgkin | Lymphoma | Hematologic Neoplasms | Virus Diseases | DNA Virus Infections | Communicable Diseases | Osteitis | Laryngitis | Laryngeal Diseases | Gastrointestinal Diseases | Digestive System Neoplasms | Digestive System Diseases | Pharyngeal Diseases | Uterine Cervical Neoplasms | Tumor Virus Infections | Soft Tissue Infections | Skin Diseases, Viral | Skin Diseases, Infectious | Skin Diseases | Papillomavirus Infections | Infection | Bone Neoplasms | Carcinoma | Lymphoma, Lymphoblastic | Leukemia, Lymphocytic, Acute, L1 | Lymphoma, Follicular | Carcinoma, Basal Cell | Leukemia, Lymphocytic | Thoracic Neoplasms | Thoracic Diseases | Respiratory Tract Diseases | Lung Neoplasms | Lung Diseases | Carcinoma, Non-Small-Cell Lung | Carcinoma, Bronchogenic | Otorhinolaryngologic Neoplasms | Leukemia, Lymphocytic, Acute | Osteosarcoma | Neoplasms, Connective Tissue | Neoplasms, Bone Tissue | Connective Tissue Diseases | Mouth Neoplasms | Lymphoma, B-Cell | Laryngeal Neoplasms | Pharyngeal Neoplasms | Lymphoma, Large-Cell, Diffuse | Lymphoma, Large-Cell | Neoplasms, Multiple Primary | Vasculitis, Hypersensitivity | Vasculitis | Vascular Diseases | Sex Chromosome Disorders | Purpura, Schoenlein-Henoch | Purpura | Peripheral Vascular Diseases | Paraproteinemias | Neoplasms, Plasma Cell | Multiple Myeloma | Lymphoproliferative Disorders | Immunoproliferative Disorders | Immune System Diseases | Hemostatic Disorders | Hemorrhage | Genetic Diseases, X-Linked | Genetic Diseases, Inborn | Cardiovascular Diseases | Blood Protein Disorders | Blood Coagulation Disorders | Autoimmune Diseases | Neoplasms, Second Primary | Translocation, Genetic | Chromosome Aberrations | Uterine Cervical Dysplasia | Cervical Intraepithelial Neoplasia | Skin Neoplasms | Retinoblastoma | Neuroendocrine Tumors | Eye Neoplasms | Eye Diseases | Plasmacytoma | Nasopharyngeal Neoplasms | Nasopharyngeal Diseases |

Carcinoma in Situ

Corresponding sum of effect sizes for each disease group of selected Phenopedia diseases
(In the same order as above)
359.10, 117.05, 97.96, 97.96, 97.96, 86.15, 63.42, 54.98, 53.63, 53.63, 53.63, 50.96, 50.92, 50.92, 50.92, 50.92, 50.20, 47.36, 45.27, 45.27, 43.89, 41.04, 41.04, 41.04, 41.04, 38.20, 38.20, 38.20, 37.09, 37.09, 37.09, 37.00, 37.00, 37.00, 34.58, 29.32, 26.85, 26.85, 26.85, 26.85, 26.85, 26.85, 26.85, 26.33, 25.41, 24.76, 22.60, 21.99, 21.95, 21.91, 21.74, 21.74, 21.74, 21.74, 21.74, 21.74, 21.74, 20.40, 19.55, 19.06, 19.06, 19.06, 19.06, 19.05, 19.04, 18.55, 17.95, 14.49, 14.23, 14.01, 12.76, 12.59, 12.51, 12.51, 12.44, 12.44, 11.43, 11.25, 11.25, 10.20, 10.20, 9.13, 5.07, 5.07, 3.46

Cluster for columns 996 to 1010, rows 233 to 305

Phenopedia diseases

Endocrine System Diseases | Diabetes Mellitus, Type 2 | Metabolic Diseases | Diabetes Mellitus | Glucose Metabolism Disorders | Neurodegenerative Diseases | Alzheimer Disease | Tauopathies | Delirium, Dementia, Amnestic, Cognitive Disorders | Dementia | Nervous System Diseases | Brain Diseases | Central Nervous System Diseases | Mental Disorders | Psychiatry

TFs

V$E2F_01 | V$CREBP1_01 | V$PITX2_Q2 | V$RREB1_01 | V$RFX1_02 | V$KROX_Q6 | V$MEF2_04 | V$GABP_B | V$ELK1_02 | V$IPF1_Q4_01 | V$NRF1_Q6 | V$CREB_01 | V$CREBATF_Q6 | V$CREBP1CJUN_01 | V$ATF_B | V$CREBP1_Q2 | V$CREB_02 | V$CREB_Q4_01 | V$CREB_Q4 | V$CREB_Q2 | V$CREB_Q2_01 | V$ATF_01 | V$ATF3_Q6 | V$ATF1_Q6 | V$ATF4_Q2 | V$TAXCREB_01 | V$AP2ALPHA_03 | V$XBP1_01 | V$ATF6_01 | V$CHOP_01 | V$FOXO1_02 | V$SP3_Q3 | V$GCM_Q2 | V$ELK1_01 | V$EFC_Q6 | V$MZF1_02 | V$NF1_Q6_01 | V$NF1_Q6 | V$GR_Q6 | V$MEIS1_01 | V$LRH1_Q5 | V$YY1_Q6 | V$RFX_Q6 | V$DEC_Q1 | V$E4F1_Q6 | V$CREB_Q3 | V$NFMUE1_Q6 | V$TBX5_01 | V$RBPJK_Q4 | V$SP1_Q2_01 | V$SP1_01 | V$SP1_Q6 | V$SP1_Q4_01 | V$SP1_Q6_01 | V$AHR_Q5 | V$AHRHIF_Q6 | V$HIF1_Q5 | V$HIF1_Q3 | V$TAACC_B | V$E2F1_Q6 | V$E2F1_Q3 | V$E2F_Q2 | V$E2F1_Q3_01 | V$AP2_Q6 | V$WT1_Q6 | V$MAZ_Q6 | V$ETF_Q6 | V$EGR_Q6 | V$AP2GAMMA_01 | V$AP2ALPHA_01 | V$AP2_Q6_01 | V$HIC1_03 | V$HIC1_02

Information

All related TFs:
(List of all TFs that are related to any of the PWMs)
120-kDa, 47-kDa, AP-2, AP-2alpha, AP-2alphaA, AP-2alphaB, AP-2beta, AP-2gamma, ARNT2, ATF, ATF-1, ATF-2, ATF-4, ATF-a, ATF-like, ATF/CREB, ATF2, ATF2-isoform2, ATF3, ATF4, ATF5, ATF6, ATFa-isoform1, AhR, AhR2, AhR:Arnt, AhRR, AhRR:Arnt, Arnt, BHLHB2, BHLHB3, C/EBP, C/EBPalpha, CHOP-10, CRE-BP1, CRE-BP2, CREB, CREBbeta, CREMalpha, CREMbeta, CREMgamma, CREMtau, CREMtau1, CREMtau2, CREMtaualpha, CTF, CTF-1, CTF-2, DEC1, DEC2, DP-1, E2F, E2F+E4, E2F-1, E2F-3a, E2F-4, E4F1, ETF, Egr-1, Egr-2, Egr-3, Egr-4, Elk-1, Elk-1-isoform1, FOXO1, GABP-alpha, GABP-alpha:GABP-beta, GABP-beta1, GABP-beta2, GCMa, GCMb, GR, GR-alpha, GR-beta, HIC-1, HIF-1, HIF-1alpha, HIF-1alpha-isoform1, IPF1, IPF1:Pbx, MAZ, MEF-2A, MZF1B-C, Meis-1, Meis-1-1, Meis-1-2, Meis-1-3, Meis-1a, Meis-1b, NF-1, NF-1/L, NF-1A, NF-muE1, NRF-1, PITX2, PITX2A, Pitx2, RBP-Jkappa, RFX1, RFX1:RFX2, RFX1:RFX3, RFX2, RFX3, RFX4, RFX5, RFX5:RFXAP:RFXANK, RFXANK, RFXAP, RREB-1, Sp1, Sp2, Sp3, Sp4, Stra13, TBX5, Tax, WT1, WT1-del2, XBP-1, YY1, aMEF-2, c-Jun, deltaCREB

Ranked gene list:
(All genes of the selected Phenopedia diseases with hits of any of the selected TFs, ranked according to the total number of TF hits)
AKT1, RXRB, GPX4, ANKMY1, EFNA3, JUNB, VAMP2, PTPN6, THRA, TNFRSF6B, DVL1, RARG, PARD6A, CDK5, DGAT1, OPRL1, PNPLA2, PAX6, LZTS2, IGF1R, GNAS, FOXO3, ADORA2A, PRDX2, CPT1A, TCF7, PTBP1, NR4A2, VEGFA, ACHE, IRS1, HRAS, RARA, LDB1, VEGF, EGR3, DDIT3, TRIM8, GNAI2, DBP, TRAF4, KCNIP2, ABCA2, VGF, SOCS1, EGR2, COL11A2, PHF1, PSENEN, CDK4, C12orf57, IRS2, GAPDH, CAMK2G, BRD2, NR3C1, LTC4S, APOM, MXI1, FOSB, ATXN1, TGFB1, EBF3, PPARGC1B, NR1H2, CTSD, S100A6, FGFR3, STRA13, TYMP, MYST4, KCNH2, HMGA1, AGER, TPT1, SH2B3, CASP9, PRDX5, GRIN3B, NOTCH4, KLF16, CCNI, SOCS3, CCND2, UBE2I, SPAG6, GRN, TNFAIP3, SREBF1, IGF2, ADAM8, RRP1, GATA2, PSD, CDKN1B, CALY, TRAF3, ARF1, USF2, TNFAIP1, PER1, CSNK1D, ATN1, SNX5, KLF13, PFKFB3, NDUFA10, DDAH2, SLC39A7, CREBBP, SLC23A3, UBQLN1, RUNX2, NFKB2, LMNA, LCAT, CCND1, CAPN10, ADM, PANK4, LBH, BAD, AHI1, RING1, PHF12, FRS3, SYNGAP1, IL11, UBL5, POU3F2, SGMS1, PPARD, FRAT1, CSNK1G2, ARSA, PSMB9, NKX2-2, CDKN1C, GALT, TFCP2, RXRA, RAGE, NP, GGT1, CYP46A1, LTBP4, IRF1, ECGF1, CYBA, TSC2, PTCH1, PRKCD, MEF2D, IL10RA, BRAF, PPP1R10, NDUFS8, NDUFB10, DYRK1A, DNAJB2, NR4A3, KLF11, ARVCF, SCA13, PARK2, MIF, MCM10, KLF6, KCNC3, ICAM4, CREM, CPT1B, ADIPOR1, SIGMAR1, RFC1, CDH23, BACH2, WNT10B, SPOCK2, PPIL2, PLCG1, PDGFB, KAZALD1, CLPTM1L, ARL5B, PCK1, MTHFR, KLF12, ESRRA, TRAF2, KCNQ1, GABARAP, BGLAP, TBL3, POU2F1, OPRS1, NR3C2, LAG3, ZFYVE27, TAF8, PTMS, PDZD8, NGFR, FYN, ENO2, ARHGAP12, LRP1, SLC2A4RG, PCNA, TUBA4A, LBXCOR1, RTEL1, SQSTM1, TRAF5, NF1, YME1L1, TBX2, TAPBP, RNPEPL1, RGS10, NOLC1, NDUFV1, KCTD15, ITPRIP, IL6R, HSPA1L, GTPBP3, DNMT3A, CTBP2, BTAF1, ARHGAP21, ADRA2A, TP73, CNTFR, C6orf47, SBF1, VEGFB, ATP6V0C, CDK6, STK11, SCAP, PEA15, MAPT, KLF10, FASTK, CLIC1, APBB1, RECQL4, ZEB1, YWHAZ, PIGQ, PHLDB1, NDUFB7, LLGL2, HSPA14, EIF4EBP2, CDK5R1, VIM, UBE2H, SEMA3B, PBX2, SST, PSRC1, PIP4K2B, CTBP1, APEX1, TSPAN9, TSPAN8, PITX3, LHB, DOM3Z, AQP10, ZNF76, VDR, STXBP3, STX1A, PPARGC1A, PPARA, NOTCH1, LEPR, HHEX, NFKBIE, TM9SF3, SEPT9, PFKP, NR1D1, NFKBIA, HNRNPF, GAPDHS, SNAP25, PRKG1, PARK7, MEN1, LY6G5B, HMGCR, GRB2, FOXO1, FASN, FADS1, CRTC2, CNDP2, CHRNB2, ABCB1, TAPBPL, SOAT1, MC1R, JARID2, GBA, THADA, PSMB8, PRKACB, HMGCS1, EXO1, AIRE, CLEC16A, CAMTA1, ZCCHC24, TPP1, SEPHS1, SEC31B, PVRL2, NDUFS6, NDUFB8, MTIF3, INSIG2, H6PD, GAB2, FUT7, DUSP5, ACSL5, UCP2, TLR9, SOCS2, SLC9A3R1, SLC26A6, PRKCZ, PNMT, PIN1, KLF5, EIF3A, CYP27B1, BCL11A, NSD1, VLDLR, TXN, STAT3, NRF1, LRP6, IGF2BP2, GCLC, FUS, CLCN6, CAMK1D, AIF1, WFS1, TH, SIT1, PTPRS, PCNT, MGMT, LGR5, LEPREL2, GSK3B, CHGB, TMEM39A, SIX5, RUNX1, PITRM1, NINJ1, MAPKAPK3, LMX1B, HIF1AN, GBF1, EGR1, CXCR4, CSTF2T, BHLHE40, SREBF2, SHB, PTGER1, PRKAA1, NPC1, LTB4R, IL18R1, FADS2, DLST, DAXX, COL1A1, APH1A, ALOX5, AGRN, NELF, CYP26B1, PRKAG2, MSH5, KNS2, KLC1, FOXO1A, DLX2, MAP3K6, WDR37, SOX2, SOS1, SLC18A2, SGTA, PPP1R1B, OPA3, NUDT1, MGAT1, LGI4, ARRB2, AMT, AGRP, XPNPEP1, USF1, TYROBP, TXNL1, TRPC4AP, SPTBN4, SLC27A5, SGPL1, SEC24C, PTPRA, PIK3R1, PBLD, OAZ1, NDUFS7, MUC1, MT2A, MMP9, MAZ, LTB, LPIN1, LDLR, KLF3, KLF2, INSIG1, INS, HNRNPH3, FTL, EIF6, DUSP13, DRD4, CYP1A1, CXCL12, CUGBP2, CUEDC2, CLCN2, CDKN1A, C10orf125, BDNF, ATE1, ANUBL1, ADK, TMEM217, PTGDS, PLXNC1, LIG1, JAG1, CDC42, BAK1, TACC3, TAF4, SMAD3, SHMT2, SCARB1, PTGIR, PRKCQ, PPRC1, PIK3CA, PCBD1, IKBKAP, ID2, HK1, GPAM, FGFR1, DCTN1, CREB5, CREB1, CHD7, ADRA1A, CFL1, ZDHHC8, TSC22D1, TOMM40, TMEM106B, SHANK3, RHOA, QKI, PPP3CA, PDXK, NR1H3, M6PR, JAG2, IMPDH2, HMOX2, HDAC4, GRK5, GALNT2, DLG4, CNTF, BAG3, ATXN3, VKORC1, TRAF7, TBXA2R, TAF1, SS18L1, NFRKB, NCSTN, MYL5, MLYCD, MLX, LOXL3, IFNGR1, HSPD1, FSTL4, FLNA, CNTNAP1, CHAF1A, BRD8, BMP1, APH1B, ACAT1, NR4A1, HES1, GRIN2D, HDAC10, ZNF248, ZNF184, UHRF1, SVIL, SUFU, SFXN4, SAMD8, POLG, PANX2, NDUFS3, NDUFA7, MYO1C, MVK, MLF1, METTL10, MAOA, KISS1R, KCNJ10, HOMER1, HCN2, GLUD1, GIPR, FOXP1, FBXO18, EPC1, CTSZ, CCS, CCND3, CCDC28B, BHLHB2, AVPI1, ADNP, ABCD1, ZDHHC17, XRCC2, SLC27A1, RGS19, RDH5, MYH9, GABBR1, EPHA4, DOT1L, CRYBA2, ACSS2, S1PR2, GPC1, DRD1IP, SLC25A1, PYGB, POLS, PLTP, LRP8, GFRA1, CLSTN2, C1orf66, C10orf116, BIRC2, ALS2, ALOXE3, TNFRSF25, SCN5A, RNF5, RGS2, RAB5B, PACRG, ICOSLG, F12, DMPK, DLL1, CDK2, ARNTL, AGPAT1, ADRA2B,

NHEJ1, MAP3K12, TP53, TNFRSF1B, TNFRSF1A, TBX21, TARDBP, TACR2, SORL1, SMAD6, SLC6A9, SIRT1, RTN3, RBBP8, PDCD1, NQO1, NOS3, NFKBIB, MMS19, MLL, MADD, KEAP1, JAZF1, IGF2AS, IGF1, IDE, HSPA4, HPS1, GCHFR, FN1, FANCD2, EN2, DUSP1, DNM2, COX4NB, COMT, CHRNB1, CHRNA4, CELSR2, CDKN2A, CDH15, CD14, CALCA, C6orf173, ASPSCR1, APOA5, AMPD2, ACP2, RAI1, PLXNA1, HDAC7, TRAF1, TNXB, SPRED2, SMAD7, SLC2A4, RBP4, PTPN1, PSEN2, PSEN1, PRNP, PRKAB2, PRKAB1, POLB, MTR, MS, MOG, LRP5, LIF, LDHA, HSD11B2, HCRT, GSTK1, GRIN1, GPR3, GNAL, GAMT, FAM167A, CNP, CLU, CIITA, CCNY, ZNF25, ZNF239, VCL, TMIE, SMC3, SLK, SLC25A16, SH3PXD2A, SDHB, SAR1A, RP11-529I10.4, RASSF4, PPIF, POLR1D, POLL, PGBD3, PAX2, NT5C2, NDUFV3, NDUFA3, NDRG1, NCAPD2, MRPS16, MARK4, KIAA1128, JMJD1C, ITGB2, HD, GPR123, DNAJB1, DMBT1, DDX21, CWF19L1, C10orf33, C10orf10, BTRC, ARNT, ALDH18A1, ADD3, ABCA7, YWHAH, TNFAIP2, TFB2M, TFB1M, SULT4A1, SPARC, SLC29A3, RAB3A, PRDM2, NDUFAB1, ING1, HOXD13, HMHA1, EGR4, ECE1, COMP, C4B, BAT1, ALPL, ALOX5AP, ADRBK1, VPS13C, PTS, MTCH2, LSP1, LEPROT, HAS2, FFAR1, CBLB, CAP1, BCAS3, AP3D1, ZC3H10, VAMP3, UBR3, TRPM7, SIAH1, SHMT1, SEMA3F, PDE11A, MDGA1, MCOLN1, LZTR1, LMO4, LIN7C, HTT, DIDO1, DGKH, CLN8, CLN3, CHRNE, C6orf25, BAP1, ATP1A1, ADAM10, ACVR1, ACTA1, AANAT, OSM, HOXA10, ENO3, ZIC2, SPRED3, SOS2, PLCB2, PI4KB, PDLIM1, NPC2, HSPA8, FTCD, E2F4, COL6A1, BAIAP2, USP37, SSTR2, PTPN2, PER2, NXNL1, IRF5, IL21R, IL16, HOXA1, HIP1, GYLTL1B, GDNF, FABP3, EIF2AK2, CUL9, CRHR1, CHRND, CDC37, CDC25B, CARTPT, BRCA2, BRCA1, BBS7, BAT2, ATF3, AMH, ADCY3, ADAMTSL5, ABCC1, XYLT2, XYLT1, XRCC6, TMEM18, STXBP1, STMN1, SLC22A18, SLC19A1, SLC11A2, SCMH1, PTPRF, PTPRE, PRKCG, PLA2G6, PIP4K2A, PDK1, PCK2, PAK4, NTRK2, NEUROG2, MSX1, LIG4, KLF7, KLF15, KLF1, INPPL1, HTR6, GRM8, GRIN2B, GPM6B, FMR1, EPHB2, DGKE, CYB5R4, COL25A1, COL18A1, CHRNA3, CHRM1, CH25H, CDCA3, CCDC6, C12orf30, BCR, ATG9B, ATF4, AP3M2, ADAM15, ACADSB, ACAA1, PRMT1, PLCD1, HECA, AKT3, ZNF365, ZC3H3, XRCC3, VDAC2, VAPB, UNC5B, TYSND1, TXN2, TSPAN7, TNKS2, THNSL1, TBX1, TBCD, TAP1, SUPV3L1, SPG7, SLC25A28, SH3TC1, SEPT3, SEMA4G, SEC61A2, RUFY2, RPP30, PRKCH, PPIG, PMS2, PIK3AP1, PICALM, PI4K2A, PHYH, PDCD4, PCGF5, PANK1, PACS2, P4HA1, NUMBL, NR2C2, NEURL, NEGR1, NDUFS5, NDUFS1, NDEL1, NDE1, NADSYN1, MPP7, MMAB, LGI1, LASP1, KIN, KIAA0913, ITGB1, IRF8, INSR, IDH2, ICMT, HSPG2, HPCAL1, HDAC1, GRIN2C, GDI2, FAT1, FAT, ECD, DYDC2, DYDC1, DNMBP, DHCR7, CSGALNACT2, COX10, COMTD1, COG2, CNNM2, CISD1, CCAR1, CBARA1, CASP7, CALU, C10orf78, C10orf76, C10orf35, C10orf119, BIK, BAX, ATPAF2, ATP5C1, ASCL1, ARMETL1, ARMC3, ABI1, PPP1R9B, HDLBP, AXIN2, UCN, TRIO, TNFRSF14, TMEM120B, STAT4, SOCS5, SDHD, RUNX3, RNF41, PURB, PRKAR1A, POLG2, PHF8, P2RY11, NPHP1, NLGN2, NHLRC1, LEF1, ITPR3, IRAK1, INHBE, IL13, GNB1L, GIGYF2, GAA, G6PD, FOS, EPO, DOCK9, CRY2, COL2A1, CDKN2C, ALDOA, AAK1, TBX4, SOD2, SOD1, SLC2A9, SH3GL2, SGK1, SDK1, SDF2, RBPJ, PTEN, PROX1, PPARG, POLD1, PLA2G7, PEX1, PEMT, OGG1, OAS3, NUP98, NR2F1, NPY, NLRP3, NFKB1, NEUROD2, NCOR2, MRE11A, MPG, MMP3, LTB4R2, KLF4, ITGA2, IARS2, HSD11B1, HFE, GSTP1, GPATCH8, GATAD1, FGF2, F7, ETV6, ETFB, ERCC6, ELMO1, DEF6, CETP, CAT, CAPN5, C6orf106, BCL2L1, AURKB, ATXN2, AR, APOA2, ANXA5, AKT2, AGTR2, AGTR1, ADRB2, ADAMTS17, ACE, ZBED4, VCAM1, TSPO, TRIB3, TIMP1, SRR, RHOG, PYY, PSMD9, PCTK2, NRP1, MYC, MYB, MTAP, MAP2K6, LRPAP1, KIAA0319L, JPH3, JAK1, ING3, IL27, HPS4, HDAC5, HBB, GCKR, FIGNL1, FGFR2, FASLG, FAS, ERC1, EIF3D, E2F2, DNMT1, DLX1, DAG1, CRLF3, CPE, CHAT, CDC34, CDC20, CDC123, CAMKK2, C6orf27, BRD1, BMPR1A, ATP7B, ADIPOR2, ABL1, A2M, TRAF6, TNNI3, TNFSF11, TNFRSF11A, TMBIM6, TIMP2, TAF4B, SOX3, SERPINC1, RNMT, PTGER4, PRF1, PHACTR2, PDGFRB, P2RY12, MTRR, MTHFD1L, LTBP1, LRAT, LCT, INHBB, IGFBP6, CEBPD, CDH11, CD40LG, CALCR, BLMH, AHR, ZNF132, WNT7A, WNK1, VAPA, UNC84A, TRAPPC4, TOLLIP, TNIK, TNFSF14, TMCO6, TG, TFDP1, TCN1, TCF25, TCF2, SUPT3H, SUNC1, SSTR5, SPTBN2, SMAD4, SLC22A4, SLC17A7, SIRT3, SGIP1, RSRC1, RASGRP1, RASD2, RAF1, RAD54L, RAC2, RAC1, RAB7A, PTPN11, PTK2B, PTK2, PRX, PRRT1, PROC, PRKCA, PPM1K, POR, POMGNT1, PNPLA4, PLXNA2, PLAGL1, PALB2, NPAS3, NCR3, NCOA6, MON1B, MKKS, MIA3, MAP2K5, MAP1A, LRP2, ITPA, ITM2C, IL2RB, IFNAR1, HNF1B, HLF, HIST1H2AG, HES6, HBA1, GTF2H1, GSTO1, GSTA4, GRK6, GCLM, GADD45A, FOXF1, FOXC2, FEM1C, FCER2, ERCC4, EGFR, DOC2A, DGCR14, DDB2, DBH, CUX2, CHP, CENPE, CAV1, CAMKV, CALM1, CACNA1D, C7orf57, C3orf10, ATP1B1, ARRB1, AKR1B1, ACTR5, ACCN2, ACACA, ZBTB37, SYN1, SSX1,

SLC18A3, SEZ6L2, POMC, OCA2, NTRK1, MRPS6, KRT8, KIAA1462, KIAA1161, IL11RA, IKBKB, HLA-B, HIRIP3, GSTO2, GAL, ERCC2, EPHA2, DAB2, COL6A2, CHUK, CCDC22, CAMK2D, CA9, C6orf129, BECN1, ADD1, ACAT2, ZNF827, ZNF678, XRCC5, VAMP4, USP4, UQCC, UBR1, TYMS, TXNIP, TRPC1, TRIM11, TMED10, TEX14, TDG, SURF1, SLCO3A1, SLC5A6, SLC25A4, SLC12A5, SHH, SEC23IP, RGS9BP, RAPGEF1, RAD51C, PREX1, PRDM16, PRC1, PPP2R2B, PLA2G4B, PIGF, PHOX2B, PHLDA2, PDE7B, PDE6C, PARD3, OPTN, OLIG2, NKX6-1, NKX2-3, NGF, NET1, NDUFV2, NCOA4, MTHFD1, MT1A, MSRA, MSH6, LPIN2, LIPE, LIPA, LINGO1, LIMK1, LHPP, LEP, LCORL, L1CAM, ITPR2, IMPA2, IGF2BP1, IDUA, HLA-C, HIST1H2BJ, HHIP, HAS3, GYS1, GSTZ1, GSK3A, GPD2, GNPDA2, GATA4, GADD45B, FXN, FN3K, ETV5, ERCC1, EFNB2, DNM3, DCTN5, CXCL1, CTSB, CTLA4, CSPG5, CPSF2, CHRNA7, CHRFAM7A, CHI3L1, CHEK1, CEP250, CD4, CCDC148, CALHM2, CALHM1, CACNB2, CABLES1, BSCL2, ATXN7, ATP1A3, ATP10A, ARL6IP5, ARHGAP18, AP3M1, ADCY5, ACP1, ABCA12, ZNF488, ZNF485, ZNF438, ZNF33B, WAPAL, VAMP8, TXNRD2, TXNRD1, TXNDC5, TUBB, TRUB1, TREX1, TPMT, TOX, TLL2, TGFBR2, TDRD1, TCTN3, STK38L, SLC29A1, SLC16A12, SELPLG, SDHC, SCGB1A1, S100A10, RSU1, RRP12, RHOBTB1, REEP3, RALBP1, PTPLA, PSMA6, PROZ, PRND, PRDX3, PRDX1, PI4KA, PCDH21, PCDH11X, OSBPL9, OGDHL, OBFC1, NPAS2, NOC3L, NDUFS4, NDUFS2, NDUFB9, NDUFB5, NDUFA6, NDST2, MX2, MSRB2, MLEC, MAPKAP1, MAP3K8, MAL, LRRC20, KRI1, KNDC1, KCNJ9, ITPK1, IREB2, INPP5F, INA, IL6ST, IFIT1, IDI2, HSPA9, HSPA5, HSPA2, HPS6, HERC4, HECTD2, GHITM, GFAP, GDF2, GALNAC4S-6ST, FTO, FBXO8, FAM63A, FAM26C, EXOC6, EXOC3L2, ETFA, ESPN, EIF2B5, DOCK1, DMXL2, DCP1A, CUL2, CSNK1E, CRABP2, CPEB3, CLRN3, CDYL2, CCT5, CCDC86, CAST, C10orf84, C10orf57, C10orf4, C10orf26, BTG1, BSN, BBS1, AVP, ATAD1, ASCC1, ARHGAP22, ANXA7, ANXA11, ACSS1, ACOT7, ACBD5, ZNF313, ZFP64, ZFHX3, USP9X, UCN2, UBE3A, UBASH3A, TTBK2, TSNAX, TRIP12, TGFBR3, TGFBR1, TERT, TAF5L, TAF3, STK32C, STAT6, ST8SIA4, SRGAP3, SMARCC1, SMAD5, SILV, SFRS15, SEMA6C, S100B, RAD52, PYGM, PTPRN2, PTPN12, PRKDC, PRKCB, POMT1, PLXNB1, PICK1, PCMT1, PARP2, PANK2, NUCKS1, NTF5, NTF4, NFIL3, NCOR1, MYO7A, MRPL23, MMACHC, MECP2, LOXL1, LDHB, KREMEN1, IL5RA, HSD17B1, HS3ST2, HS1BP3, HPN, HIVEP3, GSC, GRIK2, GPR132, GNPTAB, GLO1, GIMAP5, GCSH, GAD1, FTH1, FOXP2, EXOC4, EP300, ELAVL4, ECE2, DPYSL2, DM1, DDR1, CR2, CLOCK, CHKB, CHGA, CDKN2D, CACNA1C, BACH1, ATP2A3, ANGPTL4, AIP, ZHX2, XPC, VCP, VAV2, TUB, TMEM127, TGFA, STAG3, SRD5A1, SLC1A4, SIK3, SHBG, SBK1, RPGRIP1L, REL, PRKCB1, PREP, PEX7, PDE8A, OSBPL2, NOTCH3, MSH2, LARP7, INO80, HTF9C, HNRNPA2B1, HCRTR2, HAP1, GLIS3, FLNC, FEN1, FANK1, FAAH, F8, DLGAP4, CCNE1, CASP8, CALCB, C4orf21, BDKRB2, ATP5B, ATM, ATAD5, ZNF699, ZNF607, ZBTB40, XBP1, WNT2B, WDR55, UST, USP20, UNG, UBE3C, TRMU, TRIB1, TP53BP1, TOX2, TIMELESS, THSD4, TGFB2, TEF, TAOK2, TACR1, SV2A, STK24, STAT1, ST7, ST6GALNAC3, ST3GAL1, SPATA13, SPAST, SP4, SNAP29, SNAP23, SMPD1, SLC6A5, SLC39A2, SLC38A1, SLC25A13, SGK, SGCE, SEMA5A, SCN1B, RUVBL2, ROBO4, ROBO3, RGS12, REV1, RCAN1, RBM19, RAPGEF3, RAD51L1, RABGAP1L, PTHLH, PRSS16, PRPH, PROS1, PRKRA, PRKAR2A, PPP2R2C, PPP1R12B, PNPT1, PNPLA8, PLEKHA1, PIP5K1B, PIGG, PGF, PER3, PBX3, PAK3, NXN, NUP210, NTRK3, NR2E1, NPPC, NMB, NEUROD1, NCALD, NAPG, MYO7B, MYCBP2, MX1, MUTED, MTP18, MTO1, MT3, MST1R, MRPL10, MPZ, MFAP4, MAT2A, MAP4, MAN1A2, LYN, LTBP2, LMNB1, LGALS1, KIAA0746, KIAA0319, KHDRBS3, KCTD12, KCNN4, ITGA3, ITGA1, IRF4, IMP5, IMMP2L, IL9, IL7R, IL21, HIPK3, HINT1, HEATR5B, H2AFY, GRIK5, GPHN, GLUL, GLS2, GGCX, FXYD6, FEV, FANCA, FAM13A, FAH, ERGIC1, EPHA8, ENG, EIF2AK1, E2F1, DYX2, DSCAML1, DPP4, DLGAP2, DGCR2, DGAT2, DCUN1D1, DACT1, CRHR2, COX5B, COL9A3, COL7A1, CLINT1, CDK5RAP2, CDC42SE2, CD40, CCT2, CCNA2, CCKBR, CCKAR, CASZ1, CASP3, CARS, C4orf32, C1QTNF5, BNIP3, BMP2, BCL2, AURKA, ATF1, APOC4, APOC2, ANG, ALDH3B1, ACSL6, ABAT, ZNF7, ZNF462, ZNF236, WT1, VGLL4, UROD, UBE2D3, TSC22D3, TRIM68, TRIM25, TIMP4, THBS2, TFRC, TARS2, SYN2, SUMO3, SULT1A1, STXBP4, SPRY4, SMARCA2, SIRT4, SIAH2, SH2B1, SEC16B, SCNN1B, RHOQ, RGS9, RB1, RAD51, PXMP3, PPP3R1, PNPLA3, PLA2G12A, PKN2, PIK3CB, PGM1, PBX1, OTX2, OR52I1, NRGN, NR0B2, NPHS1, NOVA1, NIPBL, NEUROG3, NDUFB6, MUTYH, MTF1, MRPL42, MOS, MMP26, MGEA5, MCF2L2, MAP3K5, MAP3K1, LARS2, KCNJ6, KCNJ15, KALRN, INHBA, IL26, IHH, ICK, HOXD11, HACE1, GYPC, GPR126, GNE, GNA12, GALR3, GABRG2, GABRB3, GABRB1, GABRA5, FOXA2, FAIM2, FABP6, EXT2, ESRRG, ENDOGL1, ELMO2, DUSP12, DLC1, DCD, CUL1, CTH, CRH, CPLX2, COMMD5, CEP63, CDKL5, CDC73, CD274, CCNE2, CBL, CASQ1,

C18orf45, BVES, BCDIN3D, BCAT1, AUTS1, ATP6V0A1, ATP5O, ATP13A2, ATF6, ARHGEF12, ARHGEF11, AQP2, AP3B1, ANKS1A, ANAPC13, ALX4, AKAP10, ADAR, ACTN4, ABCB6, ABCA4, ZNF9, XKR6, XIAP, WNK4, VIPR1, USH1G, ULK2, TSC1, TRIM17, TRH, TPM1, TP63, TOP2B, TOM1, TNFSF8, TDRD9, TCF12, TBL2, TAGAP, TADA1L, SYNJ1, SYNGR1, STK10, SRA1, SPRY1, SPG20, SOX10, SMG6, SMC6, SMAD1, SLTM, SLC47A1, SLC12A6, SLC12A2, SLC10A7, SIX3, SELS, SEC24D, SDCCAG8, SCNN1G, SCA4, SART1, RRM2, RRAS2, RPL10, RIMS3, RFX4, RDBP, RAP1B, QDPR, PTGFRN, PRKAG1, PPARBP, POMT2, POLH, PLEKHG4, PLCG2, PIK3CG, PHTF1, PGM3, PCNX, PCM1, P2RX6, OTX1, NRXN2, NPR1, NEFH, MUT, MRAS, METAP1, MED1, MARK3, MAP1B, LPP, KIF1B, KIAA0020, KAT2B, ITGAM, ITGA4, ITGA11, IKBKG, IKBKE, IK, IDH1, IDDM12, HTRA2, HSPB1, HSP90B1, HRH2, HNRNPUL1, HMBS, HLA-DOA, HEXA, GRP, GRM4, GNRH2, GLS, GLDC, GJA1, GH2, GBE1, GARS, GALR2, GALK1, G6PC, FZD3, FREQ, FOLR2, FNTA, FLNB, FKRP, FILIP1L, FGF14, FEZ1, FEM1A, FARP1, FANCE, ERCC3, ERBB4, DUSP6, DR1, DNMT3B, DNASE1, DIAPH1, DCLRE1C, CXXC4, CSH2, CSH1, CNBP, CHST11, CES2, CEP170, CD46, CCDC60, CAPN2, CAPG, CAMK4, CABIN1, C11orf61, BCL2L13, BBX, BANK1, B4GALNT1, ATXN10, ATF5, ATF2, AS, APBA1, ANGPTL6, ALOX12B, ALDH4A1, AKAP2, ADAM22, ADAM17, ACLY, ABCG4, ZSWIM2, ZNF79, ZFP30, ZBTB20, YPEL2, YES1, YAF2, XPO4, WNT5A, WNK2, WDR7, WDR31, VPS54, UFD1L, UBE2R2, TUBG1, TPM3, TOR1B, TNFRSF21, TIPARP, TET2, TCEB3, TBPL1, SYNE1, STRN, SRC, SPTBN5, SPTB, SORBS2, SMUG1, SMAD2, SLC7A8, SLC4A3, SLC2A3, SLC26A8, SLC16A1, SLA, SHPK, SERPINB8, RORA, RMI1, RELA, RBL2, RAPGEF6, RANBP5, RAB2A, R3HDM1, PVR, PSMA4, PNLDC1, PML, PITX2, PIAS1, PDE8B, PDE5A, PDE2A, PABPN1, OSTC, OSGIN1, OPA1, NVL, NIN, NEUROG1, NCOA7, NCF4, NCF2, NANOS3, MYLIP, MTHFS, MSX2, MST1, MSH3, MMEL1, MLL3, MGAT5, MEIS2, MEGF11, MED13, ME2, MDC1, MARCH10, MAP3K7IP2, MAP3K7, MAP3K4, LYRM7, LRPPRC, LARGE, KTN1, KIFAP3, KIF6, KIF21A, KIF14, KIAA1274, KCNMB4, KCNMB3, KCNK12, ITGAE, IPO5, IL28RA, HSPE1, HOXD12, HERPUD2, GTF2H5, GRIP1, GRIA3, GPR35, GPR135, GJB6, GFRA2, GFOD1, GABRG1, GABARAPL2, FST, FPGS, FOXG1, FNIP1, FKBP5, ERO1LB, EPHB4, EN1, EME1, EIF2B4, EIF2B3, EIF2B2, EIF2B1, EFNB3, DYX1C1, DYNC1H1, DTWD2, DSCAM, DMTF1, DFNB31, DCDC2, CYP8B1, CTNNA2, CSTB, CST2, COL1A2, COBL, CLN5, CLIC5, CILP2, CHST2, CHL1, CGA, CDC25A, CDC16, CCNH, CASP2, CASK, CALCOCO1, CACNA1S, C18orf25, C13orf18, BLM, BLK, BHLHE41, BBS4, BARD1, ATP1B4, APCS, AP1AR, ANK1, ALKBH2, AKTIP, AJAP1, AIPL1, AIM1, ADSS, ADH5, ACYP2, ACVRL1, ACCN1, ABCB4, ZNF630, ZNF423, ZNF385B, YWHAE, WNK3, WDR5, VPS33B, UGDH, TSPAN12, TPK1, TP53I3, TOPBP1, TMTC2, TDP1, TCP1, TBCK, SYT11, SYBL1, STK38, STAB1, ST8SIA1, SPTBN1, SPRED1, SPEF2, SOX5, SNW1, SMARCB1, SLC9A6, SLC6A14, SLC6A1, SLC39A3, SIRT5, SHISA5, SHANK2, SH2D2A, SGOL2, SDC3, SAT1, RTN4, RPL34, RP1-21O18.1, RIMS1, RHCG, RGS16, RGMA, PUS10, PRTG, PROKR2, PRKAR2B, PREB, PPP4C, PPP3CC, PPM1L, PPA2, POLI, PNPO, PMP22, PLK2, PLEKHH2, PLD2, PLA2G1B, PI4K2B, PHB, PGCP, PDLIM5, PDE1C, PCYT1A, PCQAP, PBK, PAX9, PAWR, PALM2, PAICS, PAFAH2, P2RX4, OXT, OSBPL5, OPN3, NUFIP1, NTNG2, NTN4, NTAN1, NRP2, NRG2, NRCAM, NRAS, NIPA1, NGFB, NEUROD6, NEURL2, NECAB2, NCOA3, NCAM2, NAP1L4, MYT1L, MYOZ3, MYO1D, MYL3, MYBPC3, MTUS1, MTMR2, MPPE1, MLH3, MKS1, MFN2, METTL14, MED15, MCM6, MBP, MATN1, MAT2B, MAPK14, MAPK1, MAP2K2, MANEA, MANBA, MAFB, MAD2L1, LRRC16A, LITAF, LIG3, KIF13A, KCNK3, KCNK1, KCNIP3, JAM3, ITPR1, ITIH1, IRX1, INHBC, IL7, IGFALS, HRH3, HOMER2, HMG2L1, HBE1, GUSB, GSTT2, GRM3, GRM2, GRIA2, GPSM2, GPR55, GNL3, GNAT2, GNAQ, GMIP, GABRR1, FZD2, FNTB, FH, FBXW7, FANCC, FAM84B, FAM69A, EYA1, ENTPD7, ELOVL6, DRG1, DOCK3, DOCK2, DNM1, DND1, DLG1, DGKI, DCK, CTXN3, CSNK2A1, CSDA, CRIPT, COQ3, COL5A2, CMT1A, CHRNG, CHRNA5, CHRM5, CHML, CHCHD6, CFLAR, CDYL, CD80, CD6, CANT1, CAMK2B, CAMK2A, CAMK1, CABLES2, C17orf42, C15orf53, C13orf15, BUD13, BHLHB3, BBS9, BAZ1B, AVPR2, ATP8A1, ASTN1, ARPC1A, ARNT2, ARID4B, APOL4, ALDH3A2, AKAP13, AIMP1, AHCY, AGXT, AGBL4, ADRBK2, ADRA1B, ADCY1, ADAMTS4, ACVR2A, ACVR1B, ACTN2, ZNF638, ZNF627, ZNF45, ZNF281, ZNF224, ZIC1, WWOX, WISP1, VSNL1, VANGL2, UIMC1, UGT2A1, UACA, TYRP1, TUBAL3, TTRAP, TTF2, TTBK1, TRPM2, TRAM2, TMX3, TMEM168, TMCC3, TMC1, TLK2, TIFA, TH1L, TFR2, TFIP11, TBCE, TBCA, TAZ, TAS2R16, TAC4, STK39, STK32B, STK17B, SSTR3, SRBD1, SPO11, SPINK4, SPATA17, SOX6, SOAT2, SMYD2, SLC9A9, SLC4A7, SLC45A2, SLC37A4, SLC25A14, SLC24A5, SKP2, SGPP1, SERPINE2, SEMA3E, RYK, RPL5, RPA2, RNF152, RNF146, RNF144A, RIPK1, RGS8, RGS17,

RGS13, RBL1, RBAK, RAP1A, RAD51L3, RAD50, PTPRR, PTPN13, PSMD2, PRSS12, PROK2, POLR2K, POLQ, PLEKHB1, PLD5, PLCD4, PKIA, PELO, PAX3, PAM, ORC5L, OLIG3, NXPH1, NTN1, NSUN4, NPS, NPR2, NKX2-5, NEK4, NBEA, MYOCD, MYO1F, MTSS1, MTHFD2, MPPED2, MMRN1, MMP13, MCM7, MAS1, MARK1, MARCO, MAPK3, MAP2K4, LSM4, LNX1, LHX8, LGALS3, LDLRAP1, LCA5, LASS4, LACE1, KIF13B, KIAA0564, JAKMIP2, JAK3, IRX3, IRF9, IRAK2, INTS12, IL2RG, IL17B, IFNAR2, HSD17B7, HOMER3, HLA-DMA, HELLS, HEL308, HDAC9, HDAC3, HBS1L, HBG2, HAPLN1, HADHA, GTF2IRD1, GTF2H3, GSTCD, GRHPR, GMPS, GLP2R, GGH, GDF9, GATA6, GART, GAR1, GADD45G, FTSJ1, FOXE1, FMO5, FMO4, FMNL2, FHOD3, FGFR4, FEZF1, FERMT1, FECH, FANCI, FANCG, FAM59A, FAM129A, FAM123B, FADS3, ESAM, EPB41L1, ENOSF1, EFCAB6, E2F7, DLG3, DICER1, DDX1, DCTD, DACH2, CYP2U1, CYP2A13, COPG, CNGA3, CLRN1, CHCHD3, CENPC1, CD74, CD3G, CD3D, CCNB2, CCDC98, CCDC109B, CASP10, CAPS, CAPN3, C7orf23, C4orf31, C3orf44, C20orf26, C1orf26, C11orf79, BTD, BDH2, ATP10D, ATG16L1, ATF7IP2, ARRDC3, ANKRD44, AMD1, ALDH1B1, AKAP9, AGL, AFF3, ADCYAP1R1, ADCY8, ADAMTS19, ADAM9, ABI3BP

Corresponding total number of TF hits:
(For each gene listed above, the total number of TF hits for any of the selected TFs, multiplied by the number of selected Phenopedia diseases containing that gene)
735, 676, 588, 585, 506, 504, 495, 495, 468, 434, 430, 429, 420, 420, 416, 390, 378, 376, 369, 360, 360, 352, 350, 342, 336, 328, 325, 324, 315, 310, 308, 308, 299, 297, 294, 294, 294, 288, 288, 288, 273, 270, 270, 266, 266, 266, 266, 264, 260, 256, 253, 252, 247, 242, 242, 240, 240, 238, 234, 230, 230, 225, 225, 224, 224, 224, 221, 221, 220, 217, 216, 216, 216, 210, 208, 205, 200, 198, 198, 196, 195, 195, 192, 190, 189, 189, 187, 182, 180, 180, 180, 176, 172, 171, 170, 170, 169, 169, 168, 168, 168, 168, 168, 165, 165, 162, 162, 161, 156, 156, 155, 154, 154, 154, 154, 154, 154, 154, 153, 150, 150, 150, 150, 144, 143, 143, 141, 140, 138, 136, 135, 135, 135, 135, 132, 130, 130, 130, 128, 126, 126, 126, 126, 126, 126, 124, 124, 124, 121, 120, 120, 120, 120, 119, 119, 117, 117, 117, 117, 117, 116, 115, 115, 112, 112, 112, 112, 112, 112, 112, 112, 112, 112, 110, 110, 110, 110, 108, 108, 108, 108, 108, 108, 108, 108, 105, 105, 105, 105, 104, 104, 104, 104, 102, 100, 100, 100, 100, 99, 99, 99, 99, 99, 99, 99, 99, 98, 96, 96, 95, 95, 93, 92, 91, 91, 90, 90, 90, 90, 90, 90, 90, 90, 90, 90, 90, 90, 90, 90, 90, 90, 88, 88, 88, 87, 86, 86, 85, 84, 84, 84, 84, 84, 84, 84, 84, 82, 81, 81, 81, 81, 81, 81, 81, 81, 81, 80, 80, 80, 80, 78, 78, 78, 78, 78, 77, 77, 77, 77, 77, 77, 75, 75, 75, 75, 75, 75, 75, 75, 75, 74, 72, 72, 72, 72, 72, 72, 72, 70, 70, 70, 70, 70, 70, 70, 70, 70, 70, 70, 70, 70, 70, 66, 66, 66, 66, 66, 65, 65, 65, 65, 65, 65, 64, 64, 63, 63, 63, 63, 63, 63, 63, 63, 63, 63, 63, 63, 63, 63, 60, 60, 60, 60, 60, 60, 60, 60, 60, 60, 60, 57, 56, 56, 56, 56, 56, 56, 56, 56, 56, 56, 55, 55, 55, 55, 55, 55, 55, 55, 55, 55, 54, 54, 54, 54, 54, 54, 54, 54, 54, 54, 54, 54, 52, 52, 52, 52, 52, 52, 52, 52, 52, 52, 52, 52, 52, 52, 51, 51, 50, 50, 50, 50, 50, 50, 49, 48, 48, 48, 48, 48, 48, 48, 48, 48, 48, 48, 48, 45, 45, 45, 45, 45, 45, 45, 45, 45, 45, 45, 45, 45, 45, 45, 45, 45, 45, 45, 45, 45, 45, 45, 45, 45, 45, 45, 45, 45, 45, 45, 45, 45, 45, 45, 45, 45, 45, 45, 45, 45, 45, 45, 44, 44, 44, 44, 44, 44, 44, 43, 42, 42, 42, 42, 42, 42, 42, 42, 42, 42, 42, 42, 42, 42, 42, 42, 42, 42, 42, 42, 42, 42, 42, 41, 40, 40, 40, 40, 40, 40, 40, 40, 40, 40, 40, 40, 40, 40, 40, 40, 40, 40, 40, 40, 40, 40, 39, 39, 39, 39, 39, 39, 39, 39, 39, 39, 39, 39, 39, 39, 39, 39, 39, 39, 39, 39, 39, 38, 38, 38, 37, 36, 36, 36, 36, 36, 36, 36, 36, 36, 36, 36, 36, 36, 36, 36, 36, 36, 36, 36, 36, 36, 36, 36, 36, 36, 36, 36, 36, 36, 36, 36, 36, 36, 36, 36, 35, 35, 35, 35, 35, 35, 35, 35, 35, 35, 35, 34, 34, 34, 33, 33, 33, 33, 33, 33, 33, 33, 33, 33, 33, 33, 32, 32, 32, 32, 32, 32, 32, 32, 32, 32, 32, 32, 32, 31, 31, 30, 30, 30, 30, 30, 30, 30, 30, 30, 30, 30, 30, 30, 30, 30, 30, 30, 30, 30, 30, 30, 30, 30, 30, 30, 30, 30, 30, 30, 30, 30, 30, 30, 30, 30, 30, 30, 30, 30, 30, 30, 30, 30, 30, 30, 29, 29, 29, 28, 28, 28, 28, 28, 28, 28, 28, 28, 28, 28, 28, 28, 28, 28, 28, 28, 28, 28, 28, 28, 28, 28, 28, 28, 28, 28, 28, 28, 28, 27, 27, 27, 27, 27, 27, 27, 27, 27, 27, 27, 27, 27, 27, 27, 27, 27, 27, 27, 27, 27, 27, 27, 27, 27, 27, 27, 27, 27, 27, 27, 27, 27, 27, 27, 27, 27, 27, 27, 27, 27, 27, 26, 26, 26, 26, 26, 26, 26, 26, 26, 26, 26, 26, 26, 26, 26, 26, 26, 26, 26, 26, 26, 25, 25, 25, 25, 25, 25, 25, 25, 25, 25, 25, 24, 24, 24, 24, 24, 24, 24, 24, 24, 24, 24, 24, 24, 24, 24, 24, 24, 24, 24, 24, 24, 24, 24, 24, 24, 24, 24, 23, 23, 23, 22, 22, 22, 22, 22, 22, 22, 22, 22, 22, 22, 22, 21, 21, 21, 21, 21, 21, 21, 21, 21, 21, 21, 21, 21, 21, 21, 21, 21, 21, 21, 21, 21, 21, 21, 21, 21, 21, 21, 21, 21, 21, 21, 21, 20, 20, 20, 20, 20, 20, 20, 20, 20, 20, 20, 20, 20, 20, 20, 20, 20, 20, 20, 20, 20, 20, 20, 20, 20, 20, 20, 20, 20, 20, 20, 20, 20, 20, 20, 20, 20, 20, 20, 20, 20, 20, 20, 20, 20, 20, 19, 19, 19, 19, 18, 18, 18, 18, 18, 18, 18, 18, 18, 18, 18, 18, 18, 18, 18, 18, 18, 18, 18, 18, 18, 18, 18, 18, 18, 18, 18, 18, 18, 18, 18, 18, 18, 18, 18, 18, 18, 18, 18, 18, 18, 18, 18, 18, 18, 18, 18, 18, 18, 18, 18, 18, 18, 18, 18, 18, 18, 18, 18, 18, 18, 18, 18, 18, 18, 18, 18, 18, 18, 18, 18, 18, 18, 18, 18, 18, 18, 18, 18, 18, 18, 18, 18, 18, 18, 18, 18, 18, 18, 17, 17, 17, 16, 16, 16, 16, 16, 16, 16, 16, 16, 16, 16, 16, 16, 16, 16, 16, 16, 16, 16, 16, 16, 16, 16, 16, 16, 16, 16, 16,

16, 16, 16, 16, 16, 16, 15, 15, 15, 15, 15, 15, 15, 15, 15, 15, 15, 15, 15, 15, 15, 15, 15, 15, 15, 15, 15, 15,
15, 15, 15, 15, 15, 15, 15, 15, 15, 15, 15, 15, 15, 15, 15, 15, 15, 15, 15, 15, 15, 15, 15, 15, 15, 15, 15, 15,
15, 15, 15, 15, 15, 15, 15, 15, 15, 15, 14, 14, 14, 14, 14, 14, 14, 14, 14, 14, 14, 14, 14, 14, 14, 14, 14, 14,
14, 14, 14, 14, 14, 14, 14, 14, 14, 14, 14, 14, 14, 14, 14, 14, 14, 14, 14, 14, 14, 14, 14, 14, 14, 14, 14, 14,
14, 14, 14, 13, 13, 13, 13, 13, 13, 13, 13, 13, 13, 13, 13, 13, 13, 13, 13, 13, 13, 13, 13, 13, 13, 13, 13, 13,
13, 13, 13, 12, 12, 12, 12, 12, 12, 12, 12, 12, 12, 12, 12, 12, 12, 12, 12, 12, 12, 12, 12, 12, 12, 12, 12, 12,
12, 12, 12, 12, 12, 12, 12, 12, 12, 12, 12, 12, 12, 12, 12, 12, 12, 12, 12, 12, 12, 12, 12, 12, 12, 12, 12, 12,
12, 12, 12, 12, 12, 12, 12, 12, 12, 12, 12, 12, 12, 12, 12, 12, 12, 12, 12, 12, 12, 12, 12, 12, 12, 12, 12, 12,
12, 12, 12, 12, 12, 12, 12, 12, 12, 12, 12, 12, 12, 12, 11, 11, 11, 11, 11, 11, 11, 11, 11, 11, 11, 11, 11, 11,
11, 11, 11, 11, 11, 11, 11, 11, 11, 11, 11, 11, 11, 11, 11, 11, 10, 10, 10, 10, 10, 10, 10, 10, 10, 10, 10, 10,
10, 10, 10, 10, 10, 10, 10, 10, 10, 10, 10, 10, 10, 10, 10, 10, 10, 10, 10, 10, 10, 10, 10, 10, 10, 10, 10, 10,
10, 10, 10, 10, 10, 10, 10, 10, 10, 10, 10, 10, 10, 10, 10, 10, 10, 10, 10, 10, 10, 10, 10, 10, 10, 10, 10, 10,
10, 10, 10, 10, 10, 10, 10, 10, 10, 10, 10, 10, 10, 10, 10, 10, 10, 10, 10, 10, 10, 10, 10, 10, 10, 10, 10, 10,
10, 10, 10, 10, 10, 10, 10, 10, 9, 9, 9, 9, 9, 9, 9, 9, 9, 9, 9, 9, 9, 9, 9, 9, 9, 9, 9, 9, 9, 9, 9, 9, 9, 9, 9, 9, 9, 9,
9, 9, 9, 9, 9, 9, 9, 9, 9, 9, 9, 9, 9, 9, 9, 9, 9, 9, 9, 9, 9, 9, 9, 9, 9, 9, 9, 9, 9, 9, 9, 9, 9, 9, 9, 9, 9, 9, 9, 9, 9, 9,
9, 9, 9, 9, 9, 9, 9, 9, 9, 9, 9, 9, 9, 9, 9, 9, 9, 9, 9, 9, 9, 9, 9, 9, 9, 9, 9, 9, 9, 9, 9, 9, 9, 9, 9, 9, 9, 9, 9, 9, 9, 9,
8, 8, 8, 8, 8, 8, 8, 8, 8, 8, 8, 8, 8, 8, 8, 8, 8, 8, 8, 8, 8, 8, 8, 8, 8, 8, 8, 8, 8, 8, 8, 8, 8, 8, 8, 8, 8, 8, 8, 8, 8, 8,
8, 8, 8, 8, 8, 8, 8, 8, 8, 8, 8, 8, 8, 8, 8, 8, 8, 8, 8, 8, 8, 8, 8, 8, 8, 8, 8, 8, 8, 8, 8, 8, 8, 8, 8, 8, 8, 8, 8, 8, 7, 7,
7, 7, 7, 7, 7, 7, 7, 7, 7, 7, 7, 7, 7, 7, 7, 7, 7, 7, 7, 7, 7, 7, 7, 7, 7, 7, 7, 7, 7, 7, 7, 7, 7, 7, 7, 7, 7, 7, 7, 7, 7, 6,
6, 6, 6, 6, 6, 6, 6, 6, 6, 6, 6, 6, 6, 6, 6, 6, 6, 6, 6, 6, 6, 6, 6, 6, 6, 6, 6, 6, 6, 6, 6, 6, 6, 6, 6, 6, 6, 6, 6, 6, 6, 6,
6, 6, 6, 6, 6, 6, 6, 6, 6, 6, 6, 6, 6, 6, 6, 6, 6, 6, 6, 6, 6, 6, 6, 6, 6, 6, 6, 6, 6, 6, 6, 6, 6, 6, 6, 6, 6, 6, 6, 6, 6, 6,
6, 6, 6, 6, 6, 6, 6, 6, 6, 6, 6, 6, 6, 6, 6, 6, 6, 6, 6, 6, 6, 6, 6, 6, 6, 6, 6, 6, 6, 6, 6, 6, 6, 6, 6, 6, 6, 6, 6, 6, 6, 6,
6, 6, 6, 6, 6, 6, 6, 6, 6, 6, 6, 6, 6, 6, 6, 6, 6, 6, 6, 6, 6, 6, 6, 6, 6, 6, 6, 6, 6, 6, 6, 6, 6, 6, 6, 5, 5, 5, 5, 5, 5, 5,
5, 5, 5, 5, 5, 5, 5, 5, 5, 5, 5, 5, 5, 5, 5, 5, 5, 5, 5, 5, 5, 5, 5, 5, 5, 5, 5, 5, 5, 5, 5, 5, 5, 5, 5, 5, 5, 5, 5, 5, 5, 5,
5, 5, 5, 5, 5, 5, 5, 5, 5, 5, 5, 5, 5, 5, 5, 5, 5, 5, 5, 5, 5, 5, 5, 5, 5, 5, 5, 5, 5, 5, 5, 5, 5, 5, 5, 5, 5, 5, 5, 5, 5, 5,
5, 5, 5, 5, 5, 5, 5, 5, 5, 5, 5, 5, 5, 5, 5, 5, 5, 5, 5, 5, 5, 5, 5, 5, 5, 5, 4, 4, 4, 4, 4, 4, 4, 4, 4, 4, 4, 4, 4, 4, 4, 4,
4, 4, 4, 4, 4, 4, 4, 4, 4, 4, 4, 4, 4, 4, 4, 4, 4, 4, 4, 4, 4, 4, 4, 4, 4, 4, 4, 4, 4, 4, 4, 4, 4, 4, 4, 4, 4, 4, 4, 4, 4, 4,
4, 4, 4, 4, 4, 4, 4, 4, 4, 4, 4, 4, 4, 4, 4, 4, 4, 4, 4, 4, 4, 4, 4, 4, 4, 4, 4, 4, 4, 4, 4, 4, 4, 4, 4, 4, 4, 4, 4, 4, 4, 4,
4, 4, 4, 4, 4, 4, 4, 4, 4, 4, 4, 4, 4, 4, 4, 4, 4, 4, 4, 4, 4, 4, 4, 4, 4, 4, 4, 4, 4, 4, 4, 4, 4, 4, 4, 4, 4, 4, 4, 4, 4, 4,
4, 4, 4, 4, 4, 4, 4, 4, 4, 4, 4, 4, 4, 4, 3, 3, 3, 3, 3, 3, 3, 3, 3, 3, 3, 3, 3, 3, 3, 3, 3, 3, 3, 3, 3, 3, 3, 3, 3, 3, 3, 3,
3, 3, 3, 3, 3, 3, 3, 3, 3, 3, 3, 3, 3, 3, 3, 3, 3, 3, 3, 3, 3, 3, 3, 3, 3, 3, 3, 3, 3, 3, 3, 3, 3, 3, 3, 3, 3, 3, 3, 3, 3, 3,
3, 3, 3, 3, 3, 3, 3, 3, 3, 3, 3, 3, 3, 3, 3, 3, 3, 3, 3, 3, 3, 3, 3, 3, 3, 3, 3, 3, 3, 3, 3, 3, 3, 3, 3, 3, 3, 3, 3, 3, 3, 3,
3, 3, 3, 3, 3, 3, 3, 3, 3, 3, 3, 3, 3, 3, 3, 3, 3, 3, 3, 3, 3, 3, 3, 3, 3, 3, 3, 3, 3, 3, 3, 3, 3, 3, 3, 3, 3, 3, 3, 3, 3, 3,
3, 3, 3, 3, 3, 3, 3, 3, 3, 3, 3, 3, 3, 3, 3, 3, 3, 3, 3, 3, 2, 2, 2, 2, 2, 2, 2, 2, 2, 2, 2, 2, 2, 2, 2, 2, 2, 2, 2, 2, 2, 2,
2, 2, 2, 2, 2, 2, 2, 2, 2, 2, 2, 2, 2, 2, 2, 2, 2, 2, 2, 2, 2, 2, 2, 2, 2, 2, 2, 2, 2, 2, 2, 2, 2, 2, 2, 2, 2, 2, 2, 2, 2, 2,
2, 2, 2, 2, 2, 2, 2, 2, 2, 2, 2, 2, 2, 2, 2, 2, 2, 2, 2, 2, 2, 2, 2, 2, 2, 2, 2, 2, 2, 2, 2, 2, 2, 2, 2, 2, 2, 2, 2, 2, 2, 2,
2, 2, 2, 2, 2, 2, 2, 2, 2, 2, 2, 2, 2, 2, 2, 2, 2, 2, 2, 2, 2, 2, 2, 2, 2, 2, 2, 2, 2, 2, 2, 2, 2, 2, 2, 2, 2, 2, 2, 2, 2, 2,
2, 2, 2, 2, 2, 2, 2, 2, 2, 2, 2, 2, 2, 2, 2, 2, 2, 2, 2, 2, 2, 2, 2, 2, 2, 2, 2, 2, 2, 2, 2, 2, 2, 2, 2, 2, 2, 2, 2, 2, 2, 2,
2, 2, 2, 2, 2, 2, 2, 2, 2, 2, 2, 2, 2, 2, 2, 2, 2, 2, 2, 2, 2, 2, 2, 2, 1, 1, 1, 1, 1, 1, 1, 1, 1, 1, 1, 1, 1, 1, 1, 1, 1, 1,
1, 1, 1, 1, 1, 1, 1, 1, 1, 1, 1, 1, 1, 1, 1, 1, 1, 1, 1, 1, 1, 1, 1, 1, 1, 1, 1, 1, 1, 1, 1, 1, 1, 1, 1, 1, 1, 1, 1, 1, 1, 1,
1, 1, 1, 1, 1, 1, 1, 1, 1, 1, 1, 1, 1, 1, 1, 1, 1, 1, 1, 1, 1, 1, 1, 1, 1, 1, 1, 1, 1, 1, 1, 1, 1, 1, 1, 1, 1, 1, 1, 1, 1, 1,
1, 1, 1, 1, 1, 1, 1, 1, 1, 1, 1, 1, 1, 1, 1, 1, 1, 1, 1, 1, 1, 1, 1, 1, 1, 1, 1, 1, 1, 1, 1, 1, 1, 1, 1, 1, 1, 1, 1, 1, 1, 1,
1, 1, 1, 1, 1, 1, 1, 1, 1, 1, 1, 1, 1, 1, 1, 1, 1, 1, 1, 1, 1, 1, 1, 1, 1, 1, 1, 1, 1, 1, 1, 1, 1, 1, 1, 1, 1, 1, 1, 1, 1, 1,
1, 1, 1, 1, 1, 1, 1, 1, 1, 1, 1, 1, 1, 1, 1, 1, 1, 1, 1, 1, 1, 1, 1, 1, 1, 1, 1, 1

Corresponding number of selected Phenopedia diseases each gene is involved with:

(In the same order as above)

15, 13, 14, 13, 11, 9, 15, 11, 13, 14, 10, 13, 14, 14, 13, 10, 7, 8, 9, 15, 9, 11, 10, 9, 14, 8, 13, 9, 15, 10, 14,
7, 13, 9, 14, 14, 14, 9, 8, 8, 13, 9, 10, 14, 14, 14, 14, 11, 13, 8, 11, 14, 13, 11, 11, 12, 15, 14, 9, 10, 10, 15,
9, 14, 14, 14, 13, 13, 5, 7, 9, 8, 6, 15, 13, 5, 8, 9, 9, 14, 5, 13, 6, 5, 9, 9, 11, 14, 15, 15, 9, 11, 4, 9, 10, 10,
13, 13, 14, 14, 3, 14, 8, 11, 5, 9, 9, 7, 3, 13, 5, 14, 14, 11, 14, 14, 7, 14, 9, 5, 5, 5, 10, 4, 13, 11, 3, 14, 6, 8,
9, 15, 9, 3, 11, 10, 5, 5, 8, 14, 14, 14, 9, 9, 14, 4, 4, 4, 11, 6, 5, 5, 3, 7, 7, 9, 9, 9, 9, 9, 4, 5, 5, 4, 14, 14, 14,
14, 4, 8, 14, 14, 14, 11, 11, 10, 5, 3, 9, 9, 3, 3, 9, 12, 9, 15, 15, 5, 5, 13, 8, 2, 13, 3, 10, 10, 4, 5, 9, 11, 11,
9, 11, 11, 11, 9, 14, 3, 4, 5, 5, 3, 4, 13, 7, 9, 5, 6, 2, 10, 9, 9, 5, 9, 15, 15, 5, 3, 10, 9, 9, 15, 11, 11, 4, 3, 2,
2, 5, 14, 14, 14, 14, 6, 2, 4, 14, 2, 9, 9, 3, 3, 9, 3, 9, 9, 9, 10, 5, 2, 5, 13, 13, 13, 3, 13, 11, 7, 11, 11, 11, 11,
5, 15, 5, 15, 15, 15, 3, 15, 15, 2, 9, 3, 9, 3, 8, 9, 9, 14, 10, 10, 7, 7, 14, 14, 7, 2, 7, 5, 5, 10, 14, 11, 11, 6, 2,

11, 5, 5, 13, 13, 5, 5, 8, 8, 9, 3, 9, 9, 9, 9, 9, 9, 7, 7, 9, 3, 9, 9, 15, 6, 5, 4, 3, 5, 10, 10, 15, 10, 5, 6, 3, 14,
14, 7, 14, 14, 7, 14, 14, 8, 14, 14, 11, 11, 11, 5, 11, 11, 5, 11, 11, 5, 3, 3, 6, 9, 3, 2, 6, 9, 9, 2, 6, 9, 3, 13, 13,
13, 13, 13, 13, 13, 4, 13, 4, 13, 13, 13, 2, 1, 3, 5, 5, 10, 10, 5, 2, 7, 3, 4, 8, 6, 3, 3, 3, 8, 2, 3, 4, 6, 6, 9, 15,
3, 9, 9, 3, 5, 9, 9, 9, 15, 9, 3, 9, 3, 9, 15, 1, 5, 5, 15, 5, 5, 15, 15, 9, 9, 5, 9, 15, 15, 15, 9, 9, 3, 5, 9, 15, 9, 9,
9, 11, 4, 4, 4, 11, 4, 4, 1, 3, 14, 2, 14, 14, 14, 14, 14, 14, 6, 2, 14, 14, 14, 7, 3, 14, 3, 14, 1, 2, 5, 10, 8, 4, 5,
2, 5, 5, 5, 10, 2, 2, 10, 4, 10, 10, 2, 10, 10, 10, 13, 1, 13, 13, 3, 13, 13, 1, 13, 13, 3, 13, 13, 3, 1, 1, 3, 13,
13, 13, 13, 2, 2, 2, 1, 9, 2, 3, 9, 9, 9, 9, 9, 3, 9, 9, 1, 2, 3, 9, 12, 1, 9, 6, 3, 12, 6, 2, 9, 9, 9, 9, 12, 3, 2, 9, 2,
12, 7, 5, 5, 1, 5, 5, 5, 5, 5, 5, 5, 2, 2, 2, 1, 11, 1, 11, 11, 11, 3, 11, 11, 3, 3, 1, 4, 8, 4, 8, 4, 4, 4, 8, 8, 1, 4, 8,
4, 8, 1, 1, 15, 15, 15, 6, 10, 10, 10, 5, 6, 15, 10, 5, 6, 15, 15, 3, 10, 2, 5, 10, 5, 3, 15, 15, 15, 10, 1, 2, 2, 6,
1, 10, 3, 15, 2, 10, 10, 15, 3, 15, 6, 5, 5, 15, 2, 5, 1, 1, 1, 14, 14, 2, 1, 14, 14, 14, 14, 14, 14, 14, 14, 4, 14,
14, 7, 14, 7, 2, 4, 7, 14, 4, 1, 2, 1, 7, 14, 14, 14, 14, 9, 9, 9, 1, 9, 9, 9, 9, 3, 9, 9, 9, 9, 3, 9, 9, 9, 9, 9, 9, 9, 9,
9, 9, 9, 9, 9, 9, 9, 9, 9, 9, 9, 9, 9, 9, 9, 9, 9, 3, 2, 13, 13, 13, 2, 13, 13, 2, 13, 13, 1, 13, 1, 2, 13, 13, 13, 13,
13, 13, 1, 5, 5, 5, 1, 5, 5, 5, 5, 5, 5, 5, 1, 6, 3, 12, 4, 8, 3, 6, 2, 4, 3, 2, 6, 8, 3, 2, 3, 4, 2, 4, 3, 8, 8, 4, 12, 3,
1, 1, 1, 1, 2, 11, 2, 11, 11, 11, 11, 11, 1, 11, 2, 3, 7, 7, 3, 3, 7, 7, 3, 3, 7, 3, 7, 7, 3, 3, 7, 3, 1, 3, 7, 7, 7, 3, 7,
1, 1, 7, 3, 7, 4, 4, 4, 5, 4, 2, 2, 5, 10, 5, 5, 10, 5, 10, 10, 5, 5, 4, 10, 5, 4, 5, 5, 5, 5, 2, 10, 5, 10, 2, 10, 5, 5,
5, 10, 5, 10, 10, 10, 5, 10, 5, 10, 5, 2, 5, 2, 10, 5, 1, 1, 1, 1, 9, 3, 6, 9, 3, 9, 9, 9, 3, 9, 9, 1, 3, 9, 9, 2, 9, 1, 9,
9, 9, 9, 9, 6, 2, 3, 9, 9, 9, 9, 9, 9, 9, 3, 9, 3, 9, 9, 9, 9, 9, 2, 2, 3, 9, 1, 9, 1, 9, 9, 9, 6, 9, 3, 3, 9, 1, 9, 2, 9, 2,
2, 9, 9, 9, 9, 3, 9, 9, 9, 9, 9, 9, 9, 9, 9, 3, 9, 9, 9, 9, 6, 6, 9, 9, 6, 2, 9, 9, 1, 1, 1, 2, 2, 4, 1, 8, 4, 4, 1, 4, 1, 1,
2, 2, 1, 4, 1, 4, 1, 4, 8, 1, 8, 2, 4, 4, 8, 1, 8, 1, 8, 4, 1, 1, 4, 5, 15, 15, 5, 3, 5, 3, 5, 1, 15, 5, 15, 1, 15, 5, 5,
15, 5, 1, 1, 15, 3, 15, 5, 5, 5, 1, 15, 3, 5, 15, 5, 15, 15, 15, 3, 5, 15, 15, 5, 3, 5, 5, 5, 15, 15, 5, 5, 3, 3, 5, 15,
5, 5, 5, 15, 15, 15, 5, 15, 1, 14, 2, 14, 14, 7, 1, 7, 7, 2, 14, 14, 2, 7, 7, 14, 2, 7, 2, 1, 7, 1, 2, 7, 14, 2, 14, 14,
14, 1, 2, 2, 2, 2, 2, 7, 14, 14, 1, 1, 14, 2, 14, 2, 14, 7, 14, 14, 14, 13, 13, 13, 13, 13, 13, 1, 13, 13, 1, 13, 13,
13, 13, 13, 13, 13, 1, 13, 13, 1, 13, 13, 13, 13, 13, 13, 13, 3, 2, 4, 2, 4, 4, 3, 2, 6, 3, 4, 1, 12, 3, 6, 2, 3, 6, 4,
4, 6, 2, 6, 4, 2, 6, 2, 3, 2, 4, 4, 2, 6, 3, 3, 2, 4, 3, 4, 3, 4, 2, 2, 6, 6, 6, 3, 4, 4, 3, 6, 4, 6, 2, 3, 2, 3, 3, 4, 6, 1,
2, 2, 4, 1, 12, 12, 1, 12, 3, 3, 6, 3, 4, 12, 6, 3, 1, 6, 6, 2, 4, 1, 12, 2, 4, 4, 3, 4, 3, 2, 6, 3, 3, 3, 1, 1, 11, 11, 1,
11, 1, 11, 1, 11, 11, 11, 1, 1, 11, 1, 11, 11, 11, 1, 11, 11, 11, 11, 1, 11, 11, 1, 11, 11, 1, 5, 5, 5, 2, 5, 5, 10, 5,
5, 1, 5, 1, 1, 5, 2, 1, 5, 5, 5, 10, 2, 5, 1, 5, 1, 1, 10, 2, 5, 10, 2, 2, 10, 10, 10, 10, 5, 10, 10, 10, 10, 10, 10, 5,
10, 2, 5, 5, 10, 5, 5, 10, 10, 5, 10, 5, 5, 5, 1, 10, 2, 5, 5, 5, 10, 1, 5, 5, 2, 1, 10, 5, 5, 5, 1, 5, 5, 10, 5, 10, 2,
5, 10, 10, 10, 5, 5, 2, 1, 10, 10, 10, 5, 2, 5, 2, 5, 2, 5, 10, 5, 10, 10, 9, 9, 9, 9, 9, 3, 9, 9, 3, 3, 9, 1, 9, 3, 9, 9,
9, 9, 1, 1, 9, 3, 3, 9, 3, 9, 9, 9, 9, 3, 9, 9, 3, 9, 9, 9, 3, 9, 9, 1, 9, 9, 3, 9, 9, 9, 9, 9, 9, 9, 3, 9, 1, 3, 9, 1, 9, 1,
9, 9, 3, 9, 9, 9, 3, 9, 9, 9, 9, 9, 9, 9, 9, 9, 9, 9, 9, 9, 3, 9, 9, 9, 9, 3, 1, 3, 9, 3, 3, 9, 3, 3, 9, 9, 3, 3, 3, 9, 9, 9,
9, 9, 1, 3, 3, 3, 9, 9, 9, 9, 9, 3, 3, 9, 4, 4, 4, 1, 2, 2, 4, 4, 4, 1, 4, 8, 4, 4, 4, 1, 2, 2, 4, 2, 4, 1, 2, 2, 8, 4, 2, 4,
2, 4, 8, 4, 2, 2, 2, 1, 4, 4, 2, 2, 2, 1, 4, 1, 4, 8, 4, 2, 2, 8, 4, 2, 4, 4, 4, 4, 8, 2, 4, 8, 4, 4, 2, 4, 4, 8, 1, 4, 1, 2,
2, 2, 4, 8, 2, 2, 1, 2, 2, 1, 8, 4, 1, 7, 7, 1, 7, 1, 1, 1, 7, 7, 1, 7, 1, 7, 1, 7, 7, 7, 1, 1, 7, 7, 1, 1, 1, 1, 7, 7, 7, 1,
7, 1, 7, 7, 1, 1, 7, 1, 1, 7, 1, 7, 7, 2, 3, 1, 6, 2, 3, 3, 3, 2, 2, 6, 1, 1, 1, 3, 3, 6, 1, 1, 3, 3, 2, 2, 3, 2, 2, 3, 2, 3,
2, 6, 3, 6, 3, 1, 6, 2, 6, 6, 3, 3, 6, 6, 3, 1, 1, 3, 2, 1, 3, 2, 6, 3, 3, 2, 2, 6, 3, 6, 2, 2, 2, 3, 3, 3, 1, 3, 2, 3, 3, 2,
1, 3, 6, 6, 2, 3, 1, 2, 2, 1, 6, 3, 3, 2, 2, 2, 1, 2, 3, 6, 3, 1, 1, 2, 3, 3, 6, 3, 6, 6, 1, 6, 3, 6, 6, 6, 1, 2, 3, 2, 2, 3,
2, 2, 3, 2, 6, 3, 3, 2, 3, 2, 3, 3, 1, 3, 2, 3, 3, 1, 3, 6, 2, 2, 3, 1, 2, 2, 2, 2, 6, 3, 1, 6, 6, 3, 3, 6, 6, 2, 1, 6, 6, 2,
1, 2, 2, 3, 2, 3, 3, 5, 5, 1, 1, 1, 1, 1, 5, 5, 5, 5, 5, 1, 5, 5, 1, 5, 5, 5, 1, 5, 1, 5, 5, 5, 5, 5, 1, 5, 5, 1, 5, 1, 5, 5,
5, 5, 5, 5, 1, 5, 5, 5, 1, 5, 5, 5, 5, 5, 5, 5, 5, 5, 5, 1, 5, 5, 5, 5, 5, 5, 5, 1, 1, 5, 1, 5, 1, 5, 1, 5, 5, 5, 5, 5, 5, 5,
5, 5, 5, 5, 5, 5, 5, 1, 5, 5, 5, 5, 5, 5, 1, 5, 1, 5, 5, 5, 5, 5, 5, 1, 1, 5, 5, 5, 5, 5, 5, 5, 5, 5, 5, 5, 1, 5, 1, 5, 2, 1,
4, 4, 2, 1, 4, 4, 1, 2, 2, 1, 1, 2, 4, 2, 1, 1, 4, 4, 2, 2, 1, 1, 2, 2, 2, 2, 1, 4, 1, 4, 2, 4, 1, 2, 2, 1, 1, 2, 4, 1, 1, 4,
1, 1, 2, 4, 1, 4, 2, 2, 4, 4, 1, 4, 2, 4, 4, 2, 1, 2, 4, 1, 1, 4, 4, 2, 4, 1, 4, 1, 2, 4, 2, 1, 1, 4, 2, 2, 2, 1, 1, 4, 4, 4,
2, 1, 2, 2, 1, 4, 4, 2, 2, 2, 2, 4, 4, 2, 2, 2, 1, 4, 4, 2, 2, 1, 2, 2, 2, 1, 4, 2, 4, 4, 1, 2, 4, 1, 1, 4, 2, 1, 2, 1, 2, 2,
2, 1, 1, 1, 2, 2, 4, 4, 2, 1, 1, 2, 1, 4, 4, 4, 2, 2, 4, 2, 2, 4, 1, 2, 2, 4, 1, 2, 3, 3, 3, 3, 1, 1, 1, 1, 3, 3, 1, 3, 3, 1,
3, 1, 1, 3, 3, 1, 1, 1, 1, 3, 3, 1, 3, 3, 1, 1, 1, 3, 3, 1, 3, 1, 1, 3, 3, 3, 1, 1, 1, 1, 1, 1, 3, 3, 3, 3, 1, 1, 1, 1, 1,
1, 1, 3, 3, 3, 1, 3, 1, 3, 3, 1, 1, 1, 1, 1, 3, 1, 1, 3, 1, 3, 1, 3, 1, 3, 1, 1, 1, 1, 1, 3, 1, 3, 3, 1, 1, 1, 3, 3, 3, 3, 1,
3, 1, 1, 1, 1, 3, 3, 1, 3, 1, 3, 1, 3, 1, 1, 1, 3, 1, 3, 1, 3, 1, 3, 3, 3, 3, 3, 1, 3, 3, 1, 3, 1, 3, 3, 1, 3, 3, 3, 3, 3, 3,
3, 1, 1, 3, 3, 3, 1, 3, 1, 3, 1, 3, 1, 3, 3, 3, 3, 3, 1, 3, 3, 1, 3, 1, 1, 3, 3, 3, 1, 3, 3, 3, 3, 3, 2, 2, 2, 1, 2, 1, 2, 1,
1, 1, 1, 1, 2, 1, 1, 1, 1, 2, 2, 2, 1, 1, 2, 1, 2, 1, 1, 2, 2, 2, 2, 2, 2, 2, 2, 1, 1, 2, 2, 1, 2, 2, 2, 1, 2, 1, 2, 2, 1, 1,
1, 2, 1, 1, 1, 2, 2, 1, 2, 1, 2, 1, 1, 1, 2, 2, 1, 2, 2, 2, 2, 1, 2, 1, 2, 2, 2, 1, 1, 1, 1, 1, 1, 2, 2, 2, 2, 2, 2, 1, 2, 2,
2, 2, 1, 1, 1, 2, 2, 2, 2, 2, 2, 2, 2, 1, 2, 1, 1, 1, 1, 2, 2, 2, 2, 1, 1, 1, 1, 2, 1, 1, 2, 2, 1, 2, 1, 2, 2, 1, 2, 1, 2, 2,
2, 2, 1, 2, 2, 2, 2, 1, 1, 2, 1, 1, 2, 2, 2, 2, 2, 1, 2, 1, 1, 1, 1, 1, 2, 2, 1, 2, 1, 2, 2, 1, 2, 2, 1, 1, 1, 2, 2, 1, 2, 2,
1, 1, 1, 1, 2, 1, 2, 2, 1, 2, 1, 2, 2, 1, 1, 2, 1, 2, 2, 2, 2, 1, 2, 1, 2, 2, 2, 1, 2, 1, 1, 2, 2, 2, 2, 1, 1, 2, 1, 1, 1, 1,
1, 1, 1, 1, 1, 1, 1, 1, 1, 1, 1, 1, 1, 1, 1, 1, 1, 1, 1, 1, 1, 1, 1, 1, 1, 1, 1, 1, 1, 1, 1, 1, 1, 1, 1, 1, 1, 1, 1, 1, 1, 1,
1, 1, 1, 1, 1, 1, 1, 1, 1, 1, 1, 1, 1, 1, 1, 1, 1, 1, 1, 1, 1, 1, 1, 1, 1, 1, 1, 1, 1, 1, 1, 1, 1, 1, 1, 1, 1, 1, 1, 1, 1, 1,

1, 1, 1, 1, 1, 1, 1, 1, 1, 1, 1, 1, 1, 1, 1, 1, 1, 1, 1, 1, 1, 1, 1, 1, 1, 1, 1, 1, 1, 1, 1, 1, 1, 1, 1, 1, 1, 1, 1, 1, 1, 1, 1, 1,
1, 1, 1, 1, 1, 1, 1, 1, 1, 1, 1, 1, 1, 1, 1, 1, 1, 1, 1, 1, 1, 1, 1, 1, 1, 1, 1, 1, 1, 1, 1, 1, 1, 1, 1, 1, 1, 1, 1, 1, 1, 1, 1, 1,
1, 1, 1, 1, 1, 1, 1, 1, 1, 1, 1, 1, 1, 1, 1, 1, 1, 1, 1, 1, 1, 1, 1, 1, 1, 1, 1, 1, 1, 1, 1, 1, 1, 1, 1, 1, 1, 1, 1, 1, 1, 1, 1, 1

Corresponding number of selected TFs each gene is involved with:
(In the same order as above)
49, 52, 42, 45, 46, 56, 33, 45, 36, 31, 43, 33, 30, 30, 32, 39, 54, 47, 41, 24, 40, 32, 35, 38, 24, 41, 25, 36, 21, 31, 22, 44, 23, 33, 21, 21, 21, 32, 36, 36, 21, 30, 27, 19, 19, 19, 19, 24, 20, 32, 23, 18, 19, 22, 22, 20, 16, 17, 26, 23, 23, 15, 25, 16, 16, 16, 17, 17, 44, 31, 24, 27, 36, 14, 16, 41, 25, 22, 22, 14, 39, 15, 32, 38, 21, 21, 17, 13, 12, 12, 20, 16, 43, 19, 17, 17, 13, 13, 12, 12, 56, 12, 21, 15, 33, 18, 18, 23, 52, 12, 31, 11, 11, 14, 11, 11, 22, 11, 17, 30, 30, 30, 15, 36, 11, 13, 47, 10, 23, 17, 15, 9, 15, 45, 12, 13, 26, 26, 16, 9, 9, 9, 14, 14, 9, 31, 31, 31, 11, 20, 24, 24, 40, 17, 17, 13, 13, 13, 13, 13, 29, 23, 23, 28, 8, 8, 8, 8, 28, 14, 8, 8, 8, 10, 10, 11, 22, 36, 12, 12, 36, 36, 12, 9, 12, 7, 7, 21, 21, 8, 13, 52, 8, 34, 10, 10, 25, 20, 11, 9, 9, 11, 9, 9, 9, 11, 7, 32, 24, 19, 19, 31, 23, 7, 13, 10, 18, 15, 45, 9, 10, 10, 18, 10, 6, 6, 18, 30, 9, 10, 10, 6, 8, 8, 22, 29, 43, 43, 17, 6, 6, 6, 6, 14, 42, 21, 6, 41, 9, 9, 27, 27, 9, 27, 9, 9, 9, 8, 16, 40, 16, 6, 6, 6, 26, 6, 7, 11, 7, 7, 7, 7, 15, 5, 15, 5, 5, 5, 25, 5, 5, 37, 8, 24, 8, 24, 9, 8, 8, 5, 7, 7, 10, 10, 5, 5, 10, 35, 10, 14, 14, 7, 5, 6, 6, 11, 33, 6, 13, 13, 5, 5, 13, 13, 8, 8, 7, 21, 7, 7, 7, 7, 7, 7, 9, 9, 7, 21, 7, 7, 4, 10, 12, 15, 20, 12, 6, 6, 4, 6, 12, 10, 19, 4, 4, 8, 4, 4, 8, 4, 4, 7, 4, 4, 5, 5, 5, 11, 5, 5, 11, 5, 5, 11, 18, 18, 9, 6, 18, 27, 9, 6, 6, 27, 9, 6, 18, 4, 4, 4, 4, 4, 4, 4, 13, 4, 13, 4, 4, 4, 26, 51, 17, 10, 10, 5, 5, 10, 25, 7, 16, 12, 6, 8, 16, 16, 16, 6, 24, 16, 12, 8, 8, 5, 3, 15, 5, 5, 15, 9, 5, 5, 5, 3, 5, 15, 5, 15, 5, 3, 45, 9, 9, 3, 9, 9, 3, 3, 5, 5, 9, 5, 3, 3, 3, 5, 5, 15, 9, 5, 3, 5, 5, 5, 4, 11, 11, 11, 4, 11, 11, 43, 14, 3, 21, 3, 3, 3, 3, 3, 3, 7, 21, 3, 3, 3, 6, 14, 3, 14, 3, 41, 20, 8, 4, 5, 10, 8, 20, 8, 8, 8, 4, 20, 20, 4, 10, 4, 4, 20, 4, 4, 4, 3, 39, 3, 3, 13, 3, 3, 39, 3, 3, 13, 3, 3, 13, 39, 39, 13, 3, 3, 3, 3, 19, 19, 19, 37, 4, 18, 12, 4, 4, 4, 4, 4, 12, 4, 4, 36, 18, 12, 4, 3, 36, 4, 6, 12, 3, 6, 18, 4, 4, 4, 4, 3, 12, 18, 4, 18, 3, 5, 7, 7, 35, 7, 7, 7, 7, 7, 7, 7, 17, 17, 17, 33, 3, 33, 3, 3, 3, 11, 3, 3, 11, 11, 33, 8, 4, 8, 4, 8, 8, 8, 4, 4, 32, 8, 4, 8, 4, 31, 31, 2, 2, 2, 5, 3, 3, 3, 6, 5, 2, 3, 6, 5, 2, 2, 10, 3, 15, 6, 3, 6, 10, 2, 2, 2, 3, 30, 15, 15, 5, 30, 3, 10, 2, 15, 3, 3, 2, 10, 2, 5, 6, 6, 2, 15, 6, 29, 29, 29, 2, 2, 14, 28, 2, 2, 2, 2, 2, 2, 2, 2, 7, 2, 2, 4, 2, 4, 14, 7, 4, 2, 7, 28, 14, 28, 4, 2, 2, 2, 2, 3, 3, 3, 27, 3, 3, 3, 3, 9, 3, 3, 3, 3, 9, 3, 3, 3, 3, 3, 3, 3, 3, 3, 3, 3, 3, 3, 3, 3, 3, 3, 3, 3, 3, 3, 3, 3, 3, 9, 13, 2, 2, 2, 13, 2, 2, 13, 2, 2, 26, 2, 26, 13, 2, 2, 2, 2, 2, 2, 26, 5, 5, 5, 25, 5, 5, 5, 5, 5, 5, 5, 24, 4, 8, 2, 6, 3, 8, 4, 12, 6, 8, 12, 4, 3, 8, 12, 8, 6, 12, 6, 8, 3, 3, 6, 2, 8, 23, 23, 23, 22, 11, 2, 11, 2, 2, 2, 2, 2, 22, 2, 11, 7, 3, 3, 7, 7, 3, 3, 7, 7, 3, 7, 3, 3, 7, 7, 3, 7, 21, 7, 3, 3, 3, 7, 3, 21, 21, 3, 7, 3, 5, 5, 5, 4, 5, 10, 10, 4, 2, 4, 4, 2, 4, 2, 2, 4, 4, 5, 2, 4, 5, 4, 4, 4, 4, 10, 2, 4, 2, 10, 2, 4, 4, 4, 2, 4, 2, 2, 2, 4, 2, 4, 2, 4, 10, 4, 10, 2, 4, 19, 19, 19, 19, 2, 6, 3, 2, 6, 2, 2, 2, 6, 2, 2, 18, 6, 2, 2, 9, 2, 18, 2, 2, 2, 2, 2, 3, 9, 6, 2, 2, 2, 2, 2, 2, 2, 6, 2, 6, 2, 2, 2, 2, 2, 9, 9, 6, 2, 18, 2, 18, 2, 2, 2, 3, 2, 6, 6, 2, 18, 2, 9, 2, 9, 9, 2, 2, 2, 2, 6, 2, 2, 2, 2, 2, 2, 2, 2, 2, 6, 2, 2, 2, 2, 3, 3, 2, 2, 3, 9, 2, 2, 17, 17, 17, 8, 8, 4, 16, 2, 4, 4, 16, 4, 16, 16, 8, 8, 16, 4, 16, 4, 16, 4, 2, 16, 2, 8, 4, 4, 2, 16, 2, 16, 2, 4, 16, 16, 4, 3, 1, 1, 3, 5, 3, 5, 3, 15, 1, 3, 1, 15, 1, 3, 3, 1, 3, 15, 15, 1, 5, 1, 3, 3, 3, 15, 1, 5, 3, 1, 3, 1, 1, 1, 5, 3, 1, 1, 3, 5, 3, 3, 3, 1, 1, 3, 3, 5, 5, 3, 1, 3, 3, 3, 1, 1, 1, 3, 1, 14, 1, 7, 1, 1, 2, 14, 2, 2, 7, 1, 1, 7, 2, 2, 1, 7, 2, 7, 14, 2, 14, 7, 2, 1, 7, 1, 1, 1, 14, 7, 7, 7, 7, 7, 2, 1, 1, 14, 14, 1, 7, 1, 7, 1, 2, 1, 1, 1, 1, 1, 1, 1, 1, 1, 13, 1, 1, 13, 1, 1, 1, 1, 1, 1, 1, 13, 1, 1, 13, 1, 1, 1, 1, 1, 1, 1, 4, 6, 3, 6, 3, 3, 4, 6, 2, 4, 3, 12, 1, 4, 2, 6, 4, 2, 3, 3, 2, 6, 2, 3, 6, 2, 6, 4, 6, 3, 3, 6, 2, 4, 4, 6, 3, 4, 3, 4, 3, 6, 6, 2, 2, 2, 4, 3, 3, 4, 2, 3, 2, 6, 4, 6, 4, 4, 3, 2, 12, 6, 6, 3, 12, 1, 1, 12, 1, 4, 4, 2, 4, 3, 1, 2, 4, 12, 2, 2, 6, 3, 12, 1, 6, 3, 3, 4, 3, 4, 6, 2, 4, 4, 4, 11, 11, 1, 1, 11, 1, 11, 1, 11, 1, 1, 1, 11, 11, 1, 11, 1, 1, 1, 11, 1, 1, 1, 1, 11, 1, 1, 11, 1, 1, 10, 2, 2, 2, 5, 2, 2, 1, 2, 2, 10, 2, 10, 10, 2, 5, 10, 2, 2, 2, 1, 5, 2, 10, 2, 10, 10, 1, 5, 2, 1, 5, 5, 1, 1, 1, 1, 2, 1, 1, 1, 1, 1, 1, 2, 1, 5, 2, 2, 1, 2, 2, 1, 1, 2, 1, 2, 2, 2, 10, 1, 5, 2, 2, 2, 1, 10, 2, 2, 5, 10, 1, 2, 2, 2, 10, 2, 2, 1, 2, 1, 5, 2, 1, 1, 1, 2, 2, 5, 10, 1, 1, 1, 2, 5, 2, 5, 2, 5, 2, 1, 2, 1, 1, 1, 1, 1, 1, 1, 3, 1, 1, 3, 3, 1, 9, 1, 3, 1, 1, 1, 1, 9, 9, 1, 3, 3, 1, 3, 1, 1, 1, 1, 3, 1, 1, 3, 1, 1, 1, 3, 1, 1, 9, 1, 1, 3, 1, 1, 1, 1, 1, 1, 1, 3, 1, 9, 3, 1, 9, 1, 9, 1, 1, 3, 1, 1, 1, 3, 1, 1, 1, 1, 1, 1, 1, 1, 1, 1, 1, 1, 1, 3, 1, 1, 1, 1, 3, 9, 3, 1, 3, 3, 1, 3, 3, 1, 1, 3, 3, 3, 1, 1, 1, 1, 1, 9, 3, 3, 3, 1, 1, 1, 1, 1, 3, 3, 1, 2, 2, 2, 8, 4, 4, 2, 2, 2, 8, 2, 1, 2, 2, 2, 8, 4, 4, 2, 4, 2, 8, 4, 4, 1, 2, 4, 2, 4, 2, 1, 2, 4, 4, 4, 8, 2, 2, 4, 4, 4, 8, 2, 8, 2, 1, 2, 4, 4, 1, 2, 4, 2, 2, 2, 2, 1, 4, 2, 1, 2, 2, 4, 2, 2, 1, 8, 2, 8, 4, 4, 4, 2, 1, 4, 4, 8, 4, 4, 8, 1, 2, 7, 1, 1, 7, 1, 7, 7, 7, 1, 1, 7, 1, 7, 1, 7, 1, 1, 1, 7, 7, 1, 1, 7, 7, 7, 7, 1, 1, 1, 7, 1, 7, 1, 1, 7, 7, 1, 7, 7, 1, 7, 1, 1, 3, 2, 6, 1, 3, 2, 2, 2, 3, 3, 1, 6, 6, 6, 2, 2, 1, 6, 6, 2, 2, 3, 3, 2, 3, 3, 2, 3, 2, 3, 1, 2, 1, 2, 6, 1, 3, 1, 1, 2, 2, 1, 1, 2, 6, 6, 2, 3, 6, 2, 3, 1, 2, 2, 3, 3, 1, 2, 1, 3, 3, 3, 2, 2, 2, 6, 2, 3, 2, 2, 3, 6, 2, 1, 1, 3, 2, 6, 3, 3, 6, 1, 2, 2, 3, 3, 3, 6, 3, 2, 1, 2, 6, 6, 3, 2, 2, 1, 2, 1, 1, 6, 1, 2, 1, 1, 1, 6, 3, 2, 3, 3, 2, 3, 3, 2, 3, 1, 2, 2, 3, 2, 3, 2, 2, 6, 2, 3, 2, 2, 6, 2, 1, 3, 3, 2, 6, 3, 3, 3, 3, 1, 2, 6, 1, 1, 2, 2, 1, 1, 3, 6, 1, 1, 3, 6, 3, 3, 2, 3, 2, 2, 1, 1, 5, 5, 5, 5, 5, 1, 1, 1, 1, 1, 5, 1, 1, 5, 1, 1, 1, 5, 1, 5, 1, 1, 1, 1, 1, 5, 1, 1, 5, 1, 5, 1, 1, 1, 1, 1, 1, 5, 1, 1, 1, 5, 1, 1, 1, 1, 1, 1, 1, 1, 1, 1, 1, 5, 1, 1, 1, 1, 1, 1, 1, 1, 5, 5, 1, 5, 1, 5, 1, 5, 1, 1, 1, 1, 1, 1, 1, 1, 1, 1, 1, 1, 1, 1, 5, 1, 1, 1, 1, 1, 1, 5, 1, 5, 1, 1, 1, 1, 1, 1, 5, 5, 1, 1, 1, 1, 1,

1, 1, 1, 1, 1, 1, 5, 1, 5, 1, 2, 4, 1, 1, 2, 4, 1, 1, 4, 2, 2, 4, 4, 2, 1, 2, 4, 4, 1, 1, 2, 2, 4, 4, 2, 2, 2, 2, 4, 1, 4, 1, 2, 1, 4, 2, 2, 4, 4, 2, 1, 4, 4, 1, 4, 4, 2, 1, 4, 1, 2, 2, 1, 1, 4, 1, 2, 1, 1, 2, 4, 2, 1, 4, 4, 1, 1, 2, 1, 4, 1, 4, 2, 1, 2, 4, 4, 1, 2, 2, 2, 4, 4, 1, 1, 1, 2, 4, 2, 2, 4, 1, 1, 2, 2, 2, 2, 1, 1, 2, 2, 2, 4, 1, 1, 2, 2, 4, 2, 2, 2, 4, 1, 2, 1, 1, 4, 2, 1, 4, 4, 1, 2, 4, 2, 4, 2, 2, 2, 4, 4, 4, 2, 2, 1, 1, 2, 4, 4, 2, 4, 1, 1, 1, 2, 2, 1, 2, 2, 1, 4, 2, 2, 1, 4, 2, 1, 1, 1, 1, 3, 3, 3, 3, 1, 1, 3, 1, 1, 3, 1, 3, 3, 1, 1, 3, 3, 3, 3, 1, 1, 3, 1, 1, 3, 3, 3, 1, 1, 3, 1, 3, 3, 1, 1, 1, 3, 3, 3, 3, 3, 3, 3, 1, 1, 1, 1, 3, 3, 3, 3, 3, 3, 3, 1, 1, 1, 3, 1, 3, 1, 1, 3, 3, 3, 3, 3, 1, 3, 3, 1, 3, 1, 3, 1, 3, 1, 3, 3, 3, 3, 3, 1, 3, 1, 1, 3, 3, 3, 1, 1, 1, 1, 3, 1, 3, 3, 3, 3, 1, 1, 3, 1, 3, 1, 3, 1, 3, 3, 3, 1, 3, 1, 3, 1, 3, 1, 1, 1, 1, 1, 3, 1, 1, 3, 1, 3, 1, 1, 3, 1, 1, 1, 1, 1, 1, 1, 3, 3, 1, 1, 1, 3, 1, 3, 1, 3, 1, 3, 1, 1, 1, 1, 1, 3, 1, 1, 3, 1, 3, 3, 1, 1, 1, 3, 1, 1, 1, 1, 1, 1, 1, 1, 2, 1, 2, 1, 2, 2, 2, 2, 2, 1, 2, 2, 2, 2, 1, 1, 1, 2, 2, 1, 2, 1, 2, 2, 1, 1, 1, 1, 1, 1, 1, 1, 2, 2, 1, 1, 2, 1, 1, 1, 2, 1, 2, 1, 1, 2, 2, 2, 1, 2, 2, 2, 1, 1, 2, 1, 2, 1, 2, 2, 2, 1, 1, 2, 1, 1, 1, 1, 2, 1, 2, 1, 1, 1, 2, 2, 2, 2, 2, 2, 1, 1, 1, 1, 1, 1, 2, 1, 1, 1, 1, 2, 2, 2, 1, 1, 1, 1, 1, 1, 1, 1, 2, 1, 2, 2, 2, 2, 1, 1, 1, 1, 2, 2, 2, 2, 1, 2, 2, 1, 1, 2, 1, 2, 1, 1, 2, 1, 2, 1, 1, 1, 1, 2, 1, 1, 1, 1, 2, 2, 1, 2, 2, 1, 1, 1, 1, 1, 2, 1, 2, 2, 2, 2, 2, 1, 1, 2, 1, 2, 1, 1, 2, 1, 1, 2, 2, 2, 1, 1, 2, 1, 1, 2, 2, 2, 2, 1, 2, 1, 1, 2, 1, 2, 1, 1, 2, 2, 1, 2, 1, 1, 1, 1, 2, 1, 2, 1, 1, 1, 2, 1, 2, 2, 1, 1, 1, 1, 2, 2, 1

Phenopedia diseases ranked according to mean of effect sizes in cluster:
Nervous System Diseases | Mental Disorders | Central Nervous System Diseases | Endocrine System Diseases | Brain Diseases | Psychiatry | Neurodegenerative Diseases | Delirium, Dementia, Amnestic, Cognitive Disorders | Metabolic Diseases | Alzheimer Disease | Dementia | Tauopathies | Glucose Metabolism Disorders | Diabetes Mellitus | Diabetes Mellitus, Type 2

Corresponding mean of effect sizes of each Phenopedia disease:
(In the same order as above)
1.61, 1.35, 1.33, 1.26, 1.24, 1.13, 1.10, 0.90, 0.83, 0.82, 0.78, 0.76, 0.66, 0.58, 0.49

Corresponding total number of genes of each Phenopedia disease:
(In the same order as above)
2.6K, 2.3K, 2.0K, 1.7K, 1.9K, 1.7K, 1.5K, 1.2K, 1.9K, 1.2K, 1.2K, 1.2K, 1.3K, 1.3K, 767

Corresponding number of genes of each Phenopedia disease with at least one TF hit (of selected TFs):
(In the same order as above)
1479, 1317, 1165, 960, 1116, 913, 875, 709, 1091, 698, 703, 669, 699, 726, 421

Corresponding total number of TF hits (of selected TFs) for each Phenopedia disease (in all genes):
(In the same order as above)
8932, 7769, 6946, 6163, 6642, 5261, 5190, 4408, 6595, 4292, 4286, 4082, 4460, 4616, 2657

Corresponding number of selected TFs each Phenopedia disease is involved with:
(In the same order as above)
73, 73, 73, 73, 73, 73, 73, 73, 73, 73, 73, 73, 73, 73, 73

TFs ranked according to mean of effect sizes in cluster:
V$MEIS1_01, V$CREB_Q4_01, VATF_01, VE2F1_Q3_01, V$NFMUE1_Q6, V$AP2_Q6, V$E2F_Q2, V$RFX1_02, V$MZF1_02, V$ATF4_Q2, V$E2F1_Q3, V$AP2ALPHA_03, V$AHRHIF_Q6, V$MEF2_04, V$HIF1_Q5, V$RFX_Q6, V$SP1_Q4_01, V$SP1_Q6, VEFC_Q6, VELK1_02, V$AP2_Q6_01, V$GABP_B, V$AP2GAMMA_01, V$ATF1_Q6, V$CREBP1_Q2, V$GCM_Q2, VAHR_Q5, VE2F1_Q6, V$LRH1_Q5, V$ETF_Q6, V$NRF1_Q6, V$HIF1_Q3, V$HIC1_02, V$CREB_Q4, VATF_B, VAP2ALPHA_01, V$WT1_Q6, V$CREB_Q2, V$IPF1_Q4_01, V$MAZ_Q6, V$CHOP_01, V$YY1_Q6, V$CREBATF_Q6, V$ATF6_01, V$CREB_01, V$TBX5_01, V$ELK1_01, V$E4F1_Q6, V$RREB1_01, V$CREB_02, V$DEC_Q1,

V$KROX_Q6, V$XBP1_01, V$TAACC_B, V$CREBP1CJUN_01, V$SP1_Q6_01, V$CREBP1_01, V$CREB_Q2_01, V$SP1_01, VEGR_Q6, VATF3_Q6, V$HIC1_03, V$SP1_Q2_01, V$NF1_Q6, V$GR_Q6, V$TAXCREB_01, V$E2F_01, V$NF1_Q6_01, V$RBPJK_Q4, V$SP3_Q3, V$CREB_Q3, V$FOXO1_02, V$PITX2_Q2

Corresponding mean of effect sizes of each TF:
(In the same order as above)
1.81, 1.80, 1.78, 1.76, 1.72, 1.67, 1.63, 1.60, 1.59, 1.59, 1.48, 1.47, 1.46, 1.41, 1.40, 1.37, 1.37, 1.35, 1.33, 1.32, 1.31, 1.21, 1.20, 1.19, 1.19, 1.18, 1.15, 1.12, 1.12, 1.12, 1.10, 1.09, 1.08, 1.08, 1.06, 1.05, 1.04, 1.03, 0.98, 0.97, 0.96, 0.96, 0.95, 0.95, 0.93, 0.93, 0.91, 0.89, 0.88, 0.75, 0.72, 0.72, 0.71, 0.70, 0.69, 0.68, 0.63, 0.60, 0.54, 0.53, 0.49, 0.48, 0.46, 0.45, 0.41, 0.35, 0.32, 0.18, 0.16, 0.16, 0.03, -0.01, -0.11

Corresponding total number of TF hits for each TF (genome-wide):
(In the same order as above)
450, 421, 463, 427, 391, 472, 431, 445, 484, 390, 412, 450, 423, 487, 433, 428, 431, 431, 432, 372, 432, 391, 425, 426, 408, 413, 435, 435, 394, 422, 418, 432, 441, 418, 429, 417, 437, 435, 520, 430, 462, 424, 434, 455, 425, 458, 386, 408, 435, 401, 405, 468, 434, 415, 440, 392, 448, 438, 401, 424, 449, 451, 428, 437, 466, 448, 445, 424, 458, 446, 407, 507, 354

Corresponding total number of TF hits for each TF (in all genes in selected Phenopedia diseases):
(In the same order as above)
1322, 1049, 1069, 1067, 1101, 1282, 1214, 1158, 1299, 1059, 1117, 1220, 1217, 1247, 1102, 1206, 1085, 1157, 1140, 831, 1197, 831, 1183, 1049, 1013, 1103, 1215, 1147, 1058, 1173, 946, 1027, 1140, 971, 1055, 1156, 1275, 1123, 1456, 1236, 1365, 1112, 1039, 1130, 1037, 1178, 1006, 1187, 1213, 957, 1083, 1167, 1085, 953, 1120, 1027, 1151, 1008, 1149, 1212, 1072, 1225, 1177, 1180, 1319, 1052, 1088, 1118, 1230, 1217, 1026, 1251, 869

Corresponding number of genes (of selected Phenopedia diseases) each TF is involved with:
(In the same order as above)
223, 206, 219, 209, 210, 235, 227, 217, 231, 203, 211, 221, 216, 231, 210, 214, 204, 218, 217, 167, 212, 176, 213, 202, 204, 204, 223, 216, 205, 234, 195, 205, 215, 205, 220, 210, 232, 227, 271, 220, 250, 195, 211, 218, 200, 205, 189, 213, 216, 197, 198, 234, 211, 193, 210, 194, 218, 209, 204, 214, 222, 216, 208, 227, 232, 210, 217, 208, 226, 212, 203, 242, 147

Corresponding number of selected Phenopedia diseases each TF is involved with:
(In the same order as above)
15, 15, 15, 15, 15, 15, 15, 15, 15, 15, 15, 15, 15, 15, 15, 15, 15, 15, 15, 15, 15, 15, 15, 15, 15, 15, 15, 15, 15, 15, 15, 15, 15, 15, 15, 15, 15, 15, 15, 15, 15, 15, 15, 15, 15, 15, 15, 15, 15, 15, 15, 15, 15, 15, 15, 15, 15, 15, 15, 15, 15, 15, 15, 15, 15, 15, 15, 15, 15, 15, 15, 15, 15

Disease groups of selected Phenopedia diseases ranked according to sum of effect sizes
Nervous System Diseases | Central Nervous System Diseases | Signs and Symptoms | Brain Diseases | Metabolic Diseases | Neurodegenerative Diseases | Glucose Metabolism Disorders | Dementia | Mental Disorders | Endocrine System Diseases | Psychiatry | Diabetes Mellitus | Delirium, Dementia, Amnestic, Cognitive Disorders | Alzheimer Disease | Tauopathies | Diabetes Mellitus, Type 2

Corresponding sum of effect sizes for each disease group of selected Phenopedia diseases
(In the same order as above)
557.66, 440.18, 303.03, 207.82, 186.08, 135.60, 125.65, 116.95, 98.56, 91.92, 82.62, 77.67, 65.86, 59.66, 55.46, 35.69

Cluster for columns 1001 to 1010, rows 337 to 341

Phenopedia diseases

Neurodegenerative Diseases | Alzheimer Disease | Tauopathies | Delirium, Dementia, Amnestic, Cognitive Disorders | Dementia | Nervous System Diseases | Brain Diseases | Central Nervous System Diseases | Mental Disorders | Psychiatry

TFs

V$AHRARNT_01 | V$NFY_01 | V$ALPHACP1_01 | V$NFY_Q6_01 | V$NFY_Q6

Information

All related TFs:
(List of all TFs that are related to any of the PWMs)
AhR, Arnt, CBF(2), CBF-A, CBF-B, CBF-C, CP1, NF-Y, NF-Y', NF-YA, NF-YB, NF-YC, NF-YC-3

Ranked gene list:
(All genes of the selected Phenopedia diseases with hits of any of the selected TFs, ranked according to the total number of TF hits)
PHF1, PCNT, TOMM40, SIGMAR1, OPRS1, CNTF, CAT, AGER, TYSND1, LRRC20, LRP8, ECE1, C10orf84, BLOC1S3, SREBF2, PTPN6, PIK3R1, NET1, KEAP1, GSK3B, FOSB, C12orf57, VCP, SRF, SLC25A28, PI4K2A, NR2C2, NP, NDUFB10, MYST4, IRS1, HSPA2, GPX4, DNAJB1, CDC2, AKR1CL2, VAMP2, PPARA, PITX3, NQO1, NPC2, MTR, M6PR, INSIG1, IL11, HSPA1L, FDPS, ELAC2, DMPK, CHP, C6orf129, WAPAL, TRAF2, TLL2, SUFU, SORT1, SCD, RPP38, PRDM2, PPIL2, PANK1, NFKB2, NDUFS8, NCAPD2, MS, KLF6, KIF20B, KCNIP2, JUNB, ITPRIP, IRS2, HSPA5, HSPA14, GBF1, CUGBP2, COL1A1, CNNM2, BGLAP, POU3F2, GALT, SYNGAP1, PER1, NEUROG2, GYLTL1B, AMT, WNT3, TAF4, SIX5, RNF41, PCNA, MEF2D, GATA2, CLEC16A, CHEK2, BCL2L1, B2M, XBP1, VPS26A, VGF, USF2, UCP2, UBE2H, TSPAN9, SREBF1, SLC2A4, RRP1, PTMS, PON1, PLA2G2D, OPRL1, NTRK1, NOS3, NAT1, MAPT, MAPK8IP1, LIPA, KLF5, JAG1, IL3, IGF1R, HLA-DRA, GSTZ1, GRN, GRM8, GBA, FOXO3, FGFR1, DVL1, DNM2, COMT, CNTFR, CHRNA3, CCND3, CAMK2G, C10orf116, BRD2, BICC1, ARSA, AP3M2, AKT1, AIF1, ACE, ABCA2, ZNF25, ZNF132, UBE2I, TSPAN15, TRAF5, TRAF4, TNNI3, TNK1, TNFAIP3, STK11, SPOCK2, SMC3, SLC25A27, SGMS1, RXRB, RTN3, RBM17, RARG, PTPRA, PTGER4, PTBP1, PRKACB, PRDX5, PPP1R10, POU2F1, PLA2G3, PIP4K2B, PHEX, PDGFRB, PCDH21, PBLD, PAX2, NUMBL, NRF1, NEDD9, NADSYN1, MTRR, MTHFD1L, MTHFD1, MRPL43, MMS19, MLYCD, MLX, MCCC2, MARK4, MAP3K8, LZTS2, LIPG, LDB1, L1CAM, KCNH2, IRS4, IL1R2, HPS1, HNRNPH3, HMGCS1, HMGCR, HINT2, HIF1A, GTPBP4, GCKR, GALNT2, FAM13A, ERCC2, EIF3A, EIF2B2, EBF3, DNAJC12, DNAJB2, DLD, DGAT1, DBP, COG2, CHST3, CEP55, CEBPD, CDC123, CAMK1D, C10orf79, C10orf2, BTRC, BTAF1, BRD8, BMP1, BLMH, ATPAF2, ARNTL, ARHGAP21, APOM, APEX1, ANKMY1, ADK, ADIPOR1, ADD3, ADAMTSL5, ACBD5, ACAT2, ACAT1, ABCC8, ABCA7, ABAT, S100B, PCDH19, NR4A2, NDE1, HIVEP3, HES1, HDAC4, HDAC1, GRIK2, FTH1, FADS2, FADS1, DM1, CX3CR1, TMEM106B, NR3C1, MASP2, HIP1, WDR37, UHRF1, TCF7, TBL3, TAPBP, SLC26A6, SLC1A4, SIM1, SDK1, ROBO3, POLR1D, PNPO, PAX6, PACS2, NAP1L4, MYO7B, MFAP4, MEIS1, KCNN3, HLA-DQB1, HBA1, GCH1, FAM19A4, EPHB1, DHCR7, DDAH2, DCUN1D1, CRY2, CAMTA1, CALU, ATP1A3, ASPM, ARRB2, ACVR1, SLC25A13, OTX2, NELL1, MYH9, ITPR2, EPHA4, BRCA2, ATXN2, ARVCF, ACP2, STMN1, SPRED2, SLC25A4, SLC19A1, SHC3, SCA13, RNPEPL1, RASD2, RAC2, PTS, PTGDS, POMT2, PNPLA2, NPR1, NHLRC1, MSX1, MC1R, MAZ, LTBP4, KCNC3, IRF1, HSP90B1, HIST1H2AG, HES6, GRIN1, GLUL, GLT25D2, FPGS, ERCC8, DLX1, CELSR1, CDC25A, CALM1, CABIN1, BRD1, BAD, ATOX1, ATM, ATF4, ASTN1, ARMETL1, AIPL1, AGRN, ADAMTS13, ACACA, ABCC1, ZFP30, XRCC6, USP20, UFD1L, TSPAN7, TRIM11, TRAPPC9, TMCO6, TBCD, SUNC1, STC2, STAT3, SPTBN4, SP4, SLC39A7, SLC12A2, SCN1B, RUVBL2, RALBP1, PPM1K, PMS2, PIGQ, PEO1, PDGFB, NXNL1, NR1D1, NEUROG1, NCEH1, MVK, MRE11A, MON1B, MLF1, MLEC, MGAT5, LOXL3, LLGL2, KRT5, KIF14, IVD, ITM2C, HCN2, GLA, GCA, GAA, FUT7,

FOXG1, FDFT1, EIF2B4, EIF2AK2, DUSP6, DOC2A, DLG3, DCDC2, CUL9, CSNK1E, CHKA, CHAF1A, CDH1, CASZ1, CAPG, CACNB4, C7orf57, BRIP1, BHLHE41, BAT2, ALDH4A1, AKR1B1, AIP, AIM1, ADH5, ACSS1, ACCN1, ZNF9, ZNF423, ZBTB20, WNT7A, VPS33B, TUB, TSPAN8, TRIM17, TPP1, TMEM108, TEF, TDRD9, TAS2R38, SYNGR1, SLC12A6, SHBG, SGOL2, SAT1, SALL4, RPA2, RGS16, RECQL4, RAF1, RAD54L, QKI, PTPRG, OSM, OPA3, NR3C2, NPTX2, NLGN3, NF2, NDUFAF2, MYH7, MAPKAPK3, LMOD1, KLF10, KIF1B, KIAA0319L, KCNE1, JAG2, IL11RA, ID4, HSPB1, GSTT2, GOT1, GLS, GFRA2, GABRP, GABARAP, FZD3, FZD2, FOXO1, FN1, FKRP, FBLN5, FASTK, FABP3, ERG, EPHA8, EPHA6, EGR4, DPYSL2, DOCK9, DLX2, DLG1, DGKI, DAG1, DACT1, DAB1, CYP2U1, CNBP, CFL1, CCDC60, CCDC148, CCBE1, C7orf23, BHLHB3, ATF2, APC, VANGL1, UTRN, UNG, UBE2D3, TUBA8, TP63, TOP2B, TNFSF10, ST8SIA1, SNAP23, SLTM, SLC16A1, SEZ6L2, RGS19, PYGM, PRMT1, POLG2, PNPT1, PLEKHB1, PLCD1, PITX1, PAX3, PABPN1, OSBPL6, NSMCE2, NR2F1, NPPC, NEUROD1, MYOCD, MYB, MTSS1, MTHFD2, MSH5, MAT2B, MARK1, MAPK1, LYN, LEF1, LCA5, LAG3, INTS12, IDH3A, HOXD11, HMGA1, GSTCD, GRK6, GRID1, GPR55, GCHFR, GART, GAMT, FNIP1, FBLN2, ENTPD7, ECE2, DMTF1, DLGAP4, DICER1, DGCR2, CREB3, CRABP2, CIT, CBLB, CASP2, CAPS, CAMK2D, CALCB, CACNA1S, BNIP3, ATP1B1, ALKBH2, ALDH1B1, ADAMTS8, ACVR1B, ACLY

Corresponding total number of TF hits:
(For each gene listed above, the total number of TF hits for any of the selected TFs, multiplied by the number of selected Phenopedia diseases containing that gene)
50, 50, 40, 40, 40, 40, 40, 40, 36, 36, 36, 36, 36, 36, 30, 30, 30, 30, 30, 30, 30, 30, 28, 27, 27, 27, 27, 27, 27, 27, 27, 27, 27, 27, 27, 27, 20, 20, 20, 20, 20, 20, 20, 20, 20, 20, 20, 20, 20, 20, 18, 18, 18, 18, 18, 18, 18, 18, 18, 18, 18, 18, 18, 18, 18, 18, 18, 18, 18, 18, 18, 18, 18, 18, 18, 18, 18, 18, 16, 16, 15, 15, 15, 15, 15, 12, 12, 12, 12, 12, 12, 12, 12, 12, 12, 10, 10, 10, 10, 10, 10, 10, 10, 10, 10, 10, 10, 10, 10, 10, 10, 10, 10, 10, 10, 10, 10, 10, 10, 10, 10, 10, 10, 10, 10, 10, 10, 10, 10, 10, 10, 10, 10, 10, 10, 10, 10, 10, 10, 10, 9, 8, 8, 8, 8, 8, 8, 8, 8, 8, 8, 8, 8, 8, 8, 7, 7, 7, 7, 6, 6, 6, 6, 6, 6, 6, 6, 6, 6, 6, 6, 6, 6, 6, 6, 6, 6, 6, 6, 6, 6, 6, 6, 6, 6, 6, 6, 6, 6, 6, 6, 6, 6, 6, 5, 5, 5, 5, 5, 5, 5, 5, 5, 5, 4, 4, 4, 4, 4, 4, 4, 4, 4, 4, 4, 4, 4, 4, 4, 4, 4, 4, 4, 4, 4, 4, 4, 4, 4, 4, 4, 4, 4, 4, 4, 4, 4, 4, 4, 4, 4, 4, 4, 4, 4, 4, 4, 4, 4, 4, 4, 3, 3, 3, 3, 3, 3, 3, 3, 3, 3, 3, 3, 3, 3, 3, 3, 3, 3, 3, 3, 3, 3, 3, 3, 3, 3, 3, 3, 3, 3, 3, 3, 3, 3, 3, 3, 3, 3, 3, 3, 3, 3, 3, 3, 3, 3, 3, 3, 3, 3, 3, 3, 3, 3, 3, 3, 3, 3, 3, 3, 3, 3, 3, 3, 3, 3, 3, 3, 3, 3, 3, 3, 3, 3, 3, 3, 2, 2, 2, 2, 2, 2, 2, 2, 2, 2, 2, 2, 2, 2, 2, 2, 2, 2, 2, 2, 2, 2, 2, 2, 2, 2, 2, 2, 2, 2, 2, 2, 2, 2, 2, 2, 2, 2, 2, 2, 2, 2, 2, 2, 2, 2, 2, 2, 2, 2, 2, 2, 2, 2, 2, 2, 2, 2, 2, 2, 2, 2, 2, 2, 2, 2, 2, 2, 2, 2, 2, 2, 2, 2, 2, 2, 1, 1, 1, 1, 1, 1, 1, 1, 1, 1, 1, 1, 1, 1, 1, 1, 1, 1, 1, 1, 1, 1, 1, 1, 1, 1, 1, 1, 1, 1, 1, 1, 1, 1, 1, 1, 1, 1, 1, 1, 1, 1, 1, 1, 1, 1, 1, 1, 1, 1, 1, 1, 1, 1, 1, 1, 1, 1, 1, 1, 1, 1, 1, 1, 1, 1, 1, 1, 1, 1, 1, 1, 1, 1, 1, 1, 1, 1, 1

Corresponding number of selected Phenopedia diseases each gene is involved with:
(In the same order as above)
10, 10, 10, 10, 10, 10, 10, 10, 9, 9, 9, 9, 9, 9, 10, 10, 10, 10, 10, 10, 10, 10, 7, 9, 9, 9, 9, 9, 9, 9, 9, 9, 9, 9, 9, 9, 10, 10, 10, 10, 10, 10, 10, 10, 10, 10, 10, 10, 5, 4, 10, 9, 9, 9, 9, 9, 9, 9, 9, 9, 9, 9, 9, 9, 9, 9, 9, 9, 9, 9, 9, 9, 9, 9, 9, 9, 9, 9, 9, 8, 4, 3, 3, 5, 3, 5, 6, 3, 3, 4, 3, 3, 4, 3, 3, 3, 4, 5, 10, 10, 10, 10, 5, 10, 10, 10, 10, 10, 10, 10, 10, 10, 10, 10, 10, 10, 10, 10, 10, 10, 10, 10, 10, 5, 10, 10, 10, 10, 10, 10, 10, 10, 10, 10, 10, 10, 10, 5, 10, 10, 10, 10, 9, 3, 9, 9, 9, 9, 9, 9, 9, 9, 9, 9, 9, 9, 9, 9, 9, 9, 9, 9, 9, 9, 9, 9, 9, 9, 3, 9, 9, 9, 9, 9, 9, 3, 9, 9, 3, 9, 9, 9, 9, 9, 9, 9, 3, 9, 9, 9, 9, 9, 9, 3, 9, 9, 9, 9, 9, 9, 9, 3, 9, 9, 9, 3, 9, 9, 3, 9, 9, 9, 9, 9, 3, 9, 9, 9, 9, 9, 9, 9, 9, 9, 9, 9, 9, 9, 3, 9, 9, 9, 9, 9, 9, 9, 3, 9, 9, 9, 9, 3, 3, 8, 4, 8, 2, 4, 2, 4, 8, 8, 4, 2, 2, 2, 4, 7, 7, 7, 7, 3, 3, 3, 3, 2, 2, 6, 2, 3, 6, 3, 2, 3, 3, 2, 3, 2, 6, 6, 6, 3, 6, 3, 6, 2, 3, 6, 3, 3, 3, 2, 3, 3, 3, 5, 5, 5, 5, 5, 5, 5, 5, 5, 5, 2, 2, 4, 4, 2, 4, 2, 2, 4, 4, 4, 4, 2, 4, 4, 4, 4, 1, 4, 4, 2, 1, 2, 2, 4, 2, 4, 1, 2, 2, 4, 2, 4, 1, 2, 4, 4, 4, 2, 2, 2, 2, 2, 4, 2, 4, 3, 3, 3, 1, 3, 1, 3, 3, 3, 3, 3, 3, 3, 3, 3, 3, 3, 3, 3, 3, 3, 1, 3, 3, 3, 3, 3, 3, 3, 3, 1, 3, 3, 3, 1, 3, 3, 3, 3, 1, 3, 3, 3, 3, 1, 3, 3, 3, 1, 3, 3, 1, 3, 1, 3, 3, 3, 1, 3, 1, 3, 3, 3, 3, 3, 3, 3, 3, 1, 1, 3, 3, 3, 3, 3, 2, 2, 2, 2, 2, 2, 2, 1, 2, 2, 1, 2, 2, 2, 2, 2, 1, 2, 2, 1, 1, 1, 2, 1, 2, 2, 1, 2, 2, 2, 2, 2, 1, 2, 2, 2, 1, 2, 2, 2, 2, 1, 2, 2, 2, 1, 2, 2, 2, 2, 2, 2, 2, 2, 1, 2, 2, 2, 2, 2, 1, 2, 2, 2, 1, 2, 2, 2, 2, 2, 2, 1, 2, 1, 2, 1, 2, 1, 2, 2, 1, 1, 1, 1, 1, 1, 1, 1, 1, 1, 1, 1, 1, 1, 1, 1, 1, 1, 1, 1, 1, 1, 1, 1, 1, 1, 1, 1, 1, 1, 1, 1, 1, 1, 1, 1, 1, 1, 1, 1, 1, 1, 1, 1, 1, 1, 1, 1, 1, 1, 1, 1, 1, 1, 1, 1, 1, 1, 1, 1, 1, 1, 1, 1, 1, 1, 1, 1, 1

Corresponding number of selected TFs each gene is involved with:

(In the same order as above)
5, 5, 4, 4, 4, 4, 4, 4, 4, 4, 4, 4, 4, 4, 3, 3, 3, 3, 3, 3, 3, 3, 4, 3, 3, 3, 3, 3, 3, 3, 3, 3, 3, 3, 3, 2, 2, 2, 2, 2, 2, 2, 2, 2, 2, 2, 2, 4, 5, 2, 2, 2, 2, 2, 2, 2, 2, 2, 2, 2, 2, 2, 2, 2, 2, 2, 2, 2, 2, 2, 2, 2, 2, 2, 2, 2, 2, 2, 4, 5, 5, 3, 5, 3, 2, 4, 4, 3, 4, 4, 3, 4, 4, 4, 3, 2, 1, 1, 1, 1, 2, 1, 1, 1, 1, 1, 1, 1, 1, 1, 1, 1, 1, 1, 1, 1, 1, 1, 1, 1, 1, 2, 1, 1, 1, 1, 1, 1, 1, 1, 1, 1, 1, 1, 2, 1, 1, 1, 1, 1, 3, 1, 1, 1, 1, 1, 1, 1, 1, 1, 1, 1, 1, 1, 1, 1, 1, 1, 1, 1, 1, 1, 1, 3, 1, 1, 1, 1, 1, 1, 3, 1, 1, 3, 1, 1, 1, 1, 1, 1, 1, 3, 1, 1, 1, 1, 1, 1, 3, 1, 1, 1, 1, 1, 1, 1, 3, 1, 1, 1, 3, 1, 1, 3, 1, 1, 1, 1, 1, 3, 1, 1, 1, 1, 1, 1, 1, 1, 1, 1, 1, 1, 1, 3, 1, 1, 1, 1, 1, 1, 1, 3, 1, 1, 1, 1, 3, 3, 1, 2, 1, 4, 2, 4, 2, 1, 1, 2, 4, 4, 4, 2, 1, 1, 1, 1, 2, 2, 2, 2, 3, 3, 1, 3, 2, 1, 2, 3, 2, 2, 3, 2, 3, 1, 1, 1, 2, 1, 2, 1, 3, 2, 1, 2, 2, 2, 3, 2, 2, 2, 1, 1, 1, 1, 1, 1, 1, 1, 1, 1, 2, 2, 1, 1, 2, 1, 2, 2, 1, 1, 1, 1, 2, 1, 1, 1, 1, 4, 1, 1, 2, 4, 2, 2, 1, 2, 1, 4, 2, 2, 1, 2, 1, 4, 2, 1, 1, 1, 2, 2, 2, 2, 2, 1, 2, 1, 1, 1, 1, 3, 1, 3, 1, 1, 1, 1, 1, 1, 1, 1, 1, 1, 1, 1, 1, 3, 1, 1, 1, 1, 1, 1, 1, 3, 1, 1, 1, 3, 1, 1, 1, 1, 3, 1, 1, 1, 1, 3, 1, 1, 1, 3, 1, 1, 3, 1, 3, 1, 1, 1, 3, 1, 3, 1, 1, 1, 1, 1, 1, 1, 3, 3, 1, 1, 1, 1, 1, 1, 1, 1, 1, 1, 1, 1, 2, 1, 1, 2, 1, 1, 1, 1, 1, 2, 1, 1, 2, 2, 2, 1, 2, 1, 1, 2, 1, 1, 1, 1, 1, 2, 1, 1, 1, 2, 1, 1, 1, 1, 2, 1, 1, 1, 2, 1, 1, 1, 1, 1, 1, 1, 2, 1, 1, 1, 1, 1, 2, 1, 1, 1, 2, 1, 1, 1, 1, 1, 1, 2, 1, 2, 1, 2, 1, 2, 1, 1, 2, 1, 1, 1, 1, 1, 1, 1, 1, 1, 1, 1, 1, 1, 1, 1, 1, 1, 1, 1, 1, 1, 1, 1, 1, 1, 1, 1, 1, 1, 1, 1, 1, 1, 1, 1, 1, 1, 1, 1, 1, 1, 1, 1, 1, 1, 1, 1, 1, 1, 1, 1, 1, 1, 1, 1, 1, 1, 1, 1, 1, 1, 1, 1, 1, 1, 1, 1, 1, 1, 1, 1, 1, 1

Phenopedia diseases ranked according to mean of effect sizes in cluster:
Nervous System Diseases | Brain Diseases | Central Nervous System Diseases | Mental Disorders | Neurodegenerative Diseases | Dementia | Delirium, Dementia, Amnestic, Cognitive Disorders | Tauopathies | Alzheimer Disease | Psychiatry

Corresponding mean of effect sizes of each Phenopedia disease:
(In the same order as above)
3.02, 2.97, 2.80, 2.40, 1.90, 1.85, 1.74, 1.72, 1.55, 1.26

Corresponding total number of genes of each Phenopedia disease:
(In the same order as above)
2.6K, 1.9K, 2.0K, 2.3K, 1.5K, 1.2K, 1.2K, 1.2K, 1.2K, 1.7K

Corresponding number of genes of each Phenopedia disease with at least one TF hit (of selected TFs):
(In the same order as above)
432, 338, 347, 382, 261, 218, 219, 206, 212, 243

Corresponding total number of TF hits (of selected TFs) for each Phenopedia disease (in all genes):
(In the same order as above)
707, 549, 560, 611, 413, 352, 353, 333, 341, 389

Corresponding number of selected TFs each Phenopedia disease is involved with:
(In the same order as above)
5, 5, 5, 5, 5, 5, 5, 5, 5, 5

TFs ranked according to mean of effect sizes in cluster:
V$AHRARNT_01, V$NFY_Q6_01, VNFY_Q6, VNFY_01, V$ALPHACP1_01

Corresponding mean of effect sizes of each TF:
(In the same order as above)
3.49, 2.88, 1.77, 1.23, 1.22

Corresponding total number of TF hits for each TF (genome-wide):
(In the same order as above)
468, 433, 415, 444, 453

Corresponding total number of TF hits for each TF (in all genes in selected Phenopedia diseases):
(In the same order as above)
939, 904, 912, 872, 981

Corresponding number of genes (of selected Phenopedia diseases) each TF is involved with:
(In the same order as above)
194, 184, 180, 180, 191

Corresponding number of selected Phenopedia diseases each TF is involved with:
(In the same order as above)
10, 10, 10, 10, 10

Disease groups of selected Phenopedia diseases ranked according to sum of effect sizes
Nervous System Diseases | Central Nervous System Diseases | Brain Diseases | Neurodegenerative Diseases | Signs and Symptoms | Dementia | Mental Disorders | Delirium, Dementia, Amnestic, Cognitive Disorders | Tauopathies | Alzheimer Disease | Psychiatry

Corresponding sum of effect sizes for each disease group of selected Phenopedia diseases
(In the same order as above)
79.01, 63.92, 31.83, 18.07, 16.99, 16.99, 11.98, 8.68, 8.59, 7.75, 6.28

Cluster for columns 917 to 921, rows 347 to 352

Phenopedia diseases

Anxiety Disorders | Panic Disorder | Eating Disorders | Obsessive-Compulsive Disorder | Cocaine-Related Disorders

TFs

V$CART1_01 | V$MEF2_03 | V$MEF2_02 | V$RSRFC4_Q2 | V$RSRFC4_01 | V$CDP_02

Information

All related TFs:
(List of all TFs that are related to any of the PWMs)
CUTL1, Cart-1, MEF-2A, RSRFC4, aMEF-2

Ranked gene list:
(All genes of the selected Phenopedia diseases with hits of any of the selected TFs, ranked according to the total number of TF hits)
CHRNA5, PRL, GAD1, DLG3, ADRA1A, TRHR, SSTR3, NBEA, HCRTR2, GMFB, GABRB2, ADRB2, HOMER1, VIPR2, PNMT, P2RX7, NSF, NRP1, HOXA1, GRM3, GABRR1, CNTN3, AKAP9, AANAT, SLC6A4, PDYN, NPY5R, VIP, TAC3, SNAP91, SNAP25, SLC6A13, SLC6A12, PTN, PIK3CG, OXTR, OSM, NTRK1, NRXN2, NRG2, NPR3, NPR1, NPPC, NPPB, NOS2, NGF, NEUROD1, NCAM2, LHCGR, INPP1, INHBA, HTR2A, HDAC5, GRM8, GRIK1, GABBR1, DAOA, CRHBP, CREM, CNTF, ASTN2, ASTN1, ARMETL1, ADRB1, ADRA1B, ACHE, ABCB1, NPY1R, GHSR, GHRL, SGCE, RGS2, PDE11A, P2RX4, OLIG1, MEIS1, IMMP2L, IL10, FKBP5, DOCK9, DGKH, CREB1, CLOCK, CAMKK2, BTBD9, ADORA1, ACCN2, TTC12, PER1, NGFB, MTHFR, MOG, MLH1, MCHR2, MANEA, KCNK2, IL28RA, IGF2, DSCAM, BRCA2

Corresponding total number of TF hits:
(For each gene listed above, the total number of TF hits for any of the selected TFs, multiplied by the number of selected Phenopedia diseases containing that gene)
25, 16, 16, 16, 16, 12, 12, 12, 12, 12, 12, 12, 10, 8, 8, 8, 8, 8, 8, 8, 8, 8, 8, 5, 5, 5, 4, 4, 4, 4, 4, 4, 4, 4, 4, 4, 4, 4, 4, 4, 4, 4, 4, 4, 4, 4, 4, 4, 4, 4, 4, 4, 4, 4, 4, 4, 4, 4, 4, 4, 4, 4, 4, 4, 4, 4, 4, 3, 3, 3, 2, 2, 2, 2, 2, 2, 2, 2,

2, 2, 2, 2, 2, 2, 2, 2, 2, 1, 1, 1, 1, 1, 1, 1, 1, 1, 1, 1, 1, 1

Corresponding number of selected Phenopedia diseases each gene is involved with:
(In the same order as above)
5, 4, 4, 4, 4, 4, 4, 4, 4, 4, 4, 4, 5, 4, 4, 2, 4, 4, 4, 4, 4, 4, 4, 4, 5, 5, 5, 4, 4, 4, 4, 4, 4, 4, 4, 4, 4, 4, 4, 4, 4, 4, 4, 4, 4, 4, 4, 4, 4, 4, 4, 4, 1, 2, 4, 4, 2, 4, 2, 4, 4, 4, 1, 4, 4, 4, 4, 3, 3, 3, 2, 2, 1, 2, 2, 2, 2, 2, 2, 1, 1, 1, 2, 2, 2, 1, 2, 1, 1, 1, 1, 1, 1, 1, 1, 1, 1, 1, 1, 1

Corresponding number of selected TFs each gene is involved with:
(In the same order as above)
5, 4, 4, 4, 4, 3, 3, 3, 3, 3, 3, 3, 2, 2, 2, 4, 2, 2, 2, 2, 2, 2, 2, 2, 1, 1, 1, 1, 1, 1, 1, 1, 1, 1, 1, 1, 1, 1, 1, 1, 1, 1, 1, 1, 1, 1, 1, 1, 1, 1, 1, 1, 4, 2, 1, 1, 2, 1, 2, 1, 1, 1, 4, 1, 1, 1, 1, 1, 1, 1, 1, 1, 2, 1, 1, 1, 1, 1, 1, 2, 2, 2, 1, 1, 1, 2, 1, 1, 1, 1, 1, 1, 1, 1, 1, 1, 1, 1, 1, 1

Phenopedia diseases ranked according to mean of effect sizes in cluster:
Anxiety Disorders | Panic Disorder | Obsessive-Compulsive Disorder | Eating Disorders | Cocaine-Related Disorders

Corresponding mean of effect sizes of each Phenopedia disease:
(In the same order as above)
2.46, 2.41, 1.90, 1.70, 1.37

Corresponding total number of genes of each Phenopedia disease:
(In the same order as above)
396, 333, 318, 338, 37

Corresponding number of genes of each Phenopedia disease with at least one TF hit (of selected TFs):
(In the same order as above)
90, 73, 66, 70, 11

Corresponding total number of TF hits (of selected TFs) for each Phenopedia disease (in all genes):
(In the same order as above)
144, 120, 107, 112, 19

Corresponding number of selected TFs each Phenopedia disease is involved with:
(In the same order as above)
6, 6, 6, 6, 6

TFs ranked according to mean of effect sizes in cluster:
V$CART1_01, V$CDP_02, V$MEF2_02, V$MEF2_03, V$RSRFC4_Q2, V$RSRFC4_01

Corresponding mean of effect sizes of each TF:
(In the same order as above)
2.54, 2.11, 1.97, 1.94, 1.63, 1.63

Corresponding total number of TF hits for each TF (genome-wide):
(In the same order as above)
509, 524, 520, 505, 523, 507

Corresponding total number of TF hits for each TF (in all genes in selected Phenopedia diseases):
(In the same order as above)
88, 83, 88, 83, 82, 78

Corresponding number of genes (of selected Phenopedia diseases) each TF is involved with:
(In the same order as above)

28, 27, 27, 23, 28, 25

Corresponding number of selected Phenopedia diseases each TF is involved with:
(In the same order as above)
5, 5, 5, 5, 5, 5

Disease groups of selected Phenopedia diseases ranked according to sum of effect sizes
Anxiety Disorders | Panic Disorder | Obsessive-Compulsive Disorder | Eating Disorders | Substance-Related Disorders | Signs and Symptoms | Cocaine-Related Disorders

Corresponding sum of effect sizes for each disease group of selected Phenopedia diseases
(In the same order as above)
14.79, 14.44, 11.41, 10.22, 8.23, 8.23, 8.23

Cluster for columns 768 to 787, rows 348 to 352

Phenopedia diseases

Retinal Diseases | Macular Degeneration | Retinal Degeneration | Eye Diseases | Vision | Cardiomyopathies | Cardiomyopathy, Dilated | Heart Failure, Congestive | Hypertrophy | Cardiomegaly | Hypertrophy, Left Ventricular | Cardiomyopathy, Hypertrophic | Aortic Valve Stenosis | Heart Valve Diseases | Orientation | Arrhythmia | Atrial Fibrillation | Death, Sudden, Cardiac | Heart Arrest | Death, Sudden

TFs

V$MEF2_03 | V$MEF2_02 | V$RSRFC4_Q2 | V$RSRFC4_01 | V$CDP_02

Information

All related TFs:
(List of all TFs that are related to any of the PWMs)
CUTL1, MEF-2A, RSRFC4, aMEF-2

Ranked gene list:
(All genes of the selected Phenopedia diseases with hits of any of the selected TFs, ranked according to the total number of TF hits)
MMP3, ADRB2, LDB3, SCN5A, MYOZ2, IL2, ELAVL4, EDN2, CYBA, SCARB1, RNF207, IL10, HLA-DRB1, GUCY2D, CKB, SELE, AHR, TCAP, ADRB1, SGCD, PLN, MTHFR, MGST1, IFNG, CPT1B, AMPD1, A2M, TNMD, RDS, PRPH2, DMD, CFH, PPARGC1A, MYL3, GPR98, TSC22D1, HRC, TTN, TNNC1, TH, NOS2A, TFB2M, SOD2, SLC4A10, PRKAG2, NFKB1, IL8, CRNKL1, AKR1B1, SLN, PTPRC, PLEKHA1, NOS2, LRP6, DSC2, CFHR5, CD46, CD36, APP, USH2A, SOX5, REN, PPP1R1A, PARK2, P2RX7, KTN1, IRF1, HEY2, GP6, GINS3, ATP1B4, ATP1B2, AQP1, ADRA2B, TRAF6, SFRP2, NRL, NPY5R, NPY1R, NDP, LTC4S, IL2RA, GHSR, CPE, ATR, ADCY6, WDR36, UBAC2, TSHR, TMTC2, TFB1M, SCN4B, RARA, PTEN, PINK1, NSF, NFATC1, MYOZ1, MLH1, MEIS1, KL, IL5RA, HTR2A, GJA5, GABRR1, DCN, CYP24A1, COL8A1, CCDC28B, CBLB, C3orf10, ATP1A2, ATM, ANKS1B, ADORA1, ABCB1, UGT1A4, UGT1A1, TTC8, TRPC6, TRPC5, TNFRSF1A, TGFBI, STK11, SOD3, SNX16, SLC45A2, SLC24A5, MYD88, IGF2, FTO, FLNC, ENO3, CYP2R1, COL3A1, CFTR, CD40, CASQ2, BMP2K, ACP1

Corresponding total number of TF hits:
(For each gene listed above, the total number of TF hits for any of the selected TFs, multiplied by the number of selected Phenopedia diseases containing that gene)

48, 39, 28, 24, 20, 20, 20, 18, 18, 16, 16, 16, 16, 16, 16, 15, 15, 14, 13, 12, 12, 12, 12, 12, 12, 12, 12, 10, 10, 10, 10, 10, 9, 9, 9, 8, 8, 7, 7, 7, 7, 6, 6, 6, 6, 6, 6, 6, 5, 5, 5, 5, 5, 5, 5, 5, 5, 5, 4, 4, 4, 4, 4, 4, 4, 4, 4, 4, 4, 4, 4, 4, 4, 3, 3, 3, 3, 3, 3, 3, 3, 3, 3, 3, 2, 2, 2, 2, 2, 2, 2, 2, 2, 2, 2, 2, 2, 2, 2, 2, 2, 2, 2, 2, 2, 2, 2, 2, 2, 2, 2, 1, 1, 1, 1, 1, 1, 1, 1, 1, 1, 1, 1, 1, 1, 1, 1, 1, 1, 1, 1, 1, 1, 1

Corresponding number of selected Phenopedia diseases each gene is involved with:
(In the same order as above)
16, 13, 7, 12, 5, 5, 5, 6, 6, 4, 4, 16, 16, 4, 4, 5, 5, 7, 13, 6, 12, 12, 4, 4, 3, 3, 4, 5, 5, 5, 5, 5, 9, 9, 3, 2, 8, 7, 7, 7, 7, 2, 6, 2, 6, 6, 6, 3, 3, 5, 5, 5, 5, 5, 5, 5, 5, 5, 5, 4, 2, 2, 1, 2, 1, 1, 1, 1, 4, 4, 1, 1, 2, 4, 1, 3, 3, 3, 3, 3, 1, 3, 3, 3, 3, 3, 2, 1, 2, 1, 2, 2, 2, 2, 1, 1, 1, 1, 2, 2, 1, 1, 2, 2, 1, 2, 2, 1, 1, 1, 2, 1, 1, 2, 1, 2, 1, 1, 1, 1, 1, 1, 1, 1, 1, 1, 1, 1, 1, 1, 1, 1, 1, 1, 1, 1, 1, 1

Corresponding number of selected TFs each gene is involved with:
(In the same order as above)
3, 3, 4, 2, 4, 4, 4, 3, 3, 4, 4, 1, 1, 4, 4, 3, 3, 2, 1, 2, 1, 1, 3, 3, 4, 4, 3, 2, 2, 2, 2, 2, 1, 1, 3, 4, 1, 1, 1, 1, 1, 3, 1, 3, 1, 1, 1, 2, 2, 1, 1, 1, 1, 1, 1, 1, 1, 1, 1, 1, 2, 2, 4, 2, 4, 4, 4, 4, 1, 1, 4, 4, 2, 1, 3, 1, 1, 1, 1, 1, 3, 1, 1, 1, 1, 1, 1, 2, 1, 2, 1, 1, 1, 1, 2, 2, 2, 2, 1, 1, 2, 2, 1, 1, 2, 1, 1, 2, 2, 2, 1, 2, 2, 1, 2, 1, 1, 1, 1, 1, 1, 1, 1, 1, 1, 1, 1, 1, 1, 1, 1, 1, 1, 1, 1, 1, 1, 1, 1

Phenopedia diseases ranked according to mean of effect sizes in cluster:
Cardiomegaly | Macular Degeneration | Retinal Degeneration | Cardiomyopathies | Cardiomyopathy, Dilated | Heart Arrest | Death, Sudden, Cardiac | Heart Failure, Congestive | Cardiomyopathy, Hypertrophic | Hypertrophy | Aortic Valve Stenosis | Retinal Diseases | Heart Valve Diseases | Hypertrophy, Left Ventricular | Death, Sudden | Arrhythmia | Vision | Atrial Fibrillation | Orientation | Eye Diseases

Corresponding mean of effect sizes of each Phenopedia disease:
(In the same order as above)
2.74, 2.61, 2.45, 2.36, 1.95, 1.87, 1.81, 1.78, 1.72, 1.69, 1.53, 1.52, 1.45, 1.38, 1.00, 0.75, 0.57, 0.39, 0.37, -0.00

Corresponding total number of genes of each Phenopedia disease:
(In the same order as above)
149, 157, 179, 146, 75, 52, 54, 112, 56, 163, 63, 261, 91, 89, 97, 150, 264, 89, 49, 573

Corresponding number of genes of each Phenopedia disease with at least one TF hit (of selected TFs):
(In the same order as above)
35, 27, 28, 31, 18, 14, 14, 26, 12, 31, 12, 39, 18, 19, 18, 25, 37, 14, 9, 74

Corresponding total number of TF hits (of selected TFs) for each Phenopedia disease (in all genes):
(In the same order as above)
59, 52, 56, 58, 31, 22, 22, 49, 23, 51, 22, 75, 34, 30, 28, 36, 63, 22, 12, 138

Corresponding number of selected TFs each Phenopedia disease is involved with:
(In the same order as above)
5, 5, 5, 5, 5, 5, 5, 5, 4, 5, 4, 5, 5, 5, 5, 5, 5, 5, 5, 5

TFs ranked according to mean of effect sizes in cluster:
V$MEF2_02, V$MEF2_03, V$RSRFC4_Q2, V$RSRFC4_01, V$CDP_02

Corresponding mean of effect sizes of each TF:
(In the same order as above)
2.01, 1.91, 1.86, 1.38, 0.33

Corresponding total number of TF hits for each TF (genome-wide):

(In the same order as above)
520, 505, 523, 507, 524

Corresponding total number of TF hits for each TF (in all genes in selected Phenopedia diseases):
(In the same order as above)
196, 192, 206, 173, 116

Corresponding number of genes (of selected Phenopedia diseases) each TF is involved with:
(In the same order as above)
56, 58, 54, 48, 35

Corresponding number of selected Phenopedia diseases each TF is involved with:
(In the same order as above)
20, 20, 20, 20, 18

Disease groups of selected Phenopedia diseases ranked according to sum of effect sizes
Thoracic Diseases | Respiratory Tract Diseases | Mediastinal Diseases | Cardiovascular Diseases | Pathological Conditions, Anatomical | Hypertrophy | Retinal Diseases | Eye Diseases | Cardiomyopathies | Cardiomegaly | Retinal Degeneration | Heart Arrest | Hypertrophy, Left Ventricular | Heart Valve Diseases | Death, Sudden | Death | Macular Degeneration | Cardiomyopathy, Dilated | Death, Sudden, Cardiac | Heart Failure, Congestive | Cardiomyopathy, Hypertrophic | Ventricular Outflow Obstruction | Aortic Valve Stenosis | Arrhythmia | Vision | Atrial Fibrillation | Arrhythmias, Cardiac | Orientation

Corresponding sum of effect sizes for each disease group of selected Phenopedia diseases
(In the same order as above)
86.00, 86.00, 86.00, 86.00, 37.67, 37.67, 32.90, 32.89, 30.19, 29.24, 25.30, 18.38, 15.54, 14.89, 14.03, 14.03, 13.04, 9.76, 9.04, 8.89, 8.62, 7.66, 7.66, 3.77, 2.87, 1.93, 1.93, 1.85

Cluster for columns 799 to 801, rows 348 to 351

Phenopedia diseases

Muscular Diseases | Muscular Disorders, Atrophic | Muscular Dystrophies

TFs

V$MEF2_03 | V$MEF2_02 | V$RSRFC4_Q2 | V$RSRFC4_01

Information

All related TFs:
(List of all TFs that are related to any of the PWMs)
MEF-2A, RSRFC4, aMEF-2

Ranked gene list:
(All genes of the selected Phenopedia diseases with hits of any of the selected TFs, ranked according to the total number of TF hits)
MYOZ2, LDB3, FKRP, CLCN1, KTN1, POMGNT1, MYOZ1, CAPN3, ATP1B4, ATP1B2, AMPD1, ADRA1A, SELE, MMP3, FLNC, ENO3, DMD, ADRB2, IFNG, ATP1A2, TPM3, TNNT3, MYL3, IL10, IGF2, HTR2A

Corresponding total number of TF hits:
(For each gene listed above, the total number of TF hits for any of the selected TFs, multiplied by the

number of selected Phenopedia diseases containing that gene)
12, 12, 12, 12, 9, 6, 6, 6, 4, 4, 4, 4, 3, 3, 3, 3, 3, 3, 2, 2, 1, 1, 1, 1, 1, 1

Corresponding number of selected Phenopedia diseases each gene is involved with:
(In the same order as above)
3, 3, 3, 3, 3, 3, 3, 3, 1, 1, 1, 1, 1, 1, 3, 3, 3, 1, 1, 1, 1, 1, 1, 1, 1, 1

Corresponding number of selected TFs each gene is involved with:
(In the same order as above)
4, 4, 4, 4, 3, 2, 2, 2, 4, 4, 4, 4, 3, 3, 1, 1, 1, 3, 2, 2, 1, 1, 1, 1, 1, 1

Phenopedia diseases ranked according to mean of effect sizes in cluster:
Muscular Diseases | Muscular Dystrophies | Muscular Disorders, Atrophic

Corresponding mean of effect sizes of each Phenopedia disease:
(In the same order as above)
3.27, 3.26, 3.25

Corresponding total number of genes of each Phenopedia disease:
(In the same order as above)
158, 36, 39

Corresponding number of genes of each Phenopedia disease with at least one TF hit (of selected TFs):
(In the same order as above)
26, 11, 11

Corresponding total number of TF hits (of selected TFs) for each Phenopedia disease (in all genes):
(In the same order as above)
63, 28, 28

Corresponding number of selected TFs each Phenopedia disease is involved with:
(In the same order as above)
4, 4, 4

TFs ranked according to mean of effect sizes in cluster:
V$MEF2_02, V$MEF2_03, V$RSRFC4_01, V$RSRFC4_Q2

Corresponding mean of effect sizes of each TF:
(In the same order as above)
3.76, 3.58, 3.32, 2.39

Corresponding total number of TF hits for each TF (genome-wide):
(In the same order as above)
520, 505, 507, 523

Corresponding total number of TF hits for each TF (in all genes in selected Phenopedia diseases):
(In the same order as above)
33, 31, 29, 26

Corresponding number of genes (of selected Phenopedia diseases) each TF is involved with:
(In the same order as above)
17, 17, 15, 14

Corresponding number of selected Phenopedia diseases each TF is involved with:
(In the same order as above)

3, 3, 3, 3

Disease groups of selected Phenopedia diseases ranked according to sum of effect sizes
Signs and Symptoms | Nervous System Diseases | Musculoskeletal Diseases | Spinal Diseases | Spinal Cord Diseases | Pathological Conditions, Anatomical | Neurologic Manifestations | Nervous System Malformations | Muscular Disorders, Atrophic | Muscular Atrophy | Congenital, Hereditary, and Neonatal Diseases and Abnormalities | Central Nervous System Diseases | Bone Diseases | Atrophy | Peripheral Nervous System Diseases | Neuromuscular Junction Diseases | Neuromuscular Diseases | Muscular Diseases | Muscular Dystrophies | Genetic Diseases, Inborn

Corresponding sum of effect sizes for each disease group of selected Phenopedia diseases
(In the same order as above)
39.14, 39.14, 39.14, 26.05, 26.05, 26.05, 26.05, 26.05, 26.05, 26.05, 26.05, 26.05, 26.05, 26.05, 13.09, 13.09, 13.09, 13.09, 13.09, 13.09, 13.05, 13.05

Cluster for columns 772 to 781, rows 360 to 367

Phenopedia diseases

Vision | Cardiomyopathies | Cardiomyopathy, Dilated | Heart Failure, Congestive | Hypertrophy | Cardiomegaly | Hypertrophy, Left Ventricular | Cardiomyopathy, Hypertrophic | Aortic Valve Stenosis | Heart Valve Diseases

TFs

V$AMEF2_Q6 | V$AHRARNT_02 | V$OCT1_02 | V$MEF2_Q6_01 | V$MEF2_01 | V$HMEF2_Q6 | V$LHX3_01 | V$OCT1_01

Information

All related TFs:
(List of all TFs that are related to any of the PWMs)
AhR, Arnt, LHX3a, LHX3b, Lhx3a, Lhx3b, MEF-2A, MEF-2C/delta8, MEF-2DAB, Oct-1, POU2F1, POU2F1a, aMEF-2

Ranked gene list:
(All genes of the selected Phenopedia diseases with hits of any of the selected TFs, ranked according to the total number of TF hits)
MYL2, MTHFR, TNNC1, ADRB2, PTPRC, PLN, MMP1, ESR1, HLA-DQB1, EDN1, CCR5, RTN4, CPE, NR3C1, MYOZ2, HFE, DMD, CTLA4, TNNI3, MYH7, SLC22A5, NPPB, KCNH2, IL10, ESR2, CHRM2, AGTR1, TTN, SCN5A, PPARGC1A, NPPA, MMP9, MICB, IL1B, GLA, FKTN, ADRB1, SOD2, F5, CPT2, CPT1B, CCL2, SGCD, IRX4, APOB, VEGFA, TFB2M, SP4, RAF1, PTPN22, PPARG, NFKB1, NDP, MYOZ1, LEPR, IL2, IGF1, GNB3, EDN2, CXADR, CRP, ADRA2C, VEGF, TH, SFRS3, SFRP2, RORA, RARA, PPP3CA, HEY2, GCKR, ENO3, CRNKL1, CPT1A, CALM3, ADCY6, TTC8, SOX2, PTH, NFATC4, LUM, LEP, KERA, KCNQ1, IL1A, IL12B, FXN, FGG, CYP1B1, COL18A1, CEP290, BDNF, ATM, APOC3, ANKH, ABCB1, ABCA4, TRPC1, TMEM43, SKI, RPE65, RDS, RARB, PRPH2, PRKCB, PLEKHA1, PINK1, PDE6B, PARP1, P2RX7, OTX2, NRG2, NOS1AP, NFATC1, MTHFD1, LRAT, KTN1, KRAS, KLF5, KL, KDR, ITGB2, IMPDH1, IGF1R, HTR1A, HBA1, GNB1, GNAT2, GJA8, GDF6, FLT1, FKBP5, ERCC6, ERCC2, EPYC, EPHX1, ELAVL4, EGR1, CX3CR1, CRX, COL4A3, CHRNA4, CD59, CD46, CCDC28B, C8B, C8A, APEX1, APC, ANKS1B, AIPL1, AHR, ADAM17

Corresponding total number of TF hits:

(For each gene listed above, the total number of TF hits for any of the selected TFs, multiplied by the number of selected Phenopedia diseases containing that gene)
24, 24, 21, 21, 16, 16, 16, 16, 14, 14, 14, 12, 12, 10, 10, 10, 10, 10, 9, 9, 8, 8, 8, 8, 8, 8, 8, 7, 7, 7, 7, 7, 7, 7, 7, 7, 7, 6, 6, 6, 6, 6, 5, 5, 5, 4, 4, 4, 4, 4, 4, 4, 4, 4, 4, 4, 4, 4, 4, 4, 4, 3, 3, 3, 3, 3, 3, 3, 3, 3, 3, 3, 3, 3, 3, 2, 2, 2, 2, 2, 2, 2, 2, 2, 2, 2, 2, 2, 2, 2, 2, 2, 2, 2, 2, 1, 1, 1, 1, 1, 1, 1, 1, 1, 1, 1, 1, 1, 1, 1, 1, 1, 1, 1, 1, 1, 1, 1, 1, 1, 1, 1, 1, 1, 1, 1, 1, 1, 1, 1, 1, 1, 1, 1, 1, 1, 1, 1, 1, 1, 1, 1, 1, 1, 1, 1, 1, 1, 1, 1

Corresponding number of selected Phenopedia diseases each gene is involved with:
(In the same order as above)
8, 6, 7, 7, 4, 8, 8, 8, 7, 7, 7, 4, 3, 2, 5, 5, 5, 5, 9, 9, 4, 8, 2, 8, 8, 4, 8, 7, 7, 7, 7, 7, 7, 7, 7, 7, 7, 2, 6, 3, 3, 3, 5, 5, 5, 4, 2, 1, 4, 2, 4, 4, 1, 1, 2, 4, 2, 4, 4, 4, 4, 4, 3, 3, 1, 3, 1, 1, 1, 1, 1, 1, 3, 3, 3, 3, 1, 1, 2, 2, 1, 2, 1, 2, 2, 1, 2, 1, 1, 1, 1, 2, 1, 1, 1, 1, 1, 1, 1, 1, 1, 1, 1, 1, 1, 1, 1, 1, 1, 1, 1, 1, 1, 1, 1, 1, 1, 1, 1, 1, 1, 1, 1, 1, 1, 1, 1, 1, 1, 1, 1, 1, 1, 1, 1, 1, 1, 1, 1, 1, 1, 1, 1, 1, 1, 1, 1, 1, 1

Corresponding number of selected TFs each gene is involved with:
(In the same order as above)
3, 4, 3, 3, 4, 2, 2, 2, 2, 2, 2, 3, 4, 5, 2, 2, 2, 2, 1, 1, 2, 1, 4, 1, 1, 2, 1, 1, 1, 1, 1, 1, 1, 1, 1, 1, 3, 1, 2, 2, 2, 1, 1, 1, 1, 2, 4, 1, 2, 1, 1, 4, 4, 2, 1, 2, 1, 1, 1, 1, 1, 1, 1, 3, 1, 3, 3, 3, 3, 3, 3, 1, 1, 1, 1, 2, 2, 1, 1, 2, 1, 2, 1, 1, 2, 1, 2, 2, 2, 2, 1, 2, 2, 2, 2, 2, 1, 1, 1, 1, 1, 1, 1, 1, 1, 1, 1, 1, 1, 1, 1, 1, 1, 1, 1, 1, 1, 1, 1, 1, 1, 1, 1, 1, 1, 1, 1, 1, 1, 1, 1, 1, 1, 1, 1, 1, 1, 1, 1, 1, 1, 1, 1, 1, 1, 1, 1, 1, 1

Phenopedia diseases ranked according to mean of effect sizes in cluster:
Hypertrophy | Cardiomyopathies | Cardiomegaly | Cardiomyopathy, Dilated | Aortic Valve Stenosis | Vision | Cardiomyopathy, Hypertrophic | Hypertrophy, Left Ventricular | Heart Valve Diseases | Heart Failure, Congestive

Corresponding mean of effect sizes of each Phenopedia disease:
(In the same order as above)
2.09, 1.97, 1.96, 1.50, 1.38, 1.33, 1.28, 1.17, 0.88, 0.72

Corresponding total number of genes of each Phenopedia disease:
(In the same order as above)
163, 146, 149, 75, 63, 264, 56, 89, 91, 112

Corresponding number of genes of each Phenopedia disease with at least one TF hit (of selected TFs):
(In the same order as above)
61, 54, 53, 27, 24, 84, 23, 32, 34, 39

Corresponding total number of TF hits (of selected TFs) for each Phenopedia disease (in all genes):
(In the same order as above)
94, 90, 84, 46, 35, 126, 33, 47, 49, 63

Corresponding number of selected TFs each Phenopedia disease is involved with:
(In the same order as above)
8, 8, 8, 8, 8, 8, 8, 8, 8, 8

TFs ranked according to mean of effect sizes in cluster:
V$AMEF2_Q6, V$MEF2_Q6_01, V$LHX3_01, V$AHRARNT_02, V$MEF2_01, V$OCT1_02, V$HMEF2_Q6, V$OCT1_01

Corresponding mean of effect sizes of each TF:
(In the same order as above)
3.34, 2.11, 1.59, 1.49, 0.94, 0.83, 0.66, 0.45

Corresponding total number of TF hits for each TF (genome-wide):

(In the same order as above)
498, 528, 506, 446, 528, 515, 516, 472

Corresponding total number of TF hits for each TF (in all genes in selected Phenopedia diseases):
(In the same order as above)
123, 100, 84, 71, 75, 74, 76, 64

Corresponding number of genes (of selected Phenopedia diseases) each TF is involved with:
(In the same order as above)
37, 32, 33, 29, 27, 25, 28, 24

Corresponding number of selected Phenopedia diseases each TF is involved with:
(In the same order as above)
10, 10, 10, 10, 10, 10, 10, 10

Disease groups of selected Phenopedia diseases ranked according to sum of effect sizes
Thoracic Diseases | Respiratory Tract Diseases | Mediastinal Diseases | Cardiovascular Diseases | Pathological Conditions, Anatomical | Hypertrophy | Cardiomyopathies | Cardiomegaly | Hypertrophy, Left Ventricular | Heart Valve Diseases | Cardiomyopathy, Dilated | Ventricular Outflow Obstruction | Aortic Valve Stenosis | Vision | Cardiomyopathy, Hypertrophic | Heart Failure, Congestive

Corresponding sum of effect sizes for each disease group of selected Phenopedia diseases
(In the same order as above)
81.05, 81.05, 81.05, 81.05, 52.00, 52.00, 37.96, 35.25, 19.59, 18.06, 11.96, 11.03, 11.03, 10.64, 10.21, 5.74

Cluster for columns 328 to 559, rows 434 to 443

Phenopedia diseases

Colonic Diseases, Functional | Irritable Bowel Syndrome | Abdominal Pain | Familial Mediterranean Fever | Disorders of Excessive Somnolence | Narcolepsy | Ileal Diseases | Ileitis | Mucocutaneous Lymph Node Syndrome | Blindness | Headache | Migraine Disorders | Migraine with Aura | Migraine without Aura | Pregnancy Complications, Cardiovascular | Apnea | Sleep Apnea, Obstructive | Sleep Apnea Syndromes | Respiration Disorders | Respiratory Distress Syndrome, Newborn | HELLP Syndrome | Hemolysis | Bronchopulmonary Dysplasia | Respiratory Distress Syndrome, Adult | Bacteremia | Meningococcal Infections | Critical Illness | Multiple Organ Failure | Cadaver | Delayed Graft Function | Puerperal Disorders | Coronary Restenosis | Coronary Stenosis | Rupture | Constriction, Pathologic | Uremia | Coronary Thrombosis | Brain Damage, Chronic | Cerebral Palsy | Antiphospholipid Syndrome | Fetal Death | Aneurysm, Ruptured | Drug-Induced Liver Injury | Leukocytosis | Retinal Vein Occlusion | Gout | Hyperuricemia | Mitral Valve Prolapse | Exfoliation Syndrome | Glomerulosclerosis, Focal Segmental | Reperfusion Injury | Gallbladder Neoplasms | Papilloma | Photosensitivity Disorders | Herpesviridae Infections | Epstein-Barr Virus Infections | Neoplasms, Experimental | Constipation | Cough | Pneumonia | Emphysema | Pulmonary Emphysema | Ulcer | Duodenal Ulcer | Duodenal Diseases | Peptic Ulcer | Gastritis, Atrophic | Gastritis | Helicobacter Infections | Acute Disease | Bone Resorption | Osteoporosis, Postmenopausal | Eclampsia | Infertility, Female | Abortion, Habitual | Abortion, Spontaneous | Limb Deformities, Congenital | Retinopathy of Prematurity | alpha 1-Antitrypsin Deficiency | Dementia, Vascular | Intracranial Arteriosclerosis | Dyspepsia | Vesico-Ureteral Reflux | Urinary Tract Infections | Arthralgia | Stomach Ulcer | Enterocolitis | Idiopathic Pulmonary Fibrosis | Pulmonary Fibrosis | Lipodystrophy | Shock | Viremia | Sepsis | Systemic Inflammatory Response Syndrome | Rubella | Measles | Mumps | Tuberculosis | Mycobacterium Infections | Tuberculosis, Pulmonary | Bacterial Infections | Gram-Negative Bacterial Infections | Malaria | Parasitic Diseases | Bronchiectasis | Helminthiasis | Brucellosis | Coronary Aneurysm | Salmonella Infections | Shock, Septic | Myelitis | Deltaretrovirus Infections | HTLV-I Infections | Spinal Diseases | Spondylitis | Ankylosis | Spondylitis, Ankylosing | Spondylarthritis | Spondylarthropathies | Vasculitis | Uveitis | Behcet Syndrome |

Panuveitis | Uveitis, Anterior | Arthritis, Psoriatic | Psoriasis | Stomatitis | Epidermal Necrolysis, Toxic | Stevens-Johnson Syndrome | Erythema | Erythema Nodosum | Drug Eruptions | Drug Hypersensitivity | Encephalitis | Severe Acute Respiratory Syndrome | Meningitis | Nasal Polyps | Scleroderma, Systemic | Salivary Gland Diseases | Sjogren's Syndrome | Polymyalgia Rheumatica | Arteritis | Temporal Arteritis | Graft vs Host Disease | Purpura, Schoenlein-Henoch | Immune Complex Diseases | Vasculitis, Hypersensitivity | Streptococcal Infections | Rheumatic Fever | Rheumatic Heart Disease | Hematologic Neoplasms | Leprosy | Pneumoconiosis | Silicosis | Endometriosis | Leukemia, T-Cell, Acute | Sarcoidosis | Mycobacterium Infections, Atypical | Sarcoidosis, Pulmonary | Pregnancy Complications, Infectious | Vaginal Diseases | Vaginosis, Bacterial | Malaria, Cerebral | Malaria, Falciparum | Mycoses | Chagas Cardiomyopathy | Chagas Disease | Parasitemia | Aspergillosis | Lung Diseases, Fungal | Cytomegalovirus Infections | Opportunistic Infections | Hepatitis, Autoimmune | Urticaria | Respiratory Sounds | Paranasal Sinus Diseases | Sinusitis | Albuminuria | Conjunctival Diseases | Corneal Diseases | Conjunctivitis | Chlamydia Infections | Eye Infections, Bacterial | Trachoma | Exanthema | Liver Failure | Food Hypersensitivity | Leptospirosis | Picornaviridae Infections | Gingival Diseases | Sarcoma, Kaposi | Pemphigus | Skin Diseases, Vesiculobullous | Thymoma | Oral Submucous Fibrosis | Purpura, Thrombocytopenic | Purpura, Thrombocytopenic, Idiopathic | Otitis Media | Lichen Planus | Lichen Planus, Oral | Periodontitis, Juvenile | Periodontal Pocket | Alveolar Bone Loss | Periodontal Attachment Loss | Nephrosis | Nephrotic Syndrome | Sensation | Encephalomyelitis | Fatigue Syndrome, Chronic | Neuralgia | Intervertebral Disk Displacement | Sciatica | Multiple System Atrophy | Ventricular Dysfunction | Ventricular Dysfunction, Left | Hypotension | Weight Loss | Eosinophilia | Postoperative Complications | Pain, Postoperative | Autonomic Nervous System Diseases | Prosthesis Failure | Decision Making | Metal Metabolism, Inborn Errors | Iron Metabolism Disorders | Iron Overload | Pancreatitis | Pancreatitis, Alcoholic | Pancreatitis, Chronic | Alcohol-Induced Disorders | Liver Cirrhosis, Alcoholic | Liver Diseases, Alcoholic

TFs

V$NRF2_Q4 | V$NFE2_01 | V$HOXA7_01 | V$SMAD4_Q6 | F$STRE_01 | V$SRF_Q5_01 | V$ER_Q6 | V$TATA_01 | V$FREAC3_01 | V$PPARG_03

Information

All related TFs:
(List of all TFs that are related to any of the PWMs)
ER-alpha, ER-alpha-L, FOXC1, HOXA7, NF-E2, Nrf2, Nrf2:MafK, PPAR-gamma, PPAR-gamma1, PPAR-gamma2, SRF, SRF-I, SRF-L, SRF-M, SRF-S, Smad4, TBP

Ranked gene list:
(All genes of the selected Phenopedia diseases with hits of any of the selected TFs, ranked according to the total number of TF hits)
TNF, LTA, NOS3, IL6, IL10, TGFB1, MTHFR, HLA-B, GSTP1, VEGFA, TNFRSF1A, HLA-C, TLR9, SERPINE1, HLA-DRB1, CCR5, IL1RN, HSPA1L, HLA-DQB1, VEGF, TCF7, IRF1, IL8, APOE, CTLA4, COMT, F5, VDR, TNFRSF1B, ADD1, IL12B, GNAS, AGTR1, HMOX1, ADRB2, TP53, F2, SELE, IL10RA, CCL5, NOS2A, CISH, CYP1A1, NQO1, XRCC1, LTC4S, MEFV, NOS2, ABCB1, TAP1, EDN1, CXCL12, CRP, LST1, CYBA, G6PD, MDM2, LTB, HTR2A, PSMB9, NFKBIA, IL2RB, HIF1A, CAT, AKT1, ADORA2A, MSH2, IL6R, HSPA2, ALDH2, NLRP3, DDAH2, COL1A1, LEP, UCP2, TRADD, RUNX3, PSMB8, NR3C1, DRD2, ADRB1, SLC11A2, SIGIRR, PGC, IL8RA, IL12RB2, DUOX2, CMA1, APOC3, ADRA1A, PLAU, NFKBIZ, MC1R, LEPR, CD40LG, ANKH, ADH1B, XRCC3, VIPR1, STAT4, SOD1, OGG1, MVK, IL7R, HRAS, GCH1, GABBR1, CYP2C8, APOA1, UGT1A1, NOS1, IRAK1, CD40, APOB, UBASH3B, SQSTM1, SLC22A12, PSORS1, PROZ, PDCD1, LOXL1, KRAS, IL18R1, DRD4, CCHCR1, ATXN1, TH, TFRC, TACR1, SFTPC, RXRB, RHCE, PTPN11, NR3C2, NPPA, IFNAR1, GSTM3, GCLC, GABRB3, CLU, C4A, ADH1C, TNFRSF11A, STAT6, SH2B3, MMP11, LTBP4, LMNA, IL1F10, HMGB1, GALT, CTSZ, COL11A2, CFB, CDKN1B, CDKN1A, SP110, PPARA, HTR6, HTR1B, CYP1B1, COL6A1, UGT1A6, TPI1,

TNFSF14, TMC6, STAT5A, RARG, PRR3, NFKBIE, MYH9, MUC1, MSX2, JUN, IL2RA, IL23A, IL16, ICOS, HMHA1, GNL1, GLO1, ENPP1, CYP27B1, CSNK1D, COL9A3, CDK6, CCR7, BRCA1, BAX, BAT3, AP1B1, ANGPT2, AGER, ADRA2B, UBAC2, TCN2, SIPA1, RUNX1, PSEN1, PRF1, PPARGC1A, POMC, PAX2, MBP, LIF, KCNH2, ITGB2, IRS1, GCLM, FAM167A, CCL3, C4B, BRD2, APEX1, ADA, ZFHX3, UGT1A9, TBX21, RYR3, PNMT, PGM1, PER2, OAZ1, MX2, MAN1B1, LCAT, KIRREL, ITPKC, IGF2, HSPB6, HMBS, GRLF1, FMN1, DNAH8, CYP46A1, CXCR4, CST3, CSF1R, CPT1B, COL2A1, CLEC16A, BRAF, BMPR1A, APOA5, AKT2, ADM, VWF, UCP3, TPST2, SLC46A1, SKIV2L, SDHC, RETN, RARA, PLCE1, PIK3CA, PDGFRB, NR1D1, NQO2, NPHS1, MYB, LRPAP1, LPIN1, LIG4, LAMB2, LAMA2, LAG3, KCNS3, KCNK12, KCNJ10, INS, IMPDH1, IL3, IL11RA, HSPA9, GSTM2, GSK3B, FN1, F3, ERCC1, EPHA2, ENG, DISC1, DIDO1, CTSD, CTGF, CLIC5, CKB, CES2, CEACAM1, CD55, CARD9, CALM2, BMP4, BAT2, ATM, AIF1, AHRR, AANAT, ZNF148, XPA, XBP1, UGT1A7, UGT1A3, TREM1, TNXB, TNIP1, TNFRSF6B, TNFRSF14, TCP1, TAS2R38, TAS1R3, SULT1A1, SPINK5, SLC9A3R1, SLC22A11, SLC12A3, S100A2, RGS2, RECQL4, PRTN3, POU5F1, PLXNA2, PLA2G2A, PINK1, PEMT, PCDH21, PADI4, OXT, OBFC1, NKX2-5, NFKB2, MYH7, MMP26, MEIS2, MATN3, MAP2K5, LNX1, LHB, KIAA1217, ITGB7, IL2RG, IL21R, IFNA2, HSPA4, HOXA10, HN1L, HMGCR, HCRT, HADHA, GBA, GATA2, GABRA5, FGFR3, FGA, FBXO11, ENO1, ELA2, EGR2, ECE1, DDR1, DBP, CYFIP1, CXCR1, CRYAA, COL7A1, COL18A1, CNTF, CHRNA4, CHKB, CEBPA, CCL1, CAPN10, CALHM1, CACNA1S, BLMH, BCR, BAK1, BAG1, APOA4, AKR1B1, AIPL1, ACSL5, ACHE, ABCB6, ABCB4, ABCA7, ZP1, ZNF313, WNT10B, VTN, UGT1A8, UGT1A4, UGT1A10, UCHL1, TYMP, TSHR, TRAF2, TNR, TICAM2, THSD7A, TEF, TBX5, TAC3, SOCS1, SLIT2, SLC29A1, SLC26A2, SGCE, SEMA7A, SDC4, SCNN1G, SAA2, REN, RCBTB1, PTH2R, PTGDR, PTCH1, PRPF31, PIK3R1, PC, OPRD1, OPN1LW, NYX, NPS, NPPC, NPM1, NMB, NLRP2, NEUROD1, NEFM, NEF3, NAT9, MYO9B, MLL, MGST2, MFAP2, MCPH1, MCHR1, MATN1, MAP3K8, MADCAM1, LRAT, LIMK1, LBXCOR1, KLF12, KIF3A, KEAP1, KCNN3, KCNJ1, KCNA5, ITPR3, ITGAE, ITGA3, IMPDH2, IL20, IL13RA1, IGFBP3, HSPD1, HSPA8, HSPA5, HS3ST3A1, HRH2, HMHB1, GRM8, GRIK3, GPX4, GP9, GNAT2, GHRL, GH1, GFRA1, GABRB2, FRAP1, FPGS, FGG, FGFR1, FEZ1, FBLN5, FANCA, FADS1, ETV6, ERBB3, EIF4EBP1, DLST, DCN, DCLRE1C, CXCL16, CRABP2, COL12A1, CLCN7, CHST12, CHST11, CDKAL1, CD9, CCL7, CCL13, CBLB, CALM1, C4BPB, C1QTNF5, BTK, BGLAP, B2M, ATP1B2, ATP1B1, ATP1A1, ARHGEF3, APOC2, ALOXE3, ADAMTS13, ABCC1

Corresponding total number of TF hits:
(For each gene listed above, the total number of TF hits for any of the selected TFs, multiplied by the number of selected Phenopedia diseases containing that gene)
2310, 1200, 384, 366, 364, 298, 285, 268, 252, 204, 174, 170, 168, 160, 160, 158, 154, 152, 141, 117, 117, 85, 80, 78, 77, 70, 66, 64, 64, 64, 63, 60, 59, 54, 54, 52, 52, 51, 51, 51, 48, 48, 47, 44, 42, 38, 36, 35, 35, 30, 30, 30, 29, 28, 28, 27, 26, 25, 25, 24, 24, 24, 24, 24, 24, 24, 22, 22, 22, 22, 20, 20, 20, 19, 18, 18, 18, 18, 17, 17, 17, 16, 16, 16, 16, 16, 16, 16, 16, 14, 14, 14, 14, 14, 14, 13, 12, 12, 12, 12, 12, 12, 12, 12, 12, 12, 12, 12, 11, 11, 11, 11, 11, 10, 10, 10, 10, 10, 10, 10, 10, 10, 10, 10, 9, 9, 9, 9, 9, 9, 9, 9, 9, 9, 9, 9, 9, 9, 9, 9, 8, 8, 8, 8, 8, 8, 8, 8, 8, 8, 8, 8, 8, 8, 7, 7, 7, 7, 7, 7, 6, 6, 6, 6, 6, 6, 6, 6, 6, 6, 6, 6, 6, 6, 6, 6, 6, 6, 6, 6, 6, 6, 6, 6, 6, 6, 6, 6, 6, 6, 6, 6, 6, 5, 5, 5, 5, 5, 5, 5, 5, 5, 5, 5, 5, 5, 5, 5, 5, 5, 5, 5, 5, 5, 5, 4, 4, 4, 4, 4, 4, 4, 4, 4, 4, 4, 4, 4, 4, 4, 4, 4, 4, 4, 4, 4, 4, 4, 4, 4, 4, 4, 4, 4, 4, 4, 3, 3, 3, 3, 3, 3, 3, 3, 3, 3, 3, 3, 3, 3, 3, 3, 3, 3, 3, 3, 3, 3, 3, 3, 3, 3, 3, 3, 3, 3, 3, 3, 3, 3, 3, 3, 3, 3, 3, 3, 3, 3, 3, 3, 3, 3, 3, 3, 3, 3, 2, 2, 2, 2, 2, 2, 2, 2, 2, 2, 2, 2, 2, 2, 2, 2, 2, 2, 2, 2, 2, 2, 2, 2, 2, 2, 2, 2, 2, 2, 2, 2, 2, 2, 2, 2, 2, 2, 2, 2, 2, 2, 2, 2, 2, 2, 2, 2, 2, 2, 2, 2, 2, 2, 2, 2, 2, 2, 2, 2, 2, 2, 2, 2, 2, 2, 2, 2, 2, 2, 2, 2, 2, 2, 2, 2, 2, 2, 2, 2, 2, 2, 2, 2, 2, 2, 2, 2, 2, 2, 2, 2, 2, 2, 1

Corresponding number of selected Phenopedia diseases each gene is involved with:
(In the same order as above)
231, 120, 96, 183, 182, 149, 95, 134, 63, 68, 58, 85, 42, 80, 160, 79, 154, 38, 141, 39, 13, 17, 80, 78, 77, 35, 66, 64, 64, 16, 63, 12, 59, 27, 54, 52, 52, 51, 17, 51, 48, 8, 47, 22, 21, 19, 36, 35, 35, 30, 15, 30, 29, 7, 28, 9, 13, 5, 25, 12, 12, 12, 12, 12, 4, 6, 11, 11, 22, 22, 20, 5, 10, 19, 6, 6, 6, 9, 17, 17, 17, 8, 4, 8, 16, 16, 8, 16, 8, 8, 14, 7, 7, 14, 14, 7, 13, 6, 6, 12, 6, 12, 12, 6, 4, 6, 6, 12, 6, 11, 11, 11, 11, 11, 5, 2, 5, 5, 10, 10, 5, 10, 10, 10, 10, 2, 3, 3, 9, 9, 3, 9, 9, 3, 9, 9, 9, 3, 3, 3, 9, 9, 8, 8, 2, 2, 2, 4, 8, 8, 2, 4, 4, 4, 4, 4, 7, 7, 7, 7,

7, 7, 6, 2, 2, 1, 3, 1, 6, 2, 2, 6, 6, 6, 6, 2, 2, 3, 3, 6, 6, 6, 6, 2, 3, 2, 3, 6, 3, 6, 3, 6, 2, 6, 5, 5, 1, 5, 5, 5, 5, 5, 5, 5, 5, 1, 5, 5, 5, 5, 5, 5, 1, 5, 5, 2, 4, 2, 4, 2, 4, 2, 2, 2, 2, 1, 2, 2, 4, 2, 1, 2, 4, 4, 2, 4, 4, 2, 2, 4, 1, 4, 4, 4, 2, 2, 3, 3, 1, 3, 3, 3, 3, 1, 3, 3, 1, 1, 3, 3, 3, 3, 1, 3, 3, 3, 1, 3, 3, 3, 1, 1, 3, 3, 1, 3, 1, 3, 3, 3, 1, 3, 3, 3, 1, 3, 3, 1, 1, 3, 1, 1, 3, 3, 3, 3, 3, 1, 1, 1, 1, 2, 2, 2, 2, 1, 1, 1, 1, 2, 2, 1, 2, 2, 1, 2, 2, 1, 2, 1, 1, 1, 1, 2, 2, 2, 1, 2, 2, 2, 2, 1, 2, 2, 2, 2, 2, 2, 2, 2, 1, 1, 2, 2, 2, 1, 2, 1, 2, 2, 2, 2, 2, 2, 2, 1, 1, 1, 1, 2, 1, 2, 1, 2, 1, 2, 1, 2, 1, 2, 2, 2, 1, 2, 2, 2, 1, 2, 2, 2, 2, 1, 1, 2, 1, 2, 2, 1

Corresponding number of selected TFs each gene is involved with:
(In the same order as above)
10, 10, 4, 2, 2, 2, 3, 2, 4, 3, 3, 2, 4, 2, 1, 2, 1, 4, 1, 3, 9, 5, 1, 1, 1, 2, 1, 1, 1, 4, 1, 5, 1, 2, 1, 1, 1, 1, 3, 1, 1, 6, 1, 2, 2, 2, 1, 1, 1, 1, 2, 1, 1, 4, 1, 3, 2, 5, 1, 2, 2, 2, 2, 2, 6, 4, 2, 2, 1, 1, 1, 4, 2, 1, 3, 3, 3, 2, 1, 1, 1, 2, 4, 2, 1, 1, 2, 1, 2, 2, 1, 2, 2, 1, 1, 2, 1, 2, 2, 1, 2, 1, 1, 2, 3, 2, 2, 1, 2, 1, 1, 1, 1, 1, 2, 5, 2, 2, 1, 1, 2, 1, 1, 1, 1, 5, 3, 3, 1, 1, 3, 1, 1, 3, 1, 1, 1, 3, 3, 3, 1, 1, 1, 1, 4, 4, 4, 2, 1, 1, 4, 2, 2, 2, 2, 2, 1, 1, 1, 1, 1, 1, 1, 3, 3, 6, 2, 6, 1, 3, 3, 1, 1, 1, 1, 3, 3, 2, 2, 1, 1, 1, 1, 3, 2, 3, 2, 1, 2, 1, 2, 1, 3, 1, 1, 1, 5, 1, 1, 1, 1, 1, 1, 1, 1, 5, 1, 1, 1, 1, 1, 1, 5, 1, 1, 2, 1, 2, 1, 2, 1, 2, 2, 2, 2, 4, 2, 2, 1, 2, 4, 2, 1, 1, 2, 1, 1, 2, 2, 1, 4, 1, 1, 1, 2, 2, 1, 1, 3, 1, 1, 1, 1, 3, 1, 1, 3, 3, 1, 1, 1, 1, 3, 1, 1, 1, 3, 1, 1, 1, 3, 3, 1, 1, 3, 1, 3, 1, 1, 1, 3, 1, 1, 1, 3, 1, 1, 3, 3, 1, 3, 3, 1, 1, 1, 1, 1, 3, 3, 2, 2, 1, 1, 1, 1, 2, 2, 2, 2, 1, 1, 2, 1, 1, 2, 1, 1, 2, 1, 2, 2, 2, 2, 1, 1, 1, 2, 1, 1, 1, 1, 2, 1, 1, 1, 1, 1, 1, 1, 1, 2, 2, 1, 1, 1, 2, 1, 2, 1, 1, 1, 1, 1, 1, 2, 2, 2, 2, 1, 2, 1, 2, 1, 2, 1, 2, 1, 2, 1, 1, 1, 2, 1, 1, 1, 2, 1, 1, 1, 1, 2, 2, 1, 2, 1

Phenopedia diseases ranked according to mean of effect sizes in cluster:
Respiratory Distress Syndrome, Adult | Liver Diseases, Alcoholic | Liver Cirrhosis, Alcoholic | Constriction, Pathologic | Cerebral Palsy | Brain Damage, Chronic | Critical Illness | Alcohol-Induced Disorders | Duodenal Ulcer | Opportunistic Infections | Duodenal Diseases | Peptic Ulcer | Pancreatitis | Drug Eruptions | Pneumoconiosis | Respiration Disorders | Malaria, Falciparum | Iron Overload | Lung Diseases, Fungal | Gastritis, Atrophic | Delayed Graft Function | Eclampsia | Migraine without Aura | Coronary Thrombosis | Meningitis | Pancreatitis, Alcoholic | Parasitemia | alpha 1-Antitrypsin Deficiency | Fetal Death | Purpura, Thrombocytopenic | Decision Making | Pneumonia | Uremia | Herpesviridae Infections | Abortion, Habitual | Ulcer | HELLP Syndrome | Migraine with Aura | Liver Failure | Retinal Vein Occlusion | Hyperuricemia | Gingival Diseases | Purpura, Thrombocytopenic, Idiopathic | Chagas Cardiomyopathy | Multiple Organ Failure | Postoperative Complications | Respiratory Sounds | Purpura, Schoenlein-Henoch | Gallbladder Neoplasms | Shock | Periodontitis, Juvenile | Lipodystrophy | Hemolysis | Pulmonary Emphysema | Sarcoma, Kaposi | Vasculitis, Hypersensitivity | Cytomegalovirus Infections | Immune Complex Diseases | Albuminuria | Chagas Disease | Acute Disease | Mycoses | Meningococcal Infections | Pregnancy Complications, Cardiovascular | Stomatitis | Rupture | Stomach Ulcer | Nephrosis | Drug-Induced Liver Injury | Nephrotic Syndrome | Cadaver | Neuralgia | Exfoliation Syndrome | Abortion, Spontaneous | Iron Metabolism Disorders | Bone Resorption | Mucocutaneous Lymph Node Syndrome | Reperfusion Injury | Shock, Septic | Limb Deformities, Congenital | Bronchopulmonary Dysplasia | Headache | Skin Diseases, Vesiculobullous | Aspergillosis | Oral Submucous Fibrosis | Blindness | Gastritis | Epstein-Barr Virus Infections | Puerperal Disorders | Photosensitivity Disorders | Arthralgia | Eye Infections, Bacterial | Migraine Disorders | Severe Acute Respiratory Syndrome | Trachoma | Cough | Chlamydia Infections | Exanthema | Lichen Planus | Conjunctival Diseases | Drug Hypersensitivity | Sleep Apnea Syndromes | Helicobacter Infections | Hematologic Neoplasms | Sarcoidosis, Pulmonary | Gram-Negative Bacterial Infections | Coronary Stenosis | Emphysema | Corneal Diseases | Conjunctivitis | Myelitis | Papilloma | Leptospirosis | Uveitis | Panuveitis | HTLV-I Infections | Sarcoidosis | Intracranial Arteriosclerosis | Pain, Postoperative | Malaria, Cerebral | Pancreatitis, Chronic | Aneurysm, Ruptured | Sleep Apnea, Obstructive | Stevens-Johnson Syndrome | Epidermal Necrolysis, Toxic | Salmonella Infections | Vaginosis, Bacterial | Vaginal Diseases | Antiphospholipid Syndrome | Infertility, Female | Bacteremia | Urinary Tract Infections | Uveitis, Anterior | Leprosy | Sensation | Behcet Syndrome | Dyspepsia | Lichen Planus, Oral | Respiratory Distress Syndrome, Newborn | Glomerulosclerosis, Focal

Segmental | Narcolepsy | Picornaviridae Infections | Deltaretrovirus Infections | Pemphigus | Abdominal Pain | Osteoporosis, Postmenopausal | Leukocytosis | Tuberculosis | Thymoma | Coronary Aneurysm | Alveolar Bone Loss | Pregnancy Complications, Infectious | Malaria | Neoplasms, Experimental | Colonic Diseases, Functional | Familial Mediterranean Fever | Silicosis | Constipation | Vesico-Ureteral Reflux | Fatigue Syndrome, Chronic | Encephalitis | Sciatica | Coronary Restenosis | Erythema Nodosum | Metal Metabolism, Inborn Errors | Parasitic Diseases | Graft vs Host Disease | Sepsis | Food Hypersensitivity | Autonomic Nervous System Diseases | Disorders of Excessive Somnolence | Bronchiectasis | Viremia | Gout | Irritable Bowel Syndrome | Rheumatic Fever | Periodontal Attachment Loss | Otitis Media | Hypotension | Idiopathic Pulmonary Fibrosis | Rheumatic Heart Disease | Mycobacterium Infections, Atypical | Nasal Polyps | Apnea | Periodontal Pocket | Systemic Inflammatory Response Syndrome | Mumps | Weight Loss | Temporal Arteritis | Multiple System Atrophy | Arthritis, Psoriatic | Tuberculosis, Pulmonary | Endometriosis | Ileitis | Erythema | Scleroderma, Systemic | Retinopathy of Prematurity | Paranasal Sinus Diseases | Arteritis | Urticaria | Streptococcal Infections | Sinusitis | Helminthiasis | Mycobacterium Infections | Pulmonary Fibrosis | Encephalomyelitis | Brucellosis | Dementia, Vascular | Measles | Polymyalgia Rheumatica | Leukemia, T-Cell, Acute | Ileal Diseases | Enterocolitis | Bacterial Infections | Salivary Gland Diseases | Mitral Valve Prolapse | Intervertebral Disk Displacement | Vasculitis | Prosthesis Failure | Spondylarthritis | Spondylarthropathies | Ventricular Dysfunction | Ankylosis | Hepatitis, Autoimmune | Psoriasis | Spondylitis, Ankylosing | Sjogren's Syndrome | Rubella | Ventricular Dysfunction, Left | Spinal Diseases | Spondylitis | Eosinophilia

Corresponding mean of effect sizes of each Phenopedia disease:
(In the same order as above)
2.13, 1.95, 1.92, 1.91, 1.90, 1.90, 1.88, 1.84, 1.82, 1.79, 1.77, 1.77, 1.69, 1.66, 1.61, 1.59, 1.57, 1.56, 1.56, 1.55, 1.53, 1.53, 1.52, 1.52, 1.52, 1.51, 1.50, 1.50, 1.48, 1.48, 1.46, 1.45, 1.45, 1.42, 1.42, 1.42, 1.41, 1.40, 1.37, 1.37, 1.37, 1.36, 1.36, 1.36, 1.34, 1.33, 1.32, 1.31, 1.31, 1.31, 1.31, 1.30, 1.30, 1.30, 1.29, 1.29, 1.29, 1.29, 1.28, 1.27, 1.25, 1.25, 1.24, 1.24, 1.23, 1.22, 1.21, 1.21, 1.20, 1.20, 1.19, 1.18, 1.18, 1.18, 1.17, 1.17, 1.17, 1.17, 1.17, 1.16, 1.16, 1.16, 1.15, 1.15, 1.15, 1.15, 1.15, 1.14, 1.14, 1.14, 1.13, 1.12, 1.12, 1.11, 1.11, 1.10, 1.09, 1.08, 1.07, 1.07, 1.06, 1.06, 1.06, 1.05, 1.05, 1.04, 1.04, 1.04, 1.04, 1.03, 1.03, 1.03, 1.01, 1.00, 1.00, 1.00, 0.99, 0.99, 0.99, 0.98, 0.98, 0.98, 0.97, 0.97, 0.97, 0.97, 0.97, 0.97, 0.96, 0.96, 0.95, 0.94, 0.94, 0.94, 0.94, 0.94, 0.94, 0.93, 0.93, 0.92, 0.91, 0.91, 0.91, 0.91, 0.90, 0.89, 0.89, 0.89, 0.88, 0.87, 0.87, 0.86, 0.86, 0.85, 0.85, 0.85, 0.84, 0.84, 0.83, 0.83, 0.82, 0.82, 0.82, 0.81, 0.81, 0.80, 0.80, 0.79, 0.78, 0.78, 0.78, 0.77, 0.75, 0.74, 0.73, 0.73, 0.73, 0.72, 0.72, 0.71, 0.71, 0.70, 0.70, 0.70, 0.69, 0.67, 0.65, 0.65, 0.64, 0.64, 0.63, 0.63, 0.62, 0.60, 0.60, 0.59, 0.55, 0.55, 0.55, 0.55, 0.54, 0.54, 0.51, 0.50, 0.49, 0.48, 0.47, 0.46, 0.45, 0.43, 0.41, 0.40, 0.40, 0.39, 0.36, 0.36, 0.35, 0.34, 0.31, 0.23, 0.22, 0.21, 0.19, 0.18, 0.17, 0.16, 0.14, 0.08, 0.07, 0.06, -0.01, -0.18

Corresponding total number of genes of each Phenopedia disease:
(In the same order as above)
30, 56, 46, 66, 40, 39, 28, 94, 50, 38, 65, 72, 97, 52, 50, 81, 68, 49, 23, 47, 40, 81, 116, 29, 37, 39, 29, 21, 33, 31, 93, 80, 52, 62, 82, 84, 28, 127, 23, 22, 43, 32, 24, 22, 20, 127, 31, 33, 31, 112, 44, 23, 27, 23, 18, 34, 34, 35, 111, 36, 199, 46, 38, 30, 36, 79, 32, 39, 25, 43, 70, 32, 23, 98, 50, 72, 61, 21, 26, 22, 34, 86, 53, 22, 24, 151, 107, 25, 37, 23, 21, 39, 145, 37, 41, 29, 48, 20, 28, 51, 84, 23, 120, 57, 46, 197, 113, 59, 51, 47, 22, 32, 25, 110, 102, 29, 100, 69, 26, 39, 32, 27, 21, 21, 20, 25, 30, 32, 33, 45, 37, 32, 99, 50, 143, 95, 41, 24, 22, 22, 24, 20, 30, 34, 39, 88, 24, 159, 25, 21, 42, 44, 139, 41, 23, 32, 20, 32, 21, 43, 42, 31, 73, 38, 49, 144, 81, 185, 24, 22, 26, 36, 58, 34, 30, 30, 35, 26, 39, 36, 27, 22, 25, 41, 30, 149, 32, 89, 38, 32, 55, 93, 105, 19, 67, 84, 22, 28, 47, 38, 49, 27, 34, 142, 61, 73, 21, 40, 46, 27, 25, 22, 19, 300, 61, 20, 43, 210, 27, 111, 106, 62, 73, 22, 155, 72, 45, 48, 49, 176, 99, 52

Corresponding number of genes of each Phenopedia disease with at least one TF hit (of selected TFs):
(In the same order as above)
17, 28, 26, 29, 18, 18, 15, 38, 20, 18, 29, 30, 48, 21, 24, 35, 24, 22, 10, 23, 22, 34, 39, 11, 18, 17, 13, 9, 16, 15, 36, 31, 18, 24, 34, 34, 16, 44, 11, 10, 20, 12, 11, 10, 10, 51, 11, 20, 17, 53, 18, 14, 14, 11, 9, 20, 13, 20, 44, 17, 72, 16, 13, 14, 16, 33, 13, 19, 10, 20, 31, 12, 9, 38, 23, 31, 27, 10, 13, 12, 14, 32, 21, 8, 12, 53, 43, 15, 15, 12, 11, 19, 45, 11, 19, 13, 21, 10, 11, 24, 32, 14, 45, 26, 16, 73, 39, 21, 21, 21, 13, 16, 10, 44, 41, 17, 36, 32, 9, 12, 14, 11, 13, 8, 8, 12, 8, 10, 11, 21, 16, 12, 40, 13, 57, 39, 13, 8, 9, 10, 17, 8, 18,

15, 18, 38, 10, 57, 13, 9, 16, 16, 52, 18, 9, 15, 13, 13, 11, 16, 16, 12, 28, 18, 23, 58, 29, 68, 10, 12, 18, 13, 23, 13, 10, 13, 13, 8, 22, 16, 12, 9, 10, 24, 11, 57, 17, 38, 17, 14, 26, 33, 42, 8, 27, 32, 11, 9, 20, 15, 22, 8, 12, 43, 25, 28, 11, 18, 20, 10, 12, 6, 8, 99, 27, 10, 13, 87, 8, 44, 43, 23, 32, 11, 63, 31, 16, 20, 15, 61, 42, 19

Corresponding total number of TF hits (of selected TFs) for each Phenopedia disease (in all genes):
(In the same order as above)
51, 55, 53, 68, 47, 47, 43, 67, 54, 48, 64, 67, 90, 54, 56, 71, 62, 50, 36, 54, 49, 75, 85, 35, 36, 33, 37, 29, 33, 40, 71, 66, 45, 51, 75, 74, 43, 96, 38, 24, 44, 35, 34, 35, 37, 105, 37, 41, 40, 110, 44, 43, 37, 37, 21, 41, 30, 41, 94, 45, 131, 43, 39, 36, 40, 72, 38, 37, 25, 39, 63, 30, 28, 84, 49, 71, 54, 26, 39, 35, 44, 71, 49, 31, 35, 108, 91, 35, 33, 33, 37, 54, 99, 27, 54, 27, 56, 27, 23, 62, 72, 32, 94, 53, 43, 144, 77, 53, 57, 57, 41, 40, 24, 94, 88, 46, 79, 79, 22, 30, 27, 32, 30, 23, 23, 34, 33, 35, 26, 42, 45, 35, 86, 36, 103, 85, 30, 20, 25, 26, 32, 21, 47, 39, 44, 77, 23, 114, 40, 23, 33, 37, 114, 39, 22, 30, 28, 27, 25, 30, 36, 28, 59, 44, 48, 123, 72, 130, 25, 31, 33, 33, 47, 32, 24, 30, 29, 21, 49, 42, 29, 24, 25, 48, 25, 115, 35, 74, 37, 31, 58, 65, 95, 25, 60, 69, 30, 31, 41, 29, 46, 30, 34, 83, 56, 45, 27, 44, 43, 22, 41, 21, 24, 189, 48, 17, 29, 162, 24, 86, 85, 43, 60, 25, 122, 59, 33, 45, 31, 110, 79, 36

Corresponding number of selected TFs each Phenopedia disease is involved with:
(In the same order as above)
10, 9, 10, 10, 10, 10, 10, 10, 10, 10, 10, 10, 10, 10, 10, 10, 10, 10

TFs ranked according to mean of effect sizes in cluster:
V$NFE2_01, V$ER_Q6, V$NRF2_Q4, V$SRF_Q5_01, F$STRE_01, V$FREAC3_01, V$SMAD4_Q6, V$TATA_01, V$PPARG_03, V$HOXA7_01

Corresponding mean of effect sizes of each TF:
(In the same order as above)
1.28, 1.25, 1.21, 1.01, 0.97, 0.95, 0.92, 0.91, 0.81, 0.70

Corresponding total number of TF hits for each TF (genome-wide):
(In the same order as above)
471, 409, 470, 479, 425, 521, 445, 535, 473, 455

Corresponding total number of TF hits for each TF (in all genes in selected Phenopedia diseases):
(In the same order as above)
1322, 1244, 1243, 1128, 972, 1158, 1012, 1283, 1154, 1129

Corresponding number of genes (of selected Phenopedia diseases) each TF is involved with:
(In the same order as above)
93, 86, 85, 68, 73, 86, 78, 96, 93, 72

Corresponding number of selected Phenopedia diseases each TF is involved with:
(In the same order as above)
232, 232, 232, 231, 232, 232, 232, 232, 232, 232

Disease groups of selected Phenopedia diseases ranked according to sum of effect sizes
Signs and Symptoms | Communicable Diseases | Respiratory Tract Diseases | Cardiovascular Diseases | Digestive System Diseases | Thoracic Diseases | Vascular Diseases | Nervous System Diseases | Bacterial

Infections | Skin Diseases | Central Nervous System Diseases | Lung Diseases | Musculoskeletal Diseases | Gastrointestinal Diseases | Brain Diseases | Female Urogenital Diseases | Eye Diseases | Immune System Diseases | Neurologic Manifestations | Virus Diseases | Intestinal Diseases | Male Urogenital Diseases | Pneumonia | Gram-Negative Bacterial Infections | Connective Tissue Diseases | Mediastinal Diseases | Urologic Diseases | Exanthema | Uveal Diseases | Substance-Related Disorders | Alcohol-Related Disorders | Alcohol-Induced Disorders | Respiration Disorders | Vasculitis | Stomatognathic Diseases | Mouth Diseases | Jaw Diseases | Gram-Positive Bacterial Infections | Infection | Stomach Diseases | Neoplasms | Metabolic Diseases | Pregnancy Complications | Uveitis | Endophthalmitis | Protozoan Infections | Pulmonary Fibrosis | Lung Diseases, Interstitial | Hypersensitivity | Blood Coagulation Disorders | Bone Diseases | Liver Diseases | Duodenal Diseases | Parasitic Diseases | Arteriosclerosis | Arterial Occlusive Diseases | Peripheral Nervous System Diseases | Abdominal Pain | Dermatitis | Congenital, Hereditary, and Neonatal Diseases and Abnormalities | Peripheral Vascular Diseases | Kidney Diseases | Systemic Inflammatory Response Syndrome | Sepsis | Autoimmune Diseases | Coronary Artery Disease | Thrombocytopenia | Blood Platelet Disorders | Pancreatitis | Pancreatic Diseases | Purpura | Hemorrhage | Vascular Headaches | Migraine Disorders | Headache Disorders | Mycoses | Liver Diseases, Alcoholic | Herpesviridae Infections | Shock | Genetic Diseases, Inborn | Gastritis | Mycobacterium Infections | Actinomycetales Infections | Genital Diseases, Female | Malaria | Coccidiosis | Mucositis | Inflammation | Erythema | Hypersensitivity, Immediate | Conjunctival Diseases | Purpura, Thrombocytopenic | Colonic Diseases | Movement Disorders | Basal Ganglia Diseases | Polyps | Iron Metabolism Disorders | Apnea | Poisoning | Drug Toxicity | Otorhinolaryngologic Diseases | Trypanosomiasis | Sarcomastigophora Infections | Mastigophora Infections | Chagas Disease | Vasculitis, Hypersensitivity | Abortion, Spontaneous | Spinal Diseases | Respiratory Hypersensitivity | Pneumoconiosis | Alveolitis, Extrinsic Allergic | Autonomic Nervous System Diseases | Eye Infections, Bacterial | Eye Infections | Chlamydia Infections | Rupture | Conjunctivitis | Respiratory Distress Syndrome, Adult | Pulmonary Edema | Lung Injury | Acute Lung Injury | Hyperuricemia | Skin Diseases, Vascular | Cardiomyopathies | Skin Diseases, Vesiculobullous | Gingivitis | Gingival Overgrowth | Gingival Diseases | Bone Resorption | Sex Chromosome Disorders | Sarcoidosis | Lymphoproliferative Disorders | Genetic Diseases, X-Linked | Sleep Apnea, Obstructive | Sleep Apnea, Central | Sleep Apnea Syndromes | Lichenoid Eruptions | Lichen Planus | Streptococcal Infections | Hematologic Neoplasms | Vaginal Diseases | Liver Cirrhosis, Alcoholic | Liver Cirrhosis | Pathological Conditions, Anatomical | Constriction, Pathologic | Retroviridae Infections | Deltaretrovirus Infections | Cerebral Palsy | Brain Damage, Chronic | Disease Attributes | Critical Illness | Uveitis, Anterior | Coronary Stenosis | Coronary Occlusion | Duodenal Ulcer | Arthritis | Nose Diseases | Opportunistic Infections | Peptic Ulcer | Sleep Disorders | Dyssomnias | Disorders of Excessive Somnolence | Drug Eruptions | Periodontal Diseases | Periodontal Atrophy | Chronic Periodontitis | Malaria, Falciparum | Iron Overload | Respiratory Tract Infections | Lung Diseases, Fungal | Gastritis, Atrophic | Delayed Graft Function | Hypertension, Pregnancy-Induced | Hypertension | Eclampsia | Migraine without Aura | Coronary Thrombosis | Meningitis | Central Nervous System Infections | Tuberculosis | Pancreatitis, Alcoholic | Spinal Cord Diseases | Myelitis | Parasitemia | alpha 1-Antitrypsin Deficiency | Fetal Death | Decision Making | Uremia | Rheumatic Fever | Rheumatic Diseases | Abortion, Habitual | Ulcer | HELLP Syndrome | Migraine with Aura | Liver Failure | Venous Thrombosis | Thrombophlebitis | Retinal Vein Occlusion | Phlebitis | Purpura, Thrombocytopenic, Idiopathic | Chagas Cardiomyopathy | Multiple Organ Failure | Postoperative Complications | Respiratory Sounds | Purpura, Schoenlein-Henoch | Hemostatic Disorders | Gallbladder Neoplasms | Gallbladder Diseases | Biliary Tract Neoplasms | Biliary Tract Diseases | Abdominal Neoplasms | Periodontitis, Juvenile | Skin Diseases, Metabolic | Lipodystrophy | Lipid Metabolism Disorders | Encephalitis | Hemolysis | Pulmonary Emphysema | Pulmonary Disease, Chronic Obstructive | Lung Diseases, Obstructive | Sarcoma, Kaposi | Cytomegalovirus Infections | Immune Complex Diseases | Urination Disorders | Proteinuria | Albuminuria | Acute Disease | Neisseriaceae Infections | Meningococcal Infections | Pregnancy Complications, Cardiovascular | Stomatitis | Stomach Ulcer | Nephrosis | Drug-Induced Liver Injury | Nephrotic Syndrome | Cadaver | Sensation Disorders | Neuralgia | Myokymia | Facial Nerve Diseases | Cranial Nerve Diseases | Iris Diseases | Exfoliation Syndrome | Mucocutaneous Lymph Node Syndrome | Lymphatic Diseases | Reperfusion Injury | Staphylococcal Infections | Soft Tissue Infections | Shock, Septic | Endotoxemia | Cardiovascular Infections | Limb Deformities, Congenital | Collagen Diseases | Bronchopulmonary Dysplasia | Headache | Aspergillosis | Oral Submucous Fibrosis | Blindness | Epstein-Barr Virus Infections | Puerperal Disorders | Radiodermatitis | Photosensitivity Disorders | Arthralgia | Severe Acute Respiratory Syndrome |

Coronavirus Infections | Coronaviridae Infections | Trachoma | Conjunctivitis, Inclusion | Conjunctivitis, Bacterial | Paramyxoviridae Infections | Cough | Paranasal Sinus Diseases | Drug Hypersensitivity | Helicobacter Infections | Sarcoidosis, Pulmonary | Emphysema | Corneal Diseases | Papilloma | Neoplasms, Squamous Cell | Neoplasms, Glandular and Epithelial | Salivary Gland Diseases | Spirochaetales Infections | Leptospirosis | Panuveitis | HTLV-I Infections | Gastroenteritis | Enteritis | Intracranial Arteriosclerosis | Cerebrovascular Disorders | Pain, Postoperative | Malaria, Cerebral | Pancreatitis, Chronic | Aneurysm, Ruptured | Aneurysm | Stevens-Johnson Syndrome | Erythema Multiforme | Epidermal Necrolysis, Toxic | Salmonella Infections | Enterobacteriaceae Infections | Vaginosis, Bacterial | Vaginitis | Antiphospholipid Syndrome | Infertility, Female | Infertility | Genital Diseases, Male | Bacteremia | Urinary Tract Infections | Leprosy | Sensation | Behcet Syndrome | Dyspepsia | Lichen Planus, Oral | Respiratory Distress Syndrome, Newborn | Nephritis | Glomerulosclerosis, Focal Segmental | Glomerulonephritis | Tremor | Pharyngeal Diseases | Parasomnias | Nystagmus, Pathologic | Neuromuscular Junction Diseases | Neuromuscular Diseases | Nasopharyngeal Diseases | Narcolepsy | Myoclonus | Muscular Diseases | Infant, Newborn, Diseases | Dyskinesias | Picornaviridae Infections | Pemphigus | Reflex Sympathetic Dystrophy | Osteoporosis, Postmenopausal | Osteoporosis | Complex Regional Pain Syndromes | Leukocytosis | Leukocyte Disorders | Thymus Neoplasms | Thymoma | Thoracic Neoplasms | Neoplasms, Complex and Mixed | Mediastinal Neoplasms | Coronary Aneurysm | Alveolar Bone Loss | Pregnancy Complications, Infectious | Neoplasms, Experimental | Colonic Diseases, Functional | Familial Mediterranean Fever | Silicosis | Constipation | Vesico-Ureteral Reflux | Urinary Bladder Diseases | Fatigue Syndrome, Chronic | Sciatica | Sciatic Neuropathy | Neuritis | Mononeuropathies | Coronary Restenosis | Erythema Nodosum | Metal Metabolism, Inborn Errors | Metabolism, Inborn Errors | Skin Diseases, Papulosquamous | Psoriasis | Graft vs Host Disease | Food Hypersensitivity | Bronchiectasis | Bronchial Diseases | Viremia | Gout | Irritable Bowel Syndrome | Periodontal Attachment Loss | Otitis Media | Otitis | Hearing Disorders | Ear Diseases | Hypotension | Idiopathic Pulmonary Fibrosis | Rheumatic Heart Disease | Myocarditis | Mediastinitis | Mycobacterium Infections, Atypical | Nasal Polyps | Periodontal Pocket | Sialadenitis | Rubulavirus Infections | Parotitis | Mumps | Weight Loss | Body Weight Changes | Temporal Arteritis | Neurodegenerative Diseases | Multiple System Atrophy | Arthritis, Psoriatic | Tuberculosis, Pulmonary | Uterine Diseases | Ovarian Diseases | Ovarian Cysts | Endometriosis | Cysts | Ileitis | Spondylarthropathies | Scleroderma, Systemic | Scleroderma, Localized | Retinopathy of Prematurity | Retinal Diseases | Arteritis | Urticaria | Edema | Sinusitis | Encephalomyelitis | Brucellosis | Necrosis | Infarction | Dementia, Vascular | Dementia | Cerebral Infarction | Brain Infarction | Morbillivirus Infections | Measles | Polymyalgia Rheumatica | Leukemia, T-Cell, Acute | Ileal Diseases | Enterocolitis | Colitis | Spondylarthritis | Mitral Valve Prolapse | Heart Valve Prolapse | Heart Valve Diseases | Intervertebral Disk Displacement | Helminthiasis | Prosthesis Failure | Ventricular Dysfunction | Ankylosis | Hepatitis, Autoimmune | Hepatitis | Spondylitis, Ankylosing | Spondylitis | Osteitis | Sjogren's Syndrome | Lacrimal Apparatus Diseases | Dacryocystitis | Togaviridae Infections | Rubella | Ventricular Dysfunction, Left | Pulmonary Eosinophilia | Nematode Infections | Filariasis | Eosinophilia

Corresponding sum of effect sizes for each disease group of selected Phenopedia diseases
(In the same order as above)
988.71, 423.46, 310.08, 272.28, 239.85, 237.90, 215.91, 191.43, 182.15, 154.98, 154.71, 151.73, 150.48, 130.79, 122.83, 113.45, 111.11, 110.00, 102.10, 97.22, 91.75, 87.95, 87.35, 81.42, 79.07, 78.43, 78.34, 76.24, 73.30, 72.16, 72.16, 72.16, 69.79, 69.69, 68.47, 68.47, 68.47, 67.97, 66.87, 66.67, 64.17, 62.98, 62.25, 61.52, 61.52, 60.32, 57.85, 57.85, 55.84, 55.63, 54.45, 54.23, 53.65, 52.52, 52.44, 52.44, 52.21, 49.44, 47.99, 47.85, 47.84, 47.74, 46.59, 46.59, 46.19, 42.55, 42.50, 42.50, 41.76, 41.76, 41.52, 41.52, 40.39, 40.39, 40.39, 39.59, 38.66, 38.61, 38.09, 36.96, 36.85, 36.52, 36.52, 35.10, 34.10, 34.10, 33.72, 33.72, 33.49, 32.33, 32.05, 28.39, 28.12, 28.12, 28.12, 27.48, 27.36, 27.29, 27.19, 27.19, 27.05, 26.23, 26.23, 26.23, 26.23, 26.05, 25.94, 25.13, 24.50, 24.50, 24.50, 23.05, 22.31, 22.31, 22.00, 21.93, 21.39, 21.31, 21.31, 21.31, 21.31, 21.10, 21.06, 20.63, 20.60, 20.49, 20.49, 20.49, 20.43, 20.39, 20.39, 20.39, 20.39, 20.33, 20.33, 20.33, 20.04, 20.04, 19.79, 19.36, 19.33, 19.18, 19.18, 19.09, 19.09, 19.02, 19.02, 19.01, 19.01, 18.80, 18.80, 18.78, 18.59, 18.59, 18.23, 18.15, 17.95, 17.89, 17.70, 16.87, 16.87, 16.87, 16.58, 15.97, 15.97, 15.97, 15.67, 15.62, 15.56, 15.56, 15.47, 15.35, 15.30, 15.30, 15.30, 15.22, 15.22, 15.19, 15.19, 15.14, 15.11, 15.09, 15.09, 15.00, 14.99, 14.84, 14.64, 14.46, 14.35, 14.35, 14.18, 14.17, 14.11, 13.96, 13.71, 13.66, 13.66, 13.66, 13.66, 13.59, 13.56, 13.36, 13.31, 13.19, 13.12, 13.12, 13.08, 13.08, 13.08, 13.08, 13.08, 13.07, 13.04, 13.04, 13.04, 13.03, 13.00, 12.96, 12.96, 12.96, 12.95, 12.92,

12.90, 12.80, 12.80, 12.80, 12.51, 12.41, 12.41, 12.40, 12.33, 12.12, 12.07, 12.02, 11.98, 11.94, 11.83, 11.83, 11.83, 11.83, 11.83, 11.77, 11.77, 11.70, 11.70, 11.69, 11.66, 11.66, 11.66, 11.66, 11.66, 11.62, 11.61, 11.60, 11.56, 11.53, 11.48, 11.46, 11.44, 11.40, 11.37, 11.37, 11.27, 11.11, 11.11, 11.11, 11.07, 11.07, 11.07, 11.02, 10.99, 10.96, 10.61, 10.56, 10.48, 10.37, 10.37, 10.27, 10.27, 10.27, 10.18, 10.11, 10.11, 9.96, 9.95, 9.92, 9.92, 9.89, 9.89, 9.86, 9.84, 9.77, 9.77, 9.77, 9.69, 9.69, 9.69, 9.69, 9.69, 9.67, 9.67, 9.62, 9.61, 9.61, 9.61, 9.52, 9.45, 9.40, 9.39, 9.36, 9.36, 9.33, 9.26, 9.22, 9.22, 9.22, 9.11, 9.11, 9.11, 9.11, 9.11, 9.11, 9.11, 9.11, 9.11, 9.11, 9.11, 9.11, 9.08, 9.06, 8.90, 8.90, 8.90, 8.90, 8.89, 8.89, 8.85, 8.85, 8.85, 8.85, 8.85, 8.75, 8.70, 8.61, 8.51, 8.47, 8.46, 8.41, 8.37, 8.35, 8.35, 8.32, 8.22, 8.22, 8.22, 8.22, 8.21, 8.14, 8.11, 8.11, 8.01, 8.01, 7.96, 7.83, 7.75, 7.75, 7.54, 7.44, 7.31, 7.28, 7.25, 7.25, 7.25, 7.25, 7.17, 7.10, 7.07, 7.07, 7.07, 7.01, 6.99, 6.88, 6.53, 6.53, 6.53, 6.53, 6.50, 6.50, 6.44, 6.39, 6.39, 6.32, 6.28, 6.17, 6.17, 6.17, 6.17, 6.17, 5.96, 5.96, 5.91, 5.91, 5.55, 5.55, 5.49, 5.46, 5.46, 5.43, 4.80, 4.68, 4.63, 4.63, 4.63, 4.63, 4.63, 4.63, 4.48, 4.48, 4.30, 4.15, 4.02, 3.96, 3.96, 3.80, 3.60, 3.60, 3.60, 3.47, 3.30, 3.14, 2.80, 1.94, 1.85, 1.85, 1.62, 1.55, 1.55, 1.40, 1.40, 1.40, 0.84, 0.84, 0.71, -1.80, -1.80, -1.80, -1.80

Cluster for columns 305 to 310, rows 420 to 422

Phenopedia diseases

Glomerulonephritis | Nephritis | Kidney Diseases | Kidney Failure | Kidney Failure, Chronic | Renal Insufficiency, Chronic

TFs

V$HNF1_Q6_01 | V$HNF1_Q6 | V$HNF1_01

Information

All related TFs:
(List of all TFs that are related to any of the PWMs)
HNF-1alpha, HNF-1alpha-A, HNF-1alpha-B, HNF-1alpha-C, HNF-1beta, HNF-1beta-A, HNF-1beta-B, HNF-1beta-C

Ranked gene list:
(All genes of the selected Phenopedia diseases with hits of any of the selected TFs, ranked according to the total number of TF hits)
PPARGC1A, LIPC, TGFBR2, TCF1, SPINK1, PIK3R1, IGFBP1, IFNG, HNF4A, HNF1A, DDAH2, CNDP2, APOH, ACSM3, NR3C1, FGA, C5, TMEM60, TCF2, MTRR, MTP18, HNF1B, FABP2, UGT1A7, TRDN, SERPINA1, SELL, MYH9, IRS1, GCLM, F5, ESR1, CYP3A5, CLCN5, NQO1, AQP2, TLR1, SOX2, SMAD3, SLC34A1, SGK, RFC1, PON2, PDE4D, MYC, IGFBP3, HSPA8, HIF1A, GCKR, FN1, DACH1, CPB2, APOA5, AGER, UGT1A1, SVIL, SMAD7, RUNX1, PAX2, IL12B, FCGRT, ELMO1, C1GALT1C1, BCL2, TSPAN8, PRKCQ, PKHD1, LCP1, GNAS, DNASE2, CYP2E1, CTNNB1, STAT3, SLCO1B1, SLC1A4, SEMA4G, RXRA, PLA2G4A, PCSK2, KLF12, IL21, HPS1, GFPT1, FAM13A, DPYD, CDKN2C, CDK6, CDH4, CD244, ATF6, ARHGEF12, ADRA1B, ADRA1A

Corresponding total number of TF hits:
(For each gene listed above, the total number of TF hits for any of the selected TFs, multiplied by the number of selected Phenopedia diseases containing that gene)
18, 18, 12, 12, 12, 12, 12, 12, 12, 12, 12, 12, 12, 12, 10, 9, 9, 8, 8, 8, 8, 8, 8, 6, 6, 6, 6, 6, 6, 6, 6, 6, 6, 6, 5, 5, 4, 4, 4, 4, 4, 4, 4, 4, 4, 4, 4, 4, 4, 4, 4, 4, 4, 4, 3, 3, 3, 3, 3, 3, 3, 3, 3, 3, 2, 2, 2, 2, 2, 2, 2, 2, 1, 1, 1, 1, 1, 1, 1, 1, 1, 1, 1, 1, 1, 1, 1, 1, 1, 1, 1, 1, 1

Corresponding number of selected Phenopedia diseases each gene is involved with:
(In the same order as above)

6, 6, 4, 4, 4, 4, 4, 6, 4, 4, 4, 4, 6, 6, 5, 3, 3, 4, 4, 4, 4, 4, 4, 2, 3, 3, 6, 6, 6, 6, 6, 6, 6, 2, 5, 5, 2, 4, 4, 4, 4, 4, 4, 4, 4, 4, 4, 4, 4, 2, 4, 4, 4, 4, 1, 1, 1, 1, 3, 3, 3, 1, 3, 1, 2, 1, 1, 1, 1, 2, 1, 1, 1, 1, 1, 1, 1, 1, 1, 1, 1, 1, 1, 1, 1, 1, 1, 1, 1, 1, 1, 1, 1

Corresponding number of selected TFs each gene is involved with:
(In the same order as above)
3, 3, 3, 3, 3, 3, 3, 2, 3, 3, 3, 3, 2, 2, 2, 3, 3, 2, 2, 2, 2, 2, 2, 3, 2, 2, 1, 1, 1, 1, 1, 1, 1, 3, 1, 1, 2, 1, 1, 1, 1, 1, 1, 1, 1, 1, 1, 1, 1, 2, 1, 1, 1, 1, 3, 3, 3, 3, 1, 1, 1, 3, 1, 3, 1, 2, 2, 2, 2, 1, 2, 2, 1, 1, 1, 1, 1, 1, 1, 1, 1, 1, 1, 1, 1, 1, 1, 1, 1, 1, 1, 1, 1

Phenopedia diseases ranked according to mean of effect sizes in cluster:
Kidney Failure | Kidney Failure, Chronic | Renal Insufficiency, Chronic | Kidney Diseases | Nephritis | Glomerulonephritis

Corresponding mean of effect sizes of each Phenopedia disease:
(In the same order as above)
2.98, 2.96, 2.73, 2.31, 1.87, 1.52

Corresponding total number of genes of each Phenopedia disease:
(In the same order as above)
317, 287, 314, 716, 222, 196

Corresponding number of genes of each Phenopedia disease with at least one TF hit (of selected TFs):
(In the same order as above)
49, 46, 46, 90, 27, 22

Corresponding total number of TF hits (of selected TFs) for each Phenopedia disease (in all genes):
(In the same order as above)
83, 78, 78, 148, 46, 38

Corresponding number of selected TFs each Phenopedia disease is involved with:
(In the same order as above)
3, 3, 3, 3, 3, 3

TFs ranked according to mean of effect sizes in cluster:
V$HNF1_01, V$HNF1_Q6, V$HNF1_Q6_01

Corresponding mean of effect sizes of each TF:
(In the same order as above)
2.47, 2.38, 2.33

Corresponding total number of TF hits for each TF (genome-wide):
(In the same order as above)
499, 532, 521

Corresponding total number of TF hits for each TF (in all genes in selected Phenopedia diseases):
(In the same order as above)
153, 158, 160

Corresponding number of genes (of selected Phenopedia diseases) each TF is involved with:
(In the same order as above)
50, 52, 53

Corresponding number of selected Phenopedia diseases each TF is involved with:
(In the same order as above)

6, 6, 6

Disease groups of selected Phenopedia diseases ranked according to sum of effect sizes
Urologic Diseases | Male Urogenital Diseases | Kidney Diseases | Female Urogenital Diseases | Renal Insufficiency | Nephritis | Kidney Failure | Kidney Failure, Chronic | Renal Insufficiency, Chronic | Glomerulonephritis

Corresponding sum of effect sizes for each disease group of selected Phenopedia diseases
(In the same order as above)
34.22, 34.22, 34.22, 34.22, 17.13, 10.16, 8.95, 8.88, 8.19, 4.55

Cluster for columns 177 to 191, rows 322 to 333

Phenopedia diseases

Nervous System Malformations | Neural Tube Defects | Spinal Dysraphism | Mesothelioma | Pleural Neoplasms | Brain Injuries | Sensory Thresholds | Amino Acid Metabolism, Inborn Errors | Hyperhomocysteinemia | Vitamin B Deficiency | Vitamin D Deficiency | Vitamin K Deficiency | Malnutrition | Avitaminosis | Deficiency Diseases

TFs

V$ARNT_01 | V$MYC_Q2 | V$CLOCKBMAL_Q6 | V$USF_C | V$MYCMAX_02 | V$USF_Q6 | V$USF_02 | V$USF_Q6_01 | V$USF_01 | V$MYCMAX_03 | V$MAX_01 | V$YY1_02

Information

All related TFs:
(List of all TFs that are related to any of the PWMs)
Arnt, Clock:BMAL, Clock:BMAL1, Clock:BMAL2, Max, Max1, USF, USF-1, USF1, USF1:USF2, USF1a, USF1b, USF2, USF2a, USF2b, YY1, c-Myc

Ranked gene list:
(All genes of the selected Phenopedia diseases with hits of any of the selected TFs, ranked according to the total number of TF hits)
MTHFR, SLC19A1, COMT, CFL1, APEX1, MMACHC, CYP27B1, PRMT1, TGFB1, GAMT, DNMT3A, APOE, MAT2A, CBS, MTR, XRCC3, SHMT2, CRABP2, TAS1R3, MTHFD1, BHMT, MS, GALT, MC1R, TRPM7, SLC25A13, FTCD, AMD1, UCP2, GPX1, ZIC1, F7, ATIC, TSC2, RNMT, OPRD1, NTRK1, NOS2, MGMT, CTH, CHKA, SLC45A2, IL13, HSPD1, GSTM3, GCLC, EGR2, CD320, BMP4, SPG20, SMG6, PAH, L1CAM, HPS1, EPHB4, ANKK1

Corresponding total number of TF hits:
(For each gene listed above, the total number of TF hits for any of the selected TFs, multiplied by the number of selected Phenopedia diseases containing that gene)
120, 99, 65, 30, 30, 25, 24, 21, 20, 18, 15, 13, 12, 12, 11, 10, 10, 10, 9, 9, 9, 8, 8, 7, 6, 6, 6, 6, 5, 5, 4, 4, 4, 3, 3, 3, 3, 3, 3, 3, 3, 2, 2, 2, 2, 2, 2, 2, 2, 1, 1, 1, 1, 1, 1, 1

Corresponding number of selected Phenopedia diseases each gene is involved with:
(In the same order as above)
15, 9, 13, 3, 3, 5, 6, 3, 4, 3, 3, 13, 3, 6, 11, 5, 2, 5, 1, 9, 9, 8, 1, 1, 2, 1, 3, 3, 5, 1, 2, 4, 4, 1, 3, 1, 1, 3, 3, 3, 3, 1, 1, 1, 2, 1, 1, 1, 2, 1, 1, 1, 1, 1, 1, 1

Corresponding number of selected TFs each gene is involved with:

(In the same order as above)
8, 11, 5, 10, 10, 5, 4, 7, 5, 6, 5, 1, 4, 2, 1, 2, 5, 2, 9, 1, 1, 1, 8, 7, 3, 6, 2, 2, 1, 5, 2, 1, 1, 3, 1, 3, 3, 1, 1, 1, 1, 2, 2, 2, 1, 2, 2, 2, 1, 1, 1, 1, 1, 1, 1, 1

Phenopedia diseases ranked according to mean of effect sizes in cluster:
Amino Acid Metabolism, Inborn Errors | Neural Tube Defects | Avitaminosis | Deficiency Diseases | Spinal Dysraphism | Malnutrition | Vitamin B Deficiency | Hyperhomocysteinemia | Mesothelioma | Sensory Thresholds | Pleural Neoplasms | Brain Injuries | Nervous System Malformations | Vitamin D Deficiency | Vitamin K Deficiency

Corresponding mean of effect sizes of each Phenopedia disease:
(In the same order as above)
2.16, 2.00, 1.96, 1.95, 1.89, 1.78, 1.70, 1.36, 1.35, 1.33, 1.14, 1.04, 0.97, 0.96, 0.95

Corresponding total number of genes of each Phenopedia disease:
(In the same order as above)
59, 97, 30, 36, 77, 62, 39, 40, 30, 28, 29, 20, 146, 28, 31

Corresponding number of genes of each Phenopedia disease with at least one TF hit (of selected TFs):
(In the same order as above)
17, 31, 11, 12, 23, 14, 12, 12, 5, 5, 4, 4, 36, 7, 8

Corresponding total number of TF hits (of selected TFs) for each Phenopedia disease (in all genes):
(In the same order as above)
62, 100, 39, 42, 80, 49, 45, 35, 21, 26, 16, 15, 105, 26, 27

Corresponding number of selected TFs each Phenopedia disease is involved with:
(In the same order as above)
12, 12, 12, 12, 12, 12, 12, 12, 12, 12, 12, 12, 12, 12, 12

TFs ranked according to mean of effect sizes in cluster:
V$MYCMAX_02, V$ARNT_01, VUSF_C, VMYCMAX_03, VMAX_01, VMYC_Q2, V$CLOCKBMAL_Q6, V$USF_Q6, V$YY1_02, V$USF_Q6_01, VUSF_01, VUSF_02

Corresponding mean of effect sizes of each TF:
(In the same order as above)
2.52, 2.17, 2.11, 1.72, 1.67, 1.45, 1.36, 1.12, 1.04, 1.02, 0.96, 0.88

Corresponding total number of TF hits for each TF (genome-wide):
(In the same order as above)
457, 456, 437, 453, 451, 421, 454, 423, 392, 433, 441, 421

Corresponding total number of TF hits for each TF (in all genes in selected Phenopedia diseases):
(In the same order as above)
80, 66, 73, 63, 70, 55, 57, 50, 41, 49, 45, 39

Corresponding number of genes (of selected Phenopedia diseases) each TF is involved with:
(In the same order as above)
18, 16, 18, 19, 17, 16, 16, 14, 8, 15, 12, 9

Corresponding number of selected Phenopedia diseases each TF is involved with:
(In the same order as above)
15, 15, 15, 15, 15, 15, 15, 15, 15, 15, 15, 15

Disease groups of selected Phenopedia diseases ranked according to sum of effect sizes

Signs and Symptoms | Protein-Energy Malnutrition | Nutrition Disorders | Malnutrition | Congenital, Hereditary, and Neonatal Diseases and Abnormalities | Deficiency Diseases | Avitaminosis | Nervous System Malformations | Spinal Dysraphism | Spinal Diseases | Spinal Cord Diseases | Neural Tube Defects | Nervous System Diseases | Musculoskeletal Diseases | Central Nervous System Diseases | Bone Diseases | Metabolism, Inborn Errors | Metabolic Diseases | Genetic Diseases, Inborn | Amino Acid Metabolism, Inborn Errors | Vitamin B Deficiency | Hyperhomocysteinemia | Neoplasms, Mesothelial | Neoplasms, Glandular and Epithelial | Neoplasms | Mesothelioma | Adenoma | Sensory Thresholds | Thoracic Neoplasms | Thoracic Diseases | Respiratory Tract Diseases | Pleural Neoplasms | Pleural Diseases | Connective Tissue Diseases | Contusions | Brain Injuries | Vitamin D Deficiency | Vitamin K Deficiency

Corresponding sum of effect sizes for each disease group of selected Phenopedia diseases
(In the same order as above)
224.54, 111.55, 111.55, 111.55, 100.50, 90.19, 66.82, 58.21, 46.62, 46.62, 46.62, 46.62, 46.62, 46.62, 46.62, 46.62, 42.29, 42.29, 42.29, 42.29, 20.44, 16.36, 16.19, 16.19, 16.19, 16.19, 16.19, 15.97, 13.65, 13.65, 13.65, 13.65, 13.65, 13.65, 12.49, 12.49, 11.46, 11.45

Cluster for columns 113 to 132, rows 218 to 232

Phenopedia diseases

Breast Diseases | Breast Neoplasms | Endocrine Gland Neoplasms | Ovarian Diseases | Ovarian Neoplasms | Genital Diseases, Female | Genital Neoplasms, Female | Urogenital Neoplasms | Male Urogenital Diseases | Urologic Diseases | Hemochromatosis | Lung Diseases, Obstructive | Lung Diseases | Respiratory Tract Diseases | Urinary Bladder Neoplasms | Urologic Neoplasms | Pulmonary Disease, Chronic Obstructive | Respiratory Tract Neoplasms | Lung Neoplasms | Thoracic Neoplasms

TFs

V$E2F_Q6_01 | V$E2F_Q4_01 | V$E2F_Q3_01 | V$E2F1_Q4_01 | V$E2F_03 | V$E2F1_Q6_01 | V$E2F_Q6 | V$E2F_Q4 | V$E2F_Q3 | V$E2F4DP2_01 | V$E2F1DP2_01 | V$E2F1DP1_01 | V$E2F4DP1_01 | V$E2F1DP1RB_01 | V$E2F_02

Information

All related TFs:
(List of all TFs that are related to any of the PWMs)
DP-1, E2F, E2F+E4, E2F-1, E2F-1:DP-1, E2F-1:DP-2, E2F-2, E2F-3a, E2F-4, E2F-4:DP-1, E2F-4:DP-2, E2F-5, E2F-7, pRb:E2F-1:DP-1

Ranked gene list:
(All genes of the selected Phenopedia diseases with hits of any of the selected TFs, ranked according to the total number of TF hits)
PCNA, CDKN1B, EXO1, CCND1, CDC25B, GATA3, IRS2, GNAS, MDM2, CDKN1A, TP73, BCL6, IRS1, AKT1, CDC25A, CDK5, MTHFR, LIG4, LIG1, IRF1, E2F2, CDK6, FANCC, PIN1, PIM1, MSH3, IGF1R, RXRB, RASSF1, SEPT2, DNMT1, CCND2, SMAD7, RBL1, PIK3CA, SKP2, POLE, NUBP1, COL1A1, IFNAR1, XPC, TP53, MSH2, MLH1, EZH2, BACH1, BAT3, POLE2, BRAF, E2F1, POLRMT, MYNN, MATR3, FOXC1, TOPBP1, CDKN2C, MTHFD1, VDR, RPA2, RFC1, MTR, HELLS, GEMIN4, AHRR, CDC6, XRCC6, TACC3, CTSD, CDKN1C, PPP1R13L, MX1, LMOD1, CD81, HIF1A, APOE, DBP, ARRB2, RB1, POLA2, REV1, CD3EAP, STAT3, PRKDC, H2AFX, ARVCF, TNFRSF6B, NBN, JUN, IDE, CAMKK1, WRAP53, SLC39A2, NUBP2, MS, MPDU1, ITGB3, TFDP1, STAT1, SLC22A4, RAD51C, PTCH1, GSS, CYP11A1, CDC2, CASP7, AKT2, UNG, SUV39H2, RFC3, POLR2A, HFE, NR3C1, COL18A1, ZPBP2, SOCS3, NR3C2, JUND, HSPE1,

FOXO3, CBS, ALOXE3, ADM, SEMA3B, RRM2, RAD54L, MYB, MAPT, LTBP4, INHBB, ACP1, RRM1, GMNN, DCK, SLC20A1, RAC1, PARP4, HOXD13, HOXA10, GTF2A1, DMTF1, BUB3, ARNTL, APOA2, WDR79, REV3L, PPARA, EGR1, CLSPN, CDT1, CDKN2D, TCF7, PHLDB1, PHF11, NME1, KEAP1, AGER, XRCC5, TBCD, SLC40A1, SHC1, RING1, REV1L, RAD51, PHB, NDUFS6, IKZF3, IGF2, HRAS, CTNNB1, CHEK1, ABCB1, TBL3, SRD5A1, SH2B3, PPP1CB, PMS1, MAL, CTCF, ADORA2A, GSK3B, TSHZ3, SMARCB1, SLC39A9, PER1, PDE8A, PCGF2, NEUROD1, MYC, HSPA2, DNMT3A, DGCR8, CNTNAP1, CCNE1, ARHGEF2, TIMELESS, TAPBP, SUMO1, SIPA1, RTEL1, POLI, POLD1, MCM5, LRP5, ITGA6, HIST1H3B, HHEX, GPX4, ECE1, DDB2, SHARPIN, KPNB1, KISS1R, DMC1, C11orf30, BRD2, ATF3, ACACA, LRP6, IRF4, IGF2AS, CDC25C, ALOX5, SPOCK2, PTCH, PSMB8, PER2, PDGFA, NFKB2, HDAC7, GALT, FKBPL, ESD, DPYD, DNASE2, CCNE2, APOC1, PTBP1, FUS, YWHAQ, TET2, SMC6, RHEB, RECQL4, MAT2A, HSPG2, GAPDH, FBXL20, FASN, EGR3, EFNB2, CASP6, ATP2A3, ANXA5, AATF, TLE4, TEX14, CYFIP2, CASP2, WNT2B, WDR37, VPS52, TACR1, SOCS1, SMARCA4, SMAD6, SLC39A7, RUNX2, PREX1, OAZ1, NCOR1, EPAS1, DHX9, CPT1A, BACH2, UBE2I, TUBG1, ZWILCH, VGF, TSSK4, SLC2A4RG, SLC26A6, PSMB9, PPARGC1B, PLCB3, PCNXL3, PCMT1, P4HA2, NUMA1, MFNG, LRP8, INSIG2, HNRNPUL1, HMGB2, HMGA2, GTF2F1, GNB1, GBA, DAG1, CUL1, CIDEB, BCL2L1, ABCB6, MBD1, KCNIP3, HSPD1, HSD17B8, GPX7, GNAI2, EGR2, DDX20, ZHX2, TCF12, TARBP2, SVIL, STEAP1, SMARCA2, SEMA3F, RAD51L1, PSENEN, PARD3, NIN, NDUFS2, NCF4, MDC1, LY6G6F, KLF12, ITPR1, IFIH1, ICAM3, FANCI, EHMT2, EHBP1, DICER1, DDAH2, CXCR4, CD46, CARM1, YBX2, WDR36, TRAF3, TPT1, TACR2, SPRY4, SIK3, SCD, RAI1, PROC, NF2, MCM4, KLF5, KCNS3, HOXB6, HOXA4, HAT1, GRK5, FKBP5, FANCL, FADS2, CTTN, CHRND, CDC37, BAD, VTI1B, VTI1A, TUBB, TRPC1, TRIP12, TPMT, TOMM40, TBX2, SOX9, SNAP29, SNAI1, SLC39A4, SLC1A5, RHOC, PRMT1, PRKAG2, POR, PKD2, ORC3L, NR1H2, NPHP4, MTHFD1L, MEF2C, LASS2, ITGB1BP1, IL11RA, HMOX2, FOXA1, FMN2, ENAH, DUOX2, CTBP2, CHD7, CCR7, APH1A, ANKMY1, TFAM, RTN3, RCAN1, RARG, RARA, NTRK3, NOTCH1, MAP3K12, LGALS1, FGF8, ENO2, DLST, CYP46A1, CDC73, ACHE

Corresponding total number of TF hits:
(For each gene listed above, the total number of TF hits for any of the selected TFs, multiplied by the number of selected Phenopedia diseases containing that gene)
300, 300, 260, 260, 234, 225, 210, 210, 200, 200, 196, 195, 190, 180, 162, 156, 140, 140, 140, 136, 135, 135, 132, 130, 130, 120, 120, 117, 105, 104, 104, 96, 90, 90, 90, 88, 88, 88, 88, 84, 80, 80, 80, 80, 80, 80, 78, 70, 68, 66, 65, 65, 65, 65, 64, 64, 63, 60, 60, 60, 60, 60, 56, 56, 55, 54, 54, 54, 54, 52, 52, 52, 52, 51, 51, 48, 48, 45, 45, 44, 44, 42, 42, 42, 42, 40, 40, 40, 40, 40, 39, 39, 39, 39, 39, 38, 36, 36, 36, 36, 36, 36, 36, 36, 36, 36, 35, 35, 35, 35, 34, 33, 32, 30, 30, 30, 30, 30, 30, 30, 30, 30, 28, 28, 28, 28, 28, 28, 28, 28, 27, 27, 27, 26, 26, 26, 26, 26, 26, 26, 26, 26, 26, 24, 24, 24, 24, 24, 24, 24, 22, 21, 21, 21, 21, 21, 20, 20, 20, 20, 20, 20, 20, 20, 20, 20, 20, 20, 20, 20, 20, 18, 18, 18, 18, 18, 18, 18, 18, 17, 16, 16, 16, 16, 16, 16, 16, 16, 16, 16, 16, 16, 16, 16, 16, 15, 15, 15, 15, 15, 15, 15, 15, 15, 15, 15, 15, 15, 15, 15, 14, 14, 14, 14, 14, 14, 14, 14, 13, 13, 13, 13, 13, 12, 12, 12, 12, 12, 12, 12, 12, 12, 12, 12, 12, 12, 11, 11, 10, 10, 10, 10, 10, 10, 10, 10, 10, 10, 10, 10, 10, 10, 10, 10, 9, 9, 9, 9, 8, 8, 8, 8, 8, 8, 8, 8, 8, 8, 8, 8, 8, 8, 8, 8, 8, 7, 7, 6, 6, 6, 6, 6, 6, 6, 6, 6, 6, 6, 6, 6, 6, 6, 6, 6, 6, 6, 6, 6, 6, 6, 6, 6, 5, 5, 5, 5, 5, 5, 5, 5, 4, 4, 4, 4, 4, 4, 4, 4, 4, 4, 4, 4, 4, 4, 4, 4, 4, 4, 4, 4, 4, 4, 4, 4, 4, 4, 3, 3, 3, 3, 3, 3, 3, 3, 3, 3, 3, 3, 3, 3, 3, 3, 3, 3, 3, 3, 3, 3, 3, 3, 3, 2, 2, 2, 2, 2, 2, 2, 2, 2, 2, 2, 2, 2, 2, 2, 2, 2, 2, 2, 2, 2, 2, 2, 2, 2, 2, 2, 2, 2, 2, 2, 2, 2, 2, 1, 1, 1, 1, 1, 1, 1, 1, 1, 1, 1, 1, 1, 1

Corresponding number of selected Phenopedia diseases each gene is involved with:
(In the same order as above)
20, 20, 20, 20, 18, 15, 14, 14, 20, 20, 14, 13, 19, 20, 18, 13, 20, 20, 20, 17, 9, 15, 12, 13, 13, 20, 20, 13, 7, 13, 8, 8, 10, 6, 15, 8, 11, 8, 8, 7, 20, 20, 20, 20, 8, 8, 6, 5, 17, 6, 5, 13, 13, 13, 8, 8, 9, 20, 5, 12, 20, 6, 4, 14, 5, 18, 6, 9, 18, 13, 13, 13, 13, 17, 17, 6, 4, 15, 5, 11, 11, 14, 14, 7, 6, 8, 20, 5, 5, 5, 13, 13, 13, 13, 13, 19, 6, 18, 3, 12, 6, 6, 12, 9, 9, 6, 5, 5, 5, 5, 17, 11, 16, 3, 3, 2, 5, 6, 2, 15, 6, 2, 7, 2, 14, 4, 7, 4, 7, 7, 9, 3, 3, 2, 13, 13, 2, 2, 2, 2, 2, 2, 13, 8, 6, 8, 3, 2, 2, 8, 2, 3, 3, 7, 7, 7, 20, 2, 5, 2, 4, 5, 20, 20, 2, 2, 20, 20, 20, 20, 20, 2, 9, 3, 2, 18, 6, 2, 3, 17, 2, 4, 2, 4, 2, 2, 2, 16, 2, 8, 4, 2, 8, 2, 5, 3, 5, 5, 3, 15, 15, 1, 15, 5, 1, 5, 15, 3, 15, 2, 2, 1, 2, 2, 2, 1, 2, 13, 1, 13, 13, 13, 2, 2, 6, 2, 4, 3, 3, 6, 1, 2, 4, 2, 6, 4, 1, 1, 2, 2, 1, 5, 10, 2, 2, 1, 2, 2, 1, 2, 2, 5, 2, 2, 3, 3, 3, 3, 1, 2, 2, 4, 4, 2, 2, 1, 1, 2, 2, 8, 2, 2, 1, 2, 7, 1, 2, 1, 1, 2, 2, 3, 3, 3, 2, 2, 3, 2, 3, 2, 2, 2, 1, 3, 2, 3, 2, 2, 6, 2, 6, 2, 5, 1, 1, 1, 5, 1, 1, 5, 2, 1, 4, 4, 2, 2, 2, 2, 1, 2, 1, 2, 4, 4, 1, 4, 4, 2, 1, 2, 2,

2, 4, 2, 2, 2, 2, 1, 3, 1, 1, 3, 3, 1, 1, 3, 1, 1, 1, 1, 3, 1, 1, 3, 3, 3, 3, 1, 3, 1, 1, 1, 1, 1, 2, 2, 1, 2, 2, 2, 1, 1, 2, 1, 2, 2, 2, 2, 2, 1, 1, 2, 2, 2, 2, 2, 1, 2, 2, 2, 2, 2, 2, 1, 1, 1, 1, 1, 1, 1, 1, 1, 1, 1, 1, 1, 1, 1, 1, 1, 1

Corresponding number of selected TFs each gene is involved with:
(In the same order as above)
15, 15, 13, 13, 13, 15, 15, 15, 10, 10, 14, 15, 10, 9, 9, 12, 7, 7, 7, 8, 15, 9, 11, 10, 10, 6, 6, 9, 15, 8, 13, 12, 9, 15, 6, 11, 8, 11, 11, 12, 4, 4, 4, 4, 10, 10, 13, 14, 4, 11, 13, 5, 5, 5, 8, 8, 7, 3, 12, 5, 3, 10, 14, 4, 11, 3, 9, 6, 3, 4, 4, 4, 4, 3, 3, 8, 12, 3, 9, 4, 4, 3, 3, 6, 7, 5, 2, 8, 8, 8, 3, 3, 3, 3, 3, 2, 6, 2, 12, 3, 6, 6, 3, 4, 4, 6, 7, 7, 7, 7, 2, 3, 2, 10, 10, 15, 6, 5, 15, 2, 5, 15, 4, 14, 2, 7, 4, 7, 4, 4, 3, 9, 9, 13, 2, 2, 13, 13, 13, 13, 13, 13, 2, 3, 4, 3, 8, 12, 12, 3, 11, 7, 7, 3, 3, 3, 1, 10, 4, 10, 5, 4, 1, 1, 10, 10, 1, 1, 1, 1, 1, 9, 2, 6, 9, 1, 3, 9, 6, 1, 8, 4, 8, 4, 8, 8, 8, 1, 8, 2, 4, 8, 2, 8, 3, 5, 3, 3, 5, 1, 1, 15, 1, 3, 15, 3, 1, 5, 1, 7, 7, 14, 7, 7, 7, 14, 7, 1, 13, 1, 1, 1, 6, 6, 2, 6, 3, 4, 4, 2, 12, 6, 3, 6, 2, 3, 11, 11, 5, 5, 10, 2, 1, 5, 5, 10, 5, 5, 10, 5, 5, 2, 5, 5, 3, 3, 3, 3, 8, 4, 4, 2, 2, 4, 4, 8, 8, 4, 4, 1, 4, 4, 8, 4, 1, 7, 3, 6, 6, 3, 3, 2, 2, 2, 3, 3, 2, 3, 2, 3, 3, 3, 6, 2, 3, 2, 3, 3, 1, 3, 1, 3, 1, 5, 5, 5, 1, 5, 5, 1, 2, 4, 1, 1, 2, 2, 2, 2, 4, 2, 4, 2, 1, 1, 4, 1, 1, 2, 4, 2, 2, 2, 1, 2, 2, 2, 2, 3, 1, 3, 3, 1, 1, 3, 3, 1, 3, 3, 3, 3, 1, 3, 3, 1, 1, 1, 1, 3, 1, 3, 3, 3, 2, 2, 1, 1, 2, 1, 1, 1, 2, 2, 1, 2, 1, 1, 1, 1, 1, 1, 2, 2, 1, 1, 1, 1, 1, 1, 2, 1, 1, 1, 1, 1, 2, 2, 2, 1, 1, 1, 1, 1, 1, 1, 1, 1, 1, 1, 1, 1, 1

Phenopedia diseases ranked according to mean of effect sizes in cluster:
Ovarian Diseases | Breast Neoplasms | Breast Diseases | Ovarian Neoplasms | Genital Neoplasms, Female | Genital Diseases, Female | Urologic Neoplasms | Urinary Bladder Neoplasms | Urogenital Neoplasms | Endocrine Gland Neoplasms | Lung Neoplasms | Thoracic Neoplasms | Respiratory Tract Neoplasms | Pulmonary Disease, Chronic Obstructive | Hemochromatosis | Male Urogenital Diseases | Lung Diseases | Urologic Diseases | Respiratory Tract Diseases | Lung Diseases, Obstructive

Corresponding mean of effect sizes of each Phenopedia disease:
(In the same order as above)
2.92, 2.87, 2.87, 2.79, 2.72, 2.53, 2.15, 2.15, 2.06, 1.92, 1.62, 1.51, 1.47, 1.20, 1.19, 1.07, 0.78, 0.77, 0.66, 0.39

Corresponding total number of genes of each Phenopedia disease:
(In the same order as above)
421, 680, 680, 333, 433, 582, 575, 523, 1.0K, 585, 626, 637, 646, 506, 983, 1.4K, 1.2K, 1.0K, 1.2K, 891

Corresponding number of genes of each Phenopedia disease with at least one TF hit (of selected TFs):
(In the same order as above)
85, 140, 140, 68, 85, 105, 109, 99, 178, 111, 111, 112, 112, 84, 154, 197, 168, 148, 172, 123

Corresponding total number of TF hits (of selected TFs) for each Phenopedia disease (in all genes):
(In the same order as above)
495, 755, 755, 391, 481, 600, 576, 533, 929, 588, 549, 552, 564, 432, 808, 1008, 827, 745, 843, 628

Corresponding number of selected TFs each Phenopedia disease is involved with:
(In the same order as above)
15, 15, 15, 15, 15, 15, 15, 15, 15, 15, 15, 15, 15, 15, 15, 15, 15, 15, 15, 15

TFs ranked according to mean of effect sizes in cluster:
V$E2F1_Q6_01, V$E2F_Q6_01, V$E2F4DP2_01, V$E2F_Q4_01, V$E2F1DP1RB_01, V$E2F_03, V$E2F1DP1_01, V$E2F_Q3_01, V$E2F1DP2_01, V$E2F_02, V$E2F4DP1_01, V$E2F_Q3, V$E2F1_Q4_01, V$E2F_Q6, V$E2F_Q4

Corresponding mean of effect sizes of each TF:
(In the same order as above)
2.43, 2.27, 2.22, 1.91, 1.90, 1.86, 1.83, 1.81, 1.77, 1.73, 1.56, 1.44, 1.39, 1.36, 1.27

Corresponding total number of TF hits for each TF (genome-wide):

(In the same order as above)
447, 452, 452, 432, 448, 450, 450, 431, 454, 442, 442, 442, 437, 430, 438

Corresponding total number of TF hits for each TF (in all genes in selected Phenopedia diseases):
(In the same order as above)
954, 948, 939, 827, 925, 933, 901, 781, 908, 862, 879, 816, 774, 807, 805

Corresponding number of genes (of selected Phenopedia diseases) each TF is involved with:
(In the same order as above)
144, 144, 149, 132, 141, 146, 141, 130, 142, 131, 133, 140, 126, 140, 138

Corresponding number of selected Phenopedia diseases each TF is involved with:
(In the same order as above)
20, 20, 20, 20, 20, 20, 20, 20, 20, 20, 20, 20, 20, 20, 20

Disease groups of selected Phenopedia diseases ranked according to sum of effect sizes
Female Urogenital Diseases | Respiratory Tract Diseases | Neoplasms | Thoracic Diseases | Pelvic Neoplasms | Genital Diseases, Female | Male Urogenital Diseases | Thoracic Neoplasms | Breast Diseases | Ovarian Diseases | Genital Neoplasms, Female | Urologic Diseases | Abdominal Neoplasms | Urologic Neoplasms | Lung Diseases | Breast Neoplasms | Ovarian Neoplasms | Urinary Bladder Neoplasms | Urinary Bladder Diseases | Urogenital Neoplasms | Endocrine System Diseases | Endocrine Gland Neoplasms | Lung Neoplasms | Lung Diseases, Obstructive | Respiratory Tract Neoplasms | Respiration Disorders | Pulmonary Disease, Chronic Obstructive | Signs and Symptoms | Metabolic Diseases | Iron Overload | Iron Metabolism Disorders | Hemochromatosis

Corresponding sum of effect sizes for each disease group of selected Phenopedia diseases
(In the same order as above)
230.75, 200.65, 178.21, 168.65, 147.28, 123.76, 123.06, 90.11, 86.12, 85.78, 82.72, 76.05, 74.18, 64.56, 59.81, 43.06, 41.92, 32.26, 32.26, 30.93, 28.81, 28.81, 24.33, 23.85, 22.10, 22.10, 17.97, 17.91, 17.91, 17.91, 17.91, 17.91

Cluster for columns 44 to 100, rows 218 to 232

Phenopedia diseases

Anemia | Fanconi Anemia | Ataxia | Ataxia Telangiectasia | Nerve Sheath Neoplasms | Vestibulocochlear Nerve Diseases | Neurilemmoma | Neuroma, Acoustic | Meningeal Neoplasms | Brain Neoplasms | Central Nervous System Neoplasms | Nervous System Neoplasms | Xeroderma Pigmentosum | Neoplasms, Radiation-Induced | Radiation Injuries | Abnormalities | Abnormalities | Tooth Abnormalities | Tooth Diseases | Mouth Diseases | Craniofacial Abnormalities | Musculoskeletal Abnormalities | Cleft Lip | Cleft Palate | Jaw Abnormalities | Mouth Abnormalities | Genital Diseases, Male | Genital Neoplasms, Male | Prostatic Diseases | Prostatic Neoplasms | Gastrointestinal Diseases | Intestinal Diseases | Carcinoma, Neuroendocrine | Carcinoma, Small Cell | Melanoma | Neuroendocrine Tumors | Stomach Diseases | Stomach Neoplasms | Carcinoma, Squamous Cell | Neoplasms, Squamous Cell | Carcinoma | Esophageal Neoplasms | Warts | Colorectal Neoplasms | Intestinal Neoplasms | Adenocarcinoma | Digestive System Neoplasms | Gastrointestinal Neoplasms | Aneuploidy | Pancreatic Diseases | Pancreatic Neoplasms | Cell Transformation, Neoplastic | Genomic Instability | Microsatellite Instability | Neoplastic Syndromes, Hereditary | Colorectal Neoplasms, Hereditary Nonpolyposis | DNA Repair-Deficiency Disorders

TFs

V$E2F_Q6_01 | V$E2F_Q4_01 | V$E2F_Q3_01 | V$E2F1_Q4_01 | V$E2F_03 | V$E2F1_Q6_01 | V$E2F_Q6 | V$E2F_Q4 | V$E2F_Q3 | V$E2F4DP2_01 | V$E2F1DP2_01 | V$E2F1DP1_01 | V$E2F4DP1_01 | V$E2F1DP1RB_01 | V$E2F_02

Information

All related TFs:
(List of all TFs that are related to any of the PWMs)
DP-1, E2F, E2F+E4, E2F-1, E2F-1:DP-1, E2F-1:DP-2, E2F-2, E2F-3a, E2F-4, E2F-4:DP-1, E2F-4:DP-2, E2F-5, E2F-7, pRb:E2F-1:DP-1

Ranked gene list:
(All genes of the selected Phenopedia diseases with hits of any of the selected TFs, ranked according to the total number of TF hits)
CCND1, TP73, CDKN1B, GNAS, MDM2, PCNA, EXO1, CDKN1A, MTHFR, LIG4, IRS2, LIG1, TP53, MTHFD1, IRS1, FOXO3, XPC, MSH2, PIK3CA, IGF1R, RFC1, BRAF, RASSF1, MSH3, MLH1, AKT1, BAT3, E2F2, FANCC, PAX6, GEMIN4, MTR, DPF3, IKZF3, EZH2, CDK6, RBL1, BCL6, DNMT1, PTCH1, SMAD7, OSR1, GAD1, BCL2L11, SHMT2, SATB2, LEF1, COL1A1, GART, VDR, RRM2, CDK5, ARNTL, NBN, SLC22A4, RUNX2, CDC25A, CASP6, APOE, NR3C1, HIF1A, GTF2A1, CBS, CASP7, XRCC6, SMC6, UNG, REV1, ITGB3, IDE, FGFRL1, CAMKK1, TACC3, RXRB, STAT3, PRKDC, CYP11A1, MAT2A, C11orf30, TOPBP1, IRF1, AKT2, WNT11, SKI, RB1, MCM6, FOXC1, DCK, HFE, TUBG1, PTCH, PPARA, NF2, LTBP4, ATF3, NDUFS6, CRLF3, ATAD5, HOXD13, HOXA10, DPYD, SUMO1, REV3L, RAD51, PER2, MAPT, IFNAR1, H2AFX, DGCR8, GATA2, FUS, DLX4, CDC6, CASP2, SLC39A9, PPP1R13L, PCGF2, HSPA2, AHRR, ACP1, TYMP, RRM1, RCC2, PIN1, PIM1, MS, MDK, GNAI2, GAMT, DPYSL2, CHEK1, BACH1, ABCB1, ZIC2, SMAD6, NAT9, KISS1R, CD3EAP, BACH2, ZC3H10, XRCC5, IRF4, GSK3A, CCNO, C18orf25, ECE1, SMC3, RPA2, PER1, NIN, INSIG2, IGF2, EHMT2, CXCR4, CTNNB1, CDKN2C, CASP8AP2, TRADD, PKN2, ICK, GIPC2, COL18A1, BARX1, PHLDB1, LATS1, KEAP1, HHEX, ALKBH2, TET2, SEMA3B, RAD54L, PAX5, NFATC1, MDGA1, EGR3, ECGF1, CHAF1A, ATN1, HRAS, WRAP53, TIMELESS, TEX14, SIK3, REEP6, POLI, PARP4, NHEJ1, HMGB2, GRM8, DNMT3A, CDC37, PMS1, VPS52, TCOF1, REV1L, POLE, NEUROD1, MYC, JUN, INHBB, IHH, IFIH1, HOXB7, DBP, ATP2A3, TNFRSF6B, RTEL1, MATR3, HSPG2, GATA3, SORL1, SLC38A1, SLC22A23, RAD51L1, PSMB9, PPARGC1B, HOXD11, HMBS, GARS, CCNE1, BCL2L1, CDC25B, BUB3, ZHX2, STAT1, SMARCB1, SH2B3, NIPBL, MX1, MLL, MGRN1, ITGA6, GBA, FKBPL, CIDEB, CD81, CCND2, ARRB2, TCF7, TCF21, PAX9, MTHFD1L, GSK3B, FANCL, TPMT, SLC25A1, RING1, ICMT, HSPD1, EGR2, ALOXE3, ALOX5, WNT5A, RARA, MDC1, JAG2, HOXB6, HOXA4, GPX4, DICER1, CTCF, CHD7, CABIN1, ZNF827, VGLL4, TMEM18, TARBP2, SRD5A1, SLC40A1, SEPT2, SEMA3F, RAC1, PROX1, PCNT, NPM1, NDUFS2, MKKS, LBH, KRI1, HOXA11, HDAC7, GMPS, EHBP1, DDX20, COQ3, CFL1, APP, SUV39H2, RECQL4, POLD1, NTRK3, MYB, ITGA7, DAD1, CDC25C, BRD2, BAT4, ARVCF, ZGPAT, TSSK4, SOX9, REL, RBPJ, RAD51C, PCMT1, NMB, ING1, HEL308, GALT, FEV, FANCI, E2F7, DAG1, CTSD, CTBP2, CDKN1C, CD46, APOC1, AGER, ABCB6, NOTCH1, NCF4, MYNN, KCNIP3, IGF2AS, HSPE1, GLI1, ZEB1, WDR7, TCF12, STK38L, SPRY4, SOCS1, SNAP29, PRDM16, PMP22, PLAA, PI4KA, PARD3, P4HA2, NR2E1, LMOD1, HMGA2, HEY2, GPD2, FAM129A, ECM1, DLG4, CMT1A, CDC73, CCDC28B, ZWILCH, YBX2, WDR36, SLC39A2, SIPA1, SCA13, RAI1, PBX3, NUBP2, NME1, MPDU1, MKS1, LRP6, KRT8, KCNC3, GCH1, FGF8, EXT2, CDKN2D, CDK8, BAD, ZIC3, ZBTB16, WNK1, TRIP12, TNFRSF19, TACR2, SVIL, STX17, SPRED1, SMC1A, PSMB8, PRMT1, PRKG1, NDRG1, MEIS1, MBD1, MAP3K12, KLF12, KLF10, KIAA1274, IRX3, GOLGA1, FAF1, EN2, CSNK2A1, CSMD2, CCR7, ATXN7, ATP10A, APOA2, UBE2I, TUFT1, RTN3, PLEKHA7, PHB, PDPK1, LRP5, LGALS1, JMJD1C, ITPR1, ENAH, DDB2, ACHE

Corresponding total number of TF hits:
(For each gene listed above, the total number of TF hits for any of the selected TFs, multiplied by the number of selected Phenopedia diseases containing that gene)
494, 476, 390, 375, 370, 360, 351, 350, 322, 273, 240, 210, 196, 196, 180, 165, 164, 164, 162, 162, 160, 152, 150, 150, 144, 144, 143, 135, 132, 126, 126, 123, 121, 110, 110, 108, 105, 105, 104, 102, 99, 99, 99, 91, 90, 90, 90, 88, 85, 84, 84, 84, 78, 74, 72, 72, 72, 70, 69, 66, 66, 65, 64, 64, 63, 60, 56, 56, 56, 56, 56, 56, 54, 54, 51, 51, 51, 50, 49, 48, 48, 48, 45, 45, 45, 45, 45, 45, 44, 42, 42, 42, 42, 42, 42, 40, 40, 40, 39, 39, 39, 36, 36, 36, 36, 36, 36, 36, 36, 33, 33, 33, 33, 33, 32, 32, 32, 32, 32, 32, 30, 30, 30, 30, 30, 30, 30, 30, 30, 30, 30, 30, 30, 28, 28, 28, 28, 28, 28, 26, 26, 26, 26, 26, 26, 25, 24, 24, 24, 24, 24, 24, 24, 24, 24,

24, 24, 22, 22, 22, 22, 22, 22, 21, 21, 21, 21, 21, 20, 20, 20, 20, 20, 20, 20, 20, 20, 19, 18, 18, 18, 18, 18, 18, 18, 18, 18, 18, 18, 18, 17, 16, 16, 16, 16, 16, 16, 16, 16, 16, 16, 16, 15, 15, 15, 15, 15, 14, 14, 14, 14, 14, 14, 14, 14, 14, 14, 14, 13, 13, 12, 12, 12, 12, 12, 12, 12, 12, 12, 12, 12, 12, 12, 11, 11, 11, 11, 11, 11, 10, 10, 10, 10, 10, 10, 10, 10, 9, 9, 9, 9, 9, 9, 9, 9, 9, 9, 9, 8, 8, 8, 8, 8, 8, 8, 8, 8, 8, 8, 8, 8, 8, 8, 8, 8, 8, 8, 8, 8, 8, 8, 8, 7, 7, 7, 7, 7, 7, 7, 7, 7, 7, 7, 6, 6, 6, 6, 6, 6, 6, 6, 6, 6, 6, 6, 6, 6, 6, 6, 6, 6, 6, 6, 6, 5, 5, 5, 5, 5, 5, 5, 4, 4, 4, 4, 4, 4, 4, 4, 4, 4, 4, 4, 4, 4, 4, 4, 4, 4, 4, 4, 4, 4, 4, 4, 3, 3, 3, 3, 3, 3, 3, 3, 3, 3, 3, 3, 3, 3, 3, 3, 3, 3, 3, 3, 3, 2, 2, 2, 2, 2, 2, 2, 2, 2, 2, 2, 2, 2, 2, 2, 2, 2, 2, 2, 2, 2, 2, 2, 2, 2, 2, 2, 2, 1, 1, 1, 1, 1, 1, 1, 1, 1, 1, 1, 1, 1

Corresponding number of selected Phenopedia diseases each gene is involved with:
(In the same order as above)
38, 33, 25, 24, 36, 24, 27, 35, 45, 39, 15, 30, 48, 27, 17, 10, 40, 40, 26, 26, 31, 37, 10, 25, 35, 15, 10, 9, 11, 8, 9, 40, 10, 10, 11, 12, 7, 7, 7, 16, 10, 10, 8, 7, 8, 8, 8, 7, 16, 27, 6, 7, 5, 37, 6, 8, 8, 14, 22, 21, 22, 5, 31, 16, 21, 6, 8, 14, 27, 6, 7, 7, 6, 6, 16, 17, 16, 9, 7, 6, 6, 7, 8, 8, 15, 3, 8, 5, 21, 6, 6, 13, 14, 6, 2, 4, 3, 3, 2, 2, 13, 11, 9, 36, 5, 8, 3, 6, 9, 2, 2, 10, 3, 11, 4, 8, 4, 4, 8, 7, 3, 10, 5, 3, 2, 9, 6, 5, 1, 1, 30, 3, 29, 1, 7, 2, 1, 7, 7, 2, 26, 2, 1, 2, 2, 5, 1, 2, 5, 6, 8, 23, 12, 12, 23, 3, 6, 1, 10, 2, 10, 11, 10, 3, 7, 7, 6, 3, 4, 5, 10, 1, 1, 1, 1, 2, 4, 3, 19, 6, 5, 6, 6, 6, 18, 9, 6, 2, 2, 8, 6, 17, 4, 7, 4, 2, 1, 15, 2, 4, 3, 8, 3, 1, 8, 3, 3, 2, 2, 1, 1, 1, 2, 6, 7, 7, 1, 1, 1, 7, 14, 1, 1, 6, 6, 3, 2, 1, 3, 2, 2, 4, 3, 1, 4, 3, 1, 1, 1, 10, 10, 10, 10, 11, 10, 2, 2, 1, 1, 1, 1, 10, 8, 8, 9, 8, 2, 2, 9, 9, 1, 8, 3, 2, 2, 2, 8, 4, 2, 1, 4, 4, 1, 1, 3, 4, 1, 2, 2, 1, 2, 2, 4, 8, 1, 1, 3, 1, 6, 7, 6, 1, 7, 7, 7, 1, 7, 1, 2, 1, 2, 2, 2, 2, 1, 1, 6, 3, 2, 1, 3, 2, 1, 1, 6, 2, 2, 2, 2, 1, 4, 5, 1, 1, 5, 1, 4, 1, 2, 1, 2, 4, 2, 1, 2, 1, 1, 1, 2, 2, 1, 1, 1, 1, 1, 2, 2, 1, 1, 4, 1, 1, 1, 2, 1, 1, 1, 3, 3, 1, 1, 1, 2, 2, 3, 1, 1, 2, 1, 1, 1, 1, 1, 1, 1, 1, 1, 1, 2, 2, 2, 1, 1, 1, 1, 1, 2, 2, 2, 2, 2, 2, 2, 1, 2, 1, 1, 2, 1, 2, 1, 1, 1, 1, 1, 1, 1, 1, 1, 1, 1, 1, 1, 1

Corresponding number of selected TFs each gene is involved with:
(In the same order as above)
13, 14, 15, 15, 10, 15, 13, 10, 7, 7, 15, 7, 4, 7, 10, 15, 4, 4, 6, 6, 5, 4, 15, 6, 4, 9, 13, 15, 11, 14, 14, 3, 11, 10, 10, 9, 15, 15, 13, 6, 9, 9, 11, 13, 10, 10, 10, 11, 5, 3, 14, 12, 13, 2, 12, 8, 9, 5, 3, 3, 3, 13, 2, 4, 3, 10, 7, 4, 2, 8, 8, 8, 9, 9, 3, 3, 3, 5, 7, 8, 8, 6, 5, 5, 3, 15, 5, 9, 2, 7, 6, 3, 3, 7, 14, 10, 10, 10, 13, 13, 3, 3, 4, 1, 6, 4, 12, 6, 4, 11, 11, 3, 11, 3, 8, 4, 8, 8, 4, 4, 10, 3, 6, 10, 10, 3, 5, 5, 15, 15, 1, 10, 1, 14, 4, 14, 14, 4, 4, 13, 1, 13, 13, 13, 13, 5, 12, 12, 4, 4, 3, 1, 2, 2, 1, 8, 4, 11, 2, 11, 2, 2, 2, 7, 3, 3, 3, 7, 5, 4, 2, 10, 10, 10, 10, 10, 5, 5, 1, 3, 3, 3, 3, 3, 1, 2, 3, 6, 9, 2, 3, 1, 4, 2, 4, 8, 8, 1, 8, 4, 4, 2, 4, 8, 2, 5, 5, 5, 5, 15, 7, 7, 7, 2, 2, 2, 7, 7, 7, 2, 1, 13, 13, 2, 2, 4, 6, 6, 4, 4, 6, 3, 3, 12, 3, 4, 12, 12, 11, 1, 1, 1, 1, 1, 1, 5, 5, 5, 5, 5, 5, 1, 1, 1, 1, 1, 3, 3, 1, 1, 9, 1, 3, 4, 4, 4, 1, 2, 4, 8, 2, 2, 4, 4, 2, 2, 4, 4, 4, 4, 4, 2, 1, 4, 4, 2, 7, 1, 1, 1, 7, 1, 1, 1, 7, 1, 7, 3, 6, 2, 3, 3, 3, 3, 3, 1, 2, 2, 3, 2, 3, 3, 6, 1, 3, 2, 3, 3, 3, 1, 1, 5, 5, 1, 5, 1, 2, 2, 4, 2, 1, 2, 2, 2, 2, 4, 2, 2, 2, 2, 4, 2, 2, 2, 2, 2, 2, 2, 1, 2, 3, 3, 1, 3, 3, 3, 1, 1, 3, 3, 3, 1, 1, 1, 3, 3, 1, 3, 3, 3, 3, 1, 1, 1, 2, 2, 1, 1, 1, 1, 1, 2, 1, 1, 1, 1, 1, 1, 1, 1, 1, 1, 1, 2, 1, 1, 1, 1, 2, 1, 1, 2, 1, 1, 1, 1, 1, 1, 1, 1, 1, 1, 1, 1, 1

Phenopedia diseases ranked according to mean of effect sizes in cluster:
Neuroma, Acoustic | Neurilemmoma | Vestibulocochlear Nerve Diseases | Colorectal Neoplasms, Hereditary Nonpolyposis | Fanconi Anemia | Meningeal Neoplasms | DNA Repair-Deficiency Disorders | Neoplastic Syndromes, Hereditary | Nerve Sheath Neoplasms | Brain Neoplasms | Adenocarcinoma | Ataxia Telangiectasia | Anemia | Genomic Instability | Central Nervous System Neoplasms | Aneuploidy | Digestive System Neoplasms | Nervous System Neoplasms | Craniofacial Abnormalities | Carcinoma, Small Cell | Abnormalities | Abnormalities | Esophageal Neoplasms | Mouth Abnormalities | Jaw Abnormalities | Warts | Colorectal Neoplasms | Pancreatic Neoplasms | Intestinal Neoplasms | Gastrointestinal Neoplasms | Carcinoma, Neuroendocrine | Carcinoma, Squamous Cell | Neuroendocrine Tumors | Musculoskeletal Abnormalities | Neoplasms, Squamous Cell | Pancreatic Diseases | Cleft Palate | Xeroderma Pigmentosum | Tooth Abnormalities | Ataxia | Cleft Lip | Cell Transformation, Neoplastic | Radiation Injuries | Microsatellite Instability | Stomach Diseases | Carcinoma | Mouth Diseases | Stomach Neoplasms | Genital Diseases, Male | Tooth Diseases | Prostatic Neoplasms | Prostatic Diseases | Neoplasms, Radiation-Induced | Genital Neoplasms, Male | Gastrointestinal Diseases | Melanoma | Intestinal Diseases

Corresponding mean of effect sizes of each Phenopedia disease:
(In the same order as above)
3.08, 3.07, 3.05, 2.90, 2.85, 2.63, 2.59, 2.30, 2.29, 2.17, 2.17, 1.98, 1.94, 1.92, 1.91, 1.87, 1.84, 1.84, 1.69,

1.67, 1.66, 1.66, 1.61, 1.60, 1.59, 1.58, 1.57, 1.52, 1.50, 1.43, 1.41, 1.40, 1.40, 1.39, 1.38, 1.35, 1.31, 1.31, 1.24, 1.12, 1.08, 1.07, 1.01, 0.96, 0.96, 0.93, 0.86, 0.85, 0.75, 0.73, 0.64, 0.63, 0.53, 0.53, 0.27, -0.10, -0.16

Corresponding total number of genes of each Phenopedia disease:
(In the same order as above)
46, 47, 48, 51, 126, 105, 103, 112, 60, 132, 704, 70, 299, 161, 174, 70, 884, 182, 230, 76, 1.1K, 1.1K, 464, 196, 197, 354, 437, 219, 440, 735, 93, 306, 233, 256, 330, 318, 216, 45, 83, 102, 237, 79, 45, 105, 295, 1.2K, 485, 273, 662, 116, 504, 505, 31, 520, 1.2K, 182, 990

Corresponding number of genes of each Phenopedia disease with at least one TF hit (of selected TFs):
(In the same order as above)
13, 13, 13, 13, 33, 32, 23, 26, 16, 33, 119, 17, 50, 38, 46, 16, 139, 48, 41, 10, 167, 167, 77, 37, 37, 62, 70, 45, 70, 112, 13, 52, 33, 45, 56, 57, 37, 11, 16, 22, 38, 14, 12, 21, 43, 186, 70, 41, 91, 19, 73, 73, 7, 75, 169, 21, 127

Corresponding total number of TF hits (of selected TFs) for each Phenopedia disease (in all genes):
(In the same order as above)
81, 81, 81, 97, 175, 152, 143, 166, 87, 168, 640, 99, 244, 166, 206, 101, 738, 212, 229, 72, 892, 892, 416, 202, 202, 343, 380, 241, 380, 591, 87, 272, 218, 245, 291, 291, 202, 46, 84, 113, 203, 69, 52, 101, 240, 910, 373, 230, 466, 91, 354, 354, 29, 356, 838, 105, 627

Corresponding number of selected TFs each Phenopedia disease is involved with:
(In the same order as above)
15, 15, 15, 15, 15, 15, 15, 15, 15, 15, 15, 15, 15, 15, 15, 15, 15, 15, 15, 15, 15, 15, 15, 15, 15, 15, 15, 15, 15, 15, 15, 15, 15, 15, 15, 15, 15, 15, 15, 15, 15, 15, 15, 15, 15, 15, 15, 15, 15, 15, 15, 15, 15, 15, 15, 15, 15

TFs ranked according to mean of effect sizes in cluster:
V$E2F_03, V$E2F1_Q6_01, V$E2F_Q6_01, V$E2F1DP1RB_01, V$E2F4DP1_01, V$E2F1DP1_01, V$E2F4DP2_01, V$E2F_Q4_01, V$E2F1DP2_01, V$E2F_02, V$E2F_Q3_01, V$E2F_Q3, V$E2F1_Q4_01, V$E2F_Q4, V$E2F_Q6

Corresponding mean of effect sizes of each TF:
(In the same order as above)
2.02, 1.96, 1.92, 1.84, 1.75, 1.66, 1.51, 1.51, 1.48, 1.32, 1.31, 1.24, 1.23, 1.08, 0.89

Corresponding total number of TF hits for each TF (genome-wide):
(In the same order as above)
450, 447, 452, 448, 442, 450, 452, 432, 454, 442, 431, 442, 437, 438, 430

Corresponding total number of TF hits for each TF (in all genes in selected Phenopedia diseases):
(In the same order as above)
1157, 1162, 1145, 1141, 1106, 1106, 1064, 976, 1087, 1013, 929, 994, 941, 972, 931

Corresponding number of genes (of selected Phenopedia diseases) each TF is involved with:
(In the same order as above)
141, 150, 143, 136, 130, 147, 137, 129, 139, 131, 129, 147, 124, 149, 143

Corresponding number of selected Phenopedia diseases each TF is involved with:
(In the same order as above)
56, 56, 56, 56, 56, 56, 56, 56, 56, 56, 56, 56, 56, 56, 56

Disease groups of selected Phenopedia diseases ranked according to sum of effect sizes
Neoplasms | Nervous System Diseases | Signs and Symptoms | Musculoskeletal Diseases | Nervous

System Neoplasms | Digestive System Diseases | Gastrointestinal Diseases | Digestive System Neoplasms | Gastrointestinal Neoplasms | Central Nervous System Diseases | Congenital, Hereditary, and Neonatal Diseases and Abnormalities | Stomatognathic Diseases | Mouth Diseases | Jaw Diseases | Neuroendocrine Tumors | Soft Tissue Neoplasms | Sarcoma | Peripheral Nervous System Neoplasms | Peripheral Nervous System Diseases | Nerve Sheath Neoplasms | Neoplasms, Nerve Tissue | Central Nervous System Neoplasms | Abdominal Neoplasms | Neuroma | Neurilemmoma | Intestinal Neoplasms | Intestinal Diseases | DNA Repair-Deficiency Disorders | Bone Diseases | Brain Diseases | Neoplastic Syndromes, Hereditary | Genetic Diseases, Inborn | Musculoskeletal Abnormalities | Colorectal Neoplasms | Neoplasms, Glandular and Epithelial | Skin Diseases | Abnormalities | Craniofacial Abnormalities | Tremor | Movement Disorders | Dyskinesias | Basal Ganglia Diseases | Neuroma, Acoustic | Vestibulocochlear Nerve Diseases | Cranial Nerve Diseases | Colorectal Neoplasms, Hereditary Nonpolyposis | Genomic Instability | Pancreatic Diseases | Thrombocytopenia | Pancytopenia | Leukopenia | Leukocyte Disorders | Fanconi Anemia | Bone Marrow Diseases | Blood Platelet Disorders | Blood Coagulation Disorders | Anemia, Aplastic | Stomatognathic System Abnormalities | Spinal Diseases | Spinal Cord Neoplasms | Spinal Cord Diseases | Meningeal Neoplasms | Carcinoma | Male Urogenital Diseases | Genital Diseases, Male | Head and Neck Neoplasms | Brain Neoplasms | Adenoma, Oxyphilic | Adenocarcinoma | Vascular Diseases | Telangiectasis | Spinocerebellar Degenerations | Skin Diseases, Vascular | Peripheral Vascular Diseases | Cerebellar Diseases | Cerebellar Ataxia | Cardiovascular Diseases | Ataxia Telangiectasia | Tooth Diseases | Anemia | Chromosome Aberrations | Aneuploidy | Stomach Diseases | Carcinoma, Small Cell | Esophageal Neoplasms | Esophageal Diseases | Mouth Abnormalities | Maxillofacial Abnormalities | Jaw Abnormalities | Warts | Skin Diseases, Viral | Skin Diseases, Infectious | Pancreatic Neoplasms | Endocrine System Diseases | Endocrine Gland Neoplasms | Carcinoma, Neuroendocrine | Carcinoma, Squamous Cell | Neoplasms, Squamous Cell | Cleft Palate | Xeroderma Pigmentosum | Skin Abnormalities | Keratosis | Infant, Newborn, Diseases | Ichthyosis | Callosities | Prostatic Diseases | Tooth Abnormalities | Pelvic Neoplasms | Genital Neoplasms, Male | Ataxia | Cleft Lip | Neoplastic Processes | Cell Transformation, Neoplastic | Radiation Injuries | Microsatellite Instability | Stomach Neoplasms | Prostatic Neoplasms | Neoplasms, Radiation-Induced | Melanoma

Corresponding sum of effect sizes for each disease group of selected Phenopedia diseases
(In the same order as above)
626.74, 347.32, 272.73, 265.27, 254.90, 234.71, 214.49, 198.35, 147.86, 147.35, 144.13, 136.93, 136.93, 136.93, 134.43, 126.58, 126.58, 126.58, 126.58, 126.58, 126.58, 100.73, 99.09, 92.26, 92.26, 89.54, 87.21, 82.30, 82.15, 79.20, 77.97, 77.97, 70.11, 67.00, 59.66, 53.48, 49.71, 49.21, 46.62, 46.62, 46.62, 46.62, 46.16, 45.79, 45.79, 43.45, 43.20, 43.03, 42.70, 42.70, 42.70, 42.70, 42.70, 42.70, 42.70, 42.70, 42.70, 42.56, 39.45, 39.45, 39.45, 39.45, 39.01, 38.25, 38.25, 32.58, 32.58, 32.48, 32.48, 29.77, 29.77, 29.77, 29.77, 29.77, 29.77, 29.77, 29.77, 29.77, 29.54, 29.14, 28.09, 28.09, 27.08, 25.10, 24.21, 24.21, 23.94, 23.92, 23.92, 23.71, 23.71, 23.71, 22.82, 22.82, 22.82, 21.13, 21.05, 20.64, 19.68, 19.58, 19.58, 19.58, 19.58, 19.58, 19.58, 19.01, 18.65, 17.47, 17.47, 16.85, 16.13, 16.07, 16.07, 15.17, 14.39, 12.69, 9.55, 7.92, -1.55

Cluster for columns 1 to 4, rows 240 to 258

Phenopedia diseases

Werner Syndrome | Disease | Disease Susceptibility | Genetic Predisposition to Disease

TFs

V$GABP_B | V$ELK1_02 | V$IPF1_Q4_01 | V$NRF1_Q6 | V$CREB_01 | V$CREBATF_Q6 | V$CREBP1CJUN_01 | V$ATF_B | V$CREBP1_Q2 | V$CREB_02 | V$CREB_Q4_01 | V$CREB_Q4 | V$CREB_Q2 | V$CREB_Q2_01 | V$ATF_01 | V$ATF3_Q6 | V$ATF1_Q6 | V$ATF4_Q2 | V$TAXCREB_01

Information

All related TFs:
(List of all TFs that are related to any of the PWMs)
120-kDa, 47-kDa, ATF, ATF-1, ATF-2, ATF-4, ATF-a, ATF-like, ATF/CREB, ATF2, ATF2-isoform2, ATF3, ATF4, ATF5, ATF6, ATFa-isoform1, CRE-BP1, CRE-BP2, CREB, CREBbeta, CREMalpha, CREMbeta, CREMgamma, CREMtau, CREMtau1, CREMtau2, CREMtaualpha, Elk-1, Elk-1-isoform1, GABP-alpha, GABP-alpha:GABP-beta, GABP-beta1, GABP-beta2, IPF1, IPF1:Pbx, NRF-1, Tax, c-Jun, deltaCREB

Ranked gene list:
(All genes of the selected Phenopedia diseases with hits of any of the selected TFs, ranked according to the total number of TF hits)
PAX6, BACH2, RELB, BRAF, THADA, RXRB, IL10RA, GNAL, C16orf61, AHI1, PTPN6, PER1, MYBL2, FSTL4, FAM82A2, CREB5, SEMA3B, MGRN1, GATA2, FOSB, CCND2, ARG2, TSC2, TMEM120B, NTHL1, NF1, LTBP1, IKBKB, GPX4, FOS, FANCD2, EXO1, BIRC2, TMEM39A, NR4A2, IMPDH2, HECA, FN1, CNTNAP1, ATF3, UBQLN1, TSPAN8, GTF2H1, FADS2, CLSTN2, CDK4, CASP9, BCL6, ZC3H10, TBL3, STXBP3, STARD3NL, SPAG6, SNX5, SLC39A7, RRP1, PLCG1, PHF1, NDUFA10, JUND, THOC6, SREBF1, SDHB, PRR3, MYNN, MVK, KLF11, GTF2F1, GNL1, FRAT1, DOK1, CCR6, ALS2, GABARAP, TNFRSF12A, RASSF1, PPIL2, PBX2, NOTCH4, MUC1, MLF1, MEF2D, LDHA, JARID2, GRIN3B, GPSM3, CXCL16, CLDN6, CHGB, BAT5, AKT3, SLC18A2, RFC1, RAD51C, PLEKHG2, PCNA, PARP2, GNAS, GCHFR, GATA3, CYP27B1, AKT1, YWHAH, TNFRSF4, SYN1, SOCS1, PSENEN, PRKAR1A, PRC1, POLR2E, PINX1, PDZD8, NUP98, LGR5, IRGQ, GPR3, FADS1, DNAJC16, CSNK1G2, ARL5B, XRCC2, WNT10A, TRIM39, TDG, SYNGAP1, RAI1, PAK1, MCM10, FOXC1, FLT1, CUTA, CAMK2D, BTAF1, APTX, ZFYVE27, YME1L1, VAMP2, TACC3, OSM, NAT6, METTL1, MAF, CSNK1D, CNDP2, CDC42, ZNF184, UBE2H, SFRP2, RAD23A, DHX36, ZBTB37, YWHAZ, WDR37, UCN, TYROBP, STK38L, STAT3, SEPT2, RIPK2, RING1, REL, RECQL4, PPARD, POT1, POLS, PCK1, PARK2, PACRG, NOLC1, NFKBIB, NDUFB2, MRPL4, LOXL3, HSPA14, HSP90AB1, GAPDHS, FBXL20, FAT1, EPHA2, COX4NB, CDC37, BNIP3L, TIPRL, LY6G6F, HSD17B8, CENPE, TNFAIP1, TFCP2, SMAD7, RAGE, PRKCZ, MXI1, MLF2, KLK15, IL23A, HRAS, GTF2A1, DLX2, DBP, CHD7, TNFRSF6B, SQSTM1, SPTBN4, SH3TC1, SH3GL2, RUNX2, RCAN1, PPARGC1A, POLB, PNKP, PMS2, NR1H2, NINJ1, N4BP1, KLC1, JTV1, JAK1, HOMER1, ELOVL5, EGR1, CCNA2, CAMKK1, AMH, ZNRD1, VAPB, TMC6, SUV39H2, STRA13, SEZ6L2, RBBP8, PRKD2, ORMDL3, NFATC1, KLF13, GSDML, GSDMB, GRN, GLI1, EIF4EBP2, EGR2, EBF3, CHRNE, CD44, CALCB, C12orf43, ADRA2A, ZFP36, WNT10B, TEX14, SUPT3H, SIPA1, SHC1, RAD51L1, RAB3A, PRKG1, PRKAG2, POLD1, NFKBIE, MPG, HIRIP3, FOSL2, FAT, CHAF1A, BAT4, ADORA2A, TREX1, TRAF4, TPP1, TCIRG1, SST, SPG7, SLC25A1, SIK1, SH2B3, RTEL1, RORC, RAD18, PVRL2, PSPN, PNPLA2, PHLDB1, PDE7A, NDEL1, MYO1G, MDN1, LY6G5B, LSM2, LMO4, KNS2, KCTD15, JUN, HSPA1L, EPHA4, EIF3A, DVL1, CTCF, CRYAA, CREM, CALCA, ATRIP, APOM, AIRE, ADAT1, ZNF473, TEAD1, SGTA, PTPRU, PTPRCAP, PSMD3, PARD6A, OPA3, MYH10, IFRD1, HIVEP1, HDLBP, GOLPH3, EEF2, C7orf41, C6orf170, VPS37B, SIDT2, S1PR2, PFAS, LMCD1, KIAA1217, HOXA13, CMIP, CKB, CD2AP, CCNI, C4orf47, C3orf14, YEATS4, WT1, TXNL1, TMEM217, TM9SF3, THRA, TCF7, STK19, SPRED2, SOCS3, SMC5, SIK3, SGIP1, SF3A2, SDK1, SBF1, RGS2, PTS, PPP1R11, PPP1R10, PPM1D, PCNX, PARK7, NUBP2, NR6A1, NPPC, NP, NHEJ1, MSI2, MPDU1, MMAB, MEST, MAZ, MAOA, LPIN1, LBH, KIAA1683, JUNB, INSIG2, IL11, IKZF1, IKBKAP, IFITM3, HS3ST2, HMGCR, HDAC4, HCFC1, GSK3B, GATA4, FAM167A, F12, EIF3H, EGR4, EFNA3, DRD4, DOM3Z, DGCR14, CWF19L1, CSTF2T, CLPTM1L, CDKN1A, C2orf43, C12orf57, BRCA2, BAP1, AVPI1, ATF1, APEX1, ANKMY1, AGER, ADK, ADAR, ACSL5, ZNF34, SOX2, RBKS, MAPRE2, HPS4, DNAJB2, DAP, CREB3L2, C9orf3, BRE, XAB2, UMPS, TNFRSF18, SKI, RBM18, PDE7B, PARN, MRRF, JMJD2C, HAS1, GPM6B, GNE, GGT1, ELL2, CSPG5, CSNK2B, CDT1, C6orf47, C18orf1, BECN1, ZNF410, ZC3H3, VEGFB, TUBGCP6, TRIP12, TNNC1, TMEM129, TARBP2, SLC10A7, SIT1, SDHD, SBK1, RNMT, RHOG, RHBDF1, RARG, RARA, RANBP5, POLG2, PLCD3, PLCD1, PER2, PDLIM3, OAZ1, NUP214, NSUN6, MAL, MAGOHB, LZTS2, KIAA0020, IRAK3, HNRPDL, GRIN1, GNB5, GBA, GAB2, GAA, FAM119B, ERC1, EPHB2, ENO2, EIF2AK2, EGR3, DOCK9, DNMT1, DMBT1, DDX5, DDAH2, CTBP1, CREBBP, CHGA, CHD2, CDKN2C,

CDK5RAP2, CDK2, CCDC86, CARTPT, CAP1, C3orf26, C12orf30, BRD2, ATP2B4, ARF1, ANUBL1, AFF4, AANAT, ZZEF1, ZNF419, ZNF331, ZNF266, ZHX2, XRCC6, XPNPEP1, WNT7A, VDR, USP2, TNFSF13, TNFAIP3, TMEM127, TDP1, TAF4, TACR1, STK24, STAT4, SNAP25, SMUG1, SIAH2, SGMS1, RTN3, RPS19, RHOA, PPP1R1B, PPM1K, PPARGC1B, POLG, PLEKHA1, PKP4, PIP5K1B, NUDT1, NR3C1, NFKB2, NDUFB7, MYST4, MX1, MSH5, MRPS18B, MOG, MDM4, LRP1, LMNA, KLF12, IZUMO1, ITPRIP, ITPR3, IRF7, IRF1, HIST1H2AG, HHIP, FGFR1, FANK1, FAF1, DUSP2, DLST, DIDO1, CSTF1, COX18, CLASP2, CES2, CD81, CCDC148, CBR1, C19orf63, C16orf5, BTG1, BID, BANP, AURKA, ATG9B, APH1A, ADRA1A, ADAMTS6, ACAD10, ABCA7, SCAF1, RAB17, PTPRK, PRMT1, PRDX2, PPP2R2A, NOL4, MBNL2, LRRFIP1, HYAL1, HSPB6, FOXP1, FAHD2A, ASXL3, ACTR3B, ZWILCH, ZNF830, ZNF575, ZNF25, ZNF239, ZNF132, WNT11, WDR63, WARS, VPS52, VGF, VAMP3, USP20, UNC13D, UHRF1, UFD1L, UBL5, TYMP, TXN2, TSPO, TRIB1, TRH, TOMM40, TNRC6B, TNPO3, TNFSF12, TMF1, TMEM43, TH, TFDP1, TCTA, SUFU, STIM2, SOAT1, SNX9, SMC6, SLC39A9, SLC32A1, SLC27A5, SILV, SIK2, SHMT2, SECISBP2, RPL10, RNPEPL1, RNF39, RHOC, RFC3, RASD2, RAB5B, PTGDS, PTCH1, PSRC1, PSMB9, PSMB8, PROZ, PPM1A, PLXNA1, PLCB2, PLA2G4B, PIGG, PHRF1, PFKFB3, PDK1, PDGFB, PDF, PCNXL3, PAFAH1B2, NUPL2, NR2E1, NR1H3, NPC1, NOTCH1, NFIL3, NDUFV3, NADSYN1, MUT, MSX2, MSX1, MON1B, MAPT, MAPK11, MAP3K12, LYRM7, LTB4R, LRCH1, LLGL2, LBXCOR1, LAPTM4B, LAP3, KIAA1949, KIAA1542, KAZALD1, JAG1, ITPA, IRF6, IRAK4, ING1, HOXC4, HOXA1, HERPUD2, HDAC7, HDAC5, H6PD, GSK3A, GRP, GNAI2, GEMIN4, GALR2, FUT7, FRS3, FEZ1, FADD, ESCO2, EMILIN1, EMG1, ECE2, DYRK1A, DLX3, DLEC1, DHCR7, DDX21, DDIT3, DCTN1, DCP1A, CUX1, CUL9, CRTC2, CPT1B, CPT1A, COASY, CLIC1, CISH, CHRNB1, CDS2, CDK5R1, CDC45L, CD83, CD247, CALY, BSN, BRAP, BGLAP, BCL2L2, BCL2L11, BCCIP, ATG5, ARSA, ARPC1A, APBB1, ALOXE3, AGRP, ADAM8, ACTR5, ACSS2, ACP2, ABCF1, ABCE1, ABCA2, ZNF562, ZNF335, TRIM11, SCYL3, PGPEP1, KDM4C, HAS2, ETNK1, ENTPD5, BDP1, AIMP2, ZNF295, ZFYVE26, ZCCHC24, XRCC5, XPO5, XPC, WNK1, WISP1, VGLL4, UNG, UNC84B, UNC5B, UCP2, UBR3, UBR1, UBASH3B, UBAC2, TYSND1, TXNRD2, TSG101, TRIM26, TRIM17, TRADD, TNIP1, TNFSF8, TIMELESS, TGM1, TGFB1, TG, TBX4, TBX10, TBC1D22B, STAT1, SSTR3, SREBF2, SRBD1, SRA1, SND1, SLC30A9, SLC30A7, SLC2A9, SLC25A16, SLC23A2, SLC22A4, SLC18A3, SLC11A2, SLA, SH3PXD2A, SFRS5, SFRS3, SERPINB8, SELS, SCAP, SBNO2, SATB2, SART1, RTN4, RRP1B, RPS18, RGS9, RGS17, RFT1, REV1, RERE, RELL1, RELA, RB1, RASIP1, RAPGEF6, RAPGEF1, PTPN11, PTK2B, PTCH, PSTPIP1, PRX, PRKCH, PRKCD, PRKAB2, PPP1R12B, POU2F1, POGK, POFUT2, PIP4K2A, PIM1, PIK3CG, PGC, PGBD3, PDE11A, PCM1, PANK4, OPTN, NUMA1, NUCKS1, NTRK2, NRM, NRCAM, NR3C2, NR1D1, NDUFS8, NDUFA7, MYO9A, MYO7A, MUTYH, MTR, MTP18, MTHFS, MTHFD2, MTHFD1, MSRA, MRPL23, MFN1, MCAT, MBD1, MAPKAPK3, MAPKAP1, MADCAM1, LYN, LY6G5C, LRRC16A, LRP6, LEPREL2, LAG3, KLHL12, KLF6, KLF4, KLF16, KLF15, KIF6, KCNQ1, KALRN, JMJD1C, JAK3, ITPK1, ITGAE, ITGA4, IRS1, IRAK2, IL7R, IL13, IGFALS, IGF2AS, IGF2, IGF1R, IGF1, IFNGR1, ICAM5, HSD17B1, HK1, HIST1H3B, HIST1H2BJ, HINT1, HES1, HERC4, HEL308, HEATR5B, GTF2H3, GTDC1, GSTO2, GRAP2, GPSM2, GPR126, GPAM, GOSR2, GNRH2, GFRA1, GBF1, GABRB3, GABBR1, FPGS, FOXO3, FOSL1, FGFR1OP, FECH, FBXL17, EZH2, ETV6, ERCC6, ERCC3, ERCC1, EHMT1, E2F4, DPYSL2, DLL1, DCLRE1C, CYP1A1, CUGBP2, CTNNA2, CTLA4, CTH, COMT, COL5A2, CNTFR, CKS1B, CIDEB, CHRM5, CHAT, CGA, CEBPB, CDKN1B, CDH23, CDC34, CDC25B, CCNH, CCND1, CCDC55, CAPN10, CAMKV, CACNA1S, CACNA1C, CABIN1, C6orf26, C6orf134, C3orf67, C11orf30, BRCA1, BMP2K, BLM, BDKRB2, BCR, BAX, BAT3, BAT1, BANK1, B3GALT4, AVEN, AURKB, ATXN1, ATP1B1, ATP10A, ATG7, ATF4, ATAD1, ARVCF, ARRB2, ARNT, ARHGEF3, ARHGAP1, APOC2, APOA5, AP3B1, ANXA5, ANXA2, ANP32A, ALKBH2, ALDOA, ALDH7A1, ALDH18A1, AKAP13, ADRBK2, ADRB2, ADAM17, ACYP2, ACSS1, ACSL6, ABCC11, ABCB1, ZNF607, ZNF485, ZNF483, ZNF423, ZNF248, ZNF236, ZMYM2, ZFP64, ZEB1, ZDHHC8, ZC3H18, YPEL2, XYLT1, XPOT, XPO4, WNT2B, WHSC2, VAV3, USP37, USP13, UROD, ULK3, UIMC1, TXNDC5, TSR1, TSPAN9, TRPM7, TRIO, TRIM8, TRIM21, TRIB3, TRAPPC4, TPT1, TP53BP1, TOLLIP, TNIP2, TNIK, TMEM57, TIPARP, THEM2, TFR2, TFIP11, TFB2M, TFB1M, TDRD1, TBX6, TAPBP, TAF3, SYNGR1, STX1A, STCH, STAT6, STARD3, STAG1, SS18L1, SRR, SRD5A1, SPTB, SPSB3, SPRY4, SPOCK2, SPG11, SPAST, SP110, SMARCC1, SMAD5, SLTM, SLK, SLC9A9, SLC6A9, SLC6A1, SLC38A1, SLC35B2, SLC26A6, SLC25A4, SLC20A1, SLC17A7, SKIV2L, SHARPIN, SH2D2A, SFXN4, SFRS2IP, SFRS16, SF3A1, SETD7, SERTAD2, SERPINH1, SEC31B, SDHC, SDF4, SCFD1, SAR1A, SAMD8, S100A6, RSRC1,

RPS6KA1, RP5-1077B9.4, RP11-529I10.4, RNF160, RNF146, RHOBTB1, RHCE, RGS10, RDBP, RCOR3, RBAK, RAP1GAP2, RAN, RALGAPA1, RAB7A, PYY, PVRL1, PTK2, PTGER1, PTBP1, PSMD2, PSMA4, PSEN2, PROS1, PROC, PREB, PRCP, PPP1CB, PPIA, POLL, PMP22, PLEKHH2, PLCL2, PLA2G12A, PITX3, PITX2, PIGQ, PIGO, PIGF, PHACTR3, PGRMC2, PGM3, PFKP, PERLD1, PDLIM1, PDE4DIP, PDCD4, PBLD, PAX2, PALB2, PAK4, PACS2, ORC5L, OPRL1, OPA1, OLIG3, OGDHL, ODC1, OBFC1, NTRK1, NPM1, NPHP1, NOC3L, NLRP4, NLRP3, NLGN2, NKX2-5, NKX2-3, NKIRAS2, NHLRC1, NEURL, NEFH, NDUFV2, NDUFS6, NDUFS5, NDUFS1, NDUFB9, NDUFB8, NDUFB3, NDUFA5, NCOR1, NAPG, MYB, MTIF3, MTCH2, MST1R, MST1, MS, MPPE1, MLH3, MIF, MGST3, MFN2, MED13, MCOLN1, MC1R, MBTPS1, MAT2A, MAPK10, MANBA, MAGED2, MAGED1, MAFB, MADD, MAD1L1, LZTR1, LYRM4, LTBP4, LTBP2, LTB, LSP1, LRPAP1, LRBA, LMX1B, LMTK2, LIF, LHB, LDB1, LASP1, LAMP2, KRT18, KRI1, KNDC1, KIFAP3, KIAA0913, KEAP1, KCNIP2, KCNH2, ITGB1BP1, ITGA3, ISG15, IRF9, IRF8, IRF5, IPO5, INTS12, INS, INO80, IMPDH1, IL2RG, IL27, IL21R, IL16, IL11RA, IFNAR1, ID2, ICAM3, HSD11B2, HS1BP3, HPS1, HOXA9, HNRNPH3, HNRNPF, HMGB1, HEXA, HCRT, HBA1, H2AFY, GYLTL1B, GTPBP8, GSTCD, GRIN2D, GRIA2, GPR162, GPR132, GPA33, GNPDA2, GNB4, GNA12, GLUD1, GLG1, GEN1, GDI2, GCLC, GALT, GALR3, GALNT10, GAL, GADD45A, GABRB1, GABRA5, GABARAPL2, FUS, FST, FOXO1, FOXF1, FMR1, FLI1, FGFRL1, FGFR3, FGF14, FBXO36, FBXO3, FAM120B, FABP3, ENDOGL1, EN2, EFCAB6, ECGF1, DUSP6, DUSP27, DUSP13, DPH2, DIAPH1, DHX9, DDX20, DDX1, DDR1, DCTN5, DBH, DAXX, DAD1, DACT1, CYP2A13, CYP26B1, CXCR4, CXCL2, CXCL12, CTSD, CTRB1, CSNK1E, CSGALNACT2, CRIPT, CRH, CR2, CPA4, COX5B, CORO7, COMP, COL9A3, COL6A2, COL2A1, COL11A2, CNTF, CLPTM1, CLOCK, CISD1, CHRNB2, CHRM1, CHP, CHCHD1, CFD, CDKN2D, CDH15, CDCA3, CD72, CD5, CD46, CD19, CCL27, CCKAR, CCDC97, CCDC45, CCDC122, CBY1, CBLL1, CARS2, CARD9, CAMK2G, C6orf27, C6orf25, C4B, C1orf52, C1orf125, C1orf122, C16orf70, C13orf31, C11orf61, C10orf57, C10orf4, BLK, BIN2, BCL7C, BAT2, BAIAP2, AXIN1, ATXN7, ATRN, ATP1A1, ATG16L1, ATF5, ASPSCR1, ASCL1, ASCC3, ASCC1, ARID5B, ARHGAP10, ARFGAP3, AQP10, APOC4, AP2M1, ALOX12B, AIP, AIF1, ADAMTSL5, ACTC1, ACCN2, ACBD5, ABI1, ABCA3, ABAT, AATF, ZXDC, ZNF699, ZIC1, ZFYVE28, ZDHHC17, ZC3H11A, YWHAE, XPR1, WWP2, WIPI1, WDR27, WDHD1, ULK4, ULK2, UHRF1BP1, UBE2E2, TUBG1, TTC3, TTC23L, TTC17, TTBK2, TSC22D1, TRIM72, TRIM68, TMEM101, TMCO1, TLE4, TBX2, TAOK2, SYT8, SUMO3, STXBP1, SRP54, SPTAN1, SPIRE1, SPATA13, SPAG16, SP140, SNX16, SNAPC4, SLC39A13, SLC30A6, SLC19A2, SKP1, SIRT5, SHC4, SHANK2, SH3BP2, SGOL1, SGK493, SFRS9, SEC24C, SEC23A, SCAMP1, S100A5, RREB1, ROBO3, RNF5, RNF4, RNF165, RNF138, RNF123, RIPK4, RIMS3, RGS19, RGL2, RGL1, RFC2, REV1L, RENBP, RCSD1, RBM26, RASSF2, RABGAP1, PYGM, PUS7L, PRKAB1, PPP4C, PPP3R1, PPM1J, POMP, POLH, POLD2, PLXNB1, PLOD1, PIP4K2C, PIGP, PHACTR2, PFDN6, PEA15, PCMT1, PAX3, OVOL1, ORC3L, NOM1, NMNAT1, NKIRAS1, NDUFV1, NDUFS3, NDUFB10, NCOA7, NCK2, NAT9, NARS2, NARF, MYO1D, MTMR4, MRC2, MLYCD, MARK2, MAP3K7, MAP1B, MAN1C1, MAN1B1, LY6G6C, LTB4R2, LRRC8C, LMO1, LGALS1, LEMD2, KTN1, KLF7, KIAA0319, KCNK3, JSRP1, JARID1B, IVNS1ABP, ITFG3, INTS7, INPP4A, INHBB, ICA1, HSD17B7, HOXD12, HOXD11, HOXD10, HOXB9, HOXB7, HOOK1, HMHA1, HLA-DOA, HES6, HCG27, GP5, GOLGA1, GNAO1, GGT7, GFI1, GART, FOXO1A, FOXJ3, FNTA, FLNC, FILIP1L, FBXO8, FBXL22, FASN, FAM78B, FAM46A, FAM105B, ERBB2IP, ENO3, ENO1, EMD, ELK3, EEF2K, DUSP12, DR1, DOPEY2, DOCK3, DNAJB1, DLX1, DLG4, DHX30, DGKD, DGAT1, CYTIP, CYFIP2, CPNE5, COQ2, COMMD5, COL25A1, CLUL1, CHKB, CDCA5, CDC42BPB, CDC20, CDC16, CCDC60, CCDC25, CAPN3, CAPG, CALCOCO1, C6orf125, C3orf18, C1orf183, C1orf105, C16orf62, C16orf46, C15orf53, C12orf26, C12orf10, BRSK1, BRD9, BAMBI, ATXN7L2, ATP5G2, ATG2A, ATG10, ARID3B, ARHGEF1, ARHGAP18, AP1B1, ALDH3A2, AGXT, AGPAT4, AGPAT3, AGPAT1, AGL, ADSS, ADAM22, ADAM10, ACTC, ABR, AAK1, ZNF767, ZNF667, ZNF664, ZNF652, ZNF259, ZFP161, ZC3H7B, ZBTB43, ZBTB40, ZBTB16, ZAP70, XPO6, VIM, UXS1, USP34, USP25, USF2, ULBP3, UBR2, TULP1, TTC15, TTBK1, TSPAN2, TRPC1, TRIM9, TRIM44, TRAPPC10, TRAIP, TPST1, TPK1, TOR3A, TOPORS, TOP1MT, TMEM9, TMEM146, TMEM135, TMCO4, TMBIM6, THOC7, TDRD9, TCP11L1, TBCA, TBC1D22A, TAOK3, TAF1B, STT3B, ST8SIA4, SSBP2, SPTBN1, SPSB1, SPOP, SPATA5L1, SOBP, SNTA1, SMG6, SMARCA4, SLCO3A1, SLC6A17, SLC4A4, SLC43A2, SLC38A8, SLC25A42, SLAIN2, SKAP1, SIX6, SIPA1L3, SH3BGR, SFRS8, SEMA6C, SELI, SEC31A, SEC24D, SCFD2, SCAMP4, SCAMP2, SATB1, RTF1, RPL35A, RPL11, RNASET2, RHOH, RGS11, RCBTB2, RASSF5, RAP1B, RALB, RALA, RABEPK,

RAB35, RAB10, PTPN14, PTGIR, PRR5-ARHGAP8, PRR5, PRPH, PRKACB, PRDX5, PRDM4, PRAGMIN, PPP1R12A, PPM1B, PPIE, PPHLN1, PPAP2A, PPA2, POMT1, POMGNT1, POLR3B, POFUT1, PLXNA4, PLEKHM3, PLCXD2, PLCB3, PLAGL2, PKDCC, PIP4K2B, PHLDB2, PHLDA2, PHKB, PHF2, PHF12, PHC2, PEX5L, PDGFA, PDE8B, PCDHGA3, PCDHGA2, PAXIP1, PANK2, PAFAH2, PACS1, ODF2, NUP50, NUDT12, NUDCD3, NSUN2, NOLA1, NMNAT2, NFIA, NFE2L3, NEK6, NDUFA3, NCAPD3, NCAPD2, NAV2, NADK, N6AMT1, MUTED, MTX2, MTCH1, MRPS18C, MRPL22, MPZ, MPHOSPH6, MOCOS, MLX, MKRN1, MKNK1, MICAL2, MFAP4, MEI1, MDH2, MAP9, MAP3K14, MAP2K6, MAP1D, LTBP3, LRRFIP2, LRIG1, LRFN2, LPPR5, LPPR4, LOC729991-MEF2B, LMF1, LINS1, LHFPL2, LASS2, KISS1R, KIF21B, KIF13B, KIAA1467, KIAA1257, KIAA0427, KIAA0226, KDM4B, KDM2B, KCNK6, KCNJ14, ITFG1, ISPD, IPO11, IP6K1, ING5, IL28B, IL17C, IFI16, IDDM12, HYLS1, HUNK, HSF2BP, HS3ST4, HPCAL4, HMGA1, HMBS, HIPK2, HIP1R, HGFAC, HELQ, HBS1L, GRK6, GRAMD3, GOLGA3, GNAQ, GMPPB, GLT1D1, GLCE, GIMAP8, GBAS, GAPDH, FYCO1, FUT8, FUBP3, FTL, FTH1, FSCN2, FLCN, FGF9, FEM1A, FBXO38, FARS2, FAM24B, FAM18B2, FAM184A, FAM176A, FAM173B, FAM165B, FAM120A, EXD1, ETFA, EIF4H, EIF2B5, EIF2B2, EIF2B1, DYNLL1, DUSP23, DUSP10, DTD1, DNALI1, DNAJC8, DNAI1, DLGAP2, DIS3L2, DDOST, DDI2, DCAF5, CUL3, CTF1, CROT, COX10, CORO2B, CNTLN, CNBP, CMT1A, CMPK2, CLRN1, CLN3, CLIC5, CLGN, CLCN2, CILP2, CHID1, CHD6, CHD1, CDKL3, CD96, CD320, CCT5, CCS, CCNT2, CCM2, CCDC92, CCDC64, CCDC13, CCDC11, CAPZB, CANT1, CAD, CACNA2D4, CACHD1, C9orf25, C8orf37, C7orf60, C6orf1, C3orf31, C2orf77, C2orf63, C20orf7, C1orf101, C1QTNF1, C17orf42, C16orf75, C11orf79, C10orf137, BOD1L, BCL2L14, BCKDHA, B3GALTL, ATXN2L, ATPIF1, ATP11A, ASAH1, ARMC9, ARID3A, ANO9, ANKRD17, AMZ2, AMPD3, AMPD2, AMIGO3, AMICA1, ALG8, AIPL1, ADPGK, ADCY8, ADAM9, ADAM15, ACOT11, ABHD5, ABHD10, ABCD3, ABCB8

Corresponding total number of TF hits:
(For each gene listed above, the total number of TF hits for any of the selected TFs, multiplied by the number of selected Phenopedia diseases containing that gene)
60, 60, 56, 56, 52, 52, 52, 52, 52, 52, 48, 48, 48, 48, 48, 48, 45, 45, 45, 45, 45, 45, 44, 44, 44, 44, 44, 44, 44, 44, 44, 44, 44, 42, 42, 42, 42, 42, 42, 42, 40, 40, 40, 40, 40, 40, 40, 40, 39, 39, 39, 39, 39, 39, 39, 39, 39, 39, 39, 39, 36, 36, 36, 36, 36, 36, 36, 36, 36, 36, 36, 36, 36, 34, 33, 33, 33, 33, 33, 33, 33, 33, 33, 33, 33, 33, 33, 33, 33, 33, 33, 32, 32, 32, 32, 32, 32, 32, 32, 32, 32, 32, 30, 30, 30, 30, 30, 30, 30, 30, 30, 30, 30, 30, 30, 30, 30, 30, 30, 30, 28, 28, 28, 28, 28, 28, 28, 28, 28, 28, 28, 28, 28, 28, 27, 27, 27, 27, 27, 27, 27, 27, 27, 27, 27, 26, 26, 26, 26, 26, 24, 24, 24, 24, 24, 24, 24, 24, 24, 24, 24, 24, 24, 24, 24, 24, 24, 24, 24, 24, 24, 24, 24, 24, 24, 24, 24, 24, 24, 24, 24, 24, 22, 22, 22, 22, 21, 21, 21, 21, 21, 21, 21, 21, 21, 21, 21, 21, 21, 21, 20, 20, 20, 20, 20, 20, 20, 20, 20, 20, 20, 20, 20, 20, 20, 20, 20, 20, 20, 20, 20, 20, 20, 18, 18, 18, 18, 18, 18, 18, 18, 18, 18, 18, 18, 18, 18, 18, 18, 18, 18, 18, 18, 18, 18, 18, 18, 17, 16, 16, 16, 16, 16, 16, 16, 16, 16, 16, 16, 16, 16, 16, 16, 16, 16, 15, 15, 15, 15, 15, 15, 15, 15, 15, 15, 15, 15, 15, 15, 15, 15, 15, 15, 15, 15, 15, 15, 15, 15, 15, 15, 15, 15, 15, 15, 15, 15, 15, 15, 15, 15, 15, 15, 14, 14, 14, 14, 14, 14, 14, 14, 14, 14, 14, 14, 14, 14, 14, 14, 13, 13, 13, 13, 13, 13, 13, 13, 13, 13, 13, 13, 13, 12, 12, 12, 12, 12, 12, 12, 12, 12, 12, 12, 12, 12, 12, 12, 12, 12, 12, 12, 12, 12, 12, 12, 12, 12, 12, 12, 12, 12, 12, 12, 12, 12, 12, 12, 12, 12, 12, 12, 12, 12, 12, 12, 12, 12, 12, 12, 12, 12, 12, 12, 12, 12, 12, 12, 12, 12, 12, 12, 12, 12, 12, 12, 12, 12, 12, 12, 12, 12, 12, 12, 12, 12, 12, 12, 11, 11, 11, 11, 11, 11, 11, 11, 11, 11, 10, 10, 10, 10, 10, 10, 10, 10, 10, 10, 10, 10, 10, 10, 10, 10, 10, 10, 10, 10, 9, 9, 9, 9, 9, 9, 9, 9, 9, 9, 9, 9, 9, 9, 9, 9, 9, 9, 9, 9, 9, 9, 9, 9, 9, 9, 9, 9, 9, 9, 9, 9, 9, 9, 9, 9, 9, 9, 9, 9, 9, 9, 9, 9, 9, 9, 9, 9, 9, 9, 9, 9, 9, 9, 9, 9, 9, 9, 9, 9, 9, 9, 9, 9, 9, 9, 8, 8, 8, 8, 8, 8, 8, 8, 8, 8, 8, 8, 8, 8, 8, 8, 8, 8, 8, 8, 8, 8, 8, 8, 8, 8, 8, 8, 8, 8, 8, 8, 8, 8, 8, 8, 8, 8, 8, 8, 8, 8, 8, 8, 8, 8, 8, 8, 8, 8, 8, 8, 8, 8, 8, 8, 8, 8, 8, 8, 8, 8, 8, 8, 8, 8, 8, 8, 8, 8, 8, 8, 8, 8, 8, 8, 8, 7, 7, 7, 7, 7, 7, 7, 7, 7, 7, 7, 7, 7, 7, 7, 6, 5, 5, 5, 5, 5, 5, 5, 5, 5, 5, 5, 4,

4, 4, 4, 4, 4, 4, 4, 4, 4, 4, 4, 4, 4, 4, 4, 4, 4, 4, 3, 3, 3, 3, 3, 3, 3, 3, 3, 3, 3, 3, 3, 3, 3, 3, 3, 3, 3, 3, 3, 3, 3, 3,
3, 3, 3, 3, 3, 3, 3, 3, 3, 3, 3, 3, 3, 3, 3, 3, 3, 3, 3, 3, 3, 3, 3, 3, 3, 3, 3, 3, 3, 3, 3, 3, 3, 3, 3, 3, 3, 3, 3, 3, 3, 3,
3, 3, 3, 3, 3, 3, 3, 3, 3, 3, 3, 3, 3, 3, 3, 3, 3, 3, 3, 3, 3, 3, 3, 3, 3, 3, 3, 3, 3, 3, 3, 3, 3, 3, 3, 3, 3, 3, 3, 3, 3, 3,
3, 3, 3, 3, 3, 3, 3, 3, 3, 3, 3, 3, 3, 3, 3, 3, 3, 3, 3, 3, 3, 3, 3, 3, 3, 3, 3, 3, 3, 3, 3, 3, 3, 3, 3, 3, 3, 3, 3, 3, 3, 3,
3, 3, 3, 3, 3, 3, 3, 3, 3, 3, 3, 3, 3, 3, 3, 3, 3, 3, 3, 3, 3, 3, 3, 3, 3, 3, 3, 3, 3, 3, 3, 3, 3, 3, 3, 3, 3, 3, 3, 3, 3, 3,
3, 3, 3, 3, 3, 3, 3, 3, 3, 3, 3, 3, 3, 3, 3, 3, 3, 3, 3, 3, 3, 3, 3, 3, 3, 3, 3, 3, 3, 3, 3, 3, 3, 3, 3, 3, 3, 3, 3, 3, 3, 3,
3, 3, 3, 3, 3, 3, 3, 3, 3, 3, 3, 3, 3, 3, 3, 3, 3, 3, 3, 3, 3, 3, 3, 3, 3, 3, 3, 3, 3, 3, 3, 3, 3, 3, 3, 3, 3, 3, 3, 3, 3, 3,
3, 3, 3, 3, 3, 3, 3, 3, 3, 3, 3, 3, 3, 3, 3, 3, 3, 3, 3, 3, 3, 3, 3, 3, 3, 3, 3, 3, 3, 3, 3, 3, 3, 3, 3, 3, 3, 3, 3, 3, 3, 3,
3, 3, 3, 3, 3, 3, 3, 3, 3, 3, 3, 3, 3, 3, 3, 3, 3, 3, 3, 3, 3, 3, 3, 3, 3, 3, 3, 3, 3, 3, 3, 3, 3, 3, 3, 3, 3, 3, 3, 3, 3, 3,
3, 3, 3, 3, 3, 3, 3, 3, 3, 3, 3, 3, 3, 3, 3, 3, 3, 3, 3, 3, 3, 3, 3, 3, 3, 3, 3, 3, 3, 3, 3, 3, 3, 3, 3, 3, 3, 2, 2, 2, 2, 2,
2, 2, 2, 2, 2, 2, 2, 2, 2, 2, 2, 2, 2, 2, 2, 2, 2, 2, 2, 2, 2, 2, 2, 2, 2, 2, 2, 2, 2, 2, 2, 2, 2, 2, 2, 2, 2, 2, 2, 2, 2, 2,
2, 2, 2, 2, 2, 2, 2, 2, 2, 2, 2, 2, 2, 2, 2, 2, 2, 2, 2, 2, 2, 2, 2, 2, 2, 2, 2, 2, 2, 2, 2, 2, 2, 2, 2, 2, 2, 2, 2, 2, 2, 2,
2, 2, 2, 2, 2, 2, 2, 2, 2, 2, 2, 2, 2, 2, 2, 2, 2, 2, 2, 2, 2, 2, 2, 2, 2, 2, 2, 2, 2, 2, 2, 2, 2, 2, 2, 2, 2, 2, 2, 2, 2, 2,
2, 2, 2, 2, 2, 2, 2, 2, 2, 2, 2, 2, 2, 2, 2, 2, 2, 2, 2, 2, 2, 2, 2, 2, 2, 2, 2, 2, 2, 2, 2, 2, 2, 2, 2, 2, 2, 2, 2, 2, 2, 2,
2, 2, 2, 2, 2, 2, 2, 2, 2, 2, 2, 2, 2, 2, 2, 2, 2, 2, 2, 2, 2, 2, 2, 2, 2, 2, 2, 2, 2, 2, 2, 2, 2, 2, 2, 2, 2, 2, 2, 2, 2, 2,
2, 2, 2, 2, 2, 2, 2, 2, 2, 2, 1, 1, 1, 1, 1, 1, 1, 1, 1, 1, 1, 1, 1, 1, 1, 1, 1, 1, 1, 1, 1, 1, 1, 1, 1, 1, 1, 1, 1, 1, 1, 1,
1, 1, 1, 1, 1, 1, 1, 1, 1, 1, 1, 1, 1, 1, 1, 1, 1, 1, 1, 1, 1, 1, 1, 1, 1, 1, 1, 1, 1, 1, 1, 1, 1, 1, 1, 1, 1, 1, 1, 1, 1, 1,
1, 1, 1, 1, 1, 1, 1, 1, 1, 1, 1, 1, 1, 1, 1, 1, 1, 1, 1, 1, 1, 1, 1, 1, 1, 1, 1, 1, 1, 1, 1, 1, 1, 1, 1, 1, 1, 1, 1, 1, 1, 1,
1, 1, 1, 1, 1, 1, 1, 1, 1, 1, 1, 1, 1, 1, 1, 1, 1, 1, 1, 1, 1, 1, 1, 1, 1, 1, 1, 1, 1, 1, 1, 1, 1, 1, 1, 1, 1, 1, 1, 1, 1, 1,
1, 1, 1, 1, 1, 1, 1, 1, 1, 1, 1, 1, 1, 1, 1, 1, 1, 1, 1, 1, 1, 1, 1, 1, 1, 1, 1, 1, 1, 1, 1, 1, 1, 1, 1, 1, 1, 1, 1, 1, 1, 1,
1, 1, 1, 1, 1, 1, 1, 1, 1, 1, 1, 1, 1, 1, 1, 1, 1, 1, 1, 1, 1, 1, 1, 1, 1, 1, 1, 1, 1, 1, 1, 1, 1, 1, 1, 1, 1, 1, 1, 1, 1, 1,
1, 1, 1, 1, 1, 1, 1, 1, 1, 1, 1, 1, 1, 1, 1, 1, 1, 1, 1, 1, 1, 1, 1, 1, 1, 1, 1, 1, 1, 1, 1, 1, 1, 1, 1, 1, 1, 1, 1, 1, 1, 1,
1, 1, 1, 1, 1, 1, 1, 1, 1, 1, 1, 1, 1, 1, 1, 1, 1, 1, 1, 1, 1, 1, 1, 1, 1, 1, 1, 1, 1, 1, 1, 1, 1, 1, 1, 1, 1, 1, 1, 1, 1, 1,
1, 1, 1, 1, 1

Corresponding number of selected Phenopedia diseases each gene is involved with:
(In the same order as above)
4, 4, 4, 4, 4, 4, 4, 4, 4, 4, 3, 3, 4, 4, 3, 4, 3, 3, 3, 3, 3, 3, 4, 4, 4, 4, 4, 4, 4, 4, 4, 4, 3, 3, 3, 3, 3, 3, 3, 4, 4,
4, 4, 4, 4, 4, 4, 3, 3, 3, 3, 3, 3, 3, 3, 3, 3, 3, 3, 4, 4, 3, 4, 3, 3, 3, 3, 3, 4, 4, 2, 3, 3, 3, 3, 3, 3, 3, 3, 3, 3,
3, 3, 3, 3, 3, 3, 3, 4, 4, 4, 2, 4, 4, 4, 2, 4, 4, 4, 3, 3, 3, 3, 3, 3, 3, 3, 3, 3, 3, 3, 2, 3, 3, 2, 3, 4, 2, 2, 4, 2, 2,
2, 4, 4, 4, 2, 4, 4, 2, 3, 3, 3, 3, 3, 3, 3, 3, 3, 3, 2, 2, 2, 2, 2, 3, 4, 4, 3, 3, 3, 4, 4, 4, 2, 4, 4, 4, 4, 3, 4, 4, 4,
3, 4, 2, 3, 2, 3, 3, 3, 3, 3, 3, 3, 2, 2, 2, 2, 2, 2, 3, 3, 3, 3, 3, 3, 3, 3, 3, 3, 3, 3, 3, 3, 4, 4, 4, 4, 4, 4, 4, 4, 4, 4,
4, 4, 4, 2, 4, 4, 4, 4, 4, 2, 4, 4, 2, 3, 3, 3, 2, 3, 2, 3, 3, 3, 3, 2, 3, 3, 3, 3, 3, 3, 3, 3, 3, 3, 3, 1, 2, 2, 4, 4, 4,
4, 2, 4, 4, 4, 4, 4, 2, 2, 2, 4, 4, 4, 3, 1, 1, 3, 3, 3, 3, 1, 3, 3, 1, 3, 3, 3, 1, 3, 1, 3, 1, 3, 3, 3, 3, 3, 3, 3, 3, 3, 3,
1, 3, 3, 3, 3, 3, 3, 3, 3, 1, 1, 1, 1, 2, 2, 1, 1, 1, 1, 2, 2, 1, 1, 1, 1, 1, 1, 1, 1, 1, 1, 1, 1, 1, 1, 1, 1, 2, 4, 3, 3,
3, 3, 3, 3, 4, 3, 3, 3, 4, 1, 4, 3, 4, 3, 2, 3, 1, 4, 3, 4, 1, 3, 3, 4, 1, 4, 1, 4, 2, 4, 3, 3, 1, 1, 4, 3, 3, 3, 2, 3, 4, 3,
1, 4, 3, 4, 4, 2, 2, 3, 4, 3, 3, 4, 3, 3, 4, 1, 3, 4, 3, 3, 4, 4, 2, 4, 4, 3, 4, 1, 1, 1, 1, 1, 1, 1, 1, 1, 1, 2, 1, 2, 2, 1,
2, 1, 1, 2, 2, 1, 2, 1, 1, 2, 2, 2, 2, 2, 2, 1, 3, 3, 1, 3, 3, 1, 3, 3, 3, 3, 3, 1, 3, 1, 3, 3, 3, 3, 1, 3, 3, 1, 3, 1, 1, 3,
1, 3, 3, 3, 3, 3, 3, 3, 3, 3, 1, 1, 3, 3, 3, 3, 3, 3, 3, 3, 3, 3, 3, 3, 1, 3, 3, 3, 3, 3, 3, 1, 3, 3, 3, 3, 3, 1, 3, 1, 1, 1,
1, 4, 4, 4, 4, 4, 2, 4, 4, 2, 4, 4, 4, 4, 4, 4, 4, 2, 4, 4, 4, 4, 4, 2, 4, 4, 4, 1, 4, 4, 4, 4, 1, 4, 4, 4, 2, 4, 4, 4, 4, 4,
2, 4, 4, 2, 4, 2, 4, 4, 4, 4, 2, 4, 4, 4, 1, 1, 4, 4, 1, 4, 1, 1, 2, 4, 1, 4, 4, 4, 4, 1, 4, 4, 1, 1, 1, 1, 1, 1, 1, 1, 1, 1,
1, 1, 1, 1, 1, 2, 3, 3, 3, 3, 3, 3, 1, 3, 3, 1, 3, 3, 3, 3, 3, 2, 2, 3, 3, 3, 3, 3, 3, 3, 3, 1, 2, 3, 3, 3, 3, 1, 3, 3, 2, 2,
2, 3, 2, 1, 3, 3, 3, 1, 3, 3, 2, 2, 2, 3, 3, 3, 3, 3, 3, 1, 3, 3, 3, 3, 3, 3, 3, 3, 2, 3, 3, 1, 2, 3, 3, 3, 3, 3, 3, 3, 3, 3,
3, 3, 1, 2, 3, 3, 3, 3, 3, 3, 1, 2, 3, 3, 3, 3, 3, 3, 2, 3, 3, 3, 3, 2, 2, 3, 3, 2, 3, 3, 3, 3, 3, 3, 3, 3, 3, 3, 3, 3, 3, 3,
3, 3, 3, 3, 1, 3, 2, 3, 3, 3, 3, 3, 3, 1, 3, 3, 3, 3, 3, 3, 3, 3, 3, 3, 1, 2, 3, 3, 2, 2, 3, 2, 3, 3, 3, 2, 3, 1, 1, 1, 1,
1, 1, 1, 1, 1, 1, 1, 1, 1, 4, 4, 4, 4, 4, 4, 4, 4, 2, 4, 4, 2, 2, 4, 4, 4, 4, 4, 2, 1, 4, 4, 4, 4, 4, 4, 4, 2, 2, 1, 4, 4, 4,
4, 4, 1, 2, 2, 4, 4, 4, 4, 4, 4, 4, 4, 2, 2, 4, 4, 4, 4, 2, 2, 4, 4, 2, 4, 4, 1, 4, 4, 1, 4, 4, 1, 2, 4, 4, 4, 2, 4, 1, 4, 4,
4, 4, 4, 2, 1, 4, 4, 4, 4, 4, 4, 4, 2, 4, 4, 4, 4, 2, 4, 4, 2, 2, 1, 2, 4, 4, 4, 4, 4, 4, 4, 4, 2, 2, 2, 2, 2, 4, 2, 4, 2, 4,
4, 4, 4, 2, 4, 2, 2, 2, 4, 4, 4, 4, 4, 4, 4, 4, 4, 4, 4, 4, 4, 4, 4, 4, 4, 4, 4, 4, 4, 1, 2, 2, 2, 4, 4, 4, 4, 4, 4, 4, 2, 4,
4, 2, 4, 4, 4, 4, 4, 4, 2, 2, 4, 4, 1, 4, 4, 4, 4, 4, 4, 2, 4, 2, 4, 4, 4, 4, 4, 4, 4, 4, 4, 1, 2, 4, 4, 4, 1, 4, 4, 4, 4, 4,
4, 2, 4, 2, 4, 4, 4, 2, 2, 1, 4, 4, 2, 4, 4, 4, 4, 4, 4, 4, 2, 4, 2, 4, 4, 4, 1, 2, 4, 4, 2, 4, 4, 2, 4, 4, 4, 4, 4, 2, 4, 2,
2, 4, 4, 4, 4, 4, 4, 4, 4, 2, 4, 3, 3, 1, 3, 3, 3, 1, 3, 3, 3, 1, 3, 3, 1, 3, 3, 1, 3, 3, 1, 3, 3, 3, 3, 1, 3, 3, 3, 3, 3, 3,
3, 1, 3, 3, 3, 3, 3, 3, 3, 3, 3, 3, 3, 3, 3, 3, 3, 3, 3, 3, 1, 3, 3, 3, 3, 1, 3, 3, 1, 3, 3, 3, 3, 3, 3, 3, 3, 3, 1, 3,
3, 3, 3, 3, 3, 3, 3, 3, 1, 3, 1, 1, 1, 3, 3, 3, 3, 1, 3, 3, 3, 3, 3, 3, 3, 1, 3, 3, 3, 3, 3, 3, 3, 1, 3, 1, 1, 3, 3, 3, 3, 1,
3, 3, 3, 3, 3, 3, 3, 3, 3, 3, 3, 3, 3, 3, 3, 3, 1, 3, 1, 3, 3, 3, 3, 3, 1, 3, 3, 3, 3, 3, 3, 3, 3, 3, 3, 3, 3, 3, 3, 3, 1,
3, 3, 3, 3, 3, 3, 1, 1, 3, 3, 3, 3, 3, 3, 3, 3, 3, 3, 3, 3, 3, 3, 3, 3, 3, 3, 3, 3, 3, 3, 3, 3, 3, 1, 3, 1, 3, 3, 3, 3, 3,

1, 3, 3, 3, 3, 3, 3, 3, 1, 3, 3, 3, 3, 3, 3, 3, 3, 3, 3, 3, 3, 3, 3, 3, 3, 3, 1, 3, 3, 3, 1, 3, 3, 1, 3, 3, 3, 3, 3, 3, 3, 3,
3, 3, 3, 3, 1, 3, 3, 3, 3, 3, 3, 3, 3, 1, 3, 3, 3, 3, 3, 3, 3, 3, 3, 3, 3, 1, 3, 3, 3, 3, 1, 3, 3, 3, 3, 3, 3, 3, 3, 3, 3, 3,
1, 3, 3, 1, 1, 1, 3, 3, 3, 3, 1, 3, 3, 3, 1, 3, 3, 3, 3, 3, 3, 3, 3, 1, 3, 3, 3, 3, 3, 3, 3, 3, 3, 3, 3, 3, 3, 3, 3, 1, 3, 3,
3, 3, 3, 3, 3, 3, 3, 3, 3, 3, 1, 3, 3, 3, 3, 3, 3, 3, 3, 3, 3, 3, 1, 3, 3, 1, 1, 3, 3, 3, 3, 3, 3, 3, 3, 3, 3, 3, 3, 3, 3, 1,
3, 3, 3, 1, 3, 3, 3, 3, 3, 3, 3, 3, 3, 3, 3, 1, 3, 3, 3, 3, 3, 3, 3, 3, 3, 3, 3, 3, 3, 3, 1, 2, 2, 1, 1, 2, 2, 1, 1, 1, 1, 1,
2, 2, 2, 1, 2, 1, 1, 1, 2, 2, 2, 2, 1, 2, 2, 2, 2, 1, 2, 1, 1, 1, 1, 2, 2, 2, 2, 2, 2, 2, 2, 1, 2, 1, 2, 1, 1, 2, 2, 1, 1, 1,
2, 1, 2, 2, 1, 1, 1, 1, 1, 2, 2, 2, 1, 2, 2, 1, 2, 1, 1, 1, 1, 1, 2, 2, 1, 1, 1, 2, 1, 2, 2, 2, 1, 2, 2, 1, 2, 2, 1, 2, 1, 1,
1, 1, 1, 1, 2, 1, 1, 1, 1, 2, 1, 2, 1, 1, 2, 1, 1, 1, 2, 1, 1, 1, 2, 2, 2, 2, 2, 2, 2, 2, 2, 1, 1, 2, 2, 1, 2, 2, 2, 2, 2, 1,
1, 1, 2, 2, 2, 2, 1, 1, 1, 2, 2, 2, 1, 2, 1, 1, 2, 1, 2, 2, 2, 1, 1, 1, 1, 1, 1, 1, 2, 1, 1, 2, 1, 2, 2, 1, 1, 1, 1, 2, 1, 1,
2, 2, 1, 2, 1, 1, 2, 2, 2, 1, 2, 2, 1, 2, 1, 2, 1, 2, 1, 2, 1, 2, 1, 1, 2, 1, 2, 1, 1, 1, 2, 2, 2, 2, 1, 1, 1, 2, 2, 2, 2, 1,
2, 1, 2, 1, 1, 1, 1, 1, 1, 1, 1, 1, 1, 1, 1, 1, 1, 1, 1, 1, 1, 1, 1, 1, 1, 1, 1, 1, 1, 1, 1, 1, 1, 1, 1, 1, 1, 1, 1, 1, 1, 1,
1, 1, 1, 1, 1, 1, 1, 1, 1, 1, 1, 1, 1, 1, 1, 1, 1, 1, 1, 1, 1, 1, 1, 1, 1, 1, 1, 1, 1, 1, 1, 1, 1, 1, 1, 1, 1, 1, 1, 1, 1, 1,
1, 1, 1, 1, 1, 1, 1, 1, 1, 1, 1, 1, 1, 1, 1, 1, 1, 1, 1, 1, 1, 1, 1, 1, 1, 1, 1, 1, 1, 1, 1, 1, 1, 1, 1, 1, 1, 1, 1, 1, 1, 1,
1, 1, 1, 1, 1, 1, 1, 1, 1, 1, 1, 1, 1, 1, 1, 1, 1, 1, 1, 1, 1, 1, 1, 1, 1, 1, 1, 1, 1, 1, 1, 1, 1, 1, 1, 1, 1, 1, 1, 1, 1, 1,
1, 1, 1, 1, 1, 1, 1, 1, 1, 1, 1, 1, 1, 1, 1, 1, 1, 1, 1, 1, 1, 1, 1, 1, 1, 1, 1, 1, 1, 1, 1, 1, 1, 1, 1, 1, 1, 1, 1, 1, 1, 1,
1, 1, 1, 1, 1, 1, 1, 1, 1, 1, 1, 1, 1, 1, 1, 1, 1, 1, 1, 1, 1, 1, 1, 1, 1, 1, 1, 1, 1, 1, 1, 1, 1, 1, 1, 1, 1, 1, 1, 1, 1, 1,
1, 1, 1, 1, 1, 1, 1, 1, 1, 1, 1, 1, 1, 1, 1, 1, 1, 1, 1, 1, 1, 1, 1, 1, 1, 1, 1, 1, 1, 1, 1, 1, 1, 1, 1, 1, 1, 1, 1, 1, 1, 1,
1, 1, 1, 1, 1, 1, 1, 1, 1, 1, 1, 1, 1, 1, 1, 1, 1, 1, 1, 1, 1, 1, 1, 1, 1, 1, 1, 1, 1, 1, 1, 1, 1, 1, 1, 1, 1, 1, 1, 1, 1, 1,
1, 1, 1, 1, 1, 1, 1, 1, 1, 1, 1, 1, 1, 1, 1, 1, 1, 1, 1, 1, 1, 1, 1, 1, 1, 1, 1, 1, 1, 1, 1, 1, 1, 1, 1, 1, 1, 1, 1, 1

Corresponding number of selected TFs each gene is involved with:
(In the same order as above)
15, 15, 14, 14, 13, 13, 13, 13, 13, 13, 16, 16, 12, 12, 16, 12, 15, 15, 15, 15, 15, 15, 11, 11, 11, 11, 11, 11,
11, 11, 11, 11, 11, 14, 14, 14, 14, 14, 14, 14, 10, 10, 10, 10, 10, 10, 10, 10, 13, 13, 13, 13, 13, 13, 13, 13,
13, 13, 13, 13, 12, 9, 9, 12, 9, 12, 12, 12, 12, 12, 12, 9, 9, 17, 11, 11, 11, 11, 11, 11, 11, 11, 11, 11, 11, 11,
11, 11, 11, 11, 11, 8, 8, 8, 16, 8, 8, 8, 16, 8, 8, 8, 10, 10, 10, 10, 10, 10, 10, 10, 10, 10, 10, 10, 10, 15, 10,
10, 15, 10, 7, 14, 14, 7, 14, 14, 14, 7, 7, 7, 14, 7, 7, 14, 9, 9, 9, 9, 9, 9, 9, 9, 9, 9, 9, 13, 13, 13, 13, 13, 8, 6,
6, 8, 8, 8, 6, 6, 6, 12, 6, 6, 6, 6, 8, 6, 6, 6, 8, 6, 12, 8, 12, 8, 8, 8, 8, 8, 8, 8, 12, 12, 11, 11, 11, 11, 7, 7, 7, 7,
7, 7, 7, 7, 7, 7, 7, 7, 7, 7, 5, 5, 5, 5, 5, 5, 5, 5, 5, 5, 5, 5, 5, 10, 5, 5, 5, 5, 5, 10, 5, 5, 10, 6, 6, 6, 9, 6, 9, 6, 6,
6, 6, 9, 6, 6, 6, 6, 6, 6, 6, 6, 6, 6, 6, 6, 17, 8, 8, 4, 4, 4, 4, 8, 4, 4, 4, 4, 4, 8, 8, 8, 4, 4, 4, 5, 15, 15, 5, 5, 5, 5,
15, 5, 5, 15, 5, 5, 5, 15, 5, 15, 5, 15, 5, 5, 5, 5, 5, 5, 5, 5, 5, 5, 15, 5, 5, 5, 5, 5, 5, 5, 5, 14, 14, 14, 14, 7, 7,
14, 14, 14, 14, 7, 7, 14, 14, 14, 14, 13, 13, 13, 13, 13, 13, 13, 13, 13, 13, 13, 13, 13, 6, 3, 4, 4, 4, 4, 4, 4, 3,
4, 4, 4, 3, 12, 3, 4, 3, 4, 6, 4, 12, 3, 4, 3, 12, 4, 4, 3, 12, 3, 12, 3, 6, 3, 4, 4, 12, 12, 3, 4, 4, 4, 6, 4, 3, 4, 12,
3, 4, 3, 3, 6, 6, 4, 3, 4, 4, 3, 4, 4, 3, 12, 4, 3, 4, 4, 3, 3, 6, 3, 3, 4, 3, 11, 11, 11, 11, 11, 11, 11, 11, 11, 11, 5,
10, 5, 5, 10, 5, 10, 10, 5, 5, 10, 5, 10, 10, 5, 5, 5, 5, 5, 5, 9, 3, 3, 9, 3, 3, 9, 3, 3, 3, 3, 3, 9, 3, 9, 3, 3, 3, 3, 9,
3, 3, 9, 3, 9, 9, 3, 9, 3, 3, 3, 3, 3, 3, 3, 3, 3, 9, 9, 3, 3, 3, 3, 3, 3, 3, 3, 3, 3, 3, 3, 9, 3, 3, 3, 3, 3, 3, 9, 3, 3, 3,
3, 3, 9, 3, 8, 8, 8, 8, 2, 2, 2, 2, 2, 4, 2, 2, 4, 2, 2, 2, 2, 2, 2, 2, 4, 2, 2, 2, 2, 2, 4, 2, 2, 2, 8, 2, 2, 2, 2, 8, 2, 2,
2, 4, 2, 2, 2, 2, 2, 4, 2, 2, 4, 2, 4, 2, 2, 2, 2, 4, 2, 2, 2, 8, 8, 2, 2, 8, 2, 8, 8, 4, 2, 8, 2, 2, 2, 2, 8, 2, 2, 7, 7, 7,
7, 7, 7, 7, 7, 7, 7, 7, 7, 7, 7, 7, 3, 2, 2, 2, 2, 2, 2, 6, 2, 2, 6, 2, 2, 2, 2, 2, 3, 3, 2, 2, 2, 2, 2, 2, 2, 2, 6, 3, 2, 2,
2, 2, 6, 2, 2, 3, 3, 3, 2, 3, 6, 2, 2, 2, 6, 2, 2, 3, 3, 3, 2, 2, 2, 2, 2, 2, 6, 2, 2, 2, 2, 2, 2, 2, 2, 3, 2, 2, 6, 3, 2, 2,
2, 2, 2, 2, 2, 2, 2, 2, 2, 6, 3, 2, 2, 2, 2, 2, 2, 6, 3, 2, 2, 2, 2, 2, 2, 3, 2, 2, 2, 2, 3, 3, 2, 2, 3, 2, 2, 2, 2, 2, 2, 2,
2, 2, 2, 2, 2, 2, 2, 2, 2, 2, 2, 6, 2, 3, 2, 2, 2, 2, 2, 2, 6, 2, 2, 2, 2, 2, 2, 2, 2, 2, 2, 2, 6, 3, 2, 2, 3, 3, 2, 3, 2, 2,
2, 3, 2, 5, 5, 5, 5, 5, 5, 5, 5, 5, 5, 5, 4, 4, 1, 1, 1, 1, 1, 1, 1, 1, 2, 1, 1, 2, 2, 1, 1, 1, 1, 1, 2, 4, 1, 1, 1, 1, 1, 1,
1, 2, 2, 4, 1, 1, 1, 1, 1, 4, 2, 2, 1, 1, 1, 1, 1, 1, 1, 1, 2, 2, 1, 1, 1, 1, 2, 2, 1, 1, 2, 1, 1, 4, 1, 1, 4, 1, 1, 4, 2, 1,
1, 1, 2, 1, 4, 1, 1, 1, 1, 1, 2, 4, 1, 1, 1, 1, 1, 1, 1, 2, 1, 1, 1, 1, 2, 1, 1, 2, 2, 4, 2, 1, 1, 1, 1, 1, 1, 1, 1, 2, 2, 2,
2, 2, 1, 2, 1, 2, 1, 1, 1, 1, 2, 1, 2, 2, 2, 1, 1, 1, 1, 1, 1, 1, 1, 1, 1, 1, 1, 1, 1, 1, 1, 1, 1, 1, 1, 1, 4, 2, 2, 2, 1, 1,
1, 1, 1, 1, 1, 2, 1, 1, 2, 1, 1, 1, 1, 1, 1, 2, 2, 1, 1, 4, 1, 1, 1, 1, 1, 1, 2, 1, 2, 1, 1, 1, 1, 1, 1, 1, 1, 1, 4, 2, 1, 1,
1, 4, 1, 1, 1, 1, 1, 1, 2, 1, 2, 1, 1, 1, 2, 2, 4, 1, 1, 2, 1, 1, 1, 1, 1, 1, 1, 2, 1, 2, 1, 1, 1, 4, 2, 1, 1, 2, 1, 1, 2, 1,
1, 1, 1, 1, 2, 1, 2, 2, 1, 1, 1, 1, 1, 1, 1, 1, 2, 1, 1, 1, 3, 1, 1, 1, 3, 1, 1, 1, 3, 1, 1, 3, 1, 1, 3, 1, 1, 3, 1, 1, 1, 1,
3, 1, 1, 1, 1, 1, 1, 1, 3, 1, 1, 1, 1, 1, 1, 1, 1, 1, 1, 1, 1, 1, 1, 1, 1, 1, 1, 1, 1, 3, 1, 1, 1, 1, 3, 1, 1, 3, 1, 1, 1, 1,
1, 1, 1, 1, 1, 3, 1, 1, 1, 1, 1, 1, 1, 1, 1, 3, 1, 3, 3, 3, 1, 1, 1, 1, 3, 1, 1, 1, 1, 1, 1, 1, 3, 1, 1, 1, 1, 1, 1, 1, 3, 1,
3, 3, 1, 1, 1, 1, 3, 1, 1, 1, 1, 1, 1, 1, 1, 1, 1, 1, 1, 1, 1, 1, 1, 1, 3, 1, 3, 1, 1, 1, 1, 1, 3, 1, 1, 1, 1, 1, 1, 1, 1, 1,
1, 1, 1, 1, 1, 1, 3, 1, 1, 1, 1, 1, 1, 3, 3, 1, 1, 1, 1, 1, 1, 1, 1, 1, 1, 1, 1, 1, 1, 1, 1, 1, 1, 1, 1, 1, 1, 1, 1, 1, 1, 3,
1, 3, 1, 1, 1, 1, 1, 3, 1, 1, 1, 1, 1, 1, 1, 3, 1, 1, 1, 1, 1, 1, 1, 1, 1, 1, 1, 1, 1, 1, 1, 1, 1, 3, 1, 1, 1, 3, 1, 1, 3, 1,
1, 1, 1, 1, 1, 1, 1, 1, 1, 1, 1, 3, 1, 1, 1, 1, 1, 1, 1, 1, 3, 1, 1, 1, 1, 1, 1, 1, 1, 1, 1, 1, 3, 1, 1, 1, 1, 3, 1, 1, 1, 1,
1, 1, 1, 1, 1, 1, 1, 3, 1, 1, 3, 3, 3, 1, 1, 1, 1, 3, 1, 1, 1, 3, 1, 1, 1, 1, 1, 1, 1, 1, 3, 1, 1, 1, 1, 1, 1, 1, 1, 1, 1, 1,
1, 1, 1, 1, 3, 1, 1, 1, 1, 1, 1, 1, 1, 1, 1, 1, 1, 3, 1, 1, 1, 1, 1, 1, 1, 1, 1, 1, 1, 3, 1, 1, 3, 3, 1, 1, 1, 1, 1, 1, 1, 1,

1, 1, 1, 1, 1, 1, 3, 1, 1, 1, 3, 1, 1, 1, 1, 1, 1, 1, 1, 1, 1, 1, 3, 1, 1, 1, 1, 1, 1, 1, 1, 1, 1, 1, 1, 1, 1, 2, 1, 1, 2, 2, 1, 1, 2, 2, 2, 2, 2, 1, 1, 1, 2, 1, 2, 2, 2, 1, 1, 1, 1, 2, 1, 1, 1, 1, 2, 1, 2, 2, 2, 2, 1, 1, 1, 1, 1, 1, 1, 1, 2, 1, 2, 1, 2, 2, 1, 1, 2, 2, 2, 1, 2, 1, 1, 2, 2, 2, 2, 2, 1, 1, 1, 2, 1, 1, 2, 1, 2, 2, 2, 2, 2, 1, 1, 2, 2, 2, 1, 2, 1, 1, 1, 2, 1, 1, 2, 1, 1, 2, 1, 2, 2, 2, 2, 2, 2, 1, 2, 2, 2, 2, 1, 2, 1, 2, 2, 1, 2, 2, 2, 1, 2, 2, 2, 1, 1, 1, 1, 1, 1, 1, 1, 1, 2, 2, 1, 1, 2, 1, 1, 1, 1, 1, 2, 2, 2, 1, 1, 1, 1, 2, 2, 2, 1, 1, 1, 2, 1, 2, 2, 1, 2, 1, 1, 1, 2, 2, 2, 2, 2, 2, 2, 1, 2, 2, 1, 2, 1, 1, 2, 2, 2, 2, 1, 2, 2, 1, 1, 2, 1, 2, 2, 1, 1, 1, 2, 1, 1, 2, 1, 2, 1, 2, 1, 2, 1, 2, 1, 2, 2, 1, 2, 1, 2, 2, 2, 1, 1, 1, 1, 2, 2, 2, 1, 1, 1, 1, 2, 1, 2, 1

Phenopedia diseases ranked according to mean of effect sizes in cluster:
Disease Susceptibility | Genetic Predisposition to Disease | Disease | Werner Syndrome

Corresponding mean of effect sizes of each Phenopedia disease:
(In the same order as above)
4.10, 4.10, 3.74, 3.31

Corresponding total number of genes of each Phenopedia disease:
(In the same order as above)
4.9K, 4.9K, 4.6K, 3.9K

Corresponding number of genes of each Phenopedia disease with at least one TF hit (of selected TFs):
(In the same order as above)
1305, 1293, 1215, 914

Corresponding total number of TF hits (of selected TFs) for each Phenopedia disease (in all genes):
(In the same order as above)
4265, 4231, 3883, 2861

Corresponding number of selected TFs each Phenopedia disease is involved with:
(In the same order as above)
19, 19, 19, 19

TFs ranked according to mean of effect sizes in cluster:
VATF_01, VATF1_Q6, V$CREB_Q4_01, V$NRF1_Q6, V$CREB_Q2_01, V$ATF_B, V$ELK1_02, V$CREBATF_Q6, V$CREB_Q4, V$CREBP1_Q2, V$GABP_B, V$CREB_01, V$TAXCREB_01, V$CREB_Q2, V$ATF3_Q6, V$CREBP1CJUN_01, V$CREB_02, V$IPF1_Q4_01, V$ATF4_Q2

Corresponding mean of effect sizes of each TF:
(In the same order as above)
5.64, 4.82, 4.59, 4.57, 4.25, 4.24, 4.24, 4.08, 4.00, 3.84, 3.71, 3.70, 3.40, 3.15, 3.11, 3.09, 2.98, 2.60, 2.43

Corresponding total number of TF hits for each TF (genome-wide):
(In the same order as above)
463, 426, 421, 418, 438, 429, 372, 434, 418, 408, 391, 425, 448, 435, 449, 440, 401, 520, 390

Corresponding total number of TF hits for each TF (in all genes in selected Phenopedia diseases):
(In the same order as above)
842, 841, 781, 760, 834, 831, 659, 823, 762, 772, 658, 806, 857, 837, 843, 855, 740, 1024, 715

Corresponding number of genes (of selected Phenopedia diseases) each TF is involved with:
(In the same order as above)
364, 344, 335, 327, 349, 346, 277, 345, 320, 323, 281, 337, 355, 343, 346, 356, 308, 412, 294

Corresponding number of selected Phenopedia diseases each TF is involved with:
(In the same order as above)
4, 4, 4, 4, 4, 4, 4, 4, 4, 4, 4, 4, 4, 4, 4, 4, 4, 4, 4

Disease groups of selected Phenopedia diseases ranked according to sum of effect sizes
Disease Susceptibility | Genetic Predisposition to Disease | Disease Attributes | Disease | Werner Syndrome | Skin Diseases | Signs and Symptoms | Metabolism, Inborn Errors | Metabolic Diseases | Genetic Diseases, Inborn | Congenital, Hereditary, and Neonatal Diseases and Abnormalities | Aging, Premature

Corresponding sum of effect sizes for each disease group of selected Phenopedia diseases
(In the same order as above)
77.92, 77.87, 77.87, 71.11, 62.97, 62.97, 62.97, 62.97, 62.97, 62.97, 62.97, 62.97

Cluster for columns 27 to 35, rows 309 to 320

Phenopedia diseases

Cardiovascular Diseases | Vascular Diseases | Hemorrhage | Subarachnoid Hemorrhage | Cerebral Hemorrhage | Intracranial Hemorrhages | Cerebrovascular Disorders | Cerebrovascular Accident | Myocardial Infarction

TFs

V$AP2_Q3 | V$TAXCREB_02 | V$PAX5_02 | V$PAX5_01 | V$MTF1_Q4 | V$LMO2COM_01 | V$E12_Q6 | V$E47_01 | V$AREB6_03 | V$CACCCBINDINGFACTOR_Q6 | V$CP2_02 | V$AP4_01

Information

All related TFs:
(List of all TFs that are related to any of the PWMs)
AP-2, AP-2alpha, AP-2alphaA, AP-2alphaB, AP-2beta, AP-2gamma, AP-4, CACCC-binding, CP2, CP2a, CREB, E12, E47, Lmo2, MTF-1, Pax-5, Tax, ZEB, deltaCREB

Ranked gene list:
(All genes of the selected Phenopedia diseases with hits of any of the selected TFs, ranked according to the total number of TF hits)
SBF1, TBL3, PIGQ, LLGL2, CTBP1, GYLTL1B, SIX5, SEPT9, NADSYN1, MEF2D, FUT7, SEMA3F, PACS2, MYH9, IL21R, CDH15, ARVCF, TNFRSF4, LTC4S, LTBP4, ADORA2A, UNC84A, TP73, TNFRSF6B, SS18L1, NCOR2, ITM2C, FOXF1, CDYL2, SLC39A7, LCAT, ITGB2, TNFRSF18, TCF7, PTPN6, CYBA, CTSD, CDKN1C, C12orf57, VEGFB, AKT1, WDR37, UHRF1, TAF4, SPTBN4, RGS12, PSMA4, POLR1D, LRPAP1, ITPK1, IRF5, GATA2, EFNA3, CELSR1, CAMTA1, ADAMTSL5, UCP3, DDAH2, TRAF2, TLR9, TBXA2R, TIMP2, RGS19, NOTCH1, MMP11, GNAI2, TRADD, TGFB1, SLC19A1, SH2B3, PROC, PLEKHG2, P2RY11, OAZ1, NFKB2, LTB4R2, LTB4R, KCNQ1, IL4R, IGF1R, CLPTM1L, SREBF1, SLC7A1, RXRB, MMEL1, KCNN4, GRM8, GCLM, EMILIN1, DMPK, CX3CR1, AQP10, APP, APEX1, WNT3, WNK2, UGGT2, TRAPPC9, TOLLIP, TH, TFF1, TCF25, SORCS2, SLIT2, SDK1, PTGDS, PNPLA2, PCSK2, PANX2, OSGIN1, OGDHL, NUP210, NFKBIE, MTHFR, MCC, LZTR1, KIAA1161, JPH3, IVD, ITGB3, IGF2, HDAC4, GPR135,

GNAS, ECE1, DIDO1, DBH, CAPN10, CAMK2G, C10orf116, ATXN1, AP3D1, ANKMY1, TNFSF13, TNFRSF14, THBS1, PSTPIP1, PROZ, PRDX5, PPARA, POLG, NR3C2, LIMK1, LIF, HPCAL1, HBA1, GAS6, CPT1B, CCND1, ARHGEF2, APOB, APOA1, ADM, TUBB, TBX1, SOCS1, PDIA2, MGAT1, KCNH2, VKORC1, VEGFA, TNFRSF1B, TNFRSF1A, TNFRSF13C, SORL1, SLC20A1, SIT1, RXRA, RHOC, RARA, PTPRA, PLTP, PDLIM1, PDCD1, PAX6, MYB, MCF2L, JAG1, IRS2, IRS1, INSIG1, INS, IL4, IGF2BP2, HSPG2, HSPA1L, HPN, GPX4, FYN, FADS3, CYP27B1, CPT1A, CNNM2, CFD, CD81, BIK, BCL7A, BCL2L11, ATP1B1, ARHGEF1, ARHGDIG, WNT11, VDR, VAMP8, USF2, TNNC1, TAF3, SGIP1, SELPLG, P2RY2, NFKBIA, MIF, LTB, LRP1, ITGA3, HLA-B, HBB, GCLC, FGFR3, DM1, DHCR7, DGAT1, CYP4F2, APOC3, APOA4, ALOX5AP, XYLT2, XRCC3, VEGF, UTS2R, UCP2, TSC22D1, TRPM7, TNFRSF12A, TIAM1, THRA, TGFBR2, TGFBR1, SOX17, SORBS1, SLC23A1, RHOB, PYY, PTGER1, PLEKHA1, PIM1, OSGEP, NTAN1, NR1H2, NKX2-1, NINJ2, NDUFAB1, MTP18, LPIN1, LMNA, LMAN1, KCNB1, KALRN, IRF1, INPPL1, IMPA2, IGF2AS, ICAM4, HLA-C, HIVEP1, GPR132, GALNT2, G6PD, FN3K, CYP4V2, CYB561, CTCF, CLCN6, CEBPA, CDKN2C, BCL6, ATP1A1, ARHGEF16, ARHGEF10, ARHGAP10, AMPD2, AGER, ADA, ZNF202, UGCGL2, TNFAIP3, THPO, TAPBPL, STX1A, SLCO3A1, SLC9A3R1, SIGMAR1, SHPK, SELS, S100A6, RRP1, RNF207, RFC1, RARG, PVRL2, PTMS, PPIA, PCNT, NPHS1, NOD2, NFATC1, NAT1, MT2A, LOXL1, LEF1, ITIH3, IRX4, IL6ST, IL6R, GLI1, GAPDH, F2R, EGR3, EGFR, DAB2, CYP46A1, CXCL16, CFP, CDKN1B, CALHM1, CACNA1C, C6orf27, C6orf129, AXIN1, ATP4B, APOC2, AKAP10, ADRA2B, ABCD1, YEATS4, XYLT1, WWTR1, VGF, VAV3, VAV2, ULK3, TSC1, TNFSF9, TMEM43, TDRD5, TAP1, STK11, SOS1, SMAD4, SLC9A2, SLC7A5, SLC6A9, SLC6A19, SLC4A5, SLC3A2, SHMT1, SHB, SFT2D2, SERPINF1, SDHD, SCARB1, RGS9BP, RGS9, RET, RELB, RELA, PTGIR, PTGES2, PTCRA, PTBP1, PSMB9, PSMB8, PRMT8, PRKCB1, PRKCB, PPP1R13L, PPARD, POU2F1, POMC, POGK, PNMT, PLEKHA6, NTRK3, NR4A2, NR2F1, NR1H3, NEUROG3, NEDD4L, MYO7A, MYBPH, MVK, MT1A, MMAB, MLXIPL, MGST3, MADD, M6PR, LY86, LTBP3, KRIT1, KLF12, KCTD15, ITSN2, IQWD1, IPO7, IL11, IKBKG, HSD11B2, HN1L, HHEX, HBS1L, GUCA2B, GSTM3, GSK3A, GPAM, GCH1, GBA, FOXO1, FMOD, FBN1, ERCC6, ERBB3, EDN2, DSP, DAB2IP, CXCR4, CXADR, CTNS, CSNK2A2, CSNK1D, CREBBP, CREB1, CHRNA4, CHN2, CHD7, CEL, CD79A, CD5, CD4, CD3EAP, CD247, CAST, CASP9, CARTPT, CARD15, CAPN5, CANT1, CACNA1D, BRP44, BMP1, BHLHA15, BCR, BCL7C, BCL2L1, BAK1, ATG9B, ARHGEF3, ARHGEF12, ARHGEF11, ARHGEF10L, APOC4, ANGPTL4, ANG, ALDH1A2, ALAD, AHR, ADRBK1, ZNF536, ZEB2, ZBTB16, VAMP2, TPM1, TOMM40, TBX3, STAT6, SLC22A11, SFRP2, SCN1B, RUNX2, PROK2, PRDM2, PIK3CA, PBX4, NRF1, NPPC, NODAL, NKX2-5, NDRG4, MYL3, MLYCD, MLX, MKL1, MEIS2, MAP2K2, LST1, LITAF, LDB3, LCN1, LASS4, KCNJ5, JUP, IL1RAP, IGFBP6, HRC, HOXC4, GSTO2, GRK5, GRID1, GRB2, GIPR, GAA, FGFR1, FBLN1, ERBB4, ERBB2, EP300, EGR2, DDC, CRHR2, COMP, COL4A3, CCNI, CBR3, BRAF, BMP3, BIRC2, BCL3, ATG16L1, APBB2, APBB1

Corresponding total number of TF hits:
(For each gene listed above, the total number of TF hits for any of the selected TFs, multiplied by the number of selected Phenopedia diseases containing that gene)
99, 81, 81, 81, 72, 54, 45, 45, 45, 45, 45, 36, 36, 36, 36, 36, 36, 33, 30, 30, 30, 27, 27, 27, 27, 27, 27, 27, 27, 25, 25, 25, 24, 24, 24, 21, 21, 21, 21, 20, 20, 18, 18, 18, 18, 18, 18, 18, 18, 18, 18, 18, 18, 18, 18, 18, 16, 16, 15, 15, 15, 14, 14, 14, 14, 14, 12, 12, 12, 12, 12, 12, 12, 12, 12, 12, 12, 12, 12, 12, 10, 10, 10, 10, 10, 10, 10, 10, 10, 10, 10, 10, 10, 9, 9, 9, 9, 9, 9, 9, 9, 9, 9, 9, 9, 9, 9, 9, 9, 9, 9, 9, 9, 9, 9, 9, 9, 9, 9, 9, 9, 9, 9, 9, 9, 9, 9, 9, 9, 9, 9, 9, 9, 8, 8, 8, 8, 8, 8, 8, 8, 8, 8, 8, 8, 8, 8, 8, 8, 8, 8, 8, 8, 7, 7, 7, 7, 7, 7, 6, 6, 6, 6, 6, 6, 6, 6, 6, 6, 6, 6, 6, 6, 6, 6, 6, 6, 6, 6, 6, 6, 6, 6, 6, 6, 6, 6, 6, 6, 6, 6, 6, 6, 6, 6, 6, 6, 6, 6, 6, 6, 5, 5, 5, 5, 5, 5, 5, 5, 5, 5, 5, 5, 5, 5, 5, 5, 5, 5, 5, 5, 5, 5, 5, 5, 5, 4, 4, 4, 4, 4, 4, 4, 4, 4, 4, 4, 4, 4, 4, 4, 4, 4, 4, 4, 4, 4, 4, 4, 4, 4, 4, 4, 4, 4, 4, 4, 4, 4, 4, 4, 4, 4, 4, 4, 4, 4, 4, 4, 4, 4, 4, 4, 4, 4, 4, 4, 4, 4, 4, 4, 4, 4, 4, 3, 3, 3, 3, 3, 3, 3, 3, 3, 3, 3, 3, 3, 3, 3, 3, 3, 3, 3, 3, 3, 3, 3, 3, 3, 3, 3, 3, 3, 3, 3, 3, 3, 3, 3, 3, 3, 3, 3, 3, 3, 3, 3, 3, 3, 3, 3, 3, 3, 3, 3, 2, 1, 1, 1, 1, 1, 1, 1, 1, 1, 1, 1, 1, 1, 1, 1, 1, 1, 1, 1, 1, 1, 1, 1, 1, 1, 1, 1, 1, 1, 1, 1, 1, 1, 1, 1, 1, 1, 1, 1, 1, 1, 1, 1, 1, 1, 1, 1, 1, 1, 1, 1, 1, 1, 1, 1, 1, 1, 1, 1, 1, 1, 1, 1

Corresponding number of selected Phenopedia diseases each gene is involved with:

(In the same order as above)
9, 9, 9, 9, 9, 9, 9, 9, 9, 9, 9, 9, 9, 9, 9, 9, 9, 3, 5, 6, 3, 9, 9, 3, 9, 9, 9, 9, 9, 5, 5, 5, 2, 4, 3, 7, 3, 3, 3, 2, 2, 9, 9, 9, 9, 9, 9, 9, 9, 9, 2, 3, 9, 9, 9, 8, 8, 3, 3, 5, 7, 2, 2, 2, 2, 2, 6, 4, 3, 4, 2, 3, 3, 3, 3, 4, 3, 6, 4, 3, 2, 2, 2, 2, 5, 5, 5, 2, 2, 5, 5, 5, 5, 9, 9, 9, 9, 9, 3, 9, 9, 9, 9, 9, 3, 1, 9, 9, 9, 9, 9, 3, 9, 9, 9, 3, 9, 9, 9, 3, 3, 9, 3, 3, 9, 9, 3, 3, 3, 3, 9, 1, 2, 2, 8, 2, 4, 2, 4, 4, 2, 8, 4, 2, 4, 8, 2, 2, 2, 8, 8, 4, 7, 1, 1, 1, 1, 1, 6, 6, 6, 6, 2, 3, 2, 3, 3, 2, 1, 2, 3, 3, 3, 2, 3, 2, 3, 2, 3, 3, 2, 6, 2, 3, 6, 6, 2, 2, 2, 2, 2, 2, 2, 2, 2, 2, 2, 2, 2, 1, 1, 5, 5, 1, 1, 5, 5, 5, 5, 5, 5, 5, 5, 5, 5, 5, 5, 1, 1, 1, 1, 5, 5, 5, 5, 2, 4, 4, 2, 4, 2, 4, 2, 2, 1, 4, 4, 4, 4, 1, 2, 2, 1, 2, 2, 2, 2, 2, 4, 4, 2, 2, 2, 4, 4, 2, 4, 2, 2, 4, 2, 4, 4, 2, 2, 2, 4, 2, 2, 2, 2, 4, 2, 2, 2, 2, 2, 4, 2, 2, 4, 2, 3, 3, 3, 3, 3, 1, 3, 1, 3, 3, 3, 1, 3, 1, 3, 1, 3, 3, 3, 3, 3, 3, 1, 3, 3, 3, 1, 3, 1, 3, 3, 1, 1, 3, 1, 3, 3, 1, 3, 3, 3, 3, 3, 3, 3, 1, 1, 3, 3, 3, 3, 2, 2, 2, 1, 2, 2, 2, 2, 2, 1, 2, 2, 1, 2, 2, 2, 2, 2, 2, 2, 2, 2, 1, 2, 2, 2, 2, 2, 2, 2, 2, 2, 2, 1, 1, 1, 2, 2, 2, 2, 2, 2, 2, 2, 2, 2, 2, 2, 2, 1, 2, 2, 2, 2, 2, 2, 2, 1, 2, 2, 2, 2, 2, 2, 2, 2, 2, 1, 2, 2, 2, 1, 2, 2, 1, 2, 2, 2, 2, 2, 1, 2, 2, 2, 2, 2, 2, 2, 2, 1, 2, 2, 2, 2, 1, 2, 1, 1, 1, 2, 1, 2, 2, 2, 2, 2, 2, 2, 2, 2, 2, 2, 2, 2, 2, 1, 2, 2, 2, 2, 2, 2, 2, 2, 2, 2, 2, 2, 2, 1, 2, 2, 1, 1, 1, 1, 1, 1, 1, 1, 1, 1, 1, 1, 1, 1, 1, 1, 1, 1, 1, 1, 1, 1, 1, 1, 1, 1, 1, 1, 1, 1, 1, 1, 1, 1, 1, 1, 1, 1, 1, 1, 1, 1, 1, 1, 1, 1, 1, 1, 1, 1, 1, 1, 1, 1, 1, 1, 1, 1, 1, 1, 1, 1, 1, 1

Corresponding number of selected TFs each gene is involved with:
(In the same order as above)
11, 9, 9, 9, 8, 6, 5, 5, 5, 5, 5, 4, 4, 4, 4, 4, 4, 11, 6, 5, 10, 3, 3, 9, 3, 3, 3, 3, 3, 5, 5, 5, 12, 6, 8, 3, 7, 7, 7, 10, 10, 2, 2, 2, 2, 2, 2, 2, 2, 2, 2, 9, 6, 2, 2, 2, 2, 2, 5, 5, 3, 2, 7, 7, 7, 7, 6, 2, 3, 4, 3, 6, 4, 4, 4, 4, 3, 4, 2, 3, 4, 5, 5, 5, 5, 2, 2, 2, 5, 5, 2, 2, 2, 2, 1, 1, 1, 1, 1, 3, 1, 1, 1, 1, 1, 3, 9, 1, 1, 1, 1, 1, 3, 1, 1, 1, 3, 1, 1, 1, 3, 3, 1, 3, 3, 1, 1, 3, 3, 3, 3, 1, 9, 4, 4, 1, 4, 2, 4, 2, 2, 4, 1, 2, 4, 2, 1, 4, 4, 4, 1, 1, 2, 1, 7, 7, 7, 7, 7, 1, 1, 1, 1, 3, 2, 3, 2, 2, 3, 6, 3, 2, 2, 2, 3, 2, 3, 2, 3, 2, 2, 3, 1, 3, 2, 1, 1, 3, 3, 3, 3, 3, 3, 3, 3, 3, 3, 3, 3, 3, 6, 5, 1, 1, 5, 5, 1, 1, 1, 1, 1, 1, 1, 1, 1, 1, 1, 5, 5, 5, 5, 1, 1, 1, 1, 2, 1, 1, 2, 1, 2, 1, 2, 2, 4, 1, 1, 1, 1, 4, 2, 2, 4, 2, 2, 2, 2, 2, 1, 1, 2, 2, 2, 1, 1, 2, 1, 2, 2, 1, 2, 1, 1, 2, 2, 2, 1, 2, 2, 2, 2, 1, 2, 2, 2, 2, 2, 1, 2, 2, 1, 2, 1, 1, 1, 1, 1, 3, 1, 3, 1, 1, 1, 3, 1, 3, 1, 3, 1, 1, 1, 1, 1, 1, 3, 1, 1, 1, 3, 1, 3, 1, 1, 3, 3, 1, 3, 1, 1, 3, 1, 1, 1, 1, 1, 1, 1, 3, 3, 1, 1, 1, 1, 1, 1, 1, 2, 1, 1, 1, 1, 1, 2, 1, 1, 2, 1, 1, 1, 1, 1, 1, 1, 1, 1, 2, 1, 1, 1, 1, 1, 1, 1, 1, 1, 1, 1, 2, 2, 2, 1, 1, 1, 1, 1, 1, 1, 1, 1, 1, 1, 1, 1, 2, 1, 1, 1, 1, 1, 1, 1, 2, 1, 1, 1, 1, 1, 1, 1, 1, 1, 2, 1, 1, 1, 2, 1, 1, 2, 1, 1, 1, 1, 1, 2, 1, 1, 1, 1, 1, 1, 1, 1, 1, 2, 1, 1, 1, 1, 2, 1, 2, 2, 2, 1, 2, 1, 1, 1, 1, 1, 1, 1, 1, 1, 1, 1, 1, 1, 1, 2, 1, 1, 1, 1, 1, 1, 1, 1, 1, 1, 1, 1, 1, 2, 1, 1, 2, 1, 1, 1, 1, 1, 1, 1, 1, 1, 1, 1, 1, 1, 1, 1, 1, 1, 1, 1, 1, 1, 1, 1, 1, 1, 1, 1, 1, 1, 1, 1, 1, 1, 1, 1, 1, 1, 1, 1, 1, 1, 1, 1, 1, 1, 1, 1, 1, 1, 1, 1, 1, 1, 1, 1, 1, 1, 1, 1, 1, 1, 1, 1, 1, 1, 1

Phenopedia diseases ranked according to mean of effect sizes in cluster:
Cerebral Hemorrhage | Subarachnoid Hemorrhage | Intracranial Hemorrhages | Cardiovascular Diseases | Hemorrhage | Myocardial Infarction | Vascular Diseases | Cerebrovascular Accident | Cerebrovascular Disorders

Corresponding mean of effect sizes of each Phenopedia disease:
(In the same order as above)
2.68, 2.45, 2.38, 1.83, 1.59, 1.58, 1.54, 1.54, 1.35

Corresponding total number of genes of each Phenopedia disease:
(In the same order as above)
192, 192, 213, 1.9K, 384, 582, 1.6K, 499, 675

Corresponding number of genes of each Phenopedia disease with at least one TF hit (of selected TFs):
(In the same order as above)
71, 69, 74, 519, 109, 146, 408, 127, 179

Corresponding total number of TF hits (of selected TFs) for each Phenopedia disease (in all genes):
(In the same order as above)
185, 187, 192, 1146, 251, 333, 896, 287, 409

Corresponding number of selected TFs each Phenopedia disease is involved with:
(In the same order as above)
12, 12, 12, 12, 12, 12, 12, 12, 12

TFs ranked according to mean of effect sizes in cluster:
V$E12_Q6, V$LMO2COM_01, V$E47_01, V$PAX5_01, V$AP2_Q3, V$AP4_01, V$PAX5_02, V$CACCCBINDINGFACTOR_Q6, V$AREB6_03, V$CP2_02, V$MTF1_Q4, V$TAXCREB_02

Corresponding mean of effect sizes of each TF:
(In the same order as above)
2.87, 2.21, 2.19, 2.13, 2.11, 2.04, 2.00, 1.91, 1.64, 1.60, 1.22, 0.66

Corresponding total number of TF hits for each TF (genome-wide):
(In the same order as above)
418, 391, 387, 454, 456, 419, 456, 440, 415, 445, 449, 434

Corresponding total number of TF hits for each TF (in all genes in selected Phenopedia diseases):
(In the same order as above)
339, 338, 314, 294, 339, 332, 361, 338, 335, 301, 292, 303

Corresponding number of genes (of selected Phenopedia diseases) each TF is involved with:
(In the same order as above)
90, 99, 93, 79, 94, 105, 103, 107, 105, 99, 94, 93

Corresponding number of selected Phenopedia diseases each TF is involved with:
(In the same order as above)
9, 9, 9, 9, 9, 9, 9, 9, 9, 9, 9, 9

Disease groups of selected Phenopedia diseases ranked according to sum of effect sizes
Nervous System Diseases | Central Nervous System Diseases | Intracranial Hemorrhages | Cardiovascular Diseases | Brain Diseases | Vascular Diseases | Cerebral Hemorrhage | Subarachnoid Hemorrhage | Hemorrhage | Thoracic Diseases | Respiratory Tract Diseases | Myocardial Infarction | Mediastinal Diseases | Cardiomyopathies | Cerebrovascular Accident | Cerebrovascular Disorders

Corresponding sum of effect sizes for each disease group of selected Phenopedia diseases
(In the same order as above)
106.19, 106.19, 89.99, 75.53, 48.31, 34.65, 32.11, 29.38, 19.14, 18.94, 18.94, 18.94, 18.94, 18.94, 18.43, 16.20

Cluster for columns 52 to 58, rows 321 to 335

Phenopedia diseases

Meningeal Neoplasms | Brain Neoplasms | Central Nervous System Neoplasms | Nervous System Neoplasms | Xeroderma Pigmentosum | Neoplasms, Radiation-Induced | Radiation Injuries

TFs

V$MYCMAX_01 | V$ARNT_01 | V$MYC_Q2 | V$CLOCKBMAL_Q6 | V$USF_C | V$MYCMAX_02 | V$USF_Q6 | V$USF_02 | V$USF_Q6_01 | V$USF_01 | V$MYCMAX_03 | V$MAX_01 | V$YY1_02 | V$MAZR_01 | V$SREBP1_01

Information

All related TFs:
(List of all TFs that are related to any of the PWMs)

Arnt, Clock:BMAL, Clock:BMAL1, Clock:BMAL2, MAZR, Max, Max1, SREBP-1, SREBP-1a, SREBP-1b, SREBP-1c, USF, USF-1, USF1, USF1:USF2, USF1a, USF1b, USF2, USF2a, USF2b, YY1, c-Myc

Ranked gene list:
(All genes of the selected Phenopedia diseases with hits of any of the selected TFs, ranked according to the total number of TF hits)
HRAS, APEX1, CCND1, MTHFR, CDK4, TGFB1, RTEL1, BRCA2, TERT, GTF2H1, GNAS, ERCC6, NHEJ1, IGF2R, CHAF1A, MPG, IGF1R, GPX1, XRCC3, UNG, RECQL4, RAD54L, EME1, ALKBH2, MGMT, REV1, RAD51L1, POLD1, IL13, MC1R, CBS, TDP1, POLG, NUDT1, IGF2, GNAI2, FANCD2, MTR, ERCC1, COMT, CDKN1A, TCN2, SOD2, PCNA, MDM2, LIG1, GSTM3, TDG, POLI, PMS2, PHLDB1, NR3C1, NOS2, MSH5, HEL308, FANCA, CYP3A4, XPA, TCEAL1, RAD21, NUMBL, NF1, DDB2

Corresponding total number of TF hits:
(For each gene listed above, the total number of TF hits for any of the selected TFs, multiplied by the number of selected Phenopedia diseases containing that gene)
72, 66, 63, 50, 36, 30, 30, 30, 28, 24, 24, 21, 18, 18, 16, 15, 15, 15, 14, 12, 12, 12, 12, 12, 10, 9, 9, 9, 9, 8, 8, 6, 6, 6, 6, 6, 6, 5, 5, 5, 5, 4, 4, 4, 4, 4, 4, 3, 3, 3, 3, 3, 3, 3, 3, 3, 2, 1, 1, 1, 1, 1, 1

Corresponding number of selected Phenopedia diseases each gene is involved with:
(In the same order as above)
6, 6, 7, 5, 3, 5, 3, 5, 4, 3, 3, 3, 3, 3, 4, 3, 3, 3, 7, 3, 3, 3, 3, 3, 5, 3, 3, 3, 3, 1, 4, 3, 3, 3, 3, 3, 3, 5, 5, 1, 5, 4, 4, 4, 4, 4, 4, 3, 3, 3, 3, 3, 3, 3, 3, 3, 2, 1, 1, 1, 1, 1, 1

Corresponding number of selected TFs each gene is involved with:
(In the same order as above)
12, 11, 9, 10, 12, 6, 10, 6, 7, 8, 8, 7, 6, 6, 4, 5, 5, 5, 2, 4, 4, 4, 4, 4, 2, 3, 3, 3, 3, 8, 2, 2, 2, 2, 2, 2, 1, 1, 5, 1, 1, 1, 1, 1, 1, 1, 1, 1, 1, 1, 1, 1, 1, 1, 1, 1, 1, 1, 1, 1, 1, 1, 1

Phenopedia diseases ranked according to mean of effect sizes in cluster:
Meningeal Neoplasms | Central Nervous System Neoplasms | Nervous System Neoplasms | Radiation Injuries | Xeroderma Pigmentosum | Brain Neoplasms | Neoplasms, Radiation-Induced

Corresponding mean of effect sizes of each Phenopedia disease:
(In the same order as above)
1.94, 1.80, 1.63, 1.50, 1.47, 0.99, 0.88

Corresponding total number of genes of each Phenopedia disease:
(In the same order as above)
105, 174, 182, 45, 45, 132, 31

Corresponding number of genes of each Phenopedia disease with at least one TF hit (of selected TFs):
(In the same order as above)
39, 54, 56, 13, 10, 31, 6

Corresponding total number of TF hits (of selected TFs) for each Phenopedia disease (in all genes):
(In the same order as above)
132, 205, 207, 64, 50, 128, 36

Corresponding number of selected TFs each Phenopedia disease is involved with:
(In the same order as above)
15, 15, 15, 15, 15, 15, 15

TFs ranked according to mean of effect sizes in cluster:

V$SREBP1_01, V$MYCMAX_03, VUSF_01, VMYC_Q2, VUSF_Q6, VMAX_01, V$CLOCKBMAL_Q6, V$USF_C, V$MAZR_01, V$YY1_02, V$MYCMAX_01, V$USF_02, VUSF_Q6_01, VMYCMAX_02, V$ARNT_01

Corresponding mean of effect sizes of each TF:
(In the same order as above)
2.39, 2.31, 2.27, 2.06, 1.68, 1.48, 1.47, 1.34, 1.21, 1.15, 1.10, 1.08, 0.83, 0.79, 0.71

Corresponding total number of TF hits for each TF (genome-wide):
(In the same order as above)
441, 453, 441, 421, 423, 451, 454, 437, 419, 392, 449, 421, 433, 457, 456

Corresponding total number of TF hits for each TF (in all genes in selected Phenopedia diseases):
(In the same order as above)
68, 70, 69, 63, 57, 58, 57, 55, 49, 46, 53, 50, 42, 45, 40

Corresponding number of genes (of selected Phenopedia diseases) each TF is involved with:
(In the same order as above)
19, 20, 18, 18, 15, 15, 16, 14, 14, 14, 15, 15, 9, 12, 11

Corresponding number of selected Phenopedia diseases each TF is involved with:
(In the same order as above)
7, 7, 7, 7, 7, 7, 7, 7, 7, 7, 7, 7, 7, 7, 7

Disease groups of selected Phenopedia diseases ranked according to sum of effect sizes
Nervous System Neoplasms | Nervous System Diseases | Central Nervous System Neoplasms | Central Nervous System Diseases | Neoplasms | Signs and Symptoms | Spinal Diseases | Spinal Cord Neoplasms | Spinal Cord Diseases | Musculoskeletal Diseases | Meningeal Neoplasms | Bone Diseases | Radiation Injuries | Xeroderma Pigmentosum | Skin Abnormalities | Keratosis | Infant, Newborn, Diseases | Ichthyosis | Congenital, Hereditary, and Neonatal Diseases and Abnormalities | Callosities | Head and Neck Neoplasms | Brain Neoplasms | Brain Diseases | Neoplasms, Radiation-Induced

Corresponding sum of effect sizes for each disease group of selected Phenopedia diseases
(In the same order as above)
95.23, 95.23, 70.75, 70.75, 57.01, 35.29, 29.03, 29.03, 29.03, 29.03, 29.03, 29.03, 22.51, 22.08, 22.08, 22.08, 22.08, 22.08, 22.08, 22.08, 14.78, 14.78, 14.78, 13.21

Cluster for columns 70 to 103, rows 386 to 422

Phenopedia diseases

Genital Diseases, Male | Genital Neoplasms, Male | Prostatic Diseases | Prostatic Neoplasms | Gastrointestinal Diseases | Intestinal Diseases | Carcinoma, Neuroendocrine | Carcinoma, Small Cell | Melanoma | Neuroendocrine Tumors | Stomach Diseases | Stomach Neoplasms | Carcinoma, Squamous Cell | Neoplasms, Squamous Cell | Carcinoma | Esophageal Neoplasms | Warts | Colorectal Neoplasms | Intestinal Neoplasms | Adenocarcinoma | Digestive System Neoplasms | Gastrointestinal Neoplasms | Aneuploidy | Pancreatic Diseases | Pancreatic Neoplasms | Cell Transformation, Neoplastic | Genomic Instability | Microsatellite Instability | Neoplastic Syndromes, Hereditary | Colorectal Neoplasms, Hereditary Nonpolyposis | DNA Repair-Deficiency Disorders | Carcinoma, Papillary | Adenomatous Polyposis Coli | Intestinal Polyposis

TFs

V$TBP_Q6 | V$PBX1_01 | V$TBP_01 | V$POU1F1_Q6 | V$FOXJ2_02 | V$OCT1_06 | V$NKX62_Q2 |

V$OTX_Q1 | V$HMGIY_Q3 | V$TEF_Q6 | V$OCT1_03 | V$AFP1_Q6 | V$SRY_02 | V$FOXO1_01 | V$FOXO3A_Q1 | V$CDC5_01 | V$SRY_01 | V$FOXO4_01 | V$FREAC7_01 | V$OCT1_04 | V$TATA_C | V$GATA1_05 | V$CEBPGAMMA_Q6 | V$IPF1_Q4 | V$CDX_Q5 | V$IRF1_Q6 | V$NKX3A_01 | V$HNF3_Q6 | V$HNF3ALPHA_Q6 | V$HNF3_Q6_01 | V$FOX_Q2 | V$FOXD3_01 | V$HFH3_01 | V$FOXJ2_01 | V$HNF1_Q6_01 | V$HNF1_Q6 | V$HNF1_01

Information

All related TFs:
(List of all TFs that are related to any of the PWMs)
AFP1, C/EBPgamma, CDC5L, CDX2, Cdx-1, Cdx-2, Cdx-3, FOXD3, FOXF1, FOXF2, FOXH2, FOXI1, FOXJ1, FOXJ1a, FOXJ1b, FOXJ2, FOXL1, FOXO1, FOXO4, GATA-1, GATA-1A, HFH-3, HMG, HMG-Y, HMGI-C, HNF-1alpha, HNF-1alpha-A, HNF-1alpha-B, HNF-1alpha-C, HNF-1beta, HNF-1beta-A, HNF-1beta-B, HNF-1beta-C, HNF-3, HNF-3alpha, HNF-3beta, HNF-3gamma, HNF3(-like), IPF1, IRF-1, Nkx3-1, Nkx6-2, Oct-1, Otx1, Otx2, POU2F1, POU2F1a, Pbx1a, Pit-1, Pit-1A, Pit-1B, SRY, TBP, TEF, TEF-xbb1, TFIID

Ranked gene list:
(All genes of the selected Phenopedia diseases with hits of any of the selected TFs, ranked according to the total number of TF hits)
PIK3CA, GNAS, CDKN1B, CTNNB1, TGFBR2, NFKB1, ATM, NR3C1, PIK3R1, CTLA4, KLF6, TNF, LIG4, SMAD4, BRAF, BCL2, AHR, IRS1, KRAS, CXCR4, IL7R, MTRR, IGF1, CDK6, ESR1, BCL6, PRKCQ, IGF1R, ICOS, SMAD7, RMI1, RUNX1, ARL6IP5, PTGER4, CDC14A, MAP3K1, LRMP, BACH2, PCNA, CDK4, FYN, SSH2, APC, TLR1, DPYD, MTHFR, ACVR2A, CASP3, SMAD2, NIN, ETV1, PIK3CG, NF1, GSK3B, SLC9A9, CFLAR, FAS, IL16, SDHC, REV3L, POLG2, TNRC6B, TES, PMS1, MSH2, ARHGDIB, GTDC1, CASP2, ZHX2, RGS2, APEX1, SIK3, XPC, NEIL3, HIF1AN, PSMB9, IRS2, DAD1, BCL2L1, TBR1, CDKN2C, ELAVL4, SYNE1, DCTD, AXIN2, TET2, BRCA2, XRCC4, STAT3, MSH3, LTA, PTCH1, PPARGC1A, NDUFV2, MRE11A, INSIG2, ESRRG, CASP8, GRAP2, BRIP1, CD226, RGS1, PTPRC, GTF2A1, UCP2, PRDM2, IFNG, FANK1, DICER1, CDC73, ACVR1, RECQL, NFKBIA, CDC25C, TP53, SLC39A10, ARNTL, SERPINE1, ROCK1, NCOA3, KLF12, IMMP2L, FBXW7, SMAD3, SHFM1, SH3GLB1, HAPLN1, CASP1, YPEL2, XRCC5, TIPARP, IL6R, IL2, HIF1A, FOXP2, FGFR4, ANKRD44, UGT1A1, STAT1, PDE4B, ICK, CLEC16A, C11orf30, BIRC3, RFC1, PTGER2, PIK3C3, IL2RA, FANCM, BBX, TAGAP, PIK3C2A, MYC, LBH, UGT1A6, BARD1, TNFAIP3, PTCH, MYCBP2, CCNH, AKAP9, IL21, GABPA, FH, RAP1A, PDLIM5, NDUFB8, MGMT, ITGB1, IGFBP1, IFIH1, CYP2E1, ADRB2, ABCE1, RERE, DNMT3B, UGT1A7, PTPN22, PTPN1, B2M, ZNF827, ZNF462, VDR, HECA, AKR1C3, UGT1A9, TP73, TLR3, STK11, RB1CC1, JAK1, FAM46A, DIDO1, DHX36, CHST11, APAF1, ANTXR2, YES1, SOD2, SOD1, PTTG1, POU2F1, PARP1, UGT1A4, UGT1A10, RAP1B, ZBTB20, MTHFD1, EYA1, ATXN1, TDP1, SLC39A8, LEPR, CHEK1, SERPINI2, BCL2L11, BAT1, MTIF3, JMJD1C, ERCC3, UGT1A8, UGT1A3, RIPK1, TXNRD1, TNFSF10, TARBP2, NDUFS1, F5, CCR5, CASP9, CASP10, STK38L, RAD21, IL6ST, ERBB4, ECE1, CRY2, CDC40, CD3G, ADIPOR1, UBE2B, MLH1, ZNF24, THBS1, SLC30A7, RUNX3, NRAS, LATS1, KIFAP3, ITK, ITGA6, EHBP1, BAT4, AURKA, ATR, ATP5J, AKAP2, HSPA8, BMP4, SERPINA1, NQO1, NFYC, MDM2, JAK2, HOXB7, FIGN, CHEK2, CD3D, GATA3, ZC3H10, TOPBP1, TFRC, RYK, PTEN, PIK3C2B, PER1, LYRM7, LIPC, ING1, IL8, HNF1A, GADD45A, ERCC5, DOCK7, ATP5J2, ATG2B, ATG12, TGFB1, STK17B, SPRY1, ITPR1, CDKN1A, ZBTB37, NPAT, GNAQ, EZH2, CCDC6, CASP4, BUB3, BMPR1A, ABCC1, ABCB1, TSC1, SPINK1, SEP15, MTHFD2, HOXA10, GSTM3, EXO1, EIF3H, DNAH8, CYP3A5, COL12A1, ATP2A3, ATP1B1, ATF1, SIP1, SART1, PRKCA, PLAUR, PCTK2, NDUFS6, MTMR3, MMP9, INHBA, IDE, FANCE, CARD8, BCL2L13, POMC, IL18, IGFBP3, FASLG, SMC6, RRM1, RPRM, PTK2B, NCOA4, MYNN, MDC1, IGF2, HPGD, HFE, GPX2, GART, FURIN, EIF3A, CREB5, CDC16, CD44, CAT, BAT3, THRA, MLH3, ESR2, CFTR, ZFHX3, UBASH3A, TP53BP1, TGFBR1, TAP2, RAG1, PER2, NCOA2, MYCL1, MMP3, MED13, MANBA, LIG3, KRI1, H2AFX, GPX1, CLIC1, CASP7, CAMK1D, ATBF1, UNG, TOX3, NLRP3, NHEJ1, MATR3, JAZF1, IL18R1, FTO, EMP3, CCND2, BACH1, TGFBR3, TCF7, REL, PSMB8, POU1F1, POLK, PERP, NOD2, MYH1, MBD4, MBD2, IL12B, HDAC7, FOXC1, FAF1, EML4, E2F7, DUSP23, DNMT3A, CDX2, CDKAL1, CDA, CASP6, AKT3,

AGER, TLR9, RXRA, POLI, NEIL1, IL8RA, CAPN10, TG, TBP, TACC3, SLC10A7, RXRB, RPN1, RBPJ, RAD9A, RAD18, PTPN13, PTGS1, POLS, PIK3CD, PGC, PER3, NDUFV3, NDUFB5, NDUFA9, LPP, IL1RAP, HERPUD2, H2AFY2, GPSM2, FGF10, FAM129A, EPHA3, EHMT2, DIAPH1, CDC37, CD36, CCR2, CASP8AP2, CARD15, BUB1, BICD1, ATG5, AIFM1, AGBL4, AFF3, TRIP12, TLR10, RHOG, REV1, ODC1, NOD1, HSPA1L, CLOCK, CD55, CAV1, BRD2, BLM, WRAP53, USP1, TTK, TRDMT1, TNFRSF1B, TCN2, TCF12, STK24, STCH, SPINK7, SLC40A1, PRLR, NME7, MSH5, MMP13, MEIS1, MDM4, IL13, IFNGR1, HOXA4, HNF1B, DRD2, ASCC3, AKAP10, TGFA, SLC29A1, SLC11A1, SELE, RAD50, PUM2, PRKDC, PLA2G4A, MCL1, LMAN1, KLRK1, GSTO1, GRIK2, FGF8, FANCC, DLC1, CLPTM1L, C5, BUB1B, BMPR1B, BCCIP, YIPF5, UQCRB, TP53BP2, TMPRSS11A, TGFB2, TCF2, TBPL1, STX8, SRBD1, SOX6, SLCO1B1, SLC39A9, SLA, SILV, SH3TC1, SELS, SDHD, S100A10, RAB27A, PXN, PUS10, PIK3R3, PCNX, PBX3, NPM1, NDUFS4, NDUFS2, NCOA1, MRPS6, MET, MED1, MAP2K4, MACROD2, LAMB1, JUN, ITGAV, IL15, HSPA2, GCNT2, EMCN, CYP7A1, COX7A2L, CIDEB, CDKN2D, CD83, CCR6, BNIP1, ATP5O, ATP5G2, ACP2, ZNF624, TSPAN8, RPH3AL, POLR2B, PKP4, NR1I2, MYH3, MTHFS, JMJD2C, ITPR3, ITGA7, INPPL1, HTR1B, GSTO2, GPAM, GHSR, GFRA1, DDX25, CXCR1, CTGF, CTCF, CRNN, CHST5, CDH12, CCND3, CCNA2, CCDC98, BDNF, ALS2CR12, AKR1A1, ACYP2, ABCA4, VTN, TRPV6, TMEFF2, SVIL, STAT4, SORBS2, SMARCB1, SLC22A5, SELL, SECISBP2, RNF2, RAGE, PTHLH, PSMG1, PRNP, PPP1R3A, PPM1L, POU2AF1, PIN1, PIM1, PDCD1, ORMDL3, NSUN3, LCT, ITGAL, IRF1, IRAK4, IL23R, IL17A, IDH1, HNF4A, HLA-DPB1, GSDMB, GPC5, GLI1, GCLC, FOS, FN1, FANCL, ERLIN1, DTWD2, CTBP2, CSDA, COL3A1, CDC45L, CA6, C1orf26, BAMBI, ATIC, ZWILCH, VCAN, TNKS2, THADA, SSTR3, SIAH2, RTN3, RCC2, RBBP8, PTPRCAP, POLQ, MMS19, ITGA1, IGF2AS, HSPE1, HPN, HOXB6, HADHA, GNAI2, GC, EP300, EDNRB, DLEC1, CGA, CD80, BIRC2, BIK, ARRB2, ADAMTS9, WDR44, VPS52, TSPAN18, TSHR, TSG101, TNIP1, TMX3, TERF1, TCTA, STK39, SLC12A2, SH2B3, SERPINE2, SDHA, SBK1, S100A3, REV1L, POT1, PON2, PDE11A, PAH, NSMCE2, NR5A2, NDUFB6, NCF4, N4BP2L1, MTCP1, MSX2, MRVI1, MPPED2, MITF, MARS, MAPK1, MAP3K12, MAD2L1, LSM4, KLK10, KLHL9, KIAA1109, KIAA0020, KALRN, JAK3, INHBE, IL5RA, IL28RA, ICAM5, HPS4, HPS1, HOXA13, HNF4G, HDAC9, GRM8, GDNF, FAM110B, ESRRB, EDN1, DFFA, DCLRE1C, CUL2, CSNK1E, COX6C, CDK2, CD74, CASC1, BNIP3L, BHLHB3, BGLAP, BAI3, ATP5C1, ATP5A1, ARMC2, ACSL6, ABCA5, ZWINT, XBP1, TRAF6, TP63, TDG, STK17A, SPRY2, PLAA, PLA2G6, PHB, PDGFB, PAPSS1, NCAM1, MYO5A, MNAT1, LITAF, LIMD1, ITPA, ITGB2, IRS4, IFRD1, HTR2A, HMGCR, HLA-DPA1, HLA-DMA, GCH1, FUS, FLJ78302, FGL1, CPB2, CD4, CBR3, CARD4, ATF3, AFP, ABCA1, YAF2, WDR7, VGLL4, TWIST1, TUBAL3, TRPM3, TRIT1, TRAM2, TRAF1, TOX2, TNFSF15, TMEM18, TMEM168, THSD4, TCERG1, TAP1, STYX, STX17, SRA1, SPRED1, SPEF2, SPATA17, SOCS1, SOAT2, SNX19, SLITRK1, SLC9A3R1, SLC30A1, SLC26A3, SLC24A2, SHH, SEMA4G, SDHAF2, RXFP2, REEP3, RBM19, RASSF6, RAF1, RAD23A, R3HDM1, PSORS1C1, PSMA4, PRKAR1A, PPP1CC, POLG, POLD1, P2RX6, ORC5L, NUP98, NTNG1, NRTN, NRP2, NKX2-3, NICN1, MOBKL2B, MGRN1, MBD1, MAT2A, MARCO, LY86, LRRK2, LGR8, LDLR, LACE1, KIAA1841, KIAA1274, KCNIP4, JAKMIP2, IRAK3, IL26, IL23A, IKBKAP, IK, IGFBP6, GSR, GRM1, GCKR, GABBR1, FGFR1, FGB, ESAM, EIF5A2, DAAM2, CTH, CSF2, CS, CPS1, CLSTN2, CLEC7A, CENPE, CDC25B, CD40LG, CCNY, CA9, C11orf79, BLMH, BCL10, BAT2, AURKC, ATXN7, ATG16L1, ARPC2, APP, AP3B1, ADRA1A, ZFPM1, WDR36, UCP3, TSLP, TRIP11, TRIO, TNFRSF19, TMC6, TF, TBX21, SUV39H2, SUMO1, SULF1, SRC, SPRED2, SLC30A4, SH2D1A, SEPT2, SBF1, RASA1, RAG2, PDE3A, PCTP, PAX2, PARP2, NME1, NFKBIE, MPDU1, MEST, LRP6, LMO2, LIPE, LIMK2, LCAT, KLHL10, ITGB8, IFNK, IAPP, HOXD13, HMGB2, HAO2, GRIN2B, GPR98, GPLD1, FZD7, FANCB, EZR, EXT1, ERO1LB, DNAH5, DAZ3, DAZ2, CSTF1, CHUK, CENPF, CDYL, CDK8, CD244, BTG4, B9D2, ATXN3, ATP1B2, ATN1, ARHGEF11, APOA5, APOA2, ALDH1A1, ACO1, ACHE, ABCC4

Corresponding total number of TF hits:
(For each gene listed above, the total number of TF hits for any of the selected TFs, multiplied by the number of selected Phenopedia diseases containing that gene)
756, 720, 672, 616, 494, 484, 484, 442, 408, 396, 390, 377, 350, 342, 330, 330, 324, 320, 310, 300, 288, 275, 264, 264, 260, 259, 231, 231, 231, 225, 225, 216, 216, 210, 200, 189, 189, 182, 176, 176, 174, 170, 165, 156, 156, 155, 154, 147, 144, 144, 144, 140, 140, 135, 133, 133, 132, 130, 126, 126, 126, 120, 120, 120, 120, 120, 112, 110, 108, 104, 104, 102, 100, 99, 99, 98, 98, 98, 98, 96, 96, 95, 91, 90, 90, 88, 87, 84,

84, 84, 84, 80, 80, 80, 80, 80, 80, 80, 78, 77, 76, 75, 75, 75, 72, 72, 72, 72, 72, 72, 72, 70, 70, 70, 68, 68, 68, 66, 66, 65, 64, 64, 64, 63, 63, 63, 63, 63, 60, 60, 60, 60, 60, 60, 60, 58, 57, 56, 56, 56, 56, 56, 56, 54, 54, 54, 54, 54, 54, 52, 52, 52, 52, 51, 51, 50, 50, 50, 50, 50, 49, 49, 49, 48, 48, 48, 48, 48, 48, 48, 48, 48, 48, 46, 46, 45, 45, 45, 45, 44, 44, 44, 44, 44, 42, 42, 42, 42, 42, 42, 42, 42, 42, 42, 42, 42, 40, 40, 40, 40, 40, 40, 39, 39, 39, 38, 38, 38, 37, 36, 36, 36, 36, 35, 35, 35, 34, 34, 34, 33, 33, 33, 32, 32, 32, 32, 32, 32, 32, 32, 30, 30, 30, 30, 30, 30, 30, 30, 29, 29, 28, 28, 28, 28, 28, 28, 28, 28, 28, 28, 28, 28, 28, 28, 27, 27, 26, 26, 26, 26, 26, 26, 26, 26, 26, 25, 24, 24, 24, 24, 24, 24, 24, 24, 24, 24, 24, 24, 24, 24, 24, 24, 24, 23, 23, 23, 23, 23, 22, 22, 22, 22, 22, 22, 22, 22, 22, 22, 21, 21, 21, 21, 21, 21, 21, 21, 21, 21, 21, 21, 21, 20, 20, 20, 20, 20, 20, 20, 20, 20, 20, 20, 20, 20, 19, 19, 19, 19, 18, 18, 18, 18, 18, 18, 18, 18, 18, 18, 18, 18, 18, 18, 18, 18, 18, 18, 18, 17, 17, 17, 17, 16, 16, 16, 16, 16, 16, 16, 16, 16, 16, 16, 16, 16, 16, 16, 16, 16, 16, 16, 16, 15, 15, 15, 15, 15, 15, 15, 15, 15, 15, 15, 14, 14, 14, 14, 14, 14, 14, 14, 14, 14, 14, 14, 14, 14, 14, 14, 14, 14, 14, 14, 14, 14, 14, 14, 14, 13, 13, 13, 13, 13, 13, 12, 12, 12, 12, 12, 12, 12, 12, 12, 12, 12, 12, 12, 12, 12, 12, 12, 12, 12, 12, 12, 12, 12, 12, 12, 12, 12, 12, 12, 12, 12, 12, 12, 12, 12, 12, 12, 11, 11, 11, 11, 11, 11, 11, 11, 11, 11, 11, 11, 10, 10, 10, 10, 10, 10, 10, 10, 10, 10, 10, 10, 10, 10, 10, 10, 10, 10, 10, 10, 10, 10, 10, 10, 9, 9, 9, 9, 9, 9, 9, 9, 9, 9, 9, 9, 9, 9, 9, 9, 9, 9, 9, 9, 9, 8, 8, 8, 8, 8, 8, 8, 8, 8, 8, 8, 8, 8, 8, 8, 8, 8, 8, 8, 8, 8, 8, 8, 8, 8, 8, 8, 8, 8, 8, 8, 8, 8, 8, 8, 8, 8, 8, 8, 8, 8, 8, 8, 8, 8, 8, 8, 8, 8, 8, 7, 7, 7, 7, 7, 7, 7, 7, 7, 7, 7, 7, 7, 7, 7, 7, 7, 7, 7, 7, 7, 7, 7, 7, 7, 7, 7, 7, 7, 7, 7, 7, 6, 6, 6, 6, 6, 6, 6, 6, 6, 6, 6, 6, 6, 6, 6, 6, 6, 6, 6, 6, 6, 6, 6, 6, 6, 6, 6, 6, 6, 6, 6, 6, 6, 6, 6, 6, 6, 6, 6, 6, 6, 6, 6, 6, 6, 6, 6, 6, 6, 5, 5, 5, 5, 5, 5, 5, 5, 5, 5, 5, 5, 5, 5, 5, 5, 5, 5, 5, 5, 5, 5, 5, 5, 5, 5, 5, 5, 5, 4, 4, 4, 4, 4, 4, 4, 4, 4, 4, 4, 4, 4, 4, 4, 4, 4, 4, 4, 4, 4, 4, 4, 4, 4, 4, 4, 4, 4, 4, 4, 4, 4, 4, 4, 4, 4, 4, 4, 4, 4, 4, 4, 4, 4, 4, 4, 4, 4, 4, 4, 4, 4, 4, 4, 4, 4, 4, 4, 4, 4, 4, 4, 4, 4, 4, 4, 4, 4, 4, 4, 4, 4, 3, 3, 3, 3, 3, 3, 3, 3, 3, 3, 3, 3, 3, 3, 3, 3, 3, 3, 3, 3, 3, 3, 3, 3, 3, 3, 3, 3, 3, 3, 3, 3, 3, 3, 3, 2, 1, 1, 1, 1, 1, 1, 1, 1, 1, 1, 1, 1, 1, 1, 1, 1, 1, 1, 1, 1, 1, 1, 1, 1, 1, 1, 1, 1, 1, 1, 1, 1, 1, 1, 1, 1, 1, 1, 1, 1, 1, 1, 1, 1, 1, 1, 1, 1, 1, 1, 1, 1, 1, 1, 1, 1, 1, 1, 1, 1, 1, 1, 1, 1, 1, 1, 1, 1, 1, 1

Corresponding number of selected Phenopedia diseases each gene is involved with:
(In the same order as above)
28, 20, 21, 22, 19, 22, 22, 17, 12, 18, 13, 29, 25, 19, 30, 15, 18, 16, 31, 10, 8, 25, 24, 11, 20, 7, 7, 21, 11, 9, 15, 6, 9, 6, 10, 7, 7, 7, 11, 11, 6, 10, 33, 12, 12, 31, 11, 21, 12, 6, 6, 7, 5, 9, 7, 7, 22, 13, 14, 9, 7, 4, 4, 15, 30, 5, 7, 11, 6, 8, 26, 6, 25, 9, 11, 7, 14, 7, 14, 6, 3, 5, 7, 5, 15, 4, 29, 21, 6, 21, 21, 8, 10, 4, 10, 8, 8, 20, 3, 11, 4, 3, 3, 5, 6, 8, 24, 2, 8, 4, 4, 10, 14, 7, 34, 4, 4, 22, 6, 5, 2, 2, 4, 7, 7, 9, 7, 9, 6, 15, 2, 12, 20, 20, 2, 20, 2, 19, 4, 2, 2, 7, 7, 7, 18, 6, 9, 9, 6, 2, 2, 13, 13, 2, 17, 17, 2, 5, 2, 10, 10, 7, 7, 7, 3, 4, 4, 24, 4, 16, 8, 24, 12, 4, 2, 23, 15, 9, 5, 5, 2, 4, 22, 2, 11, 14, 21, 6, 14, 7, 3, 7, 7, 6, 6, 7, 2, 2, 20, 8, 4, 2, 20, 13, 13, 3, 2, 19, 2, 1, 6, 4, 18, 18, 7, 7, 7, 2, 1, 17, 11, 11, 11, 8, 2, 8, 4, 16, 16, 16, 16, 2, 6, 3, 15, 5, 6, 6, 2, 6, 1, 29, 7, 14, 4, 7, 14, 7, 4, 2, 4, 4, 7, 28, 14, 4, 7, 1, 9, 13, 26, 2, 26, 13, 2, 2, 26, 2, 1, 2, 6, 8, 6, 24, 4, 4, 2, 8, 6, 24, 8, 6, 24, 8, 4, 12, 12, 23, 1, 1, 1, 23, 2, 1, 2, 11, 1, 11, 1, 11, 11, 22, 7, 7, 7, 7, 1, 21, 21, 7, 7, 21, 7, 7, 7, 7, 2, 10, 10, 5, 1, 4, 10, 20, 4, 5, 5, 10, 4, 19, 19, 19, 19, 6, 9, 6, 3, 1, 1, 9, 18, 6, 18, 9, 6, 6, 6, 6, 6, 1, 18, 9, 1, 17, 17, 17, 4, 4, 16, 16, 8, 8, 4, 4, 16, 16, 1, 8, 16, 2, 4, 16, 2, 16, 4, 4, 5, 5, 3, 3, 1, 5, 5, 5, 5, 1, 1, 1, 1, 2, 1, 7, 7, 7, 14, 14, 14, 7, 14, 2, 7, 2, 2, 2, 7, 7, 7, 7, 14, 14, 1, 2, 13, 13, 13, 13, 13, 13, 3, 1, 6, 2, 6, 4, 2, 6, 12, 12, 12, 2, 4, 6, 4, 4, 4, 4, 4, 2, 2, 4, 6, 2, 2, 3, 12, 2, 6, 6, 12, 6, 12, 4, 4, 12, 4, 2, 2, 1, 11, 1, 11, 11, 11, 11, 11, 1, 11, 1, 11, 5, 5, 5, 5, 10, 10, 1, 2, 5, 10, 1, 5, 2, 2, 10, 2, 2, 10, 10, 1, 5, 10, 10, 10, 9, 9, 9, 9, 9, 1, 9, 9, 3, 3, 9, 9, 1, 1, 9, 9, 9, 3, 9, 9, 9, 2, 4, 8, 8, 2, 4, 1, 2, 2, 2, 8, 4, 2, 2, 2, 8, 8, 1, 1, 1, 2, 4, 2, 2, 2, 4, 4, 4, 2, 8, 4, 8, 2, 2, 2, 4, 8, 4, 4, 2, 8, 4, 4, 1, 4, 2, 4, 4, 4, 4, 7, 7, 7, 7, 7, 7, 7, 7, 7, 1, 7, 7, 1, 7, 7, 7, 7, 7, 7, 7, 1, 7, 7, 7, 7, 7, 7, 1, 7, 7, 7, 7, 1, 6, 2, 2, 2, 2, 3, 6, 2, 6, 3, 6, 3, 2, 6, 6, 2, 6, 3, 1, 6, 2, 2, 6, 3, 3, 6, 6, 6, 6, 2, 6, 2, 3, 3, 2, 1, 3, 6, 1, 2, 6, 2, 3, 6, 6, 2, 6, 6, 1, 5, 1, 5, 5, 1, 1, 5, 5, 5, 5, 5, 1, 5, 1, 5, 1, 5, 1, 5, 1, 5, 5, 5, 5, 1, 5, 1, 5, 2, 4, 2, 4, 1, 1, 2, 4, 1, 1, 2, 2, 2, 4, 2, 1, 4, 2, 2, 2, 4, 4, 4, 4, 2, 1, 4, 2, 4, 2, 2, 4, 1, 1, 4, 2, 4, 1, 2, 2, 4, 2, 2, 1, 2, 4, 2, 4, 2, 2, 1, 2, 4, 2, 4, 2, 2, 1, 2, 4, 4, 1, 2, 2, 4, 2, 4, 2, 4, 4, 4, 4, 1, 1, 1, 3, 1, 1, 1, 3, 1, 3, 1, 3, 3, 1, 3, 3, 1, 3, 3, 3, 1, 1, 3, 3, 3, 1, 1, 1, 3, 3, 3, 1, 1, 3, 1, 3, 1, 2, 2, 2, 1, 2, 2, 2, 2, 2, 2, 2, 2, 2, 2, 2, 2, 1, 2, 1, 1, 2, 1, 2, 1, 1, 2, 2, 1, 2, 2, 1, 2, 1, 1, 1, 2, 1, 1, 2, 2, 2, 2, 1, 1, 2, 2, 2, 2, 2, 2, 2, 2, 2, 1, 2, 2, 2, 1, 2, 2, 2, 1, 2, 2, 2, 2, 2, 2, 2, 2, 2, 2, 2, 1, 2, 2, 2, 1, 1, 2, 2, 1, 2, 1, 2, 1, 2, 2, 2, 1, 1, 1, 2, 2, 1, 2, 2, 2, 1, 1, 2, 2, 2, 2, 2, 1, 1, 1, 1, 1, 1, 1, 1, 1, 1, 1, 1, 1, 1, 1, 1, 1, 1, 1, 1, 1, 1, 1, 1, 1, 1, 1, 1, 1, 1, 1, 1, 1, 1, 1, 1, 1, 1, 1, 1, 1, 1, 1, 1, 1, 1, 1, 1, 1, 1, 1, 1, 1, 1, 1, 1, 1, 1, 1, 1, 1, 1, 1, 1, 1, 1, 1, 1, 1, 1, 1, 1, 1

Corresponding number of selected TFs each gene is involved with:

(In the same order as above)
27, 36, 32, 28, 26, 22, 22, 26, 34, 22, 30, 13, 14, 18, 11, 22, 18, 20, 10, 30, 36, 11, 11, 24, 13, 37, 33, 11, 21, 25, 15, 36, 24, 35, 20, 27, 27, 26, 16, 16, 29, 17, 5, 13, 13, 5, 14, 7, 12, 24, 24, 20, 28, 15, 19, 19, 6, 10, 9, 14, 18, 30, 30, 8, 4, 24, 16, 10, 18, 13, 4, 17, 4, 11, 9, 14, 7, 14, 7, 16, 32, 19, 13, 18, 6, 22, 3, 4, 14, 4, 4, 10, 8, 20, 8, 10, 10, 4, 26, 7, 19, 25, 25, 15, 12, 9, 3, 36, 9, 18, 18, 7, 5, 10, 2, 17, 17, 3, 11, 13, 32, 32, 16, 9, 9, 7, 9, 7, 10, 4, 30, 5, 3, 3, 30, 3, 29, 3, 14, 28, 28, 8, 8, 8, 3, 9, 6, 6, 9, 27, 26, 4, 4, 26, 3, 3, 25, 10, 25, 5, 5, 7, 7, 7, 16, 12, 12, 2, 12, 3, 6, 2, 4, 12, 23, 2, 3, 5, 9, 9, 22, 11, 2, 22, 4, 3, 2, 7, 3, 6, 14, 6, 6, 7, 7, 6, 21, 20, 2, 5, 10, 20, 2, 3, 3, 13, 19, 2, 19, 37, 6, 9, 2, 2, 5, 5, 5, 17, 34, 2, 3, 3, 3, 4, 16, 4, 8, 2, 2, 2, 2, 15, 5, 10, 2, 6, 5, 5, 15, 5, 29, 1, 4, 2, 7, 4, 2, 4, 7, 14, 7, 7, 4, 1, 2, 7, 4, 27, 3, 2, 1, 13, 1, 2, 13, 13, 1, 13, 25, 12, 4, 3, 4, 1, 6, 6, 12, 3, 4, 1, 3, 4, 1, 3, 6, 2, 2, 1, 23, 23, 23, 1, 11, 22, 11, 2, 22, 2, 22, 2, 2, 1, 3, 3, 3, 3, 21, 1, 1, 3, 3, 1, 3, 3, 3, 3, 10, 2, 2, 4, 20, 5, 2, 1, 5, 4, 4, 2, 5, 1, 1, 1, 1, 3, 2, 3, 6, 18, 18, 2, 1, 3, 1, 2, 3, 3, 3, 3, 3, 18, 1, 2, 17, 1, 1, 1, 4, 4, 1, 1, 2, 2, 4, 4, 1, 1, 16, 2, 1, 8, 4, 1, 8, 1, 4, 4, 3, 3, 5, 5, 15, 3, 3, 3, 3, 15, 15, 14, 14, 7, 14, 2, 2, 2, 1, 1, 1, 2, 1, 7, 2, 7, 7, 7, 2, 2, 2, 2, 1, 1, 14, 7, 1, 1, 1, 1, 1, 1, 4, 12, 2, 6, 2, 3, 6, 2, 1, 1, 1, 6, 3, 2, 3, 3, 3, 3, 3, 6, 6, 3, 2, 6, 6, 4, 1, 6, 2, 2, 1, 2, 1, 3, 3, 1, 3, 6, 6, 11, 1, 11, 1, 1, 1, 1, 1, 11, 1, 11, 1, 2, 2, 2, 2, 1, 1, 10, 5, 2, 1, 10, 2, 5, 5, 1, 5, 5, 1, 1, 10, 2, 1, 1, 1, 1, 1, 1, 1, 1, 9, 1, 1, 3, 3, 1, 1, 9, 9, 1, 1, 1, 3, 1, 1, 1, 4, 2, 1, 1, 4, 2, 8, 4, 4, 4, 1, 2, 4, 4, 4, 1, 1, 8, 8, 8, 4, 2, 4, 4, 4, 2, 2, 2, 4, 1, 2, 1, 4, 4, 4, 2, 1, 2, 2, 4, 1, 2, 2, 8, 2, 4, 2, 2, 2, 2, 1, 1, 1, 1, 1, 1, 1, 1, 1, 7, 1, 1, 7, 1, 1, 1, 1, 1, 1, 1, 7, 1, 1, 1, 1, 1, 1, 7, 1, 1, 1, 1, 6, 1, 3, 3, 3, 3, 2, 1, 3, 1, 2, 1, 2, 3, 1, 1, 3, 1, 2, 6, 1, 3, 3, 1, 2, 2, 1, 1, 1, 1, 3, 1, 3, 2, 2, 3, 6, 2, 1, 6, 3, 1, 3, 2, 1, 1, 3, 1, 1, 5, 1, 5, 1, 1, 5, 5, 1, 1, 1, 1, 1, 5, 1, 5, 1, 5, 1, 5, 1, 5, 1, 1, 1, 1, 5, 1, 5, 1, 2, 1, 2, 1, 4, 4, 2, 1, 4, 4, 2, 2, 2, 1, 2, 4, 1, 2, 2, 2, 1, 1, 1, 1, 2, 4, 1, 2, 1, 2, 2, 1, 4, 4, 1, 2, 1, 4, 2, 2, 1, 2, 2, 4, 2, 1, 2, 1, 2, 2, 4, 2, 1, 2, 1, 2, 2, 4, 2, 1, 1, 4, 2, 2, 1, 2, 1, 2, 1, 1, 1, 1, 4, 3, 3, 1, 3, 3, 3, 1, 3, 1, 3, 1, 1, 3, 1, 1, 3, 1, 1, 1, 3, 3, 1, 1, 1, 3, 3, 3, 1, 1, 1, 3, 3, 1, 3, 1, 3, 1, 1, 1, 2, 1, 1, 1, 1, 1, 1, 1, 1, 1, 1, 1, 1, 2, 1, 2, 2, 1, 2, 1, 2, 2, 1, 1, 2, 1, 1, 2, 1, 2, 2, 2, 1, 2, 2, 1, 1, 1, 1, 2, 2, 1, 1, 1, 1, 1, 1, 1, 1, 1, 2, 1, 1, 1, 2, 1, 1, 1, 2, 1, 1, 1, 1, 1, 1, 1, 1, 1, 1, 2, 1, 1, 1, 2, 2, 1, 1, 2, 1, 2, 1, 2, 1, 1, 1, 2, 2, 2, 1, 1, 2, 1, 1, 1, 2, 2, 1, 1, 1, 1, 1, 1, 1, 1, 1, 1, 1, 1, 1, 1, 1, 1, 1, 1, 1, 1, 1, 1, 1, 1, 1, 1, 1, 1, 1, 1, 1, 1, 1, 1, 1, 1, 1, 1, 1, 1, 1, 1, 1, 1, 1, 1, 1, 1, 1, 1, 1, 1, 1, 1, 1, 1, 1, 1, 1, 1, 1, 1, 1, 1, 1, 1, 1, 1, 1, 1, 1, 1, 1, 1, 1, 1, 1

Phenopedia diseases ranked according to mean of effect sizes in cluster:
Intestinal Diseases | Gastrointestinal Diseases | Pancreatic Neoplasms | Prostatic Neoplasms | Prostatic Diseases | Genital Neoplasms, Male | Genomic Instability | Neoplastic Syndromes, Hereditary | Genital Diseases, Male | Digestive System Neoplasms | Pancreatic Diseases | Adenocarcinoma | Microsatellite Instability | Gastrointestinal Neoplasms | Carcinoma, Neuroendocrine | Adenomatous Polyposis Coli | DNA Repair-Deficiency Disorders | Neuroendocrine Tumors | Intestinal Polyposis | Stomach Neoplasms | Colorectal Neoplasms | Stomach Diseases | Carcinoma, Squamous Cell | Intestinal Neoplasms | Esophageal Neoplasms | Carcinoma | Neoplasms, Squamous Cell | Warts | Aneuploidy | Colorectal Neoplasms, Hereditary Nonpolyposis | Carcinoma, Small Cell | Melanoma | Carcinoma, Papillary | Cell Transformation, Neoplastic

Corresponding mean of effect sizes of each Phenopedia disease:
(In the same order as above)
2.27, 2.23, 2.22, 2.09, 2.08, 2.04, 1.91, 1.78, 1.76, 1.75, 1.74, 1.40, 1.40, 1.39, 1.33, 1.30, 1.28, 1.27, 1.22, 1.21, 1.20, 1.18, 1.16, 1.15, 1.13, 1.08, 1.03, 0.97, 0.96, 0.82, 0.78, 0.73, 0.73, 0.50

Corresponding total number of genes of each Phenopedia disease:
(In the same order as above)
990, 1.2K, 219, 504, 505, 520, 161, 112, 662, 884, 318, 704, 105, 735, 93, 38, 103, 233, 37, 273, 437, 295, 306, 440, 464, 1.2K, 330, 354, 70, 51, 76, 182, 45, 79

Corresponding number of genes of each Phenopedia disease with at least one TF hit (of selected TFs):
(In the same order as above)
437, 541, 115, 230, 231, 235, 80, 61, 291, 393, 148, 327, 51, 329, 44, 22, 45, 115, 22, 125, 208, 132, 138, 209, 208, 532, 147, 165, 42, 22, 33, 83, 18, 30

Corresponding total number of TF hits (of selected TFs) for each Phenopedia disease (in all genes):
(In the same order as above)

2554, 3130, 820, 1354, 1355, 1378, 488, 443, 1644, 2229, 949, 1758, 343, 1803, 252, 135, 304, 642, 112, 758, 1096, 771, 770, 1100, 1180, 2865, 810, 940, 228, 152, 155, 422, 118, 166

Corresponding number of selected TFs each Phenopedia disease is involved with:
(In the same order as above)
37, 37, 37, 37, 37, 37, 37, 37, 37, 37, 37, 37, 37, 37, 37, 37, 37, 37, 36, 37, 37, 37, 37, 37, 37, 37, 37, 37, 37, 37, 37, 37, 35, 37

TFs ranked according to mean of effect sizes in cluster:
VTBP_01, VCDC5_01, VTBP_Q6, VNKX3A_01, V$NKX62_Q2, V$POU1F1_Q6, VSRY_02, VOTX_Q1, V$PBX1_01, V$TEF_Q6, V$AFP1_Q6, V$OCT1_04, V$FOXO3A_Q1, V$FOXJ2_02, V$FOXO4_01, V$FREAC7_01, V$CEBPGAMMA_Q6, V$HMGIY_Q3, V$OCT1_06, V$HNF1_01, V$FOXO1_01, V$HNF1_Q6, V$HNF1_Q6_01, V$TATA_C, VSRY_01, VHFH3_01, VFOX_Q2, VHNF3ALPHA_Q6, V$IRF1_Q6, V$HNF3_Q6_01, V$GATA1_05, V$CDX_Q5, V$FOXJ2_01, V$OCT1_03, V$FOXD3_01, V$HNF3_Q6, V$IPF1_Q4

Corresponding mean of effect sizes of each TF:
(In the same order as above)
2.55, 2.40, 2.24, 2.11, 2.08, 2.02, 2.01, 1.90, 1.89, 1.83, 1.77, 1.74, 1.60, 1.52, 1.50, 1.49, 1.47, 1.47, 1.37, 1.30, 1.26, 1.24, 1.23, 1.10, 1.08, 1.00, 0.99, 0.96, 0.84, 0.82, 0.80, 0.77, 0.75, 0.72, 0.64, 0.51, 0.21

Corresponding total number of TF hits for each TF (genome-wide):
(In the same order as above)
548, 538, 579, 531, 540, 564, 545, 554, 554, 574, 551, 542, 565, 548, 519, 561, 544, 570, 574, 499, 550, 532, 521, 552, 572, 546, 543, 551, 542, 519, 559, 549, 522, 513, 536, 563, 521

Corresponding total number of TF hits for each TF (in all genes in selected Phenopedia diseases):
(In the same order as above)
1006, 1004, 1052, 919, 931, 1102, 941, 938, 1035, 937, 962, 968, 904, 923, 986, 965, 980, 957, 941, 902, 815, 959, 960, 917, 973, 816, 745, 790, 845, 755, 949, 687, 779, 744, 738, 752, 647

Corresponding number of genes (of selected Phenopedia diseases) each TF is involved with:
(In the same order as above)
150, 148, 161, 133, 150, 165, 150, 145, 152, 149, 145, 160, 142, 140, 143, 149, 146, 156, 159, 132, 126, 142, 150, 153, 153, 136, 143, 136, 138, 132, 151, 123, 125, 140, 136, 140, 126

Corresponding number of selected Phenopedia diseases each TF is involved with:
(In the same order as above)
34, 34, 34, 34, 34, 34, 34, 34, 34, 34, 34, 34, 34, 34, 34, 34, 34, 34, 34, 34, 34, 33, 33, 34, 34, 34, 34, 34, 34, 34, 34, 34, 34, 34, 34, 34, 33

Disease groups of selected Phenopedia diseases ranked according to sum of effect sizes
Neoplasms | Digestive System Diseases | Gastrointestinal Diseases | Digestive System Neoplasms | Male Urogenital Diseases | Genital Diseases, Male | Gastrointestinal Neoplasms | Intestinal Diseases | Abdominal Neoplasms | Prostatic Diseases | Pelvic Neoplasms | Genital Neoplasms, Male | Pancreatic Diseases | Signs and Symptoms | Neoplasms, Glandular and Epithelial | Genomic Instability | Intestinal Neoplasms | Neuroendocrine Tumors | Neoplastic Syndromes, Hereditary | Genetic Diseases, Inborn | Carcinoma | Stomach Diseases | Pancreatic Neoplasms | Endocrine System Diseases | Endocrine Gland Neoplasms | DNA Repair-Deficiency Disorders | Prostatic Neoplasms | Colorectal Neoplasms | Neoplasms, Squamous Cell | Adenoma, Oxyphilic | Adenocarcinoma | Microsatellite Instability | Carcinoma, Neuroendocrine | Polyps | Adenomatous Polyposis Coli | Intestinal Polyposis | Stomach Neoplasms | Carcinoma, Squamous Cell | Esophageal Neoplasms | Esophageal Diseases | Warts | Skin Diseases, Viral | Skin Diseases, Infectious | Skin Diseases | Chromosome Aberrations | Aneuploidy | Colorectal Neoplasms, Hereditary Nonpolyposis | Carcinoma, Small Cell | Melanoma | Carcinoma, Papillary | Neoplastic Processes | Cell Transformation, Neoplastic

Corresponding sum of effect sizes for each disease group of selected Phenopedia diseases
(In the same order as above)
991.09, 721.40, 657.12, 401.84, 294.79, 294.79, 254.92, 246.41, 194.32, 154.29, 152.69, 152.69, 146.60, 144.18, 133.94, 122.29, 117.16, 96.37, 96.17, 96.17, 95.93, 88.38, 82.32, 82.32, 82.32, 77.49, 77.16, 74.62, 65.03, 51.81, 51.81, 51.73, 49.38, 48.01, 48.01, 45.11, 44.76, 43.06, 41.74, 41.74, 36.06, 36.06, 36.06, 36.06, 35.67, 35.67, 30.25, 28.86, 27.10, 27.02, 18.35, 18.35

Cluster for columns 5 to 20, rows 145 to 176

Phenopedia diseases

Celiac Disease | Demyelinating Diseases | Multiple Sclerosis | Sclerosis | Acquired Immunodeficiency Syndrome | HIV Seropositivity | Connective Tissue Diseases | Lupus Erythematosus, Systemic | Autoimmune Diseases | Immune System Diseases | Diabetes Mellitus, Type 1 | Arthritis, Juvenile Rheumatoid | Arthritis | Arthritis, Rheumatoid | Joint Diseases | Rheumatic Diseases

TFs

V$GATA_Q6 | V$GATA6_01 | B$CRP_C | V$PAX2_02 | V$AREB6_04 | V$BRCA_01 | V$STAT5A_04 | V$STAT5A_03 | V$STAT4_01 | V$STAT6_01 | V$STAT1_03 | V$HMGIY_Q6 | V$TST1_01 | V$HOXA4_Q2 | V$TCF4_Q5 | V$LEF1_Q2 | V$SOX10_Q6 | V$DBP_Q6 | V$CEBP_Q3 | V$HSF2_01 | V$HSF1_01 | V$NKX25_Q5 | V$ELF1_Q6 | V$CEBP_Q2 | V$CEBPB_02 | V$CEBP_Q2_01 | V$CEBPB_01 | V$CEBPA_01 | V$NFAT_Q6 | V$NFAT_Q4_01 | V$GFI1_Q6 | V$FOXP3_Q4

Information

All related TFs:
(List of all TFs that are related to any of the PWMs)
ANF-2, BRCA1, BRCA1:USF2, C/EBP, C/EBPalpha, C/EBPalpha(p20), C/EBPalpha(p30), C/EBPbeta, C/EBPbeta(LAP), C/EBPbeta(p20), C/EBPbeta(p34), C/EBPbeta(p35), C/EBPdelta, C/EBPepsilon, C/EBPgamma, DBP, ELF-1, Elf-1, FOXP3, GATA-1, GATA-1A, GATA-2, GATA-3, GATA-4, GATA-5, GATA-5A, GATA-5B, GATA-6, GATA-6A, GATA-6B, Gfi1, Gfi1b, HMG, HMG-Y, HMGI-C, HOXA4, HSF1, HSF1-L, HSF1-S, HSF1long, HSF1short, HSF2, HSF2A, LEF-1, LEF-1S, NF-AT, NF-AT1, NF-AT1C, NF-AT2, NF-AT3, NF-AT4, Nkx2-5, Nkx2.5, POU3F1, Pax-2, Pax-2.1, Pax-2.2, Pax-2a, Pax-2b, STAT1, STAT1alpha, STAT4, STAT5A, STAT6, Sox10, TCF-1, TCF-1(P), TCF-3, TCF-4, ZEB

Ranked gene list:
(All genes of the selected Phenopedia diseases with hits of any of the selected TFs, ranked according to the total number of TF hits)
TNF, PSMB8, LTA, TNFAIP3, CLEC16A, IRF1, PSMB9, TAP1, IL7R, DUSP1, C6orf47, CDK6, CTLA4, RUNX1, TAPBP, CD4, CCR7, VDR, LTB, SH2B3, TNFRSF1A, CXCR4, TAP2, PRDM1, KLF12, APOM, ZFP36, PRKCQ, STAT1, MAP3K14, GIMAP5, TCF7, CD3G, NFKBIA, CD44, MSH5, NR3C1, CLIC1, PTGER4, PTPN6, FLI1, IL11RA, COL11A2, DDAH2, AGER, NFKBIE, UCP2, TAGAP, ADRB2, CD226, TRAF5, PLCL2, LAG3, IKBKE, FYN, BTG1, NKIRAS2, BAT1, PLCG1, CD247, SYNGAP1, RXRB, RNF5, CSNK2B, BAT4, AGPAT1, RING1, CNTF, ADORA2A, HSPA1L, RBPJ, PPP1R10, MRPS18B, IKZF1, ETS1, TGFBR2, SLC9A3R1, IL10RA, CUTA, BCL2L1, MAP3K1, SLC39A7, HSD17B8, SOCS1, MGAT5, TNIP1, BRD2, MAL, LY6G5B, IL2RA, IL16, BCL2, GFI1, LST1, BACH2, PSTPIP1, PPP1R11, IL23A, BAT3, BAK1, ZNRD1, ICOS, VEGFA, GABBR1, C6orf26, IRS1, DBP, CYP27B1, STAT6, DAXX, ANP32A, PDE4B, TRADD, VEGF, STAT3, ATP1A1, HDAC7, BCL6, FANK1, RAB5B, CD3D, TNFAIP1, PFDN6, NRM, IMPDH2, IL21R, FAS, TIPARP, SMAD7, SELPLG, IDDM12, CBLB, TRAF1, TG, HLA-DMA, SMAD3, HCLS1, SPRED2, RGL2, IFNGR1, ESR1, PDGFB, IL6R, IL6, HLA-B, VPS52, RPS18, HMHA1, PTPN22, PIK3R1, KIF21B, HLA-C, TNFRSF6B, KIAA1949, IL1RAP, CFLAR, CDK2, PHF1, PDCD1, NHEJ1, EGR2,

C6orf134, B3GALT4, TAC3, NDUFS2, RUNX3, UBASH3A, TUBB, LBH, ATP6V1G2, S100A5, GNAS, CDK4, TBX21, S100A6, IL2, GATA3, ERAP1, CASP2, APEX1, CDKN1B, ORMDL3, IGF1, GSDMB, CD69, CD2, ARID5B, ANG, RNPEPL1, RASGRP1, JUN, CDKN2C, ZC3H10, TNFRSF1B, MLL, IFIH1, HLA-DPB1, CYFIP2, CHST11, WNT10B, ST8SIA1, PTPRA, HSPA8, DCTN1, UNC13D, UCP3, TP53, SOCS3, SHMT2, PCNA, NFKB1, MYD88, MMP9, IL6ST, FOXJ1, ANTXR2, MYCBP2, ETV6, DUSP2, XAB2, S100A4, ZHX2, MTIF3, ERAP2, TGFBR3, RHOG, RERE, PTPN11, PTGER2, LIF, ITPR1, ATP1B1, TGFB2, SOS1, SDCCAG8, PRRT1, PLCD1, NLRP3, IRF4, GRK5, CEP170, BDNF, ZNF827, POLS, ITGB7, EGR1, BCL2L11, ARRB2, TYROBP, TRIM72, PROX1, PBX2, ITPA, IGF1R, EHMT2, EDN1, BBX, TGFBRAP1, SOX2, RHOC, NF1, MMACHC, LIG4, CDKN1A, CAST, BSCL2, SELS, RGS1, PAX5, NOD2, ITGAV, ICAM3, HLA-DPA1, H2AFX, GYS1, GSK3B, GSDML, UNC84B, STAT4, SLA, CD28, CD14, AKT1, ZBTB9, TRIM5, SMAD5, SLC23A1, SH2D2A, PTK2B, PRF1, PPARA, PLCB2, OAZ1, NOTCH4, NOS3, NOS2A, MTRR, MDC1, LRCH1, IRF5, ICK, ECE2, CNDP2, CEBPE, CD40, CARD15, TRAF3, TLR9, SLC22A4, S100A10, PARP1, MYB, BAT2, ZAP70, TET2, STAT5A, POU2AF1, NR2F2, NOS2, MST1, MAN2A1, JAK1, HSPD1, HSP90AB1, EIF2B4, CASP8, AIF1, ADAR, ACCN2, WNK1, PSORS1, PRKCB1, ITPR3, F5, DDR1, CXXC5, CD19, BRCA2, AFF3, ZPBP2, UBE2H, TRIM21, TPMT, TNFSF10, SOCS2, RDBP, RAD51L1, RAB27A, MPG, LY6G6C, LY6G5C, IL2RB, HSPA2, HOXB9, ERBB3, COTL1, CD79A, CD27, CCR6, C6orf48, ARHGEF12, APOA2, ANKRD44, VIPR1, TTC7A, TNIP2, TNFSF4, TAX1BP1, SLC19A1, SELL, RNF39, PIK3CD, MAP3K7IP2, LCK, IL1F6, IK, FOS, FEN1, FCN3, COL6A1, B2M, ANKH, ADAM17, YES1, XYLT1, XRCC4, TRAF2, S100A3, RGS2, PRDX5, PPARGC1A, PNKP, OAS3, NGFR, MYC, LEP, KRAS, ITGB2, IRF8, IFNAR1, HMGB1, HDAC5, HCP5, GCKR, CYP2R1, C6orf25, C10orf2, ADCY7, ZMIZ1, UBAC2, TNFSF13, TNFSF12, TNFRSF4, STK38L, SPG20, RTEL1, RIPK3, POLG, PNMT, PIK3CG, PIAS1, PHRF1, PHLDB1, PGGT1B, MYH9, MERTK, MAPK14, MAPK13, MAP3K5, KIF1B, JAK2, IRF9, IMMP2L, GPR132, FAM69A, DIO2, CREB1, CD6, CASP9, C3orf18, ATXN2, ALS2, ADIPOQ, ADAM8, WDR7, TRIM39, TREX1, TRAM2, TNFRSF7, TDG, SMUG1, SKIV2L, PSRC1, PIM1, NAT1, LYRM7, LEMD2, KIAA1542, JUND, ITPR2, ITK, IDH1, GTF2H1, GRN, GPX1, GPSM3, FUS, FKBPL, FAM119B, ENG, DIAPH1, DHX16, CTSH, CREBL2, COL2A1, CD74, C1RL, ATM, ATF6B, ASXL1, ALOX5AP, ABLIM1, TNPO3, TNFSF8, TCF2, TBP, SOCS5, SMARCE1, SMAD4, SMAD1, SIGIRR, SH3TC1, RBM17, RAF1, PSMB1, PPM1L, PIK3CA, PDSS1, PCAF, NUDT1, NTRK1, NRP1, NR1H3, NOTCH1, NEUROD2, MRPS6, MPZ, LSP1, ITGB1, IGFBP7, ICOSLG, HSD11B2, HNF1B, HECA, FGR, FBXW7, DNAJB1, DCLRE1C, CXCR7, CCNY, CCL27, CALM1, BRCA1, BRAF, AS, ARRB1, AKR1B1, ADA, YPEL2, TNFRSF18, TFRC, TARDBP, STMN1, SRBD1, SQSTM1, SLC25A1, SLC10A7, SERPINE2, SDHD, SCNN1A, SBK1, S100A9, ROCK1, RHOA, RFC4, RB1, RAD52, PTPN12, PTPN1, PTP4A1, PTGER3, PRKD2, PPARD, POU2F1, PML, PLCB3, PLCB1, NTHL1, NPM1, NFKBIZ, NEIL1, MUTYH, MT2A, MGMT, MEIS1, MAPT, LRP1, KIAA0391, IL17RA, HSP90AA1, GRK4, GPX4, GDNF, FKBP4, FBXO38, FADS2, FADS1, DHCR7, DDX1, CHRNE, CD79B, CCND1, AKT2, ACADSB, ZBTB20, ZBTB16, XBP1, WNT2B, VGLL4, UNG, TUBAL3, TSC2, TRPM7, TOX2, TOPBP1, TMEM168, TLE4, TAL1, SULT1A1, STK24, STIP1, SNW1, SMYD2, SLAIN2, SIAE, SFTPC, SESTD1, S100A1, ROCK2, RGS16, RCBTB1, RBM19, RAC2, R3HDM1, PTPRE, PTGES3, PSMA6, PSEN1, PPP1R13L, PLXNA1, PLA2G3, PGRMC2, PDYN, PCNX, PAX2, OS9, OPTN, NUP98, NR1H2, NPC1, NPAS2, NCOA3, NCF4, MUC1, MTF1, MN1, MIST, MFNG, MAPK11, MAPK10, MAN1A2, LTC4S, LIG1, LDLR, KRI1, KIAA1274, KCNS3, KCNJ10, IRX3, IRS2, INPP4A, IL19, IL17C, GNB1, FBXO8, EPOR, ELF5, EIF4H, EFCAB6, DFFA, DCP1A, CYSLTR1, CXCL2, CSF2, CSDA, CRY2, CRABP2, CNPY2, CHRM1, CD5, CD3EAP, CCR4, CASP1, C1orf26, C11orf61, C11orf30, BCR, BCL2L2, BCL2A1, BAD, ARSA, APP, APOA5, AGBL4, ACSBG1, ACP5

Corresponding total number of TF hits:
(For each gene listed above, the total number of TF hits for any of the selected TFs, multiplied by the number of selected Phenopedia diseases containing that gene)
432, 416, 416, 392, 390, 372, 352, 338, 299, 290, 288, 275, 270, 264, 253, 252, 243, 240, 240, 234, 225, 224, 221, 216, 216, 208, 203, 200, 189, 189, 189, 186, 184, 180, 175, 171, 168, 162, 156, 155, 155, 154, 153, 150, 150, 147, 144, 144, 144, 143, 140, 140, 140, 140, 140, 140, 133, 132, 130, 126, 120, 120, 120, 120, 120, 120, 115, 115, 112, 108, 104, 104, 104, 104, 104, 102, 102, 100, 100, 100, 98, 96, 96, 95, 95, 92, 92, 91, 90, 90, 90, 88, 87, 84, 84, 80, 80, 80, 80, 80, 78, 78, 77, 77, 76, 75, 72, 72, 70, 70, 69, 66, 64, 63, 63, 63, 62, 62, 58, 57, 57, 56, 56, 56, 56, 56, 56, 54, 54, 54, 54, 54, 52, 52, 52, 50, 50, 49, 48, 48, 48,

45, 45, 45, 45, 44, 44, 44, 42, 42, 42, 42, 40, 40, 40, 40, 39, 36, 36, 36, 36, 36, 36, 35, 35, 34, 32, 32, 32, 32, 31, 31, 31, 30, 30, 30, 30, 30, 30, 30, 29, 28, 28, 28, 28, 28, 28, 28, 27, 27, 27, 27, 26, 26, 26, 26, 26, 26, 26, 25, 25, 25, 25, 25, 24, 24, 24, 24, 24, 24, 24, 24, 24, 24, 24, 23, 23, 23, 22, 22, 21, 21, 21, 20, 20, 20, 20, 20, 20, 20, 20, 18, 18, 18, 18, 18, 18, 18, 18, 18, 18, 17, 17, 17, 17, 17, 17, 16, 16, 16, 16, 16, 16, 16, 16, 16, 15, 15, 15, 15, 15, 15, 15, 15, 15, 14, 14, 14, 14, 14, 14, 14, 14, 14, 14, 13, 13, 13, 13, 13, 13, 12, 12, 12, 12, 12, 12, 12, 12, 12, 12, 12, 12, 12, 12, 12, 12, 12, 12, 12, 12, 12, 12, 11, 11, 11, 11, 11, 11, 11, 10, 10, 10, 10, 10, 10, 10, 10, 10, 10, 10, 10, 10, 10, 10, 10, 9, 9, 9, 9, 9, 9, 9, 9, 9, 9, 8, 8, 8, 8, 8, 8, 8, 8, 8, 8, 8, 8, 8, 8, 8, 8, 8, 8, 8, 8, 8, 8, 8, 8, 7, 7, 7, 7, 7, 7, 7, 7, 7, 7, 7, 7, 7, 7, 7, 7, 7, 7, 7, 7, 6, 6, 6, 6, 6, 6, 6, 6, 6, 6, 6, 6, 6, 6, 6, 6, 6, 6, 6, 6, 6, 6, 6, 6, 6, 5, 5, 5, 5, 5, 5, 5, 5, 5, 5, 5, 5, 5, 5, 5, 5, 5, 5, 5, 5, 5, 5, 5, 5, 5, 5, 5, 5, 5, 5, 5, 5, 5, 5, 5, 5, 4, 4, 4, 4, 4, 4, 4, 4, 4, 4, 4, 4, 4, 4, 4, 4, 4, 4, 4, 4, 4, 4, 4, 4, 4, 4, 4, 4, 4, 4, 4, 4, 4, 4, 4, 4, 4, 4, 3, 3, 3, 3, 3, 3, 3, 3, 3, 3, 3, 3, 3, 3, 3, 3, 3, 3, 3, 3, 3, 3, 3, 3, 3, 3, 3, 3, 3, 3, 3, 3, 3, 3, 3, 3, 3, 3, 3, 3, 3, 3, 3, 3, 3, 3, 2, 2, 2, 2, 2, 2, 2, 2, 2, 2, 2, 2, 2, 2, 2, 2, 2, 2, 2, 2, 2, 2, 2, 2, 2, 2, 2, 2, 2, 2, 2, 2, 2, 2, 2, 2, 2, 2, 2, 2, 2, 2, 2, 2, 2, 2, 2, 2, 2, 2, 2, 2, 2, 2, 2, 2, 1

Corresponding number of selected Phenopedia diseases each gene is involved with:
(In the same order as above)
16, 13, 16, 14, 13, 12, 11, 13, 13, 10, 9, 11, 15, 11, 11, 12, 9, 16, 8, 9, 15, 7, 13, 8, 8, 8, 7, 10, 9, 7, 9, 6, 8, 9, 7, 9, 12, 6, 6, 5, 5, 11, 9, 5, 5, 7, 12, 9, 12, 13, 7, 7, 7, 7, 5, 5, 7, 11, 5, 9, 4, 5, 5, 8, 10, 5, 5, 5, 7, 12, 8, 4, 4, 4, 4, 6, 6, 10, 4, 5, 7, 4, 4, 5, 5, 4, 4, 7, 6, 15, 5, 8, 3, 4, 4, 8, 4, 8, 10, 8, 6, 13, 11, 7, 4, 3, 6, 8, 7, 5, 3, 6, 8, 9, 7, 3, 2, 2, 2, 3, 3, 8, 4, 8, 7, 8, 14, 3, 2, 6, 3, 6, 13, 4, 13, 5, 5, 7, 4, 12, 12, 3, 5, 15, 15, 4, 4, 11, 14, 2, 2, 14, 8, 4, 8, 5, 3, 4, 12, 2, 3, 4, 4, 5, 5, 2, 4, 4, 1, 4, 1, 1, 1, 10, 1, 15, 1, 10, 5, 5, 1, 2, 7, 2, 7, 7, 1, 2, 1, 3, 3, 1, 1, 13, 1, 13, 13, 1, 1, 1, 5, 5, 1, 5, 8, 3, 12, 3, 1, 2, 12, 8, 12, 8, 8, 8, 1, 1, 1, 2, 1, 1, 1, 7, 5, 1, 2, 4, 2, 10, 1, 2, 9, 2, 6, 9, 1, 9, 6, 1, 6, 9, 1, 1, 1, 1, 1, 1, 4, 4, 2, 4, 8, 2, 4, 8, 1, 3, 3, 1, 3, 1, 3, 5, 5, 5, 7, 7, 1, 14, 7, 7, 7, 1, 2, 1, 1, 1, 13, 1, 13, 13, 1, 4, 2, 3, 2, 12, 1, 12, 6, 1, 4, 12, 12, 12, 12, 4, 3, 12, 1, 6, 3, 1, 12, 12, 1, 11, 11, 1, 11, 1, 11, 1, 1, 1, 5, 1, 10, 5, 5, 5, 10, 1, 5, 5, 10, 5, 5, 3, 3, 3, 9, 9, 9, 1, 9, 3, 9, 2, 1, 8, 8, 8, 1, 8, 2, 8, 2, 8, 8, 8, 8, 1, 8, 8, 1, 2, 8, 4, 1, 1, 1, 7, 7, 7, 7, 7, 7, 7, 7, 1, 7, 1, 7, 1, 1, 7, 7, 7, 7, 7, 7, 2, 3, 6, 2, 1, 6, 1, 3, 2, 6, 6, 1, 6, 2, 2, 6, 6, 1, 1, 6, 3, 6, 6, 3, 1, 5, 1, 5, 5, 5, 1, 5, 1, 1, 5, 5, 1, 5, 5, 1, 5, 1, 5, 1, 1, 1, 5, 5, 5, 1, 5, 5, 5, 1, 5, 5, 1, 5, 5, 5, 1, 1, 4, 4, 1, 1, 2, 2, 4, 4, 1, 4, 1, 4, 4, 1, 2, 2, 1, 2, 2, 4, 4, 1, 4, 2, 2, 1, 4, 4, 1, 4, 1, 2, 1, 4, 1, 1, 1, 3, 3, 3, 3, 3, 1, 3, 3, 1, 1, 3, 1, 3, 1, 1, 1, 1, 3, 3, 3, 1, 1, 3, 1, 1, 1, 1, 3, 3, 3, 3, 1, 1, 1, 1, 3, 1, 3, 1, 3, 3, 3, 1, 1, 3, 3, 1, 2, 2, 2, 2, 1, 1, 1, 1, 2, 1, 2, 1, 1, 1, 1, 2, 1, 2, 2, 2, 1, 2, 1, 1, 1, 1, 1, 2, 2, 1, 1, 2, 2, 1, 1, 1, 2, 2, 1, 1, 1, 1, 1, 1, 1, 1, 1, 1, 2, 1, 2, 1, 1, 1, 2, 1

Corresponding number of selected TFs each gene is involved with:
(In the same order as above)
27, 32, 26, 28, 30, 31, 32, 26, 23, 29, 32, 25, 18, 24, 23, 21, 27, 15, 30, 26, 15, 32, 17, 27, 27, 26, 29, 20, 21, 27, 21, 31, 23, 20, 25, 19, 14, 27, 26, 31, 31, 14, 17, 30, 30, 21, 12, 16, 12, 11, 20, 20, 20, 20, 28, 28, 19, 12, 26, 14, 30, 24, 24, 15, 12, 24, 23, 23, 16, 9, 13, 26, 26, 26, 26, 17, 17, 10, 25, 20, 14, 24, 24, 19, 19, 23, 23, 13, 15, 6, 18, 11, 29, 21, 21, 10, 20, 10, 8, 10, 13, 6, 7, 11, 19, 25, 12, 9, 10, 14, 23, 11, 8, 7, 9, 21, 31, 31, 29, 19, 19, 7, 14, 7, 8, 7, 4, 18, 27, 9, 18, 9, 4, 13, 4, 10, 10, 7, 12, 4, 4, 15, 9, 3, 3, 11, 11, 4, 3, 21, 21, 3, 5, 10, 5, 8, 13, 9, 3, 18, 12, 9, 9, 7, 7, 17, 8, 8, 32, 8, 31, 31, 31, 3, 30, 2, 30, 3, 6, 6, 29, 14, 4, 14, 4, 4, 28, 14, 27, 9, 9, 27, 26, 2, 26, 2, 2, 26, 26, 25, 5, 5, 25, 5, 3, 8, 2, 8, 24, 12, 2, 3, 2, 3, 3, 3, 23, 23, 23, 11, 22, 21, 21, 3, 4, 20, 10, 5, 10, 2, 20, 10, 2, 9, 3, 2, 18, 2, 3, 18, 3, 2, 17, 17, 17, 17, 17, 17, 4, 4, 8, 4, 2, 8, 4, 2, 16, 5, 5, 15, 5, 15, 5, 3, 3, 3, 2, 2, 14, 1, 2, 2, 2, 14, 7, 14, 14, 13, 1, 13, 1, 1, 13, 3, 6, 4, 6, 1, 12, 1, 2, 12, 3, 1, 1, 1, 1, 3, 4, 1, 12, 2, 4, 12, 1, 1, 11, 1, 1, 11, 1, 11, 1, 10, 10, 10, 2, 10, 1, 2, 2, 2, 1, 10, 2, 2, 1, 2, 2, 3, 3, 3, 1, 1, 1, 9, 1, 3, 1, 4, 8, 1, 1, 1, 8, 1, 4, 1, 4, 1, 1, 1, 1, 8, 1, 1, 8, 4, 1, 2, 8, 8, 8, 1, 1, 1, 1, 1, 1, 1, 1, 7, 1, 7, 1, 7, 7, 1, 1, 1, 1, 1, 1, 3, 2, 1, 3, 6, 1, 6, 2, 3, 1, 1, 6, 1, 3, 3, 1, 1, 6, 6, 1, 2, 1, 1, 2, 6, 1, 5, 1, 1, 1, 5, 1, 5, 5, 1, 1, 5, 1, 1, 5, 1, 5, 1, 5, 5, 5, 1, 1, 1, 5, 1, 1, 1, 5, 1, 1, 5, 1, 1, 1, 5, 4, 1, 1, 4, 4, 2, 2, 1, 1, 4, 1, 4, 1, 1, 4, 2, 2, 4, 2, 2, 1, 1, 4, 1, 2, 2, 4, 1, 1, 4, 1, 4, 2, 4, 1, 4, 4, 4, 1, 1, 1, 1, 1, 3, 1, 1, 3, 3, 1, 3, 1, 3, 3, 3, 3, 1, 1, 1, 3, 3, 1, 3, 3, 3, 3, 1, 1, 1, 1, 3, 3, 3, 3, 1, 3, 1, 3, 1, 1, 1, 3, 3, 1, 1, 2, 1, 1, 1, 1, 2, 2, 2, 2, 1, 2, 1, 2, 2, 2, 2, 1, 2, 1, 1, 1, 2, 1, 2, 2, 2, 2, 2, 1, 1, 2, 2, 1, 1, 2, 2, 2, 1, 1, 2, 2, 2, 2, 2, 2, 2, 2, 2,

2, 1, 2, 1, 2, 2, 2, 1

Phenopedia diseases ranked according to mean of effect sizes in cluster:
Connective Tissue Diseases | Lupus Erythematosus, Systemic | Autoimmune Diseases | Diabetes Mellitus, Type 1 | Arthritis, Rheumatoid | Joint Diseases | Arthritis | Immune System Diseases | Arthritis, Juvenile Rheumatoid | Rheumatic Diseases | HIV Seropositivity | Celiac Disease | Acquired Immunodeficiency Syndrome | Demyelinating Diseases | Multiple Sclerosis | Sclerosis

Corresponding mean of effect sizes of each Phenopedia disease:
(In the same order as above)
2.52, 2.39, 2.22, 1.99, 1.93, 1.78, 1.62, 1.61, 1.55, 1.55, 1.52, 1.51, 1.44, 1.10, 0.83, 0.51

Corresponding total number of genes of each Phenopedia disease:
(In the same order as above)
732, 416, 1.1K, 404, 425, 511, 695, 1.8K, 91, 531, 53, 380, 88, 373, 483, 634

Corresponding number of genes of each Phenopedia disease with at least one TF hit (of selected TFs):
(In the same order as above)
256, 167, 365, 143, 149, 162, 217, 554, 39, 165, 19, 123, 30, 127, 165, 199

Corresponding total number of TF hits (of selected TFs) for each Phenopedia disease (in all genes):
(In the same order as above)
2434, 1684, 3128, 1305, 1380, 1459, 1906, 4407, 449, 1435, 197, 1015, 290, 932, 1218, 1381

Corresponding number of selected TFs each Phenopedia disease is involved with:
(In the same order as above)
32, 32, 32, 32, 32, 32, 32, 32, 32, 32, 32, 32, 32, 32, 32, 32

TFs ranked according to mean of effect sizes in cluster:
V$STAT5A_03, V$STAT5A_04, V$STAT6_01, V$LEF1_Q2, V$STAT4_01, V$STAT1_03, V$CEBP_Q3, V$SOX10_Q6, V$PAX2_02, V$HMGIY_Q6, V$TCF4_Q5, V$HOXA4_Q2, V$NFAT_Q4_01, V$AREB6_04, V$CEBP_Q2_01, V$GATA6_01, V$CEBPA_01, V$GATA_Q6, V$CEBPB_01, V$NKX25_Q5, V$GFI1_Q6, V$TST1_01, V$CEBP_Q2, V$ELF1_Q6, VDBP_Q6, VNFAT_Q6, V$BRCA_01, V$FOXP3_Q4, BCRP_C, VHSF2_01, V$HSF1_01, V$CEBPB_02

Corresponding mean of effect sizes of each TF:
(In the same order as above)
2.51, 2.49, 2.32, 2.26, 2.26, 2.24, 2.17, 2.12, 2.10, 2.10, 2.07, 1.99, 1.98, 1.78, 1.77, 1.69, 1.65, 1.55, 1.48, 1.41, 1.34, 1.33, 1.30, 1.28, 1.27, 1.24, 1.10, 0.91, 0.89, 0.73, 0.58, 0.27

Corresponding total number of TF hits for each TF (genome-wide):
(In the same order as above)
527, 509, 520, 556, 523, 502, 500, 511, 529, 564, 531, 526, 536, 480, 533, 512, 528, 520, 500, 455, 460, 548, 537, 542, 519, 540, 474, 485, 497, 442, 469, 486

Corresponding total number of TF hits for each TF (in all genes in selected Phenopedia diseases):
(In the same order as above)
873, 840, 846, 856, 859, 849, 917, 814, 834, 855, 771, 803, 898, 740, 809, 787, 832, 739, 773, 621, 674, 762, 721, 733, 741, 862, 674, 637, 670, 543, 665, 622

Corresponding number of genes (of selected Phenopedia diseases) each TF is involved with:
(In the same order as above)
179, 166, 172, 173, 172, 169, 184, 160, 164, 166, 156, 164, 178, 150, 165, 155, 167, 151, 160, 138, 136,

159, 155, 167, 157, 177, 147, 141, 145, 123, 142, 148

Corresponding number of selected Phenopedia diseases each TF is involved with:
(In the same order as above)
16, 16, 16, 16, 16, 16, 16, 16, 16, 16, 16, 16, 16, 16, 16, 16, 16, 16, 16, 16, 16, 16, 16, 16, 16, 16, 16, 16, 16, 16

Disease groups of selected Phenopedia diseases ranked according to sum of effect sizes
Immune System Diseases | Autoimmune Diseases | Signs and Symptoms | Connective Tissue Diseases | Musculoskeletal Diseases | Metabolic Diseases | Arthritis, Rheumatoid | Virus Diseases | Retroviridae Infections | Lentivirus Infections | HIV Infections | Communicable Diseases | Thoracic Diseases | Respiratory Tract Diseases | Myocarditis | Mediastinitis | Mediastinal Diseases | Lupus Erythematosus, Systemic | Endocarditis | Collagen Diseases | Cardiovascular Diseases | Cardiomyopathies | Glucose Metabolism Disorders | Diabetes Mellitus, Type 1 | Diabetes Mellitus | Nervous System Diseases | Demyelinating Diseases | Joint Diseases | Arthritis | Arthritis, Juvenile Rheumatoid | Rheumatic Diseases | HIV Seropositivity | Vitamin D Deficiency | Rickets | Protein-Energy Malnutrition | Nutrition Disorders | Malnutrition | Malabsorption Syndromes | Intestinal Diseases | Gastrointestinal Diseases | Digestive System Diseases | Deficiency Diseases | Celiac Disease | Calcium Metabolism Disorders | Bone Diseases, Metabolic | Bone Diseases | Avitaminosis | Acquired Immunodeficiency Syndrome | Multiple Sclerosis | Demyelinating Autoimmune Diseases, CNS | Autoimmune Diseases of the Nervous System | Sclerosis

Corresponding sum of effect sizes for each disease group of selected Phenopedia diseases
(In the same order as above)
260.50, 209.04, 207.19, 157.31, 149.75, 112.21, 111.35, 94.98, 94.98, 94.98, 94.98, 94.98, 76.53, 76.53, 76.53, 76.53, 76.53, 76.53, 76.53, 76.53, 76.53, 76.53, 63.73, 63.73, 63.73, 61.82, 61.82, 56.91, 51.74, 49.62, 49.53, 48.76, 48.48, 48.48, 48.48, 48.48, 48.48, 48.48, 48.48, 48.48, 48.48, 48.48, 48.48, 48.48, 48.48, 48.48, 48.48, 46.22, 26.52, 26.52, 26.52, 16.32

Cluster for columns 21 to 26, rows 110 to 125

Phenopedia diseases

Cerebral Arterial Diseases | Intracranial Arterial Diseases | Neuroblastoma | Neuroectodermal Tumors, Primitive | Amyloidosis | Cerebral Amyloid Angiopathy

TFs

V$PR_Q2 | V$GR_Q6_01 | V$CEBP_01 | V$AP3_Q6 | V$PU1_Q6 | V$PEA3_Q6 | V$ETS_Q6 | V$TEF1_Q6 | V$STAT3_02 | V$STAT1_02 | V$GATA2_01 | V$TFIII_Q6 | V$MZF1_01 | V$STAT6_02 | V$GATA3_01 | V$GATA4_Q3

Information

All related TFs:
(List of all TFs that are related to any of the PWMs)
AP-3, C/EBP, C/EBPalpha, ELF-1, ELFR, Elf-1, Elk-1, Elk-1-isoform1, Erg-1, Erg-2, Ets-1, Fli-1, GABP-alpha, GABP-alpha:GABP-beta, GABP-beta1, GABP-beta2, GATA-2, GATA-3, GATA-4, GR, GR-alpha, GR-beta, MZF1B-C, NERF-1a, NERF-1b, NERF-2, Net, PEA3, PR, PR-alpha, PR-beta, PU.1, PU.1-xbb1, SAP-1a, SAP-1b, STAT1, STAT1alpha, STAT3, STAT6, Spi-B, TCF, TEF-1, TEL1, TFII-I, Tel-2a, Tel-2b, Tel-2c, Tel-2d, Tel-2e, Tel-2f, c-Ets-1, c-Ets-1A, c-Ets-1B, c-Ets-2, c-Ets-2A, c-Ets-2B, p38erg, p49erg, p55, p55erg

Ranked gene list:

(All genes of the selected Phenopedia diseases with hits of any of the selected TFs, ranked according to the total number of TF hits)
PTPN6, EFNA3, COL11A2, C12orf57, AGER, CAMK2G, VEGFA, CCND3, PTMS, BRD2, TNF, TNFRSF6B, ENO2, PITX3, PHF1, AQP10, TAPBPL, LEPREL2, VEGF, C6orf129, TGFB1, LTB, LTA, FRS3, ATXN1, LTBP4, COL6A1, TNFRSF1A, SIT1, LRP1, CNTFR, ADM, NOTCH4, MLL, NR3C1, CTSD, NOS3, TAF8, SIGMAR1, C1orf66, AIF1, LIF, LCAT, CALHM1, MMP9, NTRK1, NLRP3, IL10, IGF1R, HPN, CYBA, APOA5, ALOX5AP

Corresponding total number of TF hits:
(For each gene listed above, the total number of TF hits for any of the selected TFs, multiplied by the number of selected Phenopedia diseases containing that gene)
96, 96, 96, 96, 96, 90, 72, 72, 66, 66, 64, 60, 60, 54, 54, 54, 48, 48, 36, 36, 32, 32, 32, 30, 26, 20, 18, 15, 12, 12, 12, 12, 11, 11, 10, 9, 8, 6, 6, 6, 6, 4, 4, 4, 3, 2, 2, 2, 2, 2, 2, 2, 2

Corresponding number of selected Phenopedia diseases each gene is involved with:
(In the same order as above)
6, 6, 6, 6, 6, 6, 6, 6, 6, 6, 4, 6, 6, 6, 6, 6, 6, 6, 3, 6, 4, 2, 2, 6, 2, 2, 6, 1, 6, 4, 6, 2, 1, 1, 2, 1, 2, 6, 6, 6, 6, 2, 2, 2, 3, 2, 1, 2, 2, 2, 2, 2, 2

Corresponding number of selected TFs each gene is involved with:
(In the same order as above)
16, 16, 16, 16, 16, 15, 12, 12, 11, 11, 16, 10, 10, 9, 9, 9, 8, 8, 12, 6, 8, 16, 16, 5, 13, 10, 3, 15, 2, 3, 2, 6, 11, 11, 5, 9, 4, 1, 1, 1, 1, 2, 2, 2, 1, 1, 2, 1, 1, 1, 1, 1, 1

Phenopedia diseases ranked according to mean of effect sizes in cluster:
Amyloidosis | Cerebral Amyloid Angiopathy | Neuroblastoma | Neuroectodermal Tumors, Primitive | Intracranial Arterial Diseases | Cerebral Arterial Diseases

Corresponding mean of effect sizes of each Phenopedia disease:
(In the same order as above)
2.68, 2.54, 2.50, 2.22, 1.96, 1.95

Corresponding total number of genes of each Phenopedia disease:
(In the same order as above)
116, 95, 115, 103, 198, 197

Corresponding number of genes of each Phenopedia disease with at least one TF hit (of selected TFs):
(In the same order as above)
31, 31, 30, 27, 47, 47

Corresponding total number of TF hits (of selected TFs) for each Phenopedia disease (in all genes):
(In the same order as above)
270, 259, 265, 227, 348, 348

Corresponding number of selected TFs each Phenopedia disease is involved with:
(In the same order as above)
16, 16, 16, 16, 16, 16

TFs ranked according to mean of effect sizes in cluster:
V$MZF1_01, V$TFIII_Q6, V$AP3_Q6, V$STAT1_02, V$PU1_Q6, V$GATA3_01, V$STAT3_02, V$GATA2_01, V$TEF1_Q6, V$STAT6_02, VPR_Q2, VGATA4_Q3, V$CEBP_01, V$GR_Q6_01, VETS_Q6, VPEA3_Q6

Corresponding mean of effect sizes of each TF:
(In the same order as above)

3.64, 3.41, 2.90, 2.81, 2.78, 2.55, 2.51, 2.50, 2.44, 2.11, 2.09, 2.04, 1.67, 1.45, 1.25, 0.76

Corresponding total number of TF hits for each TF (genome-wide):
(In the same order as above)
435, 458, 479, 422, 460, 457, 434, 423, 463, 468, 457, 525, 508, 484, 466, 438

Corresponding total number of TF hits for each TF (in all genes in selected Phenopedia diseases):
(In the same order as above)
127, 125, 122, 110, 117, 114, 110, 100, 113, 112, 104, 112, 102, 98, 82, 69

Corresponding number of genes (of selected Phenopedia diseases) each TF is involved with:
(In the same order as above)
30, 29, 27, 23, 26, 28, 26, 23, 26, 27, 24, 26, 23, 23, 19, 17

Corresponding number of selected Phenopedia diseases each TF is involved with:
(In the same order as above)
6, 6, 6, 6, 6, 6, 6, 6, 6, 6, 6, 6, 6, 6, 6, 6

Disease groups of selected Phenopedia diseases ranked according to sum of effect sizes
Vascular Diseases | Nervous System Diseases | Cerebrovascular Disorders | Central Nervous System Diseases | Cardiovascular Diseases | Brain Diseases | Intracranial Arterial Diseases | Signs and Symptoms | Metabolic Diseases | Amyloidosis | Cerebral Amyloid Angiopathy | Neuroendocrine Tumors | Neuroblastoma | Neoplasms | Neuroectodermal Tumors, Primitive | Neoplasms, Neuroepithelial | Glioma | Cerebral Arterial Diseases

Corresponding sum of effect sizes for each disease group of selected Phenopedia diseases
(In the same order as above)
103.13, 103.13, 103.13, 103.13, 103.13, 103.13, 62.45, 42.82, 42.82, 42.82, 40.68, 40.06, 40.06, 40.06, 35.48, 35.48, 35.48, 31.14

Cluster for columns 159 to 172, rows 443 to 443

Phenopedia diseases

Hyperlipidemia, Familial Combined | Lipid Metabolism, Inborn Errors | Metabolism, Inborn Errors | Hyperlipoproteinemia Type II | Hyperlipoproteinemias | Hypertriglyceridemia | Lipid Metabolism Disorders | Dyslipidemias | Hyperlipidemias | Diseases in Twins | Cholecystolithiasis | Cholelithiasis | Gallbladder Diseases | Gallstones

TFs

V$PPARG_03

Information

All related TFs:
(List of all TFs that are related to any of the PWMs)
PPAR-gamma, PPAR-gamma1, PPAR-gamma2

Ranked gene list:
(All genes of the selected Phenopedia diseases with hits of any of the selected TFs, ranked according to the total number of TF hits)
APOC3, APOA4, APOA1, NR1H3, TNF, NOS3, LCAT, UGT1A1, AGTR1, ADD1, PPARA, NPC1L1,

CRABP2, APOC1, ADRA1A, IL1RN, PPARGC1A, F5, TXNIP, SLC10A2, OGG1, CAPN10, ABCB4, VEGFA, TPP1, LTA, UCP2, FADS3, CAT, ANGPTL4, ACADS, VEGF, UGT1A7, UGT1A6, UGT1A3, SILV, PNPLA2, PLXNA2, HADHA, GALT, GALK1, ETFDH, ETFB, ETFA, CYP11B1, CFB, ALDH2

Corresponding total number of TF hits:
(For each gene listed above, the total number of TF hits for any of the selected TFs, multiplied by the number of selected Phenopedia diseases containing that gene)
14, 14, 12, 10, 9, 9, 9, 8, 8, 8, 7, 7, 7, 7, 7, 6, 5, 5, 4, 4, 4, 4, 4, 3, 3, 3, 2, 2, 2, 2, 2, 1, 1, 1, 1, 1, 1, 1, 1, 1, 1, 1, 1, 1, 1, 1, 1

Corresponding number of selected Phenopedia diseases each gene is involved with:
(In the same order as above)
14, 14, 12, 10, 9, 9, 9, 8, 8, 8, 7, 7, 7, 7, 7, 6, 5, 5, 4, 4, 4, 4, 4, 3, 3, 3, 2, 2, 2, 2, 2, 1, 1, 1, 1, 1, 1, 1, 1, 1, 1, 1, 1, 1, 1, 1, 1

Corresponding number of selected TFs each gene is involved with:
(In the same order as above)
1, 1, 1, 1, 1, 1, 1, 1, 1, 1, 1, 1, 1, 1, 1, 1, 1, 1, 1, 1, 1, 1, 1, 1, 1, 1, 1, 1, 1, 1, 1, 1, 1, 1, 1, 1, 1, 1, 1, 1, 1, 1, 1, 1, 1

Phenopedia diseases ranked according to mean of effect sizes in cluster:
Cholecystolithiasis | Cholelithiasis | Gallbladder Diseases | Lipid Metabolism, Inborn Errors | Hyperlipidemia, Familial Combined | Gallstones | Hyperlipoproteinemia Type II | Lipid Metabolism Disorders | Dyslipidemias | Hyperlipoproteinemias | Hyperlipidemias | Metabolism, Inborn Errors | Hypertriglyceridemia | Diseases in Twins

Corresponding mean of effect sizes of each Phenopedia disease:
(In the same order as above)
4.76, 4.55, 4.31, 4.21, 4.19, 4.12, 4.11, 4.09, 3.79, 3.47, 3.20, 2.93, 2.40, 1.77

Corresponding total number of genes of each Phenopedia disease:
(In the same order as above)
47, 49, 58, 104, 23, 80, 54, 264, 231, 67, 204, 289, 104, 73

Corresponding number of genes of each Phenopedia disease with at least one TF hit (of selected TFs):
(In the same order as above)
9, 9, 11, 16, 6, 13, 9, 29, 24, 9, 21, 30, 11, 10

Corresponding total number of TF hits (of selected TFs) for each Phenopedia disease (in all genes):
(In the same order as above)
9, 9, 11, 16, 6, 13, 9, 29, 24, 9, 21, 30, 11, 10

Corresponding number of selected TFs each Phenopedia disease is involved with:
(In the same order as above)
1, 1, 1, 1, 1, 1, 1, 1, 1, 1, 1, 1, 1, 1

TFs ranked according to mean of effect sizes in cluster:
V$PPARG_03

Corresponding mean of effect sizes of each TF:
(In the same order as above)
3.71

Corresponding total number of TF hits for each TF (genome-wide):
(In the same order as above)

473

Corresponding total number of TF hits for each TF (in all genes in selected Phenopedia diseases):
(In the same order as above)
207

Corresponding number of genes (of selected Phenopedia diseases) each TF is involved with:
(In the same order as above)
47

Corresponding number of selected Phenopedia diseases each TF is involved with:
(In the same order as above)
14

Disease groups of selected Phenopedia diseases ranked according to sum of effect sizes
Signs and Symptoms | Metabolic Diseases | Lipid Metabolism Disorders | Dyslipidemias | Digestive System Diseases | Biliary Tract Diseases | Hyperlipidemias | Pathological Conditions, Anatomical | Gallstones | Calculi | Metabolism, Inborn Errors | Genetic Diseases, Inborn | Congenital, Hereditary, and Neonatal Diseases and Abnormalities | Gallbladder Diseases | Lipid Metabolism, Inborn Errors | Hyperlipoproteinemias | Cholecystolithiasis | Cholelithiasis | Hyperlipidemia, Familial Combined | Hyperlipoproteinemia Type II | Hypertriglyceridemia | Diseases in Twins | Disease Attributes

Corresponding sum of effect sizes for each disease group of selected Phenopedia diseases
(In the same order as above)
32.38, 32.38, 29.45, 21.16, 17.74, 17.74, 17.37, 13.43, 13.43, 13.43, 11.24, 11.24, 11.24, 9.07, 8.32, 7.58, 4.76, 4.55, 4.19, 4.11, 2.40, 1.77, 1.77

Cluster for columns 917 to 986, rows 444 to 446

Phenopedia diseases

Anxiety Disorders | Panic Disorder | Eating Disorders | Obsessive-Compulsive Disorder | Cocaine-Related Disorders | Autistic Disorder | Child Development Disorders, Pervasive | Attention | Attention Deficit Disorder with Hyperactivity | Psychiatric Status Rating Scales | Schizophrenia | Affective Disorders, Psychotic | Bipolar Disorder | Mood Disorders | Dystonia | Dystonic Disorders | Mental Status Schedule | Essential Tremor | Tremor | Epilepsies, Partial | Epilepsy, Generalized | Epilepsy | Seizures | Brief Psychiatric Rating Scale | Schizophrenia, Paranoid | Alcoholism | Conduct Disorder | Appetite | Hyperphagia | Thinness | Substance Withdrawal Syndrome | Delirium | Alcohol-Induced Disorders, Nervous System | Alcohol Withdrawal Delirium | Tobacco Use Disorder | Alcohol-Related Disorders | Substance-Related Disorders | Dominance, Cerebral | Functional Laterality | Movement Disorders | Basal Ganglia Diseases | Parkinson Disease | Parkinsonian Disorders | Learning | Memory | Neurotic Disorders | Genetics, Behavioral | Reinforcement (C0035007) | Pain Threshold | Borderline Personality Disorder | Personality Assessment | Tourette Syndrome | Tic Disorders | Tics | Postmortem Changes | Psychopathology | Heroin Dependence | Opioid-Related Disorders | Antisocial Personality Disorder | Personality Disorders | Bulimia | Bulimia Nervosa | Stress Disorders, Traumatic | Reaction Time | Cues | Memory, Short-Term | Mental Health | Thinking | Hallucinations | Perceptual Disorders

TFs

V$NRSF_Q4 | V$CAP_01 | V$MINI19_B

Information

All related TFs:
(List of all TFs that are related to any of the PWMs)
REST, REST-form1, REST-form2

Ranked gene list:
(All genes of the selected Phenopedia diseases with hits of any of the selected TFs, ranked according to the total number of TF hits)
DRD2, BDNF, DRD4, DRD3, OPRM1, HTR1A, GABRB3, GABRG2, SNAP25, NOS3, CHRNB2, HTR5A, GRIN2A, GAD1, GHSR, GRIK2, GRIN1, GAD2, IL10, GRIK4, CRH, ADA, ABCB1, SLC6A5, PENK, GRM4, NRXN1, OPRL1, LBXCOR1, KCNJ6, NRXN3, CHAT, NEFM, GRIA2, TRH, SST, SLC17A6, NEF3, L1CAM, GLRA1, TOR1A, NQO1, HCRT, GRM8, GABRD, DDC, CCKBR, HTR6, CARTPT, SYN1, NPTX2, NPPB, HTR7, UCN, PICK1, NTRK3, NGF, MAPT, CTSD, ADORA2A, VGF, TSC2, SULT4A1, PTS, MC1R, GRID1, CTNNA2, ACTA1, NPPA, ACHE, PER1, SLC6A12, PDE9A, NPR2, NF1, KCNH2, GPR78, GABBR2, DLX1, CALB1, CACNA1B, ADORA2B, VIP, NTNG1, IGF2, GRIN2C, DRG1, AGRP, XKR4, SLC12A6, PTGS2, NR2E1, NPAS3, MEIS1, INS, HCN2, GLRB, DSCAM, DFNB31, CYP2A6, RNF41, GNAS, GLRA3, CYP11B2, NEUROD1, LMX1B, FEZ1, TAAR6, SYP, SNAP91, SCN2A1, SCN2A, RTN4R, RGS7, NEFH, MTNR1B, LINGO1, JPH3, HRH3, GNB1L, DPP6, DLGAP3, CNTNAP1, CNTN2, CHGB, CAMK1, CACNG3, ATP2B2, ARRB1, ADM, ADCY1, WWC1, UGDH, RYR3, NGFB, NEFL, DGKH, BHLHE41, UBQLN1, TAPBP, SYNJ1, SLC5A4, SHC3, SCN2A2, PTPRT, PPP3CA, POU3F2, PIP4K2A, OPN4, OMG, NOTCH3, MAP3K6, LRRK1, IGF1R, GPR125, GABARAP, EPHB2, CYP11B1, APOC3, APOA1, AHI1, ADD1, XRCC3, TNXB, TNR, SYT4, SOD1, SLC26A6, PRSS12, PRLHR, NOTCH4, MYL5, MTAP, MOBKL2B, LAMP3, ITPR2, INSIG1, HTRA1, GALP, FEV, EGR4, DLGAP2, CXCL12, CPLX2, CMTM8, CHGA, CDKN2B, CAMTA1, BHLHE40, BHLHB3, BHLHB2, ARSA, APBB1, ACSL6, TRAF3IP1, SLC25A13, RUNX1, QKI, PCDHB11, OLIG3, MAP1B, LASP1, HIP1, HDAC10, GRM1, GADD45A, G6PD, F7, EN1, DYRK1A, DAXX, CYP21A2, CELSR1, CDK5R1, CDK5, BMP6, ATOX1, ARVCF, AP3B2, ALDOA, ACADS, ABCA7, TBX21, STAB1, SPRY4, SEMA3B, SCZD5, RPL34, RHCG, RHAG, RGS10, PTPRR, PSAP, PKD1, PDIA2, OPTC, NFATC2, NAP1L4, MYO7A, MDM2, LMNA, JUN, GMIP, FOXC2, FOSL2, DKK2, CAMKV, C4orf31, BRD1, AURKC, ARHGDIG, AP3M1, ALOX5

Corresponding total number of TF hits:
(For each gene listed above, the total number of TF hits for any of the selected TFs, multiplied by the number of selected Phenopedia diseases containing that gene)
189, 168, 159, 153, 84, 80, 78, 72, 66, 66, 63, 51, 51, 51, 48, 45, 42, 38, 36, 33, 33, 33, 32, 30, 30, 30, 28, 27, 27, 27, 26, 26, 24, 24, 21, 21, 21, 21, 21, 21, 20, 20, 20, 20, 20, 20, 19, 19, 18, 18, 18, 18, 16, 16, 16, 16, 16, 16, 16, 15, 15, 15, 15, 15, 15, 15, 15, 14, 14, 13, 12, 12, 12, 12, 12, 12, 12, 12, 12, 12, 12, 10, 10, 10, 10, 10, 10, 9, 9, 9, 9, 9, 9, 9, 9, 9, 9, 9, 9, 8, 8, 8, 8, 7, 7, 7, 6, 6, 6, 6, 6, 6, 6, 6, 6, 6, 6, 6, 6, 6, 6, 6, 6, 6, 6, 6, 6, 6, 6, 5, 5, 5, 5, 5, 5, 5, 4, 4, 4, 4, 4, 4, 4, 4, 4, 4, 4, 4, 4, 4, 4, 4, 4, 4, 4, 4, 4, 4, 4, 4, 3, 3, 3, 3, 3, 3, 3, 3, 3, 3, 3, 3, 3, 3, 3, 3, 3, 3, 3, 3, 3, 3, 3, 3, 3, 3, 3, 3, 3, 3, 3, 2, 2, 2, 2, 2, 2, 2, 2, 2, 2, 2, 2, 2, 2, 2, 2, 2, 2, 2, 2, 2, 2, 2, 2, 2, 2, 2, 1, 1, 1, 1, 1, 1, 1, 1, 1, 1, 1, 1, 1, 1, 1, 1, 1, 1, 1, 1, 1, 1, 1, 1, 1, 1, 1, 1, 1, 1

Corresponding number of selected Phenopedia diseases each gene is involved with:
(In the same order as above)
63, 56, 53, 51, 42, 40, 26, 24, 22, 22, 21, 17, 17, 17, 16, 15, 14, 19, 18, 11, 11, 11, 32, 10, 10, 10, 14, 9, 9, 9, 13, 13, 8, 8, 7, 7, 7, 7, 7, 7, 10, 20, 10, 20, 10, 20, 20, 19, 19, 6, 6, 6, 18, 8, 8, 16, 16, 16, 8, 16, 5, 15, 5, 5, 5, 5, 5, 7, 7, 13, 6, 6, 6, 6, 4, 4, 12, 6, 4, 4, 6, 10, 5, 10, 5, 5, 10, 3, 9, 9, 9, 9, 9, 9, 3, 9, 9, 3, 9, 4, 4, 8, 4, 7, 7, 7, 6, 2, 6, 3, 3, 6, 2, 2, 2, 2, 6, 2, 2, 3, 3, 2, 6, 6, 2, 6, 3, 2, 2, 2, 6, 5, 5, 5, 5, 5, 5, 5, 4, 2, 4, 4, 2, 2, 4, 4, 4, 4, 4, 2, 4, 2, 4, 2, 4, 2, 4, 2, 2, 2, 4, 4, 3, 1, 3, 1, 3, 1, 1, 1, 1, 3, 1, 1, 3, 3, 3, 3, 3, 1, 1, 1, 3, 3, 1, 3, 1, 1, 3, 3, 3, 3, 3, 3, 1, 2, 2, 1, 2, 2, 1, 2, 2, 2, 2, 2, 2, 1, 2, 2, 1, 1, 2, 1, 1, 1, 2, 2, 2, 1, 2, 2, 1, 1, 1, 1, 1, 1, 1, 1, 1, 1, 1, 1, 1, 1, 1, 1, 1, 1, 1, 1, 1, 1, 1, 1, 1, 1, 1, 1, 1

Corresponding number of selected TFs each gene is involved with:
(In the same order as above)

3, 3, 3, 3, 2, 2, 3, 3, 3, 3, 3, 3, 3, 3, 3, 3, 3, 2, 2, 3, 3, 3, 1, 3, 3, 3, 2, 3, 3, 3, 2, 2, 3, 3, 3, 3, 3, 3, 3, 3, 2, 1, 2, 1, 2, 1, 1, 1, 1, 3, 3, 3, 1, 2, 2, 1, 1, 1, 2, 1, 3, 1, 3, 3, 3, 3, 3, 3, 2, 2, 1, 2, 2, 2, 2, 3, 3, 1, 2, 3, 3, 2, 1, 2, 1, 2, 2, 1, 3, 1, 1, 1, 1, 1, 1, 3, 1, 1, 3, 1, 2, 2, 1, 2, 1, 1, 1, 1, 3, 1, 2, 2, 1, 3, 3, 3, 1, 3, 3, 2, 2, 3, 1, 1, 3, 1, 2, 3, 3, 3, 1, 1, 1, 1, 1, 1, 1, 1, 1, 2, 1, 1, 2, 2, 1, 1, 1, 1, 1, 2, 1, 2, 1, 2, 1, 2, 1, 2, 2, 2, 1, 1, 1, 3, 1, 3, 1, 3, 3, 3, 1, 3, 3, 1, 1, 1, 1, 1, 3, 3, 3, 1, 1, 3, 1, 3, 3, 1, 1, 1, 1, 1, 1, 3, 1, 1, 2, 1, 1, 2, 1, 1, 1, 1, 1, 1, 2, 1, 1, 2, 2, 1, 2, 2, 2, 1, 1, 1, 2, 1, 1, 2, 1, 1, 1, 1, 1, 1, 1, 1, 1, 1, 1, 1, 1, 1, 1, 1, 1, 1, 1, 1, 1, 1, 1, 1, 1, 1, 1, 1, 1

Phenopedia diseases ranked according to mean of effect sizes in cluster:
Obsessive-Compulsive Disorder | Panic Disorder | Anxiety Disorders | Eating Disorders | Epilepsy, Generalized | Pain Threshold | Reinforcement (C0035007) | Neurotic Disorders | Psychopathology | Dystonia | Genetics, Behavioral | Reaction Time | Perceptual Disorders | Seizures | Hallucinations | Cues | Mental Health | Bipolar Disorder | Borderline Personality Disorder | Dystonic Disorders | Mood Disorders | Substance Withdrawal Syndrome | Heroin Dependence | Bulimia Nervosa | Tics | Conduct Disorder | Tic Disorders | Tobacco Use Disorder | Schizophrenia | Alcohol-Related Disorders | Opioid-Related Disorders | Substance-Related Disorders | Alcoholism | Tourette Syndrome | Personality Assessment | Epilepsy | Alcohol-Induced Disorders, Nervous System | Delirium | Bulimia | Antisocial Personality Disorder | Stress Disorders, Traumatic | Psychiatric Status Rating Scales | Hyperphagia | Appetite | Brief Psychiatric Rating Scale | Movement Disorders | Thinking | Affective Disorders, Psychotic | Personality Disorders | Memory, Short-Term | Attention | Attention Deficit Disorder with Hyperactivity | Schizophrenia, Paranoid | Child Development Disorders, Pervasive | Autistic Disorder | Memory | Alcohol Withdrawal Delirium | Learning | Dominance, Cerebral | Functional Laterality | Basal Ganglia Diseases | Epilepsies, Partial | Postmortem Changes | Parkinson Disease | Cocaine-Related Disorders | Parkinsonian Disorders | Essential Tremor | Thinness | Tremor | Mental Status Schedule

Corresponding mean of effect sizes of each Phenopedia disease:
(In the same order as above)
9.33, 8.98, 8.73, 8.70, 6.75, 6.60, 6.01, 5.94, 5.89, 5.86, 5.64, 5.55, 5.40, 5.29, 5.22, 5.18, 5.12, 5.11, 4.93, 4.83, 4.72, 4.66, 4.52, 4.46, 4.41, 4.40, 4.39, 4.36, 4.23, 4.22, 4.09, 3.95, 3.95, 3.95, 3.92, 3.65, 3.51, 3.51, 3.51, 3.49, 3.37, 3.27, 3.25, 3.23, 3.05, 3.01, 2.98, 2.90, 2.84, 2.78, 2.68, 2.58, 2.56, 2.55, 2.53, 2.45, 2.34, 2.32, 2.14, 2.13, 2.12, 1.96, 1.94, 1.51, 1.35, 1.12, 0.90, 0.71, 0.56, 0.55

Corresponding total number of genes of each Phenopedia disease:
(In the same order as above)
318, 333, 396, 338, 39, 22, 24, 26, 73, 35, 20, 40, 26, 118, 50, 30, 286, 608, 20, 21, 656, 50, 36, 34, 42, 88, 42, 193, 855, 214, 48, 405, 543, 47, 25, 169, 31, 27, 37, 36, 21, 234, 26, 74, 38, 431, 45, 329, 52, 26, 437, 216, 28, 349, 341, 316, 21, 224, 63, 32, 308, 30, 28, 271, 37, 256, 24, 31, 54, 27

Corresponding number of genes of each Phenopedia disease with at least one TF hit (of selected TFs):
(In the same order as above)
63, 63, 73, 67, 14, 7, 6, 7, 17, 8, 6, 8, 7, 20, 11, 7, 47, 92, 5, 5, 102, 11, 8, 10, 8, 14, 8, 30, 124, 32, 9, 57, 74, 8, 5, 25, 6, 7, 10, 6, 4, 34, 4, 12, 8, 62, 6, 49, 7, 4, 59, 36, 6, 51, 50, 40, 4, 27, 10, 5, 43, 4, 4, 37, 5, 33, 2, 2, 5, 3

Corresponding total number of TF hits (of selected TFs) for each Phenopedia disease (in all genes):
(In the same order as above)
135, 134, 151, 137, 30, 16, 16, 17, 38, 21, 16, 21, 15, 46, 23, 18, 95, 178, 12, 14, 189, 23, 19, 21, 19, 34, 19, 58, 234, 68, 22, 109, 139, 19, 10, 52, 12, 15, 19, 15, 12, 71, 10, 26, 18, 116, 14, 95, 17, 11, 111, 70, 11, 95, 94, 76, 6, 58, 22, 13, 80, 8, 8, 66, 10, 60, 4, 4, 10, 5

Corresponding number of selected TFs each Phenopedia disease is involved with:
(In the same order as above)
3, 3, 3, 3, 3, 3, 3, 3, 3, 3, 3, 3, 3, 3, 3, 3, 3, 3, 3, 3, 3, 3, 3, 3, 3, 3, 3, 3, 3, 3, 3, 3, 3, 3, 3, 3, 3, 3, 3, 3, 3, 3, 3, 3, 3, 3, 3, 3, 3, 3, 3, 3, 3, 3, 3, 3, 3, 3, 3, 3, 3, 3, 3, 3, 3, 3, 3, 3, 3, 3

TFs ranked according to mean of effect sizes in cluster:

V$NRSF_Q4, V$CAP_01, V$MINI19_B

Corresponding mean of effect sizes of each TF:
(In the same order as above)
4.36, 4.29, 3.12

Corresponding total number of TF hits for each TF (genome-wide):
(In the same order as above)
490, 482, 488

Corresponding total number of TF hits for each TF (in all genes in selected Phenopedia diseases):
(In the same order as above)
1151, 1215, 1064

Corresponding number of genes (of selected Phenopedia diseases) each TF is involved with:
(In the same order as above)
153, 157, 143

Corresponding number of selected Phenopedia diseases each TF is involved with:
(In the same order as above)
70, 70, 70

Disease groups of selected Phenopedia diseases ranked according to sum of effect sizes
Signs and Symptoms | Nervous System Diseases | Central Nervous System Diseases | Brain Diseases | Substance-Related Disorders | Stomatognathic Diseases | Musculoskeletal Diseases | Movement Disorders | Mouth Diseases | Jaw Diseases | Basal Ganglia Diseases | Neurologic Manifestations | Tic Disorders | Epilepsy | Dystonic Disorders | Dystonia Musculorum Deformans | Alcohol-Related Disorders | Obsessive-Compulsive Disorder | Panic Disorder | Anxiety Disorders | Eating Disorders | Opioid-Related Disorders | Hallucinations | Hyperphagia | Epilepsy, Tonic-Clonic | Epilepsy, Generalized | Pain Threshold | Reinforcement (C0035007) | Neurotic Disorders | Psychopathology | Dystonia | Genetics, Behavioral | Reaction Time | Perceptual Disorders | Seizures | Cues | Mental Health | Bipolar Disorder | Borderline Personality Disorder | Mood Disorders | Substance Withdrawal Syndrome | Heroin Dependence | Bulimia Nervosa | Tics | Conduct Disorder | Tobacco Use Disorder | Schizophrenia | Alcoholism | Tourette Syndrome | Personality Assessment | Poisoning | Neurotoxicity Syndromes | Encephalitis | Alcohol-Induced Disorders, Nervous System | Alcohol-Induced Disorders | Delirium | Confusion | Bulimia | Antisocial Personality Disorder | Stress Disorders, Traumatic | Psychiatric Status Rating Scales | Appetite | Brief Psychiatric Rating Scale | Thinking | Affective Disorders, Psychotic | Personality Disorders | Memory, Short-Term | Attention | Parkinsonian Disorders | Parkinson Disease | Attention Deficit Disorder with Hyperactivity | Schizophrenia, Paranoid | Child Development Disorders, Pervasive | Autistic Disorder | Memory | Psychoses, Alcoholic | Alcohol Withdrawal Delirium | Learning | Dominance, Cerebral | Functional Laterality | Epilepsies, Partial | Postmortem Changes | Death | Tremor | Dyskinesias | Cocaine-Related Disorders | Essential Tremor | Thinness | Mental Status Schedule

Corresponding sum of effect sizes for each disease group of selected Phenopedia diseases
(In the same order as above)
213.86, 178.82, 178.82, 178.82, 110.81, 97.98, 97.98, 97.98, 97.98, 97.98, 97.98, 54.45, 38.26, 37.10, 32.07, 32.07, 30.20, 27.99, 26.94, 26.20, 26.09, 25.82, 22.68, 20.28, 20.26, 20.26, 19.80, 18.04, 17.81, 17.67, 17.57, 16.92, 16.64, 16.20, 15.86, 15.55, 15.37, 15.33, 14.78, 14.15, 13.97, 13.55, 13.37, 13.22, 13.21, 13.08, 12.70, 11.85, 11.85, 11.76, 10.53, 10.53, 10.53, 10.53, 10.53, 10.53, 10.53, 10.52, 10.47, 10.12, 9.81, 9.69, 9.14, 8.95, 8.71, 8.52, 8.34, 8.05, 7.89, 7.89, 7.73, 7.67, 7.65, 7.59, 7.36, 7.01, 7.01, 6.95, 6.41, 6.38, 5.89, 5.83, 5.83, 4.39, 4.39, 4.04, 2.71, 2.12, 1.65

Cluster for columns 39 to 41, rows 368 to 370

Phenopedia diseases

Carcinoma, Endometrioid | Cystadenocarcinoma | Cystadenocarcinoma, Serous

TFs

V$PAX1_B | V$FOXO3_01 | V$POU3F2_02

Information

All related TFs:
(List of all TFs that are related to any of the PWMs)
FOXO3, FOXO3a, FOXO3b, POU3F2, Pax-1

Ranked gene list:
(All genes of the selected Phenopedia diseases with hits of any of the selected TFs, ranked according to the total number of TF hits)
KRAS, LRP1, CYP1A1, BRAF, ABCB1, MSH6, MSH2, PTEN, CTNNB1

Corresponding total number of TF hits:
(For each gene listed above, the total number of TF hits for any of the selected TFs, multiplied by the number of selected Phenopedia diseases containing that gene)
9, 6, 3, 3, 3, 2, 2, 1, 1

Corresponding number of selected Phenopedia diseases each gene is involved with:
(In the same order as above)
3, 3, 3, 3, 3, 2, 2, 1, 1

Corresponding number of selected TFs each gene is involved with:
(In the same order as above)
3, 2, 1, 1, 1, 1, 1, 1, 1

Phenopedia diseases ranked according to mean of effect sizes in cluster:
Cystadenocarcinoma, Serous | Cystadenocarcinoma | Carcinoma, Endometrioid

Corresponding mean of effect sizes of each Phenopedia disease:
(In the same order as above)
2.93, 2.93, 2.49

Corresponding total number of genes of each Phenopedia disease:
(In the same order as above)
21, 21, 21

Corresponding number of genes of each Phenopedia disease with at least one TF hit (of selected TFs):
(In the same order as above)
7, 7, 7

Corresponding total number of TF hits (of selected TFs) for each Phenopedia disease (in all genes):
(In the same order as above)
10, 10, 10

Corresponding number of selected TFs each Phenopedia disease is involved with:
(In the same order as above)

3, 3, 3

TFs ranked according to mean of effect sizes in cluster:
V$FOXO3_01, V$PAX1_B, V$POU3F2_02

Corresponding mean of effect sizes of each TF:
(In the same order as above)
3.42, 3.33, 1.60

Corresponding total number of TF hits for each TF (genome-wide):
(In the same order as above)
519, 476, 522

Corresponding total number of TF hits for each TF (in all genes in selected Phenopedia diseases):
(In the same order as above)
12, 11, 7

Corresponding number of genes (of selected Phenopedia diseases) each TF is involved with:
(In the same order as above)
4, 4, 4

Corresponding number of selected Phenopedia diseases each TF is involved with:
(In the same order as above)
3, 3, 3

Disease groups of selected Phenopedia diseases ranked according to sum of effect sizes
Neoplasms | Adenoma, Oxyphilic | Adenocarcinoma | Neoplasms, Glandular and Epithelial | Neoplasms, Cystic, Mucinous, and Serous | Cystadenocarcinoma | Cystadenocarcinoma, Serous | Uterine Neoplasms | Uterine Diseases | Pelvic Neoplasms | Ovarian Neoplasms | Ovarian Diseases | Genital Neoplasms, Female | Genital Diseases, Female | Female Urogenital Diseases | Endometrial Neoplasms | Carcinoma, Endometrioid | Abdominal Neoplasms

Corresponding sum of effect sizes for each disease group of selected Phenopedia diseases
(In the same order as above)
25.04, 25.04, 25.04, 17.57, 17.57, 17.57, 8.79, 7.46, 7.46, 7.46, 7.46, 7.46, 7.46, 7.46, 7.46, 7.46, 7.46, 7.46

Cluster for columns 52 to 55, rows 240 to 243

Phenopedia diseases

Meningeal Neoplasms | Brain Neoplasms | Central Nervous System Neoplasms | Nervous System Neoplasms

TFs

V$GABP_B | V$ELK1_02 | V$IPF1_Q4_01 | V$NRF1_Q6

Information

All related TFs:
(List of all TFs that are related to any of the PWMs)
Elk-1, Elk-1-isoform1, GABP-alpha, GABP-alpha:GABP-beta, GABP-beta1, GABP-beta2, IPF1,

IPF1:Pbx, NRF-1

Ranked gene list:
(All genes of the selected Phenopedia diseases with hits of any of the selected TFs, ranked according to the total number of TF hits)
RECQL4, NHEJ1, GNAI2, PCNA, TDP1, TDG, SMUG1, RTEL1, POLG, POLD1, NUDT1, MSH5, HRAS, GNAS, FANCD2, CDK4, BRCA2, XRCC5, XRCC2, MUTYH, EXO1, ERCC1, CHAF1A, APEX1, XRCC6, TGFB1, RAD51L1, POMT1, PDE11A, NF1, IGF2, IGF1R, HEL308, GTF2H3, ERCC6, DCLRE1C, BLM, AURKB, AIP, ABCB1, NUMBL

Corresponding total number of TF hits:
(For each gene listed above, the total number of TF hits for any of the selected TFs, multiplied by the number of selected Phenopedia diseases containing that gene)
9, 9, 9, 8, 6, 6, 6, 6, 6, 6, 6, 6, 6, 6, 6, 6, 6, 4, 4, 4, 4, 4, 4, 4, 3, 3, 3, 3, 3, 3, 3, 3, 3, 3, 3, 3, 3, 3, 3, 3, 1

Corresponding number of selected Phenopedia diseases each gene is involved with:
(In the same order as above)
3, 3, 3, 4, 3, 3, 3, 3, 3, 3, 3, 3, 3, 3, 3, 3, 3, 4, 4, 4, 4, 4, 4, 4, 3, 3, 3, 3, 3, 1, 3, 3, 3, 3, 3, 3, 3, 3, 3, 3, 1

Corresponding number of selected TFs each gene is involved with:
(In the same order as above)
3, 3, 3, 2, 2, 2, 2, 2, 2, 2, 2, 2, 2, 2, 2, 2, 2, 1, 1, 1, 1, 1, 1, 1, 1, 1, 1, 1, 1, 3, 1, 1, 1, 1, 1, 1, 1, 1, 1, 1, 1

Phenopedia diseases ranked according to mean of effect sizes in cluster:
Nervous System Neoplasms | Meningeal Neoplasms | Central Nervous System Neoplasms | Brain Neoplasms

Corresponding mean of effect sizes of each Phenopedia disease:
(In the same order as above)
3.48, 3.48, 3.32, 1.08

Corresponding total number of genes of each Phenopedia disease:
(In the same order as above)
182, 105, 174, 132

Corresponding number of genes of each Phenopedia disease with at least one TF hit (of selected TFs):
(In the same order as above)
41, 26, 39, 21

Corresponding total number of TF hits (of selected TFs) for each Phenopedia disease (in all genes):
(In the same order as above)
63, 40, 59, 28

Corresponding number of selected TFs each Phenopedia disease is involved with:
(In the same order as above)
4, 4, 4, 4

TFs ranked according to mean of effect sizes in cluster:
V$GABP_B, V$NRF1_Q6, V$ELK1_02, V$IPF1_Q4_01

Corresponding mean of effect sizes of each TF:
(In the same order as above)
3.40, 3.31, 3.13, 1.53

Corresponding total number of TF hits for each TF (genome-wide):

(In the same order as above)
391, 418, 372, 520

Corresponding total number of TF hits for each TF (in all genes in selected Phenopedia diseases):
(In the same order as above)
47, 50, 44, 49

Corresponding number of genes (of selected Phenopedia diseases) each TF is involved with:
(In the same order as above)
16, 16, 15, 16

Corresponding number of selected Phenopedia diseases each TF is involved with:
(In the same order as above)
4, 4, 4, 4

Disease groups of selected Phenopedia diseases ranked according to sum of effect sizes
Nervous System Neoplasms | Nervous System Diseases | Central Nervous System Neoplasms | Central Nervous System Diseases | Neoplasms | Spinal Diseases | Spinal Cord Neoplasms | Spinal Cord Diseases | Musculoskeletal Diseases | Meningeal Neoplasms | Bone Diseases | Head and Neck Neoplasms | Brain Neoplasms | Brain Diseases

Corresponding sum of effect sizes for each disease group of selected Phenopedia diseases
(In the same order as above)
45.47, 45.47, 31.54, 31.54, 18.24, 13.91, 13.91, 13.91, 13.91, 13.91, 13.91, 4.32, 4.32, 4.32

Cluster for columns 635 to 638, rows 342 to 345

Phenopedia diseases

Thrombocytopenia | Neutropenia | Agranulocytosis | Leukopenia

TFs

V$P53_01 | V$E47_02 | V$IRF1_01 | V$MEIS1BHOXA9_02

Information

All related TFs:
(List of all TFs that are related to any of the PWMs)
E47, HOXA9, HOXA9B, HOXA9a, HOXA9b, IRF-1, Meis-1, Meis-1b, p53, p53-isoform-1

Ranked gene list:
(All genes of the selected Phenopedia diseases with hits of any of the selected TFs, ranked according to the total number of TF hits)
UGT1A7, UGT1A1, UGT1A9, ITPA, IRF7, ERCC2, XRCC3, TP53, STAT2, STAT1, MGMT, IRF9, IRF3, GSTP1, DDX58, ALDH3A1, UGT1A8, UGT1A10, TOP1, SLC37A4, IL18, HNF4A, HLA-A, CSF3R, CES2, XDH, SOS1, SLC28A1, CTLA4, BRCA2, SLC19A1, RET, RAD51, PTPN11, PRF1, NFKB1, IMPDH2, IFNGR1, IFNG, GIMAP5, FPGS, F5, CYCS

Corresponding total number of TF hits:
(For each gene listed above, the total number of TF hits for any of the selected TFs, multiplied by the number of selected Phenopedia diseases containing that gene)
12, 12, 9, 8, 8, 8, 6, 4, 4, 4, 4, 4, 4, 4, 4, 4, 3, 3, 3, 3, 3, 3, 3, 3, 3, 2, 2, 2, 2, 2, 1, 1, 1, 1, 1, 1, 1, 1, 1, 1, 1, 1,

1, 1

Corresponding number of selected Phenopedia diseases each gene is involved with:
(In the same order as above)
4, 4, 3, 4, 4, 4, 3, 4, 4, 4, 4, 4, 4, 4, 4, 2, 1, 1, 3, 3, 3, 3, 3, 3, 3, 2, 1, 2, 1, 2, 1, 1, 1, 1, 1, 1, 1, 1, 1, 1, 1, 1, 1

Corresponding number of selected TFs each gene is involved with:
(In the same order as above)
3, 3, 3, 2, 2, 2, 2, 1, 1, 1, 1, 1, 1, 1, 1, 2, 3, 3, 1, 1, 1, 1, 1, 1, 1, 1, 2, 1, 2, 1, 1, 1, 1, 1, 1, 1, 1, 1, 1, 1, 1, 1, 1

Phenopedia diseases ranked according to mean of effect sizes in cluster:
Neutropenia | Agranulocytosis | Thrombocytopenia | Leukopenia

Corresponding mean of effect sizes of each Phenopedia disease:
(In the same order as above)
2.17, 1.93, 1.56, 1.54

Corresponding total number of genes of each Phenopedia disease:
(In the same order as above)
114, 81, 121, 107

Corresponding number of genes of each Phenopedia disease with at least one TF hit (of selected TFs):
(In the same order as above)
32, 21, 26, 25

Corresponding total number of TF hits (of selected TFs) for each Phenopedia disease (in all genes):
(In the same order as above)
45, 31, 35, 38

Corresponding number of selected TFs each Phenopedia disease is involved with:
(In the same order as above)
4, 4, 4, 4

TFs ranked according to mean of effect sizes in cluster:
V$IRF1_01, V$E47_02, V$P53_01, V$MEIS1BHOXA9_02

Corresponding mean of effect sizes of each TF:
(In the same order as above)
2.99, 2.22, 1.24, 0.74

Corresponding total number of TF hits for each TF (genome-wide):
(In the same order as above)
499, 445, 446, 494

Corresponding total number of TF hits for each TF (in all genes in selected Phenopedia diseases):
(In the same order as above)
50, 32, 35, 32

Corresponding number of genes (of selected Phenopedia diseases) each TF is involved with:
(In the same order as above)
17, 13, 16, 14

Corresponding number of selected Phenopedia diseases each TF is involved with:
(In the same order as above)
4, 4, 4, 4

Disease groups of selected Phenopedia diseases ranked according to sum of effect sizes
Leukopenia | Leukocyte Disorders | Neutropenia | Agranulocytosis | Thrombocytopenia | Signs and Symptoms | Blood Platelet Disorders | Blood Coagulation Disorders

Corresponding sum of effect sizes for each disease group of selected Phenopedia diseases
(In the same order as above)
22.53, 22.53, 16.36, 7.70, 6.22, 6.22, 6.22, 6.22

Cluster for columns 812 to 818, rows 250 to 252

Phenopedia diseases

Cerebellar Ataxia | Cerebellar Diseases | Ocular Motility Disorders | Ophthalmoplegia | Language Disorders | Communication Disorders | Learning Disorders

TFs

V$CREB_Q4_01 | V$CREB_Q4 | V$CREB_Q2

Information

All related TFs:
(List of all TFs that are related to any of the PWMs)
CREB, CREBbeta, CREMalpha, CREMbeta, CREMgamma, CREMtau, CREMtau1, CREMtau2, CREMtaualpha, deltaCREB

Ranked gene list:
(All genes of the selected Phenopedia diseases with hits of any of the selected TFs, ranked according to the total number of TF hits)
GRN, PARK2, ADRA2A, SYNGAP1, SCA13, NPHP1, NF1, KCNC3, AHI1, KIF21A, GBA, FOXP1

Corresponding total number of TF hits:
(For each gene listed above, the total number of TF hits for any of the selected TFs, multiplied by the number of selected Phenopedia diseases containing that gene)
12, 9, 9, 6, 6, 6, 6, 6, 6, 2, 2, 2

Corresponding number of selected Phenopedia diseases each gene is involved with:
(In the same order as above)
4, 3, 3, 2, 2, 2, 2, 2, 2, 2, 2, 1

Corresponding number of selected TFs each gene is involved with:
(In the same order as above)
3, 3, 3, 3, 3, 3, 3, 3, 3, 1, 1, 2

Phenopedia diseases ranked according to mean of effect sizes in cluster:
Ophthalmoplegia | Ocular Motility Disorders | Communication Disorders | Cerebellar Ataxia | Learning Disorders | Language Disorders | Cerebellar Diseases

Corresponding mean of effect sizes of each Phenopedia disease:
(In the same order as above)
2.54, 2.34, 1.98, 1.92, 1.90, 1.86, 1.04

Corresponding total number of genes of each Phenopedia disease:
(In the same order as above)
18, 21, 39, 37, 26, 27, 43

Corresponding number of genes of each Phenopedia disease with at least one TF hit (of selected TFs):
(In the same order as above)
4, 4, 4, 5, 3, 3, 4

Corresponding total number of TF hits (of selected TFs) for each Phenopedia disease (in all genes):
(In the same order as above)
8, 8, 12, 15, 9, 8, 12

Corresponding number of selected TFs each Phenopedia disease is involved with:
(In the same order as above)
3, 3, 3, 3, 3, 3, 3

TFs ranked according to mean of effect sizes in cluster:
V$CREB_Q4, V$CREB_Q4_01, V$CREB_Q2

Corresponding mean of effect sizes of each TF:
(In the same order as above)
2.22, 1.88, 1.73

Corresponding total number of TF hits for each TF (genome-wide):
(In the same order as above)
418, 421, 435

Corresponding total number of TF hits for each TF (in all genes in selected Phenopedia diseases):
(In the same order as above)
25, 23, 24

Corresponding number of genes (of selected Phenopedia diseases) each TF is involved with:
(In the same order as above)
11, 10, 10

Corresponding number of selected Phenopedia diseases each TF is involved with:
(In the same order as above)
7, 7, 7

Disease groups of selected Phenopedia diseases ranked according to sum of effect sizes
Signs and Symptoms | Communication Disorders | Nervous System Diseases | Ophthalmoplegia | Eye Diseases | Cerebellar Diseases | Central Nervous System Diseases | Brain Diseases | Vision, Low | Vision Disorders | Ocular Motility Disorders | Cranial Nerve Diseases | Tremor | Stomatognathic Diseases | Musculoskeletal Diseases | Movement Disorders | Mouth Diseases | Jaw Diseases | Dyskinesias | Cerebellar Ataxia | Basal Ganglia Diseases | Learning Disorders | Language Disorders

Corresponding sum of effect sizes for each disease group of selected Phenopedia diseases
(In the same order as above)
17.24, 17.24, 15.91, 14.65, 14.65, 8.88, 8.88, 8.88, 7.03, 7.03, 7.03, 7.03, 5.76, 5.76, 5.76, 5.76, 5.76, 5.76, 5.76, 5.76, 5.76, 5.71, 5.58

Cluster for columns 290 to 299, rows 7 to 7

Phenopedia diseases

Pregnancy Complications | Hypertension, Pregnancy-Induced | Pre-Eclampsia | Premature Birth | Placenta Diseases | Chorioamnionitis | Fetal Membranes, Premature Rupture | Fetal Diseases | Obstetric Labor, Premature | Obstetric Labor Complications

TFs

V$HSF1_Q6

Information

All related TFs:
(List of all TFs that are related to any of the PWMs)
HSF1, HSF1-L, HSF1-S, HSF1long, HSF1short

Ranked gene list:
(All genes of the selected Phenopedia diseases with hits of any of the selected TFs, ranked according to the total number of TF hits)
TNFRSF1B, THBS1, PTGER4, PDGFB, MMP17, MGP, IRS1, HLA-G, HLA-E, FGF2, CXCR1, CSF3, CRHR2, COL4A3, COL4A2, COL4A1, MTHFD1, CTLA4, HSPA1L, FASLG, SERPINH1, RAN, IL8RA, IL10RB, HSPA6, HLA-A, CBS, SLC19A1, PTGDR, HLA-B, HLA-C, ENG, DRD4, CYP11B2, APOC2, ACP1, TYMS, TACR2, KCNQ1, ITGB2, IDE, HMOX1, HMGB1, GATA3, FTO, CXCL9

Corresponding total number of TF hits:
(For each gene listed above, the total number of TF hits for any of the selected TFs, multiplied by the number of selected Phenopedia diseases containing that gene)
10, 10, 10, 10, 10, 10, 10, 10, 10, 10, 10, 10, 10, 10, 10, 10, 8, 8, 7, 7, 6, 5, 5, 5, 5, 5, 5, 4, 4, 4, 3, 3, 3, 3, 3, 2, 1, 1, 1, 1, 1, 1, 1, 1, 1, 1

Corresponding number of selected Phenopedia diseases each gene is involved with:
(In the same order as above)
10, 10, 10, 10, 10, 10, 10, 10, 10, 10, 10, 10, 10, 10, 10, 10, 8, 8, 7, 7, 6, 5, 5, 5, 5, 5, 5, 4, 4, 4, 3, 3, 3, 3, 3, 2, 1, 1, 1, 1, 1, 1, 1, 1, 1, 1

Corresponding number of selected TFs each gene is involved with:
(In the same order as above)
1, 1, 1, 1, 1, 1, 1, 1, 1, 1, 1, 1, 1, 1, 1, 1, 1, 1, 1, 1, 1, 1, 1, 1, 1, 1, 1, 1, 1, 1, 1, 1, 1, 1, 1, 1, 1, 1, 1, 1, 1, 1, 1, 1, 1, 1

Phenopedia diseases ranked according to mean of effect sizes in cluster:
Obstetric Labor Complications | Pregnancy Complications | Obstetric Labor, Premature | Placenta Diseases | Hypertension, Pregnancy-Induced | Pre-Eclampsia | Fetal Diseases | Fetal Membranes, Premature Rupture | Premature Birth | Chorioamnionitis

Corresponding mean of effect sizes of each Phenopedia disease:
(In the same order as above)
3.78, 3.38, 3.33, 2.87, 2.82, 2.75, 2.59, 2.32, 2.16, 1.73

Corresponding total number of genes of each Phenopedia disease:
(In the same order as above)
296, 485, 289, 204, 278, 282, 289, 198, 413, 197

**Corresponding number of genes of each Phenopedia disease with at least one TF hit (of selected

TFs):
(In the same order as above)
31, 42, 29, 20, 25, 26, 26, 18, 32, 16

Corresponding total number of TF hits (of selected TFs) for each Phenopedia disease (in all genes):
(In the same order as above)
31, 42, 29, 20, 25, 26, 26, 18, 32, 16

Corresponding number of selected TFs each Phenopedia disease is involved with:
(In the same order as above)
1, 1, 1, 1, 1, 1, 1, 1, 1, 1

TFs ranked according to mean of effect sizes in cluster:
V$HSF1_Q6

Corresponding mean of effect sizes of each TF:
(In the same order as above)
2.77

Corresponding total number of TF hits for each TF (genome-wide):
(In the same order as above)
499

Corresponding total number of TF hits for each TF (in all genes in selected Phenopedia diseases):
(In the same order as above)
265

Corresponding number of genes (of selected Phenopedia diseases) each TF is involved with:
(In the same order as above)
46

Corresponding number of selected Phenopedia diseases each TF is involved with:
(In the same order as above)
10

Disease groups of selected Phenopedia diseases ranked according to sum of effect sizes
Pregnancy Complications | Vascular Diseases | Hypertension, Pregnancy-Induced | Hypertension | Cardiovascular Diseases | Uterine Diseases | Placenta Diseases | Genital Diseases, Female | Female Urogenital Diseases | Fetal Membranes, Premature Rupture | Obstetric Labor Complications | Obstetric Labor, Premature | Pre-Eclampsia | Fetal Diseases | Premature Birth | Signs and Symptoms | Congenital, Hereditary, and Neonatal Diseases and Abnormalities | Chorioamnionitis

Corresponding sum of effect sizes for each disease group of selected Phenopedia diseases
(In the same order as above)
15.16, 5.57, 5.57, 5.57, 5.57, 4.61, 4.61, 4.61, 4.61, 4.05, 3.78, 3.33, 2.75, 2.59, 2.16, 1.73, 1.73, 1.73

Cluster for columns 400 to 403, rows 12 to 14

Phenopedia diseases

Eclampsia | Infertility, Female | Abortion, Habitual | Abortion, Spontaneous

TFs

V$EGR3_01 | V$EGR2_01 | V$EGR1_01

Information

All related TFs:
(List of all TFs that are related to any of the PWMs)
Egr-1, Egr-2, Egr-3

Ranked gene list:
(All genes of the selected Phenopedia diseases with hits of any of the selected TFs, ranked according to the total number of TF hits)
TGFB1, COL1A1, ANXA5, VEGFA, VEGF, F12, ACHE, SERPINE1, IGF2, HLA-DQB1, F7, F5, CD46, IFNGR1, CYP1A1, APEX1, TCN2, ITGA2, IL4R, IGFBP4, SLC19A1, LEPR, DRD4, DDAH1

Corresponding total number of TF hits:
(For each gene listed above, the total number of TF hits for any of the selected TFs, multiplied by the number of selected Phenopedia diseases containing that gene)
12, 9, 9, 8, 6, 6, 6, 4, 4, 4, 4, 4, 4, 3, 3, 3, 2, 2, 2, 2, 1, 1, 1, 1

Corresponding number of selected Phenopedia diseases each gene is involved with:
(In the same order as above)
4, 3, 3, 4, 3, 3, 2, 4, 2, 4, 2, 4, 2, 1, 3, 1, 2, 2, 1, 2, 1, 1, 1, 1

Corresponding number of selected TFs each gene is involved with:
(In the same order as above)
3, 3, 3, 2, 2, 2, 3, 1, 2, 1, 2, 1, 2, 3, 1, 3, 1, 1, 2, 1, 1, 1, 1, 1

Phenopedia diseases ranked according to mean of effect sizes in cluster:
Eclampsia | Abortion, Habitual | Abortion, Spontaneous | Infertility, Female

Corresponding mean of effect sizes of each Phenopedia disease:
(In the same order as above)
3.64, 3.10, 2.65, 0.81

Corresponding total number of genes of each Phenopedia disease:
(In the same order as above)
81, 82, 98, 45

Corresponding number of genes of each Phenopedia disease with at least one TF hit (of selected TFs):
(In the same order as above)
17, 15, 17, 7

Corresponding total number of TF hits (of selected TFs) for each Phenopedia disease (in all genes):
(In the same order as above)
31, 29, 31, 10

Corresponding number of selected TFs each Phenopedia disease is involved with:
(In the same order as above)
3, 3, 3, 3

TFs ranked according to mean of effect sizes in cluster:
V$EGR3_01, V$EGR1_01, V$EGR2_01

Corresponding mean of effect sizes of each TF:
(In the same order as above)
3.11, 2.45, 2.09

Corresponding total number of TF hits for each TF (genome-wide):
(In the same order as above)
452, 452, 457

Corresponding total number of TF hits for each TF (in all genes in selected Phenopedia diseases):
(In the same order as above)
39, 31, 31

Corresponding number of genes (of selected Phenopedia diseases) each TF is involved with:
(In the same order as above)
15, 15, 13

Corresponding number of selected Phenopedia diseases each TF is involved with:
(In the same order as above)
4, 4, 4

Disease groups of selected Phenopedia diseases ranked according to sum of effect sizes
Pregnancy Complications | Abortion, Spontaneous | Vascular Diseases | Hypertension, Pregnancy-Induced | Hypertension | Eclampsia | Cardiovascular Diseases | Abortion, Habitual | Male Urogenital Diseases | Infertility, Female | Infertility | Genital Diseases, Male | Genital Diseases, Female | Female Urogenital Diseases

Corresponding sum of effect sizes for each disease group of selected Phenopedia diseases
(In the same order as above)
28.17, 17.24, 10.92, 10.92, 10.92, 10.92, 10.92, 9.31, 2.42, 2.42, 2.42, 2.42, 2.42, 2.42

Cluster for columns 612 to 613, rows 289 to 290

Phenopedia diseases

Barrett Esophagus | Digestive System Abnormalities

TFs

V$HIF1_Q5 | V$HIF1_Q3

Information

All related TFs:
(List of all TFs that are related to any of the PWMs)
HIF-1, HIF-1alpha, HIF-1alpha-isoform1

Ranked gene list:
(All genes of the selected Phenopedia diseases with hits of any of the selected TFs, ranked according to the total number of TF hits)
PIK3CA, IGF1R, CCND1, HOXB7, HLA-B, HFE, GDNF, EGFR

Corresponding total number of TF hits:

(For each gene listed above, the total number of TF hits for any of the selected TFs, multiplied by the number of selected Phenopedia diseases containing that gene)
4, 4, 4, 2, 2, 2, 2, 2

Corresponding number of selected Phenopedia diseases each gene is involved with:
(In the same order as above)
2, 2, 2, 1, 2, 2, 1, 2

Corresponding number of selected TFs each gene is involved with:
(In the same order as above)
2, 2, 2, 2, 1, 1, 2, 1

Phenopedia diseases ranked according to mean of effect sizes in cluster:
Barrett Esophagus | Digestive System Abnormalities

Corresponding mean of effect sizes of each Phenopedia disease:
(In the same order as above)
3.27, 3.18

Corresponding total number of genes of each Phenopedia disease:
(In the same order as above)
42, 73

Corresponding number of genes of each Phenopedia disease with at least one TF hit (of selected TFs):
(In the same order as above)
6, 8

Corresponding total number of TF hits (of selected TFs) for each Phenopedia disease (in all genes):
(In the same order as above)
9, 13

Corresponding number of selected TFs each Phenopedia disease is involved with:
(In the same order as above)
2, 2

TFs ranked according to mean of effect sizes in cluster:
V$HIF1_Q3, V$HIF1_Q5

Corresponding mean of effect sizes of each TF:
(In the same order as above)
3.67, 2.78

Corresponding total number of TF hits for each TF (genome-wide):
(In the same order as above)
432, 433

Corresponding total number of TF hits for each TF (in all genes in selected Phenopedia diseases):
(In the same order as above)
12, 10

Corresponding number of genes (of selected Phenopedia diseases) each TF is involved with:
(In the same order as above)
7, 6

Corresponding number of selected Phenopedia diseases each TF is involved with:

(In the same order as above)
2, 2

Disease groups of selected Phenopedia diseases ranked according to sum of effect sizes
Digestive System Diseases | Gastrointestinal Diseases | Esophagitis | Esophageal Diseases | Barrett Esophagus | Digestive System Abnormalities

Corresponding sum of effect sizes for each disease group of selected Phenopedia diseases
(In the same order as above)
12.89, 6.53, 6.53, 6.53, 6.53, 6.36
