# Supplementary material for: The differential disease regulome
Source: BMC Genomics. 2011 Jul 7;12:353. doi: 10.1186/1471-2164-12-353 (PMC3160420; doi:10.1186/1471-2164-12-353)
Supplement: Additional file 3 — TF-GO clusters. A listing of 105 manually indentified TF-GO clusters in the Gene Ontology regulome. [file 1471-2164-12-353-S3.PDF]

Cluster for columns 60 to 76, rows 97 to 111

Gene ontology terms

regulation of gene expression | transcription factor binding | sequence-specific DNA binding | histone acetylation | regulation of transcription | chromatin remodeling complex | gene silencing | histone deacetylase activity | histone deacetylation | DNA methylation | methylation | centromeric heterochromatin | histone methylation | histone modification | chromatin modification | euchromatin | chromatin silencing

TFs

V$STAT6_01 | V$STAT5A_03 | V$STAT4_01 | V$STAT5A_04 | V$STAT1_03 | V$CEBP_Q3 | V$PAX2_02 | V$HMGIY_Q6 | V$HOXA4_Q2 | V$TST1_01 | V$NFAT_Q6 | V$NFAT_Q4_01 | V$CEBP_Q2_01 | V$CEBPA_01 | V$CEBP_Q2

Information

All related TFs:
(List of all TFs that are related to any of the PWMs)
ANF-2, C/EBP, C/EBPalpha, C/EBPalpha(p20), C/EBPalpha(p30), C/EBPbeta, C/EBPbeta(LAP), C/EBPbeta(p20), C/EBPbeta(p34), C/EBPbeta(p35), C/EBPdelta, C/EBPepsilon, C/EBPgamma, HMG, HMG-Y, HMGI-C, HOXA4, NF-AT, NF-AT1, NF-AT1C, NF-AT2, NF-AT3, NF-AT4, POU3F1, Pax-2, Pax-2.1, Pax-2.2, Pax-2a, Pax-2b, STAT1, STAT1alpha, STAT4, STAT5A, STAT6

Ranked gene list:
(All genes of the selected Gene ontology terms with hits of any of the selected TFs, ranked according to the total number of TFBS)
HIST1H4I, CBX5, SMARCA2, HIST4H4, NR3C1, IKZF1, SIN3A, RASSF1, EGR1, ETS1, STAT1, SSSCA1, LBX1, TFAP2A, SETD7, RUNX1, MLL, IRF1, CDKN1B, BMI1, ID2, TIPARP, SLC38A2, HOXA5, HIST1H3E, NNAT, LEF1, JMJD3, GATA3, CISH, ZMYND10, RASSF5, MYB, MBD1, USF1, SOCS1, RUNX3, PAX5, MTA2, JUN, POLR2A, VDR, TNF, RING1, MNT, IL23A, ESR1, CREB1, TAF8, S100A4, MYC, JUNB, CHD3, ANXA6, TRERF1, TGFBR2, SATB1, RASSF2, RARA, PTPN6, MLL2, KAT2B, HOXA9, HOXA7, HOXA10, HOXA1, HDAC7, H2AFZ, GNAS, EGR2, CDK4, CCND2, BCL11B, BAZ2A, H2AFX, CDK6, CBX8, TNFAIP8, CHD4, CBX4, STAT3, RBPJ, MAZ, KLF9, CTNNB1, BCL2, SP1, CDKN1A, CBX3, PSMP, PLEC1, EHMT2, STAT5A, SRF, SETD1A, MAPK14, HMGA1, CADM1, PCNA, MAF, E2F3, CYP27B1, THRA, TCF7, TARBP2, TAF10, ST8SIA4, SMAD7, PRDM1, PAX6, NR4A2, NFKBIA, NFIA, MXI1, KLF6, HOXC8, GFI1, GADD45A, FLI1, EOMES, E2F4, DDAH2, CXCR4, BTG1, ARID1A, UBE2B, PPP2CA, NR4A1, LDB1, ID3, H3F3B, DCTN3, CDKN2C, CD44, BCL2L1, TP53, PURA, EP300, CFL1, ANP32A, SOX2, SMAD3, PPP1R1B, PCGF1, LAPTM5, HSD17B8, HOXB5, HIST1H3C, HDAC5, GTF3A, CHD1, TUSC4, PTCH1, POU4F1, NAB2, IRF2, HNRNPL, HNRNPA2B1, CDK2, SATB2, PRMT1, POU2F1, MAP3K1, ISCU, GATA2, COL11A2, ARNTL, UBC, OTUD4, KCNIP2, HNRNPC, FOSB, WBP7, PELP1, ING1, HMGN4, CTCF, CAMK2G, AKT1, ACTR1A, ZEB1, TMEM115, TBL1XR1, REPIN1, MZF1, MYST4, MEIS2, LPIN1, HOXD11, HIST1H1A, GLI1, FBXL10, DAXX, UROD, STAT6, SIL1, RUVBL2, RERE, RB1, PML, IL6, HNRNPK, HIST1H1E, HIC1, FLOT2, CNOT7, CD47, CCND1, ZFP161, TTF2, STX16, RGP1, PSMC5, MAX, GCS1, GAPDH, FOS, EMP3, CASP2, BAK1, APBB3, AHSA1, WNK1, TDG, STK4, SRCAP, SRA1, SOCS3, SLC20A1, SGCE, SEMA3B, SELPLG, RHOQ, POU3F2, PHF1, PEG10, PCGF2, NF1, MLLT3, IGF1R, HOXA4, GYPC, FUS, FOXO1, ETV1, EIF4G2, CST6, CHPT1, CALM2, APEX1, VEGFA, TES, STAT5B, SLC5A6, PRMT5, PITX2, PARP1, NRF1, NFIC, LMNA, JARID1B, HIST1H2BB, HEMK1, GNASAS, ETV5, E2F2, CD82, CAPG, CALM1, C2orf28, BRD4, BRAF, ATF4, ADIPOR1, TBPL1, SUPT4H1, SP4, SMC4, SMAD4, SKP2, RRBP1, RHOA, PPARA, POLR3A, PIK3CA, PIH1D1, PEA15, PAX3, NCOA3, NBPF1, MYST2, MTRR,

MT2A, MEST, MEIS1, KRAS, IRF4, INTS6, IL2, HLA-DMA, H3F3A, GTF2I, GNB2L1, GLUL, EPB41, ELF1, EFNB2, EEF1A1, CXXC1, CD40, BRCA2, ATF2, TPMT, TMEM43, TLE4, TAL1, REEP5, PIK3CG, NOS2, NAT6, MMAA, MAD1L1, LGALS1, KLF5, JMJD2C, IWS1, IRF8, HNRNPUL1, HNRNPU, HMBS, HIST1H2BO, GLIPR1, GATAD2B, GADD45B, FBL, ERAP2, EPHB6, DNAJC14, DLX5, CGGBP1, CENPE, CCR6, CBX1, BTAF1, BRE, BDNF, BANF1, ASCL1

Corresponding total number of TFBS:
(For each gene listed above, the total number of TFBS for any of the selected TFs, multiplied by the number of selected Gene ontology terms containing that gene)
240, 156, 112, 112, 108, 105, 104, 98, 80, 75, 70, 70, 66, 65, 60, 60, 60, 60, 60, 60, 56, 48, 48, 48, 48, 45, 45, 45, 45, 45, 42, 42, 42, 42, 40, 40, 40, 40, 40, 40, 39, 36, 36, 36, 36, 36, 36, 36, 35, 33, 33, 33, 33, 32, 30, 30, 30, 30, 30, 30, 30, 30, 30, 30, 30, 30, 30, 30, 30, 30, 30, 30, 30, 28, 28, 28, 26, 26, 26, 24, 24, 24, 24, 24, 24, 22, 22, 22, 20, 20, 20, 18, 18, 18, 18, 18, 18, 16, 16, 16, 16, 15, 15, 15, 15, 15, 15, 15, 15, 15, 15, 15, 15, 15, 15, 15, 15, 15, 15, 15, 15, 15, 15, 15, 14, 14, 14, 14, 14, 14, 14, 14, 14, 13, 13, 13, 13, 13, 12, 12, 12, 12, 12, 12, 12, 12, 12, 12, 12, 11, 11, 11, 11, 11, 11, 11, 11, 10, 10, 10, 10, 10, 10, 10, 9, 9, 9, 9, 9, 8, 8, 8, 8, 8, 8, 8, 8, 7, 7, 7, 7, 7, 7, 7, 7, 7, 7, 7, 7, 6, 6, 6, 6, 6, 6, 6, 6, 6, 6, 6, 6, 6, 5, 5, 5, 5, 5, 5, 5, 5, 5, 5, 5, 5, 5, 5, 4, 4, 4, 4, 4, 4, 4, 4, 4, 4, 4, 4, 4, 4, 4, 4, 4, 4, 4, 4, 4, 4, 4, 4, 4, 4, 4, 3, 3, 3, 3, 3, 3, 3, 3, 3, 3, 3, 3, 3, 3, 3, 3, 3, 3, 3, 3, 3, 3, 3, 3, 2, 2, 2, 2, 2, 2, 2, 2, 2, 2, 2, 2, 2, 2, 2, 2, 2, 2, 2, 2, 2, 2, 2, 2, 2, 2, 2, 2, 2, 2, 2, 2, 2, 2, 2, 2, 2, 2, 2, 1, 1, 1, 1, 1, 1, 1, 1, 1, 1, 1, 1, 1, 1, 1, 1, 1, 1, 1, 1, 1, 1, 1, 1, 1, 1, 1, 1, 1, 1, 1, 1, 1, 1, 1, 1

Corresponding number of selected Gene ontology terms each gene is involved with:
(In the same order as above)
16, 13, 8, 16, 9, 7, 8, 7, 8, 5, 5, 5, 6, 5, 6, 4, 4, 4, 4, 5, 4, 4, 4, 4, 4, 3, 3, 3, 3, 3, 3, 3, 7, 7, 5, 4, 4, 5, 4, 10, 3, 4, 3, 3, 3, 6, 9, 9, 5, 3, 11, 3, 3, 4, 2, 3, 2, 2, 3, 2, 2, 10, 2, 2, 2, 3, 2, 10, 2, 3, 2, 2, 2, 2, 4, 2, 2, 2, 2, 2, 4, 3, 2, 2, 3, 4, 11, 11, 2, 4, 4, 10, 3, 6, 9, 6, 3, 3, 4, 2, 2, 2, 1, 1, 1, 1, 1, 1, 1, 1, 1, 1, 1, 1, 1, 1, 5, 1, 1, 5, 1, 1, 1, 1, 1, 1, 7, 1, 1, 1, 1, 1, 1, 1, 13, 1, 13, 1, 1, 3, 3, 1, 1, 1, 1, 1, 4, 6, 6, 4, 1, 1, 1, 1, 1, 1, 1, 1, 1, 5, 5, 1, 2, 2, 1, 1, 9, 3, 1, 3, 3, 2, 4, 2, 2, 8, 1, 2, 1, 1, 1, 1, 1, 1, 1, 1, 1, 1, 1, 1, 1, 3, 2, 3, 2, 1, 6, 6, 3, 3, 1, 3, 2, 6, 3, 6, 1, 5, 1, 1, 1, 1, 1, 1, 5, 1, 1, 1, 5, 5, 4, 4, 4, 4, 4, 1, 4, 2, 2, 1, 1, 1, 1, 2, 1, 2, 1, 1, 1, 1, 2, 2, 1, 1, 4, 2, 2, 2, 1, 1, 3, 1, 1, 1, 3, 3, 3, 1, 1, 1, 1, 3, 1, 1, 1, 1, 1, 1, 1, 3, 3, 1, 2, 1, 2, 1, 2, 1, 1, 2, 2, 1, 2, 1, 1, 1, 2, 2, 1, 2, 1, 2, 1, 2, 2, 1, 2, 1, 2, 1, 1, 2, 1, 1, 1, 2, 2, 2, 1, 2, 1, 1, 1, 1, 1, 1, 1, 1, 1, 1, 1, 1, 1, 1, 1, 1, 1, 1, 1, 1, 1, 1, 1, 1, 1, 1, 1, 1, 1, 1, 1, 1, 1, 1

Corresponding number of selected TFs each gene is involved with:
(In the same order as above)
15, 12, 14, 7, 12, 15, 13, 14, 10, 15, 14, 14, 11, 13, 10, 15, 15, 15, 15, 12, 14, 12, 12, 12, 12, 15, 15, 15, 15, 15, 14, 14, 6, 6, 8, 10, 10, 8, 10, 4, 13, 9, 12, 12, 12, 6, 4, 4, 7, 11, 3, 11, 11, 8, 15, 10, 15, 15, 10, 15, 15, 3, 15, 15, 15, 10, 15, 3, 15, 10, 15, 15, 15, 15, 7, 14, 14, 13, 13, 13, 6, 8, 12, 12, 8, 6, 2, 2, 11, 5, 5, 2, 6, 3, 2, 3, 6, 6, 4, 8, 8, 8, 15, 15, 15, 15, 15, 15, 15, 15, 15, 15, 15, 15, 15, 15, 15, 3, 15, 15, 3, 15, 15, 15, 15, 14, 14, 2, 14, 14, 14, 14, 14, 14, 14, 1, 13, 1, 13, 13, 4, 4, 12, 12, 12, 12, 12, 3, 2, 2, 3, 11, 11, 11, 11, 11, 11, 11, 11, 10, 2, 2, 10, 5, 5, 10, 10, 1, 3, 9, 3, 3, 4, 2, 4, 4, 1, 8, 4, 8, 7, 7, 7, 7, 7, 7, 7, 7, 7, 7, 7, 7, 2, 3, 2, 3, 6, 1, 1, 2, 2, 6, 2, 3, 1, 2, 1, 5, 1, 5, 5, 5, 5, 5, 5, 1, 5, 5, 5, 1, 1, 1, 1, 1, 1, 1, 4, 1, 2, 2, 4, 4, 4, 4, 2, 4, 2, 4, 4, 4, 4, 2, 2, 4, 4, 1, 2, 2, 2, 3, 3, 1, 3, 3, 3, 1, 1, 1, 3, 3, 3, 3, 1, 3, 3, 3, 3, 3, 3, 3, 1, 1, 3, 1, 2, 1, 2, 1, 2, 2, 1, 1, 2, 1, 2, 2, 2, 1, 1, 2, 1, 2, 1, 2, 1, 1, 2, 1, 2, 1, 2, 2, 1, 2, 2, 2, 1, 1, 1, 2, 1, 1, 1, 1, 1, 1, 1, 1, 1, 1, 1, 1, 1, 1, 1, 1, 1, 1, 1, 1, 1, 1, 1, 1, 1, 1, 1, 1, 1, 1, 1, 1

Gene ontology terms ranked according to mean of effect sizes in cluster:
DNA methylation | regulation of gene expression | chromatin remodeling complex | histone acetylation | sequence-specific DNA binding | chromatin silencing | transcription factor binding | regulation of transcription | histone deacetylation | histone modification | centromeric heterochromatin | histone deacetylase activity | methylation | chromatin modification | histone methylation | gene silencing | euchromatin

Corresponding mean of effect sizes of each Gene ontology term:
(In the same order as above)
1.74, 1.71, 1.62, 1.55, 1.32, 1.22, 1.22, 1.21, 1.19, 1.04, 1.01, 0.98, 0.93, 0.86, 0.81, 0.63, 0.35

Corresponding total number of genes of each Gene ontology term:
(In the same order as above)
437, 310, 105, 283, 118, 55, 231, 187, 144, 115, 51, 108, 715, 82, 101, 355, 62

Corresponding number of genes of each Gene ontology term with at least one TFBS (of selected TFs):
(In the same order as above)
99, 95, 32, 91, 36, 15, 75, 63, 44, 34, 12, 33, 152, 24, 27, 82, 17

Corresponding total number of TFBS (of selected TFs) for each Gene ontology term (in all genes):
(In the same order as above)
669, 619, 236, 513, 234, 124, 404, 350, 298, 244, 93, 226, 925, 163, 172, 544, 88

Corresponding number of selected TFs each Gene ontology term is involved with:
(In the same order as above)
15, 15, 15, 15, 15, 15, 15, 15, 15, 15, 15, 15, 15, 15, 15, 15, 15

TFs ranked according to mean of effect sizes in cluster:
V$STAT6_01, V$TST1_01, V$PAX2_02, V$NFAT_Q6, V$CEBP_Q2, V$HOXA4_Q2, V$CEBP_Q3, V$HMGIY_Q6, V$STAT5A_03, V$STAT4_01, V$CEBPA_01, V$NFAT_Q4_01, V$STAT5A_04, V$CEBP_Q2_01, V$STAT1_03

Corresponding mean of effect sizes of each TF:
(In the same order as above)
1.54, 1.45, 1.43, 1.27, 1.25, 1.21, 1.21, 1.20, 1.12, 1.12, 1.11, 0.83, 0.83, 0.79, 0.76

Corresponding total number of TFBS for each TF (genome-wide):
(In the same order as above)
732, 730, 741, 719, 718, 719, 744, 753, 749, 743, 711, 736, 740, 716, 751

Corresponding total number of TFBS for each TF (in all genes in selected Gene ontology terms):
(In the same order as above)
419, 414, 430, 432, 391, 385, 415, 398, 393, 401, 378, 385, 356, 344, 361

Corresponding number of genes (of selected Gene ontology terms) each TF is involved with:
(In the same order as above)
148, 160, 159, 154, 149, 153, 150, 149, 150, 153, 146, 144, 142, 135, 152

Corresponding number of selected Gene ontology terms each TF is involved with:
(In the same order as above)
17, 17, 17, 17, 17, 17, 17, 17, 17, 17, 17, 17, 17, 17, 17

Cluster for columns 416 to 422, rows 139 to 144

Gene ontology terms

mast cell activation | mast cell degranulation | leukotriene biosynthetic process | icosanoid metabolic process | arachidonic acid metabolic process | cyclooxygenase pathway | lipoxygenase pathway

TFs

V$SMAD4_Q6 | F$STRE_01 | V$SREBP1_02 | V$MYOGNF1_01 | V$PEA3_Q6 | V$ETS_Q6

Information

All related TFs:
(List of all TFs that are related to any of the PWMs)
ELF-1, ELFR, Elf-1, Elk-1, Elk-1-isoform1, Erg-1, Erg-2, Ets-1, Fli-1, GABP-alpha, GABP-alpha:GABP-beta, GABP-beta1, GABP-beta2, NERF-1a, NERF-1b, NERF-2, NF-1, NF-1/L, NF-1/Red1, NF-1A1, NF-1B1, NF-1B2, NF-1C2, NF-1X, Net, PEA3, PU.1, PU.1-xbb1, SAP-1a, SAP-1b, SREBP-1, SREBP-1a, SREBP-1b, SREBP-1c, Smad4, Spi-B, TCF, TEL1, Tel-2a, Tel-2b, Tel-2c, Tel-2d, Tel-2e, Tel-2f, c-Ets-1, c-Ets-1A, c-Ets-1B, c-Ets-2, c-Ets-2A, c-Ets-2B, p38erg, p49erg, p55, p55erg

Ranked gene list:
(All genes of the selected Gene ontology terms with hits of any of the selected TFs, ranked according to the total number of TFBS)
TNF, LTB, LTC4S, LTA, PTGDS, PLA2G6, VAV1, PGF, ALOX5, SH3BP2, PTPN6, BGN, AKT1, LMNA, ACVRL1, PTGS1, MAPK14, LCP2, ITGB2, GUSB, F2, EGR1, CYSLTR1, PSMP, PLCG1, MARK2, IRF6, INPP5D, IL3, IL13, DOK2, AHSA1, UCN, TNFSF13, SRC, PGD, MTHFR, LTB4R, KIT, IL4, FYN, ERAF, BDKRB2, PIK3CD, PAF1, MMP2, LTB4R2, LAT2, HRH2, GPX4, FGFR3, EPHB2, CERK, B3GAT1, AVP, ADORA3

Corresponding total number of TFBS:
(For each gene listed above, the total number of TFBS for any of the selected TFs, multiplied by the number of selected Gene ontology terms containing that gene)
30, 30, 18, 15, 14, 12, 8, 8, 7, 6, 6, 6, 6, 5, 5, 4, 4, 4, 4, 4, 4, 4, 4, 3, 3, 3, 3, 3, 3, 3, 3, 3, 2, 2, 2, 2, 2, 2, 2, 2, 2, 2, 2, 1, 1, 1, 1, 1, 1, 1, 1, 1, 1, 1, 1, 1

Corresponding number of selected Gene ontology terms each gene is involved with:
(In the same order as above)
5, 5, 6, 3, 7, 6, 2, 4, 7, 1, 1, 2, 1, 5, 5, 4, 4, 2, 1, 4, 4, 1, 4, 1, 1, 1, 3, 1, 3, 3, 1, 3, 2, 1, 2, 2, 2, 2, 2, 2, 2, 2, 2, 1, 1, 1, 1, 1, 1, 1, 1, 1, 1, 1, 1, 1

Corresponding number of selected TFs each gene is involved with:
(In the same order as above)
6, 6, 3, 5, 2, 2, 4, 2, 1, 6, 6, 3, 6, 1, 1, 1, 1, 2, 4, 1, 1, 4, 1, 3, 3, 3, 1, 3, 1, 1, 3, 1, 1, 2, 1, 1, 1, 1, 1, 1, 1, 1, 1, 1, 1, 1, 1, 1, 1, 1, 1, 1, 1, 1, 1, 1

Gene ontology terms ranked according to mean of effect sizes in cluster:
cyclooxygenase pathway | arachidonic acid metabolic process | mast cell degranulation | lipoxygenase pathway | icosanoid metabolic process | mast cell activation | leukotriene biosynthetic process

Corresponding mean of effect sizes of each Gene ontology term:
(In the same order as above)
2.45, 1.95, 1.91, 1.90, 1.85, 1.85, 1.74

Corresponding total number of genes of each Gene ontology term:
(In the same order as above)
42, 88, 90, 43, 24, 91, 50

Corresponding number of genes of each Gene ontology term with at least one TFBS (of selected TFs):
(In the same order as above)
18, 23, 26, 15, 9, 27, 17

Corresponding total number of TFBS (of selected TFs) for each Gene ontology term (in all genes):
(In the same order as above)
35, 46, 44, 29, 19, 62, 35

Corresponding number of selected TFs each Gene ontology term is involved with:
(In the same order as above)
6, 6, 6, 6, 6, 6, 6

TFs ranked according to mean of effect sizes in cluster:
V$SREBP1_02, F$STRE_01, V$MYOGNF1_01, V$ETS_Q6, V$SMAD4_Q6, V$PEA3_Q6

Corresponding mean of effect sizes of each TF:
(In the same order as above)
2.22, 2.09, 2.06, 1.97, 1.86, 1.50

Corresponding total number of TFBS for each TF (genome-wide):
(In the same order as above)
693, 651, 654, 721, 666, 717

Corresponding total number of TFBS for each TF (in all genes in selected Gene ontology terms):
(In the same order as above)
51, 43, 47, 46, 42, 41

Corresponding number of genes (of selected Gene ontology terms) each TF is involved with:
(In the same order as above)
21, 17, 21, 19, 17, 18

Corresponding number of selected Gene ontology terms each TF is involved with:
(In the same order as above)
7, 7, 7, 7, 7, 7

Cluster for columns 906 to 915, rows 400 to 408

Gene ontology terms

isoprenoid biosynthetic process | protein-glutamine gamma-glutamyltransferase activity | binding | methionine biosynthetic process | oocyte growth | oocyte growth in germarium-derived egg chamber | phospholipid metabolic process | polysaccharide biosynthetic process | phosphatidylcholine biosynthetic process | phospholipid biosynthetic process

TFs

V$PPAR_DR1_Q2 | V$DR1_Q3 | V$HNF4_DR1_Q3 | V$COUP_DR1_Q6 | V$COUP_01 | V$HNF4_01 | V$HNF4_Q6_01 | V$HNF4ALPHA_Q6 | V$COUPTF_Q6

Information

All related TFs:
(List of all TFs that are related to any of the PWMs)
COUP, COUP-TF1, COUP-TF2, HNF-4, HNF-4alpha, HNF-4alpha1, HNF-4alpha2, HNF-4alpha3, HNF-4alpha4, HNF-4alpha7, HNF-4gamma, PPAR-alpha, PPAR-alpha:RXR-alpha, PPAR-beta, PPAR-gamma, PPAR-gamma1, PPAR-gamma2, PPAR-gamma2:RXR-alpha, PPAR-gamma:RXR-alpha, RAR-alpha:RXR-alpha

Ranked gene list:
(All genes of the selected Gene ontology terms with hits of any of the selected TFs, ranked according to the total number of TFBS)

CTSD, SLC25A1, RHOA, MDK, SSSCA1, ZP3, POU5F1, HSD17B8, CHKB, MMP11, ISYNA1, CSNK2B, GALE, DGAT1, PISD, CALM3, FASN, CDIPT, APEH, TCF7, RXRB, RPS6KA1, RGS19, RBKS, RASGRP2, PNPLA2, PLEC1, PGP, PFN1, OPRL1, MC1R, MAP2K7, LTBP4, KIAA0664, JUND, IL2RB, GIT1, CSK, CRIP1, CD151, BRE, BCL9L, APOC3, APOA1, ZYX, ZBTB7B, VEGFA, UCN2, TRAF4, SYTL1, SLC25A22, RING1, RGS14, POLD4, PDPK1, NOXO1, NISCH, METTL1, MEF2D, MAP3K11, MAFG, GYLTL1B, GOLGA4, GBF1, FUT7, FLJ32987, ESRRA, DNMT3A, DDX41, CYP1A1, CSF1R, CRABP2, CORO1A, CDK2, CBX4, TPI1, SMPD2, SILV, SHBG, SH3BP2, SH3BP1, SCRIB, PSD, PRKCSH, PPP1R10, PPARA, PCP2, NPDC1, MYBBP1A, KCNH2, KAT2A, GMNN, GALK1, ELOVL1, EFNA2, DVL1, DNAJA2, CHRNA4, CFB, CDC34, CCDC88B, TFEB, TAPBP, SHMT2, SFTPC, SFRS6, RPL32, PSMC5, PRKCA, PNMT, PLXNB1, OGFR, NCF4, MAFK, LPIN1, LMAN2, KCNH4, IL23A, GNAI2, GCK, EMG1, ELF3, EDC4, E4F1, CTRL, CISH, CIC, CDK4, CDC20, CD248, C19orf26, ABHD14B, TUSC2, TNK2, TNFRSF1B, TNFRSF12A, THRA, SLC2A4RG, RGS3, PAX6, PARD6A, MYO1C, MPG, MAPRE1, LRP1, IRF8, IGFBP6, FGFR4, CRIP2, CFL1, CD63, BGN, AXIN1, ARRB2, AGMAT, VAMP8, TPM2, TAP2, SIRT2, SEMA3B, SDC3, RIN1, RBMS1, RARG, RALGDS, PTBP2, PSENEN, PRKCG, PNPLA6, PLA2G1B, PDLIM7, PDK3, NR4A1, NR2F6, NFKBIB, NECAB3, MGC138368, MGAT1, MAZ, LTB4R, LDB1, JUNB, ITGB7, INTU, IL2RG, HSPE1, HSPD1, HNF1B, GYPC, FOSL2, FLNC, F2RL3, EARS2, DLG4, DAXX, CLCF1, ARHGEF2, ANG, AKT1, ADORA2A, ADM, ADAT2, ADAM8, VASP, UBTF, UAP1, TSPO, TNFRSF4, TIMM44, TAP1, SQSTM1, SP2, SOCS3, SOCS2, SNRPA, SNRNP40, SMAD7, SLURP1, SLC2A1, RASGRF1, PTPN6, PTP4A3, PTGER1, PLCG1, PARD3B, OSM, NPFF, NOS3, NFE2L2, NCOA2, NAT6, MTRR, MBD2, MAPK8IP1, MAP2K2, MADCAM1, KLF6, KHSRP, KCNJ5, IRF3, ILVBL, IGF2R, HRH3, HMGCS1, GPI, GAL, FLII, FGR, E2F4, DECR1, CYP11A1, CUGBP1, CTDSP2, CSTB, CSNK1A1, CPT1A, CDKN1A, BAX, ATF2, ANAPC2, ALDOA, ADRBK1, ACY1, ZFPM1, WIPF1, TRIM28, TRADD, TPM3, TNKS1BP1, TNF, SUMO1, STK24, STAT5A, SREBF1, SRC, SP7, SMAD3, SLC9A3R1, SKI, SH2B3, RIT1, RARA, PYY, PRDX6, PRDX2, PLD2, PLCD1, PITX1, PICK1, PDGFA, PCSK7, PBX2, OPRS1, OBSCN, NR6A1, NR2F1, NHP2L1, NFIA, NDUFAB1, NCOR2, MUC1, MRPL28, MNT, MARK2, LTA, ITGB2, IRF9, INSR, ILK, ID1, HRAS, HNF1A, HBEGF, HAMP, GRIN2D, GPR132, GLP1R, GFI1, GALT, GADD45A, FSTL3, FRS2, FKBP5, FGF4, ERF, ERBB2, EPHB2, EMD, EGR1, DNAJC14, DNAI1, DLX2, DDX21, DDB1, CTSB, CPZ, CLCA2, CDK5, CD2BP2, CASP9, C16orf38, AKT1S1, AGFG1, ACTB, ACTA1, ACSL1, ACHE, ZNF148, ZBTB33, ZAP70, WDR1, UTS2, USF2, UNC13A, UBE4A, UBE2M, U2AF2, TST, TSC2, TRIM63, TREH, TRAF5, TPM1, TP73, TP53, TNFRSF25, TNFRSF1A, TMEM201, TM2D1, TLR2, TCF7L2, TAL1, SULT2B1, STX6, STMN1, STAT6, STAT3, STARD3, SSR1, SP6, SNX1, SNRPG, SNAI1, SLC9A3R2, SLC7A7, SLC5A1, SLC36A1, SKP1, SHC1, SFRS1, SF4, SF3B2, SEMA3F, SELPLG, SCP2, SCGB1A1, SCD, SAR1A, S100A9, S100A6, S100A4, RXRA, RTN4R, RPS8, RPS27A, ROBLD3, RGS4, RFX1, RERE, REPIN1, RENBP, RBM47, RASA3, RAMP2, PURA, PTRH1, PSME2, PSME1, POU3F3, POU2F2, PLD1, PIN1, PIK3R2, PIK3CG, PIAS1, PGLYRP1, PFKP, PELP1, PDGFRB, PDCD6, PDCD4, PCSK4, PCDH8, PAPSS1, ORC5L, NUP43, NTRK1, NR5A2, NR1H2, NQO2, NPTN, NME2, NID2, NGFR, NFYA, NFATC1, NEDD9, NDOR1, MXI1, MXD3, MST1, MSL2, MRPL41, MPI, MFI2, MFGE8, MBNL1, MAPT, MAPK1, MAL, MAFA, MAF, MAD1L1, M6PR, LTB, LPIN3, LMNA, LGALS9, LCN1, LARS2, LAMP1, KEAP1, KCNIP3, JAG2, ITIH4, ITGAL, ITGA3, IRF2, IPO13, ING2, IL4R, IL1B, IL18BP, ICAM2, HSPA5, HSPA4, HRH1, HPCA, HOXA5, HNRNPL, HNRNPC, GZMM, GUCA2A, GRM2, GPSM1, GP2, GNRH2, GLI1, GFM1, GCKR, GCC2, GATA4, GAPDH, GALNS, FUBP3, FOXO1, FLAD1, FBLIM1, F2R, EZR, EXOC1, ERBB2IP, EPO, ELAVL1, EIF4G1, EGR4, EFEMP2, E2F3, DUSP2, DSC3, DLX4, DLL1, DKFZp434P0672, DCTN3, CYTH1, CYC1, CRHR1, COX17, COPG, CLTB, CKS1B, CHPF, CHMP4B, CDX1, CDKN2C, CD82, CD72, CD5, CD4, CARM1, CAPG, CAND1, BRD2, BNIP2, BCL2L11, BAK1, B4GALT1, ATF4, ARHGAP1, ARF1, ANXA9, ANXA2, AMT, ALPI, AGRN, ADRM1, ADORA3, ACSL5

Corresponding total number of TFBS:
(For each gene listed above, the total number of TFBS for any of the selected TFs, multiplied by the number of selected Gene ontology terms containing that gene)
40, 30, 27, 27, 21, 18, 18, 18, 18, 16, 15, 15, 14, 14, 12, 12, 10, 10, 10, 9, 9, 9, 9, 9, 9, 9, 9, 9, 9, 9, 9, 9, 9, 9, 9, 9, 9, 9, 9, 9, 9, 9, 9, 9, 8, 8, 8, 8, 8, 8, 8, 8, 8, 8, 8, 8, 8, 8, 8, 8, 8, 8, 8, 8, 8, 8, 8, 8, 8, 8, 8, 8, 8, 8, 7, 7, 7, 7, 7, 7, 7, 7, 7, 7, 7, 7, 7, 7, 7, 7, 7, 7, 7, 7, 7, 7, 7, 7, 7, 7, 7, 6, 6, 6, 6, 6, 6, 6, 6, 6, 6, 6, 6, 6, 6, 6, 6, 6, 6, 6, 6, 6, 6, 6, 6, 6, 6, 6, 6, 6, 6, 5, 5, 5, 5, 5, 5, 5, 5, 5, 5, 5, 5, 5, 5, 5, 5, 5, 5, 5, 5, 5, 5, 5, 4, 4, 4, 4,

4, 4, 4, 4, 4, 4, 4, 4, 4, 4, 4, 4, 4, 4, 4, 4, 4, 4, 4, 4, 4, 4, 4, 4, 4, 4, 4, 4, 4, 4, 4, 4, 4, 4, 4, 4, 4, 4, 4, 4, 4, 4, 4, 4, 3, 3, 3, 3, 3, 3, 3, 3, 3, 3, 3, 3, 3, 3, 3, 3, 3, 3, 3, 3, 3, 3, 3, 3, 3, 3, 3, 3, 3, 3, 3, 3, 3, 3, 3, 3, 3, 3, 3, 3, 3, 3, 3, 3, 3, 3, 3, 3, 3, 3, 3, 3, 3, 3, 3, 3, 3, 3, 2, 2, 2, 2, 2, 2, 2, 2, 2, 2, 2, 2, 2, 2, 2, 2, 2, 2, 2, 2, 2, 2, 2, 2, 2, 2, 2, 2, 2, 2, 2, 2, 2, 2, 2, 2, 2, 2, 2, 2, 2, 2, 2, 2, 2, 2, 2, 2, 2, 2, 2, 2, 2, 2, 2, 2, 2, 2, 2, 2, 2, 2, 2, 2, 2, 2, 2, 2, 2, 2, 2, 2, 2, 2, 2, 2, 2, 2, 2, 2, 2, 2, 1, 1

Corresponding number of selected Gene ontology terms each gene is involved with:
(In the same order as above)
5, 5, 3, 3, 3, 3, 3, 2, 3, 2, 3, 3, 2, 2, 4, 4, 2, 2, 2, 1, 1, 1, 1, 1, 1, 1, 1, 1, 1, 1, 1, 1, 1, 1, 1, 1, 1, 1, 1, 1, 1, 1, 1, 1, 1, 1, 1, 1, 1, 1, 1, 1, 1, 1, 1, 1, 1, 1, 1, 1, 1, 1, 1, 1, 1, 1, 1, 2, 1, 1, 1, 1, 1, 1, 1, 1, 1, 1, 1, 1, 1, 1, 1, 1, 1, 1, 1, 1, 1, 1, 1, 1, 1, 1, 1, 1, 1, 1, 1, 1, 1, 1, 1, 2, 2, 1, 1, 1, 2, 1, 1, 1, 1, 1, 1, 1, 1, 1, 1, 1, 1, 1, 1, 1, 1, 1, 1, 1, 1, 1, 1, 1, 1, 1, 1, 1, 1, 1, 1, 1, 1, 1, 1, 1, 1, 1, 1, 1, 1, 1, 1, 1, 1, 1, 1, 1, 1, 1, 1, 1, 1, 1, 1, 1, 1, 1, 1, 1, 1, 4, 1, 1, 1, 1, 1, 1, 1, 1, 1, 1, 1, 1, 1, 1, 1, 1, 1, 1, 2, 1, 1, 1, 1, 1, 1, 1, 1, 1, 1, 1, 1, 2, 1, 1, 3, 1, 1, 1, 1, 1, 1, 1, 1, 1, 1, 1, 1, 1, 3, 1, 1, 1, 1, 1, 1, 1, 1, 1, 1, 1, 1, 1, 1, 1, 1, 1, 1, 1, 1, 1, 1, 3, 1, 1, 1, 1, 1, 1, 1, 3, 3, 1, 1, 1, 1, 3, 1, 1, 1, 1, 1, 1, 1, 1, 1, 1, 1, 1, 1, 1, 1, 1, 1, 2, 1, 2, 1, 1, 1, 1, 1, 1, 1, 2, 1, 2, 1, 1, 1, 1, 1, 1, 1, 1, 1, 1, 1, 1, 1, 1, 1, 1, 1, 1, 1, 1, 1, 1, 1, 1, 2, 1, 1, 2, 1, 1, 1, 1, 1, 1, 1, 1, 1, 1, 1, 1, 1, 1, 1, 1, 1, 1, 1, 1, 2, 1, 1, 1, 1, 1, 1, 1, 1, 1, 2, 1

Corresponding number of selected TFs each gene is involved with:
(In the same order as above)
8, 6, 9, 9, 7, 6, 6, 9, 6, 8, 5, 5, 7, 7, 3, 3, 5, 5, 5, 9, 9, 9, 9, 9, 9, 9, 9, 9, 9, 9, 9, 9, 9, 9, 9, 9, 9, 9, 9, 9, 9, 9, 9, 9, 8, 8, 8, 8, 8, 8, 8, 8, 8, 8, 8, 8, 8, 8, 8, 8, 8, 8, 8, 8, 8, 8, 8, 4, 8, 8, 8, 8, 8, 8, 8, 7, 7, 7, 7, 7, 7, 7, 7, 7, 7, 7, 7, 7, 7, 7, 7, 7, 7, 7, 7, 7, 7, 7, 7, 7, 7, 6, 6, 3, 3, 6, 6, 6, 3, 6, 6, 6, 6, 6, 6, 6, 6, 6, 6, 6, 6, 6, 6, 6, 6, 6, 6, 6, 6, 6, 6, 6, 6, 5, 5, 5, 5, 5, 5, 5, 5, 5, 5, 5, 5, 5, 5, 5, 5, 5, 5, 5, 5, 5, 5, 5, 4, 4, 4, 4, 4, 4, 4, 4, 4, 4, 4, 4, 4, 4, 1, 4, 4, 4, 4, 4, 4, 4, 4, 4, 4, 4, 4, 4, 4, 4, 4, 4, 4, 2, 4, 4, 4, 4, 4, 4, 4, 4, 4, 4, 4, 4, 2, 4, 3, 1, 3, 3, 3, 3, 3, 3, 3, 3, 3, 3, 3, 3, 3, 1, 3, 3, 3, 3, 3, 3, 3, 3, 3, 3, 3, 3, 3, 3, 3, 3, 3, 3, 3, 3, 3, 3, 1, 3, 3, 3, 3, 3, 3, 3, 1, 1, 3, 3, 3, 3, 1, 3, 3, 3, 3, 3, 3, 3, 2, 2, 2, 2, 2, 2, 2, 2, 2, 2, 1, 2, 1, 2, 2, 2, 2, 2, 2, 2, 1, 2, 1, 2, 2, 2, 2, 2, 2, 2, 2, 2, 2, 2, 2, 2, 2, 2, 2, 2, 2, 2, 2, 2, 2, 2, 2, 1, 2, 2, 1, 2, 2, 2, 2, 2, 2, 2, 2, 2, 2, 2, 2, 2, 2, 2, 2, 2, 2, 2, 2, 1, 2, 2, 2, 2, 2, 2, 2, 2, 2, 1, 2, 2, 1

Gene ontology terms ranked according to mean of effect sizes in cluster:
phospholipid metabolic process | protein-glutamine gamma-glutamyltransferase activity | binding | phosphatidylcholine biosynthetic process | phospholipid biosynthetic process | oocyte growth in germarium-derived egg chamber | oocyte growth | polysaccharide biosynthetic process | isoprenoid biosynthetic process | methionine biosynthetic process

Corresponding mean of effect sizes of each Gene ontology term:
(In the same order as above)
1.62, 1.49, 1.46, 1.44, 1.37, 1.12, 1.12, 1.09, 0.85, 0.72

Corresponding total number of genes of each Gene ontology term:

(In the same order as above)
44, 39, 3.2K, 45, 88, 61, 61, 41, 35, 23

Corresponding number of genes of each Gene ontology term with at least one TFBS (of selected TFs):
(In the same order as above)
13, 6, 529, 13, 22, 11, 11, 9, 6, 4

Corresponding total number of TFBS (of selected TFs) for each Gene ontology term (in all genes):
(In the same order as above)
38, 38, 1673, 48, 76, 48, 48, 36, 29, 18

Corresponding number of selected TFs each Gene ontology term is involved with:
(In the same order as above)
9, 9, 9, 9, 9, 9, 9, 9, 9, 8

TFs ranked according to mean of effect sizes in cluster:
V$PPAR_DR1_Q2, V$HNF4_DR1_Q3, V$HNF4_01, V$COUP_01, V$HNF4_Q6_01, V$COUP_DR1_Q6, V$DR1_Q3, V$COUPTF_Q6, V$HNF4ALPHA_Q6

Corresponding mean of effect sizes of each TF:
(In the same order as above)
1.63, 1.52, 1.46, 1.30, 1.27, 1.16, 1.11, 1.10, 0.52

Corresponding total number of TFBS for each TF (genome-wide):
(In the same order as above)
684, 666, 674, 660, 687, 651, 667, 696, 688

Corresponding total number of TFBS for each TF (in all genes in selected Gene ontology terms):
(In the same order as above)
243, 233, 222, 226, 233, 218, 227, 219, 231

Corresponding number of genes (of selected Gene ontology terms) each TF is involved with:
(In the same order as above)
206, 203, 190, 195, 203, 192, 199, 190, 208

Corresponding number of selected Gene ontology terms each TF is involved with:
(In the same order as above)
10, 10, 10, 10, 10, 10, 10, 9, 10

Cluster for columns 670 to 677, rows 423 to 428

Gene ontology terms

ubiquitin-dependent protein catabolic process | cellular homeostasis | DNA catabolic process | autophagic cell death | response to ionizing radiation | protein stabilization | regulation of smooth muscle cell proliferation | response to radiation

TFs

V$E2F_Q6_01 | V$E2F_Q4_01 | V$E2F_Q3_01 | V$E2F1_Q4_01 | V$E2F1_Q6_01 | V$E2F_03

Information

All related TFs:
(List of all TFs that are related to any of the PWMs)
DP-1, E2F, E2F+E4, E2F-1, E2F-1:DP-1, E2F-3a, E2F-4, E2F-7

Ranked gene list:
(All genes of the selected Gene ontology terms with hits of any of the selected TFs, ranked according to the total number of TFBS)
JUN, CDKN1A, AKT1, H2AFX, CDKN1B, TP53, CCND1, TP73, PCNA, MDM2, FASN, BACH1, ANXA5, MYB, MARCKSL1, GEMIN4, CDT1, CDC25B, CAD, UBB, SKP2, RRAS, GMNN, FBXO5, EXO1, CUX1, BCL2L1, SFRS2, SFRS1, POLA1, PIK3CA, HSPA2, PSMC5, PRKDC, CDC25A, UNG, ULK1, SOCS1, RAD51, RAC1, NPM1, HSPD1, HMOX2, EGR1, DUSP1, CDK5R1, CCNE1, CBX7, TMEM11, SMC1A, RBX1, NOLC1, MLH1, MDC1, ING1, HSP90B1, DMXL2, CUL1, CHEK1, CDC25C, CASP7

Corresponding total number of TFBS:
(For each gene listed above, the total number of TFBS for any of the selected TFs, multiplied by the number of selected Gene ontology terms containing that gene)
40, 36, 25, 24, 24, 21, 18, 12, 12, 12, 10, 10, 8, 6, 6, 6, 6, 6, 6, 5, 5, 5, 5, 5, 5, 5, 5, 4, 4, 4, 4, 4, 3, 3, 3, 2, 2, 2, 2, 2, 2, 2, 2, 2, 2, 2, 2, 2, 1, 1, 1, 1, 1, 1, 1, 1, 1, 1, 1, 1, 1

Corresponding number of selected Gene ontology terms each gene is involved with:
(In the same order as above)
8, 6, 5, 4, 4, 7, 3, 2, 2, 3, 2, 2, 2, 1, 1, 1, 1, 1, 1, 5, 1, 1, 1, 1, 1, 1, 5, 1, 1, 1, 2, 1, 1, 3, 1, 1, 1, 1, 2, 1, 1, 1, 1, 1, 1, 1, 1, 1, 1, 1, 1, 1, 1, 1, 1, 1, 1, 1, 1, 1, 1

Corresponding number of selected TFs each gene is involved with:
(In the same order as above)
5, 6, 5, 6, 6, 3, 6, 6, 6, 4, 5, 5, 4, 6, 6, 6, 6, 6, 6, 1, 5, 5, 5, 5, 5, 5, 1, 4, 4, 4, 2, 4, 3, 1, 3, 2, 2, 2, 1, 2, 2, 2, 2, 2, 2, 2, 2, 2, 1, 1, 1, 1, 1, 1, 1, 1, 1, 1, 1, 1, 1

Gene ontology terms ranked according to mean of effect sizes in cluster:
regulation of smooth muscle cell proliferation | response to radiation | cellular homeostasis | protein stabilization | DNA catabolic process | response to ionizing radiation | ubiquitin-dependent protein catabolic process | autophagic cell death

Corresponding mean of effect sizes of each Gene ontology term:
(In the same order as above)
2.71, 2.65, 1.90, 1.75, 1.47, 1.40, 1.23, 0.95

Corresponding total number of genes of each Gene ontology term:
(In the same order as above)
27, 41, 91, 37, 71, 94, 89, 79

Corresponding number of genes of each Gene ontology term with at least one TFBS (of selected TFs):
(In the same order as above)
6, 12, 18, 8, 13, 21, 18, 14

Corresponding total number of TFBS (of selected TFs) for each Gene ontology term (in all genes):
(In the same order as above)
35, 49, 60, 35, 45, 62, 65, 45

Corresponding number of selected TFs each Gene ontology term is involved with:
(In the same order as above)

6, 6, 6, 6, 6, 6, 6, 6

TFs ranked according to mean of effect sizes in cluster:
V$E2F_Q6_01, V$E2F_Q4_01, V$E2F_03, V$E2F1_Q4_01, V$E2F_Q3_01, V$E2F1_Q6_01

Corresponding mean of effect sizes of each TF:
(In the same order as above)
2.50, 2.12, 1.70, 1.58, 1.45, 1.20

Corresponding total number of TFBS for each TF (genome-wide):
(In the same order as above)
686, 667, 688, 681, 690, 700

Corresponding total number of TFBS for each TF (in all genes in selected Gene ontology terms):
(In the same order as above)
81, 69, 68, 61, 59, 58

Corresponding number of genes (of selected Gene ontology terms) each TF is involved with:
(In the same order as above)
38, 32, 37, 30, 29, 32

Corresponding number of selected Gene ontology terms each TF is involved with:
(In the same order as above)
8, 8, 8, 8, 8, 8

Cluster for columns 820 to 830, rows 342 to 345

Gene ontology terms

angiogenesis | vascular endothelial growth factor production | endothelial cell migration | regulation of angiogenesis | lymphangiogenesis | vascular endothelial growth factor receptor activity | vascular endothelial growth factor receptor binding | endothelial cell proliferation | regulation of vascular endothelial growth factor production | negative regulation of transferase activity | skeletal muscle regeneration

TFs

V$RP58_01 | V$FREAC3_01 | V$EGR3_01 | V$EGR2_01

Information

All related TFs:
(List of all TFs that are related to any of the PWMs)
Egr-2, Egr-3, FOXC1, RP58

Ranked gene list:
(All genes of the selected Gene ontology terms with hits of any of the selected TFs, ranked according to the total number of TFBS)
VEGFA, AKT1, MKI67, FGFR1, THBS1, TM7SF2, TNF, TYMP, PDGFB, HGF, GGPS1, FGF2, CD44, PLAU, HIF1A, CD34, ANGPT2, VTN, SCARB2, PDGFA, ID2, HRAS, EGR1, CDH5, TNFSF15, TNFSF13, MMP2, IL8, TIE1, TGFB1, SSSCA1, PROX1, PLAUR, PIK3CA, PIGF, NODAL, LIF, FAM126A, EFNA1, CYR61, ANXA5, SLC35A1, SERPINE1, RAF1, PSMP, JUN, SPARC, SMAD2, SEMA3B, RNH1, RAC1, PTGES, PIK3R2, HDAC7, EPAS1, CXCL3, BNIP3, SP4, SOX18, SEMA3G,

SDC4, S100A4, PROK1, MMP14, MGC39830, IL6ST, HSP90AA1, GIPC1, F3, ETS1, EPHA1, DCN, CDKN1B, WNT5A, WDHD1, UBASH3B, TRADD, TP73, TNFSF11, TGFBR2, TFAP2A, STAT1, SMAD5, SMAD3, SHB, RYK, RTN4R, RPS6KA1, RHOC, PTGER4, PLCG1, MARK2, KRAS, JUNB, ISYNA1, ING1, IGF2, HOXD10, HMGCR, HIF1AN, GDF2, FH, FGF6, FAS, EPHB4, EPHB1, CXCL12, CTSD, CSF1, CD46, CD248, BAI2, ATF3, AREG, AQP1, AKT3, XPC, VCL, TRAF1, TNXB, TNFSF12, TGFB3, TCEB2, STAB1, S1PR2, S100A9, S100A13, RALA, PTPN11, PRKCZ, PPP1R15A, PGP, PCSK5, NR2F2, NOL3, MYOM2, MFI2, MDM2, MAPK9, MAPK7, MAP2K5, KLK1, KLF5, IL13, HEY2, GRLF1, FLII, FLI1, FGF10, ELF2, EIF4EBP1, CRP, CLIP1, CITED2, CDH2, CCM2, C20orf85, BRAF, BGLAP, AXL, ANPEP, AKT2, ABCB1

Corresponding total number of TFBS:
(For each gene listed above, the total number of TFBS for any of the selected TFs, multiplied by the number of selected Gene ontology terms containing that gene)
33, 30, 24, 21, 18, 16, 14, 12, 10, 10, 10, 10, 10, 9, 9, 9, 9, 8, 8, 8, 8, 8, 8, 8, 7, 7, 7, 7, 6, 6, 6, 6, 6, 6, 6, 6, 6, 6, 6, 6, 6, 5, 5, 5, 5, 5, 4, 4, 4, 4, 4, 4, 4, 4, 4, 4, 4, 3, 3, 3, 3, 3, 3, 3, 3, 3, 3, 3, 3, 3, 3, 3, 3, 2, 2, 2, 2, 2, 2, 2, 2, 2, 2, 2, 2, 2, 2, 2, 2, 2, 2, 2, 2, 2, 2, 2, 2, 2, 2, 2, 2, 2, 2, 2, 2, 2, 2, 2, 2, 2, 1, 1, 1, 1, 1, 1, 1, 1, 1, 1, 1, 1, 1, 1, 1, 1, 1, 1, 1, 1, 1, 1, 1, 1, 1, 1, 1, 1, 1, 1, 1, 1, 1, 1, 1, 1, 1, 1, 1, 1, 1, 1, 1, 1

Corresponding number of selected Gene ontology terms each gene is involved with:
(In the same order as above)
11, 10, 6, 7, 9, 8, 7, 6, 5, 10, 5, 10, 5, 9, 9, 9, 9, 4, 4, 4, 2, 4, 4, 8, 7, 7, 7, 7, 6, 3, 6, 6, 6, 6, 3, 3, 6, 3, 3, 3, 3, 5, 5, 5, 5, 5, 4, 2, 2, 2, 4, 2, 2, 2, 4, 2, 2, 3, 3, 1, 3, 1, 3, 3, 1, 1, 3, 3, 3, 3, 3, 3, 3, 2, 1, 1, 1, 1, 1, 2, 2, 1, 2, 2, 2, 1, 1, 1, 1, 2, 1, 2, 1, 1, 2, 1, 2, 1, 2, 2, 1, 1, 2, 2, 2, 1, 2, 1, 2, 1, 1, 2, 1, 2, 2, 1, 1, 1, 1, 1, 1, 1, 1, 1, 1, 1, 1, 1, 1, 1, 1, 1, 1, 1, 1, 1, 1, 1, 1, 1, 1, 1, 1, 1, 1, 1, 1, 1, 1, 1, 1, 1, 1, 1, 1, 1

Corresponding number of selected TFs each gene is involved with:
(In the same order as above)
3, 3, 4, 3, 2, 2, 2, 2, 2, 1, 2, 1, 2, 1, 1, 1, 1, 2, 2, 2, 4, 2, 2, 1, 1, 1, 1, 1, 1, 2, 1, 1, 1, 1, 2, 2, 1, 2, 2, 2, 2, 1, 1, 1, 1, 1, 1, 2, 2, 2, 1, 2, 2, 2, 1, 2, 2, 1, 1, 3, 1, 3, 1, 1, 3, 3, 1, 1, 1, 1, 1, 1, 1, 1, 2, 2, 2, 2, 2, 1, 1, 2, 1, 1, 1, 2, 2, 2, 2, 1, 2, 1, 2, 2, 1, 2, 1, 2, 1, 1, 2, 2, 1, 1, 1, 2, 1, 2, 1, 2, 2, 1, 2, 1, 1, 2, 1, 1, 1, 1, 1, 1, 1, 1, 1, 1, 1, 1, 1, 1, 1, 1, 1, 1, 1, 1, 1, 1, 1, 1, 1, 1, 1, 1, 1, 1, 1, 1, 1, 1, 1, 1, 1, 1, 1, 1

Gene ontology terms ranked according to mean of effect sizes in cluster:
regulation of angiogenesis | endothelial cell migration | lymphangiogenesis | vascular endothelial growth factor receptor binding | vascular endothelial growth factor receptor activity | endothelial cell proliferation | regulation of vascular endothelial growth factor production | vascular endothelial growth factor production | negative regulation of transferase activity | angiogenesis | skeletal muscle regeneration

Corresponding mean of effect sizes of each Gene ontology term:
(In the same order as above)
2.26, 2.19, 2.10, 1.60, 1.54, 1.36, 1.17, 1.00, 0.93, 0.71, 0.36

Corresponding total number of genes of each Gene ontology term:
(In the same order as above)
86, 168, 66, 92, 89, 161, 71, 515, 60, 678, 48

Corresponding number of genes of each Gene ontology term with at least one TFBS (of selected TFs):
(In the same order as above)
25, 41, 22, 26, 25, 38, 21, 102, 14, 128, 11

Corresponding total number of TFBS (of selected TFs) for each Gene ontology term (in all genes):
(In the same order as above)
36, 61, 34, 40, 39, 59, 30, 147, 23, 183, 14

Corresponding number of selected TFs each Gene ontology term is involved with:

(In the same order as above)
4, 4, 4, 4, 4, 4, 4, 4, 4, 4, 4

TFs ranked according to mean of effect sizes in cluster:
V$RP58_01, V$EGR3_01, V$EGR2_01, V$FREAC3_01

Corresponding mean of effect sizes of each TF:
(In the same order as above)
2.02, 1.42, 1.35, 0.74

Corresponding total number of TFBS for each TF (genome-wide):
(In the same order as above)
675, 627, 637, 710

Corresponding total number of TFBS for each TF (in all genes in selected Gene ontology terms):
(In the same order as above)
193, 156, 171, 146

Corresponding number of genes (of selected Gene ontology terms) each TF is involved with:
(In the same order as above)
64, 58, 59, 52

Corresponding number of selected Gene ontology terms each TF is involved with:
(In the same order as above)
11, 11, 11, 11

Cluster for columns 58 to 70, rows 267 to 269

Gene ontology terms

spermatogenesis | protein sumoylation | regulation of gene expression | transcription factor binding | sequence-specific DNA binding | histone acetylation | regulation of transcription | chromatin remodeling complex | gene silencing | histone deacetylase activity | histone deacetylation | DNA methylation | methylation

TFs

V$ETF_Q6 | V$E2F_Q2 | V$E2F1_Q6

Information

All related TFs:
(List of all TFs that are related to any of the PWMs)
DP-1, E2F, E2F+E4, E2F-1, E2F-3a, E2F-4, ETF

Ranked gene list:
(All genes of the selected Gene ontology terms with hits of any of the selected TFs, ranked according to the total number of TFBS)
NR3C1, EGR1, YY1, SIN3A, LBX1, SP3, H2AFZ, DNMT3A, CTCF, CCND1, TP73, TCF3, ID2, E2F4, SSSCA1, SMARCA2, LEF1, JUND, JUNB, HIST1H4I, EZH2, EHMT2, CDKN1C, EP300, CREBBP, SETD1A, IRF1, CDKN1A, ACTB, SMARCA4, RBPJ, PLEC1, NFIC, MYB, HRAS, HMGA1, HIC1, DGAT1, CREB1, CCND2, CBX4, UBE2I, SOCS1, RUNX3, RASSF1, PCNA, MTA1, HNRPDL, CDKN1B, MBD3, ZMYND10, ZFPM1, SRCAP, MNT, MAZ, JMJD3, ING1, HOXA9, HOXA7,

HOXA10, HDAC7, GNAS, GATA3, GATA2, EGR2, CACNA1G, BMI1, BCL11B, AKT1, TFAP2A, RARA, POU2F1, IKZF1, ZBTB16, UHRF1, SLC20A1, SKIL, SEMA3B, RUNX2, RNF4, RING1, RCOR1, PTPN6, PELP1, PAX5, IRF4, HOXA5, HMGB1, FOXO1, ENOPH1, CISH, CDKN2D, CDK6, BAZ2A, APRT, TMEM201, TCF7, SOCS3, SMAD7, SLC25A22, SIRT7, SATB2, SAMD4A, REPIN1, RBL1, PURA, PTCH1, POU3F2, PCGF1, PAX6, NSD1, NOTCH1, NNAT, NFATC1, NAB2, MDK, MAPK12, MAFG, MAFA, LRDD, KISS1R, IGF1R, HNRNPL, H3F3A, GPX4, GGNBP2, GFI1, GAMT, FOSB, FLJ32987, FLI1, FASN, EGR3, EBF3, DAZAP1, CTDSP1, CHD3, CHAF1A, CFL1, CDKN2C, CBX8, C19orf6, BCL6B, BCL2L11, ANP32A, ZFP161, WBP7, USF2, UBL4A, TUBB2C, TRIM28, TRERF1, THRA, TGFB1, TARBP2, SPAG6, SOX1, SMARCD3, SMARCC2, SERBP1, RXRA, RRBP1, POU4F1, PCGF2, PAFAH1B1, OAZ1, NR4A2, NFYB, NFIX, MYST4, MLL, MEF2D, IRF2, HSD17B8, H2AFX, GTF2I, DAXX, CTBP1, CNOT1, CHD4, CENPB, CDK9, CDK4, CARM1, CAMK2G, CALM1, ARSG, ARHGDIA, ZEB1, VIM, TNRC6A, TGIF1, TCF12, SMAD2, SLC7A1, SLC5A6, SEPT7, SALL3, PPP1R7, PPIA, PIH1D1, PDPK1, PATZ1, PASK, NFIB, LMX1B, LMNB1, LDB1, INTS6, ILF3, ID3, ICMT, HSP90AA1, HOXD11, HOXB5, HMBS, GTF2A1, GGN, GEMIN4, GAPDH, FUS, FLOT2, FBXL10, EOMES, EFNB2, E2F2, DDAH2, CTDSPL, CTBP2, CREB3L4, COL11A2, CGGBP1, CDC2L6, CDC25C, CBX3, CASP9, C2orf28, BCL2L2, AXIN1, ATXN7, ATF3, ARNTL, ARID1A, AKT3

Corresponding total number of TFBS:
(For each gene listed above, the total number of TFBS for any of the selected TFs, multiplied by the number of selected Gene ontology terms containing that gene)
30, 24, 22, 21, 21, 20, 18, 18, 18, 18, 16, 15, 15, 15, 12, 12, 12, 12, 12, 12, 12, 12, 12, 11, 11, 10, 10, 10, 10, 9, 9, 9, 9, 9, 9, 9, 9, 9, 9, 9, 9, 8, 8, 8, 8, 8, 8, 8, 8, 7, 6, 6, 6, 6, 6, 6, 6, 6, 6, 6, 6, 6, 6, 6, 6, 6, 6, 6, 5, 5, 5, 5, 4, 4, 4, 4, 4, 4, 4, 4, 4, 4, 4, 4, 4, 4, 4, 4, 4, 4, 4, 4, 4, 3, 3, 3, 3, 3, 3, 3, 3, 3, 3, 3, 3, 3, 3, 3, 3, 3, 3, 3, 3, 3, 3, 3, 3, 3, 3, 3, 3, 3, 3, 3, 3, 3, 3, 3, 3, 3, 3, 3, 3, 3, 3, 3, 3, 3, 3, 3, 2, 2, 2, 2, 2, 2, 2, 2, 2, 2, 2, 2, 2, 2, 2, 2, 2, 2, 2, 2, 2, 2, 2, 2, 2, 2, 2, 2, 2, 2, 2, 2, 2, 2, 2, 2, 2, 2, 2, 2, 2, 2, 2, 2, 1, 1, 1, 1, 1, 1, 1, 1, 1, 1, 1, 1, 1, 1, 1, 1, 1, 1, 1, 1, 1, 1, 1, 1, 1, 1, 1, 1, 1, 1, 1, 1, 1, 1, 1, 1, 1, 1, 1, 1, 1, 1, 1, 1, 1, 1, 1, 1, 1

Corresponding number of selected Gene ontology terms each gene is involved with:
(In the same order as above)
10, 8, 11, 7, 7, 10, 6, 6, 6, 6, 8, 5, 5, 5, 4, 6, 4, 4, 4, 12, 6, 6, 4, 11, 11, 5, 5, 10, 5, 9, 3, 3, 3, 9, 3, 3, 3, 3, 9, 3, 3, 4, 4, 4, 4, 4, 4, 4, 4, 7, 3, 2, 3, 2, 2, 2, 2, 2, 2, 2, 3, 2, 2, 2, 3, 3, 3, 2, 2, 5, 5, 5, 5, 4, 2, 4, 2, 2, 2, 2, 2, 2, 2, 4, 4, 2, 4, 2, 2, 2, 4, 4, 2, 2, 2, 3, 1, 1, 1, 1, 1, 1, 1, 1, 3, 1, 1, 1, 1, 1, 1, 1, 3, 1, 1, 1, 1, 1, 1, 1, 1, 1, 1, 3, 1, 1, 1, 1, 3, 1, 1, 1, 1, 1, 1, 1, 3, 3, 1, 1, 1, 1, 1, 1, 1, 1, 1, 1, 2, 1, 2, 1, 1, 2, 1, 1, 1, 1, 2, 2, 1, 1, 1, 2, 2, 1, 1, 1, 1, 1, 2, 1, 2, 1, 2, 1, 2, 1, 2, 2, 1, 2, 2, 2, 1, 1, 1, 1, 1, 1, 1, 1, 1, 1, 1, 1, 1, 1, 1, 1, 1, 1, 1, 1, 1, 1, 1, 1, 1, 1, 1, 1, 1, 1, 1, 1, 1, 1, 1, 1, 1, 1, 1, 1, 1, 1, 1, 1, 1, 1, 1, 1, 1, 1, 1, 1, 1, 1, 1, 1, 1, 1, 1, 1

Corresponding number of selected TFs each gene is involved with:
(In the same order as above)
3, 3, 2, 3, 3, 2, 3, 3, 3, 3, 2, 3, 3, 3, 3, 2, 3, 3, 3, 1, 2, 2, 3, 1, 1, 2, 2, 1, 2, 1, 3, 3, 3, 1, 3, 3, 3, 3, 1, 3, 3, 2, 2, 2, 2, 2, 2, 2, 2, 1, 2, 3, 2, 3, 3, 3, 3, 3, 3, 3, 2, 3, 3, 3, 2, 2, 2, 3, 3, 1, 1, 1, 1, 1, 2, 1, 2, 2, 2, 2, 2, 2, 2, 1, 1, 2, 1, 2, 2, 2, 1, 1, 2, 2, 2, 1, 3, 3, 3, 3, 3, 3, 3, 3, 1, 3, 3, 3, 3, 3, 3, 3, 1, 3, 3, 3, 3, 3, 3, 3, 3, 3, 3, 1, 3, 3, 3, 3, 1, 3, 3, 3, 3, 3, 3, 3, 1, 1, 3, 3, 3, 3, 3, 3, 3, 2, 2, 2, 1, 2, 1, 2, 2, 1, 2, 2, 2, 2, 1, 1, 2, 2, 2, 1, 1, 2, 2, 2, 2, 2, 1, 2, 1, 2, 1, 2, 1, 2, 1, 1, 2, 1, 1, 1, 2, 2, 2, 2, 1, 1, 1, 1, 1, 1, 1, 1, 1, 1, 1, 1, 1, 1, 1, 1, 1, 1, 1, 1, 1, 1, 1, 1, 1, 1, 1, 1, 1, 1, 1, 1, 1, 1, 1, 1, 1, 1, 1, 1, 1, 1, 1, 1, 1, 1, 1, 1, 1, 1, 1, 1, 1, 1, 1, 1, 1

Gene ontology terms ranked according to mean of effect sizes in cluster:
transcription factor binding | sequence-specific DNA binding | regulation of gene expression | histone deacetylation | histone deacetylase activity | spermatogenesis | protein sumoylation | chromatin remodeling complex | gene silencing | DNA methylation | regulation of transcription | histone acetylation | methylation

Corresponding mean of effect sizes of each Gene ontology term:
(In the same order as above)
3.02, 2.40, 2.24, 2.22, 2.11, 2.07, 2.02, 2.02, 1.93, 1.71, 1.65, 1.59, 1.45

Corresponding total number of genes of each Gene ontology term:
(In the same order as above)
231, 118, 310, 144, 108, 397, 166, 105, 355, 437, 187, 283, 715

Corresponding number of genes of each Gene ontology term with at least one TFBS (of selected TFs):
(In the same order as above)
45, 26, 62, 35, 26, 51, 35, 25, 57, 60, 37, 46, 86

Corresponding total number of TFBS (of selected TFs) for each Gene ontology term (in all genes):
(In the same order as above)
93, 51, 116, 63, 50, 96, 64, 45, 114, 116, 66, 90, 170

Corresponding number of selected TFs each Gene ontology term is involved with:
(In the same order as above)
3, 3, 3, 3, 3, 3, 3, 3, 3, 3, 3, 3, 3

TFs ranked according to mean of effect sizes in cluster:
V$E2F_Q2, V$E2F1_Q6, V$ETF_Q6

Corresponding mean of effect sizes of each TF:
(In the same order as above)
2.30, 1.92, 1.89

Corresponding total number of TFBS for each TF (genome-wide):
(In the same order as above)
714, 691, 699

Corresponding total number of TFBS for each TF (in all genes in selected Gene ontology terms):
(In the same order as above)
397, 380, 357

Corresponding number of genes (of selected Gene ontology terms) each TF is involved with:
(In the same order as above)
159, 159, 153

Corresponding number of selected Gene ontology terms each TF is involved with:
(In the same order as above)
13, 13, 13

Cluster for columns 457 to 539, rows 307 to 312

Gene ontology terms

chemotaxis | leukocyte migration | cell chemotaxis | positive chemotaxis | B cell activation | immunoglobulin production | B cell proliferation | immunoglobulin secretion | interferon-gamma production | T-helper 1 type immune response | response to host immune response | adaptive immune response | adaptive immune response based on somatic recombination of immune receptors built from immunoglobulin superfamily domains | chemokine production | interleukin-10 production | interleukin-12 production | cell maturation | MHC class I biosynthetic process | MHC class I protein binding | MHC class II biosynthetic process | MHC class II protein binding | antigen processing and presentation | immune response | type IV hypersensitivity | lymphocyte proliferation | tolerance induction | lymphocyte activation | interleukin-2 production | interleukin-2 receptor activity | T cell proliferation | interleukin-4 production | cytokine production | cytokine secretion | pathogenesis | interleukin-4 receptor activity |

interleukin-5 production | evasion or tolerance of immune response of other organism during symbiotic interaction | tryptophan catabolic process | defense response to virus | innate immune response | tumor necrosis factor receptor activity | tumor necrosis factor receptor binding | positive regulation of NF-kappaB transcription factor activity | response to tumor necrosis factor | IkappaB kinase complex | NF-kappaB binding | interleukin-6 receptor activity | interleukin-1 beta production | interleukin-1 production | cytolysis | natural killer cell mediated cytotoxicity | natural killer cell receptor activity | naringenin-chalcone synthase activity | immature T cell proliferation in the thymus | eosinophil activation | leukocyte activation | monocyte activation | mucosal immune response | granulocyte macrophage colony-stimulating factor biosynthetic process | granulocyte macrophage colony-stimulating factor production | interleukin-1 receptor activity | interleukin-6 production | tumor necrosis factor production | macrophage activation | inflammatory response | response to lipopolysaccharide | negative regulation of inflammatory response | regulation of tumor necrosis factor production | negative regulation of tumor necrosis factor production | positive regulation of tumor necrosis factor production | interleukin-1 receptor antagonist activity | acute inflammatory response | granuloma formation | lipopolysaccharide binding | cytokine biosynthetic process | interleukin-8 production | mRNA transcription | chronic inflammatory response | monocyte differentiation | hypersensitivity | hyaluronic acid binding | cytokine activity | rosetting

TFs

V$NFKB_Q6_01 | V$NFKAPPAB_01 | V$NFKAPPAB65_01 | V$CREL_01 | V$NFKB_Q6 | V$NFKB_C

Information

All related TFs:
(List of all TFs that are related to any of the PWMs)
NF-TNF, NF-kappaB, NF-kappaB(-like), NF-kappaB2, RelA-p65, c-Rel, p100, p105, p50, p52

Ranked gene list:
(All genes of the selected Gene ontology terms with hits of any of the selected TFs, ranked according to the total number of TFBS)
TNF, ICAM1, CD4, LTA, IRF6, CD40, CSF1, NFKBIA, CD69, REL, STAT1, CXCL10, NOD2, LTB, AKT1, IL2RA, TNFRSF4, STAT6, IRF1, RELA, EDC4, CD58, TNFRSF18, CD83, NFKB1, TNFRSF9, TNFSF13, TGFB1, RELB, IL17C, FLT3LG, ITGAM, CD86, CD5, PER1, DPP4, CD70, TRAF2, NFATC2, CD27, PTPN6, PSMB8, MAPK14, IRF8, CREB1, IL6ST, IL1RN, IL11, GPR132, FAS, CXCR5, TRAF3, PTMA, MYD88, PDLIM7, ISYNA1, TRADD, TNFRSF6B, TNFAIP3, NFKBIB, MAP2K3, IL27, IKBKE, B2M, NFATC1, IL23A, ERCC8, EPHB2, PDCD1, PDCD1LG2, NFKB2, LAG3, JUNB, IRF5, HLA-A, CXCL1, IRF3, IL16, EGR1, CXCL5, MYB, MIF, IL7R, STAT3, PTGS1, PDE4A, PAX5, LCK, IFIH1, TNFRSF1B, TNIP1, PSMB9, LMNA, IRF2, CFLAR, BIRC3, VEGFA, SOCS3, KLRK1, TRIM63, TRAF1, TAPBP, TAP1, FGR, CISH, MAP3K8, GADD45B, CYLD, CCND1, AGER, JAK1, HLA-B, CXCL6, CCL21, RPS6KA1, ROBLD3, PLEKHF1, PLAU, PARP1, NR3C1, NFKBIE, NCAPG2, MAP4K4, LGALS3, IKBKB, ICOSLG, HIVEP2, DLX2, CXCL2, CX3CL1, TNFRSF25, ZAP70, TNFRSF14, TNFRSF11B, TMPO, STXBP3, PRG2, NDUFA2, JAK3, HSP90B1, DUSP2, SLC19A1, PSME3, PSME2, MPRIP, IL19, IL12B, HLA-DMB, GATA3, CCR10, TYROBP, TSHR, TOLLIP, SMPD1, SLC25A22, RXRB, RAB27A, PTGER4, NBPF1, IL32, HLA-C, EIF4G2, EDAR, CXCL16, COL11A2, CDKN2C, CD300A, MUC1, ICAM2, ZC3H12A, TRAF4, TMEM201, TAP2, SSSCA1, SEMA3B, RNASEK, PTGDS, PLCG1, PIBF1, PGF, PACS1, NGFR, NFKBIZ, MAZ, MAP3K14, MAP3K11, MAL, HSPA1A, HIVEP1, HCST, GABARAP, FLNA, EHD1, CYSLTR1, CXCL3, CSK, CREBBP, CRADD, CDC37, CD55, CCL25, CANX, CAMK2G, BCL3, ABCB9, ZFP36, SP1, SMPD2, SHH, RUNX3, RTEL1, PRDX2, MAP3K1, IL27RA, FYN, ERAP1, CCL1, ACTB, WDR26, TUBGCP2, TSPAN4, TSC22D3, TRPV3, TNFRSF12A, TIFA, TCIRG1, STAG1, SPATA2, SPAG1, SIRT1, SEPSECS, S1PR2, PTGER1, ORAI1, NSFL1C, NR4A2, NOTCH1, NANP, MR1, LSP1, KSR1, JUND, ITGA5, ILK, HSP90AA1, HIF1A, HAMP, GSTP1, ESAM, EGR2, DCTN3, CTSS, CRY2, CRTC2, CORO7, CFL1, BCL6, ART3, AHR, VIM, VDAC1, UBD, TUSC2, TSC2, STMN1,

SH2D3C, S1PR4, RUNX1, RIPK3, RHOF, RAC2, PVRL1, PTPN7, PLP1, PLAUR, PAWR, NTRK1, NR4A1, NOTCH4, MLLT10, LRDD, KLF6, IRS1, IL4R, HFE, FOXP1, FOSB, FLJ32987, ETV6, EPB41, DCXR, DAXX, COL6A1, CDKN2A, CDK4, CCND3, CCND2, CASP6, BRAF, BDNF, ATXN1, ATN1, AIRE, AFF3, ADRBK2, ZMYND10, ZFPM1, XRCC1, WHSC1, UNC13D, UBE2L6, TNIP3, TCEA2, STRA13, SMAD7, SFRS2, SERPINH1, SERPINA1, SEC62, RTN2, REV3L, RASSF5, RASSF1, RARA, PTGES, PSMP, PPP3CA, PPARGC1B, POLDIP3, PNKD, PIM1, PIK3CD, PAK4, P21, OTP, NXT1, NTRK3, NRAS, NFYA, NFKBID, MEN1, LRP2, KIAA1524, INHBB, GSK3B, GPR172B, GDNF, GBA, GATA4, GALNT6, FDXR, EOMES, EFNA1, ECE1, EBAG9, DUSP28, DNAH1, DIABLO, DHX58, DAPP1, DAD1, CYP27B1, CXCR7, CKLF, CKAP4, CIR, CHI3L2, CDK5R2, CACNB3, BTRC, ANKRA2, ALCAM, ADA, ZBTB16, VCAN, USF2, USF1, UNC5A, TRIM69, TCEB2, TBP, TBC1D1, SRF, SREBF1, SP2, SNCG, SNCB, SLC36A2, SLC25A18, SKAP1, SERPINB9, SERPINA4, SEMA4A, S100A6, RPS6KA3, RET, PTHLH, PLTP, PDGFRA, PDGFB, PDE4DIP, PARK7, PAOX, PAFAH1B1, OGFR, MVD, MSN, MAPK12, MAPK11, MAG, LY6E, LSM2, ITM2B, IFIT3, IER3, HDC, H2AFX, GNB2L1, FUT8, FOSL1, FHL3, FGF8, FGF1, FBL, ERN1, ERCC2, ELF3, EIF4E, DRD4, DMPK, DISC1, CYBA, CDK9, CDC2L5, CD3D, CD37, CARD8, C11orf2, BCL9, BATF, AMH, ALOX5AP, ALG1, AGPAT1

Corresponding total number of TFBS:
(For each gene listed above, the total number of TFBS for any of the selected TFs, multiplied by the number of selected Gene ontology terms containing that gene)
492, 372, 280, 258, 219, 186, 174, 150, 144, 138, 136, 128, 126, 120, 120, 112, 105, 102, 95, 93, 84, 72, 70, 70, 66, 64, 63, 60, 60, 60, 60, 57, 56, 54, 50, 48, 48, 45, 45, 42, 40, 40, 40, 40, 40, 39, 39, 39, 39, 39, 36, 35, 35, 34, 33, 31, 30, 30, 30, 30, 30, 30, 30, 30, 28, 28, 26, 26, 25, 24, 24, 24, 24, 24, 24, 24, 22, 22, 22, 22, 21, 21, 21, 20, 20, 20, 20, 20, 20, 19, 18, 18, 18, 18, 18, 18, 17, 17, 17, 16, 16, 16, 16, 16, 16, 15, 15, 15, 15, 15, 14, 14, 14, 13, 12, 12, 12, 12, 12, 12, 12, 12, 12, 12, 12, 12, 12, 12, 12, 11, 10, 10, 10, 10, 10, 10, 10, 10, 10, 10, 9, 9, 9, 9, 9, 9, 9, 9, 9, 8, 8, 8, 8, 8, 8, 8, 8, 8, 8, 8, 8, 8, 8, 8, 8, 7, 7, 6, 6, 6, 6, 6, 6, 6, 6, 6, 6, 6, 6, 6, 6, 6, 6, 6, 6, 6, 6, 6, 6, 6, 6, 6, 6, 6, 6, 6, 6, 6, 6, 6, 6, 5, 5, 5, 5, 5, 5, 5, 5, 5, 5, 5, 5, 5, 4, 4, 4, 4, 4, 4, 4, 4, 4, 4, 4, 4, 4, 4, 4, 4, 4, 4, 4, 4, 4, 4, 4, 4, 4, 4, 4, 4, 4, 4, 4, 4, 4, 4, 4, 4, 4, 4, 4, 4, 3, 3, 3, 3, 3, 3, 3, 3, 3, 3, 3, 3, 3, 3, 3, 3, 3, 3, 3, 3, 3, 3, 3, 3, 3, 3, 3, 3, 3, 3, 3, 3, 3, 3, 3, 3, 3, 3, 3, 3, 3, 3, 3, 3, 3, 3, 3, 3, 3, 2, 2, 2, 2, 2, 2, 2, 2, 2, 2, 2, 2, 2, 2, 2, 2, 2, 2, 2, 2, 2, 2, 2, 2, 2, 2, 2, 2, 2, 2, 2, 2, 2, 2, 2, 2, 2, 2, 2, 2, 2, 2, 2, 2, 2, 2, 2, 2, 2, 2, 2, 2, 2, 2, 2, 2, 2, 2, 2, 2, 2, 2, 2, 2, 2, 2, 1, 1, 1, 1, 1, 1, 1, 1, 1, 1, 1, 1, 1, 1, 1, 1, 1, 1, 1, 1, 1, 1, 1, 1, 1, 1, 1, 1, 1, 1, 1, 1, 1, 1, 1, 1, 1, 1, 1, 1, 1, 1, 1, 1, 1, 1, 1, 1, 1, 1, 1, 1, 1, 1, 1, 1, 1, 1, 1, 1, 1, 1, 1, 1, 1, 1, 1

Corresponding number of selected Gene ontology terms each gene is involved with:
(In the same order as above)
82, 62, 70, 43, 73, 62, 29, 25, 48, 23, 34, 32, 21, 20, 20, 56, 21, 17, 19, 31, 21, 24, 14, 35, 11, 16, 63, 12, 15, 15, 12, 57, 56, 18, 10, 12, 12, 9, 9, 21, 10, 8, 40, 10, 8, 13, 39, 13, 13, 39, 9, 7, 7, 34, 11, 31, 6, 5, 6, 6, 6, 10, 5, 5, 7, 14, 26, 26, 5, 12, 4, 6, 4, 4, 12, 12, 11, 11, 11, 11, 7, 21, 7, 20, 5, 5, 4, 20, 4, 19, 3, 9, 6, 6, 6, 6, 17, 17, 17, 8, 4, 4, 8, 4, 8, 5, 3, 3, 5, 3, 7, 7, 7, 13, 2, 2, 2, 6, 4, 6, 2, 12, 6, 4, 12, 12, 4, 3, 12, 12, 11, 5, 5, 10, 5, 2, 10, 10, 10, 5, 2, 3, 3, 3, 3, 3, 9, 3, 3, 3, 4, 8, 8, 4, 2, 2, 2, 8, 4, 2, 8, 4, 4, 4, 4, 2, 4, 7, 7, 1, 3, 2, 6, 3, 1, 1, 6, 3, 3, 2, 2, 2, 2, 1, 6, 1, 2, 6, 2, 3, 3, 1, 2, 6, 6, 3, 6, 3, 1, 3, 3, 6, 1, 1, 3, 1, 5, 5, 1, 1, 1, 1, 5, 5, 5, 5, 5, 5, 1, 4, 1, 2, 1, 4, 1, 2, 1, 2, 2, 1, 2, 1, 2, 2, 4, 2, 2, 4, 4, 1, 1, 1, 4, 1, 4, 4, 4, 1, 4, 1, 2, 4, 1, 1, 2, 2, 4, 1, 4, 1, 3, 3, 1, 1, 1, 1, 3, 3, 3, 1, 1, 3, 1, 3, 3, 3, 3, 1, 1, 1, 1, 1, 3, 3, 3, 3, 1, 3, 1, 1, 3, 1, 1, 3, 1, 3, 3, 3, 1, 1, 1, 1, 1, 1, 1, 1, 1, 1, 1, 2, 1, 2, 1, 1, 2, 1, 1, 2, 1, 2, 1, 2, 1, 2, 2, 2, 1, 1, 2, 1, 1, 1, 1, 1, 1, 1, 2, 1, 2, 1, 1, 1, 2, 1, 1, 1, 2, 1, 1, 2, 1, 1, 1, 1, 2, 1, 1, 1, 2, 1, 2, 1, 2, 1, 1, 2, 1, 2, 1, 2, 2, 2, 1, 1, 1, 1, 1, 1, 1, 1, 1, 1, 1, 1, 1, 1, 1, 1, 1, 1, 1, 1, 1, 1, 1, 1, 1, 1, 1, 1, 1, 1, 1, 1, 1, 1, 1, 1, 1, 1, 1, 1, 1, 1, 1, 1, 1, 1, 1, 1, 1, 1, 1, 1, 1, 1, 1, 1, 1, 1, 1, 1, 1, 1, 1, 1, 1, 1, 1, 1

Corresponding number of selected TFs each gene is involved with:
(In the same order as above)
6, 6, 4, 6, 3, 3, 6, 6, 3, 6, 4, 4, 6, 6, 6, 2, 5, 6, 5, 3, 4, 3, 5, 2, 6, 4, 1, 5, 4, 4, 5, 1, 1, 3, 5, 4, 4, 5, 5, 2, 4, 5, 1, 4, 5, 3, 1, 3, 3, 1, 4, 5, 5, 1, 3, 1, 5, 6, 5, 5, 5, 3, 6, 6, 4, 2, 1, 1, 5, 2, 6, 4, 6, 6, 2, 2, 2, 2, 2, 2, 3, 1, 3, 1, 4, 4, 5, 1, 5, 1, 6, 2, 3, 3, 3, 3, 1, 1, 1, 2, 4, 4, 2, 4, 2, 3, 5, 5, 3, 5, 2, 2, 2, 1, 6, 6, 6, 2, 3, 2, 6, 1, 2, 3, 1, 1, 3, 4, 1, 1, 1, 2, 2, 1, 2, 5, 1, 1, 1, 2, 5, 3, 3, 3, 3, 3, 1, 3, 3, 3, 2, 1, 1, 2, 4, 4, 4, 1, 2, 4, 1, 2, 2, 2, 2, 4, 2, 1, 1, 6, 2, 3, 1, 2, 6, 6, 1, 2, 2, 3, 3, 3, 3, 6, 1, 6, 3, 1, 3, 2, 2, 6, 3, 1, 1, 2, 1, 2, 6, 2, 2, 1, 6, 6, 2, 5, 1, 1, 5, 5,

5, 5, 1, 1, 1, 1, 1, 1, 4, 1, 4, 2, 4, 1, 4, 2, 4, 2, 2, 4, 2, 4, 2, 2, 1, 2, 2, 1, 1, 4, 4, 4, 1, 4, 1, 1, 1, 4, 1, 4, 2, 1, 4, 4, 2, 2, 1, 4, 1, 3, 1, 1, 3, 3, 3, 3, 1, 1, 1, 3, 3, 1, 3, 1, 1, 1, 1, 3, 3, 3, 3, 3, 1, 1, 1, 1, 3, 1, 3, 3, 1, 3, 3, 1, 3, 1, 1, 1, 3, 3, 3, 3, 3, 3, 3, 2, 2, 2, 2, 1, 2, 1, 2, 2, 1, 2, 2, 1, 2, 1, 2, 1, 2, 1, 1, 1, 2, 2, 1, 2, 2, 2, 2, 2, 2, 2, 1, 2, 1, 2, 2, 2, 1, 2, 2, 2, 1, 2, 2, 1, 2, 2, 2, 2, 1, 2, 2, 2, 1, 2, 1, 2, 1, 2, 2, 1, 2, 1, 2, 1, 1, 1, 2, 1, 1, 1, 1, 1, 1, 1, 1, 1, 1, 1, 1, 1, 1, 1, 1, 1, 1, 1, 1, 1, 1, 1, 1, 1, 1, 1, 1, 1, 1, 1, 1, 1, 1, 1, 1, 1, 1, 1, 1, 1, 1, 1, 1, 1, 1, 1, 1, 1, 1, 1, 1, 1, 1, 1, 1, 1, 1, 1, 1, 1, 1, 1, 1, 1, 1, 1, 1, 1, 1

Gene ontology terms ranked according to mean of effect sizes in cluster:
IkappaB kinase complex | response to tumor necrosis factor | tumor necrosis factor receptor activity | tumor necrosis factor receptor binding | immune response | interleukin-12 production | NF-kappaB binding | chemokine production | positive regulation of NF-kappaB transcription factor activity | response to lipopolysaccharide | MHC class I protein binding | MHC class I biosynthetic process | cytokine secretion | interleukin-8 production | granulocyte macrophage colony-stimulating factor production | interleukin-6 production | T-helper 1 type immune response | granulocyte macrophage colony-stimulating factor biosynthetic process | cytokine production | innate immune response | inflammatory response | granuloma formation | adaptive immune response based on somatic recombination of immune receptors built from immunoglobulin superfamily domains | regulation of tumor necrosis factor production | interleukin-10 production | response to host immune response | mucosal immune response | T cell proliferation | chronic inflammatory response | evasion or tolerance of immune response of other organism during symbiotic interaction | macrophage activation | adaptive immune response | interferon-gamma production | naringenin-chalcone synthase activity | monocyte activation | interleukin-4 production | interleukin-2 receptor activity | positive chemotaxis | interleukin-6 receptor activity | interleukin-2 production | B cell activation | negative regulation of tumor necrosis factor production | antigen processing and presentation | cytokine biosynthetic process | lymphocyte proliferation | defense response to virus | tumor necrosis factor production | interleukin-1 receptor antagonist activity | B cell proliferation | mRNA transcription | interleukin-5 production | leukocyte activation | immunoglobulin production | acute inflammatory response | immature T cell proliferation in the thymus | cell maturation | positive regulation of tumor necrosis factor production | interleukin-4 receptor activity | type IV hypersensitivity | MHC class II biosynthetic process | negative regulation of inflammatory response | cytokine activity | MHC class II protein binding | cytolysis | pathogenesis | rosetting | immunoglobulin secretion | lymphocyte activation | tryptophan catabolic process | tolerance induction | leukocyte migration | interleukin-1 receptor activity | interleukin-1 beta production | hypersensitivity | natural killer cell receptor activity | monocyte differentiation | chemotaxis | eosinophil activation | hyaluronic acid binding | lipopolysaccharide binding | interleukin-1 production | cell chemotaxis | natural killer cell mediated cytotoxicity

Corresponding mean of effect sizes of each Gene ontology term:
(In the same order as above)
4.04, 3.96, 3.84, 3.77, 3.59, 3.58, 3.54, 3.50, 3.41, 3.31, 3.18, 3.09, 3.06, 3.00, 2.98, 2.95, 2.94, 2.88, 2.74, 2.71, 2.68, 2.67, 2.67, 2.59, 2.56, 2.45, 2.43, 2.37, 2.31, 2.21, 2.20, 2.20, 2.14, 2.13, 2.12, 2.11, 2.09, 2.06, 2.06, 2.03, 2.03, 2.02, 2.02, 1.98, 1.95, 1.89, 1.81, 1.80, 1.78, 1.72, 1.68, 1.68, 1.64, 1.64, 1.62, 1.60, 1.60, 1.56, 1.53, 1.53, 1.47, 1.47, 1.42, 1.42, 1.41, 1.35, 1.34, 1.25, 1.22, 1.21, 1.18, 1.16, 1.07, 0.98, 0.97, 0.96, 0.91, 0.77, 0.59, 0.58, 0.48, 0.46, 0.43

Corresponding total number of genes of each Gene ontology term:
(In the same order as above)
107, 185, 213, 214, 530, 150, 146, 155, 550, 226, 197, 195, 243, 144, 107, 165, 59, 109, 455, 246, 425, 77, 131, 86, 166, 37, 52, 188, 22, 141, 178, 148, 106, 50, 117, 123, 189, 87, 104, 182, 116, 61, 315, 108, 158, 110, 209, 70, 88, 130, 57, 108, 155, 68, 42, 224, 61, 87, 131, 181, 40, 42, 178, 179, 1.1K, 63, 51, 232, 41, 109, 145, 128, 21, 234, 77, 102, 371, 71, 37, 61, 31, 94, 33

Corresponding number of genes of each Gene ontology term with at least one TFBS (of selected TFs):
(In the same order as above)
34, 53, 61, 61, 110, 41, 46, 41, 131, 56, 56, 55, 52, 35, 26, 42, 17, 25, 94, 51, 82, 17, 28, 25, 42, 10, 11, 42, 6, 34, 38, 29, 21, 10, 23, 26, 43, 21, 24, 46, 27, 17, 67, 27, 32, 30, 40, 15, 20, 33, 13, 18, 28, 14, 11,

48, 17, 24, 30, 38, 12, 9, 37, 32, 192, 12, 11, 50, 10, 21, 26, 23, 4, 31, 14, 17, 56, 9, 6, 12, 4, 17, 5

Corresponding total number of TFBS (of selected TFs) for each Gene ontology term (in all genes):
(In the same order as above)
119, 168, 180, 180, 295, 122, 134, 105, 377, 175, 156, 154, 162, 102, 80, 123, 49, 79, 261, 137, 218, 51, 69, 71, 107, 32, 38, 120, 25, 88, 111, 75, 60, 35, 61, 82, 125, 53, 66, 138, 76, 48, 176, 74, 93, 80, 115, 47, 62, 96, 44, 51, 72, 35, 34, 131, 43, 62, 78, 98, 29, 32, 95, 81, 458, 37, 32, 138, 33, 60, 67, 62, 13, 90, 33, 46, 128, 27, 17, 21, 11, 40, 15

Corresponding number of selected TFs each Gene ontology term is involved with:
(In the same order as above)
6, 6, 6, 6, 6, 6, 6, 6, 6, 6, 6, 6, 6, 6, 6, 6, 6, 6, 6, 6, 6, 6, 6, 6, 6, 6, 6, 6, 6, 6, 6, 6, 6, 6, 6, 6, 6, 6, 6, 6, 6, 6, 6, 6, 6, 6, 6, 6, 6, 6, 6, 6, 6, 6, 6, 6, 6, 6, 6, 6, 6, 6, 6, 6, 6, 6, 6, 6, 6, 6, 6, 6, 6, 6, 6, 6, 6, 6, 6, 6, 6, 6, 6

TFs ranked according to mean of effect sizes in cluster:
V$NFKAPPAB_01, V$NFKB_C, V$NFKAPPAB65_01, V$NFKB_Q6_01, V$NFKB_Q6, V$CREL_01

Corresponding mean of effect sizes of each TF:
(In the same order as above)
2.36, 2.30, 2.18, 2.15, 1.96, 1.43

Corresponding total number of TFBS for each TF (genome-wide):
(In the same order as above)
702, 685, 716, 713, 689, 713

Corresponding total number of TFBS for each TF (in all genes in selected Gene ontology terms):
(In the same order as above)
1420, 1308, 1346, 1313, 1283, 1193

Corresponding number of genes (of selected Gene ontology terms) each TF is involved with:
(In the same order as above)
177, 165, 189, 175, 182, 178

Corresponding number of selected Gene ontology terms each TF is involved with:
(In the same order as above)
83, 83, 83, 83, 83, 83

Cluster for columns 1296 to 1311, rows 97 to 108

Gene ontology terms

gene expression | DBD domain binding | DNA binding | chordate embryonic development | embryonic development | limb development | central nervous system development | central nervous system neuron development | gastrulation | somitogenesis | anatomical structure morphogenesis | organ development | neurogenesis | regionalization | cell fate determination | segmentation

TFs

V$STAT6_01 | V$STAT5A_03 | V$STAT4_01 | V$STAT5A_04 | V$STAT1_03 | V$CEBP_Q3 | V$PAX2_02 | V$HMGIY_Q6 | V$HOXA4_Q2 | V$TST1_01 | V$NFAT_Q6 | V$NFAT_Q4_01

Information

All related TFs:
(List of all TFs that are related to any of the PWMs)
ANF-2, C/EBP, C/EBPalpha, C/EBPalpha(p20), C/EBPalpha(p30), C/EBPbeta, C/EBPbeta(LAP), C/EBPbeta(p20), C/EBPbeta(p34), C/EBPbeta(p35), C/EBPdelta, C/EBPepsilon, C/EBPgamma, HMG, HMG-Y, HMGI-C, HOXA4, NF-AT, NF-AT1, NF-AT1C, NF-AT2, NF-AT3, NF-AT4, POU3F1, Pax-2, Pax-2.1, Pax-2.2, Pax-2a, Pax-2b, STAT1, STAT1alpha, STAT4, STAT5A, STAT6

Ranked gene list:
(All genes of the selected Gene ontology terms with hits of any of the selected TFs, ranked according to the total number of TFBS)
LBX1, PAX6, LEF1, WNT1, HAND2, DLL1, RUNX1, FGF8, CTNNB1, NR2F1, HOXA9, HES1, ID2, DLX2, NR4A2, HOXA7, CXCR4, LHX1, TFAP2A, HOXD13, HMX3, VIM, RARG, HOXB7, HOXA11, HOXA10, ETS1, EOMES, EGR2, CDC25C, HOXA1, NKX2-2, ID3, GLI1, EPHA4, RARA, PRDM1, POU4F1, FLI1, DLX1, RBPJ, NEUROG2, CD44, PTCH1, IL23A, HOXA13, RUNX3, NR3C1, JARID2, MEIS2, TCF7, SOX2, SMAD7, NFIA, LMO4, HOXC8, HOXC6, HOXB5, HOXB4, HOXB3, HMX2, GATA3, CDKN1B, CCND2, BRD2, HOXA5, EVX2, TLE3, SSSCA1, SMARCA2, LDB1, HSPA8, HOXB6, HOXB2, CITED2, BHLHB2, TGFBR2, NR2F2, NFATC1, JUN, CDK5, ZFP36L2, ZEB2, TIPARP, THRA, TGIF1, NFATC3, MXI1, MEF2D, LFNG, KLF6, IRF1, HOXB8, HIST1H4I, HES7, GSK3B, GFI1, EGR1, DAAM1, CSK, CDK4, CDK2, PROX1, GDF11, STAT1, SIN3A, SHC1, RXRB, NR3C2, MBNL1, MAP3K14, LHX9, ETV6, BMI1, STAT3, NFYA, NFE2L1, MYB, HOXA2, ETV3, COL11A2, BCL2, ZIC2, WNT10B, TAF8, SMAD3, FOXD3, ETV1, ANXA6, AKT1, JUNB, PDLIM5, PAX3, MAF, HOXB9, FGF9, ZBTB7B, USF1, TNF, SIX1, SATB1, RREB1, PPP1R1B, NR4A3, NFKBIA, MYC, MLL, MBNL2, LTB, LTA, IRX5, IRF2, IKZF1, HOXC9, HOXC5, HOXC4, HEY2, H3F3B, FST, FOXP1, FOXG1, EBF1, CISH, BUB3, ARID1A, ARHGEF2, ZHX2, UBE2B, OTX2, OSR1, NAB2, KLF7, FOXJ2, FOSL2, CHD3, CFL1, CDKN2C, CCR7, BCL2L1, BACH2, ZIC1, ZEB1, VEGFA, PITX2, FLJ32987, E2F3, SSBP3, SOX4, PURA, NFKBIE, MBD1, MAP2K3, KLF9, ILK, HSPA1B, HOXD11, HMGA1, HIVEP1, GATA2, DUSP6, DDX5, CREB1, CDC42SE1, ACVR1, VDR, SOS1, SKP1, MNT, MIER1, MAZ, MARK2, LCP1, HOXA3, HNRNPA1, HIST4H4, CBX5, ARNTL, ADNP, SOCS1, S100A10, PIK3R1, PCNA, NEDD9, HOXD8, BCL2L11, BATF, STAT5A, SPRY1, PSMC5, NFYC, NDRG1, UBTF, TNFRSF1A, PAX5, MAP3K1, GDNF, DDIT3, CYP27B1, CDKN1A, ADAMTS6, ZFP36L1, ZFP36, VEGFB, TUSC2, TRERF1, TNFAIP3, TAP1, SRF, SHOX2, SFMBT2, S100A6, RORC, RGMB, RERE, PTMA, PTGER4, PSMB8, PSENEN, POU3F2, PITX3, PHF20, PER1, PDCD4, PAX2, OSM, NR1D1, NNAT, NIN, MZF1, MYL6, MLL2, MGAT1, MAPKAPK3, MAP4K4, MAP3K12, KLF12, JMJD3, IRS1, HOXD12, HNRNPC, HIVEP2, HDAC7, FYN, FOXN3, EVL, DUSP1, DLX6, DKFZp779C0757, CYP26B1, CYFIP2, CUGBP2, CHST11, CDK6, CAP1, BTG1, BCL6, BCL11B, ATXN1, ARID5B, ALDOA, AGER, TNIP1, RING1, RASSF1, PSMB9, PHF12, MVP, IL7R, HOXC10, GBF1, DCTN3, CRTC2, CD4, BCL9L, ATP1A1, AGPAT1, TRIB2, TNFAIP8, SLCO3A1, SGK1, SATB2, PSMP, PPOX, POLR2A, PBXIP1, OTP, OSR2, MYNN, MTA2, MLLT11, LRRFIP1, ITGB1, HOXA6, HNRNPL, FMNL1, EFNB2, DVL2, DBP, CRIP1, CKS1B, CD68, CBX4, CASP2, BMF, ANP32A, ANG, ADM, ADD3, TTF2, TAP2, TAGLN2, SUFU, SLC39A1, SLC38A2, SILV, SHMT2, S100A4, LAPTM5, ITPR1, IKBKE, IGF1R, GSK3A, GPS2, ESR1, ENO3, EFNA1, DYRK2, DYRK1A, DUSP5, CCND3, CBX3, BCOR, BCL11A, ARHGDIB, ADIPOR1, WDR1, WBP7, UNCX, TPP1, SOCS3, SLC4A2, S1PR1, RPL19, RHOC, RAB13, POU2F1, PAX9, NP, MGAT5, MAX, LASP1, IL16, HYAL2, HOXD10, HOXA4, GTF3A, GIT1, GABPB1, ENC1, ELK3, EGLN2, CYTIP, CUL1, CTLA4, CTDSP2, CLCN3, CD3D, BAK1, ARID1B, APOA2, WIPF1, VAMP8, UBC, TRIM3, TRAF3, SOCS2, SNAPC5, SMAD4, RPS18, REPIN1, POU3F3, OTX1, LRDD, HSP90B1, GAD1, EFNA3, DAXX, CSF1, CIRBP, BAT1, ARHGAP5, ZC3H12D, ZC3H12A, UGP2, TP53, TLE4, TGFB3, TCF7L2, SNRK, SLC20A1, RTN4, RPS6KA1, REV3L, RB1, PRKCE, PEA15, NR4A1, NF1, MEIS1, MAPRE2, MAL, LCK, KAT2B, H2AFZ, H2AFJ, GGPS1, GABBR1, FUS, FOXO1, FOXN2, FHOD1, FHL3, FBRS, ETV5, EP300, EHMT2, DPH1, DAD1, COL2A1, CEBPE, CDC42EP3, C2orf28, ABI3, ABCF3, ZNF384, TPM3, TG, TBPL1, POLD4, PLEC1, PDGFRA, PDE4B, PBX2, NR2E1, MGA, LPIN1, HIST1H1E, HIST1H1A, HIF1AN, HCLS1, GAPDH, EMP3, ELF5, EIF5A, DCTD, CCND1, CAMK2G, CACNB3, ALCAM, ADAMTS4, ACVR2A, ABCD2, ZAP70, UBL3, TWIST1, TUBB, TRADD, TPT1, TBX15, TBL1XR1,

SYTL1, STAT6, SIM1, SEMA3B, SART3, QKI, PXN, PTGER2, PIK3CG, PIK3CA, PCGF2, NRGN, MYLIP, MYD88, MT2A, MSI2, MAPK14, KLF2, JAK1, IL6R, IL2RA, IER2, HSPA1A, HOXD3, HOXC11, HNRNPH1, GRB2, GRAP2, FOS, ERBB2IP, ENO2, ELF1, E2F4, E2F2, DUSP10, DLG4, DHH, CXCR5, CREB3, CD79A, CD63, CADM1, ATP1B1, APEX1, ADORA2A, XAB2, WNK1, UCP2, TXNIP, STAT5B, SP4, SOX13, SMEK2, SLC25A22, PTK2B, PPARA, POU2AF1, PML, PHB2, PELP1, PDCD10, PCP2, PCDH17, PARP1, ORC4L, NUMB, NFAT5, MAT2B, LGALS1, LCP2, KRIT1, ITGA6, IRF8, ING1, IKZF3, IDI1, HSP90AB1, HNRNPK, HECA, HDGF, GIT2, FOXP4, FOXO3, FOSB, ERGIC2, EIF4A1, EDAR, DR1, CFLAR, CD82, CD40, CCNL1, CAMK2D, BHLHB3, BCLAF1, ATF2, ZYX, ZNF274, ZIC4, ZFP161, YWHAZ, VGF, TSC22D1, TRAF1, TOB1, TGFBR3, TBK1, TAF12, STK38L, SSRP1, SSBP1, SRI, SLC26A8, SIL1, SETD1A, SESN1, SERPINI1, SEPT7, SEPHS1, RGS10, RAF1, PRG4, PPP3R1, POFUT1, PLAGL2, PHIP, PEG10, PDGFB, PCSK7, OTUD4, NR1H2, NOS2, NFIL3, NBPF1, MLC-B, MED21, MCAM, MAPKAPK2, MAP3K5, LIG4, KLF5, KLF13, JARID1B, IVNS1ABP, ISYNA1, IMPDH2, IL2, IGF2BP3, HSPE1, HSPD1, HS2ST1, HNRNPD, HIPK3, HIC1, GNB2L1, FRAS1, FBXW11, FAM110A, EIF4A2, EFNA4, DEDD, CXCR7, CHIC2, CD69, CD47, CD27, CALR, CALM2, CALM1, BTAF1, BRCA2, BACH1, ATF4, AQP3, AHSA1, ABHD2, ABCG1, ABCF2, ZNF260, YES1, WWP1, WASF1, USO1, UEVLD, TOX, TNFSF8, TNFRSF1B, TMSB4X, TGFBRAP1, TAX1BP1, ST6GAL1, SSR1, SRCAP, SPTBN1, SPRY4, SNRPA, SIPA1, SFRS2IP, SEP15, SELPLG, RPS9, RPL4, RPL37, RPL28, ROCK1, RNF24, RGS16, RBM12, PUM1, PTTG1, PTGES3, PRMT1, PRKCD, PRCP, PPARGC1A, PNKD, PKNOX2, PHLDB1, PFKFB2, PER2, PARP14, OPTN, NUP37, NRN1, NCOA4, NAB1, MRPS6, MIA, MAPK13, MAP3K8, MAP2K6, LTBP2, LRBA, LMNA, LLGL2, LDLR, KREMEN2, KRAS, ITPR2, ITGA1, IMP3, IL10RA, IGF1, IFNGR1, IFITM2, IFITM1, IFIH1, HYAL3, HS6ST1, HNRNPU, HMGN3, HCRT, H3F3A, GSX1, GPR183, GLIPR1, FUBP1, FBL, FAM102A, EXOSC7, ESRRA, EPB41, EEF1A1, ECE1, DUSP4, DOCK10, DNAJC14, DDX42, DDX1, DDIT4, DBF4, CST6, CSRP1, CSNK1A1, CRIP2, CPT1A, CPNE1, CNTFR, CLDN12, CHD6, CEP57, CDC25B, CD53, CD200R1, CCR6, CBX1, CAD, C7orf23, BRAF, BBC3, BANF1, ATP5G2, ATP5B, ASXL1, ARMCX2, ANXA1, ADIPOR2, ACSL5

Corresponding total number of TFBS:
(For each gene listed above, the total number of TFBS for any of the selected TFs, multiplied by the number of selected Gene ontology terms containing that gene)
176, 132, 132, 121, 110, 110, 108, 108, 104, 99, 96, 90, 88, 88, 84, 84, 84, 80, 77, 77, 77, 72, 72, 72, 72, 72, 72, 72, 72, 72, 70, 66, 66, 66, 64, 63, 60, 60, 60, 60, 56, 55, 55, 54, 54, 54, 50, 50, 50, 49, 48, 48, 48, 48, 48, 48, 48, 48, 48, 48, 48, 48, 48, 48, 48, 45, 45, 44, 44, 44, 44, 44, 44, 44, 44, 44, 42, 42, 40, 40, 40, 36, 36, 36, 36, 36, 36, 36, 36, 36, 36, 36, 36, 36, 36, 36, 36, 36, 36, 36, 36, 36, 35, 35, 33, 33, 33, 33, 33, 33, 33, 33, 33, 33, 30, 30, 30, 30, 30, 30, 30, 30, 28, 28, 28, 28, 28, 28, 28, 28, 27, 26, 26, 25, 25, 25, 24, 24, 24, 24, 24, 24, 24, 24, 24, 24, 24, 24, 24, 24, 24, 24, 24, 24, 24, 24, 24, 24, 24, 24, 24, 24, 24, 24, 24, 24, 22, 22, 22, 22, 22, 22, 22, 22, 22, 22, 22, 22, 22, 22, 21, 21, 21, 21, 21, 21, 20, 20, 20, 20, 20, 20, 20, 20, 20, 20, 20, 20, 20, 20, 20, 20, 20, 20, 18, 18, 18, 18, 18, 18, 18, 18, 18, 18, 18, 18, 18, 18, 16, 16, 16, 16, 16, 16, 16, 16, 15, 15, 15, 15, 15, 14, 14, 14, 14, 14, 14, 14, 14, 14, 12, 12, 12, 12, 12, 12, 12, 12, 12, 12, 12, 12, 12, 12, 12, 12, 12, 12, 12, 12, 12, 12, 12, 12, 12, 12, 12, 12, 12, 12, 12, 12, 12, 12, 12, 12, 12, 12, 12, 12, 12, 12, 12, 12, 12, 12, 12, 12, 12, 12, 12, 12, 12, 12, 12, 12, 12, 12, 12, 12, 12, 11, 11, 11, 11, 11, 11, 11, 11, 11, 11, 11, 11, 11, 11, 11, 10, 10, 10, 10, 10, 10, 10, 10, 10, 10, 10, 10, 10, 10, 10, 10, 10, 10, 10, 10, 10, 10, 10, 10, 10, 10, 10, 10, 10, 10, 10, 10, 9, 9, 9, 9, 9, 9, 9, 9, 9, 9, 9, 9, 9, 9, 9, 9, 9, 9, 9, 9, 9, 9, 9, 9, 9, 9, 9, 8, 8, 8, 8, 8, 8, 8, 8, 8, 8, 8, 8, 8, 8, 8, 8, 8, 8, 8, 8, 8, 8, 8, 8, 8, 8, 8, 8, 8, 8, 8, 8, 8, 8, 8, 7, 7, 7, 7, 7, 7, 7, 7, 7, 7, 7, 7, 7, 7, 7, 7, 7, 7, 7, 7, 7, 6, 6, 6, 6, 6, 6, 6, 6, 6, 6, 6, 6, 6, 6, 6, 6, 6, 6, 6, 6, 6, 6, 6, 6, 6, 6, 6, 6, 6, 6, 6, 6, 6, 6, 6, 6, 6, 6, 6, 6, 6, 6, 6, 5, 5, 5, 5, 5, 5, 5, 5, 5, 5, 5, 5, 5, 5, 5, 5, 5, 5, 5, 5, 5, 5, 5, 5, 5, 5, 5, 5, 4, 4, 4, 4, 4, 4, 4, 4, 4, 4, 4, 4, 4, 4, 4, 4, 4, 4, 4, 4, 4, 4, 4, 4, 4, 4, 4, 4, 4, 4, 4, 4, 4, 4, 4, 4, 4, 4, 4, 4, 4, 4, 4, 4, 4, 4, 4, 4, 4, 4, 4, 4, 4, 3, 3, 3, 3, 3, 3, 3, 3, 3, 3, 3, 3, 3, 3, 3, 3, 3, 3, 3, 3, 3, 3, 3, 3, 3, 3, 3, 3, 3, 3, 3, 3, 3, 3, 3, 3, 3, 3, 3, 3, 3, 3, 3, 3, 3, 3, 3, 3, 3, 3, 3, 2, 2, 2, 2, 2, 2, 2, 2, 2, 2, 2, 2, 2, 2, 2, 2, 2, 2, 2, 2, 2, 2, 2, 2, 2, 2, 2, 2, 2, 2, 2, 2, 2, 2, 2, 2, 2, 2, 2, 2, 2, 2, 2, 2, 2, 2, 2, 2, 2, 2, 2, 2, 2, 2, 2, 2, 2, 2, 2, 2, 2, 2, 2, 2, 2, 2, 2, 2, 2, 2, 2, 2, 2, 2, 2, 2, 2, 2, 2, 2, 2, 2, 1

Corresponding number of selected Gene ontology terms each gene is involved with:

(In the same order as above)
16, 11, 11, 11, 10, 10, 9, 12, 13, 9, 8, 10, 8, 8, 7, 7, 7, 8, 7, 7, 7, 6, 6, 6, 6, 6, 6, 6, 8, 6, 7, 6, 6, 11, 8, 9, 5, 6, 5, 6, 8, 5, 5, 6, 9, 6, 5, 5, 5, 7, 4, 12, 4, 4, 4, 4, 4, 4, 4, 4, 4, 4, 4, 4, 4, 5, 5, 4, 4, 4, 4, 4, 4, 4, 4, 4, 6, 7, 5, 10, 5, 3, 3, 4, 3, 3, 3, 3, 3, 9, 3, 3, 4, 3, 4, 4, 3, 4, 4, 3, 3, 4, 7, 5, 3, 3, 3, 3, 3, 3, 3, 3, 3, 3, 6, 3, 3, 6, 5, 3, 3, 6, 4, 4, 4, 7, 4, 7, 4, 7, 3, 13, 13, 5, 5, 5, 2, 3, 2, 6, 2, 2, 2, 2, 2, 8, 2, 2, 2, 2, 4, 3, 2, 2, 2, 2, 6, 2, 6, 2, 6, 3, 2, 2, 2, 2, 2, 2, 11, 2, 2, 2, 2, 2, 2, 2, 2, 2, 2, 2, 7, 3, 7, 7, 3, 3, 2, 5, 2, 2, 4, 2, 2, 2, 2, 4, 4, 2, 4, 2, 2, 5, 2, 5, 3, 6, 3, 2, 9, 2, 2, 2, 3, 2, 3, 2, 2, 3, 2, 2, 2, 8, 2, 2, 2, 2, 3, 3, 3, 3, 3, 2, 2, 2, 2, 7, 2, 2, 7, 2, 1, 1, 3, 1, 1, 1, 1, 6, 1, 1, 1, 1, 1, 2, 1, 1, 1, 2, 3, 1, 3, 1, 1, 12, 4, 1, 1, 1, 3, 1, 1, 1, 1, 3, 1, 1, 1, 1, 3, 4, 1, 1, 1, 1, 2, 1, 4, 2, 4, 1, 1, 1, 1, 2, 1, 1, 1, 1, 1, 2, 1, 1, 1, 1, 1, 1, 1, 1, 1, 1, 1, 1, 1, 1, 1, 1, 1, 1, 1, 2, 1, 1, 1, 1, 2, 1, 1, 1, 1, 5, 1, 1, 1, 5, 2, 2, 1, 1, 1, 1, 2, 1, 1, 1, 1, 1, 9, 1, 1, 3, 1, 1, 1, 1, 1, 1, 1, 1, 3, 1, 1, 3, 1, 3, 1, 3, 1, 1, 1, 1, 1, 1, 3, 2, 2, 4, 2, 2, 1, 1, 1, 1, 2, 4, 8, 1, 1, 2, 1, 1, 2, 4, 2, 8, 1, 2, 1, 1, 2, 1, 2, 1, 1, 1, 1, 2, 2, 2, 1, 1, 7, 1, 1, 1, 1, 7, 1, 1, 1, 7, 1, 1, 1, 1, 1, 1, 1, 1, 1, 1, 1, 1, 6, 6, 3, 6, 1, 6, 1, 3, 2, 6, 1, 3, 3, 3, 6, 1, 1, 2, 3, 2, 1, 1, 1, 3, 3, 2, 1, 1, 1, 3, 6, 3, 2, 2, 6, 3, 1, 2, 3, 1, 1, 5, 1, 5, 1, 1, 5, 1, 5, 5, 1, 1, 1, 1, 1, 1, 1, 1, 5, 1, 1, 5, 1, 1, 1, 1, 5, 1, 1, 1, 4, 1, 2, 1, 2, 1, 4, 2, 4, 4, 1, 1, 4, 1, 4, 4, 1, 1, 2, 2, 2, 1, 2, 2, 2, 2, 1, 1, 2, 4, 2, 1, 2, 1, 4, 1, 1, 2, 2, 2, 1, 1, 4, 2, 2, 1, 1, 1, 1, 2, 1, 1, 3, 1, 1, 3, 3, 3, 1, 1, 1, 3, 3, 3, 1, 3, 1, 1, 3, 3, 1, 1, 3, 1, 3, 1, 3, 3, 3, 1, 1, 1, 1, 3, 1, 3, 1, 1, 3, 3, 3, 1, 3, 3, 1, 1, 3, 1, 1, 3, 1, 3, 1, 2, 1, 1, 1, 2, 1, 2, 1, 1, 2, 2, 2, 2, 2, 1, 1, 1, 1, 1, 1, 2, 1, 1, 2, 1, 1, 1, 1, 2, 1, 1, 1, 1, 2, 2, 2, 2, 1, 2, 1, 1, 2, 2, 2, 2, 1, 1, 2, 1, 2, 1, 2, 2, 2, 1, 1, 1, 1, 2, 2, 1, 1, 1, 2, 1, 2, 1, 1, 1, 1, 1, 2, 2, 2, 2, 1, 2, 1

Corresponding number of selected TFs each gene is involved with:
(In the same order as above)
11, 12, 12, 11, 11, 11, 12, 9, 8, 11, 12, 9, 11, 11, 12, 12, 12, 10, 11, 11, 11, 12, 12, 12, 12, 12, 12, 12, 9, 12, 10, 11, 11, 6, 8, 7, 12, 10, 12, 10, 7, 11, 11, 9, 6, 9, 10, 10, 10, 7, 12, 4, 12, 12, 12, 12, 12, 12, 12, 12, 12, 12, 12, 12, 12, 9, 9, 11, 11, 11, 11, 11, 11, 11, 11, 11, 7, 6, 8, 4, 8, 12, 12, 9, 12, 12, 12, 12, 12, 4, 12, 12, 9, 12, 9, 9, 12, 9, 9, 12, 12, 9, 5, 7, 11, 11, 11, 11, 11, 11, 11, 11, 11, 11, 5, 10, 10, 5, 6, 10, 10, 5, 7, 7, 7, 4, 7, 4, 7, 4, 9, 2, 2, 5, 5, 5, 12, 8, 12, 4, 12, 12, 12, 12, 12, 3, 12, 12, 12, 12, 6, 8, 12, 12, 12, 12, 4, 12, 4, 12, 4, 8, 12, 12, 12, 12, 11, 11, 2, 11, 11, 11, 11, 11, 11, 11, 11, 11, 11, 11, 3, 7, 3, 3, 7, 7, 10, 4, 10, 10, 5, 10, 10, 10, 10, 5, 5, 10, 5, 10, 10, 4, 10, 4, 6, 3, 6, 9, 2, 9, 9, 9, 6, 9, 6, 9, 9, 6, 8, 8, 8, 2, 8, 8, 8, 8, 5, 5, 5, 5, 5, 7, 7, 7, 7, 2, 7, 7, 2, 7, 12, 12, 4, 12, 12, 12, 12, 2, 12, 12, 12, 12, 12, 6, 12, 12, 12, 6, 4, 12, 4, 12, 12, 1, 3, 12, 12, 12, 4, 12, 12, 12, 12, 4, 12, 12, 12, 12, 4, 3, 12, 12, 12, 12, 6, 12, 3, 6, 3, 12, 12, 12, 12, 6, 12, 12, 12, 12, 12, 6, 12, 11, 11, 11, 11, 11, 11, 11, 11, 11, 11, 11, 11, 11, 11, 11, 10, 10, 10, 10, 10, 5, 10, 10, 10, 10, 5, 10, 10, 10, 10, 2, 10, 10, 10, 2, 5, 5, 10, 10, 10, 10, 5, 10, 10, 10, 10, 10, 1, 9, 9, 3, 9, 9, 9, 9, 9, 9, 9, 9, 3, 9, 9, 3, 9, 3, 9, 3, 9, 9, 9, 9, 9, 9, 3, 4, 4, 2, 4, 4, 8, 8, 8, 8, 4, 2, 1, 8, 8, 4, 8, 8, 4, 2, 4, 1, 8, 4, 8, 8, 4, 8, 4, 8, 8, 8, 8, 4, 4, 4, 7, 7, 1, 7, 7, 7, 7, 1, 7, 7, 7, 1, 7, 7, 7, 7, 7, 7, 7, 7, 7, 6, 6, 6, 1, 1, 2, 1, 6, 1, 6, 2, 3, 1, 6, 2, 2, 2, 1, 6, 6, 3, 2, 3, 6, 6, 6, 2, 2, 3, 6, 6, 6, 2, 1, 2, 3, 3, 1, 2, 6, 3, 2, 6, 5, 1, 5, 1, 5, 5, 1, 5, 1, 1, 5, 5, 5, 5, 5, 5, 5, 5, 1, 5, 5, 1, 5, 5, 5, 5, 1, 5, 4, 4, 1, 4, 2, 4, 2, 4, 1, 2, 1, 1, 4, 4, 1, 4, 1, 1, 4, 4, 2, 2, 2, 4, 2, 2, 2, 2, 4, 4, 2, 1, 2, 4, 2, 4, 1, 4, 4, 2, 2, 2, 4, 4, 1, 2, 2, 4, 4, 4, 4, 2, 4, 3, 1, 3, 3, 1, 1, 1, 3, 3, 3, 1, 1, 1, 3, 1, 3, 3, 1, 1, 3, 3, 1, 3, 1, 3, 1, 1, 1, 3, 3, 3, 3, 1, 3, 1, 3, 3, 1, 1, 1, 3, 1, 1, 3, 3, 1, 3, 3, 1, 3, 1, 2, 1, 2, 2, 2, 1, 2, 1, 2, 2, 1, 1, 1, 1, 1, 2, 2, 2, 2, 2, 2, 1, 2, 2, 1, 2, 2, 2, 2, 1, 2, 2, 2, 2, 1, 1, 1, 1, 2, 1, 2, 2, 1, 1, 1, 1, 2, 2, 1, 2, 1, 2, 1, 1, 1, 2, 2, 2, 2, 1, 1, 2, 2, 2, 1, 2, 1, 2, 2, 2, 2, 2, 2, 1, 1, 1, 1, 2, 1, 2, 2, 2, 1

Gene ontology terms ranked according to mean of effect sizes in cluster:
organ development | DNA binding | gene expression | embryonic development | limb development | segmentation | chordate embryonic development | regionalization | anatomical structure morphogenesis | cell fate determination | neurogenesis | DBD domain binding | central nervous system development | central nervous system neuron development | gastrulation | somitogenesis

Corresponding mean of effect sizes of each Gene ontology term:
(In the same order as above)
3.05, 2.74, 2.56, 2.51, 2.50, 2.32, 2.29, 2.22, 2.13, 2.06, 1.83, 1.63, 1.46, 1.44, 1.32, 0.97

Corresponding total number of genes of each Gene ontology term:
(In the same order as above)
339, 1.2K, 3.8K, 1.2K, 129, 109, 671, 62, 583, 145, 409, 411, 110, 37, 293, 116

Corresponding number of genes of each Gene ontology term with at least one TFBS (of selected TFs):
(In the same order as above)
89, 281, 679, 250, 30, 35, 146, 25, 120, 42, 94, 111, 24, 9, 60, 26

Corresponding total number of TFBS (of selected TFs) for each Gene ontology term (in all genes):
(In the same order as above)
577, 1627, 3825, 1536, 212, 255, 930, 172, 749, 292, 579, 597, 179, 77, 376, 165

Corresponding number of selected TFs each Gene ontology term is involved with:
(In the same order as above)
12, 12, 12, 12, 12, 12, 12, 12, 12, 12, 12, 12, 12, 12, 12, 12

TFs ranked according to mean of effect sizes in cluster:
V$HMGIY_Q6, V$PAX2_02, V$STAT1_03, V$STAT6_01, V$TST1_01, V$STAT5A_03, V$STAT4_01, V$STAT5A_04, V$HOXA4_Q2, V$NFAT_Q4_01, V$NFAT_Q6, V$CEBP_Q3

Corresponding mean of effect sizes of each TF:
(In the same order as above)
2.82, 2.65, 2.55, 2.44, 2.39, 2.18, 2.01, 1.96, 1.62, 1.61, 1.58, 0.96

Corresponding total number of TFBS for each TF (genome-wide):
(In the same order as above)
753, 741, 751, 732, 730, 749, 743, 740, 719, 736, 719, 744

Corresponding total number of TFBS for each TF (in all genes in selected Gene ontology terms):
(In the same order as above)
1092, 1096, 1023, 1051, 1031, 1020, 1026, 940, 949, 981, 1011, 928

Corresponding number of genes (of selected Gene ontology terms) each TF is involved with:
(In the same order as above)
388, 387, 368, 390, 370, 380, 384, 359, 368, 371, 373, 348

Corresponding number of selected Gene ontology terms each TF is involved with:
(In the same order as above)
16, 16, 16, 16, 16, 16, 16, 16, 16, 16, 16, 16

Cluster for columns 1 to 6, rows 176 to 186

Gene ontology terms

peptide transport | Hedgehog signaling complex | hemopoiesis | cell differentiation | T cell differentiation | cell development

TFs

V$MYB_Q5_01 | P$GBP_Q6 | V$MYB_Q3 | V$TFIIA_Q6 | V$RFX_Q6 | V$TEF1_Q6 | V$STAT6_02 | V$STAT3_02 | V$NCX_01 | V$PXRRXR_02 | V$PXRRXR_01

Information

All related TFs:
(List of all TFs that are related to any of the PWMs)
B-Myb, NCX, Ncx, RFX1, RFX1:RFX2, RFX1:RFX3, RFX2, RFX3, RFX4, RFX5, RFX5:RFXAP:RFXANK, RFXANK, RFXAP, STAT3, STAT6, TEF-1, TFIIA, TFIIA-alpha/beta, TFIIA-gamma, c-Myb, c-Myb-isoform1

Ranked gene list:
(All genes of the selected Gene ontology terms with hits of any of the selected TFs, ranked according to the total number of TFBS)
GFI1, CXCR4, CDKN1B, FASN, CD4, SSSCA1, SH2B3, RUNX3, TNF, LBX1, LEF1, GATA2, NOTCH1, TCF7, PTPN6, NFATC1, IRF1, HOXA9, GATA3, FLI1, CISH, CCND2, AKT1, ZFPM1, SHC1, HOXA10, FLII, CCR7, LYL1, ITGA5, EGR1, SOCS1, HOXB4, RARA, PAX6, OSM, NR4A2, DLL1, CSK, CD68, BCL6, BCL11B, ATXN1, ID2, SMAD7, NKX2-2, LCK, FLJ32987, BCL2L1, SOCS3, GLI1, DLL4, VEGFA, TNFRSF4, MLL5, HES5, DNMT3A, STAT5A, RUNX1, ETS1, POU2F2, LMO4, ITGB2, IKZF1, ID3, HOXB3, HES1, ZBTB7B, WNT10B, TCIRG1, STK16, RORC, RARG, PTMA, PSMB8, PLEKHG2, NR4A1, MAP3K12, LTB, LFNG, LDB1, JUNB, HOXC9, HOXA7, HOXA11, CRIP2, CRIP1, CFL1, CDK5, CDH23, ACTB, ABCB9, TNFSF13, TNFRSF18, STRA13, RPS6KA1, RING1, NFATC3, MLL, EOMES, CDK4, BMI1, BHLHB2, ARHGEF1, TGFB1, TAPBP, SMAD3, RPS6KB2, PSMB10, PAX5, MDK, LTA, IRS1, HOXB6, BCL2L11, ZAP70, NR3C2, LAG3, JUND, IL11RA, E2F4, CDKN2C, TAP1, PITX3, MAL, HSP90AB1, HOXC6, HOXC5, HOXC4, BAK1, TLR9, SATB1, NOTCH4, NGFR, JUN, ING1, HRAS, GGNBP2, FGF9, FAM65B, CD79A, CD3G, CCND3, TNFRSF6B, FOSL2, CXXC1, CD44, CALR, UNK, SOCS2, PSMB9, PCGF2, PARD6A, NFIA, MZF1, MCAM, LMNA, FOXJ1, ENO2, EBF1, DUSP6, DAXX, CDK6, C2orf28, BCL11A, ASB2, ZNF385A, VIM, TBX21, TAP2, SETD1A, PSMC5, PBX2, NDRG1, INPP5D, HAND2, CSF1, CD3D, C21orf33, VDR, TSPAN32, SP1, PRDM1, POU4F1, PIM1, ONECUT1, NEUROG2, MYD88, MAFG, LEPREL2, KLF2, IRF4, IL23A, IL17C, IGF1R, HIST1H4I, HBA2, GADD45G, FYN, CORO1A, CD81, CCND1, BACH2, ANXA6, AMH, ALCAM, AKTIP, UBE2I, TRIM28, TRAF3, TFAP2A, TAF8, SOX4, SERPINH1, SDCBP2, RASGRP1, PKNOX1, PCNA, MUC1, MMP14, MAFA, LHX9, LGALS1, KLF7, HOXA1, HIPK3, GSK3B, FIT1, FGR, EDC4, E2F2, CUX1, CIAPIN1, CHRNE, CDK2, CBX5, BTG2

Corresponding total number of TFBS:
(For each gene listed above, the total number of TFBS for any of the selected TFs, multiplied by the number of selected Gene ontology terms containing that gene)
55, 55, 50, 45, 45, 44, 44, 44, 40, 40, 36, 36, 35, 33, 33, 33, 33, 33, 33, 33, 33, 33, 33, 30, 30, 30, 30, 30, 27, 27, 27, 24, 24, 22, 22, 22, 22, 22, 22, 22, 22, 22, 22, 21, 20, 20, 20, 20, 20, 18, 18, 18, 16, 16, 16, 16, 16, 15, 15, 15, 14, 12, 12, 12, 12, 12, 12, 11, 11, 11, 11, 11, 11, 11, 11, 11, 11, 11, 11, 11, 11, 11, 11, 11, 11, 11, 11, 11, 11, 11, 11, 11, 10, 10, 10, 10, 10, 10, 10, 10, 10, 10, 10, 9, 9, 9, 9, 9, 9, 9, 9, 9, 9, 9, 8, 8, 8, 8, 8, 8, 8, 7, 7, 7, 7, 7, 7, 7, 7, 6, 6, 6, 6, 6, 6, 6, 6, 6, 6, 6, 6, 6, 5, 5, 5, 5, 5, 4, 4, 4, 4, 4, 4, 4, 4, 4, 4, 4, 4, 4, 4, 4, 4, 4, 4, 3, 3, 3, 3, 3, 3, 3, 3, 3, 3, 3, 3, 3, 2, 2, 2, 2, 2, 2, 2, 2, 2, 2, 2, 2, 2, 2, 2, 2, 2, 2, 2, 2, 2, 2, 2, 2, 2, 2, 2, 2, 1, 1, 1, 1, 1, 1, 1, 1, 1, 1, 1, 1, 1, 1, 1, 1, 1, 1, 1, 1, 1, 1, 1, 1, 1, 1, 1, 1

Corresponding number of selected Gene ontology terms each gene is involved with:
(In the same order as above)
5, 5, 5, 5, 5, 4, 4, 4, 4, 5, 4, 4, 5, 3, 3, 3, 3, 3, 3, 3, 3, 3, 3, 3, 3, 3, 3, 3, 3, 3, 3, 3, 2, 2, 2, 2, 2, 2, 2, 2, 2, 2, 3, 2, 2, 5, 2, 5, 2, 2, 3, 4, 2, 2, 2, 2, 5, 5, 3, 2, 2, 4, 4, 2, 3, 4, 1, 1, 1, 1, 1, 1, 1, 1, 1, 1, 1, 1, 1, 1, 1, 1, 1, 1, 1, 1, 1, 1, 1, 1, 1, 1, 5, 1, 1, 1, 1, 1, 1, 1, 1, 2, 1, 1, 3, 1, 3, 1, 1, 3, 1, 1, 1, 1, 1, 2, 1, 1, 2, 1, 1, 2, 1, 1, 1, 1, 1, 1, 1, 1, 1, 3, 3, 3, 3, 1, 1, 1, 1, 1, 2, 2, 2, 1, 1, 1, 5, 1, 1, 2, 1, 1, 1, 1, 1, 1, 1, 1, 1, 4, 1, 1, 1, 2, 1, 1, 1, 1, 3, 1, 1, 1, 1, 1, 1, 1, 3, 1, 3, 1, 1, 2, 2, 1, 2, 1, 1, 2, 1, 1, 2, 2, 2, 2, 1, 1, 2, 2, 2, 2, 2, 2, 1, 2, 2, 1, 1, 1, 1, 1, 1, 1, 1, 1, 1, 1, 1, 1, 1, 1, 1, 1, 1, 1, 1, 1, 1, 1, 1, 1, 1, 1, 1, 1, 1

Corresponding number of selected TFs each gene is involved with:
(In the same order as above)
11, 11, 10, 9, 9, 11, 11, 11, 10, 8, 9, 9, 7, 11, 11, 11, 11, 11, 11, 11, 11, 11, 11, 10, 10, 10, 10, 10, 9, 9, 9, 8,

8, 11, 11, 11, 11, 11, 11, 11, 11, 11, 11, 7, 10, 10, 4, 10, 4, 9, 9, 6, 4, 8, 8, 8, 8, 3, 3, 5, 7, 6, 3, 3, 6, 4, 3, 11, 11, 11, 11, 11, 11, 11, 11, 11, 11, 11, 11, 11, 11, 11, 11, 11, 11, 11, 11, 11, 11, 11, 2, 10, 10, 10, 10, 10, 10, 10, 10, 5, 10, 10, 3, 9, 3, 9, 9, 3, 9, 9, 9, 9, 9, 4, 8, 8, 4, 8, 8, 4, 7, 7, 7, 7, 7, 7, 7, 7, 6, 2, 2, 2, 2, 6, 6, 6, 6, 6, 3, 3, 3, 5, 5, 5, 1, 5, 4, 2, 4, 4, 4, 4, 4, 4, 4, 4, 4, 1, 4, 4, 4, 2, 4, 4, 3, 3, 1, 3, 3, 3, 3, 3, 3, 3, 1, 3, 1, 2, 2, 1, 1, 2, 1, 2, 2, 1, 2, 2, 1, 1, 1, 1, 2, 2, 1, 1, 1, 1, 1, 2, 1, 1, 2, 2, 1, 1, 1, 1, 1, 1, 1, 1, 1, 1, 1, 1, 1, 1, 1, 1, 1, 1, 1, 1, 1, 1, 1, 1, 1, 1, 1, 1

Gene ontology terms ranked according to mean of effect sizes in cluster:
hemopoiesis | cell development | Hedgehog signaling complex | peptide transport | cell differentiation | T cell differentiation

Corresponding mean of effect sizes of each Gene ontology term:
(In the same order as above)
2.24, 2.03, 1.98, 1.89, 1.88, 1.46

Corresponding total number of genes of each Gene ontology term:
(In the same order as above)
343, 507, 230, 32, 871, 172

Corresponding number of genes of each Gene ontology term with at least one TFBS (of selected TFs):
(In the same order as above)
80, 100, 49, 9, 159, 40

Corresponding total number of TFBS (of selected TFs) for each Gene ontology term (in all genes):
(In the same order as above)
497, 622, 319, 69, 932, 251

Corresponding number of selected TFs each Gene ontology term is involved with:
(In the same order as above)
11, 11, 11, 11, 11, 11

TFs ranked according to mean of effect sizes in cluster:
V$PXRRXR_01, V$PXRRXR_02, V$STAT3_02, V$STAT6_02, V$TEF1_Q6, V$MYB_Q5_01, VMYB_Q3, VNCX_01, PGBP_Q6, VRFX_Q6, V$TFIIA_Q6

Corresponding mean of effect sizes of each TF:
(In the same order as above)
2.43, 2.32, 2.11, 2.09, 2.04, 1.88, 1.79, 1.73, 1.61, 1.56, 1.46

Corresponding total number of TFBS for each TF (genome-wide):
(In the same order as above)
723, 727, 718, 737, 722, 693, 691, 701, 699, 713, 691

Corresponding total number of TFBS for each TF (in all genes in selected Gene ontology terms):
(In the same order as above)
267, 261, 263, 279, 261, 237, 237, 223, 223, 218, 221

Corresponding number of genes (of selected Gene ontology terms) each TF is involved with:
(In the same order as above)
137, 133, 133, 145, 134, 122, 116, 117, 110, 115, 130

Corresponding number of selected Gene ontology terms each TF is involved with:
(In the same order as above)
6, 6, 6, 6, 6, 6, 6, 6, 6, 6, 6

Cluster for columns 757 to 784, rows 147 to 150

Gene ontology terms

Janus kinase activity | macrophage differentiation | protein tyrosine kinase inhibitor activity | transmembrane receptor protein tyrosine kinase activity | protein tyrosine kinase activator activity | protein tyrosine kinase activity | focal adhesion | platelet-derived growth factor receptor activity | platelet-derived growth factor receptor binding | 1-phosphatidylinositol-3-kinase activity | epidermal growth factor receptor activity | epidermal growth factor receptor binding | SAP kinase activity | protein kinase cascade | JUN kinase activity | mitogen-activated protein kinase p38 binding | MAPKKK cascade | MAP kinase 1 activity | MAP kinase 2 activity | activation of MAPKKK activity | activation of MAPK activity | mitogen-activated protein kinase kinase kinase kinase binding | mitogen-activated protein kinase kinase binding | mitogen-activated protein kinase kinase kinase binding | MAP kinase kinase activity | MAP kinase kinase kinase activity | MAP kinase activity | MAP kinase kinase kinase kinase activity

TFs

V$TEL2_Q6 | V$ETS_Q4 | V$ELF1_Q6 | V$NKX25_Q5

Information

All related TFs:
(List of all TFs that are related to any of the PWMs)
ELF-1, ERF, Elf-1, Elk-1, Erg-1, Ets-1, Fli-1, NERF, NERF-1a, NERF-1b, NERF-2, Nkx2-5, Nkx2.5, SAP-1a, Tel-2a, Tel-2b, Tel-2c, c-Ets-1, c-Ets-2

Ranked gene list:
(All genes of the selected Gene ontology terms with hits of any of the selected TFs, ranked according to the total number of TFBS)
SHC1, JUNB, AKT1, CSK, TNF, EGR1, LCK, RPS6KA1, PTPN11, PTPN6, MAP2K3, PTK2B, PIK3R1, IRS1, PKN1, NTRK1, SSSCA1, MMP9, COL11A2, ERBB2, ZAP70, PLCG1, JUN, FASN, STAT3, RAF1, MAPK14, GRB2, RAC2, FGR, CXCR4, CAMK2G, PIK3R2, CANX, ARHGEF7, JAK2, CDKN1B, TNFRSF1A, TFRC, SOCS3, HRAS, EPHA1, LYN, ZC3H12D, STAT5A, ILK, CISH, CDK5, CDK4, CCND3, CASP8, ANXA6, STAT5B, CREB1, STK24, PTPN7, PRKCD, PIK3CG, MAP3K1, IRF1, RAP1GAP, MAPKAPK3, MAPK11, LIF, CSF1, VAV1, PLA2G6, MAPK12, MAP4K1, MAP3K5, ITGB2, KAT5, SORBS3, PRKCA, MOCOS, MARK2, FYN, DLG4, AKT2, ADRBK1, SLC33A1, EIF4EBP1, BAX, VAV2, SART3, NFKBIA, CDK2, TNK2, SOCS1, NFATC1, NCK1, MAP3K12, LTBR, FAS, CCND2, TUSC2, THPO, SLC9A3R1, RASSF1, NLRP3, FOXO3, DUSP5, CD2BP2, BAK1, ARRB2, ARHGAP4, GGPS1, ZFP36, TYROBP, TXNDC12, TLR9, SMAD3, RGS3, PSMP, PRIM2, PDE4A, NR4A1, NR2C2, MMP14, LCP2, ITGA5, INPP5D, FLJ32987, DUSP2, DAPP1, CD82, ARHGEF2, TNFRSF1B, SPRY2, MAPK10, IL2RB, TRAF1, STAT6, STAT4, RIN1, RARA, PTGER4, NR4A2, NFKBIE, NAB2, MEF2D, MAP3K11, LMNA, ITGAV, GRN, GATA3, EPHA2, CTTN, CTSD, ARHGEF1, ACVR1, ACTB, VIM, TRAF5, TLN1, SH3BP2, SELPLG, RHOG, RALB, PPP1R9B, PLCB1, PEA15, PDLIM7, NFKB1, LASP1, KISS1R, JUND, IRF9, IL23A, GIT1, GADD45A, ETV6, DGAT1, DCTN3, CTF1, CCR7, AGTRAP, ADAMTS4, ZYX, UBE2B, TSG101, TRIP11, TRADD, TPP1, TNFRSF6B, SUB1, STAM2, SNX13, SNX1, SLC2A4RG, RHOC, RERE, RAP1B, PSMC5, PRF1, PRDM1, PKN2, PIK3CD, PHB2, PDK2, NFATC3, NDOR1, MUC4, MR1, MIA, MAP4K5, LSM4, KRT13, KIAA0101, HCST, GPS2, GLI1, GFI1, FURIN, FERMT3, ERBB2IP, EGLN2, ECE1, DVL2, DIAPH1, DIABLO, DDIT3, DBNL, CXCR5, CRTC2, CDK9, CD68, CD19, CCBL1, CALR, BRMS1, BRCA1, ATF3, ARHGAP1, ACTN1, ABI1, WNT10B, WNT10A, WDR1, WASF2, VASP, TXNIP, TSC2, TM7SF2, SPEN, SOCS7, SLC5A5, SH2B2, SELL, RIPK3, RASGRP2, PRKCB, PLEKHA2, PIK3IP1, PARVG, OSR1, NPTN, NF1, NCOR2, NCF4, NCCRP1, MZF1, MPZ, LHX1, LGALS8, KHSRP, KCNA3, IRAK4, IL1RAP, HSP90B1, FOXP1, FOSL2, FHL3, ERN1, EGR4,

DUSP22, DUSP16, DOCK2, DFFA, DCTN5, CTLA4, CLIP1, CD180, CCL27, C9orf95, BANF1, ARL6IP5, ARHGEF12, ARAP3, AKT1S1, AIF1, ACAA2, ABCF2

Corresponding total number of TFBS:
(For each gene listed above, the total number of TFBS for any of the selected TFs, multiplied by the number of selected Gene ontology terms containing that gene)
104, 76, 75, 68, 63, 63, 57, 54, 54, 52, 51, 46, 46, 46, 45, 44, 36, 36, 33, 30, 27, 27, 27, 27, 26, 26, 25, 25, 24, 24, 24, 24, 23, 23, 23, 22, 22, 21, 20, 20, 20, 20, 19, 18, 18, 18, 18, 18, 18, 18, 18, 18, 17, 17, 16, 16, 16, 16, 16, 16, 15, 15, 15, 15, 15, 14, 14, 14, 14, 14, 14, 13, 12, 12, 12, 12, 12, 12, 12, 11, 11, 11, 10, 10, 10, 10, 9, 9, 9, 9, 9, 9, 9, 9, 8, 8, 8, 8, 8, 8, 8, 8, 8, 8, 8, 7, 6, 6, 6, 6, 6, 6, 6, 6, 6, 6, 6, 6, 6, 6, 6, 6, 6, 6, 6, 5, 5, 5, 5, 4, 4, 4, 4, 4, 4, 4, 4, 4, 4, 4, 4, 4, 4, 4, 4, 4, 4, 4, 4, 3, 3, 3, 3, 3, 3, 3, 3, 3, 3, 3, 3, 3, 3, 3, 3, 3, 3, 3, 3, 3, 3, 3, 3, 3, 2, 2, 2, 2, 2, 2, 2, 2, 2, 2, 2, 2, 2, 2, 2, 2, 2, 2, 2, 2, 2, 2, 2, 2, 2, 2, 2, 2, 2, 2, 2, 2, 2, 2, 2, 2, 2, 2, 2, 2, 2, 2, 2, 2, 2, 2, 2, 2, 2, 2, 2, 2, 1, 1, 1, 1, 1, 1, 1, 1, 1, 1, 1, 1, 1, 1, 1, 1, 1, 1, 1, 1, 1, 1, 1, 1, 1, 1, 1, 1, 1, 1, 1, 1, 1, 1, 1, 1, 1, 1, 1, 1, 1, 1, 1, 1, 1, 1, 1, 1, 1, 1, 1

Corresponding number of selected Gene ontology terms each gene is involved with:
(In the same order as above)
26, 19, 25, 17, 21, 21, 19, 18, 18, 13, 17, 23, 23, 23, 15, 22, 12, 12, 11, 15, 9, 9, 27, 9, 26, 26, 25, 25, 8, 6, 12, 6, 23, 23, 23, 22, 11, 7, 20, 10, 20, 10, 19, 6, 18, 6, 6, 6, 6, 6, 9, 18, 17, 17, 8, 4, 8, 16, 16, 4, 5, 5, 15, 5, 5, 14, 7, 14, 7, 7, 7, 13, 3, 12, 12, 4, 12, 4, 6, 4, 11, 11, 11, 5, 5, 10, 10, 3, 3, 3, 9, 3, 3, 9, 3, 2, 4, 2, 2, 4, 4, 4, 4, 4, 2, 4, 7, 2, 3, 6, 3, 6, 3, 6, 3, 3, 3, 6, 6, 3, 2, 6, 3, 2, 3, 2, 2, 5, 5, 5, 5, 4, 4, 4, 2, 1, 4, 1, 1, 2, 1, 4, 2, 2, 2, 1, 2, 4, 2, 1, 2, 2, 3, 3, 1, 1, 1, 1, 1, 1, 3, 3, 3, 3, 1, 1, 3, 1, 3, 1, 3, 3, 3, 3, 1, 1, 3, 1, 1, 1, 2, 2, 2, 1, 2, 2, 2, 2, 2, 1, 1, 2, 1, 1, 1, 1, 2, 2, 1, 1, 1, 1, 2, 2, 2, 2, 2, 2, 2, 1, 1, 2, 1, 2, 1, 1, 1, 1, 1, 1, 1, 2, 1, 2, 1, 2, 1, 1, 1, 1, 2, 2, 2, 1, 1, 2, 1, 1, 1, 1, 1, 1, 1, 1, 1, 1, 1, 1, 1, 1, 1, 1, 1, 1, 1, 1, 1, 1, 1, 1, 1, 1, 1, 1, 1, 1, 1, 1, 1, 1, 1, 1, 1, 1, 1, 1, 1, 1, 1, 1, 1, 1, 1, 1, 1, 1, 1

Corresponding number of selected TFs each gene is involved with:
(In the same order as above)
4, 4, 3, 4, 3, 3, 3, 3, 3, 4, 3, 2, 2, 2, 3, 2, 3, 3, 3, 2, 3, 3, 1, 3, 1, 1, 1, 1, 3, 4, 2, 4, 1, 1, 1, 1, 2, 3, 1, 2, 1, 2, 1, 3, 1, 3, 3, 3, 3, 3, 2, 1, 1, 1, 2, 4, 2, 1, 1, 4, 3, 3, 1, 3, 3, 1, 2, 1, 2, 2, 2, 1, 4, 1, 1, 3, 1, 3, 2, 3, 1, 1, 1, 2, 2, 1, 1, 3, 3, 3, 1, 3, 3, 1, 3, 4, 2, 4, 4, 2, 2, 2, 2, 2, 4, 2, 1, 3, 2, 1, 2, 1, 2, 1, 2, 2, 2, 1, 1, 2, 3, 1, 2, 3, 2, 3, 3, 1, 1, 1, 1, 1, 1, 1, 2, 4, 1, 4, 4, 2, 4, 1, 2, 2, 2, 4, 2, 1, 2, 4, 2, 2, 1, 1, 3, 3, 3, 3, 3, 3, 1, 1, 1, 1, 3, 3, 1, 3, 1, 3, 1, 1, 1, 1, 3, 3, 1, 3, 2, 2, 1, 1, 1, 2, 1, 1, 1, 1, 1, 2, 2, 1, 2, 2, 2, 2, 1, 1, 2, 2, 2, 2, 1, 1, 1, 1, 1, 1, 1, 2, 2, 1, 2, 1, 2, 2, 2, 2, 2, 2, 2, 1, 2, 1, 2, 1, 2, 2, 2, 2, 1, 1, 1, 2, 2, 1, 1, 1, 1, 1, 1, 1, 1, 1, 1, 1, 1, 1, 1, 1, 1, 1, 1, 1, 1, 1, 1, 1, 1, 1, 1, 1, 1, 1, 1, 1, 1, 1, 1, 1, 1, 1, 1, 1, 1, 1, 1, 1, 1, 1, 1, 1, 1, 1, 1, 1, 1, 1, 1

Gene ontology terms ranked according to mean of effect sizes in cluster:
protein tyrosine kinase activity | Janus kinase activity | MAP kinase 2 activity | activation of MAPK activity | protein tyrosine kinase activator activity | MAPKKK cascade | protein tyrosine kinase inhibitor activity | 1-phosphatidylinositol-3-kinase activity | focal adhesion | activation of MAPKKK activity | platelet-derived growth factor receptor activity | mitogen-activated protein kinase kinase kinase kinase binding | mitogen-activated protein kinase kinase kinase binding | mitogen-activated protein kinase kinase binding | transmembrane receptor protein tyrosine kinase activity | MAP kinase 1 activity | MAP kinase activity | mitogen-activated protein kinase p38 binding | MAP kinase kinase kinase kinase activity | platelet-derived growth factor receptor binding | macrophage differentiation | MAP kinase kinase kinase activity | epidermal growth factor receptor binding | epidermal growth factor receptor activity | MAP kinase kinase activity | JUN kinase activity | protein kinase cascade | SAP kinase activity

Corresponding mean of effect sizes of each Gene ontology term:
(In the same order as above)
2.31, 2.02, 1.94, 1.93, 1.84, 1.77, 1.75, 1.57, 1.49, 1.45, 1.37, 1.34, 1.34, 1.34, 1.28, 1.25, 1.23, 1.19, 1.17, 1.01, 0.92, 0.89, 0.88, 0.86, 0.86, 0.81, 0.75, -0.07

Corresponding total number of genes of each Gene ontology term:
(In the same order as above)
103, 139, 300, 408, 96, 218, 144, 553, 404, 155, 153, 121, 121, 121, 155, 223, 175, 426, 113, 177, 151,

335, 459, 557, 334, 717, 161, 95

Corresponding number of genes of each Gene ontology term with at least one TFBS (of selected TFs):
(In the same order as above)
32, 41, 72, 86, 27, 55, 38, 114, 80, 39, 36, 35, 35, 35, 32, 53, 38, 90, 26, 37, 31, 69, 88, 101, 68, 139, 35, 23

Corresponding total number of TFBS (of selected TFs) for each Gene ontology term (in all genes):
(In the same order as above)
59, 80, 144, 163, 50, 104, 72, 209, 152, 75, 70, 66, 66, 66, 60, 96, 71, 168, 49, 71, 63, 125, 155, 183, 124, 255, 61, 36

Corresponding number of selected TFs each Gene ontology term is involved with:
(In the same order as above)
4, 4, 4, 4, 4, 4, 4, 4, 4, 4, 4, 4, 4, 4, 4, 4, 4, 4, 4, 4, 4, 4, 4, 4, 4, 4, 4, 4

TFs ranked according to mean of effect sizes in cluster:
V$TEL2_Q6, V$ETS_Q4, V$ELF1_Q6, V$NKX25_Q5

Corresponding mean of effect sizes of each TF:
(In the same order as above)
1.55, 1.44, 1.30, 0.92

Corresponding total number of TFBS for each TF (genome-wide):
(In the same order as above)
707, 718, 717, 695

Corresponding total number of TFBS for each TF (in all genes in selected Gene ontology terms):
(In the same order as above)
745, 751, 758, 639

Corresponding number of genes (of selected Gene ontology terms) each TF is involved with:
(In the same order as above)
132, 143, 138, 118

Corresponding number of selected Gene ontology terms each TF is involved with:
(In the same order as above)
28, 28, 28, 28

Cluster for columns 416 to 422, rows 139 to 144

Gene ontology terms

mast cell activation | mast cell degranulation | leukotriene biosynthetic process | icosanoid metabolic process | arachidonic acid metabolic process | cyclooxygenase pathway | lipoxygenase pathway

TFs

V$SMAD4_Q6 | F$STRE_01 | V$SREBP1_02 | V$MYOGNF1_01 | V$PEA3_Q6 | V$ETS_Q6

Information

All related TFs:
(List of all TFs that are related to any of the PWMs)
ELF-1, ELFR, Elf-1, Elk-1, Elk-1-isoform1, Erg-1, Erg-2, Ets-1, Fli-1, GABP-alpha, GABP-alpha:GABP-beta, GABP-beta1, GABP-beta2, NERF-1a, NERF-1b, NERF-2, NF-1, NF-1/L, NF-1/Red1, NF-1A1, NF-1B1, NF-1B2, NF-1C2, NF-1X, Net, PEA3, PU.1, PU.1-xbb1, SAP-1a, SAP-1b, SREBP-1, SREBP-1a, SREBP-1b, SREBP-1c, Smad4, Spi-B, TCF, TEL1, Tel-2a, Tel-2b, Tel-2c, Tel-2d, Tel-2e, Tel-2f, c-Ets-1, c-Ets-1A, c-Ets-1B, c-Ets-2, c-Ets-2A, c-Ets-2B, p38erg, p49erg, p55, p55erg

Ranked gene list:
(All genes of the selected Gene ontology terms with hits of any of the selected TFs, ranked according to the total number of TFBS)
TNF, LTB, LTC4S, LTA, PTGDS, PLA2G6, VAV1, PGF, ALOX5, SH3BP2, PTPN6, BGN, AKT1, LMNA, ACVRL1, PTGS1, MAPK14, LCP2, ITGB2, GUSB, F2, EGR1, CYSLTR1, PSMP, PLCG1, MARK2, IRF6, INPP5D, IL3, IL13, DOK2, AHSA1, UCN, TNFSF13, SRC, PGD, MTHFR, LTB4R, KIT, IL4, FYN, ERAF, BDKRB2, PIK3CD, PAF1, MMP2, LTB4R2, LAT2, HRH2, GPX4, FGFR3, EPHB2, CERK, B3GAT1, AVP, ADORA3

Corresponding total number of TFBS:
(For each gene listed above, the total number of TFBS for any of the selected TFs, multiplied by the number of selected Gene ontology terms containing that gene)
30, 30, 18, 15, 14, 12, 8, 8, 7, 6, 6, 6, 6, 5, 5, 4, 4, 4, 4, 4, 4, 4, 4, 3, 3, 3, 3, 3, 3, 3, 3, 3, 2, 2, 2, 2, 2, 2, 2, 2, 2, 2, 2, 1, 1, 1, 1, 1, 1, 1, 1, 1, 1, 1, 1, 1

Corresponding number of selected Gene ontology terms each gene is involved with:
(In the same order as above)
5, 5, 6, 3, 7, 6, 2, 4, 7, 1, 1, 2, 1, 5, 5, 4, 4, 2, 1, 4, 4, 1, 4, 1, 1, 1, 3, 1, 3, 3, 1, 3, 2, 1, 2, 2, 2, 2, 2, 2, 2, 2, 2, 1, 1, 1, 1, 1, 1, 1, 1, 1, 1, 1, 1, 1

Corresponding number of selected TFs each gene is involved with:
(In the same order as above)
6, 6, 3, 5, 2, 2, 4, 2, 1, 6, 6, 3, 6, 1, 1, 1, 1, 2, 4, 1, 1, 4, 1, 3, 3, 3, 1, 3, 1, 1, 3, 1, 1, 2, 1, 1, 1, 1, 1, 1, 1, 1, 1, 1, 1, 1, 1, 1, 1, 1, 1, 1, 1, 1, 1, 1

Gene ontology terms ranked according to mean of effect sizes in cluster:
cyclooxygenase pathway | arachidonic acid metabolic process | mast cell degranulation | lipoxygenase pathway | icosanoid metabolic process | mast cell activation | leukotriene biosynthetic process

Corresponding mean of effect sizes of each Gene ontology term:
(In the same order as above)
2.45, 1.95, 1.91, 1.90, 1.85, 1.85, 1.74

Corresponding total number of genes of each Gene ontology term:
(In the same order as above)
42, 88, 90, 43, 24, 91, 50

Corresponding number of genes of each Gene ontology term with at least one TFBS (of selected TFs):
(In the same order as above)
18, 23, 26, 15, 9, 27, 17

Corresponding total number of TFBS (of selected TFs) for each Gene ontology term (in all genes):
(In the same order as above)
35, 46, 44, 29, 19, 62, 35

Corresponding number of selected TFs each Gene ontology term is involved with:
(In the same order as above)
6, 6, 6, 6, 6, 6, 6

TFs ranked according to mean of effect sizes in cluster:
V$SREBP1_02, F$STRE_01, V$MYOGNF1_01, V$ETS_Q6, V$SMAD4_Q6, V$PEA3_Q6

Corresponding mean of effect sizes of each TF:
(In the same order as above)
2.22, 2.09, 2.06, 1.97, 1.86, 1.50

Corresponding total number of TFBS for each TF (genome-wide):
(In the same order as above)
693, 651, 654, 721, 666, 717

Corresponding total number of TFBS for each TF (in all genes in selected Gene ontology terms):
(In the same order as above)
51, 43, 47, 46, 42, 41

Corresponding number of genes (of selected Gene ontology terms) each TF is involved with:
(In the same order as above)
21, 17, 21, 19, 17, 18

Corresponding number of selected Gene ontology terms each TF is involved with:
(In the same order as above)
7, 7, 7, 7, 7, 7

Cluster for columns 1296 to 1300, rows 50 to 65

Gene ontology terms

gene expression | DBD domain binding | DNA binding | chordate embryonic development | embryonic development

TFs

V$TBP_Q6 | V$NKX62_Q2 | V$OTX_Q1 | V$PBX1_01 | V$SRY_01 | V$HMGIY_Q3 | V$OCT1_06 | V$TBP_01 | V$TEF_Q6 | V$FOXJ2_02 | V$AFP1_Q6 | V$OCT1_03 | V$SRY_02 | V$FOXO1_01 | V$FOXO3A_Q1 | V$FOXO4_01

Information

All related TFs:
(List of all TFs that are related to any of the PWMs)
AFP1, FOXJ2, FOXO1, FOXO4, HMG, HMG-Y, HMGI-C, Nkx6-2, Oct-1, Otx1, Otx2, POU2F1, POU2F1a, Pbx1a, SRY, TBP, TEF, TEF-xbb1, TFIID

Ranked gene list:
(All genes of the selected Gene ontology terms with hits of any of the selected TFs, ranked according to the total number of TFBS)
RUNX1, LEF1, NR4A2, MAF, ID2, PRDM1, ETS1, TIPARP, NR3C1, MYB, CTNNB1, TGFBR2, SMARCA2, PIK3CG, PIK3CA, TBR1, POU4F1, FLI1, AHR, SOX2, RUNX2, NR3C2, NFIA, JARID2, HSPA8, HIST1H4I, ETV1, CXCR4, CITED2, BMI1, BCL2, ZEB2, SMAD4, NR2F1, NF1, PAX6,

GATA3, TGIF1, POU2F1, MIER1, HOXA7, WNK1, VIM, MXI1, FST, ZEB1, SMAD7, NEUROG2, MEF2C, HOXB4, HOXA9, GSK3B, STAT3, SMAD2, ZFP36L2, STAT5B, HNRNPK, FOXG1, TCF7, SATB1, PURA, PIK3R1, PHIP, MBNL2, MBNL1, LMO4, KLF6, IKZF1, HOXA10, FOXP2, ELF1, CDKN2C, CDKN1B, THRA, TBP, RFX3, OTX2, NFYC, NFYA, MEIS2, LCP1, HIST4H4, EPHA4, DDX5, BUB3, ADAMTS6, TLE3, PITX2, PDLIM5, HOXA1, HNRNPA1, CBX5, BACH2, ARID1B, ARID1A, TLE4, RUNX1T1, NFIB, HOXA5, ETV3, EOMES, DR1, CD44, ACVR2A, SKP1, RERE, NFKB1, MAP3K1, ETV6, ARNTL, TCF4, STAT1, PCNA, NFE2L2, NFAT5, MEF2D, ITGB1, IRF2, HOXC8, HOXA11, HBP1, HAND2, GABPB1, CDK4, CCND2, CCNB1IP1, REV3L, MLL, MAP3K14, FOXJ2, ERGIC2, DYRK1A, DAD1, PTCH1, NFATC3, IGF1R, FOXO1, DLX2, TCF7L2, TBPL1, TBK1, SUB1, SMAD3, RORA, REL, NCOA3, HSPA1B, GTF2A1, CUL1, CREB1, CHD1, ATM, NR4A3, NEDD9, MYLIP, MED13, LPHN2, HIVEP1, ESR1, EFNB2, DLX1, DLL1, CDC25C, BACH1, UBE2B, UBC, TRIB2, TBL1XR1, SOS1, SHOX2, RREB1, RBL2, RARG, PTGER4, PDCD4, NRF1, MAX, MAP4K4, LHX9, KLF12, JAK1, IRX5, IL7R, HOXD8, HNRPDL, HNRNPC, HIVEP2, HIST1H1C, HEY2, FYN, FOXN3, CYTIP, CDC2, BTG1, BCL6, BCL11B, ATXN1, ARID5B, ADD3, ACVR1, TNFAIP3, SUFU, SP1, SOX4, RBM12, PROX1, PDE4B, NR2F2, KAT2B, HOXB7, GPR183, EED, CUGBP2, CTLA4, CPNE1, CDK6, CBX3, BRD2, BCLAF1, ZNF10, MAT2B, MAPRE2, KLF7, HOXA4, HECA, GRAP2, FOXP1, ERBB2IP, ENC1, EBF1, CLCN3, CD69, CASP2, ATF7IP, ARHGDIB, ANXA1, ABCD2, TNFAIP8, SPRY1, S1PR1, RGMB, PTPRC, MGAT5, LRMP, IRS1, IKZF3, HIST1H1E, ZNF148, UGP2, SOD1, RBPJ, RBM15, PXMP3, PRH1, PRG4, PDE3B, OTUD4, NFATC1, NAB1, MLC-B, MEIS1, ITPR1, ITK, HOXB3, HOXA2, HIST1H1A, H2AFZ, GADD45A, GABPA, FGF10, EVI1, ETV5, EGR2, E2F3, DENND4A, DAAM1, CD47, CADM1, BIRC3, BHLHB2, BDNF, ATF2, ANTXR2, ZFP36L1, TRERF1, SNRK, RTN4, ORC4L, HNRNPD, H3F3B, GIT2, EVL, DMTF1, DCTD, CDC42EP3, CAMK2D, YES1, UBL3, TXNIP, TTF2, TOB1, SSBP3, SNAPC5, SLC38A2, SART3, RGS1, PRKACB, POU3F3, NPAT, NCOA4, MYNN, LMO3, JUN, IL6ST, HSF2, HOXD10, HNRNPU, HNRNPH1, HMX2, HIVEP3, H2AFJ, GTF3A, GLIPR1, GFI1, FRS2, FAS, EP300, DUSP6, DUSP1, CHIC2, CHD3, CD53, BCL2L1, ANP32A, ZNF281, WWP1, SMAD5, SGK1, NKX2-2, MAP2K6, IVNS1ABP, IGF2BP3, HIPK3, GGPS1, FOXO3, EYA1, ENOPH1, DUSP5, DPYD, DOCK10, CCNL1, CAMK4, CALM2, CALM1, APC, ADNP, ADIPOR1, ZNF569, ZHX2, YBX1, UNCX, UBE4A, TNF, TGFBR3, SUPT3H, SOX5, SIX1, RPL34, RGS2, PTP4A1, PLAG1, PBX1, OSR1, NEGR1, MDM4, LIG4, INSIG2, IGF1, ID3, HOXC9, HOXC6, HOXC5, HOXC4, HOXA3, HIST2H2AC, GTF2I, GREM2, GBP2, FOSL2, FGF9, FAF1, DLX6, CREM, CASP1, CAP1, BCL2L11, ATP1A1, ARID4A, AKT3, TOX, TANK, SLCO3A1, QKI, PTGER2, PSMB8, PMS1, PKIA, MGAT1, LRRFIP2, HOXA6, FUBP1, FIGN, ESRRG, EIF4A2, DYRK2, CFLAR, CD200R1, BCL11A, B2M, ZNF521, ZIC1, ZFYVE9, XRCC5, XPO1, TWIST1, TNFSF10, TLR1, TFB1M, SRI, SP8, SP4, SMARCA5, SHC1, SEMA3A, RUNX3, RPL37, ROCK1, REST, RBM47, PTTG1, POU3F2, PIK3C3, PAX5, NNAT, NFKBIA, NFIL3, NFATC2, MORF4L1, MGA, MED21, MANEA, LIN54, LHX1, LDB1, KLF9, KLF5, KIAA1524, ITGA6, IRF1, HSPH1, HOXB8, HOXB5, HOXB2, HMX3, HMGCS1, HMGB1, HIST2H2BE, HIST1H4C, HIF1AN, HCLS1, GLUL, FZD8, FUS, FOS, FBXO32, EPHA7, EPHA3, DUSP10, CUX1, CISH, CD55, CD3D, CCNG1, CASP8, BRIP1, BMP5, BIRC2, APEX1, ANAPC10, AFF1, ZFPM2, ZFP161, TP63, TNPO1, TFAP2A, TAX1BP1, SMAD1, SLC39A1, SKP2, SFMBT2, RARA, RAB3GAP1, PUM2, PUM1, PRDM2, POU1F1, MYC, LBX1, ING3, IL23A, HSP90B1, HSF1, H3F3A, GBP5, GBF1, CRTC2, CREBBP, CPS1, CDKN1A, CAST, ASXL1, ANG, ACTA2, ZBTB7B, XBP1, TTK, TSC22D1, TFB2M, TAF8, SOD2, SLC16A10, SATB2, SAMD4A, RYBP, RPE, RDH10, PSMD1, PSMC2, PPP3R1, POLR2A, PITX3, PIAS1, PFKFB2, PER1, PEG10, PCDH17, PBXIP1, PAX9, NR2C2, NR1D1, NP, NEDD1, NCOA2, MYF6, MYF5, MYCN, MTPN, MSL2, LRRFIP1, LRBA, KRIT1, KRAS, IL2, IFRD1, IFIH1, ID1, HSP90AA1, HOXD3, HOXD13, HOXB6, HMGN3, HIST2H2AA3, HIF1A, HEG1, HDAC9, FOXN2, FOXF2, FOXD3, FILIP1L, EPHB6, EPAS1, ENPP2, ELF2, EFNA5, EFNA1, DNMT3A, DICER1, DEK, DEDD, COPS2, COL19A1, CLK4, CLDND1, CENPC1, CEBPG, CDK2, CDC5L, CDC42SE1, CCNG2, BTF3, BTAF1, BRAF, BNIP2, BBC3, BATF, BAT1, ARHGEF2, ANXA2, ZNF143, ZFP36, ZFHX3, ZC3H12D, WNT1, VCAN, USO1, USF1, UCP2, TPT1, TMBIM6, TGFBR1, TGFB2, TCHP, TCF12, ST6GAL1, SOX6, SIN3A, SESN1, SDHC, S100A6, RXRB, RNASEN, PRKAB1, PPP3CA, PPARGC1A, PEA15, PDGFRA, NUP37, NUDT6, NR2E1, NPM1, NFE2L1, NAP1L4, MRPS6, MRE11A, MPZ, MGEA5, MBD2, MBD1, MARCKS, MAPKAPK3, MAGOHB, JUNB, ITGB3BP, IRS2, INHBA, IL2RA, HMMR, HDAC4, HAPLN1, GTF2B, GSC, GRIK2, FOSB, FGFR1, FGF8, FBXO8, FAM129A, ETF1, EIF4E, EGR1, EEF1A1, DDX42, CWC22, CTDSP2, CTCF, CSK, COL11A2, CKS1B, CDR2, CDC42,

CD2, CCDC91, CAND1, CAMTA1, CALCRL, BRD4, BMP4, BIN2, BHLHB3, APAF1, ANKH, ALCAM, ACSL5, ABCF2, ZNF24, ZNF146, ZMAT3, ZBTB7A, ZBTB17, YWHAZ, XRN1, XRCC4, WHSC1, VPS72, UNC5C, UBP1, UBE3A, TRAF1, TPBG, TNIP1, TIAM1, TG, TFRC, TFEB, TFAP2B, TCF21, TAGLN2, TAF6, SYTL2, SUDS3, STK38, SSB, SPTBN1, SPRY2, SNRPN, SMARCB1, SLC25A4, SIM1, SGPL1, SFRP2, SEPT7, SEPHS1, SELPLG, SAP30BP, SAP18, RPS6KA5, RPL7, RHOA, RBBP4, RAG1, RAF1, PTMA, PTK2B, PRKCA, PRKAR1A, PRKAA2, PPRC1, POU4F2, POT1, PHF20, PHF12, PERP, PCDHGB4, PBX3, PARP14, ORC6L, OPTN, NXT1, NUP43, NFIX, NET1, NELL2, NDRG1, MYEF2, MLLT11, MLL2, MEOX2, MED23, MCM2, MCL1, MBD4, MAZ, MAT2A, MAPK10, MAP4K3, MAP3K5, MAP3K12, MAP2K3, MAFF, LYST, LTA, LSM2, LRPPRC, LPIN1, LHX2, LDHB, LCP2, LCK, KPNA1, JMJD3, ITPR2, ITGA1, INVS, INSIG1, ING2, IMP3, ILK, IL6R, IL16, IFI44, HSPE1, HSPA2, HSPA1A, HOXC10, HIST2H3C, HIST1H1T, HELLS, HDAC7, GZMA, GTF2F2, GRB2, GINS4, GFM1, FZD1, FEN1, FBXW4, FAM126A, EZH1, EXTL2, ERRFI1, ERCC5, ERBB4, ENSA, ELOVL5, EIF3A, EDN1, EDAR, DVL2, DRGX, DPH1, DLL4, DDIT4, DDIT3, DCN, DACH1, CSDA, CPT1A, CLDN12, CHUK, CFL1, CDH6, CCR7, CCNH, CASP9, CASP3, CALU, C1orf107, C13orf15, BRCA2, BMF, BCOR, BCL9L, BANP, BAG3, ATR, ATP6V0A1, ATP2A2, ATP1B1, ATF6, ASPM, APOA2, ALDOA, AGER, ADAR, ADAMTS4, ACHE, ABHD2, ABCG1, ZNF395, ZNF217, ZNF202, YWHAQ, YWHAG, XPR1, XAB2, WDR48, WDR1, VTA1, VGLL3, UQCRH, UNG, UBR2, UBE2C, TUSC2, TSN, TSHR, TRIM3, TRDMT1, TRAF3, TP53INP1, TNRC6A, TMSB4X, TIA1, TBX4, TAF9, TAC1, STK11, STC1, SRA1, SQSTM1, SPATA2, SNF1LK2, SLIT2, SLCO1A2, SLC40A1, SLC35A2, SLC30A1, SLC25A3, SLC16A7, SIX6, SILV, SIL1, SIDT1, SIAH1, SHMT2, SFRS2IP, SERPINI2, SEMA6D, SEMA6A, SCG2, SC5DL, SC4MOL, S100A4, S100A13, S100A10, RPS3A, RPL4, RPL21, RPL13A, RORC, ROD1, RNF169, RHOT1, RGS10, REPIN1, REEP5, RBM27, RAGE, RAD17, RAB13, PTPRO, PTPN12, PSMC1, PRDX4, PPP1R1B, PPID, PPAP2A, PNKD, PMAIP1, PLOD1, PLN, PLAGL2, PKNOX2, PIM2, PFKFB3, PDK4, PCSK7, PCP2, PAM, PAK3, PABPC1, OXR1, NSL1, NRP2, NRAS, NR1H3, NLK, NEO1, NDRG2, NAP1L1, MTTP, MTERF, MRS2, MRFAP1, MOG, MGST2, MED8, MBTPS1, MAPRE1, MAPKAPK2, MAPK6, MAP3K8, MAP3K3, MAG, LTB, LMNA, LHX8, LEPROT, LEPR, LDHA, KLRA1, ITGA2B, IMMT, IL1RAP, IL18R1, IL18, IKBKE, ICAM2, IAPP, HSPD1, HSP90AB1, HNRNPF, HMOX2, HK1, HIST2H3A, HERPUD1, HBEGF, HAT1, H2AFY, GSPT1, GRM1, GPX1, GPS2, GPR19, GNRH2, GNA13, GMCL1, GCG, GBA, GAPDH, G3BP1, FRAT2, FRAT1, FOXD1, FKBP5, FHIT, FAR1, FABP5, EXT1, EXOSC7, ESCO2, EPHA6, EIF6, EIF5A, EIF2S2, EIF1, EGLN2, EFNA4, EEF1E1, ECE1, E2F7, DYNLL1, DST, DCTN3, CXCR5, CWC15, CTSS, CTSL1, CSNK1A1, CRY2, CRY1, CREB5, COX4I1, COL8A1, CLK1, CHPT1, CHMP2B, CHD7, CHD6, CENPF, CDON, CDCA7L, CDC123, CD83, CD68, CD59, CD4, CCT5, CCR6, CCR5, CCR4, CCNC, CBLN3, CASP10, CARD16, CAPNS1, CAMK2G, CA5A, C5orf13, C10orf137, BRMS1L, BOC, BNIP3L, BMP2K, BIK, BAMBI, ATIC, ASH1L, APBB3, AGPAT1, ADIPOR2, ADFP, ACSL6, ACOX1, ACAT2, ABI2, ABI1, ABCF3, ABCC5

Corresponding total number of TFBS:
(For each gene listed above, the total number of TFBS for any of the selected TFs, multiplied by the number of selected Gene ontology terms containing that gene)
80, 80, 75, 70, 70, 64, 64, 60, 60, 60, 60, 56, 56, 56, 56, 55, 55, 55, 55, 50, 50, 48, 48, 48, 48, 48, 48, 48, 48, 48, 48, 45, 45, 45, 45, 44, 44, 42, 40, 40, 40, 39, 39, 39, 39, 36, 36, 36, 36, 36, 36, 36, 35, 35, 33, 33, 33, 33, 32, 32, 32, 32, 32, 32, 32, 32, 32, 32, 32, 32, 32, 32, 32, 30, 30, 30, 30, 30, 30, 30, 30, 30, 30, 30, 30, 28, 28, 28, 28, 28, 28, 28, 28, 28, 27, 27, 27, 27, 27, 27, 27, 27, 27, 26, 26, 26, 26, 26, 26, 24, 24, 24, 24, 24, 24, 24, 24, 24, 24, 24, 24, 24, 24, 24, 24, 22, 22, 22, 22, 22, 22, 22, 21, 21, 21, 21, 21, 20, 20, 20, 20, 20, 20, 20, 20, 20, 20, 20, 20, 20, 20, 18, 18, 18, 18, 18, 18, 18, 18, 18, 18, 18, 18, 16, 16, 16, 16, 16, 16, 16, 16, 16, 16, 16, 16, 16, 16, 16, 16, 16, 16, 16, 16, 16, 16, 16, 16, 16, 16, 16, 16, 16, 16, 16, 16, 16, 16, 15, 15, 15, 15, 15, 15, 15, 15, 15, 15, 15, 15, 15, 15, 15, 15, 15, 15, 15, 15, 14, 14, 14, 14, 14, 14, 14, 14, 14, 14, 14, 14, 14, 14, 14, 14, 14, 14, 14, 13, 13, 13, 13, 13, 13, 13, 13, 13, 13, 12, 12, 12, 12, 12, 12, 12, 12, 12, 12, 12, 12, 12, 12, 12, 12, 12, 12, 12, 12, 12, 12, 12, 12, 12, 12, 12, 12, 12, 12, 12, 12, 12, 12, 11, 11, 11, 11, 11, 11, 11, 11, 11, 11, 11, 11, 11, 10, 10, 10, 10, 10, 10, 10, 10, 10, 10, 10, 10, 10, 10, 10, 10, 10, 10, 10, 10, 10, 10, 10, 10, 10, 10, 10, 10, 10, 10, 10, 10, 10, 10, 10, 10, 10, 9, 9, 9, 9, 9, 9, 9, 9, 9, 9, 9, 9, 9, 9, 9, 9, 9, 9, 9, 9, 9, 9, 9, 9, 8, 8, 8, 8, 8, 8, 8, 8, 8, 8, 8, 8, 8, 8, 8, 8, 8, 8, 8, 8, 8, 8, 8, 8, 8, 8, 8, 8, 8, 8, 8, 8, 8, 8, 8, 8, 8, 8, 8, 8, 8, 8, 8, 8, 8, 8, 7, 7, 7, 7, 7, 7, 7, 7, 7, 7, 7, 7, 7, 7, 7, 7, 7, 7, 7, 7, 7, 7, 6, 6, 6, 6, 6, 6, 6, 6, 6, 6, 6, 6, 6, 6, 6, 6, 6, 6, 6, 6, 6, 6, 6, 6, 6, 6, 6, 6, 6, 6, 6, 6, 6, 6, 6, 6, 6, 6, 6,

6, 6, 6, 6, 6, 6, 6, 6, 6, 6, 6, 6, 6, 6, 6, 6, 6, 6, 6, 6, 6, 6, 6, 6, 6, 6, 6, 5, 5, 5, 5, 5, 5, 5, 5, 5, 5, 5, 5, 5, 5, 5,
5, 5, 5, 5, 5, 5, 5, 5, 5, 5, 5, 5, 5, 5, 5, 5, 5, 5, 4, 4, 4, 4, 4, 4, 4, 4, 4, 4, 4, 4, 4, 4, 4, 4, 4, 4, 4, 4, 4, 4, 4, 4,
4, 4, 4, 4, 4, 4, 4, 4, 4, 4, 4, 4, 4, 4, 4, 4, 4, 4, 4, 4, 4, 4, 4, 4, 4, 4, 4, 4, 4, 4, 4, 4, 4, 4, 4, 4, 4, 4, 4, 4, 4, 4,
4, 4, 4, 4, 4, 4, 4, 4, 4, 4, 4, 4, 4, 4, 4, 4, 4, 4, 4, 3, 3, 3, 3, 3, 3, 3, 3, 3, 3, 3, 3, 3, 3, 3, 3, 3, 3, 3, 3, 3, 3, 3,
3, 3, 3, 3, 3, 3, 3, 3, 3, 3, 3, 3, 3, 3, 3, 3, 3, 3, 3, 3, 3, 3, 3, 3, 3, 3, 3, 3, 3, 3, 3, 3, 3, 3, 3, 3, 3, 3, 3, 3, 3, 3,
3, 3, 3, 3, 3, 3, 3, 3, 3, 3, 3, 3, 3, 3, 3, 3, 3, 3, 3, 3, 3, 2, 2, 2, 2, 2, 2, 2, 2, 2, 2, 2, 2, 2, 2, 2, 2, 2, 2, 2, 2, 2,
2, 2, 2, 2, 2, 2, 2, 2, 2, 2, 2, 2, 2, 2, 2, 2, 2, 2, 2, 2, 2, 2, 2, 2, 2, 2, 2, 2, 2, 2, 2, 2, 2, 2, 2, 2, 2, 2, 2, 2, 2, 2,
2, 2, 2, 2, 2, 2, 2, 2, 2, 2, 2, 2, 2, 2, 2, 2, 2, 2, 2, 2, 2, 2, 2, 2, 2, 2, 2, 2, 2, 2, 2, 2, 2, 2, 2, 2, 2, 2, 2, 2, 2, 2,
2, 2, 2, 2, 2, 2, 2, 2, 2, 2, 2, 2, 2, 2, 2, 2, 2, 2, 2, 2, 2, 2, 2, 2, 2, 2, 2, 2, 2, 2, 2, 2, 2, 2, 2, 2, 2, 2, 2, 2, 2, 2,
2, 2, 2, 2, 2, 2, 2, 2, 2, 2, 2, 2, 2, 2, 2, 2, 2, 2, 2, 2, 2, 2, 2, 2, 2, 2, 2, 1, 1, 1, 1, 1, 1, 1, 1, 1, 1, 1, 1, 1, 1, 1,
1, 1, 1, 1, 1, 1, 1, 1, 1, 1, 1, 1, 1, 1, 1, 1, 1, 1, 1, 1, 1, 1, 1, 1, 1, 1, 1, 1, 1, 1, 1, 1, 1, 1, 1, 1, 1, 1, 1, 1, 1, 1,
1, 1, 1, 1, 1, 1, 1, 1, 1, 1, 1, 1, 1, 1, 1, 1, 1, 1, 1, 1, 1, 1, 1, 1, 1, 1, 1, 1, 1, 1, 1, 1, 1, 1, 1, 1, 1, 1, 1, 1, 1, 1,
1, 1, 1, 1, 1, 1, 1, 1, 1, 1, 1, 1, 1, 1, 1, 1, 1, 1, 1, 1, 1, 1, 1, 1, 1, 1, 1, 1, 1, 1, 1, 1, 1, 1, 1, 1, 1, 1, 1, 1, 1, 1,
1, 1, 1, 1, 1, 1, 1, 1, 1, 1, 1, 1, 1, 1, 1, 1, 1, 1, 1, 1, 1, 1, 1, 1, 1, 1, 1, 1, 1, 1, 1, 1, 1, 1, 1, 1, 1, 1, 1, 1, 1, 1,
1, 1, 1, 1, 1, 1, 1, 1, 1, 1, 1, 1, 1, 1, 1, 1, 1, 1, 1, 1, 1, 1, 1, 1, 1, 1, 1, 1, 1, 1, 1, 1, 1, 1, 1, 1, 1, 1, 1, 1, 1, 1,
1, 1, 1, 1, 1, 1

Corresponding number of selected Gene ontology terms each gene is involved with:
(In the same order as above)
5, 5, 5, 5, 5, 4, 4, 4, 4, 5, 5, 4, 4, 4, 4, 5, 5, 5, 5, 5, 5, 3, 3, 4, 3, 3, 4, 3, 3, 3, 4, 3, 5, 5, 3, 4, 4, 3, 4, 5, 4, 3,
3, 3, 3, 3, 3, 3, 4, 4, 4, 3, 5, 5, 3, 3, 3, 3, 4, 2, 2, 2, 2, 2, 2, 2, 2, 4, 2, 2, 2, 2, 3, 5, 2, 3, 3, 3, 3, 2, 3, 3, 2,
2, 2, 2, 4, 4, 4, 2, 2, 2, 2, 2, 3, 3, 3, 3, 3, 3, 3, 3, 3, 2, 2, 2, 2, 2, 2, 4, 3, 4, 3, 3, 3, 3, 3, 3, 3, 2, 4, 2, 3, 3, 3,
2, 2, 2, 2, 2, 2, 2, 3, 3, 3, 3, 3, 5, 5, 2, 5, 5, 2, 5, 2, 2, 4, 2, 4, 2, 2, 2, 2, 2, 2, 2, 2, 3, 3, 3, 3, 3, 2, 1, 4, 1, 1,
4, 1, 1, 4, 4, 1, 1, 4, 2, 2, 2, 1, 2, 2, 1, 2, 4, 4, 1, 2, 2, 1, 1, 1, 4, 1, 1, 1, 1, 1, 2, 1, 3, 5, 5, 1, 3, 1, 5, 3, 3,
1, 3, 1, 1, 1, 1, 1, 3, 1, 2, 1, 1, 1, 2, 1, 1, 1, 1, 1, 2, 1, 1, 2, 1, 1, 1, 1, 1, 1, 1, 1, 1, 1, 1, 1, 1, 3, 1, 3, 4, 1,
1, 2, 1, 1, 1, 3, 1, 1, 4, 1, 3, 3, 3, 1, 2, 3, 3, 3, 4, 2, 4, 2, 1, 1, 1, 1, 2, 4, 3, 3, 1, 1, 1, 1, 1, 1, 1, 1, 1, 1, 1,
1, 1, 1, 1, 1, 5, 1, 2, 1, 1, 1, 1, 1, 1, 1, 1, 1, 5, 2, 5, 2, 1, 1, 1, 2, 1, 5, 1, 2, 2, 2, 5, 2, 1, 2, 2, 1, 2, 1, 1, 1,
3, 1, 3, 1, 1, 1, 1, 1, 3, 1, 3, 1, 1, 1, 1, 1, 1, 1, 3, 1, 3, 4, 1, 4, 2, 2, 2, 1, 1, 1, 2, 1, 1, 1, 1, 4, 2, 1, 2, 1, 1, 1,
4, 1, 2, 2, 2, 1, 4, 4, 2, 1, 2, 2, 2, 2, 2, 2, 1, 2, 1, 1, 1, 1, 1, 1, 1, 1, 1, 1, 1, 1, 1, 1, 1, 1, 1, 1, 1, 1, 1, 1, 1, 1,
3, 2, 2, 2, 3, 1, 1, 2, 1, 1, 3, 3, 2, 2, 3, 1, 1, 3, 3, 1, 3, 2, 2, 1, 2, 2, 3, 3, 1, 2, 1, 1, 2, 2, 2, 2, 2, 2, 3, 1, 2, 2,
3, 3, 2, 3, 3, 3, 1, 1, 2, 2, 3, 3, 1, 2, 3, 1, 2, 2, 1, 1, 2, 2, 2, 3, 2, 2, 1, 1, 5, 1, 5, 1, 5, 1, 5, 1, 1, 1, 5, 5, 1, 1,
1, 5, 5, 5, 1, 5, 1, 5, 1, 1, 1, 1, 5, 1, 5, 1, 1, 1, 1, 2, 2, 4, 1, 1, 4, 2, 1, 1, 2, 2, 1, 2, 2, 1, 1, 1, 1, 2, 1, 1, 1, 2,
1, 4, 4, 1, 1, 2, 2, 4, 4, 4, 1, 1, 1, 1, 2, 1, 2, 4, 1, 4, 2, 2, 4, 4, 1, 4, 4, 2, 2, 2, 2, 2, 1, 2, 2, 2, 1, 4, 2, 4, 1, 2,
2, 1, 1, 1, 1, 1, 2, 4, 2, 2, 1, 1, 2, 1, 1, 1, 2, 1, 1, 1, 3, 1, 1, 1, 3, 3, 1, 3, 1, 1, 1, 3, 3, 3, 3, 1, 1, 3, 1, 1, 1, 3,
1, 1, 1, 1, 3, 3, 1, 3, 3, 3, 3, 1, 1, 1, 1, 3, 3, 3, 1, 1, 1, 3, 1, 1, 1, 1, 1, 1, 1, 3, 3, 1, 3, 3, 3, 1, 1, 3, 3, 3, 1, 1,
1, 1, 3, 3, 3, 1, 1, 3, 1, 1, 1, 1, 1, 1, 3, 1, 3, 1, 1, 1, 1, 1, 2, 1, 1, 2, 2, 1, 2, 1, 2, 1, 2, 2, 1, 2, 2, 1, 1, 1, 2, 2,
2, 2, 1, 1, 1, 1, 1, 2, 1, 2, 1, 2, 2, 2, 2, 2, 1, 1, 1, 2, 2, 2, 1, 2, 2, 2, 2, 1, 1, 2, 2, 2, 2, 2, 2, 2, 1, 1, 1, 2, 1, 1,
1, 2, 2, 2, 1, 2, 1, 2, 1, 1, 2, 1, 2, 2, 2, 2, 1, 1, 1, 2, 1, 2, 2, 1, 1, 2, 2, 1, 2, 1, 1, 2, 2, 1, 1, 1, 2, 1, 2, 1, 1, 1,
1, 2, 2, 2, 2, 1, 2, 2, 1, 1, 1, 2, 1, 2, 2, 2, 2, 1, 1, 1, 2, 1, 2, 2, 1, 1, 1, 1, 1, 1, 2, 2, 2, 1, 2, 2, 1, 2, 1, 1, 2, 2,
2, 1, 1, 2, 2, 1, 2, 1, 2, 1, 1, 1, 1, 1, 2, 1, 1, 1, 2, 1, 2, 2, 1, 2, 1, 2, 1, 1, 1, 1, 1, 1, 1, 1, 1, 1, 1, 1, 1, 1, 1, 1,
1, 1, 1, 1, 1, 1, 1, 1, 1, 1, 1, 1, 1, 1, 1, 1, 1, 1, 1, 1, 1, 1, 1, 1, 1, 1, 1, 1, 1, 1, 1, 1, 1, 1, 1, 1, 1, 1, 1, 1, 1, 1,
1, 1, 1, 1, 1, 1, 1, 1, 1, 1, 1, 1, 1, 1, 1, 1, 1, 1, 1, 1, 1, 1, 1, 1, 1, 1, 1, 1, 1, 1, 1, 1, 1, 1, 1, 1, 1, 1, 1, 1, 1, 1,
1, 1, 1, 1, 1, 1, 1, 1, 1, 1, 1, 1, 1, 1, 1, 1, 1, 1, 1, 1, 1, 1, 1, 1, 1, 1, 1, 1, 1, 1, 1, 1, 1, 1, 1, 1, 1, 1, 1, 1, 1, 1,
1, 1, 1, 1, 1, 1, 1, 1, 1, 1, 1, 1, 1, 1, 1, 1, 1, 1, 1, 1, 1, 1, 1, 1, 1, 1, 1, 1, 1, 1, 1, 1, 1, 1, 1, 1, 1, 1, 1, 1, 1, 1,
1, 1, 1, 1, 1, 1, 1, 1, 1, 1, 1, 1, 1, 1, 1, 1, 1, 1, 1, 1, 1, 1, 1, 1, 1, 1, 1, 1, 1, 1, 1, 1, 1, 1, 1, 1, 1, 1, 1, 1, 1, 1,
1, 1, 1, 1, 1, 1, 1

Corresponding number of selected TFs each gene is involved with:
(In the same order as above)
16, 16, 15, 14, 14, 16, 16, 15, 15, 12, 12, 14, 14, 14, 14, 11, 11, 11, 11, 10, 10, 16, 16, 12, 16, 16, 12, 16,
16, 16, 12, 15, 9, 9, 15, 11, 11, 14, 10, 8, 10, 13, 13, 13, 13, 12, 12, 12, 9, 9, 9, 12, 7, 7, 11, 11, 11, 11, 8,
16, 16, 16, 16, 16, 16, 16, 16, 16, 8, 16, 16, 16, 16, 10, 6, 15, 10, 10, 10, 10, 15, 10, 10, 15, 15, 15, 14, 7,
7, 7, 14, 14, 14, 14, 14, 9, 9, 9, 9, 9, 9, 9, 9, 9, 13, 13, 13, 13, 13, 13, 6, 8, 6, 8, 8, 8, 8, 8, 8, 8, 12, 6, 12, 8,
8, 8, 11, 11, 11, 11, 11, 11, 11, 7, 7, 7, 7, 7, 4, 4, 10, 4, 4, 10, 4, 10, 10, 5, 10, 5, 10, 10, 9, 9, 9, 9, 9, 9, 6, 6,
6, 6, 6, 9, 16, 4, 16, 16, 4, 16, 16, 4, 4, 16, 16, 4, 8, 8, 8, 16, 8, 8, 16, 8, 4, 4, 16, 8, 8, 16, 16, 16, 4, 16, 16,
16, 16, 16, 16, 8, 15, 5, 3, 3, 15, 5, 15, 3, 5, 5, 15, 5, 15, 15, 15, 15, 15, 5, 15, 7, 14, 14, 14, 7, 14, 14, 14,

14, 14, 7, 14, 14, 7, 14, 14, 14, 14, 13, 13, 13, 13, 13, 13, 13, 13, 13, 13, 4, 12, 4, 3, 12, 12, 6, 12, 12, 12, 4, 12, 12, 3, 12, 4, 4, 4, 12, 6, 4, 4, 4, 3, 6, 3, 6, 12, 12, 12, 12, 6, 3, 4, 4, 12, 11, 11, 11, 11, 11, 11, 11, 11, 11, 11, 11, 11, 11, 10, 10, 10, 2, 10, 5, 10, 10, 10, 10, 10, 10, 10, 10, 10, 10, 2, 5, 2, 5, 10, 10, 10, 5, 10, 2, 10, 5, 5, 5, 2, 5, 10, 5, 5, 10, 5, 10, 9, 9, 3, 9, 3, 9, 9, 9, 9, 9, 3, 9, 3, 9, 9, 9, 9, 9, 9, 9, 3, 9, 3, 2, 8, 2, 4, 4, 4, 8, 8, 8, 4, 8, 8, 8, 8, 2, 4, 8, 4, 8, 8, 8, 2, 8, 4, 4, 4, 8, 2, 2, 4, 8, 4, 4, 4, 4, 4, 4, 8, 4, 8, 8, 8, 7, 7, 7, 7, 7, 7, 7, 7, 7, 7, 7, 7, 7, 7, 7, 7, 7, 7, 7, 7, 6, 2, 3, 3, 3, 2, 6, 6, 3, 6, 6, 2, 2, 3, 3, 2, 6, 6, 2, 2, 6, 2, 3, 3, 6, 3, 3, 2, 2, 6, 3, 6, 6, 3, 3, 3, 3, 3, 3, 2, 6, 3, 3, 2, 2, 3, 2, 2, 2, 6, 6, 3, 3, 2, 2, 6, 3, 2, 6, 3, 3, 6, 6, 3, 3, 3, 2, 3, 3, 6, 6, 1, 5, 1, 5, 1, 5, 1, 5, 5, 5, 1, 1, 5, 5, 5, 1, 1, 1, 5, 1, 5, 1, 5, 5, 5, 5, 1, 5, 1, 5, 5, 5, 5, 2, 2, 1, 4, 4, 1, 2, 4, 4, 2, 2, 4, 2, 2, 4, 4, 4, 4, 2, 4, 4, 4, 2, 4, 1, 1, 4, 4, 2, 2, 1, 1, 1, 4, 4, 4, 4, 2, 4, 2, 1, 4, 1, 2, 2, 1, 1, 4, 1, 1, 2, 2, 2, 2, 2, 4, 2, 2, 2, 4, 1, 2, 1, 4, 2, 2, 4, 4, 4, 4, 4, 2, 1, 2, 2, 4, 4, 2, 4, 4, 4, 2, 4, 4, 4, 1, 3, 3, 3, 1, 1, 3, 1, 3, 3, 3, 1, 1, 1, 1, 3, 3, 1, 3, 3, 3, 1, 3, 3, 3, 3, 1, 1, 3, 1, 1, 1, 1, 3, 3, 3, 3, 1, 1, 1, 3, 3, 3, 1, 3, 3, 3, 3, 3, 3, 3, 1, 1, 3, 1, 1, 1, 3, 3, 1, 1, 1, 3, 3, 3, 3, 1, 1, 1, 3, 3, 1, 3, 3, 3, 3, 3, 3, 1, 3, 1, 3, 3, 3, 3, 3, 1, 2, 2, 1, 1, 2, 1, 2, 1, 2, 1, 1, 2, 1, 1, 2, 2, 2, 1, 1, 1, 1, 2, 2, 2, 2, 2, 1, 2, 1, 2, 1, 1, 1, 1, 1, 2, 2, 2, 1, 1, 1, 2, 1, 1, 1, 1, 2, 2, 1, 1, 1, 1, 1, 1, 1, 2, 2, 2, 1, 2, 2, 2, 1, 1, 1, 2, 1, 2, 1, 2, 2, 1, 2, 1, 1, 1, 1, 2, 2, 2, 1, 2, 1, 1, 2, 2, 1, 1, 2, 1, 2, 2, 1, 1, 2, 2, 2, 1, 2, 1, 2, 2, 2, 2, 1, 1, 1, 1, 2, 1, 1, 2, 2, 2, 1, 2, 1, 1, 1, 1, 2, 2, 2, 1, 2, 1, 1, 2, 2, 2, 2, 2, 2, 1, 1, 1, 2, 1, 1, 2, 1, 2, 2, 1, 1, 1, 2, 2, 1, 1, 2, 1, 2, 1, 2, 2, 2, 2, 2, 1, 2, 2, 2, 1, 2, 1, 1, 2, 1, 2, 1, 2, 2, 1

Gene ontology terms ranked according to mean of effect sizes in cluster:
DNA binding | gene expression | embryonic development | DBD domain binding | chordate embryonic development

Corresponding mean of effect sizes of each Gene ontology term:
(In the same order as above)
2.74, 2.33, 1.86, 1.84, 1.47

Corresponding total number of genes of each Gene ontology term:
(In the same order as above)
1.2K, 3.8K, 1.2K, 411, 671

Corresponding number of genes of each Gene ontology term with at least one TFBS (of selected TFs):
(In the same order as above)
377, 957, 329, 138, 187

Corresponding total number of TFBS (of selected TFs) for each Gene ontology term (in all genes):
(In the same order as above)
1979, 4594, 1756, 747, 1040

Corresponding number of selected TFs each Gene ontology term is involved with:
(In the same order as above)
16, 16, 16, 16, 16

TFs ranked according to mean of effect sizes in cluster:
V$OCT1_03, V$OTX_Q1, VTBP_Q6, VNKX62_Q2, VSRY_01, VFOXO1_01, V$FOXO3A_Q1, V$TBP_01, V$HMGIY_Q3, V$FOXO4_01, VSRY_02, VPBX1_01, V$AFP1_Q6, V$OCT1_06, VTEF_Q6, VFOXJ2_02

Corresponding mean of effect sizes of each TF:
(In the same order as above)
3.34, 3.19, 3.14, 3.05, 2.77, 2.21, 2.14, 2.05, 1.89, 1.83, 1.80, 1.55, 1.25, 1.16, 1.00, 0.40

Corresponding total number of TFBS for each TF (genome-wide):
(In the same order as above)
704, 701, 723, 698, 743, 680, 689, 685, 716, 696, 693, 704, 682, 717, 684, 680

Corresponding total number of TFBS for each TF (in all genes in selected Gene ontology terms):
(In the same order as above)
661, 664, 706, 645, 735, 613, 625, 594, 655, 704, 636, 616, 569, 625, 534, 534

Corresponding number of genes (of selected Gene ontology terms) each TF is involved with:
(In the same order as above)
329, 326, 347, 330, 363, 334, 325, 308, 333, 359, 338, 330, 307, 344, 306, 292

Corresponding number of selected Gene ontology terms each TF is involved with:
(In the same order as above)
5, 5, 5, 5, 5, 5, 5, 5, 5, 5, 5, 5, 5, 5, 5, 5

Cluster for columns 177 to 185, rows 154 to 167

Gene ontology terms

exocytosis | synaptic transmission | postsynaptic membrane | receptor clustering | postsynaptic density | N-methyl-D-aspartate selective glutamate receptor activity | dendritic spine | receptor activity | synaptic transmission, glutamatergic

TFs

V$SMAD_Q6 | V$AP2REP_01 | V$HNF4_Q6_03 | V$HNF4_Q6_02 | V$T3R_Q6 | V$PAX8_B | V$PAX8_01 | V$USF2_Q6 | V$ZIC2_01 | V$TTF1_Q6 | V$VDR_Q6 | V$LRF_Q2 | V$LBP1_Q6 | V$AP4_Q6_01

Information

All related TFs:
(List of all TFs that are related to any of the PWMs)
AP-2rep, AP-4, FBI-1, HNF-4, HNF-4alpha, HNF-4alpha1, HNF-4alpha2, HNF-4alpha3, HNF-4alpha4, LBP-1, LRF, Nkx2-1, OCZF, Pax-8, RAR-alpha, RAR-alpha1, RAR-alpha:RXR-alpha, RAR-alpha:RXR-gamma, RAR-beta, RAR-beta2, RAR-gamma, RXR-alpha, RXR-beta, RXR-beta2, RXR-gamma, Smad1, Smad1.1, Smad2, Smad2-L, Smad3, Smad3:Smad4, Smad4, T3R-alpha, T3R-alpha1, T3R-alpha2, T3R-beta, T3R-beta1, T3R-beta2, USF1:USF2, USF2, USF2a, VDR, ZIC2, Zic2

Ranked gene list:
(All genes of the selected Gene ontology terms with hits of any of the selected TFs, ranked according to the total number of TFBS)
GUK1, CDK5, AKT1, PPP1R9B, MGAT1, SYNGAP1, PSD, FGFR3, DLG4, CAMK2G, PER1, NDOR1, ACHE, FBXL15, SLC2A4RG, RHBDL1, GABARAP, FASN, ARHGEF2, GRIN2D, VAMP2, EGR1, REPIN1, ITGB2, GRIN2C, AGRN, SYT8, STXBP2, SLC25A22, SEPT5, RARA, OPRL1, MAP3K11, MAP2K3, JUNB, ISYNA1, GIT1, FLJ32987, EFNA3, CTSD, TAS1R3, BAIAP3, PPP1R1B, PLCG1, MC1R, ADM2, ADM, SYTL1, SQSTM1, SLC9A3R1, CAMKK1, ACD, UNC13D, ENTPD2, SYT7, RNASEK, LASP1, GRM2, GRIN1, RAB37, FHL3, CAPN10, APEH, SHANK3, KCNC3, GRN, CHRNE, ARHGDIA, TNFSF13, SLC17A7, NR3C2, DBNL, VGF, TNFRSF1B, TNFRSF1A, SLC9A3R2, ProSAPiP1, HCRT, FOSB, CACNA1G, BRD2, ARF1, ACCN4, OPRS1, NRGN, GRIN3B, CD81, ARC, ADRM1, TSC2, RAB3A, NR3C1, MUC1, KCNA2, INS, DKFZp434P0672, CPLX1,

CDK5R1, VAV1, VAMP8, TFAP2A, SHANK1, RHOG, RAB25, MAP1A, IGF1R, HRH3, EPB41, CYB561, CLCN3, CHRNB1, CD63, AQP5, ACCN2

Corresponding total number of TFBS:
(For each gene listed above, the total number of TFBS for any of the selected TFs, multiplied by the number of selected Gene ontology terms containing that gene)
78, 70, 70, 65, 65, 60, 55, 54, 48, 44, 42, 40, 36, 35, 28, 28, 28, 28, 26, 24, 20, 20, 18, 18, 16, 15, 14, 14, 14, 14, 14, 14, 14, 14, 14, 14, 14, 14, 14, 14, 13, 13, 12, 12, 12, 12, 12, 11, 11, 11, 11, 11, 10, 10, 8, 8, 8, 8, 8, 7, 7, 7, 7, 6, 6, 6, 6, 6, 5, 5, 5, 5, 4, 4, 4, 4, 4, 4, 4, 4, 4, 4, 4, 3, 3, 3, 3, 3, 3, 2, 2, 2, 2, 2, 2, 2, 2, 2, 1, 1, 1, 1, 1, 1, 1, 1, 1, 1, 1, 1, 1, 1, 1, 1

Corresponding number of selected Gene ontology terms each gene is involved with:
(In the same order as above)
6, 5, 5, 5, 5, 5, 5, 6, 8, 4, 3, 8, 3, 5, 2, 2, 2, 2, 2, 4, 2, 2, 2, 2, 4, 3, 1, 1, 1, 1, 1, 1, 1, 1, 1, 1, 1, 1, 1, 1, 1, 1, 1, 1, 1, 1, 1, 1, 1, 1, 1, 1, 2, 1, 1, 1, 8, 8, 1, 1, 1, 1, 3, 1, 1, 1, 1, 5, 5, 1, 1, 1, 1, 2, 1, 1, 1, 1, 1, 1, 1, 1, 1, 3, 1, 1, 3, 1, 1, 2, 1, 1, 2, 2, 1, 2, 2, 1, 1, 1, 1, 1, 1, 1, 1, 1, 1, 1, 1, 1, 1, 1

Corresponding number of selected TFs each gene is involved with:
(In the same order as above)
13, 14, 14, 13, 13, 12, 11, 9, 6, 11, 14, 5, 12, 7, 14, 14, 14, 14, 13, 6, 10, 10, 9, 9, 4, 5, 14, 14, 14, 14, 14, 14, 14, 14, 14, 14, 14, 14, 14, 14, 13, 13, 12, 12, 12, 12, 12, 11, 11, 11, 11, 11, 10, 5, 8, 8, 8, 1, 1, 7, 7, 7, 7, 2, 6, 6, 6, 6, 1, 1, 5, 5, 4, 4, 2, 4, 4, 4, 4, 4, 4, 4, 4, 3, 1, 3, 3, 1, 3, 2, 1, 2, 2, 1, 1, 2, 1, 1, 1, 1, 1, 1, 1, 1, 1, 1, 1, 1, 1, 1, 1, 1, 1, 1

Gene ontology terms ranked according to mean of effect sizes in cluster:
postsynaptic membrane | dendritic spine | N-methyl-D-aspartate selective glutamate receptor activity | synaptic transmission | postsynaptic density | receptor clustering | exocytosis | synaptic transmission, glutamatergic | receptor activity

Corresponding mean of effect sizes of each Gene ontology term:
(In the same order as above)
1.99, 1.54, 1.44, 1.31, 1.20, 1.14, 0.97, 0.69, 0.68

Corresponding total number of genes of each Gene ontology term:
(In the same order as above)
62, 91, 208, 251, 162, 79, 381, 38, 187

Corresponding number of genes of each Gene ontology term with at least one TFBS (of selected TFs):
(In the same order as above)
17, 18, 32, 36, 27, 16, 48, 7, 24

Corresponding total number of TFBS (of selected TFs) for each Gene ontology term (in all genes):
(In the same order as above)
110, 156, 257, 245, 173, 102, 344, 40, 175

Corresponding number of selected TFs each Gene ontology term is involved with:
(In the same order as above)
14, 14, 14, 14, 14, 14, 14, 14, 14

TFs ranked according to mean of effect sizes in cluster:
V$ZIC2_01, V$AP4_Q6_01, V$USF2_Q6, V$HNF4_Q6_02, V$HNF4_Q6_03, V$LRF_Q2, VVDR_Q6, VAP2REP_01, V$TTF1_Q6, V$SMAD_Q6, V$LBP1_Q6, V$T3R_Q6, V$PAX8_01, V$PAX8_B

Corresponding mean of effect sizes of each TF:

(In the same order as above)
1.57, 1.49, 1.45, 1.42, 1.40, 1.32, 1.32, 1.25, 1.24, 1.16, 1.13, 1.01, 0.70, 0.58

Corresponding total number of TFBS for each TF (genome-wide):
(In the same order as above)
716, 699, 703, 699, 701, 719, 692, 700, 714, 729, 691, 698, 728, 712

Corresponding total number of TFBS for each TF (in all genes in selected Gene ontology terms):
(In the same order as above)
127, 116, 127, 118, 117, 121, 109, 117, 113, 114, 109, 107, 107, 100

Corresponding number of genes (of selected Gene ontology terms) each TF is involved with:
(In the same order as above)
61, 57, 60, 57, 60, 59, 55, 56, 58, 61, 57, 53, 58, 53

Corresponding number of selected Gene ontology terms each TF is involved with:
(In the same order as above)
9, 9, 9, 9, 9, 9, 9, 9, 9, 9, 9, 9, 9, 9

Cluster for columns 210 to 246, rows 400 to 409

Gene ontology terms

phospholipase activity | bile acid biosynthetic process | bile acid metabolic process | lipid binding | sequestering of lipid | cholesterol biosynthetic process | fatty acid biosynthetic process | fatty acid beta-oxidation | fatty acid binding | fatty acid transport | lipid biosynthetic process | triacylglycerol biosynthetic process | lipid catabolic process | fatty acid oxidation | lipid metabolic process | lipid oxidation | peroxisome | peroxisome proliferator activated receptor binding | low-density lipoprotein particle | cholesterol absorption | cholesterol esterification | low-density lipoprotein receptor activity | cholesterol homeostasis | cholesterol metabolic process | cholesterol efflux | cholesterol transport | reverse cholesterol transport | lipoprotein metabolic process | high-density lipoprotein particle | phosphatidylcholine-sterol O-acyltransferase activity | lipid homeostasis | lipid transport | lipoprotein lipase activity | acyltransferase activity | chylomicron | lipoprotein biosynthetic process | triacylglycerol metabolic process

TFs

V$PPAR_DR1_Q2 | V$DR1_Q3 | V$HNF4_DR1_Q3 | V$COUP_DR1_Q6 | V$COUP_01 | V$HNF4_01 | V$HNF4_Q6_01 | V$HNF4ALPHA_Q6 | V$COUPTF_Q6 | V$PPARG_03

Information

All related TFs:
(List of all TFs that are related to any of the PWMs)
COUP, COUP-TF1, COUP-TF2, HNF-4, HNF-4alpha, HNF-4alpha1, HNF-4alpha2, HNF-4alpha3, HNF-4alpha4, HNF-4alpha7, HNF-4gamma, PPAR-alpha, PPAR-alpha:RXR-alpha, PPAR-beta, PPAR-gamma, PPAR-gamma1, PPAR-gamma2, PPAR-gamma2:RXR-alpha, PPAR-gamma:RXR-alpha, RAR-alpha:RXR-alpha

Ranked gene list:
(All genes of the selected Gene ontology terms with hits of any of the selected TFs, ranked according to the total number of TFBS)
APOA1, APOC3, PPARA, APOA4, LCAT, LIPE, FASN, MT1B, DGAT1, CPT1B, CHKB, PNPLA2, ABCA2, SOAT2, LPIN1, HMGCS1, ESRRA, AKT1, ACAT2, LRP1, APOM, GCK, SREBF1, SMPD2,

CPT1A, STK11, CTDSP2, NPC1L1, RHOA, HSD17B8, GLP-1, SLC27A5, SKIV2L, RXRB, CRABP2, AGPAT2, TNFSF13, SCP2, PRKCSH, HSD11B1, HADHA, AGPAT1, ACOT8, TNF, NPC1, MGAT1, ACADVL, SHBG, INSR, ACSL1, SCD, PPARD, UCP3, HMGCS2, GNAI2, DBP, CIDEB, APOC1, NUCB2, UCP2, SNX17, PLA2G1B, HADHB, FGFR4, VEGFA, SEC14L2, PEX16, DECR1, CYP27B1, ACADS, SLC25A22, SLC10A2, SELPLG, PNPLA6, PITPNM1, NOS3, NFE2L2, CYP1A1, CHDH, CDH15, ACTA1, ABCB4, XPR1, SSSCA1, SLC25A1, NR5A2, MYBBP1A, FOXO1, CAPN10, ACSL5, ACBD3, SOD2, SFTPC, RXRA, RPL32, RERE, RARA, PPARGC1A, PISD, NR1H2, CSNK2B, CDK4, PCK1, MYO1C, LTB4R, ISYNA1, ETFA, DGAT2, CYP11A1, CAT, ARSA, ANGPTL4, TSPO, SOCS3, SLC2A1, SLC25A20, PDK3, NDUFAB1, NCOA2, MPV17, HSPD1, ECHS1, CLIC1, SLC27A3, RPLP2, PYY, PRKCA, PEX10, PDZK1, PCSK7, PC, OSM, OSBPL2, MBD2, IDH1, CDKN1A, CALM3, BDH1, BAX, AMACR, VTA1, VPS52, RAB7A, PNPLA3, PLD1, PIGW, PICK1, PEX3, PCDH8, NR2F2, NCOR2, MAPK1, LPIN3, LLGL2, INPP5K, HSD3B7, HSD17B10, HNF1A, GYPC, GAPDH, GABPA, EPHB2, ELOVL3, EGR1, DGKQ, ANXA2, AGFG1, ACTB, ACOT7, VSX1, TXNIP, TMEM201, TM7SF2, SULT2B1, STAT5B, STAT3, STARD4, STARD3, SCARF1, S100A9, RPS27A, PURB, PLD2, PGC, OTC, OSTalpha, NKX6-1, MUC17, MST1, MGC138228, LTB, LCN1, KATNB1, HRAS, HEXA, GSPT1, GPT, GLRX, GATA4, GALNS, GADD45B, FH, FBLIM1, CYTH3, CCHCR1, CAPG, CAMKK2, ASPA, ARNTL, ALOX12B, AHSA1, AGTR1

Corresponding total number of TFBS:
(For each gene listed above, the total number of TFBS for any of the selected TFs, multiplied by the number of selected Gene ontology terms containing that gene)
300, 250, 232, 190, 168, 114, 96, 88, 70, 63, 63, 60, 60, 50, 42, 42, 36, 36, 36, 35, 32, 30, 28, 28, 26, 24, 24, 22, 20, 20, 20, 18, 18, 18, 18, 18, 17, 16, 16, 16, 16, 16, 16, 15, 15, 15, 15, 14, 14, 14, 13, 13, 12, 12, 12, 12, 12, 12, 11, 10, 10, 10, 10, 10, 9, 9, 9, 9, 9, 9, 8, 8, 8, 8, 8, 8, 8, 8, 8, 8, 8, 8, 7, 7, 7, 7, 7, 7, 7, 7, 7, 6, 6, 6, 6, 6, 6, 6, 6, 6, 6, 6, 5, 5, 5, 5, 5, 5, 5, 5, 5, 5, 4, 4, 4, 4, 4, 4, 4, 4, 4, 4, 4, 3, 3, 3, 3, 3, 3, 3, 3, 3, 3, 3, 3, 3, 3, 3, 3, 3, 2, 2, 2, 2, 2, 2, 2, 2, 2, 2, 2, 2, 2, 2, 2, 2, 2, 2, 2, 2, 2, 2, 2, 2, 2, 2, 2, 2, 1, 1, 1, 1, 1, 1, 1, 1, 1, 1, 1, 1, 1, 1, 1, 1, 1, 1, 1, 1, 1, 1, 1, 1, 1, 1, 1, 1, 1, 1, 1, 1, 1, 1, 1, 1, 1, 1, 1

Corresponding number of selected Gene ontology terms each gene is involved with:
(In the same order as above)
30, 25, 29, 19, 24, 19, 16, 11, 10, 9, 9, 6, 6, 10, 7, 14, 4, 9, 9, 7, 8, 5, 28, 4, 13, 3, 6, 11, 2, 2, 4, 3, 2, 2, 2, 3, 17, 16, 2, 16, 8, 2, 2, 5, 15, 3, 3, 2, 7, 7, 13, 13, 12, 12, 2, 3, 2, 12, 11, 10, 1, 10, 5, 2, 1, 1, 1, 9, 1, 3, 1, 8, 8, 2, 2, 2, 2, 1, 8, 4, 4, 8, 7, 1, 1, 7, 1, 7, 1, 7, 1, 2, 2, 6, 1, 3, 3, 6, 2, 6, 1, 1, 5, 1, 1, 1, 1, 5, 5, 5, 1, 5, 1, 1, 4, 4, 1, 2, 1, 1, 1, 2, 1, 1, 3, 1, 1, 1, 3, 1, 3, 1, 1, 1, 1, 1, 1, 3, 1, 3, 1, 2, 2, 2, 2, 1, 1, 1, 2, 2, 1, 2, 2, 1, 1, 1, 2, 1, 1, 2, 2, 1, 1, 1, 1, 2, 1, 1, 1, 1, 1, 1, 1, 1, 1, 1, 1, 1, 1, 1, 1, 1, 1, 1, 1, 1, 1, 1, 1, 1, 1, 1, 1, 1, 1, 1, 1, 1, 1, 1, 1, 1, 1, 1, 1, 1, 1, 1, 1, 1, 1, 1

Corresponding number of selected TFs each gene is involved with:
(In the same order as above)
10, 10, 8, 10, 7, 6, 6, 8, 7, 7, 7, 10, 10, 5, 6, 3, 9, 4, 4, 5, 4, 6, 1, 7, 2, 8, 4, 2, 10, 10, 5, 6, 9, 9, 9, 6, 1, 1, 8, 1, 2, 8, 8, 3, 1, 5, 5, 7, 2, 2, 1, 1, 1, 1, 6, 4, 6, 1, 1, 1, 10, 1, 2, 5, 9, 9, 9, 1, 9, 3, 8, 1, 1, 4, 4, 4, 4, 8, 1, 2, 2, 1, 1, 7, 7, 1, 7, 1, 7, 1, 7, 3, 3, 1, 6, 2, 2, 1, 3, 1, 6, 6, 1, 5, 5, 5, 5, 1, 1, 1, 5, 1, 4, 4, 1, 1, 4, 2, 4, 4, 4, 2, 4, 3, 1, 3, 3, 3, 1, 3, 1, 3, 3, 3, 3, 3, 3, 1, 3, 1, 2, 1, 1, 1, 1, 2, 2, 2, 1, 1, 2, 1, 1, 2, 2, 2, 1, 2, 2, 1, 1, 2, 2, 2, 2, 1, 2, 2, 2, 1, 1, 1, 1, 1, 1, 1, 1, 1, 1, 1, 1, 1, 1, 1, 1, 1, 1, 1, 1, 1, 1, 1, 1, 1, 1, 1, 1, 1, 1, 1, 1, 1, 1, 1, 1, 1, 1, 1, 1, 1

Gene ontology terms ranked according to mean of effect sizes in cluster:
lipoprotein biosynthetic process | cholesterol esterification | cholesterol absorption | chylomicron | triacylglycerol metabolic process | phospholipase activity | cholesterol homeostasis | lipid transport | phosphatidylcholine-sterol O-acyltransferase activity | lipid homeostasis | lipoprotein metabolic process | lipoprotein lipase activity | reverse cholesterol transport | cholesterol transport | cholesterol efflux | high-density lipoprotein particle | lipid biosynthetic process | lipid metabolic process | cholesterol metabolic process | low-density lipoprotein receptor activity | lipid binding | fatty acid biosynthetic process | fatty acid transport | cholesterol biosynthetic process | fatty acid oxidation | lipid oxidation | acyltransferase activity | sequestering of lipid | peroxisome proliferator activated receptor binding | bile acid biosynthetic process | fatty acid beta-oxidation | low-density lipoprotein particle | lipid catabolic process | bile acid

metabolic process | triacylglycerol biosynthetic process | fatty acid binding | peroxisome

Corresponding mean of effect sizes of each Gene ontology term:
(In the same order as above)
4.69, 4.24, 3.67, 3.66, 3.56, 3.48, 3.47, 3.34, 3.09, 3.07, 2.93, 2.82, 2.77, 2.74, 2.69, 2.66, 2.61, 2.60, 2.51, 2.33, 2.33, 2.31, 2.20, 2.08, 1.95, 1.90, 1.85, 1.84, 1.75, 1.73, 1.72, 1.67, 1.33, 1.21, 1.08, 1.01, 0.80

Corresponding total number of genes of each Gene ontology term:
(In the same order as above)
25, 53, 65, 56, 47, 49, 110, 85, 34, 123, 91, 53, 76, 96, 93, 35, 114, 270, 102, 112, 125, 134, 68, 126, 162, 68, 44, 69, 102, 92, 80, 41, 174, 39, 63, 135, 336

Corresponding number of genes of each Gene ontology term with at least one TFBS (of selected TFs):
(In the same order as above)
7, 15, 18, 14, 12, 12, 30, 21, 8, 31, 18, 16, 20, 25, 19, 8, 34, 63, 27, 26, 29, 35, 21, 29, 47, 21, 9, 14, 28, 23, 25, 7, 33, 9, 15, 33, 74

Corresponding total number of TFBS (of selected TFs) for each Gene ontology term (in all genes):
(In the same order as above)
52, 72, 66, 76, 63, 69, 114, 93, 41, 127, 79, 76, 75, 94, 92, 44, 131, 221, 91, 101, 105, 127, 85, 106, 148, 68, 45, 51, 102, 64, 84, 31, 120, 21, 54, 93, 213

Corresponding number of selected TFs each Gene ontology term is involved with:
(In the same order as above)
10, 10, 10, 10, 10, 10, 10, 10, 10, 10, 10, 10, 10, 10, 10, 10, 10, 10, 10, 10, 10, 10, 10, 10, 10, 10, 10, 10, 10, 10, 10, 10, 10, 10, 10, 10, 10

TFs ranked according to mean of effect sizes in cluster:
V$PPARG_03, V$HNF4_01, V$HNF4_DR1_Q3, V$HNF4ALPHA_Q6, V$COUPTF_Q6, V$DR1_Q3, V$HNF4_Q6_01, V$PPAR_DR1_Q2, V$COUP_DR1_Q6, V$COUP_01

Corresponding mean of effect sizes of each TF:
(In the same order as above)
3.30, 2.98, 2.71, 2.59, 2.58, 2.45, 2.42, 2.16, 1.92, 1.67

Corresponding total number of TFBS for each TF (genome-wide):
(In the same order as above)
647, 674, 666, 688, 696, 667, 687, 684, 651, 660

Corresponding total number of TFBS for each TF (in all genes in selected Gene ontology terms):
(In the same order as above)
415, 356, 336, 353, 314, 329, 323, 316, 278, 274

Corresponding number of genes (of selected Gene ontology terms) each TF is involved with:
(In the same order as above)
77, 73, 67, 67, 62, 66, 69, 70, 63, 68

Corresponding number of selected Gene ontology terms each TF is involved with:
(In the same order as above)
37, 37, 37, 37, 37, 37, 37, 37, 37, 37

Cluster for columns 681 to 684, rows 9 to 17

Gene ontology terms

peptidase activity | protein digestion | endopeptidase activity | secretory granule

TFs

V$CREB_Q4_01 | V$CREB_Q2_01 | V$CREB_Q4 | V$CREBP1_Q2 | V$CREB_Q2 | V$CREB_01 | V$CREBP1CJUN_01 | V$CREBATF_Q6 | V$ATF_B

Information

All related TFs:
(List of all TFs that are related to any of the PWMs)
120-kDa, 47-kDa, ATF, ATF-1, ATF-2, ATF-4, ATF-a, ATF-like, ATF/CREB, ATF2, ATF2-isoform2, ATF3, ATF4, ATF5, ATF6, ATFa-isoform1, CRE-BP1, CRE-BP2, CREB, CREBbeta, CREMalpha, CREMbeta, CREMgamma, CREMtau, CREMtau1, CREMtau2, CREMtaualpha, c-Jun, deltaCREB

Ranked gene list:
(All genes of the selected Gene ontology terms with hits of any of the selected TFs, ranked according to the total number of TFBS)
TPP1, CYCS, MUC1, VAMP2, THOP1, LAP3, UBC, STXBP3, RCE1, PNRC1, FN1, CNDP2, CHGB, BRAF, CALCA, SH2D3C, CDC42, YME1L1, SLC18A2, RTN2, PSENEN, CTSL1, CASP9, SST, SNAP25, SCG2, RAB3A, PNPLA4, VGF, PCOLCE, IKBKAP, CGB, APPBP2, PVRL2, OSGEP, CHGA, CALM2, UNC13D, STX2, SH2D3A, PARK7, ICA1

Corresponding total number of TFBS:
(For each gene listed above, the total number of TFBS for any of the selected TFs, multiplied by the number of selected Gene ontology terms containing that gene)
18, 18, 16, 12, 12, 12, 9, 9, 9, 9, 9, 9, 9, 9, 8, 7, 7, 6, 6, 6, 6, 6, 6, 4, 4, 4, 4, 4, 3, 3, 3, 3, 3, 2, 2, 2, 2, 1, 1, 1, 1, 1

Corresponding number of selected Gene ontology terms each gene is involved with:
(In the same order as above)
2, 2, 2, 2, 2, 2, 1, 1, 1, 1, 1, 1, 1, 1, 2, 1, 1, 1, 1, 1, 1, 2, 1, 2, 2, 1, 1, 1, 1, 1, 1, 1, 1, 1, 1, 1, 1, 1, 1, 1, 1, 1

Corresponding number of selected TFs each gene is involved with:
(In the same order as above)
9, 9, 8, 6, 6, 6, 9, 9, 9, 9, 9, 9, 9, 9, 4, 7, 7, 6, 6, 6, 6, 3, 6, 2, 2, 4, 4, 4, 3, 3, 3, 3, 3, 2, 2, 2, 2, 1, 1, 1, 1, 1

Gene ontology terms ranked according to mean of effect sizes in cluster:
endopeptidase activity | secretory granule | peptidase activity | protein digestion

Corresponding mean of effect sizes of each Gene ontology term:
(In the same order as above)
1.99, 1.82, 1.65, 1.06

Corresponding total number of genes of each Gene ontology term:
(In the same order as above)
56, 117, 246, 26

Corresponding number of genes of each Gene ontology term with at least one TFBS (of selected TFs):
(In the same order as above)
10, 19, 21, 2

Corresponding total number of TFBS (of selected TFs) for each Gene ontology term (in all genes):
(In the same order as above)
49, 78, 127, 12

Corresponding number of selected TFs each Gene ontology term is involved with:
(In the same order as above)
9, 9, 9, 9

TFs ranked according to mean of effect sizes in cluster:
VATF_B, VCREB_Q4_01, V$CREBP1_Q2, V$CREBP1CJUN_01, V$CREB_Q4, V$CREB_01, V$CREB_Q2_01, V$CREBATF_Q6, V$CREB_Q2

Corresponding mean of effect sizes of each TF:
(In the same order as above)
2.57, 2.02, 1.99, 1.64, 1.55, 1.54, 1.34, 1.22, 0.80

Corresponding total number of TFBS for each TF (genome-wide):
(In the same order as above)
665, 667, 679, 652, 671, 659, 679, 671, 676

Corresponding total number of TFBS for each TF (in all genes in selected Gene ontology terms):
(In the same order as above)
37, 34, 33, 28, 29, 27, 26, 27, 25

Corresponding number of genes (of selected Gene ontology terms) each TF is involved with:
(In the same order as above)
29, 28, 26, 21, 22, 21, 21, 22, 21

Corresponding number of selected Gene ontology terms each TF is involved with:
(In the same order as above)
4, 4, 4, 4, 4, 4, 4, 4, 4

Cluster for columns 801 to 813, rows 267 to 282

Gene ontology terms

insulin-like growth factor binding protein complex | insulin-like growth factor receptor binding | insulin-like growth factor binding | insulin-like growth factor II binding | insulin-like growth factor I binding | insulin-like growth factor receptor activity | growth factor binding | regulation of growth | glucose transport | glucose homeostasis | insulin binding | insulin receptor binding | response to wounding

TFs

V$ETF_Q6 | V$E2F_Q2 | V$E2F1_Q6 | V$E2F1_Q3 | V$AP2GAMMA_01 | V$AP2ALPHA_01 | V$AP2_Q6_01 | V$AP2_Q6 | V$WT1_Q6 | V$EGR_Q6 | V$MAZ_Q6 | V$MAZR_01 | V$E2F1_Q3_01 | V$HES1_Q2 | V$ACAAT_B | V$AP2_Q3

Information

All related TFs:
(List of all TFs that are related to any of the PWMs)
AP-2, AP-2alpha, AP-2alphaA, AP-2alphaB, AP-2beta, AP-2gamma, DP-1, E2F, E2F+E4, E2F-1, E2F-

3a, E2F-4, ETF, Egr-1, Egr-2, Egr-3, HES-1, MAZ, MAZR, WT1, WT1-del2

Ranked gene list:
(All genes of the selected Gene ontology terms with hits of any of the selected TFs, ranked according to the total number of TFBS)
IGF1R, AKT1, IRS1, CCND1, LBX1, IRS2, TGFB1, IGF2, FOXO3, FOXO1, VEGFA, ACTB, HRAS, HOXA10, RARG, PER1, TNFSF13, SOCS3, MDK, IRF1, CTSD, PAX6, PDGFB, TFAP2A, GNAI2, UBTF, MYO1C, OGFR, HOXC6, FASN, BLVRB, SSSCA1, NKX2-2, MAPK11, MAFA, CUX1, RTN4RL1, GPC1, GFI1, SHC1, PIK3R2, HMHA1, FLJ32987, WNT10B, HBA2, PTPN6, PRKCZ, CISH, SH2B2, PPP1R13B, SP3, SOCS2, RASSF1, NUTF2, NR3C1, HMGB1, CORO7, CAMK2G, TNF, GAPDH, EGR1, BRD1, TRIP10, STMN1, SOS1, RPS6KB2, MAPK1, LRP5, HOXA11, GRB7, CDKN1A, BAX, SRC, SOCS1, RARA, PTP4A1, MPG, MAP2K3, IGF2BP3, CDKN1B, CDC20, ST3GAL4, SLC20A1, PTPRU, KLK1, HBA1, GOT2, ATF3, AKT3, SLC2A1, PPP3CA, PIK3CA, LEPROT, KCNJ4, HSP90AA1, GAB2, CREB1, CALM1, VAMP2, STAT5B, SMAD2, SLC38A2, SDC3, RHOA, PRKCA, PPARD, PPARA, PIGP, PDPK1, PAOX, NUCB2, NOL3, NEDD4, LEPR, GPI, ELAVL1, CYP11A1, CRTC2, CNBP, CASP9, AKT2, ADIPOR1, ADA, TFRC, STX6, STX16, STAT5A, SCARB1, RHOQ, RFTN1, RAPGEF1, RAB40B, PTK2, PPM2C, PCK2, ONECUT1, ODC1, NR0B2, MTHFR, MAPK7, MACF1, LRBA, LPIN1, KCNJ11, IHH, IDE, HK1, HDAC3, G6PC3, FOS, EP300, CAPN10, CALM3, CALM2, BIK, ARF6, APPL1

Corresponding total number of TFBS:
(For each gene listed above, the total number of TFBS for any of the selected TFs, multiplied by the number of selected Gene ontology terms containing that gene)
192, 176, 140, 84, 66, 66, 60, 60, 56, 54, 48, 48, 45, 40, 36, 36, 33, 28, 28, 27, 27, 24, 22, 20, 20, 16, 16, 15, 15, 15, 15, 14, 14, 14, 14, 14, 13, 13, 13, 12, 12, 12, 12, 11, 11, 10, 10, 10, 9, 9, 8, 8, 8, 8, 8, 8, 8, 8, 7, 7, 7, 7, 6, 6, 6, 6, 6, 6, 6, 6, 6, 6, 5, 5, 5, 5, 5, 5, 5, 5, 5, 4, 4, 4, 4, 4, 4, 4, 4, 3, 3, 3, 3, 3, 3, 3, 3, 3, 2, 2, 2, 2, 2, 2, 2, 2, 2, 2, 2, 2, 2, 2, 2, 2, 2, 2, 2, 2, 2, 2, 2, 1, 1, 1, 1, 1, 1, 1, 1, 1, 1, 1, 1, 1, 1, 1, 1, 1, 1, 1, 1, 1, 1, 1, 1, 1, 1, 1, 1, 1, 1, 1, 1, 1

Corresponding number of selected Gene ontology terms each gene is involved with:
(In the same order as above)
12, 11, 10, 6, 6, 6, 6, 12, 4, 6, 8, 4, 3, 5, 4, 4, 11, 2, 2, 3, 3, 2, 2, 5, 2, 1, 1, 1, 5, 1, 5, 1, 1, 1, 1, 1, 1, 1, 1, 3, 3, 1, 1, 1, 1, 1, 2, 2, 1, 3, 1, 1, 1, 1, 1, 1, 1, 1, 7, 1, 1, 1, 1, 3, 2, 1, 6, 2, 2, 3, 6, 6, 5, 1, 1, 5, 1, 1, 5, 1, 1, 4, 1, 1, 4, 4, 2, 1, 1, 3, 1, 3, 3, 1, 1, 1, 1, 1, 1, 2, 1, 1, 1, 1, 2, 1, 2, 1, 1, 1, 2, 1, 2, 2, 1, 2, 2, 1, 1, 1, 2, 1, 2, 1, 1, 1, 1, 1, 1, 1, 1, 1, 1, 1, 1, 1, 1, 1, 1, 1, 1, 1, 1, 1, 1, 1, 1, 1, 1, 1, 1, 1

Corresponding number of selected TFs each gene is involved with:
(In the same order as above)
16, 16, 14, 14, 11, 11, 10, 5, 14, 9, 6, 12, 15, 8, 9, 9, 3, 14, 14, 9, 9, 12, 11, 4, 10, 16, 16, 15, 3, 15, 3, 14, 14, 14, 14, 14, 13, 13, 13, 4, 4, 12, 12, 11, 11, 10, 5, 5, 9, 3, 8, 8, 8, 8, 8, 8, 8, 8, 1, 7, 7, 7, 6, 2, 3, 6, 1, 3, 3, 2, 1, 1, 1, 5, 5, 1, 5, 5, 1, 5, 5, 1, 4, 4, 1, 1, 2, 4, 4, 1, 3, 1, 1, 3, 3, 3, 3, 3, 2, 1, 2, 2, 2, 2, 1, 2, 1, 2, 2, 2, 1, 2, 1, 1, 2, 1, 1, 2, 2, 2, 1, 2, 1, 1, 1, 1, 1, 1, 1, 1, 1, 1, 1, 1, 1, 1, 1, 1, 1, 1, 1, 1, 1, 1, 1, 1, 1, 1, 1, 1, 1, 1, 1, 1, 1, 1

Gene ontology terms ranked according to mean of effect sizes in cluster:
insulin-like growth factor receptor activity | insulin-like growth factor receptor binding | insulin-like growth factor I binding | insulin binding | insulin-like growth factor binding | insulin receptor binding | insulin-like growth factor II binding | growth factor binding | insulin-like growth factor binding protein complex | glucose transport | glucose homeostasis | response to wounding | regulation of growth

Corresponding mean of effect sizes of each Gene ontology term:
(In the same order as above)
1.53, 1.44, 1.33, 1.26, 1.25, 1.16, 1.12, 1.11, 0.96, 0.94, 0.87, 0.80, 0.79

Corresponding total number of genes of each Gene ontology term:
(In the same order as above)

71, 173, 132, 33, 149, 20, 132, 93, 88, 209, 197, 26, 107

Corresponding number of genes of each Gene ontology term with at least one TFBS (of selected TFs):
(In the same order as above)
19, 40, 27, 11, 33, 10, 26, 20, 19, 56, 48, 7, 37

Corresponding total number of TFBS (of selected TFs) for each Gene ontology term (in all genes):
(In the same order as above)
141, 247, 182, 66, 196, 62, 183, 134, 126, 270, 251, 52, 185

Corresponding number of selected TFs each Gene ontology term is involved with:
(In the same order as above)
16, 16, 16, 16, 16, 16, 16, 16, 16, 16, 16, 16, 16

TFs ranked according to mean of effect sizes in cluster:
VEGR_Q6, VWT1_Q6, V$MAZR_01, V$ACAAT_B, V$AP2GAMMA_01, V$AP2ALPHA_01, V$E2F1_Q3, V$AP2_Q6_01, V$E2F1_Q6, V$ETF_Q6, VMAZ_Q6, VHES1_Q2, V$AP2_Q6, V$E2F_Q2, V$AP2_Q3, V$E2F1_Q3_01

Corresponding mean of effect sizes of each TF:
(In the same order as above)
1.83, 1.65, 1.56, 1.28, 1.23, 1.20, 1.19, 1.06, 1.05, 1.02, 0.99, 0.99, 0.94, 0.88, 0.84, 0.22

Corresponding total number of TFBS for each TF (genome-wide):
(In the same order as above)
695, 729, 705, 692, 695, 686, 709, 690, 691, 699, 734, 690, 688, 714, 683, 680

Corresponding total number of TFBS for each TF (in all genes in selected Gene ontology terms):
(In the same order as above)
155, 160, 133, 148, 128, 126, 130, 126, 133, 127, 124, 134, 129, 129, 113, 100

Corresponding number of genes (of selected Gene ontology terms) each TF is involved with:
(In the same order as above)
52, 56, 43, 54, 50, 50, 52, 44, 52, 50, 48, 53, 50, 56, 46, 35

Corresponding number of selected Gene ontology terms each TF is involved with:
(In the same order as above)
13, 13, 13, 13, 13, 13, 13, 13, 13, 13, 13, 13, 13, 13, 13, 13

Cluster for columns 228 to 245, rows 262 to 265

Gene ontology terms

low-density lipoprotein particle | cholesterol absorption | cholesterol esterification | low-density lipoprotein receptor activity | cholesterol homeostasis | cholesterol metabolic process | cholesterol efflux | cholesterol transport | reverse cholesterol transport | lipoprotein metabolic process | high-density lipoprotein particle | phosphatidylcholine-sterol O-acyltransferase activity | lipid homeostasis | lipid transport | lipoprotein lipase activity | acyltransferase activity | chylomicron | lipoprotein biosynthetic process

TFs

V$BLIMP1_Q6 | V$CACCCBINDINGFACTOR_Q6 | V$FXR_Q3 | V$FXR_IR1_Q6

Information

All related TFs:
(List of all TFs that are related to any of the PWMs)
Blimp-1, CACCC-binding, FOR1, FOR2, FXR, FXR-alpha, FXR:RXR-alpha

Ranked gene list:
(All genes of the selected Gene ontology terms with hits of any of the selected TFs, ranked according to the total number of TFBS)
PLTP, APOA1, TNFSF13, NLRP3, APOB, CETP, NR1H3, APOC3, INS, APOA2, SCARB1, NR0B2, RXRB, DHCR7, MT1B, APOM, SCAP, PPARD, PER1, APP, ABCA2, MGAT1, HDLBP, SLC25A22, PON2, MUC17, LEP, KRT81, GOT2, GNAI2, FASN, CYP11A1, COG2, SNX17, PPAT, PNPLA6, MLXIPL, LRP2, FABP6, DGAT1, CD68, ANXA2, ANGPTL4, TRERF1, TNF, TFAP2A, SLC27A1, SLC16A1, SKIV2L, SFTPC, SCARF1, PITPNA, PCTP, PCSK7, OSM, MST1, MGLL, LTB, LRPAP1, LDLRAP1, HBA2, GALNS, FOXA2, FGF21, FABP3, ENPP7, DAB2, CPT1A, COL4A3BP, COASY, CDT1, ADIPOR2, ACTB

Corresponding total number of TFBS:
(For each gene listed above, the total number of TFBS for any of the selected TFs, multiplied by the number of selected Gene ontology terms containing that gene)
48, 36, 20, 18, 18, 17, 16, 16, 15, 13, 12, 12, 8, 8, 7, 7, 6, 6, 6, 6, 5, 4, 4, 3, 3, 3, 3, 3, 3, 3, 3, 3, 3, 2, 2, 2, 2, 2, 2, 2, 2, 2, 2, 1, 1, 1, 1, 1, 1, 1, 1, 1, 1, 1, 1, 1, 1, 1, 1, 1, 1, 1, 1, 1, 1, 1, 1, 1, 1, 1, 1, 1, 1

Corresponding number of selected Gene ontology terms each gene is involved with:
(In the same order as above)
16, 18, 10, 9, 18, 17, 8, 16, 15, 13, 12, 6, 2, 4, 7, 7, 6, 6, 2, 6, 5, 2, 4, 1, 3, 1, 3, 3, 3, 1, 3, 3, 3, 1, 1, 1, 2, 2, 1, 2, 1, 1, 2, 1, 1, 1, 1, 1, 1, 1, 1, 1, 1, 1, 1, 1, 1, 1, 1, 1, 1, 1, 1, 1, 1, 1, 1, 1, 1, 1, 1, 1, 1

Corresponding number of selected TFs each gene is involved with:
(In the same order as above)
3, 2, 2, 2, 1, 1, 2, 1, 1, 1, 1, 2, 4, 2, 1, 1, 1, 1, 3, 1, 1, 2, 1, 3, 1, 3, 1, 1, 1, 3, 1, 1, 1, 2, 2, 2, 1, 1, 2, 1, 2, 2, 1, 1, 1, 1, 1, 1, 1, 1, 1, 1, 1, 1, 1, 1, 1, 1, 1, 1, 1, 1, 1, 1, 1, 1, 1, 1, 1, 1, 1, 1, 1

Gene ontology terms ranked according to mean of effect sizes in cluster:
cholesterol efflux | cholesterol absorption | reverse cholesterol transport | high-density lipoprotein particle | cholesterol transport | low-density lipoprotein particle | phosphatidylcholine-sterol O-acyltransferase activity | lipoprotein lipase activity | cholesterol homeostasis | lipoprotein metabolic process | lipid transport | cholesterol metabolic process | low-density lipoprotein receptor activity | chylomicron | lipid homeostasis | cholesterol esterification | lipoprotein biosynthetic process | acyltransferase activity

Corresponding mean of effect sizes of each Gene ontology term:
(In the same order as above)
2.37, 2.19, 2.09, 2.04, 1.82, 1.75, 1.69, 1.64, 1.60, 1.59, 1.31, 1.26, 1.19, 1.16, 1.07, 0.95, 0.93, 0.72

Corresponding total number of genes of each Gene ontology term:
(In the same order as above)
93, 65, 76, 35, 96, 41, 34, 53, 110, 91, 85, 102, 112, 56, 123, 53, 25, 44

Corresponding number of genes of each Gene ontology term with at least one TFBS (of selected TFs):
(In the same order as above)
23, 12, 17, 11, 21, 9, 7, 15, 20, 18, 17, 20, 26, 13, 26, 9, 6, 7

Corresponding total number of TFBS (of selected TFs) for each Gene ontology term (in all genes):
(In the same order as above)
35, 19, 26, 15, 31, 13, 11, 23, 31, 23, 23, 27, 31, 16, 33, 12, 7, 12

Corresponding number of selected TFs each Gene ontology term is involved with:
(In the same order as above)
4, 4, 4, 4, 4, 4, 4, 4, 4, 4, 4, 4, 4, 4, 4, 4, 3, 4

TFs ranked according to mean of effect sizes in cluster:
V$CACCCBINDINGFACTOR_Q6, V$BLIMP1_Q6, VFXR_IR1_Q6, VFXR_Q3

Corresponding mean of effect sizes of each TF:
(In the same order as above)
2.46, 1.55, 1.11, 0.95

Corresponding total number of TFBS for each TF (genome-wide):
(In the same order as above)
684, 679, 659, 666

Corresponding total number of TFBS for each TF (in all genes in selected Gene ontology terms):
(In the same order as above)
122, 96, 89, 81

Corresponding number of genes (of selected Gene ontology terms) each TF is involved with:
(In the same order as above)
29, 21, 25, 24

Corresponding number of selected Gene ontology terms each TF is involved with:
(In the same order as above)
18, 18, 18, 17

Cluster for columns 461 to 539, rows 133 to 138

Gene ontology terms

B cell activation | immunoglobulin production | B cell proliferation | immunoglobulin secretion | interferon-gamma production | T-helper 1 type immune response | response to host immune response | adaptive immune response | adaptive immune response based on somatic recombination of immune receptors built from immunoglobulin superfamily domains | chemokine production | interleukin-10 production | interleukin-12 production | cell maturation | MHC class I biosynthetic process | MHC class I protein binding | MHC class II biosynthetic process | MHC class II protein binding | antigen processing and presentation | immune response | type IV hypersensitivity | lymphocyte proliferation | tolerance induction | lymphocyte activation | interleukin-2 production | interleukin-2 receptor activity | T cell proliferation | interleukin-4 production | cytokine production | cytokine secretion | pathogenesis | interleukin-4 receptor activity | interleukin-5 production | evasion or tolerance of immune response of other organism during symbiotic interaction | tryptophan catabolic process | defense response to virus | innate immune response | tumor necrosis factor receptor activity | tumor necrosis factor receptor binding | positive regulation of NF-kappaB transcription factor activity | response to tumor necrosis factor | IkappaB kinase complex | NF-kappaB binding | interleukin-6 receptor activity | interleukin-1 beta production | interleukin-1 production | cytolysis | natural killer cell mediated cytotoxicity | natural killer cell receptor activity | naringenin-chalcone synthase activity | immature T cell proliferation in the thymus | eosinophil activation | leukocyte activation | monocyte activation | mucosal immune response | granulocyte macrophage colony-stimulating factor biosynthetic process | granulocyte macrophage colony-stimulating factor production | interleukin-1 receptor activity | interleukin-6 production | tumor necrosis factor production | macrophage activation | inflammatory response | response to lipopolysaccharide | negative regulation of inflammatory response | regulation of tumor necrosis factor production | negative regulation of tumor necrosis factor production | positive regulation of tumor necrosis factor production | interleukin-1 receptor antagonist activity | acute inflammatory response | granuloma formation |

lipopolysaccharide binding | cytokine biosynthetic process | interleukin-8 production | mRNA transcription | chronic inflammatory response | monocyte differentiation | hypersensitivity | hyaluronic acid binding | cytokine activity | rosetting

TFs

V$IRF_Q6_01 | V$IRF_Q6 | V$IRF7_01 | V$ISRE_01 | V$ICSBP_Q6 | V$IRF1_01

Information

All related TFs:
(List of all TFs that are related to any of the PWMs)
IRF-1, IRF-10, IRF-2, IRF-3, IRF-4, IRF-5, IRF-6, IRF-7, IRF-7A, IRF-7H, IRF-8, IRF-9, ISGF-3

Ranked gene list:
(All genes of the selected Gene ontology terms with hits of any of the selected TFs, ranked according to the total number of TFBS)
STAT1, IL15, TNFSF13, FAS, ISG20, IFNB1, TLR3, PARP9, IL7, NR3C2, CXCL10, TLR4, IFNG, NFKBIA, IL6, IL2, MYD88, CD4, CD40, HLA-A, CSF1, TNFSF13B, STAT5A, NOD1, ITGB2, IL27, CD80, IL18, CCL2, SOCS1, IL3, NOD2, TAP1, PSMB8, CASP1, TFRC, LCK, WNK1, EIF2AK2, EGR1, CXCR4, TNFSF10, CD2BP2, TAP2, NR3C1, IRF2, CD274, B2M, SILV, SCARB2, PIK3CG, PIK3CA, TLR1, FOXP3, CD44, CCR5, CTLA4, RFX5, RAF1, PSMB10, MAP2K3, LMNA, HLA-C, FLT3LG, EPHB2, CADM1, ANXA6, TBX21, PTMA, IL7R, HLA-B, STAT3, PTPN6, OSM, NLRP3, MAP3K5, IRF7, FYN, ERAP1, CD27, BCL6, IRF1, PSMB9, LAG3, ITGA4, ISG15, FOXP1, CIITA, CD5, CCR4, CCL21, BIRC3, AKT1, STAT6, TAPBP, SLC17A5, PTGER4, PRDM1, MX1, CCR6, TNFSF4, RELB, MAP3K1, IRS1, HSPD1, DDX58, CCL3, TBK1, MYB, INDO, IL17C, HLA-G, DUSP1, CD74, CD38, STAT5B, SOAT1, OMG, UBC, TNFAIP3, TFAP2A, RIPK1, PDCD1LG2, NR4A2, MAP4K4, MAP3K14, LY86, LY75, KARS, IRF5, IL1RAP, IFIH1, HSPA8, HLA-DRB1, HIVEP2, GATA3, DHX58, CTRL, CSK, CFLAR, CDKN2A, CDKN1B, CASP8, TNFSF11, NFKB1, LIF, KCNA3, IRF3, CCR3, B3GAT1, TTF2, PIK3R1, PER1, PDCD1, NEDD9, IKBKE, IFI44, GALNT6, CCND1, ARL4C, TNFSF15, TNFRSF13C, TNFRSF10A, TLR8, PTK2B, ITGB1, DLX2, CD47, WARS, TSLP, TRAF5, TCIRG1, TAF8, SLC25A22, PCNA, PAX5, MAP3K7IP1, JUNB, GRAP2, CREB1, CISH, CDKN2D, CD63, CD48, CD200R1, BAK1, APP, ADM, SOD1, PSMC1, IL6R, CTBS, CARD11, UNK, TRIM21, TMEM201, STATH, SOS1, RUNX1, RPE, RGS1, PYCARD, PTCH1, PSME1, PSMB5, PDGFA, NFKBIZ, MEFV, MARK2, LOXL1, IRF4, INPP5D, IFIT3, IFIT1, HSPA1B, HLA-DMA, HIVEP1, GADD45B, EHD1, ATM, AGRN, ZAP70, TREX1, TNFRSF17, SP1, SOD2, SLC26A3, PLAU, PDE4A, P2RX7, MAPKAPK2, MAP2K1, LRDD, IFIT2, GRB10, FADD, EOMES, EDARADD, DDR1, CHI3L2, CASP9, BCL11A, BATF, ARID3B, AIMP2, VWF, VHLL, USF1, TXNRD3, TBP, SP140, SIGLEC1, SELPLG, SCGB1A1, SART3, RXRB, RHOF, PSMC5, PML, PLXNC1, OGFR, NUDT6, NFYA, MT2A, MBNL1, MAT2B, LY6E, LILRB4, LAP3, ITGB7, IL18R1, HIF1A, GTPBP1, GGPS1, GABPA, FGL2, ERAP2, EIF4G2, EDN1, DPYSL2, DAXX, CTSS, CSF3R, COL11A2, CIR, CD6, CD300A, ATF3, ARID4B, ARF6, ALOX5AP, ALCAM, ZFP36, XDH, USP18, UCP2, UBE2L6, TNIP1, TIPARP, TICAM2, TGM2, TFPI, STK16, SMAD4, SFRS2, RSAD2, RAPGEF3, PKD2L1, PAK2, NTRK1, NF1, MED25, MAP3K11, LRP2, LEF1, KSR1, ITGA1, IL12RB1, IFT122, HLA-DOA, HLA-DMB, HIST2H2BE, GNAS, FMR1, FLJ32987, FAF1, ETV6, DCN, DCLRE1C, DAPP1, CNTFR, CD37, CCRL2, CCND3, CCND2, CASP2, CALCA, ART3, ANXA5, AKR1B1, AGPAT4, ZC3H12A, XRCC5, WAS, UVRAG, USO1, ULBP2, UBA7, TXNIP, TRIM69, TRAF3IP2, TIMD4, THBS1, TBC1D1, SUB1, ST5, S1PR1, RUNX3, RNASEL, PSMC2, PRKD1, PRKAB1, PPIH, PON1, POLD4, PLUNC, PLEKHF1, PINK1, PIK3CD, NPPA, NP, NOTCH1, NFKBIE, NCSTN, MYO1A, MX2, MTMR11, MIA, MED15, MAL, LTB4R, LRMP, LMLN, LDHB, KLRG1, KIFC1, KHDRBS1, IVNS1ABP, IRF9, IL29, IL20RA, IL1RAPL1, IL18RAP, HSH2D, HPSE, HLA-F, HIVEP3, GPR172B, GLUL, GLIPR2, GFI1, GBP1, GAPDH, GALT, FOXO3, FOXE1, FHL3, EZR, ERN1, EIF2S1, ECE1, DYM, DLAT, CYLD, CD84, CD79A, CCR10, CAST, CAMK2G, CALM3, C8orf4, C1QBP, BAG1, ATP7B, ASRGL1, APOE, APEX1, AIF1, AGMAT, ACD, ABCD1, ABCA1,

ZBTB16, YARS, YAP1, WNK4, VCP, UTS2R, TSKU, TRAFD1, TP63, TNFSF14, TIFA, TCEA3, TBC1D9, SULT1E1, SUFU, STRA13, STAG2, STAG1, ST6GAL1, ST3GAL4, SNRNP70, SMARCA2, SLC2A1, SKP1, SHH, SH3BGRL, SH3BGR, SEMA4D, SCN2A, RPS14, RASA1, RAD52, PTPN7, PRDX5, PRDX2, PPFIBP1, POLD3, PLAA, PHOX2B, PDE4DIP, PDCD5, PCDHA@, PCDHA4, PAK1, NXT1, NRP1, NLRP6, NFE2, NEU4, NDUFAB1, NBN, NAMPT, MYBPC3, MLL, MGEA5, MBNL2, MBD4, MAZ, MAP3K13, LOX, KRIT1, KMO, IL28A, IER3, ID1, HSPE1, HMMR, GUK1, GNB2L1, GHRL, GBP2, GBA, FTH1, FKBP5, FIP1L1, FHL2, FBL, FANCD2, ETS1, ERCC4, ERCC2, ELL, ELF4, ELA1, EIF2AK4, EHF, DYSF, DUSP28, DIABLO, DDX5, DAPK1, CYBA, CUGBP1, CSRP1, CRX, COPB1, COL6A1, CHRNB2, CDK5, CD3D, CCNT1, CCNB1, CARD8, CARD6, CALM2, CALM1, BSG, BLMH, BHLHB2, BCL3, BACE1, ATXN7, ATXN3, ATXN1, APC, AGFG1, ADORA3, ADAR, ACTN4, AASS

Corresponding total number of TFBS:
(For each gene listed above, the total number of TFBS for any of the selected TFs, multiplied by the number of selected Gene ontology terms containing that gene)
170, 129, 118, 117, 110, 98, 96, 95, 90, 84, 84, 82, 76, 75, 73, 73, 68, 66, 62, 60, 54, 52, 52, 52, 50, 50, 49, 47, 47, 44, 43, 42, 40, 40, 40, 38, 38, 34, 33, 33, 33, 32, 32, 30, 30, 30, 30, 30, 28, 28, 28, 28, 27, 26, 26, 26, 25, 24, 24, 24, 24, 24, 24, 24, 24, 24, 23, 21, 21, 21, 21, 20, 20, 20, 20, 20, 20, 20, 20, 20, 20, 19, 18, 18, 18, 18, 18, 18, 18, 18, 18, 18, 18, 17, 16, 16, 16, 16, 16, 16, 15, 15, 15, 15, 15, 15, 15, 14, 14, 14, 14, 14, 14, 14, 14, 13, 13, 13, 12, 12, 12, 12, 12, 12, 12, 12, 12, 12, 12, 12, 12, 12, 12, 12, 12, 12, 12, 12, 12, 12, 12, 12, 12, 11, 11, 11, 11, 11, 11, 11, 10, 10, 10, 10, 10, 10, 10, 10, 10, 9, 9, 9, 9, 9, 9, 9, 9, 8, 8, 8, 8, 8, 8, 8, 8, 8, 8, 8, 8, 8, 8, 8, 8, 8, 8, 7, 7, 7, 7, 7, 6, 6, 6, 6, 6, 6, 6, 6, 6, 6, 6, 6, 6, 6, 6, 6, 6, 6, 6, 6, 6, 6, 6, 6, 6, 6, 6, 6, 5, 5, 5, 5, 5, 5, 5, 5, 5, 5, 5, 5, 5, 5, 5, 5, 5, 5, 5, 5, 5, 5, 5, 5, 4, 4, 4, 4, 4, 4, 4, 4, 4, 4, 4, 4, 4, 4, 4, 4, 4, 4, 4, 4, 4, 4, 4, 4, 4, 4, 4, 4, 4, 4, 4, 4, 4, 4, 4, 4, 4, 4, 4, 4, 4, 4, 4, 4, 4, 4, 4, 3, 3, 3, 3, 3, 3, 3, 3, 3, 3, 3, 3, 3, 3, 3, 3, 3, 3, 3, 3, 3, 3, 3, 3, 3, 3, 3, 3, 3, 3, 3, 3, 3, 3, 3, 3, 3, 3, 3, 3, 3, 3, 3, 3, 3, 3, 3, 3, 3, 2, 2, 2, 2, 2, 2, 2, 2, 2, 2, 2, 2, 2, 2, 2, 2, 2, 2, 2, 2, 2, 2, 2, 2, 2, 2, 2, 2, 2, 2, 2, 2, 2, 2, 2, 2, 2, 2, 2, 2, 2, 2, 2, 2, 2, 2, 2, 2, 2, 2, 2, 2, 2, 2, 2, 2, 2, 2, 2, 2, 2, 2, 2, 2, 2, 2, 2, 2, 2, 2, 2, 2, 2, 2, 2, 2, 2, 2, 2, 2, 2, 2, 2, 2, 2, 2, 2, 2, 2, 2, 2, 1

Corresponding number of selected Gene ontology terms each gene is involved with:
(In the same order as above)
34, 43, 59, 39, 55, 49, 24, 19, 30, 21, 28, 41, 76, 25, 73, 73, 34, 66, 62, 12, 27, 13, 13, 13, 50, 10, 49, 47, 47, 11, 43, 21, 8, 8, 20, 19, 19, 34, 11, 11, 11, 8, 32, 6, 6, 6, 15, 5, 14, 14, 14, 14, 27, 26, 26, 26, 25, 6, 8, 6, 6, 6, 8, 12, 24, 12, 23, 21, 7, 7, 7, 20, 10, 5, 4, 5, 10, 5, 5, 20, 4, 19, 9, 6, 18, 3, 3, 9, 18, 9, 9, 6, 18, 17, 4, 4, 8, 8, 4, 8, 15, 15, 5, 3, 15, 5, 15, 7, 7, 14, 14, 14, 7, 7, 14, 13, 13, 13, 12, 6, 4, 6, 12, 2, 6, 6, 3, 12, 12, 4, 4, 4, 3, 12, 4, 3, 2, 3, 3, 6, 3, 4, 12, 11, 11, 11, 11, 11, 11, 11, 10, 5, 10, 5, 5, 5, 2, 2, 5, 10, 3, 9, 9, 9, 3, 9, 3, 3, 2, 8, 4, 2, 2, 2, 2, 4, 2, 4, 4, 8, 8, 4, 8, 8, 4, 2, 4, 2, 7, 7, 7, 7, 7, 2, 2, 2, 6, 6, 3, 2, 2, 3, 2, 3, 3, 2, 2, 6, 2, 3, 6, 2, 1, 1, 3, 3, 2, 3, 2, 3, 1, 5, 1, 5, 5, 5, 5, 5, 5, 5, 5, 5, 1, 1, 5, 5, 1, 5, 1, 1, 5, 1, 1, 1, 5, 4, 4, 1, 2, 1, 1, 4, 4, 4, 1, 2, 1, 1, 4, 1, 1, 4, 2, 1, 1, 2, 1, 4, 1, 1, 2, 4, 2, 1, 4, 2, 1, 4, 4, 1, 1, 4, 4, 4, 2, 2, 4, 2, 1, 2, 1, 2, 1, 3, 3, 1, 1, 3, 3, 1, 3, 3, 3, 1, 1, 1, 1, 3, 3, 3, 1, 3, 1, 1, 1, 1, 3, 1, 3, 3, 3, 3, 1, 1, 3, 3, 1, 3, 1, 1, 1, 1, 1, 3, 3, 3, 3, 1, 3, 3, 1, 1, 1, 2, 1, 1, 2, 1, 1, 1, 2, 2, 1, 1, 2, 1, 2, 1, 1, 2, 2, 1, 1, 1, 1, 2, 2, 1, 1, 1, 2, 2, 2, 1, 1, 1, 1, 1, 1, 2, 2, 1, 1, 2, 2, 2, 2, 2, 2, 2, 1, 1, 2, 2, 2, 2, 1, 1, 1, 1, 1, 1, 2, 2, 1, 2, 1, 1, 1, 2, 1, 2, 2, 2, 2, 2, 1, 2, 1, 1, 2, 2, 2, 1, 2, 2, 1, 2, 2, 2, 1, 2, 1

Corresponding number of selected TFs each gene is involved with:
(In the same order as above)
5, 3, 2, 3, 2, 2, 4, 5, 3, 4, 3, 2, 1, 3, 1, 1, 2, 1, 1, 5, 2, 4, 4, 4, 1, 5, 1, 1, 1, 4, 1, 2, 5, 5, 2, 2, 2, 1, 3, 3, 3, 4, 1, 5, 5, 5, 2, 6, 2, 2, 2, 2, 1, 1, 1, 1, 1, 4, 3, 4, 4, 4, 3, 2, 1, 2, 1, 1, 3, 3, 3, 1, 2, 4, 5, 4, 2, 4, 4, 1, 5, 1, 2, 3, 1, 6, 6, 2, 1, 2, 2, 3, 1, 1, 4, 4, 2, 2, 4, 2, 1, 1, 3, 5, 1, 3, 1, 2, 2, 1, 1, 1, 2, 2, 1, 1, 1, 1, 1, 2, 3, 2, 1, 6, 2, 2, 4, 1, 1, 3, 3, 3, 4, 1, 3, 4, 6, 4, 4, 2, 4, 3, 1, 1, 1, 1, 1, 1, 1, 1, 1, 2, 1, 2, 2, 2, 5, 5, 2, 1, 3, 1, 1, 1, 3, 1, 3, 3,

4, 1, 2, 4, 4, 4, 4, 2, 4, 2, 2, 1, 1, 2, 1, 1, 2, 4, 2, 4, 1, 1, 1, 1, 1, 3, 3, 3, 1, 1, 2, 3, 3, 2, 3, 2, 2, 3, 3, 1, 3, 2, 1, 3, 6, 6, 2, 2, 3, 2, 3, 2, 6, 1, 5, 1, 1, 1, 1, 1, 1, 1, 1, 1, 5, 5, 1, 1, 5, 1, 5, 5, 1, 5, 5, 5, 1, 1, 1, 4, 2, 4, 4, 1, 1, 1, 4, 2, 4, 4, 1, 4, 4, 1, 2, 4, 4, 2, 4, 1, 4, 4, 2, 1, 2, 4, 1, 2, 4, 1, 1, 4, 4, 1, 1, 1, 2, 2, 1, 2, 4, 2, 4, 2, 3, 1, 1, 3, 3, 1, 1, 3, 1, 1, 1, 3, 3, 3, 3, 1, 1, 1, 3, 1, 3, 3, 3, 3, 1, 3, 1, 1, 1, 1, 3, 3, 1, 1, 3, 1, 3, 3, 3, 3, 3, 1, 1, 1, 1, 3, 1, 1, 3, 2, 2, 1, 2, 2, 1, 2, 2, 2, 1, 1, 2, 2, 1, 2, 1, 2, 2, 1, 1, 2, 2, 2, 2, 1, 1, 2, 2, 2, 1, 1, 1, 2, 2, 2, 2, 2, 2, 1, 1, 2, 2, 1, 1, 1, 1, 1, 1, 1, 2, 2, 1, 1, 1, 1, 2, 2, 2, 2, 2, 2, 1, 1, 2, 1, 2, 2, 2, 1, 2, 1, 1, 1, 1, 1, 2, 1, 2, 2, 1, 1, 1, 2, 1, 1, 2, 1, 1, 1, 2, 1

Gene ontology terms ranked according to mean of effect sizes in cluster:
defense response to virus | innate immune response | antigen processing and presentation | adaptive immune response based on somatic recombination of immune receptors built from immunoglobulin superfamily domains | evasion or tolerance of immune response of other organism during symbiotic interaction | adaptive immune response | immune response | MHC class I biosynthetic process | MHC class I protein binding | cytokine secretion | chemokine production | T-helper 1 type immune response | response to host immune response | interleukin-12 production | interleukin-10 production | interleukin-1 receptor activity | response to lipopolysaccharide | positive regulation of NF-kappaB transcription factor activity | cell maturation | B cell activation | lymphocyte proliferation | positive regulation of tumor necrosis factor production | pathogenesis | MHC class II biosynthetic process | MHC class II protein binding | negative regulation of tumor necrosis factor production | interleukin-6 receptor activity | IkappaB kinase complex | tumor necrosis factor production | NF-kappaB binding | interferon-gamma production | monocyte differentiation | interleukin-6 production | interleukin-8 production | immunoglobulin secretion | tryptophan catabolic process | response to tumor necrosis factor | rosetting | natural killer cell receptor activity | cytokine production | interleukin-4 receptor activity | immunoglobulin production | regulation of tumor necrosis factor production | interleukin-5 production | tumor necrosis factor receptor activity | mRNA transcription | inflammatory response | interleukin-2 receptor activity | cytokine activity | tumor necrosis factor receptor binding | B cell proliferation | lymphocyte activation | T cell proliferation | monocyte activation | mucosal immune response | granulocyte macrophage colony-stimulating factor production | granuloma formation | granulocyte macrophage colony-stimulating factor biosynthetic process | macrophage activation | cytokine biosynthetic process | cytolysis | chronic inflammatory response | interleukin-1 production | interleukin-2 production | natural killer cell mediated cytotoxicity | hypersensitivity | leukocyte activation | interleukin-1 receptor antagonist activity | interleukin-4 production | tolerance induction | type IV hypersensitivity | immature T cell proliferation in the thymus | eosinophil activation | naringenin-chalcone synthase activity | interleukin-1 beta production | hyaluronic acid binding | lipopolysaccharide binding | acute inflammatory response | negative regulation of inflammatory response

Corresponding mean of effect sizes of each Gene ontology term:
(In the same order as above)
4.25, 3.42, 3.04, 2.76, 2.66, 2.61, 2.50, 2.19, 2.19, 2.14, 1.96, 1.90, 1.62, 1.54, 1.52, 1.49, 1.44, 1.41, 1.40, 1.36, 1.32, 1.28, 1.27, 1.25, 1.24, 1.20, 1.18, 1.10, 1.07, 1.05, 1.04, 1.00, 0.99, 0.99, 0.97, 0.87, 0.84, 0.83, 0.83, 0.80, 0.78, 0.74, 0.70, 0.70, 0.63, 0.61, 0.59, 0.59, 0.59, 0.58, 0.56, 0.55, 0.54, 0.54, 0.54, 0.48, 0.45, 0.45, 0.43, 0.42, 0.38, 0.36, 0.35, 0.33, 0.31, 0.24, 0.18, 0.17, 0.05, -0.05, -0.11, -0.14, -0.15, -0.19, -0.22, -0.23, -0.24, -0.38, -0.62

Corresponding total number of genes of each Gene ontology term:
(In the same order as above)
110, 246, 315, 131, 141, 148, 530, 195, 197, 243, 155, 59, 37, 150, 166, 128, 226, 550, 224, 116, 158, 61, 1.1K, 181, 178, 61, 104, 107, 209, 146, 106, 102, 165, 144, 51, 41, 185, 63, 77, 455, 87, 155, 86, 57, 213, 130, 425, 189, 42, 214, 88, 232, 188, 117, 52, 107, 77, 109, 178, 108, 179, 22, 31, 182, 33, 234, 108, 70, 123, 109, 131, 42, 71, 50, 21, 37, 61, 68, 40

Corresponding number of genes of each Gene ontology term with at least one TFBS (of selected TFs):

(In the same order as above)
50, 67, 96, 37, 44, 43, 129, 60, 60, 79, 40, 21, 14, 48, 51, 38, 65, 163, 62, 33, 42, 21, 240, 50, 49, 20, 28, 37, 54, 47, 30, 32, 47, 34, 18, 18, 53, 15, 20, 114, 25, 33, 26, 20, 60, 40, 93, 56, 12, 60, 25, 69, 55, 27, 13, 27, 16, 27, 45, 30, 34, 8, 6, 58, 10, 49, 25, 16, 34, 29, 36, 12, 12, 11, 4, 6, 9, 8, 9

Corresponding total number of TFBS (of selected TFs) for each Gene ontology term (in all genes):
(In the same order as above)
119, 150, 202, 70, 95, 81, 258, 133, 133, 140, 79, 38, 25, 84, 87, 67, 130, 300, 126, 65, 81, 39, 444, 92, 91, 38, 53, 68, 99, 85, 45, 46, 84, 67, 28, 29, 99, 31, 31, 198, 50, 57, 44, 32, 105, 74, 157, 92, 23, 105, 44, 120, 82, 39, 20, 43, 26, 43, 75, 49, 62, 11, 10, 98, 14, 74, 31, 27, 47, 40, 50, 16, 17, 13, 5, 10, 13, 14, 9

Corresponding number of selected TFs each Gene ontology term is involved with:
(In the same order as above)
6, 6, 6, 6, 6, 6, 6, 6, 6, 6, 6, 6, 5, 6, 6, 6, 6, 6, 6, 6, 6, 6, 6, 6, 6, 6, 6, 6, 6, 6, 6, 6, 6, 6, 6, 6, 6, 6, 6, 6, 6, 6, 6, 6, 6, 6, 6, 6, 6, 6, 6, 6, 6, 6, 6, 6, 6, 6, 5, 6, 6, 4, 6, 6, 6, 6, 6, 6, 5, 6, 6, 4, 5, 6, 6, 6

TFs ranked according to mean of effect sizes in cluster:
VIRF_Q6_01, VIRF_Q6, V$IRF7_01, V$IRF1_01, V$ICSBP_Q6, V$ISRE_01

Corresponding mean of effect sizes of each TF:
(In the same order as above)
1.95, 1.60, 1.18, 0.86, 0.38, -0.20

Corresponding total number of TFBS for each TF (genome-wide):
(In the same order as above)
690, 707, 680, 648, 683, 701

Corresponding total number of TFBS for each TF (in all genes in selected Gene ontology terms):
(In the same order as above)
1254, 1240, 1018, 891, 873, 695

Corresponding number of genes (of selected Gene ontology terms) each TF is involved with:
(In the same order as above)
202, 202, 164, 151, 187, 171

Corresponding number of selected Gene ontology terms each TF is involved with:
(In the same order as above)
79, 79, 79, 78, 76, 75

Cluster for columns 951 to 966, rows 139 to 141

Gene ontology terms

relaxation of vascular smooth muscle | GMP biosynthetic process | cGMP biosynthetic process | cyclase activity | guanylate cyclase activity | 11-beta-hydroxysteroid dehydrogenase activity | cortisol secretion | immunoglobulin binding | regulation of blood pressure | vasopressin secretion | penile erection | vasoconstriction | vasodilation | glomerular filtration | diuresis | natriuresis

TFs

V$SMAD4_Q6 | F$STRE_01 | V$SREBP1_02

Information

All related TFs:
(List of all TFs that are related to any of the PWMs)
SREBP-1, SREBP-1a, SREBP-1b, SREBP-1c, Smad4

Ranked gene list:
(All genes of the selected Gene ontology terms with hits of any of the selected TFs, ranked according to the total number of TFBS)
S100A6, INS, PER1, GUCA2A, NPPC, NOS3, CNP, AVP, ISYNA1, ADM, FGFR3, ERAF, TNF, COMT, CCHCR1, TPM2, MTHFR, HGS, GUCA2B, BDKRB2, VASP, PTGDS, OSM, FLJ32987, EMD, CALM3, CALM1, BGN, TNFSF13, SCNN1G, RHOA, PGF, PAF1, NR3C2, HSPA9, F2, AKT1, ZNF395, PTGS1, P2RY2, KLK1, GNAI2, CYP11A1, CEBPZ, CALR, ADORA2A, ADK, UCN, UBTF, SRC, SLC5A2, S1PR2, RYR1, RENBP, PTPRCAP, PTGER1, PROP1, PNMT, PIGR, PDE4A, MT2A, MBTPS1, LRSAM1, LLGL2, KCNS3, KCNN3, KCNA5, KCNA2, IGF2, HTR2A, GUK1, GEM, GAD1, FLNA, EGR1, ECE1, DPEP1, CYBA, COL1A1, CLCNKB, CILP2, CCL27, CBX1, ATP2A3, ALOX5, ADRBK1, ADD1

Corresponding total number of TFBS:
(For each gene listed above, the total number of TFBS for any of the selected TFs, multiplied by the number of selected Gene ontology terms containing that gene)
20, 16, 15, 12, 11, 9, 9, 9, 8, 8, 7, 7, 6, 6, 6, 5, 5, 5, 5, 5, 4, 4, 4, 4, 4, 4, 4, 4, 3, 3, 3, 3, 3, 3, 3, 3, 3, 2, 2, 2, 2, 2, 2, 2, 2, 2, 2, 1, 1, 1, 1, 1, 1, 1, 1, 1, 1, 1, 1, 1, 1, 1, 1, 1, 1, 1, 1, 1, 1, 1, 1, 1, 1, 1, 1, 1, 1, 1, 1, 1, 1, 1, 1, 1, 1, 1, 1

Corresponding number of selected Gene ontology terms each gene is involved with:
(In the same order as above)
10, 8, 5, 6, 11, 9, 9, 9, 8, 8, 7, 7, 2, 2, 6, 5, 5, 5, 5, 5, 2, 2, 4, 2, 4, 4, 4, 2, 3, 3, 3, 3, 3, 3, 3, 3, 3, 1, 2, 2, 2, 2, 1, 2, 2, 1, 1, 2, 1, 1, 1, 1, 1, 1, 1, 1, 1, 1, 1, 1, 1, 1, 1, 1, 1, 1, 1, 1, 1, 1, 1, 1, 1, 1, 1, 1, 1, 1, 1, 1, 1, 1, 1, 1, 1, 1, 1

Corresponding number of selected TFs each gene is involved with:
(In the same order as above)
2, 2, 3, 2, 1, 1, 1, 1, 1, 1, 1, 1, 3, 3, 1, 1, 1, 1, 1, 1, 2, 2, 1, 2, 1, 1, 1, 2, 1, 1, 1, 1, 1, 1, 1, 1, 3, 1, 1, 1, 1, 2, 1, 1, 2, 2, 1, 1, 1, 1, 1, 1, 1, 1, 1, 1, 1, 1, 1, 1, 1, 1, 1, 1, 1, 1, 1, 1, 1, 1, 1, 1, 1, 1, 1, 1, 1, 1, 1, 1, 1, 1, 1, 1, 1, 1, 1

Gene ontology terms ranked according to mean of effect sizes in cluster:
natriuresis | vasoconstriction | vasodilation | guanylate cyclase activity | GMP biosynthetic process | diuresis | cyclase activity | cGMP biosynthetic process | relaxation of vascular smooth muscle | regulation of blood pressure | glomerular filtration | 11-beta-hydroxysteroid dehydrogenase activity | immunoglobulin binding | cortisol secretion | vasopressin secretion | penile erection

Corresponding mean of effect sizes of each Gene ontology term:
(In the same order as above)
2.27, 2.08, 1.83, 1.67, 1.59, 1.53, 1.48, 1.29, 1.20, 1.11, 0.99, 0.64, 0.45, 0.30, -0.06, -0.10

Corresponding total number of genes of each Gene ontology term:
(In the same order as above)
83, 171, 213, 46, 30, 72, 53, 70, 50, 99, 145, 73, 23, 68, 26, 32

Corresponding number of genes of each Gene ontology term with at least one TFBS (of selected TFs):
(In the same order as above)
19, 36, 39, 12, 9, 14, 11, 15, 11, 19, 20, 12, 3, 10, 3, 5

Corresponding total number of TFBS (of selected TFs) for each Gene ontology term (in all genes):
(In the same order as above)
26, 42, 52, 13, 9, 17, 12, 18, 11, 23, 25, 16, 5, 15, 4, 5

Corresponding number of selected TFs each Gene ontology term is involved with:
(In the same order as above)
3, 3, 3, 3, 3, 3, 3, 3, 3, 3, 3, 3, 3, 3, 3, 3

TFs ranked according to mean of effect sizes in cluster:
F$STRE_01, V$SMAD4_Q6, V$SREBP1_02

Corresponding mean of effect sizes of each TF:
(In the same order as above)
1.39, 1.05, 0.98

Corresponding total number of TFBS for each TF (genome-wide):
(In the same order as above)
651, 666, 693

Corresponding total number of TFBS for each TF (in all genes in selected Gene ontology terms):
(In the same order as above)
98, 94, 101

Corresponding number of genes (of selected Gene ontology terms) each TF is involved with:
(In the same order as above)
33, 34, 38

Corresponding number of selected Gene ontology terms each TF is involved with:
(In the same order as above)
16, 16, 16

Cluster for columns 2 to 4, rows 267 to 277

Gene ontology terms

Hedgehog signaling complex | hemopoiesis | cell differentiation

TFs

V$ETF_Q6 | V$E2F_Q2 | V$E2F1_Q6 | V$E2F1_Q3 | V$AP2GAMMA_01 | V$AP2ALPHA_01 | V$AP2_Q6_01 | V$AP2_Q6 | V$WT1_Q6 | V$EGR_Q6 | V$MAZ_Q6

Information

All related TFs:
(List of all TFs that are related to any of the PWMs)
AP-2, AP-2alpha, AP-2alphaA, AP-2alphaB, AP-2beta, AP-2gamma, DP-1, E2F, E2F+E4, E2F-1, E2F-3a, E2F-4, ETF, Egr-1, Egr-2, Egr-3, MAZ, WT1, WT1-del2

Ranked gene list:
(All genes of the selected Gene ontology terms with hits of any of the selected TFs, ranked according to the total number of TFBS)
SSSCA1, NOTCH1, GATA2, FASN, HOXA9, GFI1, LBX1, ZFPM1, SH2B3, AKT1, HOXA10, SOCS3,

SMAD7, ID2, HES1, CCND1, BMI1, PDGFA, HBA2, FLII, IL11, TGFB1, TCF3, SOCS2, RBPJ, PTPN6, MAFK, IRF1, EGR1, ATXN1, VEGFA, RUNX3, JUND, CREBBP, CDKN1B, TCF7, STRA13, PTMA, PLEKHG2, PAX6, NKX2-2, NFATC1, MDK, MAPK12, MAFG, JUNB, JARID2, IRS1, IGF1R, HRAS, HOXC9, GATA3, DNMT3A, CRIP2, CRIP1, CFL1, CDKN1C, CDK5, PSMA7, NR3C2, MAFA, LEF1, HES5, FGF9, DLL1, CISH, CDKN2C, CD68, WNT10B, TNFRSF4, SOS1, NR4A2, LDB1, FLJ32987, CYP26B1, ACTB, UBE2I, RING1, RARA, PAX5, LYL1, HOXA7, FLI1, CCND2, CBFA2T3, SP3, RORC, PHF13, MLL, ING1, FOSL2, E2F4, DLL4, BCL6, YY1, WNT7B, TCIRG1, SOCS1, SHC1, RARG, PRKCE, POU3F2, PAFAH1B1, NGFR, MYB, MLL5, MAP3K12, LMO4, HMGB1, GGNBP2, FOXO1, EGFL7, CUL4A, BACH2, TRIM28, TRAF3, SETD1A, RRBP1, RPS6KB2, LAG3, HOXB6, CDK6, RUNX2, NOL3, LHX3, ITGA5, IL17C, IGF2, IDUA, HSP90AB1, HHEX, FSTL3, CREB1, CORO1A, CLEC11A, CDK4, CD81, ZBTB16, WNT4, UBL4A, TNFSF13, STAT5A, SRC, PRKCZ, PKNOX1, PCNA, PCGF2, PARD6A, MCAM, IRF2, ID3, HOXB4, HOXA11, HES6, EZH2, CDKN1A, CDK5R1, ARHGEF1, AMH, ZNF385A, TSPAN32, TNFRSF13C, TFRC, TFAP2A, TCF12, STMN1, SMAD3, SLC20A1, SDC3, S1PR2, RHOA, PPARA, PIM1, OSM, NDRG2, MYST3, MUC1, LEPREL2, IRF6, IRF4, IKZF1, HOXC6, HOXC5, HOXC4, HMBS, H3F3A, GLI1, GADD45B, GAB2, EP300, EOMES, E2F2, CRABP2, CEBPB, CDKN2D, CBFB, C2orf28, C21orf33, BHLHB2, ACVR1, VIM, TMEM201, SMARCA4, SMAD2, SFTPC, ROBLD3, RBL1, PRKCA, PDGFRA, PBX2, PAX2, OTX1, ONECUT1, ODC1, NFIA, NEUROG2, MAPK7, LHX9, LEPR, IL17D, IL11RA, HOXA1, HIST1H4I, FOS, FBXW7, ENO2, CDK2, CCNY

Corresponding total number of TFBS:
(For each gene listed above, the total number of TFBS for any of the selected TFs, multiplied by the number of selected Gene ontology terms containing that gene)
33, 33, 33, 33, 30, 30, 27, 22, 22, 22, 21, 20, 20, 20, 20, 20, 20, 16, 16, 16, 15, 14, 14, 14, 14, 14, 14, 14, 14, 14, 12, 12, 12, 12, 12, 11, 11, 11, 11, 11, 11, 11, 11, 11, 11, 11, 11, 11, 11, 11, 11, 11, 11, 11, 11, 11, 11, 10, 10, 10, 10, 10, 10, 10, 10, 10, 10, 9, 9, 9, 9, 9, 9, 9, 9, 8, 8, 8, 8, 8, 8, 8, 8, 8, 7, 7, 7, 7, 7, 7, 7, 7, 7, 6, 6, 6, 6, 6, 6, 6, 6, 6, 6, 6, 6, 6, 6, 6, 6, 6, 6, 6, 5, 5, 5, 5, 5, 5, 5, 5, 4, 4, 4, 4, 4, 4, 4, 4, 4, 4, 4, 4, 4, 4, 4, 3, 3, 3, 3, 3, 3, 3, 3, 3, 3, 3, 3, 3, 3, 3, 3, 3, 3, 3, 3, 3, 3, 3, 2, 2, 2, 2, 2, 2, 2, 2, 2, 2, 2, 2, 2, 2, 2, 2, 2, 2, 2, 2, 2, 2, 2, 2, 2, 2, 2, 2, 2, 2, 2, 2, 2, 2, 2, 2, 2, 2, 2, 2, 2, 2, 1, 1, 1, 1, 1, 1, 1, 1, 1, 1, 1, 1, 1, 1, 1, 1, 1, 1, 1, 1, 1, 1, 1, 1, 1, 1, 1, 1

Corresponding number of selected Gene ontology terms each gene is involved with:
(In the same order as above)
3, 3, 3, 3, 3, 3, 3, 2, 2, 2, 3, 2, 2, 2, 2, 2, 2, 2, 2, 2, 3, 2, 2, 2, 2, 2, 2, 2, 2, 2, 3, 2, 2, 2, 3, 1, 1, 1, 1, 1, 1, 1, 1, 1, 1, 1, 1, 1, 1, 1, 1, 1, 1, 1, 1, 1, 1, 1, 1, 1, 1, 2, 1, 1, 2, 1, 1, 1, 2, 2, 2, 1, 1, 3, 1, 1, 1, 1, 1, 1, 1, 2, 2, 1, 1, 2, 1, 2, 1, 1, 1, 1, 1, 1, 1, 1, 1, 1, 1, 1, 2, 2, 1, 1, 1, 2, 2, 2, 2, 1, 2, 1, 1, 1, 1, 1, 1, 1, 1, 1, 1, 1, 1, 1, 1, 1, 1, 2, 2, 1, 2, 1, 1, 1, 1, 1, 1, 2, 2, 2, 1, 1, 1, 1, 1, 3, 3, 3, 1, 1, 1, 1, 1, 1, 1, 1, 3, 1, 1, 1, 3, 1, 1, 1, 1, 1, 1, 2, 1, 2, 1, 2, 1, 2, 1, 2, 2, 1, 2, 1, 1, 1, 1, 2, 1, 2, 1, 1, 1, 2, 1, 1, 1, 1, 2, 1, 1, 1, 2, 2, 1, 2, 2, 1, 1, 1, 1, 1, 1, 1, 1, 1, 1, 1, 1, 1, 1, 1, 1, 1, 1, 1, 1, 1, 1, 1, 1, 1, 1, 1, 1, 1, 1

Corresponding number of selected TFs each gene is involved with:
(In the same order as above)
11, 11, 11, 11, 10, 10, 9, 11, 11, 11, 7, 10, 10, 10, 10, 10, 10, 8, 8, 8, 5, 7, 7, 7, 7, 7, 7, 7, 7, 7, 4, 6, 6, 6, 4, 11, 11, 11, 11, 11, 11, 11, 11, 11, 11, 11, 11, 11, 11, 11, 11, 11, 11, 11, 11, 11, 5, 10, 10, 5, 10, 10, 10, 5, 5, 5, 9, 9, 3, 9, 9, 9, 9, 9, 8, 8, 4, 4, 8, 8, 4, 8, 4, 7, 7, 7, 7, 7, 7, 7, 7, 7, 6, 6, 6, 3, 3, 6, 6, 6, 3, 3, 3, 3, 6, 3, 6, 6, 6, 6, 6, 6, 5, 5, 5, 5, 5, 5, 5, 5, 2, 2, 4, 2, 4, 4, 4, 4, 4, 4, 2, 2, 2, 4, 4, 3, 3, 3, 1, 1, 1, 3, 3, 3, 3, 3, 3, 3, 3, 1, 3, 3, 3, 1, 3, 3, 3, 2, 2, 2, 1, 2, 1, 2, 1, 2, 1, 2, 1, 1, 2, 1, 2, 2, 2, 2, 1, 2, 1, 2, 2, 2, 1, 2, 2, 2, 2, 1, 2, 2, 2, 1, 1, 2, 1, 1, 2, 2, 1, 1, 1, 1, 1, 1, 1, 1, 1, 1, 1, 1, 1, 1, 1, 1, 1, 1, 1, 1, 1, 1, 1, 1, 1, 1

Gene ontology terms ranked according to mean of effect sizes in cluster:
cell differentiation | hemopoiesis | Hedgehog signaling complex

Corresponding mean of effect sizes of each Gene ontology term:
(In the same order as above)
2.14, 1.58, 1.40

Corresponding total number of genes of each Gene ontology term:
(In the same order as above)
871, 343, 230

Corresponding number of genes of each Gene ontology term with at least one TFBS (of selected TFs):
(In the same order as above)
175, 94, 54

Corresponding total number of TFBS (of selected TFs) for each Gene ontology term (in all genes):
(In the same order as above)
919, 431, 281

Corresponding number of selected TFs each Gene ontology term is involved with:
(In the same order as above)
11, 11, 11

TFs ranked according to mean of effect sizes in cluster:
VETF_Q6, VAP2ALPHA_01, V$E2F1_Q6, V$E2F1_Q3, V$AP2GAMMA_01, V$E2F_Q2, VMAZ_Q6, VAP2_Q6, V$WT1_Q6, V$EGR_Q6, V$AP2_Q6_01

Corresponding mean of effect sizes of each TF:
(In the same order as above)
2.23, 2.23, 2.14, 1.89, 1.85, 1.83, 1.82, 1.62, 1.51, 0.87, 0.78

Corresponding total number of TFBS for each TF (genome-wide):
(In the same order as above)
699, 686, 691, 709, 695, 714, 734, 688, 729, 695, 690

Corresponding total number of TFBS for each TF (in all genes in selected Gene ontology terms):
(In the same order as above)
157, 148, 162, 146, 143, 156, 148, 149, 156, 134, 132

Corresponding number of genes (of selected Gene ontology terms) each TF is involved with:
(In the same order as above)
107, 102, 112, 103, 98, 110, 104, 105, 107, 97, 97

Corresponding number of selected Gene ontology terms each TF is involved with:
(In the same order as above)
3, 3, 3, 3, 3, 3, 3, 3, 3, 3, 3

Cluster for columns 1053 to 1071, rows 161 to 167

Gene ontology terms

tectum | axon guidance | nervous system development | synaptic cleft | terminal button | cognition | toxin binding | choline O-acetyltransferase activity | neurotransmitter biosynthetic process | basal lamina | sarcoplasm | response to ATP | response to histamine | serotonin receptor activity | interchromatin granule | acetylcholinesterase activity | choline transport | angiotensin-converting enzyme inhibitor activity | saliva secretion

TFs

V$USF2_Q6 | V$ZIC2_01 | V$TTF1_Q6 | V$VDR_Q6 | V$LRF_Q2 | V$LBP1_Q6 | V$AP4_Q6_01

Information

All related TFs:
(List of all TFs that are related to any of the PWMs)
AP-4, FBI-1, LBP-1, LRF, Nkx2-1, OCZF, USF1:USF2, USF2, USF2a, VDR, ZIC2, Zic2

Ranked gene list:
(All genes of the selected Gene ontology terms with hits of any of the selected TFs, ranked according to the total number of TFBS)
FGFR3, ACHE, AGRN, AKT1, THRA, SEMA3B, PPP1R9B, PLXNA1, PER1, GPT, FLJ32987, EFNA3, CDK5, SLC25A1, PAX6, DPAGT1, LBX1, VEGFA, PNPLA6, ENTPD2, EFNA2, WNT1, SLC4A2, SLC2A4RG, SCRIB, RARA, PPP1R1B, PLEC1, NTN3, NOTCH1, NOC2L, NKX2-2, MDK, GNAI2, GFI1, FGF8, DVL1, ACTG1, WBP7, S100A6, PTDSS2, PNMT, PGP, PDE4A, MGAT1, KCNH2, EGR1, ARHGEF2, TNFSF13, SH2B2, PSD, P2RY11, JUND, DBP, CDT1, CDH15, PARD6A, NGFR, EPHB3, EPB49, EFNA1, AQP3, VASP, NR3C2, NDOR1, LAMA5, HES1, GPR132, FBXL15, ENO3, DDR1, POU2F2, NFIX, MICAL1, LIF, HRH3, HBA2, GPC1, GALR3, GALNS, FERMT3, DLG4, BRD1, TFAP2A, SEMA7A, SEMA3G, PYGM, PAX5, NOTCH4, MUC1, MAP1A, KCNA2, INS, IGF2, GRM2, GRIN1, FXYD1, AQP5, AMD1

Corresponding total number of TFBS:
(For each gene listed above, the total number of TFBS for any of the selected TFs, multiplied by the number of selected Gene ontology terms containing that gene)
98, 91, 25, 21, 14, 14, 14, 14, 14, 14, 14, 14, 14, 12, 12, 10, 9, 8, 8, 8, 8, 7, 7, 7, 7, 7, 7, 7, 7, 7, 7, 7, 7, 7, 7, 7, 7, 7, 6, 6, 6, 6, 6, 6, 6, 6, 6, 6, 5, 5, 5, 5, 5, 5, 5, 5, 4, 4, 4, 4, 4, 4, 3, 3, 3, 3, 3, 3, 3, 3, 3, 2, 2, 2, 2, 2, 2, 2, 2, 2, 2, 2, 2, 1, 1, 1, 1, 1, 1, 1, 1, 1, 1, 1, 1, 1, 1, 1

Corresponding number of selected Gene ontology terms each gene is involved with:
(In the same order as above)
14, 13, 5, 3, 2, 2, 2, 2, 2, 2, 2, 2, 2, 2, 3, 2, 3, 2, 2, 2, 2, 1, 1, 1, 1, 1, 1, 1, 1, 1, 1, 1, 1, 1, 1, 1, 1, 1, 1, 1, 2, 1, 1, 1, 1, 1, 1, 5, 1, 1, 1, 1, 1, 1, 1, 1, 4, 2, 1, 1, 1, 1, 1, 1, 1, 1, 1, 1, 1, 1, 1, 1, 2, 2, 2, 1, 1, 1, 2, 1, 1, 2, 1, 1, 1, 1, 1, 1, 1, 1, 1, 1, 1, 1, 1, 1, 1, 1

Corresponding number of selected TFs each gene is involved with:
(In the same order as above)
7, 7, 5, 7, 7, 7, 7, 7, 7, 7, 7, 7, 7, 6, 4, 5, 3, 4, 4, 4, 4, 7, 7, 7, 7, 7, 7, 7, 7, 7, 7, 7, 7, 7, 7, 7, 7, 7, 6, 6, 6, 3, 6, 6, 6, 6, 6, 6, 1, 5, 5, 5, 5, 5, 5, 5, 4, 1, 2, 4, 4, 4, 3, 3, 3, 3, 3, 3, 3, 3, 3, 2, 2, 1, 1, 1, 2, 2, 2, 1, 2, 2, 1, 1, 1, 1, 1, 1, 1, 1, 1, 1, 1, 1, 1, 1, 1, 1

Gene ontology terms ranked according to mean of effect sizes in cluster:
saliva secretion | acetylcholinesterase activity | neurotransmitter biosynthetic process | choline transport | angiotensin-converting enzyme inhibitor activity | toxin binding | terminal button | synaptic cleft | interchromatin granule | sarcoplasm | choline O-acetyltransferase activity | cognition | tectum | serotonin receptor activity | axon guidance | response to ATP | nervous system development | basal lamina | response to histamine

Corresponding mean of effect sizes of each Gene ontology term:
(In the same order as above)
2.40, 2.08, 1.89, 1.72, 1.63, 1.52, 1.51, 1.49, 1.46, 1.40, 1.26, 1.19, 1.16, 1.13, 1.05, 1.03, 1.02, 0.91, 0.85

Corresponding total number of genes of each Gene ontology term:
(In the same order as above)
27, 27, 27, 26, 63, 33, 21, 66, 35, 24, 37, 69, 116, 49, 187, 47, 185, 112, 37

Corresponding number of genes of each Gene ontology term with at least one TFBS (of selected TFs):
(In the same order as above)

7, 6, 5, 4, 11, 5, 2, 8, 5, 5, 6, 7, 15, 6, 26, 4, 26, 11, 4

Corresponding total number of TFBS (of selected TFs) for each Gene ontology term (in all genes):
(In the same order as above)
38, 29, 27, 22, 49, 22, 14, 38, 27, 23, 20, 37, 70, 27, 103, 22, 112, 50, 16

Corresponding number of selected TFs each Gene ontology term is involved with:
(In the same order as above)
7, 7, 7, 7, 7, 7, 7, 7, 7, 7, 7, 7, 7, 7, 7, 7, 7, 7, 7

TFs ranked according to mean of effect sizes in cluster:
VVDR_Q6, VLRF_Q2, V$AP4_Q6_01, V$LBP1_Q6, V$TTF1_Q6, V$ZIC2_01, V$USF2_Q6

Corresponding mean of effect sizes of each TF:
(In the same order as above)
1.60, 1.52, 1.51, 1.46, 1.35, 1.26, 1.13

Corresponding total number of TFBS for each TF (genome-wide):
(In the same order as above)
692, 719, 699, 691, 714, 716, 703

Corresponding total number of TFBS for each TF (in all genes in selected Gene ontology terms):
(In the same order as above)
109, 115, 109, 109, 99, 103, 102

Corresponding number of genes (of selected Gene ontology terms) each TF is involved with:
(In the same order as above)
58, 65, 62, 61, 57, 57, 56

Corresponding number of selected Gene ontology terms each TF is involved with:
(In the same order as above)
19, 19, 19, 19, 19, 19, 19

Cluster for columns 138 to 140, rows 241 to 243

Gene ontology terms

hemoglobin biosynthetic process | heme biosynthetic process | heme metabolic process

TFs

V$LMO2COM_02 | V$GATA1_04 | V$GATA1_02

Information

All related TFs:
(List of all TFs that are related to any of the PWMs)
GATA-1, GATA-1A, Lmo2

Ranked gene list:
(All genes of the selected Gene ontology terms with hits of any of the selected TFs, ranked according to the total number of TFBS)
HMBS, PPOX, HFE, ZFPM1, DCTN3, BLVRA, ALAD, AHSA1, UROD, RAB8A, NFE2, HBA2,

GATA1, MYB, KLF1, HMOX2, GCLC, EPOR, DLX3

Corresponding total number of TFBS:
(For each gene listed above, the total number of TFBS for any of the selected TFs, multiplied by the number of selected Gene ontology terms containing that gene)
9, 6, 6, 3, 3, 3, 3, 3, 2, 2, 2, 2, 2, 1, 1, 1, 1, 1, 1

Corresponding number of selected Gene ontology terms each gene is involved with:
(In the same order as above)
3, 2, 2, 1, 1, 1, 3, 1, 2, 2, 2, 2, 2, 1, 1, 1, 1, 1, 1

Corresponding number of selected TFs each gene is involved with:
(In the same order as above)
3, 3, 3, 3, 3, 3, 1, 3, 1, 1, 1, 1, 1, 1, 1, 1, 1, 1, 1

Gene ontology terms ranked according to mean of effect sizes in cluster:
hemoglobin biosynthetic process | heme metabolic process | heme biosynthetic process

Corresponding mean of effect sizes of each Gene ontology term:
(In the same order as above)
4.56, 3.87, 2.07

Corresponding total number of genes of each Gene ontology term:
(In the same order as above)
37, 22, 73

Corresponding number of genes of each Gene ontology term with at least one TFBS (of selected TFs):
(In the same order as above)
12, 7, 11

Corresponding total number of TFBS (of selected TFs) for each Gene ontology term (in all genes):
(In the same order as above)
20, 13, 19

Corresponding number of selected TFs each Gene ontology term is involved with:
(In the same order as above)
3, 3, 3

TFs ranked according to mean of effect sizes in cluster:
V$LMO2COM_02, V$GATA1_04, V$GATA1_02

Corresponding mean of effect sizes of each TF:
(In the same order as above)
3.74, 3.39, 3.37

Corresponding total number of TFBS for each TF (genome-wide):
(In the same order as above)
687, 698, 694

Corresponding total number of TFBS for each TF (in all genes in selected Gene ontology terms):
(In the same order as above)
18, 18, 16

Corresponding number of genes (of selected Gene ontology terms) each TF is involved with:
(In the same order as above)

12, 11, 10

Corresponding number of selected Gene ontology terms each TF is involved with:
(In the same order as above)
3, 3, 3

Cluster for columns 1169 to 1172, rows 122 to 127

Gene ontology terms

odontogenesis | transdifferentiation | kidney development | mesoderm formation

TFs

V$HNF3_Q6 | V$HNF3ALPHA_Q6 | V$HNF3_Q6_01 | V$HFH3_01 | V$FOX_Q2 | V$FOXD3_01

Information

All related TFs:
(List of all TFs that are related to any of the PWMs)
FOXD3, FOXF1, FOXF2, FOXH2, FOXI1, FOXJ1, FOXJ1a, FOXJ1b, HFH-3, HNF-3, HNF-3alpha, HNF-3beta, HNF-3gamma, HNF3(-like)

Ranked gene list:
(All genes of the selected Gene ontology terms with hits of any of the selected TFs, ranked according to the total number of TFBS)
FST, FGF8, LEF1, PCNA, CTNNB1, ACVR1, RUNX2, SMAD4, SMAD3, SMAD1, PIK3CA, OTX2, MAF, ID2, CITED2, TLE4, HOXA11, GRB2, GAS1, FOXD1, ATM, VIM, TGFB1, TCF7L2, SPRY1, SMAD7, SIX1, SHH, PROX1, LBX1, KIF2A, HAND2, FOXG1, FOXC2, CASP3, BMP4, VEZF1, STX3, SSSCA1, PTPRC, PRPH, POU2F1, NFAT5, NEUROD1, MAPK1, EYA1, CXCR4, TJP1, TGFB3, STAT3, SOS1, SLC33A1, PTHLH, PIK3C2A, ONECUT1, NKX2-5, NCAM1, LHX1, ITK, IL6ST, HOXB7, GSC, GLP-1, FOXL2, EOMES, EDAR, CD44, BMPR2, WNT10A, TRAF3, TPM3, TGFBR2, SRF, SLIT2, SFRP2, SERPINE1, PTCH1, PITX3, PAX9, PAX6, OSR1, NRTN, NR1H3, NKX2-2, NEUROG3, LSM4, LRPPRC, KIAA0319, KAT2B, HS2ST1, HES1, GLI1, GFRA1, GDF11, FOXD3, FOS, DMTF1, CSRP2, CORO7, CDX2, CD68, AHR, ADFP

Corresponding total number of TFBS:
(For each gene listed above, the total number of TFBS for any of the selected TFs, multiplied by the number of selected Gene ontology terms containing that gene)
20, 20, 15, 12, 12, 12, 10, 6, 6, 6, 6, 6, 6, 6, 6, 5, 5, 5, 5, 5, 5, 4, 4, 4, 4, 4, 4, 4, 4, 4, 4, 4, 4, 4, 4, 4, 3, 3, 3, 3, 3, 3, 3, 3, 3, 3, 3, 2, 2, 2, 2, 2, 2, 2, 2, 2, 2, 2, 2, 2, 2, 2, 2, 2, 2, 2, 2, 1, 1, 1, 1, 1, 1, 1, 1, 1, 1, 1, 1, 1, 1, 1, 1, 1, 1, 1, 1, 1, 1, 1, 1, 1, 1, 1, 1, 1, 1, 1, 1, 1, 1, 1

Corresponding number of selected Gene ontology terms each gene is involved with:
(In the same order as above)
4, 4, 3, 2, 4, 3, 2, 2, 2, 3, 1, 1, 1, 1, 1, 1, 1, 1, 1, 1, 1, 2, 4, 1, 1, 1, 1, 4, 1, 4, 1, 1, 1, 1, 1, 4, 1, 1, 1, 1, 1, 1, 1, 1, 1, 1, 1, 1, 1, 1, 2, 2, 2, 1, 1, 1, 2, 1, 2, 1, 1, 1, 1, 1, 1, 1, 1, 2, 2, 1, 1, 1, 1, 1, 1, 1, 1, 1, 1, 1, 1, 1, 1, 1, 1, 1, 1, 1, 1, 1, 1, 1, 1, 1, 1, 1, 1, 1, 1, 1, 1, 1

Corresponding number of selected TFs each gene is involved with:
(In the same order as above)
5, 5, 5, 6, 3, 4, 5, 3, 3, 2, 6, 6, 6, 6, 6, 5, 5, 5, 5, 5, 5, 2, 1, 4, 4, 4, 4, 1, 4, 1, 4, 4, 4, 4, 4, 1, 3, 3, 3, 3, 3, 3, 3, 3, 3, 3, 3, 2, 2, 2, 1, 1, 1, 2, 2, 1, 2, 1, 2, 2, 2, 2, 2, 2, 2, 2, 2, 1, 1, 1, 1, 1, 1, 1, 1, 1, 1, 1, 1, 1, 1, 1, 1, 1, 1, 1,

1, 1, 1, 1, 1, 1, 1, 1, 1, 1, 1, 1, 1, 1, 1, 1, 1, 1, 1

Gene ontology terms ranked according to mean of effect sizes in cluster:
transdifferentiation | kidney development | mesoderm formation | odontogenesis

Corresponding mean of effect sizes of each Gene ontology term:
(In the same order as above)
2.18, 2.18, 1.38, 0.56

Corresponding total number of genes of each Gene ontology term:
(In the same order as above)
204, 139, 66, 122

Corresponding number of genes of each Gene ontology term with at least one TFBS (of selected TFs):
(In the same order as above)
56, 37, 22, 27

Corresponding total number of TFBS (of selected TFs) for each Gene ontology term (in all genes):
(In the same order as above)
143, 94, 49, 63

Corresponding number of selected TFs each Gene ontology term is involved with:
(In the same order as above)
6, 6, 6, 6

TFs ranked according to mean of effect sizes in cluster:
V$HNF3_Q6_01, V$HNF3ALPHA_Q6, V$HFH3_01, V$FOXD3_01, V$HNF3_Q6, V$FOX_Q2

Corresponding mean of effect sizes of each TF:
(In the same order as above)
1.89, 1.86, 1.79, 1.35, 1.34, 1.23

Corresponding total number of TFBS for each TF (genome-wide):
(In the same order as above)
676, 706, 689, 686, 707, 681

Corresponding total number of TFBS for each TF (in all genes in selected Gene ontology terms):
(In the same order as above)
59, 62, 60, 57, 56, 55

Corresponding number of genes (of selected Gene ontology terms) each TF is involved with:
(In the same order as above)
42, 45, 45, 39, 41, 38

Corresponding number of selected Gene ontology terms each TF is involved with:
(In the same order as above)
4, 4, 4, 4, 4, 4

Cluster for columns 177 to 185, rows 154 to 167

Gene ontology terms

exocytosis | synaptic transmission | postsynaptic membrane | receptor clustering | postsynaptic density | N-methyl-D-aspartate selective glutamate receptor activity | dendritic spine | receptor activity | synaptic

transmission, glutamatergic

TFs

V$SMAD_Q6 | V$AP2REP_01 | V$HNF4_Q6_03 | V$HNF4_Q6_02 | V$T3R_Q6 | V$PAX8_B | V$PAX8_01 | V$USF2_Q6 | V$ZIC2_01 | V$TTF1_Q6 | V$VDR_Q6 | V$LRF_Q2 | V$LBP1_Q6 | V$AP4_Q6_01

Information

All related TFs:
(List of all TFs that are related to any of the PWMs)
AP-2rep, AP-4, FBI-1, HNF-4, HNF-4alpha, HNF-4alpha1, HNF-4alpha2, HNF-4alpha3, HNF-4alpha4, LBP-1, LRF, Nkx2-1, OCZF, Pax-8, RAR-alpha, RAR-alpha1, RAR-alpha:RXR-alpha, RAR-alpha:RXR-gamma, RAR-beta, RAR-beta2, RAR-gamma, RXR-alpha, RXR-beta, RXR-beta2, RXR-gamma, Smad1, Smad1.1, Smad2, Smad2-L, Smad3, Smad3:Smad4, Smad4, T3R-alpha, T3R-alpha1, T3R-alpha2, T3R-beta, T3R-beta1, T3R-beta2, USF1:USF2, USF2, USF2a, VDR, ZIC2, Zic2

Ranked gene list:
(All genes of the selected Gene ontology terms with hits of any of the selected TFs, ranked according to the total number of TFBS)
GUK1, CDK5, AKT1, PPP1R9B, MGAT1, SYNGAP1, PSD, FGFR3, DLG4, CAMK2G, PER1, NDOR1, ACHE, FBXL15, SLC2A4RG, RHBDL1, GABARAP, FASN, ARHGEF2, GRIN2D, VAMP2, EGR1, REPIN1, ITGB2, GRIN2C, AGRN, SYT8, STXBP2, SLC25A22, SEPT5, RARA, OPRL1, MAP3K11, MAP2K3, JUNB, ISYNA1, GIT1, FLJ32987, EFNA3, CTSD, TAS1R3, BAIAP3, PPP1R1B, PLCG1, MC1R, ADM2, ADM, SYTL1, SQSTM1, SLC9A3R1, CAMKK1, ACD, UNC13D, ENTPD2, SYT7, RNASEK, LASP1, GRM2, GRIN1, RAB37, FHL3, CAPN10, APEH, SHANK3, KCNC3, GRN, CHRNE, ARHGDIA, TNFSF13, SLC17A7, NR3C2, DBNL, VGF, TNFRSF1B, TNFRSF1A, SLC9A3R2, ProSAPiP1, HCRT, FOSB, CACNA1G, BRD2, ARF1, ACCN4, OPRS1, NRGN, GRIN3B, CD81, ARC, ADRM1, TSC2, RAB3A, NR3C1, MUC1, KCNA2, INS, DKFZp434P0672, CPLX1, CDK5R1, VAV1, VAMP8, TFAP2A, SHANK1, RHOG, RAB25, MAP1A, IGF1R, HRH3, EPB41, CYB561, CLCN3, CHRNB1, CD63, AQP5, ACCN2

Corresponding total number of TFBS:
(For each gene listed above, the total number of TFBS for any of the selected TFs, multiplied by the number of selected Gene ontology terms containing that gene)
78, 70, 70, 65, 65, 60, 55, 54, 48, 44, 42, 40, 36, 35, 28, 28, 28, 28, 26, 24, 20, 20, 18, 18, 16, 15, 14, 14, 14, 14, 14, 14, 14, 14, 14, 14, 14, 14, 14, 14, 13, 13, 12, 12, 12, 12, 12, 11, 11, 11, 11, 11, 10, 10, 8, 8, 8, 8, 8, 7, 7, 7, 7, 6, 6, 6, 6, 6, 5, 5, 5, 5, 4, 4, 4, 4, 4, 4, 4, 4, 4, 4, 4, 3, 3, 3, 3, 3, 3, 2, 2, 2, 2, 2, 2, 2, 2, 2, 1, 1, 1, 1, 1, 1, 1, 1, 1, 1, 1, 1, 1, 1, 1, 1

Corresponding number of selected Gene ontology terms each gene is involved with:
(In the same order as above)
6, 5, 5, 5, 5, 5, 5, 6, 8, 4, 3, 8, 3, 5, 2, 2, 2, 2, 2, 4, 2, 2, 2, 2, 4, 3, 1, 1, 1, 1, 1, 1, 1, 1, 1, 1, 1, 1, 1, 1, 1, 1, 1, 1, 1, 1, 1, 1, 1, 1, 1, 1, 2, 1, 1, 1, 8, 8, 1, 1, 1, 1, 3, 1, 1, 1, 1, 5, 5, 1, 1, 1, 1, 2, 1, 1, 1, 1, 1, 1, 1, 1, 1, 3, 1, 1, 3, 1, 1, 2, 1, 1, 2, 2, 1, 2, 2, 1, 1, 1, 1, 1, 1, 1, 1, 1, 1, 1, 1, 1, 1, 1

Corresponding number of selected TFs each gene is involved with:
(In the same order as above)
13, 14, 14, 13, 13, 12, 11, 9, 6, 11, 14, 5, 12, 7, 14, 14, 14, 14, 13, 6, 10, 10, 9, 9, 4, 5, 14, 14, 14, 14, 14, 14, 14, 14, 14, 14, 14, 14, 14, 14, 13, 13, 12, 12, 12, 12, 12, 11, 11, 11, 11, 11, 10, 5, 8, 8, 8, 1, 1, 7, 7, 7, 7, 2, 6, 6, 6, 6, 1, 1, 5, 5, 4, 4, 2, 4, 4, 4, 4, 4, 4, 4, 4, 3, 1, 3, 3, 1, 3, 2, 1, 2, 2, 1, 1, 2, 1, 1, 1, 1, 1, 1, 1, 1, 1, 1, 1, 1, 1, 1, 1, 1, 1, 1

Gene ontology terms ranked according to mean of effect sizes in cluster:
postsynaptic membrane | dendritic spine | N-methyl-D-aspartate selective glutamate receptor activity | synaptic transmission | postsynaptic density | receptor clustering | exocytosis | synaptic transmission, glutamatergic | receptor activity

Corresponding mean of effect sizes of each Gene ontology term:
(In the same order as above)
1.99, 1.54, 1.44, 1.31, 1.20, 1.14, 0.97, 0.69, 0.68

Corresponding total number of genes of each Gene ontology term:
(In the same order as above)
62, 91, 208, 251, 162, 79, 381, 38, 187

Corresponding number of genes of each Gene ontology term with at least one TFBS (of selected TFs):
(In the same order as above)
17, 18, 32, 36, 27, 16, 48, 7, 24

Corresponding total number of TFBS (of selected TFs) for each Gene ontology term (in all genes):
(In the same order as above)
110, 156, 257, 245, 173, 102, 344, 40, 175

Corresponding number of selected TFs each Gene ontology term is involved with:
(In the same order as above)
14, 14, 14, 14, 14, 14, 14, 14, 14

TFs ranked according to mean of effect sizes in cluster:
V$ZIC2_01, V$AP4_Q6_01, V$USF2_Q6, V$HNF4_Q6_02, V$HNF4_Q6_03, V$LRF_Q2, VVDR_Q6, VAP2REP_01, V$TTF1_Q6, V$SMAD_Q6, V$LBP1_Q6, V$T3R_Q6, V$PAX8_01, V$PAX8_B

Corresponding mean of effect sizes of each TF:
(In the same order as above)
1.57, 1.49, 1.45, 1.42, 1.40, 1.32, 1.32, 1.25, 1.24, 1.16, 1.13, 1.01, 0.70, 0.58

Corresponding total number of TFBS for each TF (genome-wide):
(In the same order as above)
716, 699, 703, 699, 701, 719, 692, 700, 714, 729, 691, 698, 728, 712

Corresponding total number of TFBS for each TF (in all genes in selected Gene ontology terms):
(In the same order as above)
127, 116, 127, 118, 117, 121, 109, 117, 113, 114, 109, 107, 107, 100

Corresponding number of genes (of selected Gene ontology terms) each TF is involved with:
(In the same order as above)
61, 57, 60, 57, 60, 59, 55, 56, 58, 61, 57, 53, 58, 53

Corresponding number of selected Gene ontology terms each TF is involved with:
(In the same order as above)
9, 9, 9, 9, 9, 9, 9, 9, 9, 9, 9, 9, 9, 9

Cluster for columns 961 to 966, rows 247 to 261

Gene ontology terms

penile erection | vasoconstriction | vasodilation | glomerular filtration | diuresis | natriuresis

TFs

V$AML_Q6 | V$PAX_Q6 | V$NRF2_Q4 | V$MAF_Q6_01 | V$AP1_Q6_01 | V$AP1_Q4_01 | V$AP1_Q6 | V$AP1_C | V$AP1_01 | V$AP1_Q2 | V$AP1FJ_Q2 | V$AP1_Q4 | V$AP1_Q2_01 | V$BACH2_01 | V$NFE2_01

Information

All related TFs:
(List of all TFs that are related to any of the PWMs)
AML1, AML1a, AML1b, AML1c, AML2, AML3, AML3-isoform1, AML3-isoform2, AP-1, Bach1, Bach1:MafK, Bach2, FosB, Fra-1, Fra-2, JunB, JunB:Fra-1, JunB:Fra-2, JunD, JunD:Fra-2, JunD:deltaFosB, LCR-F1, MAF, Maf, MafB, MafF, MafG, MafG:MafG, MafK, NF-E2, Nrf1, Nrf1:MafG, Nrf1:MafK, Nrf2, Nrf2:MafG, Nrf2:MafK, Nrf3, Nrf3:MafK, PEBP2, PEBP2alphaA1, PEBP2alphaA2, PEBP2alphaB1, PEBP2alphaB2, Pax-1, Pax-2, Pax-2a, Pax-3, Pax-4a, Pax-4c, Pax-5, Pax-6, Pax-8, Pax6-1, RUNX2-isoform2, YAP1, c-Fos, c-Jun, c-Jun:FosB, c-Jun:JunD, c-Jun:c-Fos, c-Maf, deltaFosB, v-Maf

Ranked gene list:
(All genes of the selected Gene ontology terms with hits of any of the selected TFs, ranked according to the total number of TFBS)
ADM, HGS, S100A6, NLRP3, DDR1, ISYNA1, OSM, NOS2, ZFP91, MTHFR, HMOX1, HSPA9, GPI, TNF, LMNA, VASP, PER1, CPA6, AQP2, AKT1, ADORA2A, VGF, RHOA, PTPRCAP, INS, ENO1, EDN2, S1PR2, FLNA, FLJ32987, CNP, NQO1, NOS3, ECE1, UBTF, NPR1, MT2A, HMOX2, NR1D2, NPPC, NOS1, MME, KNG1, ADA, PTK2B, PAOX, OPA1, NUDT2, NPPB, KLF6, EMD, DCTN3, VEGFA, PTTG1IP, PCYT2, GUCA2B, AQP1, TNFRSF9, TES, TCN2, SLC35A1, SCGB1A1, S100A12, PTGER4, PTGDS, P2RX1, NFAT5, MGEA5, IRF6, IL6, DBP, CYBA, CABIN1, C1QL1, AMBP, ADRA1A, VWF, VIPR1, TRIM25, TALDO1, TAC4, SERPINE1, RENBP, RAMP2, RAMP1, PYY, PTGIR, PTGER3, MFSD11, LRSAM1, EMP1, CYGB, CILP2, BGLAP, ARSA, AKR1B1, ADRB2, ACTN4

Corresponding total number of TFBS:
(For each gene listed above, the total number of TFBS for any of the selected TFs, multiplied by the number of selected Gene ontology terms containing that gene)
78, 65, 60, 60, 60, 40, 33, 24, 16, 16, 16, 15, 14, 13, 13, 12, 12, 12, 12, 12, 12, 11, 10, 10, 10, 10, 10, 9, 9, 9, 9, 8, 8, 8, 7, 6, 6, 6, 5, 5, 5, 5, 5, 5, 4, 4, 4, 4, 4, 4, 4, 4, 3, 3, 3, 3, 3, 2, 2, 2, 2, 2, 2, 2, 2, 2, 2, 2, 2, 2, 2, 2, 2, 2, 2, 2, 1, 1, 1, 1, 1, 1, 1, 1, 1, 1, 1, 1, 1, 1, 1, 1, 1, 1, 1, 1, 1, 1

Corresponding number of selected Gene ontology terms each gene is involved with:
(In the same order as above)
6, 5, 5, 5, 5, 4, 3, 4, 2, 2, 2, 3, 2, 1, 1, 1, 4, 4, 3, 1, 1, 1, 2, 1, 5, 1, 2, 1, 1, 1, 3, 2, 4, 1, 1, 6, 1, 3, 5, 5, 5, 5, 5, 1, 1, 2, 2, 2, 4, 1, 1, 1, 1, 1, 1, 3, 3, 1, 2, 1, 2, 1, 2, 1, 2, 2, 1, 2, 2, 1, 2, 1, 2, 2, 1, 1, 1, 1, 1, 1, 1, 1, 1, 1, 1, 1, 1, 1, 1, 1, 1, 1, 1, 1, 1, 1, 1, 1

Corresponding number of selected TFs each gene is involved with:
(In the same order as above)
13, 13, 12, 12, 12, 10, 11, 6, 8, 8, 8, 5, 7, 13, 13, 12, 3, 3, 4, 12, 12, 11, 5, 10, 2, 10, 5, 9, 9, 9, 3, 4, 2, 8, 7, 1, 6, 2, 1, 1, 1, 1, 1, 5, 4, 2, 2, 2, 1, 4, 4, 4, 3, 3, 3, 1, 1, 2, 1, 2, 1, 2, 1, 2, 1, 1, 2, 1, 1, 2, 1, 2, 1, 1, 2, 2, 1, 1, 1, 1, 1, 1, 1, 1, 1, 1, 1, 1, 1, 1, 1, 1, 1, 1, 1, 1, 1, 1

Gene ontology terms ranked according to mean of effect sizes in cluster:
vasoconstriction | diuresis | vasodilation | penile erection | natriuresis | glomerular filtration

Corresponding mean of effect sizes of each Gene ontology term:
(In the same order as above)
2.03, 1.87, 1.25, 1.24, 1.05, 1.00

Corresponding total number of genes of each Gene ontology term:
(In the same order as above)
171, 72, 213, 32, 83, 145

Corresponding number of genes of each Gene ontology term with at least one TFBS (of selected TFs):
(In the same order as above)
50, 22, 57, 10, 22, 39

Corresponding total number of TFBS (of selected TFs) for each Gene ontology term (in all genes):
(In the same order as above)
227, 102, 255, 56, 102, 136

Corresponding number of selected TFs each Gene ontology term is involved with:
(In the same order as above)
15, 15, 15, 15, 15, 15

TFs ranked according to mean of effect sizes in cluster:
V$NFE2_01, V$AP1_01, V$AP1_Q6_01, V$AP1_C, V$AP1FJ_Q2, V$AP1_Q4_01, VMAF_Q6_01, VNRF2_Q4, VPAX_Q6, VAP1_Q6, V$AP1_Q4, V$AP1_Q2_01, V$AP1_Q2, V$AML_Q6, V$BACH2_01

Corresponding mean of effect sizes of each TF:
(In the same order as above)
2.60, 2.30, 1.67, 1.64, 1.61, 1.60, 1.42, 1.38, 1.26, 1.22, 1.11, 1.01, 0.85, 0.85, 0.58

Corresponding total number of TFBS for each TF (genome-wide):
(In the same order as above)
729, 721, 726, 720, 710, 719, 700, 683, 683, 721, 712, 714, 692, 680, 686

Corresponding total number of TFBS for each TF (in all genes in selected Gene ontology terms):
(In the same order as above)
80, 72, 66, 64, 63, 64, 59, 55, 52, 58, 56, 49, 51, 46, 43

Corresponding number of genes (of selected Gene ontology terms) each TF is involved with:
(In the same order as above)
38, 34, 27, 28, 29, 29, 22, 24, 24, 27, 26, 20, 23, 21, 22

Corresponding number of selected Gene ontology terms each TF is involved with:
(In the same order as above)
6, 6, 6, 6, 6, 6, 6, 6, 6, 6, 6, 6, 6, 6, 6

Cluster for columns 951 to 966, rows 139 to 141

Gene ontology terms

relaxation of vascular smooth muscle | GMP biosynthetic process | cGMP biosynthetic process | cyclase activity | guanylate cyclase activity | 11-beta-hydroxysteroid dehydrogenase activity | cortisol secretion |

immunoglobulin binding | regulation of blood pressure | vasopressin secretion | penile erection | vasoconstriction | vasodilation | glomerular filtration | diuresis | natriuresis

TFs

V$SMAD4_Q6 | F$STRE_01 | V$SREBP1_02

Information

All related TFs:
(List of all TFs that are related to any of the PWMs)
SREBP-1, SREBP-1a, SREBP-1b, SREBP-1c, Smad4

Ranked gene list:
(All genes of the selected Gene ontology terms with hits of any of the selected TFs, ranked according to the total number of TFBS)
S100A6, INS, PER1, GUCA2A, NPPC, NOS3, CNP, AVP, ISYNA1, ADM, FGFR3, ERAF, TNF, COMT, CCHCR1, TPM2, MTHFR, HGS, GUCA2B, BDKRB2, VASP, PTGDS, OSM, FLJ32987, EMD, CALM3, CALM1, BGN, TNFSF13, SCNN1G, RHOA, PGF, PAF1, NR3C2, HSPA9, F2, AKT1, ZNF395, PTGS1, P2RY2, KLK1, GNAI2, CYP11A1, CEBPZ, CALR, ADORA2A, ADK, UCN, UBTF, SRC, SLC5A2, S1PR2, RYR1, RENBP, PTPRCAP, PTGER1, PROP1, PNMT, PIGR, PDE4A, MT2A, MBTPS1, LRSAM1, LLGL2, KCNS3, KCNN3, KCNA5, KCNA2, IGF2, HTR2A, GUK1, GEM, GAD1, FLNA, EGR1, ECE1, DPEP1, CYBA, COL1A1, CLCNKB, CILP2, CCL27, CBX1, ATP2A3, ALOX5, ADRBK1, ADD1

Corresponding total number of TFBS:
(For each gene listed above, the total number of TFBS for any of the selected TFs, multiplied by the number of selected Gene ontology terms containing that gene)
20, 16, 15, 12, 11, 9, 9, 9, 8, 8, 7, 7, 6, 6, 6, 5, 5, 5, 5, 5, 4, 4, 4, 4, 4, 4, 4, 4, 3, 3, 3, 3, 3, 3, 3, 3, 3, 2, 2, 2, 2, 2, 2, 2, 2, 2, 2, 1, 1, 1, 1, 1, 1, 1, 1, 1, 1, 1, 1, 1, 1, 1, 1, 1, 1, 1, 1, 1, 1, 1, 1, 1, 1, 1, 1, 1, 1, 1, 1, 1, 1, 1, 1, 1, 1, 1, 1

Corresponding number of selected Gene ontology terms each gene is involved with:
(In the same order as above)
10, 8, 5, 6, 11, 9, 9, 9, 8, 8, 7, 7, 2, 2, 6, 5, 5, 5, 5, 5, 2, 2, 4, 2, 4, 4, 4, 2, 3, 3, 3, 3, 3, 3, 3, 3, 1, 2, 2, 2, 2, 1, 2, 2, 1, 1, 2, 1, 1, 1, 1, 1, 1, 1, 1, 1, 1, 1, 1, 1, 1, 1, 1, 1, 1, 1, 1, 1, 1, 1, 1, 1, 1, 1, 1, 1, 1, 1, 1, 1, 1, 1, 1, 1, 1, 1, 1

Corresponding number of selected TFs each gene is involved with:
(In the same order as above)
2, 2, 3, 2, 1, 1, 1, 1, 1, 1, 1, 1, 3, 3, 1, 1, 1, 1, 1, 1, 2, 2, 1, 2, 1, 1, 1, 2, 1, 1, 1, 1, 1, 1, 1, 1, 3, 1, 1, 1, 1, 2, 1, 1, 2, 2, 1, 1, 1, 1, 1, 1, 1, 1, 1, 1, 1, 1, 1, 1, 1, 1, 1, 1, 1, 1, 1, 1, 1, 1, 1, 1, 1, 1, 1, 1, 1, 1, 1, 1, 1, 1, 1, 1, 1, 1, 1

Gene ontology terms ranked according to mean of effect sizes in cluster:
natriuresis | vasoconstriction | vasodilation | guanylate cyclase activity | GMP biosynthetic process | diuresis | cyclase activity | cGMP biosynthetic process | relaxation of vascular smooth muscle | regulation of blood pressure | glomerular filtration | 11-beta-hydroxysteroid dehydrogenase activity | immunoglobulin binding | cortisol secretion | vasopressin secretion | penile erection

Corresponding mean of effect sizes of each Gene ontology term:
(In the same order as above)
2.27, 2.08, 1.83, 1.67, 1.59, 1.53, 1.48, 1.29, 1.20, 1.11, 0.99, 0.64, 0.45, 0.30, -0.06, -0.10

Corresponding total number of genes of each Gene ontology term:
(In the same order as above)
83, 171, 213, 46, 30, 72, 53, 70, 50, 99, 145, 73, 23, 68, 26, 32

Corresponding number of genes of each Gene ontology term with at least one TFBS (of selected TFs):
(In the same order as above)
19, 36, 39, 12, 9, 14, 11, 15, 11, 19, 20, 12, 3, 10, 3, 5

Corresponding total number of TFBS (of selected TFs) for each Gene ontology term (in all genes):
(In the same order as above)
26, 42, 52, 13, 9, 17, 12, 18, 11, 23, 25, 16, 5, 15, 4, 5

Corresponding number of selected TFs each Gene ontology term is involved with:
(In the same order as above)
3, 3, 3, 3, 3, 3, 3, 3, 3, 3, 3, 3, 3, 3, 3, 3

TFs ranked according to mean of effect sizes in cluster:
F$STRE_01, V$SMAD4_Q6, V$SREBP1_02

Corresponding mean of effect sizes of each TF:
(In the same order as above)
1.39, 1.05, 0.98

Corresponding total number of TFBS for each TF (genome-wide):
(In the same order as above)
651, 666, 693

Corresponding total number of TFBS for each TF (in all genes in selected Gene ontology terms):
(In the same order as above)
98, 94, 101

Corresponding number of genes (of selected Gene ontology terms) each TF is involved with:
(In the same order as above)
33, 34, 38

Corresponding number of selected Gene ontology terms each TF is involved with:
(In the same order as above)
16, 16, 16

Cluster for columns 365 to 370, rows 27 to 32

Gene ontology terms

Golgi membrane | Golgi stack | Golgi apparatus | trans-Golgi network | endoplasmic reticulum | protein transport

TFs

V$GABP_B | V$ELK1_02 | V$TAXCREB_01 | V$ATF6_01 | V$HLF_01 | V$E4BP4_01

Information

All related TFs:
(List of all TFs that are related to any of the PWMs)
ATF6, CREB, E4BP4, Elk-1, Elk-1-isoform1, GABP-alpha, GABP-alpha:GABP-beta, GABP-beta1, GABP-beta2, Hlf, Tax, deltaCREB

Ranked gene list:
(All genes of the selected Gene ontology terms with hits of any of the selected TFs, ranked according to the total number of TFBS)
STX5, ARF1, UBB, GOLGA5, LMAN1, ING1, CDK5R1, ARHGAP4, ARF4, RPS27A, RAB6A, GOLGB1, GOLGA3, SCFD1, RER1, RAB4A, MGAT1, GOLGA1, GABARAPL2, COPG, COPE, TPT1, TNFSF13, SEC62, PER1, MTG1, JUN, GABARAP, ENOPH1, COPB1, ATAD2, UBC, UBA52, TG, SAR1A, RAB2A, NRSN1, IQGAP1, VPS53, VIM, VAMP3, VAMP2, TFRC, SLC30A7, NFE2L2, NEU1, MBTPS1, LAMP2, KDELR1, INSIG2, GOSR2, GCS1, GBF1, FLJ21865, DPM1, CHPF, CALR, ATF6, ARFGEF2, ARF6, APP, ACP2, ACHE, ABL2, WASL, VPS45, TMEM201, SEC24A, RPN1, RABGEF1, RAB34, PRNP, POFUT2, PCSK7, NPC1, NCSTN, LMAN2, LDLR, GTF3A, GPSN2, GORASP1, GBA2, FANCD2, DDIT3, CYB5R4, CTBP1, CSNK1A1, CPE, COPB2, CLTC, CANX, ATF4, ZFYVE9, YIF1A, VPS29, VAPB, VAMP4, UFD1L, UBE2J1, TXNDC12, TRIM3, TIMM44, TFAP2A, STMN3, SRPR, SRP54, SRP19, SREBF1, SNAP25, SLC31A1, SLC30A3, SLC27A5, SERP1, SEC31A, SEC23A, SCAMP1, RPS19, RPN2, RERE, RASGRP1, RANBP1, RAB11A, PPIG, POR, POFUT1, PMM1, PLEKHA8, PIK3C3, PIGW, PFN1, P4HB, OSBP, NMNAT1, NDOR1, MDM4, MARCH6, MAP4, LNPEP, KTN1, KIF3C, ITPR2, IKBKAP, HYOU1, HSP90AA1, HAT1, GPAA1, GGA2, GCC1, GBA, GABARAPL1, FKBP2, FAM168B, EXOC3, ERF, ERC1, EIF2A, EDC4, DPM3, DPAGT1, DNAJB11, DNAJB1, DHRS11, CYTIP, CXCL17, CSNK2B, CORO7, CLGN, CDC42, CASP9, CALM1, C1QBP, BNIP1, BET1L, B3GALTL, B3GALT4, ATP7A, ATG7, ATG5, ARID2, AP2B1, AP1S1, AP1M1, AKT1, AHCYL1, ABCB9, WBP1, VPS54, VKORC1, VCP, USP3, UBXN11, TXNDC5, TSG101, TRIM21, TRIB3, TPX2, TPP2, TMED2, TLR9, TAPBP, SYVN1, STX10, STMN1, STK24, STIM1, ST3GAL5, SREBF2, SPTLC2, SORL1, SLC39A7, SLC30A5, SLC2A4RG, SLC25A1, SERPINH1, SEPS1, SEC61G, SEC24D, SEC24C, SEC16A, SEC11A, SCP2, SC4MOL, SASH3, RTN4, RFT1, RABGAP1, RAB43, RAB21, PTDSS2, PSMB8, PSENEN, PRKCZ, PLEKHF1, PJA2, PITPNM1, PIGC, PIGB, PEX16, PAK4, OSBPL2, ORMDL3, ORAI2, ORAI1, OPTN, NDFIP1, NDEL1, NAPA, MT1B, MON2, MINPP1, MICAL1, MCFD2, MBIP, MAP1LC3A, MAN2B1, LCLAT1, KDELR2, KCNC1, ITPR3, IL6R, IL27, IFRD1, ICA1, HSF4, HRAS, HPS1, HIP1R, HERPUD1, HCST, HAX1, HAVCR1, GYLTL1B, GUK1, GSK3B, GRIN1, GPER, GPAM, GOLPH3, GGA3, GAK, FKRP, FKBP14, FGFR3, FASN, FAP, EXOC1, ERP29, EPOR, EIF4E, EGFL7, EDEM2, DPM2, DNAJC10, DNAJB9, DNAJB2, DDOST, DCTN2, DBNL, DBN1, CYCS, CSGALNACT2, CPD, CP, COPA, COG8, COG3, CLINT1, CHERP, CGB, CERK, CD81, CD47, CD4, CBLN3, CASP7, CASP3, CABP1, BSCL2, BFAR, BAT2, B4GALT6, ATF3, ASAP1, ARL6IP5, ARG2, ARFGAP3, APBA3, AP2S1, ALG8, ALG5, ALG13, ALG1, ALDH3A2, AFTPH, ADAMTS3

Corresponding total number of TFBS:
(For each gene listed above, the total number of TFBS for any of the selected TFs, multiplied by the number of selected Gene ontology terms containing that gene)
18, 18, 15, 15, 12, 12, 12, 12, 12, 10, 10, 10, 10, 9, 8, 8, 8, 8, 8, 8, 8, 6, 6, 6, 6, 6, 6, 6, 6, 6, 5, 5, 5, 5, 5, 5, 5, 4, 4, 4, 4, 4, 4, 4, 4, 4, 4, 4, 4, 4, 4, 4, 4, 4, 4, 4, 4, 4, 4, 4, 4, 4, 4, 3, 3, 3, 3, 3, 3, 3, 3, 3, 3, 3, 3, 3, 3, 3, 3, 3, 3, 3, 3, 3, 3, 3, 3, 3, 3, 3, 3, 2, 2, 2, 2, 2, 2, 2, 2, 2, 2, 2, 2, 2, 2, 2, 2, 2, 2, 2, 2, 2, 2, 2, 2, 2, 2, 2, 2, 2, 2, 2, 2, 2, 2, 2, 2, 2, 2, 2, 2, 2, 2, 2, 2, 2, 2, 2, 2, 2, 2, 2, 2, 2, 2, 2, 2, 2, 2, 2, 2, 2, 2, 2, 2, 2, 2, 2, 2, 2, 2, 2, 2, 2, 2, 2, 2, 2, 2, 2, 2, 2, 2, 2, 2, 2, 2, 2, 2, 2, 2, 2, 2, 2, 2, 2, 2, 2, 1

Corresponding number of selected Gene ontology terms each gene is involved with:
(In the same order as above)

6, 6, 5, 5, 6, 3, 3, 6, 4, 5, 5, 5, 5, 3, 4, 4, 4, 4, 4, 4, 4, 3, 3, 2, 1, 6, 2, 2, 3, 6, 3, 5, 5, 5, 5, 5, 5, 5, 2, 2, 2, 2, 4, 2, 1, 4, 2, 4, 4, 2, 4, 4, 4, 4, 1, 4, 4, 2, 2, 4, 4, 2, 2, 2, 3, 3, 3, 1, 3, 1, 3, 1, 1, 1, 3, 3, 3, 3, 1, 3, 3, 1, 1, 1, 1, 1, 1, 3, 3, 3, 3, 1, 1, 2, 2, 2, 2, 1, 1, 1, 2, 1, 2, 1, 2, 2, 1, 1, 2, 1, 1, 1, 1, 1, 2, 2, 1, 2, 2, 1, 1, 2, 1, 1, 1, 2, 2, 2, 1, 1, 1, 2, 1, 1, 1, 1, 1, 2, 1, 1, 1, 2, 2, 2, 1, 1, 2, 2, 2, 2, 1, 1, 1, 1, 1, 1, 2, 1, 1, 1, 2, 1, 1, 1, 1, 1, 2, 2, 1, 1, 1, 2, 2, 2, 1, 2, 1, 2, 1, 2, 2, 1

Corresponding number of selected TFs each gene is involved with:
(In the same order as above)
3, 3, 3, 3, 2, 4, 4, 2, 3, 2, 2, 2, 2, 3, 2, 2, 2, 2, 2, 2, 2, 2, 2, 3, 6, 1, 3, 3, 2, 1, 2, 1, 1, 1, 1, 1, 1, 1, 2, 2, 2, 2, 1, 2, 4, 1, 2, 1, 1, 2, 1, 1, 1, 1, 4, 1, 1, 2, 2, 1, 1, 2, 2, 2, 1, 1, 1, 3, 1, 3, 1, 3, 3, 3, 1, 1, 1, 1, 3, 1, 1, 3, 3, 3, 3, 3, 3, 1, 1, 1, 1, 3, 2, 1, 1, 1, 1, 2, 2, 2, 1, 2, 1, 2, 1, 1, 2, 2, 1, 2, 2, 2, 2, 2, 1, 1, 2, 1, 1, 2, 2, 1, 2, 2, 2, 1, 1, 1, 2, 2, 2, 1, 2, 2, 2, 2, 2, 1, 2, 2, 2, 1, 1, 1, 2, 2, 1, 1, 1, 1, 2, 2, 2, 2, 2, 2, 1, 2, 2, 2, 1, 2, 2, 2, 2, 2, 1, 1, 2, 2, 2, 1, 1, 1, 2, 1, 2, 1, 2, 1, 1, 2, 2, 2, 2, 1

Gene ontology terms ranked according to mean of effect sizes in cluster:
endoplasmic reticulum | protein transport | Golgi membrane | Golgi apparatus | Golgi stack | trans-Golgi network

Corresponding mean of effect sizes of each Gene ontology term:
(In the same order as above)
2.96, 2.88, 2.61, 2.42, 1.96, 1.90

Corresponding total number of genes of each Gene ontology term:
(In the same order as above)
771, 146, 93, 482, 100, 300

Corresponding number of genes of each Gene ontology term with at least one TFBS (of selected TFs):
(In the same order as above)
227, 54, 31, 139, 37, 85

Corresponding total number of TFBS (of selected TFs) for each Gene ontology term (in all genes):
(In the same order as above)
337, 79, 56, 201, 58, 119

Corresponding number of selected TFs each Gene ontology term is involved with:
(In the same order as above)
6, 6, 6, 6, 6, 6

TFs ranked according to mean of effect sizes in cluster:
V$ATF6_01, V$ELK1_02, V$E4BP4_01, V$GABP_B, V$TAXCREB_01, V$HLF_01

Corresponding mean of effect sizes of each TF:
(In the same order as above)
4.37, 3.14, 2.38, 1.92, 1.78, 1.13

Corresponding total number of TFBS for each TF (genome-wide):
(In the same order as above)
670, 627, 635, 625, 674, 657

Corresponding total number of TFBS for each TF (in all genes in selected Gene ontology terms):
(In the same order as above)
194, 138, 143, 117, 138, 120

Corresponding number of genes (of selected Gene ontology terms) each TF is involved with:
(In the same order as above)
99, 79, 77, 74, 82, 70

Corresponding number of selected Gene ontology terms each TF is involved with:
(In the same order as above)
6, 6, 6, 6, 6, 6

Cluster for columns 349 to 368, rows 47 to 49

Gene ontology terms

cell envelope | outer membrane | porin activity | abscission | ethylene biosynthetic process | merozoite dense granule | melanosome | organelle | oligosaccharide biosynthetic process | checkpoint clamp complex | transpiration | vacuolar membrane | vacuole | multivesicular body | autophagic vacuole | autophagy | Golgi membrane | Golgi stack | Golgi apparatus | trans-Golgi network

TFs

V$SREBP1_01 | V$ARNT_02 | V$XBP1_01

Information

All related TFs:
(List of all TFs that are related to any of the PWMs)
Arnt, SREBP-1, SREBP-1a, SREBP-1b, SREBP-1c, XBP-1

Ranked gene list:
(All genes of the selected Gene ontology terms with hits of any of the selected TFs, ranked according to the total number of TFBS)
LAMP1, RAB5A, CALR, RPS27A, RAB7A, GABARAP, CTSA, GAA, BECN1, BAX, CD63, VPS26A, STX6, RRAGB, PSAP, MGAT1, IGF2R, ATP6V0C, ARFGEF2, APEX1, TMEM201, SFRS5, GOLGA1, GEMIN4, GALT, VPS11, UNC13D, TOM1, TNFSF13, SNX2, PDCD6IP, MTHFR, MDM4, MCOLN1, ING1, HSPD1, HPS1, GOLGA3, GAPDH, GALNS, CHMP4A, ATG3, ALG1, ABCB6, VDAC1, RPN1, RAB4A, PRIM2, MYD88, KDELR1, HPS5, HPS3, GGA2, GCS1, DHDDS, CTRL, COPG, CAV1, CASP3, ACTA1, VPS16, VLDLR, UVRAG, UBE4B, TSC2, TSC1, TRPV1, TPT1, TFAP2A, TBC1D20, SUB1, SLC7A1, SLC24A5, SDF4, S100A6, RPLP2, RNASEH2C, RGS20, RERE, RER1, RABAC1, RAB21, QTRT1, PRDX2, PNKD, PLDN, PLA2G1B, PLA2G15, PAICS, P76, OSBP, NR2F1, NPTN, NAPA, NAGPA, MGAT3, MARCH8, LONP1, INVS, INSIG1, GCC1, FADD, EXOC3, ERF, ENOPH1, EEF1A1, DSCR3, DNASE1, CYTH3, CUX1, CPT1A, COPB2, CNO, CHAF1A, CD164, CCL18, CASP9, BNIP3L, ATXN3, ATP6V1H, ATP6V1A, ATP6V0D1, AP1GBP1, AIDA, AGPAT1, ACOX1, VPS52, USP14, TP53INP1, TOM1L2, TMED10, TM9SF2, TIMM50, TIMM13, STX18, STMN1, SRRM2, SLC30A3, SHCBP1, SCAP, S100B, RPS9, RHEB, RANBP2, RAD17, RABEP1, RAB12, PRKAR1A, PLEKHA8, PIM1, PHF20, PEX7, PEX3, PCDH8, MYOC, MON2, MAPK1, LSS, LOXL1, INSIG2, HSPE1, HRAS, HMGB1, HLF, HADHB, GPR3, GPR132, GLTSCR2, GBA, FTMT, FTCD, FOXM1, FLVCR2, FBL, FAM168B, DOLK, CTDSP2, CORO7, CLN3, CLCN5, CERK, CASP7, C18orf8, BTG3, B4GALT7, B3GALT4, ATP5A1, ATG9B, ARFIP2, ARAP1, ANK1, ADAM7

Corresponding total number of TFBS:

(For each gene listed above, the total number of TFBS for any of the selected TFs, multiplied by the number of selected Gene ontology terms containing that gene)
30, 14, 10, 9, 9, 9, 9, 8, 8, 8, 7, 6, 6, 6, 6, 6, 6, 6, 6, 6, 5, 5, 5, 5, 5, 4, 4, 4, 4, 4, 4, 4, 4, 4, 4, 4, 4, 4, 4, 4, 4, 4, 4, 3, 3, 3, 3, 3, 3, 3, 3, 3, 3, 3, 3, 3, 3, 3, 3, 2, 2, 2, 2, 2, 2, 2, 2, 2, 2, 2, 2, 2, 2, 2, 2, 2, 2, 2, 2, 2, 2, 2, 2, 2, 2, 2, 2, 2, 2, 2, 2, 2, 2, 2, 2, 2, 2, 2, 2, 2, 2, 2, 2, 2, 2, 2, 2, 2, 2, 2, 2, 2, 2, 2, 2, 2, 2, 2, 2, 1, 1, 1, 1, 1, 1, 1, 1, 1, 1, 1, 1, 1, 1, 1, 1, 1, 1, 1, 1, 1, 1, 1, 1, 1, 1, 1, 1, 1, 1, 1, 1, 1, 1, 1, 1, 1, 1, 1, 1, 1, 1, 1, 1, 1, 1, 1, 1, 1, 1, 1, 1, 1, 1, 1, 1, 1, 1, 1, 1, 1, 1, 1, 1, 1, 1, 1, 1, 1, 1

Corresponding number of selected Gene ontology terms each gene is involved with:
(In the same order as above)
10, 7, 5, 9, 9, 3, 9, 4, 4, 4, 7, 3, 3, 2, 3, 3, 3, 3, 2, 3, 5, 5, 5, 5, 5, 2, 2, 2, 4, 2, 4, 2, 2, 2, 4, 4, 4, 4, 2, 2, 2, 2, 2, 2, 3, 3, 3, 3, 3, 3, 1, 3, 3, 3, 1, 1, 3, 3, 3, 3, 2, 2, 2, 1, 1, 1, 2, 2, 2, 1, 1, 2, 2, 1, 2, 2, 1, 1, 2, 2, 2, 1, 1, 1, 2, 2, 2, 1, 2, 1, 2, 2, 1, 1, 2, 2, 1, 2, 1, 1, 2, 1, 2, 1, 2, 2, 1, 2, 1, 2, 1, 2, 2, 1, 1, 2, 1, 1, 1, 1, 2, 2, 1, 1, 1, 1, 1, 1, 1, 1, 1, 1, 1, 1, 1, 1, 1, 1, 1, 1, 1, 1, 1, 1, 1, 1, 1, 1, 1, 1, 1, 1, 1, 1, 1, 1, 1, 1, 1, 1, 1, 1, 1, 1, 1, 1, 1, 1, 1, 1, 1, 1, 1, 1, 1, 1, 1, 1, 1, 1, 1, 1, 1, 1, 1, 1, 1, 1, 1, 1, 1, 1, 1, 1, 1, 1

Corresponding number of selected TFs each gene is involved with:
(In the same order as above)
3, 2, 2, 1, 1, 3, 1, 2, 2, 2, 1, 2, 2, 3, 2, 2, 2, 2, 3, 2, 1, 1, 1, 1, 1, 2, 2, 2, 1, 2, 1, 2, 2, 2, 1, 1, 1, 1, 2, 2, 2, 2, 2, 2, 1, 1, 1, 1, 1, 1, 3, 1, 1, 1, 3, 3, 1, 1, 1, 1, 1, 1, 1, 2, 2, 2, 1, 1, 1, 2, 2, 1, 1, 2, 1, 1, 2, 2, 1, 1, 1, 2, 2, 2, 1, 1, 1, 2, 1, 2, 1, 1, 2, 2, 1, 1, 2, 1, 2, 2, 1, 2, 1, 2, 1, 1, 2, 1, 2, 1, 2, 1, 1, 2, 2, 1, 2, 2, 2, 2, 1, 1, 2, 2, 2, 2, 1, 1, 1, 1, 1, 1, 1, 1, 1, 1, 1, 1, 1, 1, 1, 1, 1, 1, 1, 1, 1, 1, 1, 1, 1, 1, 1, 1, 1, 1, 1, 1, 1, 1, 1, 1, 1, 1, 1, 1, 1, 1, 1, 1, 1, 1, 1, 1, 1, 1, 1, 1, 1, 1, 1, 1, 1, 1, 1, 1, 1, 1, 1, 1, 1, 1, 1, 1, 1, 1

Gene ontology terms ranked according to mean of effect sizes in cluster:
outer membrane | cell envelope | autophagy | melanosome | trans-Golgi network | vacuolar membrane | autophagic vacuole | multivesicular body | vacuole | Golgi apparatus | porin activity | organelle | oligosaccharide biosynthetic process | merozoite dense granule | transpiration | checkpoint clamp complex | ethylene biosynthetic process | Golgi membrane | Golgi stack | abscission

Corresponding mean of effect sizes of each Gene ontology term:
(In the same order as above)
2.73, 2.46, 2.27, 2.18, 2.16, 2.05, 2.04, 1.78, 1.77, 1.73, 1.60, 1.56, 1.54, 1.36, 1.22, 0.72, 0.53, 0.39, 0.16, -0.41

Corresponding total number of genes of each Gene ontology term:
(In the same order as above)
259, 91, 195, 56, 300, 72, 63, 67, 208, 482, 77, 255, 25, 40, 23, 42, 25, 93, 100, 32

Corresponding number of genes of each Gene ontology term with at least one TFBS (of selected TFs):
(In the same order as above)
35, 13, 34, 11, 42, 16, 12, 13, 33, 66, 12, 39, 6, 6, 3, 6, 3, 13, 13, 2

Corresponding total number of TFBS (of selected TFs) for each Gene ontology term (in all genes):
(In the same order as above)
51, 17, 54, 16, 60, 24, 21, 18, 46, 88, 15, 48, 8, 7, 5, 7, 5, 14, 16, 2

Corresponding number of selected TFs each Gene ontology term is involved with:
(In the same order as above)
3, 3, 3, 3, 3, 3, 3, 3, 3, 3, 3, 3, 3, 3, 3, 3, 3, 3, 3, 2

TFs ranked according to mean of effect sizes in cluster:
V$ARNT_02, V$SREBP1_01, V$XBP1_01

Corresponding mean of effect sizes of each TF:

(In the same order as above)
1.69, 1.69, 1.09

Corresponding total number of TFBS for each TF (genome-wide):
(In the same order as above)
625, 629, 639

Corresponding total number of TFBS for each TF (in all genes in selected Gene ontology terms):
(In the same order as above)
184, 178, 160

Corresponding number of genes (of selected Gene ontology terms) each TF is involved with:
(In the same order as above)
91, 92, 81

Corresponding number of selected Gene ontology terms each TF is involved with:
(In the same order as above)
20, 19, 20

Cluster for columns 98 to 108, rows 423 to 437

Gene ontology terms

nucleotide biosynthetic process | nucleotide metabolic process | PCNA complex | single-stranded DNA binding | DNA replication | DNA duplex unwinding | DNA replication factor A complex | DNA repair | replication fork | cytoplasmic replication fork | nuclear replication fork

TFs

V$E2F_Q6_01 | V$E2F_Q4_01 | V$E2F_Q3_01 | V$E2F1_Q4_01 | V$E2F1_Q6_01 | V$E2F_03 | V$E2F4DP2_01 | V$E2F1DP2_01 | V$E2F1DP1_01 | V$E2F_Q6 | V$E2F_Q4 | V$E2F_Q3 | V$E2F4DP1_01 | V$E2F1DP1RB_01 | V$E2F_02

Information

All related TFs:
(List of all TFs that are related to any of the PWMs)
DP-1, E2F, E2F+E4, E2F-1, E2F-1:DP-1, E2F-1:DP-2, E2F-2, E2F-3a, E2F-4, E2F-4:DP-1, E2F-4:DP-2, E2F-5, E2F-7, pRb:E2F-1:DP-1

Ranked gene list:
(All genes of the selected Gene ontology terms with hits of any of the selected TFs, ranked according to the total number of TFBS)
MCM7, PCNA, MCM2, EXO1, MCM6, RPA2, CDC6, CLSPN, MCM5, LIG1, FUS, DUT, CCNO, UNG, TOPBP1, APRT, ISCU, CAD, E2F1, RRM2, POLA1, LIG4, H2AFX, DHFR, SLC25A1, RFC1, CDKN1A, CCND1, PPAT, DTYMK, DCK, CDT1, PURA, CHAF1A, CDC7, TIPIN, GINS1, UHRF1, TYMP, RBL1, HIST1H4I, CDKN1B, TP53, POLE2, MCM3, GMNN, CDC25A, HNRNPA1, SMC3, REV1, NR4A1, MUS81, JUN, E2F3, CD3EAP, MYCN, CUX1, PROC, MTHFR, MCM4, SMC6, SHMT2, SFRS1, REV3L, MDM2, MCM10, HMGB2, XPC, MLH1, HIST4H4, DBP, CETN3, CDC2, RASSF1, PTMA, PRPF19, LMNB1, HIST1H2AM, H2AFZ, FOXO3, E2F2, CBX3, TP73, RFC3, MYB, GPS2, DMC1, PPM1D, CDC25B, ZFP36, RAD54L, PAICS, NBN, MSH3, MSH2, MARCKSL1, CKB, CDK5, CCND2, ADK, SKP2, SF3B3, RRP1, RANBP1, NCL, LRBA, FBXO5, FANCC, CD68, STMN1, NME4, NFIB, MXI1, LEF1, IRS1, ING3, HIST1H3H, HCFC1, GAPDH, DTL, DPAGT1, BACH1,

SLBP, RB1, RAD51, PTPRU, PSMD9, PRKDC, CDK6, CCNB1IP1, AKT1, POLE, NOLC1, NEUROD1, HIST1H3F, HIST1H3E, GINS2, FANCI, SLC38A1, RNF34, PPP1R14B, MTHFD1, MCM9, LMNB2, CHEK1, ALKBH2, ZNRF1, XRCC6, UBB, TFDP1, TCF3, SUMO1, SRCAP, SMC1A, SAP130, RRM1, RNF4, RECQL4, PTCH1, NR1H2, IGF1R, GUK1, CUL4A, CTSD, CDKN1C, WAPAL, NFIA, MYC, MAT2A, GART, FBXL20, FASN, CLPX, CBX5, ANXA5, VPRBP, TEAD1, SP1, SMARCA4, RDH5, POLE4, PER1, NOL3, NFIC, NDRG1, NADK, MKKS, MIB1, MGC20604, MEI1, LMOD1, KCTD13, HNRNPC, H3F3B, GMPS, GLTSCR2, FOXM1, ACTB, XRCC5, VIM, UGP2, TRRAP, TFAM, STAT3, SSSCA1, SPSB3, SIRT6, RAD51C, POLI, POLD1, PDGFA, MTR, MND1, MDC1, LONP1, ING1, HRAS, HIST1H3C, HIF1A, GPR180, E2F7, DPYD, ATP6V0D1, APOE, AK2, YY1, UBE2I, SMARCA2, RAD51L1, PSMD4, PSAT1, PRPS1, PPAN, PMS1, PKMYT1, PARP4, ORC3L, OBFC2B, NPM1, IMMT, HLTF, HIST1H3G, DNMT3A, DDB2, DCLRE1A, CDK5R2, CDK5R1, CDC25C, CDC23, CCNE2, BCL2L1, ADAMTS13, URG4, TACR2, PRMT1, PPP2CA, POLR2I, PDXK, NAPRT1, KRT8, ISYNA1, IGF2, HIRA, FBXO18, FANCL, ENO2, CTNNB1, CENPK, BTAF1, AK5, ABCB1

Corresponding total number of TFBS:
(For each gene listed above, the total number of TFBS for any of the selected TFs, multiplied by the number of selected Gene ontology terms containing that gene)
135, 120, 105, 104, 90, 84, 77, 72, 60, 56, 55, 54, 52, 49, 48, 48, 45, 45, 44, 42, 42, 42, 42, 42, 40, 40, 40, 39, 36, 36, 36, 36, 35, 35, 35, 32, 32, 30, 30, 30, 30, 30, 28, 28, 28, 27, 27, 25, 24, 24, 24, 24, 24, 24, 24, 22, 22, 21, 21, 21, 20, 20, 20, 20, 20, 18, 18, 16, 16, 16, 16, 16, 16, 15, 15, 15, 15, 15, 15, 15, 15, 15, 14, 14, 14, 14, 14, 13, 13, 12, 12, 12, 12, 12, 12, 12, 12, 12, 12, 12, 11, 11, 11, 11, 11, 11, 11, 11, 11, 10, 10, 10, 10, 10, 10, 10, 10, 10, 10, 10, 10, 9, 9, 9, 9, 9, 9, 9, 9, 9, 8, 8, 8, 8, 8, 8, 8, 7, 7, 7, 7, 7, 7, 7, 7, 6, 6, 6, 6, 6, 6, 6, 6, 6, 6, 6, 6, 6, 6, 6, 6, 6, 5, 5, 5, 5, 5, 5, 5, 5, 5, 5, 4, 4, 4, 4, 4, 4, 4, 4, 4, 4, 4, 4, 4, 4, 4, 4, 4, 4, 4, 4, 4, 4, 4, 3, 3, 3, 3, 3, 3, 3, 3, 3, 3, 3, 3, 3, 3, 3, 3, 3, 3, 3, 3, 3, 3, 3, 3, 3, 3, 3, 2, 2, 2, 2, 2, 2, 2, 2, 2, 2, 2, 2, 2, 2, 2, 2, 2, 2, 2, 2, 2, 2, 2, 2, 2, 2, 2, 1, 1, 1, 1, 1, 1, 1, 1, 1, 1, 1, 1, 1, 1, 1, 1, 1, 1, 1

Corresponding number of selected Gene ontology terms each gene is involved with:
(In the same order as above)
9, 8, 7, 8, 6, 7, 7, 6, 4, 8, 5, 6, 4, 7, 6, 4, 5, 3, 4, 3, 6, 6, 7, 7, 8, 8, 4, 3, 3, 4, 4, 3, 5, 7, 7, 4, 4, 2, 3, 2, 2, 2, 7, 2, 4, 3, 3, 5, 2, 6, 4, 6, 3, 2, 6, 2, 2, 7, 3, 7, 2, 2, 2, 5, 2, 6, 3, 4, 4, 2, 2, 4, 4, 1, 1, 1, 1, 1, 1, 1, 1, 1, 1, 2, 2, 1, 2, 1, 1, 2, 6, 1, 6, 2, 3, 2, 1, 1, 1, 2, 1, 1, 1, 1, 1, 1, 1, 1, 1, 1, 2, 1, 2, 1, 1, 2, 1, 1, 1, 1, 1, 1, 1, 1, 3, 9, 1, 3, 3, 1, 3, 1, 1, 2, 1, 1, 1, 4, 4, 1, 1, 1, 1, 1, 1, 7, 1, 1, 2, 6, 1, 1, 2, 1, 6, 1, 2, 1, 6, 1, 3, 1, 1, 2, 1, 2, 1, 1, 5, 1, 1, 1, 1, 1, 1, 1, 1, 2, 2, 1, 1, 1, 1, 1, 2, 4, 1, 1, 1, 2, 1, 1, 2, 1, 1, 1, 1, 1, 2, 3, 1, 1, 1, 3, 1, 3, 1, 1, 1, 3, 3, 1, 1, 1, 3, 1, 3, 3, 1, 1, 1, 1, 1, 1, 1, 1, 2, 2, 1, 1, 1, 1, 1, 2, 2, 1, 1, 1, 1, 1, 2, 1, 1, 1, 2, 1, 1, 1, 2, 1, 1, 2, 2, 1, 1, 1, 1, 1, 1, 1, 1, 1, 1, 1, 1, 1, 1, 1, 1, 1, 1, 1

Corresponding number of selected TFs each gene is involved with:
(In the same order as above)
15, 15, 15, 13, 15, 12, 11, 12, 15, 7, 11, 9, 13, 7, 8, 12, 9, 15, 11, 14, 7, 7, 6, 6, 5, 5, 10, 13, 12, 9, 9, 12, 7, 5, 5, 8, 8, 15, 10, 15, 15, 15, 4, 14, 7, 9, 9, 5, 12, 4, 6, 4, 8, 12, 4, 11, 11, 3, 7, 3, 10, 10, 10, 4, 10, 3, 6, 4, 4, 8, 8, 4, 4, 15, 15, 15, 15, 15, 15, 15, 15, 15, 14, 7, 7, 14, 7, 13, 13, 6, 2, 12, 2, 6, 4, 6, 12, 12, 12, 6, 11, 11, 11, 11, 11, 11, 11, 11, 11, 10, 5, 10, 5, 10, 10, 5, 10, 10, 10, 10, 10, 10, 9, 3, 1, 9, 3, 3, 9, 3, 9, 8, 4, 8, 8, 8, 2, 2, 7, 7, 7, 7, 7, 7, 1, 7, 6, 3, 1, 6, 6, 3, 6, 1, 6, 3, 6, 1, 6, 2, 6, 6, 3, 6, 3, 5, 5, 1, 5, 5, 5, 5, 5, 5, 5, 4, 2, 2, 4, 4, 4, 4, 4, 2, 1, 4, 4, 4, 2, 4, 4, 2, 4, 4, 4, 4, 4, 2, 1, 3, 3, 3, 1, 3, 1, 3, 3, 3, 1, 1, 3, 3, 3, 1, 3, 1, 1, 3, 3, 3, 3, 3, 3, 3, 3, 1, 1, 2, 2, 2, 2, 2, 1, 1, 2, 2, 2, 2, 2, 1, 2, 2, 2, 1, 2, 2, 2, 1, 2, 2, 1, 1, 1, 1, 1, 1, 1, 1, 1, 1, 1, 1, 1, 1, 1, 1, 1, 1, 1, 1, 1

Gene ontology terms ranked according to mean of effect sizes in cluster:
DNA replication | replication fork | nuclear replication fork | cytoplasmic replication fork | DNA replication factor A complex | nucleotide metabolic process | DNA duplex unwinding | nucleotide biosynthetic process | DNA repair | PCNA complex | single-stranded DNA binding

Corresponding mean of effect sizes of each Gene ontology term:
(In the same order as above)
5.57, 4.11, 4.11, 4.11, 3.78, 3.75, 3.63, 3.14, 3.07, 2.70, 1.28

Corresponding total number of genes of each Gene ontology term:
(In the same order as above)
589, 156, 156, 156, 146, 57, 52, 73, 569, 340, 78

Corresponding number of genes of each Gene ontology term with at least one TFBS (of selected TFs):
(In the same order as above)
165, 46, 46, 46, 41, 24, 15, 24, 135, 82, 19

Corresponding total number of TFBS (of selected TFs) for each Gene ontology term (in all genes):
(In the same order as above)
1042, 294, 294, 294, 248, 183, 110, 167, 783, 506, 119

Corresponding number of selected TFs each Gene ontology term is involved with:
(In the same order as above)
15, 15, 15, 15, 15, 15, 15, 15, 15, 15, 15

TFs ranked according to mean of effect sizes in cluster:
V$E2F_03, V$E2F1DP1RB_01, V$E2F4DP1_01, V$E2F4DP2_01, V$E2F_02, V$E2F_Q6_01, V$E2F_Q3_01, V$E2F1_Q4_01, V$E2F1DP2_01, V$E2F_Q4_01, V$E2F1_Q6_01, V$E2F_Q3, V$E2F_Q4, V$E2F1DP1_01, V$E2F_Q6

Corresponding mean of effect sizes of each TF:
(In the same order as above)
5.21, 4.49, 4.47, 4.13, 4.12, 3.63, 3.44, 3.37, 3.27, 3.19, 3.18, 3.04, 2.87, 2.73, 2.36

Corresponding total number of TFBS for each TF (genome-wide):
(In the same order as above)
688, 662, 657, 656, 658, 686, 690, 681, 658, 667, 700, 688, 702, 682, 699

Corresponding total number of TFBS for each TF (in all genes in selected Gene ontology terms):
(In the same order as above)
358, 292, 286, 274, 277, 288, 267, 262, 243, 254, 273, 253, 251, 235, 227

Corresponding number of genes (of selected Gene ontology terms) each TF is involved with:
(In the same order as above)
134, 114, 111, 114, 108, 114, 109, 109, 104, 103, 113, 107, 103, 99, 94

Corresponding number of selected Gene ontology terms each TF is involved with:
(In the same order as above)
11, 11, 11, 11, 11, 11, 11, 11, 11, 11, 11, 11, 11, 11, 11

Cluster for columns 385 to 390, rows 50 to 113

Gene ontology terms

T cell differentiation in the thymus | B cell differentiation | lymphocyte differentiation | tissue development | cell recognition | thyroid-stimulating hormone receptor activity

TFs

V$TBP_Q6 | V$NKX62_Q2 | V$OTX_Q1 | V$PBX1_01 | V$SRY_01 | V$HMGIY_Q3 | V$OCT1_06 | V$TBP_01 | V$TEF_Q6 | V$FOXJ2_02 | V$AFP1_Q6 | V$OCT1_03 | V$SRY_02 | V$FOXO1_01 |

V$FOXO3A_Q1 | V$FOXO4_01 | V$IRF1_Q6 | V$CEBPGAMMA_Q6 | V$CDC5_01 | V$BRN2_01 | V$CDX_Q5 | V$POU1F1_Q6 | V$OCT1_04 | V$TATA_C | V$FREAC7_01 | V$PIT1_Q6 | V$IPF1_Q4 | V$OCT1_Q6 | V$OCT1_05 | V$OCT_Q6 | V$OCT1_Q5_01 | V$OCT1_B | V$OCT_C | V$GATA1_05 | V$FAC1_01 | V$TITF1_Q3 | B$CRP_C | V$GATA_Q6 | V$GATA6_01 | V$TCF4_Q5 | V$LEF1_Q2 | V$BRCA_01 | V$AREB6_04 | V$SOX10_Q6 | V$DBP_Q6 | V$CEBP_01 | V$AP3_Q6 | V$STAT6_01 | V$STAT5A_03 | V$STAT4_01 | V$STAT5A_04 | V$STAT1_03 | V$CEBP_Q3 | V$PAX2_02 | V$HMGIY_Q6 | V$HOXA4_Q2 | V$TST1_01 | V$NFAT_Q6 | V$NFAT_Q4_01 | V$CEBP_Q2_01 | V$CEBPA_01 | V$CEBP_Q2 | V$CEBPB_01 | V$GATA4_Q3

Information

All related TFs:
(List of all TFs that are related to any of the PWMs)
AFP1, ANF-2, AP-3, BRCA1, BRCA1:USF2, Brn1, C/EBP, C/EBPalpha, C/EBPalpha(p20), C/EBPalpha(p30), C/EBPbeta, C/EBPbeta(LAP), C/EBPbeta(p20), C/EBPbeta(p34), C/EBPbeta(p35), C/EBPdelta, C/EBPepsilon, C/EBPgamma, CDC5L, CDX2, Cdx-1, Cdx-2, Cdx-3, DBP, FAC1, FOXJ2, FOXL1, FOXO1, FOXO4, GATA-1, GATA-1A, GATA-2, GATA-3, GATA-4, GATA-5, GATA-5A, GATA-5B, GATA-6, GATA-6A, GATA-6B, HMG, HMG-Y, HMGI-C, HOXA4, IPF1, IRF-1, LEF-1, LEF-1S, NF-AT, NF-AT1, NF-AT1C, NF-AT2, NF-AT3, NF-AT4, Nkx2-1, Nkx6-2, OCA-B, Oct-1, Oct-10, Oct-2, Oct-2.1, Oct-2.2, Oct-2.5, Oct-9, Oct-R, Octa-factor, Otx1, Otx2, POU2F1, POU2F1a, POU2F1b, POU2F1c, POU2F2, POU2F2B, POU2F3, POU3F1, POU3F2, POU4F1, POU4F1(l), POU5F1, POU5F1A, POU5F1B, POU5F1C, Pax-2, Pax-2.1, Pax-2.2, Pax-2a, Pax-2b, Pbx1a, Pit-1, Pit-1-xbb3, Pit-1A, Pit-1B, SRY, STAT1, STAT1alpha, STAT4, STAT5A, STAT6, Sox10, TBP, TCF-1, TCF-1(P), TCF-3, TCF-4, TEF, TEF-xbb1, TFIID, ZEB, oct-B2, oct-B3, octamer-binding

Ranked gene list:
(All genes of the selected Gene ontology terms with hits of any of the selected TFs, ranked according to the total number of TFBS)
CD44, BCL6, IKZF1, PRDM1, CD4, NR3C2, BCL2, CTLA4, PTPRC, RERE, ETS1, GNAS, TG, CXCR4, RUNX1, LEF1, CD68, ID2, BACH2, EBF1, MYB, FOXP1, NP, FLI1, VIM, TCF7, ARL4C, PAX5, CD2, MAML2, GFI1, CTNNB1, CCR7, CD226, TNF, CD69, CD247, PTMA, GRK5, CD96, CADM1, TAP1, LPIN1, IL16, PCNA, MYC, BCL2L1, TAPBP, SILV, IL2RA, TNFSF13, ITGA5, STAT5B, IKZF3, ID3, WNT10B, STAT3, LCK, TAP2, RUNX3, POU2F1, ATM, LBX1, UBC, ITGB1, ITGA4, CD27, SLC3A2, PRH1, DR1, KRAS, FAS, SELL, POU2F2, IL2, TLR1, REL, ITGA6, ENO2, STAT6, ITGB2, CNTNAP1, TNC, STAT5A, STAT4, SSFA2, PRDM2, IL6R, CXCR5, CD5, XBP1, LBR, FOS, RBMS1, CD79A, BMP2, TSHR, TCF4, TBX21, RBM45, RAG2, RAG1, LYN, IL6, IL10, EVI1, CSNK1A1, CDR2, CD40, ABL2, TNFSF13B, PRLR, CKAP4, TAOK3, MYCN, IRF4, HCST, GZMA, EDC4, ALPK1, TP63, TH1L, SH3BP5, NCR3, MAG, LY75, KIRREL3, IL2RB, GPD2, EEF2, CD7, CD52, CD28, C1R, ATP6V1H, TNFRSF25, TMPO, TFRC, TAL1, STRN3, SNRPA, RTEL1, RPL22, RNH1, POU2AF1, POLA1, NOTCH1, MSC, MPG, MMP11, MBP, LSAMP, KLRA1, IRF6, ID1, HSPD1, HRAS, HLA-C, HLA-B, GPR160, FLNB, ERBB2, DST, CGA, CDX2, CDH4, CD79B, CD40LG, CD37, BRWD2, BLNK, BCL10, ADA, VTN, ULBP2, UBE2D1, TSLP, TP53, SPIB, SLC4A3, SEPN1, RPS27A, RHO, PRKCDBP, PPARG, PAX8, OLIG2, NUDT6, NRCAM, NCAN, MYOG, MUC1, KRT31, ITIH4, IL3, FGF2, FGB, EWSR1, ENTPD1, EGF, DSG3, DLEU7, CNTNAP2, CLTA, CEACAM5, CD86, CALCA, C6, BCR, ADK

Corresponding total number of TFBS:
(For each gene listed above, the total number of TFBS for any of the selected TFs, multiplied by the number of selected Gene ontology terms containing that gene)
282, 186, 177, 120, 104, 98, 96, 90, 88, 62, 62, 61, 60, 60, 59, 59, 56, 54, 53, 52, 50, 50, 48, 46, 44, 44, 43, 40, 40, 37, 37, 37, 36, 35, 34, 33, 32, 31, 31, 30, 30, 29, 29, 28, 27, 27, 27, 26, 26, 26, 25, 25, 24, 24, 24, 22, 22, 22, 20, 20, 20, 20, 18, 17, 17, 15, 15, 14, 14, 14, 13, 13, 12, 12, 12, 11, 11, 11, 11, 10, 10, 10, 9, 9, 9, 9, 9, 9, 9, 9, 8, 8, 8, 7, 7, 7, 6, 6, 6, 6, 6, 6, 6, 6, 6, 6, 6, 6, 6, 5, 5, 5, 4, 4, 4, 4, 4, 4, 4, 3, 3, 3, 3, 3, 3, 3, 3, 3, 3, 3, 3, 3, 3, 3, 3, 3, 3, 3, 3, 2, 2, 2, 2, 2, 2, 2, 2, 2, 2, 2, 2, 2, 2, 2, 2, 2, 2, 2, 2, 2, 2, 2, 2, 2, 2, 2, 2, 2, 2, 2, 2, 2, 2, 2, 2, 2, 1, 1, 1, 1, 1, 1, 1, 1, 1, 1, 1, 1, 1, 1, 1, 1, 1, 1, 1, 1, 1, 1, 1, 1, 1, 1, 1, 1, 1, 1, 1, 1, 1, 1,

1, 1

Corresponding number of selected Gene ontology terms each gene is involved with:
(In the same order as above)
6, 3, 3, 2, 4, 2, 3, 2, 4, 2, 1, 1, 3, 1, 1, 1, 2, 1, 1, 2, 2, 1, 2, 1, 1, 1, 1, 2, 4, 1, 1, 1, 1, 1, 1, 1, 1, 1, 1, 1, 1, 1, 1, 1, 1, 3, 1, 1, 1, 2, 5, 1, 1, 1, 1, 1, 1, 2, 1, 1, 1, 1, 1, 1, 3, 3, 2, 1, 1, 1, 1, 2, 2, 4, 1, 1, 1, 1, 1, 2, 1, 3, 1, 1, 1, 1, 1, 1, 3, 2, 2, 1, 1, 1, 1, 1, 1, 1, 1, 3, 3, 1, 2, 2, 1, 1, 1, 3, 2, 1, 1, 1, 1, 1, 2, 1, 1, 2, 1, 1, 3, 1, 1, 1, 1, 1, 3, 1, 1, 3, 1, 3, 1, 1, 1, 2, 1, 1, 1, 1, 1, 1, 1, 1, 1, 1, 1, 1, 1, 1, 1, 2, 1, 1, 1, 1, 1, 2, 1, 2, 1, 1, 1, 1, 1, 2, 2, 1, 2, 1, 2, 1, 1, 1, 1, 1, 1, 1, 1, 1, 1, 1, 1, 1, 1, 1, 1, 1, 1, 1, 1, 1, 1, 1, 1, 1, 1, 1, 1, 1, 1, 1, 1, 1, 1, 1, 1

Corresponding number of selected TFs each gene is involved with:
(In the same order as above)
47, 62, 59, 60, 26, 49, 32, 45, 22, 31, 62, 61, 20, 60, 59, 59, 28, 54, 53, 26, 25, 50, 24, 46, 44, 44, 43, 20, 10, 37, 37, 37, 36, 35, 34, 33, 32, 31, 31, 30, 30, 29, 29, 28, 27, 9, 27, 26, 26, 13, 5, 25, 24, 24, 24, 22, 22, 11, 20, 20, 20, 20, 18, 17, 17, 5, 5, 7, 14, 14, 13, 13, 6, 6, 3, 11, 11, 11, 11, 10, 5, 10, 3, 9, 9, 9, 9, 9, 9, 3, 4, 4, 8, 7, 7, 7, 6, 6, 6, 6, 2, 2, 6, 3, 3, 6, 6, 6, 2, 3, 5, 5, 5, 4, 4, 2, 4, 4, 2, 4, 3, 1, 3, 3, 3, 3, 3, 1, 3, 3, 1, 3, 1, 3, 3, 2, 1, 2, 2, 2, 2, 2, 2, 2, 2, 2, 2, 2, 2, 2, 2, 2, 2, 1, 2, 2, 2, 2, 2, 1, 2, 1, 2, 2, 2, 2, 2, 1, 1, 2, 1, 2, 1, 1, 1, 1, 1, 1, 1, 1, 1, 1, 1, 1, 1, 1, 1, 1, 1, 1, 1, 1, 1, 1, 1, 1, 1, 1, 1, 1, 1, 1, 1, 1, 1, 1, 1, 1, 1

Gene ontology terms ranked according to mean of effect sizes in cluster:
B cell differentiation | T cell differentiation in the thymus | thyroid-stimulating hormone receptor activity | lymphocyte differentiation | cell recognition | tissue development

Corresponding mean of effect sizes of each Gene ontology term:
(In the same order as above)
1.44, 1.23, 1.23, 1.18, 0.96, 0.78

Corresponding total number of genes of each Gene ontology term:
(In the same order as above)
132, 67, 39, 80, 152, 134

Corresponding number of genes of each Gene ontology term with at least one TFBS (of selected TFs):
(In the same order as above)
67, 39, 25, 49, 65, 48

Corresponding total number of TFBS (of selected TFs) for each Gene ontology term (in all genes):
(In the same order as above)
911, 663, 394, 655, 669, 570

Corresponding number of selected TFs each Gene ontology term is involved with:
(In the same order as above)
64, 64, 64, 64, 64, 64

TFs ranked according to mean of effect sizes in cluster:
V$OCT1_06, V$OCT1_05, V$LEF1_Q2, V$OCT1_Q5_01, V$NFAT_Q6, V$OCT_Q6, V$IRF1_Q6, V$HMGIY_Q6, V$SOX10_Q6, V$AFP1_Q6, VOTX_Q1, VOCT1_B, V$GATA4_Q3, V$STAT6_01, V$TCF4_Q5, V$DBP_Q6, V$CEBP_Q2_01, V$POU1F1_Q6, V$HMGIY_Q3, V$CEBP_Q3, V$NKX62_Q2, V$HOXA4_Q2, VCDX_Q5, VCEBP_Q2, VTBP_Q6, VBRN2_01, V$IPF1_Q4, V$PAX2_02, V$NFAT_Q4_01, V$PBX1_01, V$CEBP_01, V$GATA_Q6, V$OCT1_04, V$FOXJ2_02, V$CEBPA_01, V$SRY_01, V$OCT1_Q6, V$STAT5A_03, V$STAT4_01, V$STAT1_03, V$TST1_01, V$AREB6_04, VSRY_02, VCEBPGAMMA_Q6, BCRP_C, VOCT1_03, V$GATA6_01, V$GATA1_05, V$CEBPB_01, V$OCT_C, VTBP_01, VPIT1_Q6, V$FOXO3A_Q1, V$TEF_Q6, V$AP3_Q6, V$STAT5A_04, V$FAC1_01, V$TATA_C, V$FOXO1_01, V$TITF1_Q3, V$FOXO4_01, V$CDC5_01, V$FREAC7_01, V$BRCA_01

Corresponding mean of effect sizes of each TF:
(In the same order as above)
2.17, 2.13, 1.97, 1.91, 1.84, 1.78, 1.68, 1.66, 1.64, 1.58, 1.56, 1.56, 1.55, 1.54, 1.52, 1.52, 1.50, 1.49, 1.48, 1.44, 1.42, 1.41, 1.41, 1.40, 1.39, 1.38, 1.38, 1.35, 1.34, 1.33, 1.29, 1.23, 1.18, 1.17, 1.15, 1.14, 1.13, 1.10, 1.09, 1.01, 1.00, 0.97, 0.97, 0.92, 0.90, 0.86, 0.80, 0.73, 0.67, 0.65, 0.64, 0.60, 0.60, 0.54, 0.54, 0.53, 0.52, 0.47, 0.37, 0.23, 0.20, 0.15, -0.01, -0.05

Corresponding total number of TFBS for each TF (genome-wide):
(In the same order as above)
717, 672, 723, 698, 719, 687, 697, 753, 729, 682, 701, 665, 713, 732, 716, 714, 716, 694, 716, 744, 698, 719, 707, 718, 723, 659, 704, 741, 736, 704, 740, 703, 662, 680, 711, 743, 679, 749, 743, 751, 730, 740, 693, 688, 716, 704, 722, 715, 703, 654, 685, 672, 689, 684, 741, 740, 722, 712, 680, 707, 696, 669, 669, 730

Corresponding total number of TFBS for each TF (in all genes in selected Gene ontology terms):
(In the same order as above)
77, 77, 80, 71, 79, 71, 71, 73, 70, 62, 64, 65, 66, 70, 72, 67, 66, 66, 67, 71, 60, 66, 57, 66, 64, 57, 57, 69, 68, 62, 67, 63, 57, 57, 62, 65, 63, 63, 64, 61, 61, 60, 57, 52, 56, 53, 57, 60, 50, 48, 47, 49, 49, 44, 51, 51, 51, 49, 44, 46, 51, 42, 39, 42

Corresponding number of genes (of selected Gene ontology terms) each TF is involved with:
(In the same order as above)
48, 51, 52, 49, 51, 49, 47, 50, 45, 41, 43, 43, 43, 46, 46, 43, 45, 44, 42, 48, 40, 46, 36, 43, 43, 37, 34, 47, 46, 41, 45, 43, 42, 39, 43, 44, 45, 43, 43, 41, 42, 43, 40, 37, 36, 37, 39, 40, 34, 35, 32, 33, 34, 32, 36, 38, 37, 34, 32, 33, 38, 29, 30, 35

Corresponding number of selected Gene ontology terms each TF is involved with:
(In the same order as above)
6, 6, 6, 6, 6, 6, 6, 6, 6, 6, 6, 6, 6, 6, 6, 6, 6, 6, 6, 6, 6, 6, 6, 6, 6, 6, 6, 6, 6, 6, 6, 6, 6, 6, 6, 6, 6, 6, 6, 6, 6, 6, 6, 6, 6, 6, 6, 6, 6, 6, 6, 6, 6, 6, 6, 6, 6, 6, 6, 6, 6, 6, 6, 6

Cluster for columns 961 to 966, rows 247 to 261

Gene ontology terms

penile erection | vasoconstriction | vasodilation | glomerular filtration | diuresis | natriuresis

TFs

V$AML_Q6 | V$PAX_Q6 | V$NRF2_Q4 | V$MAF_Q6_01 | V$AP1_Q6_01 | V$AP1_Q4_01 | V$AP1_Q6 | V$AP1_C | V$AP1_01 | V$AP1_Q2 | V$AP1FJ_Q2 | V$AP1_Q4 | V$AP1_Q2_01 | V$BACH2_01 | V$NFE2_01

Information

All related TFs:
(List of all TFs that are related to any of the PWMs)
AML1, AML1a, AML1b, AML1c, AML2, AML3, AML3-isoform1, AML3-isoform2, AP-1, Bach1, Bach1:MafK, Bach2, FosB, Fra-1, Fra-2, JunB, JunB:Fra-1, JunB:Fra-2, JunD, JunD:Fra-2, JunD:deltaFosB, LCR-F1, MAF, Maf, MafB, MafF, MafG, MafG:MafG, MafK, NF-E2, Nrf1, Nrf1:MafG, Nrf1:MafK, Nrf2, Nrf2:MafG, Nrf2:MafK, Nrf3, Nrf3:MafK, PEBP2, PEBP2alphaA1, PEBP2alphaA2, PEBP2alphaB1, PEBP2alphaB2, Pax-1, Pax-2, Pax-2a, Pax-3, Pax-4a, Pax-4c, Pax-5, Pax-6, Pax-8, Pax6-1, RUNX2-isoform2, YAP1, c-Fos, c-Jun, c-Jun:FosB, c-Jun:JunD, c-Jun:c-Fos, c-

Maf, deltaFosB, v-Maf

Ranked gene list:
(All genes of the selected Gene ontology terms with hits of any of the selected TFs, ranked according to the total number of TFBS)
ADM, HGS, S100A6, NLRP3, DDR1, ISYNA1, OSM, NOS2, ZFP91, MTHFR, HMOX1, HSPA9, GPI, TNF, LMNA, VASP, PER1, CPA6, AQP2, AKT1, ADORA2A, VGF, RHOA, PTPRCAP, INS, ENO1, EDN2, S1PR2, FLNA, FLJ32987, CNP, NQO1, NOS3, ECE1, UBTF, NPR1, MT2A, HMOX2, NR1D2, NPPC, NOS1, MME, KNG1, ADA, PTK2B, PAOX, OPA1, NUDT2, NPPB, KLF6, EMD, DCTN3, VEGFA, PTTG1IP, PCYT2, GUCA2B, AQP1, TNFRSF9, TES, TCN2, SLC35A1, SCGB1A1, S100A12, PTGER4, PTGDS, P2RX1, NFAT5, MGEA5, IRF6, IL6, DBP, CYBA, CABIN1, C1QL1, AMBP, ADRA1A, VWF, VIPR1, TRIM25, TALDO1, TAC4, SERPINE1, RENBP, RAMP2, RAMP1, PYY, PTGIR, PTGER3, MFSD11, LRSAM1, EMP1, CYGB, CILP2, BGLAP, ARSA, AKR1B1, ADRB2, ACTN4

Corresponding total number of TFBS:
(For each gene listed above, the total number of TFBS for any of the selected TFs, multiplied by the number of selected Gene ontology terms containing that gene)
78, 65, 60, 60, 60, 40, 33, 24, 16, 16, 16, 15, 14, 13, 13, 12, 12, 12, 12, 12, 12, 11, 10, 10, 10, 10, 10, 9, 9, 9, 9, 8, 8, 8, 7, 6, 6, 6, 5, 5, 5, 5, 5, 5, 4, 4, 4, 4, 4, 4, 4, 4, 3, 3, 3, 3, 3, 2, 2, 2, 2, 2, 2, 2, 2, 2, 2, 2, 2, 2, 2, 2, 2, 2, 2, 2, 1, 1, 1, 1, 1, 1, 1, 1, 1, 1, 1, 1, 1, 1, 1, 1, 1, 1, 1, 1, 1

Corresponding number of selected Gene ontology terms each gene is involved with:
(In the same order as above)
6, 5, 5, 5, 5, 4, 3, 4, 2, 2, 2, 3, 2, 1, 1, 1, 4, 4, 3, 1, 1, 1, 2, 1, 5, 1, 2, 1, 1, 1, 3, 2, 4, 1, 1, 6, 1, 3, 5, 5, 5, 5, 5, 1, 1, 2, 2, 2, 4, 1, 1, 1, 1, 1, 1, 3, 3, 1, 2, 1, 2, 1, 2, 1, 2, 2, 1, 2, 2, 1, 2, 1, 2, 2, 1, 1, 1, 1, 1, 1, 1, 1, 1, 1, 1, 1, 1, 1, 1, 1, 1, 1, 1, 1, 1, 1, 1

Corresponding number of selected TFs each gene is involved with:
(In the same order as above)
13, 13, 12, 12, 12, 10, 11, 6, 8, 8, 8, 5, 7, 13, 13, 12, 3, 3, 4, 12, 12, 11, 5, 10, 2, 10, 5, 9, 9, 9, 3, 4, 2, 8, 7, 1, 6, 2, 1, 1, 1, 1, 1, 5, 4, 2, 2, 2, 1, 4, 4, 4, 3, 3, 3, 1, 1, 2, 1, 2, 1, 2, 1, 2, 1, 1, 2, 1, 1, 2, 1, 2, 1, 1, 2, 2, 1, 1, 1, 1, 1, 1, 1, 1, 1, 1, 1, 1, 1, 1, 1, 1, 1, 1, 1, 1, 1

Gene ontology terms ranked according to mean of effect sizes in cluster:
vasoconstriction | diuresis | vasodilation | penile erection | natriuresis | glomerular filtration

Corresponding mean of effect sizes of each Gene ontology term:
(In the same order as above)
2.03, 1.87, 1.25, 1.24, 1.05, 1.00

Corresponding total number of genes of each Gene ontology term:
(In the same order as above)
171, 72, 213, 32, 83, 145

Corresponding number of genes of each Gene ontology term with at least one TFBS (of selected TFs):
(In the same order as above)
50, 22, 57, 10, 22, 39

Corresponding total number of TFBS (of selected TFs) for each Gene ontology term (in all genes):
(In the same order as above)
227, 102, 255, 56, 102, 136

Corresponding number of selected TFs each Gene ontology term is involved with:
(In the same order as above)

15, 15, 15, 15, 15, 15

TFs ranked according to mean of effect sizes in cluster:
V$NFE2_01, V$AP1_01, V$AP1_Q6_01, V$AP1_C, V$AP1FJ_Q2, V$AP1_Q4_01, VMAF_Q6_01, VNRF2_Q4, VPAX_Q6, VAP1_Q6, V$AP1_Q4, V$AP1_Q2_01, V$AP1_Q2, V$AML_Q6, V$BACH2_01

Corresponding mean of effect sizes of each TF:
(In the same order as above)
2.60, 2.30, 1.67, 1.64, 1.61, 1.60, 1.42, 1.38, 1.26, 1.22, 1.11, 1.01, 0.85, 0.85, 0.58

Corresponding total number of TFBS for each TF (genome-wide):
(In the same order as above)
729, 721, 726, 720, 710, 719, 700, 683, 683, 721, 712, 714, 692, 680, 686

Corresponding total number of TFBS for each TF (in all genes in selected Gene ontology terms):
(In the same order as above)
80, 72, 66, 64, 63, 64, 59, 55, 52, 58, 56, 49, 51, 46, 43

Corresponding number of genes (of selected Gene ontology terms) each TF is involved with:
(In the same order as above)
38, 34, 27, 28, 29, 29, 22, 24, 24, 27, 26, 20, 23, 21, 22

Corresponding number of selected Gene ontology terms each TF is involved with:
(In the same order as above)
6, 6, 6, 6, 6, 6, 6, 6, 6, 6, 6, 6, 6, 6, 6

Cluster for columns 1132 to 1135, rows 423 to 426

Gene ontology terms

transcription factor activity | termination of RNA polymerase III transcription | transcription initiation | acetyltransferase activity

TFs

V$E2F_Q6_01 | V$E2F_Q4_01 | V$E2F_Q3_01 | V$E2F1_Q4_01

Information

All related TFs:
(List of all TFs that are related to any of the PWMs)
DP-1, E2F, E2F+E4, E2F-1, E2F-1:DP-1, E2F-3a, E2F-4, E2F-7

Ranked gene list:
(All genes of the selected Gene ontology terms with hits of any of the selected TFs, ranked according to the total number of TFBS)
ZFP36L1, JUN, RBL1, PELP1, JUND, HIST1H4I, GTF2A1, ETV4, CDKN1A, ZFP36L2, GTF2F1, UBTF, TP73, TP53, TCF3, SMC3, SLC20A1, RPS15, RPS10, RPL8, PCNA, NFATC1, NASP, NARG1, MYB, MDM2, MAZ, MARCKSL1, MAF1, LRBA, IRF1, HOXA9, HIST4H4, FASN, E2F3, E2F1, DRAP1, CUX1, CREBZF, CNOT1, CKB, CBX4, CAD, ATP5B, AKT1, TRRAP, TFAP2A, TCF7, SP1, SHMT2, PURA, PPP1R8, POLRMT, NOL3, LBX1, KLF13, JUNB, FOSB, CTDP1, CLPX, ATF3, TRIM26, TEAD1, TAF4, SUPT3H, PCSK4, MORF4L1, ISCU, DHFR, COBRA1, CDK8, ACTB, ZEB1,

SMARCA4, NFIC, ABHD14B

Corresponding total number of TFBS:
(For each gene listed above, the total number of TFBS for any of the selected TFs, multiplied by the number of selected Gene ontology terms containing that gene)
12, 12, 8, 8, 8, 8, 8, 8, 8, 6, 6, 4, 4, 4, 4, 4, 4, 4, 4, 4, 4, 4, 4, 4, 4, 4, 4, 4, 4, 4, 4, 4, 4, 4, 4, 4, 4, 4, 4, 4, 4, 4, 4, 4, 3, 3, 3, 3, 3, 3, 3, 3, 3, 3, 3, 3, 3, 3, 3, 2, 2, 2, 2, 2, 2, 2, 2, 2, 2, 2, 1, 1, 1, 1

Corresponding number of selected Gene ontology terms each gene is involved with:
(In the same order as above)
3, 3, 2, 2, 2, 2, 2, 2, 2, 2, 2, 1, 1, 2, 2, 1, 1, 1, 1, 1, 1, 1, 1, 1, 1, 1, 1, 1, 1, 1, 1, 1, 1, 2, 1, 1, 2, 1, 1, 1, 1, 1, 1, 1, 1, 1, 1, 1, 1, 3, 1, 1, 1, 1, 1, 1, 1, 1, 1, 1, 1, 1, 1, 2, 1, 1, 1, 1, 1, 1, 1, 1, 1, 1, 1, 1

Corresponding number of selected TFs each gene is involved with:
(In the same order as above)
4, 4, 4, 4, 4, 4, 4, 4, 4, 3, 3, 4, 4, 2, 2, 4, 4, 4, 4, 4, 4, 4, 4, 4, 4, 4, 4, 4, 4, 4, 4, 4, 4, 2, 4, 4, 2, 4, 4, 4, 4, 4, 4, 4, 4, 4, 3, 3, 3, 1, 3, 3, 3, 3, 3, 3, 3, 3, 3, 3, 3, 3, 2, 1, 2, 2, 2, 2, 2, 2, 2, 2, 2, 1, 1, 1, 1

Gene ontology terms ranked according to mean of effect sizes in cluster:
transcription initiation | termination of RNA polymerase III transcription | transcription factor activity | acetyltransferase activity

Corresponding mean of effect sizes of each Gene ontology term:
(In the same order as above)
2.63, 2.41, 2.29, 1.48

Corresponding total number of genes of each Gene ontology term:
(In the same order as above)
407, 52, 83, 112

Corresponding number of genes of each Gene ontology term with at least one TFBS (of selected TFs):
(In the same order as above)
56, 8, 18, 14

Corresponding total number of TFBS (of selected TFs) for each Gene ontology term (in all genes):
(In the same order as above)
173, 29, 57, 43

Corresponding number of selected TFs each Gene ontology term is involved with:
(In the same order as above)
4, 4, 4, 4

TFs ranked according to mean of effect sizes in cluster:
V$E2F1_Q4_01, V$E2F_Q3_01, V$E2F_Q4_01, V$E2F_Q6_01

Corresponding mean of effect sizes of each TF:
(In the same order as above)
2.52, 2.47, 2.35, 1.47

Corresponding total number of TFBS for each TF (genome-wide):
(In the same order as above)
681, 690, 667, 686

Corresponding total number of TFBS for each TF (in all genes in selected Gene ontology terms):
(In the same order as above)

81, 81, 74, 66

Corresponding number of genes (of selected Gene ontology terms) each TF is involved with:
(In the same order as above)
65, 65, 60, 51

Corresponding number of selected Gene ontology terms each TF is involved with:
(In the same order as above)
4, 4, 4, 4

Cluster for columns 1225 to 1251, rows 384 to 387

Gene ontology terms

blood coagulation | high molecular weight kininogen binding | generation of a signal involved in cell-cell signaling | phospholipid binding | ribonuclease activity | carbohydrate binding | pore complex biogenesis | gamma-glutamyltransferase activity | amino acid catabolic process | amino acid metabolic process | arginine biosynthetic process | urea cycle | fibronectin binding | insemination | parturition | progesterone secretion | receptor binding | receptor biosynthetic process | cortisol biosynthetic process | drinking behavior | cation transport | amiloride-sensitive sodium channel activity | sodium ion transport | water homeostasis | water transport | quorum sensing | transcription, RNA-dependent

TFs

V$HNF1_Q6_01 | V$HNF1_Q6 | V$HNF1_01 | V$HNF1_C

Information

All related TFs:
(List of all TFs that are related to any of the PWMs)
HNF-1alpha, HNF-1alpha-A, HNF-1alpha-B, HNF-1alpha-C, HNF-1beta, HNF-1beta-A, HNF-1beta-B, HNF-1beta-C

Ranked gene list:
(All genes of the selected Gene ontology terms with hits of any of the selected TFs, ranked according to the total number of TFBS)
RERE, ALB, ABL2, NR3C1, MBNL1, IGFBP1, ATM, SLC35A1, FN1, TRA@, AGT, SLC5A1, PRLR, HSPD1, FGA, DPYD, BCL2, AQP5, AQP2, RUNX2, PTGER4, LRPPRC, IFNG, GLB1, CLCN5, CAP1, AQP7, SLC12A1, PROC, PLB1, PLA2G4A, ESR1, UGT1A6, TGFBR2, TBXAS1, SS18, RUNX1, RIPK4, PIK3R1, PGR, NR3C2, MRPL39, LAD1, HSPE1, GRM3, G6PC, FGFR4, ETS1, CYP2E1, CXCR4, CSH1, CP, C1orf116, APOH, ANXA11, ANTXR2, WWP1, WNK1, VIM, UGT1A1, TRIM5, SRC, SERPINA4, PTPRC, PPIG, PLXNA2, PDE4DIP, OTC, KCNJ1, IGFBP3, GNRHR, FBXW11, EGR1, DR1, CSH2, COMMD3, BTD, ANPEP, AKR1C3, AFM, TRDN, TGFBR3, TGFA, TBK1, SGK1, SERPINA10, SERPINA1, SELL, SCAMP2, PVRL1, NRBP1, NR1I2, MSH3, MGP, LBR, GUCA2B, GRK5, GFM1, FLJ32987, FCGRT, F5, F2RL1, DHRS2, CTNNB1, CSF1, CS, CNTF, CD44, BAX, ATP5E, ATP2B1, ASL, ARID4B, ANXA1, ANGPT1, A2M, UHMK1, TRAF3, TNFSF18, TLR3, TLR10, THBS1, TG, SSFA2, SNRPB, SMAD3, SLCO3A1, SLCO1B1, SLC25A13, SLC25A12, SLC22A5, SLC22A2, SLC20A1, SLC1A4, SERPINH1, SERPINC1, SDHA, RPL7, RARS, PTTG1, PTK2B, POU2F1, PLA2G7, PLA2G6, PAG1, ODZ1, NR2F2, NQO1, NARS, MYC, MCF2, MAN2A1, MAN1A1, LNPEP, LCT, JAK2, JAK1, ITGB1, ITGA5, IGFBP4, HOXB2, HIF1A, HGFAC, GZMB, GSR, GCDH, FGL2, FAM126A, FABP12, ETV1, ERN1, ERBB2, DUT, DNAJB9, DMD, DDX5, CYSLTR1, CPNE1, CPB2, CDA, CCND3, CCL8, CASP3, BMP4, B2M, ARHGAP24, ANXA2, ALCAM, AHR, AFP, ADH5, ACY1, ACVR2A

Corresponding total number of TFBS:
(For each gene listed above, the total number of TFBS for any of the selected TFs, multiplied by the number of selected Gene ontology terms containing that gene)
48, 27, 26, 21, 12, 12, 12, 10, 10, 9, 9, 8, 8, 8, 8, 8, 8, 8, 8, 6, 6, 6, 6, 6, 6, 6, 6, 5, 5, 5, 5, 5, 4, 4, 4, 4, 4, 4, 4, 4, 4, 4, 4, 4, 4, 4, 4, 4, 4, 4, 4, 4, 4, 4, 4, 4, 4, 4, 3, 3, 3, 3, 3, 3, 3, 3, 3, 3, 3, 3, 3, 3, 3, 3, 3, 3, 3, 3, 3, 3, 3, 3, 2, 2, 2, 2, 2, 2, 2, 2, 2, 2, 2, 2, 2, 2, 2, 2, 2, 2, 2, 2, 2, 2, 2, 2, 2, 2, 2, 2, 2, 2, 2, 2, 2, 2, 1, 1, 1, 1, 1, 1, 1, 1, 1, 1, 1, 1, 1, 1, 1, 1, 1, 1, 1, 1, 1, 1, 1, 1, 1, 1, 1, 1, 1, 1, 1, 1, 1, 1, 1, 1, 1, 1, 1, 1, 1, 1, 1, 1, 1, 1, 1, 1, 1, 1, 1, 1, 1, 1, 1, 1, 1, 1, 1, 1, 1, 1, 1, 1, 1, 1, 1, 1, 1, 1, 1, 1

Corresponding number of selected Gene ontology terms each gene is involved with:
(In the same order as above)
12, 9, 13, 7, 3, 3, 6, 10, 5, 3, 9, 2, 4, 2, 2, 4, 2, 4, 4, 3, 3, 2, 2, 2, 2, 2, 2, 5, 5, 5, 5, 5, 1, 1, 1, 2, 1, 1, 1, 4, 4, 1, 1, 1, 2, 2, 1, 1, 2, 2, 4, 2, 1, 2, 1, 2, 1, 3, 1, 1, 1, 3, 1, 1, 1, 1, 1, 3, 3, 3, 3, 1, 1, 1, 3, 1, 1, 3, 1, 1, 1, 2, 2, 1, 2, 1, 1, 1, 1, 1, 2, 2, 2, 1, 2, 1, 2, 2, 2, 2, 2, 1, 1, 2, 1, 1, 1, 2, 1, 1, 2, 2, 1, 1, 2, 1, 1, 1, 1, 1, 1, 1, 1, 1, 1, 1, 1, 1, 1, 1, 1, 1, 1, 1, 1, 1, 1, 1, 1, 1, 1, 1, 1, 1, 1, 1, 1, 1, 1, 1, 1, 1, 1, 1, 1, 1, 1, 1, 1, 1, 1, 1, 1, 1, 1, 1, 1, 1, 1, 1, 1, 1, 1, 1, 1, 1, 1, 1, 1, 1, 1, 1, 1, 1, 1, 1, 1, 1, 1

Corresponding number of selected TFs each gene is involved with:
(In the same order as above)
4, 3, 2, 3, 4, 4, 2, 1, 2, 3, 1, 4, 2, 4, 4, 2, 4, 2, 2, 2, 2, 3, 3, 3, 3, 3, 3, 1, 1, 1, 1, 1, 4, 4, 4, 2, 4, 4, 4, 1, 1, 4, 4, 4, 2, 2, 4, 4, 2, 2, 1, 2, 4, 2, 4, 2, 3, 1, 3, 3, 3, 1, 3, 3, 3, 3, 3, 1, 1, 1, 1, 3, 3, 3, 1, 3, 3, 1, 3, 3, 2, 1, 1, 2, 1, 2, 2, 2, 2, 2, 2, 1, 1, 1, 2, 1, 2, 1, 1, 1, 1, 1, 2, 2, 1, 2, 2, 2, 1, 2, 2, 1, 1, 2, 2, 1, 1, 1, 1, 1, 1, 1, 1, 1, 1, 1, 1, 1, 1, 1, 1, 1, 1, 1, 1, 1, 1, 1, 1, 1, 1, 1, 1, 1, 1, 1, 1, 1, 1, 1, 1, 1, 1, 1, 1, 1, 1, 1, 1, 1, 1, 1, 1, 1, 1, 1, 1, 1, 1, 1, 1, 1, 1, 1, 1, 1, 1, 1, 1, 1, 1, 1, 1, 1, 1, 1, 1, 1, 1, 1, 1, 1, 1

Gene ontology terms ranked according to mean of effect sizes in cluster:
high molecular weight kininogen binding | blood coagulation | water transport | pore complex biogenesis | parturition | gamma-glutamyltransferase activity | amino acid metabolic process | drinking behavior | amino acid catabolic process | progesterone secretion | sodium ion transport | insemination | urea cycle | generation of a signal involved in cell-cell signaling | quorum sensing | transcription, RNA-dependent | amiloride-sensitive sodium channel activity | carbohydrate binding | ribonuclease activity | water homeostasis | phospholipid binding | arginine biosynthetic process | receptor binding | cation transport | cortisol biosynthetic process | receptor biosynthetic process | fibronectin binding

Corresponding mean of effect sizes of each Gene ontology term:
(In the same order as above)
4.58, 3.70, 2.82, 2.60, 2.40, 2.26, 2.26, 2.26, 2.13, 2.10, 2.09, 2.04, 1.99, 1.93, 1.89, 1.73, 1.65, 1.64, 1.46, 1.38, 1.33, 1.05, 0.74, 0.60, 0.41, 0.32, -0.01

Corresponding total number of genes of each Gene ontology term:
(In the same order as above)
48, 118, 63, 45, 102, 23, 50, 27, 30, 48, 54, 33, 48, 22, 34, 582, 70, 59, 23, 50, 90, 30, 417, 28, 25, 48, 30

Corresponding number of genes of each Gene ontology term with at least one TFBS (of selected TFs):
(In the same order as above)
11, 24, 13, 10, 18, 5, 10, 5, 6, 9, 13, 4, 9, 5, 6, 83, 16, 8, 4, 9, 19, 5, 47, 5, 2, 7, 5

Corresponding total number of TFBS (of selected TFs) for each Gene ontology term (in all genes):
(In the same order as above)
27, 40, 27, 22, 30, 8, 19, 10, 13, 15, 22, 14, 19, 10, 14, 147, 30, 18, 8, 14, 30, 9, 77, 6, 4, 11, 6

Corresponding number of selected TFs each Gene ontology term is involved with:
(In the same order as above)
4, 4, 4, 4, 4, 4, 4, 4, 4, 4, 4, 4, 4, 4, 4, 4, 4, 4, 4, 4, 4, 4, 4, 4, 3, 4, 4

TFs ranked according to mean of effect sizes in cluster:
V$HNF1_C, V$HNF1_Q6, V$HNF1_Q6_01, V$HNF1_01

Corresponding mean of effect sizes of each TF:
(In the same order as above)
2.41, 1.93, 1.68, 1.30

Corresponding total number of TFBS for each TF (genome-wide):
(In the same order as above)
651, 675, 679, 646

Corresponding total number of TFBS for each TF (in all genes in selected Gene ontology terms):
(In the same order as above)
180, 170, 164, 136

Corresponding number of genes (of selected Gene ontology terms) each TF is involved with:
(In the same order as above)
83, 88, 91, 77

Corresponding number of selected Gene ontology terms each TF is involved with:
(In the same order as above)
27, 27, 27, 26

Cluster for columns 514 to 536, rows 348 to 352

Gene ontology terms

mucosal immune response | granulocyte macrophage colony-stimulating factor biosynthetic process | granulocyte macrophage colony-stimulating factor production | interleukin-1 receptor activity | interleukin-6 production | tumor necrosis factor production | macrophage activation | inflammatory response | response to lipopolysaccharide | negative regulation of inflammatory response | regulation of tumor necrosis factor production | negative regulation of tumor necrosis factor production | positive regulation of tumor necrosis factor production | interleukin-1 receptor antagonist activity | acute inflammatory response | granuloma formation | lipopolysaccharide binding | cytokine biosynthetic process | interleukin-8 production | mRNA transcription | chronic inflammatory response | monocyte differentiation | hypersensitivity

TFs

V$STAT5B_01 | V$STAT5A_01 | V$STAT_01 | V$STAT3_01 | V$STAT1_01

Information

All related TFs:
(List of all TFs that are related to any of the PWMs)
STAT1alpha, STAT1beta, STAT2, STAT3, STAT4, STAT5A, STAT5B, STAT6

Ranked gene list:
(All genes of the selected Gene ontology terms with hits of any of the selected TFs, ranked according to the total number of TFBS)
ICAM1, CCL2, IL10, JUN, IL2RA, TNFRSF1A, CD14, SOCS3, EGR1, RELA, IRF1, TNF, FAS, CXCL10, LTA, IRF8, STAT3, AKT1, PTGER4, CD40LG, CCR1, S100A9, JUNB, CISH, IL2RB, CD69,

TLR5, NOD2, NOD1, HSPD1, GPR132, TNFRSF1B, CEL, SOCS1, SLC7A2, PRKCDBP, PIK3CG, OSM, NOS3, NOS1, NDUFA2, IGL@, ICAM2, FOS, IL6ST, IL11, H2AFX, FANCM, DPEP1, CD46, THPO, TBCA, TAF8, STAT5A, RUNX1, RNASE2, RAB27A, NR3C1, MUC1, MLANA, LMNA, ERBB2, CHUK, ZFP36, TNFSF11, TNFRSF6B, TCEA2, SCARB1, MAP3K8, LIG4, FKBP4, F2RL1, CCR7, VIPR2, VIP, TOP1, TCEAL1, SYNJ2BP, STAT5B, SOD2, SHMT1, SELPLG, REV3L, RBM47, PTGES, PIGR, NSFL1C, NEDD9, MMP2, MMP12, MEFV, MAP4K4, MAP2K4, ITIH4, ING1, IL23A, IFNGR1, HPD, GLTSCR2, GJB6, GABARAP, FOXP1, CXCR4, CXCL11, CLEC11A, CDKN1A, CDK5R1, CCR6, BRCA2, ATF3, ANTXR1, ACAA2, VWF, TWIST1, TPPP, TIPARP, SFTPB, SERPING1, RPS14, PROC, PRAP1, POU4F1, PIK3R2, NFKB2, NDUFAB1, MSMB, MRE11A, MCHR2, MARK2, MAP3K14, LTC4S, KCNA3, IRF2, IL19, HSP90AA1, HNRNPC, HMGB1, HLF, GPI, GAPDH, GALNT6, DDX5, CTF1, CREBBP, CORO7, CD63, CD19, ATP2C1, ARG1, ADM

Corresponding total number of TFBS:
(For each gene listed above, the total number of TFBS for any of the selected TFs, multiplied by the number of selected Gene ontology terms containing that gene)
105, 84, 69, 60, 44, 36, 36, 35, 28, 26, 25, 23, 18, 16, 15, 15, 14, 14, 12, 12, 12, 10, 10, 10, 9, 9, 8, 8, 8, 8, 8, 7, 7, 6, 6, 6, 6, 6, 6, 6, 6, 6, 6, 6, 5, 5, 5, 5, 5, 5, 4, 4, 4, 4, 4, 4, 4, 4, 4, 4, 4, 4, 4, 3, 3, 3, 3, 3, 3, 3, 3, 3, 3, 2, 2, 2, 2, 2, 2, 2, 2, 2, 2, 2, 2, 2, 2, 2, 2, 2, 2, 2, 2, 2, 2, 2, 2, 2, 2, 2, 2, 2, 2, 2, 2, 2, 2, 2, 2, 1, 1, 1, 1, 1, 1, 1, 1, 1, 1, 1, 1, 1, 1, 1, 1, 1, 1, 1, 1, 1, 1, 1, 1, 1, 1, 1, 1, 1, 1, 1, 1, 1, 1, 1, 1, 1, 1

Corresponding number of selected Gene ontology terms each gene is involved with:
(In the same order as above)
21, 21, 23, 15, 11, 12, 18, 7, 7, 13, 5, 23, 6, 8, 15, 5, 7, 7, 4, 12, 3, 5, 2, 2, 9, 9, 8, 8, 4, 4, 2, 7, 7, 3, 2, 3, 6, 3, 2, 3, 6, 3, 3, 6, 5, 5, 1, 1, 5, 1, 2, 4, 2, 2, 2, 2, 1, 4, 1, 2, 1, 2, 2, 1, 3, 1, 1, 3, 3, 1, 1, 3, 3, 1, 1, 1, 2, 1, 2, 1, 1, 1, 1, 1, 2, 1, 2, 2, 2, 2, 1, 1, 1, 2, 2, 2, 1, 2, 1, 2, 2, 1, 2, 1, 2, 2, 2, 1, 1, 2, 1, 1, 1, 1, 1, 1, 1, 1, 1, 1, 1, 1, 1, 1, 1, 1, 1, 1, 1, 1, 1, 1, 1, 1, 1, 1, 1, 1, 1, 1, 1, 1, 1, 1, 1, 1

Corresponding number of selected TFs each gene is involved with:
(In the same order as above)
5, 4, 3, 4, 4, 3, 2, 5, 4, 2, 5, 1, 3, 2, 1, 3, 2, 2, 3, 1, 4, 2, 5, 5, 1, 1, 1, 1, 2, 2, 4, 1, 1, 2, 3, 2, 1, 2, 3, 2, 1, 2, 2, 1, 1, 1, 5, 5, 1, 5, 2, 1, 2, 2, 2, 2, 4, 1, 4, 2, 4, 2, 2, 3, 1, 3, 3, 1, 1, 3, 3, 1, 1, 2, 2, 2, 1, 2, 1, 2, 2, 2, 2, 2, 1, 2, 1, 1, 1, 1, 2, 2, 2, 1, 1, 1, 2, 1, 2, 1, 1, 2, 1, 2, 1, 1, 1, 2, 2, 1, 2, 2, 1, 1, 1, 1, 1, 1, 1, 1, 1, 1, 1, 1, 1, 1, 1, 1, 1, 1, 1, 1, 1, 1, 1, 1, 1, 1, 1, 1, 1, 1, 1, 1, 1, 1, 1

Gene ontology terms ranked according to mean of effect sizes in cluster:
granulocyte macrophage colony-stimulating factor production | granulocyte macrophage colony-stimulating factor biosynthetic process | positive regulation of tumor necrosis factor production | chronic inflammatory response | macrophage activation | hypersensitivity | negative regulation of tumor necrosis factor production | mRNA transcription | granuloma formation | response to lipopolysaccharide | inflammatory response | regulation of tumor necrosis factor production | acute inflammatory response | mucosal immune response | interleukin-1 receptor antagonist activity | interleukin-1 receptor activity | interleukin-6 production | negative regulation of inflammatory response | lipopolysaccharide binding | tumor necrosis factor production | interleukin-8 production | cytokine biosynthetic process | monocyte differentiation

Corresponding mean of effect sizes of each Gene ontology term:
(In the same order as above)
1.75, 1.72, 1.69, 1.66, 1.65, 1.61, 1.38, 1.28, 1.25, 1.22, 1.18, 1.16, 1.15, 1.14, 1.12, 0.93, 0.92, 0.86, 0.84, 0.81, 0.36, 0.29, 0.28

Corresponding total number of genes of each Gene ontology term:
(In the same order as above)
107, 109, 61, 22, 178, 234, 61, 130, 77, 226, 425, 86, 68, 52, 70, 128, 165, 40, 61, 209, 144, 108, 102

Corresponding number of genes of each Gene ontology term with at least one TFBS (of selected TFs):

(In the same order as above)
22, 22, 13, 6, 34, 39, 12, 31, 13, 46, 70, 15, 9, 10, 12, 21, 33, 7, 10, 37, 20, 15, 13

Corresponding total number of TFBS (of selected TFs) for each Gene ontology term (in all genes):
(In the same order as above)
48, 48, 34, 16, 77, 79, 31, 67, 27, 96, 136, 39, 23, 20, 30, 45, 64, 18, 18, 72, 43, 36, 28

Corresponding number of selected TFs each Gene ontology term is involved with:
(In the same order as above)
5, 5, 5, 5, 5, 5, 5, 5, 5, 5, 5, 5, 5, 5, 5, 5, 5, 5, 5, 5, 5, 5, 5

TFs ranked according to mean of effect sizes in cluster:
V$STAT3_01, V$STAT5B_01, V$STAT1_01, V$STAT_01, V$STAT5A_01

Corresponding mean of effect sizes of each TF:
(In the same order as above)
1.38, 1.34, 1.16, 0.94, 0.88

Corresponding total number of TFBS for each TF (genome-wide):
(In the same order as above)
609, 642, 647, 645, 678

Corresponding total number of TFBS for each TF (in all genes in selected Gene ontology terms):
(In the same order as above)
205, 237, 212, 223, 218

Corresponding number of genes (of selected Gene ontology terms) each TF is involved with:
(In the same order as above)
51, 59, 56, 52, 58

Corresponding number of selected Gene ontology terms each TF is involved with:
(In the same order as above)
23, 23, 23, 23, 23

Cluster for columns 365 to 370, rows 27 to 32

Gene ontology terms

Golgi membrane | Golgi stack | Golgi apparatus | trans-Golgi network | endoplasmic reticulum | protein transport

TFs

V$GABP_B | V$ELK1_02 | V$TAXCREB_01 | V$ATF6_01 | V$HLF_01 | V$E4BP4_01

Information

All related TFs:
(List of all TFs that are related to any of the PWMs)
ATF6, CREB, E4BP4, Elk-1, Elk-1-isoform1, GABP-alpha, GABP-alpha:GABP-beta, GABP-beta1, GABP-beta2, Hlf, Tax, deltaCREB

Ranked gene list:

(All genes of the selected Gene ontology terms with hits of any of the selected TFs, ranked according to the total number of TFBS)
STX5, ARF1, UBB, GOLGA5, LMAN1, ING1, CDK5R1, ARHGAP4, ARF4, RPS27A, RAB6A, GOLGB1, GOLGA3, SCFD1, RER1, RAB4A, MGAT1, GOLGA1, GABARAPL2, COPG, COPE, TPT1, TNFSF13, SEC62, PER1, MTG1, JUN, GABARAP, ENOPH1, COPB1, ATAD2, UBC, UBA52, TG, SAR1A, RAB2A, NRSN1, IQGAP1, VPS53, VIM, VAMP3, VAMP2, TFRC, SLC30A7, NFE2L2, NEU1, MBTPS1, LAMP2, KDELR1, INSIG2, GOSR2, GCS1, GBF1, FLJ21865, DPM1, CHPF, CALR, ATF6, ARFGEF2, ARF6, APP, ACP2, ACHE, ABL2, WASL, VPS45, TMEM201, SEC24A, RPN1, RABGEF1, RAB34, PRNP, POFUT2, PCSK7, NPC1, NCSTN, LMAN2, LDLR, GTF3A, GPSN2, GORASP1, GBA2, FANCD2, DDIT3, CYB5R4, CTBP1, CSNK1A1, CPE, COPB2, CLTC, CANX, ATF4, ZFYVE9, YIF1A, VPS29, VAPB, VAMP4, UFD1L, UBE2J1, TXNDC12, TRIM3, TIMM44, TFAP2A, STMN3, SRPR, SRP54, SRP19, SREBF1, SNAP25, SLC31A1, SLC30A3, SLC27A5, SERP1, SEC31A, SEC23A, SCAMP1, RPS19, RPN2, RERE, RASGRP1, RANBP1, RAB11A, PPIG, POR, POFUT1, PMM1, PLEKHA8, PIK3C3, PIGW, PFN1, P4HB, OSBP, NMNAT1, NDOR1, MDM4, MARCH6, MAP4, LNPEP, KTN1, KIF3C, ITPR2, IKBKAP, HYOU1, HSP90AA1, HAT1, GPAA1, GGA2, GCC1, GBA, GABARAPL1, FKBP2, FAM168B, EXOC3, ERF, ERC1, EIF2A, EDC4, DPM3, DPAGT1, DNAJB11, DNAJB1, DHRS11, CYTIP, CXCL17, CSNK2B, CORO7, CLGN, CDC42, CASP9, CALM1, C1QBP, BNIP1, BET1L, B3GALTL, B3GALT4, ATP7A, ATG7, ATG5, ARID2, AP2B1, AP1S1, AP1M1, AKT1, AHCYL1, ABCB9, WBP1, VPS54, VKORC1, VCP, USP3, UBXN11, TXNDC5, TSG101, TRIM21, TRIB3, TPX2, TPP2, TMED2, TLR9, TAPBP, SYVN1, STX10, STMN1, STK24, STIM1, ST3GAL5, SREBF2, SPTLC2, SORL1, SLC39A7, SLC30A5, SLC2A4RG, SLC25A1, SERPINH1, SEPS1, SEC61G, SEC24D, SEC24C, SEC16A, SEC11A, SCP2, SC4MOL, SASH3, RTN4, RFT1, RABGAP1, RAB43, RAB21, PTDSS2, PSMB8, PSENEN, PRKCZ, PLEKHF1, PJA2, PITPNM1, PIGC, PIGB, PEX16, PAK4, OSBPL2, ORMDL3, ORAI2, ORAI1, OPTN, NDFIP1, NDEL1, NAPA, MT1B, MON2, MINPP1, MICAL1, MCFD2, MBIP, MAP1LC3A, MAN2B1, LCLAT1, KDELR2, KCNC1, ITPR3, IL6R, IL27, IFRD1, ICA1, HSF4, HRAS, HPS1, HIP1R, HERPUD1, HCST, HAX1, HAVCR1, GYLTL1B, GUK1, GSK3B, GRIN1, GPER, GPAM, GOLPH3, GGA3, GAK, FKRP, FKBP14, FGFR3, FASN, FAP, EXOC1, ERP29, EPOR, EIF4E, EGFL7, EDEM2, DPM2, DNAJC10, DNAJB9, DNAJB2, DDOST, DCTN2, DBNL, DBN1, CYCS, CSGALNACT2, CPD, CP, COPA, COG8, COG3, CLINT1, CHERP, CGB, CERK, CD81, CD47, CD4, CBLN3, CASP7, CASP3, CABP1, BSCL2, BFAR, BAT2, B4GALT6, ATF3, ASAP1, ARL6IP5, ARG2, ARFGAP3, APBA3, AP2S1, ALG8, ALG5, ALG13, ALG1, ALDH3A2, AFTPH, ADAMTS3

Corresponding total number of TFBS:
(For each gene listed above, the total number of TFBS for any of the selected TFs, multiplied by the number of selected Gene ontology terms containing that gene)
18, 18, 15, 15, 12, 12, 12, 12, 12, 10, 10, 10, 10, 9, 8, 8, 8, 8, 8, 8, 8, 6, 6, 6, 6, 6, 6, 6, 6, 6, 5, 5, 5, 5, 5, 5, 5, 4, 4, 4, 4, 4, 4, 4, 4, 4, 4, 4, 4, 4, 4, 4, 4, 4, 4, 4, 4, 4, 4, 4, 4, 4, 4, 3, 3, 3, 3, 3, 3, 3, 3, 3, 3, 3, 3, 3, 3, 3, 3, 3, 3, 3, 3, 3, 3, 3, 3, 3, 3, 3, 3, 2, 2, 2, 2, 2, 2, 2, 2, 2, 2, 2, 2, 2, 2, 2, 2, 2, 2, 2, 2, 2, 2, 2, 2, 2, 2, 2, 2, 2, 2, 2, 2, 2, 2, 2, 2, 2, 2, 2, 2, 2, 2, 2, 2, 2, 2, 2, 2, 2, 2, 2, 2, 2, 2, 2, 2, 2, 2, 2, 2, 2, 2, 2, 2, 2, 2, 2, 2, 2, 2, 2, 2, 2, 2, 2, 2, 2, 2, 2, 2, 2, 2, 2, 2, 2, 2, 2, 2, 2, 2, 2, 2, 2, 1

Corresponding number of selected Gene ontology terms each gene is involved with:
(In the same order as above)
6, 6, 5, 5, 6, 3, 3, 6, 4, 5, 5, 5, 5, 3, 4, 4, 4, 4, 4, 4, 4, 3, 3, 2, 1, 6, 2, 2, 3, 6, 3, 5, 5, 5, 5, 5, 5, 5, 2, 2, 2, 2, 4, 2, 1, 4, 2, 4, 4, 2, 4, 4, 4, 4, 1, 4, 4, 2, 2, 4, 4, 2, 2, 2, 3, 3, 3, 1, 3, 1, 3, 1, 1, 1, 3, 3, 3, 3, 1, 3, 3, 1, 1, 1, 1, 1, 1, 3, 3, 3, 3, 1, 1, 2, 2, 2, 2, 1, 1, 1, 2, 1, 2, 1, 2, 2, 1, 1, 2, 1, 1, 1, 1, 1, 2, 2, 1, 2, 2, 1, 1, 2, 1, 1, 1, 2, 2, 2, 1, 1, 1, 2, 1, 1, 1, 1, 1, 2, 1, 1, 1, 2, 2, 2, 1, 1, 2, 2, 2, 2, 1, 1, 1, 1, 1, 1, 2, 1, 1, 1, 2, 1, 1, 1, 1, 1, 2, 2, 1, 1, 1, 2, 2, 2, 1, 2, 1, 2, 1, 2, 2, 1

Corresponding number of selected TFs each gene is involved with:
(In the same order as above)
3, 3, 3, 3, 2, 4, 4, 2, 3, 2, 2, 2, 2, 3, 2, 2, 2, 2, 2, 2, 2, 2, 3, 6, 1, 3, 3, 2, 1, 2, 1, 1, 1, 1, 1, 1, 1, 2, 2, 2, 2, 1, 2, 4, 1, 2, 1, 1, 2, 1, 1, 1, 1, 4, 1, 1, 2, 2, 1, 1, 2, 2, 2, 1, 1, 1, 3, 1, 3, 1, 3, 3, 3, 1, 1, 1, 1, 3, 1, 1, 3, 3, 3, 3, 3, 3, 1, 1, 1, 1, 3, 2, 1, 1, 1, 1, 2, 2, 2, 1, 2, 1, 2, 1, 1, 2, 2, 1, 2, 2, 2, 2, 2, 1, 1, 2, 1, 1, 2, 2, 1, 2, 2, 2, 1, 1, 1, 2, 2, 2, 1, 2, 2, 2, 2, 2, 1, 2, 2, 2, 1, 1, 1, 2, 2, 1, 1, 1, 1, 2, 2, 2, 2, 2, 1, 2, 2, 2, 1, 2, 2, 2, 2, 2, 1, 1, 2, 2, 2, 1, 1, 1, 2, 1, 2, 1, 2, 1, 1, 2, 2, 2, 2, 1

Gene ontology terms ranked according to mean of effect sizes in cluster:
endoplasmic reticulum | protein transport | Golgi membrane | Golgi apparatus | Golgi stack | trans-Golgi network

Corresponding mean of effect sizes of each Gene ontology term:
(In the same order as above)
2.96, 2.88, 2.61, 2.42, 1.96, 1.90

Corresponding total number of genes of each Gene ontology term:
(In the same order as above)
771, 146, 93, 482, 100, 300

Corresponding number of genes of each Gene ontology term with at least one TFBS (of selected TFs):
(In the same order as above)
227, 54, 31, 139, 37, 85

Corresponding total number of TFBS (of selected TFs) for each Gene ontology term (in all genes):
(In the same order as above)
337, 79, 56, 201, 58, 119

Corresponding number of selected TFs each Gene ontology term is involved with:
(In the same order as above)
6, 6, 6, 6, 6, 6

TFs ranked according to mean of effect sizes in cluster:
V$ATF6_01, V$ELK1_02, V$E4BP4_01, V$GABP_B, V$TAXCREB_01, V$HLF_01

Corresponding mean of effect sizes of each TF:
(In the same order as above)
4.37, 3.14, 2.38, 1.92, 1.78, 1.13

Corresponding total number of TFBS for each TF (genome-wide):
(In the same order as above)
670, 627, 635, 625, 674, 657

Corresponding total number of TFBS for each TF (in all genes in selected Gene ontology terms):
(In the same order as above)
194, 138, 143, 117, 138, 120

Corresponding number of genes (of selected Gene ontology terms) each TF is involved with:
(In the same order as above)
99, 79, 77, 74, 82, 70

Corresponding number of selected Gene ontology terms each TF is involved with:
(In the same order as above)
6, 6, 6, 6, 6, 6

Cluster for columns 718 to 727, rows 397 to 399

Gene ontology terms

progesterone biosynthetic process | luteinization | 3beta-hydroxysteroid dehydrogenase activity | regulation of steroid biosynthetic process | steroid biosynthetic process | hormone biosynthetic process | estrogen biosynthetic process | unspecific monooxygenase activity | androgen biosynthetic process | steroid metabolic process

TFs

V$MEF2_02 | V$RSRFC4_Q2 | V$RSRFC4_01

Information

All related TFs:
(List of all TFs that are related to any of the PWMs)
MEF-2A, RSRFC4, aMEF-2

Ranked gene list:
(All genes of the selected Gene ontology terms with hits of any of the selected TFs, ranked according to the total number of TFBS)
NR5A1, PRL, PPIG, SCARB1, NR4A2, NR4A1, STAR, AHR, MTHFR, INS, NR0B1, GDF9, SF1, FST, SCN5A, PTP4A3, IGF2, ATM, TP63, STARD4, REN, POU1F1, PLAT, HSD11B1, DIO1, CREM, CREB1, ACVR2A, SULT2B1, SNF1LK2, SMARCA1, S100A6, KIAA1804, JUN, GADD45B, CDKN1B, CAMK2G, ARSG, AKR1B1, ACVR1

Corresponding total number of TFBS:
(For each gene listed above, the total number of TFBS for any of the selected TFs, multiplied by the number of selected Gene ontology terms containing that gene)
30, 27, 18, 12, 9, 9, 8, 8, 7, 7, 6, 6, 4, 4, 3, 3, 3, 3, 2, 2, 2, 2, 2, 2, 2, 2, 2, 2, 1, 1, 1, 1, 1, 1, 1, 1, 1, 1, 1, 1

Corresponding number of selected Gene ontology terms each gene is involved with:
(In the same order as above)
10, 9, 6, 4, 3, 3, 8, 4, 7, 7, 6, 3, 4, 4, 3, 1, 3, 3, 2, 1, 1, 1, 2, 1, 1, 1, 1, 2, 1, 1, 1, 1, 1, 1, 1, 1, 1, 1, 1, 1

Corresponding number of selected TFs each gene is involved with:
(In the same order as above)
3, 3, 3, 3, 3, 3, 1, 2, 1, 1, 1, 2, 1, 1, 1, 3, 1, 1, 1, 2, 2, 2, 1, 2, 2, 2, 2, 1, 1, 1, 1, 1, 1, 1, 1, 1, 1, 1, 1, 1

Gene ontology terms ranked according to mean of effect sizes in cluster:
progesterone biosynthetic process | hormone biosynthetic process | steroid metabolic process | androgen biosynthetic process | estrogen biosynthetic process | unspecific monooxygenase activity | steroid biosynthetic process | luteinization | 3beta-hydroxysteroid dehydrogenase activity | regulation of steroid biosynthetic process

Corresponding mean of effect sizes of each Gene ontology term:
(In the same order as above)
3.49, 3.11, 2.95, 2.81, 2.52, 2.19, 2.16, 2.15, 1.44, 1.30

Corresponding total number of genes of each Gene ontology term:
(In the same order as above)
42, 91, 51, 54, 48, 71, 213, 50, 68, 40

Corresponding number of genes of each Gene ontology term with at least one TFBS (of selected TFs):
(In the same order as above)
9, 12, 6, 9, 9, 11, 32, 11, 8, 6

Corresponding total number of TFBS (of selected TFs) for each Gene ontology term (in all genes):
(In the same order as above)
17, 23, 13, 17, 16, 18, 51, 18, 14, 12

Corresponding number of selected TFs each Gene ontology term is involved with:
(In the same order as above)
3, 3, 3, 3, 3, 3, 3, 3, 3, 3

TFs ranked according to mean of effect sizes in cluster:
V$RSRFC4_Q2, V$MEF2_02, V$RSRFC4_01

Corresponding mean of effect sizes of each TF:
(In the same order as above)
2.54, 2.52, 2.17

Corresponding total number of TFBS for each TF (genome-wide):
(In the same order as above)
655, 700, 655

Corresponding total number of TFBS for each TF (in all genes in selected Gene ontology terms):
(In the same order as above)
69, 73, 57

Corresponding number of genes (of selected Gene ontology terms) each TF is involved with:
(In the same order as above)
23, 22, 18

Corresponding number of selected Gene ontology terms each TF is involved with:
(In the same order as above)
10, 10, 10

Cluster for columns 874 to 879, rows 9 to 27

Gene ontology terms

cell morphogenesis | cell cortex | microtubule cytoskeleton | invasive growth in response to glucose limitation | cleavage furrow | nuclear migration

TFs

V$CREB_Q4_01 | V$CREB_Q2_01 | V$CREB_Q4 | V$CREBP1_Q2 | V$CREB_Q2 | V$CREB_01 | V$CREBP1CJUN_01 | V$CREBATF_Q6 | V$ATF_B | V$CREB_02 | V$ATF_01 | V$ATF3_Q6 | V$ATF1_Q6 | V$CREBP1_01 | V$YY1_Q6_02 | V$YY1_Q6 | V$NFMUE1_Q6 | V$YY1_02 | V$GABP_B

Information

All related TFs:
(List of all TFs that are related to any of the PWMs)
120-kDa, 47-kDa, ATF, ATF-1, ATF-2, ATF-4, ATF-a, ATF-like, ATF/CREB, ATF2, ATF2-isoform2, ATF3, ATF4, ATF5, ATF6, ATFa-isoform1, CRE-BP1, CRE-BP2, CREB, CREBbeta, CREMalpha, CREMbeta, CREMgamma, CREMtau, CREMtau1, CREMtau2, CREMtaualpha, GABP-alpha, GABP-alpha:GABP-beta, GABP-beta1, GABP-beta2, NF-muE1, YY1, c-Jun, deltaCREB, factor

Ranked gene list:
(All genes of the selected Gene ontology terms with hits of any of the selected TFs, ranked according to the total number of TFBS)
MAPRE2, CDC42, PAFAH1B1, AKT1, PAK1, FN1, PAX6, C14orf4, TRAP1, TPP1, SEMA3B, PARD6A, ILK, TUBGCP2, RAB11FIP3, DVL1, CNTNAP1, SEPT7, RAC1, IKBKAP, TUBB2A, PITPNM1, PHF1, MUC1, MARK2, IL32, VASP, PRC1, SEPT2, RNMT, CLASP2, CALM2, ACTR3, PRKCZ, PCNA, EPHA2, CD68, PCK1, HRAS, AURKA, ARHGEF2, APC, PLK4, NDEL1, MAPRE1, CD44, WDR48, RHOA, PFN1, PCNT, MTTP, MMP14, DCTN3, CALM1, C10orf27, ANAPC2, WASL, TUBA4B, TMEM201, STK24, RAB7A, MOAP1, GNB2L1, EZR, EPHB2, ACTR1A, WDR1, SLC25A23, RAX2, RAF1, PKN1, PIGG, MARCKS, MAP1B, GFM1, ETV5, CTNNB1, CD248, AXIN1, AURKB, ARHGAP1, TUBB, TPK1, TP53, SEPT9, RPS6KA3, PPT1, PCYOX1, NUMA1, NCKAP1, MIA, MGAM, MAPT, MAPRE3, MAP4, MAP3K11, KIAA1804, KDELR1, ITPR3, IQGAP1, HIRA, HGFAC, GSK3B, GPSM2, EGLN2, DYNC1H1, CYFIP2, CTSD, COIL, CEP120, ACTR2, ACTB, ABL2, ABI2

Corresponding total number of TFBS:
(For each gene listed above, the total number of TFBS for any of the selected TFs, multiplied by the number of selected Gene ontology terms containing that gene)
60, 60, 45, 30, 28, 26, 16, 15, 14, 14, 14, 14, 14, 13, 13, 13, 13, 12, 12, 12, 11, 11, 11, 11, 10, 10, 9, 9, 8, 8, 8, 8, 8, 7, 7, 7, 7, 6, 6, 6, 6, 6, 5, 5, 5, 5, 4, 4, 4, 4, 4, 4, 4, 4, 4, 4, 3, 3, 3, 3, 3, 3, 3, 3, 3, 3, 2, 2, 2, 2, 2, 2, 2, 2, 2, 2, 2, 2, 2, 2, 2, 1, 1, 1, 1, 1, 1, 1, 1, 1, 1, 1, 1, 1, 1, 1, 1, 1, 1, 1, 1, 1, 1, 1, 1, 1, 1, 1, 1, 1, 1, 1, 1

Corresponding number of selected Gene ontology terms each gene is involved with:
(In the same order as above)
5, 6, 3, 3, 2, 2, 1, 1, 1, 1, 1, 1, 1, 1, 1, 1, 1, 4, 4, 3, 1, 1, 1, 1, 2, 1, 1, 1, 1, 1, 1, 2, 2, 1, 1, 1, 1, 1, 1, 2, 2, 2, 1, 1, 5, 1, 1, 4, 1, 1, 1, 1, 1, 2, 4, 1, 1, 1, 1, 3, 1, 1, 1, 3, 1, 3, 1, 1, 1, 1, 2, 1, 1, 1, 2, 2, 2, 1, 1, 1, 2, 1, 1, 1, 1, 1, 1, 1, 1, 1, 1, 1, 1, 1, 1, 1, 1, 1, 1, 1, 1, 1, 1, 1, 1, 1, 1, 1, 1, 1, 1, 1, 1

Corresponding number of selected TFs each gene is involved with:
(In the same order as above)
12, 10, 15, 10, 14, 13, 16, 15, 14, 14, 14, 14, 14, 13, 13, 13, 13, 3, 3, 4, 11, 11, 11, 11, 5, 10, 9, 9, 8, 8, 8, 4, 4, 7, 7, 7, 7, 6, 6, 3, 3, 3, 5, 5, 1, 5, 4, 1, 4, 4, 4, 4, 4, 2, 1, 4, 3, 3, 3, 1, 3, 3, 3, 1, 3, 1, 2, 2, 2, 2, 1, 2, 2, 2, 1, 1, 1, 2, 2, 2, 1, 1, 1, 1, 1, 1, 1, 1, 1, 1, 1, 1, 1, 1, 1, 1, 1, 1, 1, 1, 1, 1, 1, 1, 1, 1, 1, 1, 1, 1, 1, 1, 1

Gene ontology terms ranked according to mean of effect sizes in cluster:
cell cortex | microtubule cytoskeleton | nuclear migration | cell morphogenesis | invasive growth in response to glucose limitation | cleavage furrow

Corresponding mean of effect sizes of each Gene ontology term:
(In the same order as above)
1.71, 1.36, 1.27, 0.80, 0.76, 0.71

Corresponding total number of genes of each Gene ontology term:
(In the same order as above)
93, 63, 32, 83, 88, 68

Corresponding number of genes of each Gene ontology term with at least one TFBS (of selected

TFs):
(In the same order as above)
42, 27, 13, 32, 26, 25

Corresponding total number of TFBS (of selected TFs) for each Gene ontology term (in all genes):
(In the same order as above)
183, 126, 81, 139, 129, 113

Corresponding number of selected TFs each Gene ontology term is involved with:
(In the same order as above)
19, 19, 18, 19, 19, 19

TFs ranked according to mean of effect sizes in cluster:
V$CREB_Q2_01, V$CREB_Q4_01, V$ATF3_Q6, V$CREB_01, V$CREB_Q4, V$ATF1_Q6, V$CREBP1CJUN_01, V$ATF_B, V$CREBATF_Q6, V$GABP_B, V$CREBP1_01, V$CREB_Q2, V$CREB_02, V$ATF_01, V$CREBP1_Q2, V$YY1_Q6_02, V$NFMUE1_Q6, V$YY1_Q6, V$YY1_02

Corresponding mean of effect sizes of each TF:
(In the same order as above)
1.64, 1.53, 1.49, 1.48, 1.35, 1.35, 1.31, 1.30, 1.09, 1.09, 1.07, 1.01, 0.98, 0.87, 0.86, 0.76, 0.65, 0.58, 0.52

Corresponding total number of TFBS for each TF (genome-wide):
(In the same order as above)
679, 667, 663, 659, 671, 671, 652, 665, 671, 625, 662, 676, 656, 660, 679, 688, 658, 684, 639

Corresponding total number of TFBS for each TF (in all genes in selected Gene ontology terms):
(In the same order as above)
46, 44, 49, 45, 42, 44, 42, 43, 39, 36, 40, 41, 41, 38, 37, 38, 34, 35, 37

Corresponding number of genes (of selected Gene ontology terms) each TF is involved with:
(In the same order as above)
31, 31, 33, 30, 29, 28, 28, 28, 29, 22, 26, 31, 32, 28, 27, 20, 23, 23, 26

Corresponding number of selected Gene ontology terms each TF is involved with:
(In the same order as above)
6, 6, 6, 6, 6, 6, 6, 6, 6, 6, 6, 6, 6, 6, 6, 6, 6, 6, 5

Cluster for columns 293 to 307, rows 289 to 297

Gene ontology terms

anaerobic glycolysis | fermentation | glycerol-3-phosphate dehydrogenase activity | phosphoglycerate mutase activity | hexokinase activity | tricarboxylic acid cycle | glucose-6-phosphate dehydrogenase activity | pentose-phosphate shunt | lactate dehydrogenase activity | glucokinase activity | glucose catabolic process | glycolysis | gluconeogenesis | carbohydrate metabolic process | glucose metabolic process

TFs

V$SP1_01 | V$SP1_Q6 | V$SP1_Q4_01 | V$SP1_Q6_01 | V$KROX_Q6 | V$AHR_Q5 | V$AHRHIF_Q6 | V$HIF1_Q5 | V$HIF1_Q3

Information

All related TFs:
(List of all TFs that are related to any of the PWMs)
ARNT2, AhR, AhR2, AhR:Arnt, AhRR, AhRR:Arnt, Arnt, Egr-1, Egr-2, Egr-3, Egr-4, HIF-1, HIF-1alpha, HIF-1alpha-isoform1, Sp1, Sp2, Sp3, Sp4

Ranked gene list:
(All genes of the selected Gene ontology terms with hits of any of the selected TFs, ranked according to the total number of TFBS)
GPI, PER1, GAPDH, H6PD, TPI1, PFKL, FASN, UBL4A, TKT, PFKP, PCK2, ACHE, LDHA, GK, PPM2C, PFN2, IRS2, AKT1, VEGFA, PC, PFKFB2, MAP4K2, HBA2, KHK, HK1, GPD2, FOLR2, CYCS, LDHB, GFER, TNK2, PGP, IMMT, CPT1A, BNIP3, SLC2A1, SCO2, RAG1AP1, SLC16A3, HIF1AN, GLUD1, ADIPOR1, ACSS2, ACSL1, NOL3, GPT, DBP, VDAC1, UGDH, TXN, SLC2A3, PPARG, PFKFB3, PDK3, NOC2L, IGF1R, GNAI2, COL11A2, ALDOA, ACACA, RPL22, PKN1, NR4A3, NR4A1, LPHN1, LEPROT, LEPR, HOOK1, ENO1, DHX9, DGAT1, ADIPOR2, ZFYVE9, ZDHHC23, UCP2, TGFB1, SUFU, STK11, SOD2, SLC37A4, SLC16A1, SFI1, SCARB1, PTPRF, PODXL, PIK3CA, PGM2, PGAM1, PDPK1, ONECUT1, NR3C1, MTG1, MAPT, KCNJ11, HM13, HDAC4, HBA1, GNAS, BSG, A1BG, TIPARP, SEPHS1, RPE, PPP1R13B, PPAN, POMC, PDK1, PDF, NRF1, MUC1, MBP, LPIN1, LAP3, JPH3, INVS, HGS, GCLC, FXN, ENO2, DYNLRB1, DPP4, CSH2, CRTC2, COL25A1, CKB, CFD, CBFA2T2, CA11, ATF1, AQP3, AKT3, AKT2, ACY1

Corresponding total number of TFBS:
(For each gene listed above, the total number of TFBS for any of the selected TFs, multiplied by the number of selected Gene ontology terms containing that gene)
52, 48, 42, 40, 36, 35, 25, 21, 20, 18, 18, 18, 16, 16, 15, 15, 15, 15, 14, 14, 12, 12, 12, 10, 10, 10, 10, 10, 9, 9, 8, 8, 8, 8, 8, 7, 7, 7, 6, 6, 6, 6, 6, 6, 5, 5, 5, 4, 4, 4, 4, 4, 4, 4, 4, 4, 4, 4, 4, 4, 3, 3, 3, 3, 3, 3, 3, 3, 3, 3, 3, 3, 2, 2, 2, 2, 2, 2, 2, 2, 2, 2, 2, 2, 2, 2, 2, 2, 2, 2, 2, 2, 2, 2, 2, 2, 2, 2, 2, 2, 1, 1, 1, 1, 1, 1, 1, 1, 1, 1, 1, 1, 1, 1, 1, 1, 1, 1, 1, 1, 1, 1, 1, 1, 1, 1, 1, 1, 1, 1, 1, 1

Corresponding number of selected Gene ontology terms each gene is involved with:
(In the same order as above)
13, 6, 14, 10, 6, 7, 5, 5, 7, 10, 6, 9, 2, 8, 8, 5, 3, 3, 3, 2, 7, 4, 3, 2, 2, 5, 10, 5, 5, 3, 1, 4, 1, 8, 4, 2, 7, 1, 1, 1, 3, 6, 2, 2, 2, 1, 1, 1, 2, 2, 4, 4, 4, 4, 2, 1, 1, 2, 1, 2, 2, 1, 1, 1, 1, 1, 1, 1, 3, 1, 1, 1, 3, 1, 1, 2, 1, 1, 2, 2, 2, 2, 1, 1, 1, 2, 1, 2, 2, 1, 2, 1, 2, 1, 1, 1, 1, 2, 1, 1, 1, 1, 1, 1, 1, 1, 1, 1, 1, 1, 1, 1, 1, 1, 1, 1, 1, 1, 1, 1, 1, 1, 1, 1, 1, 1, 1, 1, 1, 1, 1, 1, 1

Corresponding number of selected TFs each gene is involved with:
(In the same order as above)
4, 8, 3, 4, 6, 5, 5, 3, 2, 3, 2, 9, 2, 2, 3, 5, 5, 5, 7, 2, 3, 4, 6, 5, 2, 1, 2, 2, 3, 9, 2, 8, 1, 2, 4, 1, 7, 7, 6, 2, 1, 3, 3, 3, 5, 5, 5, 2, 2, 1, 1, 1, 1, 2, 4, 4, 2, 4, 2, 2, 3, 3, 3, 3, 3, 3, 3, 1, 3, 3, 3, 1, 2, 2, 1, 2, 2, 1, 1, 1, 1, 2, 2, 2, 1, 2, 1, 1, 2, 1, 2, 1, 2, 2, 2, 2, 1, 2, 2, 2, 1, 1, 1, 1, 1, 1, 1, 1, 1, 1, 1, 1, 1, 1, 1, 1, 1, 1, 1, 1, 1, 1, 1, 1, 1, 1, 1, 1, 1, 1, 1, 1

Gene ontology terms ranked according to mean of effect sizes in cluster:
glycolysis | carbohydrate metabolic process | hexokinase activity | glucose metabolic process | phosphoglycerate mutase activity | tricarboxylic acid cycle | glucose-6-phosphate dehydrogenase activity | anaerobic glycolysis | glucose catabolic process | glucokinase activity | gluconeogenesis | pentose-phosphate shunt | lactate dehydrogenase activity | glycerol-3-phosphate dehydrogenase activity | fermentation

Corresponding mean of effect sizes of each Gene ontology term:
(In the same order as above)
1.70, 1.65, 1.63, 1.28, 1.10, 1.05, 1.03, 1.02, 0.96, 0.94, 0.80, 0.71, 0.58, 0.57, 0.38

Corresponding total number of genes of each Gene ontology term:

(In the same order as above)
200, 99, 40, 215, 77, 144, 67, 37, 39, 30, 144, 78, 33, 79, 137

Corresponding number of genes of each Gene ontology term with at least one TFBS (of selected TFs):
(In the same order as above)
51, 26, 14, 55, 18, 32, 14, 11, 10, 9, 36, 16, 9, 15, 24

Corresponding total number of TFBS (of selected TFs) for each Gene ontology term (in all genes):
(In the same order as above)
144, 70, 32, 136, 50, 72, 38, 26, 33, 31, 87, 41, 25, 38, 63

Corresponding number of selected TFs each Gene ontology term is involved with:
(In the same order as above)
9, 9, 9, 9, 9, 9, 9, 7, 9, 8, 9, 9, 9, 9, 9

TFs ranked according to mean of effect sizes in cluster:
V$HIF1_Q3, V$HIF1_Q5, V$SP1_Q4_01, V$AHR_Q5, V$SP1_Q6, V$SP1_Q6_01, V$AHRHIF_Q6, V$KROX_Q6, V$SP1_01

Corresponding mean of effect sizes of each TF:
(In the same order as above)
2.10, 1.51, 1.14, 1.09, 1.07, 1.05, 0.98, 0.34, -0.06

Corresponding total number of TFBS for each TF (genome-wide):
(In the same order as above)
644, 654, 656, 666, 664, 663, 671, 675, 696

Corresponding total number of TFBS for each TF (in all genes in selected Gene ontology terms):
(In the same order as above)
131, 117, 89, 111, 92, 93, 103, 79, 71

Corresponding number of genes (of selected Gene ontology terms) each TF is involved with:
(In the same order as above)
44, 35, 31, 42, 33, 32, 42, 30, 36

Corresponding number of selected Gene ontology terms each TF is involved with:
(In the same order as above)
15, 15, 15, 15, 15, 14, 15, 14, 14

Cluster for columns 391 to 399, rows 143 to 151

Gene ontology terms

actin binding | actomyosin | cell motility | establishment of cell polarity | leading edge | cytoskeleton | filamentous actin | actin cytoskeleton | actin filament polymerization

TFs

V$PEA3_Q6 | V$ETS_Q6 | V$PU1_Q6 | V$PXR_Q2 | V$TEL2_Q6 | V$ETS_Q4 | V$ELF1_Q6 | V$NKX25_Q5 | V$ETS2_B

Information

All related TFs:
(List of all TFs that are related to any of the PWMs)
CAR, CAR2:RXR-alpha, CAR:RXR-alpha, ELF-1, ELFR, ERF, Elf-1, Elk-1, Elk-1-isoform1, Erg-1, Erg-2, Ets-1, FXR, FXR:RXR-alpha, Fli-1, GABP-alpha, GABP-alpha:GABP-beta, GABP-beta1, GABP-beta2, LXR-alpha:RXR-alpha, LXR-beta:RXR-alpha, NERF, NERF-1a, NERF-1b, NERF-2, Net, Nkx2-5, Nkx2.5, PEA3, PU.1, PU.1-xbb1, PXR-1, PXR-1A, PXR-2, PXR:RXR-alpha, SAP-1a, SAP-1b, Spi-B, TCF, TEL1, Tel-2a, Tel-2b, Tel-2c, Tel-2d, Tel-2e, Tel-2f, c-Ets-1, c-Ets-1A, c-Ets-1B, c-Ets-2, c-Ets-2A, c-Ets-2B, p38erg, p49erg, p55, p55erg

Ranked gene list:
(All genes of the selected Gene ontology terms with hits of any of the selected TFs, ranked according to the total number of TFBS)
AKT1, ARHGEF2, PFN1, RAC2, ITGB2, RHOG, ZYX, PKN1, LASP1, ILK, ACTB, VASP, SLC9A3R1, SLC2A4RG, PLCG1, LCK, CDK5, ARHGAP1, VAV1, RASSF5, MARK2, FGR, CSK, MYO1C, TNF, SHC1, MMP9, LCP1, GIT1, RHOC, PTK2B, PPP1R9B, LSP1, DLG4, DBNL, CYFIP2, ACTN1, SRF, PTPN6, PSTPIP1, LCP2, FHOD1, CXCR4, CTDSP1, CORO1A, CAPZA1, AHNAK, PDLIM2, CRIP1, CORO1B, CCR7, RALB, WIPF1, TNFSF13, S100A4, PIK3CG, CFL1, CALM2, VAV2, PIK3R1, ITGA5, FERMT3, EPB49, CRTC2, CD82, CAP1, ARHGDIA, ANXA6, UNC84B, ITPR3, GAPDH, FYN, DIAPH1, CSF1, ARFIP2, ACTA2, WDR1, RHOA, PXN, PLEC1, MAP3K11, MAP3K1, LTBR, CAMK2G, ARHGEF1, ZAP70, VILL, TNK2, TNFRSF1A, SSSCA1, SORBS3, S1PR2, PER1, NFATC1, MAP3K12, EVL, DGKZ, CTTN, CEBPE, CALR, TUBA4A, TLN1, MYL6, MTG1, FLNA, CD44, ACTR3, VIPR1, TYROBP, TPP1, STK24, SMTN, SLC9A5, RIN1, RAP1GAP, PTPN11, PIK3R2, PFDN5, PELP1, MAPK14, JUN, ITGB1, ICAM3, EPHA1, ELMO1, EGR1, EFNA1, DKFZp779C0757, CRK, CNTNAP1, ARPC1B, ARHGEF7, ACTR2, WASF2, WASF1, VIM, SPN, SKAP1, RAB3GAP1, PRKCD, NCK1, MYO1F, MYH9, MAP4K1, LYN, INPP5D, GRB2, EPB41, EDC4, DVL2, CRIP2, CLIP1, CD63, C3orf58, ACTG1, ZBP1, STOML2, SLC25A1, SEMA3B, SELL, S1PR4, RND2, RALGDS, RAC3, PSMP, PRKCA, PPP1R12A, PLEKHO2, MTHFR, MMP14, MAPRE2, LMNA, IRS1, HCLS1, FLII, FBXW7, F11R, ERBB2, DOCK2, DLGAP1, CD4, CD248, ARHGAP4, ABI1, ZC3H12D, VAV3, TBX21, SHANK3, S100A11, RHOH, RALA, RAB13, PKN2, PARD6A, NFKBIA, NCF4, MICAL1, MGC138368, ITGA4, IGSF8, GUK1, GLI1, ERBB2IP, ELMO3, DEF6, DAPP1, CLCF1, CDKN1B, CD97, CD2BP2, CAPG, ARHGEF12, APC, AKT2, p63RhoGEF, VEGFA, TRIP10, TRAF3IP2, TNNT1, TNNI3, TNNI2, TNIK, TMOD3, TM7SF2, SYN2, STAT5A, SQSTM1, SLC9A3R2, RAPGEF3, RAP1B, RAF1, PRKCZ, PRKCSH, PRIM2, PRF1, PREX1, PLEKHO1, PFN3, PDLIM7, PARVG, NOS3, MCAM, MARCKSL1, MAPK11, LPXN, LIMK2, JAK2, ITGA7, ICAM2, HRAS, HAX1, GGPS1, FGD3, FAS, DLEC1, DLC1, DIAPH3, DDX17, CASP8, CANX, ALCAM, AIF1, VEZT, TUBB2C, TSPAN4, TGFBRAP1, TGFA, TFRC, STAT5B, STAT4, STAT3, S100A10, RIC3, RERE, REM2, REEP5, PVRL1, PTP4A3, PSD, PRKCB, PLEK2, PAK3, NRP1, NBPF1, MRC2, MBP, MAP4K2, LPAR2, LHX1, LGALS3, KIAA0101, JUB, IL23A, HOXA13, HISPPD1, HGFAC, GPR172B, GPI, GIT2, FXYD5, FOSL1, FGD2, FBXL15, EXOC3, ETS1, ELMO2, DVL1, DDX5, CNP, CDC42SE1, CDC42EP1, CDC14A, C1orf9, BIN3, ATF3, ASCL1, ALS2, ADAR

Corresponding total number of TFBS:
(For each gene listed above, the total number of TFBS for any of the selected TFs, multiplied by the number of selected Gene ontology terms containing that gene)
72, 64, 63, 56, 49, 48, 42, 42, 42, 42, 42, 40, 40, 40, 40, 40, 35, 35, 30, 28, 28, 27, 27, 25, 24, 24, 24, 24, 24, 21, 21, 21, 20, 20, 20, 20, 20, 18, 18, 18, 18, 18, 18, 18, 18, 18, 18, 16, 16, 16, 16, 15, 14, 14, 14, 14, 14, 14, 12, 12, 12, 12, 12, 12, 12, 12, 12, 12, 10, 10, 10, 10, 10, 10, 10, 10, 9, 9, 9, 9, 9, 9, 9, 9, 9, 8, 8, 8, 8, 8, 8, 8, 8, 8, 8, 8, 8, 8, 8, 8, 7, 7, 7, 7, 7, 7, 7, 6, 6, 6, 6, 6, 6, 6, 6, 6, 6, 6, 6, 6, 6, 6, 6, 6, 6, 6, 6, 6, 6, 6, 6, 5, 5, 5, 5, 5, 5, 5, 5, 5, 5, 5, 5, 5, 5, 5, 5, 5, 5, 5, 5, 5, 5, 4, 4, 4, 4, 4, 4, 4, 4, 4, 4, 4, 4, 4, 4, 4, 4, 4, 4, 4, 4, 4, 4, 4, 4, 4, 4, 4, 4, 4, 4, 4, 3, 3, 3, 3, 3, 3, 3, 3, 3, 3, 3, 3, 3, 3, 3, 3, 3, 3, 3, 3, 3, 3, 3, 3, 3, 3, 3, 3, 3, 3, 2, 2, 2, 2, 2, 2, 2, 2, 2, 2, 2, 2, 2, 2, 2, 2, 2, 2, 2, 2, 2, 2, 2, 2, 2, 2, 2, 2, 2, 2, 2, 2, 2, 2, 2, 2, 2, 2, 2, 2, 2, 2, 2, 2, 2, 2, 1, 1, 1, 1, 1, 1, 1, 1, 1, 1, 1, 1, 1, 1, 1, 1, 1, 1, 1, 1, 1, 1, 1, 1, 1, 1, 1, 1, 1, 1, 1, 1, 1, 1, 1, 1, 1, 1, 1, 1, 1, 1, 1, 1, 1, 1, 1, 1, 1, 1, 1, 1, 1, 1, 1, 1, 1, 1, 1, 1, 1

Corresponding number of selected Gene ontology terms each gene is involved with:
(In the same order as above)
9, 8, 7, 8, 7, 6, 7, 7, 7, 6, 7, 8, 5, 8, 5, 5, 5, 7, 6, 4, 4, 3, 3, 5, 3, 3, 4, 4, 3, 3, 7, 3, 5, 4, 5, 5, 5, 6, 2, 3, 3, 3, 6, 3, 3, 6, 2, 2, 2, 2, 2, 3, 7, 7, 2, 7, 2, 7, 6, 6, 2, 2, 3, 2, 2, 3, 4, 3, 2, 2, 2, 5, 5, 2, 2, 5, 3, 9, 9, 1, 3, 3, 3, 1, 1, 1, 2, 1, 1, 1, 2, 2, 1, 1, 1, 4, 1, 8, 2, 2, 1, 1, 1, 7, 7, 7, 7, 1, 1, 1, 3, 2, 1, 1, 1, 2, 6, 1, 2, 6, 6, 6, 3, 3, 2, 1, 2, 1, 6, 3, 3, 6, 6, 5, 5, 5, 5, 1, 5, 1, 5, 1, 1, 1, 5, 1, 5, 5, 1, 1, 1, 5, 1, 5, 5, 2, 1, 1, 2, 4, 2, 2, 1, 2, 1, 4, 4, 1, 4, 4, 4, 1, 2, 2, 1, 1, 1, 2, 4, 2, 1, 1, 2, 4, 1, 3, 1, 3, 1, 1, 3, 3, 3, 3, 1, 1, 1, 1, 3, 1, 3, 1, 1, 1, 1, 1, 1, 1, 1, 1, 3, 3, 3, 1, 1, 1, 1, 2, 1, 1, 1, 2, 2, 1, 2, 1, 2, 2, 2, 1, 2, 2, 2, 1, 1, 2, 1, 2, 1, 1, 1, 1, 1, 1, 1, 2, 2, 2, 1, 2, 2, 1, 1, 1, 2, 2, 2, 2, 1, 1, 1, 2, 1, 1, 1, 1, 1, 1, 1, 1, 1, 1, 1, 1, 1, 1, 1, 1, 1, 1, 1, 1, 1, 1, 1, 1, 1, 1, 1, 1, 1, 1, 1, 1, 1, 1, 1, 1, 1, 1, 1, 1, 1, 1, 1, 1, 1, 1, 1, 1, 1, 1

Corresponding number of selected TFs each gene is involved with:
(In the same order as above)
8, 8, 9, 7, 7, 8, 6, 6, 6, 7, 6, 5, 8, 5, 8, 8, 7, 5, 5, 7, 7, 9, 9, 5, 8, 8, 6, 6, 8, 7, 3, 7, 4, 5, 4, 4, 4, 3, 9, 6, 6, 6, 3, 6, 6, 3, 9, 8, 8, 8, 8, 5, 2, 2, 7, 2, 7, 2, 2, 2, 6, 6, 4, 6, 6, 4, 3, 4, 5, 5, 5, 2, 2, 5, 5, 2, 3, 1, 1, 9, 3, 3, 3, 9, 9, 8, 4, 8, 8, 8, 4, 4, 8, 8, 8, 2, 8, 1, 4, 4, 7, 7, 7, 1, 1, 1, 1, 6, 6, 6, 2, 3, 6, 6, 6, 3, 1, 6, 3, 1, 1, 1, 2, 2, 3, 6, 3, 6, 1, 2, 2, 1, 1, 1, 1, 1, 1, 5, 1, 5, 1, 5, 5, 5, 1, 5, 1, 1, 5, 5, 5, 1, 5, 1, 1, 2, 4, 4, 2, 1, 2, 2, 4, 2, 4, 1, 1, 4, 1, 1, 1, 4, 2, 2, 4, 4, 4, 2, 1, 2, 4, 4, 2, 1, 3, 1, 3, 1, 3, 3, 1, 1, 1, 1, 3, 3, 3, 3, 1, 3, 1, 3, 3, 3, 3, 3, 3, 3, 3, 3, 1, 1, 1, 3, 2, 2, 2, 1, 2, 2, 2, 1, 1, 2, 1, 2, 1, 1, 1, 2, 1, 1, 1, 2, 2, 1, 2, 1, 2, 2, 2, 2, 2, 2, 2, 1, 1, 1, 2, 1, 1, 2, 2, 2, 1, 1, 1, 1, 2, 2, 2, 1, 1, 1, 1, 1, 1, 1, 1, 1, 1, 1, 1, 1, 1, 1, 1, 1, 1, 1, 1, 1, 1, 1, 1, 1, 1, 1, 1, 1, 1, 1, 1, 1, 1, 1, 1, 1, 1, 1, 1, 1, 1, 1, 1, 1, 1, 1, 1, 1, 1, 1, 1

Gene ontology terms ranked according to mean of effect sizes in cluster:
actin filament polymerization | actin cytoskeleton | leading edge | filamentous actin | cytoskeleton | establishment of cell polarity | cell motility | actin binding | actomyosin

Corresponding mean of effect sizes of each Gene ontology term:
(In the same order as above)
2.70, 2.48, 2.07, 2.03, 2.00, 1.75, 1.58, 1.24, 0.78

Corresponding total number of genes of each Gene ontology term:
(In the same order as above)
300, 605, 195, 439, 612, 152, 478, 142, 118

Corresponding number of genes of each Gene ontology term with at least one TFBS (of selected TFs):
(In the same order as above)
95, 168, 59, 120, 145, 38, 136, 33, 26

Corresponding total number of TFBS (of selected TFs) for each Gene ontology term (in all genes):
(In the same order as above)
318, 542, 206, 375, 485, 150, 400, 129, 92

Corresponding number of selected TFs each Gene ontology term is involved with:
(In the same order as above)
9, 9, 9, 9, 9, 9, 9, 9, 9

TFs ranked according to mean of effect sizes in cluster:
V$PEA3_Q6, V$ETS_Q4, V$TEL2_Q6, V$ETS_Q6, V$ETS2_B, V$PU1_Q6, V$ELF1_Q6, V$PXR_Q2, V$NKX25_Q5

Corresponding mean of effect sizes of each TF:
(In the same order as above)
2.47, 2.39, 2.24, 2.17, 2.06, 1.76, 1.76, 1.17, 0.61

Corresponding total number of TFBS for each TF (genome-wide):

(In the same order as above)
717, 718, 707, 721, 722, 724, 717, 684, 695

Corresponding total number of TFBS for each TF (in all genes in selected Gene ontology terms):
(In the same order as above)
324, 325, 317, 298, 317, 298, 311, 271, 236

Corresponding number of genes (of selected Gene ontology terms) each TF is involved with:
(In the same order as above)
114, 122, 107, 113, 120, 114, 121, 100, 90

Corresponding number of selected Gene ontology terms each TF is involved with:
(In the same order as above)
9, 9, 9, 9, 9, 9, 9, 9, 9

Cluster for columns 1295 to 1311, rows 267 to 278

Gene ontology terms

Notch signaling pathway | gene expression | DBD domain binding | DNA binding | chordate embryonic development | embryonic development | limb development | central nervous system development | central nervous system neuron development | gastrulation | somitogenesis | anatomical structure morphogenesis | organ development | neurogenesis | regionalization | cell fate determination | segmentation

TFs

V$ETF_Q6 | V$E2F_Q2 | V$E2F1_Q6 | V$E2F1_Q3 | V$AP2GAMMA_01 | V$AP2ALPHA_01 | V$AP2_Q6_01 | V$AP2_Q6 | V$WT1_Q6 | V$EGR_Q6 | V$MAZ_Q6 | V$MAZR_01

Information

All related TFs:
(List of all TFs that are related to any of the PWMs)
AP-2, AP-2alpha, AP-2alphaA, AP-2alphaB, AP-2beta, AP-2gamma, DP-1, E2F, E2F+E4, E2F-1, E2F-3a, E2F-4, ETF, Egr-1, Egr-2, Egr-3, MAZ, MAZR, WT1, WT1-del2

Ranked gene list:
(All genes of the selected Gene ontology terms with hits of any of the selected TFs, ranked according to the total number of TFBS)
LBX1, NOTCH1, FGF8, PAX6, LFNG, HES5, HES1, DLL1, TBX2, WNT1, ID2, HOXA9, AKT1, LHX1, NKX2-2, MDK, DVL1, PTCH1, CCND1, TGFB1, TCF3, DLX2, RBPJ, NR4A2, NR2F1, TLE3, NFATC1, CDKN1C, CDK5, AGRN, HOXA7, HMX3, LEF1, JARID2, DGAT1, PDGFA, FLII, FGF9, UNCX, HMGA1, GATA2, DNMT3A, RARA, HES7, TCF7, SSSCA1, SMAD7, MAFG, GATA3, RARG, HOXA10, ZIC2, WNT11, NR2F6, LDB1, FOXC1, EFNA2, DLL4, ZFP36L2, YY1, WNT10B, TBX1, RXRB, NRARP, NFIC, MAPK12, JUNB, IRX5, IGF1R, HOXB7, CYP26B1, CREBBP, CCND2, ACTB, SP3, NR3C1, ZFPM1, VEGFB, SOX8, SKI, GPX4, GLI1, FOXO3, BMI1, VEGFA, EGR2, SMARCD3, SIN3A, RUNX3, NR3C2, MMP11, LMX1B, GFI1, EGR3, AXIN1, ACHE, SEMA3B, RXRA, MAFK, LHX3, HOXC8, FOXD3, EGR1, UBE2I, THRA, PLXNA1, MEF2D, HES3, GPC1, FLJ32987, EFNB2, ZBTB7B, ZBTB7A, WNT7B, USF2, UBTF, SOX1, SNAPC2, SAMD4A, PCNA, NFKBIE, MNT, MMP23B, MAZ, ISYNA1, ISG15, HOXC9, HOXB6, HIVEP1, HES6, ERF, EHMT2, CDC25C, CBX4, BRD2, ARID3A, STRA13, SOCS3, REXO1, NELF, KLF13, HRAS, FASN, DPH1, CFL1, WNT4, TFAP2A, IRX4, IRF1, EGFL7, DYRK1A, CTCF, CORO7, TRADD, TFEB, SMAD6, RREB1, RRBP1, PBX3, MAFA, JAG2, IGF2, FLI1, DVL2, CUX1, BCL2L11, ACVR2B, ZNF362,

TP73, SOS1, SCRIB, POU3F2, MYB, MTA1, LYL1, ING2, ID3, HOXA11, HMGB1, FOXO1, DBP, CDK5R1, ABCA2, TM7SF2, SOX18, SKIL, RING1, REST, PURA, POU2F1, NAB2, MLL, LMO4, HOXB8, HHEX, CTBP1, CDKN1B, SHH, PATZ1, NGFR, MBD3, MAP2K7, FURIN, COL11A2, ADNP, WHSC1, WBP7, RUNX2, NOL3, NFKB2, JUND, FOSL2, E2F4, TNFSF13, PAX2, ZFP36, ZEB1, ZBTB16, WNT7A, TEAD3, SLC2A4RG, SLC25A22, SLC20A1, SCRT1, POU4F1, PLEC1, PKNOX1, PGP, PARD6A, OGFR, NR4A1, NEO1, NDOR1, MXI1, MAPK11, LRDD, KREMEN2, KISS1R, JMJD3, IRS1, HOXC6, HOXB5, HNRPDL, HMX2, HIVEP2, HIC1, H2AFZ, GIT1, FZD8, FGFRL1, FASTK, EZH2, EOMES, EFNA3, EFNA1, CUL4A, CSK, CRIP2, CRIP1, CKB, CIRBP, CDKN2C, CDK4, CD151, BCOR, BBC3, BACH2, ACTG1, TYMP, TNFRSF18, TNFRSF12A, SQSTM1, SLC4A2, SIPA1, REPIN1, RCOR2, PTMA, PNPLA2, PIGQ, NPDC1, LLGL2, IER2, HCFC1R1, GAMT, CEND1, BCL11B, ANP32A, WNT2B, TUSC2, TUBB3, TNFRSF4, TLE2, PBX2, NR4A3, LEPREL2, KLF10, HYAL2, HAND2, DDIT3, CREB1, CISH, CELSR3, CDK9, C21orf33, BCL3, ARHGEF2, ACVR1, ZEB2, TUBB2C, TGIF1, SHC1, PTBP1, PDGFB, NFYB, KLF12, IRF2, FBRS, ENOPH1, CLDN6, CHRD, ATP6V0C, AMH, ZFP161, WNT10A, VGF, STAU1, SNF1LK, SMARCA2, SMAD2, SLC7A1, SIX5, SHMT2, SATB2, RPS2, RPL8, QKI, POLR2A, PER1, PAX5, ONECUT1, NFIX, NFIB, NFIA, MXD3, MSL1, MKX, MAP4K2, MAP2K3, ING1, HS6ST1, HOXD11, HOXB4, HNRNPL, HDAC7, HCN2, HBA2, GTF2I, GPS2, GBX2, FMNL1, FGF17, ESRRA, ENO3, EFNA4, E4F1, DKFZp779C0757, CTSD, CTBP2, CPT1A, CPLX1, CORO1A, CHD3, CDKN1A, CBFA2T3, CARM1, BHLHB2, BCL9L, ATF3, APRT, ADORA2A, TIAM1, SPHK1, SOCS2, SMAD3, RORC, PHLDB1, PFKFB3, PCYT2, OTX1, OAZ1, NOTCH4, NKD2, NCOA6, MXD4, MARCKSL1, LTC4S, IRS2, HOXD13, HOXA1, GRIN2C, EP300, EMX1, EIF5A, CTSW, CAMK2G, CACNA1G, BCL6, ATXN1, WNT9A, VIM, VDAC1, TRERF1, TRAF4, TMEM201, TLE1, TAS1R3, SSBP3, SOCS1, SMARCA4, SETD1A, RHOA, PTPRU, PRKCE, PRKCD, PRDM16, PPP1R13B, PPARGC1B, PIK3R2, PELP1, PCID2, OCA2, NKX6-2, NFATC3, MCM2, MC1R, MAP3K12, LRP1, KLF11, HOXC5, HOXC4, HDGF, FZD7, FUS, FOXP1, FOXF2, FOXF1, FHOD1, ELAVL3, EEF1A2, DLL3, DAG1, CUL1, CRABP2, CHD7, CENPB, CD68, CBX2, CACNB3, BUB3, BCL11A, ADIPOR1, USP7, TRIM28, TRAF3, TBPL1, SRC, SFMBT2, RPL19, ROM1, REL, RCOR1, RBL1, RASSF1, PSMA7, PDGFRA, PCP2, NR2C2, NPTX1, NEUROG2, MGAT1, MAPKAPK3, LASP1, IL11, HOXB9, HOXA5, HOXA2, HES4, GRN, GAPDH, EVX2, DYRK2, CTDSP2, CDK6, ADM, ZFYVE9, ZFP36L1, XAB2, TWSG1, SSPO, SOX12, SIAH1, SEMA3F, RPL28, RHOC, PTPN12, PTK6, PPP1R1B, POLRMT, PIK3CA, PHIP, OSM, NRF1, MUC1, MSL2, MRPS6, MEN1, LRP5, KREMEN1, KLF2, KAT2A, IRF4, INTS1, IL17C, HSP90AB1, HSP90AA1, HOXB2, GTF2A1, GPSM1, FOS, FAM152A, EGR4, E2F2, DPYSL2, DAXX, CDK2, CDH15, CDC20, CBFB, CASP9, BRF1, BHLHB3, BCL2L2, ARNTL, ARID1B, ARID1A, APLP2, ALDOA, AIRE, YWHAZ, VAV2, TSPAN32, TSC22D1, TP53I3, TGFB3, TCF12, TBL1XR1, STAT5A, SRCAP, SPIB, SFTPC, SDC3, RPLP0, ROBLD3, RNF4, PTP4A3, PRKCZ, PPARA, POU3F3, PLXND1, PIN1, PIAS4, PHOX2A, PFN2, PDLIM7, PCSK4, PCGF2, NR1D1, NF1, NCLN, MSH6, MORF4L1, MFNG, MCAM, MAPK7, MAP3K14, LPHN1, LHX9, LGALS1, KCNH8, IRF7, Ht006, HOXA6, HIST1H4I, GTF2IRD1, GRIN2D, GEMIN4, GADD45B, FOSB, DUSP1, CTF1, CLEC11A, CEBPB, CCNE1, CBX3, CALM1, BAX, ARFRP1, AKT3, ZHX2, YES1, XPO1, UQCRH, TSTA3, TRRAP, TNFRSF25, TNFAIP3, TFRC, TAGLN2, STMN1, SMARCC2, SLCO3A1, SLC39A1, SLC19A1, SLC16A8, SEPT7, SEPHS1, RTN2, RPS6KA2, RND2, RGS10, REV3L, PTH1R, PSENEN, PRKCA, PRKACA, PPP3CA, PMF1, PFKP, OSR1, ODC1, NFATC4, NDRG2, NCOA2, MYST3, MYADM, MVP, MSI2, MIB1, MED13, MAX, MAFF, LY6E, LTBP3, LNX2, LHB, L1CAM, KLK1, KLF5, IRF6, IKZF1, HNRNPH1, HDLBP, HCFC1, H3F3B, H3F3A, GRB7, GAB2, FOXN2, FOLR2, FAM168B, FAF1, EPB41, EGLN2, DLG4, DHX58, DCHS1, DBN1, CYBA, CRTC2, CRB3, CNOT1, CHIC2, CDKN2D, CDC42EP4, CDC25B, CAMTA1, CALR, CACNA1C, C2orf28, BRMS1, BMP1, BACH1, ARHGAP5, AGER, ADRA2B, ZYX, ZNF521, ZC3H12A, YWHAG, YBX2, VLDLR, UQCR, TUBA1B, TRIM3, TPT1, TPM1, TOX2, TNRC6A, THPO, THBS3, TGFBRAP1, TADA2L, SYNCRIP, SRXN1, SMURF2, SMTN, SMEK1, SLC38A2, SLC29A1, SLC1A4, SLC12A5, SILV, SHOX2, SERTAD3, SCARB1, SBNO2, SALL3, S100A6, S100A4, RGS2, REPS1, RBM15, PXMP2, PVRL1, PUM1, PSMB8, PRMT1, PPP1R15B, PPP1R12C, PPIA, PPFIA1, PPARD, PNMT, PILRB, PHF7, PHC1, PER2, PDPK1, PDF, PBXIP1, PBX4, PAPD1, ORC6L, NRGN, NPPC, NPAS3, NPAS1, NNAT, NKG7, NFE2L3, NDRG3, NAB1, MYL6, MTHFR, MST1R, MSL3, MPP6, MLL2, MGC40146, MGA, MFRP, MADCAM1, LRBA, LMNA, LEPROT, LEPR, LDHA, KCNB1, JAKMIP1, ITPR3, IMPDH2, ILF3, IL17D, IGF2BP3, IDI1, HYAL1, HSPB6, HPN, HOXC10, HNRNPU, HNRNPD, HMGN1, HMBS, HES2, HDAC5, HDAC4, H6PD, GSK3A,

GPC2, GOT2, GNA13, GCLM, GABBR1, FTL, FSCN1, FRAT2, FOXP4, FHL3, FCGRT, FAM110A, ERBB2IP, ENO2, EMP3, EIF4G1, EIF4A1, EBF2, DYNLL1, DIO3, DICER1, DENND4A, DCTN3, CTNNA1, CRHR2, COL6A1, CNN1, CD82, CCNE2, CCL27, CAST, CAD, C21orf2, C1QTNF5, C17orf85, C17orf28, BMP2K, BANP, ATP1B1, ATP1A1, ASXL1, ARID5B, APOBEC3D, APBB3, ANKH, AKT2, ADA2B, ACSL6, ACACA, ABCA3

Corresponding total number of TFBS:
(For each gene listed above, the total number of TFBS for any of the selected TFs, multiplied by the number of selected Gene ontology terms containing that gene)
160, 156, 144, 132, 120, 110, 110, 110, 108, 99, 88, 88, 84, 81, 77, 72, 72, 66, 66, 64, 64, 64, 63, 63, 63, 60, 60, 60, 60, 60, 56, 56, 55, 55, 55, 54, 54, 50, 48, 48, 48, 48, 45, 45, 44, 44, 44, 44, 44, 42, 42, 40, 40, 40, 40, 40, 40, 40, 36, 36, 36, 36, 36, 36, 36, 36, 36, 36, 36, 36, 36, 36, 36, 36, 35, 35, 33, 33, 33, 33, 33, 33, 33, 33, 32, 32, 30, 30, 30, 30, 30, 30, 30, 30, 30, 30, 28, 28, 28, 28, 28, 28, 28, 27, 27, 27, 27, 27, 27, 27, 25, 24, 24, 24, 24, 24, 24, 24, 24, 24, 24, 24, 24, 24, 24, 24, 24, 24, 24, 24, 24, 24, 24, 24, 24, 24, 22, 22, 22, 22, 22, 22, 22, 22, 22, 21, 21, 21, 21, 21, 21, 21, 21, 20, 20, 20, 20, 20, 20, 20, 20, 20, 20, 20, 20, 20, 20, 18, 18, 18, 18, 18, 18, 18, 18, 18, 18, 18, 18, 18, 18, 16, 16, 16, 16, 16, 16, 16, 16, 16, 16, 16, 16, 16, 16, 16, 16, 15, 15, 15, 15, 15, 15, 15, 15, 14, 14, 14, 14, 14, 14, 14, 14, 13, 13, 12, 12, 12, 12, 12, 12, 12, 12, 12, 12, 12, 12, 12, 12, 12, 12, 12, 12, 12, 12, 12, 12, 12, 12, 12, 12, 12, 12, 12, 12, 12, 12, 12, 12, 12, 12, 12, 12, 12, 12, 12, 12, 12, 12, 12, 12, 12, 12, 12, 12, 12, 12, 12, 11, 11, 11, 11, 11, 11, 11, 11, 11, 11, 11, 11, 11, 11, 11, 11, 11, 11, 11, 10, 10, 10, 10, 10, 10, 10, 10, 10, 10, 10, 10, 10, 10, 10, 10, 10, 10, 10, 10, 9, 9, 9, 9, 9, 9, 9, 9, 9, 9, 9, 9, 9, 9, 9, 8, 8, 8, 8, 8, 8, 8, 8, 8, 8, 8, 8, 8, 8, 8, 8, 8, 8, 8, 8, 8, 8, 8, 8, 8, 8, 8, 8, 8, 8, 8, 8, 8, 8, 8, 8, 8, 8, 8, 8, 8, 8, 8, 8, 8, 8, 8, 8, 8, 8, 8, 8, 8, 8, 8, 8, 8, 8, 8, 8, 8, 8, 7, 7, 7, 7, 7, 7, 7, 7, 7, 7, 7, 7, 7, 7, 7, 7, 7, 7, 7, 7, 7, 7, 7, 7, 7, 7, 7, 7, 6, 6, 6, 6, 6, 6, 6, 6, 6, 6, 6, 6, 6, 6, 6, 6, 6, 6, 6, 6, 6, 6, 6, 6, 6, 6, 6, 6, 6, 6, 6, 6, 6, 6, 6, 6, 6, 6, 6, 6, 6, 6, 6, 6, 6, 6, 6, 6, 6, 6, 6, 6, 6, 6, 6, 6, 6, 5, 5, 5, 5, 5, 5, 5, 5, 5, 5, 5, 5, 5, 5, 5, 5, 5, 5, 5, 5, 5, 5, 5, 5, 5, 5, 5, 5, 5, 5, 5, 5, 5, 5, 5, 4, 4, 4, 4, 4, 4, 4, 4, 4, 4, 4, 4, 4, 4, 4, 4, 4, 4, 4, 4, 4, 4, 4, 4, 4, 4, 4, 4, 4, 4, 4, 4, 4, 4, 4, 4, 4, 4, 4, 4, 4, 4, 4, 4, 4, 4, 4, 4, 4, 4, 4, 4, 4, 4, 4, 4, 3, 3, 3, 3, 3, 3, 3, 3, 3, 3, 3, 3, 3, 3, 3, 3, 3, 3, 3, 3, 3, 3, 3, 3, 3, 3, 3, 3, 3, 3, 3, 3, 3, 3, 3, 3, 3, 3, 3, 3, 3, 3, 3, 3, 3, 3, 3, 3, 3, 3, 3, 3, 3, 3, 3, 3, 3, 3, 3, 3, 3, 3, 2, 2, 2, 2, 2, 2, 2, 2, 2, 2, 2, 2, 2, 2, 2, 2, 2, 2, 2, 2, 2, 2, 2, 2, 2, 2, 2, 2, 2, 2, 2, 2, 2, 2, 2, 2, 2, 2, 2, 2, 2, 2, 2, 2, 2, 2, 2, 2, 2, 2, 2, 2, 2, 2, 2, 2, 2, 2, 2, 2, 2, 2, 2, 2, 2, 2, 2, 2, 2, 2, 2, 2, 2, 2, 2, 2, 2, 2, 2, 2, 2, 2, 2, 2, 2, 2, 2, 2, 2, 2, 2, 1

Corresponding number of selected Gene ontology terms each gene is involved with:
(In the same order as above)
16, 13, 12, 11, 10, 11, 11, 11, 9, 11, 8, 8, 7, 9, 7, 6, 6, 6, 6, 8, 8, 8, 9, 7, 9, 5, 5, 5, 5, 5, 7, 7, 11, 5, 5, 6, 6, 5, 4, 4, 4, 4, 9, 5, 4, 4, 4, 4, 4, 6, 6, 4, 5, 4, 4, 4, 5, 5, 3, 6, 4, 4, 3, 3, 3, 3, 3, 4, 3, 6, 4, 6, 4, 4, 5, 5, 3, 3, 3, 3, 3, 11, 3, 3, 8, 8, 3, 3, 5, 3, 5, 6, 3, 3, 6, 3, 4, 4, 4, 7, 4, 4, 4, 3, 3, 3, 3, 3, 3, 3, 5, 2, 2, 4, 3, 2, 4, 2, 3, 8, 2, 2, 3, 2, 2, 2, 2, 4, 2, 8, 3, 3, 6, 2, 4, 2, 2, 2, 2, 2, 2, 2, 2, 2, 2, 7, 7, 3, 3, 3, 3, 3, 3, 2, 2, 5, 2, 4, 4, 2, 2, 4, 5, 2, 2, 2, 5, 3, 6, 6, 2, 3, 6, 3, 2, 2, 6, 6, 3, 3, 2, 6, 2, 4, 2, 2, 2, 4, 2, 4, 2, 2, 4, 4, 4, 2, 4, 15, 3, 5, 3, 3, 5, 3, 3, 2, 2, 7, 7, 2, 2, 2, 2, 13, 13, 1, 3, 4, 6, 3, 1, 1, 6, 1, 6, 1, 4, 1, 4, 1, 3, 4, 2, 3, 1, 1, 1, 1, 1, 1, 4, 4, 4, 4, 1, 1, 2, 1, 2, 1, 1, 4, 6, 1, 3, 2, 3, 1, 1, 1, 1, 2, 3, 1, 1, 1, 2, 1, 1, 1, 1, 1, 1, 1, 1, 1, 1, 1, 1, 1, 1, 1, 1, 1, 1, 1, 1, 5, 1, 5, 1, 5, 5, 2, 5, 2, 2, 10, 2, 5, 2, 2, 2, 10, 2, 2, 5, 3, 1, 3, 3, 1, 1, 3, 1, 3, 1, 3, 1, 9, 1, 3, 1, 2, 2, 4, 1, 4, 8, 4, 2, 1, 1, 1, 1, 1, 1, 1, 2, 8, 2, 4, 4, 2, 2, 2, 2, 2, 1, 1, 4, 4, 1, 1, 1, 1, 4, 1, 8, 1, 8, 1, 1, 1, 1, 2, 1, 1, 1, 1, 4, 2, 8, 2, 4, 4, 1, 2, 1, 1, 1, 1, 1, 7, 1, 1, 1, 1, 7, 1, 7, 1, 7, 1, 1, 1, 1, 7, 7, 1, 7, 7, 1, 1, 1, 1, 1, 1, 3, 6, 2, 1, 2, 6, 6, 1, 2, 2, 6, 1, 6, 3, 1, 1, 1, 2, 1, 2, 3, 1, 2, 1, 3, 3, 1, 1, 3, 2, 2, 2, 3, 6, 3, 2, 2, 6, 1, 1, 1, 6, 3, 2, 2, 1, 2, 1, 2, 1, 2, 1, 3, 1, 1, 1, 5, 5, 1, 1, 1, 5, 1, 5, 1, 1, 5, 1, 5, 1, 5, 1, 1, 1, 1, 5, 5, 5, 1, 1, 1, 5, 1, 1, 1, 1, 2, 1, 1, 4, 2, 1, 1, 1, 1, 1, 1, 2, 2, 1, 4, 2, 4, 4, 2, 1, 1, 1, 4, 2, 2, 4, 2, 1, 1, 1, 2, 4, 4, 1, 4, 2, 2, 2, 2, 1, 4, 2, 1, 2, 2, 1, 4, 1, 2, 2, 2, 1, 2, 1, 1, 1, 1, 1, 3, 3, 3, 1, 3, 1, 3, 3, 3, 1, 3, 1, 1, 1, 3, 1, 1, 3, 1, 3, 1, 1, 3, 1, 1, 3, 1, 3, 3, 3, 1, 3, 3, 1, 3, 3, 3, 3, 3, 1, 3, 3, 1, 1, 1, 3, 1, 3, 1, 3, 3, 1, 1, 3, 1, 1, 2, 1, 2, 1, 1, 1, 2, 1, 2, 1, 1, 2, 1, 1, 1, 2, 2, 1, 1, 2, 1, 1, 2, 1, 2, 2, 1, 1, 1, 1, 2, 2, 2, 1, 2, 1, 1, 1, 1, 2, 2, 2, 2, 1, 1, 1, 1, 2, 2, 2, 2, 2, 1, 1, 2, 2, 1, 1, 1, 2, 1, 2, 2, 1, 2, 1, 1, 1, 2, 1, 1, 1, 2, 2, 1, 1, 1, 1, 1, 1, 2, 1, 2, 2, 1,

1, 1, 1, 1, 1, 1, 1, 1, 1, 1, 1, 1, 1, 1, 1

Corresponding number of selected TFs each gene is involved with:
(In the same order as above)
10, 12, 12, 12, 12, 10, 10, 10, 12, 9, 11, 11, 12, 9, 11, 12, 12, 11, 11, 8, 8, 8, 7, 9, 7, 12, 12, 12, 12, 12, 8, 8, 5, 11, 11, 9, 9, 10, 12, 12, 12, 12, 5, 9, 11, 11, 11, 11, 11, 7, 7, 10, 8, 10, 10, 10, 8, 8, 12, 6, 9, 9, 12, 12, 12, 12, 12, 9, 12, 6, 9, 6, 9, 9, 7, 7, 11, 11, 11, 11, 11, 3, 11, 11, 4, 4, 10, 10, 6, 10, 6, 5, 10, 10, 5, 10, 7, 7, 7, 4, 7, 7, 7, 9, 9, 9, 9, 9, 9, 9, 5, 12, 12, 6, 8, 12, 6, 12, 8, 3, 12, 12, 8, 12, 12, 12, 12, 6, 12, 3, 8, 8, 4, 12, 6, 12, 11, 11, 11, 11, 11, 11, 11, 11, 11, 3, 3, 7, 7, 7, 7, 7, 7, 10, 10, 4, 10, 5, 5, 10, 10, 5, 4, 10, 10, 10, 4, 6, 3, 3, 9, 6, 3, 6, 9, 9, 3, 3, 6, 6, 9, 3, 9, 4, 8, 8, 8, 4, 8, 4, 8, 8, 4, 4, 4, 8, 4, 1, 5, 3, 5, 5, 3, 5, 5, 7, 7, 2, 2, 7, 7, 7, 7, 1, 1, 12, 4, 3, 2, 4, 12, 12, 2, 12, 2, 12, 3, 12, 3, 12, 4, 3, 6, 4, 12, 12, 12, 12, 12, 12, 3, 3, 3, 3, 12, 12, 6, 12, 6, 12, 12, 3, 2, 12, 4, 6, 4, 12, 12, 12, 12, 6, 4, 12, 12, 12, 6, 12, 11, 11, 11, 11, 11, 11, 11, 11, 11, 11, 11, 11, 11, 11, 11, 11, 11, 11, 11, 2, 10, 2, 10, 2, 2, 5, 2, 5, 5, 1, 5, 2, 5, 5, 5, 1, 5, 5, 2, 3, 9, 3, 3, 9, 9, 3, 9, 3, 9, 3, 9, 1, 9, 3, 8, 4, 4, 2, 8, 2, 1, 2, 4, 8, 8, 8, 8, 8, 8, 8, 4, 1, 4, 2, 2, 4, 4, 4, 4, 4, 8, 8, 2, 2, 8, 8, 8, 8, 2, 8, 1, 8, 1, 8, 8, 8, 8, 4, 8, 8, 8, 8, 2, 4, 1, 4, 2, 2, 8, 4, 8, 8, 7, 7, 7, 1, 7, 7, 7, 7, 1, 7, 1, 7, 1, 7, 7, 7, 7, 1, 1, 7, 1, 1, 7, 7, 7, 7, 7, 7, 2, 1, 3, 6, 3, 1, 1, 6, 3, 3, 1, 6, 1, 2, 6, 6, 6, 3, 6, 3, 2, 6, 3, 6, 2, 2, 6, 6, 2, 3, 3, 3, 2, 1, 2, 3, 3, 1, 6, 6, 6, 1, 2, 3, 3, 6, 3, 6, 3, 6, 3, 6, 2, 5, 5, 5, 1, 1, 5, 5, 5, 1, 5, 1, 5, 5, 1, 5, 1, 5, 1, 5, 5, 5, 5, 1, 1, 1, 5, 5, 5, 1, 5, 5, 5, 5, 2, 4, 4, 1, 2, 4, 4, 4, 4, 4, 4, 2, 2, 4, 1, 2, 1, 1, 2, 4, 4, 4, 1, 2, 2, 1, 2, 4, 4, 4, 2, 1, 1, 4, 1, 2, 2, 2, 2, 4, 1, 2, 4, 2, 2, 4, 1, 4, 2, 2, 2, 4, 2, 4, 3, 3, 3, 3, 1, 1, 1, 3, 1, 3, 1, 1, 1, 3, 1, 3, 3, 3, 1, 3, 3, 1, 3, 1, 3, 3, 1, 3, 3, 1, 3, 1, 1, 1, 3, 1, 1, 3, 1, 1, 1, 1, 1, 3, 1, 1, 3, 3, 3, 1, 3, 1, 3, 1, 1, 3, 3, 1, 3, 3, 1, 2, 1, 2, 2, 2, 1, 2, 1, 2, 2, 1, 2, 2, 2, 1, 1, 2, 2, 1, 2, 2, 1, 2, 1, 1, 2, 2, 2, 2, 1, 1, 1, 2, 1, 2, 2, 2, 2, 1, 1, 1, 1, 2, 2, 2, 2, 1, 1, 1, 1, 1, 2, 2, 1, 1, 2, 2, 2, 1, 2, 1, 1, 2, 1, 2, 2, 2, 1, 2, 2, 2, 1, 1, 2, 2, 2, 2, 2, 2, 1, 2, 1, 1, 2, 2, 2, 1

Gene ontology terms ranked according to mean of effect sizes in cluster:
embryonic development | chordate embryonic development | Notch signaling pathway | DNA binding | neurogenesis | segmentation | gene expression | somitogenesis | DBD domain binding | cell fate determination | anatomical structure morphogenesis | regionalization | gastrulation | organ development | central nervous system development | central nervous system neuron development | limb development

Corresponding mean of effect sizes of each Gene ontology term:
(In the same order as above)
4.01, 3.55, 3.23, 2.66, 2.57, 2.56, 2.46, 2.36, 2.30, 2.09, 2.03, 2.01, 1.97, 1.88, 1.41, 1.34, 1.27

Corresponding total number of genes of each Gene ontology term:
(In the same order as above)
1.2K, 671, 103, 1.2K, 409, 109, 3.8K, 116, 411, 145, 583, 62, 293, 339, 110, 37, 129

Corresponding number of genes of each Gene ontology term with at least one TFBS (of selected TFs):
(In the same order as above)
295, 173, 38, 269, 104, 39, 688, 30, 111, 42, 125, 24, 71, 83, 29, 10, 29

Corresponding total number of TFBS (of selected TFs) for each Gene ontology term (in all genes):
(In the same order as above)
1425, 882, 231, 1355, 534, 227, 3149, 187, 545, 249, 622, 141, 349, 421, 150, 64, 140

Corresponding number of selected TFs each Gene ontology term is involved with:
(In the same order as above)
12, 12, 12, 12, 12, 12, 12, 12, 12, 12, 12, 12, 12, 12, 12, 12, 12

TFs ranked according to mean of effect sizes in cluster:
V$E2F_Q2, V$E2F1_Q6, V$AP2GAMMA_01, V$AP2ALPHA_01, VETF_Q6, VAP2_Q6,

V$E2F1_Q3, V$AP2_Q6_01, V$WT1_Q6, V$EGR_Q6, V$MAZR_01, V$MAZ_Q6

Corresponding mean of effect sizes of each TF:
(In the same order as above)
2.98, 2.88, 2.74, 2.73, 2.72, 2.35, 2.31, 2.21, 1.95, 1.76, 1.72, 1.64

Corresponding total number of TFBS for each TF (genome-wide):
(In the same order as above)
714, 691, 695, 686, 699, 688, 709, 690, 729, 695, 705, 734

Corresponding total number of TFBS for each TF (in all genes in selected Gene ontology terms):
(In the same order as above)
993, 970, 880, 870, 945, 919, 851, 884, 919, 840, 766, 834

Corresponding number of genes (of selected Gene ontology terms) each TF is involved with:
(In the same order as above)
346, 327, 315, 309, 344, 337, 315, 327, 337, 314, 296, 323

Corresponding number of selected Gene ontology terms each TF is involved with:
(In the same order as above)
17, 17, 17, 17, 17, 17, 17, 17, 17, 17, 17, 17

Cluster for columns 354 to 364, rows 33 to 39

Gene ontology terms

merozoite dense granule | melanosome | organelle | oligosaccharide biosynthetic process | checkpoint clamp complex | transpiration | vacuolar membrane | vacuole | multivesicular body | autophagic vacuole | autophagy

TFs

V$USF_01 | V$MAX_01 | V$USF_Q6 | V$USF_02 | V$USF_Q6_01 | V$MYCMAX_03 | V$ARNT_01

Information

All related TFs:
(List of all TFs that are related to any of the PWMs)
Arnt, Max, Max1, USF, USF-1, USF1, USF1:USF2, USF1a, USF1b, USF2, USF2a, USF2b, c-Myc

Ranked gene list:
(All genes of the selected Gene ontology terms with hits of any of the selected TFs, ranked according to the total number of TFBS)
LAMP1, CD63, BAX, ATP6V0C, RAB5A, CTSA, CALR, APEX1, SQSTM1, MCOLN1, GABARAP, MTHFR, MAPRE2, GAA, CTSD, VPS16, VPS11, S100A6, PPA1, KIF2A, FIS1, MFSD10, ANXA6, QTRT1, GALNS, CTDSP2, SGTA, RNASEH2C, NOD2, HPS5, C18orf8, ATXN3, ATP6V1A, ATP6V0E1, ALG1, AKT1, USP14, SUMF1, RAB24, PHF20, MARCH8, IGF2R, GPSM1, CRTC2, PRKAR2B, PDIA2, NCL, MYD88, MUC1, MC1R, GAPDH, GALT, ATP6V1H, SYTL1, SLC31A2, RGS19, PLA2G1B, MYO1C, MAP1LC3A, HPS1, FADD, EEF2K, CLN3, CASP9, BNIP3L, ATG5, ARFRP1, VPS8, VPS36, UVRAG, USP3, TSC2, TCIRG1, RCC1, PROC, PISD, PEX10, MLPH, MARCKSL1, LSS, HSPA8, HSP90AA1, GGA2, FGFR3, CRTC1, CHMP4A, CHMP2A, BNIP3, BAK1, ATG4B, ATG3, ARVCF, ARF1, ACD, VAC14, UGT8, UCHL1, TOM1L2, TOM1, TICAM1, TBX1, SOS1, SLC25A1, RTN4, RABAC1, RAB4A, PEX7, PDCD6, PAWR, PARVA, OXSM, NKX2-3,

NGLY1, MPG, MAPK1, LONP1, HSP90B1, HMGB1, HMBS, GGT1, GADD45B, FOXO3, FLVCR2, EXOC3, CDKN2D, ATIC, APC, ALG12, ACOX1

Corresponding total number of TFBS:
(For each gene listed above, the total number of TFBS for any of the selected TFs, multiplied by the number of selected Gene ontology terms containing that gene)
49, 35, 21, 21, 20, 18, 18, 18, 14, 14, 14, 12, 12, 12, 12, 10, 10, 10, 10, 10, 10, 8, 8, 7, 7, 7, 6, 6, 6, 6, 6, 6, 6, 6, 6, 6, 5, 5, 5, 5, 5, 5, 5, 5, 4, 4, 4, 4, 4, 4, 4, 4, 3, 3, 3, 3, 3, 3, 3, 3, 3, 3, 3, 3, 3, 3, 2, 2, 2, 2, 2, 2, 2, 2, 2, 2, 2, 2, 2, 2, 2, 2, 2, 2, 2, 2, 2, 2, 2, 2, 2, 2, 1, 1, 1, 1, 1, 1, 1, 1, 1, 1, 1, 1, 1, 1, 1, 1, 1, 1, 1, 1, 1, 1, 1, 1, 1, 1, 1, 1, 1, 1, 1, 1

Corresponding number of selected Gene ontology terms each gene is involved with:
(In the same order as above)
7, 5, 3, 3, 5, 6, 3, 3, 2, 2, 2, 2, 2, 2, 4, 2, 2, 2, 2, 2, 2, 2, 2, 1, 1, 1, 1, 1, 1, 1, 1, 1, 2, 2, 2, 2, 1, 1, 1, 1, 1, 1, 1, 1, 1, 1, 1, 1, 2, 1, 1, 1, 1, 1, 1, 1, 1, 3, 3, 1, 1, 1, 1, 1, 3, 1, 1, 2, 2, 2, 1, 2, 2, 1, 1, 1, 2, 1, 1, 1, 2, 1, 1, 2, 2, 1, 2, 2, 2, 2, 2, 2, 1, 1, 1, 1, 1, 1, 1, 1, 1, 1, 1, 1, 1, 1, 1, 1, 1, 1, 1, 1, 1, 1, 1, 1, 1, 1, 1, 1, 1, 1, 1, 1, 1, 1, 1

Corresponding number of selected TFs each gene is involved with:
(In the same order as above)
7, 7, 7, 7, 4, 3, 6, 6, 7, 7, 7, 6, 6, 6, 3, 5, 5, 5, 5, 5, 5, 4, 4, 7, 7, 7, 6, 6, 6, 6, 6, 6, 3, 3, 3, 3, 5, 5, 5, 5, 5, 5, 5, 5, 4, 4, 4, 4, 2, 4, 4, 4, 4, 3, 3, 3, 3, 3, 1, 1, 3, 3, 3, 3, 3, 1, 3, 2, 1, 1, 1, 2, 1, 1, 2, 2, 2, 1, 2, 2, 2, 1, 2, 2, 1, 1, 2, 1, 1, 1, 1, 1, 1, 2, 1, 1, 1, 1, 1, 1, 1, 1, 1, 1, 1, 1, 1, 1, 1, 1, 1, 1, 1, 1, 1, 1, 1, 1, 1, 1, 1, 1, 1, 1, 1, 1, 1

Gene ontology terms ranked according to mean of effect sizes in cluster:
autophagy | vacuole | vacuolar membrane | melanosome | organelle | merozoite dense granule | multivesicular body | autophagic vacuole | transpiration | checkpoint clamp complex | oligosaccharide biosynthetic process

Corresponding mean of effect sizes of each Gene ontology term:
(In the same order as above)
3.14, 2.42, 2.18, 2.12, 1.64, 1.63, 1.60, 1.55, 1.35, 1.30, 1.06

Corresponding total number of genes of each Gene ontology term:
(In the same order as above)
195, 208, 72, 56, 255, 40, 67, 63, 23, 42, 25

Corresponding number of genes of each Gene ontology term with at least one TFBS (of selected TFs):
(In the same order as above)
49, 40, 17, 9, 33, 5, 13, 14, 3, 10, 6

Corresponding total number of TFBS (of selected TFs) for each Gene ontology term (in all genes):
(In the same order as above)
153, 128, 61, 38, 117, 19, 42, 45, 13, 22, 16

Corresponding number of selected TFs each Gene ontology term is involved with:
(In the same order as above)
7, 7, 7, 7, 7, 7, 7, 7, 6, 7, 6

TFs ranked according to mean of effect sizes in cluster:
V$MYCMAX_03, V$USF_02, VUSF_01, VUSF_Q6_01, VUSF_Q6, VARNT_01, V$MAX_01

Corresponding mean of effect sizes of each TF:
(In the same order as above)

2.51, 2.06, 2.05, 1.69, 1.69, 1.44, 1.26

Corresponding total number of TFBS for each TF (genome-wide):
(In the same order as above)
650, 649, 647, 664, 672, 655, 647

Corresponding total number of TFBS for each TF (in all genes in selected Gene ontology terms):
(In the same order as above)
110, 101, 103, 87, 92, 82, 79

Corresponding number of genes (of selected Gene ontology terms) each TF is involved with:
(In the same order as above)
64, 58, 59, 53, 49, 48, 49

Corresponding number of selected Gene ontology terms each TF is involved with:
(In the same order as above)
11, 11, 11, 11, 11, 10, 10

Cluster for columns 1161 to 1204, rows 97 to 108

Gene ontology terms

lung development | ossification | osteoblast differentiation | cartilage development | chondrocyte differentiation | skeletal development | gut development | neuron development | odontogenesis | transdifferentiation | kidney development | mesoderm formation | heart development | skeletal muscle development | gliogenesis | stem cell differentiation | epithelial to mesenchymal transition | fibroblast growth factor receptor activity | fibroblast growth factor receptor binding | neural tube closure | compound eye development | camera-type eye development | eye development | neural tube formation | system development | cell-cell signaling | cell fate specification | lens development in camera-type eye | neuron migration | N-acetyltransferase activity | developmental process | cell | growth | provirus | induction | apoptosis | cell proliferation | response to osmotic stress | luciferin monooxygenase activity | transcription factor complex | decidualization | liver development | myoblast differentiation | regulation of cell proliferation

TFs

V$STAT6_01 | V$STAT5A_03 | V$STAT4_01 | V$STAT5A_04 | V$STAT1_03 | V$CEBP_Q3 | V$PAX2_02 | V$HMGIY_Q6 | V$HOXA4_Q2 | V$TST1_01 | V$NFAT_Q6 | V$NFAT_Q4_01

Information

All related TFs:
(List of all TFs that are related to any of the PWMs)
ANF-2, C/EBP, C/EBPalpha, C/EBPalpha(p20), C/EBPalpha(p30), C/EBPbeta, C/EBPbeta(LAP), C/EBPbeta(p20), C/EBPbeta(p34), C/EBPbeta(p35), C/EBPdelta, C/EBPepsilon, C/EBPgamma, HMG, HMG-Y, HMGI-C, HOXA4, NF-AT, NF-AT1, NF-AT1C, NF-AT2, NF-AT3, NF-AT4, POU3F1, Pax-2, Pax-2.1, Pax-2.2, Pax-2a, Pax-2b, STAT1, STAT1alpha, STAT4, STAT5A, STAT6

Ranked gene list:
(All genes of the selected Gene ontology terms with hits of any of the selected TFs, ranked according to the total number of TFBS)
LBX1, CTNNB1, RUNX1, FGF8, PAX6, CD44, LEF1, VIM, CDKN1B, HES1, STAT1, SSSCA1, SMAD7, CXCR4, GLI1, RUNX3, ID2, RARG, PTGER4, ETS1, TGFBR2, ID3, TNF, NFKBIA,

CDC25C, RARA, PTCH1, MAP2K3, TFAP2A, SHC1, PITX3, NR4A2, IRS1, HIST1H4I, FYN, CDK6, CDK4, STAT3, WNT1, BCL2L1, MEF2D, KLF6, JUN, IRF1, IL23A, GFI1, CSK, CISH, CDK2, CCND3, CCND2, NR3C1, ILK, DUSP6, COL11A2, NKX2-2, DLX2, DLL1, CFL1, CDKN2C, SMAD3, NFATC1, JUNB, EGR1, VEGFA, SOX2, PTPN6, PTMA, PROX1, PRDM1, PER1, NFATC3, MAF, LTB, LTA, ITGA5, DUSP1, BTG1, ACVR1, WNT10B, TNFRSF1A, RBPJ, PIK3R1, AKT1, NEUROG2, HSPA8, HAND2, CITED2, CD4, BHLHB2, S100A4, NR2F2, GSK3B, STAT5A, PLCG1, DDX5, CD68, ADM, VDR, PDCD4, MXI1, GATA3, FST, CDK5, BRD2, BCL2L11, ARID2, PSMP, MYB, MARK2, EGR2, BCL2, RASSF1, NR3C2, NR2F1, CCR7, TAF8, MYC, MAP3K1, HIST4H4, GGPS1, FLJ32987, E2F3, DDIT3, CSF1, SOCS1, SGK1, S1PR1, RHOC, NFYA, JARID2, CTLA4, CREB1, ARL4C, IGF1R, TUSC2, TNFAIP3, TIPARP, RASSF5, MAPK14, MAP3K12, HOXB7, HOXB4, HOXA10, GNAS, FOXP1, FBRS, BCL6, PAX5, FGF9, ANXA6, ALCAM, SOS1, SMARCA2, PSMB9, MBNL1, IL7R, HOXD13, FOSL2, DCTN3, SIX1, PTGER2, NEDD9, IRF2, EPHA4, ZC3H12D, PDLIM5, NFKBIE, LHX1, HSPA1B, ETV3, ESR1, CKS1B, CDKN1A, ANG, TRAF3, SOCS2, MEIS2, H2AFX, FOXD3, DAXX, CYP27B1, SILV, PITX2, OSM, HOXA5, BARHL2, SPRY1, HMGA1, GAPDH, CASP2, ZFP36L2, ZFP36L1, ZFP36, WIBG, USF1, UNK, TRERF1, TRAF5, TGIF1, TCF7, TAP1, SOCS3, SHOX2, SATB1, S100A6, S100A10, RREB1, RHOF, RERE, PSMB8, PRKCE, NR4A3, NNAT, NIN, MLL2, MLL, MGAT5, LMO4, IL2RA, IKZF1, HOXA9, HOXA11, HDAC7, GIMAP5, EOMES, ENO2, DPAGT1, CUGBP2, CLIC1, CLCN3, BUB3, BCL11B, ATP2B4, ARL6IP5, ARHGEF2, TGFB3, SRF, SH2B3, RNASEK, PPP2CA, PAX3, OSR1, NAB2, MAP3K14, LDB1, HOXB2, CYB561D2, CBX8, BMI1, BACH2, ZEB1, TTF2, PTK2B, LCK, KSR1, HSP90B1, SLA, SATB2, PURA, POU4F1, PCNA, KLF9, GRAP2, GDNF, EIF5A, DLX1, BMF, BAK1, ANP32A, ADAMTS4, TXNIP, SLC39A1, SLC38A2, RTN4, OTX2, MNT, MAZ, JAK1, ITPR1, IKBKE, HOXA2, GRB2, GABARAP, EFNA1, DUSP5, C2orf28, BCL11A, ALDOA, AHSA1, STK16, SLC4A2, SART3, NARG1, MAP4K4, IL16, GIT1, FOXO1, FOXG1, EP300, EBF1, CD3D, CADM1, ZIC1, TG, PSMC5, PRDX2, POLD4, PDE4B, NDRG1, ING1, GATA2, DVL2, CYP26B1, ZNRD1, ZNF292, ZIC2, WIPF1, UBTF, TRIM3, PAX2, KAT2B, GDF11, CACYBP, TP53, SMAD4, CCND1, ZMYND10, ZEB2, VEGFB, TSHZ1, TPT1, THRA, TGFBR3, STAT6, SKP1, RORC, RASSF2, PPP1R1B, PIK3IP1, PIK3CA, PFN1, PDGFB, NR4A1, MT2A, MGAT1, MAPRE2, MAPKAPK3, LBH, KLF3, ITGB1, ICOS, HOXC8, HOXC6, HOXC4, HOXB5, HOXB3, HOXA7, HNRNPC, HIVEP2, H3F3B, FOS, FLI1, ETV1, E2F4, E2F2, DIAPH1, CUL1, CFLAR, CD69, CALM2, BAT3, ATXN1, ARID1B, ARID1A, AHNAK, UBC, TUSC4, TNIP1, SLC38A1, RHOG, RFTN1, PPP1R10, PIK3CG, PDE6D, MYO1G, MVP, MLLT10, KLF7, HOXB6, FYB, FOXJ2, ETV6, CRTC2, CDKL3, CD3G, C16orf53, AGPAT1, TOB2, TNFAIP8, TAPBP, STOML2, STAT5B, SLC35B2, SKAP1, SERTAD2, RPS6KA1, POU2F1, PLK3, PLEC1, NFYC, MYD88, HOXD11, HOXB9, HOXA1, HNRNPL, HNRNPH3, HIVEP1, GALT, CRIP1, CDC42SE1, CD247, CAMK2G, ADD3, VEZF1, TAP2, TAOK2, TAGLN2, SHMT2, RB1, RAG1AP1, RAF1, PKM2, PHB2, NIPBL, MAT2B, LCP1, LAG3, HSP90AB1, HNRNPA2B1, HECA, GNAI2, ENO3, DYRK2, DUSP2, DLX6, CD82, AXUD1, ARHGEF1, ARHGDIB, ADIPOR1, WNK1, WDR1, WBP7, TPP1, TMC6, TCF7L2, STIM1, SOX4, SLC20A1, PPARA, PIK3CD, PHF20, NP, NF1, NAT13, MZF1, MGAT4A, MCAM, MAX, MAPKAPK2, MAP2K6, MAML2, LRIG1, IL6R, IGF1, HYAL2, HSPA1A, HEY2, GTF3A, GIN1, FUS, FBXW11, ETV5, ENC1, CYTIP, CYLD, CTDSP2, CD47, CD27, CALM1, APEX1, VAMP8, TPM3, TMEM115, SLC9A3R1, SGMS1, REPIN1, PTPN7, PDGFRA, NOS2, MIA, MDGA1, LRDD, LGALS1, ISYNA1, ICK, GAD1, FOSB, EMILIN1, CIDEB, CD40, ARFIP2, ACVR2A, VAMP2, UGP2, UCP2, TXNDC12, TWIST1, TRADD, TOB1, STK17B, RNF31, PXN, PSME2, PML, PEA15, MEIS1, MAL, LTBP4, LCP2, KRAS, KLF2, KCNA3, ITGB7, IL2, GNB2L1, GIT2, GGNBP2, FHL3, EVL, EIF4A2, EFNB2, DKFZp779C0757, DGKZ, DAD1, CXCR5, CNTNAP1, CCR10, CAP1, CALR, B3GNT2, ATF4, ARID4B, AKTIP, ADNP, ZNF384, UROD, TNFRSF1B, RGL2, PTTG1, PRKCD, PPP1R9B, PIM1, PELP1, PCF11, PARP1, OSR2, NFAT5, NCOA4, MKNK1, MGA, MBD1, MAP3K5, HSPD1, HDGF, HCLS1, GLG1, FOXO3, ESRRA, EMP3, DCTD, DBP, COL2A1, CD5, C17orf81, ANXA1, AKT1S1, ABCD2, ZAP70, VASP, TUBB, TRAF1, TNFSF8, SLAMF1, SH3BGRL3, SELPLG, RNF41, QKI, PRG4, PPM1B, POU3F2, PHIP, PHF1, OBFC2B, NDE1, MLLT3, MLC-B, MDC1, MAPK13, MADD, LFNG, LDLR, KLF5, ITGA6, HOXA4, GLRX, GCS1, GALE, FGF11, FAIM3, ERBB2IP, EIF4G2, EGLN2, DUSP10, CDC25B, CD79A, CD63, CCR6, BRAF, ATF2, AQP3, ADORA2A, ACTN1, ABI3, ABCG1, AATF, WWP1, VGF, TBPL1, SUFU, STAG1, ST8SIA1, SLC5A6, SLC25A22, SLC12A6, SEPT7, SEMA3B, REV3L, PTGES3, PRMT5, PPP1R16B, PNKD, PDCD10, PCP2, NRM, NFIL3, NBPF1, MEA1, MAP3K8, LRBA, LMNA, IRF8, IL11RA, IFNGR1, HSPE1, HNRNPK, FRS3, FRAG1, FBXW7, FBL, EDAR, DDIT4, CNTFR, CD226, CAD, BECN1,

BCLAF1, BANF1, ACAA1, ZYX, VCP, TMSB4X, TLX2, TLE4, TCIRG1, TBX15, TBK1, TAX1BP1, SSBP1, SPSB2, SPRY4, SP4, SLC26A8, SERPINI1, ROCK1, RGS16, REV1, RBBP6, RASGRP1, RAD51L1, PTPN22, PRCP, PPP3R1, POFUT1, PLAGL2, PEG10, PCSK7, PAX9, P2RX3, OTX1, OSGEP, NR1H2, NOG, NLRP3, NAB1, MTP18, MAP3K11, MAP1A, JMJD2C, JARID1B, IVNS1ABP, ITGA7, ITGA1, INTS6, IMPDH2, IMP3, IL10RA, IFITM1, IFIH1, IDH1, HS2ST1, HNRNPU, HNRNPD, HLA-B, HIST1H2AE, HIPK3, HIPK1, HAX1, GSX1, FXYD5, FUBP1, ELF5, ELAVL4, ECE1, DUT, DR1, DNAJC14, CXCR7, CST6, CSRP1, CRIP2, CLCN2, CLCF1, CEBPE, CD53, CD200R1, BPGM, BBC3, AIM1, ACTR3, ACSL5, ABCF2, ZIK1, ZBTB6, YES1, WDR26, USO1, UNC13D, TYROBP, TUBGCP2, TTC9, TPMT, TOPBP1, TMEM102, TGFBRAP1, ST6GAL1, SSR1, SRP54, SPRED2, SOX13, SLC23A1, SIM1, SIAH2, SENP1, SACM1L, RCHY1, RBKS, RAB6A, RAB27A, PVRL3, PRPF4B, POU2AF1, POMGNT1, PLEKHG5, PFDN5, PER2, OVCA2, OPTN, NXPH4, NUP37, NR2E1, NMT1, NCKAP1L, MTMR11, MRO, MAGI3, LTBP2, LIG4, KIF2C, KCTD10, KCNN4, ITPR2, HSF4, HS6ST1, HLA-DMA, HLA-C, HIST1H2AM, HCST, H3F3A, GYPC, FGF14, EXOSC7, ERGIC2, EPB41L2, EPB41, EMP1, ELL2, EIF5B, EEF2, EEF1A1, DYNLRB1, DUSP4, DIDO1, DHH, DEDD2, DEDD, DDX1, CTSC, CSNK1A1, CLDN12, CHSY1, CHIC2, CEP57, CCDC72, C7orf23, C1orf103, C10orf4, BRCA2, BID, BACH1, ARMCX2, APPL1, ANKHD1, ADIPOR2, AAMP

Corresponding total number of TFBS:

(For each gene listed above, the total number of TFBS for any of the selected TFs, multiplied by the number of selected Gene ontology terms containing that gene)

374, 264, 204, 198, 192, 176, 168, 156, 132, 126, 121, 121, 120, 120, 114, 110, 110, 108, 108, 108, 105, 99, 96, 96, 96, 91, 90, 90, 88, 88, 84, 84, 84, 84, 84, 84, 84, 80, 77, 77, 72, 72, 72, 72, 72, 72, 72, 72, 72, 72, 72, 70, 70, 70, 70, 66, 66, 66, 66, 66, 64, 64, 63, 63, 60, 60, 60, 60, 60, 60, 60, 60, 60, 60, 60, 60, 60, 60, 60, 56, 56, 56, 56, 56, 55, 55, 55, 55, 55, 55, 54, 54, 54, 50, 50, 50, 50, 50, 48, 48, 48, 48, 48, 48, 48, 48, 48, 45, 45, 45, 45, 45, 44, 44, 44, 44, 42, 42, 42, 42, 42, 42, 42, 42, 42, 40, 40, 40, 40, 40, 40, 40, 40, 40, 39, 36, 36, 36, 36, 36, 36, 36, 36, 36, 36, 36, 36, 36, 35, 35, 35, 35, 33, 33, 33, 33, 33, 33, 33, 33, 32, 32, 32, 32, 32, 30, 30, 30, 30, 30, 30, 30, 30, 30, 30, 28, 28, 28, 28, 28, 28, 28, 27, 27, 27, 27, 27, 25, 25, 25, 25, 24, 24, 24, 24, 24, 24, 24, 24, 24, 24, 24, 24, 24, 24, 24, 24, 24, 24, 24, 24, 24, 24, 24, 24, 24, 24, 24, 24, 24, 24, 24, 24, 24, 24, 24, 24, 24, 24, 24, 24, 24, 24, 24, 24, 24, 22, 22, 22, 22, 22, 22, 22, 22, 22, 22, 22, 22, 22, 22, 22, 21, 21, 21, 21, 21, 21, 20, 20, 20, 20, 20, 20, 20, 20, 20, 20, 20, 20, 20, 20, 18, 18, 18, 18, 18, 18, 18, 18, 18, 18, 18, 18, 18, 18, 18, 18, 18, 18, 18, 16, 16, 16, 16, 16, 16, 16, 16, 16, 16, 16, 16, 16, 15, 15, 15, 15, 15, 15, 15, 15, 15, 15, 15, 14, 14, 14, 14, 14, 14, 14, 14, 14, 14, 13, 13, 13, 12, 12, 12, 12, 12, 12, 12, 12, 12, 12, 12, 12, 12, 12, 12, 12, 12, 12, 12, 12, 12, 12, 12, 12, 12, 12, 12, 12, 12, 12, 12, 12, 12, 12, 12, 12, 12, 12, 12, 12, 12, 12, 12, 12, 12, 12, 12, 12, 12, 12, 12, 12, 11, 11, 11, 11, 11, 11, 11, 11, 11, 11, 11, 11, 11, 11, 11, 11, 11, 11, 11, 11, 11, 11, 10, 10, 10, 10, 10, 10, 10, 10, 10, 10, 10, 10, 10, 10, 10, 10, 10, 10, 10, 10, 10, 10, 10, 10, 10, 10, 9, 9, 9, 9, 9, 9, 9, 9, 9, 9, 9, 9, 9, 9, 9, 9, 9, 9, 9, 9, 9, 9, 9, 9, 9, 9, 9, 8, 8, 8, 8, 8, 8, 8, 8, 8, 8, 8, 8, 8, 8, 8, 8, 8, 8, 8, 8, 8, 8, 8, 8, 8, 8, 8, 8, 8, 8, 8, 8, 8, 8, 8, 8, 8, 8, 8, 8, 8, 7, 7, 7, 7, 7, 7, 7, 7, 7, 7, 7, 7, 7, 7, 7, 7, 7, 7, 7, 7, 7, 7, 6, 6, 6, 6, 6, 6, 6, 6, 6, 6, 6, 6, 6, 6, 6, 6, 6, 6, 6, 6, 6, 6, 6, 6, 6, 6, 6, 6, 6, 6, 6, 6, 6, 6, 6, 6, 6, 6, 6, 6, 6, 6, 5, 5, 5, 5, 5, 5, 5, 5, 5, 5, 5, 5, 5, 5, 5, 5, 5, 5, 5, 5, 5, 5, 5, 5, 5, 5, 5, 5, 5, 5, 5, 5, 5, 4, 4, 4, 4, 4, 4, 4, 4, 4, 4, 4, 4, 4, 4, 4, 4, 4, 4, 4, 4, 4, 4, 4, 4, 4, 4, 4, 4, 4, 4, 4, 4, 4, 4, 4, 4, 4, 4, 4, 4, 4, 4, 4, 4, 4, 4, 4, 4, 3, 3, 3, 3, 3, 3, 3, 3, 3, 3, 3, 3, 3, 3, 3, 3, 3, 3, 3, 3, 3, 3, 3, 3, 3, 3, 3, 3, 3, 3, 3, 3, 3, 3, 3, 3, 3, 3, 3, 3, 3, 3, 3, 2, 2, 2, 2, 2, 2, 2, 2, 2, 2, 2, 2, 2, 2, 2, 2, 2, 2, 2, 2, 2, 2, 2, 2, 2, 2, 2, 2, 2, 2, 2, 2, 2, 2, 2, 2, 2, 2, 2, 2, 2, 2, 2, 2, 2, 2, 2, 2, 2, 2, 2, 2, 2, 2, 2, 2, 2, 2, 2, 2, 2, 2, 2, 2, 2, 2, 2, 2, 2, 2, 2, 2, 2, 2, 2, 2, 2, 2, 2, 2, 2, 1, 1, 1, 1, 1, 1, 1, 1, 1, 1, 1, 1, 1, 1, 1, 1, 1, 1, 1, 1, 1, 1, 1, 1, 1, 1, 1, 1, 1, 1, 1, 1, 1, 1, 1, 1, 1, 1, 1, 1, 1, 1, 1, 1, 1, 1, 1, 1, 1, 1, 1, 1, 1, 1, 1, 1, 1, 1, 1, 1, 1, 1, 1, 1, 1, 1, 1, 1, 1, 1, 1, 1, 1, 1, 1, 1, 1, 1, 1, 1, 1, 1, 1, 1, 1, 1, 1, 1, 1, 1, 1, 1, 1

Corresponding number of selected Gene ontology terms each gene is involved with:

(In the same order as above)

34, 33, 17, 22, 16, 16, 14, 13, 11, 14, 11, 11, 10, 10, 19, 11, 10, 9, 9, 9, 15, 9, 8, 8, 8, 13, 10, 9, 8, 8, 7, 7, 7, 7, 7, 7, 7, 16, 7, 7, 6, 6, 18, 6, 12, 6, 6, 6, 8, 8, 6, 7, 7, 7, 7, 6, 6, 6, 6, 6, 16, 8, 7, 7, 20, 15, 5, 5, 12, 5, 5, 5, 12, 5, 5, 5, 5, 5, 15, 8, 8, 8, 7, 14, 5, 5, 5, 5, 5, 5, 6, 9, 6, 10, 5, 5, 5, 5, 8, 4, 4, 4, 12, 6, 4, 6, 4, 9, 9, 5, 5, 9, 4, 4, 4, 4, 6, 14, 6, 7, 7, 6, 6, 6, 6, 5, 4, 5, 5, 4, 4, 5, 10, 5, 13, 3, 3, 4, 3, 18, 3, 3, 3, 3, 3, 3, 6, 3, 5, 7, 5, 7, 11, 3, 3, 3, 3, 3, 3, 3, 8, 8, 4, 4, 4, 5, 15, 3, 3, 3, 3, 10, 3, 15, 3, 4, 4, 4, 4, 4, 4, 4, 3, 9, 9, 3, 3, 5, 5, 5, 5, 2, 2, 2, 2, 3, 2, 2, 3, 2, 2, 2, 6, 2, 2, 2, 3, 2, 2, 4, 2, 4, 2, 2, 2, 2, 2, 3, 2, 6, 2, 2, 2, 2, 2, 2, 6, 2, 2, 2, 3, 2, 2,

2, 2, 2, 11, 11, 2, 2, 2, 11, 2, 2, 2, 2, 2, 2, 2, 2, 2, 3, 21, 7, 7, 7, 3, 4, 2, 2, 2, 10, 2, 5, 10, 4, 2, 2, 5, 2, 4, 6, 2, 2, 3, 9, 2, 2, 9, 2, 2, 3, 9, 2, 6, 2, 6, 2, 3, 18, 2, 2, 4, 2, 4, 2, 2, 8, 4, 16, 2, 2, 4, 5, 3, 3, 3, 3, 3, 3, 5, 3, 3, 5, 2, 2, 2, 2, 2, 2, 14, 7, 2, 2, 13, 13, 13, 1, 1, 3, 1, 3, 1, 6, 6, 2, 1, 1, 1, 1, 12, 1, 6, 6, 6, 1, 2, 1, 1, 1, 6, 3, 1, 1, 1, 1, 1, 1, 4, 1, 1, 12, 1, 3, 6, 6, 6, 3, 4, 6, 6, 2, 1, 3, 1, 1, 11, 1, 1, 1, 1, 1, 1, 11, 1, 1, 1, 1, 1, 1, 1, 1, 1, 1, 1, 1, 1, 1, 1, 1, 1, 1, 10, 1, 1, 1, 5, 5, 2, 2, 2, 5, 2, 2, 1, 1, 1, 1, 2, 1, 1, 2, 2, 1, 1, 1, 3, 1, 1, 9, 1, 9, 1, 3, 1, 3, 1, 1, 3, 1, 3, 1, 1, 1, 1, 3, 3, 1, 1, 1, 3, 8, 2, 2, 2, 1, 8, 1, 2, 8, 8, 4, 2, 1, 4, 1, 2, 1, 4, 2, 4, 8, 1, 2, 4, 8, 2, 4, 2, 8, 4, 4, 8, 4, 1, 1, 4, 1, 4, 4, 4, 4, 1, 7, 1, 1, 1, 1, 1, 7, 7, 7, 1, 1, 7, 7, 1, 1, 7, 1, 1, 7, 1, 7, 1, 1, 2, 3, 6, 3, 3, 2, 1, 6, 1, 6, 3, 6, 1, 2, 2, 6, 3, 3, 1, 6, 3, 2, 1, 1, 1, 3, 3, 1, 1, 2, 3, 2, 2, 1, 3, 1, 6, 1, 3, 1, 1, 5, 5, 1, 5, 5, 1, 5, 5, 1, 5, 1, 5, 5, 1, 1, 1, 5, 5, 5, 1, 5, 5, 5, 1, 1, 1, 5, 5, 1, 5, 1, 1, 1, 2, 1, 4, 4, 2, 1, 4, 1, 1, 2, 2, 1, 4, 1, 1, 1, 1, 2, 2, 4, 2, 1, 4, 4, 4, 1, 4, 1, 1, 1, 1, 1, 1, 1, 1, 4, 1, 1, 4, 4, 4, 2, 1, 1, 2, 2, 2, 3, 3, 3, 1, 1, 1, 1, 1, 1, 3, 3, 1, 3, 1, 1, 3, 1, 1, 1, 3, 3, 1, 3, 3, 3, 3, 1, 3, 3, 3, 3, 3, 3, 3, 3, 3, 1, 3, 3, 1, 3, 1, 1, 2, 2, 2, 2, 1, 1, 2, 2, 2, 1, 2, 2, 1, 1, 2, 2, 2, 2, 2, 1, 2, 2, 1, 1, 1, 1, 1, 2, 2, 2, 1, 2, 2, 2, 2, 1, 2, 1, 2, 1, 1, 1, 2, 1, 1, 2, 2, 2, 2, 1, 2, 2, 1, 1, 1, 1, 2, 2, 2, 1, 2, 2, 1, 2, 2, 2, 2, 1, 2, 2, 2, 2, 2, 1, 2, 2, 1, 2, 2, 2, 2, 1, 1, 1, 1, 1, 1, 1, 1, 1, 1, 1, 1, 1, 1, 1, 1, 1, 1, 1, 1, 1, 1, 1, 1, 1, 1, 1, 1, 1, 1, 1, 1, 1, 1, 1, 1, 1, 1, 1, 1, 1, 1, 1, 1, 1, 1, 1, 1, 1, 1, 1, 1, 1, 1, 1, 1, 1, 1, 1, 1, 1, 1, 1, 1, 1, 1, 1, 1, 1, 1, 1, 1, 1, 1, 1, 1, 1, 1, 1, 1, 1, 1, 1, 1, 1, 1, 1, 1, 1, 1, 1, 1, 1

Corresponding number of selected TFs each gene is involved with:
(In the same order as above)
11, 8, 12, 9, 12, 11, 12, 12, 12, 9, 11, 11, 12, 12, 6, 10, 11, 12, 12, 12, 7, 11, 12, 12, 12, 7, 9, 10, 11, 11, 12, 12, 12, 12, 12, 12, 12, 5, 11, 11, 12, 12, 4, 12, 6, 12, 12, 12, 9, 9, 12, 10, 10, 10, 10, 11, 11, 11, 11, 11, 4, 8, 9, 9, 3, 4, 12, 12, 5, 12, 12, 12, 5, 12, 12, 12, 12, 12, 4, 7, 7, 7, 8, 4, 11, 11, 11, 11, 11, 11, 9, 6, 9, 5, 10, 10, 10, 10, 6, 12, 12, 12, 4, 8, 12, 8, 12, 5, 5, 9, 9, 5, 11, 11, 11, 11, 7, 3, 7, 6, 6, 7, 7, 7, 7, 8, 10, 8, 8, 10, 10, 8, 4, 8, 3, 12, 12, 9, 12, 2, 12, 12, 12, 12, 12, 12, 6, 12, 7, 5, 7, 5, 3, 11, 11, 11, 11, 11, 11, 11, 4, 4, 8, 8, 8, 6, 2, 10, 10, 10, 10, 3, 10, 2, 10, 7, 7, 7, 7, 7, 7, 7, 9, 3, 3, 9, 9, 5, 5, 5, 5, 12, 12, 12, 12, 8, 12, 12, 8, 12, 12, 12, 4, 12, 12, 12, 8, 12, 12, 6, 12, 6, 12, 12, 12, 12, 12, 8, 12, 4, 12, 12, 12, 12, 12, 12, 4, 12, 12, 12, 8, 12, 12, 12, 12, 12, 2, 2, 11, 11, 11, 2, 11, 11, 11, 11, 11, 11, 11, 11, 11, 7, 1, 3, 3, 3, 7, 5, 10, 10, 10, 2, 10, 4, 2, 5, 10, 10, 4, 10, 5, 3, 9, 9, 6, 2, 9, 9, 2, 9, 9, 6, 2, 9, 3, 9, 3, 9, 6, 1, 8, 8, 4, 8, 4, 8, 8, 2, 4, 1, 8, 8, 4, 3, 5, 5, 5, 5, 5, 5, 3, 5, 5, 3, 7, 7, 7, 7, 7, 7, 1, 2, 7, 7, 1, 1, 1, 12, 12, 4, 12, 4, 12, 2, 2, 6, 12, 12, 12, 12, 1, 12, 2, 2, 2, 12, 6, 12, 12, 12, 2, 4, 12, 12, 12, 12, 12, 12, 3, 12, 12, 1, 12, 4, 2, 2, 2, 4, 3, 2, 2, 6, 12, 4, 12, 12, 1, 11, 11, 11, 11, 11, 11, 1, 11, 11, 11, 11, 11, 11, 11, 11, 11, 11, 11, 11, 11, 11, 10, 10, 10, 10, 1, 10, 10, 10, 2, 2, 5, 5, 5, 2, 5, 5, 10, 10, 10, 10, 5, 10, 10, 5, 5, 10, 9, 9, 3, 9, 9, 1, 9, 1, 9, 3, 9, 3, 9, 9, 3, 9, 3, 9, 9, 9, 9, 3, 3, 9, 9, 9, 3, 1, 4, 4, 4, 8, 1, 8, 4, 1, 1, 2, 4, 8, 2, 8, 4, 8, 2, 4, 2, 1, 8, 4, 2, 1, 4, 2, 4, 1, 2, 2, 1, 2, 8, 8, 2, 8, 2, 2, 2, 2, 7, 1, 7, 7, 7, 7, 7, 1, 1, 1, 7, 7, 1, 1, 7, 7, 1, 7, 7, 1, 7, 1, 6, 6, 3, 2, 1, 2, 2, 3, 6, 1, 6, 1, 2, 1, 6, 3, 3, 1, 2, 2, 6, 1, 2, 3, 6, 6, 6, 2, 2, 6, 6, 3, 2, 3, 3, 6, 2, 6, 1, 6, 2, 6, 5, 1, 1, 5, 1, 1, 5, 1, 1, 5, 1, 5, 1, 1, 5, 5, 5, 1, 1, 1, 5, 1, 1, 1, 5, 5, 5, 1, 1, 5, 1, 5, 5, 4, 2, 4, 1, 1, 2, 4, 1, 4, 4, 2, 2, 4, 1, 4, 4, 4, 4, 2, 2, 1, 2, 4, 1, 1, 1, 4, 1, 4, 4, 4, 4, 4, 4, 4, 4, 1, 4, 4, 1, 1, 1, 2, 4, 4, 2, 2, 2, 1, 1, 1, 3, 3, 3, 3, 3, 3, 1, 1, 3, 1, 3, 3, 1, 3, 3, 3, 1, 1, 3, 1, 1, 1, 1, 3, 1, 1, 1, 1, 1, 1, 1, 1, 1, 3, 1, 1, 3, 1, 3, 2, 1, 1, 1, 1, 2, 2, 1, 1, 1, 2, 1, 1, 2, 2, 1, 1, 1, 1, 1, 2, 1, 1, 2, 2, 2, 2, 2, 1, 1, 1, 2, 1, 1, 1, 1, 2, 1, 2, 1, 2, 2, 2, 1, 2, 2, 1, 1, 1, 1, 2, 1, 1, 2, 2, 2, 2, 1, 1, 1, 2, 1, 1, 2, 1, 1, 1, 1, 2, 1, 1, 1, 1, 1, 2, 1, 1, 2, 1, 1, 1, 1, 2, 1, 1, 1, 1, 1, 1, 1, 1, 1, 1, 1, 1, 1, 1, 1, 1, 1, 1, 1, 1, 1, 1, 1, 1, 1, 1, 1, 1, 1, 1, 1, 1, 1, 1, 1, 1, 1, 1, 1, 1, 1, 1, 1, 1, 1, 1, 1, 1, 1, 1, 1, 1, 1, 1, 1, 1, 1, 1, 1, 1, 1, 1, 1, 1, 1, 1, 1, 1, 1, 1, 1, 1, 1, 1, 1, 1, 1, 1, 1, 1, 1, 1, 1

Gene ontology terms ranked according to mean of effect sizes in cluster:
neural tube formation | provirus | neuron development | cell fate specification | kidney development | gut development | regulation of cell proliferation | transcription factor complex | cartilage development | apoptosis | cell proliferation | luciferin monooxygenase activity | osteoblast differentiation | mesoderm formation | developmental process | lung development | odontogenesis | myoblast differentiation | chondrocyte differentiation | heart development | decidualization | transdifferentiation | N-acetyltransferase activity | gliogenesis | liver development | stem cell differentiation | neural tube closure | system development | ossification | eye development | camera-type eye development | compound eye development | fibroblast growth factor receptor binding | skeletal muscle development | fibroblast growth factor receptor activity | cell-cell signaling | skeletal development | induction | lens development in camera-type eye | epithelial to mesenchymal transition | growth | cell | response to osmotic stress | neuron migration

Corresponding mean of effect sizes of each Gene ontology term:

(In the same order as above)
1.84, 1.78, 1.55, 1.49, 1.46, 1.45, 1.42, 1.33, 1.31, 1.31, 1.30, 1.28, 1.24, 1.22, 1.16, 1.16, 1.13, 1.12, 1.10, 1.01, 0.96, 0.88, 0.84, 0.83, 0.81, 0.76, 0.75, 0.73, 0.70, 0.67, 0.67, 0.66, 0.62, 0.62, 0.58, 0.58, 0.57, 0.49, 0.45, 0.41, 0.37, 0.23, 0.21, -0.13

Corresponding total number of genes of each Gene ontology term:
(In the same order as above)
79, 135, 69, 74, 139, 54, 165, 117, 199, 1.9K, 1.5K, 289, 239, 66, 26, 159, 122, 112, 135, 144, 117, 204, 19, 65, 100, 93, 75, 21, 283, 140, 140, 141, 182, 64, 193, 26, 133, 2.1K, 80, 229, 2.2K, 4.1K, 46, 187

Corresponding number of genes of each Gene ontology term with at least one TFBS (of selected TFs):
(In the same order as above)
25, 41, 21, 19, 29, 9, 49, 41, 40, 360, 288, 73, 61, 19, 6, 34, 24, 31, 31, 28, 21, 43, 3, 13, 21, 17, 21, 6, 50, 25, 25, 25, 47, 13, 49, 7, 21, 364, 15, 53, 372, 637, 12, 22

Corresponding total number of TFBS (of selected TFs) for each Gene ontology term (in all genes):
(In the same order as above)
170, 241, 136, 147, 175, 81, 270, 212, 240, 2033, 1624, 359, 320, 101, 52, 201, 158, 177, 176, 198, 134, 245, 24, 88, 112, 115, 103, 41, 253, 151, 151, 151, 229, 85, 223, 40, 142, 1954, 93, 272, 1984, 3486, 50, 167

Corresponding number of selected TFs each Gene ontology term is involved with:
(In the same order as above)
12, 12, 12, 12, 12, 12, 12, 12, 12, 12, 12, 12, 12, 12, 12, 12, 12, 12, 12, 12, 12, 12, 12, 12, 12, 12, 12, 12, 12, 12, 12, 12, 12, 12, 12, 12, 12, 12, 12, 12, 12, 12, 12, 12

TFs ranked according to mean of effect sizes in cluster:
V$PAX2_02, V$TST1_01, V$HMGIY_Q6, V$STAT1_03, V$NFAT_Q6, V$STAT6_01, V$STAT5A_03, V$NFAT_Q4_01, V$HOXA4_Q2, V$STAT4_01, V$STAT5A_04, V$CEBP_Q3

Corresponding mean of effect sizes of each TF:
(In the same order as above)
1.27, 1.16, 1.15, 1.03, 1.02, 1.02, 0.99, 0.83, 0.79, 0.72, 0.61, 0.58

Corresponding total number of TFBS for each TF (genome-wide):
(In the same order as above)
741, 730, 753, 751, 719, 732, 749, 736, 719, 743, 740, 744

Corresponding total number of TFBS for each TF (in all genes in selected Gene ontology terms):
(In the same order as above)
1549, 1452, 1488, 1424, 1524, 1459, 1462, 1444, 1361, 1440, 1322, 1439

Corresponding number of genes (of selected Gene ontology terms) each TF is involved with:
(In the same order as above)
392, 378, 387, 386, 390, 387, 399, 392, 370, 391, 380, 374

Corresponding number of selected Gene ontology terms each TF is involved with:
(In the same order as above)
44, 44, 44, 44, 44, 44, 44, 44, 44, 44, 44, 44

Cluster for columns 425 to 431, rows 139 to 151

Gene ontology terms

specific granule | phagocytosis | respiratory burst | piecemeal microautophagy of nucleus | neutrophil degranulation | neutrophil activation | neutrophil chemotaxis

TFs

V$SMAD4_Q6 | F$STRE_01 | V$SREBP1_02 | V$MYOGNF1_01 | V$PEA3_Q6 | V$ETS_Q6 | V$PU1_Q6 | V$PXR_Q2 | V$TEL2_Q6 | V$ETS_Q4 | V$ELF1_Q6 | V$NKX25_Q5 | V$ETS2_B

Information

All related TFs:
(List of all TFs that are related to any of the PWMs)
CAR, CAR2:RXR-alpha, CAR:RXR-alpha, ELF-1, ELFR, ERF, Elf-1, Elk-1, Elk-1-isoform1, Erg-1, Erg-2, Ets-1, FXR, FXR:RXR-alpha, Fli-1, GABP-alpha, GABP-alpha:GABP-beta, GABP-beta1, GABP-beta2, LXR-alpha:RXR-alpha, LXR-beta:RXR-alpha, NERF, NERF-1a, NERF-1b, NERF-2, NF-1, NF-1/L, NF-1/Red1, NF-1A1, NF-1B1, NF-1B2, NF-1C2, NF-1X, Net, Nkx2-5, Nkx2.5, PEA3, PU.1, PU.1-xbb1, PXR-1, PXR-1A, PXR-2, PXR:RXR-alpha, SAP-1a, SAP-1b, SREBP-1, SREBP-1a, SREBP-1b, SREBP-1c, Smad4, Spi-B, TCF, TEL1, Tel-2a, Tel-2b, Tel-2c, Tel-2d, Tel-2e, Tel-2f, c-Ets-1, c-Ets-1A, c-Ets-1B, c-Ets-2, c-Ets-2A, c-Ets-2B, p38erg, p49erg, p55, p55erg

Ranked gene list:
(All genes of the selected Gene ontology terms with hits of any of the selected TFs, ranked according to the total number of TFBS)
TNF, LTB, ITGB2, FGR, PER1, AKT1, RAC2, LTBR, PTPN6, CD63, MMP9, TNFSF13, PML, VAV1, GUSB, TLR9, PLA2G6, MTHFR, CSF1, RARA, IRF1, MAPK14, NCF4, ISYNA1, CORO1B, CCR7, TNFRSF1A, SHC1, RHOG, PIK3CG, LTB4R, LTA, ITGA5, FASN, CORO1A, CD68, SELL, PHB2, LSP1, HOXA10, CIC, ALOX5, TYROBP, SPN, MYO1C, KIAA0101, IRF6, INPP5D, CALR, CALM2, CTSD, CLCF1, CEBPE, AHSA1, ST3GAL4, NOS2, LYN, ING1, ICAM2, HGS, FAS, ELMO3, DCTN3, CLEC11A, CD4, CAPG, SRC, SELPLG, PTK2B, PRKCSH, PDE4A, MARCKSL1, IL4, IL3, F2, ELMO1, DPEP1, CYBA, CDK9, CD44, CD2BP2, CALM3, CALM1, ARHGDIA, WIPF1, WDR26, RHOA, RERE, PXN, P2RY2, MBP, LGALS3, ITGAV, ITGA4, IL13, ICAM3, GMFG, EPHB2, ENPP3, CD46, C4B, BCKDHA, ADRM1, ACTR3, VSIG4, VAV3, TRPM2, TFRC, STAT3, SLC5A5, PRKCA, PLD2, PIGR, NOXO1, NDUFAB1, MYST1, MYD88, MTG1, MMP2, LAMP1, KAT5, HPN, GFAP, ELMO2, DAGLA, CUX1, CTRL, CRK, CERK, CEBPA, CD40, CCL24, CASP1, CAMK1D, BSG, ARF6, AIF1

Corresponding total number of TFBS:
(For each gene listed above, the total number of TFBS for any of the selected TFs, multiplied by the number of selected Gene ontology terms containing that gene)
84, 72, 63, 50, 48, 48, 42, 28, 26, 25, 24, 20, 20, 14, 14, 12, 12, 12, 12, 11, 11, 10, 9, 9, 9, 9, 8, 8, 8, 8, 8, 8, 8, 8, 8, 8, 7, 7, 7, 7, 7, 7, 6, 6, 6, 6, 6, 6, 6, 6, 5, 5, 5, 5, 4, 4, 4, 4, 4, 4, 4, 4, 4, 4, 4, 4, 3, 3, 3, 3, 3, 3, 3, 3, 3, 3, 3, 3, 3, 3, 3, 3, 3, 3, 2, 2, 2, 2, 2, 2, 2, 2, 2, 2, 2, 2, 2, 2, 2, 2, 2, 2, 2, 1, 1, 1, 1, 1, 1, 1, 1, 1, 1, 1, 1, 1, 1, 1, 1, 1, 1, 1, 1, 1, 1, 1, 1, 1, 1, 1, 1, 1, 1

Corresponding number of selected Gene ontology terms each gene is involved with:
(In the same order as above)
7, 6, 7, 5, 4, 4, 6, 7, 2, 5, 4, 5, 4, 2, 7, 2, 3, 6, 2, 1, 1, 5, 3, 3, 1, 1, 1, 1, 1, 4, 4, 1, 1, 1, 1, 1, 7, 1, 1, 1, 1, 7, 1, 3, 1, 6, 6, 1, 1, 3, 1, 1, 1, 5, 2, 4, 4, 4, 1, 2, 2, 1, 1, 4, 1, 2, 3, 1, 1, 1, 1, 1, 3, 3, 3, 1, 1, 3, 1, 1, 1, 3, 3, 1, 1, 2, 1, 2, 2, 2, 2, 1, 1, 2, 2, 1, 1, 2, 1, 2, 1, 2, 1, 1, 1, 1, 1, 1, 1, 1, 1, 1, 1, 1, 1, 1, 1, 1, 1, 1, 1, 1, 1, 1, 1, 1, 1, 1, 1, 1, 1, 1, 1, 1, 1, 1, 1, 1

Corresponding number of selected TFs each gene is involved with:

(In the same order as above)
12, 12, 9, 10, 12, 12, 7, 4, 13, 5, 6, 4, 5, 7, 2, 6, 4, 2, 6, 11, 11, 2, 3, 3, 9, 9, 8, 8, 8, 2, 2, 8, 8, 8, 8, 8, 1, 7, 7, 7, 7, 1, 6, 2, 6, 1, 1, 6, 6, 2, 5, 5, 5, 1, 2, 1, 1, 1, 4, 2, 2, 4, 4, 1, 4, 2, 1, 3, 3, 3, 3, 3, 1, 1, 1, 3, 3, 1, 3, 3, 3, 1, 1, 3, 2, 1, 2, 1, 1, 1, 1, 2, 2, 1, 1, 2, 2, 1, 2, 1, 2, 1, 2, 2, 1, 1, 1, 1, 1, 1, 1, 1, 1, 1, 1, 1, 1, 1, 1, 1, 1, 1, 1, 1, 1, 1, 1, 1, 1, 1, 1, 1, 1, 1, 1, 1

Gene ontology terms ranked according to mean of effect sizes in cluster:
specific granule | neutrophil degranulation | phagocytosis | respiratory burst | neutrophil activation | neutrophil chemotaxis | piecemeal microautophagy of nucleus

Corresponding mean of effect sizes of each Gene ontology term:
(In the same order as above)
2.12, 1.84, 1.67, 1.51, 1.32, 1.31, 1.30

Corresponding total number of genes of each Gene ontology term:
(In the same order as above)
62, 52, 304, 195, 147, 86, 200

Corresponding number of genes of each Gene ontology term with at least one TFBS (of selected TFs):
(In the same order as above)
21, 17, 83, 59, 35, 25, 50

Corresponding total number of TFBS (of selected TFs) for each Gene ontology term (in all genes):
(In the same order as above)
86, 64, 296, 215, 135, 96, 167

Corresponding number of selected TFs each Gene ontology term is involved with:
(In the same order as above)
13, 13, 13, 13, 13, 13, 13

TFs ranked according to mean of effect sizes in cluster:
V$ETS2_B, V$PU1_Q6, VETS_Q6, VPXR_Q2, V$PEA3_Q6, V$ETS_Q4, V$TEL2_Q6, F$STRE_01, V$SREBP1_02, V$ELF1_Q6, V$NKX25_Q5, V$SMAD4_Q6, V$MYOGNF1_01

Corresponding mean of effect sizes of each TF:
(In the same order as above)
2.14, 2.14, 2.03, 1.87, 1.83, 1.65, 1.46, 1.37, 1.33, 1.29, 1.25, 1.16, 1.05

Corresponding total number of TFBS for each TF (genome-wide):
(In the same order as above)
722, 724, 721, 684, 717, 718, 707, 651, 693, 717, 695, 666, 654

Corresponding total number of TFBS for each TF (in all genes in selected Gene ontology terms):
(In the same order as above)
94, 94, 87, 90, 89, 83, 80, 70, 79, 80, 72, 69, 72

Corresponding number of genes (of selected Gene ontology terms) each TF is involved with:
(In the same order as above)
41, 39, 40, 42, 40, 36, 35, 31, 33, 34, 33, 30, 27

Corresponding number of selected Gene ontology terms each TF is involved with:
(In the same order as above)
7, 7, 7, 7, 7, 7, 7, 7, 7, 7, 7, 7, 7

Cluster for columns 172 to 185, rows 199 to 203

Gene ontology terms

isochorismate pyruvate lyase activity | axon | dendrite | synapse | synaptogenesis | exocytosis | synaptic transmission | postsynaptic membrane | receptor clustering | postsynaptic density | N-methyl-D-aspartate selective glutamate receptor activity | dendritic spine | receptor activity | synaptic transmission, glutamatergic

TFs

V$TFIII_Q6 | V$MZF1_01 | V$VDR_Q3 | V$MZF1_02 | V$SMAD_Q6_01

Information

All related TFs:
(List of all TFs that are related to any of the PWMs)
MZF1B-C, Smad1, Smad1.1, Smad10, Smad2, Smad2-L, Smad3, Smad3:Smad4, Smad4, Smad4alpha, Smad4beta, Smad4delta3, Smad4delta4-6, Smad4delta4-7, Smad4delta5-6, Smad4delta6, Smad5, Smad6, Smad7, Smad8, TFII-I, VDR

Ranked gene list:
(All genes of the selected Gene ontology terms with hits of any of the selected TFs, ranked according to the total number of TFBS)
DLG4, PPP1R9B, CDK5, MGAT1, AKT1, PSD, GRIN2D, ACHE, FBXL15, SYNGAP1, AGRN, CAMK2G, ARHGEF2, SLC17A7, SHANK3, VAMP2, PNMT, PER1, GRM2, TNFSF13, RHBDL1, NDOR1, GRIN2C, PPP1R1B, GIT1, EFNA3, ITGB2, VGF, NRGN, NLGN2, GUK1, GABARAP, EGR1, EFNA2, BRD2, TPM3, SV2A, SLC2A4RG, SHANK1, FASN, STXBP2, SLC25A22, SEMA3B, RARA, OPRL1, NELF, JUNB, ISYNA1, COMP, CACNA1G, p63RhoGEF, TNFRSF1A, TFAP2A, SYT7, SLC22A18, SEPT5, RTN4RL2, REPIN1, MUC1, LBX1, KCNC3, GRIK5, GABBR1, ENO2, DVL1, DKFZp434P0672, CTSD, CNTNAP1, CAMKK1, BAIAP3, B3GAT1, ACCN2, TAS1R3, SYT8, SLC9A3R1, SLC12A5, RTN4RL1, RNASEK, PLXNA1, PGP, PARD6A, NGFR, MAPRE3, MAP1A, LASP1, KCNC4, IL23A, ACD, ACCN4, ZIC2, ZAP70, UNC13D, SYTL1, SQSTM1, SEMA3F, SCAMP2, RAB25, PTPRU, PTPRF, PLCG1, PAX6, NR3C2, MEA1, MC1R, LPHN1, KIAA1684, JUN, HS6ST1, FLJ32987, FHL3, ERBB3, ENTPD2, DNM2, DAGLA, CTBP2, CPLX1, CHRNE, CDKN1C, CD81, CD63, CD44, ADM2, ADM, ACTB, VIM, UBE4B, SYNJ2, STX6, SRRM2, SLC9A3R2, SLC12A2, SEMA7A, SCRIB, RAB37, PTGIR, PSMP, PNPLA6, PELP1, OPRS1, NRTN, NCOR2, MPZ, MAP3K11, MAP2K3, LCK, KLF7, KIF5A, KCNJ11, IGF1R, ICAM5, HRH3, GRN, GRIN3B, GRASP, GAD1, FOSB, EPHB3, EPB41, DPYSL3, CTNNBIP1, CTDSPL, CSAD, CORO1A, CHPT1, CAPN10, ATP6V0D1, ARL6IP5, ARID4B, ARF1, APEH, AGRP, ABR

Corresponding total number of TFBS:
(For each gene listed above, the total number of TFBS for any of the selected TFs, multiplied by the number of selected Gene ontology terms containing that gene)
52, 40, 40, 35, 35, 32, 30, 28, 27, 24, 24, 20, 20, 18, 18, 15, 15, 15, 13, 12, 12, 12, 12, 10, 10, 10, 9, 8, 8, 8, 8, 8, 8, 8, 8, 7, 6, 6, 6, 6, 5, 5, 5, 5, 5, 5, 5, 5, 5, 5, 4, 4, 4, 4, 4, 4, 4, 4, 4, 4, 4, 4, 4, 4, 4, 4, 4, 4, 4, 4, 4, 3, 3, 3, 3, 3, 3, 3, 3, 3, 3, 3, 3, 3, 3, 3, 3, 3, 2, 2, 2, 2, 2, 2, 2, 2, 2, 2, 2, 2, 2, 2, 2, 2, 2, 2, 2, 2, 2, 2, 2, 2, 2, 2, 2, 2, 2, 2, 2, 2, 2, 2, 2, 1, 1, 1, 1, 1, 1, 1, 1, 1, 1, 1, 1, 1, 1, 1, 1, 1, 1, 1, 1, 1, 1, 1, 1, 1, 1, 1, 1, 1, 1, 1, 1, 1, 1, 1, 1, 1, 1, 1, 1, 1, 1, 1, 1

Corresponding number of selected Gene ontology terms each gene is involved with:
(In the same order as above)
13, 8, 8, 7, 7, 8, 6, 7, 9, 6, 6, 5, 4, 9, 6, 5, 3, 3, 13, 6, 3, 12, 6, 2, 2, 2, 3, 2, 4, 2, 8, 2, 2, 2, 2, 7, 6, 2, 2, 2, 1, 1, 1, 1, 1, 1, 1, 1, 5, 1, 1, 2, 2, 1, 1, 1, 1, 2, 1, 1, 1, 4, 1, 2, 1, 1, 1, 1, 1, 1, 2, 1, 1, 1, 1, 3, 1, 1, 1, 1, 1, 3, 3, 3, 1, 1, 3, 1, 1, 1, 1, 1, 1, 1, 1, 2, 1, 1, 2, 2, 1, 1, 1, 1, 1, 2, 1, 2, 1, 1, 1, 2, 2, 2, 2, 2, 2, 2, 1, 2, 1, 2, 1, 1, 1, 1, 1, 1, 1, 1, 1, 1, 1, 1, 1, 1, 1, 1, 1, 1, 1, 1, 1, 1, 1, 1, 1, 1, 1, 1, 1, 1, 1, 1, 1, 1, 1, 1, 1, 1, 1, 1, 1, 1, 1, 1, 1, 1,

1, 1, 1, 1, 1

Corresponding number of selected TFs each gene is involved with:
(In the same order as above)
4, 5, 5, 5, 5, 4, 5, 4, 3, 4, 4, 4, 5, 2, 3, 3, 5, 5, 1, 2, 4, 1, 2, 5, 5, 5, 3, 4, 2, 4, 1, 4, 4, 4, 4, 1, 1, 3, 3, 3, 5, 5, 5, 5, 5, 5, 5, 5, 1, 5, 4, 2, 2, 4, 4, 4, 4, 2, 4, 4, 4, 1, 4, 2, 4, 4, 4, 4, 4, 4, 2, 4, 3, 3, 3, 1, 3, 3, 3, 3, 3, 1, 1, 1, 3, 3, 1, 3, 3, 2, 2, 2, 2, 2, 1, 2, 2, 1, 1, 2, 2, 2, 2, 2, 1, 2, 1, 2, 2, 2, 1, 1, 1, 1, 1, 1, 1, 2, 1, 2, 1, 2, 2, 2, 1, 1, 1, 1, 1, 1, 1, 1, 1, 1, 1, 1, 1, 1, 1, 1, 1, 1, 1, 1, 1, 1, 1, 1, 1, 1, 1, 1, 1, 1, 1, 1, 1, 1, 1, 1, 1, 1, 1, 1, 1, 1, 1, 1, 1, 1, 1, 1, 1

Gene ontology terms ranked according to mean of effect sizes in cluster:
synaptic transmission | synaptogenesis | N-methyl-D-aspartate selective glutamate receptor activity | postsynaptic membrane | axon | isochorismate pyruvate lyase activity | postsynaptic density | dendritic spine | dendrite | synapse | synaptic transmission, glutamatergic | exocytosis | receptor clustering | receptor activity

Corresponding mean of effect sizes of each Gene ontology term:
(In the same order as above)
2.52, 1.93, 1.51, 1.42, 1.41, 1.40, 1.37, 1.25, 1.23, 1.21, 1.14, 0.74, 0.71, 0.60

Corresponding total number of genes of each Gene ontology term:
(In the same order as above)
251, 200, 208, 62, 382, 69, 162, 91, 82, 318, 38, 381, 79, 187

Corresponding number of genes of each Gene ontology term with at least one TFBS (of selected TFs):
(In the same order as above)
39, 35, 33, 13, 52, 13, 27, 18, 14, 41, 6, 47, 14, 28

Corresponding total number of TFBS (of selected TFs) for each Gene ontology term (in all genes):
(In the same order as above)
113, 82, 97, 35, 134, 28, 67, 54, 37, 105, 18, 123, 33, 64

Corresponding number of selected TFs each Gene ontology term is involved with:
(In the same order as above)
5, 5, 5, 5, 5, 5, 5, 5, 5, 5, 5, 5, 5, 5

TFs ranked according to mean of effect sizes in cluster:
V$MZF1_01, V$VDR_Q3, V$SMAD_Q6_01, V$TFIII_Q6, V$MZF1_02

Corresponding mean of effect sizes of each TF:
(In the same order as above)
1.58, 1.46, 1.32, 1.13, 1.10

Corresponding total number of TFBS for each TF (genome-wide):
(In the same order as above)
729, 720, 699, 726, 747

Corresponding total number of TFBS for each TF (in all genes in selected Gene ontology terms):
(In the same order as above)
211, 192, 191, 196, 200

Corresponding number of genes (of selected Gene ontology terms) each TF is involved with:
(In the same order as above)
84, 80, 82, 82, 79

Corresponding number of selected Gene ontology terms each TF is involved with:
(In the same order as above)
14, 14, 14, 14, 14

Cluster for columns 293 to 307, rows 289 to 297

Gene ontology terms

anaerobic glycolysis | fermentation | glycerol-3-phosphate dehydrogenase activity | phosphoglycerate mutase activity | hexokinase activity | tricarboxylic acid cycle | glucose-6-phosphate dehydrogenase activity | pentose-phosphate shunt | lactate dehydrogenase activity | glucokinase activity | glucose catabolic process | glycolysis | gluconeogenesis | carbohydrate metabolic process | glucose metabolic process

TFs

V$SP1_01 | V$SP1_Q6 | V$SP1_Q4_01 | V$SP1_Q6_01 | V$KROX_Q6 | V$AHR_Q5 | V$AHRHIF_Q6 | V$HIF1_Q5 | V$HIF1_Q3

Information

All related TFs:
(List of all TFs that are related to any of the PWMs)
ARNT2, AhR, AhR2, AhR:Arnt, AhRR, AhRR:Arnt, Arnt, Egr-1, Egr-2, Egr-3, Egr-4, HIF-1, HIF-1alpha, HIF-1alpha-isoform1, Sp1, Sp2, Sp3, Sp4

Ranked gene list:
(All genes of the selected Gene ontology terms with hits of any of the selected TFs, ranked according to the total number of TFBS)
GPI, PER1, GAPDH, H6PD, TPI1, PFKL, FASN, UBL4A, TKT, PFKP, PCK2, ACHE, LDHA, GK, PPM2C, PFN2, IRS2, AKT1, VEGFA, PC, PFKFB2, MAP4K2, HBA2, KHK, HK1, GPD2, FOLR2, CYCS, LDHB, GFER, TNK2, PGP, IMMT, CPT1A, BNIP3, SLC2A1, SCO2, RAG1AP1, SLC16A3, HIF1AN, GLUD1, ADIPOR1, ACSS2, ACSL1, NOL3, GPT, DBP, VDAC1, UGDH, TXN, SLC2A3, PPARG, PFKFB3, PDK3, NOC2L, IGF1R, GNAI2, COL11A2, ALDOA, ACACA, RPL22, PKN1, NR4A3, NR4A1, LPHN1, LEPROT, LEPR, HOOK1, ENO1, DHX9, DGAT1, ADIPOR2, ZFYVE9, ZDHHC23, UCP2, TGFB1, SUFU, STK11, SOD2, SLC37A4, SLC16A1, SFI1, SCARB1, PTPRF, PODXL, PIK3CA, PGM2, PGAM1, PDPK1, ONECUT1, NR3C1, MTG1, MAPT, KCNJ11, HM13, HDAC4, HBA1, GNAS, BSG, A1BG, TIPARP, SEPHS1, RPE, PPP1R13B, PPAN, POMC, PDK1, PDF, NRF1, MUC1, MBP, LPIN1, LAP3, JPH3, INVS, HGS, GCLC, FXN, ENO2, DYNLRB1, DPP4, CSH2, CRTC2, COL25A1, CKB, CFD, CBFA2T2, CA11, ATF1, AQP3, AKT3, AKT2, ACY1

Corresponding total number of TFBS:
(For each gene listed above, the total number of TFBS for any of the selected TFs, multiplied by the number of selected Gene ontology terms containing that gene)
52, 48, 42, 40, 36, 35, 25, 21, 20, 18, 18, 18, 16, 16, 15, 15, 15, 15, 14, 14, 12, 12, 12, 10, 10, 10, 10, 10, 9, 9, 8, 8, 8, 8, 8, 7, 7, 7, 6, 6, 6, 6, 6, 6, 5, 5, 5, 4, 4, 4, 4, 4, 4, 4, 4, 4, 4, 4, 4, 4, 3, 3, 3, 3, 3, 3, 3, 3, 3, 3, 3, 3, 2, 2, 2, 2, 2, 2, 2, 2, 2, 2, 2, 2, 2, 2, 2, 2, 2, 2, 2, 2, 2, 2, 2, 2, 2, 2, 2, 1, 1, 1, 1, 1, 1, 1, 1, 1, 1, 1, 1, 1, 1, 1, 1, 1, 1, 1, 1, 1, 1, 1, 1, 1, 1, 1, 1, 1, 1, 1, 1, 1

Corresponding number of selected Gene ontology terms each gene is involved with:
(In the same order as above)
13, 6, 14, 10, 6, 7, 5, 7, 10, 6, 9, 2, 8, 8, 5, 3, 3, 3, 2, 7, 4, 3, 2, 2, 5, 10, 5, 5, 3, 1, 4, 1, 8, 4, 2, 7, 1, 1, 1, 3, 6, 2, 2, 2, 1, 1, 1, 2, 2, 4, 4, 4, 4, 2, 1, 1, 2, 1, 2, 2, 1, 1, 1, 1, 1, 1, 1, 3, 1, 1, 1, 3, 1, 1, 2, 1, 1, 2, 2, 2, 2, 1,

1, 1, 2, 1, 2, 2, 1, 2, 1, 2, 1, 1, 1, 1, 2, 1, 1, 1, 1, 1, 1, 1, 1, 1, 1, 1, 1, 1, 1, 1, 1, 1, 1, 1, 1, 1, 1, 1, 1, 1, 1, 1, 1, 1, 1, 1, 1, 1, 1, 1, 1

Corresponding number of selected TFs each gene is involved with:
(In the same order as above)
4, 8, 3, 4, 6, 5, 5, 3, 2, 3, 2, 9, 2, 2, 3, 5, 5, 5, 7, 2, 3, 4, 6, 5, 2, 1, 2, 2, 3, 9, 2, 8, 1, 2, 4, 1, 7, 7, 6, 2, 1, 3, 3, 3, 5, 5, 5, 2, 2, 1, 1, 1, 1, 2, 4, 4, 2, 4, 2, 2, 3, 3, 3, 3, 3, 3, 3, 1, 3, 3, 3, 1, 2, 2, 1, 2, 2, 1, 1, 1, 1, 2, 2, 2, 1, 2, 1, 1, 2, 1, 2, 1, 2, 2, 2, 2, 1, 2, 2, 2, 1, 1, 1, 1, 1, 1, 1, 1, 1, 1, 1, 1, 1, 1, 1, 1, 1, 1, 1, 1, 1, 1, 1, 1, 1, 1, 1, 1, 1, 1, 1, 1

Gene ontology terms ranked according to mean of effect sizes in cluster:
glycolysis | carbohydrate metabolic process | hexokinase activity | glucose metabolic process | phosphoglycerate mutase activity | tricarboxylic acid cycle | glucose-6-phosphate dehydrogenase activity | anaerobic glycolysis | glucose catabolic process | glucokinase activity | gluconeogenesis | pentose-phosphate shunt | lactate dehydrogenase activity | glycerol-3-phosphate dehydrogenase activity | fermentation

Corresponding mean of effect sizes of each Gene ontology term:
(In the same order as above)
1.70, 1.65, 1.63, 1.28, 1.10, 1.05, 1.03, 1.02, 0.96, 0.94, 0.80, 0.71, 0.58, 0.57, 0.38

Corresponding total number of genes of each Gene ontology term:
(In the same order as above)
200, 99, 40, 215, 77, 144, 67, 37, 39, 30, 144, 78, 33, 79, 137

Corresponding number of genes of each Gene ontology term with at least one TFBS (of selected TFs):
(In the same order as above)
51, 26, 14, 55, 18, 32, 14, 11, 10, 9, 36, 16, 9, 15, 24

Corresponding total number of TFBS (of selected TFs) for each Gene ontology term (in all genes):
(In the same order as above)
144, 70, 32, 136, 50, 72, 38, 26, 33, 31, 87, 41, 25, 38, 63

Corresponding number of selected TFs each Gene ontology term is involved with:
(In the same order as above)
9, 9, 9, 9, 9, 9, 9, 7, 9, 8, 9, 9, 9, 9, 9

TFs ranked according to mean of effect sizes in cluster:
V$HIF1_Q3, V$HIF1_Q5, V$SP1_Q4_01, V$AHR_Q5, V$SP1_Q6, V$SP1_Q6_01, V$AHRHIF_Q6, V$KROX_Q6, V$SP1_01

Corresponding mean of effect sizes of each TF:
(In the same order as above)
2.10, 1.51, 1.14, 1.09, 1.07, 1.05, 0.98, 0.34, -0.06

Corresponding total number of TFBS for each TF (genome-wide):
(In the same order as above)
644, 654, 656, 666, 664, 663, 671, 675, 696

Corresponding total number of TFBS for each TF (in all genes in selected Gene ontology terms):
(In the same order as above)
131, 117, 89, 111, 92, 93, 103, 79, 71

Corresponding number of genes (of selected Gene ontology terms) each TF is involved with:
(In the same order as above)

44, 35, 31, 42, 33, 32, 42, 30, 36

Corresponding number of selected Gene ontology terms each TF is involved with:
(In the same order as above)
15, 15, 15, 15, 15, 14, 15, 14, 14

Cluster for columns 454 to 531, rows 192 to 198

Gene ontology terms

crotonyl-CoA reductase activity | monocyte chemotaxis | localization of cell | chemotaxis | leukocyte migration | cell chemotaxis | positive chemotaxis | B cell activation | immunoglobulin production | B cell proliferation | immunoglobulin secretion | interferon-gamma production | T-helper 1 type immune response | response to host immune response | adaptive immune response | adaptive immune response based on somatic recombination of immune receptors built from immunoglobulin superfamily domains | chemokine production | interleukin-10 production | interleukin-12 production | cell maturation | MHC class I biosynthetic process | MHC class I protein binding | MHC class II biosynthetic process | MHC class II protein binding | antigen processing and presentation | immune response | type IV hypersensitivity | lymphocyte proliferation | tolerance induction | lymphocyte activation | interleukin-2 production | interleukin-2 receptor activity | T cell proliferation | interleukin-4 production | cytokine production | cytokine secretion | pathogenesis | interleukin-4 receptor activity | interleukin-5 production | evasion or tolerance of immune response of other organism during symbiotic interaction | tryptophan catabolic process | defense response to virus | innate immune response | tumor necrosis factor receptor activity | tumor necrosis factor receptor binding | positive regulation of NF-kappaB transcription factor activity | response to tumor necrosis factor | IkappaB kinase complex | NF-kappaB binding | interleukin-6 receptor activity | interleukin-1 beta production | interleukin-1 production | cytolysis | natural killer cell mediated cytotoxicity | natural killer cell receptor activity | naringenin-chalcone synthase activity | immature T cell proliferation in the thymus | eosinophil activation | leukocyte activation | monocyte activation | mucosal immune response | granulocyte macrophage colony-stimulating factor biosynthetic process | granulocyte macrophage colony-stimulating factor production | interleukin-1 receptor activity | interleukin-6 production | tumor necrosis factor production | macrophage activation | inflammatory response | response to lipopolysaccharide | negative regulation of inflammatory response | regulation of tumor necrosis factor production | negative regulation of tumor necrosis factor production | positive regulation of tumor necrosis factor production | interleukin-1 receptor antagonist activity | acute inflammatory response | granuloma formation | lipopolysaccharide binding | cytokine biosynthetic process

TFs

V$HNF4_Q6 | V$AR_Q6 | V$PR_Q2 | V$GR_Q6_01 | V$AML1_Q6 | V$AML1_01 | V$OSF2_Q6

Information

All related TFs:
(List of all TFs that are related to any of the PWMs)
AML1, AML1a, AML3, AML3-isoform2, AR, COUP, COUP-TF1, COUP-TF2, GR, GR-alpha, GR-beta, HNF-4, HNF-4alpha, HNF-4alpha1, HNF-4alpha2, HNF-4alpha3, HNF-4alpha4, HNF-4alpha7, HNF-4gamma, PR, PR-alpha, PR-beta, RUNX2-isoform2

Ranked gene list:
(All genes of the selected Gene ontology terms with hits of any of the selected TFs, ranked according to the total number of TFBS)
TNF, CD4, ITGB2, LTA, CCR7, TLR9, FASN, CSF2, LTB, TNFRSF4, IRF1, ISYNA1, VEGFA, AKT1, IL2RA, CXCR4, MYD88, STAT1, TNFRSF18, TNFRSF1A, LCK, SOCS1, PTPN6, PER1, STAT6,

CD27, IL17C, PSMB8, CISH, CD44, GPR132, TG, PSMB10, MAP3K14, EDC4, STAT5A, TNFRSF1B, PTMA, NFATC1, MAP2K3, SOCS3, BCL2, CDK5R1, TAP2, LAG3, IKBKE, SILV, PTGER4, DUSP1, BCL6, ACTB, STAT5B, CSF1, CD68, FOXP3, CDKN1B, PLCG1, JUNB, IL7R, IL6R, FLJ32987, CTRL, CSK, CD7, CD63, TNFRSF6B, SELPLG, PDCD1, OSM, IL16, EGR1, CCR10, CAPG, BCR, GATA3, CD5, APOM, ABCB9, TRAF1, TAP1, PTK2B, PRDM1, CTSD, ZAP70, TRAF6, SSSCA1, SIGIRR, S1PR4, PDE4A, ITGA5, ING1, FYN, TCIRG1, STK16, RPS6KA1, RARA, POU2F2, PNPLA2, PHB2, NFKBIE, IL23A, EHD1, DUSP2, CIC, CFL1, VIPR1, UBC, TAPBP, SMAD7, RXRB, RUNX1, RTEL1, RASSF5, PXN, NLRP3, LIF, HIVEP2, GABARAP, COL11A2, CCND2, CALR, BAK1, AGER, ADM, TNFRSF25, FOS, SLA, PIK3R1, NEDD9, DCTN3, CXCR5, CORO7, TUBGCP2, TNIP1, TNFRSF13C, SHC1, PKN1, UNC13D, TRAF5, SQSTM1, PTGER2, MAL, MADD, INPP5D, FGR, DECR1, CD300A, ZMYND10, ZFP36, ZC3H12A, TUSC2, SOS1, SLC25A22, S100A6, S100A4, RASSF1, PTPN7, PRDX2, MUC1, MAZ, LSP1, LRDD, ITGB7, GAPDH, ETS1, DUSP28, CDK5, ARVCF, TNFAIP3, SKP1, RNASEK, RIPK3, PSME1, PRAP1, NR4A1, MARK2, MAPKAPK2, LTB4R, LEF1, LCP2, ICAM3, HLA-DPB1, HLA-DMB, GUK1, GFI1, GALT, GALNT6, FHL3, FGF8, EOMES, DHX58, CFLAR, CDK4, CD74, CD226, CAMK2G, AMH, TSPAN4, TRADD, TPP1, TNFRSF14, TIRAP, STRA13, SFTPC, SEMA4D, RPLP2, PTGDS, PDGFB, LMNA, HSP90B1, CD79B, CAPN10, ATXN1, ZFPM1, TNFRSF12A, SKAP1, RUNX3, PRF1, PLEKHF1, PAX5, P2RY11, NOS3, MST1, MMP11, LY6E, LTB4R2, IL10RA, HRAS, GRK6, GNAS, CD47, CD247, ACSL6, VIM, VDAC1, USF1, UROD, TRAIP, TNFSF14, TIPARP, PSME2, PSENEN, PML, PIN1, NGFR, MRPL28, MC1R, MAP3K11, MAG, KLF6, KCNN4, IRS1, ILK, HSPA1B, FUT7, FOXO3, EFNA1, DLX2, DEAF1, DCXR, CRTC2, CD3D, CCND3, CAMKV, BCL9, ATN1, ANGPT2, ACD, VAC14, TRERF1, SSFA2, SPON2, SPHK2, SEMA3B, PSMP, POLD4, PLXNC1, PCSK7, NR4A2, NOTCH1, NOL3, MTX1, MT2A, MLL, MBNL2, LTC4S, ILF3, HIC1, GORASP2, FOXP1, FLNA, FLII, ETV6, DPCR1, DGCR2, DDR1, CDKN2C, CD79A, CD6, CCL27, CACNB3, BHLHB2, BCL2L11, AGPAT1, UCP2, TXNIP, TCEB2, TAC3, SUFU, ROBLD3, PTCH1, PNKD, NOTCH4, NHP2L1, MAPK12, MAPK11, LMAN2, LEPREL2, HLA-DPA1, HIVEP3, GNB2L1, EXOSC1, EFNA2, ECE1, DIABLO, DHCR7, DAPP1, CNTFR, CEACAM1, CDKN1C, CDC37, BATF, APBB1, AIRE

Corresponding total number of TFBS:
(For each gene listed above, the total number of TFBS for any of the selected TFs, multiplied by the number of selected Gene ontology terms containing that gene)
532, 476, 350, 287, 196, 182, 180, 174, 133, 126, 119, 116, 112, 108, 106, 102, 99, 93, 84, 81, 80, 77, 70, 70, 64, 63, 60, 56, 56, 56, 48, 45, 42, 42, 40, 39, 38, 36, 35, 35, 34, 33, 32, 30, 30, 30, 28, 28, 28, 28, 28, 26, 26, 26, 24, 24, 21, 21, 21, 21, 21, 21, 21, 21, 21, 20, 20, 20, 20, 20, 20, 20, 20, 20, 18, 18, 18, 18, 16, 16, 16, 16, 16, 15, 15, 15, 15, 15, 15, 15, 15, 15, 14, 14, 14, 14, 14, 14, 14, 14, 14, 14, 14, 14, 12, 12, 12, 12, 12, 12, 12, 12, 12, 12, 12, 12, 12, 12, 12, 12, 12, 12, 11, 11, 10, 10, 10, 10, 10, 10, 9, 9, 9, 9, 9, 8, 8, 8, 8, 8, 8, 8, 8, 8, 8, 7, 7, 7, 7, 7, 7, 7, 7, 7, 7, 7, 7, 7, 7, 7, 7, 7, 7, 7, 7, 7, 6, 6, 6, 6, 6, 6, 6, 6, 6, 6, 6, 6, 6, 6, 6, 6, 6, 6, 6, 6, 6, 6, 6, 6, 6, 6, 6, 6, 6, 5, 5, 5, 5, 5, 5, 5, 5, 5, 5, 5, 5, 5, 5, 5, 4, 4, 4, 4, 4, 4, 4, 4, 4, 4, 4, 4, 4, 4, 4, 4, 4, 4, 4, 4, 3, 3, 3, 3, 3, 3, 3, 3, 3, 3, 3, 3, 3, 3, 3, 3, 3, 3, 3, 3, 3, 3, 3, 3, 3, 3, 3, 3, 3, 3, 3, 3, 3, 3, 2, 2, 2, 2, 2, 2, 2, 2, 2, 2, 2, 2, 2, 2, 2, 2, 2, 2, 2, 2, 2, 2, 2, 2, 2, 2, 2, 2, 2, 2, 2, 2, 2, 2, 1, 1, 1, 1, 1, 1, 1, 1, 1, 1, 1, 1, 1, 1, 1, 1, 1, 1, 1, 1, 1, 1, 1, 1, 1, 1, 1, 1

Corresponding number of selected Gene ontology terms each gene is involved with:
(In the same order as above)
76, 68, 50, 41, 28, 26, 30, 58, 19, 21, 17, 29, 16, 18, 53, 17, 33, 31, 14, 27, 20, 11, 10, 10, 16, 21, 15, 8, 8, 28, 12, 9, 6, 6, 20, 13, 19, 6, 7, 5, 17, 11, 16, 6, 6, 5, 14, 7, 7, 4, 4, 13, 26, 13, 24, 4, 3, 3, 7, 7, 3, 3, 3, 7, 7, 5, 5, 5, 4, 10, 10, 4, 10, 10, 3, 18, 3, 3, 4, 8, 4, 8, 4, 5, 15, 3, 3, 3, 5, 5, 5, 5, 2, 2, 2, 2, 2, 2, 7, 2, 14, 2, 2, 2, 2, 6, 12, 4, 2, 2, 3, 2, 2, 3, 4, 12, 4, 2, 4, 3, 6, 2, 3, 2, 11, 11, 2, 5, 5, 2, 10, 2, 3, 3, 9, 3, 3, 2, 4, 8, 8, 2, 2, 2, 4, 8, 4, 1, 1, 1, 1, 7, 1, 1, 1, 1, 1, 1, 1, 7, 1, 1, 1, 1, 1, 1, 1, 1, 1, 6, 1, 1, 3, 3, 6, 1, 3, 6, 3, 1, 6, 6, 6, 3, 1, 1, 2, 2, 1, 1, 1, 2, 6, 1, 6, 3, 1, 1, 1, 5, 1, 5, 5, 1, 5, 1, 1, 5, 1, 5, 5, 5, 1, 1, 1, 4, 1, 1, 2, 2, 4, 1, 4, 2, 1, 1, 1, 4, 1, 1, 1, 4, 4, 1, 1, 3, 1, 1, 3, 1, 3, 3, 1, 3, 1, 3, 1, 1, 1, 1, 1, 1, 3, 1, 3, 1, 1, 1, 3, 1, 3, 1, 1, 3, 1, 1, 1, 3, 1, 2, 1, 2, 1, 2, 1, 2, 1, 1, 1, 2, 2, 2, 1, 1, 1, 1, 1, 2, 1, 2, 2, 1, 1, 1, 1, 2, 1, 2, 2, 1, 2, 1, 1, 1, 1, 1, 1, 1, 1, 1, 1, 1, 1, 1, 1, 1, 1, 1, 1, 1, 1, 1, 1, 1, 1, 1, 1, 1, 1, 1, 1, 1

Corresponding number of selected TFs each gene is involved with:
(In the same order as above)

7, 7, 7, 7, 7, 7, 6, 3, 7, 6, 7, 4, 7, 6, 2, 6, 3, 3, 6, 3, 4, 7, 7, 7, 4, 3, 4, 7, 7, 2, 4, 5, 7, 7, 2, 3, 2, 6, 5, 7, 2, 3, 2, 5, 5, 6, 2, 4, 4, 7, 7, 2, 1, 2, 1, 6, 7, 7, 3, 3, 7, 7, 7, 3, 3, 4, 4, 4, 5, 2, 2, 5, 2, 2, 6, 1, 6, 6, 4, 2, 4, 2, 4, 3, 1, 5, 5, 5, 3, 3, 3, 3, 7, 7, 7, 7, 7, 7, 2, 7, 1, 7, 7, 7, 7, 2, 1, 3, 6, 6, 4, 6, 6, 4, 3, 1, 3, 6, 3, 4, 2, 6, 4, 6, 1, 1, 5, 2, 2, 5, 1, 5, 3, 3, 1, 3, 3, 4, 2, 1, 1, 4, 4, 4, 2, 1, 2, 7, 7, 7, 7, 1, 7, 7, 7, 7, 7, 7, 1, 7, 7, 7, 7, 7, 7, 7, 7, 7, 1, 6, 6, 2, 2, 1, 6, 2, 1, 2, 6, 1, 1, 1, 2, 6, 6, 3, 3, 6, 6, 6, 3, 1, 6, 1, 2, 6, 6, 5, 1, 5, 1, 1, 5, 1, 5, 5, 1, 5, 1, 1, 1, 5, 5, 4, 1, 4, 4, 2, 2, 1, 4, 1, 2, 4, 4, 4, 1, 4, 4, 4, 1, 1, 4, 3, 1, 3, 3, 1, 3, 1, 1, 3, 1, 3, 1, 3, 3, 3, 3, 3, 3, 1, 3, 1, 3, 3, 3, 1, 3, 1, 3, 3, 1, 3, 3, 3, 1, 3, 1, 2, 1, 2, 1, 2, 1, 2, 2, 2, 1, 1, 1, 2, 2, 2, 2, 2, 1, 2, 1, 1, 2, 2, 2, 2, 1, 2, 1, 1, 2, 1, 2, 2, 2, 2, 1, 1, 1, 1, 1, 1, 1, 1, 1, 1, 1, 1, 1, 1, 1, 1, 1, 1, 1, 1, 1, 1, 1, 1, 1, 1, 1, 1

Gene ontology terms ranked according to mean of effect sizes in cluster:
cell maturation | acute inflammatory response | adaptive immune response | immature T cell proliferation in the thymus | response to host immune response | cell chemotaxis | chemokine production | positive chemotaxis | T-helper 1 type immune response | cytokine production | cytokine secretion | interleukin-4 production | MHC class II biosynthetic process | MHC class II protein binding | tryptophan catabolic process | immune response | antigen processing and presentation | leukocyte migration | T cell proliferation | granulocyte macrophage colony-stimulating factor production | innate immune response | immunoglobulin production | interleukin-2 receptor activity | granulocyte macrophage colony-stimulating factor biosynthetic process | granuloma formation | adaptive immune response based on somatic recombination of immune receptors built from immunoglobulin superfamily domains | type IV hypersensitivity | lymphocyte activation | macrophage activation | interleukin-4 receptor activity | interleukin-12 production | response to lipopolysaccharide | localization of cell | positive regulation of tumor necrosis factor production | positive regulation of NF-kappaB transcription factor activity | interleukin-5 production | mucosal immune response | leukocyte activation | monocyte activation | natural killer cell receptor activity | regulation of tumor necrosis factor production | interleukin-6 production | naringenin-chalcone synthase activity | eosinophil activation | interleukin-1 receptor antagonist activity | cytokine biosynthetic process | interleukin-10 production | monocyte chemotaxis | crotonyl-CoA reductase activity | interleukin-1 receptor activity | B cell activation | response to tumor necrosis factor | cytolysis | evasion or tolerance of immune response of other organism during symbiotic interaction | inflammatory response | natural killer cell mediated cytotoxicity | interleukin-2 production | lymphocyte proliferation | B cell proliferation | negative regulation of inflammatory response | tolerance induction | interferon-gamma production | negative regulation of tumor necrosis factor production | MHC class I biosynthetic process | MHC class I protein binding | lipopolysaccharide binding | tumor necrosis factor production | pathogenesis | tumor necrosis factor receptor binding | tumor necrosis factor receptor activity | chemotaxis | defense response to virus | interleukin-1 production | NF-kappaB binding | immunoglobulin secretion | interleukin-1 beta production | interleukin-6 receptor activity | IkappaB kinase complex

Corresponding mean of effect sizes of each Gene ontology term:
(In the same order as above)
1.93, 1.92, 1.81, 1.75, 1.75, 1.74, 1.74, 1.73, 1.71, 1.64, 1.55, 1.54, 1.46, 1.46, 1.44, 1.44, 1.41, 1.40, 1.39, 1.31, 1.31, 1.30, 1.29, 1.28, 1.28, 1.26, 1.25, 1.24, 1.24, 1.18, 1.18, 1.18, 1.18, 1.17, 1.17, 1.10, 1.08, 1.06, 1.05, 1.03, 1.02, 1.01, 1.00, 0.99, 0.98, 0.97, 0.97, 0.97, 0.97, 0.96, 0.96, 0.93, 0.93, 0.91, 0.90, 0.85, 0.82, 0.82, 0.81, 0.81, 0.76, 0.75, 0.68, 0.67, 0.67, 0.67, 0.67, 0.67, 0.66, 0.60, 0.59, 0.49, 0.47, 0.38, 0.35, 0.33, 0.24, -0.07

Corresponding total number of genes of each Gene ontology term:
(In the same order as above)
224, 68, 148, 42, 37, 94, 155, 87, 59, 455, 243, 123, 181, 178, 41, 530, 315, 145, 188, 107, 246, 155, 189, 109, 77, 131, 131, 232, 178, 87, 150, 226, 38, 61, 550, 57, 52, 108, 117, 77, 86, 165, 50, 71, 70, 108, 166, 52, 52, 128, 116, 185, 179, 141, 425, 33, 182, 158, 88, 40, 109, 106, 61, 195, 197, 61, 209, 1.1K, 214, 213, 371, 110, 31, 146, 51, 21, 104, 107

Corresponding number of genes of each Gene ontology term with at least one TFBS (of selected TFs):
(In the same order as above)
42, 8, 23, 9, 7, 15, 22, 14, 11, 72, 40, 22, 30, 29, 9, 77, 55, 20, 32, 18, 27, 22, 35, 18, 11, 17, 21, 43, 25, 18, 22, 40, 11, 10, 97, 9, 7, 11, 12, 7, 14, 26, 7, 10, 11, 18, 24, 5, 7, 20, 22, 31, 21, 21, 56, 4, 35, 24, 16, 5,

15, 14, 9, 30, 30, 7, 25, 152, 41, 40, 45, 16, 3, 21, 8, 2, 17, 18

Corresponding total number of TFBS (of selected TFs) for each Gene ontology term (in all genes):
(In the same order as above)
165, 45, 82, 42, 31, 69, 90, 58, 43, 269, 151, 86, 115, 114, 42, 274, 195, 84, 119, 66, 123, 79, 128, 66, 42, 59, 87, 164, 109, 67, 93, 148, 36, 45, 349, 44, 30, 51, 55, 40, 58, 101, 29, 35, 44, 69, 92, 29, 35, 70, 70, 121, 86, 78, 199, 22, 131, 86, 57, 27, 63, 49, 38, 119, 119, 26, 108, 503, 128, 125, 145, 67, 13, 86, 26, 10, 47, 58

Corresponding number of selected TFs each Gene ontology term is involved with:
(In the same order as above)
7, 7, 7, 7, 7, 7, 7, 7, 7, 7, 7, 7, 7, 7, 7, 7, 7, 7, 7, 7, 7, 7, 7, 7, 7, 7, 7, 7, 7, 7, 7, 7, 7, 7, 7, 7, 7, 7, 7, 7, 7, 7, 7, 7, 7, 7, 7, 7, 7, 7, 7, 7, 7, 7, 7, 7, 7, 7, 7, 7, 7, 7, 7, 7, 7, 7, 7, 7, 7, 7, 7, 7, 7, 7, 7, 7, 7, 7

TFs ranked according to mean of effect sizes in cluster:
V$AML1_Q6, V$AML1_01, V$HNF4_Q6, V$GR_Q6_01, VPR_Q2, VAR_Q6, V$OSF2_Q6

Corresponding mean of effect sizes of each TF:
(In the same order as above)
1.49, 1.38, 1.23, 0.97, 0.85, 0.85, 0.78

Corresponding total number of TFBS for each TF (genome-wide):
(In the same order as above)
718, 710, 710, 720, 698, 699, 710

Corresponding total number of TFBS for each TF (in all genes in selected Gene ontology terms):
(In the same order as above)
1134, 1119, 992, 1021, 984, 966, 908

Corresponding number of genes (of selected Gene ontology terms) each TF is involved with:
(In the same order as above)
161, 168, 152, 177, 175, 159, 146

Corresponding number of selected Gene ontology terms each TF is involved with:
(In the same order as above)
78, 78, 78, 78, 78, 78, 78

Cluster for columns 61 to 94, rows 423 to 433

Gene ontology terms

transcription factor binding | sequence-specific DNA binding | histone acetylation | regulation of transcription | chromatin remodeling complex | gene silencing | histone deacetylase activity | histone deacetylation | DNA methylation | methylation | centromeric heterochromatin | histone methylation | histone modification | chromatin modification | euchromatin | chromatin silencing | heterochromatin formation | nucleolus | nucleoplasm | nucleus | nuclear import | cytoplasm | nuclear export | autosome | sex chromosome | nuclear periphery | nucleus organization | protein binding | nucleosome positioning | dosage compensation, by hyperactivation of X chromosome | dosage compensation, by hypoactivation of X chromosome | histone acetyltransferase activity | protein-DNA complex | 5'-nucleotidase activity

TFs

V$E2F_Q6_01 | V$E2F_Q4_01 | V$E2F_Q3_01 | V$E2F1_Q4_01 | V$E2F1_Q6_01 | V$E2F_03 | V$E2F4DP2_01 | V$E2F1DP2_01 | V$E2F1DP1_01 | V$E2F_Q6 | V$E2F_Q4

Information

All related TFs:
(List of all TFs that are related to any of the PWMs)
DP-1, E2F, E2F+E4, E2F-1, E2F-1:DP-1, E2F-1:DP-2, E2F-3a, E2F-4, E2F-4:DP-2, E2F-7

Ranked gene list:
(All genes of the selected Gene ontology terms with hits of any of the selected TFs, ranked according to the total number of TFBS)
HIST1H4I, HIST4H4, CDKN1A, JUN, H2AFZ, CTCF, TP73, CCND1, RASSF1, PCNA, CDKN1B, CBX5, EZH2, SIN3A, MYB, MNT, E2F1, TP53, EGR1, TFAP2A, RBBP4, RBL1, MDM2, LEF1, LBX1, H2AFX, PAX5, NOL3, ID2, ANP32A, AKT1, NFATC1, UHRF1, SMARCA4, RANBP1, LMNB1, BAZ2A, APRT, STMN1, SETD7, PELP1, NCL, RRM2, GEMIN4, ERBB2IP, TCF3, SLC20A1, NR3C1, NAB2, MARCKSL1, CHAF1A, BMI1, ZMYND10, SLC39A1, GATA3, FOXO3, UBTF, SFRS2, NUTF2, LMOD1, JUNB, IRF1, CCND2, PLEC1, PEG3, HMGA1, CDC25B, SMAD7, SKP2, NOLC1, MYCN, IKZF1, EHMT2, CDC6, SUMO1, STAT3, RB1, HOXA10, HIC1, EIF5A, CENPB, CAMK2G, ARNTL, SP1, DAXX, SLBP, SFN, SERBP1, REL, NUP153, MORF4L1, JUND, FANCC, CALM2, RPL23A, PTMA, NR4A1, NASP, MCM7, MCM2, MAZ, IRF4, GNAS, E2F3, CBX4, CBX3, JMJD3, DHX9, CDKN2D, SRCAP, SOCS3, SMARCA2, SIAH1, IRS1, HNRNPA1, HMGB2, HIST1H3E, FOSB, CDK5, ATF3, VDR, STAT1, NUMA1, NNAT, MYC, HOXA9, GATA2, DNMT3A, CTDSP1, CBX8, BCL11B, SMC3, RPA2, HOXA7, HNRNPF, HMGN2, HDLBP, HDAC7, CDK6, YWHAQ, UBB, RING1, POLR2A, ISCU, DHFR, DCK, CCNB1IP1, RUNX2, POU3F2, MSL1, ILF3, HSPA2, HNRNPD, DTL, CUX1, ACTB, YY1, TRRAP, TADA2L, SNF1LK, SLC38A2, SGCE, SAMD4A, RNF4, RBPJ, RANBP3, PRKDC, PGD, PER2, PEG10, NFE2L2, NFATC3, NEUROD1, MTHFR, MSL2, MSH2, MLL, MCM3, MAT2A, MAF1, KEAP1, HELLS, HCFC1, GAPDH, CDKN1C, CD47, CALM1, BNIP3, BACH2, SLC5A6, PAX6, NR3C2, MCM5, HNRNPA2B1, HIST1H2BO, HES1, GFI1, GAMT, FLJ32987, EBF3, E2F2, CRTC2, CDT1, C2orf28, WNT1, WNK1, TRIM26, TRERF1, TMPO, TEAD1, SF3A3, SART3, RRAS, NPM1, MDK, KISS1R, HCN3, ETV4, EMD, EIF4A3, EGR2, CDC25A, CAST, ATAD2, VIM, TYMP, SSSCA1, PPIA, NOL5A, MKNK2, LEMD3, KLF1, KCNH4, IPO7, HIF1A, GTF2A1, CIRBP, CASP2, CACNA1G, ZFYVE9, YEATS4, SLC22A4, SLC17A7, SEMA3B, SCARF2, RPL35, RAVER1, RAB8A, PTPN6, PTBP1, PRMT1, PIK3CA, PIH1D1, PHF17, PER1, PBRM1, NFYA, NFKB2, NCOR1, MXI1, MLL2, MBD1, LMNB2, LIN9, GRM8, FLI1, EFNA5, DOCK10, DMRT1, CYCS, CTBP1, ANXA5, ANXA4, ADK, ZFP36L2, ZFP36L1, XRCC5, UBE2I, RRP1, RARA, PCGF1, IPO11, GAD1, FUS, FLII, DUX4, CTNNB1, CSNK2A1, COL1A1, CNOT1, CDKN2C, CD68, TPT1, TGFB1I1, SUV39H2, STAU1, SOCS1, SNF1LK2, SLC25A10, SIRT6, SAP130, RPS10, PURA, PSMC5, PRKCZ, POU4F1, PLAGL1, PGP, PCGF2, NUP43, NR4A2, NFIC, NFATC4, MTR, MSH3, MGC111011, MCM4, KPNB1, KLF9, IGF1R, HOXD11, HMBS, HLTF, GNL3, FAR1, EARS2, DYRK1B, DUSP4, CD3EAP, CASP7, BACH1, ACTL6A, ABCB1, ZFP90, VTA1, TRIM37, SNRNP70, SALL3, RFC1, PTCH1, PRKCE, PCIF1, MATR3, MARCH7, MAD2L1BP, LIG1, KANK1, IGF2, ICMT, HSD17B8, HIRA, GMNN, FASN, EFNB2, CDC2L5, CDC25C, ATN1, ARID2, ZBTB16, UNG, UBE2E3, U2AF2, TRIP6, SUPT3H, SNRPD1, SLC7A1, SKI, RAC1, PSMD4, POLE4, POLA1, PI4KA, PCNT, NSUN2, NADK, MAPT, MAP3K11, LLGL2, LGALS1, LEPREL2, JMJD1A, ING1, HSPD1, HRAS, HOXA5, HCRT, H3F3B, GLI1, FBXL10, ENTPD2, DLX5, DICER1, DGCR8, CHMP1A, CFL1, CETN3, CDK5R1, CDC7, BTG1, ARHGEF2, AKAP8, YBX2, WDR68, WBP7, UTX, UBE2E1, TLE4, TIAL1, SYNCRIP, SMCHD1, SMAD6, SLC2A4RG, SFRS7, RANBP9, PPP1R8, PCMT1, OPRL1, NR1H2, NOTCH1, NME2, NME1-NME2, NMB, NF2, NDC80, MLH1, MEF2C, MCM10, KLF5, INTS6, HOXB5, HNRNPUL1, HIST1H1A, HHEX, HAT1, GTF2F1, GSK3B, GRIN2C, GLTSCR2, GARS, FOXL2, FBRS, ENO2, EIF5, EIF4ENIF1, EEF1A1, DUSP1, DPYD, DMC1, DIS3, DEDD2, DAG1, CTDP1, CPSF3, CLPX, CLK1, CLIC4, CHEK1, CDK8, CDC37, CDC2L6, CD248, CADM1, CABIN1, BCL2L1, ATXN7, ALDOA, AKT2, AHNAK, AGPAT1, ADORA2A, ZFYVE20, ZEB1, VGF, ULK1, THOC2, TFDP1, TCOF1, TCF21, TACR1, SYNE2, STX16, SRP54, SMC1A, SENP1, RBX1, RAD51, PPOX, PMP22, PKN2, PKMYT1, PHF20, PDPK1, OBFC2B, NR2C2, NCKIPSD, MCRS1, MAPK12, KPNA3, ITPR1, ISYNA1, IRF8, HSPH1, HSPB11, HOXC8, GCS1, GATAD2B, FKBP5, FAF1, EID1, DUSP6, DDX20, DDB2, DDAH2, CXCR4, CTSD, CTBP2, COPS3, CNTFR, CDC5L, CD46, CASP6, CAPNS1, BRSK2, ATP6V0D1, ANP32B, ANO9, ANG, AGRP, ACTR6, ACHE, ZFP161, WAPAL, TSNAX, TPMT, TARBP2, SYNJ2, SP5, SLC1A5, SIRT7, SFRS18,

RPE, RNF123, RCHY1, PSMB8, PRKG1, PPP5C, PPP2CA, PPIG, POU6F1, PMS1, PKD2, PHF1, PHB, PES1, NTRK3, NDRG1, MKS1, MIB1, MEA1, MAFA, LRRC6, LDHA, KRT8, JAG2, IRX3, HSP90B1, HN1L, GORASP2, FSCN1, FOXA1, ETV5, ELAVL2, EFNA2, DLST, DDX39, CUL1, CTTN, COX17, CENPK, CALCB, C19orf6, BTD, BTAF1, BOP1, BAT4, ATP6V0C, ALOX5, ABT1

Corresponding total number of TFBS:
(For each gene listed above, the total number of TFBS for any of the selected TFs, multiplied by the number of selected Gene ontology terms containing that gene)
297, 216, 160, 136, 132, 112, 90, 90, 88, 88, 88, 85, 80, 77, 77, 77, 77, 76, 63, 60, 56, 55, 54, 54, 54, 54, 48, 48, 48, 48, 48, 45, 44, 44, 44, 44, 44, 44, 42, 42, 42, 42, 40, 40, 40, 36, 36, 36, 36, 36, 35, 35, 33, 33, 33, 33, 32, 32, 32, 32, 32, 32, 32, 30, 30, 30, 30, 28, 28, 28, 28, 28, 28, 28, 27, 27, 27, 27, 27, 27, 27, 27, 27, 26, 25, 24, 24, 24, 24, 24, 24, 24, 24, 24, 22, 22, 22, 22, 22, 22, 22, 22, 22, 22, 22, 22, 21, 21, 21, 20, 20, 20, 20, 20, 20, 20, 20, 20, 20, 20, 18, 18, 18, 18, 18, 18, 18, 18, 18, 18, 18, 16, 16, 16, 16, 16, 16, 16, 16, 15, 15, 15, 15, 15, 15, 15, 15, 14, 14, 14, 14, 14, 14, 14, 14, 14, 12, 12, 12, 12, 12, 12, 12, 12, 12, 12, 12, 12, 12, 12, 12, 12, 12, 12, 12, 12, 12, 12, 12, 12, 12, 12, 12, 12, 12, 12, 12, 12, 12, 12, 11, 11, 11, 11, 11, 11, 11, 11, 11, 11, 11, 11, 11, 11, 11, 10, 10, 10, 10, 10, 10, 10, 10, 10, 10, 10, 10, 10, 10, 10, 10, 10, 10, 10, 10, 9, 9, 9, 9, 9, 9, 9, 9, 9, 9, 9, 9, 9, 9, 9, 8, 8, 8, 8, 8, 8, 8, 8, 8, 8, 8, 8, 8, 8, 8, 8, 8, 8, 8, 8, 8, 8, 8, 8, 8, 8, 8, 8, 8, 8, 8, 8, 8, 7, 7, 7, 7, 7, 7, 7, 7, 7, 7, 7, 7, 7, 7, 7, 7, 7, 6, 6, 6, 6, 6, 6, 6, 6, 6, 6, 6, 6, 6, 6, 6, 6, 6, 6, 6, 6, 6, 6, 6, 6, 6, 6, 6, 6, 6, 6, 6, 6, 6, 6, 6, 6, 6, 6, 5, 5, 5, 5, 5, 5, 5, 5, 5, 5, 5, 5, 5, 5, 5, 5, 5, 5, 5, 5, 5, 5, 5, 5, 5, 4, 4, 4, 4, 4, 4, 4, 4, 4, 4, 4, 4, 4, 4, 4, 4, 4, 4, 4, 4, 4, 4, 4, 4, 4, 4, 4, 4, 4, 4, 4, 4, 4, 4, 4, 4, 4, 4, 4, 4, 4, 3, 3, 3, 3, 3, 3, 3, 3, 3, 3, 3, 3, 3, 3, 3, 3, 3, 3, 3, 3, 3, 3, 3, 3, 3, 3, 3, 3, 3, 3, 3, 3, 3, 3, 3, 3, 3, 3, 3, 3, 3, 3, 3, 3, 3, 3, 3, 3, 3, 3, 3, 3, 3, 3, 3, 3, 3, 3, 3, 3, 3, 3, 3, 3, 2, 2, 2, 2, 2, 2, 2, 2, 2, 2, 2, 2, 2, 2, 2, 2, 2, 2, 2, 2, 2, 2, 2, 2, 2, 2, 2, 2, 2, 2, 2, 2, 2, 2, 2, 2, 2, 2, 2, 2, 2, 2, 2, 2, 2, 2, 2, 2, 2, 2, 2, 2, 2, 2, 2, 2, 2, 2, 1, 1, 1, 1, 1, 1, 1, 1, 1, 1, 1, 1, 1, 1, 1, 1, 1, 1, 1, 1, 1, 1, 1, 1, 1, 1, 1, 1, 1, 1, 1, 1, 1, 1, 1, 1, 1, 1, 1, 1, 1, 1, 1, 1, 1, 1, 1, 1, 1, 1, 1, 1, 1, 1, 1, 1

Corresponding number of selected Gene ontology terms each gene is involved with:
(In the same order as above)
27, 27, 16, 17, 12, 14, 9, 10, 8, 8, 8, 17, 10, 7, 11, 7, 11, 19, 9, 6, 8, 5, 9, 6, 9, 9, 6, 12, 6, 6, 6, 5, 4, 11, 4, 4, 4, 4, 6, 6, 6, 6, 4, 4, 4, 6, 4, 12, 4, 6, 7, 5, 3, 3, 3, 3, 4, 8, 4, 8, 4, 4, 4, 5, 3, 3, 3, 4, 4, 7, 4, 7, 14, 4, 9, 9, 9, 3, 3, 3, 3, 3, 3, 13, 5, 3, 4, 4, 8, 6, 4, 4, 3, 4, 2, 2, 11, 2, 2, 2, 2, 2, 2, 2, 2, 2, 3, 7, 7, 4, 2, 10, 2, 2, 4, 4, 4, 4, 2, 2, 6, 9, 6, 3, 18, 2, 2, 9, 2, 2, 2, 2, 2, 2, 2, 4, 4, 2, 3, 15, 3, 3, 3, 5, 3, 5, 2, 2, 2, 2, 2, 2, 2, 2, 7, 12, 4, 3, 3, 4, 2, 2, 2, 4, 3, 4, 2, 2, 2, 4, 4, 3, 4, 3, 3, 4, 3, 3, 2, 4, 2, 2, 2, 4, 3, 4, 4, 3, 1, 1, 1, 1, 1, 1, 1, 1, 1, 1, 1, 1, 1, 1, 1, 10, 2, 2, 2, 5, 2, 2, 2, 5, 2, 1, 1, 2, 5, 2, 2, 2, 2, 2, 3, 1, 9, 3, 3, 1, 3, 3, 3, 3, 3, 1, 1, 3, 3, 4, 2, 1, 1, 2, 2, 1, 2, 4, 2, 1, 8, 4, 1, 1, 2, 4, 2, 2, 8, 2, 2, 8, 2, 4, 1, 1, 2, 1, 2, 4, 4, 2, 2, 2, 1, 1, 7, 7, 1, 7, 1, 1, 1, 1, 1, 1, 7, 7, 1, 1, 1, 1, 2, 3, 2, 2, 3, 1, 1, 2, 1, 1, 1, 2, 2, 2, 3, 1, 1, 2, 1, 3, 3, 2, 2, 1, 2, 1, 2, 1, 1, 1, 3, 3, 3, 1, 2, 1, 2, 2, 1, 3, 6, 1, 1, 1, 1, 1, 1, 1, 1, 1, 1, 1, 1, 1, 5, 1, 1, 5, 1, 1, 1, 1, 5, 1, 1, 4, 1, 4, 2, 2, 2, 2, 1, 1, 2, 2, 1, 1, 2, 1, 2, 1, 2, 2, 1, 4, 2, 1, 4, 1, 4, 4, 1, 1, 4, 1, 1, 1, 4, 1, 1, 1, 1, 2, 1, 1, 1, 1, 1, 3, 3, 3, 1, 1, 3, 1, 1, 1, 1, 1, 3, 1, 1, 1, 3, 3, 1, 1, 1, 1, 1, 3, 3, 1, 1, 3, 1, 1, 1, 1, 3, 1, 3, 1, 1, 1, 1, 1, 3, 3, 1, 3, 1, 1, 1, 1, 1, 1, 1, 3, 1, 3, 1, 3, 1, 1, 1, 1, 3, 1, 3, 3, 1, 1, 1, 1, 1, 1, 1, 1, 1, 1, 1, 2, 1, 1, 1, 1, 2, 2, 2, 2, 1, 1, 1, 1, 1, 2, 1, 2, 1, 2, 2, 2, 2, 2, 1, 2, 1, 1, 1, 1, 2, 2, 2, 2, 2, 2, 1, 1, 1, 2, 1, 1, 1, 1, 2, 1, 1, 1, 1, 1, 1, 2, 2, 1, 1, 1, 1, 1, 1, 1, 1, 1, 1, 1, 1, 1, 1, 1, 1, 1, 1, 1, 1, 1, 1, 1, 1, 1, 1, 1, 1, 1, 1, 1, 1, 1, 1, 1, 1, 1, 1, 1, 1, 1, 1, 1, 1, 1, 1, 1, 1, 1, 1, 1, 1, 1, 1, 1, 1, 1, 1

Corresponding number of selected TFs each gene is involved with:
(In the same order as above)
11, 8, 10, 8, 11, 8, 10, 9, 11, 11, 11, 5, 8, 11, 7, 11, 7, 4, 7, 10, 7, 11, 6, 9, 6, 6, 8, 4, 8, 8, 8, 9, 11, 4, 11, 11, 11, 11, 7, 7, 7, 7, 10, 10, 10, 6, 9, 3, 9, 6, 5, 7, 11, 11, 11, 11, 8, 4, 8, 4, 8, 8, 8, 6, 10, 10, 10, 7, 7, 4, 7, 4, 2, 7, 3, 3, 3, 9, 9, 9, 9, 9, 9, 2, 5, 8, 6, 6, 3, 4, 6, 6, 8, 6, 11, 11, 2, 11, 11, 11, 11, 11, 11, 11, 11, 11, 7, 3, 3, 5, 10, 2, 10, 10, 5, 5, 5, 5, 10, 10, 3, 2, 3, 6, 1, 9, 9, 2, 9, 9, 9, 8, 8, 8, 8, 8, 4, 4, 8, 5, 1, 5, 5, 5, 3, 5, 3, 7, 7, 7, 7, 7, 7, 7, 7, 2, 1, 3, 4, 4, 3, 6, 6, 6, 3, 4, 3, 6, 6, 6, 3, 3, 4, 3, 4, 4, 3, 4, 4, 6, 3, 6, 6, 6, 3, 4, 3, 3, 4, 11, 11, 11, 11, 11, 11, 11, 11, 11, 11, 11, 11, 11, 11, 11, 10, 1, 5, 5, 5, 2, 5, 5, 5, 2, 5, 10, 10, 5, 2, 5, 5, 5, 5, 5, 3, 9, 1, 3, 3, 9, 3, 3, 3, 3, 3, 9, 9, 3, 3, 2, 4, 8, 8, 4, 4, 8, 4, 2, 4, 8, 1, 2, 8, 8, 4, 2, 4, 4, 1, 4, 4, 1, 4, 2, 8, 8, 4, 8, 4, 2, 2, 4, 4, 4, 7, 7, 1, 1, 7, 1, 7, 7, 7, 7, 7, 7, 1, 1, 7, 7, 7, 7, 3, 2, 3, 3, 2, 6, 6, 3, 6, 6, 6, 3, 3, 3, 2, 6, 6, 3, 6, 2, 2, 3, 3, 6, 3, 6, 3, 6, 6, 6, 2, 2, 2, 6, 3, 6, 3, 3, 6, 2, 1, 5, 5, 5, 5, 5, 5, 5, 5, 5, 5, 5, 5, 5, 5, 1, 5, 5, 1, 5, 5, 5, 5, 1, 5, 5, 1, 4, 1, 2, 2, 2, 2, 4, 4, 2, 2, 4, 4, 2, 4, 2, 4, 2, 2, 4, 1, 2, 4, 1, 4, 1, 1, 4, 4, 1, 4, 4, 4, 1,

4, 4, 4, 4, 2, 4, 4, 4, 4, 3, 1, 1, 1, 3, 3, 1, 3, 3, 3, 3, 3, 1, 3, 3, 3, 1, 1, 3, 3, 3, 3, 3, 1, 1, 3, 3, 1, 3, 3, 3, 3, 1, 3, 1, 3, 3, 3, 3, 3, 1, 1, 3, 1, 3, 3, 3, 3, 3, 3, 3, 1, 3, 1, 3, 1, 3, 3, 3, 3, 1, 3, 1, 1, 3, 3, 3, 3, 3, 2, 2, 2, 2, 2, 2, 2, 1, 2, 2, 2, 2, 1, 1, 1, 1, 2, 2, 2, 2, 2, 1, 2, 1, 2, 1, 1, 1, 1, 1, 2, 1, 2, 2, 2, 2, 1, 1, 1, 1, 1, 1, 2, 2, 2, 1, 2, 2, 2, 2, 1, 2, 2, 2, 2, 2, 2, 2, 1, 1, 1, 1, 1, 1, 1, 1, 1, 1, 1, 1, 1, 1, 1, 1, 1, 1, 1, 1, 1, 1, 1, 1, 1, 1, 1, 1, 1, 1, 1, 1, 1, 1, 1, 1, 1, 1, 1, 1, 1, 1, 1, 1, 1, 1, 1, 1, 1, 1, 1, 1, 1, 1, 1, 1, 1, 1, 1

Gene ontology terms ranked according to mean of effect sizes in cluster:
DNA methylation | nuclear periphery | chromatin silencing | heterochromatin formation | chromatin modification | euchromatin | sequence-specific DNA binding | nucleoplasm | methylation | transcription factor binding | histone deacetylase activity | histone acetylation | nucleus | protein binding | dosage compensation, by hypoactivation of X chromosome | dosage compensation, by hyperactivation of X chromosome | chromatin remodeling complex | centromeric heterochromatin | regulation of transcription | autosome | histone acetyltransferase activity | gene silencing | nucleus organization | nuclear import | histone deacetylation | histone methylation | 5'-nucleotidase activity | nuclear export | protein-DNA complex | nucleolus | histone modification | nucleosome positioning | sex chromosome | cytoplasm

Corresponding mean of effect sizes of each Gene ontology term:
(In the same order as above)
3.10, 2.81, 2.68, 2.44, 2.39, 2.31, 2.30, 2.24, 2.22, 2.19, 2.16, 2.02, 1.96, 1.83, 1.78, 1.78, 1.70, 1.68, 1.66, 1.63, 1.62, 1.51, 1.44, 1.41, 1.37, 1.34, 1.28, 1.24, 1.23, 1.22, 1.15, 0.96, 0.87, 0.53

Corresponding total number of genes of each Gene ontology term:
(In the same order as above)
437, 90, 55, 78, 82, 62, 118, 172, 715, 231, 108, 283, 1.2K, 228, 61, 61, 105, 51, 187, 47, 99, 355, 35, 296, 144, 101, 23, 448, 84, 222, 115, 38, 62, 1.2K

Corresponding number of genes of each Gene ontology term with at least one TFBS (of selected TFs):
(In the same order as above)
98, 27, 21, 23, 21, 17, 34, 48, 146, 55, 35, 74, 230, 47, 12, 12, 31, 13, 51, 9, 27, 87, 10, 67, 40, 25, 7, 104, 21, 45, 31, 9, 10, 217

Corresponding total number of TFBS (of selected TFs) for each Gene ontology term (in all genes):
(In the same order as above)
509, 135, 115, 119, 150, 101, 185, 211, 686, 305, 186, 355, 1036, 219, 71, 71, 155, 74, 245, 61, 126, 397, 48, 296, 199, 127, 32, 445, 99, 191, 161, 48, 51, 905

Corresponding number of selected TFs each Gene ontology term is involved with:
(In the same order as above)
11, 11, 11, 11, 11, 11, 11, 11, 11, 11, 11, 11, 11, 11, 11, 11, 11, 11, 11, 11, 11, 11, 11, 11, 11, 11, 11, 11, 11, 11, 11, 11, 11, 11

TFs ranked according to mean of effect sizes in cluster:
V$E2F_Q3_01, V$E2F1_Q4_01, V$E2F_03, V$E2F4DP2_01, V$E2F_Q6_01, V$E2F_Q4_01, V$E2F1DP2_01, V$E2F1DP1_01, V$E2F_Q4, V$E2F1_Q6_01, V$E2F_Q6

Corresponding mean of effect sizes of each TF:
(In the same order as above)
2.27, 2.27, 2.14, 1.88, 1.82, 1.78, 1.60, 1.55, 1.54, 1.29, 1.29

Corresponding total number of TFBS for each TF (genome-wide):
(In the same order as above)
690, 681, 688, 656, 686, 667, 658, 682, 702, 700, 699

Corresponding total number of TFBS for each TF (in all genes in selected Gene ontology terms):
(In the same order as above)

800, 794, 819, 690, 781, 722, 666, 711, 734, 701, 696

Corresponding number of genes (of selected Gene ontology terms) each TF is involved with:
(In the same order as above)
257, 248, 258, 213, 239, 230, 201, 222, 247, 230, 232

Corresponding number of selected Gene ontology terms each TF is involved with:
(In the same order as above)
34, 34, 34, 34, 34, 34, 34, 34, 34, 34, 34

Cluster for columns 1295 to 1311, rows 267 to 278

Gene ontology terms

Notch signaling pathway | gene expression | DBD domain binding | DNA binding | chordate embryonic development | embryonic development | limb development | central nervous system development | central nervous system neuron development | gastrulation | somitogenesis | anatomical structure morphogenesis | organ development | neurogenesis | regionalization | cell fate determination | segmentation

TFs

V$ETF_Q6 | V$E2F_Q2 | V$E2F1_Q6 | V$E2F1_Q3 | V$AP2GAMMA_01 | V$AP2ALPHA_01 | V$AP2_Q6_01 | V$AP2_Q6 | V$WT1_Q6 | V$EGR_Q6 | V$MAZ_Q6 | V$MAZR_01

Information

All related TFs:
(List of all TFs that are related to any of the PWMs)
AP-2, AP-2alpha, AP-2alphaA, AP-2alphaB, AP-2beta, AP-2gamma, DP-1, E2F, E2F+E4, E2F-1, E2F-3a, E2F-4, ETF, Egr-1, Egr-2, Egr-3, MAZ, MAZR, WT1, WT1-del2

Ranked gene list:
(All genes of the selected Gene ontology terms with hits of any of the selected TFs, ranked according to the total number of TFBS)
LBX1, NOTCH1, FGF8, PAX6, LFNG, HES5, HES1, DLL1, TBX2, WNT1, ID2, HOXA9, AKT1, LHX1, NKX2-2, MDK, DVL1, PTCH1, CCND1, TGFB1, TCF3, DLX2, RBPJ, NR4A2, NR2F1, TLE3, NFATC1, CDKN1C, CDK5, AGRN, HOXA7, HMX3, LEF1, JARID2, DGAT1, PDGFA, FLII, FGF9, UNCX, HMGA1, GATA2, DNMT3A, RARA, HES7, TCF7, SSSCA1, SMAD7, MAFG, GATA3, RARG, HOXA10, ZIC2, WNT11, NR2F6, LDB1, FOXC1, EFNA2, DLL4, ZFP36L2, YY1, WNT10B, TBX1, RXRB, NRARP, NFIC, MAPK12, JUNB, IRX5, IGF1R, HOXB7, CYP26B1, CREBBP, CCND2, ACTB, SP3, NR3C1, ZFPM1, VEGFB, SOX8, SKI, GPX4, GLI1, FOXO3, BMI1, VEGFA, EGR2, SMARCD3, SIN3A, RUNX3, NR3C2, MMP11, LMX1B, GFI1, EGR3, AXIN1, ACHE, SEMA3B, RXRA, MAFK, LHX3, HOXC8, FOXD3, EGR1, UBE2I, THRA, PLXNA1, MEF2D, HES3, GPC1, FLJ32987, EFNB2, ZBTB7B, ZBTB7A, WNT7B, USF2, UBTF, SOX1, SNAPC2, SAMD4A, PCNA, NFKBIE, MNT, MMP23B, MAZ, ISYNA1, ISG15, HOXC9, HOXB6, HIVEP1, HES6, ERF, EHMT2, CDC25C, CBX4, BRD2, ARID3A, STRA13, SOCS3, REXO1, NELF, KLF13, HRAS, FASN, DPH1, CFL1, WNT4, TFAP2A, IRX4, IRF1, EGFL7, DYRK1A, CTCF, CORO7, TRADD, TFEB, SMAD6, RREB1, RRBP1, PBX3, MAFA, JAG2, IGF2, FLI1, DVL2, CUX1, BCL2L11, ACVR2B, ZNF362, TP73, SOS1, SCRIB, POU3F2, MYB, MTA1, LYL1, ING2, ID3, HOXA11, HMGB1, FOXO1, DBP, CDK5R1, ABCA2, TM7SF2, SOX18, SKIL, RING1, REST, PURA, POU2F1, NAB2, MLL, LMO4, HOXB8, HHEX, CTBP1, CDKN1B, SHH, PATZ1, NGFR, MBD3, MAP2K7, FURIN, COL11A2, ADNP, WHSC1, WBP7, RUNX2, NOL3, NFKB2, JUND, FOSL2, E2F4, TNFSF13, PAX2, ZFP36, ZEB1, ZBTB16, WNT7A, TEAD3, SLC2A4RG, SLC25A22, SLC20A1, SCRT1, POU4F1, PLEC1,

PKNOX1, PGP, PARD6A, OGFR, NR4A1, NEO1, NDOR1, MXI1, MAPK11, LRDD, KREMEN2, KISS1R, JMJD3, IRS1, HOXC6, HOXB5, HNRPDL, HMX2, HIVEP2, HIC1, H2AFZ, GIT1, FZD8, FGFRL1, FASTK, EZH2, EOMES, EFNA3, EFNA1, CUL4A, CSK, CRIP2, CRIP1, CKB, CIRBP, CDKN2C, CDK4, CD151, BCOR, BBC3, BACH2, ACTG1, TYMP, TNFRSF18, TNFRSF12A, SQSTM1, SLC4A2, SIPA1, REPIN1, RCOR2, PTMA, PNPLA2, PIGQ, NPDC1, LLGL2, IER2, HCFC1R1, GAMT, CEND1, BCL11B, ANP32A, WNT2B, TUSC2, TUBB3, TNFRSF4, TLE2, PBX2, NR4A3, LEPREL2, KLF10, HYAL2, HAND2, DDIT3, CREB1, CISH, CELSR3, CDK9, C21orf33, BCL3, ARHGEF2, ACVR1, ZEB2, TUBB2C, TGIF1, SHC1, PTBP1, PDGFB, NFYB, KLF12, IRF2, FBRS, ENOPH1, CLDN6, CHRD, ATP6V0C, AMH, ZFP161, WNT10A, VGF, STAU1, SNF1LK, SMARCA2, SMAD2, SLC7A1, SIX5, SHMT2, SATB2, RPS2, RPL8, QKI, POLR2A, PER1, PAX5, ONECUT1, NFIX, NFIB, NFIA, MXD3, MSL1, MKX, MAP4K2, MAP2K3, ING1, HS6ST1, HOXD11, HOXB4, HNRNPL, HDAC7, HCN2, HBA2, GTF2I, GPS2, GBX2, FMNL1, FGF17, ESRRA, ENO3, EFNA4, E4F1, DKFZp779C0757, CTSD, CTBP2, CPT1A, CPLX1, CORO1A, CHD3, CDKN1A, CBFA2T3, CARM1, BHLHB2, BCL9L, ATF3, APRT, ADORA2A, TIAM1, SPHK1, SOCS2, SMAD3, RORC, PHLDB1, PFKFB3, PCYT2, OTX1, OAZ1, NOTCH4, NKD2, NCOA6, MXD4, MARCKSL1, LTC4S, IRS2, HOXD13, HOXA1, GRIN2C, EP300, EMX1, EIF5A, CTSW, CAMK2G, CACNA1G, BCL6, ATXN1, WNT9A, VIM, VDAC1, TRERF1, TRAF4, TMEM201, TLE1, TAS1R3, SSBP3, SOCS1, SMARCA4, SETD1A, RHOA, PTPRU, PRKCE, PRKCD, PRDM16, PPP1R13B, PPARGC1B, PIK3R2, PELP1, PCID2, OCA2, NKX6-2, NFATC3, MCM2, MC1R, MAP3K12, LRP1, KLF11, HOXC5, HOXC4, HDGF, FZD7, FUS, FOXP1, FOXF2, FOXF1, FHOD1, ELAVL3, EEF1A2, DLL3, DAG1, CUL1, CRABP2, CHD7, CENPB, CD68, CBX2, CACNB3, BUB3, BCL11A, ADIPOR1, USP7, TRIM28, TRAF3, TBPL1, SRC, SFMBT2, RPL19, ROM1, REL, RCOR1, RBL1, RASSF1, PSMA7, PDGFRA, PCP2, NR2C2, NPTX1, NEUROG2, MGAT1, MAPKAPK3, LASP1, IL11, HOXB9, HOXA5, HOXA2, HES4, GRN, GAPDH, EVX2, DYRK2, CTDSP2, CDK6, ADM, ZFYVE9, ZFP36L1, XAB2, TWSG1, SSPO, SOX12, SIAH1, SEMA3F, RPL28, RHOC, PTPN12, PTK6, PPP1R1B, POLRMT, PIK3CA, PHIP, OSM, NRF1, MUC1, MSL2, MRPS6, MEN1, LRP5, KREMEN1, KLF2, KAT2A, IRF4, INTS1, IL17C, HSP90AB1, HSP90AA1, HOXB2, GTF2A1, GPSM1, FOS, FAM152A, EGR4, E2F2, DPYSL2, DAXX, CDK2, CDH15, CDC20, CBFB, CASP9, BRF1, BHLHB3, BCL2L2, ARNTL, ARID1B, ARID1A, APLP2, ALDOA, AIRE, YWHAZ, VAV2, TSPAN32, TSC22D1, TP53I3, TGFB3, TCF12, TBL1XR1, STAT5A, SRCAP, SPIB, SFTPC, SDC3, RPLP0, ROBLD3, RNF4, PTP4A3, PRKCZ, PPARA, POU3F3, PLXND1, PIN1, PIAS4, PHOX2A, PFN2, PDLIM7, PCSK4, PCGF2, NR1D1, NF1, NCLN, MSH6, MORF4L1, MFNG, MCAM, MAPK7, MAP3K14, LPHN1, LHX9, LGALS1, KCNH8, IRF7, Ht006, HOXA6, HIST1H4I, GTF2IRD1, GRIN2D, GEMIN4, GADD45B, FOSB, DUSP1, CTF1, CLEC11A, CEBPB, CCNE1, CBX3, CALM1, BAX, ARFRP1, AKT3, ZHX2, YES1, XPO1, UQCRH, TSTA3, TRRAP, TNFRSF25, TNFAIP3, TFRC, TAGLN2, STMN1, SMARCC2, SLCO3A1, SLC39A1, SLC19A1, SLC16A8, SEPT7, SEPHS1, RTN2, RPS6KA2, RND2, RGS10, REV3L, PTH1R, PSENEN, PRKCA, PRKACA, PPP3CA, PMF1, PFKP, OSR1, ODC1, NFATC4, NDRG2, NCOA2, MYST3, MYADM, MVP, MSI2, MIB1, MED13, MAX, MAFF, LY6E, LTBP3, LNX2, LHB, L1CAM, KLK1, KLF5, IRF6, IKZF1, HNRNPH1, HDLBP, HCFC1, H3F3B, H3F3A, GRB7, GAB2, FOXN2, FOLR2, FAM168B, FAF1, EPB41, EGLN2, DLG4, DHX58, DCHS1, DBN1, CYBA, CRTC2, CRB3, CNOT1, CHIC2, CDKN2D, CDC42EP4, CDC25B, CAMTA1, CALR, CACNA1C, C2orf28, BRMS1, BMP1, BACH1, ARHGAP5, AGER, ADRA2B, ZYX, ZNF521, ZC3H12A, YWHAG, YBX2, VLDLR, UQCR, TUBA1B, TRIM3, TPT1, TPM1, TOX2, TNRC6A, THPO, THBS3, TGFBRAP1, TADA2L, SYNCRIP, SRXN1, SMURF2, SMTN, SMEK1, SLC38A2, SLC29A1, SLC1A4, SLC12A5, SILV, SHOX2, SERTAD3, SCARB1, SBNO2, SALL3, S100A6, S100A4, RGS2, REPS1, RBM15, PXMP2, PVRL1, PUM1, PSMB8, PRMT1, PPP1R15B, PPP1R12C, PPIA, PPFIA1, PPARD, PNMT, PILRB, PHF7, PHC1, PER2, PDPK1, PDF, PBXIP1, PBX4, PAPD1, ORC6L, NRGN, NPPC, NPAS3, NPAS1, NNAT, NKG7, NFE2L3, NDRG3, NAB1, MYL6, MTHFR, MST1R, MSL3, MPP6, MLL2, MGC40146, MGA, MFRP, MADCAM1, LRBA, LMNA, LEPROT, LEPR, LDHA, KCNB1, JAKMIP1, ITPR3, IMPDH2, ILF3, IL17D, IGF2BP3, IDI1, HYAL1, HSPB6, HPN, HOXC10, HNRNPU, HNRNPD, HMGN1, HMBS, HES2, HDAC5, HDAC4, H6PD, GSK3A, GPC2, GOT2, GNA13, GCLM, GABBR1, FTL, FSCN1, FRAT2, FOXP4, FHL3, FCGRT, FAM110A, ERBB2IP, ENO2, EMP3, EIF4G1, EIF4A1, EBF2, DYNLL1, DIO3, DICER1, DENND4A, DCTN3, CTNNA1, CRHR2, COL6A1, CNN1, CD82, CCNE2, CCL27, CAST, CAD, C21orf2, C1QTNF5, C17orf85, C17orf28, BMP2K, BANP, ATP1B1, ATP1A1, ASXL1, ARID5B, APOBEC3D, APBB3, ANKH, AKT2, ADA2B, ACSL6, ACACA, ABCA3

Corresponding total number of TFBS:
(For each gene listed above, the total number of TFBS for any of the selected TFs, multiplied by the number of selected Gene ontology terms containing that gene)
160, 156, 144, 132, 120, 110, 110, 110, 108, 99, 88, 88, 84, 81, 77, 72, 72, 66, 66, 64, 64, 64, 63, 63, 63, 60, 60, 60, 60, 60, 56, 56, 55, 55, 55, 54, 54, 50, 48, 48, 48, 48, 45, 45, 44, 44, 44, 44, 44, 42, 42, 40, 40, 40, 40, 40, 40, 40, 36, 36, 36, 36, 36, 36, 36, 36, 36, 36, 36, 36, 36, 36, 36, 36, 35, 35, 33, 33, 33, 33, 33, 33, 33, 33, 32, 32, 30, 30, 30, 30, 30, 30, 30, 30, 30, 30, 28, 28, 28, 28, 28, 28, 28, 27, 27, 27, 27, 27, 27, 27, 25, 24, 24, 24, 24, 24, 24, 24, 24, 24, 24, 24, 24, 24, 24, 24, 24, 24, 24, 24, 24, 24, 24, 24, 24, 24, 22, 22, 22, 22, 22, 22, 22, 22, 22, 21, 21, 21, 21, 21, 21, 21, 21, 20, 20, 20, 20, 20, 20, 20, 20, 20, 20, 20, 20, 20, 20, 18, 18, 18, 18, 18, 18, 18, 18, 18, 18, 18, 18, 18, 18, 18, 18, 16, 16, 16, 16, 16, 16, 16, 16, 16, 16, 16, 16, 16, 16, 15, 15, 15, 15, 15, 15, 15, 15, 14, 14, 14, 14, 14, 14, 14, 13, 13, 12, 12, 12, 12, 12, 12, 12, 12, 12, 12, 12, 12, 12, 12, 12, 12, 12, 12, 12, 12, 12, 12, 12, 12, 12, 12, 12, 12, 12, 12, 12, 12, 12, 12, 12, 12, 12, 12, 12, 12, 12, 12, 12, 12, 12, 12, 12, 12, 12, 12, 12, 12, 12, 11, 11, 11, 11, 11, 11, 11, 11, 11, 11, 11, 11, 11, 11, 11, 11, 11, 11, 11, 10, 10, 10, 10, 10, 10, 10, 10, 10, 10, 10, 10, 10, 10, 10, 10, 10, 10, 10, 10, 9, 9, 9, 9, 9, 9, 9, 9, 9, 9, 9, 9, 9, 9, 9, 8, 8, 8, 8, 8, 8, 8, 8, 8, 8, 8, 8, 8, 8, 8, 8, 8, 8, 8, 8, 8, 8, 8, 8, 8, 8, 8, 8, 8, 8, 8, 8, 8, 8, 8, 8, 8, 8, 8, 8, 8, 8, 8, 8, 8, 8, 8, 8, 8, 8, 8, 8, 8, 8, 8, 8, 8, 8, 7, 7, 7, 7, 7, 7, 7, 7, 7, 7, 7, 7, 7, 7, 7, 7, 7, 7, 7, 7, 7, 7, 7, 7, 7, 7, 7, 7, 6, 6, 6, 6, 6, 6, 6, 6, 6, 6, 6, 6, 6, 6, 6, 6, 6, 6, 6, 6, 6, 6, 6, 6, 6, 6, 6, 6, 6, 6, 6, 6, 6, 6, 6, 6, 6, 6, 6, 6, 6, 6, 6, 6, 6, 6, 6, 6, 6, 6, 6, 6, 6, 5, 5, 5, 5, 5, 5, 5, 5, 5, 5, 5, 5, 5, 5, 5, 5, 5, 5, 5, 5, 5, 5, 5, 5, 5, 5, 5, 5, 5, 5, 5, 5, 5, 4, 4, 4, 4, 4, 4, 4, 4, 4, 4, 4, 4, 4, 4, 4, 4, 4, 4, 4, 4, 4, 4, 4, 4, 4, 4, 4, 4, 4, 4, 4, 4, 4, 4, 4, 4, 4, 4, 4, 4, 4, 4, 4, 4, 4, 4, 4, 4, 4, 4, 4, 4, 4, 4, 3, 3, 3, 3, 3, 3, 3, 3, 3, 3, 3, 3, 3, 3, 3, 3, 3, 3, 3, 3, 3, 3, 3, 3, 3, 3, 3, 3, 3, 3, 3, 3, 3, 3, 3, 3, 3, 3, 3, 3, 3, 3, 3, 3, 3, 3, 3, 3, 3, 3, 3, 3, 3, 3, 3, 3, 3, 3, 3, 3, 2, 2, 2, 2, 2, 2, 2, 2, 2, 2, 2, 2, 2, 2, 2, 2, 2, 2, 2, 2, 2, 2, 2, 2, 2, 2, 2, 2, 2, 2, 2, 2, 2, 2, 2, 2, 2, 2, 2, 2, 2, 2, 2, 2, 2, 2, 2, 2, 2, 2, 2, 2, 2, 2, 2, 2, 2, 2, 2, 2, 2, 2, 2, 2, 2, 2, 2, 2, 2, 2, 2, 2, 2, 2, 2, 2, 2, 2, 2, 2, 2, 2, 2, 2, 2, 2, 2, 1

Corresponding number of selected Gene ontology terms each gene is involved with:
(In the same order as above)
16, 13, 12, 11, 10, 11, 11, 11, 9, 11, 8, 8, 7, 9, 7, 6, 6, 6, 6, 8, 8, 8, 9, 7, 9, 5, 5, 5, 5, 5, 7, 7, 11, 5, 5, 6, 6, 5, 4, 4, 4, 4, 9, 5, 4, 4, 4, 4, 4, 6, 6, 4, 5, 4, 4, 4, 5, 5, 3, 6, 4, 4, 3, 3, 3, 3, 3, 4, 3, 6, 4, 6, 4, 4, 5, 5, 3, 3, 3, 3, 3, 11, 3, 3, 8, 8, 3, 3, 5, 3, 5, 6, 3, 3, 6, 3, 4, 4, 4, 7, 4, 4, 4, 3, 3, 3, 3, 3, 3, 3, 5, 2, 2, 4, 3, 2, 4, 2, 3, 8, 2, 2, 3, 2, 2, 2, 2, 4, 2, 8, 3, 3, 6, 2, 4, 2, 2, 2, 2, 2, 2, 2, 2, 2, 2, 7, 7, 3, 3, 3, 3, 3, 3, 2, 2, 5, 2, 4, 4, 2, 2, 4, 5, 2, 2, 2, 5, 3, 6, 6, 2, 3, 6, 3, 2, 2, 6, 6, 3, 3, 2, 6, 2, 4, 2, 2, 2, 4, 2, 4, 2, 2, 4, 4, 4, 2, 4, 15, 3, 5, 3, 3, 5, 3, 3, 2, 2, 7, 7, 2, 2, 2, 2, 13, 13, 1, 3, 4, 6, 3, 1, 1, 6, 1, 6, 1, 4, 1, 4, 1, 3, 4, 2, 3, 1, 1, 1, 1, 1, 1, 4, 4, 4, 4, 1, 1, 2, 1, 2, 1, 1, 4, 6, 1, 3, 2, 3, 1, 1, 1, 1, 2, 3, 1, 1, 1, 2, 1, 1, 1, 1, 1, 1, 1, 1, 1, 1, 1, 1, 1, 1, 1, 1, 1, 1, 5, 1, 5, 1, 5, 5, 2, 5, 2, 2, 10, 2, 5, 2, 2, 2, 10, 2, 2, 5, 3, 1, 3, 3, 1, 1, 3, 1, 3, 1, 3, 1, 9, 1, 3, 1, 2, 2, 4, 1, 4, 8, 4, 2, 1, 1, 1, 1, 1, 1, 1, 2, 8, 2, 4, 4, 2, 2, 2, 2, 2, 1, 1, 4, 4, 1, 1, 1, 1, 4, 1, 8, 1, 8, 1, 1, 1, 1, 2, 1, 1, 1, 1, 4, 2, 8, 2, 4, 4, 1, 2, 1, 1, 1, 1, 1, 7, 1, 1, 1, 1, 7, 1, 7, 1, 7, 1, 1, 1, 1, 7, 7, 1, 7, 7, 1, 1, 1, 1, 1, 1, 3, 6, 2, 1, 2, 6, 6, 1, 2, 2, 6, 1, 6, 3, 1, 1, 1, 2, 1, 2, 3, 1, 2, 1, 3, 3, 1, 1, 3, 2, 2, 2, 3, 6, 3, 2, 2, 6, 1, 1, 1, 6, 3, 2, 2, 1, 2, 1, 2, 1, 2, 1, 3, 1, 1, 1, 5, 5, 1, 1, 1, 5, 1, 5, 1, 1, 5, 1, 5, 1, 5, 1, 1, 1, 1, 5, 5, 5, 1, 1, 1, 5, 1, 1, 1, 1, 2, 1, 1, 4, 2, 1, 1, 1, 1, 1, 1, 2, 2, 1, 4, 2, 4, 4, 2, 1, 1, 1, 4, 2, 2, 4, 2, 1, 1, 1, 2, 4, 4, 1, 4, 2, 2, 2, 2, 1, 4, 2, 1, 2, 2, 1, 4, 1, 2, 2, 2, 1, 2, 1, 1, 1, 1, 1, 3, 3, 3, 1, 3, 1, 3, 3, 3, 1, 3, 1, 1, 1, 3, 1, 1, 3, 1, 3, 1, 1, 3, 1, 1, 3, 1, 3, 3, 3, 1, 3, 3, 1, 3, 3, 3, 3, 3, 1, 3, 3, 1, 1, 1, 3, 1, 3, 1, 3, 3, 1, 1, 3, 1, 1, 2, 1, 2, 1, 1, 1, 2, 1, 2, 1, 1, 2, 1, 1, 1, 2, 2, 1, 1, 2, 1, 1, 2, 1, 2, 2, 1, 1, 1, 1, 2, 2, 2, 1, 2, 1, 1, 1, 1, 2, 2, 2, 2, 1, 1, 1, 1, 2, 2, 2, 2, 2, 1, 1, 2, 2, 1, 1, 1, 2, 1, 2, 2, 1, 2, 1, 1, 1, 2, 1, 1, 1, 2, 2, 1, 1, 1, 1, 1, 1, 2, 1, 2, 2, 1

Corresponding number of selected TFs each gene is involved with:
(In the same order as above)
10, 12, 12, 12, 12, 10, 10, 10, 12, 9, 11, 11, 12, 9, 11, 12, 12, 11, 11, 8, 8, 8, 7, 9, 7, 12, 12, 12, 12, 12, 8,

8, 5, 11, 11, 9, 9, 10, 12, 12, 12, 12, 5, 9, 11, 11, 11, 11, 11, 7, 7, 10, 8, 10, 10, 10, 8, 8, 12, 6, 9, 9, 12, 12, 12, 12, 12, 9, 12, 6, 9, 6, 9, 9, 7, 7, 11, 11, 11, 11, 11, 3, 11, 11, 4, 4, 10, 10, 6, 10, 6, 5, 10, 10, 5, 10, 7, 7, 7, 4, 7, 7, 7, 9, 9, 9, 9, 9, 9, 9, 5, 12, 12, 6, 8, 12, 6, 12, 8, 3, 12, 12, 8, 12, 12, 12, 12, 6, 12, 3, 8, 8, 4, 12, 6, 12, 11, 11, 11, 11, 11, 11, 11, 11, 11, 3, 3, 7, 7, 7, 7, 7, 7, 10, 10, 4, 10, 5, 5, 10, 10, 5, 4, 10, 10, 10, 4, 6, 3, 3, 9, 6, 3, 6, 9, 9, 3, 3, 6, 6, 9, 3, 9, 4, 8, 8, 8, 4, 8, 4, 8, 8, 4, 4, 4, 8, 4, 1, 5, 3, 5, 5, 3, 5, 5, 7, 7, 2, 2, 7, 7, 7, 7, 1, 1, 12, 4, 3, 2, 4, 12, 12, 2, 12, 2, 12, 3, 12, 3, 12, 4, 3, 6, 4, 12, 12, 12, 12, 12, 12, 3, 3, 3, 3, 12, 12, 6, 12, 6, 12, 12, 3, 2, 12, 4, 6, 4, 12, 12, 12, 12, 6, 4, 12, 12, 12, 6, 12, 11, 11, 11, 11, 11, 11, 11, 11, 11, 11, 11, 11, 11, 11, 11, 11, 11, 11, 11, 2, 10, 2, 10, 2, 2, 5, 2, 5, 5, 1, 5, 2, 5, 5, 5, 1, 5, 5, 2, 3, 9, 3, 3, 9, 9, 3, 9, 3, 9, 3, 9, 1, 9, 3, 8, 4, 4, 2, 8, 2, 1, 2, 4, 8, 8, 8, 8, 8, 8, 8, 4, 1, 4, 2, 2, 4, 4, 4, 4, 4, 8, 8, 2, 2, 8, 8, 8, 8, 2, 8, 1, 8, 1, 8, 8, 8, 8, 4, 8, 8, 8, 8, 2, 4, 1, 4, 2, 2, 8, 4, 8, 8, 7, 7, 7, 1, 7, 7, 7, 7, 1, 7, 1, 7, 1, 7, 7, 7, 7, 1, 1, 7, 1, 1, 7, 7, 7, 7, 7, 7, 2, 1, 3, 6, 3, 1, 1, 6, 3, 3, 1, 6, 1, 2, 6, 6, 6, 3, 6, 3, 2, 6, 3, 6, 2, 2, 6, 6, 2, 3, 3, 3, 2, 1, 2, 3, 3, 1, 6, 6, 6, 1, 2, 3, 3, 6, 3, 6, 3, 6, 3, 6, 2, 5, 5, 5, 1, 1, 5, 5, 5, 1, 5, 1, 5, 5, 1, 5, 1, 5, 1, 5, 5, 5, 5, 1, 1, 1, 5, 5, 5, 1, 5, 5, 5, 5, 2, 4, 4, 1, 2, 4, 4, 4, 4, 4, 4, 2, 2, 4, 1, 2, 1, 1, 2, 4, 4, 4, 1, 2, 2, 1, 2, 4, 4, 4, 2, 1, 1, 4, 1, 2, 2, 2, 2, 4, 1, 2, 4, 2, 2, 4, 1, 4, 2, 2, 2, 4, 2, 4, 3, 3, 3, 3, 1, 1, 1, 3, 1, 3, 1, 1, 1, 3, 1, 3, 3, 3, 1, 3, 3, 1, 3, 1, 3, 3, 1, 3, 3, 1, 3, 1, 1, 1, 3, 1, 1, 3, 1, 1, 1, 1, 1, 3, 1, 1, 3, 3, 3, 1, 3, 1, 3, 1, 1, 3, 3, 1, 3, 3, 1, 2, 1, 2, 2, 2, 1, 2, 1, 2, 2, 1, 2, 2, 2, 1, 1, 2, 2, 1, 2, 2, 1, 2, 1, 1, 2, 2, 2, 2, 1, 1, 1, 2, 1, 2, 2, 2, 2, 1, 1, 1, 1, 2, 2, 2, 2, 1, 1, 1, 1, 1, 2, 2, 1, 1, 2, 2, 2, 1, 2, 1, 1, 2, 1, 2, 2, 2, 1, 2, 2, 2, 1, 1, 2, 2, 2, 2, 2, 2, 1, 2, 1, 1, 2, 2, 2, 1

Gene ontology terms ranked according to mean of effect sizes in cluster:
embryonic development | chordate embryonic development | Notch signaling pathway | DNA binding | neurogenesis | segmentation | gene expression | somitogenesis | DBD domain binding | cell fate determination | anatomical structure morphogenesis | regionalization | gastrulation | organ development | central nervous system development | central nervous system neuron development | limb development

Corresponding mean of effect sizes of each Gene ontology term:
(In the same order as above)
4.01, 3.55, 3.23, 2.66, 2.57, 2.56, 2.46, 2.36, 2.30, 2.09, 2.03, 2.01, 1.97, 1.88, 1.41, 1.34, 1.27

Corresponding total number of genes of each Gene ontology term:
(In the same order as above)
1.2K, 671, 103, 1.2K, 409, 109, 3.8K, 116, 411, 145, 583, 62, 293, 339, 110, 37, 129

Corresponding number of genes of each Gene ontology term with at least one TFBS (of selected TFs):
(In the same order as above)
295, 173, 38, 269, 104, 39, 688, 30, 111, 42, 125, 24, 71, 83, 29, 10, 29

Corresponding total number of TFBS (of selected TFs) for each Gene ontology term (in all genes):
(In the same order as above)
1425, 882, 231, 1355, 534, 227, 3149, 187, 545, 249, 622, 141, 349, 421, 150, 64, 140

Corresponding number of selected TFs each Gene ontology term is involved with:
(In the same order as above)
12, 12, 12, 12, 12, 12, 12, 12, 12, 12, 12, 12, 12, 12, 12, 12, 12

TFs ranked according to mean of effect sizes in cluster:
V$E2F_Q2, V$E2F1_Q6, V$AP2GAMMA_01, V$AP2ALPHA_01, VETF_Q6, VAP2_Q6, V$E2F1_Q3, V$AP2_Q6_01, V$WT1_Q6, V$EGR_Q6, V$MAZR_01, V$MAZ_Q6

Corresponding mean of effect sizes of each TF:
(In the same order as above)
2.98, 2.88, 2.74, 2.73, 2.72, 2.35, 2.31, 2.21, 1.95, 1.76, 1.72, 1.64

Corresponding total number of TFBS for each TF (genome-wide):
(In the same order as above)
714, 691, 695, 686, 699, 688, 709, 690, 729, 695, 705, 734

Corresponding total number of TFBS for each TF (in all genes in selected Gene ontology terms):
(In the same order as above)
993, 970, 880, 870, 945, 919, 851, 884, 919, 840, 766, 834

Corresponding number of genes (of selected Gene ontology terms) each TF is involved with:
(In the same order as above)
346, 327, 315, 309, 344, 337, 315, 327, 337, 314, 296, 323

Corresponding number of selected Gene ontology terms each TF is involved with:
(In the same order as above)
17, 17, 17, 17, 17, 17, 17, 17, 17, 17, 17, 17

Cluster for columns 1210 to 1213, rows 392 to 410

Gene ontology terms

contractile ring | estrogen metabolic process | drug metabolic process | xenobiotic metabolic process

TFs

V$TAL1BETAE47_01 | V$RFX1_02 | V$HFH4_01 | V$AIRE_02 | V$MEF2_03 | V$MEF2_02 | V$RSRFC4_Q2 | V$RSRFC4_01 | V$PPAR_DR1_Q2 | V$DR1_Q3 | V$HNF4_DR1_Q3 | V$COUP_DR1_Q6 | V$COUP_01 | V$HNF4_01 | V$HNF4_Q6_01 | V$HNF4ALPHA_Q6 | V$COUPTF_Q6 | V$PPARG_03 | V$PPARG_01

Information

All related TFs:
(List of all TFs that are related to any of the PWMs)
AIRE, COUP, COUP-TF1, COUP-TF2, E47, FOXJ1, FOXJ1a, FOXJ1b, HNF-4, HNF-4alpha, HNF-4alpha1, HNF-4alpha2, HNF-4alpha3, HNF-4alpha4, HNF-4alpha7, HNF-4gamma, MEF-2A, PPAR-alpha, PPAR-alpha:RXR-alpha, PPAR-beta, PPAR-gamma, PPAR-gamma1, PPAR-gamma2, PPAR-gamma2:RXR-alpha, PPAR-gamma:RXR-alpha, RAR-alpha:RXR-alpha, RFX1, RSRFC4, Tal-1beta, aMEF-2

Ranked gene list:
(All genes of the selected Gene ontology terms with hits of any of the selected TFs, ranked according to the total number of TFBS)
SLC35A2, CYP1A1, PLEC1, CYP27B1, PPARA, COMT, SULT1A3, PPIG, NFE2L2, GABPA, AHR, RHOA, PGP, PPIF, EDC4, XPR1, UGT1A9, UGT1A6, SHBG, MYBBP1A, MBD2, PKP4, DBP, MPG, CYP2E1, CES2, UGT1A7, UGT1A10, SULT1A1, SOD2, SLC45A2, PCNT, MTHFR, INTU, FMO4, BRD2, TEF, TBC1D9, NR6A1, NR1H4, NQO1, MGST1, GADD45B, CYP7A1, CYP17A1, CHST6, CALM3, ABCB4, UGT1A8, UGT1A5, UGT1A4, TPMT, TNFSF13, SULT2B1, SLC38A1, RXRA, NR3C1, NR2F2, NGEF, JAM3, IBSP, HNF4A, GSTT2B, GSTT2, FOXO1, FBXW7, ESR1, DECR1, CYCS, CDC7, ARR3, ALDH2, ALAS1, ABCC1, XRCC1, UGT1A3, TBC1D8, TAT, SLC40A1, SLC26A2, PON3, PLEKHG6, PHIP, PELP1, PC, OGG1, NDOR1, MED1, MDM4, MAP3K8, KEAP1, INSM1, HTR1A, HSPBP1, GUSB, GUCY1B3, GRIN1, GPX2, GPT, GLB1, GHRHR, FUT1, ESR2, DPYD, DMPK, CXADR, CSH1, CLSTN2, CHST7, CDC42, CDC14A, CD46, CD247, CCL4L2,

CCL4L1, CAT, CALM1, BCHE, BAMBI

Corresponding total number of TFBS:
(For each gene listed above, the total number of TFBS for any of the selected TFs, multiplied by the number of selected Gene ontology terms containing that gene)
36, 36, 32, 30, 24, 24, 20, 20, 15, 15, 12, 11, 11, 9, 9, 8, 8, 8, 8, 8, 8, 7, 7, 6, 6, 5, 4, 4, 4, 4, 4, 4, 4, 4, 4, 4, 3, 3, 3, 3, 3, 3, 3, 3, 3, 3, 3, 3, 2, 2, 2, 2, 2, 2, 2, 2, 2, 2, 2, 2, 2, 2, 2, 2, 2, 2, 2, 2, 2, 2, 2, 2, 1, 1, 1, 1, 1, 1, 1, 1, 1, 1, 1, 1, 1, 1, 1, 1, 1, 1, 1, 1, 1, 1, 1, 1, 1, 1, 1, 1, 1, 1, 1, 1, 1, 1, 1, 1, 1, 1, 1, 1, 1, 1, 1, 1, 1

Corresponding number of selected Gene ontology terms each gene is involved with:
(In the same order as above)
4, 4, 2, 3, 3, 3, 2, 4, 3, 3, 4, 1, 1, 3, 1, 2, 4, 4, 1, 1, 2, 1, 1, 1, 3, 1, 2, 2, 4, 1, 4, 2, 2, 1, 1, 2, 1, 3, 1, 3, 3, 1, 1, 3, 3, 1, 1, 1, 1, 1, 1, 1, 2, 1, 2, 1, 1, 1, 1, 1, 1, 2, 2, 2, 1, 1, 1, 2, 2, 1, 1, 1, 1, 2, 1, 1, 1, 1, 1, 1, 1, 1, 1, 1, 1, 1, 1, 1, 1, 1, 1, 1, 1, 1, 1, 1, 1, 1, 1, 1, 1, 1, 1, 1, 1, 1, 1, 1, 1, 1, 1, 1, 1, 1, 1, 1, 1

Corresponding number of selected TFs each gene is involved with:
(In the same order as above)
9, 9, 16, 10, 8, 8, 10, 5, 5, 5, 3, 11, 11, 3, 9, 4, 2, 2, 8, 8, 4, 7, 7, 6, 2, 5, 2, 2, 1, 4, 1, 2, 2, 4, 4, 2, 3, 1, 3, 1, 1, 3, 3, 1, 1, 3, 3, 3, 2, 2, 2, 2, 1, 2, 1, 2, 2, 2, 2, 2, 2, 1, 1, 1, 2, 2, 2, 1, 1, 2, 2, 2, 2, 1, 1, 1, 1, 1, 1, 1, 1, 1, 1, 1, 1, 1, 1, 1, 1, 1, 1, 1, 1, 1, 1, 1, 1, 1, 1, 1, 1, 1, 1, 1, 1, 1, 1, 1, 1, 1, 1, 1, 1, 1, 1, 1, 1

Gene ontology terms ranked according to mean of effect sizes in cluster:
estrogen metabolic process | xenobiotic metabolic process | drug metabolic process | contractile ring

Corresponding mean of effect sizes of each Gene ontology term:
(In the same order as above)
2.52, 2.10, 1.90, 1.54

Corresponding total number of genes of each Gene ontology term:
(In the same order as above)
37, 128, 150, 150

Corresponding number of genes of each Gene ontology term with at least one TFBS (of selected TFs):
(In the same order as above)
18, 51, 59, 56

Corresponding total number of TFBS (of selected TFs) for each Gene ontology term (in all genes):
(In the same order as above)
66, 141, 180, 169

Corresponding number of selected TFs each Gene ontology term is involved with:
(In the same order as above)
19, 19, 19, 19

TFs ranked according to mean of effect sizes in cluster:
V$PPARG_01, V$HNF4_DR1_Q3, V$COUP_01, V$DR1_Q3, V$HNF4_Q6_01, V$AIRE_02, V$PPAR_DR1_Q2, V$HNF4_01, V$TAL1BETAE47_01, V$PPARG_03, V$COUP_DR1_Q6, V$MEF2_03, V$RFX1_02, V$RSRFC4_01, V$HNF4ALPHA_Q6, V$RSRFC4_Q2, V$COUPTF_Q6, V$HFH4_01, V$MEF2_02

Corresponding mean of effect sizes of each TF:
(In the same order as above)
3.22, 3.13, 2.71, 2.56, 2.43, 2.30, 2.26, 2.24, 2.23, 2.21, 2.15, 2.14, 1.92, 1.55, 1.45, 1.37, 1.02, 0.74, 0.63

Corresponding total number of TFBS for each TF (genome-wide):

(In the same order as above)
642, 666, 660, 667, 687, 607, 684, 674, 684, 647, 651, 650, 613, 655, 688, 655, 696, 619, 700

Corresponding total number of TFBS for each TF (in all genes in selected Gene ontology terms):
(In the same order as above)
31, 35, 32, 31, 32, 44, 29, 29, 29, 44, 27, 30, 26, 24, 26, 24, 20, 21, 22

Corresponding number of genes (of selected Gene ontology terms) each TF is involved with:
(In the same order as above)
21, 19, 18, 15, 18, 26, 15, 14, 15, 25, 14, 15, 16, 13, 15, 14, 14, 15, 14

Corresponding number of selected Gene ontology terms each TF is involved with:
(In the same order as above)
4, 4, 4, 4, 4, 4, 4, 4, 4, 4, 4, 4, 4, 4, 4, 4, 4, 4, 4

Cluster for columns 7 to 11, rows 143 to 151

Gene ontology terms

cell activation | T cell activation | T cell receptor complex | immunological synapse | integrin activation

TFs

V$PEA3_Q6 | V$ETS_Q6 | V$PU1_Q6 | V$PXR_Q2 | V$TEL2_Q6 | V$ETS_Q4 | V$ELF1_Q6 | V$NKX25_Q5 | V$ETS2_B

Information

All related TFs:
(List of all TFs that are related to any of the PWMs)
CAR, CAR2:RXR-alpha, CAR:RXR-alpha, ELF-1, ELFR, ERF, Elf-1, Elk-1, Elk-1-isoform1, Erg-1, Erg-2, Ets-1, FXR, FXR:RXR-alpha, Fli-1, GABP-alpha, GABP-alpha:GABP-beta, GABP-beta1, GABP-beta2, LXR-alpha:RXR-alpha, LXR-beta:RXR-alpha, NERF, NERF-1a, NERF-1b, NERF-2, Net, Nkx2-5, Nkx2.5, PEA3, PU.1, PU.1-xbb1, PXR-1, PXR-1A, PXR-2, PXR:RXR-alpha, SAP-1a, SAP-1b, Spi-B, TCF, TEL1, Tel-2a, Tel-2b, Tel-2c, Tel-2d, Tel-2e, Tel-2f, c-Ets-1, c-Ets-1A, c-Ets-1B, c-Ets-2, c-Ets-2A, c-Ets-2B, p38erg, p49erg, p55, p55erg

Ranked gene list:
(All genes of the selected Gene ontology terms with hits of any of the selected TFs, ranked according to the total number of TFBS)
PLCG1, LCK, CCR7, AKT1, PTPN6, CSK, ITGB2, ZAP70, LCP2, VAV1, TNF, SHC1, PER1, NFATC1, LTB, LAG3, ITGA5, CISH, SKAP1, SH2B3, PTPN7, LCP1, JUNB, TNFRSF1A, TCIRG1, TCF7, CD4, BCL11B, FASN, EDC4, CXCR4, RASSF5, DUSP2, CD68, TYROBP, TNFRSF18, PTK2B, PSTPIP1, LTBR, HCST, GFI1, EGR1, CD82, CD2BP2, CCND3, TNFSF13, TLR9, SOCS1, MAP4K1, LTA, FYN, CD63, ZBTB7B, TNFRSF4, TBX21, TBC1D10C, NFKBIA, MEF2D, IRF1, GATA3, DGKA, CD3G, CD19, SSSCA1, SH3BP2, SATB1, PIK3CG, ISG20, IL16, DKFZp434P0672, ARHGEF2, ANXA6, XAB2, SIPA1, RAC2, PPP1R9B, MYO1G, ITGB7, ILK, CD79B, STAT5A, SH2D2A, RAP1GAP, PTPN11, PKN1, NFATC3, FERMT3, FAS, EFNA2, CD53, CD3D, BHLHB2, BCL6, SOCS3, SLC2A4RG, RASGRP2, PRDM1, NR4A1, ITPR3, INPP5D, GPR132, CSF1, CD37, WIPF1, WASF2, VAV2, TNFRSF6B, TNFRSF1B, STIM1, STAT6, SPN, SELL, RAF1, POU2F2, POLD4, PIK3R1, NCK1, MAPK14, LYL1, KCNA3, ITGB1, ITGA4, IKZF1, ICAM3, ICAM2, HMHA1, GIMAP5, EXTL3, DOK2, CXCR5, CTLA4, CASP8, CAP1, CALR, CALM2, ALCAM, VAV3, TFRC, TAPBP, TAP1, STAT5B, STAT4, SRF, SLC19A2, SEMA4D, SELPLG, PIK3CD, NFATC2, MBP, MAP3K1,

LEF1, JUN, JAK3, IL2RB, ID3, GRB2, ETS1, DIABLO, DEF6, DAPP1, CRK, CDKN1B, CD5, CD44, CD27, CD247, VEGFA, TSPAN4, SMAD3, SILV, RHOA, RAP1B, PSMB9, PRIM2, PRDX5, PIK3R2, PHLDA1, PEA15, PDLIM7, PDE4A, ORAI1, NR1H2, NCR3, MYB, MR1, MARCKSL1, KCNN4, ISYNA1, IL7R, IL23A, IL21R, HRAS, HNRNPL, EPHA1, EGR2, DPP8, DOCK2, CREB1, CD74, CD180, CANX, BTLA, ARHGEF7, WDR48, UNC13D, TRAF1, TNFRSF25, TGFA, TCF12, STRA13, STAT3, SQSTM1, RNF114, RERE, RAB3GAP1, PXN, PLEKHA2, NFKB1, MYD88, MTHFR, LYN, LAMP1, KIAA0101, KAT5, JUND, ITPR1, IL18R1, ICAM5, GUK1, GPR172B, FKBP1A, ERCC6, DNTT, CDH15, CD3E, CBLB, BTN3A1, BSG, AIF1

Corresponding total number of TFBS:
(For each gene listed above, the total number of TFBS for any of the selected TFs, multiplied by the number of selected Gene ontology terms containing that gene)
40, 40, 40, 40, 36, 36, 35, 32, 30, 25, 24, 24, 24, 24, 24, 24, 24, 24, 20, 18, 18, 18, 18, 16, 16, 16, 16, 16, 15, 15, 15, 14, 14, 14, 12, 12, 12, 12, 12, 12, 12, 12, 12, 12, 10, 10, 10, 10, 10, 10, 10, 9, 9, 9, 9, 9, 9, 9, 9, 9, 9, 9, 8, 8, 8, 8, 8, 8, 8, 8, 8, 7, 7, 7, 7, 7, 7, 7, 7, 6, 6, 6, 6, 6, 6, 6, 6, 6, 6, 6, 6, 5, 5, 5, 5, 5, 5, 5, 5, 5, 5, 4, 4, 4, 4, 4, 4, 4, 4, 4, 4, 4, 4, 4, 4, 4, 4, 4, 4, 4, 4, 4, 4, 4, 4, 4, 4, 4, 4, 4, 4, 4, 4, 3, 3, 3, 3, 3, 3, 3, 3, 3, 3, 3, 3, 3, 3, 3, 3, 3, 3, 3, 3, 3, 3, 3, 3, 3, 3, 3, 3, 2, 2, 2, 2, 2, 2, 2, 2, 2, 2, 2, 2, 2, 2, 2, 2, 2, 2, 2, 2, 2, 2, 2, 2, 2, 2, 2, 2, 2, 2, 2, 2, 2, 2, 2, 1, 1, 1, 1, 1, 1, 1, 1, 1, 1, 1, 1, 1, 1, 1, 1, 1, 1, 1, 1, 1, 1, 1, 1, 1, 1, 1, 1, 1, 1, 1, 1, 1, 1

Corresponding number of selected Gene ontology terms each gene is involved with:
(In the same order as above)
5, 5, 5, 5, 4, 4, 5, 4, 5, 5, 3, 3, 3, 3, 3, 3, 3, 4, 3, 4, 2, 2, 3, 2, 2, 2, 2, 4, 2, 3, 3, 5, 2, 2, 2, 2, 3, 4, 2, 4, 2, 2, 2, 2, 4, 2, 5, 2, 2, 2, 2, 5, 2, 1, 3, 3, 1, 3, 1, 1, 1, 1, 3, 3, 1, 1, 2, 4, 4, 2, 2, 1, 2, 1, 1, 1, 1, 1, 1, 1, 1, 3, 3, 1, 2, 1, 1, 1, 3, 2, 2, 2, 1, 1, 1, 1, 1, 1, 1, 1, 1, 1, 1, 1, 2, 4, 2, 1, 2, 2, 2, 4, 4, 4, 1, 1, 2, 4, 4, 1, 4, 4, 4, 1, 2, 2, 1, 1, 2, 1, 2, 4, 2, 1, 1, 2, 2, 3, 3, 1, 1, 3, 3, 1, 3, 3, 1, 3, 3, 3, 1, 1, 3, 3, 3, 1, 3, 3, 1, 1, 1, 3, 1, 3, 3, 3, 3, 1, 2, 1, 1, 2, 1, 1, 1, 1, 2, 2, 1, 1, 1, 1, 1, 1, 2, 2, 1, 2, 1, 2, 2, 1, 2, 1, 1, 2, 1, 2, 2, 1, 1, 1, 2, 2, 1, 1, 1, 1, 1, 1, 1, 1, 1, 1, 1, 1, 1, 1, 1, 1, 1, 1, 1, 1, 1, 1, 1, 1, 1, 1, 1, 1, 1, 1, 1, 1, 1, 1, 1

Corresponding number of selected TFs each gene is involved with:
(In the same order as above)
8, 8, 8, 8, 9, 9, 7, 8, 6, 5, 8, 8, 8, 8, 8, 8, 6, 8, 5, 9, 9, 6, 9, 8, 8, 8, 4, 8, 5, 5, 3, 7, 7, 7, 6, 4, 3, 6, 3, 6, 6, 6, 6, 3, 6, 2, 5, 5, 5, 5, 5, 2, 5, 9, 3, 3, 9, 3, 9, 9, 9, 9, 3, 3, 8, 8, 4, 2, 2, 4, 4, 8, 4, 7, 7, 7, 7, 7, 7, 7, 7, 2, 2, 6, 3, 6, 6, 6, 2, 3, 3, 3, 6, 6, 5, 5, 5, 5, 5, 5, 5, 5, 5, 5, 2, 1, 2, 4, 2, 2, 2, 1, 1, 1, 4, 4, 2, 1, 1, 4, 1, 1, 1, 4, 2, 2, 4, 4, 2, 4, 2, 1, 2, 4, 4, 2, 2, 1, 1, 3, 3, 1, 1, 3, 1, 1, 3, 1, 1, 1, 3, 3, 1, 1, 1, 3, 1, 1, 3, 3, 3, 1, 3, 1, 1, 1, 1, 2, 1, 2, 2, 1, 2, 2, 2, 2, 1, 1, 2, 2, 2, 2, 2, 2, 1, 1, 2, 1, 2, 1, 1, 2, 1, 2, 2, 1, 2, 1, 1, 2, 2, 2, 1, 1, 1, 1, 1, 1, 1, 1, 1, 1, 1, 1, 1, 1, 1, 1, 1, 1, 1, 1, 1, 1, 1, 1, 1, 1, 1, 1, 1, 1, 1, 1, 1, 1, 1, 1, 1

Gene ontology terms ranked according to mean of effect sizes in cluster:
cell activation | integrin activation | T cell receptor complex | immunological synapse | T cell activation

Corresponding mean of effect sizes of each Gene ontology term:
(In the same order as above)
2.57, 2.46, 2.33, 2.11, 1.82

Corresponding total number of genes of each Gene ontology term:
(In the same order as above)
596, 155, 478, 112, 287

Corresponding number of genes of each Gene ontology term with at least one TFBS (of selected TFs):
(In the same order as above)
163, 48, 136, 41, 88

Corresponding total number of TFBS (of selected TFs) for each Gene ontology term (in all genes):
(In the same order as above)

565, 181, 485, 156, 307

Corresponding number of selected TFs each Gene ontology term is involved with:
(In the same order as above)
9, 9, 9, 9, 9

TFs ranked according to mean of effect sizes in cluster:
V$ETS2_B, V$ETS_Q6, V$ELF1_Q6, V$ETS_Q4, V$PEA3_Q6, V$TEL2_Q6, V$PU1_Q6, V$NKX25_Q5, V$PXR_Q2

Corresponding mean of effect sizes of each TF:
(In the same order as above)
2.56, 2.56, 2.56, 2.54, 2.42, 2.40, 2.15, 1.91, 1.21

Corresponding total number of TFBS for each TF (genome-wide):
(In the same order as above)
722, 721, 717, 718, 717, 707, 724, 695, 684

Corresponding total number of TFBS for each TF (in all genes in selected Gene ontology terms):
(In the same order as above)
200, 190, 202, 199, 189, 187, 190, 172, 165

Corresponding number of genes (of selected Gene ontology terms) each TF is involved with:
(In the same order as above)
95, 93, 96, 95, 89, 91, 91, 91, 85

Corresponding number of selected Gene ontology terms each TF is involved with:
(In the same order as above)
5, 5, 5, 5, 5, 5, 5, 5, 5

Cluster for columns 856 to 873, rows 251 to 258

Gene ontology terms

response to hypoxia | activation of protein kinase C activity | protein kinase C activity | soluble NSF attachment protein activity | arginase activity | nitric oxide biosynthetic process | D-nopaline dehydrogenase activity | nitric-oxide synthase activity | nitric-oxide synthase regulator activity | nascent polypeptide-associated complex | response to reactive oxygen species | heme catabolic process | superoxide release | myosin light chain kinase activity | stress fiber formation | smooth muscle contraction | regulation of vascular smooth muscle contraction | vascular smooth muscle contraction

TFs

V$AP1_Q6_01 | V$AP1_Q4_01 | V$AP1_Q6 | V$AP1_C | V$AP1_01 | V$AP1_Q2 | V$AP1FJ_Q2 | V$AP1_Q4

Information

All related TFs:
(List of all TFs that are related to any of the PWMs)
AP-1, FosB, Fra-1, Fra-2, JunB, JunB:Fra-1, JunB:Fra-2, JunD, JunD:Fra-2, JunD:deltaFosB, YAP1, c-Fos, c-Jun, c-Jun:FosB, c-Jun:JunD, c-Jun:c-Fos, deltaFosB

Ranked gene list:
(All genes of the selected Gene ontology terms with hits of any of the selected TFs, ranked according to the total number of TFBS)
TNF, MTHFR, AKT1, ISYNA1, CALM1, CDKN1A, NOS2, HMOX1, DDAH2, RHOA, ILK, VASP, PKM2, NLRP3, VEGFA, CALM3, PTK2B, MARK2, SSSCA1, LMNA, JUNB, DGKZ, ARHGEF2, ADM, SLC2A1, RELA, TNFRSF1A, NQO1, CD44, DGKA, FBRS, VCL, CSF2, ZYX, VDR, SQSTM1, SPSB2, SLC2A4RG, SIAH2, SHC1, SFN, S100A6, RHOC, RALGDS, PXN, PRKCD, PFN1, NFKBIA, LTB, IRF1, HMOX2, HGS, H2AFX, GAPDH, FHOD1, EFNA3, EDN2, DKFZp779C0757, DDR1, CNTNAP1, BHLHB2, BAG3, BACH1, ARHGEF1, ACY1, MYL6, LDHA, LCK, CSF1, BUD31, SOD1, PSMD9, PPP1R14A, PLCG1, PKN1, PELP1, NUP43, IL10, HSPA9, FLJ32987, CNP, ZC3H12D, UBC, SPINT1, S1PR2, RPS6KA1, RND1, NDRG1, MAD2L1BP, KSR1, IL2RB, GCLC, FOS, EGLN2, CNN1, STX1A, SNAPC2, RGS3, PRAP1, MPRIP, MAPK3, MAP2K1, JAG2, HSPB6, EGR1, DCTN3, CASP9, AMT, VAMP8, SEC61A1, PICK1, PER1, NFE2L2, NACC1, MAPK13, JUND, COL11A2, BCL2L1, BAX, ADA, UROD, SDC4, RAB5A, PTPN1, PRX, PLAU, PLA2G6, PHB2, MXI1, MAPKAPK2, KLF6, KCNJ11, ITGA3, GSTP1, GSR, GLTSCR2, FASN, CYBA, CLCN3, CA9, ADRBK1, TIPARP, TINAGL1, STRA13, SND1, SLC35A2, S1PR1, RND2, RGS2, PRKAB1, PPP1R15A, PAOX, OGG1, OBSCN, MRLC3, MAPK12, MAPK11, MAP3K1, LBR, IVL, HIF1AN, FECH, DYNC1H1, DDIT4, ARF1, ADRA1A

Corresponding total number of TFBS:
(For each gene listed above, the total number of TFBS for any of the selected TFs, multiplied by the number of selected Gene ontology terms containing that gene)
96, 91, 91, 78, 70, 48, 42, 30, 30, 28, 25, 24, 24, 24, 21, 20, 18, 18, 16, 16, 16, 16, 16, 16, 15, 15, 14, 14, 14, 12, 10, 9, 9, 8, 8, 8, 8, 8, 8, 8, 8, 8, 8, 8, 8, 8, 8, 8, 8, 8, 8, 8, 8, 8, 8, 8, 8, 8, 8, 8, 8, 8, 8, 8, 7, 7, 7, 7, 7, 6, 6, 6, 6, 6, 6, 6, 6, 6, 6, 6, 5, 5, 5, 5, 5, 5, 5, 5, 5, 5, 5, 5, 5, 5, 4, 4, 4, 4, 4, 4, 4, 4, 4, 4, 4, 4, 4, 3, 3, 3, 3, 3, 3, 3, 3, 3, 3, 3, 3, 2, 2, 2, 2, 2, 2, 2, 2, 2, 2, 2, 2, 2, 2, 2, 2, 2, 2, 2, 2, 1, 1, 1, 1, 1, 1, 1, 1, 1, 1, 1, 1, 1, 1, 1, 1, 1, 1, 1, 1, 1, 1, 1, 1

Corresponding number of selected Gene ontology terms each gene is involved with:
(In the same order as above)
12, 13, 13, 13, 10, 6, 14, 6, 5, 7, 5, 3, 3, 3, 7, 10, 6, 3, 2, 2, 2, 2, 2, 2, 3, 3, 2, 7, 2, 2, 2, 3, 3, 1, 1, 1, 1, 1, 1, 1, 1, 1, 1, 1, 4, 2, 1, 2, 1, 1, 4, 1, 2, 1, 1, 1, 2, 1, 1, 1, 1, 1, 1, 1, 1, 1, 1, 1, 1, 1, 2, 2, 6, 1, 2, 2, 2, 6, 2, 1, 2, 1, 1, 1, 1, 1, 1, 1, 1, 1, 1, 1, 5, 1, 1, 1, 1, 1, 1, 1, 2, 1, 1, 2, 2, 1, 2, 1, 1, 1, 3, 3, 3, 1, 1, 1, 3, 1, 3, 1, 1, 2, 1, 1, 2, 2, 2, 1, 1, 1, 1, 1, 1, 1, 2, 1, 2, 2, 1, 1, 1, 1, 1, 1, 1, 1, 1, 1, 1, 1, 1, 1, 1, 1, 1, 1, 1, 1, 1, 1, 1, 1, 1, 1

Corresponding number of selected TFs each gene is involved with:
(In the same order as above)
8, 7, 7, 6, 7, 8, 3, 5, 6, 4, 5, 8, 8, 8, 3, 2, 3, 6, 8, 8, 8, 8, 8, 8, 5, 5, 7, 2, 7, 6, 5, 3, 3, 8, 8, 8, 8, 8, 8, 8, 8, 8, 8, 8, 2, 4, 8, 4, 8, 8, 2, 8, 4, 8, 8, 8, 4, 8, 8, 8, 8, 8, 8, 8, 8, 7, 7, 7, 7, 7, 3, 3, 1, 6, 3, 3, 3, 1, 3, 6, 3, 5, 5, 5, 5, 5, 5, 5, 5, 5, 5, 5, 1, 5, 5, 4, 4, 4, 4, 4, 2, 4, 4, 2, 2, 4, 2, 4, 3, 3, 1, 1, 1, 3, 3, 3, 1, 3, 1, 3, 2, 1, 2, 2, 1, 1, 1, 2, 2, 2, 2, 2, 2, 2, 1, 2, 1, 1, 2, 2, 2, 1, 1, 1, 1, 1, 1, 1, 1, 1, 1, 1, 1, 1, 1, 1, 1, 1, 1, 1, 1, 1, 1, 1

Gene ontology terms ranked according to mean of effect sizes in cluster:
stress fiber formation | nascent polypeptide-associated complex | protein kinase C activity | regulation of vascular smooth muscle contraction | myosin light chain kinase activity | nitric oxide biosynthetic process | response to hypoxia | vascular smooth muscle contraction | nitric-oxide synthase regulator activity | smooth muscle contraction | activation of protein kinase C activity | response to reactive oxygen species | heme catabolic process | soluble NSF attachment protein activity | nitric-oxide synthase activity | arginase activity | D-nopaline dehydrogenase activity | superoxide release

Corresponding mean of effect sizes of each Gene ontology term:
(In the same order as above)
1.95, 1.70, 1.55, 1.47, 1.44, 1.35, 1.27, 1.25, 1.24, 1.21, 1.13, 1.06, 1.05, 0.88, 0.87, 0.76, 0.75, 0.47

Corresponding total number of genes of each Gene ontology term:
(In the same order as above)

165, 136, 157, 51, 130, 101, 124, 27, 43, 105, 302, 115, 57, 119, 56, 44, 77, 48

Corresponding number of genes of each Gene ontology term with at least one TFBS (of selected TFs):
(In the same order as above)
34, 33, 29, 10, 24, 13, 32, 8, 10, 15, 54, 26, 14, 23, 11, 7, 14, 6

Corresponding total number of TFBS (of selected TFs) for each Gene ontology term (in all genes):
(In the same order as above)
170, 122, 140, 47, 117, 74, 123, 32, 49, 62, 229, 89, 49, 95, 50, 32, 63, 25

Corresponding number of selected TFs each Gene ontology term is involved with:
(In the same order as above)
8, 8, 8, 8, 8, 8, 8, 8, 8, 8, 8, 8, 8, 8, 8, 8, 8, 8

TFs ranked according to mean of effect sizes in cluster:
V$AP1_Q4, V$AP1_Q6_01, V$AP1_Q2, V$AP1FJ_Q2, V$AP1_01, V$AP1_Q4_01, V$AP1_Q6, V$AP1_C

Corresponding mean of effect sizes of each TF:
(In the same order as above)
1.47, 1.35, 1.33, 1.30, 1.24, 1.17, 0.85, 0.81

Corresponding total number of TFBS for each TF (genome-wide):
(In the same order as above)
712, 726, 692, 710, 721, 719, 721, 720

Corresponding total number of TFBS for each TF (in all genes in selected Gene ontology terms):
(In the same order as above)
213, 212, 203, 196, 193, 196, 181, 174

Corresponding number of genes (of selected Gene ontology terms) each TF is involved with:
(In the same order as above)
87, 91, 82, 82, 92, 89, 82, 84

Corresponding number of selected Gene ontology terms each TF is involved with:
(In the same order as above)
18, 18, 18, 18, 18, 18, 18, 18

Cluster for columns 5 to 11, rows 193 to 198

Gene ontology terms

T cell differentiation | cell development | cell activation | T cell activation | T cell receptor complex | immunological synapse | integrin activation

TFs

V$AR_Q6 | V$PR_Q2 | V$GR_Q6_01 | V$AML1_Q6 | V$AML1_01 | V$OSF2_Q6

Information

All related TFs:

(List of all TFs that are related to any of the PWMs)
AML1, AML1a, AML3, AML3-isoform2, AR, GR, GR-alpha, GR-beta, PR, PR-alpha, PR-beta, RUNX2-isoform2

Ranked gene list:
(All genes of the selected Gene ontology terms with hits of any of the selected TFs, ranked according to the total number of TFBS)
ITGB2, CCR7, CSK, CD4, CXCR4, PTPN6, PLCG1, AKT1, NFATC1, FASN, TNF, TCF7, LTB, ETS1, CISH, BCL11B, LCK, TNFRSF4, TNFRSF18, SH2B3, PTK2B, GFI1, FYB, ZAP70, SOCS1, PER1, LTA, LEF1, FYN, CDKN1B, SKAP1, IL17C, SEMA4D, LYL1, LAG3, GATA3, CD7, CD3G, CD27, BCL2, ZBTB7B, VEGFA, TLR9, TCIRG1, STAT1, SHC1, RUNX1, RASSF5, PTPN7, POU2F2, LCP1, JUNB, IRF1, IL7R, IL2RA, DUSP2, BCL6, STAT5B, STAT5A, NR4A1, NFATC3, ITGA5, CD82, CD6, CD44, STAT6, IKZF1, CD3D, TSPAN4, SSSCA1, SATB1, LY6E, GIMAP5, EDC4, CSF2, LCP2, TNFRSF1A, TAL1, STK16, SH3BP2, RUNX3, PSTPIP1, PIK3R1, PAX6, MYO1G, MEF2D, LFNG, KCNN4, ITGB7, IL21R, HRAS, EGR1, DLL1, CDK5R1, CD63, CCND2, BCR, BCL2L11, ARHGEF2, SLC2A4RG, PPP1R9B, HMHA1, HES5, GUK1, FOXP3, DEF6, CD5, AMH, TRAF1, TBC1D10C, STRA13, STIM1, SIPA1, SELPLG, PXN, PRDM1, PDCD1, MYD88, LBX1, LAT, ITGA6, IL16, HNRNPL, FLI1, DGKA, CDH23, CD247, CD226, ZFPM1, VAV3, UNC13D, UBC, TNFSF14, TNFRSF6B, TAPBP, PKN1, PDE4A, NOTCH1, MAL, ISYNA1, INPP5D, ILK, IL23A, IFITM3, GPR132, GORASP2, GLI1, CXCR5, C21orf33, ZEB1, XAB2, TNFRSF1B, TNFRSF13C, TAP1, SOCS3, SILV, RASGRP2, NOTCH4, NOL3, NKX2-2, NGFR, NEDD9, MAT2A, ID3, ICAM3, FOS, FGR, EGR3, EBF1, CSF1, CFLAR, CD79B, CD68, CD47, CD300A, CCND3, BHLHB2, AIRE, XPC, SSFA2, PROX1, POU4F1, POU3F2, POLD4, PAX5, NR4A2, MUC1, ILF3, ID2, GATA2, FLII, FERMT3, DOK2, DNMT3A, DIABLO, DECR1, DCXR, DCTN2, DAPP1, CDH15, CD74, CBLB, CAP1, CALR, BATF

Corresponding total number of TFBS:
(For each gene listed above, the total number of TFBS for any of the selected TFs, multiplied by the number of selected Gene ontology terms containing that gene)
42, 42, 36, 36, 35, 30, 30, 30, 25, 25, 24, 24, 24, 24, 24, 24, 21, 20, 20, 20, 20, 20, 20, 18, 18, 18, 18, 18, 18, 18, 16, 16, 15, 15, 15, 15, 15, 15, 15, 15, 12, 12, 12, 12, 12, 12, 12, 12, 12, 12, 12, 12, 12, 12, 12, 12, 10, 10, 10, 10, 10, 10, 10, 10, 9, 9, 9, 8, 8, 8, 8, 8, 8, 8, 7, 6, 6, 6, 6, 6, 6, 6, 6, 6, 6, 6, 6, 6, 6, 6, 6, 6, 6, 6, 6, 6, 6, 5, 5, 5, 5, 5, 5, 5, 5, 5, 5, 4, 4, 4, 4, 4, 4, 4, 4, 4, 4, 4, 4, 4, 4, 4, 4, 4, 4, 4, 4, 3, 3, 3, 3, 3, 3, 3, 3, 3, 3, 3, 3, 3, 3, 3, 3, 3, 3, 3, 3, 3, 3, 2, 2, 2, 2, 2, 2, 2, 2, 2, 2, 2, 2, 2, 2, 2, 2, 2, 2, 2, 2, 2, 2, 2, 2, 2, 2, 2, 2, 2, 1, 1, 1, 1, 1, 1, 1, 1, 1, 1, 1, 1, 1, 1, 1, 1, 1, 1, 1, 1, 1, 1, 1, 1, 1

Corresponding number of selected Gene ontology terms each gene is involved with:
(In the same order as above)
7, 7, 6, 6, 7, 5, 5, 6, 5, 5, 4, 4, 4, 4, 4, 4, 7, 4, 4, 4, 5, 4, 5, 6, 3, 3, 3, 3, 6, 3, 4, 4, 3, 3, 3, 3, 5, 5, 5, 5, 2, 2, 2, 2, 4, 4, 3, 2, 2, 2, 3, 2, 2, 4, 6, 2, 2, 5, 5, 2, 2, 5, 2, 5, 5, 3, 3, 3, 2, 2, 4, 2, 2, 4, 4, 7, 2, 3, 1, 1, 2, 2, 3, 1, 1, 1, 1, 2, 1, 2, 2, 3, 1, 3, 2, 2, 3, 3, 1, 1, 1, 1, 1, 1, 5, 1, 5, 1, 1, 1, 1, 2, 1, 1, 1, 2, 1, 2, 2, 2, 4, 2, 1, 1, 1, 1, 4, 2, 1, 3, 1, 3, 1, 1, 1, 1, 1, 3, 1, 1, 1, 1, 3, 1, 1, 3, 1, 3, 1, 1, 1, 2, 2, 1, 1, 1, 1, 2, 2, 1, 2, 1, 1, 2, 2, 2, 1, 2, 2, 2, 2, 2, 2, 2, 1, 2, 1, 2, 1, 1, 1, 1, 1, 1, 1, 1, 1, 1, 1, 1, 1, 1, 1, 1, 1, 1, 1, 1, 1, 1, 1, 1, 1

Corresponding number of selected TFs each gene is involved with:
(In the same order as above)
6, 6, 6, 6, 5, 6, 6, 5, 5, 5, 6, 6, 6, 6, 6, 6, 3, 5, 5, 5, 4, 5, 4, 3, 6, 6, 6, 6, 3, 6, 4, 4, 5, 5, 5, 5, 3, 3, 3, 3, 6, 6, 6, 6, 3, 3, 4, 6, 6, 6, 4, 6, 6, 3, 2, 6, 6, 2, 2, 5, 5, 2, 5, 2, 2, 3, 3, 3, 4, 4, 2, 4, 4, 2, 2, 1, 3, 2, 6, 6, 3, 3, 2, 6, 6, 6, 6, 3, 6, 3, 3, 2, 6, 2, 3, 3, 2, 2, 6, 5, 5, 5, 5, 5, 1, 5, 1, 5, 4, 4, 4, 2, 4, 4, 4, 2, 4, 2, 2, 2, 1, 2, 4, 4, 4, 4, 1, 2, 3, 1, 3, 1, 3, 3, 3, 3, 3, 1, 3, 3, 3, 3, 1, 3, 3, 1, 3, 1, 3, 2, 2, 1, 1, 2, 2, 2, 2, 1, 1, 2, 1, 2, 2, 1, 1, 1, 2, 1, 1, 1, 1, 1, 1, 1, 2, 1, 2, 1, 1, 1, 1, 1, 1, 1, 1, 1, 1, 1, 1, 1, 1, 1, 1, 1, 1, 1, 1, 1, 1, 1, 1, 1

Gene ontology terms ranked according to mean of effect sizes in cluster:
T cell receptor complex | cell activation | immunological synapse | T cell activation | integrin activation | cell development | T cell differentiation

Corresponding mean of effect sizes of each Gene ontology term:
(In the same order as above)
2.59, 2.35, 2.34, 2.33, 2.19, 2.18, 2.10

Corresponding total number of genes of each Gene ontology term:
(In the same order as above)
478, 596, 112, 287, 155, 507, 172

Corresponding number of genes of each Gene ontology term with at least one TFBS (of selected TFs):
(In the same order as above)
97, 118, 30, 61, 30, 111, 48

Corresponding total number of TFBS (of selected TFs) for each Gene ontology term (in all genes):
(In the same order as above)
338, 374, 110, 223, 117, 359, 158

Corresponding number of selected TFs each Gene ontology term is involved with:
(In the same order as above)
6, 6, 6, 6, 6, 6, 6

TFs ranked according to mean of effect sizes in cluster:
VGR_Q6_01, VAR_Q6, VPR_Q2, VAML1_Q6, V$AML1_01, V$OSF2_Q6

Corresponding mean of effect sizes of each TF:
(In the same order as above)
2.97, 2.64, 2.41, 2.06, 1.93, 1.79

Corresponding total number of TFBS for each TF (genome-wide):
(In the same order as above)
720, 699, 698, 718, 710, 710

Corresponding total number of TFBS for each TF (in all genes in selected Gene ontology terms):
(In the same order as above)
304, 287, 288, 279, 273, 248

Corresponding number of genes (of selected Gene ontology terms) each TF is involved with:
(In the same order as above)
115, 107, 110, 114, 107, 94

Corresponding number of selected Gene ontology terms each TF is involved with:
(In the same order as above)
7, 7, 7, 7, 7, 7

Cluster for columns 17 to 56, rows 322 to 322

Gene ontology terms

small nuclear ribonucleoprotein complex | spliceosome | spliceosome assembly | mRNA processing | heterogeneous nuclear ribonucleoprotein complex | RNA binding | ribonucleoprotein complex | nuclear transport | RNA metabolic process | helicase activity | spindle | spindle assembly | kinetochore | anaphase | chromosome segregation | sister chromatid cohesion | telophase | meiosis | prophase | origin recognition complex | cell cycle arrest | cell cycle | regulation of cell cycle | cell division | mitosis | interphase | metaphase | cell cycle checkpoint | cellular response to DNA damage stimulus | DNA damage checkpoint | response to DNA damage stimulus | nuclear matrix | chromatin assembly | nucleosome assembly |

heterochromatin | chromatin remodeling | nucleosome | spermatid development | macronucleus | male pronucleus

TFs

V$NKX3A_01

Information

All related TFs:
(List of all TFs that are related to any of the PWMs)
Nkx3-1

Ranked gene list:
(All genes of the selected Gene ontology terms with hits of any of the selected TFs, ranked according to the total number of TFBS)
HIST1H4I, HIST1H4C, HIST2H2AA3, CDC2, PTTG1, CHEK2, CHEK1, BUB3, CENPC1, CENPE, CBX5, HNRPDL, CDK2, AURKA, MDM2, MCM2, TOP1, SKP1, SEPT7, DDX5, CDC27, TPX2, SMC4, SMARCA5, MAPRE2, HIST1H3E, HIST1H3C, H2AFZ, FBXW7, DBF4, CTNNB1, BCL2, SMARCA2, SKP2, SFRS11, RACGAP1, HIST1H1T, H3F3B, ERBB2IP, CLIP1, CKS1B, ATM, TP73, SLU7, PAFAH1B1, NFYA, LIG4, HNRNPA1, UBE2B, SSB, SNRPN, ORC1L, NPAT, NET1, HIST2H3C, UBE2K, TP63, STAG2, RUNX2, RUNX1, RPS6KA3, RAF1, PRPF4B, PIK3CA, HNRNPH1, FILIP1L, ETS1, CHD1, CDKN2C, CDK6, BMI1, BCL6, ANAPC10, TLK1, STRN3, SMC6, PIK3C3, NFKB1, NEK1, NEDD9, LSM4, LATS1, IL2, HNRNPU, HNRNPD, ENOPH1, CAMK4, TXNIP, TGFBR2, SMAD2, SAMD4A, REL, PTPRC, PRH1, PIK3R1, NOVA1, ING3, IKZF3, ID2, HNRNPR, HMGB2, HIF1A, HBP1, ELAVL4, DUT, DCLRE1A, CREM, CD69, CD44, BTG1, AHR, ZNF638, XRCC4, VCPIP1, UBE3A, TNFSF10, SUPT3H, SP3, SNRPG, SHC1, SERTAD2, PXMP3, PURA, PSMC2, PPP2CA, ORC4L, NIPBL, NFYC, MLL5, MBNL1, IFNG, HNRNPH3, HIST2H3A, FYN, FOXG1, EIF2S1, EHMT2, EFTUD2, DICER1, DHX15, DCP2, COLEC12, CHORDC1, CEP57, BIRC2, ARID4A, ADNP, ZWINT, ZNRD1, ZNF238, ZFX, ZFP36L2, YTHDC1, XPO7, TGFB2, TES, STK38L, SRPK2, SLC38A2, SILV, SETDB2, SERBP1, RREB1, RPL15, RBM45, PRKCH, PRKAR1A, PRDM2, PRDM1, POLR2B, PHIP, PHC3, PDE3A, PCF11, PAWR, NT5C3, NF1, NEUROG2, NDUFB6, NAT13, NARG1, MAPKSP1, MAP3K4, LSM6, KPNA4, KLRK1, KIAA0419, ITPR1, HLTF, HIST1H3H, HIST1H3G, HIST1H2BF, HIST1H2BB, HIST1H1D, HIST1H1B, HIPK3, HDAC7, GSK3B, GOLGA1, GBP2, GABPA, FIP1L1, ETV1, ENO1, EID1, EGR2, EED, DYRK1A, DNAJB9, DMTF1, DHX32, CYP51A1, CSTF3, CSTF1, CLDN1, CLCN3, CIT, CHD2, CEP170, CBFB, CA2, BTD, BCL9, ATP6V1A, ATF7IP, ARSG, ARID2, ANAPC4, AFF1, ACO1, AATF, AASDHPPT

Corresponding total number of TFBS:
(For each gene listed above, the total number of TFBS for any of the selected TFs, multiplied by the number of selected Gene ontology terms containing that gene)
29, 29, 26, 24, 18, 18, 18, 18, 17, 16, 16, 15, 15, 15, 13, 12, 11, 11, 11, 10, 10, 9, 9, 9, 9, 9, 9, 9, 9, 9, 9, 9, 8, 8, 8, 8, 8, 8, 8, 8, 8, 8, 7, 7, 7, 7, 7, 7, 6, 6, 6, 6, 6, 6, 6, 5, 5, 5, 5, 5, 5, 5, 5, 5, 5, 5, 5, 5, 5, 5, 5, 5, 4, 4, 4, 4, 4, 4, 4, 4, 4, 4, 4, 4, 4, 3, 3, 3, 3, 3, 3, 3, 3, 3, 3, 3, 3, 3, 3, 3, 3, 3, 3, 3, 3, 3, 3, 3, 3, 2, 2, 2, 2, 2, 2, 2, 2, 2, 2, 2, 2, 2, 2, 2, 2, 2, 2, 2, 2, 2, 2, 2, 2, 2, 2, 2, 2, 2, 2, 2, 2, 2, 1, 1, 1, 1, 1, 1, 1, 1, 1, 1, 1, 1, 1, 1, 1, 1, 1, 1, 1, 1, 1, 1, 1, 1, 1, 1, 1, 1, 1, 1, 1, 1, 1, 1, 1, 1, 1, 1, 1, 1, 1, 1, 1, 1, 1, 1, 1, 1, 1, 1, 1, 1, 1, 1, 1, 1, 1, 1, 1, 1, 1, 1, 1, 1, 1, 1, 1, 1, 1, 1, 1, 1, 1, 1, 1, 1, 1, 1

Corresponding number of selected Gene ontology terms each gene is involved with:
(In the same order as above)
29, 29, 26, 24, 18, 18, 18, 18, 17, 16, 16, 15, 15, 15, 13, 12, 11, 11, 11, 10, 10, 9, 9, 9, 9, 9, 9, 9, 9, 9, 9, 9, 8, 8, 8, 8, 8, 8, 8, 8, 8, 8, 7, 7, 7, 7, 7, 7, 6, 6, 6, 6, 6, 6, 6, 5, 5, 5, 5, 5, 5, 5, 5, 5, 5, 5, 5, 5, 5, 5, 5, 5, 4, 4, 4, 4, 4, 4, 4, 4, 4, 4, 4, 4, 4, 3, 3, 3, 3, 3, 3, 3, 3, 3, 3, 3, 3, 3, 3, 3, 3, 3, 3, 3, 3, 3, 3, 3, 3, 2, 2, 2, 2, 2, 2, 2, 2, 2, 2, 2, 2, 2, 2, 2, 2, 2, 2, 2, 2, 2, 2, 2, 2, 2, 2, 2, 2, 2, 2, 2, 2, 2, 1, 1, 1, 1, 1, 1, 1, 1, 1, 1,

1, 1, 1, 1, 1, 1, 1, 1, 1, 1, 1, 1, 1, 1, 1, 1, 1, 1, 1, 1, 1, 1, 1, 1, 1, 1, 1, 1, 1, 1, 1, 1, 1, 1, 1, 1, 1, 1, 1, 1, 1, 1, 1, 1, 1, 1, 1, 1, 1, 1, 1, 1, 1, 1, 1, 1, 1, 1, 1, 1, 1, 1, 1, 1, 1, 1, 1, 1, 1, 1, 1, 1, 1, 1

Corresponding number of selected TFs each gene is involved with:
(In the same order as above)
1, 1

Gene ontology terms ranked according to mean of effect sizes in cluster:
chromatin assembly | anaphase | heterochromatin | nucleosome assembly | metaphase | kinetochore | mRNA processing | chromosome segregation | small nuclear ribonucleoprotein complex | regulation of cell cycle | mitosis | origin recognition complex | cellular response to DNA damage stimulus | macronucleus | cell cycle checkpoint | prophase | interphase | telophase | male pronucleus | nucleosome | sister chromatid cohesion | spindle | nuclear transport | spermatid development | cell cycle | nuclear matrix | meiosis | chromatin remodeling | heterogeneous nuclear ribonucleoprotein complex | spliceosome | RNA binding | cell division | helicase activity | spindle assembly | RNA metabolic process | cell cycle arrest | DNA damage checkpoint | ribonucleoprotein complex | response to DNA damage stimulus | spliceosome assembly

Corresponding mean of effect sizes of each Gene ontology term:
(In the same order as above)
5.14, 4.86, 4.61, 4.52, 4.39, 4.10, 4.00, 3.98, 3.90, 3.52, 3.50, 3.37, 3.12, 3.05, 2.82, 2.79, 2.78, 2.77, 2.36, 2.30, 2.24, 2.23, 2.16, 2.09, 1.88, 1.75, 1.63, 1.48, 1.44, 1.34, 1.33, 1.31, 1.24, 1.24, 1.20, 1.07, 0.88, 0.29, 0.15, 0.09

Corresponding total number of genes of each Gene ontology term:
(In the same order as above)
101, 263, 196, 83, 295, 151, 109, 278, 197, 648, 635, 64, 111, 31, 171, 212, 382, 128, 21, 190, 93, 302, 185, 119, 1.3K, 197, 369, 410, 240, 119, 448, 451, 90, 161, 98, 628, 168, 339, 303, 73

Corresponding number of genes of each Gene ontology term with at least one TFBS (of selected TFs):
(In the same order as above)
21, 32, 29, 16, 36, 20, 14, 31, 23, 72, 61, 11, 15, 6, 21, 21, 37, 15, 5, 21, 10, 22, 17, 10, 102, 20, 26, 38, 21, 10, 31, 38, 7, 12, 7, 54, 13, 20, 21, 4

Corresponding total number of TFBS (of selected TFs) for each Gene ontology term (in all genes):
(In the same order as above)
21, 32, 29, 16, 36, 20, 14, 31, 23, 72, 61, 11, 15, 6, 21, 21, 37, 15, 5, 21, 10, 22, 17, 10, 102, 20, 26, 38, 21, 10, 31, 38, 7, 12, 7, 54, 13, 20, 21, 4

Corresponding number of selected TFs each Gene ontology term is involved with:
(In the same order as above)
1, 1, 1, 1, 1, 1, 1, 1, 1, 1, 1, 1, 1, 1, 1, 1, 1, 1, 1, 1, 1, 1, 1, 1, 1, 1, 1, 1, 1, 1, 1, 1, 1, 1, 1, 1, 1, 1, 1, 1

TFs ranked according to mean of effect sizes in cluster:
V$NKX3A_01

Corresponding mean of effect sizes of each TF:
(In the same order as above)
2.47

Corresponding total number of TFBS for each TF (genome-wide):
(In the same order as above)
674

Corresponding total number of TFBS for each TF (in all genes in selected Gene ontology terms):
(In the same order as above)
990

Corresponding number of genes (of selected Gene ontology terms) each TF is involved with:
(In the same order as above)
232

Corresponding number of selected Gene ontology terms each TF is involved with:
(In the same order as above)
40

Cluster for columns 1294 to 1295, rows 154 to 170

Gene ontology terms

signal transduction | Notch signaling pathway

TFs

V$SMAD_Q6 | V$AP2REP_01 | V$HNF4_Q6_03 | V$HNF4_Q6_02 | V$T3R_Q6 | V$PAX8_B | V$PAX8_01 | V$USF2_Q6 | V$ZIC2_01 | V$TTF1_Q6 | V$VDR_Q6 | V$LRF_Q2 | V$LBP1_Q6 | V$AP4_Q6_01 | V$AP4_Q6 | V$AP4_Q5 | V$LFA1_Q6

Information

All related TFs:
(List of all TFs that are related to any of the PWMs)
AP-2rep, AP-4, FBI-1, HNF-4, HNF-4alpha, HNF-4alpha1, HNF-4alpha2, HNF-4alpha3, HNF-4alpha4, LBP-1, LF-A1, LRF, Nkx2-1, OCZF, Pax-8, RAR-alpha, RAR-alpha1, RAR-alpha:RXR-alpha, RAR-alpha:RXR-gamma, RAR-beta, RAR-beta2, RAR-gamma, RXR-alpha, RXR-beta, RXR-beta2, RXR-gamma, Smad1, Smad1.1, Smad2, Smad2-L, Smad3, Smad3:Smad4, Smad4, T3R-alpha, T3R-alpha1, T3R-alpha2, T3R-beta, T3R-beta1, T3R-beta2, USF1:USF2, USF2, USF2a, VDR, ZIC2, Zic2

Ranked gene list:
(All genes of the selected Gene ontology terms with hits of any of the selected TFs, ranked according to the total number of TFBS)
LFNG, HES5, NRARP, NOTCH1, MFNG, TLE3, JAG2, HES7, HES3, DLL1, LHX1, NKX2-2, VEGFA, PTBP1, DLL4, C21orf33, ZFPM1, ZFP36, WNT10B, WNT1, VEGFB, TUSC2, TSPAN4, TSPAN32, TNK2, TNFRSF6B, TNFRSF4, TNFRSF18, TNFRSF12A, TNF, TCF7, TBC1D10C, STRA13, SSSCA1, SLC2A4RG, SKI, SIPA1, SH3BP2, SH3BP1, SH2B3, SEMA3B, SCRIB, SBF1, RPS6KB2, RORC, RGS19, RASSF2, RASGRP2, RARG, RARA, RAC3, PTPN7, PTPN6, PNPLA2, PLXNA1, PLCB3, PFN1, PER1, PDGFB, OVOL1, OPRL1, NR4A1, NPHP4, NPDC1, NFATC1, MGC111011, MEF2D, MDK, MAZ, MAPKAPK3, MAP3K12, MAP3K11, MAP2K3, MAF1, LTBP4, LTB, LSP1, KREMEN2, JUNB, ISYNA1, HRAS, GPX4, GNAI2, GIT1, GFI1, GATA3, FLNA, FLJ32987, FLII, FGFRL1, FGF8, FBRS, FASN, EFNA4, EFNA3, DUSP7, DUSP2, DGKZ, DGAT1, CXXC5, CSK, CISH, CFL1, CDK5, CD151, CBX4, BCL9L, ANAPC2, AKT1, AHNAK, ADORA2A, ABHD14B, TRADD, TAS1R3, TAGLN2, SMAD7, SIGIRR, S100A4, RIN1, PRKCD, PLK3, NFKBIE, MXD4, MCAM, KISS1R, IRF1, HDAC7, GATA2, DVL1, CNTNAP1, CLCF1, C19orf26, BCL11B, ARHGEF2, AMH, ACTB, WBP7,

UBE2I, RXRB, RASSF1, RALGDS, PPP1R9B, PPP1R1B, PLCG1, P2RY11, MC1R, MAPK12, MAPK11, IL17C, HS6ST1, GUK1, GRK6, GNAS, EHD1, E2F4, CTBP1, BBC3, APC2, ADRBK1, TLR9, SOCS3, PTP4A3, NXPH4, LLGL2, HOXA10, FOXC1, CCND2, BCL6, ADM, WNT10A, SYNGAP1, SOCS1, SLC9A3R1, SLC12A7, SBNO2, LTA, GPS2, EMD, EGR1, CAMK2G, p63RhoGEF, TBX2, STMN3, SQSTM1, RGS14, PYGO2, PSD, PAX6, PARD6A, OSM, GAPDH, EFNA2, EFNA1, DVL2, CCND1, ARVCF, ZAP70, S1PR4, RING1, RHOC, NFKB2, LBX1, HOXA11, FLI1, CYBA, ARHGEF1, WNT11, VAMP2, RFNG, PTK6, PSENEN, POU2F2, GLI1, FHOD1, FGFR3, DGKQ, DGKA, CDKN1C, BHLHB2, BCL2L11, ATP2A3, AGAP2, SH2B2, RPS6KA1, PDE4A, MXD3, MRPL28, LAG3, ITGB2, FGF22, CDK4, CDC34, AQP3, AKT1S1, AGER, TNFRSF25, NXPH3, NR4A2, MAP2K7, LEF1, LASP1, GARNL4, FBXL15, CORO1A, CDK9, ARHGDIA, ZYX, UCN2, TENC1, RPS6KA4, PTGDS, PKN1, NR3C2, MAL, LCK, IKBKE, FOXO3, FGF9, ESRRA, DLX2, DLG4, DCTN3, CRTC2, COL11A2, CD82, CALY, CALR, BMF, BAD, ARRB2, ADCY7, TRAF4, TNIP1, TGFB1, PDCD1, MAP3K14, LTBP3, JUND, ITGA5, IRS1, INPP5D, IL11RA, ID3, HES1, EOMES, DHX58, DBNL, CRLF1, CDC20, ZIC2, SLC9A3R2, S100A3, RAD9A, NR4A3, NKD2, NDOR1, MXI1, KLF2, HCST, GPSM1, GPR132, GGNBP2, ENTPD2, DDR1, DDIT3, CDKN2C, CD248, ASB2, AGRN, VIPR1, VGF, VASP, TSC2, TRIP10, TNFRSF1B, TNFRSF14, TM7SF2, TLX2, STAT5A, SEMA3G, RUNX3, PTCH1, PRKACA, NPPC, NFKBID, NAB2, MGC4238, LEFTY1, IFITM1, ID2, HOXB4, HCRT, GSK3A, FOSB, FERMT3, EPHB3, DUSP1, CDK6, CD79B, CCR7, BRD2, BAK1, ARF1, ANP32A, UCP2, TRIM28, TPP1, TNFRSF13C, SLC12A4, RGL2, PTH1R, PMEPA1, PHB2, PAX5, NPW, NEUROG2, MTA2, MST1, MAPK15, MAPK13, LPAR2, LAT, LAMA5, KLF6, ITPR3, IL11, ICAM4, GPI, GPC1, FSTL3, ERF, ELMO3, E2F2, DHCR7, CTF1, CMIP, CDK5R1, CDC25B, CD81, CD7, WBP2, TYROBP, TNFSF13, TNFRSF1A, SNX20, SMAD2, SLC29A1, SLC17A7, SAMD4A, RRAS, RGS11, RGS10, RAP1GAP, PRMT1, PIN1, PIK3R2, PDLIM7, PDCD6, PCNA, NR3C1, NOTCH4, NOL3, NISCH, NFATC3, MUC1, MTA1, MICAL1, MGRN1, LTB4R2, LNX2, LIMD1, KIAA0319L, KCNH4, ISG15, IRF7, INS, ING1, IDUA, HGFAC, HES6, GALR3, FOXJ1, FLOT2, DLX4, DAAM1, CXCR4, CNP, CERK, CDKN1B, CDC37, CD79A, CD4, CARD9, CAMK1D, BID, AXIN1, ARC, ADCY4, ZHX2, ZFP36L1, ZBTB16, WNT7B, WNT7A, WNT4, VAV1, TRRAP, TRIM3, TRERF1, TRAP1, TRAF2, TGM1, TGIF2, TFDP1, TFAP2A, TCF3, TAOK3, STMN1, STK25, STAT6, STARD3, SREBF1, SRA1, SPHK1, SORL1, SORBS3, SNF1LK, SKP1, SHC1, SH3RF3, S100A13, ROBLD3, RHOG, RAD23A, PVRL1, PTPRA, PTGER1, PTCHD2, PSMB9, PRRG2, PRKCB, PPP3CA, PPARA, PLEKHO2, NUDT1, NRGN, NR2F6, NR2F1, NPM1, NOG, NKX3-2, NGFR, NDRG1, MYD88, MT2A, MIF, MARCKSL1, MAP4K2, MAP3K6, MAP2K2, MAD1L1, LMNA, LIF, LGALS1, KLF10, KCNA2, IRF5, IL32, IL21R, IL10RA, IKZF1, IGF2, IGF1R, HOXC6, HOXC4, HIVEP1, HES4, HDAC5, GRM2, GRIN1, GRB7, GRASP, GLTSCR2, GIT2, GEMIN4, GADD45B, FRS3, FOSL2, FBXW7, EPB41, EGR4, EGR2, ECE1, DUSP6, DTX1, DPYSL2, DOK2, DIABLO, CUX1, CRABP2, CPLX1, CPEB1, CLEC11A, CDKN2D, CDC25C, CD63, CD3G, CAMKK2, ATF4, ARRB1, ARNTL, AQP5, AGTRAP, ACTN1, ACAA1, ABI3

Corresponding total number of TFBS:
(For each gene listed above, the total number of TFBS for any of the selected TFs, multiplied by the number of selected Gene ontology terms containing that gene)
34, 34, 32, 32, 32, 28, 28, 28, 28, 28, 26, 24, 20, 20, 20, 20, 17, 17, 17, 17, 17, 17, 17, 17, 17, 17, 17, 17, 17, 17, 17, 17, 17, 17, 17, 17, 17, 17, 17, 17, 17, 17, 17, 17, 17, 17, 17, 17, 17, 17, 17, 17, 17, 17, 17, 17, 17, 17, 17, 17, 17, 17, 17, 17, 17, 17, 17, 17, 17, 17, 17, 17, 17, 17, 17, 17, 17, 17, 17, 17, 17, 17, 17, 17, 17, 17, 17, 17, 17, 17, 17, 17, 17, 17, 17, 17, 17, 17, 17, 17, 17, 17, 17, 17, 17, 17, 17, 17, 17, 17, 17, 17, 16, 16, 16, 16, 16, 16, 16, 16, 16, 16, 16, 16, 16, 16, 16, 16, 16, 16, 16, 16, 16, 16, 16, 16, 15, 15, 15, 15, 15, 15, 15, 15, 15, 15, 15, 15, 15, 15, 15, 15, 15, 15, 15, 15, 15, 15, 15, 14, 14, 14, 14, 14, 14, 14, 14, 14, 14, 13, 13, 13, 13, 13, 13, 13, 13, 13, 13, 13, 12, 12, 12, 12, 12, 12, 12, 12, 12, 12, 12, 12, 12, 12, 12, 12, 11, 11, 11, 11, 11, 11, 11, 11, 11, 11, 10, 10, 10, 10, 10, 10, 10, 10, 10, 10, 10, 10, 10, 10, 10, 10, 9, 9, 9, 9, 9, 9, 9, 9, 9, 9, 9, 9, 9, 8, 8, 8, 8, 8, 8, 8, 8, 8, 8, 8, 7, 7, 7, 7, 7, 7, 7, 7, 7, 7, 7, 7, 7, 7, 7, 7, 7, 7, 7, 7, 7, 7, 7, 7, 7, 6, 6, 6, 6, 6, 6, 6, 6, 6, 6, 6, 6, 6, 6, 6, 6, 6, 6, 5, 5, 5, 5, 5, 5, 5, 5, 5, 5, 5, 5, 5, 5, 5, 5, 5, 5, 5, 5, 4, 4, 4, 4, 4, 4, 4, 4, 4, 4, 4, 4, 4, 4, 4, 4, 4, 4, 4, 4, 4, 4, 4, 4, 4, 4, 4, 4, 4, 4, 4, 4, 4, 4, 4, 3, 3, 3, 3, 3, 3, 3, 3, 3, 3, 3, 3, 3, 3, 3, 3, 3, 3, 3, 3, 3, 3, 3, 3, 3, 3, 3, 3, 3, 3, 3, 3, 3, 3, 3, 3, 2, 2, 2, 2, 2, 2, 2, 2, 2, 2, 2, 2, 2, 2, 2, 2, 2, 2, 2, 2, 2, 2, 2, 2, 2, 2, 2, 2, 2, 2, 2, 2, 2, 2, 2, 2, 2, 2, 2, 2, 2, 2, 2, 2, 2, 2, 2, 2, 2, 2, 2, 2, 2, 2, 2, 2, 2, 2, 1, 1, 1, 1, 1, 1, 1, 1, 1, 1, 1, 1, 1, 1, 1, 1, 1, 1, 1, 1, 1, 1, 1, 1, 1, 1, 1, 1, 1, 1, 1, 1, 1, 1, 1, 1, 1, 1, 1, 1, 1, 1, 1, 1, 1, 1, 1, 1, 1, 1, 1, 1, 1, 1, 1, 1, 1, 1, 1, 1, 1, 1, 1, 1, 1, 1, 1, 1, 1, 1, 1, 1, 1, 1, 1, 1, 1, 1, 1, 1, 1, 1,

1, 1, 1, 1, 1, 1, 1, 1, 1, 1, 1, 1, 1, 1, 1, 1, 1, 1, 1, 1, 1, 1, 1, 1, 1, 1, 1, 1, 1, 1, 1, 1, 1, 1, 1

Corresponding number of selected Gene ontology terms each gene is involved with:
(In the same order as above)
2, 2, 2, 2, 2, 2, 2, 2, 2, 2, 2, 2, 2, 2, 2, 2, 1, 1, 1, 1, 1, 1, 1, 1, 1, 1, 1, 1, 1, 1, 1, 1, 1, 1, 1, 1, 1, 1, 1, 1, 1, 1,
1, 1, 1, 1, 1, 1, 1, 1, 1, 1, 1, 1, 1, 1, 1, 1, 1, 1, 1, 1, 1, 1, 1, 1, 1, 1, 1, 1, 1, 1, 1, 1, 1, 1, 1, 1, 1, 1, 1, 1, 1, 1,
1, 1, 1, 1, 1, 1, 1, 1, 1, 1, 1, 1, 1, 1, 1, 1, 1, 1, 1, 1, 1, 1, 1, 1, 1, 1, 1, 1, 1, 1, 1, 1, 1, 1, 1, 1, 1, 1, 1, 1, 1, 1,
1, 1, 1, 1, 1, 1, 1, 1, 1, 1, 1, 1, 1, 1, 1, 1, 1, 1, 1, 1, 1, 1, 1, 1, 1, 1, 1, 1, 1, 1, 1, 1, 1, 1, 1, 1, 1, 1, 1, 2, 1, 1,
1, 1, 1, 1, 1, 1, 1, 1, 1, 1, 1, 1, 1, 1, 1, 1, 1, 1, 1, 1, 1, 1, 1, 1, 1, 1, 2, 1, 1, 1, 1, 1, 1, 1, 1, 1, 1, 1, 1, 1, 2, 1,
1, 1, 1, 1, 1, 1, 1, 1, 1, 1, 1, 1, 1, 1, 1, 1, 1, 1, 1, 1, 1, 1, 1, 1, 1, 1, 1, 1, 1, 1, 1, 1, 1, 1, 1, 1, 1, 1, 1, 1, 1, 1,
1, 1, 1, 1, 1, 1, 1, 1, 1, 1, 1, 1, 1, 1, 1, 1, 1, 1, 1, 1, 1, 1, 1, 1, 1, 1, 1, 1, 1, 1, 1, 2, 1, 1, 1, 1, 1, 1, 1, 1, 1, 1,
1, 1, 1, 1, 1, 1, 1, 1, 1, 1, 1, 1, 1, 1, 1, 1, 1, 1, 1, 1, 1, 1, 1, 1, 1, 1, 1, 1, 1, 1, 1, 1, 1, 1, 1, 1, 1, 1, 1, 1, 1, 1,
1, 1, 1, 1, 1, 1, 1, 1, 1, 1, 1, 1, 1, 1, 1, 1, 1, 1, 1, 1, 1, 1, 1, 1, 1, 1, 1, 1, 1, 1, 1, 1, 1, 1, 1, 1, 1, 1, 1, 1, 1, 1,
1, 1, 1, 1, 2, 1, 1, 1, 1, 1, 1, 1, 1, 1, 1, 1, 1, 1, 1, 1, 1, 1, 2, 1, 1, 1, 1, 1, 1, 1, 1, 1, 1, 1, 1, 1, 1, 1, 1, 1, 1, 2,
1, 1, 1, 1, 1, 1, 1, 1, 1, 1, 1, 1, 1, 1, 1, 1, 1, 1, 1, 1, 1, 1, 1, 1, 1, 1, 1, 1, 1, 1, 1, 1, 1, 1, 1, 1, 1, 1, 1, 1, 1, 1,
1, 1, 1, 1, 1, 1, 1, 1, 1, 1, 1, 1, 1, 1, 1, 1, 1, 1, 1, 1, 1, 1, 1, 1, 1, 1, 1, 1, 1, 1, 1, 1, 1, 1, 1, 1, 1, 1, 1, 1, 1, 1,
1, 1, 1, 1, 1, 1, 1, 1, 1, 1, 1, 1, 1, 1, 1, 1, 1, 1, 1, 1, 1, 1, 1, 1, 1, 1, 1, 1, 1, 1, 1, 1, 1, 1, 1, 1, 1, 1, 1, 1, 1, 1,
1, 1, 1, 1, 1, 1, 1, 1, 1

Corresponding number of selected TFs each gene is involved with:
(In the same order as above)
17, 17, 16, 16, 16, 14, 14, 14, 14, 14, 13, 12, 10, 10, 10, 10, 17, 17, 17, 17, 17, 17, 17, 17, 17, 17, 17, 17,
17, 17, 17, 17, 17, 17, 17, 17, 17, 17, 17, 17, 17, 17, 17, 17, 17, 17, 17, 17, 17, 17, 17, 17, 17, 17, 17, 17,
17, 17, 17, 17, 17, 17, 17, 17, 17, 17, 17, 17, 17, 17, 17, 17, 17, 17, 17, 17, 17, 17, 17, 17, 17, 17, 17, 17,
17, 17, 17, 17, 17, 17, 17, 17, 17, 17, 17, 17, 17, 17, 17, 17, 17, 17, 17, 17, 17, 17, 17, 17, 17, 17, 17, 17,
16, 16, 16, 16, 16, 16, 16, 16, 16, 16, 16, 16, 16, 16, 16, 16, 16, 16, 16, 16, 16, 16, 16, 16, 15, 15, 15, 15,
15, 15, 15, 15, 15, 15, 15, 15, 15, 15, 15, 15, 15, 15, 15, 15, 15, 15, 15, 14, 14, 14, 14, 14, 14, 7, 14, 14,
14, 13, 13, 13, 13, 13, 13, 13, 13, 13, 13, 13, 12, 12, 12, 12, 12, 12, 12, 12, 12, 12, 12, 12, 12, 12, 6, 12,
11, 11, 11, 11, 11, 11, 11, 11, 11, 11, 10, 10, 5, 10, 10, 10, 10, 10, 10, 10, 10, 10, 10, 10, 10, 10, 9, 9, 9, 9,
9, 9, 9, 9, 9, 9, 9, 9, 9, 8, 8, 8, 8, 8, 8, 8, 8, 8, 8, 8, 7, 7, 7, 7, 7, 7, 7, 7, 7, 7, 7, 7, 7, 7, 7, 7, 7, 7, 7, 7, 7, 7,
7, 7, 7, 6, 6, 6, 6, 6, 6, 6, 6, 6, 6, 6, 6, 3, 6, 6, 6, 6, 6, 5, 5, 5, 5, 5, 5, 5, 5, 5, 5, 5, 5, 5, 5, 5, 5, 5, 5, 5, 5, 4,
4, 4, 4, 4, 4, 4, 4, 4, 4, 4, 4, 4, 4, 4, 4, 4, 4, 4, 4, 4, 4, 4, 4, 4, 4, 4, 4, 4, 4, 4, 4, 4, 4, 4, 3, 3, 3, 3, 3, 3, 3, 3,
3, 3, 3, 3, 3, 3, 3, 3, 3, 3, 3, 3, 3, 3, 3, 3, 3, 3, 3, 3, 3, 3, 3, 3, 3, 3, 3, 3, 2, 2, 1, 2, 2, 2, 2, 2, 2, 2, 2, 2, 2, 2,
2, 2, 2, 2, 2, 2, 1, 2, 2, 2, 2, 2, 2, 2, 2, 2, 2, 2, 2, 2, 2, 2, 2, 2, 2, 2, 1, 2, 2, 2, 2, 2, 2, 2, 2, 2, 2, 2, 2, 2, 2, 2,
2, 2, 1, 1, 1, 1, 1, 1, 1, 1, 1, 1, 1, 1, 1, 1, 1, 1, 1, 1, 1, 1, 1, 1, 1, 1, 1, 1, 1, 1, 1, 1, 1, 1, 1, 1, 1, 1, 1, 1, 1, 1,
1, 1, 1, 1, 1, 1, 1, 1, 1, 1, 1, 1, 1, 1, 1, 1, 1, 1, 1, 1, 1, 1, 1, 1, 1, 1, 1, 1, 1, 1, 1, 1, 1, 1, 1, 1, 1, 1, 1, 1, 1, 1,
1, 1, 1, 1, 1, 1, 1, 1, 1, 1, 1, 1, 1, 1, 1, 1, 1, 1, 1, 1, 1, 1, 1, 1, 1, 1, 1, 1, 1, 1, 1, 1, 1, 1, 1

Gene ontology terms ranked according to mean of effect sizes in cluster:
Notch signaling pathway | signal transduction

Corresponding mean of effect sizes of each Gene ontology term:
(In the same order as above)
2.49, 1.92

Corresponding total number of genes of each Gene ontology term:
(In the same order as above)
103, 3.9K

Corresponding number of genes of each Gene ontology term with at least one TFBS (of selected TFs):
(In the same order as above)
28, 550

Corresponding total number of TFBS (of selected TFs) for each Gene ontology term (in all genes):
(In the same order as above)

284, 4325

Corresponding number of selected TFs each Gene ontology term is involved with:
(In the same order as above)
17, 17

TFs ranked according to mean of effect sizes in cluster:
V$AP4_Q6_01, V$LRF_Q2, V$LBP1_Q6, V$AP4_Q5, V$USF2_Q6, V$VDR_Q6, V$SMAD_Q6, V$AP4_Q6, V$AP2REP_01, V$PAX8_01, V$HNF4_Q6_03, V$LFA1_Q6, V$ZIC2_01, V$PAX8_B, V$T3R_Q6, V$TTF1_Q6, V$HNF4_Q6_02

Corresponding mean of effect sizes of each TF:
(In the same order as above)
3.01, 3.00, 2.86, 2.75, 2.71, 2.63, 2.33, 2.20, 2.17, 2.09, 1.98, 1.94, 1.74, 1.63, 1.60, 1.51, 1.36

Corresponding total number of TFBS for each TF (genome-wide):
(In the same order as above)
699, 719, 691, 702, 703, 692, 729, 692, 700, 728, 701, 711, 716, 712, 698, 714, 699

Corresponding total number of TFBS for each TF (in all genes in selected Gene ontology terms):
(In the same order as above)
278, 289, 275, 266, 279, 261, 278, 258, 280, 285, 267, 263, 275, 268, 265, 254, 268

Corresponding number of genes (of selected Gene ontology terms) each TF is involved with:
(In the same order as above)
262, 273, 258, 251, 258, 245, 265, 245, 265, 268, 255, 253, 261, 254, 254, 243, 258

Corresponding number of selected Gene ontology terms each TF is involved with:
(In the same order as above)
2, 2, 2, 2, 2, 2, 2, 2, 2, 2, 2, 2, 2, 2, 2, 2, 2

Cluster for columns 1054 to 1073, rows 267 to 293

Gene ontology terms

axon guidance | nervous system development | synaptic cleft | terminal button | cognition | toxin binding | choline O-acetyltransferase activity | neurotransmitter biosynthetic process | basal lamina | sarcoplasm | response to ATP | response to histamine | serotonin receptor activity | interchromatin granule | acetylcholinesterase activity | choline transport | angiotensin-converting enzyme inhibitor activity | saliva secretion | antigenic variation | serotonin biosynthetic process

TFs

V$ETF_Q6 | V$E2F_Q2 | V$E2F1_Q6 | V$E2F1_Q3 | V$AP2GAMMA_01 | V$AP2ALPHA_01 | V$AP2_Q6_01 | V$AP2_Q6 | V$WT1_Q6 | V$EGR_Q6 | V$MAZ_Q6 | V$MAZR_01 | V$E2F1_Q3_01 | V$HES1_Q2 | V$ACAAT_B | V$AP2_Q3 | V$AP2ALPHA_02 | V$HIC1_03 | V$HIC1_02 | V$PAX5_01 | V$ZNF219_01 | V$SP1_Q2_01 | V$SP1_01 | V$SP1_Q6 | V$SP1_Q4_01 | V$SP1_Q6_01 | V$KROX_Q6

Information

All related TFs:
(List of all TFs that are related to any of the PWMs)

AP-2, AP-2alpha, AP-2alphaA, AP-2alphaB, AP-2beta, AP-2gamma, DP-1, E2F, E2F+E4, E2F-1, E2F-3a, E2F-4, ETF, Egr-1, Egr-2, Egr-3, Egr-4, HES-1, HIC-1, MAZ, MAZR, Pax-5, Sp1, Sp2, Sp3, Sp4, WT1, WT1-del2, ZNF219

Ranked gene list:
(All genes of the selected Gene ontology terms with hits of any of the selected TFs, ranked according to the total number of TFBS)
ACHE, FGFR3, AGRN, AKT1, PLXNA1, CDK5, SLC25A1, GPT, NKX2-2, THRA, PER1, ENTPD2, ACTG1, RHOF, PAX6, NTN3, SLC2A4RG, PLEC1, GIT1, PGP, LBX1, VEGFA, SEMA3B, MDK, EFNA3, DVL1, NOC2L, FLNA, NGFR, HBA2, SLC4A2, PPP1R9B, IGF1R, GPC1, DBP, NR3C2, KCNH2, PDE4A, NEO1, JUND, ING2, HES1, GNAI2, GFI1, ENO3, CDT1, BRD1, TNFSF13, TMEM121, SH2D3C, SH2B2, NOTCH1, FLJ32987, WBP7, SCRIB, EGR3, TIAM1, EFNA2, PSD, SOX1, PXN, NR3C1, LHX3, GALNS, FXYD1, EPHA1, SLC30A3, NTN1, EFNB2, EFNA1, CTF1, VASP, PRKCE, POU2F1, PLXND1, NPTX1, LMX1B, IGF2, CASP9, RHOG, RARA, PRKCZ, NFIB, NFIA, EGR1, CDH15, ARHGEF2, ZMYM2, VIM, VDAC1, SLC17A7, SEMA3F, RHOA, NFIX, MUC1, MGAT1, HNRPDL, EPB49, DPYSL2, DPAGT1, DAG1, B3GAT1, ARSA, WNT4, PITRM1, P2RY11, KCNJ11, FBXL15, EMX1, DLG4, BDNF, TFAP2A, SLC7A1, SLC12A2, SHH, RABEP2, PRKCA, PPP1R14A, NT5C2, MICAL1, LIMK1, HRH3, CRY1, CDH2, ARHGEF11, UNC5A, TPM1, SMURF1, SCARB1, PXMP2, PLXNA3, PARD6A, NTF4, NCAN, MAPT, MAP1B, LRP5, LHX4, FOSL1, FGF2, DMPK, DBN1, ACCN2, SYN1, STX1A, SRC, SNAP25, SLC17A5, SFRS2, SEMA7A, SDC3, RPE, RGMA, PTPRF, PSEN1, PPFIA1, PNMT, PLXNA4, NTNG2, NRP2, NRP1, NOTCH3, NGEF, NF2, MCF2L, LMNA, L1CAM, KCNB1, HTR6, HBA1, GALR3, FYN, EPHB2, EPHA4, ENAH, EIF3B, EFNA5, DPYSL5, DPYSL3, DNER, DLX3, CYLD, CRAT, COMP, AMT, WNT5A, WNT3, VPS13A, VCL, UAP1, TRPC1, TIMP2, STX2, STX1B, ST3GAL4, SNAI1, SLIT3, SLC7A10, SLC6A9, SLC32A1, SEMA4G, SDHB, S100A6, RYK, RPS15A, PTK2, PSMD1, POMGNT1, PNPLA6, PMP22, PLXNB1, P2RX5, ODC1, NTRK3, NOTCH4, NEFM, NCK1, MSH3, MMP14, MARCKS, MAPK8IP3, MAPK1, MAP1A, LHX2, LAMA5, KIT, JUN, ITGB4, ITGB1, IQGAP1, HTT, GAA, FOS, EZR, ERBB4, EPHB4, ENO2, ELK3, E2F5, DRD4, CSE1L, CRMP1, CNTFR, CNP, CENPV, CELSR2, CELSR1, ATP7B, ARL6IP5, ARID4B, ARHGEF12, AQP3, ANXA7, AGFG1, ADA, ACE, ABL1

Corresponding total number of TFBS:
(For each gene listed above, the total number of TFBS for any of the selected TFs, multiplied by the number of selected Gene ontology terms containing that gene)
252, 238, 95, 66, 42, 40, 34, 32, 30, 28, 28, 28, 27, 26, 26, 26, 25, 25, 25, 24, 24, 22, 22, 22, 22, 22, 21, 21, 20, 20, 19, 18, 18, 18, 18, 17, 17, 16, 16, 16, 16, 16, 16, 16, 16, 16, 15, 15, 15, 15, 15, 15, 14, 14, 14, 12, 12, 11, 10, 10, 10, 10, 10, 10, 10, 9, 9, 9, 9, 9, 8, 8, 8, 8, 8, 8, 8, 8, 7, 7, 7, 7, 7, 7, 7, 7, 6, 6, 6, 6, 6, 6, 6, 6, 6, 6, 6, 6, 6, 6, 6, 6, 5, 5, 5, 5, 5, 5, 5, 5, 4, 4, 4, 4, 4, 4, 4, 4, 4, 4, 4, 4, 4, 4, 3, 3, 3, 3, 3, 3, 3, 3, 3, 3, 3, 3, 3, 3, 3, 3, 3, 3, 2, 2, 2, 2, 2, 2, 2, 2, 2, 2, 2, 2, 2, 2, 2, 2, 2, 2, 2, 2, 2, 2, 2, 2, 2, 2, 2, 2, 2, 2, 2, 2, 2, 2, 2, 2, 2, 2, 2, 1, 1, 1, 1, 1, 1, 1, 1, 1, 1, 1, 1, 1, 1, 1, 1, 1, 1, 1, 1, 1, 1, 1, 1, 1, 1, 1, 1, 1, 1, 1, 1, 1, 1, 1, 1, 1, 1, 1, 1, 1, 1, 1, 1, 1, 1, 1, 1, 1, 1, 1, 1, 1, 1, 1, 1, 1, 1, 1, 1, 1, 1, 1, 1, 1, 1, 1, 1, 1

Corresponding number of selected Gene ontology terms each gene is involved with:
(In the same order as above)
12, 14, 5, 3, 2, 2, 2, 2, 2, 2, 2, 2, 1, 1, 2, 1, 1, 1, 1, 1, 2, 2, 2, 1, 1, 1, 1, 1, 4, 1, 1, 2, 1, 1, 1, 1, 1, 1, 2, 1, 1, 1, 1, 1, 1, 1, 2, 5, 1, 1, 1, 1, 1, 1, 1, 1, 1, 1, 1, 1, 1, 2, 1, 2, 2, 1, 2, 1, 3, 1, 1, 1, 1, 1, 1, 1, 1, 1, 1, 1, 1, 1, 1, 1, 1, 1, 1, 1, 1, 1, 1, 2, 1, 2, 1, 2, 1, 1, 1, 1, 1, 2, 2, 2, 3, 1, 1, 1, 1, 1, 1, 1, 1, 1, 5, 1, 1, 1, 2, 1, 2, 1, 4, 2, 2, 2, 1, 4, 2, 1, 1, 1, 1, 1, 1, 1, 3, 3, 1, 3, 1, 1, 1, 3, 1, 1, 1, 2, 1, 2, 2, 2, 1, 1, 1, 2, 2, 1, 2, 1, 2, 1, 1, 1, 2, 1, 2, 2, 1, 1, 2, 1, 2, 1, 1, 1, 2, 1, 2, 1, 1, 1, 2, 1, 1, 1, 2, 2, 1, 1, 1, 1, 1, 1, 1, 1, 1, 1, 1, 1, 1, 1, 1, 1, 1, 1, 1, 1, 1, 1, 1, 1, 1, 1, 1, 1, 1, 1, 1, 1, 1, 1, 1, 1, 1, 1, 1, 1, 1, 1, 1, 1, 1, 1, 1, 1, 1, 1, 1, 1, 1, 1, 1, 1, 1, 1, 1, 1, 1, 1, 1, 1, 1, 1, 1, 1, 1, 1, 1, 1, 1, 1, 1, 1, 1, 1

Corresponding number of selected TFs each gene is involved with:
(In the same order as above)
21, 17, 19, 22, 21, 20, 17, 16, 15, 14, 14, 14, 27, 26, 13, 26, 25, 25, 25, 24, 12, 11, 11, 22, 22, 22, 21, 21, 5, 20, 19, 9, 18, 18, 18, 17, 17, 16, 8, 16, 16, 16, 16, 16, 16, 16, 8, 3, 15, 15, 15, 15, 15, 14, 14, 14, 12, 12, 11, 10, 5, 10, 5, 5, 10, 5, 9, 3, 9, 9, 9, 8, 8, 8, 8, 8, 8, 8, 8, 7, 7, 7, 7, 7, 7, 7, 7, 6, 3, 6, 3, 6, 3, 6, 6, 6, 6, 6,

3, 3, 3, 2, 6, 5, 5, 5, 5, 5, 5, 5, 1, 4, 4, 4, 2, 4, 2, 4, 1, 2, 2, 2, 4, 1, 2, 3, 3, 3, 3, 3, 3, 3, 1, 1, 3, 1, 3, 3, 3, 1, 3, 3, 3, 1, 2, 1, 1, 1, 2, 2, 2, 1, 1, 2, 1, 2, 1, 2, 2, 2, 1, 2, 1, 1, 2, 2, 1, 2, 1, 2, 2, 2, 1, 2, 1, 2, 2, 2, 1, 2, 2, 2, 1, 1, 2, 1, 1, 1, 1, 1, 1, 1, 1, 1, 1, 1, 1, 1, 1, 1, 1, 1, 1, 1, 1, 1, 1, 1, 1, 1, 1, 1, 1, 1, 1, 1, 1, 1, 1, 1, 1, 1, 1, 1, 1, 1, 1, 1, 1, 1, 1, 1, 1, 1, 1, 1, 1, 1, 1, 1, 1, 1, 1, 1, 1, 1, 1, 1, 1, 1, 1, 1, 1, 1, 1, 1, 1, 1, 1, 1, 1, 1, 1

Gene ontology terms ranked according to mean of effect sizes in cluster:
axon guidance | sarcoplasm | nervous system development | interchromatin granule | choline transport | response to histamine | neurotransmitter biosynthetic process | toxin binding | synaptic cleft | saliva secretion | terminal button | angiotensin-converting enzyme inhibitor activity | response to ATP | antigenic variation | basal lamina | serotonin biosynthetic process | cognition | choline O-acetyltransferase activity | acetylcholinesterase activity | serotonin receptor activity

Corresponding mean of effect sizes of each Gene ontology term:
(In the same order as above)
1.77, 1.56, 1.44, 1.18, 1.11, 1.06, 1.04, 1.03, 1.00, 0.98, 0.76, 0.72, 0.66, 0.66, 0.61, 0.60, 0.56, 0.53, 0.51, 0.39

Corresponding total number of genes of each Gene ontology term:
(In the same order as above)
187, 24, 185, 35, 26, 37, 27, 33, 66, 27, 21, 63, 47, 37, 112, 34, 69, 37, 27, 49

Corresponding number of genes of each Gene ontology term with at least one TFBS (of selected TFs):
(In the same order as above)
82, 9, 78, 11, 5, 8, 9, 9, 21, 10, 5, 17, 10, 8, 32, 8, 18, 11, 5, 9

Corresponding total number of TFBS (of selected TFs) for each Gene ontology term (in all genes):
(In the same order as above)
450, 92, 462, 92, 65, 66, 74, 68, 122, 90, 36, 137, 70, 75, 169, 60, 110, 53, 58, 73

Corresponding number of selected TFs each Gene ontology term is involved with:
(In the same order as above)
27, 27, 27, 27, 27, 26, 27, 26, 27, 27, 21, 27, 27, 27, 27, 26, 27, 25, 27, 26

TFs ranked according to mean of effect sizes in cluster:
V$WT1_Q6, V$E2F_Q2, V$SP1_01, V$AP2ALPHA_01, V$AP2GAMMA_01, V$EGR_Q6, V$E2F1_Q3, V$AP2_Q6_01, V$HES1_Q2, V$ETF_Q6, V$AP2_Q6, V$SP1_Q6_01, V$KROX_Q6, V$HIC1_02, V$SP1_Q2_01, V$PAX5_01, V$E2F1_Q3_01, V$MAZ_Q6, V$SP1_Q4_01, V$SP1_Q6, V$AP2_Q3, V$ACAAT_B, V$AP2ALPHA_02, V$MAZR_01, V$HIC1_03, V$ZNF219_01, V$E2F1_Q6

Corresponding mean of effect sizes of each TF:
(In the same order as above)
1.44, 1.40, 1.39, 1.36, 1.31, 1.31, 1.30, 1.24, 1.23, 1.21, 1.17, 1.03, 0.95, 0.91, 0.85, 0.82, 0.80, 0.77, 0.75, 0.65, 0.62, 0.51, 0.48, 0.39, 0.31, 0.23, 0.09

Corresponding total number of TFBS for each TF (genome-wide):
(In the same order as above)
729, 714, 696, 686, 695, 695, 709, 690, 690, 699, 688, 663, 675, 664, 695, 671, 680, 734, 656, 664, 683, 692, 663, 705, 660, 689, 691

Corresponding total number of TFBS for each TF (in all genes in selected Gene ontology terms):
(In the same order as above)
121, 110, 99, 102, 102, 109, 101, 108, 104, 103, 103, 78, 90, 90, 85, 75, 90, 92, 70, 69, 81, 88, 67, 76, 67, 70, 72

Corresponding number of genes (of selected Gene ontology terms) each TF is involved with:
(In the same order as above)
70, 68, 58, 61, 61, 65, 59, 65, 63, 61, 60, 53, 55, 53, 57, 44, 50, 62, 46, 45, 54, 62, 45, 57, 44, 52, 54

Corresponding number of selected Gene ontology terms each TF is involved with:
(In the same order as above)
20, 20, 20, 20, 20, 20, 20, 20, 20, 20, 20, 20, 19, 20, 20, 20, 20, 19, 19, 20, 20, 19, 20, 18, 20, 16, 18

Cluster for columns 391 to 392, rows 377 to 382

Gene ontology terms

actin binding | actomyosin

TFs

V$SRF_Q6 | V$SRF_C | V$SRF_Q5_01 | V$SRF_Q4 | V$SRF_Q5_02 | V$SRF_01

Information

All related TFs:
(List of all TFs that are related to any of the PWMs)
SRF, SRF-I, SRF-L, SRF-M, SRF-S

Ranked gene list:
(All genes of the selected Gene ontology terms with hits of any of the selected TFs, ranked according to the total number of TFBS)
VCL, TPM1, TPM3, SRF, PFN1, MYL6, FLNA, CFL1, CD248, AKT1, ACTB, ZYX, TPM4, TAGLN, PPP1R12A, PDLIM5, MYL9, MYL3, CNN1, C3orf58, AK1, ACTC1, SBDS, DKFZp779C0757, CALD1, C10orf27, BAG2, TNNI1, SYCP1, MYO1E, MYH7, LSP1, DIAPH1, ARHGAP1, SYNPO, RND3, RAB27A, PXN, PER1, MYO1C, MYO1B, MYLK, MAP3K11, KEAP1, FN1, EPB41, ENAH, CAPZA2, VASP, UTRN, TXN, TMOD1, SLC25A1, SDHB, ROCK1, NRAP, MYO9B, MYL1, MARCKS, MAP2, LASP1, DSTN, DMD, CORO1A, ACTR3

Corresponding total number of TFBS:
(For each gene listed above, the total number of TFBS for any of the selected TFs, multiplied by the number of selected Gene ontology terms containing that gene)
12, 10, 6, 6, 6, 6, 6, 6, 6, 6, 6, 5, 5, 5, 5, 5, 5, 5, 5, 5, 5, 5, 4, 4, 4, 4, 4, 3, 3, 3, 3, 3, 3, 3, 2, 2, 2, 2, 2, 2, 2, 2, 2, 2, 2, 2, 2, 2, 1, 1, 1, 1, 1, 1, 1, 1, 1, 1, 1, 1, 1, 1, 1, 1, 1

Corresponding number of selected Gene ontology terms each gene is involved with:
(In the same order as above)
2, 2, 1, 1, 1, 1, 1, 1, 1, 2, 1, 1, 1, 1, 1, 1, 1, 1, 1, 1, 1, 1, 2, 1, 2, 2, 1, 1, 1, 1, 1, 1, 1, 1, 1, 1, 1, 2, 1, 2, 2, 2, 1, 1, 2, 1, 1, 2, 1, 1, 1, 1, 1, 1, 1, 1, 1, 1, 1, 1, 1, 1, 1, 1, 1

Corresponding number of selected TFs each gene is involved with:
(In the same order as above)
6, 5, 6, 6, 6, 6, 6, 6, 6, 3, 6, 5, 5, 5, 5, 5, 5, 5, 5, 5, 5, 5, 2, 4, 2, 2, 4, 3, 3, 3, 3, 3, 3, 3, 2, 2, 2, 1, 2, 1, 1, 1, 2, 2, 1, 2, 2, 1, 1, 1, 1, 1, 1, 1, 1, 1, 1, 1, 1, 1, 1, 1, 1, 1, 1

Gene ontology terms ranked according to mean of effect sizes in cluster:
actin binding | actomyosin

Corresponding mean of effect sizes of each Gene ontology term:
(In the same order as above)
3.10, 2.97

Corresponding total number of genes of each Gene ontology term:
(In the same order as above)
142, 118

Corresponding number of genes of each Gene ontology term with at least one TFBS (of selected TFs):
(In the same order as above)
45, 32

Corresponding total number of TFBS (of selected TFs) for each Gene ontology term (in all genes):
(In the same order as above)
120, 97

Corresponding number of selected TFs each Gene ontology term is involved with:
(In the same order as above)
6, 6

TFs ranked according to mean of effect sizes in cluster:
VSRF_Q5_02, VSRF_Q5_01, VSRF_Q6, VSRF_C, VSRF_Q4, VSRF_01

Corresponding mean of effect sizes of each TF:
(In the same order as above)
4.45, 3.11, 3.03, 3.01, 2.82, 1.80

Corresponding total number of TFBS for each TF (genome-wide):
(In the same order as above)
683, 693, 703, 687, 664, 659

Corresponding total number of TFBS for each TF (in all genes in selected Gene ontology terms):
(In the same order as above)
44, 37, 35, 37, 35, 29

Corresponding number of genes (of selected Gene ontology terms) each TF is involved with:
(In the same order as above)
37, 32, 32, 34, 32, 24

Corresponding number of selected Gene ontology terms each TF is involved with:
(In the same order as above)
2, 2, 2, 2, 2, 2

Cluster for columns 801 to 805, rows 373 to 383

Gene ontology terms

insulin-like growth factor binding protein complex | insulin-like growth factor receptor binding | insulin-like growth factor binding | insulin-like growth factor II binding | insulin-like growth factor I binding

TFs

V$P53_01 | V$CHX10_01 | V$POU3F2_01 | V$MEF2_04 | V$SRF_Q6 | V$SRF_C | V$SRF_Q5_01 |

V$SRF_Q4 | V$SRF_Q5_02 | V$SRF_01 | V$TATA_01

Information

All related TFs:
(List of all TFs that are related to any of the PWMs)
Chx10, MEF-2A, POU3F2, SRF, SRF-I, SRF-L, SRF-M, SRF-S, TBP, aMEF-2, p53, p53-isoform-1

Ranked gene list:
(All genes of the selected Gene ontology terms with hits of any of the selected TFs, ranked according to the total number of TFBS)
LBX1, FHL2, CYR61, ACTB, PTP4A1, HOXC6, CTGF, GHRL, BLVRB, IGF1R, FN1, DCN, AKT1, PDLIM5, THBS1, RARG, MMP2, TNF, SBDS, LEP, IGFBP3, IGF1, CLU, VTN, PSMP, PRH1, NDC80, FOXO3, VEGFA, STAT5B, SCN5A, S100P, PTRH1, PRL, PAEP, LHCGR, LATS1, IRS1, IGFBP7, IGFBP5, IGFBP2, IGF2, GH1, FOXO1, ESR1, CDKN1A, CCND1, BAX, TTR, SPP1, SERPINE1, RETN, PRH2, MMP3, HOXA10, GCG, ERBB2, CSH2, BEST1, STMN1, SCN2A, PIK3R1, IRF1, DUOXA1, CEACAM1, BHMT, BCL2, ANXA6, SSSCA1, KIAA1804, HRAS, HOXA11, HIF1A, HBA1, GRB2, GRB10, GAB1, FCGR1A, DMD, CYP11A1, CADM1, SMAD2, SHC1, SDCBP, RASSF1, PTPRF, PTEN, PSMD9, PIK3R2, PDGFRB, MYH6, LEPROT, ITGB5, IL17F, GRB7, GADD45A, EPHB2, CDH13, CDH1, CDC20, CD44, CASP3, CASP10, ATP8A2

Corresponding total number of TFBS:
(For each gene listed above, the total number of TFBS for any of the selected TFs, multiplied by the number of selected Gene ontology terms containing that gene)
35, 35, 28, 24, 20, 20, 20, 16, 16, 15, 15, 15, 15, 14, 12, 12, 12, 10, 10, 10, 10, 10, 10, 8, 8, 8, 8, 8, 5, 5, 5, 5, 5, 5, 5, 5, 5, 5, 5, 5, 5, 5, 5, 5, 5, 5, 5, 5, 4, 4, 4, 4, 4, 4, 4, 4, 4, 4, 4, 3, 3, 3, 3, 3, 3, 3, 3, 3, 2, 2, 2, 2, 2, 2, 2, 2, 2, 2, 2, 2, 2, 2, 2, 2, 2, 1, 1, 1, 1, 1, 1, 1, 1, 1, 1, 1, 1, 1, 1, 1, 1, 1, 1, 1, 1, 1

Corresponding number of selected Gene ontology terms each gene is involved with:
(In the same order as above)
5, 5, 4, 3, 5, 5, 5, 4, 4, 5, 5, 5, 5, 2, 3, 4, 4, 5, 5, 5, 5, 5, 5, 2, 4, 4, 4, 4, 5, 1, 5, 5, 5, 5, 5, 5, 5, 5, 5, 5, 5, 5, 5, 5, 5, 5, 5, 5, 4, 4, 4, 4, 4, 4, 4, 4, 4, 4, 4, 3, 1, 1, 3, 3, 1, 3, 3, 1, 1, 2, 1, 1, 2, 2, 1, 1, 1, 2, 1, 2, 1, 1, 1, 1, 1, 1, 1, 1, 1, 1, 1, 1, 1, 1, 1, 1, 1, 1, 1, 1, 1, 1, 1, 1, 1, 1

Corresponding number of selected TFs each gene is involved with:
(In the same order as above)
7, 7, 7, 8, 4, 4, 4, 4, 4, 3, 3, 3, 3, 7, 4, 3, 3, 2, 2, 2, 2, 2, 2, 4, 2, 2, 2, 2, 1, 5, 1, 1, 1, 1, 1, 1, 1, 1, 1, 1, 1, 1, 1, 1, 1, 1, 1, 1, 1, 1, 1, 1, 1, 1, 1, 1, 1, 1, 1, 3, 3, 1, 1, 3, 1, 1, 3, 2, 1, 2, 2, 1, 1, 2, 2, 2, 1, 2, 1, 2, 1, 1, 1, 1, 1, 1, 1, 1, 1, 1, 1, 1, 1, 1, 1, 1, 1, 1, 1, 1, 1, 1, 1, 1, 1, 1

Gene ontology terms ranked according to mean of effect sizes in cluster:
insulin-like growth factor binding | insulin-like growth factor II binding | insulin-like growth factor binding protein complex | insulin-like growth factor I binding | insulin-like growth factor receptor binding

Corresponding mean of effect sizes of each Gene ontology term:
(In the same order as above)
1.52, 1.18, 1.14, 1.09, 0.75

Corresponding total number of genes of each Gene ontology term:
(In the same order as above)
149, 132, 88, 132, 173

Corresponding number of genes of each Gene ontology term with at least one TFBS (of selected TFs):
(In the same order as above)

69, 64, 46, 64, 78

Corresponding total number of TFBS (of selected TFs) for each Gene ontology term (in all genes):
(In the same order as above)
148, 132, 93, 122, 149

Corresponding number of selected TFs each Gene ontology term is involved with:
(In the same order as above)
11, 11, 11, 11, 11

TFs ranked according to mean of effect sizes in cluster:
VSRF_Q5_02, VTATA_01, V$MEF2_04, V$SRF_C, VSRF_Q4, VPOU3F2_01, V$P53_01, V$SRF_Q6, VSRF_Q5_01, VSRF_01, V$CHX10_01

Corresponding mean of effect sizes of each TF:
(In the same order as above)
2.63, 2.09, 2.04, 1.58, 1.02, 1.00, 0.88, 0.66, 0.45, 0.36, -0.19

Corresponding total number of TFBS for each TF (genome-wide):
(In the same order as above)
683, 736, 621, 687, 664, 630, 615, 703, 693, 659, 669

Corresponding total number of TFBS for each TF (in all genes in selected Gene ontology terms):
(In the same order as above)
81, 86, 69, 68, 58, 51, 48, 50, 51, 48, 34

Corresponding number of genes (of selected Gene ontology terms) each TF is involved with:
(In the same order as above)
23, 22, 21, 19, 18, 20, 16, 16, 18, 14, 11

Corresponding number of selected Gene ontology terms each TF is involved with:
(In the same order as above)
5, 5, 5, 5, 5, 5, 5, 5, 5, 5, 5

Cluster for columns 397 to 401, rows 377 to 382

Gene ontology terms

filamentous actin | actin cytoskeleton | actin filament polymerization | actin filament binding | actin filament

TFs

V$SRF_Q6 | V$SRF_C | V$SRF_Q5_01 | V$SRF_Q4 | V$SRF_Q5_02 | V$SRF_01

Information

All related TFs:
(List of all TFs that are related to any of the PWMs)
SRF, SRF-I, SRF-L, SRF-M, SRF-S

Ranked gene list:
(All genes of the selected Gene ontology terms with hits of any of the selected TFs, ranked according to

the total number of TFBS)
VCL, FLNA, PDLIM5, SRF, PFN1, ACTG1, ACTB, ZYX, TAGLN, SVIL, MAPKAPK2, ACTA1, TPM1, RHOQ, PPP1R12A, ILK, C3orf58, ARPC1B, VTN, TPM3, SLC9A3R1, LIMA1, ITGB1, CFL1, CAP1, AKT1, CALD1, ARC, ACTA2, LSP1, HCLS1, DIAPH1, CTGF, ARHGAP1, ANXA6, SYNPO, EPB41, DLG4, CRIP1, C10orf27, ABLIM1, TNS1, SYCP1, SNAP25, RHOH, RHOG, MYO1E, ENAH, DNM2, VASP, UNC84B, TXN, TPM4, TNIK, SORBS1, SMTN, MYL9, DSTN, CSRP1, CNN1, ARHGEF19, ACTR3, ACTC1, SMURF1, SLC2A4, PXN, PPP2CA, PIK3R1, NBPF1, MYLK, MTTP, MAPK11, LTBR, LCP2, LASP1, GIT1, FN1, EPHB2, CCR5, CAPZA2, ARF5, AQP2, ZAP70, VILL, TMOD1, THBS1, SORBS3, SLC9A1, ROCK1, RHOB, MYO1C, MYO1B, MTHFR, MMP2, MARCKS, MAP2, LYN, LCK, GRB2, FYN, FERMT2, DMD, CYLD, CTSA, CORO1A, CLIC5, CDH5, CAMK2G, BIN3, BCR, ASCL1, ANXA2, UTRN, TNF, TMOD4, TGFB1I1, STK24, SBDS, S1PR1, RND3, RAB7A, RAB4A, RAB27B, RAB27A, PTGDR, PLAUR, PIP5K3, NCKIPSD, MYO9B, MBTPS1, MARK2, MAPKAPK5, GAB1, FBLIM1, EPHA1, DEF6, CYTH1, CTNNA1, CORO1C, CEBPE, CCDC91, ARHGEF1, AMPH, AKAP1, AGRN, ACTN1, ABL2, UBC, TNK2, TLN1, TFDP1, SYNE1, STMN1, SSH3, SQSTM1, SOS1, SLC22A3, SHC1, SH3KBP1, S1PR5, RPS27A, RND2, RHOV, PVRL1, PTPRU, PTPN6, PLXNB1, PARD3, PAK3, NUP43, MST1, MAPRE1, KALRN, ITGB8, HISPPD1, GGPS1, FTH1, FMN2, FLOT1, FCGR3A, FBXW7, ETV6, ERBB2, ELMO1, DYNLL1, DOCK8, CSNK2A1, CSK, COL18A1, CDC42SE1, CD82, CD69, CASP8, CASK, AHNAK, ACSBG2

Corresponding total number of TFBS:
(For each gene listed above, the total number of TFBS for any of the selected TFs, multiplied by the number of selected Gene ontology terms containing that gene)
30, 30, 25, 24, 24, 24, 24, 20, 20, 20, 20, 20, 15, 15, 15, 15, 15, 15, 12, 12, 12, 12, 12, 12, 12, 12, 10, 10, 10, 9, 9, 9, 9, 9, 9, 8, 8, 8, 8, 8, 8, 6, 6, 6, 6, 6, 6, 6, 6, 5, 5, 5, 5, 5, 5, 5, 5, 5, 5, 5, 5, 5, 5, 4, 4, 4, 4, 4, 4, 4, 4, 4, 4, 4, 4, 4, 4, 4, 4, 4, 4, 4, 3, 3, 3, 3, 3, 3, 3, 3, 3, 3, 3, 3, 3, 3, 3, 3, 3, 3, 3, 3, 3, 3, 3, 3, 3, 3, 3, 3, 3, 2, 2, 2, 2, 2, 2, 2, 2, 2, 2, 2, 2, 2, 2, 2, 2, 2, 2, 2, 2, 2, 2, 2, 2, 2, 2, 2, 2, 2, 2, 2, 2, 2, 2, 1, 1, 1, 1, 1, 1, 1, 1, 1, 1, 1, 1, 1, 1, 1, 1, 1, 1, 1, 1, 1, 1, 1, 1, 1, 1, 1, 1, 1, 1, 1, 1, 1, 1, 1, 1, 1, 1, 1, 1, 1, 1, 1, 1, 1

Corresponding number of selected Gene ontology terms each gene is involved with:
(In the same order as above)
5, 5, 5, 4, 4, 4, 4, 4, 4, 4, 4, 4, 3, 3, 3, 3, 3, 3, 3, 2, 4, 2, 3, 2, 4, 4, 5, 2, 5, 3, 3, 3, 3, 3, 3, 4, 4, 4, 2, 4, 2, 3, 2, 1, 1, 3, 2, 3, 3, 5, 1, 5, 1, 1, 1, 1, 1, 5, 1, 1, 1, 5, 1, 1, 4, 4, 1, 4, 1, 4, 4, 1, 2, 2, 4, 1, 4, 4, 1, 4, 2, 2, 1, 3, 3, 1, 1, 3, 3, 3, 3, 3, 3, 1, 3, 3, 3, 3, 3, 3, 1, 3, 1, 1, 3, 1, 3, 1, 1, 1, 1, 3, 2, 2, 1, 1, 2, 1, 1, 1, 2, 1, 2, 1, 1, 2, 2, 2, 2, 2, 2, 2, 1, 1, 1, 1, 1, 1, 2, 2, 2, 1, 2, 1, 1, 2, 2, 1, 1, 1, 1, 1, 1, 1, 1, 1, 1, 1, 1, 1, 1, 1, 1, 1, 1, 1, 1, 1, 1, 1, 1, 1, 1, 1, 1, 1, 1, 1, 1, 1, 1, 1, 1, 1, 1, 1, 1, 1, 1, 1, 1

Corresponding number of selected TFs each gene is involved with:
(In the same order as above)
6, 6, 5, 6, 6, 6, 6, 5, 5, 5, 5, 5, 5, 5, 5, 5, 5, 5, 4, 6, 3, 6, 4, 6, 3, 3, 2, 5, 2, 3, 3, 3, 3, 3, 3, 2, 2, 2, 4, 2, 4, 2, 3, 6, 6, 2, 3, 2, 2, 1, 5, 1, 5, 5, 5, 5, 5, 1, 5, 5, 5, 1, 5, 4, 1, 1, 4, 1, 4, 1, 1, 4, 2, 2, 1, 4, 1, 1, 4, 1, 2, 2, 3, 1, 1, 3, 3, 1, 1, 1, 1, 1, 1, 3, 1, 1, 1, 1, 1, 1, 3, 1, 3, 3, 1, 3, 1, 3, 3, 3, 3, 1, 1, 1, 2, 2, 1, 2, 2, 2, 1, 2, 1, 2, 2, 1, 1, 1, 1, 1, 1, 1, 2, 2, 2, 2, 2, 2, 1, 1, 1, 2, 1, 2, 2, 1, 1, 1, 1, 1, 1, 1, 1, 1, 1, 1, 1, 1, 1, 1, 1, 1, 1, 1, 1, 1, 1, 1, 1, 1, 1, 1, 1, 1, 1, 1, 1, 1, 1, 1, 1, 1, 1, 1, 1, 1, 1, 1, 1, 1, 1

Gene ontology terms ranked according to mean of effect sizes in cluster:
actin filament | filamentous actin | actin filament polymerization | actin cytoskeleton | actin filament binding

Corresponding mean of effect sizes of each Gene ontology term:
(In the same order as above)
3.10, 2.68, 2.23, 2.15, 2.02

Corresponding total number of genes of each Gene ontology term:
(In the same order as above)
177, 439, 300, 605, 62

Corresponding number of genes of each Gene ontology term with at least one TFBS (of selected TFs):
(In the same order as above)
48, 106, 79, 150, 26

Corresponding total number of TFBS (of selected TFs) for each Gene ontology term (in all genes):
(In the same order as above)
137, 267, 196, 342, 60

Corresponding number of selected TFs each Gene ontology term is involved with:
(In the same order as above)
6, 6, 6, 6, 6

TFs ranked according to mean of effect sizes in cluster:
VSRF_C, VSRF_Q6, VSRF_01, VSRF_Q5_02, VSRF_Q4, VSRF_Q5_01

Corresponding mean of effect sizes of each TF:
(In the same order as above)
3.05, 2.97, 2.57, 2.24, 1.97, 1.81

Corresponding total number of TFBS for each TF (genome-wide):
(In the same order as above)
687, 703, 659, 683, 664, 693

Corresponding total number of TFBS for each TF (in all genes in selected Gene ontology terms):
(In the same order as above)
186, 174, 162, 163, 157, 160

Corresponding number of genes (of selected Gene ontology terms) each TF is involved with:
(In the same order as above)
84, 77, 69, 76, 69, 75

Corresponding number of selected Gene ontology terms each TF is involved with:
(In the same order as above)
5, 5, 5, 5, 5, 5

Cluster for columns 1210 to 1213, rows 392 to 410

Gene ontology terms

contractile ring | estrogen metabolic process | drug metabolic process | xenobiotic metabolic process

TFs

V$TAL1BETAE47_01 | V$RFX1_02 | V$HFH4_01 | V$AIRE_02 | V$MEF2_03 | V$MEF2_02 | V$RSRFC4_Q2 | V$RSRFC4_01 | V$PPAR_DR1_Q2 | V$DR1_Q3 | V$HNF4_DR1_Q3 | V$COUP_DR1_Q6 | V$COUP_01 | V$HNF4_01 | V$HNF4_Q6_01 | V$HNF4ALPHA_Q6 | V$COUPTF_Q6 | V$PPARG_03 | V$PPARG_01

Information

All related TFs:
(List of all TFs that are related to any of the PWMs)

AIRE, COUP, COUP-TF1, COUP-TF2, E47, FOXJ1, FOXJ1a, FOXJ1b, HNF-4, HNF-4alpha, HNF-4alpha1, HNF-4alpha2, HNF-4alpha3, HNF-4alpha4, HNF-4alpha7, HNF-4gamma, MEF-2A, PPAR-alpha, PPAR-alpha:RXR-alpha, PPAR-beta, PPAR-gamma, PPAR-gamma1, PPAR-gamma2, PPAR-gamma2:RXR-alpha, PPAR-gamma:RXR-alpha, RAR-alpha:RXR-alpha, RFX1, RSRFC4, Tal-1beta, aMEF-2

Ranked gene list:
(All genes of the selected Gene ontology terms with hits of any of the selected TFs, ranked according to the total number of TFBS)
SLC35A2, CYP1A1, PLEC1, CYP27B1, PPARA, COMT, SULT1A3, PPIG, NFE2L2, GABPA, AHR, RHOA, PGP, PPIF, EDC4, XPR1, UGT1A9, UGT1A6, SHBG, MYBBP1A, MBD2, PKP4, DBP, MPG, CYP2E1, CES2, UGT1A7, UGT1A10, SULT1A1, SOD2, SLC45A2, PCNT, MTHFR, INTU, FMO4, BRD2, TEF, TBC1D9, NR6A1, NR1H4, NQO1, MGST1, GADD45B, CYP7A1, CYP17A1, CHST6, CALM3, ABCB4, UGT1A8, UGT1A5, UGT1A4, TPMT, TNFSF13, SULT2B1, SLC38A1, RXRA, NR3C1, NR2F2, NGEF, JAM3, IBSP, HNF4A, GSTT2B, GSTT2, FOXO1, FBXW7, ESR1, DECR1, CYCS, CDC7, ARR3, ALDH2, ALAS1, ABCC1, XRCC1, UGT1A3, TBC1D8, TAT, SLC40A1, SLC26A2, PON3, PLEKHG6, PHIP, PELP1, PC, OGG1, NDOR1, MED1, MDM4, MAP3K8, KEAP1, INSM1, HTR1A, HSPBP1, GUSB, GUCY1B3, GRIN1, GPX2, GPT, GLB1, GHRHR, FUT1, ESR2, DPYD, DMPK, CXADR, CSH1, CLSTN2, CHST7, CDC42, CDC14A, CD46, CD247, CCL4L2, CCL4L1, CAT, CALM1, BCHE, BAMBI

Corresponding total number of TFBS:
(For each gene listed above, the total number of TFBS for any of the selected TFs, multiplied by the number of selected Gene ontology terms containing that gene)
36, 36, 32, 30, 24, 24, 20, 20, 15, 15, 12, 11, 11, 9, 9, 8, 8, 8, 8, 8, 8, 7, 7, 6, 6, 5, 4, 4, 4, 4, 4, 4, 4, 4, 4, 4, 3, 3, 3, 3, 3, 3, 3, 3, 3, 3, 3, 3, 2, 2, 2, 2, 2, 2, 2, 2, 2, 2, 2, 2, 2, 2, 2, 2, 2, 2, 2, 2, 2, 2, 2, 2, 2, 2, 1, 1, 1, 1, 1, 1, 1, 1, 1, 1, 1, 1, 1, 1, 1, 1, 1, 1, 1, 1, 1, 1, 1, 1, 1, 1, 1, 1, 1, 1, 1, 1, 1, 1, 1, 1, 1, 1, 1, 1, 1, 1, 1, 1, 1, 1, 1

Corresponding number of selected Gene ontology terms each gene is involved with:
(In the same order as above)
4, 4, 2, 3, 3, 3, 2, 4, 3, 3, 4, 1, 1, 3, 1, 2, 4, 4, 1, 1, 2, 1, 1, 1, 3, 1, 2, 2, 4, 1, 4, 2, 2, 1, 1, 2, 1, 3, 1, 3, 3, 1, 1, 3, 3, 1, 1, 1, 1, 1, 1, 1, 2, 1, 2, 1, 1, 1, 1, 1, 2, 2, 2, 1, 1, 1, 2, 2, 1, 1, 1, 1, 2, 1, 1, 1, 1, 1, 1, 1, 1, 1, 1, 1, 1, 1, 1, 1, 1, 1, 1, 1, 1, 1, 1, 1, 1, 1, 1, 1, 1, 1, 1, 1, 1, 1, 1, 1, 1, 1, 1, 1, 1, 1, 1, 1

Corresponding number of selected TFs each gene is involved with:
(In the same order as above)
9, 9, 16, 10, 8, 8, 10, 5, 5, 5, 3, 11, 11, 3, 9, 4, 2, 2, 8, 8, 4, 7, 7, 6, 2, 5, 2, 2, 1, 4, 1, 2, 2, 4, 4, 2, 3, 1, 3, 1, 1, 3, 3, 1, 1, 3, 3, 3, 2, 2, 2, 2, 1, 2, 1, 2, 2, 2, 2, 2, 1, 1, 1, 2, 2, 2, 1, 1, 2, 2, 2, 2, 1, 1, 1, 1, 1, 1, 1, 1, 1, 1, 1, 1, 1, 1, 1, 1, 1, 1, 1, 1, 1, 1, 1, 1, 1, 1, 1, 1, 1, 1, 1, 1, 1, 1, 1, 1, 1, 1, 1, 1, 1, 1, 1, 1, 1, 1, 1, 1, 1

Gene ontology terms ranked according to mean of effect sizes in cluster:
estrogen metabolic process | xenobiotic metabolic process | drug metabolic process | contractile ring

Corresponding mean of effect sizes of each Gene ontology term:
(In the same order as above)
2.52, 2.10, 1.90, 1.54

Corresponding total number of genes of each Gene ontology term:
(In the same order as above)
37, 128, 150, 150

Corresponding number of genes of each Gene ontology term with at least one TFBS (of selected TFs):
(In the same order as above)
18, 51, 59, 56

Corresponding total number of TFBS (of selected TFs) for each Gene ontology term (in all genes):
(In the same order as above)
66, 141, 180, 169

Corresponding number of selected TFs each Gene ontology term is involved with:
(In the same order as above)
19, 19, 19, 19

TFs ranked according to mean of effect sizes in cluster:
V$PPARG_01, V$HNF4_DR1_Q3, V$COUP_01, V$DR1_Q3, V$HNF4_Q6_01, V$AIRE_02, V$PPAR_DR1_Q2, V$HNF4_01, V$TAL1BETAE47_01, V$PPARG_03, V$COUP_DR1_Q6, V$MEF2_03, V$RFX1_02, V$RSRFC4_01, V$HNF4ALPHA_Q6, V$RSRFC4_Q2, V$COUPTF_Q6, V$HFH4_01, V$MEF2_02

Corresponding mean of effect sizes of each TF:
(In the same order as above)
3.22, 3.13, 2.71, 2.56, 2.43, 2.30, 2.26, 2.24, 2.23, 2.21, 2.15, 2.14, 1.92, 1.55, 1.45, 1.37, 1.02, 0.74, 0.63

Corresponding total number of TFBS for each TF (genome-wide):
(In the same order as above)
642, 666, 660, 667, 687, 607, 684, 674, 684, 647, 651, 650, 613, 655, 688, 655, 696, 619, 700

Corresponding total number of TFBS for each TF (in all genes in selected Gene ontology terms):
(In the same order as above)
31, 35, 32, 31, 32, 44, 29, 29, 29, 44, 27, 30, 26, 24, 26, 24, 20, 21, 22

Corresponding number of genes (of selected Gene ontology terms) each TF is involved with:
(In the same order as above)
21, 19, 18, 15, 18, 26, 15, 14, 15, 25, 14, 15, 16, 13, 15, 14, 14, 15, 14

Corresponding number of selected Gene ontology terms each TF is involved with:
(In the same order as above)
4, 4, 4, 4, 4, 4, 4, 4, 4, 4, 4, 4, 4, 4, 4, 4, 4, 4, 4

Cluster for columns 1094 to 1122, rows 23 to 42

Gene ontology terms

polyamine biosynthetic process | polyamine metabolic process | RNA catabolic process | mitochondrial DNA replication | leaf senescence | mRNA transport | nucleocytoplasmic transport | nuclear-transcribed mRNA catabolic process, nonsense-mediated decay | polysome | mRNA binding | eukaryotic translation initiation factor 3 complex | translation | translational initiation | mRNA catabolic process | nucleic acid binding | RNA biosynthetic process | RNA transport | nucleolus organizer region | rRNA transcription | rRNA processing | ribosome assembly | ribosome biogenesis | small ribosomal subunit | translational elongation | eukaryotic translation initiation factor 2 complex | regulation of translation | ribosome binding | ribosomal subunit | ribosome

TFs

V$YY1_Q6_02 | V$YY1_Q6 | V$NFMUE1_Q6 | V$YY1_02 | V$GABP_B | V$ELK1_02 | V$TAXCREB_01 | V$ATF6_01 | V$HLF_01 | V$E4BP4_01 | V$USF_01 | V$MAX_01 | V$USF_Q6 | V$USF_02 | V$USF_Q6_01 | V$MYCMAX_03 | V$ARNT_01 | V$MYC_Q2 | V$CLOCKBMAL_Q6 | V$MYCMAX_02

Information

All related TFs:
(List of all TFs that are related to any of the PWMs)
ATF6, Arnt, CREB, Clock:BMAL, Clock:BMAL1, Clock:BMAL2, E4BP4, Elk-1, Elk-1-isoform1, GABP-alpha, GABP-alpha:GABP-beta, GABP-beta1, GABP-beta2, Hlf, Max, Max1, NF-muE1, Tax, USF, USF-1, USF1, USF1:USF2, USF1a, USF1b, USF2, USF2a, USF2b, YY1, c-Myc, deltaCREB, factor

Ranked gene list:
(All genes of the selected Gene ontology terms with hits of any of the selected TFs, ranked according to the total number of TFBS)
EIF5A, EIF4G1, HNRPDL, EIF3A, KIAA0664, EIF4A1, NPM1, UBTF, HNRNPA1, NCL, PER1, EIF4E, DDX5, RPS15, ZFP36, ATF4, RPS2, PTBP1, ODC1, EEF1A1, EIF3B, ANXA6, PCBP1, ILF3, EIF5, EIF4A2, GAPDH, NP, KHDRBS1, GIT1, AKT1, ACTB, SYNCRIP, EIF1, CIRBP, SQSTM1, CDK5R1, ADAM8, UBA52, SSR1, RPL13A, PROC, CRTC2, CALR, SFRS2, RPL3, NR1H2, MKNK2, KHSRP, HNRNPL, GNB2L1, EEF1B2, CCND1, SLC20A1, EEF2, YBX1, XPO1, UBB, SLC2A4RG, RPS5, UPF1, STXBP1, SART3, RPL23A, RPL13, CSDE1, ATP5E, TARBP2, SRM, SLBP, RPL11, NR3C1, JUN, HSPA8, HDLBP, GTF3A, GCN1L1, ETF1, CUX1, AMD1, RHBDL1, MTG1, USP7, RPS27A, RPL14, RCC1, MVK, IRF9, EIF3K, DOHH, ATF1, UTS2R, TSC2, TFRC, RPS18, NECAB3, ING1, DDIT3, CDK4, BNIP1, ATP5A1, ABL2, UBC, HMHA1, ERCC6, ELAVL1, QTRT1, OSM, FASN, EEF1D, DPH2, DPH1, CISH, UPF2, THNSL1, TCEA2, TBX1, SLC25A1, RPS6KA1, RPS24, RPS21, RPN1, RPLP0, RORC, RNH1, PKN1, NXT1, NUP98, NOL5A, NCOA6, MPG, IRS1, HNRNPC, ERF, EMG1, EEF2K, EBNA1BP2, CRIP1, ARHGEF2, ZFP36L1, HOXA7, CFL1, ADK, ACTG1, XPOT, TYMP, TUFM, TAF1C, SUPV3L1, RPS16, RERE, PDGFB, OAZ1, NAPRT1, MTHFR, GPX1, GGT1, EPB41, DDX21, CSDA, CARS2, BAX, ATAD2, RPS6KB1, RPL10, MGC111011, MARCKSL1, KAT5, JUNB, EXOSC8, BBS9, ARRB2, APEX1, VARS, SPSB1, SLC36A1, SLC16A1, SECISBP2, SBNO2, RPS8, RPS28, RPS19, RPL23, RHOF, POLRMT, POLR2A, POLG2, PNPT1, PCK2, P4HB, MRPL28, GUSB, GPS1, FARSA, EIF6, EIF4H, EIF4G2, EIF2A, DDX6, CTRL, CTF1, CSNK2A1, BOP1, ATF6, ADAR, SUMO3, STAT5A, SSSCA1, PSMC4, NME2, MAT2A, HNRNPK, GTF2A1, FBL, ENOPH1, EDC4, DYM, YBX2, TSHZ1, TFB2M, SRRM1, SLC38A2, RPS23, RPS12, RPL36, RPL35A, RPL30, RPL24, RPL22, RPAIN, RPA1, RBM19, RBM15, RARA, RABGEF1, PURA, PTK2B, PSMD8, PSMC5, POLD3, PARN, PABPN1, NFE2L2, NCBP2, MPHOSPH10, MKNK1, MGC126674, LTB, LONP1, JAG2, ISYNA1, INTS6, IGF2BP1, HSPD1, HNRNPD, GLTSCR2, GFM1, FUS, FHL1, FBXO32, FAM60A, FAM49B, FAM168B, ESCO2, DHPS, DCP1B, CTSD, COTL1, CORO1A, CEBPE, CDKN2C, CCNH, CCNB1, CASP9, C1QBP, C14orf4, BRCA2, ARID5A, APRT, APC, ALG1, WDR77, UQCRH, TGFB1, TERT, TAF8, SLC33A1, SH3BP4, RPSA, RPS3, RPS14, PRKCZ, PGK1, PDAP1, PCNA, NOL3, NAB2, MVP, MARCH8, MAPK1, LIF, JMJD6, HSP90B1, GEMIN4, FMR1, ERP29, EIF4G3, EIF3G, EIF3E, EEFSEC, DHFR, DGCR2, CYTH2, CPEB1, CANX, C2orf28, VIM, TRIM25, TPP1, TNPO1, TNFSF13, TERF2IP, TAF15, SUMO1, STAT5B, STAT3, SSBP1, SNRPA, SKIV2L, SHMT2, SAT1, SAMD4A, S100A10, RPLP1, RPL32, RPL26, RPL18, RPL17, RBM3, RAF1, PTPN11, PTBP2, POLR2G, POLE4, PECI, PCBP2, NUP62, NRF1, NFKBIA, MYB, MXD1, MX1, MORF4, MBNL1, MARVELD2, MARS, MAPKAPK2, MAPK3, IRF1, IL6R, IGF1R, HSPC120, HSP90AA1, HMOX1, HIPK3, GRIN1, GPX4, GNL2, GCDH, GAR1, G3BP1, FOS, FKBP4, EXOSC4, ETV5, EIF4EBP2, EIF3H, DUSP1, DTYMK, DKC1, DHODH, DGAT1, DDX19B, DDX1, DCP1A, CLN3, CASC3, CALM1, C14orf172, BXDC1, BAG3, ATF5, ASCL1, ARID2, ANP32A, XIAP, TRMT6, TOP1, TNFRSF14, TFB1M, TEF, TAT, STK11, SRPR, SRP54, SMG7, SCARF2, RRAS, RPP30, RPL9, RPL15, RHEB, REXO2, RELA, REL, RANBP1, PSMP, PRNP, PRKG1, PRKAR1A, PRAP1, PPY, POLG, POLDIP3, POLA1, PLA2G1B, PIK3R2, NUP88, NUP210, NSFL1C, NRAS, NR4A1, NR3C2, NLRP3, NKX6-2, NARS, MSTN, MSL2, MGC111084, MDM2, MCAT, MAN2C1, LIN28, LAT, LASS2, IMP4, IL2, IL13, HMGA1, HGFAC, GDE1, FTL, FTH1, ELL, EIF4E2, EIF3I, EGR1, EDC3, DLG4, CYCS, CYC1, COPA, COL9A3, CLEC11A, CHRM1, CDKN2D, CDKN1C, CASP3, BCL2L11, BCKDK, ATP6V0D1, ATG5, ZNRD1, ZFYVE9, WDR61, WDR48, WARS, TXNRD1, TXNDC15, TRIM37, TRIM3, TRIM21, TPT1, TPI1, TNFRSF1A, TNF, TMEM201, TK2, TGFBR2, TGFB2, TG, TFAP2A, TCF3, SYF2, STAT6, STAT1, SRP9, SRP19, SPG7, SPAG9, SNRPG, SMG6, SLC31A1, SLC30A9, SKIL, SERBP1, RTN4, RPS6KB2, RPN2, RPL6, RPL41,

RNPS1, RNF39, RELB, RCL1, RARS2, PTCHD3, PRMT3, PRKAB1, PPAT, POLR2B, PIK3C3, PHAX, PDGFA, PDF, PDCD11, PABPC4, OSBP, OBFC2B, NXF1, NT5C2, NR4A3, NPM3, NOS2, NOP10, NOLC1, NME1, NFIL3, NDOR1, MTR, MTIF3, MOCOS, MKI67IP, METTL3, MCL1, MBP, MARS2, MAD1L1, LSM14A, LIN9, KTN1, KRR1, KARS, JUND, IVNS1ABP, ISG15, IMP3, IMMP1L, IL27, IKBKAP, IGF2, IARS, HSP90AB1, HSF1, HOXD13, HIST1H4I, HIST1H4C, HIST1H3F, HIST1H3C, HIRA, HIF1A, HEXIM2, GTPBP3, GRIA1, GADD45A, FTSJ1, FEN1, FDXR, FAU, FARS2, ELF2, EIF5A2, EIF4ENIF1, EIF4EBP3, EIF2B3, EIF2B2, EIF2B1, DTD1, DR1, DPH3, DNAJC5, DNAJC2, DNAJB9, DNAJB11, DNAJB1, DICER1, DHRS11, DDX3X, DDX25, DCTN3, DAP3, DAP, CUGBP2, CUGBP1, CSRP1, CSF2, CRTC1, CPSF3, CPE, COPS5, COL6A2, COL6A1, COL2A1, COL11A2, COIL, CNBP, CLPP, CDKN1B, CD44, C10orf27, C10orf2, ATP7A, ATF2, ARNT, AIRE, AHR, ZNF74, ZNF167, ZC3H7B, YRDC, YAP1, XPC, XPA, WT1, WNK1, WDR36, VDAC1, UGT8, TYRO3, TRNT1, TPX2, TNFSF10, TKT, TEAD3, TEAD2, TCOF1, TBP, TAS1R1, TAF9, SUMO2, STK19, SRP68, SRGN, SPTB, SPECC1, SPARC, SOS1, SLC5A10, SLC3A2, SLC25A4, SLC25A19, SFRS5, SERPINH1, SERPINB8, RPP38, RPL34, RPH3AL, RHOBTB2, RHOA, RARS, RANGRF, RANGAP1, RANBP3, RANBP2, PSMD14, PSMC2, PRG2, PRDX5, PPP2R4, PPAN, POM121, PLAA, PIK3R1, PET112L, PDLIM4, PARVA, PAK1IP1, P11, OBFC2A, NUTF2, NUP214, NTRK2, NT5C, NRSN1, NPTXR, NPTN, NPPA, NPC1, NOL12, NIP7, NHP2, NGLY1, NFE2, NEU1, NCOA5, NAF1, MRE11A, MMP3, MMP13, MIPEP, MANBA, LGALS1, LBX1, LARS2, KLF3, JAK2, ITPR3, IPO4, IMMP2L, IL6ST, HSPA9, HLCS, HIVEP3, HIST1H3H, HIST1H3G, HBEGF, HBA2, GZMM, GTF2A2, GSPT2, GRN, GLUD1, GLS, GAST, GARS, FUBP1, FOXK1, FOSL1, FLJ21865, FGFR1, FBF1, FAS, FAP, EXOSC9, ERCC3, EIF4E3, EIF2C4, EARS2, DYNC1H1, DOM3Z, DNAJC24, DKFZp686G1042, DHX15, DDX47, DDC, CWC15, CPS1, CP, CIB1, CHPF, CHERP, CGN, CDK7, CDC25C, CD47, CD46, CD3EAP, CAST, CASP7, CAPNS1, CABP1, C1orf102, C18orf10, AZI1, AUH, ATR, ATF3, ARG2, ARG1, APOE, ANXA5, ACSL5, ACLY

Corresponding total number of TFBS:
(For each gene listed above, the total number of TFBS for any of the selected TFs, multiplied by the number of selected Gene ontology terms containing that gene)
160, 105, 91, 91, 90, 90, 81, 80, 80, 77, 76, 76, 72, 64, 56, 56, 55, 54, 54, 52, 50, 45, 44, 42, 42, 42, 40, 36, 36, 36, 36, 36, 35, 33, 32, 30, 30, 30, 28, 28, 28, 27, 27, 26, 25, 24, 24, 24, 24, 24, 24, 24, 24, 22, 22, 21, 21, 21, 21, 21, 20, 20, 20, 20, 20, 20, 20, 18, 18, 18, 18, 18, 18, 18, 18, 18, 18, 18, 18, 18, 17, 17, 16, 16, 16, 16, 16, 16, 16, 16, 16, 15, 15, 15, 15, 15, 15, 15, 15, 15, 15, 15, 14, 14, 14, 14, 13, 13, 13, 13, 13, 13, 13, 12, 12, 12, 12, 12, 12, 12, 12, 12, 12, 12, 12, 12, 12, 12, 12, 12, 12, 12, 12, 12, 12, 12, 12, 12, 12, 11, 11, 11, 11, 11, 10, 10, 10, 10, 10, 10, 10, 10, 10, 10, 10, 10, 10, 10, 10, 10, 10, 10, 10, 9, 9, 9, 9, 9, 9, 9, 9, 9, 9, 8, 8, 8, 8, 8, 8, 8, 8, 8, 8, 8, 8, 8, 8, 8, 8, 8, 8, 8, 8, 8, 8, 8, 8, 8, 8, 8, 8, 8, 8, 8, 8, 7, 7, 7, 7, 7, 7, 7, 7, 7, 7, 7, 7, 6, 6, 6, 6, 6, 6, 6, 6, 6, 6, 6, 6, 6, 6, 6, 6, 6, 6, 6, 6, 6, 6, 6, 6, 6, 6, 6, 6, 6, 6, 6, 6, 6, 6, 6, 6, 6, 6, 6, 6, 6, 6, 6, 6, 6, 6, 6, 6, 6, 6, 6, 6, 6, 6, 6, 6, 6, 6, 6, 6, 6, 6, 6, 6, 5, 5, 5, 5, 5, 5, 5, 5, 5, 5, 5, 5, 5, 5, 5, 5, 5, 5, 5, 5, 5, 5, 5, 5, 5, 5, 5, 5, 5, 5, 5, 5, 5, 5, 5, 4, 4, 4, 4, 4, 4, 4, 4, 4, 4, 4, 4, 4, 4, 4, 4, 4, 4, 4, 4, 4, 4, 4, 4, 4, 4, 4, 4, 4, 4, 4, 4, 4, 4, 4, 4, 4, 4, 4, 4, 4, 4, 4, 4, 4, 4, 4, 4, 4, 4, 4, 4, 4, 4, 4, 4, 4, 4, 4, 4, 4, 4, 4, 4, 4, 4, 4, 4, 4, 4, 4, 4, 4, 4, 4, 4, 4, 4, 4, 3, 3, 3, 3, 3, 3, 3, 3, 3, 3, 3, 3, 3, 3, 3, 3, 3, 3, 3, 3, 3, 3, 3, 3, 3, 3, 3, 3, 3, 3, 3, 3, 3, 3, 3, 3, 3, 3, 3, 3, 3, 3, 3, 3, 3, 3, 3, 3, 3, 3, 3, 3, 3, 3, 3, 3, 3, 3, 3, 3, 3, 3, 3, 3, 3, 3, 3, 3, 3, 3, 3, 3, 3, 3, 3, 3, 3, 2, 1

Corresponding number of selected Gene ontology terms each gene is involved with:
(In the same order as above)
16, 15, 13, 13, 9, 15, 9, 10, 10, 11, 4, 19, 8, 8, 4, 4, 11, 6, 6, 13, 10, 9, 4, 3, 14, 14, 5, 6, 9, 3, 3, 9, 7, 11, 2, 3, 5, 10, 7, 4, 4, 9, 3, 2, 5, 8, 4, 3, 3, 3, 6, 4, 4, 2, 11, 7, 7, 7, 3, 7, 10, 4, 5, 2, 4, 4, 5, 2, 2, 3, 9, 3, 6, 6, 3, 6, 6, 9, 6, 6, 1, 17, 2, 8, 4, 4, 2, 4, 4, 2, 2, 15, 3, 5, 5, 5, 3, 3, 1, 5, 3, 5, 7, 2, 2, 7, 1, 1, 1, 1, 1, 1, 1, 4, 3, 2, 4, 3, 6, 4, 4, 3, 4, 1, 3, 1, 2, 3, 2, 4, 3, 3, 12, 3, 3, 3, 4, 1, 4, 11, 1, 1, 1, 1, 2, 1, 10, 5, 2, 5, 5, 1, 2, 1, 1, 2, 5, 1,

2, 5, 2, 1, 5, 9, 3, 1, 3, 1, 1, 3, 9, 1, 1, 4, 4, 1, 1, 4, 1, 4, 2, 4, 4, 1, 2, 2, 1, 8, 1, 2, 2, 4, 2, 2, 8, 4, 4, 4, 4, 1, 1, 4, 4, 4, 2, 1, 1, 1, 7, 1, 1, 7, 1, 7, 1, 7, 1, 2, 3, 3, 2, 1, 2, 6, 2, 3, 2, 3, 2, 2, 2, 1, 1, 6, 2, 3, 2, 2, 2, 6, 2, 3, 1, 6, 3, 6, 1, 3, 3, 1, 2, 3, 2, 3, 6, 3, 3, 3, 3, 2, 3, 3, 3, 3, 2, 6, 1, 2, 3, 2, 1, 6, 3, 1, 3, 2, 1, 2, 1, 2, 1, 5, 1, 1, 1, 1, 1, 5, 5, 5, 5, 1, 5, 5, 1, 5, 1, 1, 1, 5, 1, 1, 1, 5, 5, 1, 5, 5, 5, 5, 5, 1, 5, 1, 5, 1, 2, 2, 2, 1, 2, 2, 2, 2, 1, 1, 4, 1, 4, 1, 2, 4, 4, 2, 2, 4, 1, 1, 2, 2, 4, 4, 1, 2, 2, 4, 1, 4, 2, 2, 1, 2, 4, 1, 1, 4, 2, 1, 1, 2, 1, 1, 2, 1, 1, 1, 2, 2, 4, 2, 2, 4, 2, 2, 4, 2, 4, 1, 1, 4, 2, 1, 2, 2, 4, 1, 1, 2, 1, 1, 2, 1, 2, 1, 1, 3, 3, 1, 1, 3, 1, 3, 1, 3, 3, 1, 3, 1, 1, 3, 3, 3, 3, 3, 3, 1, 1, 1, 1, 1, 3, 1, 1, 3, 3, 1, 1, 1, 1, 3, 3, 1, 1, 1, 3, 1, 3, 3, 1, 3, 1, 1, 3, 3, 1, 1, 3, 1, 1, 1, 1, 1, 1, 1, 3, 3, 1, 1, 1, 3, 3, 3, 1, 3, 1, 3, 3, 3, 1, 1, 3, 1, 1, 1, 2, 1, 1, 2, 2, 1, 2, 2, 1, 1, 1, 2, 2, 1, 2, 1, 2, 2, 1, 2, 1, 2, 2, 1, 1, 1, 2, 1, 1, 1, 1, 1, 1, 2, 2, 2, 2, 1, 1, 2, 2, 2, 1, 2, 1, 2, 2, 1, 1, 2, 1, 1, 1, 2, 2, 2, 1, 1, 2, 1, 2, 1, 1, 1, 2, 2, 2, 2, 1, 1, 2, 1, 1, 2, 1, 1, 1, 1, 1, 2, 1, 1, 2, 2, 2, 2, 2, 1, 1, 2, 2, 2, 1, 1, 1, 2, 1, 1, 1, 1, 2, 1, 2, 1, 2, 2, 1, 2, 2, 2, 2, 2, 2, 1, 1, 2, 2, 1, 1, 2, 1, 1, 2, 1, 1, 1, 2, 1, 1, 2, 2, 2, 1, 2, 2, 1, 1, 1, 1, 2, 1, 2, 1, 2, 2, 1, 2, 1

Corresponding number of selected TFs each gene is involved with:
(In the same order as above)
10, 7, 7, 7, 10, 6, 9, 8, 8, 7, 19, 4, 9, 8, 14, 14, 5, 9, 9, 4, 5, 5, 11, 14, 3, 3, 8, 6, 4, 12, 12, 4, 5, 3, 16, 10, 6, 3, 4, 7, 7, 3, 9, 13, 5, 3, 6, 8, 8, 8, 4, 6, 6, 11, 2, 3, 3, 3, 7, 3, 2, 5, 4, 10, 5, 5, 4, 9, 9, 6, 2, 6, 3, 3, 6, 3, 3, 2, 3, 3, 17, 1, 8, 2, 4, 4, 8, 4, 4, 8, 8, 1, 5, 3, 3, 3, 5, 5, 15, 3, 5, 3, 2, 7, 7, 2, 13, 13, 13, 13, 13, 13, 13, 3, 4, 6, 3, 4, 2, 3, 3, 4, 3, 12, 4, 12, 6, 4, 6, 3, 4, 4, 1, 4, 4, 4, 3, 12, 3, 1, 11, 11, 11, 11, 5, 10, 1, 2, 5, 2, 2, 10, 5, 10, 10, 5, 2, 10, 5, 2, 5, 10, 2, 1, 3, 9, 3, 9, 9, 3, 1, 9, 9, 2, 2, 8, 8, 2, 8, 2, 4, 2, 2, 8, 4, 4, 8, 1, 8, 4, 4, 2, 4, 4, 1, 2, 2, 2, 2, 8, 8, 2, 2, 2, 4, 7, 7, 7, 1, 7, 7, 1, 7, 1, 7, 1, 7, 3, 2, 2, 3, 6, 3, 1, 3, 2, 3, 2, 3, 3, 3, 6, 6, 1, 3, 2, 3, 3, 3, 1, 3, 2, 6, 1, 2, 1, 6, 2, 2, 6, 3, 2, 3, 2, 1, 2, 2, 2, 2, 3, 2, 2, 2, 2, 3, 1, 6, 3, 2, 3, 6, 1, 2, 6, 2, 3, 6, 3, 6, 3, 6, 1, 5, 5, 5, 5, 5, 1, 1, 1, 1, 5, 1, 1, 5, 1, 5, 5, 5, 1, 5, 5, 5, 1, 1, 5, 1, 1, 1, 1, 1, 5, 1, 5, 1, 5, 2, 2, 2, 4, 2, 2, 2, 2, 4, 4, 1, 4, 1, 4, 2, 1, 1, 2, 2, 1, 4, 4, 2, 2, 1, 1, 4, 2, 2, 1, 4, 1, 2, 2, 4, 2, 1, 4, 4, 1, 2, 4, 4, 2, 4, 4, 2, 4, 4, 4, 2, 2, 1, 2, 2, 1, 2, 2, 1, 2, 1, 4, 4, 1, 2, 4, 2, 2, 1, 4, 4, 2, 4, 4, 2, 4, 2, 4, 4, 1, 1, 3, 3, 1, 3, 1, 3, 1, 1, 3, 1, 3, 3, 1, 1, 1, 1, 1, 1, 3, 3, 3, 3, 3, 1, 3, 3, 1, 1, 3, 3, 3, 3, 1, 1, 3, 3, 3, 1, 3, 1, 1, 3, 1, 3, 3, 1, 1, 3, 3, 1, 3, 3, 3, 3, 3, 3, 3, 1, 1, 3, 3, 3, 1, 1, 1, 3, 1, 3, 1, 1, 1, 3, 3, 1, 3, 2, 2, 1, 2, 2, 1, 1, 2, 1, 1, 2, 2, 2, 1, 1, 2, 1, 2, 1, 1, 2, 1, 2, 1, 1, 2, 2, 2, 1, 2, 2, 2, 2, 2, 2, 2, 1, 1, 1, 1, 2, 2, 1, 1, 1, 2, 1, 2, 1, 1, 2, 2, 1, 2, 2, 2, 1, 1, 1, 2, 2, 1, 2, 1, 2, 2, 2, 1, 1, 1, 1, 2, 2, 1, 2, 2, 1, 2, 2, 2, 2, 2, 1, 2, 2, 1, 1, 1, 1, 1, 2, 2, 1, 1, 1, 2, 2, 2, 1, 2, 2, 2, 2, 1, 2, 1, 2, 1, 1, 2, 1, 1, 1, 1, 1, 1, 2, 2, 1, 1, 2, 2, 1, 2, 2, 1, 2, 2, 2, 1, 2, 2, 1, 1, 1, 2, 1, 1, 2, 2, 2, 2, 1, 2, 1, 2, 1, 1, 2, 1, 2, 2, 2, 2, 1

Gene ontology terms ranked according to mean of effect sizes in cluster:
ribosome | ribosome biogenesis | translational initiation | polysome | translational elongation | polyamine biosynthetic process | eukaryotic translation initiation factor 2 complex | nuclear-transcribed mRNA catabolic process, nonsense-mediated decay | nucleocytoplasmic transport | ribosome binding | mRNA binding | mRNA transport | polyamine metabolic process | ribosomal subunit | small ribosomal subunit | rRNA processing | mitochondrial DNA replication | translation | regulation of translation | RNA biosynthetic process | ribosome assembly | nucleic acid binding | mRNA catabolic process | leaf senescence | RNA transport | RNA catabolic process | eukaryotic translation initiation factor 3 complex | rRNA transcription | nucleolus organizer region

Corresponding mean of effect sizes of each Gene ontology term:
(In the same order as above)
1.94, 1.68, 1.47, 1.45, 1.27, 1.26, 1.23, 1.21, 1.20, 1.20, 1.16, 1.12, 1.11, 1.07, 1.03, 1.02, 0.99, 0.93, 0.88, 0.87, 0.77, 0.74, 0.71, 0.64, 0.57, 0.56, 0.55, 0.44, 0.08

Corresponding total number of genes of each Gene ontology term:
(In the same order as above)

335, 166, 449, 88, 109, 57, 93, 81, 84, 106, 106, 64, 32, 131, 53, 67, 50, 1.1K, 82, 176, 61, 94, 165, 37, 36, 50, 132, 33, 29

Corresponding number of genes of each Gene ontology term with at least one TFBS (of selected TFs):
(In the same order as above)
167, 97, 212, 45, 56, 28, 46, 41, 46, 57, 59, 31, 9, 65, 29, 32, 27, 494, 40, 76, 33, 50, 79, 14, 19, 20, 67, 16, 13

Corresponding total number of TFBS (of selected TFs) for each Gene ontology term (in all genes):
(In the same order as above)
502, 296, 677, 146, 205, 89, 187, 124, 137, 179, 200, 104, 42, 194, 95, 92, 79, 1467, 137, 250, 107, 166, 234, 50, 68, 72, 202, 48, 38

Corresponding number of selected TFs each Gene ontology term is involved with:
(In the same order as above)
20, 20, 20, 20, 20, 20, 20, 20, 20, 20, 20, 20, 19, 20, 20, 20, 20, 20, 20, 20, 20, 20, 20, 19, 20, 20, 20, 20, 19

TFs ranked according to mean of effect sizes in cluster:
V$GABP_B, V$ELK1_02, VUSF_Q6_01, VUSF_Q6, V$MYCMAX_03, V$HLF_01, V$YY1_Q6_02, V$CLOCKBMAL_Q6, V$E4BP4_01, V$YY1_Q6, VUSF_01, VMAX_01, V$MYCMAX_02, V$MYC_Q2, V$ARNT_01, V$USF_02, V$YY1_02, V$TAXCREB_01, V$NFMUE1_Q6, V$ATF6_01

Corresponding mean of effect sizes of each TF:
(In the same order as above)
1.74, 1.69, 1.45, 1.35, 1.34, 1.31, 1.20, 1.20, 1.13, 1.12, 1.09, 1.02, 0.94, 0.80, 0.65, 0.62, 0.53, 0.46, 0.38, 0.07

Corresponding total number of TFBS for each TF (genome-wide):
(In the same order as above)
625, 627, 664, 672, 650, 657, 688, 679, 635, 684, 647, 647, 694, 676, 655, 649, 639, 674, 658, 670

Corresponding total number of TFBS for each TF (in all genes in selected Gene ontology terms):
(In the same order as above)
333, 310, 325, 328, 321, 362, 342, 340, 334, 325, 318, 312, 319, 291, 286, 266, 287, 289, 250, 249

Corresponding number of genes (of selected Gene ontology terms) each TF is involved with:
(In the same order as above)
121, 113, 115, 118, 121, 133, 127, 119, 122, 114, 118, 120, 121, 109, 109, 106, 107, 114, 103, 102

Corresponding number of selected Gene ontology terms each TF is involved with:
(In the same order as above)
29, 29, 29, 29, 29, 29, 29, 29, 29, 29, 29, 28, 29, 29, 28, 29, 29, 29, 28, 29

Cluster for columns 7 to 11, rows 143 to 151

Gene ontology terms

cell activation | T cell activation | T cell receptor complex | immunological synapse | integrin activation

TFs

V$PEA3_Q6 | V$ETS_Q6 | V$PU1_Q6 | V$PXR_Q2 | V$TEL2_Q6 | V$ETS_Q4 | V$ELF1_Q6 | V$NKX25_Q5 | V$ETS2_B

Information

All related TFs:
(List of all TFs that are related to any of the PWMs)
CAR, CAR2:RXR-alpha, CAR:RXR-alpha, ELF-1, ELFR, ERF, Elf-1, Elk-1, Elk-1-isoform1, Erg-1, Erg-2, Ets-1, FXR, FXR:RXR-alpha, Fli-1, GABP-alpha, GABP-alpha:GABP-beta, GABP-beta1, GABP-beta2, LXR-alpha:RXR-alpha, LXR-beta:RXR-alpha, NERF, NERF-1a, NERF-1b, NERF-2, Net, Nkx2-5, Nkx2.5, PEA3, PU.1, PU.1-xbb1, PXR-1, PXR-1A, PXR-2, PXR:RXR-alpha, SAP-1a, SAP-1b, Spi-B, TCF, TEL1, Tel-2a, Tel-2b, Tel-2c, Tel-2d, Tel-2e, Tel-2f, c-Ets-1, c-Ets-1A, c-Ets-1B, c-Ets-2, c-Ets-2A, c-Ets-2B, p38erg, p49erg, p55, p55erg

Ranked gene list:
(All genes of the selected Gene ontology terms with hits of any of the selected TFs, ranked according to the total number of TFBS)
PLCG1, LCK, CCR7, AKT1, PTPN6, CSK, ITGB2, ZAP70, LCP2, VAV1, TNF, SHC1, PER1, NFATC1, LTB, LAG3, ITGA5, CISH, SKAP1, SH2B3, PTPN7, LCP1, JUNB, TNFRSF1A, TCIRG1, TCF7, CD4, BCL11B, FASN, EDC4, CXCR4, RASSF5, DUSP2, CD68, TYROBP, TNFRSF18, PTK2B, PSTPIP1, LTBR, HCST, GFI1, EGR1, CD82, CD2BP2, CCND3, TNFSF13, TLR9, SOCS1, MAP4K1, LTA, FYN, CD63, ZBTB7B, TNFRSF4, TBX21, TBC1D10C, NFKBIA, MEF2D, IRF1, GATA3, DGKA, CD3G, CD19, SSSCA1, SH3BP2, SATB1, PIK3CG, ISG20, IL16, DKFZp434P0672, ARHGEF2, ANXA6, XAB2, SIPA1, RAC2, PPP1R9B, MYO1G, ITGB7, ILK, CD79B, STAT5A, SH2D2A, RAP1GAP, PTPN11, PKN1, NFATC3, FERMT3, FAS, EFNA2, CD53, CD3D, BHLHB2, BCL6, SOCS3, SLC2A4RG, RASGRP2, PRDM1, NR4A1, ITPR3, INPP5D, GPR132, CSF1, CD37, WIPF1, WASF2, VAV2, TNFRSF6B, TNFRSF1B, STIM1, STAT6, SPN, SELL, RAF1, POU2F2, POLD4, PIK3R1, NCK1, MAPK14, LYL1, KCNA3, ITGB1, ITGA4, IKZF1, ICAM3, ICAM2, HMHA1, GIMAP5, EXTL3, DOK2, CXCR5, CTLA4, CASP8, CAP1, CALR, CALM2, ALCAM, VAV3, TFRC, TAPBP, TAP1, STAT5B, STAT4, SRF, SLC19A2, SEMA4D, SELPLG, PIK3CD, NFATC2, MBP, MAP3K1, LEF1, JUN, JAK3, IL2RB, ID3, GRB2, ETS1, DIABLO, DEF6, DAPP1, CRK, CDKN1B, CD5, CD44, CD27, CD247, VEGFA, TSPAN4, SMAD3, SILV, RHOA, RAP1B, PSMB9, PRIM2, PRDX5, PIK3R2, PHLDA1, PEA15, PDLIM7, PDE4A, ORAI1, NR1H2, NCR3, MYB, MR1, MARCKSL1, KCNN4, ISYNA1, IL7R, IL23A, IL21R, HRAS, HNRNPL, EPHA1, EGR2, DPP8, DOCK2, CREB1, CD74, CD180, CANX, BTLA, ARHGEF7, WDR48, UNC13D, TRAF1, TNFRSF25, TGFA, TCF12, STRA13, STAT3, SQSTM1, RNF114, RERE, RAB3GAP1, PXN, PLEKHA2, NFKB1, MYD88, MTHFR, LYN, LAMP1, KIAA0101, KAT5, JUND, ITPR1, IL18R1, ICAM5, GUK1, GPR172B, FKBP1A, ERCC6, DNTT, CDH15, CD3E, CBLB, BTN3A1, BSG, AIF1

Corresponding total number of TFBS:
(For each gene listed above, the total number of TFBS for any of the selected TFs, multiplied by the number of selected Gene ontology terms containing that gene)
40, 40, 40, 40, 36, 36, 35, 32, 30, 25, 24, 24, 24, 24, 24, 24, 24, 24, 20, 18, 18, 18, 18, 16, 16, 16, 16, 16, 15, 15, 15, 14, 14, 14, 12, 12, 12, 12, 12, 12, 12, 12, 12, 12, 12, 10, 10, 10, 10, 10, 10, 10, 9, 9, 9, 9, 9, 9, 9, 9, 9, 9, 9, 8, 8, 8, 8, 8, 8, 8, 8, 8, 7, 7, 7, 7, 7, 7, 7, 7, 6, 6, 6, 6, 6, 6, 6, 6, 6, 6, 6, 6, 6, 5, 5, 5, 5, 5, 5, 5, 5, 5, 5, 4, 4, 4, 4, 4, 4, 4, 4, 4, 4, 4, 4, 4, 4, 4, 4, 4, 4, 4, 4, 4, 4, 4, 4, 4, 4, 4, 4, 4, 4, 4, 4, 3, 3, 3, 3, 3, 3, 3, 3, 3, 3, 3, 3, 3, 3, 3, 3, 3, 3, 3, 3, 3, 3, 3, 3, 3, 3, 3, 3, 3, 2, 2, 2, 2, 2, 2, 2, 2, 2, 2, 2, 2, 2, 2, 2, 2, 2, 2, 2, 2, 2, 2, 2, 2, 2, 2, 2, 2, 2, 2, 2, 2, 2, 2, 2, 2, 2, 2, 2, 2, 1, 1, 1, 1, 1, 1, 1, 1, 1, 1, 1, 1, 1, 1, 1, 1, 1, 1, 1, 1, 1, 1, 1, 1, 1, 1, 1, 1, 1, 1, 1, 1, 1, 1, 1, 1, 1, 1, 1, 1

Corresponding number of selected Gene ontology terms each gene is involved with:
(In the same order as above)
5, 5, 5, 5, 4, 4, 5, 4, 5, 5, 3, 3, 3, 3, 3, 3, 4, 3, 4, 2, 2, 3, 2, 2, 2, 2, 2, 4, 2, 3, 3, 5, 2, 2, 2, 2, 3, 4, 2, 4, 2, 2, 2,

2, 4, 2, 5, 2, 2, 2, 2, 5, 2, 1, 3, 3, 1, 3, 1, 1, 1, 1, 3, 3, 1, 1, 2, 4, 4, 2, 2, 1, 2, 1, 1, 1, 1, 1, 1, 1, 1, 3, 3, 1, 2, 1, 1, 1, 3, 2, 2, 2, 1, 1, 1, 1, 1, 1, 1, 1, 1, 1, 1, 2, 4, 2, 1, 2, 2, 2, 4, 4, 4, 1, 1, 2, 4, 4, 1, 4, 4, 4, 1, 2, 2, 1, 1, 2, 1, 2, 4, 2, 1, 1, 2, 2, 3, 3, 1, 1, 3, 3, 1, 3, 3, 1, 3, 3, 3, 1, 1, 3, 3, 3, 1, 3, 3, 1, 1, 1, 3, 1, 3, 3, 3, 3, 1, 2, 1, 1, 2, 1, 1, 1, 1, 2, 2, 1, 1, 1, 1, 1, 1, 2, 2, 1, 2, 1, 2, 2, 1, 2, 1, 1, 2, 1, 2, 2, 1, 1, 1, 2, 2, 1, 1, 1, 1, 1, 1, 1, 1, 1, 1, 1, 1, 1, 1, 1, 1, 1, 1, 1, 1, 1, 1, 1, 1, 1, 1, 1, 1, 1, 1, 1, 1, 1, 1

Corresponding number of selected TFs each gene is involved with:
(In the same order as above)
8, 8, 8, 8, 9, 9, 7, 8, 6, 5, 8, 8, 8, 8, 8, 8, 6, 8, 5, 9, 9, 6, 9, 8, 8, 8, 4, 8, 5, 5, 3, 7, 7, 7, 6, 4, 3, 6, 3, 6, 6, 6, 6, 3, 6, 2, 5, 5, 5, 5, 2, 5, 9, 3, 3, 9, 3, 9, 9, 9, 9, 3, 3, 8, 8, 4, 2, 2, 4, 4, 8, 4, 7, 7, 7, 7, 7, 7, 7, 7, 2, 2, 6, 3, 6, 6, 6, 2, 3, 3, 3, 6, 6, 5, 5, 5, 5, 5, 5, 5, 5, 5, 5, 2, 1, 2, 4, 2, 2, 2, 1, 1, 1, 4, 4, 2, 1, 1, 4, 1, 1, 1, 4, 2, 2, 4, 4, 2, 4, 2, 1, 2, 4, 4, 2, 2, 1, 1, 3, 3, 1, 1, 3, 1, 1, 3, 1, 1, 1, 3, 3, 1, 1, 1, 3, 1, 1, 3, 3, 3, 1, 3, 1, 1, 1, 1, 2, 1, 2, 2, 1, 2, 2, 2, 2, 1, 1, 2, 2, 2, 2, 2, 2, 1, 1, 2, 1, 2, 1, 1, 2, 1, 2, 2, 1, 2, 1, 1, 2, 2, 2, 1, 1, 1, 1, 1, 1, 1, 1, 1, 1, 1, 1, 1, 1, 1, 1, 1, 1, 1, 1, 1, 1, 1, 1, 1, 1, 1, 1, 1, 1, 1, 1, 1, 1

Gene ontology terms ranked according to mean of effect sizes in cluster:
cell activation | integrin activation | T cell receptor complex | immunological synapse | T cell activation

Corresponding mean of effect sizes of each Gene ontology term:
(In the same order as above)
2.57, 2.46, 2.33, 2.11, 1.82

Corresponding total number of genes of each Gene ontology term:
(In the same order as above)
596, 155, 478, 112, 287

Corresponding number of genes of each Gene ontology term with at least one TFBS (of selected TFs):
(In the same order as above)
163, 48, 136, 41, 88

Corresponding total number of TFBS (of selected TFs) for each Gene ontology term (in all genes):
(In the same order as above)
565, 181, 485, 156, 307

Corresponding number of selected TFs each Gene ontology term is involved with:
(In the same order as above)
9, 9, 9, 9, 9

TFs ranked according to mean of effect sizes in cluster:
V$ETS2_B, V$ETS_Q6, V$ELF1_Q6, V$ETS_Q4, V$PEA3_Q6, V$TEL2_Q6, V$PU1_Q6, V$NKX25_Q5, V$PXR_Q2

Corresponding mean of effect sizes of each TF:
(In the same order as above)
2.56, 2.56, 2.56, 2.54, 2.42, 2.40, 2.15, 1.91, 1.21

Corresponding total number of TFBS for each TF (genome-wide):
(In the same order as above)
722, 721, 717, 718, 717, 707, 724, 695, 684

Corresponding total number of TFBS for each TF (in all genes in selected Gene ontology terms):
(In the same order as above)
200, 190, 202, 199, 189, 187, 190, 172, 165

Corresponding number of genes (of selected Gene ontology terms) each TF is involved with:

(In the same order as above)
95, 93, 96, 95, 89, 91, 91, 91, 85

Corresponding number of selected Gene ontology terms each TF is involved with:
(In the same order as above)
5, 5, 5, 5, 5, 5, 5, 5, 5

Cluster for columns 1295 to 1311, rows 423 to 434

Gene ontology terms

Notch signaling pathway | gene expression | DBD domain binding | DNA binding | chordate embryonic development | embryonic development | limb development | central nervous system development | central nervous system neuron development | gastrulation | somitogenesis | anatomical structure morphogenesis | organ development | neurogenesis | regionalization | cell fate determination | segmentation

TFs

V$E2F_Q6_01 | V$E2F_Q4_01 | V$E2F_Q3_01 | V$E2F1_Q4_01 | V$E2F1_Q6_01 | V$E2F_03 | V$E2F4DP2_01 | V$E2F1DP2_01 | V$E2F1DP1_01 | V$E2F_Q6 | V$E2F_Q4 | V$E2F_Q3

Information

All related TFs:
(List of all TFs that are related to any of the PWMs)
DP-1, E2F, E2F+E4, E2F-1, E2F-1:DP-1, E2F-1:DP-2, E2F-3a, E2F-4, E2F-4:DP-2, E2F-7

Ranked gene list:
(All genes of the selected Gene ontology terms with hits of any of the selected TFs, ranked according to the total number of TFBS)
PAX6, HES1, WNT1, LEF1, LHX1, HES5, PCNA, LBX1, NKX2-2, HMX3, JUN, HOXA9, CDKN1A, TFAP2A, MEIS2, DLL1, ID2, EOMES, HOXD13, TP73, HOXA7, AKT1, SLC20A1, RBL1, LFNG, HOXA10, FGF9, CCND1, RUNX2, MYCN, CDK5, ID3, TLE3, NFATC1, EVX2, NR4A2, TCF3, HMX2, GATA3, FLII, E2F1, CDKN1B, NR2F1, NEUROD1, FLI1, ZIC2, WNT10B, OTX1, MYB, WNT2B, UNCX, TCF7, ONECUT1, HMGA1, HAND2, GTF2A1, GATA2, EGR2, SIN3A, PTCH1, NR3C2, MCM7, MCM2, LHX9, HIST1H4I, GFI1, FOXO3, FOXF1, E2F3, CCND2, PDGFRA, IRX4, FLJ32987, CORO7, SMAD7, NFIB, IRX5, FOXD3, EZH2, EGR1, DLX2, MDK, PROX1, NOL3, MDM2, HOXD11, DLX5, BRD2, RBPJ, PTPRU, CTCF, WNT11, ETV4, EFNB2, EFNA5, DLL4, ZFP36L2, TP53, SOX8, SMARCA4, RXRB, POU3F2, PELP1, MNT, MAZ, JUNB, IRF4, IRF1, HOXB7, HOXA11, HIST4H4, HES3, H2AFZ, FUS, E2F2, DPYSL2, DAAM1, CBX4, C2orf28, BUB3, BMI1, BCL2L11, ZNF274, ATF3, ARID3A, VEGFB, SHC1, PIN1, MORF4L1, EGR3, SOCS3, SMAD6, PEG3, NOLC1, NEUROG2, NAB2, HES7, FOXC1, CENPB, ARNTL, ZBTB7B, VIM, UBTF, TRADD, TLE4, TCF25, STAT3, SNAPC2, SAMD4A, RPA2, RB1, POU4F1, PAX5, NRARP, MXD3, MKX, IGF1R, CDC25A, STAU1, SLC7A1, SKP2, SEMA3B, RBBP4, NCL, HSPA2, HOXC9, HOXB5, HIVEP1, DVL2, DMRT1, CUX1, CNOT1, CDKN2C, CDC6, SKI, REL, NR3C1, NKX3-2, MXI1, MMP11, FOSB, CDKN1C, CCNB1IP1, AGRN, WHSC1, SSBP3, PURA, PGD, MSL1, HEY2, HCFC1, DBP, CXCR4, BACH1, NOTCH1, CTNNB1, VPRBP, TIAM1, TCF7L1, SLC39A1, SFMBT2, RASSF1, PTMA, PNPLA2, PIK3CA, OSR1, NFYA, MIP, MCM6, MAP3K7, LRDD, KREMEN2, JUND, IRS2, HOXD12, HOXB6, HIF1A, HHEX, GAMT, FZD8, FGF8, FASTK, DPH1, DLX4, CRTC2, CKB, CDK5R1, CD151, CBX3, CAD, BCOR, BCL6, APRT, ADM, SPHK1, SLC4A2, SIAH1, RRM2, RPL8, KISS1R, GPS2, GLI1, GEMIN4, ERBB2IP, CREBZF, CDC25B, ZNF260, TYMP, TIPIN, SP1, SATB2, RPS2, RING1, RFC1, POU4F3, POLRMT, NR2E1, MBTPS1, LEPREL2, KLF13, IRS1, IKZF1, IER2, HSPE1, HSPD1, HNRNPA1, HMGB2, HIC1, GSK3A, FOXN2, FASN, EIF5A, DMTF1, CRIP1, CIRBP,

CBX5, CAMK2G, BCL11B, ARHGEF2, ZNF362, VDR, TFDP1, TBX2, SUMO1, SRPR, RARA, PSMC5, PCSK4, PAICS, NR4A1, NFE2L2, NFATC3, LPHN1, KLF1, KCNH4, IFNAR1, HCFC1R1, EFNA3, EBF2, DOCK10, DAG1, CTF1, CSK, CRIP2, CDK6, ATP6V0D1, ANP32A, ZFP36L1, XPC, VAV2, UBB, TUSC2, TUBA1B, TNFRSF12A, TEAD1, SMARCA2, SFRS2, REXO1, REV3L, RAC1, PTCHD2, PTBP1, PSMD1, PSENEN, PPRC1, PAX9, OSR2, NFKB2, NFIA, MYC, MGA, MAP4K1, LRBA, LMOD1, LIG4, KLF9, JMJD3, ILF3, HOXD10, HOXC8, HOXB8, HNRNPF, HNRNPD, H3F3B, H2AFJ, GIT1, GBX2, GAD1, FUSIP1, FOXA1, FGFRL1, FBXO5, EPAS1, DNMT3A, CPT1A, COL1A1, CFL1, CEP57, CD68, BARX1, BACH2, ATP5B, ANXA4, ADAM9, ACTB, WNT5A, STMN1, SHMT2, RASD1, PHLDB1, PEG10, PDHX, PCGF2, PBXIP1, LRP6, IKZF3, IHH, HOXC10, HOXA6, HMBS, HELLS, GAPDH, FAM105A, DUSP4, CLDN6, CALM2, ARID4A, ZFP36, ZEB1, YY1, VTA1, VGF, TRIM26, TRERF1, TGFB3, TBX15, STAT1, SRCAP, SOX15, SNF1LK2, SFN, SART3, RNF4, RARG, PSMD4, PRKDC, POLR2A, PLEC1, PLAGL1, PHF20, PGP, PER2, NUP43, NPM1, NNAT, NME2, NME1-NME2, NFIC, MLL, MFNG, MEIS1, MEF2D, MEF2C, MARCKSL1, LRP8, LONP1, KLF5, ISG15, IRF8, GTF2F1, GMNN, FOSL2, EHMT2, DHFR, DEK, DCK, CUL4A, CELSR3, CDK10, CDC25C, CCNF, CCNE1, CASP2, C7orf23, APP, ALDOA, ZNF384, YWHAQ, UNG, TMPO, TCP1, SLC31A1, SALL3, S1PR1, PXMP2, PRKCE, NR2C2, NLE1, MYNN, KCNIP3, JARID2, HOXA5, HIVEP2, HDLBP, GSPT1, FOXM1, FHOD1, FBXL20, EFNA2, ECE1, DRAP1, DPYSL5, DENND4A, DAXX, CD47, CAST, BRMS1L, BARHL1, ANXA5, ACTG1, ZHX2, ZFYVE9, ZBTB16, UQCRH, TGFBRAP1, TCF21, TADA2L, TACR1, SSSCA1, SP5, SOCS1, SNF1LK, RPL19, RAB8A, PRPF40A, POLE4, PER1, PCDH8, PBX3, OAZ1, NTRK3, NR1H2, NPDC1, NFATC4, NCOR1, NAB1, MYL6, MTHFR, MSL2, MSH2, MPP6, MLL2, MFSD11, MBD1, MAT2A, LRP5, LLGL2, KRT8, IRX3, IGF2, HYAL2, HNRNPC, HDAC7, HCRT, GSK3B, G3BP1, ENO1, EN2, DUSP10, DUSP1, DMC1, CTBP1, COBRA1, CDC5L, CDC2, CCR7, CASP7, CASP6, C5orf13, BTG1, BSG, BMF, ATP1A1, ASXL2, ACO2, ACACA, YBX2, WNK1, UGP2, UBE2I, TRRAP, TRAF3, TPT1, TPK1, TCF12, TAGLN2, SYNCRIP, SNRPB, SND1, SLC3A2, SLC38A2, SLC2A4RG, SFRS2IP, RRM1, RAD51, PTPLAD1, PRKCZ, PPIA, PKD2, PFKFB3, PCID2, NMB, NDRG1, MTR, MSH3, MGAT1, MAPK12, MAL, LGALS1, KEAP1, INSIG2, IGF2BP3, IDI1, HSPB11, HOXA3, HIST1H1A, HIRA, GUK1, GSS, GRIN2C, GPX4, GCDH, GBA, FOXL2, FBRS, FAM110A, ETV5, ENO3, EFNA1, EEF1E1, DPYD, DHX9, DGAT2, CYFIP2, CYCS, CTSW, CTSD, CLK4, CLIC4, CDKN2D, CDK8, CAMTA1, CALM1, CACNB3, CACNA1G, CABIN1, BNIP3, BANP, ARFRP1, APLP2, ALLC, AKT2, AK2, AGRP, AGPAT1, AGER, ADORA2A, ACHE, ZNF76, ZBTB7A, XRCC5, WBP7, TUFT1, TNFRSF19, TMSB10, TCOF1, SUPT3H, STRA13, ST3GAL4, SRD5A1, SPON2, SPEN, SON, SNAPC5, SMARCB1, SLCO3A1, SLC39A4, SLC25A22, SIX5, SEMA3F, RPL28, RAD54L, PSMB8, PRKG1, PRDM16, PPOX, PPARGC1B, PMP22, PKMYT1, PBRM1, MIB1, MAFA, LPAR1, LIN9, KLF2, KLF10, JAG2, ITM2C, ISYNA1, ING3, IFIH1, HRAS, HMOX2, HLTF, GPD2, GINS2, FMN2, FAR1, FAM129A, FAF1, ELF2, EIF4A1, EGR4, DUSP6, DLG4, DKFZp779C0757, DDB2, DAND5, DAD1, CUL1, CSRP1, COX8A, CNTFR, CHRNE, CHEK1, CD46, CCNE2, CBLN3, CBFA2T3, CAPNS1, CALU, BTAF1, BCL2L1, BBC3, ARID1A, ARHGAP1, ANG, ADAMTS4, ACAA2, ABCF3, ZNF146, ZIC4, ZIC3, ZFP161, YME1L1, WDR68, TUBB, TSNAX, TRPC1, TIAL1, SPRY4, SLC7A3, SLC40A1, SLC37A4, SLC25A36, SIVA1, SHOX2, SERPINH1, SCD, SALL1, RPS18, RPE, RHOC, RGMB, RCOR2, RCAN1, RBX1, RBM15, PRMT1, PPP5C, PPP3CA, PPIG, PMS1, PIGQ, PGLYRP1, PDPK1, PCYT2, PCDH21, PAPD1, NRN1, NPTX1, NIN, NID2, MX1, MT1F, MLH1, MKL2, MAP3K12, LDHA, KLF12, ITPR1, ING1, IMMT, IFT74, HSPH1, HSP90B1, HIPK3, HAT1, FSCN1, FKBP5, EZH1, ERRFI1, ENO2, ENAH, EFNA4, EEF1A1, EAF2, DOPEY1, DMXL2, DICER1, DDX39, CTBP2, CSTF2T, CSNK2A1, CLK1, CHD7, CENPK, CDC42EP4, CAND1, CADM1, BRAF, ATP6V0C, ATP2A2, ANKH, ALOX5, ALCAM, ADAMTS2, ACTR6, ACTR5, ABCD3, ABCB1

Corresponding total number of TFBS:
(For each gene listed above, the total number of TFBS for any of the selected TFs, multiplied by the number of selected Gene ontology terms containing that gene)
132, 132, 121, 110, 108, 99, 96, 96, 84, 84, 80, 80, 80, 77, 77, 77, 72, 72, 70, 66, 63, 63, 60, 60, 60, 60, 60, 60, 56, 56, 55, 54, 50, 50, 50, 49, 48, 48, 48, 48, 48, 48, 45, 45, 45, 44, 44, 42, 42, 40, 40, 40, 40, 40, 40, 40, 40, 40, 36, 36, 36, 36, 36, 36, 36, 36, 36, 36, 36, 36, 35, 33, 33, 33, 32, 32, 32, 32, 32, 32, 32, 30, 28, 28, 28, 28, 28, 28, 27, 27, 27, 25, 25, 25, 25, 25, 24, 24, 24, 24, 24, 24, 24, 24, 24, 24, 24, 24, 24, 24, 24, 24, 24, 24, 24, 24, 24, 24, 24, 24, 24, 24, 22, 22, 22, 21, 21, 21, 21, 21, 20, 20, 20, 20, 20, 20, 20, 20, 20, 20, 18, 18, 18, 18, 18, 18, 18, 18, 18, 18, 18, 18, 18, 18, 18, 18, 18, 18, 16, 16, 16, 16, 16, 16, 16, 16,

16, 16, 16, 16, 16, 16, 16, 16, 15, 15, 15, 15, 15, 15, 15, 15, 15, 15, 14, 14, 14, 14, 14, 14, 14, 14, 14, 14, 13, 13, 12, 12, 12, 12, 12, 12, 12, 12, 12, 12, 12, 12, 12, 12, 12, 12, 12, 12, 12, 12, 12, 12, 12, 12, 12, 12, 12, 12, 12, 12, 12, 12, 12, 12, 12, 12, 12, 12, 11, 11, 11, 11, 11, 11, 11, 11, 11, 11, 11, 11, 10, 10, 10, 10, 10, 10, 10, 10, 10, 10, 10, 10, 10, 10, 10, 10, 10, 10, 10, 10, 10, 10, 10, 10, 10, 10, 10, 10, 10, 10, 10, 10, 10, 9, 9, 9, 9, 9, 9, 9, 9, 9, 9, 9, 9, 9, 9, 9, 9, 9, 9, 9, 9, 9, 9, 9, 9, 9, 9, 9, 8, 8, 8, 8, 8, 8, 8, 8, 8, 8, 8, 8, 8, 8, 8, 8, 8, 8, 8, 8, 8, 8, 8, 8, 8, 8, 8, 8, 8, 8, 8, 8, 8, 8, 8, 8, 8, 8, 8, 8, 8, 8, 8, 8, 8, 8, 8, 8, 8, 8, 8, 8, 8, 8, 8, 8, 8, 8, 8, 7, 7, 7, 7, 7, 7, 7, 7, 7, 7, 7, 7, 7, 7, 7, 7, 7, 7, 7, 7, 7, 7, 6, 6, 6, 6, 6, 6, 6, 6, 6, 6, 6, 6, 6, 6, 6, 6, 6, 6, 6, 6, 6, 6, 6, 6, 6, 6, 6, 6, 6, 6, 6, 6, 6, 6, 6, 6, 6, 6, 6, 6, 6, 6, 6, 6, 6, 6, 6, 6, 6, 6, 6, 6, 6, 6, 6, 6, 6, 6, 6, 6, 6, 6, 6, 6, 5, 5, 5, 5, 5, 5, 5, 5, 5, 5, 5, 5, 5, 5, 5, 5, 5, 5, 5, 5, 5, 5, 5, 5, 5, 5, 5, 5, 5, 5, 5, 5, 5, 5, 4, 4, 4, 4, 4, 4, 4, 4, 4, 4, 4, 4, 4, 4, 4, 4, 4, 4, 4, 4, 4, 4, 4, 4, 4, 4, 4, 4, 4, 4, 4, 4, 4, 4, 4, 4, 4, 4, 4, 4, 4, 4, 4, 4, 4, 4, 4, 4, 4, 4, 4, 4, 4, 4, 4, 4, 4, 4, 4, 4, 4, 4, 4, 3, 3, 3, 3, 3, 3, 3, 3, 3, 3, 3, 3, 3, 3, 3, 3, 3, 3, 3, 3, 3, 3, 3, 3, 3, 3, 3, 3, 3, 3, 3, 3, 3, 3, 3, 3, 3, 3, 3, 3, 3, 3, 3, 3, 3, 3, 3, 3, 3, 3, 3, 3, 3, 3, 3, 3, 3, 3, 3, 3, 3, 3, 3, 3, 3, 3, 3, 3, 3, 3, 3, 3, 3, 3, 3, 3, 3, 3, 2, 2, 2, 2, 2, 2, 2, 2, 2, 2, 2, 2, 2, 2, 2, 2, 2, 2, 2, 2, 2, 2, 2, 2, 2, 2, 2, 2, 2, 2, 2, 2, 2, 2, 2, 2, 2, 2, 2, 2, 2, 2, 2, 2, 2, 2, 2, 2, 2, 2, 2, 2, 2, 2, 2, 2, 2, 2, 2, 2, 2, 2, 2, 2, 2, 2, 2, 2, 2, 2, 2, 2, 2, 2, 1, 1, 1, 1, 1, 1, 1, 1, 1, 1, 1, 1, 1, 1, 1, 1, 1, 1, 1, 1, 1, 1, 1, 1, 1, 1, 1, 1, 1, 1, 1, 1, 1, 1, 1, 1, 1, 1, 1, 1, 1, 1, 1, 1, 1, 1, 1, 1, 1, 1, 1, 1, 1, 1, 1, 1, 1, 1, 1, 1, 1, 1, 1, 1, 1, 1, 1, 1, 1, 1, 1, 1, 1, 1, 1, 1, 1, 1, 1, 1, 1, 1, 1, 1

Corresponding number of selected Gene ontology terms each gene is involved with:
(In the same order as above)
11, 11, 11, 11, 9, 11, 8, 16, 7, 7, 10, 8, 8, 7, 7, 11, 8, 6, 7, 6, 7, 7, 6, 5, 10, 6, 5, 6, 7, 7, 5, 6, 5, 5, 5, 7, 8, 4, 4, 6, 6, 4, 9, 9, 5, 4, 4, 7, 6, 5, 4, 4, 8, 4, 10, 4, 4, 8, 3, 6, 3, 3, 3, 3, 3, 3, 3, 6, 3, 4, 5, 3, 3, 3, 4, 4, 4, 4, 4, 4, 8, 6, 7, 7, 4, 4, 7, 4, 9, 3, 3, 5, 5, 5, 5, 5, 3, 6, 3, 6, 3, 3, 3, 2, 2, 3, 2, 3, 6, 6, 3, 3, 2, 3, 2, 2, 4, 2, 2, 2, 3, 2, 2, 2, 2, 3, 3, 3, 3, 3, 2, 5, 2, 5, 5, 2, 5, 4, 2, 2, 2, 6, 2, 2, 6, 2, 6, 2, 3, 2, 6, 6, 2, 3, 2, 2, 3, 3, 4, 4, 2, 4, 2, 2, 2, 2, 4, 2, 2, 4, 2, 2, 2, 2, 3, 5, 5, 3, 3, 5, 3, 5, 5, 5, 2, 2, 2, 2, 2, 7, 2, 2, 7, 2, 13, 13, 3, 1, 6, 1, 1, 1, 1, 1, 4, 2, 3, 4, 1, 4, 1, 1, 2, 1, 3, 4, 4, 4, 1, 2, 12, 1, 2, 4, 1, 1, 6, 1, 1, 1, 1, 1, 1, 1, 1, 1, 1, 1, 1, 1, 11, 1, 1, 1, 1, 1, 1, 2, 5, 1, 1, 2, 2, 5, 1, 5, 2, 5, 2, 1, 2, 1, 2, 2, 2, 2, 1, 1, 2, 2, 1, 1, 1, 1, 2, 1, 1, 2, 3, 3, 3, 9, 3, 1, 9, 3, 3, 1, 3, 3, 3, 1, 3, 3, 1, 1, 1, 1, 1, 3, 3, 3, 1, 1, 3, 1, 1, 2, 1, 8, 1, 1, 1, 4, 4, 2, 2, 2, 4, 1, 1, 2, 2, 2, 8, 2, 2, 4, 8, 1, 2, 1, 2, 2, 2, 1, 1, 4, 4, 4, 1, 1, 2, 1, 1, 8, 1, 1, 8, 1, 1, 2, 4, 1, 1, 2, 1, 1, 4, 2, 1, 2, 2, 4, 7, 1, 1, 1, 1, 1, 7, 1, 1, 7, 1, 7, 1, 1, 1, 1, 1, 1, 1, 1, 1, 1, 1, 3, 6, 1, 2, 1, 1, 3, 2, 3, 1, 2, 1, 1, 1, 1, 6, 3, 2, 1, 1, 3, 3, 1, 1, 2, 3, 1, 2, 2, 3, 2, 3, 6, 3, 6, 1, 2, 2, 2, 2, 3, 2, 1, 2, 3, 2, 2, 1, 2, 2, 1, 6, 1, 3, 2, 1, 3, 2, 1, 1, 1, 1, 1, 5, 1, 1, 1, 1, 5, 1, 1, 1, 5, 5, 1, 1, 1, 5, 1, 1, 5, 1, 1, 1, 1, 1, 1, 1, 5, 1, 1, 2, 2, 4, 1, 1, 4, 1, 2, 4, 4, 2, 1, 1, 2, 1, 1, 1, 4, 4, 1, 4, 2, 1, 2, 4, 1, 1, 1, 1, 1, 1, 1, 4, 1, 4, 1, 4, 4, 4, 2, 4, 1, 1, 4, 1, 2, 4, 1, 1, 1, 2, 1, 2, 4, 2, 1, 2, 1, 1, 1, 1, 1, 1, 1, 1, 1, 3, 1, 3, 1, 1, 1, 1, 3, 1, 1, 1, 1, 1, 1, 1, 1, 3, 1, 1, 1, 3, 1, 1, 1, 3, 1, 1, 1, 3, 1, 3, 1, 1, 1, 1, 1, 3, 1, 3, 1, 1, 1, 3, 1, 1, 1, 1, 1, 3, 1, 3, 1, 1, 1, 1, 1, 1, 1, 1, 1, 1, 1, 1, 1, 1, 1, 1, 1, 1, 1, 1, 1, 1, 1, 1, 1, 1, 1, 3, 1, 2, 2, 2, 2, 1, 1, 1, 1, 2, 1, 1, 2, 1, 1, 1, 2, 1, 1, 1, 2, 1, 1, 1, 1, 2, 1, 1, 1, 1, 1, 1, 2, 2, 2, 1, 2, 2, 2, 1, 2, 1, 1, 2, 1, 1, 1, 1, 2, 1, 1, 2, 1, 1, 2, 2, 1, 2, 2, 1, 2, 2, 1, 1, 1, 1, 2, 1, 1, 1, 2, 1, 1, 2, 2, 1, 2, 1, 1, 1, 1, 1, 1, 1, 1, 1, 1, 1, 1, 1, 1, 1, 1, 1, 1, 1, 1, 1, 1, 1, 1, 1, 1, 1, 1, 1, 1, 1, 1, 1, 1, 1, 1, 1, 1, 1, 1, 1, 1, 1, 1, 1, 1, 1, 1, 1, 1, 1, 1, 1, 1, 1, 1, 1, 1, 1, 1, 1, 1, 1, 1, 1, 1, 1, 1, 1, 1, 1, 1, 1, 1, 1, 1, 1, 1, 1, 1, 1, 1, 1, 1, 1, 1, 1, 1, 1, 1, 1

Corresponding number of selected TFs each gene is involved with:
(In the same order as above)
12, 12, 11, 10, 12, 9, 12, 6, 12, 12, 8, 10, 10, 11, 11, 7, 9, 12, 10, 11, 9, 9, 10, 12, 6, 10, 12, 10, 8, 8, 11, 9, 10, 10, 10, 7, 6, 12, 12, 8, 8, 12, 5, 5, 9, 11, 11, 6, 7, 8, 10, 10, 5, 10, 4, 10, 10, 5, 12, 6, 12, 12, 12, 12, 12, 12, 12, 6, 12, 9, 7, 11, 11, 11, 8, 8, 8, 8, 8, 8, 4, 5, 4, 4, 7, 7, 4, 7, 3, 9, 9, 5, 5, 5, 5, 5, 8, 4, 8, 4, 8, 8, 8, 12, 12, 8, 12, 8, 4, 4, 8, 8, 12, 8, 12, 12, 6, 12, 12, 12, 8, 12, 11, 11, 11, 7, 7, 7, 7, 7, 10, 4, 10, 4, 4, 10, 4, 5, 10, 10, 9, 3, 9, 9, 3, 9, 3, 9, 6, 9, 3, 3, 9, 6, 9, 9, 6, 6, 4, 4, 8, 4, 8, 8, 8, 8, 4, 8, 8, 4, 8, 8, 8, 8, 5, 3, 3, 5, 5, 3, 5, 3, 3, 3, 7, 7, 7, 7, 7, 2, 7, 7, 2, 7, 1, 1, 4, 12, 2, 12, 12, 12, 12, 12, 3, 6, 4, 3, 12, 3, 12, 12, 6, 12, 4, 3, 3, 3, 12, 6, 1, 12, 6, 3, 12, 12, 2, 12, 12, 12, 12, 12, 12, 12, 11, 11, 11, 11, 11, 11, 11, 1, 11, 11, 11, 11, 10, 10, 5, 2, 10, 10, 5, 5, 2, 10, 2, 5, 2, 5, 10, 5, 10, 5, 5, 5, 5, 10, 10, 5, 5, 10, 10, 10, 10, 5, 10, 10, 5, 3, 3, 3, 1, 3, 9, 1, 3, 3, 9, 3, 3, 3, 9, 3, 3, 9, 9, 9, 9, 9, 3, 3, 3, 9, 9, 3, 9, 8, 4, 8, 1, 8, 8, 8, 2, 2, 4, 4, 4, 2, 8, 8, 4, 4, 4, 1, 4, 4, 2, 1, 8, 4, 8, 4, 4, 4, 8, 8, 2, 2, 2, 8, 8, 4, 8, 8, 1, 8, 8, 1, 8, 8, 4, 2, 8, 8, 4, 8, 8, 2, 4, 8, 4, 4, 2, 1, 7, 7, 7, 7, 7, 1, 7, 7, 1, 7, 1, 7, 7, 7, 7, 7, 7, 7, 7, 7, 7, 6, 2, 1, 6, 3, 6, 6, 2, 3, 2, 6, 3, 6, 6, 6, 6, 1, 2, 3, 6, 6, 2, 2, 6, 6, 3, 2, 6, 3, 3, 2, 3, 2, 1, 2, 1, 6, 3, 3, 3, 3, 2, 3, 6, 3, 2, 3, 3, 6, 3, 3, 6, 1, 6, 2, 3, 6, 2, 3, 5, 5, 5, 5, 5, 1, 5, 5, 5, 5, 1, 5, 5, 5, 1, 1, 5, 5, 5, 1, 5, 5, 1, 5, 5, 5, 5, 5, 5, 5, 5, 1, 5, 5, 2, 2, 1, 4, 4, 1, 4, 2, 1, 1, 2, 4,

4, 2, 4, 4, 4, 1, 1, 4, 1, 2, 4, 2, 1, 4, 4, 4, 4, 4, 4, 4, 4, 1, 4, 1, 4, 1, 1, 1, 2, 1, 4, 4, 1, 4, 2, 1, 4, 4, 4, 2, 4, 2, 1, 2, 4, 2, 4, 4, 4, 4, 4, 4, 4, 4, 3, 1, 3, 1, 3, 3, 3, 3, 1, 3, 3, 3, 3, 3, 3, 3, 3, 1, 3, 3, 3, 1, 3, 3, 3, 1, 3, 3, 3, 1, 3, 1, 3, 3, 3, 3, 3, 1, 3, 1, 3, 3, 3, 1, 3, 3, 3, 3, 3, 1, 3, 1, 3, 3, 3, 3, 3, 3, 3, 3, 3, 3, 3, 3, 3, 3, 3, 3, 3, 3, 3, 3, 3, 3, 3, 3, 3, 3, 3, 3, 3, 1, 2, 1, 1, 1, 1, 2, 2, 2, 2, 1, 2, 2, 1, 2, 2, 2, 1, 2, 2, 2, 1, 2, 2, 2, 2, 1, 2, 2, 2, 2, 2, 2, 1, 1, 1, 2, 1, 1, 1, 2, 1, 2, 2, 1, 2, 2, 2, 2, 1, 2, 2, 1, 2, 2, 1, 1, 2, 1, 1, 2, 1, 1, 2, 2, 2, 2, 1, 2, 2, 2, 1, 2, 2, 1, 1, 2, 1, 2, 2, 2, 2, 2, 1, 1, 1, 1, 1, 1, 1, 1, 1, 1, 1, 1, 1, 1, 1, 1, 1, 1, 1, 1, 1, 1, 1, 1, 1, 1, 1, 1, 1, 1, 1, 1, 1, 1, 1, 1, 1, 1, 1, 1, 1, 1, 1, 1, 1, 1, 1, 1, 1, 1, 1, 1, 1, 1, 1, 1, 1, 1, 1, 1, 1, 1, 1, 1, 1, 1, 1, 1, 1, 1, 1, 1, 1, 1, 1, 1, 1, 1, 1, 1, 1, 1, 1, 1, 1, 1, 1, 1, 1, 1

Gene ontology terms ranked according to mean of effect sizes in cluster:
neurogenesis | DBD domain binding | embryonic development | DNA binding | central nervous system neuron development | cell fate determination | regionalization | Notch signaling pathway | central nervous system development | gene expression | chordate embryonic development | organ development | limb development | segmentation | anatomical structure morphogenesis | somitogenesis | gastrulation

Corresponding mean of effect sizes of each Gene ontology term:
(In the same order as above)
2.11, 2.10, 1.97, 1.90, 1.71, 1.48, 1.22, 1.18, 1.12, 1.11, 1.06, 0.92, 0.88, 0.84, 0.83, 0.83, 0.43

Corresponding total number of genes of each Gene ontology term:
(In the same order as above)
409, 411, 1.2K, 1.2K, 37, 145, 62, 103, 110, 3.8K, 671, 339, 129, 109, 583, 116, 293

Corresponding number of genes of each Gene ontology term with at least one TFBS (of selected TFs):
(In the same order as above)
94, 106, 259, 260, 9, 41, 19, 28, 23, 655, 150, 77, 32, 29, 116, 27, 59

Corresponding total number of TFBS (of selected TFs) for each Gene ontology term (in all genes):
(In the same order as above)
523, 550, 1259, 1328, 74, 231, 121, 162, 145, 3083, 708, 378, 132, 167, 557, 139, 279

Corresponding number of selected TFs each Gene ontology term is involved with:
(In the same order as above)
12, 12, 12, 12, 12, 12, 12, 12, 12, 12, 12, 12, 12, 12, 12, 12, 12

TFs ranked according to mean of effect sizes in cluster:
V$E2F1_Q6_01, V$E2F_Q6, V$E2F_Q3_01, V$E2F_Q4, V$E2F1DP1_01, V$E2F1_Q4_01, V$E2F_Q4_01, V$E2F_Q3, V$E2F_Q6_01, V$E2F4DP2_01, V$E2F1DP2_01, V$E2F_03

Corresponding mean of effect sizes of each TF:
(In the same order as above)
2.44, 1.99, 1.66, 1.40, 1.40, 1.33, 1.21, 1.19, 1.07, 0.81, 0.60, 0.22

Corresponding total number of TFBS for each TF (genome-wide):
(In the same order as above)
700, 699, 690, 702, 682, 681, 667, 688, 686, 656, 658, 688

Corresponding total number of TFBS for each TF (in all genes in selected Gene ontology terms):
(In the same order as above)
942, 894, 870, 853, 825, 830, 790, 810, 846, 709, 704, 763

Corresponding number of genes (of selected Gene ontology terms) each TF is involved with:
(In the same order as above)
324, 325, 325, 326, 311, 321, 305, 315, 333, 284, 278, 315

Corresponding number of selected Gene ontology terms each TF is involved with:
(In the same order as above)
17, 17, 17, 17, 17, 17, 17, 17, 17, 17, 17, 17

Cluster for columns 1054 to 1073, rows 267 to 293

Gene ontology terms

axon guidance | nervous system development | synaptic cleft | terminal button | cognition | toxin binding | choline O-acetyltransferase activity | neurotransmitter biosynthetic process | basal lamina | sarcoplasm | response to ATP | response to histamine | serotonin receptor activity | interchromatin granule | acetylcholinesterase activity | choline transport | angiotensin-converting enzyme inhibitor activity | saliva secretion | antigenic variation | serotonin biosynthetic process

TFs

V$ETF_Q6 | V$E2F_Q2 | V$E2F1_Q6 | V$E2F1_Q3 | V$AP2GAMMA_01 | V$AP2ALPHA_01 | V$AP2_Q6_01 | V$AP2_Q6 | V$WT1_Q6 | V$EGR_Q6 | V$MAZ_Q6 | V$MAZR_01 | V$E2F1_Q3_01 | V$HES1_Q2 | V$ACAAT_B | V$AP2_Q3 | V$AP2ALPHA_02 | V$HIC1_03 | V$HIC1_02 | V$PAX5_01 | V$ZNF219_01 | V$SP1_Q2_01 | V$SP1_01 | V$SP1_Q6 | V$SP1_Q4_01 | V$SP1_Q6_01 | V$KROX_Q6

Information

All related TFs:
(List of all TFs that are related to any of the PWMs)
AP-2, AP-2alpha, AP-2alphaA, AP-2alphaB, AP-2beta, AP-2gamma, DP-1, E2F, E2F+E4, E2F-1, E2F-3a, E2F-4, ETF, Egr-1, Egr-2, Egr-3, Egr-4, HES-1, HIC-1, MAZ, MAZR, Pax-5, Sp1, Sp2, Sp3, Sp4, WT1, WT1-del2, ZNF219

Ranked gene list:
(All genes of the selected Gene ontology terms with hits of any of the selected TFs, ranked according to the total number of TFBS)
ACHE, FGFR3, AGRN, AKT1, PLXNA1, CDK5, SLC25A1, GPT, NKX2-2, THRA, PER1, ENTPD2, ACTG1, RHOF, PAX6, NTN3, SLC2A4RG, PLEC1, GIT1, PGP, LBX1, VEGFA, SEMA3B, MDK, EFNA3, DVL1, NOC2L, FLNA, NGFR, HBA2, SLC4A2, PPP1R9B, IGF1R, GPC1, DBP, NR3C2, KCNH2, PDE4A, NEO1, JUND, ING2, HES1, GNAI2, GFI1, ENO3, CDT1, BRD1, TNFSF13, TMEM121, SH2D3C, SH2B2, NOTCH1, FLJ32987, WBP7, SCRIB, EGR3, TIAM1, EFNA2, PSD, SOX1, PXN, NR3C1, LHX3, GALNS, FXYD1, EPHA1, SLC30A3, NTN1, EFNB2, EFNA1, CTF1, VASP, PRKCE, POU2F1, PLXND1, NPTX1, LMX1B, IGF2, CASP9, RHOG, RARA, PRKCZ, NFIB, NFIA, EGR1, CDH15, ARHGEF2, ZMYM2, VIM, VDAC1, SLC17A7, SEMA3F, RHOA, NFIX, MUC1, MGAT1, HNRPDL, EPB49, DPYSL2, DPAGT1, DAG1, B3GAT1, ARSA, WNT4, PITRM1, P2RY11, KCNJ11, FBXL15, EMX1, DLG4, BDNF, TFAP2A, SLC7A1, SLC12A2, SHH, RABEP2, PRKCA, PPP1R14A, NT5C2, MICAL1, LIMK1, HRH3, CRY1, CDH2, ARHGEF11, UNC5A, TPM1, SMURF1, SCARB1, PXMP2, PLXNA3, PARD6A, NTF4, NCAN, MAPT, MAP1B, LRP5, LHX4, FOSL1, FGF2, DMPK, DBN1, ACCN2, SYN1, STX1A, SRC, SNAP25, SLC17A5, SFRS2, SEMA7A, SDC3, RPE, RGMA, PTPRF, PSEN1, PPFIA1, PNMT, PLXNA4, NTNG2, NRP2, NRP1, NOTCH3, NGEF, NF2, MCF2L, LMNA, L1CAM, KCNB1, HTR6, HBA1, GALR3, FYN, EPHB2, EPHA4, ENAH, EIF3B, EFNA5, DPYSL5, DPYSL3, DNER, DLX3, CYLD, CRAT, COMP, AMT, WNT5A, WNT3, VPS13A, VCL, UAP1, TRPC1, TIMP2, STX2, STX1B, ST3GAL4, SNAI1, SLIT3, SLC7A10, SLC6A9, SLC32A1, SEMA4G, SDHB, S100A6, RYK, RPS15A, PTK2, PSMD1, POMGNT1, PNPLA6, PMP22, PLXNB1, P2RX5, ODC1, NTRK3, NOTCH4, NEFM, NCK1, MSH3, MMP14, MARCKS, MAPK8IP3, MAPK1, MAP1A, LHX2, LAMA5, KIT, JUN, ITGB4, ITGB1, IQGAP1, HTT, GAA, FOS,

EZR, ERBB4, EPHB4, ENO2, ELK3, E2F5, DRD4, CSE1L, CRMP1, CNTFR, CNP, CENPV, CELSR2, CELSR1, ATP7B, ARL6IP5, ARID4B, ARHGEF12, AQP3, ANXA7, AGFG1, ADA, ACE, ABL1

Corresponding total number of TFBS:
(For each gene listed above, the total number of TFBS for any of the selected TFs, multiplied by the number of selected Gene ontology terms containing that gene)
252, 238, 95, 66, 42, 40, 34, 32, 30, 28, 28, 28, 27, 26, 26, 26, 25, 25, 25, 24, 24, 22, 22, 22, 22, 22, 21, 21, 20, 20, 19, 18, 18, 18, 18, 17, 17, 16, 16, 16, 16, 16, 16, 16, 16, 16, 15, 15, 15, 15, 15, 15, 14, 14, 14, 12, 12, 11, 10, 10, 10, 10, 10, 10, 10, 9, 9, 9, 9, 9, 8, 8, 8, 8, 8, 8, 8, 8, 7, 7, 7, 7, 7, 7, 7, 7, 6, 6, 6, 6, 6, 6, 6, 6, 6, 6, 6, 6, 6, 6, 6, 5, 5, 5, 5, 5, 5, 5, 5, 4, 4, 4, 4, 4, 4, 4, 4, 4, 4, 4, 4, 4, 4, 3, 3, 3, 3, 3, 3, 3, 3, 3, 3, 3, 3, 3, 3, 3, 3, 3, 3, 2, 2, 2, 2, 2, 2, 2, 2, 2, 2, 2, 2, 2, 2, 2, 2, 2, 2, 2, 2, 2, 2, 2, 2, 2, 2, 2, 2, 2, 2, 2, 2, 2, 2, 2, 2, 2, 2, 2, 1, 1, 1, 1, 1, 1, 1, 1, 1, 1, 1, 1, 1, 1, 1, 1, 1, 1, 1, 1, 1, 1, 1, 1, 1, 1, 1, 1, 1, 1, 1, 1, 1, 1, 1, 1, 1, 1, 1, 1, 1, 1, 1, 1, 1, 1, 1, 1, 1, 1, 1, 1, 1, 1, 1, 1, 1, 1, 1, 1, 1, 1, 1, 1, 1, 1, 1, 1, 1, 1, 1

Corresponding number of selected Gene ontology terms each gene is involved with:
(In the same order as above)
12, 14, 5, 3, 2, 2, 2, 2, 2, 2, 2, 2, 1, 1, 2, 1, 1, 1, 1, 1, 2, 2, 2, 1, 1, 1, 1, 1, 4, 1, 1, 2, 1, 1, 1, 1, 1, 1, 2, 1, 1, 1, 1, 1, 1, 1, 2, 5, 1, 1, 1, 1, 1, 1, 1, 1, 1, 1, 1, 1, 2, 1, 2, 2, 1, 2, 1, 3, 1, 1, 1, 1, 1, 1, 1, 1, 1, 1, 1, 1, 1, 1, 1, 1, 1, 1, 1, 1, 1, 2, 1, 2, 1, 2, 1, 1, 1, 1, 1, 2, 2, 2, 3, 1, 1, 1, 1, 1, 1, 1, 1, 5, 1, 1, 1, 2, 1, 2, 1, 4, 2, 2, 2, 1, 4, 2, 1, 1, 1, 1, 1, 1, 1, 3, 3, 1, 3, 1, 1, 1, 3, 1, 1, 1, 2, 1, 2, 2, 2, 1, 1, 1, 2, 2, 1, 2, 1, 2, 1, 1, 1, 2, 1, 2, 2, 1, 1, 2, 1, 2, 1, 1, 1, 2, 1, 2, 1, 1, 1, 2, 1, 1, 1, 2, 2, 1, 1, 1, 1, 1, 1, 1, 1, 1, 1, 1, 1, 1, 1, 1, 1, 1, 1, 1, 1, 1, 1, 1, 1, 1, 1, 1, 1, 1, 1, 1, 1, 1, 1, 1, 1, 1, 1, 1, 1, 1, 1, 1, 1, 1, 1, 1, 1, 1, 1, 1, 1, 1, 1, 1, 1, 1, 1, 1, 1, 1, 1, 1, 1, 1, 1, 1, 1, 1, 1, 1, 1, 1, 1, 1, 1

Corresponding number of selected TFs each gene is involved with:
(In the same order as above)
21, 17, 19, 22, 21, 20, 17, 16, 15, 14, 14, 14, 27, 26, 13, 26, 25, 25, 25, 24, 12, 11, 11, 22, 22, 22, 21, 21, 5, 20, 19, 9, 18, 18, 18, 17, 17, 16, 8, 16, 16, 16, 16, 16, 16, 16, 8, 3, 15, 15, 15, 15, 15, 14, 14, 14, 12, 12, 11, 10, 5, 10, 5, 5, 10, 5, 9, 3, 9, 9, 9, 8, 8, 8, 8, 8, 8, 8, 8, 7, 7, 7, 7, 7, 7, 7, 7, 6, 3, 6, 3, 6, 3, 6, 6, 6, 6, 6, 3, 3, 3, 2, 6, 5, 5, 5, 5, 5, 5, 5, 1, 4, 4, 4, 2, 4, 2, 4, 1, 2, 2, 2, 4, 1, 2, 3, 3, 3, 3, 3, 3, 3, 1, 1, 3, 1, 3, 3, 3, 1, 3, 3, 3, 1, 2, 1, 1, 1, 2, 2, 2, 1, 1, 2, 1, 2, 1, 2, 2, 2, 1, 2, 1, 1, 2, 2, 1, 2, 1, 2, 2, 2, 1, 2, 1, 2, 2, 2, 1, 2, 2, 2, 1, 1, 2, 1, 1, 1, 1, 1, 1, 1, 1, 1, 1, 1, 1, 1, 1, 1, 1, 1, 1, 1, 1, 1, 1, 1, 1, 1, 1, 1, 1, 1, 1, 1, 1, 1, 1, 1, 1, 1, 1, 1, 1, 1, 1, 1, 1, 1, 1, 1, 1, 1, 1, 1, 1, 1, 1, 1, 1, 1, 1, 1, 1, 1, 1, 1, 1, 1, 1, 1, 1, 1, 1, 1, 1, 1

Gene ontology terms ranked according to mean of effect sizes in cluster:
axon guidance | sarcoplasm | nervous system development | interchromatin granule | choline transport | response to histamine | neurotransmitter biosynthetic process | toxin binding | synaptic cleft | saliva secretion | terminal button | angiotensin-converting enzyme inhibitor activity | response to ATP | antigenic variation | basal lamina | serotonin biosynthetic process | cognition | choline O-acetyltransferase activity | acetylcholinesterase activity | serotonin receptor activity

Corresponding mean of effect sizes of each Gene ontology term:
(In the same order as above)
1.77, 1.56, 1.44, 1.18, 1.11, 1.06, 1.04, 1.03, 1.00, 0.98, 0.76, 0.72, 0.66, 0.66, 0.61, 0.60, 0.56, 0.53, 0.51, 0.39

Corresponding total number of genes of each Gene ontology term:
(In the same order as above)
187, 24, 185, 35, 26, 37, 27, 33, 66, 27, 21, 63, 47, 37, 112, 34, 69, 37, 27, 49

Corresponding number of genes of each Gene ontology term with at least one TFBS (of selected TFs):
(In the same order as above)
82, 9, 78, 11, 5, 8, 9, 9, 21, 10, 5, 17, 10, 8, 32, 8, 18, 11, 5, 9

Corresponding total number of TFBS (of selected TFs) for each Gene ontology term (in all genes):

(In the same order as above)
450, 92, 462, 92, 65, 66, 74, 68, 122, 90, 36, 137, 70, 75, 169, 60, 110, 53, 58, 73

Corresponding number of selected TFs each Gene ontology term is involved with:
(In the same order as above)
27, 27, 27, 27, 27, 26, 27, 26, 27, 27, 21, 27, 27, 27, 27, 26, 27, 25, 27, 26

TFs ranked according to mean of effect sizes in cluster:
V$WT1_Q6, V$E2F_Q2, V$SP1_01, V$AP2ALPHA_01, V$AP2GAMMA_01, V$EGR_Q6, V$E2F1_Q3, V$AP2_Q6_01, V$HES1_Q2, V$ETF_Q6, V$AP2_Q6, V$SP1_Q6_01, V$KROX_Q6, V$HIC1_02, V$SP1_Q2_01, V$PAX5_01, V$E2F1_Q3_01, V$MAZ_Q6, V$SP1_Q4_01, V$SP1_Q6, V$AP2_Q3, V$ACAAT_B, V$AP2ALPHA_02, V$MAZR_01, V$HIC1_03, V$ZNF219_01, V$E2F1_Q6

Corresponding mean of effect sizes of each TF:
(In the same order as above)
1.44, 1.40, 1.39, 1.36, 1.31, 1.31, 1.30, 1.24, 1.23, 1.21, 1.17, 1.03, 0.95, 0.91, 0.85, 0.82, 0.80, 0.77, 0.75, 0.65, 0.62, 0.51, 0.48, 0.39, 0.31, 0.23, 0.09

Corresponding total number of TFBS for each TF (genome-wide):
(In the same order as above)
729, 714, 696, 686, 695, 695, 709, 690, 690, 699, 688, 663, 675, 664, 695, 671, 680, 734, 656, 664, 683, 692, 663, 705, 660, 689, 691

Corresponding total number of TFBS for each TF (in all genes in selected Gene ontology terms):
(In the same order as above)
121, 110, 99, 102, 102, 109, 101, 108, 104, 103, 103, 78, 90, 90, 85, 75, 90, 92, 70, 69, 81, 88, 67, 76, 67, 70, 72

Corresponding number of genes (of selected Gene ontology terms) each TF is involved with:
(In the same order as above)
70, 68, 58, 61, 61, 65, 59, 65, 63, 61, 60, 53, 55, 53, 57, 44, 50, 62, 46, 45, 54, 62, 45, 57, 44, 52, 54

Corresponding number of selected Gene ontology terms each TF is involved with:
(In the same order as above)
20, 20, 20, 20, 20, 20, 20, 20, 20, 20, 20, 20, 19, 20, 20, 20, 20, 19, 19, 20, 20, 19, 20, 18, 20, 16, 18

Cluster for columns 325 to 327, rows 32 to 38

Gene ontology terms

oxygen and reactive oxygen species metabolic process | catalase activity | superoxide dismutase activity

TFs

V$E4BP4_01 | V$USF_01 | V$MAX_01 | V$USF_Q6 | V$USF_02 | V$USF_Q6_01 | V$MYCMAX_03

Information

All related TFs:
(List of all TFs that are related to any of the PWMs)
E4BP4, Max, Max1, USF, USF-1, USF1, USF1:USF2, USF1a, USF1b, USF2, USF2a, USF2b, c-Myc

Ranked gene list:
(All genes of the selected Gene ontology terms with hits of any of the selected TFs, ranked according to the total number of TFBS)
MTHFR, GPX1, APEX1, SIRT1, RCBTB1, KATNB1, GCLC, ACHE, PLA2G1B, NOS2, HMHA1, GPX3, FTH1, CYCS, CASP3, ST3GAL4, NUDT1, ESRRA, YAP1, PRNP, PRDX4, POR, PDSS1, NFE2L2, LOC100131187, HIF1A, H2AFX

Corresponding total number of TFBS:
(For each gene listed above, the total number of TFBS for any of the selected TFs, multiplied by the number of selected Gene ontology terms containing that gene)
18, 15, 12, 6, 6, 6, 4, 4, 3, 3, 3, 3, 3, 3, 3, 2, 2, 2, 1, 1, 1, 1, 1, 1, 1, 1, 1

Corresponding number of selected Gene ontology terms each gene is involved with:
(In the same order as above)
3, 3, 2, 1, 1, 1, 2, 1, 1, 3, 1, 3, 1, 3, 3, 2, 1, 1, 1, 1, 1, 1, 1, 1, 1, 1, 1

Corresponding number of selected TFs each gene is involved with:
(In the same order as above)
6, 5, 6, 6, 6, 6, 2, 4, 3, 1, 3, 1, 3, 1, 1, 1, 2, 2, 1, 1, 1, 1, 1, 1, 1, 1, 1

Gene ontology terms ranked according to mean of effect sizes in cluster:
superoxide dismutase activity | catalase activity | oxygen and reactive oxygen species metabolic process

Corresponding mean of effect sizes of each Gene ontology term:
(In the same order as above)
3.31, 2.12, 1.61

Corresponding total number of genes of each Gene ontology term:
(In the same order as above)
40, 77, 79

Corresponding number of genes of each Gene ontology term with at least one TFBS (of selected TFs):
(In the same order as above)
10, 15, 17

Corresponding total number of TFBS (of selected TFs) for each Gene ontology term (in all genes):
(In the same order as above)
29, 40, 38

Corresponding number of selected TFs each Gene ontology term is involved with:
(In the same order as above)
7, 7, 7

TFs ranked according to mean of effect sizes in cluster:
V$MYCMAX_03, V$USF_01, VUSF_Q6, VMAX_01, VUSF_02, VE4BP4_01, V$USF_Q6_01

Corresponding mean of effect sizes of each TF:
(In the same order as above)
3.72, 2.52, 2.49, 2.19, 1.91, 1.81, 1.80

Corresponding total number of TFBS for each TF (genome-wide):
(In the same order as above)
650, 647, 672, 647, 649, 635, 664

Corresponding total number of TFBS for each TF (in all genes in selected Gene ontology terms):

(In the same order as above)
21, 16, 16, 15, 13, 13, 13

Corresponding number of genes (of selected Gene ontology terms) each TF is involved with:
(In the same order as above)
14, 9, 10, 10, 8, 8, 9

Corresponding number of selected Gene ontology terms each TF is involved with:
(In the same order as above)
3, 3, 3, 3, 3, 3, 3

Cluster for columns 1296 to 1310, rows 199 to 202

Gene ontology terms

gene expression | DBD domain binding | DNA binding | chordate embryonic development | embryonic development | limb development | central nervous system development | central nervous system neuron development | gastrulation | somitogenesis | anatomical structure morphogenesis | organ development | neurogenesis | regionalization | cell fate determination

TFs

V$TFIII_Q6 | V$MZF1_01 | V$VDR_Q3 | V$MZF1_02

Information

All related TFs:
(List of all TFs that are related to any of the PWMs)
MZF1B-C, TFII-I, VDR

Ranked gene list:
(All genes of the selected Gene ontology terms with hits of any of the selected TFs, ranked according to the total number of TFBS)
LBX1, FGF8, WNT1, GLI1, RARA, LEF1, LFNG, VEGFA, HOXA9, AKT1, NR2F1, HES5, TNFSF13, TGFB1, TCF3, RARG, PAX2, NKX2-2, MDK, HOXB7, CDC25C, PAX6, NOTCH1, PBX2, NFATC1, MMP11, LEPREL2, GDF11, DVL1, DGAT1, CDK5, AGRN, HOXA10, DLL1, CHRD, SSSCA1, SEMA3B, OSM, LHX1, LDB1, HOXC8, HOXC6, HOXB8, HOXB6, HOXB4, HMGA1, GATA3, GATA2, EGR1, DLL4, CCND2, BRD2, BHLHB2, LIF, FLI1, FGF9, EFNA2, TFAP2A, OTX1, NR4A2, HMX3, ZFPM1, ZEB2, VEGFB, UNCX, TLE3, THRA, TCF7, SMARCD3, SMAD7, SIN3A, RXRB, NR4A1, NOTCH4, NFIC, MEF2D, MAPK12, MAP2K7, LRP1, JUNB, HOXB5, HOXA7, HES7, ERF, DNMT3A, COL11A2, CDK4, SP1, JUN, STAT5A, NFATC3, MXI1, IL23A, HOXB3, HDGF, GFI1, EHMT2, EFNA1, CORO7, ADNP, ACHE, ZBTB7B, ZBTB7A, WNT10A, VGF, UBTF, TRADD, SSPO, SOCS3, SNAPC2, RREB1, PURA, PPP1R1B, POU2F1, PARD6A, NFKBIE, NFKB2, NELF, NAB2, MUC1, MNT, MAZ, LYL1, LTB, ISYNA1, ISG15, HOXC5, HOXC4, HIVEP1, FOXP1, FGF17, DPH1, DKFZp779C0757, DBP, CISH, CHD3, CFL1, ARID3A, ARHGEF2, ABCA2, PROX1, PITX1, NR2F2, NOL3, MEIS2, DLX2, CXCR4, ZIC2, ZFP36L2, ZEB1, WNT7A, WNT6, WNK1, WBP7, VIM, TNF, TGIF1, TEAD3, TCF7L2, STRA13, SP4, SOX18, SLC20A1, SIX3, SHC1, PTCH1, PLXNA1, NR3C2, MSL1, MLL, MAP4K2, KLF6, IRF1, HOXD12, HOXC9, HOXA11, FST, FOXO3, FLII, EGR3, EGLN2, DDIT3, CSK, CRABP2, BCL3, B3GAT1, ARID1A, ALDOA, WNT2B, WNT11, TPM3, SP3, NGFR, JARID2, HOXB9, FURIN, EN1, COMP, CDKN1C, CCND1, ZFP36L1, ZFP36, ZBTB16, WNT10B, TYMP, TRERF1, TNFRSF25, TNFRSF1A, TNFRSF18, TNFRSF12A, TFEB, TBX1, STAT6, SSBP3, SMARCA2, SLC4A2, SLC25A22, SHMT2, SCRT1, RPL19, RORC, RING1, RHOC, REXO1, RCOR2, PRMT1, POLR2A, PNMT, PLEC1, PIAS3, PER1, PCP2, PCGF2, NRARP, NR2F6, NR1D1,

NFIA, MVP, MGAT1, MAPKAPK3, MAP3K12, MAFG, LTC4S, LTA, LRDD, LMO4, LLGL2, LIN28, KREMEN2, KISS1R, KAT2A, JUND, JMJD3, IRX5, ING2, IGF2, HYAL2, HNRPDL, HNRNPL, HIVEP2, HIC1, HDAC7, HCFC1R1, GRIN2D, GPS2, GIT1, GAPDH, GABBR1, FOXJ1, FOXD3, FMNL1, FGFRL1, FASTK, FASN, ENO3, EFNA4, EFNA3, DVL2, DLX6, DLX4, DLG4, CRIP2, CRIP1, CORO1A, CLDN6, CKB, CIRBP, CEND1, CDK9, CD151, CBX4, CACNB3, CACNA1G, BCOR, BCL9L, BCL6, BCL11B, BCL11A, BBC3, BAK1, ATXN1, ANP32A, AGER, ACTB, ZNF148, UBE2I, TSPAN32, TNFRSF4, TGFB3, TCF12, TAGLN2, SYTL1, SPIB, SOX8, SKI, SIPA1, SAMD4A, S100A6, S100A4, RPS6KA1, ROBLD3, RND2, PRKCD, POU3F2, POU2F2, PNPLA2, PHLDB1, PELP1, PEA15, PATZ1, PARN, NFATC2, NCLN, MLL2, MARCKSL1, MAPK11, LTBP3, LGALS1, LASP1, KLF1, IRX4, IRS1, IRF7, IRF2, IGF1R, HOXC10, HIST1H4I, GPX4, GPC1, FOXP4, FOSB, FLJ32987, FBXW4, FBRS, ETV5, EPHB3, ENOPH1, EMP3, EIF5A, EGFL7, EDAR, E2F3, DCHS1, DAXX, CTSD, CRIM1, CLEC11A, CEBPE, CDK6, CD68, CAMK2G, ATP6V0D1, ADORA2A, ZNF274, ZNF24, ZHX2, XRCC6, XAB2, VIPR1, UBE4B, TUSC2, TRIM3, TRIM28, TRAF4, TOB1, TNNT1, THPO, TAS1R3, SQSTM1, SOCS1, SLC2A4RG, SIX5, SETD1A, SEPT1, SATB2, SATB1, REV3L, REPIN1, PTMA, PSMP, PSENEN, PRKCE, POU3F3, PKNOX2, PIK3R2, PIGQ, PHF12, PGP, PDGFB, PCDH1, ORC4L, OGFR, NRTN, NRGN, NNAT, NFKBIA, NFATC4, NEUROD2, MYLIP, MYL6, MSI2, MFNG, MCAM, MARK2, MAFA, LY6E, LMNA, LAMA5, KLF7, ITGB2, IL11, IGF2BP1, IER2, HYAL3, HSP90AB1, HOXA6, HNRNPD, HNRNPA1, HMG20B, GAMT, FZD2, FOXJ2, FOXF2, FOSL2, FHOD1, ESRRA, ERBB3, ENO2, EGR4, DDR1, DCTN3, CTSW, CTDSP2, CRB3, COL6A1, CNTFR, CHD1, CDH15, CDC20, CD63, CCR7, CBX5, BMF, BCL2L11, ATF3, AKT3, AIRE, AIF1, ADM, ZYX, ZNF2, ZC3H12A, ZAP70, YBX2, XAF1, UNG, UBR5, TUBB2C, TPM1, TOX4, TLR9, SP7, SOX30, SOCS2, SLCO4A1, SLC29A1, SLC12A5, SKIL, SHOX2, SFMBT2, SERTAD3, SERPINH1, SDHC, RPS2, RPL8, RNASEH2C, RBM35A, RASSF1, PTP4A3, PTH1R, PTGER1, PRPF40A, PRDM16, PPP1R15B, PPP1R12C, POLD4, PNKD, PBXIP1, NUDT1, NKG7, MYADM, MXD4, MTA2, MPZ, MEN1, MC1R, MAPK13, MAD1L1, LPHN1, LHX4, LAT, LAPTM5, KAT5, JAG2, ITM2C, INHBA, IMP3, IL2RB, IL17C, IGFBP6, IDI1, ICAM2, HYAL1, HS6ST1, H2AFY, GRIN2C, GRIK5, GRB7, GPSM1, GPER, GGPS1, GCDH, GADD45B, FUSIP1, FHL3, ERBB2IP, EPB41, ELL, ELAVL3, EIF4G1, EIF4A1, EEF1A2, DYRK2, DUSP1, DPYSL3, CYBA, CUL7, CTNNBIP1, CTBP2, CRTC2, CPLX1, CHST11, CHRNE, CHPT1, CFLAR, CD79A, CD3D, CD27, CCND3, CBX3, C1QL1, BAT2, ATP6V0C, ASXL1, ARID5B, ARID4A, ARHGAP5, AGRP, ADRA2B, ACTG1

Corresponding total number of TFBS:
(For each gene listed above, the total number of TFBS for any of the selected TFs, multiplied by the number of selected Gene ontology terms containing that gene)
45, 44, 40, 40, 36, 33, 32, 28, 28, 28, 27, 27, 24, 24, 24, 24, 24, 24, 24, 24, 24, 22, 22, 20, 20, 20, 20, 20, 20, 20, 20, 20, 18, 18, 18, 16, 16, 16, 16, 16, 16, 16, 16, 16, 16, 16, 16, 16, 16, 16, 16, 16, 16, 15, 15, 15, 15, 14, 14, 14, 14, 12, 12, 12, 12, 12, 12, 12, 12, 12, 12, 12, 12, 12, 12, 12, 12, 12, 12, 12, 12, 12, 12, 12, 12, 12, 12, 10, 10, 9, 9, 9, 9, 9, 9, 9, 9, 9, 9, 9, 9, 8, 8, 8, 8, 8, 8, 8, 8, 8, 8, 8, 8, 8, 8, 8, 8, 8, 8, 8, 8, 8, 8, 8, 8, 8, 8, 8, 8, 8, 8, 8, 8, 8, 8, 8, 8, 8, 8, 8, 7, 7, 7, 7, 7, 7, 7, 6, 6, 6, 6, 6, 6, 6, 6, 6, 6, 6, 6, 6, 6, 6, 6, 6, 6, 6, 6, 6, 6, 6, 6, 6, 6, 6, 6, 6, 6, 6, 6, 6, 6, 6, 6, 6, 6, 6, 6, 6, 6, 6, 6, 5, 5, 5, 5, 5, 5, 5, 5, 5, 5, 5, 5, 4, 3, 3, 3, 3, 3, 3, 3, 3, 3, 3, 3, 3, 3, 3, 3, 3, 3, 3, 3, 3, 3, 3, 3, 3, 3, 3, 3, 3, 3, 3, 3, 3, 3, 3, 3, 3, 3, 3, 3, 3, 3, 3, 3, 3, 3, 3, 3, 3, 3, 3, 3, 3, 3, 3, 3, 3, 3, 3, 3, 3, 3, 3, 3, 3, 3, 3, 3, 3, 3, 2, 2, 2, 2, 2, 2, 2, 2, 2, 2, 2, 2, 2, 2, 2, 2, 2, 2, 2, 2, 2, 2, 2, 2, 2, 2, 2, 2, 2, 2, 2, 2, 2, 2, 2, 2, 2, 2, 2, 2, 2, 2, 2, 2, 2, 2, 2, 2, 2, 2, 2, 2, 2, 2, 2, 2, 2, 2, 2, 2, 2, 2, 2, 2, 2, 2, 2, 2, 2, 2, 2, 2, 2, 2, 2, 2, 2, 2, 2, 2, 2, 2, 2, 2, 2, 2, 2, 2, 2, 2, 2, 2, 2, 2, 2, 2, 1

Corresponding number of selected Gene ontology terms each gene is involved with:
(In the same order as above)
15, 11, 10, 10, 9, 11, 8, 7, 7, 7, 9, 9, 12, 8, 8, 6, 12, 6, 6, 6, 6, 11, 11, 5, 5, 5, 5, 5, 5, 5, 5, 5, 6, 9, 9, 4, 4, 4, 8, 4, 4, 4, 4, 4, 4, 4, 4, 4, 4, 4, 4, 4, 4, 5, 5, 5, 5, 7, 7, 7, 7, 3, 3, 3, 3, 3, 3, 4, 3, 4, 3, 3, 3, 6, 3, 3, 3, 3, 3, 3, 3, 6, 3, 3, 4, 3, 3, 5, 10, 3, 3, 3, 9, 3, 3, 3, 3, 3, 3, 3, 3, 2, 2, 2, 2, 2, 2, 2, 2, 2, 2, 2, 2, 4, 4, 2, 2, 2, 2, 2, 2, 2,

2, 2, 2, 2, 2, 2, 2, 2, 8, 2, 2, 2, 2, 2, 2, 2, 2, 2, 7, 7, 7, 7, 7, 7, 7, 3, 3, 3, 6, 6, 3, 2, 6, 2, 3, 3, 6, 2, 3, 2, 6, 6, 3, 6, 3, 3, 2, 2, 2, 3, 3, 3, 2, 6, 6, 3, 6, 3, 2, 2, 3, 2, 2, 3, 2, 2, 5, 5, 5, 5, 5, 5, 5, 5, 5, 5, 5, 1, 1, 4, 4, 1, 1, 2, 2, 1, 1, 2, 4, 2, 2, 4, 1, 1, 1, 1, 1, 1, 1, 1, 2, 1, 1, 1, 1, 1, 2, 1, 1, 1, 2, 4, 1, 4, 1, 1, 1, 1, 4, 1, 2, 1, 4, 1, 4, 1, 1, 4, 2, 1, 4, 2, 4, 2, 4, 1, 1, 1, 1, 1, 1, 1, 1, 1, 1, 4, 4, 1, 1, 1, 2, 1, 1, 1, 1, 4, 4, 1, 1, 1, 4, 1, 1, 1, 1, 2, 1, 1, 1, 1, 1, 1, 1, 1, 1, 1, 2, 1, 1, 1, 4, 3, 3, 1, 1, 3, 3, 1, 3, 3, 3, 3, 1, 3, 1, 1, 3, 3, 1, 1, 3, 3, 1, 1, 3, 3, 3, 3, 3, 1, 1, 1, 1, 1, 3, 1, 3, 3, 1, 3, 3, 3, 1, 3, 3, 3, 1, 3, 3, 3, 1, 3, 3, 3, 1, 1, 3, 3, 3, 1, 1, 1, 3, 1, 3, 1, 1, 1, 3, 1, 2, 2, 2, 2, 1, 2, 2, 1, 1, 1, 2, 1, 2, 1, 1, 1, 2, 1, 2, 1, 1, 1, 2, 2, 1, 1, 2, 2, 1, 1, 1, 2, 1, 1, 1, 1, 1, 1, 1, 2, 1, 1, 2, 2, 2, 2, 1, 1, 2, 1, 2, 2, 1, 1, 2, 2, 1, 1, 2, 1, 1, 1, 1, 1, 2, 2, 1, 2, 2, 2, 2, 1, 1, 2, 1, 2, 1, 1, 1, 1, 1, 1, 1, 2, 2, 1, 1, 2, 2, 1, 2, 2, 1, 1, 2, 1

Corresponding number of selected TFs each gene is involved with:
(In the same order as above)
3, 4, 4, 4, 4, 3, 4, 4, 4, 4, 3, 3, 2, 3, 3, 4, 2, 4, 4, 4, 4, 2, 2, 4, 4, 4, 4, 4, 4, 4, 4, 4, 3, 2, 2, 4, 4, 4, 2, 4, 4, 4, 4, 4, 4, 4, 4, 4, 4, 4, 4, 4, 4, 3, 3, 3, 3, 2, 2, 2, 2, 4, 4, 4, 4, 4, 4, 3, 4, 3, 4, 4, 4, 2, 4, 4, 4, 4, 4, 4, 4, 2, 4, 4, 3, 4, 4, 2, 1, 3, 3, 3, 1, 3, 3, 3, 3, 3, 3, 3, 3, 4, 4, 4, 4, 4, 4, 4, 4, 4, 4, 4, 2, 2, 4, 4, 4, 4, 4, 4, 4, 4, 4, 4, 4, 4, 4, 4, 4, 1, 4, 4, 4, 4, 4, 4, 4, 4, 4, 1, 1, 1, 1, 1, 1, 1, 2, 2, 2, 1, 1, 2, 3, 1, 3, 2, 2, 1, 3, 2, 3, 1, 1, 2, 1, 2, 2, 3, 3, 3, 2, 2, 2, 3, 1, 1, 2, 1, 2, 3, 3, 2, 3, 3, 2, 3, 3, 1, 1, 1, 1, 1, 1, 1, 1, 1, 1, 1, 4, 4, 1, 1, 4, 4, 2, 2, 4, 4, 2, 1, 2, 2, 1, 4, 4, 4, 4, 4, 4, 4, 4, 2, 4, 4, 4, 4, 4, 2, 4, 4, 4, 2, 1, 4, 1, 4, 4, 4, 4, 1, 4, 2, 4, 1, 4, 1, 4, 4, 1, 2, 4, 1, 2, 1, 2, 1, 4, 4, 4, 4, 4, 4, 4, 4, 4, 4, 1, 1, 4, 4, 4, 2, 4, 4, 4, 4, 1, 1, 4, 4, 4, 1, 4, 4, 4, 4, 2, 4, 4, 4, 4, 4, 4, 4, 4, 4, 4, 2, 4, 4, 4, 1, 1, 1, 3, 3, 1, 1, 3, 1, 1, 1, 1, 3, 1, 3, 3, 1, 1, 3, 3, 1, 1, 3, 3, 1, 1, 1, 1, 1, 3, 3, 3, 3, 3, 1, 3, 1, 1, 3, 1, 1, 1, 3, 1, 1, 1, 3, 1, 1, 1, 3, 1, 1, 1, 3, 3, 1, 1, 1, 3, 3, 3, 1, 3, 1, 3, 3, 3, 1, 3, 1, 1, 1, 1, 2, 1, 1, 2, 2, 2, 1, 2, 1, 2, 2, 2, 1, 2, 1, 2, 2, 2, 1, 1, 2, 2, 1, 1, 2, 2, 2, 1, 2, 2, 2, 2, 2, 2, 2, 1, 2, 2, 1, 1, 1, 1, 2, 2, 1, 2, 1, 1, 2, 2, 1, 1, 2, 2, 1, 2, 2, 2, 2, 2, 1, 1, 2, 1, 1, 1, 1, 2, 2, 1, 2, 1, 2, 2, 2, 2, 2, 2, 2, 1, 1, 2, 2, 1, 1, 2, 1, 1, 2, 2, 1, 2, 1, 1, 1, 1, 1, 1, 1, 1, 1, 1, 1, 1, 1, 1, 1, 1, 1, 1, 1, 1, 1, 1, 1, 1, 1, 1, 1, 1, 1, 1, 1, 1, 1, 1, 1, 1, 1, 1, 1, 1, 1, 1, 1, 1, 1, 1, 1, 1, 1, 1, 1, 1, 1, 1, 1, 1, 1, 1, 1, 1, 1, 1, 1, 1, 1, 1, 1, 1, 1, 1, 1, 1, 1, 1, 1, 1, 1, 1, 1, 1, 1, 1, 1, 1, 1, 1, 1, 1, 1, 1, 1, 1, 1, 1, 1, 1, 1, 1, 1

Gene ontology terms ranked according to mean of effect sizes in cluster:
embryonic development | regionalization | neurogenesis | chordate embryonic development | central nervous system development | anatomical structure morphogenesis | organ development | gene expression | DBD domain binding | limb development | DNA binding | cell fate determination | somitogenesis | central nervous system neuron development | gastrulation

Corresponding mean of effect sizes of each Gene ontology term:
(In the same order as above)
3.25, 2.52, 2.30, 2.27, 2.00, 1.93, 1.75, 1.64, 1.53, 1.48, 1.27, 1.20, 1.04, 1.04, 0.76

Corresponding total number of genes of each Gene ontology term:
(In the same order as above)
1.2K, 62, 409, 671, 110, 583, 339, 3.8K, 411, 129, 1.2K, 145, 116, 37, 293

Corresponding number of genes of each Gene ontology term with at least one TFBS (of selected TFs):
(In the same order as above)
203, 22, 77, 119, 26, 97, 60, 463, 76, 24, 179, 30, 20, 7, 46

Corresponding total number of TFBS (of selected TFs) for each Gene ontology term (in all genes):
(In the same order as above)
472, 54, 179, 270, 59, 213, 143, 1064, 171, 51, 422, 73, 49, 20, 99

Corresponding number of selected TFs each Gene ontology term is involved with:
(In the same order as above)
4, 4, 4, 4, 4, 4, 4, 4, 4, 4, 4, 4, 4, 4, 4

TFs ranked according to mean of effect sizes in cluster:
V$MZF1_02, V$MZF1_01, V$TFIII_Q6, V$VDR_Q3

Corresponding mean of effect sizes of each TF:
(In the same order as above)
2.06, 1.85, 1.52, 1.50

Corresponding total number of TFBS for each TF (genome-wide):
(In the same order as above)
747, 729, 726, 720

Corresponding total number of TFBS for each TF (in all genes in selected Gene ontology terms):
(In the same order as above)
909, 840, 821, 769

Corresponding number of genes (of selected Gene ontology terms) each TF is involved with:
(In the same order as above)
349, 325, 330, 315

Corresponding number of selected Gene ontology terms each TF is involved with:
(In the same order as above)
15, 15, 15, 15

Cluster for columns 624 to 627, rows 9 to 22

Gene ontology terms

microtubule organizing center | centrosome | microtubule nucleation | microtubule binding

TFs

V$CREB_Q4_01 | V$CREB_Q2_01 | V$CREB_Q4 | V$CREBP1_Q2 | V$CREB_Q2 | V$CREB_01 | V$CREBP1CJUN_01 | V$CREBATF_Q6 | V$ATF_B | V$CREB_02 | V$ATF_01 | V$ATF3_Q6 | V$ATF1_Q6 | V$CREBP1_01

Information

All related TFs:
(List of all TFs that are related to any of the PWMs)
120-kDa, 47-kDa, ATF, ATF-1, ATF-2, ATF-4, ATF-a, ATF-like, ATF/CREB, ATF2, ATF2-isoform2, ATF3, ATF4, ATF5, ATF6, ATFa-isoform1, CRE-BP1, CRE-BP2, CREB, CREBbeta, CREMalpha, CREMbeta, CREMgamma, CREMtau, CREMtau1, CREMtau2, CREMtaualpha, c-Jun, deltaCREB

Ranked gene list:
(All genes of the selected Gene ontology terms with hits of any of the selected TFs, ranked according to the total number of TFBS)
MAPRE2, TUBGCP2, PARD6A, UBC, MXI1, NEDD1, CENPE, CDC42, NDC80, PAK1, PAFAH1B1, NEK1, GABARAPL1, KIF1A, IKBKAP, ID1, CDC14B, CALM2, UBQLN1, SSSCA1, CEP57, CCDC41, TMEM201, RAD51C, PRC1, TUBGCP6, NF1, IFT20, CNTROB, TACC3, RASSF1, XRCC2, SQSTM1, PARK2, MOAP1, MAP1B, CCNA2, CALM1, AURKA, PLK4, NDEL1, BCAS2, RAB6A, NXF1, EPB41, BRCA1, TRIP10, RAD51L1, MCM2, CDKN1A, CDK5RAP2, CDK2, UBE2E2, TUBGCP5, SKP1, SGOL1, SGMS1, NUMA1, NEK9, MAPT, HSP90AA1, FGFR1, FBL, AURKB, ZBTB16, TP53, SSNA1, SLC25A23, RARA, PTTG1, PIK3CG, NPM1, MAP2K1, GSK3B, GSK3A,

EDC4, E2F3, CROCC, CETN3, ATF4, APC, ANAPC2

Corresponding total number of TFBS:
(For each gene listed above, the total number of TFBS for any of the selected TFs, multiplied by the number of selected Gene ontology terms containing that gene)
48, 36, 26, 24, 24, 22, 22, 20, 16, 14, 14, 14, 14, 12, 12, 12, 12, 12, 11, 10, 10, 10, 9, 9, 9, 8, 8, 8, 8, 7, 7, 6, 6, 6, 6, 6, 6, 6, 6, 5, 5, 5, 4, 4, 4, 4, 3, 3, 3, 3, 3, 3, 2, 2, 2, 2, 2, 2, 2, 2, 2, 2, 2, 2, 1, 1, 1, 1, 1, 1, 1, 1, 1, 1, 1, 1, 1, 1, 1, 1, 1

Corresponding number of selected Gene ontology terms each gene is involved with:
(In the same order as above)
4, 3, 2, 2, 3, 2, 2, 2, 2, 1, 1, 1, 1, 1, 3, 1, 1, 3, 1, 1, 1, 1, 3, 1, 1, 1, 1, 1, 1, 1, 1, 1, 2, 1, 2, 3, 1, 3, 3, 1, 1, 1, 1, 1, 1, 2, 1, 1, 1, 1, 1, 1, 1, 2, 1, 1, 1, 2, 1, 2, 1, 1, 1, 1, 1, 1, 1, 1, 1, 1, 1, 1, 1, 1, 1, 1, 1, 1, 1, 1, 1

Corresponding number of selected TFs each gene is involved with:
(In the same order as above)
12, 12, 13, 12, 8, 11, 11, 10, 8, 14, 14, 14, 14, 12, 4, 12, 12, 4, 11, 10, 10, 10, 3, 9, 9, 8, 8, 8, 8, 7, 7, 6, 3, 6, 3, 2, 6, 2, 2, 5, 5, 5, 4, 4, 4, 2, 3, 3, 3, 3, 3, 3, 2, 1, 2, 2, 2, 1, 2, 1, 2, 2, 2, 2, 1, 1, 1, 1, 1, 1, 1, 1, 1, 1, 1, 1, 1, 1, 1, 1, 1

Gene ontology terms ranked according to mean of effect sizes in cluster:
centrosome | microtubule organizing center | microtubule nucleation | microtubule binding

Corresponding mean of effect sizes of each Gene ontology term:
(In the same order as above)
2.83, 1.78, 1.63, 1.59

Corresponding total number of genes of each Gene ontology term:
(In the same order as above)
243, 46, 42, 69

Corresponding number of genes of each Gene ontology term with at least one TFBS (of selected TFs):
(In the same order as above)
68, 11, 12, 22

Corresponding total number of TFBS (of selected TFs) for each Gene ontology term (in all genes):
(In the same order as above)
349, 84, 66, 108

Corresponding number of selected TFs each Gene ontology term is involved with:
(In the same order as above)
14, 14, 14, 14

TFs ranked according to mean of effect sizes in cluster:
V$CREB_01, V$CREBP1_01, V$CREBATF_Q6, V$CREBP1CJUN_01, V$ATF3_Q6, V$ATF1_Q6, V$CREB_Q4, V$CREBP1_Q2, VATF_B, VCREB_Q4_01, VATF_01, VCREB_Q2, V$CREB_Q2_01, V$CREB_02

Corresponding mean of effect sizes of each TF:
(In the same order as above)
3.04, 2.78, 2.60, 2.51, 2.37, 2.10, 1.93, 1.86, 1.75, 1.65, 1.45, 1.42, 1.23, 0.75

Corresponding total number of TFBS for each TF (genome-wide):
(In the same order as above)
659, 662, 671, 652, 663, 671, 671, 679, 665, 667, 660, 676, 679, 656

Corresponding total number of TFBS for each TF (in all genes in selected Gene ontology terms):
(In the same order as above)
53, 54, 49, 47, 48, 45, 41, 40, 42, 39, 39, 39, 35, 36

Corresponding number of genes (of selected Gene ontology terms) each TF is involved with:
(In the same order as above)
36, 38, 34, 31, 31, 31, 29, 28, 29, 28, 28, 27, 25, 26

Corresponding number of selected Gene ontology terms each TF is involved with:
(In the same order as above)
4, 4, 4, 4, 4, 4, 4, 4, 4, 4, 4, 4, 4, 4

Cluster for columns 589 to 612, rows 423 to 437

Gene ontology terms

meiotic recombination | synapsis | synaptonemal complex | pronucleus | male meiosis | diplotene | leptotene | zygotene | cell aging | senescence | dephosphorylation | hyperphosphorylation | centrosome duplication | mitotic cell cycle | cell cycle phase | negative regulation of cell cycle | positive regulation of cell cycle | regulation of meiotic cell cycle | regulation of mitotic cell cycle | nuclear membrane | nuclear inner membrane | nuclear lamina | nuclear envelope | nuclear pore

TFs

V$E2F_Q6_01 | V$E2F_Q4_01 | V$E2F_Q3_01 | V$E2F1_Q4_01 | V$E2F1_Q6_01 | V$E2F_03 | V$E2F4DP2_01 | V$E2F1DP2_01 | V$E2F1DP1_01 | V$E2F_Q6 | V$E2F_Q4 | V$E2F_Q3 | V$E2F4DP1_01 | V$E2F1DP1RB_01 | V$E2F_02

Information

All related TFs:
(List of all TFs that are related to any of the PWMs)
DP-1, E2F, E2F+E4, E2F-1, E2F-1:DP-1, E2F-1:DP-2, E2F-2, E2F-3a, E2F-4, E2F-4:DP-1, E2F-4:DP-2, E2F-5, E2F-7, pRb:E2F-1:DP-1

Ranked gene list:
(All genes of the selected Gene ontology terms with hits of any of the selected TFs, ranked according to the total number of TFBS)
RBL1, CDKN1B, CCND1, FOXO3, CDKN1A, CDC25B, E2F1, CDK6, PCNA, MDM2, AKT1, H2AFX, LMNB1, CDC25A, CDC2, CTDSP1, CDC6, ERBB2IP, JUN, SMC3, MCM2, E2F3, C2orf28, NUP155, DMC1, PIK3CA, CDK5, CCND2, MCM7, CD151, TP53, SKP2, NCL, MYCN, GEMIN4, TMPO, STMN1, PIN1, SLC39A1, BUB3, PSMD9, NOLC1, ATAD2, LMNB2, RB1, NOL3, CDKN2C, RASSF1, MCM5, IRS2, DPYSL2, TP73, MLH1, LMOD1, NUTF2, ISCU, CCNB1IP1, PPM1D, GSK3A, EXO1, BCL2L11, CASP6, VIM, LOXL3, JUNB, HSPA2, RANBP1, MCM3, LIG4, SHC1, NUP153, MXI1, MORF4L1, IRS1, EZH2, CDC7, CBX5, TFDP1, PTPRU, ID2, CYCS, SFRS2, HNRNPC, EGR1, CFL1, BMI1, ZMYND10, TRAF3, PRPF19, LEMD3, HIST1H4I, HIST1H1B, FASN, E2F2, CBX3, ANXA5, TFAP2A, SYCP2, MAF1, LIG1, KPNB1, HES1, NAB2, LRDD, EVX2, EIF5A, CRTC2, CAMK2G, UBB, SUMO1, STAG1, SSSCA1, RPA2, PRKDC, PPP1R8, PAICS, NUP43, NUMA1, MEI1, MARCKSL1, ID3, DOK1, DNAJA3, CDT1, ARRB2, LRBA, FUSIP1, CUX1, CHEK1, CDC25C, CD68, YWHAQ, SF3B1, PTPN6, NPM1, NFATC1, ING3, HELLS, GORASP2, EMD, DUSP4, CAST, CALM2, ARID4A, UBTF, STAT3, SLC39A2, PROC, MYC, MND1, METAP2, MCM4, GMNN, DTYMK, DEK, CDKN1C, ANP32A, SYCE2, SMARCB1, PCNT, PCGF2, NIN, NBN,

MBTPS1, MAPT, HIST4H4, GLTSCR2, FOXM1, FBXL10, DUSP1, CD47, CAMKK1, ZMYND11, TRPT1, RHEBL1, RFC3, RAD51, POLR2A, MTHFR, IPO7, ZFYVE20, XRCC6, TGFB1I1, SP1, SMC1A, SIRT6, RAD54L, RAD51C, PSMC5, NR3C1, MSH3, MMACHC, IGF1R, HMGB2, GCA, FBRS, EIF5, CSK, CDKN2D, CDK5R2, ATP6V0D1, AKT2, ADK, WAPAL, UBE2I, SLC25A1, RFC1, NFATC4, MAD2L1BP, ICMT, GSPT1, CTNNB1, XPC, SYNE2, SMARCA4, SLC7A1, SLC39A4, SHISA5, RARA, RAG1AP1, RAC1, PPM1G, POLE4, PKMYT1, PER1, NFATC3, MUS81, MSH2, MEF2D, MAP3K11, ENTPD2, DUSP10, CUL1, CETN3, CDK5R1, CASP8AP2, BRSK2, BRAF, BCL2L1, APP, ANXA4, ALCAM, ACP1, YBX2, XRCC5, SMG5, SH3BP2, SFRS10, REL, RCAN1, PSMP, PRKCZ, PPP1R16B, PCMT1, NUP160, NF2, MAP3K7, HRAS, HNRNPUL1, GTF2F2, GTF2F1, FIS1, DYSF, DUSP22, CYLD, CTDP1, CLIC4, CDK8, CASP2, CALM1, CABIN1, APOE, WNK1, TNK2, TGFB3, TBX2, STAT1, SASS6, RHEB, RAD51L1, PPP2CA, PPM1B, PHF20, PHB, PDPK1, NR2C2, MGC20604, MAPK12, ITPR1, ING1, HMGA2, HIRA, GSK3B, DUSP6, DNMT3A, CXCR4, CTTN, CSNK2A1, CENPK, CCNE1, CARHSP1, CADM1, ATP2A3, APOA2, ALOX5, ZBTB16, UBL4A, STAG2, SLC36A1, SLC31A1, RPL31, RANBP9, PTPRA, PRKG1, PPP5C, PMS1, PHF1, PDXK, NOTCH1, MRLC3, MDC1, KRT8, KIAA0419, ITPKB, GRK5, FBXL11, DLST, DKFZp779C0757, CSTF2T, CSNK2B, CLK1, ACHE

Corresponding total number of TFBS:
(For each gene listed above, the total number of TFBS for any of the selected TFs, multiplied by the number of selected Gene ontology terms containing that gene)
150, 150, 130, 120, 120, 104, 99, 99, 90, 90, 90, 78, 75, 72, 72, 66, 66, 65, 64, 60, 60, 60, 60, 56, 49, 48, 48, 48, 45, 45, 44, 44, 44, 44, 42, 40, 40, 40, 39, 39, 36, 36, 36, 35, 33, 32, 32, 30, 30, 30, 30, 28, 28, 28, 27, 27, 27, 26, 26, 26, 26, 25, 24, 24, 24, 24, 22, 21, 21, 20, 20, 20, 20, 20, 20, 20, 20, 18, 18, 18, 18, 16, 16, 16, 16, 16, 15, 15, 15, 15, 15, 15, 15, 15, 15, 15, 14, 14, 14, 14, 14, 14, 13, 13, 13, 13, 13, 13, 12, 12, 12, 12, 12, 12, 12, 12, 12, 12, 12, 12, 12, 12, 12, 12, 11, 11, 11, 11, 11, 11, 10, 10, 10, 10, 10, 10, 10, 10, 10, 10, 10, 10, 10, 9, 9, 9, 9, 9, 9, 9, 9, 9, 9, 9, 9, 9, 8, 8, 8, 8, 8, 8, 8, 8, 8, 8, 8, 8, 8, 8, 8, 7, 7, 7, 7, 7, 7, 7, 7, 6, 6, 6, 6, 6, 6, 6, 6, 6, 6, 6, 6, 6, 6, 6, 6, 6, 6, 6, 6, 6, 6, 5, 5, 5, 5, 5, 5, 5, 5, 5, 4, 4, 4, 4, 4, 4, 4, 4, 4, 4, 4, 4, 4, 4, 4, 4, 4, 4, 4, 4, 4, 4, 4, 4, 4, 4, 4, 4, 4, 3, 3, 3, 3, 3, 3, 3, 3, 3, 3, 3, 3, 3, 3, 3, 3, 3, 3, 3, 3, 3, 3, 3, 3, 3, 3, 3, 3, 3, 2, 2, 2, 2, 2, 2, 2, 2, 2, 2, 2, 2, 2, 2, 2, 2, 2, 2, 2, 2, 2, 2, 2, 2, 2, 2, 2, 2, 2, 2, 2, 1, 1, 1, 1, 1, 1, 1, 1, 1, 1, 1, 1, 1, 1, 1, 1, 1, 1, 1, 1, 1, 1, 1, 1, 1, 1, 1, 1

Corresponding number of selected Gene ontology terms each gene is involved with:
(In the same order as above)
10, 10, 10, 8, 12, 8, 9, 11, 6, 9, 10, 13, 5, 8, 18, 6, 6, 5, 8, 5, 4, 5, 4, 4, 7, 8, 4, 4, 3, 3, 11, 4, 4, 4, 3, 5, 4, 4, 3, 3, 12, 9, 4, 5, 11, 8, 4, 2, 2, 2, 2, 2, 7, 7, 3, 3, 9, 2, 2, 2, 2, 5, 8, 2, 3, 3, 2, 3, 3, 2, 5, 4, 2, 2, 2, 4, 4, 3, 2, 2, 3, 4, 4, 2, 4, 2, 1, 5, 1, 5, 1, 1, 3, 1, 1, 3, 1, 2, 2, 2, 2, 1, 1, 1, 1, 1, 1, 1, 12, 4, 1, 12, 1, 4, 2, 1, 4, 4, 3, 2, 1, 1, 1, 1, 1, 1, 1, 1, 11, 11, 1, 2, 2, 2, 5, 1, 2, 1, 2, 5, 1, 2, 1, 1, 1, 3, 3, 3, 9, 3, 1, 3, 1, 1, 3, 3, 1, 2, 2, 2, 1, 2, 4, 1, 2, 1, 2, 2, 1, 2, 1, 1, 1, 1, 1, 1, 7, 1, 1, 1, 1, 2, 2, 3, 6, 2, 3, 2, 2, 2, 1, 1, 1, 1, 2, 2, 6, 2, 2, 3, 2, 1, 1, 1, 5, 1, 1, 1, 1, 1, 1, 5, 1, 2, 1, 1, 2, 1, 4, 1, 2, 1, 1, 2, 1, 1, 1, 1, 2, 2, 1, 1, 4, 1, 2, 1, 2, 1, 4, 2, 1, 1, 1, 1, 3, 1, 1, 1, 1, 3, 3, 1, 1, 1, 1, 1, 1, 3, 1, 1, 1, 1, 1, 1, 1, 1, 1, 1, 1, 1, 1, 2, 1, 1, 2, 1, 1, 1, 1, 2, 1, 1, 2, 2, 1, 1, 2, 2, 2, 1, 2, 2, 2, 1, 1, 2, 2, 2, 1, 1, 2, 1, 1, 2, 1, 1, 1, 1, 1, 1, 1, 1, 1, 1, 1, 1, 1, 1, 1, 1, 1, 1, 1, 1, 1, 1, 1, 1, 1, 1, 1

Corresponding number of selected TFs each gene is involved with:
(In the same order as above)
15, 15, 13, 15, 10, 13, 11, 9, 15, 10, 9, 6, 15, 9, 4, 11, 11, 13, 8, 12, 15, 12, 15, 14, 7, 6, 12, 12, 15, 15, 4, 11, 11, 11, 14, 8, 10, 10, 13, 13, 3, 4, 9, 7, 3, 4, 8, 15, 15, 15, 15, 14, 4, 4, 9, 9, 3, 13, 13, 13, 13, 5, 3, 12, 8, 8, 11, 7, 7, 10, 4, 5, 10, 10, 10, 5, 5, 6, 9, 9, 6, 4, 4, 8, 4, 8, 15, 3, 15, 3, 15, 15, 5, 15, 15, 5, 14, 7, 7, 7, 7, 14, 13, 13, 13, 13, 13, 13, 1, 3, 12, 1, 12, 3, 6, 12, 3, 3, 4, 6, 12, 12, 12, 12, 12, 11, 11, 11, 1, 1, 11, 5, 5, 5, 2, 10, 5, 10, 5, 2, 10, 5, 10, 10, 9, 3, 3, 3, 1, 3, 9, 3, 9, 9, 3, 3, 9, 4, 4, 4, 8, 4, 2, 8, 4, 8, 4, 4, 8, 4, 8, 8, 7, 7, 7, 7, 1, 7, 7, 7, 6, 3, 3, 2, 1, 3, 2, 3, 3, 3, 6, 6, 6, 6, 3, 3, 1, 3, 3, 2, 3, 6, 6, 5, 1, 5, 5, 5, 5, 5, 5, 1, 4, 2, 4, 4, 2, 4, 1, 4, 2, 4, 4, 2, 4, 4, 4, 4, 2, 2, 4, 4, 1, 4, 2, 4, 2, 4, 1, 2, 4, 4, 4, 3, 1, 3, 3, 3, 3, 1, 1, 3, 3, 3, 3, 3, 3, 1, 3, 3, 3, 3, 3, 3, 3, 3, 3, 3, 3, 3, 3, 1, 2, 2, 1, 2, 2, 2, 2, 1, 2, 2, 1, 1, 2, 2, 1, 1, 1, 2, 1, 1, 1, 2, 2, 1, 1, 1, 2, 2, 1, 2, 2, 1, 1, 1, 1, 1, 1, 1, 1, 1, 1, 1, 1, 1, 1, 1, 1, 1, 1, 1, 1, 1, 1, 1, 1, 1, 1

Gene ontology terms ranked according to mean of effect sizes in cluster:

cell cycle phase | senescence | synapsis | meiotic recombination | regulation of mitotic cell cycle | negative regulation of cell cycle | positive regulation of cell cycle | mitotic cell cycle | nuclear pore | regulation of meiotic cell cycle | diplotene | synaptonemal complex | nuclear envelope | cell aging | nuclear membrane | leptotene | nuclear lamina | zygotene | nuclear inner membrane | centrosome duplication | hyperphosphorylation | dephosphorylation | pronucleus | male meiosis

Corresponding mean of effect sizes of each Gene ontology term:
(In the same order as above)
3.22, 3.01, 2.42, 2.39, 2.30, 2.15, 2.00, 1.93, 1.85, 1.79, 1.78, 1.64, 1.63, 1.62, 1.51, 1.36, 1.33, 1.26, 1.17, 0.98, 0.97, 0.95, -0.03, -0.05

Corresponding total number of genes of each Gene ontology term:
(In the same order as above)
104, 327, 83, 144, 116, 87, 86, 71, 238, 101, 32, 70, 203, 182, 110, 36, 70, 24, 56, 72, 351, 689, 22, 48

Corresponding number of genes of each Gene ontology term with at least one TFBS (of selected TFs):
(In the same order as above)
42, 89, 24, 36, 39, 33, 32, 22, 55, 34, 12, 18, 43, 52, 30, 13, 19, 7, 18, 24, 91, 153, 4, 8

Corresponding total number of TFBS (of selected TFs) for each Gene ontology term (in all genes):
(In the same order as above)
287, 578, 143, 223, 262, 225, 211, 134, 365, 214, 63, 108, 270, 282, 175, 68, 109, 45, 105, 121, 504, 880, 15, 26

Corresponding number of selected TFs each Gene ontology term is involved with:
(In the same order as above)
15, 15, 15, 15, 15, 15, 15, 15, 15, 15, 15, 15, 15, 15, 15, 15, 15, 15, 15, 15, 15, 15, 11, 12

TFs ranked according to mean of effect sizes in cluster:
V$E2F_Q6_01, V$E2F_03, V$E2F1DP1RB_01, V$E2F_02, V$E2F4DP1_01, V$E2F_Q3, V$E2F1_Q6_01, V$E2F1_Q4_01, V$E2F_Q3_01, V$E2F_Q4_01, V$E2F4DP2_01, V$E2F1DP1_01, V$E2F_Q4, V$E2F1DP2_01, V$E2F_Q6

Corresponding mean of effect sizes of each TF:
(In the same order as above)
2.31, 2.15, 2.10, 1.94, 1.84, 1.70, 1.67, 1.64, 1.63, 1.54, 1.40, 1.27, 1.22, 1.09, 1.00

Corresponding total number of TFBS for each TF (genome-wide):
(In the same order as above)
686, 688, 662, 658, 657, 688, 700, 681, 690, 667, 656, 682, 702, 658, 699

Corresponding total number of TFBS for each TF (in all genes in selected Gene ontology terms):
(In the same order as above)
429, 416, 360, 358, 338, 372, 378, 366, 367, 354, 327, 347, 355, 309, 337

Corresponding number of genes (of selected Gene ontology terms) each TF is involved with:
(In the same order as above)
134, 141, 129, 127, 123, 129, 128, 127, 128, 120, 122, 125, 126, 116, 116

Corresponding number of selected Gene ontology terms each TF is involved with:
(In the same order as above)
24, 24, 24, 24, 24, 24, 24, 24, 24, 24, 22, 22, 24, 23, 22

Cluster for columns 50 to 53, rows 50 to 64

Gene ontology terms

nucleosome assembly | heterochromatin | chromatin remodeling | nucleosome

TFs

V$TBP_Q6 | V$NKX62_Q2 | V$OTX_Q1 | V$PBX1_01 | V$SRY_01 | V$HMGIY_Q3 | V$OCT1_06 | V$TBP_01 | V$TEF_Q6 | V$FOXJ2_02 | V$AFP1_Q6 | V$OCT1_03 | V$SRY_02 | V$FOXO1_01 | V$FOXO3A_Q1

Information

All related TFs:
(List of all TFs that are related to any of the PWMs)
AFP1, FOXJ2, FOXO1, HMG, HMG-Y, HMGI-C, Nkx6-2, Oct-1, Otx1, Otx2, POU2F1, POU2F1a, Pbx1a, SRY, TBP, TEF, TEF-xbb1, TFIID

Ranked gene list:
(All genes of the selected Gene ontology terms with hits of any of the selected TFs, ranked according to the total number of TFBS)
HIST1H4I, HIST1H3E, CBX5, SATB1, HIST1H3C, CHD1, SMARCA2, IKZF3, HIST4H4, NR3C1, IKZF1, BMI1, TIPARP, HIST1H2AE, HIST1H1E, PXMP3, H2AFZ, SMARCE1, H3F3B, TBP, ANP32A, SETD7, ISCU, SUPT3H, ZNF238, UBE2B, RUNX1, PCNA, NF1, KAT2B, ETS1, CHD2, CBX3, TES, HIST1H2AM, BUB3, MAF, JMJD1A, HIST1H3H, HIST1H3B, BCL9, ATF7IP, ARNTL, ARID1B, ARID1A, WNK1, MXI1, HIST1H1A, ESR1, CTNNB1, MLL, HNRNPK, TRERF1, TBR1, STAT5B, SOX2, RUNX2, NFYC, NFYA, HIST1H1D, H3F3A, GTF2A1, GATA3, CREB1, CHD3, SLC38A2, POU2F1, NCOA3, ADNP, TLK1, TBPL1, STAT1, SMARCA5, HMGN4, HIST2H2BE, HIST2H2AC, HIST1H4C, HIST1H1C, H2AFX, CENPC1, PSMA3, HIST1H2BO, HIST1H2BB, BAZ2A, ARID4A, ACTR1A, HDAC9, EP300, CHD4, ADIPOR1, ING3, HIST1H3F, EED, DNAJB9, SP1, SATB2, MGEA5, IPMK, HIST2H2AA3, HELLS, FOS, DICER1, CREM, CDC2, ASXL1, XRCC5, UBC, RBBP4, NAP1L4, MSL2, MCM3, MBD2, HMGB1, HIST2H3C, HIST1H1T, HDAC4, H2AFY, CTCF, CHD8, BDNF, UBE3A, TCHP, SUPT16H, SS18, SMARCB1, SETDB2, RPS6KA5, REST, PRKCH, OPTN, MYC, MRE11A, MORF4L1, JMJD1B, HIST1H2BC, HDAC7, HAT1, GZMA, GTF3A, GTF2I, GTF2B, DEK, CREBBP, COL11A2, BTAF1, ATR, ACTR8, ZBTB17, WAPAL, UBE2L6, TAF9, TACC3, SRA1, SALL1, RARA, RAC3, PTMA, POT1, PAK3, NUF2, NPM3, NAP1L1, MRFAP1, MBD1, LTB, LSM2, LMNA, LBX1, JUN, INVS, ING2, IL23A, HSP90AA1, HIST1H3G, HABP4, GTF2F2, FHIT, EZH1, DYNLL1, CHORDC1, CDKN1A, CDK2, CD4, C11orf30, BRMS1L, ASH1L, APBB3

Corresponding total number of TFBS:
(For each gene listed above, the total number of TFBS for any of the selected TFs, multiplied by the number of selected Gene ontology terms containing that gene)
60, 60, 52, 45, 44, 40, 39, 39, 36, 30, 30, 30, 28, 26, 26, 24, 24, 22, 22, 20, 20, 18, 18, 16, 15, 15, 15, 15, 15, 15, 15, 15, 15, 14, 14, 14, 13, 13, 13, 13, 13, 13, 13, 13, 13, 12, 12, 12, 12, 12, 11, 11, 10, 10, 10, 10, 10, 10, 10, 10, 10, 10, 10, 10, 10, 9, 9, 9, 9, 8, 8, 8, 8, 8, 8, 8, 8, 8, 8, 8, 7, 7, 7, 7, 7, 7, 6, 6, 6, 6, 5, 5, 5, 5, 4, 4, 4, 4, 4, 4, 4, 4, 4, 4, 4, 3, 3, 3, 3, 3, 3, 3, 3, 3, 3, 3, 3, 3, 3, 3, 2, 2, 2, 2, 2, 2, 2, 2, 2, 2, 2, 2, 2, 2, 2, 2, 2, 2, 2, 2, 2, 2, 2, 2, 2, 2, 2, 1, 1, 1, 1, 1, 1, 1, 1, 1, 1, 1, 1, 1, 1, 1, 1, 1, 1, 1, 1, 1, 1, 1, 1, 1, 1, 1, 1, 1, 1, 1, 1, 1, 1, 1, 1, 1, 1

Corresponding number of selected Gene ontology terms each gene is involved with:
(In the same order as above)
4, 4, 4, 3, 4, 4, 3, 3, 4, 2, 2, 2, 2, 2, 2, 2, 4, 2, 2, 4, 2, 2, 2, 2, 1, 1, 1, 3, 1, 3, 1, 1, 1, 1, 2, 1, 1, 1, 1, 1, 1, 1, 1, 1, 1, 1, 1, 1, 1, 2, 1, 1, 1, 1, 1, 1, 1, 1, 1, 1, 1, 2, 2, 1, 2, 2, 1, 1, 1, 1, 1, 2, 1, 4, 1, 4, 4, 4, 1, 4, 2, 1, 1, 1, 1, 1, 1, 3, 3, 2, 2, 1, 1, 1, 1, 2, 1, 4, 1, 4, 2, 2, 1, 1, 1, 1, 1, 1, 3, 1, 1, 1, 3, 3, 3, 3, 1, 3, 3, 1, 1, 1, 2, 2, 1, 2, 1,

2, 1, 1, 1, 2, 1, 1, 1, 1, 1, 2, 1, 2, 1, 2, 1, 2, 2, 1, 2, 2, 1, 1, 1, 1, 1, 1, 1, 1, 1, 1, 1, 1, 1, 1, 1, 1, 1, 1, 1, 1, 1, 1, 1, 1, 1, 1, 1, 1, 1, 1, 1, 1, 1, 1, 1, 1, 1, 1

Corresponding number of selected TFs each gene is involved with:
(In the same order as above)
15, 15, 13, 15, 11, 10, 13, 13, 9, 15, 15, 15, 14, 13, 13, 12, 6, 11, 11, 5, 10, 9, 9, 8, 15, 15, 15, 5, 15, 5, 15, 15, 15, 14, 7, 14, 13, 13, 13, 13, 13, 13, 13, 13, 13, 12, 12, 12, 6, 12, 11, 11, 10, 10, 10, 10, 10, 10, 10, 10, 5, 5, 10, 5, 5, 9, 9, 9, 9, 8, 4, 8, 2, 8, 2, 2, 2, 8, 2, 4, 7, 7, 7, 7, 7, 7, 2, 2, 3, 3, 5, 5, 5, 5, 2, 4, 1, 4, 1, 2, 2, 4, 4, 4, 4, 3, 3, 1, 3, 3, 3, 1, 1, 1, 1, 3, 1, 1, 3, 3, 2, 1, 1, 2, 1, 2, 1, 2, 2, 2, 1, 2, 2, 2, 2, 2, 1, 2, 1, 2, 1, 2, 1, 1, 2, 1, 1, 1, 1, 1, 1, 1, 1, 1, 1, 1, 1, 1, 1, 1, 1, 1, 1, 1, 1, 1, 1, 1, 1, 1, 1, 1, 1, 1, 1, 1, 1, 1, 1, 1, 1, 1, 1, 1, 1, 1, 1

Gene ontology terms ranked according to mean of effect sizes in cluster:
heterochromatin | nucleosome assembly | chromatin remodeling | nucleosome

Corresponding mean of effect sizes of each Gene ontology term:
(In the same order as above)
3.09, 2.96, 2.66, 2.59

Corresponding total number of genes of each Gene ontology term:
(In the same order as above)
196, 83, 410, 190

Corresponding number of genes of each Gene ontology term with at least one TFBS (of selected TFs):
(In the same order as above)
60, 38, 133, 67

Corresponding total number of TFBS (of selected TFs) for each Gene ontology term (in all genes):
(In the same order as above)
386, 201, 720, 361

Corresponding number of selected TFs each Gene ontology term is involved with:
(In the same order as above)
15, 15, 15, 15

TFs ranked according to mean of effect sizes in cluster:
V$FOXO3A_Q1, V$TBP_01, VTBP_Q6, VOTX_Q1, VSRY_02, VPBX1_01, V$OCT1_03, V$SRY_01, V$FOXO1_01, V$OCT1_06, V$NKX62_Q2, V$FOXJ2_02, V$AFP1_Q6, V$TEF_Q6, V$HMGIY_Q3

Corresponding mean of effect sizes of each TF:
(In the same order as above)
4.11, 4.06, 3.37, 3.32, 3.06, 2.96, 2.95, 2.94, 2.85, 2.67, 2.36, 2.20, 2.16, 1.91, 1.45

Corresponding total number of TFBS for each TF (genome-wide):
(In the same order as above)
689, 685, 723, 701, 693, 704, 704, 743, 680, 717, 698, 680, 682, 684, 716

Corresponding total number of TFBS for each TF (in all genes in selected Gene ontology terms):
(In the same order as above)
130, 125, 126, 118, 121, 116, 110, 125, 105, 113, 99, 98, 95, 92, 95

Corresponding number of genes (of selected Gene ontology terms) each TF is involved with:
(In the same order as above)
78, 74, 80, 74, 73, 75, 66, 77, 65, 71, 61, 63, 61, 57, 61

Corresponding number of selected Gene ontology terms each TF is involved with:
(In the same order as above)
4, 4, 4, 4, 4, 4, 4, 4, 4, 4, 4, 4, 4, 4, 4

Cluster for columns 1094 to 1122, rows 23 to 42

Gene ontology terms

polyamine biosynthetic process | polyamine metabolic process | RNA catabolic process | mitochondrial DNA replication | leaf senescence | mRNA transport | nucleocytoplasmic transport | nuclear-transcribed mRNA catabolic process, nonsense-mediated decay | polysome | mRNA binding | eukaryotic translation initiation factor 3 complex | translation | translational initiation | mRNA catabolic process | nucleic acid binding | RNA biosynthetic process | RNA transport | nucleolus organizer region | rRNA transcription | rRNA processing | ribosome assembly | ribosome biogenesis | small ribosomal subunit | translational elongation | eukaryotic translation initiation factor 2 complex | regulation of translation | ribosome binding | ribosomal subunit | ribosome

TFs

V$YY1_Q6_02 | V$YY1_Q6 | V$NFMUE1_Q6 | V$YY1_02 | V$GABP_B | V$ELK1_02 | V$TAXCREB_01 | V$ATF6_01 | V$HLF_01 | V$E4BP4_01 | V$USF_01 | V$MAX_01 | V$USF_Q6 | V$USF_02 | V$USF_Q6_01 | V$MYCMAX_03 | V$ARNT_01 | V$MYC_Q2 | V$CLOCKBMAL_Q6 | V$MYCMAX_02

Information

All related TFs:
(List of all TFs that are related to any of the PWMs)
ATF6, Arnt, CREB, Clock:BMAL, Clock:BMAL1, Clock:BMAL2, E4BP4, Elk-1, Elk-1-isoform1, GABP-alpha, GABP-alpha:GABP-beta, GABP-beta1, GABP-beta2, Hlf, Max, Max1, NF-muE1, Tax, USF, USF-1, USF1, USF1:USF2, USF1a, USF1b, USF2, USF2a, USF2b, YY1, c-Myc, deltaCREB, factor

Ranked gene list:
(All genes of the selected Gene ontology terms with hits of any of the selected TFs, ranked according to the total number of TFBS)
EIF5A, EIF4G1, HNRPDL, EIF3A, KIAA0664, EIF4A1, NPM1, UBTF, HNRNPA1, NCL, PER1, EIF4E, DDX5, RPS15, ZFP36, ATF4, RPS2, PTBP1, ODC1, EEF1A1, EIF3B, ANXA6, PCBP1, ILF3, EIF5, EIF4A2, GAPDH, NP, KHDRBS1, GIT1, AKT1, ACTB, SYNCRIP, EIF1, CIRBP, SQSTM1, CDK5R1, ADAM8, UBA52, SSR1, RPL13A, PROC, CRTC2, CALR, SFRS2, RPL3, NR1H2, MKNK2, KHSRP, HNRNPL, GNB2L1, EEF1B2, CCND1, SLC20A1, EEF2, YBX1, XPO1, UBB, SLC2A4RG, RPS5, UPF1, STXBP1, SART3, RPL23A, RPL13, CSDE1, ATP5E, TARBP2, SRM, SLBP, RPL11, NR3C1, JUN, HSPA8, HDLBP, GTF3A, GCN1L1, ETF1, CUX1, AMD1, RHBDL1, MTG1, USP7, RPS27A, RPL14, RCC1, MVK, IRF9, EIF3K, DOHH, ATF1, UTS2R, TSC2, TFRC, RPS18, NECAB3, ING1, DDIT3, CDK4, BNIP1, ATP5A1, ABL2, UBC, HMHA1, ERCC6, ELAVL1, QTRT1, OSM, FASN, EEF1D, DPH2, DPH1, CISH, UPF2, THNSL1, TCEA2, TBX1, SLC25A1, RPS6KA1, RPS24, RPS21, RPN1, RPLP0, RORC, RNH1, PKN1, NXT1, NUP98, NOL5A, NCOA6, MPG, IRS1, HNRNPC, ERF, EMG1, EEF2K, EBNA1BP2, CRIP1, ARHGEF2, ZFP36L1, HOXA7, CFL1, ADK, ACTG1, XPOT, TYMP, TUFM, TAF1C, SUPV3L1, RPS16, RERE, PDGFB, OAZ1, NAPRT1, MTHFR, GPX1, GGT1, EPB41, DDX21, CSDA, CARS2, BAX, ATAD2, RPS6KB1, RPL10, MGC111011, MARCKSL1, KAT5, JUNB, EXOSC8, BBS9, ARRB2, APEX1, VARS, SPSB1, SLC36A1, SLC16A1, SECISBP2, SBNO2, RPS8, RPS28, RPS19, RPL23, RHOF, POLRMT, POLR2A, POLG2, PNPT1, PCK2, P4HB, MRPL28, GUSB, GPS1, FARSA, EIF6, EIF4H, EIF4G2, EIF2A, DDX6, CTRL, CTF1,

CSNK2A1, BOP1, ATF6, ADAR, SUMO3, STAT5A, SSSCA1, PSMC4, NME2, MAT2A, HNRNPK, GTF2A1, FBL, ENOPH1, EDC4, DYM, YBX2, TSHZ1, TFB2M, SRRM1, SLC38A2, RPS23, RPS12, RPL36, RPL35A, RPL30, RPL24, RPL22, RPAIN, RPA1, RBM19, RBM15, RARA, RABGEF1, PURA, PTK2B, PSMD8, PSMC5, POLD3, PARN, PABPN1, NFE2L2, NCBP2, MPHOSPH10, MKNK1, MGC126674, LTB, LONP1, JAG2, ISYNA1, INTS6, IGF2BP1, HSPD1, HNRNPD, GLTSCR2, GFM1, FUS, FHL1, FBXO32, FAM60A, FAM49B, FAM168B, ESCO2, DHPS, DCP1B, CTSD, COTL1, CORO1A, CEBPE, CDKN2C, CCNH, CCNB1, CASP9, C1QBP, C14orf4, BRCA2, ARID5A, APRT, APC, ALG1, WDR77, UQCRH, TGFB1, TERT, TAF8, SLC33A1, SH3BP4, RPSA, RPS3, RPS14, PRKCZ, PGK1, PDAP1, PCNA, NOL3, NAB2, MVP, MARCH8, MAPK1, LIF, JMJD6, HSP90B1, GEMIN4, FMR1, ERP29, EIF4G3, EIF3G, EIF3E, EEFSEC, DHFR, DGCR2, CYTH2, CPEB1, CANX, C2orf28, VIM, TRIM25, TPP1, TNPO1, TNFSF13, TERF2IP, TAF15, SUMO1, STAT5B, STAT3, SSBP1, SNRPA, SKIV2L, SHMT2, SAT1, SAMD4A, S100A10, RPLP1, RPL32, RPL26, RPL18, RPL17, RBM3, RAF1, PTPN11, PTBP2, POLR2G, POLE4, PECI, PCBP2, NUP62, NRF1, NFKBIA, MYB, MXD1, MX1, MORF4, MBNL1, MARVELD2, MARS, MAPKAPK2, MAPK3, IRF1, IL6R, IGF1R, HSPC120, HSP90AA1, HMOX1, HIPK3, GRIN1, GPX4, GNL2, GCDH, GAR1, G3BP1, FOS, FKBP4, EXOSC4, ETV5, EIF4EBP2, EIF3H, DUSP1, DTYMK, DKC1, DHODH, DGAT1, DDX19B, DDX1, DCP1A, CLN3, CASC3, CALM1, C14orf172, BXDC1, BAG3, ATF5, ASCL1, ARID2, ANP32A, XIAP, TRMT6, TOP1, TNFRSF14, TFB1M, TEF, TAT, STK11, SRPR, SRP54, SMG7, SCARF2, RRAS, RPP30, RPL9, RPL15, RHEB, REXO2, RELA, REL, RANBP1, PSMP, PRNP, PRKG1, PRKAR1A, PRAP1, PPY, POLG, POLDIP3, POLA1, PLA2G1B, PIK3R2, NUP88, NUP210, NSFL1C, NRAS, NR4A1, NR3C2, NLRP3, NKX6-2, NARS, MSTN, MSL2, MGC111084, MDM2, MCAT, MAN2C1, LIN28, LAT, LASS2, IMP4, IL2, IL13, HMGA1, HGFAC, GDE1, FTL, FTH1, ELL, EIF4E2, EIF3I, EGR1, EDC3, DLG4, CYCS, CYC1, COPA, COL9A3, CLEC11A, CHRM1, CDKN2D, CDKN1C, CASP3, BCL2L11, BCKDK, ATP6V0D1, ATG5, ZNRD1, ZFYVE9, WDR61, WDR48, WARS, TXNRD1, TXNDC15, TRIM37, TRIM3, TRIM21, TPT1, TPI1, TNFRSF1A, TNF, TMEM201, TK2, TGFBR2, TGFB2, TG, TFAP2A, TCF3, SYF2, STAT6, STAT1, SRP9, SRP19, SPG7, SPAG9, SNRPG, SMG6, SLC31A1, SLC30A9, SKIL, SERBP1, RTN4, RPS6KB2, RPN2, RPL6, RPL41, RNPS1, RNF39, RELB, RCL1, RARS2, PTCHD3, PRMT3, PRKAB1, PPAT, POLR2B, PIK3C3, PHAX, PDGFA, PDF, PDCD11, PABPC4, OSBP, OBFC2B, NXF1, NT5C2, NR4A3, NPM3, NOS2, NOP10, NOLC1, NME1, NFIL3, NDOR1, MTR, MTIF3, MOCOS, MKI67IP, METTL3, MCL1, MBP, MARS2, MAD1L1, LSM14A, LIN9, KTN1, KRR1, KARS, JUND, IVNS1ABP, ISG15, IMP3, IMMP1L, IL27, IKBKAP, IGF2, IARS, HSP90AB1, HSF1, HOXD13, HIST1H4I, HIST1H4C, HIST1H3F, HIST1H3C, HIRA, HIF1A, HEXIM2, GTPBP3, GRIA1, GADD45A, FTSJ1, FEN1, FDXR, FAU, FARS2, ELF2, EIF5A2, EIF4ENIF1, EIF4EBP3, EIF2B3, EIF2B2, EIF2B1, DTD1, DR1, DPH3, DNAJC5, DNAJC2, DNAJB9, DNAJB11, DNAJB1, DICER1, DHRS11, DDX3X, DDX25, DCTN3, DAP3, DAP, CUGBP2, CUGBP1, CSRP1, CSF2, CRTC1, CPSF3, CPE, COPS5, COL6A2, COL6A1, COL2A1, COL11A2, COIL, CNBP, CLPP, CDKN1B, CD44, C10orf27, C10orf2, ATP7A, ATF2, ARNT, AIRE, AHR, ZNF74, ZNF167, ZC3H7B, YRDC, YAP1, XPC, XPA, WT1, WNK1, WDR36, VDAC1, UGT8, TYRO3, TRNT1, TPX2, TNFSF10, TKT, TEAD3, TEAD2, TCOF1, TBP, TAS1R1, TAF9, SUMO2, STK19, SRP68, SRGN, SPTB, SPECC1, SPARC, SOS1, SLC5A10, SLC3A2, SLC25A4, SLC25A19, SFRS5, SERPINH1, SERPINB8, RPP38, RPL34, RPH3AL, RHOBTB2, RHOA, RARS, RANGRF, RANGAP1, RANBP3, RANBP2, PSMD14, PSMC2, PRG2, PRDX5, PPP2R4, PPAN, POM121, PLAA, PIK3R1, PET112L, PDLIM4, PARVA, PAK1IP1, P11, OBFC2A, NUTF2, NUP214, NTRK2, NT5C, NRSN1, NPTXR, NPTN, NPPA, NPC1, NOL12, NIP7, NHP2, NGLY1, NFE2, NEU1, NCOA5, NAF1, MRE11A, MMP3, MMP13, MIPEP, MANBA, LGALS1, LBX1, LARS2, KLF3, JAK2, ITPR3, IPO4, IMMP2L, IL6ST, HSPA9, HLCS, HIVEP3, HIST1H3H, HIST1H3G, HBEGF, HBA2, GZMM, GTF2A2, GSPT2, GRN, GLUD1, GLS, GAST, GARS, FUBP1, FOXK1, FOSL1, FLJ21865, FGFR1, FBF1, FAS, FAP, EXOSC9, ERCC3, EIF4E3, EIF2C4, EARS2, DYNC1H1, DOM3Z, DNAJC24, DKFZp686G1042, DHX15, DDX47, DDC, CWC15, CPS1, CP, CIB1, CHPF, CHERP, CGN, CDK7, CDC25C, CD47, CD46, CD3EAP, CAST, CASP7, CAPNS1, CABP1, C1orf102, C18orf10, AZI1, AUH, ATR, ATF3, ARG2, ARG1, APOE, ANXA5, ACSL5, ACLY

Corresponding total number of TFBS:
(For each gene listed above, the total number of TFBS for any of the selected TFs, multiplied by the number of selected Gene ontology terms containing that gene)
160, 105, 91, 91, 90, 90, 81, 80, 80, 77, 76, 76, 72, 64, 56, 56, 55, 54, 54, 52, 50, 45, 44, 42, 42, 42, 40,

36, 36, 36, 36, 36, 35, 33, 32, 30, 30, 30, 28, 28, 28, 27, 27, 26, 25, 24, 24, 24, 24, 24, 24, 24, 24, 22, 22,
21, 21, 21, 21, 21, 20, 20, 20, 20, 20, 20, 20, 18, 18, 18, 18, 18, 18, 18, 18, 18, 18, 18, 18, 18, 17, 17, 16,
16, 16, 16, 16, 16, 16, 16, 16, 15, 15, 15, 15, 15, 15, 15, 15, 15, 15, 14, 14, 14, 14, 13, 13, 13, 13, 13,
13, 13, 12, 12, 12, 12, 12, 12, 12, 12, 12, 12, 12, 12, 12, 12, 12, 12, 12, 12, 12, 12, 12, 12, 12, 12, 12, 12,
11, 11, 11, 11, 11, 10, 10, 10, 10, 10, 10, 10, 10, 10, 10, 10, 10, 10, 10, 10, 10, 10, 10, 10, 9, 9, 9, 9, 9, 9,
9, 9, 9, 9, 8, 8, 8, 8, 8, 8, 8, 8, 8, 8, 8, 8, 8, 8, 8, 8, 8, 8, 8, 8, 8, 8, 8, 8, 8, 8, 8, 8, 8, 8, 8, 8, 7, 7, 7, 7, 7, 7,
7, 7, 7, 7, 7, 7, 6, 6, 6, 6, 6, 6, 6, 6, 6, 6, 6, 6, 6, 6, 6, 6, 6, 6, 6, 6, 6, 6, 6, 6, 6, 6, 6, 6, 6, 6, 6, 6, 6, 6, 6, 6,
6, 6, 6, 6, 6, 6, 6, 6, 6, 6, 6, 6, 6, 6, 6, 6, 6, 6, 6, 6, 6, 6, 6, 6, 6, 6, 6, 6, 5, 5, 5, 5, 5, 5, 5, 5, 5, 5, 5, 5, 5, 5,
5, 5, 5, 5, 5, 5, 5, 5, 5, 5, 5, 5, 5, 5, 5, 5, 5, 5, 5, 5, 5, 4, 4, 4, 4, 4, 4, 4, 4, 4, 4, 4, 4, 4, 4, 4, 4, 4, 4, 4, 4, 4,
4, 4, 4, 4, 4, 4, 4, 4, 4, 4, 4, 4, 4, 4, 4, 4, 4, 4, 4, 4, 4, 4, 4, 4, 4, 4, 4, 4, 4, 4, 4, 4, 4, 4, 4, 4, 4, 4, 4, 4, 4, 4,
4, 4, 4, 4, 4, 4, 4, 4, 4, 4, 4, 4, 4, 4, 4, 4, 3, 3, 3, 3, 3, 3, 3, 3, 3, 3, 3, 3, 3, 3, 3, 3, 3, 3, 3, 3, 3, 3, 3, 3, 3, 3,
3, 3, 3, 3, 3, 3, 3, 3, 3, 3, 3, 3, 3, 3, 3, 3, 3, 3, 3, 3, 3, 3, 3, 3, 3, 3, 3, 3, 3, 3, 3, 3, 3, 3, 3, 3, 3, 3, 3, 3, 3, 3,
3, 3, 3, 3, 3, 3, 3, 3, 3, 2, 2, 2, 2, 2, 2, 2, 2, 2, 2, 2, 2, 2, 2, 2, 2, 2, 2, 2, 2, 2, 2, 2, 2, 2, 2, 2, 2, 2, 2, 2, 2, 2,
2, 2, 2, 2, 2, 2, 2, 2, 2, 2, 2, 2, 2, 2, 2, 2, 2, 2, 2, 2, 2, 2, 2, 2, 2, 2, 2, 2, 2, 2, 2, 2, 2, 2, 2, 2, 2, 2, 2, 2, 2, 2,
2, 2, 2, 2, 2, 2, 2, 2, 2, 2, 2, 2, 2, 2, 2, 2, 2, 2, 2, 2, 2, 2, 2, 2, 2, 2, 2, 2, 2, 2, 2, 2, 2, 2, 2, 2, 2, 2, 2, 2, 2, 2,
2, 2, 2, 2, 2, 2, 2, 2, 2, 2, 2, 2, 2, 2, 2, 2, 2, 2, 2, 2, 2, 2, 2, 2, 2, 2, 2, 2, 2, 2, 2, 2, 2, 2, 2, 2, 2, 1, 1, 1, 1, 1,
1, 1, 1, 1, 1, 1, 1, 1, 1, 1, 1, 1, 1, 1, 1, 1, 1, 1, 1, 1, 1, 1, 1, 1, 1, 1, 1, 1, 1, 1, 1, 1, 1, 1, 1, 1, 1, 1, 1, 1, 1, 1,
1, 1, 1, 1, 1, 1, 1, 1, 1, 1, 1, 1, 1, 1, 1, 1, 1, 1, 1, 1, 1, 1, 1, 1, 1, 1, 1, 1, 1, 1, 1, 1, 1, 1, 1, 1, 1, 1, 1, 1, 1, 1,
1, 1, 1, 1, 1, 1, 1, 1, 1, 1, 1, 1, 1, 1, 1, 1, 1, 1, 1, 1, 1, 1, 1, 1, 1, 1, 1, 1, 1, 1, 1, 1, 1, 1, 1, 1, 1, 1, 1, 1, 1, 1,
1, 1, 1, 1, 1, 1, 1, 1, 1, 1, 1, 1, 1, 1, 1, 1, 1, 1, 1, 1, 1, 1, 1, 1, 1, 1

Corresponding number of selected Gene ontology terms each gene is involved with:
(In the same order as above)
16, 15, 13, 13, 9, 15, 9, 10, 10, 11, 4, 19, 8, 8, 4, 4, 11, 6, 6, 13, 10, 9, 4, 3, 14, 14, 5, 6, 9, 3, 3, 9, 7, 11, 2,
3, 5, 10, 7, 4, 4, 9, 3, 2, 5, 8, 4, 3, 3, 3, 6, 4, 4, 2, 11, 7, 7, 7, 3, 7, 10, 4, 5, 2, 4, 4, 5, 2, 2, 3, 9, 3, 6, 6, 3, 6,
6, 9, 6, 6, 1, 17, 2, 8, 4, 4, 2, 4, 4, 2, 2, 15, 3, 5, 5, 5, 3, 3, 1, 5, 3, 5, 7, 2, 2, 7, 1, 1, 1, 1, 1, 1, 1, 4, 3, 2, 4,
3, 6, 4, 4, 3, 4, 1, 3, 1, 2, 3, 2, 4, 3, 3, 12, 3, 3, 3, 4, 1, 4, 11, 1, 1, 1, 1, 2, 1, 10, 5, 2, 5, 5, 1, 2, 1, 1, 2, 5, 1,
2, 5, 2, 1, 5, 9, 3, 1, 3, 1, 1, 3, 9, 1, 1, 4, 4, 1, 1, 4, 1, 4, 2, 4, 4, 1, 2, 2, 1, 8, 1, 2, 2, 4, 2, 2, 8, 4, 4, 4, 4, 1,
1, 4, 4, 4, 2, 1, 1, 1, 7, 1, 1, 7, 1, 7, 1, 7, 1, 2, 3, 3, 2, 1, 2, 6, 2, 3, 2, 3, 2, 2, 2, 1, 1, 6, 2, 3, 2, 2, 2, 6, 2, 3,
1, 6, 3, 6, 1, 3, 3, 1, 2, 3, 2, 3, 6, 3, 3, 3, 3, 2, 3, 3, 3, 3, 2, 6, 1, 2, 3, 2, 1, 6, 3, 1, 3, 2, 1, 2, 1, 2, 1, 5, 1, 1,
1, 1, 1, 5, 5, 5, 5, 1, 5, 5, 1, 5, 1, 1, 1, 5, 1, 1, 1, 5, 5, 1, 5, 5, 5, 5, 5, 1, 5, 1, 5, 1, 2, 2, 2, 1, 2, 2, 2, 2, 1, 1,
4, 1, 4, 1, 2, 4, 4, 2, 2, 4, 1, 1, 2, 2, 4, 4, 1, 2, 2, 4, 1, 4, 2, 2, 1, 2, 4, 1, 1, 4, 2, 1, 1, 2, 1, 1, 2, 1, 1, 1, 2, 2,
4, 2, 2, 4, 2, 2, 4, 2, 4, 1, 1, 4, 2, 1, 2, 2, 4, 1, 1, 2, 1, 1, 2, 1, 2, 1, 1, 3, 3, 1, 1, 3, 1, 3, 1, 3, 3, 1, 3, 1, 1, 3,
3, 3, 3, 3, 3, 1, 1, 1, 1, 1, 3, 1, 1, 3, 3, 1, 1, 1, 1, 3, 3, 1, 1, 1, 3, 1, 3, 3, 1, 3, 1, 1, 3, 3, 1, 1, 3, 1, 1, 1, 1, 1,
1, 1, 3, 3, 1, 1, 1, 3, 3, 3, 1, 3, 1, 3, 3, 3, 1, 1, 3, 1, 1, 1, 2, 1, 1, 2, 2, 1, 2, 2, 1, 1, 1, 2, 2, 1, 2, 1, 2, 2, 1, 2,
1, 2, 2, 1, 1, 1, 2, 1, 1, 1, 1, 1, 1, 2, 2, 2, 2, 1, 1, 2, 2, 2, 1, 2, 1, 2, 2, 1, 1, 2, 1, 1, 1, 2, 2, 2, 1, 1, 2, 1, 2,
1, 1, 1, 2, 2, 2, 2, 1, 1, 2, 1, 1, 2, 1, 1, 1, 1, 1, 2, 1, 1, 2, 2, 2, 2, 2, 1, 1, 2, 2, 2, 1, 1, 1, 2, 1, 1, 1, 1, 2, 1, 2,
1, 2, 2, 1, 2, 2, 2, 2, 2, 2, 1, 1, 2, 2, 1, 1, 2, 1, 1, 2, 1, 1, 1, 2, 1, 1, 2, 2, 2, 1, 2, 2, 1, 1, 1, 1, 2, 1, 2, 1, 2, 2,
1, 2, 1, 1, 1, 1, 1, 1, 1, 1, 1, 1, 1, 1, 1, 1, 1, 1, 1, 1, 1, 1, 1, 1, 1, 1, 1, 1, 1, 1, 1, 1, 1, 1, 1, 1, 1, 1, 1, 1, 1, 1,
1, 1, 1, 1, 1, 1, 1, 1, 1, 1, 1, 1, 1, 1, 1, 1, 1, 1, 1, 1, 1, 1, 1, 1, 1, 1, 1, 1, 1, 1, 1, 1, 1, 1, 1, 1, 1, 1, 1, 1, 1, 1,
1, 1, 1, 1, 1, 1, 1, 1, 1, 1, 1, 1, 1, 1, 1, 1, 1, 1, 1, 1, 1, 1, 1, 1, 1, 1, 1, 1, 1, 1, 1, 1, 1, 1, 1, 1, 1, 1, 1, 1, 1, 1,
1, 1, 1, 1, 1, 1, 1, 1, 1, 1, 1, 1, 1, 1, 1, 1, 1, 1, 1, 1, 1, 1, 1, 1, 1, 1, 1, 1, 1, 1, 1, 1, 1, 1, 1, 1, 1

Corresponding number of selected TFs each gene is involved with:
(In the same order as above)
10, 7, 7, 7, 10, 6, 9, 8, 8, 7, 19, 4, 9, 8, 14, 14, 5, 9, 9, 4, 5, 5, 11, 14, 3, 3, 8, 6, 4, 12, 12, 4, 5, 3, 16, 10, 6,
3, 4, 7, 7, 3, 9, 13, 5, 3, 6, 8, 8, 8, 4, 6, 6, 11, 2, 3, 3, 3, 7, 3, 2, 5, 4, 10, 5, 5, 4, 9, 9, 6, 2, 6, 3, 3, 6, 3, 3, 2,
3, 3, 17, 1, 8, 2, 4, 4, 8, 4, 4, 8, 8, 1, 5, 3, 3, 3, 5, 5, 15, 3, 5, 3, 2, 7, 7, 2, 13, 13, 13, 13, 13, 13, 13, 3, 4, 6,
3, 4, 2, 3, 3, 4, 3, 12, 4, 12, 6, 4, 6, 3, 4, 4, 1, 4, 4, 4, 3, 12, 3, 1, 11, 11, 11, 11, 5, 10, 1, 2, 5, 2, 2, 10, 5,
10, 10, 5, 2, 10, 5, 2, 5, 10, 2, 1, 3, 9, 3, 9, 9, 3, 1, 9, 9, 2, 2, 8, 8, 2, 8, 2, 4, 2, 2, 8, 4, 4, 8, 1, 8, 4, 4, 2, 4,
4, 1, 2, 2, 2, 2, 8, 8, 2, 2, 2, 4, 7, 7, 7, 1, 7, 7, 1, 7, 1, 7, 1, 7, 3, 2, 2, 3, 6, 3, 1, 3, 2, 3, 2, 3, 3, 3, 6, 6, 1, 3,
2, 3, 3, 3, 1, 3, 2, 6, 1, 2, 1, 6, 2, 2, 6, 3, 2, 3, 2, 1, 2, 2, 2, 2, 3, 2, 2, 2, 2, 3, 1, 6, 3, 2, 3, 6, 1, 2, 6, 2, 3, 6,
3, 6, 3, 6, 1, 5, 5, 5, 5, 5, 1, 1, 1, 1, 5, 1, 1, 5, 1, 5, 5, 5, 1, 5, 5, 5, 1, 1, 5, 1, 1, 1, 1, 1, 5, 1, 5, 1, 5, 2, 2, 2,
4, 2, 2, 2, 2, 4, 4, 1, 4, 1, 4, 2, 1, 1, 2, 2, 1, 4, 4, 2, 2, 1, 1, 4, 2, 2, 1, 4, 1, 2, 2, 4, 2, 1, 4, 4, 1, 2, 4, 4, 2, 4,
4, 2, 4, 4, 4, 2, 2, 1, 2, 2, 1, 2, 2, 1, 2, 1, 4, 4, 1, 2, 4, 2, 2, 1, 4, 4, 2, 4, 4, 2, 4, 2, 4, 4, 1, 1, 3, 3, 1, 3, 1, 3,
1, 1, 3, 1, 3, 3, 1, 1, 1, 1, 1, 1, 3, 3, 3, 3, 3, 1, 3, 3, 1, 1, 3, 3, 3, 3, 1, 1, 3, 3, 3, 1, 3, 1, 1, 3, 1, 3, 3, 1, 1, 3,

3, 1, 3, 3, 3, 3, 3, 3, 3, 1, 1, 3, 3, 3, 1, 1, 1, 3, 1, 3, 1, 1, 1, 3, 3, 1, 3, 2, 2, 1, 2, 2, 1, 1, 2, 1, 1, 2, 2, 2, 1, 1, 2, 1, 2, 1, 1, 2, 1, 2, 1, 1, 2, 2, 2, 1, 2, 2, 2, 2, 2, 2, 2, 1, 1, 1, 1, 2, 2, 1, 1, 1, 2, 1, 2, 1, 1, 2, 2, 1, 2, 2, 2, 1, 1, 1, 2, 2, 1, 2, 1, 2, 2, 2, 1, 1, 1, 1, 2, 2, 1, 2, 2, 1, 2, 2, 2, 2, 2, 1, 2, 2, 1, 1, 1, 1, 1, 2, 2, 1, 1, 1, 2, 2, 2, 1, 2, 2, 2, 2, 1, 2, 1, 2, 1, 1, 2, 1, 1, 1, 1, 1, 1, 2, 2, 1, 1, 2, 2, 1, 2, 2, 1, 2, 2, 2, 1, 2, 2, 1, 1, 1, 2, 1, 1, 2, 2, 2, 2, 1, 2, 1, 2, 1, 1, 2, 1, 2, 2, 2, 2, 1

Gene ontology terms ranked according to mean of effect sizes in cluster:
ribosome | ribosome biogenesis | translational initiation | polysome | translational elongation | polyamine biosynthetic process | eukaryotic translation initiation factor 2 complex | nuclear-transcribed mRNA catabolic process, nonsense-mediated decay | nucleocytoplasmic transport | ribosome binding | mRNA binding | mRNA transport | polyamine metabolic process | ribosomal subunit | small ribosomal subunit | rRNA processing | mitochondrial DNA replication | translation | regulation of translation | RNA biosynthetic process | ribosome assembly | nucleic acid binding | mRNA catabolic process | leaf senescence | RNA transport | RNA catabolic process | eukaryotic translation initiation factor 3 complex | rRNA transcription | nucleolus organizer region

Corresponding mean of effect sizes of each Gene ontology term:
(In the same order as above)
1.94, 1.68, 1.47, 1.45, 1.27, 1.26, 1.23, 1.21, 1.20, 1.20, 1.16, 1.12, 1.11, 1.07, 1.03, 1.02, 0.99, 0.93, 0.88, 0.87, 0.77, 0.74, 0.71, 0.64, 0.57, 0.56, 0.55, 0.44, 0.08

Corresponding total number of genes of each Gene ontology term:
(In the same order as above)
335, 166, 449, 88, 109, 57, 93, 81, 84, 106, 106, 64, 32, 131, 53, 67, 50, 1.1K, 82, 176, 61, 94, 165, 37, 36, 50, 132, 33, 29

Corresponding number of genes of each Gene ontology term with at least one TFBS (of selected TFs):
(In the same order as above)
167, 97, 212, 45, 56, 28, 46, 41, 46, 57, 59, 31, 9, 65, 29, 32, 27, 494, 40, 76, 33, 50, 79, 14, 19, 20, 67, 16, 13

Corresponding total number of TFBS (of selected TFs) for each Gene ontology term (in all genes):
(In the same order as above)
502, 296, 677, 146, 205, 89, 187, 124, 137, 179, 200, 104, 42, 194, 95, 92, 79, 1467, 137, 250, 107, 166, 234, 50, 68, 72, 202, 48, 38

Corresponding number of selected TFs each Gene ontology term is involved with:
(In the same order as above)
20, 20, 20, 20, 20, 20, 20, 20, 20, 20, 20, 20, 19, 20, 20, 20, 20, 20, 20, 20, 20, 20, 20, 19, 20, 20, 20, 20, 19

TFs ranked according to mean of effect sizes in cluster:
V$GABP_B, V$ELK1_02, VUSF_Q6_01, VUSF_Q6, V$MYCMAX_03, V$HLF_01, V$YY1_Q6_02, V$CLOCKBMAL_Q6, V$E4BP4_01, V$YY1_Q6, VUSF_01, VMAX_01, V$MYCMAX_02, V$MYC_Q2, V$ARNT_01, V$USF_02, V$YY1_02, V$TAXCREB_01, V$NFMUE1_Q6, V$ATF6_01

Corresponding mean of effect sizes of each TF:
(In the same order as above)
1.74, 1.69, 1.45, 1.35, 1.34, 1.31, 1.20, 1.20, 1.13, 1.12, 1.09, 1.02, 0.94, 0.80, 0.65, 0.62, 0.53, 0.46, 0.38, 0.07

Corresponding total number of TFBS for each TF (genome-wide):
(In the same order as above)
625, 627, 664, 672, 650, 657, 688, 679, 635, 684, 647, 647, 694, 676, 655, 649, 639, 674, 658, 670

Corresponding total number of TFBS for each TF (in all genes in selected Gene ontology terms):
(In the same order as above)
333, 310, 325, 328, 321, 362, 342, 340, 334, 325, 318, 312, 319, 291, 286, 266, 287, 289, 250, 249

Corresponding number of genes (of selected Gene ontology terms) each TF is involved with:
(In the same order as above)
121, 113, 115, 118, 121, 133, 127, 119, 122, 114, 118, 120, 121, 109, 109, 106, 107, 114, 103, 102

Corresponding number of selected Gene ontology terms each TF is involved with:
(In the same order as above)
29, 29, 29, 29, 29, 29, 29, 29, 29, 29, 29, 28, 29, 29, 28, 29, 29, 29, 28, 29

Cluster for columns 906 to 915, rows 400 to 408

Gene ontology terms

isoprenoid biosynthetic process | protein-glutamine gamma-glutamyltransferase activity | binding | methionine biosynthetic process | oocyte growth | oocyte growth in germarium-derived egg chamber | phospholipid metabolic process | polysaccharide biosynthetic process | phosphatidylcholine biosynthetic process | phospholipid biosynthetic process

TFs

V$PPAR_DR1_Q2 | V$DR1_Q3 | V$HNF4_DR1_Q3 | V$COUP_DR1_Q6 | V$COUP_01 | V$HNF4_01 | V$HNF4_Q6_01 | V$HNF4ALPHA_Q6 | V$COUPTF_Q6

Information

All related TFs:
(List of all TFs that are related to any of the PWMs)
COUP, COUP-TF1, COUP-TF2, HNF-4, HNF-4alpha, HNF-4alpha1, HNF-4alpha2, HNF-4alpha3, HNF-4alpha4, HNF-4alpha7, HNF-4gamma, PPAR-alpha, PPAR-alpha:RXR-alpha, PPAR-beta, PPAR-gamma, PPAR-gamma1, PPAR-gamma2, PPAR-gamma2:RXR-alpha, PPAR-gamma:RXR-alpha, RAR-alpha:RXR-alpha

Ranked gene list:
(All genes of the selected Gene ontology terms with hits of any of the selected TFs, ranked according to the total number of TFBS)
CTSD, SLC25A1, RHOA, MDK, SSSCA1, ZP3, POU5F1, HSD17B8, CHKB, MMP11, ISYNA1, CSNK2B, GALE, DGAT1, PISD, CALM3, FASN, CDIPT, APEH, TCF7, RXRB, RPS6KA1, RGS19, RBKS, RASGRP2, PNPLA2, PLEC1, PGP, PFN1, OPRL1, MC1R, MAP2K7, LTBP4, KIAA0664, JUND, IL2RB, GIT1, CSK, CRIP1, CD151, BRE, BCL9L, APOC3, APOA1, ZYX, ZBTB7B, VEGFA, UCN2, TRAF4, SYTL1, SLC25A22, RING1, RGS14, POLD4, PDPK1, NOXO1, NISCH, METTL1, MEF2D, MAP3K11, MAFG, GYLTL1B, GOLGA4, GBF1, FUT7, FLJ32987, ESRRA, DNMT3A, DDX41, CYP1A1, CSF1R, CRABP2, CORO1A, CDK2, CBX4, TPI1, SMPD2, SILV, SHBG, SH3BP2, SH3BP1, SCRIB, PSD, PRKCSH, PPP1R10, PPARA, PCP2, NPDC1, MYBBP1A, KCNH2, KAT2A, GMNN, GALK1, ELOVL1, EFNA2, DVL1, DNAJA2, CHRNA4, CFB, CDC34, CCDC88B, TFEB, TAPBP, SHMT2, SFTPC, SFRS6, RPL32, PSMC5, PRKCA, PNMT, PLXNB1, OGFR, NCF4, MAFK,

LPIN1, LMAN2, KCNH4, IL23A, GNAI2, GCK, EMG1, ELF3, EDC4, E4F1, CTRL, CISH, CIC, CDK4, CDC20, CD248, C19orf26, ABHD14B, TUSC2, TNK2, TNFRSF1B, TNFRSF12A, THRA, SLC2A4RG, RGS3, PAX6, PARD6A, MYO1C, MPG, MAPRE1, LRP1, IRF8, IGFBP6, FGFR4, CRIP2, CFL1, CD63, BGN, AXIN1, ARRB2, AGMAT, VAMP8, TPM2, TAP2, SIRT2, SEMA3B, SDC3, RIN1, RBMS1, RARG, RALGDS, PTBP2, PSENEN, PRKCG, PNPLA6, PLA2G1B, PDLIM7, PDK3, NR4A1, NR2F6, NFKBIB, NECAB3, MGC138368, MGAT1, MAZ, LTB4R, LDB1, JUNB, ITGB7, INTU, IL2RG, HSPE1, HSPD1, HNF1B, GYPC, FOSL2, FLNC, F2RL3, EARS2, DLG4, DAXX, CLCF1, ARHGEF2, ANG, AKT1, ADORA2A, ADM, ADAT2, ADAM8, VASP, UBTF, UAP1, TSPO, TNFRSF4, TIMM44, TAP1, SQSTM1, SP2, SOCS3, SOCS2, SNRPA, SNRNP40, SMAD7, SLURP1, SLC2A1, RASGRF1, PTPN6, PTP4A3, PTGER1, PLCG1, PARD3B, OSM, NPFF, NOS3, NFE2L2, NCOA2, NAT6, MTRR, MBD2, MAPK8IP1, MAP2K2, MADCAM1, KLF6, KHSRP, KCNJ5, IRF3, ILVBL, IGF2R, HRH3, HMGCS1, GPI, GAL, FLII, FGR, E2F4, DECR1, CYP11A1, CUGBP1, CTDSP2, CSTB, CSNK1A1, CPT1A, CDKN1A, BAX, ATF2, ANAPC2, ALDOA, ADRBK1, ACY1, ZFPM1, WIPF1, TRIM28, TRADD, TPM3, TNKS1BP1, TNF, SUMO1, STK24, STAT5A, SREBF1, SRC, SP7, SMAD3, SLC9A3R1, SKI, SH2B3, RIT1, RARA, PYY, PRDX6, PRDX2, PLD2, PLCD1, PITX1, PICK1, PDGFA, PCSK7, PBX2, OPRS1, OBSCN, NR6A1, NR2F1, NHP2L1, NFIA, NDUFAB1, NCOR2, MUC1, MRPL28, MNT, MARK2, LTA, ITGB2, IRF9, INSR, ILK, ID1, HRAS, HNF1A, HBEGF, HAMP, GRIN2D, GPR132, GLP1R, GFI1, GALT, GADD45A, FSTL3, FRS2, FKBP5, FGF4, ERF, ERBB2, EPHB2, EMD, EGR1, DNAJC14, DNAI1, DLX2, DDX21, DDB1, CTSB, CPZ, CLCA2, CDK5, CD2BP2, CASP9, C16orf38, AKT1S1, AGFG1, ACTB, ACTA1, ACSL1, ACHE, ZNF148, ZBTB33, ZAP70, WDR1, UTS2, USF2, UNC13A, UBE4A, UBE2M, U2AF2, TST, TSC2, TRIM63, TREH, TRAF5, TPM1, TP73, TP53, TNFRSF25, TNFRSF1A, TMEM201, TM2D1, TLR2, TCF7L2, TAL1, SULT2B1, STX6, STMN1, STAT6, STAT3, STARD3, SSR1, SP6, SNX1, SNRPG, SNAI1, SLC9A3R2, SLC7A7, SLC5A1, SLC36A1, SKP1, SHC1, SFRS1, SF4, SF3B2, SEMA3F, SELPLG, SCP2, SCGB1A1, SCD, SAR1A, S100A9, S100A6, S100A4, RXRA, RTN4R, RPS8, RPS27A, ROBLD3, RGS4, RFX1, RERE, REPIN1, RENBP, RBM47, RASA3, RAMP2, PURA, PTRH1, PSME2, PSME1, POU3F3, POU2F2, PLD1, PIN1, PIK3R2, PIK3CG, PIAS1, PGLYRP1, PFKP, PELP1, PDGFRB, PDCD6, PDCD4, PCSK4, PCDH8, PAPSS1, ORC5L, NUP43, NTRK1, NR5A2, NR1H2, NQO2, NPTN, NME2, NID2, NGFR, NFYA, NFATC1, NEDD9, NDOR1, MXI1, MXD3, MST1, MSL2, MRPL41, MPI, MFI2, MFGE8, MBNL1, MAPT, MAPK1, MAL, MAFA, MAF, MAD1L1, M6PR, LTB, LPIN3, LMNA, LGALS9, LCN1, LARS2, LAMP1, KEAP1, KCNIP3, JAG2, ITIH4, ITGAL, ITGA3, IRF2, IPO13, ING2, IL4R, IL1B, IL18BP, ICAM2, HSPA5, HSPA4, HRH1, HPCA, HOXA5, HNRNPL, HNRNPC, GZMM, GUCA2A, GRM2, GPSM1, GP2, GNRH2, GLI1, GFM1, GCKR, GCC2, GATA4, GAPDH, GALNS, FUBP3, FOXO1, FLAD1, FBLIM1, F2R, EZR, EXOC1, ERBB2IP, EPO, ELAVL1, EIF4G1, EGR4, EFEMP2, E2F3, DUSP2, DSC3, DLX4, DLL1, DKFZp434P0672, DCTN3, CYTH1, CYC1, CRHR1, COX17, COPG, CLTB, CKS1B, CHPF, CHMP4B, CDX1, CDKN2C, CD82, CD72, CD5, CD4, CARM1, CAPG, CAND1, BRD2, BNIP2, BCL2L11, BAK1, B4GALT1, ATF4, ARHGAP1, ARF1, ANXA9, ANXA2, AMT, ALPI, AGRN, ADRM1, ADORA3, ACSL5

Corresponding total number of TFBS:
(For each gene listed above, the total number of TFBS for any of the selected TFs, multiplied by the number of selected Gene ontology terms containing that gene)
40, 30, 27, 27, 21, 18, 18, 18, 18, 16, 15, 15, 14, 14, 12, 12, 10, 10, 10, 9, 9, 9, 9, 9, 9, 9, 9, 9, 9, 9, 9, 9, 9, 9, 9, 9, 9, 9, 9, 9, 9, 9, 9, 9, 9, 8, 8, 8, 8, 8, 8, 8, 8, 8, 8, 8, 8, 8, 8, 8, 8, 8, 8, 8, 8, 8, 8, 8, 8, 8, 8, 8, 8, 8, 8, 8, 7, 7, 7, 7, 7, 7, 7, 7, 7, 7, 7, 7, 7, 7, 7, 7, 7, 7, 7, 7, 7, 7, 7, 7, 7, 7, 6, 6, 6, 6, 6, 6, 6, 6, 6, 6, 6, 6, 6, 6, 6, 6, 6, 6, 6, 6, 6, 6, 6, 6, 6, 6, 6, 6, 6, 6, 6, 5, 5, 5, 5, 5, 5, 5, 5, 5, 5, 5, 5, 5, 5, 5, 5, 5, 5, 5, 5, 5, 5, 5, 4, 4, 4, 4, 4, 4, 4, 4, 4, 4, 4, 4, 4, 4, 4, 4, 4, 4, 4, 4, 4, 4, 4, 4, 4, 4, 4, 4, 4, 4, 4, 4, 4, 4, 4, 4, 4, 4, 4, 4, 4, 4, 4, 4, 4, 4, 4, 4, 3, 3, 3, 3, 3, 3, 3, 3, 3, 3, 3, 3, 3, 3, 3, 3, 3, 3, 3, 3, 3, 3, 3, 3, 3, 3, 3, 3, 3, 3, 3, 3, 3, 3, 3, 3, 3, 3, 3, 3, 3, 3, 3, 3, 3, 3, 3, 3, 3, 3, 3, 3, 3, 3, 3, 3, 3, 3, 3, 3, 2, 2, 2, 2, 2, 2, 2, 2, 2, 2, 2, 2, 2, 2, 2, 2, 2, 2, 2, 2, 2, 2, 2, 2, 2, 2, 2, 2, 2, 2, 2, 2, 2, 2, 2, 2, 2, 2, 2, 2, 2, 2, 2, 2, 2, 2, 2, 2, 2, 2, 2, 2, 2, 2, 2, 2, 2, 2, 2, 2, 2, 2, 2, 2, 2, 2, 2, 2, 2, 2, 2, 2, 2, 2, 2, 2, 2, 2, 2, 2, 2, 2, 2, 2, 1

Corresponding number of selected Gene ontology terms each gene is involved with:
(In the same order as above)
5, 5, 3, 3, 3, 3, 3, 2, 3, 2, 3, 3, 2, 2, 4, 4, 2, 2, 2, 1, 1, 1, 1, 1, 1, 1, 1, 1, 1, 1, 1, 1, 1, 1, 1, 1, 1, 1, 1, 1, 1, 1, 1, 1, 1, 1, 1, 1, 1, 1, 1, 1, 1, 1, 1, 1, 1, 1, 1, 1, 1, 1, 1, 1, 1, 1, 1, 2, 1, 1, 1, 1, 1, 1, 1, 1, 1, 1, 1, 1, 1, 1, 1, 1, 1, 1, 1, 1, 1, 1, 1, 1, 1, 1, 1, 1, 1, 1, 1, 1, 1, 1, 1, 2, 2, 1, 1, 1, 2, 1, 1, 1, 1, 1, 1, 1, 1, 1, 1, 1, 1, 1, 1, 1, 1, 1, 1, 1, 1, 1, 1, 1, 1, 1, 1, 1, 1, 1, 1, 1, 1, 1, 1, 1, 1, 1, 1, 1, 1, 1, 1, 1, 1, 1, 1, 1, 1, 1, 1, 1, 1, 1, 1, 1, 1, 1, 1, 4, 1, 1, 1, 1, 1, 1, 1, 1, 1, 1, 1, 1, 1, 1, 1, 1, 1, 1, 2, 1, 1, 1, 1, 1, 1, 1, 1, 1, 1, 1, 1, 2, 1, 1, 3, 1, 1, 1, 1, 1, 1, 1, 1, 1, 1, 1, 1, 1, 3, 1, 1, 1, 1, 1, 1, 1, 1, 1, 1, 1, 1, 1, 1, 1, 1, 1, 1, 1, 1, 1, 1, 3, 1, 1, 1, 1, 1, 1, 1, 3, 3, 1, 1, 1, 1, 3, 1, 1, 1, 1, 1, 1, 1, 1, 1, 1, 1, 1, 1, 1, 1, 1, 1, 2, 1, 2, 1, 1, 1, 1, 1, 1, 1, 2, 1, 2, 1, 1, 1, 1, 1, 1, 1, 1, 1, 1, 1, 1, 1, 1, 1, 1, 1, 1, 1, 1, 1, 1, 1, 1, 1, 1, 2, 1, 1, 2, 1, 1, 1, 1, 1, 1, 1, 1, 1, 1, 1, 1, 1, 1, 1, 1, 1, 1, 1, 1, 2, 1, 1, 1, 1, 1, 1, 1, 1, 1, 2, 1

Corresponding number of selected TFs each gene is involved with:
(In the same order as above)
8, 6, 9, 9, 7, 6, 6, 9, 6, 8, 5, 5, 7, 7, 3, 3, 5, 5, 5, 9, 9, 9, 9, 9, 9, 9, 9, 9, 9, 9, 9, 9, 9, 9, 9, 9, 9, 9, 9, 9, 9, 9, 9, 9, 8, 8, 8, 8, 8, 8, 8, 8, 8, 8, 8, 8, 8, 8, 8, 8, 8, 8, 8, 8, 8, 8, 8, 4, 8, 8, 8, 8, 8, 8, 8, 7, 7, 7, 7, 7, 7, 7, 7, 7, 7, 7, 7, 7, 7, 7, 7, 7, 7, 7, 7, 7, 7, 7, 7, 7, 7, 6, 6, 3, 3, 6, 6, 6, 3, 6, 6, 6, 6, 6, 6, 6, 6, 6, 6, 6, 6, 6, 6, 6, 6, 6, 6, 6, 6, 6, 6, 6, 5, 5, 5, 5, 5, 5, 5, 5, 5, 5, 5, 5, 5, 5, 5, 5, 5, 5, 5, 5, 5, 5, 5, 4, 4, 4, 4, 4, 4, 4, 4, 4, 4, 4, 4, 4, 4, 1, 4, 4, 4, 4, 4, 4, 4, 4, 4, 4, 4, 4, 4, 4, 4, 4, 4, 4, 2, 4, 4, 4, 4, 4, 4, 4, 4, 4, 4, 4, 4, 2, 4, 3, 1, 3, 3, 3, 3, 3, 3, 3, 3, 3, 3, 3, 3, 3, 1, 3, 3, 3, 3, 3, 3, 3, 3, 3, 3, 3, 3, 3, 3, 3, 3, 3, 3, 3, 3, 3, 3, 1, 3, 3, 3, 3, 3, 3, 3, 1, 1, 3, 3, 3, 3, 1, 3, 3, 3, 3, 3, 3, 3, 2, 2, 2, 2, 2, 2, 2, 2, 2, 2, 1, 2, 1, 2, 2, 2, 2, 2, 2, 2, 1, 2, 1, 2, 2, 2, 2, 2, 2, 2, 2, 2, 2, 2, 2, 2, 2, 2, 2, 2, 2, 2, 2, 2, 2, 2, 2, 1, 2, 2, 1, 2, 2, 2, 2, 2, 2, 2, 2, 2, 2, 2, 2, 2, 2, 2, 2, 2, 2, 2, 2, 1, 2, 2, 2, 2, 2, 2, 2, 2, 2, 1, 2, 2, 1

Gene ontology terms ranked according to mean of effect sizes in cluster:
phospholipid metabolic process | protein-glutamine gamma-glutamyltransferase activity | binding | phosphatidylcholine biosynthetic process | phospholipid biosynthetic process | oocyte growth in germarium-derived egg chamber | oocyte growth | polysaccharide biosynthetic process | isoprenoid biosynthetic process | methionine biosynthetic process

Corresponding mean of effect sizes of each Gene ontology term:
(In the same order as above)
1.62, 1.49, 1.46, 1.44, 1.37, 1.12, 1.12, 1.09, 0.85, 0.72

Corresponding total number of genes of each Gene ontology term:
(In the same order as above)
44, 39, 3.2K, 45, 88, 61, 61, 41, 35, 23

Corresponding number of genes of each Gene ontology term with at least one TFBS (of selected TFs):
(In the same order as above)
13, 6, 529, 13, 22, 11, 11, 9, 6, 4

Corresponding total number of TFBS (of selected TFs) for each Gene ontology term (in all genes):
(In the same order as above)

38, 38, 1673, 48, 76, 48, 48, 36, 29, 18

Corresponding number of selected TFs each Gene ontology term is involved with:
(In the same order as above)
9, 9, 9, 9, 9, 9, 9, 9, 9, 8

TFs ranked according to mean of effect sizes in cluster:
V$PPAR_DR1_Q2, V$HNF4_DR1_Q3, V$HNF4_01, V$COUP_01, V$HNF4_Q6_01, V$COUP_DR1_Q6, V$DR1_Q3, V$COUPTF_Q6, V$HNF4ALPHA_Q6

Corresponding mean of effect sizes of each TF:
(In the same order as above)
1.63, 1.52, 1.46, 1.30, 1.27, 1.16, 1.11, 1.10, 0.52

Corresponding total number of TFBS for each TF (genome-wide):
(In the same order as above)
684, 666, 674, 660, 687, 651, 667, 696, 688

Corresponding total number of TFBS for each TF (in all genes in selected Gene ontology terms):
(In the same order as above)
243, 233, 222, 226, 233, 218, 227, 219, 231

Corresponding number of genes (of selected Gene ontology terms) each TF is involved with:
(In the same order as above)
206, 203, 190, 195, 203, 192, 199, 190, 208

Corresponding number of selected Gene ontology terms each TF is involved with:
(In the same order as above)
10, 10, 10, 10, 10, 10, 10, 9, 10

Cluster for columns 895 to 922, rows 154 to 174

Gene ontology terms

oligodendrocyte differentiation | asymmetric cell division | leaf development | methylated-DNA-[protein]-cysteine S-methyltransferase activity | viral capsid | Gram-negative-bacterium-type cell wall biogenesis | cell wall biogenesis | cell growth | kinase activity | phosphorylation | glycosaminoglycan biosynthetic process | isoprenoid biosynthetic process | protein-glutamine gamma-glutamyltransferase activity | binding | methionine biosynthetic process | oocyte growth | oocyte growth in germarium-derived egg chamber | phospholipid metabolic process | polysaccharide biosynthetic process | phosphatidylcholine biosynthetic process | phospholipid biosynthetic process | Rho guanyl-nucleotide exchange factor activity | guanyl-nucleotide exchange factor activity | GTPase activator activity | GTP catabolic process | GTPase activity | lipid modification | regulator of G-protein signaling activity

TFs

V$SMAD_Q6 | V$AP2REP_01 | V$HNF4_Q6_03 | V$HNF4_Q6_02 | V$T3R_Q6 | V$PAX8_B | V$PAX8_01 | V$USF2_Q6 | V$ZIC2_01 | V$TTF1_Q6 | V$VDR_Q6 | V$LRF_Q2 | V$LBP1_Q6 | V$AP4_Q6_01 | V$AP4_Q6 | V$AP4_Q5 | V$LFA1_Q6 | V$P53_DECAMER_Q2 | V$TAL1_Q6 | V$HEB_Q6 | V$TBX5_02

Information

All related TFs:
(List of all TFs that are related to any of the PWMs)
AP-2rep, AP-4, DeltaNp63alpha, FBI-1, HNF-4, HNF-4alpha, HNF-4alpha1, HNF-4alpha2, HNF-4alpha3, HNF-4alpha4, HTF4, HTF4gamma, LBP-1, LF-A1, LRF, Nkx2-1, OCZF, Pax-8, RAR-alpha, RAR-alpha1, RAR-alpha:RXR-alpha, RAR-alpha:RXR-gamma, RAR-beta, RAR-beta2, RAR-gamma, RXR-alpha, RXR-beta, RXR-beta2, RXR-gamma, Smad1, Smad1.1, Smad2, Smad2-L, Smad3, Smad3:Smad4, Smad4, T3R-alpha, T3R-alpha1, T3R-alpha2, T3R-beta, T3R-beta1, T3R-beta2, TBX5, Tal-1, Tal-1alpha, USF1:USF2, USF2, USF2a, VDR, ZIC2, Zic2, p53, p53-isoform-1, p63alpha, p63gamma, p73alpha, p73beta

Ranked gene list:
(All genes of the selected Gene ontology terms with hits of any of the selected TFs, ranked according to the total number of TFBS)
HRAS, SLC2A4RG, AKT1, ARHGEF2, SSSCA1, CNTNAP1, TNK2, CTSD, CDK5, TUSC2, SLC25A1, RGS19, PTPN6, GIT1, ISYNA1, RALGDS, PLCG1, NOTCH1, ADRBK1, UBTF, RARA, MDK, FASN, CFL1, GRK6, UBE2I, CHKB, TNF, SH3BP1, SCRIB, PER1, JUNB, GALE, DGAT1, CSK, CISH, TRADD, RIN1, MAP3K11, ARHGEF1, CDKN1C, E2F4, SOCS3, RGS14, PISD, SEMA3B, RORC, RASGRP2, PNPLA2, PLXNA1, PDGFB, NR4A1, MKNK2, MEF2D, GFER, SQSTM1, RPS6KB2, PTBP1, PRKCD, PARD6A, MRPL28, MGC111011, LTB, KIAA0664, ITGB2, GPT, CLCF1, CCND2, ACTB, TNFRSF12A, IRF1, DVL1, SOCS1, EGR1, PPP1R9B, MAPK12, LCK, GUK1, SMAD7, PTP4A3, CORO1A, CD248, VEGFA, RPS6KA1, RAB1B, PKN1, FOXO3, CAMK2G, BCL2L11, ARHGDIA, OSM, EFNA1, CDK4, TSC2, SH3BP2, SH2B3, RHOC, NPDC1, NOXO1, MAP3K12, MAP2K3, LBX1, GAPDH, FLJ32987, FLII, DUSP2, BCL9L, ADAM8, TACC3, SHMT2, S100A4, PLK3, PFN1, MXI1, DNMT3A, DLG4, AKT1S1, ZAP70, GLI1, TYMP, RASSF1, RARG, OGFR, NFKBIE, NFATC1, MYO1C, SH2B2, PTK6, GNAS, DPAGT1, TGFB1, NPPC, BRD2, TMEM115, PSD4, MAPK11, JAG2, SLC9A3R1, NFIC, NECAB3, MPG, MMP11, MAP3K14, MAFA, TPP1, SYNGAP1, POLR2A, MAP2K7, IRS1, HMGA1, EMD, CD82, ARF1, ADM, p63RhoGEF, PLCD1, HSD17B8, EFNA2, DDR1, CYBA, COL11A2, NUTF2, MXD3, TPI1, RAB24, PSD, CDKN2C, PURA, PNKP, NFKB2, MLL2, INPP5D, DGKA, DDIT3, DCTN3, CPT1A, CDK9, CDC34, CCND1, CALR, C21orf33, AGRN, U2AF2, PIGW, FGFR3, BHLHB2, APEH, ZFPM1, ZFP36, ZBTB7B, VEGFB, UCN2, TNFRSF4, TMC6, THRA, TCF7, SLC25A22, SKI, SGMS1, SEPT9, RAC3, PTPN7, PTMA, PLEC1, OPRL1, NOC2L, NELF, MNT, MAZ, MAPKAPK3, MAF1, LSP1, KAT2A, JUND, ITGA5, INS, ID3, HES5, HDAC10, GNAI2, FUT7, FLNA, FGF8, DGKZ, CRIP2, CRIP1, CKB, CIRBP, CIC, CDC20, CD151, CBX4, C16orf53, ANAPC2, AHNAK, ADORA2A, ABHD14B, ZMYND10, STAT5A, S100A6, RPS15, RASSF2, PDE4A, PCP2, MAFG, LDB1, HOXA7, HIC1, HDAC7, GFI1, FBRS, E2F2, DKFZp779C0757, CDC25B, RAB34, PRDX2, MYL5, MXD4, MGAT1, LTBP4, KCNH2, HOXA9, GATA2, CYB561D2, CACNB3, ACY1, ACTG1, ZYX, WBP7, TLR9, SYTL1, RPS2, PPP1R1B, PGP, NR4A2, MC1R, LEF1, ENTPD2, DLL1, C19orf26, AXIN1, ARHGAP4, TFEB, SLC12A7, SF3A2, RXRB, PLEKHG2, MCAM, IL17C, GABARAP, CTBP1, BBC3, TNFSF13, SLC39A7, SH2D3C, POLD4, PIK3R2, LTA, LASP1, HOXA10, GPR132, GPI, FHL3, FGF9, FBXL15, DBNL, CDK6, SH3BGRL3, RUNX3, MST1, GYLTL1B, GBF1, FOSB, DUSP1, CTDSP1, CAMKK1, BAK1, REPIN1, RAP1GAP, RAD9A, PFKL, PAX6, NR3C2, NKX2-2, MAFK, GALT, DDX41, CRTC2, CRLF1, ARRB2, CDT1, BRMS1, B3GALT6, ARFGAP2, VASP, VAMP2, SOX8, SHC1, RASA3, PYGM, POU2F2, PDLIM7, PCNA, NOL3, MAPK15, LPAR2, IRF7, ILK, IFITM1, ID2, HES1, GPSM1, GALK1, EPB49, CTRL, CDKN1B, CDK5R1, ACHE, ACAA1, RING1, PSENEN, NPFF, HNRNPL, GRN, CLCN2, VIPR1, UBE2M, TNFRSF1B, SLC9A3R2, SAMD4A, RPS6KA4, RHOG, PNPLA6, PIN1, NTRK1, NDOR1, NAT6, MAPK13, LAT, KLF13, IKBKE, GSK3A, GARNL4, CDKN1A, ARAP1, TRAF4, TNFRSF25, SIN3A, PTGER1, MAL, GPC1, ESRRA, DUSP6, ARFGAP1, ACAP1, ZC3H12D, TSHZ1, TNFRSF1A, SMAD2, RGS11, RGS10, RANBP1, PRKCB, PHF1, PHB2, NGFR, MKNK1, KCNH4, ITPR3, ING1, GALNS, FAM62A, DLX2, CTF1, CD7, CD4, VAV1, STOML2, SLBP, RAB3A, PTGDS, OPRS1, LPIN1, HMX2, GRIN2D, CUX1, CDC25C, BAD, TRAF2, TNFRSF14, TM7SF2, SOS1, SLC2A1, SLC29A1, S100A3, RRAS, PTH1R, PSME2, PNMT, NTHL1, NSD1, NR3C1, NPM1, NDRG1, MYL6, MYD88, MCM2, MARCKSL1, MAP2K2, LIF, LGALS1, LEPREL2, KLF6, KHK, KCNA2, IL11, IDUA, GYPC, GRM2, GRB7, FERMT3, EPHB3, ELOVL1, EIF6, EEF2,

CYC1, CXCR4, CTCF, CNP, CDC37, CD81, CACNA1G, ATF4, ANXA9, ALDOA, TLX2, TGM1, SLC12A4, SCARF2, RGS16, NLGN2, LMAN2, ISG15, IGF2, HCST, EPOR, DDX5, CSNK2B, CCNL2, ADRM1, ZFP36L1, WBP2, VEZF1, TYROBP, TRIM28, TNFRSF13C, STMN1, STK11, SKP1, ROBLD3, RNF4, PTCH1, PSTPIP1, PRMT1, PRKD2, PPARA, PLA2G6, PKM2, NCBP2, NADK, MIF4GD, MBD1, MAP4K2, MAD1L1, IL2RB, IKZF1, IGF1R, IDI1, HOXB4, HK1, HGS, GRIN2C, GPR3, GLTSCR2, GADD45G, FXYD1, ETS1, EMG1, ELMO3, EIF5A, E2F1, DOHH, DDIT4, CSF1, CLEC11A, CERK, CDKN2D, CD79B, CD6, CD44, CCR7, CCND3, CASP9, BRSK2, BCL2L1, BACH2, ANXA6, ANP32A, ADAM15, ACVR1, TFDP1, TCF3, TAF8, STAT6, SREBF2, SREBF1, SPHK1, SEMA4D, RGL2, PTPRA, PSME1, PRAP1, PPP1R7, PMEPA1, PFKFB3, PEA15, PDK2, PAX5, PAOX, NOS3, NFE2L1, MLL5, MARK2, JARID2, ITGB7, ICAM4, HRH3, HDAC4, GIT2, GADD45B, FURIN, FSTL3, FRS3, FBXW7, ERF, DOK2, DNM2, DKFZp434P0672, DDB1, CTDSP2, CGB, CENPB, CD79A, CD63, CCDC88B, BLVRB, BID, ARHGAP9, ZBTB16, WDR1, VDAC1, VAMP8, USF2, USF1, ULK1, TPM1, TOB2, TFAP2A, TCEB2, STARD3, SORBS3, SNCG, SH2D2A, SCN5A, RRM1, RNH1, RAD23A, PPP3CA, PHF12, PFDN5, PER2, PDP2, PDCD6, NRGN, NR5A1, NR2F1, NISCH, NFATC2, NAGK, NACC1, MUC1, MT2A, MIF, MIB2, MBD3, MAP4K1, MADCAM1, LTB4R2, LMNA, KLF10, ITPKA, IL32, IL10RA, HGFAC, HDAC5, HBA2, GRIN1, GRASP, GMEB2, GEMIN4, FOSL2, FARSA, EPB41, EGR4, EFEMP2, EDC4, ECE1, DYRK1B, DLX4, DIABLO, DAK, CTSA, CSRP1, CPEB1, COASY, CEBPE, BRD4, ARNTL, APBA2, AP2M1, AMD1, AHRR, ADRA2B, TSPO, TRRAP, TMSB10, TGFB3, TEAD3, TAPBP, TAP1, SURF2, SNF1LK, SLC38A3, SF3B2, S100A13, RPS19, PVRL1, PTRH1, PPP4C, PPP1R16A, PPIF, POU3F3, PIAS4, PCOLCE, PCID2, PBX2, P2RX7, OAZ1, NUP62, NR2F6, NR1H2, NQO1, NOTCH4, NCOA4, NCAN, MZF1, MYBPC3, MTF1, MSL2, MGMT, MGAT3, MFI2, MCRS1, MCAT, MARK4, MAP1A, LOXL1, KLF3, ITPKB, ITPK1, ITGA7, ITGA6, ISCU, IRF5, IPO13, INSRR, HDGF, GZMM, GATAD2A, FZR1, FYB, F7, EIF4ENIF1, EGR3, EGR2, DYRK2, DPYSL2, DHRS11, CYB5A, CST6, CRABP2, CNTFR, CHRNB1, CAMKK2, C6orf25, C1orf38, BRMS1L, BRF1, BRD1, BCLAF1, B4GALT7, B3GAT3, ATRIP, ARRB1, AP2B1, ANXA11, AGTRAP, ACSS2, ACSL5, ABCG1

Corresponding total number of TFBS:
(For each gene listed above, the total number of TFBS for any of the selected TFs, multiplied by the number of selected Gene ontology terms containing that gene)
252, 240, 231, 228, 168, 162, 147, 133, 133, 126, 126, 126, 126, 126, 120, 114, 114, 114, 114, 105, 105, 105, 105, 105, 95, 85, 85, 84, 84, 84, 84, 84, 84, 84, 84, 84, 80, 80, 80, 78, 70, 68, 64, 64, 64, 63, 63, 63, 63, 63, 63, 63, 63, 63, 63, 60, 60, 60, 60, 60, 60, 60, 60, 60, 60, 60, 60, 60, 60, 57, 57, 57, 56, 56, 54, 54, 54, 54, 51, 51, 50, 49, 48, 48, 48, 48, 48, 48, 48, 48, 45, 45, 44, 42, 42, 42, 42, 42, 42, 42, 42, 42, 42, 42, 42, 42, 42, 42, 40, 40, 40, 40, 40, 40, 40, 40, 40, 39, 39, 38, 38, 38, 38, 38, 38, 38, 36, 36, 36, 36, 35, 35, 35, 34, 34, 34, 34, 32, 32, 32, 32, 32, 32, 32, 30, 30, 30, 30, 30, 30, 30, 30, 30, 30, 28, 28, 28, 28, 28, 28, 28, 27, 27, 26, 26, 26, 25, 24, 24, 24, 24, 24, 24, 24, 24, 24, 24, 24, 24, 24, 24, 24, 22, 22, 22, 22, 22, 21, 21, 21, 21, 21, 21, 21, 21, 21, 21, 21, 21, 21, 21, 21, 21, 21, 21, 21, 21, 21, 21, 21, 21, 21, 21, 21, 21, 21, 21, 21, 21, 21, 21, 21, 21, 21, 21, 21, 21, 21, 21, 21, 21, 21, 21, 21, 21, 21, 20, 20, 20, 20, 20, 20, 20, 20, 20, 20, 20, 20, 20, 20, 20, 20, 20, 19, 19, 19, 19, 19, 19, 19, 19, 19, 19, 19, 19, 19, 18, 18, 18, 18, 18, 18, 18, 18, 18, 18, 18, 18, 18, 18, 18, 17, 17, 17, 17, 17, 17, 17, 17, 17, 17, 16, 16, 16, 16, 16, 16, 16, 16, 16, 16, 16, 16, 16, 16, 16, 15, 15, 15, 15, 15, 15, 15, 15, 15, 15, 14, 14, 14, 14, 14, 14, 14, 14, 14, 14, 14, 14, 14, 13, 13, 13, 13, 12, 12, 12, 12, 12, 12, 12, 12, 12, 12, 12, 12, 12, 12, 12, 12, 12, 12, 12, 12, 12, 12, 12, 12, 12, 11, 11, 11, 11, 11, 11, 10, 10, 10, 10, 10, 10, 10, 10, 10, 10, 10, 10, 10, 10, 10, 10, 10, 10, 10, 10, 9, 9, 9, 9, 9, 9, 9, 9, 9, 9, 8, 8, 8, 8, 8, 8, 8, 8, 8, 8, 8, 8, 8, 8, 8, 8, 8, 8, 8, 8, 8, 7, 7, 7, 7, 7, 7, 7, 7, 7, 7, 7, 7, 6, 6, 6, 6, 6, 6, 6, 6, 6, 6, 6, 6, 6, 6, 6, 6, 6, 6, 6, 6, 6, 6, 6, 6, 6, 6, 6, 6, 6, 6, 6, 6, 6, 6, 6, 6, 6, 6, 6, 6, 6, 6, 6, 6, 6, 6, 6, 6, 5, 5, 5, 5, 5, 5, 5, 5, 5, 5, 5, 5, 5, 5, 5, 4, 4, 4, 4, 4, 4, 4, 4, 4, 4, 4, 4, 4, 4, 4, 4, 4, 4, 4, 4, 4, 4, 4, 4, 4, 4, 4, 4, 4, 4, 4, 4, 4, 4, 4, 4, 4, 4, 4, 4, 4, 4, 4, 4, 4, 4, 4, 4, 4, 4, 4, 4, 4, 4, 4, 4, 4, 4, 4, 4, 3, 3, 3, 3, 3, 3, 3, 3, 3, 3, 3, 3, 3, 3, 3, 3, 3, 3, 3, 3, 3, 3, 3, 3, 3, 3, 3, 3, 3, 3, 3, 3, 3, 3, 3, 3, 3, 3, 3, 3, 3, 3, 3, 3, 3, 3, 3, 3, 2, 2, 2, 2, 2, 2, 2, 2, 2, 2, 2, 2, 2, 2, 2, 2, 2, 2, 2, 2, 2, 2, 2, 2, 2, 2, 2, 2, 2, 2, 2, 2, 2, 2, 2, 2, 2, 2, 2, 2, 2, 2, 2, 2, 2, 2, 2, 2, 2, 2, 2, 2, 2, 2, 2, 2, 2, 2, 2, 2, 2, 2, 2, 2, 2, 2, 2, 2, 2, 2, 2, 2, 2, 2, 2, 1, 1, 1, 1, 1, 1, 1, 1, 1, 1, 1, 1, 1, 1, 1, 1, 1, 1, 1, 1, 1, 1, 1, 1, 1, 1, 1, 1, 1, 1, 1, 1, 1, 1, 1, 1, 1, 1, 1, 1, 1, 1, 1, 1, 1, 1, 1, 1, 1, 1, 1, 1, 1, 1, 1, 1, 1, 1, 1, 1, 1, 1, 1, 1, 1, 1, 1, 1, 1, 1, 1, 1, 1, 1, 1, 1, 1, 1, 1, 1, 1, 1, 1, 1, 1, 1, 1

Corresponding number of selected Gene ontology terms each gene is involved with:
(In the same order as above)

12, 12, 11, 12, 8, 9, 7, 7, 7, 6, 9, 6, 6, 6, 6, 6, 6, 6, 5, 5, 5, 5, 5, 5, 5, 5, 4, 4, 4, 4, 4, 4, 4, 4, 4, 4, 4, 4, 6, 5, 4, 4, 4, 4, 3, 3, 3, 3, 3, 3, 3, 3, 3, 3, 4, 3, 5, 3, 5, 5, 3, 3, 3, 5, 3, 3, 4, 3, 3, 3, 3, 4, 4, 3, 3, 6, 3, 3, 3, 5, 7, 4, 4, 3, 6, 6, 3, 4, 6, 3, 3, 4, 7, 2, 2, 3, 2, 2, 2, 2, 3, 3, 2, 2, 2, 2, 2, 2, 2, 2, 2, 2, 5, 2, 5, 4, 3, 3, 2, 2, 2, 2, 2, 2, 2, 4, 3, 2, 3, 5, 5, 7, 2, 2, 2, 2, 2, 2, 2, 2, 2, 4, 2, 5, 2, 2, 3, 5, 2, 2, 3, 6, 2, 2, 2, 2, 2, 4, 2, 4, 3, 3, 2, 2, 2, 5, 2, 2, 2, 2, 3, 2, 4, 3, 3, 3, 2, 4, 3, 2, 3, 2, 2, 2, 2, 2, 1, 1, 1, 1, 3, 1, 1, 1, 1, 1, 1, 3, 1, 1, 1, 1, 1, 1, 1, 1, 1, 1, 1, 1, 1, 3, 3, 3, 7, 3, 1, 1, 1, 1, 1, 1, 1, 1, 1, 1, 1, 1, 3, 1, 1, 1, 1, 1, 1, 1, 1, 4, 1, 1, 1, 2, 1, 1, 1, 1, 1, 1, 1, 1, 4, 1, 4, 1, 1, 1, 1, 1, 1, 1, 1, 1, 1, 1, 1, 1, 2, 1, 1, 1, 1, 1, 1, 2, 1, 2, 2, 1, 1, 6, 9, 1, 1, 1, 1, 1, 1, 1, 1, 1, 1, 8, 1, 1, 2, 8, 1, 2, 1, 2, 4, 2, 2, 2, 2, 4, 1, 3, 5, 1, 3, 3, 3, 1, 1, 3, 1, 7, 2, 1, 1, 2, 1, 1, 1, 1, 2, 2, 2, 1, 1, 1, 1, 3, 1, 1, 6, 1, 4, 1, 4, 4, 4, 3, 3, 4, 6, 2, 3, 4, 2, 2, 1, 1, 6, 4, 1, 6, 1, 1, 1, 1, 1, 1, 2, 1, 2, 2, 5, 1, 5, 2, 5, 5, 2, 1, 2, 2, 1, 1, 2, 1, 10, 1, 1, 1, 1, 3, 1, 3, 1, 3, 3, 1, 4, 1, 4, 4, 4, 4, 4, 4, 2, 2, 4, 4, 4, 2, 4, 2, 1, 1, 2, 2, 4, 7, 1, 1, 7, 1, 1, 1, 1, 1, 7, 7, 1, 3, 1, 1, 6, 6, 2, 1, 3, 2, 1, 1, 1, 1, 3, 6, 2, 1, 3, 3, 3, 6, 3, 3, 2, 2, 1, 3, 2, 2, 2, 2, 3, 1, 1, 1, 3, 6, 2, 3, 2, 3, 3, 2, 1, 2, 1, 1, 1, 5, 1, 1, 5, 1, 1, 1, 5, 1, 5, 5, 5, 1, 1, 4, 1, 1, 2, 1, 1, 4, 4, 2, 4, 1, 1, 1, 2, 1, 4, 4, 2, 2, 2, 1, 1, 2, 4, 4, 2, 4, 1, 1, 2, 1, 1, 2, 4, 2, 2, 4, 1, 1, 1, 4, 1, 2, 4, 4, 2, 4, 1, 2, 4, 1, 4, 4, 1, 4, 1, 4, 1, 2, 4, 3, 3, 3, 3, 3, 3, 3, 3, 1, 3, 1, 3, 3, 1, 1, 3, 3, 1, 1, 3, 1, 1, 3, 1, 1, 1, 1, 3, 3, 3, 3, 1, 3, 3, 1, 1, 3, 1, 3, 1, 1, 1, 1, 1, 1, 1, 1, 1, 2, 2, 2, 1, 1, 1, 2, 2, 1, 2, 1, 1, 2, 2, 2, 2, 2, 1, 1, 2, 1, 1, 1, 1, 1, 2, 2, 1, 1, 2, 2, 1, 1, 2, 2, 2, 1, 2, 1, 1, 1, 2, 1, 2, 2, 1, 2, 1, 2, 2, 1, 2, 2, 1, 2, 2, 1, 1, 1, 2, 1, 2, 2, 2, 1, 2, 1, 1, 2, 1, 2, 1, 1, 1, 1, 1, 1, 1, 1, 1, 1, 1, 1, 1, 1, 1, 1, 1, 1, 1, 1, 1, 1, 1, 1, 1, 1, 1, 1, 1, 1, 1, 1, 1, 1, 1, 1, 1, 1, 1, 1, 1, 1, 1, 1, 1, 1, 1, 1, 1, 1, 1, 1, 1, 1, 1, 1, 1, 1, 1, 1, 1, 1, 1, 1, 1, 1, 1, 1, 1, 1, 1, 1, 1, 1, 1, 1, 1, 1, 1, 1, 1, 1, 1, 1, 1, 1

Corresponding number of selected TFs each gene is involved with:
(In the same order as above)
21, 20, 21, 19, 21, 18, 21, 19, 19, 21, 14, 21, 21, 21, 20, 19, 19, 19, 19, 21, 21, 21, 21, 21, 19, 17, 17, 21, 21, 21, 21, 21, 21, 21, 21, 21, 20, 20, 20, 13, 14, 17, 16, 16, 16, 21, 21, 21, 21, 21, 21, 21, 21, 21, 21, 15, 20, 12, 20, 12, 12, 20, 20, 20, 12, 20, 20, 15, 20, 19, 19, 19, 14, 14, 18, 18, 9, 18, 17, 17, 10, 7, 12, 12, 16, 8, 8, 16, 12, 8, 15, 15, 11, 6, 21, 21, 14, 21, 21, 21, 21, 14, 14, 21, 21, 21, 21, 21, 20, 20, 20, 20, 20, 8, 20, 8, 10, 13, 13, 19, 19, 19, 19, 19, 19, 19, 9, 12, 18, 12, 7, 7, 5, 17, 17, 17, 17, 16, 16, 16, 16, 16, 8, 16, 6, 15, 15, 10, 6, 15, 15, 10, 5, 15, 14, 14, 14, 14, 7, 14, 7, 9, 9, 13, 13, 13, 5, 12, 12, 12, 12, 8, 12, 6, 8, 8, 8, 12, 6, 8, 12, 8, 11, 11, 11, 11, 11, 21, 21, 21, 21, 7, 21, 21, 21, 21, 21, 21, 7, 21, 21, 21, 21, 21, 21, 21, 21, 21, 21, 21, 21, 21, 7, 7, 7, 3, 7, 21, 21, 21, 21, 21, 21, 21, 21, 21, 21, 21, 21, 7, 21, 21, 21, 21, 21, 21, 21, 20, 5, 20, 20, 20, 10, 20, 20, 20, 20, 20, 20, 20, 20, 5, 20, 5, 19, 19, 19, 19, 19, 19, 19, 19, 19, 19, 19, 19, 19, 9, 18, 18, 18, 18, 18, 18, 9, 18, 9, 9, 18, 18, 3, 2, 17, 17, 17, 17, 17, 17, 17, 17, 17, 17, 2, 16, 16, 8, 2, 16, 8, 16, 8, 4, 8, 8, 8, 8, 4, 15, 5, 3, 15, 5, 5, 5, 15, 15, 5, 14, 2, 7, 14, 14, 7, 14, 14, 14, 14, 7, 7, 7, 13, 13, 13, 13, 4, 12, 12, 2, 12, 3, 12, 3, 3, 3, 4, 4, 3, 2, 6, 4, 3, 6, 6, 12, 12, 2, 3, 12, 2, 11, 11, 11, 11, 11, 11, 5, 10, 5, 5, 2, 10, 2, 5, 2, 2, 5, 10, 5, 5, 10, 10, 5, 10, 1, 10, 9, 9, 9, 3, 9, 3, 9, 3, 3, 9, 2, 8, 2, 2, 2, 2, 2, 2, 4, 4, 2, 2, 2, 4, 2, 4, 8, 8, 4, 4, 2, 1, 7, 7, 1, 7, 7, 7, 7, 7, 1, 1, 7, 2, 6, 6, 1, 1, 3, 6, 2, 3, 6, 6, 6, 6, 2, 1, 3, 6, 2, 2, 2, 1, 2, 2, 3, 3, 6, 2, 3, 3, 3, 3, 2, 6, 6, 6, 2, 1, 3, 2, 3, 2, 2, 3, 6, 3, 6, 6, 5, 1, 5, 5, 1, 5, 5, 5, 1, 5, 1, 1, 1, 5, 5, 1, 4, 4, 2, 4, 4, 1, 1, 2, 1, 4, 4, 4, 2, 4, 1, 1, 2, 2, 2, 4, 4, 2, 1, 1, 2, 1, 4, 4, 2, 4, 4, 2, 1, 2, 2, 1, 4, 4, 4, 1, 4, 2, 1, 1, 2, 1, 4, 2, 1, 4, 1, 1, 4, 1, 4, 1, 4, 2, 1, 1, 1, 1, 1, 1, 1, 1, 3, 1, 3, 1, 1, 3, 3, 1, 1, 3, 3, 1, 3, 3, 1, 3, 3, 3, 3, 1, 1, 1, 1, 3, 1, 1, 3, 3, 1, 3, 1, 3, 3, 3, 3, 3, 3, 3, 3, 3, 1, 1, 1, 2, 2, 2, 1, 1, 2, 1, 2, 2, 1, 1, 1, 1, 1, 2, 2, 1, 2, 2, 2, 2, 2, 1, 1, 2, 2, 1, 1, 2, 2, 1, 1, 1, 2, 1, 2, 2, 2, 1, 2, 1, 1, 2, 1, 2, 1, 1, 2, 1, 1, 2, 1, 1, 2, 2, 2, 1, 2, 1, 1, 1, 2, 1, 2, 2, 1, 2, 1, 2, 2, 2, 2, 1, 1, 1, 1, 1, 1, 1, 1, 1, 1, 1, 1, 1, 1, 1, 1, 1, 1, 1, 1, 1, 1, 1, 1, 1, 1, 1, 1, 1, 1, 1, 1, 1, 1, 1, 1, 1, 1, 1, 1, 1, 1, 1, 1, 1, 1, 1, 1, 1, 1, 1, 1, 1, 1, 1, 1, 1, 1, 1, 1, 1, 1, 1, 1, 1, 1, 1, 1, 1, 1, 1, 1, 1, 1, 1, 1, 1, 1, 1, 1, 1, 1, 1, 1, 1, 1, 1, 1

Gene ontology terms ranked according to mean of effect sizes in cluster:
leaf development | asymmetric cell division | regulator of G-protein signaling activity | phosphatidylcholine biosynthetic process | oligodendrocyte differentiation | cell wall biogenesis | polysaccharide biosynthetic process | lipid modification | GTP catabolic process | GTPase activity | cell growth | binding | GTPase activator activity | Gram-negative-bacterium-type cell wall biogenesis | oocyte growth in germarium-derived egg chamber | oocyte growth | phospholipid biosynthetic process | kinase activity | viral capsid | guanyl-nucleotide exchange factor activity | methylated-DNA-[protein]-cysteine S-methyltransferase activity | methionine biosynthetic process | glycosaminoglycan biosynthetic process | isoprenoid biosynthetic process | phospholipid metabolic process | protein-glutamine gamma-glutamyltransferase activity | phosphorylation | Rho guanyl-nucleotide exchange factor activity

Corresponding mean of effect sizes of each Gene ontology term:
(In the same order as above)
1.87, 1.84, 1.37, 1.36, 1.32, 1.23, 1.21, 1.20, 1.19, 1.11, 1.04, 1.02, 1.01, 0.95, 0.92, 0.92, 0.90, 0.83, 0.77, 0.75, 0.74, 0.66, 0.61, 0.47, 0.45, 0.44, 0.42, 0.41

Corresponding total number of genes of each Gene ontology term:
(In the same order as above)
26, 46, 102, 45, 75, 72, 41, 31, 178, 240, 1.3K, 3.2K, 146, 62, 61, 61, 88, 860, 60, 195, 174, 23, 37, 35, 44, 39, 2.4K, 365

Corresponding number of genes of each Gene ontology term with at least one TFBS (of selected TFs):
(In the same order as above)
8, 12, 20, 11, 17, 13, 7, 5, 37, 47, 254, 502, 34, 11, 11, 11, 20, 175, 13, 38, 31, 3, 9, 4, 5, 7, 411, 62

Corresponding total number of TFBS (of selected TFs) for each Gene ontology term (in all genes):
(In the same order as above)
101, 174, 227, 116, 208, 173, 93, 87, 316, 450, 2131, 4073, 326, 134, 111, 111, 165, 1437, 147, 387, 305, 43, 53, 60, 58, 61, 3306, 582

Corresponding number of selected TFs each Gene ontology term is involved with:
(In the same order as above)
21, 21, 21, 21, 21, 21, 21, 21, 21, 21, 21, 21, 21, 21, 21, 21, 21, 21, 21, 21, 21, 21, 21, 21, 21, 21, 21, 21

TFs ranked according to mean of effect sizes in cluster:
V$LFA1_Q6, V$AP4_Q6_01, V$HNF4_Q6_02, V$LBP1_Q6, VHEB_Q6, VAP4_Q6, V$SMAD_Q6, V$TTF1_Q6, V$TAL1_Q6, V$HNF4_Q6_03, V$AP4_Q5, V$VDR_Q6, V$AP2REP_01, V$PAX8_01, V$USF2_Q6, V$TBX5_02, V$ZIC2_01, V$T3R_Q6, V$PAX8_B, V$LRF_Q2, V$P53_DECAMER_Q2

Corresponding mean of effect sizes of each TF:
(In the same order as above)
1.27, 1.17, 1.16, 1.14, 1.13, 1.11, 1.10, 1.05, 1.05, 1.03, 1.00, 1.00, 0.95, 0.89, 0.88, 0.85, 0.82, 0.82, 0.81, 0.64, 0.38

Corresponding total number of TFBS for each TF (genome-wide):
(In the same order as above)
711, 699, 699, 691, 681, 692, 729, 714, 686, 701, 702, 692, 700, 728, 703, 704, 716, 698, 712, 719, 709

Corresponding total number of TFBS for each TF (in all genes in selected Gene ontology terms):
(In the same order as above)
723, 729, 768, 735, 713, 699, 753, 717, 730, 735, 698, 710, 769, 782, 755, 722, 751, 720, 734, 727, 765

Corresponding number of genes (of selected Gene ontology terms) each TF is involved with:
(In the same order as above)
304, 303, 315, 301, 293, 294, 307, 305, 299, 306, 294, 302, 313, 314, 305, 291, 301, 297, 307, 296, 320

Corresponding number of selected Gene ontology terms each TF is involved with:
(In the same order as above)
28, 28, 28, 28, 28, 28, 28, 28, 28, 28, 28, 28, 28, 28, 28, 28, 28, 28, 28, 28, 28

Cluster for columns 3 to 11, rows 83 to 111

Gene ontology terms

hemopoiesis | cell differentiation | T cell differentiation | cell development | cell activation | T cell

activation | T cell receptor complex | immunological synapse | integrin activation

TFs

V$GATA1_05 | V$FAC1_01 | V$TITF1_Q3 | B$CRP_C | V$GATA_Q6 | V$GATA6_01 | V$TCF4_Q5 | V$LEF1_Q2 | V$BRCA_01 | V$AREB6_04 | V$SOX10_Q6 | V$DBP_Q6 | V$CEBP_01 | V$AP3_Q6 | V$STAT6_01 | V$STAT5A_03 | V$STAT4_01 | V$STAT5A_04 | V$STAT1_03 | V$CEBP_Q3 | V$PAX2_02 | V$HMGIY_Q6 | V$HOXA4_Q2 | V$TST1_01 | V$NFAT_Q6 | V$NFAT_Q4_01 | V$CEBP_Q2_01 | V$CEBPA_01 | V$CEBP_Q2

Information

All related TFs:
(List of all TFs that are related to any of the PWMs)
ANF-2, AP-3, BRCA1, BRCA1:USF2, C/EBP, C/EBPalpha, C/EBPalpha(p20), C/EBPalpha(p30), C/EBPbeta, C/EBPbeta(LAP), C/EBPbeta(p20), C/EBPbeta(p34), C/EBPbeta(p35), C/EBPdelta, C/EBPepsilon, C/EBPgamma, DBP, FAC1, GATA-1, GATA-1A, GATA-2, GATA-3, GATA-4, GATA-5, GATA-5A, GATA-5B, GATA-6, GATA-6A, GATA-6B, HMG, HMG-Y, HMGI-C, HOXA4, LEF-1, LEF-1S, NF-AT, NF-AT1, NF-AT1C, NF-AT2, NF-AT3, NF-AT4, Nkx2-1, POU3F1, Pax-2, Pax-2.1, Pax-2.2, Pax-2a, Pax-2b, STAT1, STAT1alpha, STAT4, STAT5A, STAT6, Sox10, TCF-1, TCF-1(P), TCF-3, TCF-4, ZEB

Ranked gene list:
(All genes of the selected Gene ontology terms with hits of any of the selected TFs, ranked according to the total number of TFBS)
CXCR4, CCR7, PTPN6, FYN, CD4, CD44, ETS1, CISH, GFI1, CSK, TNF, TCF7, LEF1, SHC1, SATB1, ITGA5, FYB, CDKN1B, BCL2L1, RUNX1, IL7R, IKZF1, STAT1, PLCG1, CTLA4, BCL11B, SH2B3, GATA3, CD3G, SSSCA1, LTB, SKAP1, NR3C2, IRF1, CD68, LCP1, BCL2, CCND2, BCL6, SOCS1, PRDM1, PER1, ID2, CCND3, AKT1, LTA, PTK2B, JUNB, LAG3, CD247, ANXA6, NFATC1, LBX1, RUNX3, PIK3R1, MYB, EGR1, ZBTB7B, HOXA10, FLI1, ID3, STAT5B, STAT5A, NR4A2, LMO4, ZAP70, PAX6, NFKBIA, LCK, CD3D, ARL4C, MAF, HOXA9, HES1, BCL2L11, RASSF5, NFATC3, HOXB4, RARA, BHLHB2, PAX5, HOXB3, GIMAP5, EBF1, NKX2-2, STAT6, ICOS, DUSP2, DLL1, RBPJ, EGR2, STIM1, PTPN7, IL16, STAT3, SMAD3, RASGRP1, ITGB2, PIK3CG, LCP2, IL2RA, GATA2, VEGFA, IL23A, CSF1, TBX21, MYC, JUN, GLI1, CFLAR, SMAD7, MLL5, MEF2D, HOXC9, HOXA11, ATXN1, RARG, MLL, LDB1, FAM65B, EOMES, DGKA, CXCR5, CDK4, CD69, CD27, ARHGEF2, PTMA, PITX3, HOXA7, CDKN2C, ALCAM, TNFRSF1A, RORC, MAP3K12, MAP3K1, IRF2, HIST1H4I, CDK6, CD226, PIK3CD, MYO1G, GRB2, ARL6IP5, WIPF1, UNK, TNFAIP8, TAP1, NFIA, LYL1, KLF7, CFL1, CD2, CBX5, IRS1, ILK, BACH2, VIM, SOS1, OSM, ETV6, DOK2, CTNNB1, CRIP1, CD82, BMI1, TCIRG1, TAPBP, SOCS2, MAPK14, ITGB1, HOXC4, FAS, SLAMF1, RING1, PXN, PSMB9, LHX9, HOXC6, HOXC5, HAND2, CREB1, CD63, STK16, NEUROG2, ITPR1, HNRNPL, DUSP6, ZFPM1, S1PR1, PSTPIP1, POU2F2, ITK, POU4F1, PLEKHG2, JARID2, ITGB7, UBC, TFAP2A, POU3F2, NEDD9, NDRG1, IL11RA, FLJ32987, CDK5, CD53, CD47, TXK, STK17B, SOCS3, SILV, ITGA6, HOXB6, CDK2, TAF8, PROX1, MAP4K4, KLF2, IL6, GSK3B, CD79A, CADM1, BCL11A, BATF, PPP1R9B, POLD4, MAL, HOXA1, GRAP2, VDR, TRAF3, TLR9, SOX2, SEMA4D, PDLIM5, PCNA, MYD88, KCNN4, KCNA3, IL21R, CSF2, CD5, CD14, CALM2, CALM1, AHSA1, NR2F2, LFNG, HIST4H4, GGNBP2, DAXX, XAB2, TNFRSF4, STIM2, SMAD5, RAF1, PTPN11, NR4A1, HMBS, CAP1, TNFSF13, FST, FOS, FLII, FGF9, ENO2, CDH23, CALR, BAK1, ZEB1, WNK1, TNFRSF18, SELPLG, SCARB2, RPL17, RERE, PIM1, PAG1, MZF1, MEIS2, IL2, IKZF3, HES5, HCST, E2F4, E2F2, DAD1, CD28, CBLB, ASB2, ACVR1, SH3BP2, RASGRP2, RAG1, PCGF2, IL10, IGF1R, DEF6, DAPP1, CYP26B1, CD7, CD40, ACTB, TPT1, TFRC, RUNX1T1, RHOA, RBM39, PSMC5, PRKCE, PIK3CA, PIK3C3, JUND, ITGA1, IRF6, INPP5D, IL2RB, CDKN1A, C2orf28, TAL1, SRF, SOX4, RAPGEF1, PEA15, PDGFRA, OTX2, NOTCH1, ISYNA1, HIPK3, GUK1, GTF3A, ESR1, ACVR2A, WASF2, UBASH3B, TYROBP, TRIM28, TNFSF8, TNFSF13B, TNFSF10, TNFRSF1B, STRA13, SP1, RFX3, RBM45, PTPN4, PML, PLEKHA2, PDCD1,

NKX6-1, NFE2, MBP, LMO2, LMNA, IRF4, ICAM3, GDF9, FOXO1, ETV5, ELF1, EDC4, DR1, CXXC1, CD83, CD3E, CCR5, BMP4, AKTIP, VAV3, TRAF1, TPM3, TBC1D10C, SIPA1, SETD1A, RGS1, RAP1B, PTPN22, PRKCB, PDE4A, PBX2, NR0B2, NOTCH4, MTHFR, MET, MCAM, JMJD1A, JAK2, IRF8, IL6ST, IGF1, HRAS, HMHA1, HLA-C, GADD45A, FGF2, EPO, EP300, EGF, CRK, CIB1, CCBP2, APBB1IP, ADA, ZNF385A, ZBTB16, WNT7B, UNC13D, TSPAN32, TGFB2, STMN1, SOX5, SMAD4, RRBP1, RRAS, RB1, PRH1, PPARA, POU5F1, POU4F2, POU2F1, POU2AF1, POLR2B, PKN1, PIK3R2, PHLDA1, PDHX, NXT1, NGRN, NFKB1, MUC1, MR1, MEIS1, MAP4K1, MALT1, MAFK, LGALS1, LAT, JAK1, ISL1, ILF3, HLA-B, HBP1, H3F3A, GLIPR1, GDNF, GATA6, FERMT3, EPHB6, DNMT3A, DKFZp434P0672, DHH, DCTN2, CTCF, CDC2L5, CD74, CD37, CD200R1, CD180, CCR6, CASP8, CALM3, ASCL1, ANXA1, XPC, VTN, UBE2I, TTF2, TRAF2, TP53, TCF7L2, TBP, SOX13, SOCS7, SOCS5, SLC2A3, SLC2A1, SLC20A1, SIX3, SIM1, SFTPC, ROBLD3, RAB27A, PUM1, PSMA7, PPIB, PPA1, POU3F1, PDE5A, PAX2, PARP1, OTX1, NR5A2, NR1H2, NOS2, NOD2, NDRG2, MN1, MDK, LYST, LY75, LRRC8A, ITPR3, ITGA3, HSPD1, HNRNPU, GPD2, GIMAP4, GADD45B, FKBP1A, FGR, EED, DDX25, CRTAM, CHRNE, CEBPB, CDR2, CCND1, CASP1, CANX, C1R, BTG2, BDNF, ALAS1

Corresponding total number of TFBS:
(For each gene listed above, the total number of TFBS for any of the selected TFs, multiplied by the number of selected Gene ontology terms containing that gene)
261, 216, 196, 196, 184, 175, 174, 168, 162, 162, 150, 145, 145, 144, 140, 140, 140, 140, 140, 135, 130, 125, 120, 120, 119, 116, 115, 112, 110, 108, 104, 100, 100, 100, 100, 92, 91, 87, 87, 85, 81, 81, 81, 80, 77, 75, 72, 69, 68, 68, 68, 66, 64, 60, 60, 60, 60, 58, 58, 58, 57, 56, 56, 56, 56, 54, 54, 54, 54, 54, 54, 52, 52, 52, 51, 50, 50, 50, 48, 48, 45, 44, 44, 44, 42, 40, 40, 40, 40, 39, 39, 38, 38, 38, 36, 36, 36, 36, 35, 35, 35, 33, 32, 32, 32, 30, 30, 30, 30, 30, 29, 29, 29, 29, 29, 29, 28, 28, 28, 28, 28, 28, 28, 28, 28, 28, 28, 27, 27, 27, 27, 27, 26, 26, 26, 26, 26, 26, 26, 25, 25, 25, 25, 24, 24, 24, 24, 24, 24, 24, 24, 24, 23, 23, 23, 22, 22, 22, 22, 22, 22, 22, 22, 22, 21, 21, 21, 21, 21, 21, 21, 20, 20, 20, 20, 20, 20, 20, 20, 20, 20, 19, 19, 19, 19, 19, 18, 18, 18, 18, 18, 17, 17, 17, 17, 16, 16, 16, 16, 16, 16, 16, 16, 16, 15, 15, 15, 15, 15, 15, 15, 14, 14, 14, 14, 14, 14, 14, 14, 14, 14, 13, 13, 13, 13, 13, 12, 12, 12, 12, 12, 12, 12, 12, 12, 12, 12, 12, 12, 12, 12, 12, 11, 11, 11, 11, 11, 10, 10, 10, 10, 10, 10, 10, 10, 9, 9, 9, 9, 9, 9, 9, 9, 8, 8, 8, 8, 8, 8, 8, 8, 8, 8, 8, 8, 8, 8, 8, 8, 8, 8, 8, 8, 8, 7, 7, 7, 7, 7, 7, 7, 7, 7, 7, 7, 7, 6, 6, 6, 6, 6, 6, 6, 6, 6, 6, 6, 6, 6, 6, 6, 5, 5, 5, 5, 5, 5, 5, 5, 5, 5, 5, 5, 5, 4, 4, 4, 4, 4, 4, 4, 4, 4, 4, 4, 4, 4, 4, 4, 4, 4, 4, 4, 4, 4, 4, 4, 4, 4, 4, 4, 4, 4, 4, 4, 4, 4, 4, 3, 3, 3, 3, 3, 3, 3, 3, 3, 3, 3, 3, 3, 3, 3, 3, 3, 3, 3, 3, 3, 3, 3, 3, 3, 3, 3, 3, 3, 3, 3, 3, 3, 3, 3, 2, 2, 2, 2, 2, 2, 2, 2, 2, 2, 2, 2, 2, 2, 2, 2, 2, 2, 2, 2, 2, 2, 2, 2, 2, 2, 2, 2, 2, 2, 2, 2, 2, 2, 2, 2, 2, 2, 2, 2, 2, 2, 2, 2, 2, 2, 2, 2, 2, 2, 2, 2, 2, 2, 2, 1, 1, 1, 1, 1, 1, 1, 1, 1, 1, 1, 1, 1, 1, 1, 1, 1, 1, 1, 1, 1, 1, 1, 1, 1, 1, 1, 1, 1, 1, 1, 1, 1, 1, 1, 1, 1, 1, 1, 1, 1, 1, 1, 1, 1, 1, 1, 1, 1, 1, 1, 1, 1, 1, 1, 1, 1

Corresponding number of selected Gene ontology terms each gene is involved with:
(In the same order as above)
9, 8, 7, 7, 8, 7, 6, 6, 6, 6, 6, 5, 5, 6, 5, 7, 5, 5, 7, 5, 5, 5, 6, 5, 7, 4, 5, 4, 5, 4, 4, 4, 4, 4, 4, 4, 7, 3, 3, 5, 3, 3, 3, 4, 7, 3, 6, 3, 4, 4, 4, 6, 4, 4, 3, 6, 5, 2, 2, 2, 3, 7, 7, 2, 2, 6, 2, 3, 9, 3, 3, 4, 2, 4, 3, 2, 2, 2, 2, 2, 3, 2, 2, 4, 2, 4, 5, 2, 2, 3, 3, 2, 2, 2, 4, 3, 4, 9, 7, 7, 7, 3, 4, 4, 4, 6, 6, 5, 2, 3, 1, 1, 1, 1, 1, 1, 1, 1, 1, 1, 1, 1, 4, 1, 7, 7, 1, 1, 1, 1, 1, 3, 2, 1, 1, 2, 2, 1, 1, 2, 5, 1, 5, 1, 2, 1, 1, 1, 1, 3, 1, 1, 8, 1, 1, 1, 1, 1, 2, 2, 1, 1, 2, 1, 2, 1, 3, 1, 3, 7, 7, 1, 7, 5, 1, 2, 1, 1, 1, 1, 1, 5, 2, 1, 1, 1, 1, 1, 3, 1, 2, 3, 6, 1, 1, 1, 1, 4, 1, 2, 1, 1, 1, 1, 1, 2, 2, 5, 3, 3, 1, 5, 1, 1, 1, 2, 1, 2, 7, 1, 2, 2, 1, 1, 1, 1, 1, 1, 1, 1, 1, 3, 2, 3, 4, 1, 3, 2, 4, 2, 6, 6, 6, 2, 2, 6, 1, 1, 1, 1, 1, 1, 5, 1, 2, 5, 5, 2, 2, 1, 9, 1, 3, 3, 1, 1, 1, 1, 1, 1, 4, 4, 1, 2, 4, 1, 2, 1, 1, 1, 8, 2, 2, 2, 1, 1, 1, 8, 1, 1, 1, 1, 1, 7, 1, 7, 1, 1, 1, 1, 7, 7, 1, 1, 6, 2, 3, 1, 1, 1, 6, 6, 2, 3, 6, 2, 6, 3, 1, 5, 1, 1, 1, 1, 1, 1, 5, 1, 1, 1, 1, 1, 1, 4, 4, 2, 1, 2, 4, 2, 2, 2, 2, 1, 1, 2, 2, 1, 1, 2, 2, 4, 4, 1, 2, 2, 2, 2, 1, 1, 4, 2, 1, 4, 2, 4, 4, 1, 3, 1, 1, 1, 1, 1, 1, 1, 3, 1, 1, 1, 3, 3, 1, 3, 1, 1, 3, 3, 3, 1, 3, 1, 3, 1, 3, 3, 3, 3, 3, 3, 3, 1, 3, 1, 2, 1, 1, 1, 2, 1, 1, 1, 1, 2, 1, 2, 2, 2, 2, 1, 2, 1, 1, 2, 2, 2, 1, 1, 1, 2, 2, 1, 2, 2, 2, 2, 2, 1, 2, 1, 1, 1, 2, 1, 1, 1, 1, 1, 2, 2, 2, 1, 1, 1, 1, 2, 1, 1, 1, 2, 1, 2, 1, 1, 1, 1, 1, 1, 1, 1, 1, 1, 1, 1, 1, 1, 1, 1, 1, 1, 1, 1, 1, 1, 1, 1, 1, 1, 1, 1, 1, 1, 1, 1, 1, 1, 1, 1, 1, 1, 1, 1, 1, 1, 1, 1, 1, 1, 1, 1, 1, 1, 1, 1, 1, 1, 1, 1, 1, 1, 1

Corresponding number of selected TFs each gene is involved with:
(In the same order as above)
29, 27, 28, 28, 23, 25, 29, 28, 27, 27, 25, 29, 29, 24, 28, 20, 28, 28, 20, 27, 26, 25, 20, 24, 17, 29, 23, 28, 22, 27, 26, 25, 25, 25, 25, 23, 13, 29, 29, 17, 27, 27, 27, 20, 11, 25, 12, 23, 17, 17, 17, 11, 16, 15, 20, 10,

12, 29, 29, 29, 19, 8, 8, 28, 28, 9, 27, 18, 6, 18, 18, 13, 26, 13, 17, 25, 25, 25, 24, 24, 15, 22, 22, 11, 21, 10, 8, 20, 20, 13, 13, 19, 19, 19, 9, 12, 9, 4, 5, 5, 5, 11, 8, 8, 8, 5, 5, 6, 15, 10, 29, 29, 29, 29, 29, 29, 28, 28, 28, 28, 28, 28, 7, 28, 4, 4, 28, 27, 27, 27, 27, 9, 13, 26, 26, 13, 13, 26, 26, 13, 5, 25, 5, 25, 12, 24, 24, 24, 24, 8, 24, 24, 3, 24, 23, 23, 23, 22, 11, 11, 22, 22, 11, 22, 11, 22, 7, 21, 7, 3, 3, 21, 3, 4, 20, 10, 20, 20, 20, 20, 20, 4, 10, 19, 19, 19, 19, 19, 6, 18, 9, 6, 3, 17, 17, 17, 17, 4, 16, 8, 16, 16, 16, 16, 16, 8, 8, 3, 5, 5, 15, 3, 15, 15, 14, 7, 14, 7, 2, 14, 7, 7, 14, 14, 13, 13, 13, 13, 13, 12, 12, 4, 6, 4, 3, 12, 4, 6, 3, 6, 2, 2, 2, 6, 6, 2, 11, 11, 11, 11, 11, 10, 2, 10, 5, 2, 2, 5, 5, 10, 1, 9, 3, 3, 9, 9, 9, 9, 9, 8, 2, 2, 8, 4, 2, 8, 4, 8, 8, 8, 1, 4, 4, 4, 8, 8, 8, 1, 8, 8, 8, 7, 7, 1, 7, 1, 7, 7, 7, 7, 1, 1, 7, 6, 1, 3, 2, 6, 6, 6, 1, 1, 3, 2, 1, 3, 1, 2, 6, 1, 5, 5, 5, 5, 5, 5, 1, 5, 5, 5, 5, 5, 5, 1, 1, 2, 4, 2, 1, 2, 2, 2, 2, 4, 4, 2, 2, 4, 4, 2, 2, 1, 1, 4, 2, 2, 2, 2, 4, 4, 1, 2, 4, 1, 2, 1, 1, 4, 1, 3, 3, 3, 3, 3, 3, 3, 1, 3, 3, 3, 1, 1, 3, 1, 3, 3, 1, 1, 1, 3, 1, 3, 1, 3, 1, 1, 1, 1, 1, 1, 1, 3, 1, 2, 1, 2, 2, 2, 1, 2, 2, 2, 2, 1, 2, 1, 1, 1, 1, 2, 1, 2, 2, 1, 1, 1, 2, 2, 2, 1, 1, 2, 1, 1, 1, 1, 1, 2, 1, 2, 2, 2, 1, 2, 2, 2, 2, 2, 1, 1, 1, 2, 2, 2, 2, 1, 2, 2, 2, 1, 2, 1, 2, 1, 1, 1, 1, 1, 1, 1, 1, 1, 1, 1, 1, 1, 1, 1, 1, 1, 1, 1, 1, 1, 1, 1, 1, 1, 1, 1, 1, 1, 1, 1, 1, 1, 1, 1, 1, 1, 1, 1, 1, 1, 1, 1, 1, 1, 1, 1, 1, 1, 1, 1, 1, 1, 1, 1, 1, 1, 1, 1

Gene ontology terms ranked according to mean of effect sizes in cluster:
cell differentiation | T cell receptor complex | cell development | hemopoiesis | T cell activation | cell activation | T cell differentiation | immunological synapse | integrin activation

Corresponding mean of effect sizes of each Gene ontology term:
(In the same order as above)
2.59, 2.32, 2.29, 2.13, 2.04, 1.98, 1.96, 1.26, 0.96

Corresponding total number of genes of each Gene ontology term:
(In the same order as above)
871, 478, 507, 343, 287, 596, 172, 112, 155

Corresponding number of genes of each Gene ontology term with at least one TFBS (of selected TFs):
(In the same order as above)
291, 175, 189, 132, 111, 200, 73, 47, 53

Corresponding total number of TFBS (of selected TFs) for each Gene ontology term (in all genes):
(In the same order as above)
2943, 1710, 1896, 1447, 1118, 1877, 806, 463, 478

Corresponding number of selected TFs each Gene ontology term is involved with:
(In the same order as above)
29, 29, 29, 29, 29, 29, 29, 29, 29

TFs ranked according to mean of effect sizes in cluster:
V$NFAT_Q6, V$NFAT_Q4_01, V$HMGIY_Q6, V$DBP_Q6, V$CEBP_Q2_01, V$STAT6_01, V$TCF4_Q5, V$CEBP_01, V$PAX2_02, V$AP3_Q6, V$STAT5A_03, V$AREB6_04, V$CEBP_Q3, V$HOXA4_Q2, V$CEBPA_01, V$STAT5A_04, V$LEF1_Q2, V$SOX10_Q6, V$STAT1_03, V$STAT4_01, V$CEBP_Q2, V$BRCA_01, V$GATA_Q6, B$CRP_C, V$TITF1_Q3, V$TST1_01, V$GATA1_05, V$FAC1_01, V$GATA6_01

Corresponding mean of effect sizes of each TF:
(In the same order as above)
2.71, 2.71, 2.44, 2.41, 2.38, 2.33, 2.25, 2.23, 2.23, 2.17, 2.13, 2.12, 2.12, 2.08, 2.08, 2.07, 2.04, 2.00, 1.97, 1.95, 1.93, 1.54, 1.49, 1.25, 1.25, 1.24, 1.18, 1.14, 1.06

Corresponding total number of TFBS for each TF (genome-wide):
(In the same order as above)
719, 736, 753, 714, 716, 732, 716, 740, 741, 741, 749, 740, 744, 719, 711, 740, 723, 729, 751, 743, 718, 730, 703, 716, 707, 730, 715, 722, 722

Corresponding total number of TFBS for each TF (in all genes in selected Gene ontology terms):
(In the same order as above)
505, 498, 480, 444, 444, 471, 473, 459, 480, 428, 460, 442, 476, 437, 437, 428, 470, 429, 440, 462, 428, 397, 399, 376, 378, 406, 421, 377, 393

Corresponding number of genes (of selected Gene ontology terms) each TF is involved with:
(In the same order as above)
183, 194, 179, 168, 173, 181, 183, 174, 185, 171, 180, 170, 178, 166, 176, 171, 178, 167, 174, 181, 165, 160, 144, 147, 147, 168, 154, 151, 156

Corresponding number of selected Gene ontology terms each TF is involved with:
(In the same order as above)
9, 9, 9, 9, 9, 9, 9, 9, 9, 9, 9, 9, 9, 9, 9, 9, 9, 9, 9, 9, 9, 9, 9, 9, 9, 9, 9, 9, 9

Cluster for columns 1161 to 1204, rows 267 to 278

Gene ontology terms

lung development | ossification | osteoblast differentiation | cartilage development | chondrocyte differentiation | skeletal development | gut development | neuron development | odontogenesis | transdifferentiation | kidney development | mesoderm formation | heart development | skeletal muscle development | gliogenesis | stem cell differentiation | epithelial to mesenchymal transition | fibroblast growth factor receptor activity | fibroblast growth factor receptor binding | neural tube closure | compound eye development | camera-type eye development | eye development | neural tube formation | system development | cell-cell signaling | cell fate specification | lens development in camera-type eye | neuron migration | N-acetyltransferase activity | developmental process | cell | growth | provirus | induction | apoptosis | cell proliferation | response to osmotic stress | luciferin monooxygenase activity | transcription factor complex | decidualization | liver development | myoblast differentiation | regulation of cell proliferation

TFs

V$ETF_Q6 | V$E2F_Q2 | V$E2F1_Q6 | V$E2F1_Q3 | V$AP2GAMMA_01 | V$AP2ALPHA_01 | V$AP2_Q6_01 | V$AP2_Q6 | V$WT1_Q6 | V$EGR_Q6 | V$MAZ_Q6 | V$MAZR_01

Information

All related TFs:
(List of all TFs that are related to any of the PWMs)
AP-2, AP-2alpha, AP-2alphaA, AP-2alphaB, AP-2beta, AP-2gamma, DP-1, E2F, E2F+E4, E2F-1, E2F-3a, E2F-4, ETF, Egr-1, Egr-2, Egr-3, MAZ, MAZR, WT1, WT1-del2

Ranked gene list:
(All genes of the selected Gene ontology terms with hits of any of the selected TFs, ranked according to the total number of TFBS)
LBX1, FGF8, TGFB1, NOTCH1, PAX6, CDKN1C, AKT1, IGF1R, CCND1, HES1, SSSCA1, SMAD7, PTCH1, ID2, PDGFA, NFATC1, MAPK12, AGRN, HES5, HRAS, MDK, JUNB, ISYNA1, IRS1, FGFR3, VEGFA, IGF2, SQSTM1, WNT10B, TCF3, TBX2, CDK5, LEF1, FGF9, SOCS3, RUNX3, NKX2-2, CFL1, RARA, WNT11, WNT1, RARG, NR4A2, JUND, HMGA1, GFI1, DVL1, DLL1, CUX1, CREBBP, BCL2L11, GLI1, SP3, RBPJ, TYMP, TNFRSF18, TNFRSF12A, PTMA, IL11, FOXO3, FASN, UBE2I, PDGFB, MEF2D, FLJ32987, FBRS, CCND2, SMAD6, TNFRSF4, SPHK1, NR3C1, EGR1, WNT7B, WNT4, PGP, MAPK11, FOXO1, FGFRL1, DNMT3A, DLX2, CYP26B1, ACTB, SKI, JARID2, GATA3, DGAT1, CDKN1B, RXRA, IRS2, IRF1, E2F4, TFEB, PTPN6, PER1,

NR3C2, ING1, FOXC1, ESRRA, EGR3, SCRIB, PTBP1, OGFR, NFKBIE, MAP2K3, ISG15, GPC1, GNAS, GATA2, CDKN2C, ARID2, COL11A2, CDK6, RUNX2, VEGFB, SOS1, PRDX2, PNPLA2, HMHA1, CTSD, CDC25C, TUSC2, TRADD, PRKCD, PDE4A, PCNA, KLF10, DVL2, DDIT3, CISH, CD68, BAD, AXIN1, ACVR1, WHSC1, TIAM1, SOCS2, SHH, NR2F1, FOXD3, EIF5A, CDK4, TNFSF13, MYB, LHX1, ING2, ID3, PLCG1, GAPDH, CDK9, ADM, ZFP36L2, ZFP36, YY1, UBTF, TSPAN4, TFAP2A, SLC2A4RG, SHC1, RHOF, PRKCE, PLEC1, NR4A1, NGFR, MNT, MAZ, LTBP4, KISS1R, HMGB1, GIT1, GFER, ERF, EFNA1, DLL4, CSK, CRIP2, CKB, CBX8, BRD2, BBC3, ATF3, ACVR2B, ZFPM1, TCF7, STRA13, SOX8, SLC4A2, SH2B3, RPLP2, RAC3, NELF, HOXA9, GPX4, GABARAP, FGF22, CORO1A, BMI1, BCL11B, ANP32A, SEMA3B, PPP1R13B, PIK3R2, NFKB2, MARCKSL1, HOXA10, FOSL2, BCL6, ZIC2, TRAF3, RREB1, RRBP1, RHOC, RASSF1, POU2F1, PAX5, NSD1, LMX1B, LDB1, JAG2, IL17C, EGR2, CREB1, CCR10, TP73, SOX1, PTH1R, PLXNA1, PDLIM7, NDOR1, MMP11, MAP3K12, HOXB7, HES3, GADD45B, CNTNAP1, CDK5R1, USF2, TM7SF2, SMAD3, SLC20A1, SKIL, SATB2, PURA, NOL3, NAB2, MXI1, MLL, MEN1, HDAC7, GPT, EP300, EFNA2, DAXX, APRT, SRC, SOCS1, S1PR2, PRKCZ, MAP2K7, ITGA5, FRS3, EZH2, EFNB2, DUSP1, CRABP2, CDKN1A, WNT2B, WBP7, TNK2, STMN1, RNASEK, PFKFB3, PAX2, MXD4, MUC1, HSP90AA1, CTCF, CORO7, CLCN2, CDKN2D, CAMK2G, VIM, ZEB1, ZBTB16, VGF, VDAC1, TSHZ1, TRERF1, SMAD2, SLC25A22, SLC16A3, SIX5, SEMA3F, RASSF7, PTPRU, PTP4A3, PRDM16, PIK3CA, PAFAH1B1, NFIC, MGAT4B, MCAM, MAP4K2, MAP3K11, MAP1LC3B, LRDD, LPAR2, LHX3, LFNG, KLF11, IRF2, HSP90AB1, HNRPDL, HIVEP2, HIVEP1, GAB2, FURIN, FOS, FLNA, FASTK, E2F2, DUSP8, CRLF1, CRIP1, CLEC11A, CD151, CALM1, C16orf53, BCL11A, BACH2, ARVCF, AKT3, ACTG1, TMC6, TGFB3, SLC25A1, REXO1, REPIN1, RCOR2, NPDC1, MAFG, KCNH2, ENTPD2, CEND1, C21orf33, WNT9A, TMEM121, TLX2, STK16, STAT5A, RTEL1, RHOA, REL, PTK6, PPP3CA, PIM1, PFN1, PELP1, OVCA2, NR4A3, NFATC3, MPG, MAFA, Ht006, HYAL2, HDGF, GRN, GNAI2, FSTL3, CLCF1, CHRD, CELSR3, CDC37, CASP9, BHLHB2, ARHGEF2, AHNAK, VAV2, TRAF4, THRA, TBX1, TACC3, PFN2, OSM, NEO1, MLLT10, LYL1, FOXP1, FLII, DCXR, DBP, CUL1, CEBPB, AXUD1, AMH, ABCA2, ZMYM2, ZFP36L1, WNT7A, WNT10A, VASP, SPRED2, SIAH1, SHMT2, SAMD4A, RPS2, QKI, PTPN12, PPARA, PLK3, PHIP, NOG, MXD3, MMP23B, MCM2, LRP1, LMO4, LBH, IRF4, INHBB, HSF4, HS6ST1, HOXA7, HNRNPL, HCN2, HBA2, GRB7, FUS, ENO3, EMILIN1, DYRK1B, DUSP2, CLIC1, CDK2, CDC25B, CDC20, CD81, CBFB, C17orf81, BCL2L2, AIRE, ADORA2A, TCIRG1, SH3BP2, SBF1, RORC, RBL1, PHF13, PDGFRA, ODC1, OAZ1, NOTCH4, MAFK, LTC4S, LGALS1, IRX4, HOXC8, HIST1H4I, HDAC4, FUT7, FOSB, CNKSR1, ATXN1, ARHGDIA, TUBB3, TRRAP, TNFRSF13C, TNFAIP3, THPO, TGIF1, TFRC, SMARCA2, SLC7A1, S100A4, RAP1GAP, RAG1AP1, PRKCA, POU3F2, PARD6A, NXPH4, NKX6-2, NFYB, MTA1, MAPK7, LTBP3, LPHN1, LEPREL2, LAG3, KLF2, IRF6, HOXB6, HOXB4, HOXA11, GGNBP2, GEMIN4, GALE, FZD8, ENO2, ELAVL3, EEF1A2, EBF4, DPAGT1, DIO3, DAG1, CDH15, CALR, C2orf28, BUB3, BAX, BAIAP3, ARID1B, ALDOA, ADIPOR1, USP7, TMEM201, TAPBP, SMARCA4, SLC35B2, SGMS1, SFTPC, SDF4, SDC3, RPS6KA2, ROBLD3, PSMA7, PPP1R9B, PPARD, PLEKHG5, PCP2, NPPC, NEUROG2, MTHFR, MSH6, MIB1, MGAT1, MDGA1, MBD3, MAPKAPK3, LRP5, LEPR, L1CAM, KLF3, KCNH8, INTS6, INS-IGF2, IL17D, HAND2, EMX1, EIF4G1, DYRK2, DPP7, CTF1, CTDSP2, CSF3R, CAST, BRD3, ATP2A3, ARL4C, ANKH, AKT2, ADNP, ZNF292, ZMYND10, ZFYVE9, XPO1, VLDLR, ULK1, UHRF1, TUBGCP2, TP53I3, TCF12, SLC39A1, SLC30A3, SCARB1, REV1, REST, RAPGEF1, POU4F1, PLD2, PEX10, PDPK1, PDLIM2, PC, PAOX, ONECUT1, NR2C2, NFATC4, NF1, NDRG2, MAP1S, KLF5, KAT2A, IDUA, ICMT, HOXD11, HHEX, H2AFX, GCLM, GBX2, FLI1, EPC2, EOMES, EGR4, EEA1, DPYSL2, DNAJA3, DKFZp779C0757, DDX6, CYBA, CYB561D2, CCNO, CCNE1, CBFA2T3, BRMS1, APLP2, AMFR, AKAP1, AES, ZEB2, WDR26, UBL4A, TWSG1, TPT1, TOB2, TNFRSF6B, TNFRSF25, TBPL1, STAG1, SPSB2, SLTM, SLC23A1, SILV, SEPT7, RPLP0, RBM15, RAD9A, PPIA, PNMT, PLXNA3, PIN1, PIAS4, PAK4, NRF1, MTX1, MST1R, MBOAT7, LRBA, LMNA, LIG1, KCNN1, INPP5D, ICOSLG, HOXD13, HOXC6, HOXC4, HOXB5, HOXA5, HOXA2, HNRNPA2B1, HES6, H6PD, FGF17, FGF11, FCGRT, FBXW7, FAM168B, ENOPH1, DKFZp434P0672, DICER1, DCTN3, CSNK2A2, CLIP1, CGB, CD82, CCNL2, CCNE2, CAD, C21orf2, BMP1, ARHGEF1, ACTN1, ACD, YES1, WIBG, VAMP2, UNK, UNC5A, TUSC4, TSTA3, TRIM3, TPM1, TAGLN2, STK3, SMURF2, SLC38A2, SLC1A4, SLC19A1, SLC12A7, SLC12A4, SHOX2, SDHB, SCN8A, S1PR4, S100A6, RHOG, RGS2, REPS1, RAB25, PVRL1, PTPN7, PSMB8, PPP1R1B, PMEPA1, PLXNB2, PHOX2A, PHF1, PCSK4, PARK7, OTX1, OSR1, ONECUT2, NPB, NNAT, NME4, NFIB, NAT13, NAB1, MVP, MLL2, MAX, MAP3K14, MADD, MADCAM1, LY6E, LSP1, LMNB1,

KREMEN1, KAZALD1, ITPR3, IRF7, IKZF1, HYAL1, HOXB9, HOXB2, HNRNPU, HIP1, HDLBP, HDAC5, H3F3A, GTF2I, GALT, FZD7, FSCN1, FOXF1, FOLR2, FAU, EPB41, DYNLL1, DNAJA1, DHX58, DGKZ, DBN1, CRTC2, CRTC1, CNN1, BAT3, ARID1A, ARF1, ADAM15, ACSL6, ABCA3, AATF, ZYX, ZNF250, YWHAG, VPREB3, VEZF1, UNC13D, TYRO3, TPM4, TPD52L2, TMEM187, TMEM115, TLE1, TGFBRAP1, TCEB2, SPIB, SOS2, SMTN, SLC9A3R1, SLC5A6, SLC29A1, SLC12A5, SLC12A2, SERTAD2, SERBP1, SCARF1, RNH1, RGL2, REV3L, RAP1B, RAD51C, PXMP2, PSD2, PPP1R16B, PPP1R12C, PPM2C, POMGNT1, PIP5KL1, PHLDA2, PER2, PDS5B, PDE6D, PARP3, NRM, NKG7, NCOA6, NCOA2, NCDN, NCAN, NAPB, NANOS1, MORF4L1, MGAT4A, MGA, MFAP2, MEA1, MAPT, MAD2L2, LEPROT, LEPRE1, LDHA, LARGE, KLHL35, KCNJ4, KANK2, IMPDH2, ILF3, IL11RA, IDE, HSPB6, HSD11B2, HPN, HOXA1, HNRNPD, HNRNPAB, HMBS, HCFC1, H3F3B, GTF2A1, GOT2, FTL, FHL3, FEZF2, FAM3C, FAM162A, FAF1, ESPN, ERBB2IP, EMP3, EML4, EIF4G3, EGLN2, EBF2, DNM1, DLL3, DENND4A, DAPK2, CTNNA1, COL6A1, CLCN6, CHIC2, CDC73, CDC2L6, CARD9, C3orf58, C11orf31, BOP1, BID, BCL7C, BAP1, BACH1, ATE1, ARFIP2, ANKHD1, AGMAT, ADRM1, ABTB1, ABHD5

Corresponding total number of TFBS:
(For each gene listed above, the total number of TFBS for any of the selected TFs, multiplied by the number of selected Gene ontology terms containing that gene)
340, 264, 216, 204, 192, 168, 168, 156, 143, 140, 121, 110, 110, 110, 99, 96, 96, 96, 90, 88, 84, 84, 84, 84, 81, 80, 80, 77, 72, 72, 72, 72, 70, 70, 66, 66, 66, 66, 65, 64, 63, 63, 63, 63, 60, 60, 60, 60, 60, 60, 60, 57, 56, 56, 55, 55, 55, 55, 55, 55, 55, 54, 54, 54, 54, 54, 54, 52, 50, 49, 49, 49, 48, 48, 48, 48, 48, 48, 48, 48, 45, 45, 44, 44, 44, 44, 44, 42, 42, 42, 42, 40, 40, 40, 40, 40, 40, 40, 40, 36, 36, 36, 36, 36, 36, 36, 36, 36, 36, 36, 35, 35, 34, 33, 33, 33, 33, 33, 32, 32, 30, 30, 30, 30, 30, 30, 30, 30, 30, 30, 30, 30, 30, 28, 28, 28, 28, 28, 28, 28, 28, 27, 27, 27, 27, 27, 25, 25, 25, 25, 24, 24, 24, 24, 24, 24, 24, 24, 24, 24, 24, 24, 24, 24, 24, 24, 24, 24, 24, 24, 24, 24, 24, 24, 24, 24, 24, 24, 24, 24, 24, 22, 22, 22, 22, 22, 22, 22, 22, 22, 22, 22, 22, 22, 22, 22, 22, 22, 21, 21, 21, 21, 21, 21, 21, 21, 20, 20, 20, 20, 20, 20, 20, 20, 20, 20, 20, 20, 20, 20, 20, 20, 18, 18, 18, 18, 18, 18, 18, 18, 18, 18, 18, 18, 18, 16, 16, 16, 16, 16, 16, 16, 16, 16, 16, 16, 16, 16, 16, 16, 16, 16, 16, 15, 15, 15, 15, 15, 15, 15, 15, 15, 15, 15, 15, 14, 14, 14, 14, 14, 14, 14, 14, 14, 14, 14, 14, 14, 14, 14, 13, 12, 12, 12, 12, 12, 12, 12, 12, 12, 12, 12, 12, 12, 12, 12, 12, 12, 12, 12, 12, 12, 12, 12, 12, 12, 12, 12, 12, 12, 12, 12, 12, 12, 12, 12, 12, 12, 12, 12, 12, 12, 12, 12, 12, 12, 12, 12, 12, 12, 12, 12, 12, 12, 12, 12, 11, 11, 11, 11, 11, 11, 11, 11, 11, 11, 11, 11, 10, 10, 10, 10, 10, 10, 10, 10, 10, 10, 10, 10, 10, 10, 10, 10, 10, 10, 10, 10, 10, 10, 10, 10, 10, 10, 10, 10, 10, 10, 10, 10, 9, 9, 9, 9, 9, 9, 9, 9, 9, 9, 9, 9, 9, 9, 9, 9, 9, 9, 9, 8, 8, 8, 8, 8, 8, 8, 8, 8, 8, 8, 8, 8, 8, 8, 8, 8, 8, 8, 8, 8, 8, 8, 8, 8, 8, 8, 8, 8, 8, 8, 8, 8, 8, 8, 8, 8, 8, 8, 8, 8, 8, 8, 8, 8, 8, 7, 7, 7, 7, 7, 7, 7, 7, 7, 7, 7, 7, 7, 7, 7, 7, 7, 7, 7, 7, 7, 7, 6, 6, 6, 6, 6, 6, 6, 6, 6, 6, 6, 6, 6, 6, 6, 6, 6, 6, 6, 6, 6, 6, 6, 6, 6, 6, 6, 6, 6, 6, 6, 6, 6, 6, 6, 6, 6, 6, 6, 6, 6, 6, 6, 6, 6, 6, 6, 6, 6, 5, 5, 5, 5, 5, 5, 5, 5, 5, 5, 5, 5, 5, 5, 5, 5, 5, 5, 5, 5, 5, 5, 5, 5, 5, 5, 5, 5, 5, 5, 5, 5, 5, 5, 5, 5, 5, 5, 5, 5, 5, 5, 5, 5, 5, 5, 5, 5, 4, 4, 4, 4, 4, 4, 4, 4, 4, 4, 4, 4, 4, 4, 4, 4, 4, 4, 4, 4, 4, 4, 4, 4, 4, 4, 4, 4, 4, 4, 4, 4, 4, 4, 4, 4, 4, 4, 4, 4, 4, 4, 4, 4, 4, 4, 4, 4, 4, 4, 4, 4, 4, 4, 4, 4, 4, 3, 3, 3, 3, 3, 3, 3, 3, 3, 3, 3, 3, 3, 3, 3, 3, 3, 3, 3, 3, 3, 3, 3, 3, 3, 3, 3, 3, 3, 3, 3, 3, 3, 3, 3, 3, 3, 3, 3, 3, 3, 3, 3, 3, 3, 3, 3, 3, 3, 3, 3, 3, 3, 3, 3, 3, 3, 3, 3, 3, 3, 3, 3, 3, 2, 2, 2, 2, 2, 2, 2, 2, 2, 2, 2, 2, 2, 2, 2, 2, 2, 2, 2, 2, 2, 2, 2, 2, 2, 2, 2, 2, 2, 2, 2, 2, 2, 2, 2, 2, 2, 2, 2, 2, 2, 2, 2, 2, 2, 2, 2, 2, 2, 2, 2, 2, 2, 2, 2, 2, 2, 2, 2, 2, 2, 2, 2, 2, 2, 2, 2, 2, 2, 2, 2, 2, 2, 2, 2, 2, 2, 2, 2, 2, 2, 2, 2, 2, 2, 2, 2, 2, 2, 2, 1

Corresponding number of selected Gene ontology terms each gene is involved with:
(In the same order as above)
34, 22, 27, 17, 16, 14, 14, 13, 13, 14, 11, 10, 10, 10, 11, 8, 8, 8, 9, 8, 7, 7, 7, 7, 9, 20, 16, 7, 8, 9, 6, 6, 14, 7, 6, 11, 6, 6, 13, 8, 7, 9, 7, 9, 5, 6, 5, 6, 6, 10, 6, 19, 8, 8, 5, 5, 5, 5, 11, 5, 5, 6, 6, 6, 6, 6, 6, 13, 5, 7, 7, 7, 8, 16, 4, 4, 8, 4, 4, 6, 5, 5, 4, 4, 4, 4, 11, 6, 6, 6, 6, 4, 5, 5, 4, 5, 4, 5, 4, 4, 4, 3, 3, 9, 3, 4, 3, 3, 6, 4, 7, 7, 17, 3, 11, 3, 3, 3, 4, 8, 3, 3, 5, 3, 10, 6, 3, 6, 6, 5, 3, 6, 15, 4, 4, 4, 28, 4, 4, 4, 7, 27, 9, 3, 3, 9, 5, 5, 5, 5, 2, 2, 4, 2, 2, 8, 2, 8, 2, 4, 2, 6, 8, 2, 2, 2, 2, 4, 2, 2, 3, 6, 3, 6, 2, 2, 2, 4, 2, 6, 6, 2, 2, 2, 2, 2, 2, 2, 2, 2, 2, 2, 2, 2, 11, 2, 2, 2, 3, 7, 7, 3, 3, 3, 3, 3, 2, 4, 2, 4, 5, 4, 5, 5, 2, 4, 2, 2, 5, 5, 10, 2, 6, 3, 9, 2, 6, 3, 3, 3, 3, 2, 6, 2, 6, 2, 4, 16, 8, 2, 2, 2, 8, 2, 4, 2, 4, 2, 2, 16, 2, 4, 2, 15, 5, 5, 5, 3, 5, 3, 5, 3, 5, 5, 15, 7, 2, 2, 7, 2, 2, 14, 2, 7, 7, 2, 2, 2, 7, 2, 13, 3, 4, 3, 4, 1, 2, 12, 1, 1, 3, 3, 1, 6, 4, 2, 12, 4, 1, 1, 4, 3, 2, 2, 1, 4, 3, 1, 4, 4, 3, 4, 1, 1, 6, 4, 12, 1, 1, 6, 1, 1, 1, 4, 1, 4, 1, 2, 2, 2, 4, 1, 1, 11, 1, 1, 1, 1, 1, 1, 1, 1, 1, 11, 5, 1, 2, 2, 10, 1, 10, 10, 5, 5, 5, 1, 5,

1, 2, 5, 2, 1, 10, 2, 5, 2, 1, 2, 2, 10, 2, 5, 5, 5, 2, 1, 3, 3, 1, 1, 1, 3, 9, 3, 1, 1, 3, 1, 1, 1, 3, 9, 1, 3, 1, 2, 2, 4, 2, 2, 1, 2, 1, 1, 1, 1, 2, 8, 2, 4, 2, 2, 1, 4, 4, 2, 1, 4, 1, 1, 1, 1, 1, 1, 1, 4, 4, 1, 1, 4, 1, 2, 8, 4, 2, 2, 4, 1, 2, 2, 1, 1, 1, 1, 1, 7, 1, 7, 7, 1, 7, 1, 1, 7, 1, 1, 7, 7, 1, 7, 7, 1, 1, 3, 3, 3, 3, 6, 2, 6, 3, 3, 6, 3, 1, 6, 1, 2, 1, 1, 2, 1, 6, 3, 2, 3, 1, 3, 6, 1, 3, 2, 1, 2, 1, 1, 6, 1, 1, 1, 2, 6, 3, 3, 3, 6, 2, 6, 1, 3, 3, 3, 1, 5, 1, 5, 1, 1, 5, 1, 5, 5, 5, 1, 1, 5, 1, 1, 5, 5, 5, 5, 5, 1, 1, 1, 1, 5, 5, 5, 1, 5, 1, 1, 5, 5, 5, 5, 1, 1, 5, 1, 5, 5, 1, 1, 5, 5, 5, 1, 2, 1, 2, 4, 4, 2, 1, 1, 4, 4, 2, 1, 4, 2, 1, 4, 2, 4, 1, 4, 1, 1, 2, 4, 4, 4, 4, 2, 2, 4, 4, 1, 2, 2, 1, 4, 4, 4, 1, 2, 2, 2, 2, 2, 2, 1, 2, 2, 2, 2, 4, 1, 2, 1, 2, 1, 1, 1, 1, 1, 3, 3, 1, 3, 3, 3, 1, 1, 3, 1, 3, 3, 1, 3, 3, 3, 3, 3, 1, 3, 1, 3, 3, 3, 3, 1, 3, 3, 1, 1, 3, 3, 3, 1, 1, 1, 3, 3, 1, 1, 3, 3, 1, 3, 3, 3, 1, 1, 3, 3, 1, 3, 1, 3, 1, 3, 3, 3, 3, 1, 1, 1, 1, 2, 1, 2, 2, 1, 1, 2, 2, 1, 2, 2, 2, 2, 1, 1, 1, 2, 2, 1, 1, 2, 1, 2, 2, 2, 2, 1, 2, 1, 2, 2, 2, 1, 2, 2, 2, 2, 2, 1, 2, 2, 1, 1, 2, 1, 2, 2, 2, 2, 2, 1, 1, 2, 1, 1, 2, 2, 2, 2, 2, 2, 2, 2, 1, 2, 1, 1, 2, 2, 2, 2, 1, 2, 1, 2, 2, 1, 1, 2, 1, 1, 2, 2, 1, 1, 1, 2, 2, 2, 1

Corresponding number of selected TFs each gene is involved with:
(In the same order as above)
10, 12, 8, 12, 12, 12, 12, 12, 11, 10, 11, 11, 11, 11, 9, 12, 12, 12, 10, 11, 12, 12, 12, 12, 9, 4, 5, 11, 9, 8, 12, 12, 5, 10, 11, 6, 11, 11, 5, 8, 9, 7, 9, 7, 12, 10, 12, 10, 10, 6, 10, 3, 7, 7, 11, 11, 11, 11, 5, 11, 11, 9, 9, 9, 9, 9, 9, 4, 10, 7, 7, 7, 6, 3, 12, 12, 6, 12, 12, 8, 9, 9, 11, 11, 11, 11, 4, 7, 7, 7, 7, 10, 8, 8, 10, 8, 10, 8, 10, 9, 9, 12, 12, 4, 12, 9, 12, 12, 6, 9, 5, 5, 2, 11, 3, 11, 11, 11, 8, 4, 10, 10, 6, 10, 3, 5, 10, 5, 5, 6, 10, 5, 2, 7, 7, 7, 1, 7, 7, 7, 4, 1, 3, 9, 9, 3, 5, 5, 5, 5, 12, 12, 6, 12, 12, 3, 12, 3, 12, 6, 12, 4, 3, 12, 12, 12, 12, 6, 12, 12, 8, 4, 8, 4, 12, 12, 12, 6, 12, 4, 4, 11, 11, 11, 11, 11, 11, 11, 11, 11, 11, 11, 11, 2, 11, 11, 11, 7, 3, 3, 7, 7, 7, 7, 7, 10, 5, 10, 5, 4, 5, 4, 4, 10, 5, 10, 10, 4, 4, 2, 10, 3, 6, 2, 9, 3, 6, 6, 6, 6, 9, 3, 9, 3, 8, 4, 1, 2, 8, 8, 8, 2, 8, 4, 8, 4, 8, 8, 1, 8, 4, 8, 1, 3, 3, 3, 5, 3, 5, 3, 5, 3, 3, 1, 2, 7, 7, 2, 7, 7, 1, 7, 2, 2, 7, 7, 7, 2, 7, 1, 4, 3, 4, 3, 12, 6, 1, 12, 12, 4, 4, 12, 2, 3, 6, 1, 3, 12, 12, 3, 4, 6, 6, 12, 3, 4, 12, 3, 3, 4, 3, 12, 12, 2, 3, 1, 12, 12, 2, 12, 12, 12, 3, 12, 3, 12, 6, 6, 6, 3, 12, 11, 1, 11, 11, 11, 11, 11, 11, 11, 11, 11, 1, 2, 10, 5, 5, 1, 10, 1, 1, 2, 2, 2, 10, 2, 10, 5, 2, 5, 10, 1, 5, 2, 5, 10, 5, 5, 1, 5, 2, 2, 2, 5, 10, 3, 3, 9, 9, 9, 3, 1, 3, 9, 9, 3, 9, 9, 9, 3, 1, 9, 3, 9, 4, 4, 2, 4, 4, 8, 4, 8, 8, 8, 8, 4, 1, 4, 2, 4, 4, 8, 2, 2, 4, 8, 2, 8, 8, 8, 8, 8, 8, 8, 2, 2, 8, 8, 2, 8, 4, 1, 2, 4, 4, 2, 8, 4, 4, 8, 7, 7, 7, 7, 1, 7, 1, 1, 7, 1, 7, 7, 1, 7, 7, 1, 1, 7, 1, 1, 7, 7, 2, 2, 2, 2, 1, 3, 1, 2, 2, 1, 2, 6, 1, 6, 3, 6, 6, 3, 6, 1, 2, 3, 2, 6, 2, 1, 6, 2, 3, 6, 3, 6, 6, 1, 6, 6, 6, 3, 1, 2, 2, 2, 1, 3, 1, 6, 2, 2, 2, 5, 1, 5, 1, 5, 5, 1, 5, 1, 1, 1, 5, 5, 1, 5, 5, 1, 1, 1, 1, 1, 5, 5, 5, 5, 1, 1, 1, 5, 1, 5, 5, 1, 1, 1, 1, 5, 5, 1, 5, 1, 1, 5, 5, 1, 1, 1, 5, 2, 4, 2, 1, 1, 2, 4, 4, 1, 1, 2, 4, 1, 2, 4, 1, 2, 1, 4, 1, 4, 4, 2, 1, 1, 1, 1, 2, 2, 1, 1, 4, 2, 2, 4, 1, 1, 1, 4, 2, 2, 2, 2, 2, 2, 4, 2, 2, 2, 2, 1, 4, 2, 4, 2, 4, 4, 3, 3, 3, 1, 1, 3, 1, 1, 1, 3, 3, 1, 3, 1, 1, 3, 1, 1, 1, 1, 3, 1, 3, 1, 1, 1, 1, 3, 1, 1, 3, 3, 1, 1, 1, 3, 3, 3, 1, 1, 3, 3, 1, 1, 3, 1, 1, 1, 3, 3, 1, 1, 3, 1, 3, 1, 3, 1, 1, 1, 1, 3, 3, 3, 2, 1, 2, 1, 1, 2, 2, 1, 1, 2, 1, 1, 1, 2, 2, 2, 1, 1, 2, 2, 1, 2, 1, 1, 1, 1, 2, 1, 2, 1, 1, 1, 2, 1, 1, 1, 1, 1, 2, 1, 1, 2, 2, 1, 2, 1, 1, 1, 1, 1, 2, 2, 1, 2, 2, 1, 1, 1, 1, 1, 1, 1, 1, 2, 1, 2, 2, 1, 1, 1, 1, 2, 1, 2, 1, 1, 2, 2, 1, 2, 2, 1, 1, 2, 2, 2, 1

Gene ontology terms ranked according to mean of effect sizes in cluster:
cell fate specification | lens development in camera-type eye | gliogenesis | skeletal development | cell-cell signaling | stem cell differentiation | fibroblast growth factor receptor activity | growth | fibroblast growth factor receptor binding | heart development | regulation of cell proliferation | neural tube closure | neuron migration | myoblast differentiation | epithelial to mesenchymal transition | ossification | lung development | chondrocyte differentiation | decidualization | cartilage development | osteoblast differentiation | eye development | camera-type eye development | compound eye development | odontogenesis | neuron development | neural tube formation | liver development | cell | system development | cell proliferation | kidney development | skeletal muscle development | developmental process | gut development | induction | transdifferentiation | luciferin monooxygenase activity | mesoderm formation | provirus | transcription factor complex | response to osmotic stress | apoptosis | N-acetyltransferase activity

Corresponding mean of effect sizes of each Gene ontology term:
(In the same order as above)
1.96, 1.74, 1.70, 1.65, 1.60, 1.59, 1.53, 1.52, 1.49, 1.43, 1.40, 1.28, 1.27, 1.27, 1.24, 1.22, 1.21, 1.20, 1.18,

1.12, 1.12, 1.05, 1.05, 1.04, 1.02, 1.01, 0.93, 0.92, 0.91, 0.88, 0.74, 0.70, 0.69, 0.66, 0.65, 0.64, 0.56, 0.55, 0.44, 0.41, 0.36, 0.24, 0.02, -0.26

Corresponding total number of genes of each Gene ontology term:
(In the same order as above)
74, 80, 65, 133, 26, 93, 193, 2.2K, 182, 144, 165, 75, 187, 112, 229, 283, 159, 135, 117, 199, 239, 140, 140, 141, 122, 69, 79, 100, 4.1K, 21, 1.5K, 139, 64, 26, 54, 2.1K, 204, 289, 66, 135, 117, 46, 1.9K, 19

Corresponding number of genes of each Gene ontology term with at least one TFBS (of selected TFs):
(In the same order as above)
25, 20, 15, 31, 8, 21, 41, 392, 41, 29, 47, 20, 41, 33, 53, 52, 36, 33, 27, 41, 54, 24, 24, 24, 24, 18, 22, 23, 654, 6, 283, 24, 14, 9, 11, 335, 41, 54, 16, 29, 27, 7, 336, 2

Corresponding total number of TFBS (of selected TFs) for each Gene ontology term (in all genes):
(In the same order as above)
139, 113, 96, 155, 51, 121, 226, 1774, 228, 182, 226, 101, 194, 154, 265, 234, 171, 151, 119, 194, 262, 139, 139, 139, 130, 100, 116, 97, 2951, 37, 1279, 122, 73, 36, 52, 1624, 190, 263, 66, 150, 141, 42, 1530, 8

Corresponding number of selected TFs each Gene ontology term is involved with:
(In the same order as above)
12, 12, 12, 12, 12, 12, 12, 12, 12, 12, 12, 12, 12, 12, 12, 12, 12, 12, 12, 12, 12, 12, 12, 12, 12, 12, 12, 12, 12, 12, 12, 12, 12, 12, 12, 12, 12, 12, 12, 12, 12, 12, 12, 7

TFs ranked according to mean of effect sizes in cluster:
V$AP2ALPHA_01, V$AP2GAMMA_01, VETF_Q6, VE2F1_Q6, V$AP2_Q6_01, V$E2F_Q2, V$AP2_Q6, V$WT1_Q6, V$MAZR_01, V$E2F1_Q3, VMAZ_Q6, VEGR_Q6

Corresponding mean of effect sizes of each TF:
(In the same order as above)
1.33, 1.31, 1.21, 1.11, 1.10, 1.09, 1.09, 1.01, 0.93, 0.81, 0.66, 0.60

Corresponding total number of TFBS for each TF (genome-wide):
(In the same order as above)
686, 695, 699, 691, 690, 714, 688, 729, 705, 709, 734, 695

Corresponding total number of TFBS for each TF (in all genes in selected Gene ontology terms):
(In the same order as above)
1176, 1187, 1252, 1293, 1199, 1298, 1266, 1315, 1100, 1159, 1175, 1160

Corresponding number of genes (of selected Gene ontology terms) each TF is involved with:
(In the same order as above)
323, 325, 334, 341, 328, 342, 339, 346, 309, 332, 330, 329

Corresponding number of selected Gene ontology terms each TF is involved with:
(In the same order as above)
43, 43, 44, 44, 43, 43, 44, 44, 44, 44, 44, 43

Cluster for columns 1296 to 1310, rows 199 to 202

Gene ontology terms

gene expression | DBD domain binding | DNA binding | chordate embryonic development | embryonic development | limb development | central nervous system development | central nervous system neuron

development | gastrulation | somitogenesis | anatomical structure morphogenesis | organ development | neurogenesis | regionalization | cell fate determination

TFs

V$TFIII_Q6 | V$MZF1_01 | V$VDR_Q3 | V$MZF1_02

Information

All related TFs:
(List of all TFs that are related to any of the PWMs)
MZF1B-C, TFII-I, VDR

Ranked gene list:
(All genes of the selected Gene ontology terms with hits of any of the selected TFs, ranked according to the total number of TFBS)
LBX1, FGF8, WNT1, GLI1, RARA, LEF1, LFNG, VEGFA, HOXA9, AKT1, NR2F1, HES5, TNFSF13, TGFB1, TCF3, RARG, PAX2, NKX2-2, MDK, HOXB7, CDC25C, PAX6, NOTCH1, PBX2, NFATC1, MMP11, LEPREL2, GDF11, DVL1, DGAT1, CDK5, AGRN, HOXA10, DLL1, CHRD, SSSCA1, SEMA3B, OSM, LHX1, LDB1, HOXC8, HOXC6, HOXB8, HOXB6, HOXB4, HMGA1, GATA3, GATA2, EGR1, DLL4, CCND2, BRD2, BHLHB2, LIF, FLI1, FGF9, EFNA2, TFAP2A, OTX1, NR4A2, HMX3, ZFPM1, ZEB2, VEGFB, UNCX, TLE3, THRA, TCF7, SMARCD3, SMAD7, SIN3A, RXRB, NR4A1, NOTCH4, NFIC, MEF2D, MAPK12, MAP2K7, LRP1, JUNB, HOXB5, HOXA7, HES7, ERF, DNMT3A, COL11A2, CDK4, SP1, JUN, STAT5A, NFATC3, MXI1, IL23A, HOXB3, HDGF, GFI1, EHMT2, EFNA1, CORO7, ADNP, ACHE, ZBTB7B, ZBTB7A, WNT10A, VGF, UBTF, TRADD, SSPO, SOCS3, SNAPC2, RREB1, PURA, PPP1R1B, POU2F1, PARD6A, NFKBIE, NFKB2, NELF, NAB2, MUC1, MNT, MAZ, LYL1, LTB, ISYNA1, ISG15, HOXC5, HOXC4, HIVEP1, FOXP1, FGF17, DPH1, DKFZp779C0757, DBP, CISH, CHD3, CFL1, ARID3A, ARHGEF2, ABCA2, PROX1, PITX1, NR2F2, NOL3, MEIS2, DLX2, CXCR4, ZIC2, ZFP36L2, ZEB1, WNT7A, WNT6, WNK1, WBP7, VIM, TNF, TGIF1, TEAD3, TCF7L2, STRA13, SP4, SOX18, SLC20A1, SIX3, SHC1, PTCH1, PLXNA1, NR3C2, MSL1, MLL, MAP4K2, KLF6, IRF1, HOXD12, HOXC9, HOXA11, FST, FOXO3, FLII, EGR3, EGLN2, DDIT3, CSK, CRABP2, BCL3, B3GAT1, ARID1A, ALDOA, WNT2B, WNT11, TPM3, SP3, NGFR, JARID2, HOXB9, FURIN, EN1, COMP, CDKN1C, CCND1, ZFP36L1, ZFP36, ZBTB16, WNT10B, TYMP, TRERF1, TNFRSF25, TNFRSF1A, TNFRSF18, TNFRSF12A, TFEB, TBX1, STAT6, SSBP3, SMARCA2, SLC4A2, SLC25A22, SHMT2, SCRT1, RPL19, RORC, RING1, RHOC, REXO1, RCOR2, PRMT1, POLR2A, PNMT, PLEC1, PIAS3, PER1, PCP2, PCGF2, NRARP, NR2F6, NR1D1, NFIA, MVP, MGAT1, MAPKAPK3, MAP3K12, MAFG, LTC4S, LTA, LRDD, LMO4, LLGL2, LIN28, KREMEN2, KISS1R, KAT2A, JUND, JMJD3, IRX5, ING2, IGF2, HYAL2, HNRPDL, HNRNPL, HIVEP2, HIC1, HDAC7, HCFC1R1, GRIN2D, GPS2, GIT1, GAPDH, GABBR1, FOXJ1, FOXD3, FMNL1, FGFRL1, FASTK, FASN, ENO3, EFNA4, EFNA3, DVL2, DLX6, DLX4, DLG4, CRIP2, CRIP1, CORO1A, CLDN6, CKB, CIRBP, CEND1, CDK9, CD151, CBX4, CACNB3, CACNA1G, BCOR, BCL9L, BCL6, BCL11B, BCL11A, BBC3, BAK1, ATXN1, ANP32A, AGER, ACTB, ZNF148, UBE2I, TSPAN32, TNFRSF4, TGFB3, TCF12, TAGLN2, SYTL1, SPIB, SOX8, SKI, SIPA1, SAMD4A, S100A6, S100A4, RPS6KA1, ROBLD3, RND2, PRKCD, POU3F2, POU2F2, PNPLA2, PHLDB1, PELP1, PEA15, PATZ1, PARN, NFATC2, NCLN, MLL2, MARCKSL1, MAPK11, LTBP3, LGALS1, LASP1, KLF1, IRX4, IRS1, IRF7, IRF2, IGF1R, HOXC10, HIST1H4I, GPX4, GPC1, FOXP4, FOSB, FLJ32987, FBXW4, FBRS, ETV5, EPHB3, ENOPH1, EMP3, EIF5A, EGFL7, EDAR, E2F3, DCHS1, DAXX, CTSD, CRIM1, CLEC11A, CEBPE, CDK6, CD68, CAMK2G, ATP6V0D1, ADORA2A, ZNF274, ZNF24, ZHX2, XRCC6, XAB2, VIPR1, UBE4B, TUSC2, TRIM3, TRIM28, TRAF4, TOB1, TNNT1, THPO, TAS1R3, SQSTM1, SOCS1, SLC2A4RG, SIX5, SETD1A, SEPT1, SATB2, SATB1, REV3L, REPIN1, PTMA, PSMP, PSENEN, PRKCE, POU3F3, PKNOX2, PIK3R2, PIGQ, PHF12, PGP, PDGFB, PCDH1, ORC4L, OGFR, NRTN, NRGN, NNAT, NFKBIA, NFATC4, NEUROD2, MYLIP, MYL6, MSI2, MFNG, MCAM, MARK2, MAFA, LY6E, LMNA, LAMA5, KLF7, ITGB2, IL11, IGF2BP1, IER2, HYAL3, HSP90AB1, HOXA6, HNRNPD, HNRNPA1, HMG20B, GAMT, FZD2,

FOXJ2, FOXF2, FOSL2, FHOD1, ESRRA, ERBB3, ENO2, EGR4, DDR1, DCTN3, CTSW, CTDSP2, CRB3, COL6A1, CNTFR, CHD1, CDH15, CDC20, CD63, CCR7, CBX5, BMF, BCL2L11, ATF3, AKT3, AIRE, AIF1, ADM, ZYX, ZNF2, ZC3H12A, ZAP70, YBX2, XAF1, UNG, UBR5, TUBB2C, TPM1, TOX4, TLR9, SP7, SOX30, SOCS2, SLCO4A1, SLC29A1, SLC12A5, SKIL, SHOX2, SFMBT2, SERTAD3, SERPINH1, SDHC, RPS2, RPL8, RNASEH2C, RBM35A, RASSF1, PTP4A3, PTH1R, PTGER1, PRPF40A, PRDM16, PPP1R15B, PPP1R12C, POLD4, PNKD, PBXIP1, NUDT1, NKG7, MYADM, MXD4, MTA2, MPZ, MEN1, MC1R, MAPK13, MAD1L1, LPHN1, LHX4, LAT, LAPTM5, KAT5, JAG2, ITM2C, INHBA, IMP3, IL2RB, IL17C, IGFBP6, IDI1, ICAM2, HYAL1, HS6ST1, H2AFY, GRIN2C, GRIK5, GRB7, GPSM1, GPER, GGPS1, GCDH, GADD45B, FUSIP1, FHL3, ERBB2IP, EPB41, ELL, ELAVL3, EIF4G1, EIF4A1, EEF1A2, DYRK2, DUSP1, DPYSL3, CYBA, CUL7, CTNNBIP1, CTBP2, CRTC2, CPLX1, CHST11, CHRNE, CHPT1, CFLAR, CD79A, CD3D, CD27, CCND3, CBX3, C1QL1, BAT2, ATP6V0C, ASXL1, ARID5B, ARID4A, ARHGAP5, AGRP, ADRA2B, ACTG1

Corresponding total number of TFBS:
(For each gene listed above, the total number of TFBS for any of the selected TFs, multiplied by the number of selected Gene ontology terms containing that gene)
45, 44, 40, 40, 36, 33, 32, 28, 28, 28, 27, 27, 24, 24, 24, 24, 24, 24, 24, 24, 24, 22, 22, 20, 20, 20, 20, 20, 20, 20, 20, 20, 18, 18, 18, 16, 16, 16, 16, 16, 16, 16, 16, 16, 16, 16, 16, 16, 16, 16, 16, 16, 16, 15, 15, 15, 15, 14, 14, 14, 14, 12, 12, 12, 12, 12, 12, 12, 12, 12, 12, 12, 12, 12, 12, 12, 12, 12, 12, 12, 12, 12, 12, 12, 12, 12, 12, 10, 10, 9, 9, 9, 9, 9, 9, 9, 9, 9, 9, 9, 8, 8, 8, 8, 8, 8, 8, 8, 8, 8, 8, 8, 8, 8, 8, 8, 8, 8, 8, 8, 8, 8, 8, 8, 8, 8, 8, 8, 8, 8, 8, 8, 8, 8, 8, 8, 8, 8, 8, 8, 7, 7, 7, 7, 7, 7, 7, 6, 6, 6, 6, 6, 6, 6, 6, 6, 6, 6, 6, 6, 6, 6, 6, 6, 6, 6, 6, 6, 6, 6, 6, 6, 6, 6, 6, 6, 6, 6, 6, 6, 6, 6, 6, 6, 6, 6, 6, 6, 5, 5, 5, 5, 5, 5, 5, 5, 5, 5, 5, 5, 4, 4, 4, 4, 4, 4, 4, 4, 4, 4, 4, 4, 4, 4, 4, 4, 4, 4, 4, 4, 4, 4, 4, 4, 4, 4, 4, 4, 4, 4, 4, 4, 4, 4, 4, 4, 4, 4, 4, 4, 4, 4, 4, 4, 4, 4, 4, 4, 4, 4, 4, 4, 4, 4, 4, 4, 4, 4, 4, 4, 4, 4, 4, 4, 4, 4, 4, 4, 4, 4, 4, 4, 4, 4, 4, 4, 4, 4, 4, 4, 4, 4, 4, 4, 4, 4, 4, 4, 4, 4, 4, 4, 4, 4, 4, 4, 4, 4, 3, 3, 3, 3, 3, 3, 3, 3, 3, 3, 3, 3, 3, 3, 3, 3, 3, 3, 3, 3, 3, 3, 3, 3, 3, 3, 3, 3, 3, 3, 3, 3, 3, 3, 3, 3, 3, 3, 3, 3, 3, 3, 3, 3, 3, 3, 3, 3, 3, 3, 3, 3, 3, 3, 3, 3, 3, 3, 3, 3, 3, 3, 3, 3, 3, 3, 3, 3, 3, 2, 2, 2, 2, 2, 2, 2, 2, 2, 2, 2, 2, 2, 2, 2, 2, 2, 2, 2, 2, 2, 2, 2, 2, 2, 2, 2, 2, 2, 2, 2, 2, 2, 2, 2, 2, 2, 2, 2, 2, 2, 2, 2, 2, 2, 2, 2, 2, 2, 2, 2, 2, 2, 2, 2, 2, 2, 2, 2, 2, 2, 2, 2, 2, 2, 2, 2, 2, 2, 2, 2, 2, 2, 2, 2, 2, 2, 2, 2, 2, 2, 2, 2, 2, 2, 2, 2, 2, 2, 2, 2, 2, 2, 2, 2, 2, 2, 1

Corresponding number of selected Gene ontology terms each gene is involved with:
(In the same order as above)
15, 11, 10, 10, 9, 11, 8, 7, 7, 7, 9, 9, 12, 8, 8, 6, 12, 6, 6, 6, 6, 11, 11, 5, 5, 5, 5, 5, 5, 5, 5, 5, 6, 9, 9, 4, 4, 4, 8, 4, 4, 4, 4, 4, 4, 4, 4, 4, 4, 4, 4, 4, 4, 5, 5, 5, 5, 7, 7, 7, 7, 3, 3, 3, 3, 3, 3, 4, 3, 4, 3, 3, 3, 6, 3, 3, 3, 3, 3, 3, 3, 6, 3, 3, 4, 3, 3, 5, 10, 3, 3, 3, 9, 3, 3, 3, 3, 3, 3, 3, 3, 2, 2, 2, 2, 2, 2, 2, 2, 2, 2, 2, 2, 4, 4, 2, 2, 2, 2, 2, 2, 2, 2, 2, 2, 2, 2, 2, 2, 2, 8, 2, 2, 2, 2, 2, 2, 2, 2, 2, 7, 7, 7, 7, 7, 7, 7, 3, 3, 3, 6, 6, 3, 2, 6, 2, 3, 3, 6, 2, 3, 2, 6, 6, 3, 6, 3, 3, 2, 2, 2, 3, 3, 3, 2, 6, 6, 3, 6, 3, 2, 2, 3, 2, 2, 3, 2, 2, 5, 5, 5, 5, 5, 5, 5, 5, 5, 5, 5, 5, 1, 1, 4, 4, 1, 1, 2, 2, 1, 1, 2, 4, 2, 2, 4, 1, 1, 1, 1, 1, 1, 1, 1, 2, 1, 1, 1, 1, 1, 2, 1, 1, 1, 2, 4, 1, 4, 1, 1, 1, 1, 4, 1, 2, 1, 4, 1, 4, 1, 1, 4, 2, 1, 4, 2, 4, 2, 4, 1, 1, 1, 1, 1, 1, 1, 1, 1, 1, 4, 4, 1, 1, 1, 2, 1, 1, 1, 1, 4, 4, 1, 1, 1, 4, 1, 1, 1, 1, 2, 1, 1, 1, 1, 1, 1, 1, 1, 1, 1, 2, 1, 1, 1, 4, 3, 3, 1, 1, 3, 3, 1, 3, 3, 3, 3, 1, 3, 1, 1, 3, 3, 1, 1, 3, 3, 1, 1, 3, 3, 3, 3, 3, 1, 1, 1, 1, 1, 3, 1, 3, 3, 1, 3, 3, 3, 1, 3, 3, 3, 1, 3, 3, 3, 1, 3, 3, 3, 1, 1, 3, 3, 3, 1, 1, 1, 3, 1, 3, 1, 1, 1, 3, 1, 2, 2, 2, 2, 1, 2, 2, 1, 1, 1, 2, 1, 2, 1, 1, 1, 2, 1, 2, 1, 1, 1, 2, 2, 1, 1, 2, 2, 1, 1, 1, 2, 1, 1, 1, 1, 1, 1, 1, 2, 1, 1, 2, 2, 2, 2, 1, 1, 2, 1, 2, 2, 1, 1, 2, 2, 1, 1, 2, 1, 1, 1, 1, 1, 2, 2, 1, 2, 2, 2, 2, 1, 1, 2, 1, 2, 1, 1, 1, 1, 1, 1, 1, 2, 2, 1, 1, 2, 2, 1, 2, 2, 1, 1, 2, 1

Corresponding number of selected TFs each gene is involved with:
(In the same order as above)
3, 4, 4, 4, 4, 3, 4, 4, 4, 4, 3, 3, 2, 3, 3, 4, 2, 4, 4, 4, 4, 2, 2, 4, 4, 4, 4, 4, 4, 4, 4, 4, 3, 2, 2, 4, 4, 4, 2, 4, 4, 4, 4, 4, 4, 4, 4, 4, 4, 4, 4, 4, 4, 3, 3, 3, 3, 2, 2, 2, 2, 4, 4, 4, 4, 4, 4, 3, 4, 3, 4, 4, 4, 2, 4, 4, 4, 4, 4, 4, 4, 2, 4, 4, 3, 4, 4, 2, 1, 3, 3, 3, 1, 3, 3, 3, 3, 3, 3, 3, 3, 4, 4, 4, 4, 4, 4, 4, 4, 4, 4, 4, 4, 2, 2, 4, 4, 4, 4, 4, 4, 4, 4, 4, 4, 4, 4, 4, 4, 4, 1, 4, 4, 4, 4, 4, 4, 4, 4, 4, 1, 1, 1, 1, 1, 1, 1, 2, 2, 2, 1, 1, 2, 3, 1, 3, 2, 2, 1, 3, 2, 3, 1, 1, 2, 1, 2, 2,

3, 3, 3, 2, 2, 2, 3, 1, 1, 2, 1, 2, 3, 3, 2, 3, 3, 2, 3, 3, 1, 1, 1, 1, 1, 1, 1, 1, 1, 1, 1, 4, 4, 1, 1, 4, 4, 2, 2, 4, 4, 2, 1, 2, 2, 1, 4, 4, 4, 4, 4, 4, 4, 4, 2, 4, 4, 4, 4, 4, 2, 4, 4, 4, 2, 1, 4, 1, 4, 4, 4, 4, 1, 4, 2, 4, 1, 4, 1, 4, 4, 1, 2, 4, 1, 2, 1, 2, 1, 4, 4, 4, 4, 4, 4, 4, 4, 4, 4, 1, 1, 4, 4, 4, 2, 4, 4, 4, 4, 1, 1, 4, 4, 4, 1, 4, 4, 4, 4, 2, 4, 4, 4, 4, 4, 4, 4, 4, 4, 4, 2, 4, 4, 4, 1, 1, 1, 3, 3, 1, 1, 3, 1, 1, 1, 1, 3, 1, 3, 3, 1, 1, 3, 3, 1, 1, 3, 3, 1, 1, 1, 1, 1, 3, 3, 3, 3, 3, 1, 3, 1, 1, 3, 1, 1, 1, 3, 1, 1, 1, 3, 1, 1, 1, 3, 1, 1, 1, 3, 3, 1, 1, 1, 3, 3, 3, 1, 3, 1, 3, 3, 3, 1, 3, 1, 1, 1, 1, 2, 1, 1, 2, 2, 2, 1, 2, 1, 2, 2, 2, 1, 2, 1, 2, 2, 2, 1, 1, 2, 2, 1, 1, 2, 2, 2, 1, 2, 2, 2, 2, 2, 2, 2, 1, 2, 2, 1, 1, 1, 1, 2, 2, 1, 2, 1, 1, 2, 2, 1, 1, 2, 2, 1, 2, 2, 2, 2, 2, 1, 1, 2, 1, 1, 1, 1, 2, 2, 1, 2, 1, 2, 2, 2, 2, 2, 2, 2, 1, 1, 2, 2, 1, 1, 2, 1, 1, 2, 2, 1, 2, 1

Gene ontology terms ranked according to mean of effect sizes in cluster:
embryonic development | regionalization | neurogenesis | chordate embryonic development | central nervous system development | anatomical structure morphogenesis | organ development | gene expression | DBD domain binding | limb development | DNA binding | cell fate determination | somitogenesis | central nervous system neuron development | gastrulation

Corresponding mean of effect sizes of each Gene ontology term:
(In the same order as above)
3.25, 2.52, 2.30, 2.27, 2.00, 1.93, 1.75, 1.64, 1.53, 1.48, 1.27, 1.20, 1.04, 1.04, 0.76

Corresponding total number of genes of each Gene ontology term:
(In the same order as above)
1.2K, 62, 409, 671, 110, 583, 339, 3.8K, 411, 129, 1.2K, 145, 116, 37, 293

Corresponding number of genes of each Gene ontology term with at least one TFBS (of selected TFs):
(In the same order as above)
203, 22, 77, 119, 26, 97, 60, 463, 76, 24, 179, 30, 20, 7, 46

Corresponding total number of TFBS (of selected TFs) for each Gene ontology term (in all genes):
(In the same order as above)
472, 54, 179, 270, 59, 213, 143, 1064, 171, 51, 422, 73, 49, 20, 99

Corresponding number of selected TFs each Gene ontology term is involved with:
(In the same order as above)
4, 4, 4, 4, 4, 4, 4, 4, 4, 4, 4, 4, 4, 4, 4

TFs ranked according to mean of effect sizes in cluster:
V$MZF1_02, V$MZF1_01, V$TFIII_Q6, V$VDR_Q3

Corresponding mean of effect sizes of each TF:
(In the same order as above)
2.06, 1.85, 1.52, 1.50

Corresponding total number of TFBS for each TF (genome-wide):
(In the same order as above)
747, 729, 726, 720

Corresponding total number of TFBS for each TF (in all genes in selected Gene ontology terms):
(In the same order as above)
909, 840, 821, 769

Corresponding number of genes (of selected Gene ontology terms) each TF is involved with:
(In the same order as above)
349, 325, 330, 315

Corresponding number of selected Gene ontology terms each TF is involved with:
(In the same order as above)
15, 15, 15, 15

Cluster for columns 58 to 70, rows 267 to 269

Gene ontology terms

spermatogenesis | protein sumoylation | regulation of gene expression | transcription factor binding | sequence-specific DNA binding | histone acetylation | regulation of transcription | chromatin remodeling complex | gene silencing | histone deacetylase activity | histone deacetylation | DNA methylation | methylation

TFs

V$ETF_Q6 | V$E2F_Q2 | V$E2F1_Q6

Information

All related TFs:
(List of all TFs that are related to any of the PWMs)
DP-1, E2F, E2F+E4, E2F-1, E2F-3a, E2F-4, ETF

Ranked gene list:
(All genes of the selected Gene ontology terms with hits of any of the selected TFs, ranked according to the total number of TFBS)
NR3C1, EGR1, YY1, SIN3A, LBX1, SP3, H2AFZ, DNMT3A, CTCF, CCND1, TP73, TCF3, ID2, E2F4, SSSCA1, SMARCA2, LEF1, JUND, JUNB, HIST1H4I, EZH2, EHMT2, CDKN1C, EP300, CREBBP, SETD1A, IRF1, CDKN1A, ACTB, SMARCA4, RBPJ, PLEC1, NFIC, MYB, HRAS, HMGA1, HIC1, DGAT1, CREB1, CCND2, CBX4, UBE2I, SOCS1, RUNX3, RASSF1, PCNA, MTA1, HNRPDL, CDKN1B, MBD3, ZMYND10, ZFPM1, SRCAP, MNT, MAZ, JMJD3, ING1, HOXA9, HOXA7, HOXA10, HDAC7, GNAS, GATA3, GATA2, EGR2, CACNA1G, BMI1, BCL11B, AKT1, TFAP2A, RARA, POU2F1, IKZF1, ZBTB16, UHRF1, SLC20A1, SKIL, SEMA3B, RUNX2, RNF4, RING1, RCOR1, PTPN6, PELP1, PAX5, IRF4, HOXA5, HMGB1, FOXO1, ENOPH1, CISH, CDKN2D, CDK6, BAZ2A, APRT, TMEM201, TCF7, SOCS3, SMAD7, SLC25A22, SIRT7, SATB2, SAMD4A, REPIN1, RBL1, PURA, PTCH1, POU3F2, PCGF1, PAX6, NSD1, NOTCH1, NNAT, NFATC1, NAB2, MDK, MAPK12, MAFG, MAFA, LRDD, KISS1R, IGF1R, HNRNPL, H3F3A, GPX4, GGNBP2, GFI1, GAMT, FOSB, FLJ32987, FLI1, FASN, EGR3, EBF3, DAZAP1, CTDSP1, CHD3, CHAF1A, CFL1, CDKN2C, CBX8, C19orf6, BCL6B, BCL2L11, ANP32A, ZFP161, WBP7, USF2, UBL4A, TUBB2C, TRIM28, TRERF1, THRA, TGFB1, TARBP2, SPAG6, SOX1, SMARCD3, SMARCC2, SERBP1, RXRA, RRBP1, POU4F1, PCGF2, PAFAH1B1, OAZ1, NR4A2, NFYB, NFIX, MYST4, MLL, MEF2D, IRF2, HSD17B8, H2AFX, GTF2I, DAXX, CTBP1, CNOT1, CHD4, CENPB, CDK9, CDK4, CARM1, CAMK2G, CALM1, ARSG, ARHGDIA, ZEB1, VIM, TNRC6A, TGIF1, TCF12, SMAD2, SLC7A1, SLC5A6, SEPT7, SALL3, PPP1R7, PPIA, PIH1D1, PDPK1, PATZ1, PASK, NFIB, LMX1B, LMNB1, LDB1, INTS6, ILF3, ID3, ICMT, HSP90AA1, HOXD11, HOXB5, HMBS, GTF2A1, GGN, GEMIN4, GAPDH, FUS, FLOT2, FBXL10, EOMES, EFNB2, E2F2, DDAH2, CTDSPL, CTBP2, CREB3L4, COL11A2, CGGBP1, CDC2L6, CDC25C, CBX3, CASP9, C2orf28, BCL2L2, AXIN1, ATXN7, ATF3, ARNTL, ARID1A, AKT3

Corresponding total number of TFBS:
(For each gene listed above, the total number of TFBS for any of the selected TFs, multiplied by the number of selected Gene ontology terms containing that gene)

30, 24, 22, 21, 21, 20, 18, 18, 18, 18, 16, 15, 15, 15, 12, 12, 12, 12, 12, 12, 12, 12, 12, 11, 11, 10, 10, 10, 10, 9, 9, 9, 9, 9, 9, 9, 9, 9, 9, 9, 9, 8, 8, 8, 8, 8, 8, 8, 8, 7, 6, 6, 6, 6, 6, 6, 6, 6, 6, 6, 6, 6, 6, 6, 6, 6, 6, 6, 5, 5, 5, 5, 4, 4, 4, 4, 4, 4, 4, 4, 4, 4, 4, 4, 4, 4, 4, 4, 4, 4, 4, 4, 3, 3, 3, 3, 3, 3, 3, 3, 3, 3, 3, 3, 3, 3, 3, 3, 3, 3, 3, 3, 3, 3, 3, 3, 3, 3, 3, 3, 3, 3, 3, 3, 3, 3, 3, 3, 3, 3, 3, 3, 3, 3, 3, 3, 3, 3, 3, 2, 2, 2, 2, 2, 2, 2, 2, 2, 2, 2, 2, 2, 2, 2, 2, 2, 2, 2, 2, 2, 2, 2, 2, 2, 2, 2, 2, 2, 2, 2, 2, 2, 2, 2, 2, 2, 2, 1, 1, 1, 1, 1, 1, 1, 1, 1, 1, 1, 1, 1, 1, 1, 1, 1, 1, 1, 1, 1, 1, 1, 1, 1, 1, 1, 1, 1, 1, 1, 1, 1, 1, 1, 1, 1, 1, 1, 1, 1, 1, 1, 1, 1, 1

Corresponding number of selected Gene ontology terms each gene is involved with:
(In the same order as above)
10, 8, 11, 7, 7, 10, 6, 6, 6, 6, 8, 5, 5, 5, 4, 6, 4, 4, 4, 12, 6, 6, 4, 11, 11, 5, 5, 10, 5, 9, 3, 3, 3, 9, 3, 3, 3, 3, 9, 3, 3, 4, 4, 4, 4, 4, 4, 4, 4, 7, 3, 2, 3, 2, 2, 2, 2, 2, 2, 2, 3, 2, 2, 2, 3, 3, 3, 2, 2, 5, 5, 5, 5, 4, 2, 4, 2, 2, 2, 2, 2, 2, 2, 4, 4, 2, 4, 2, 2, 2, 4, 4, 2, 2, 2, 3, 1, 1, 1, 1, 1, 1, 1, 3, 1, 1, 1, 1, 1, 1, 3, 1, 1, 1, 1, 1, 1, 1, 1, 1, 3, 1, 1, 1, 1, 3, 1, 1, 1, 1, 1, 1, 1, 3, 3, 1, 1, 1, 1, 1, 1, 1, 1, 1, 2, 1, 2, 1, 1, 2, 1, 1, 1, 1, 2, 2, 1, 1, 1, 2, 2, 1, 1, 1, 1, 1, 2, 1, 2, 1, 2, 1, 2, 1, 2, 2, 1, 2, 2, 2, 1, 1, 1, 1, 1, 1, 1, 1, 1, 1, 1, 1, 1, 1, 1, 1, 1, 1, 1, 1, 1, 1, 1, 1, 1, 1, 1, 1, 1, 1, 1, 1, 1, 1, 1, 1, 1, 1, 1, 1, 1, 1, 1, 1, 1, 1, 1, 1, 1, 1, 1, 1, 1, 1, 1, 1, 1, 1, 1

Corresponding number of selected TFs each gene is involved with:
(In the same order as above)
3, 3, 2, 3, 3, 2, 3, 3, 3, 3, 2, 3, 3, 3, 3, 2, 3, 3, 3, 1, 2, 2, 3, 1, 1, 2, 2, 1, 2, 1, 3, 3, 3, 1, 3, 3, 3, 3, 1, 3, 3, 2, 2, 2, 2, 2, 2, 2, 2, 1, 2, 3, 2, 3, 3, 3, 3, 3, 3, 3, 2, 3, 3, 3, 2, 2, 2, 3, 3, 1, 1, 1, 1, 1, 2, 1, 2, 2, 2, 2, 2, 2, 2, 1, 1, 2, 1, 2, 2, 2, 1, 1, 2, 2, 2, 1, 3, 3, 3, 3, 3, 3, 3, 3, 1, 3, 3, 3, 3, 3, 3, 3, 1, 3, 3, 3, 3, 3, 3, 3, 3, 3, 3, 1, 3, 3, 3, 3, 1, 3, 3, 3, 3, 3, 3, 3, 1, 1, 3, 3, 3, 3, 3, 3, 3, 2, 2, 2, 1, 2, 1, 2, 2, 1, 2, 2, 2, 2, 1, 1, 2, 2, 2, 1, 1, 2, 2, 2, 2, 2, 1, 2, 1, 2, 1, 2, 1, 2, 1, 1, 2, 1, 1, 1, 2, 2, 2, 2, 1, 1, 1, 1, 1, 1, 1, 1, 1, 1, 1, 1, 1, 1, 1, 1, 1, 1, 1, 1, 1, 1, 1, 1, 1, 1, 1, 1, 1, 1, 1, 1, 1, 1, 1, 1, 1, 1, 1, 1, 1, 1, 1, 1, 1, 1, 1, 1, 1, 1

Gene ontology terms ranked according to mean of effect sizes in cluster:
transcription factor binding | sequence-specific DNA binding | regulation of gene expression | histone deacetylation | histone deacetylase activity | spermatogenesis | protein sumoylation | chromatin remodeling complex | gene silencing | DNA methylation | regulation of transcription | histone acetylation | methylation

Corresponding mean of effect sizes of each Gene ontology term:
(In the same order as above)
3.02, 2.40, 2.24, 2.22, 2.11, 2.07, 2.02, 2.02, 1.93, 1.71, 1.65, 1.59, 1.45

Corresponding total number of genes of each Gene ontology term:
(In the same order as above)
231, 118, 310, 144, 108, 397, 166, 105, 355, 437, 187, 283, 715

Corresponding number of genes of each Gene ontology term with at least one TFBS (of selected TFs):
(In the same order as above)
45, 26, 62, 35, 26, 51, 35, 25, 57, 60, 37, 46, 86

Corresponding total number of TFBS (of selected TFs) for each Gene ontology term (in all genes):
(In the same order as above)
93, 51, 116, 63, 50, 96, 64, 45, 114, 116, 66, 90, 170

Corresponding number of selected TFs each Gene ontology term is involved with:
(In the same order as above)
3, 3, 3, 3, 3, 3, 3, 3, 3, 3, 3, 3, 3

TFs ranked according to mean of effect sizes in cluster:
V$E2F_Q2, V$E2F1_Q6, V$ETF_Q6

Corresponding mean of effect sizes of each TF:
(In the same order as above)
2.30, 1.92, 1.89

Corresponding total number of TFBS for each TF (genome-wide):
(In the same order as above)
714, 691, 699

Corresponding total number of TFBS for each TF (in all genes in selected Gene ontology terms):
(In the same order as above)
397, 380, 357

Corresponding number of genes (of selected Gene ontology terms) each TF is involved with:
(In the same order as above)
159, 159, 153

Corresponding number of selected Gene ontology terms each TF is involved with:
(In the same order as above)
13, 13, 13

Cluster for columns 820 to 830, rows 342 to 345

Gene ontology terms

angiogenesis | vascular endothelial growth factor production | endothelial cell migration | regulation of angiogenesis | lymphangiogenesis | vascular endothelial growth factor receptor activity | vascular endothelial growth factor receptor binding | endothelial cell proliferation | regulation of vascular endothelial growth factor production | negative regulation of transferase activity | skeletal muscle regeneration

TFs

V$RP58_01 | V$FREAC3_01 | V$EGR3_01 | V$EGR2_01

Information

All related TFs:
(List of all TFs that are related to any of the PWMs)
Egr-2, Egr-3, FOXC1, RP58

Ranked gene list:
(All genes of the selected Gene ontology terms with hits of any of the selected TFs, ranked according to the total number of TFBS)
VEGFA, AKT1, MKI67, FGFR1, THBS1, TM7SF2, TNF, TYMP, PDGFB, HGF, GGPS1, FGF2, CD44, PLAU, HIF1A, CD34, ANGPT2, VTN, SCARB2, PDGFA, ID2, HRAS, EGR1, CDH5, TNFSF15, TNFSF13, MMP2, IL8, TIE1, TGFB1, SSSCA1, PROX1, PLAUR, PIK3CA, PIGF, NODAL, LIF, FAM126A, EFNA1, CYR61, ANXA5, SLC35A1, SERPINE1, RAF1, PSMP, JUN, SPARC, SMAD2, SEMA3B, RNH1, RAC1, PTGES, PIK3R2, HDAC7, EPAS1, CXCL3, BNIP3, SP4, SOX18, SEMA3G, SDC4, S100A4, PROK1, MMP14, MGC39830, IL6ST, HSP90AA1, GIPC1, F3, ETS1, EPHA1, DCN, CDKN1B, WNT5A, WDHD1, UBASH3B, TRADD, TP73, TNFSF11, TGFBR2, TFAP2A, STAT1, SMAD5, SMAD3, SHB, RYK, RTN4R, RPS6KA1, RHOC, PTGER4, PLCG1, MARK2, KRAS, JUNB, ISYNA1, ING1, IGF2, HOXD10, HMGCR, HIF1AN, GDF2, FH, FGF6, FAS, EPHB4, EPHB1, CXCL12, CTSD, CSF1, CD46, CD248, BAI2, ATF3, AREG, AQP1, AKT3, XPC, VCL, TRAF1, TNXB,

TNFSF12, TGFB3, TCEB2, STAB1, S1PR2, S100A9, S100A13, RALA, PTPN11, PRKCZ, PPP1R15A, PGP, PCSK5, NR2F2, NOL3, MYOM2, MFI2, MDM2, MAPK9, MAPK7, MAP2K5, KLK1, KLF5, IL13, HEY2, GRLF1, FLII, FLI1, FGF10, ELF2, EIF4EBP1, CRP, CLIP1, CITED2, CDH2, CCM2, C20orf85, BRAF, BGLAP, AXL, ANPEP, AKT2, ABCB1

Corresponding total number of TFBS:
(For each gene listed above, the total number of TFBS for any of the selected TFs, multiplied by the number of selected Gene ontology terms containing that gene)
33, 30, 24, 21, 18, 16, 14, 12, 10, 10, 10, 10, 10, 9, 9, 9, 9, 8, 8, 8, 8, 8, 8, 8, 7, 7, 7, 7, 6, 6, 6, 6, 6, 6, 6, 6, 6, 6, 6, 6, 6, 5, 5, 5, 5, 5, 4, 4, 4, 4, 4, 4, 4, 4, 4, 4, 4, 3, 3, 3, 3, 3, 3, 3, 3, 3, 3, 3, 3, 3, 3, 3, 3, 2, 2, 2, 2, 2, 2, 2, 2, 2, 2, 2, 2, 2, 2, 2, 2, 2, 2, 2, 2, 2, 2, 2, 2, 2, 2, 2, 2, 2, 2, 2, 2, 2, 2, 2, 2, 2, 2, 2, 2, 2, 2, 1, 1, 1, 1, 1, 1, 1, 1, 1, 1, 1, 1, 1, 1, 1, 1, 1, 1, 1, 1, 1, 1, 1, 1, 1, 1, 1, 1, 1, 1, 1, 1, 1, 1, 1, 1, 1, 1, 1, 1, 1, 1, 1, 1, 1, 1, 1, 1

Corresponding number of selected Gene ontology terms each gene is involved with:
(In the same order as above)
11, 10, 6, 7, 9, 8, 7, 6, 5, 10, 5, 10, 5, 9, 9, 9, 9, 4, 4, 4, 2, 4, 4, 8, 7, 7, 7, 7, 6, 3, 6, 6, 6, 6, 3, 3, 6, 3, 3, 3, 3, 5, 5, 5, 5, 5, 4, 2, 2, 2, 4, 2, 2, 2, 4, 2, 2, 3, 3, 1, 3, 1, 3, 3, 1, 1, 3, 3, 3, 3, 3, 3, 3, 3, 2, 1, 1, 1, 1, 1, 2, 2, 1, 2, 2, 2, 1, 1, 1, 1, 2, 1, 2, 1, 1, 2, 1, 2, 1, 2, 2, 1, 1, 2, 2, 2, 1, 2, 1, 2, 1, 1, 2, 1, 2, 2, 1, 1, 1, 1, 1, 1, 1, 1, 1, 1, 1, 1, 1, 1, 1, 1, 1, 1, 1, 1, 1, 1, 1, 1, 1, 1, 1, 1, 1, 1, 1, 1, 1, 1, 1, 1, 1, 1, 1, 1, 1, 1, 1, 1, 1, 1, 1, 1

Corresponding number of selected TFs each gene is involved with:
(In the same order as above)
3, 3, 4, 3, 2, 2, 2, 2, 2, 1, 2, 1, 2, 1, 1, 1, 1, 2, 2, 2, 4, 2, 2, 1, 1, 1, 1, 1, 1, 2, 1, 1, 1, 1, 2, 2, 1, 2, 2, 2, 2, 1, 1, 1, 1, 1, 1, 2, 2, 2, 1, 2, 2, 2, 1, 2, 2, 1, 1, 3, 1, 3, 1, 1, 3, 3, 1, 1, 1, 1, 1, 1, 1, 1, 2, 2, 2, 2, 2, 1, 1, 2, 1, 1, 1, 2, 2, 2, 2, 1, 2, 1, 2, 2, 1, 2, 1, 2, 1, 1, 2, 2, 1, 1, 1, 2, 1, 2, 1, 2, 2, 1, 2, 1, 1, 2, 1, 1, 1, 1, 1, 1, 1, 1, 1, 1, 1, 1, 1, 1, 1, 1, 1, 1, 1, 1, 1, 1, 1, 1, 1, 1, 1, 1, 1, 1, 1, 1, 1, 1, 1, 1, 1, 1, 1, 1, 1, 1, 1, 1, 1, 1, 1, 1

Gene ontology terms ranked according to mean of effect sizes in cluster:
regulation of angiogenesis | endothelial cell migration | lymphangiogenesis | vascular endothelial growth factor receptor binding | vascular endothelial growth factor receptor activity | endothelial cell proliferation | regulation of vascular endothelial growth factor production | vascular endothelial growth factor production | negative regulation of transferase activity | angiogenesis | skeletal muscle regeneration

Corresponding mean of effect sizes of each Gene ontology term:
(In the same order as above)
2.26, 2.19, 2.10, 1.60, 1.54, 1.36, 1.17, 1.00, 0.93, 0.71, 0.36

Corresponding total number of genes of each Gene ontology term:
(In the same order as above)
86, 168, 66, 92, 89, 161, 71, 515, 60, 678, 48

Corresponding number of genes of each Gene ontology term with at least one TFBS (of selected TFs):
(In the same order as above)
25, 41, 22, 26, 25, 38, 21, 102, 14, 128, 11

Corresponding total number of TFBS (of selected TFs) for each Gene ontology term (in all genes):
(In the same order as above)
36, 61, 34, 40, 39, 59, 30, 147, 23, 183, 14

Corresponding number of selected TFs each Gene ontology term is involved with:
(In the same order as above)
4, 4, 4, 4, 4, 4, 4, 4, 4, 4, 4

TFs ranked according to mean of effect sizes in cluster:
V$RP58_01, V$EGR3_01, V$EGR2_01, V$FREAC3_01

Corresponding mean of effect sizes of each TF:
(In the same order as above)
2.02, 1.42, 1.35, 0.74

Corresponding total number of TFBS for each TF (genome-wide):
(In the same order as above)
675, 627, 637, 710

Corresponding total number of TFBS for each TF (in all genes in selected Gene ontology terms):
(In the same order as above)
193, 156, 171, 146

Corresponding number of genes (of selected Gene ontology terms) each TF is involved with:
(In the same order as above)
64, 58, 59, 52

Corresponding number of selected Gene ontology terms each TF is involved with:
(In the same order as above)
11, 11, 11, 11

Cluster for columns 757 to 784, rows 147 to 150

Gene ontology terms

Janus kinase activity | macrophage differentiation | protein tyrosine kinase inhibitor activity | transmembrane receptor protein tyrosine kinase activity | protein tyrosine kinase activator activity | protein tyrosine kinase activity | focal adhesion | platelet-derived growth factor receptor activity | platelet-derived growth factor receptor binding | 1-phosphatidylinositol-3-kinase activity | epidermal growth factor receptor activity | epidermal growth factor receptor binding | SAP kinase activity | protein kinase cascade | JUN kinase activity | mitogen-activated protein kinase p38 binding | MAPKKK cascade | MAP kinase 1 activity | MAP kinase 2 activity | activation of MAPKKK activity | activation of MAPK activity | mitogen-activated protein kinase kinase kinase kinase binding | mitogen-activated protein kinase kinase binding | mitogen-activated protein kinase kinase kinase binding | MAP kinase kinase activity | MAP kinase kinase kinase activity | MAP kinase activity | MAP kinase kinase kinase kinase activity

TFs

V$TEL2_Q6 | V$ETS_Q4 | V$ELF1_Q6 | V$NKX25_Q5

Information

All related TFs:
(List of all TFs that are related to any of the PWMs)
ELF-1, ERF, Elf-1, Elk-1, Erg-1, Ets-1, Fli-1, NERF, NERF-1a, NERF-1b, NERF-2, Nkx2-5, Nkx2.5, SAP-1a, Tel-2a, Tel-2b, Tel-2c, c-Ets-1, c-Ets-2

Ranked gene list:
(All genes of the selected Gene ontology terms with hits of any of the selected TFs, ranked according to the total number of TFBS)
SHC1, JUNB, AKT1, CSK, TNF, EGR1, LCK, RPS6KA1, PTPN11, PTPN6, MAP2K3, PTK2B, PIK3R1, IRS1, PKN1, NTRK1, SSSCA1, MMP9, COL11A2, ERBB2, ZAP70, PLCG1, JUN, FASN, STAT3, RAF1, MAPK14, GRB2, RAC2, FGR, CXCR4, CAMK2G, PIK3R2, CANX, ARHGEF7, JAK2,

CDKN1B, TNFRSF1A, TFRC, SOCS3, HRAS, EPHA1, LYN, ZC3H12D, STAT5A, ILK, CISH, CDK5, CDK4, CCND3, CASP8, ANXA6, STAT5B, CREB1, STK24, PTPN7, PRKCD, PIK3CG, MAP3K1, IRF1, RAP1GAP, MAPKAPK3, MAPK11, LIF, CSF1, VAV1, PLA2G6, MAPK12, MAP4K1, MAP3K5, ITGB2, KAT5, SORBS3, PRKCA, MOCOS, MARK2, FYN, DLG4, AKT2, ADRBK1, SLC33A1, EIF4EBP1, BAX, VAV2, SART3, NFKBIA, CDK2, TNK2, SOCS1, NFATC1, NCK1, MAP3K12, LTBR, FAS, CCND2, TUSC2, THPO, SLC9A3R1, RASSF1, NLRP3, FOXO3, DUSP5, CD2BP2, BAK1, ARRB2, ARHGAP4, GGPS1, ZFP36, TYROBP, TXNDC12, TLR9, SMAD3, RGS3, PSMP, PRIM2, PDE4A, NR4A1, NR2C2, MMP14, LCP2, ITGA5, INPP5D, FLJ32987, DUSP2, DAPP1, CD82, ARHGEF2, TNFRSF1B, SPRY2, MAPK10, IL2RB, TRAF1, STAT6, STAT4, RIN1, RARA, PTGER4, NR4A2, NFKBIE, NAB2, MEF2D, MAP3K11, LMNA, ITGAV, GRN, GATA3, EPHA2, CTTN, CTSD, ARHGEF1, ACVR1, ACTB, VIM, TRAF5, TLN1, SH3BP2, SELPLG, RHOG, RALB, PPP1R9B, PLCB1, PEA15, PDLIM7, NFKB1, LASP1, KISS1R, JUND, IRF9, IL23A, GIT1, GADD45A, ETV6, DGAT1, DCTN3, CTF1, CCR7, AGTRAP, ADAMTS4, ZYX, UBE2B, TSG101, TRIP11, TRADD, TPP1, TNFRSF6B, SUB1, STAM2, SNX13, SNX1, SLC2A4RG, RHOC, RERE, RAP1B, PSMC5, PRF1, PRDM1, PKN2, PIK3CD, PHB2, PDK2, NFATC3, NDOR1, MUC4, MR1, MIA, MAP4K5, LSM4, KRT13, KIAA0101, HCST, GPS2, GLI1, GFI1, FURIN, FERMT3, ERBB2IP, EGLN2, ECE1, DVL2, DIAPH1, DIABLO, DDIT3, DBNL, CXCR5, CRTC2, CDK9, CD68, CD19, CCBL1, CALR, BRMS1, BRCA1, ATF3, ARHGAP1, ACTN1, ABI1, WNT10B, WNT10A, WDR1, WASF2, VASP, TXNIP, TSC2, TM7SF2, SPEN, SOCS7, SLC5A5, SH2B2, SELL, RIPK3, RASGRP2, PRKCB, PLEKHA2, PIK3IP1, PARVG, OSR1, NPTN, NF1, NCOR2, NCF4, NCCRP1, MZF1, MPZ, LHX1, LGALS8, KHSRP, KCNA3, IRAK4, IL1RAP, HSP90B1, FOXP1, FOSL2, FHL3, ERN1, EGR4, DUSP22, DUSP16, DOCK2, DFFA, DCTN5, CTLA4, CLIP1, CD180, CCL27, C9orf95, BANF1, ARL6IP5, ARHGEF12, ARAP3, AKT1S1, AIF1, ACAA2, ABCF2

Corresponding total number of TFBS:
(For each gene listed above, the total number of TFBS for any of the selected TFs, multiplied by the number of selected Gene ontology terms containing that gene)
104, 76, 75, 68, 63, 63, 57, 54, 54, 52, 51, 46, 46, 46, 45, 44, 36, 36, 33, 30, 27, 27, 27, 27, 26, 26, 25, 25, 24, 24, 24, 24, 23, 23, 23, 22, 22, 21, 20, 20, 20, 20, 19, 18, 18, 18, 18, 18, 18, 18, 18, 18, 17, 17, 16, 16, 16, 16, 16, 16, 15, 15, 15, 15, 15, 14, 14, 14, 14, 14, 14, 13, 12, 12, 12, 12, 12, 12, 12, 12, 11, 11, 11, 10, 10, 10, 10, 9, 9, 9, 9, 9, 9, 9, 9, 8, 8, 8, 8, 8, 8, 8, 8, 8, 8, 8, 7, 6, 6, 6, 6, 6, 6, 6, 6, 6, 6, 6, 6, 6, 6, 6, 6, 6, 6, 6, 5, 5, 5, 5, 4, 4, 4, 4, 4, 4, 4, 4, 4, 4, 4, 4, 4, 4, 4, 4, 4, 4, 4, 4, 4, 3, 3, 3, 3, 3, 3, 3, 3, 3, 3, 3, 3, 3, 3, 3, 3, 3, 3, 3, 3, 3, 3, 3, 3, 3, 3, 3, 3, 2, 2, 2, 2, 2, 2, 2, 2, 2, 2, 2, 2, 2, 2, 2, 2, 2, 2, 2, 2, 2, 2, 2, 2, 2, 2, 2, 2, 2, 2, 2, 2, 2, 2, 2, 2, 2, 2, 2, 2, 2, 2, 2, 2, 2, 2, 2, 2, 2, 2, 2, 2, 2, 2, 2, 1, 1, 1, 1, 1, 1, 1, 1, 1, 1, 1, 1, 1, 1, 1, 1, 1, 1, 1, 1, 1, 1, 1, 1, 1, 1, 1, 1, 1, 1, 1, 1, 1, 1, 1, 1, 1, 1, 1, 1, 1, 1, 1, 1, 1, 1, 1, 1, 1, 1, 1

Corresponding number of selected Gene ontology terms each gene is involved with:
(In the same order as above)
26, 19, 25, 17, 21, 21, 19, 18, 18, 13, 17, 23, 23, 23, 15, 22, 12, 12, 11, 15, 9, 9, 27, 9, 26, 26, 25, 25, 8, 6, 12, 6, 23, 23, 23, 22, 11, 7, 20, 10, 20, 10, 19, 6, 18, 6, 6, 6, 6, 6, 9, 18, 17, 17, 8, 4, 8, 16, 16, 4, 5, 5, 15, 5, 5, 14, 7, 14, 7, 7, 7, 13, 3, 12, 12, 4, 12, 4, 6, 4, 11, 11, 11, 5, 5, 10, 10, 3, 3, 3, 9, 3, 3, 9, 3, 2, 4, 2, 2, 4, 4, 4, 4, 4, 2, 4, 7, 2, 3, 6, 3, 6, 3, 6, 3, 3, 3, 6, 6, 3, 2, 6, 3, 2, 3, 2, 2, 5, 5, 5, 5, 4, 4, 4, 2, 1, 4, 1, 1, 2, 1, 4, 2, 2, 2, 1, 2, 4, 2, 1, 2, 2, 3, 3, 1, 1, 1, 1, 1, 1, 3, 3, 3, 3, 1, 1, 3, 1, 3, 1, 3, 3, 3, 3, 1, 1, 3, 1, 1, 1, 2, 2, 2, 1, 2, 2, 2, 2, 2, 1, 1, 2, 1, 1, 1, 1, 2, 2, 1, 1, 1, 1, 2, 2, 2, 2, 2, 2, 2, 1, 1, 2, 1, 2, 1, 1, 1, 1, 1, 1, 1, 2, 1, 2, 1, 2, 1, 1, 1, 1, 2, 2, 2, 1, 1, 2, 1, 1, 1, 1, 1, 1, 1, 1, 1, 1, 1, 1, 1, 1, 1, 1, 1, 1, 1, 1, 1, 1, 1, 1, 1, 1, 1, 1, 1, 1, 1, 1, 1, 1, 1, 1, 1, 1, 1, 1, 1, 1, 1, 1, 1, 1, 1, 1, 1, 1, 1, 1, 1

Corresponding number of selected TFs each gene is involved with:
(In the same order as above)
4, 4, 3, 4, 3, 3, 3, 3, 3, 4, 3, 2, 2, 2, 3, 2, 3, 3, 3, 2, 3, 3, 1, 3, 1, 1, 1, 1, 3, 4, 2, 4, 1, 1, 1, 1, 2, 3, 1, 2, 1, 2, 1, 3, 1, 3, 3, 3, 3, 3, 2, 1, 1, 1, 2, 4, 2, 1, 1, 4, 3, 3, 1, 3, 3, 1, 2, 1, 2, 2, 2, 1, 4, 1, 1, 3, 1, 3, 2, 3, 1, 1, 1, 2, 2, 1, 1, 3, 3, 3, 1, 3, 3, 1, 3, 4, 2, 4, 4, 2, 2, 2, 2, 2, 4, 2, 1, 3, 2, 1, 2, 1, 2, 1, 2, 2, 2, 1, 1, 2, 3, 1, 2, 3, 2, 3, 3, 1, 1, 1, 1, 1, 1, 1, 2, 4, 1, 4, 4, 2, 4, 1, 2, 2, 2, 4, 2, 1, 2, 4, 2, 2, 1, 1, 3, 3, 3, 3, 3, 3, 1, 1, 1, 1, 3, 3, 1, 3, 1, 3, 1, 1, 1, 1, 3, 3, 1, 3, 2, 2, 1, 1, 1, 2, 1, 1, 1, 1, 1, 2, 2, 1, 2, 2, 2, 2, 1, 1, 2, 2, 2, 2, 1, 1, 1, 1, 1, 1, 1, 2, 2, 1, 2, 1, 2, 2, 2, 2, 2, 2, 2, 2, 1, 2, 1, 2, 1, 2, 2, 2, 2, 1, 1, 1, 2, 2, 1, 1, 1, 1, 1, 1, 1, 1, 1, 1, 1, 1, 1, 1, 1, 1, 1, 1, 1, 1, 1, 1, 1, 1, 1, 1, 1, 1, 1, 1, 1, 1, 1, 1, 1, 1, 1, 1, 1, 1, 1, 1, 1, 1, 1, 1, 1, 1, 1, 1, 1, 1, 1, 1

Gene ontology terms ranked according to mean of effect sizes in cluster:
protein tyrosine kinase activity | Janus kinase activity | MAP kinase 2 activity | activation of MAPK activity | protein tyrosine kinase activator activity | MAPKKK cascade | protein tyrosine kinase inhibitor activity | 1-phosphatidylinositol-3-kinase activity | focal adhesion | activation of MAPKKK activity | platelet-derived growth factor receptor activity | mitogen-activated protein kinase kinase kinase kinase binding | mitogen-activated protein kinase kinase kinase binding | mitogen-activated protein kinase kinase binding | transmembrane receptor protein tyrosine kinase activity | MAP kinase 1 activity | MAP kinase activity | mitogen-activated protein kinase p38 binding | MAP kinase kinase kinase kinase activity | platelet-derived growth factor receptor binding | macrophage differentiation | MAP kinase kinase kinase activity | epidermal growth factor receptor binding | epidermal growth factor receptor activity | MAP kinase kinase activity | JUN kinase activity | protein kinase cascade | SAP kinase activity

Corresponding mean of effect sizes of each Gene ontology term:
(In the same order as above)
2.31, 2.02, 1.94, 1.93, 1.84, 1.77, 1.75, 1.57, 1.49, 1.45, 1.37, 1.34, 1.34, 1.34, 1.28, 1.25, 1.23, 1.19, 1.17, 1.01, 0.92, 0.89, 0.88, 0.86, 0.86, 0.81, 0.75, -0.07

Corresponding total number of genes of each Gene ontology term:
(In the same order as above)
103, 139, 300, 408, 96, 218, 144, 553, 404, 155, 153, 121, 121, 121, 155, 223, 175, 426, 113, 177, 151, 335, 459, 557, 334, 717, 161, 95

Corresponding number of genes of each Gene ontology term with at least one TFBS (of selected TFs):
(In the same order as above)
32, 41, 72, 86, 27, 55, 38, 114, 80, 39, 36, 35, 35, 35, 32, 53, 38, 90, 26, 37, 31, 69, 88, 101, 68, 139, 35, 23

Corresponding total number of TFBS (of selected TFs) for each Gene ontology term (in all genes):
(In the same order as above)
59, 80, 144, 163, 50, 104, 72, 209, 152, 75, 70, 66, 66, 66, 60, 96, 71, 168, 49, 71, 63, 125, 155, 183, 124, 255, 61, 36

Corresponding number of selected TFs each Gene ontology term is involved with:
(In the same order as above)
4, 4, 4, 4, 4, 4, 4, 4, 4, 4, 4, 4, 4, 4, 4, 4, 4, 4, 4, 4, 4, 4, 4, 4, 4, 4, 4, 4

TFs ranked according to mean of effect sizes in cluster:
V$TEL2_Q6, V$ETS_Q4, V$ELF1_Q6, V$NKX25_Q5

Corresponding mean of effect sizes of each TF:
(In the same order as above)
1.55, 1.44, 1.30, 0.92

Corresponding total number of TFBS for each TF (genome-wide):
(In the same order as above)
707, 718, 717, 695

Corresponding total number of TFBS for each TF (in all genes in selected Gene ontology terms):
(In the same order as above)
745, 751, 758, 639

Corresponding number of genes (of selected Gene ontology terms) each TF is involved with:
(In the same order as above)
132, 143, 138, 118

Corresponding number of selected Gene ontology terms each TF is involved with:
(In the same order as above)
28, 28, 28, 28

Cluster for columns 391 to 538, rows 244 to 263

Gene ontology terms

actin binding | actomyosin | cell motility | establishment of cell polarity | leading edge | cytoskeleton | filamentous actin | actin cytoskeleton | actin filament polymerization | actin filament binding | actin filament | actin filament depolymerization | fibrinolysis | fibrinogen complex | coagulation | hemostasis | fibrinogen binding | platelet activation | protein secretion by the type III secretion system | collagen catabolic process | bone resorption | osteoclast differentiation | sensitization | nerve growth factor receptor activity | nerve growth factor receptor binding | mast cell activation | mast cell degranulation | leukotriene biosynthetic process | icosanoid metabolic process | arachidonic acid metabolic process | cyclooxygenase pathway | lipoxygenase pathway | prostaglandin biosynthetic process | prostanoid biosynthetic process | specific granule | phagocytosis | respiratory burst | piecemeal microautophagy of nucleus | neutrophil degranulation | neutrophil activation | neutrophil chemotaxis | cell surface | membrane raft | receptor complex | cell adhesion | cell migration | basement membrane | proteinaceous extracellular matrix | extracellular matrix | middle lamella-containing extracellular matrix | diapedesis | integrin binding | U-plasminogen activator receptor activity | endothelial cell activation | luteolysis | cell fraction | leukocyte adhesion | membrane attack complex | antibody-dependent cellular cytotoxicity | complement activation | opsonization | neutrophil apoptosis | leukocyte chemotaxis | crotonyl-CoA reductase activity | monocyte chemotaxis | localization of cell | chemotaxis | leukocyte migration | cell chemotaxis | positive chemotaxis | B cell activation | immunoglobulin production | B cell proliferation | immunoglobulin secretion | interferon-gamma production | T-helper 1 type immune response | response to host immune response | adaptive immune response | adaptive immune response based on somatic recombination of immune receptors built from immunoglobulin superfamily domains | chemokine production | interleukin-10 production | interleukin-12 production | cell maturation | MHC class I biosynthetic process | MHC class I protein binding | MHC class II biosynthetic process | MHC class II protein binding | antigen processing and presentation | immune response | type IV hypersensitivity | lymphocyte proliferation | tolerance induction | lymphocyte activation | interleukin-2 production | interleukin-2 receptor activity | T cell proliferation | interleukin-4 production | cytokine production | cytokine secretion | pathogenesis | interleukin-4 receptor activity | interleukin-5 production | evasion or tolerance of immune response of other organism during symbiotic interaction | tryptophan catabolic process | defense response to virus | innate immune response | tumor necrosis factor receptor activity | tumor necrosis factor receptor binding | positive regulation of NF-kappaB transcription factor activity | response to tumor necrosis factor | IkappaB kinase complex | NF-kappaB binding | interleukin-6 receptor activity | interleukin-1 beta production | interleukin-1 production | cytolysis | natural killer cell mediated cytotoxicity | natural killer cell receptor activity | naringenin-chalcone synthase activity | immature T cell proliferation in the thymus | eosinophil activation | leukocyte activation | monocyte activation | mucosal immune response | granulocyte macrophage colony-stimulating factor biosynthetic process | granulocyte macrophage colony-stimulating factor production | interleukin-1 receptor activity | interleukin-6 production | tumor necrosis factor production | macrophage activation | inflammatory response | response to lipopolysaccharide | negative regulation of inflammatory response | regulation of tumor necrosis factor production | negative regulation of tumor necrosis factor production | positive regulation of tumor necrosis factor production | interleukin-1 receptor antagonist activity | acute inflammatory response | granuloma formation | lipopolysaccharide binding | cytokine biosynthetic process | interleukin-8 production | mRNA transcription | chronic inflammatory response | monocyte differentiation | hypersensitivity | hyaluronic acid binding | cytokine activity

TFs

V$FOXP3_Q4 | V$DR3_Q4 | V$PEBP_Q6 | V$AML_Q6 | V$PAX_Q6 | V$NRF2_Q4 | V$MAF_Q6_01 | V$AP1_Q6_01 | V$AP1_Q4_01 | V$AP1_Q6 | V$AP1_C | V$AP1_01 | V$AP1_Q2 | V$AP1FJ_Q2 | V$AP1_Q4 | V$AP1_Q2_01 | V$BACH2_01 | V$NFE2_01 | V$BLIMP1_Q6 | V$CACCCBINDINGFACTOR_Q6

Information

All related TFs:
(List of all TFs that are related to any of the PWMs)
AML1, AML1a, AML1b, AML1c, AML2, AML3, AML3-isoform1, AML3-isoform2, AP-1, Bach1, Bach1:MafK, Bach2, Blimp-1, CACCC-binding, CAR, CAR2:RXR-alpha, CAR:RXR-alpha, FOXP3, FosB, Fra-1, Fra-2, JunB, JunB:Fra-1, JunB:Fra-2, JunD, JunD:Fra-2, JunD:deltaFosB, LCR-F1, MAF, Maf, MafB, MafF, MafG, MafG:MafG, MafK, NF-E2, Nrf1, Nrf1:MafG, Nrf1:MafK, Nrf2, Nrf2:MafG, Nrf2:MafK, Nrf3, Nrf3:MafK, PEBP2, PEBP2alpha, PEBP2alphaA1, PEBP2alphaA2, PEBP2alphaB1, PEBP2alphaB2, PEBP2beta, PEBP2beta1, PEBP2beta2, PEBP2beta3, PXR-1, PXR-1:RXR-alpha, PXR-1A, PXR-1A:RXR-alpha, PXR-1A:RXR-beta, PXR-2, PXR-2:RXR-alpha, Pax-1, Pax-2, Pax-2a, Pax-3, Pax-4a, Pax-4c, Pax-5, Pax-6, Pax-8, Pax6-1, RUNX2-isoform2, RUNX3, RXR-alpha, RXR-beta, Runx3, SXR:RXR-alpha, VDR, YAP1, c-Fos, c-Jun, c-Jun:FosB, c-Jun:JunD, c-Jun:c-Fos, c-Maf, deltaFosB, v-Maf

Ranked gene list:
(All genes of the selected Gene ontology terms with hits of any of the selected TFs, ranked according to the total number of TFBS)
TNF, AKT1, LTA, CSF2, ISYNA1, LTB, TNFRSF1A, CSF1, CD44, ITGB2, CCR7, RELA, CD68, PTPN6, IL2RB, NOS2, IRF1, IL3, IL2RA, LCK, FASN, CDKN1A, PSAP, CD4, TNFRSF4, ISG20, VIM, IL6, TLR9, VASP, SHC1, LBR, SQSTM1, MTHFR, IL23A, CTSD, TNFSF13, VAV1, HGS, TNFRSF18, CD63, ITGAX, MMP9, CSK, PLCG1, MARK2, IL11, MAP2K3, HSPA9, ZYX, RHOA, GPR132, CXCR4, TNFRSF1B, PXN, DUSP2, ICAM2, OSM, MAPK14, LMNA, IL6R, CISH, NFKBIA, SLC2A4RG, SSSCA1, PER1, JUNB, ITGA5, ILK, CALM1, FLNA, DLG4, ARHGEF2, HLA-E, FLNC, TG, PTK2B, PFN1, PSMB8, GAPDH, STAT3, SOCS1, S100A4, PDLIM7, EPB41, TBX21, RASSF5, PSMP, VEGFA, SFTPC, PRAP1, ZAP70, CXCR5, IRF6, CAPG, TRAF2, IL10, STAT6, CD7, ACTB, LASP1, PTMA, TSPAN4, NR3C2, HMOX1, GPI, CCR10, ANXA2, IL2, CD2, ICAM1, DDR1, UBC, S1PR4, FGR, CALR, MAP3K14, PRDX2, PRDM1, ITGA7, IL7R, EZR, CNTNAP1, STAT1, NTRK1, NLRP3, MIF, ACTG1, PIK3CA, VCL, MAP2K1, CD55, SEMA3B, RARA, TNFAIP3, SLC3A2, PDGFB, MYO1C, CFL1, VTN, STAT5B, GIT1, PLAUR, PDE4A, NUP43, MAPK3, EFNA1, RHOC, PTPRCAP, SLC35A1, ITGB7, CD151, ITIH4, TCIRG1, SPHK1, NEU1, LIF, LGALS1, FHOD1, CTNNB1, CD40LG, ANXA6, SH3BP2, EGR1, TRAF3, PPP1R9B, PKN1, LSP1, LAG3, F3, DUSP1, CLEC11A, TNFRSF12A, S1PR1, CD82, PSMD9, IL17C, HBEGF, EPHB2, MAPKAPK2, GLTSCR2, SLC25A22, SOD1, GPR56, WDR1, FOSL1, NBPF1, MDK, FLII, FBRS, EDC4, CIC, BUD31, BCL2L1, ARF1, SCARB1, MYC, HCK, SLC9A3R1, SLC2A1, S1PR2, ROCK2, IKBKE, CD276, CAP1, ARL4C, WNK1, FOS, CD79A, VASN, TBK1, IL10RA, GATA3, ETS1, CD247, ADM, PLAT, RUNX1, RTN4, RAPGEF1, RAB5A, PLEC1, PIK3CG, HLA-B, ENO1, CFLAR, STK16, S100A6, PSTPIP1, IRAK1, IL16, FLI1, CLCF1, TNFRSF9, S100A9, PIK3R2, MYD88, ETV5, AHNAK, ABHD14B, TRADD, TNIP1, TNFRSF13C, SOS1, SFN, RHOG, PDGFRB, NFKB2, HRAS, GADD45B, FYN, FERMT3, CD81, BAX, ALCAM, TGFBR2, STAT5A, LCP2, CRK, VEGFB, UBASH3B, SELPLG, RASGRP2, MBP, LPAR2, GALT, FLJ32987, ECE1, ATF3, PTGDS, PLA2G6, LYN, CORO7, CDK5, CALM3, RAC3, PSMB10, NOS3, MUC1, LTBP3, LAMA3, GYLTL1B, FLT3LG, COL6A1, BCR, ARAP3, SLC25A10, RXRB, PLEKHF1, MMP11, LMLN, FOXP3, CTLA4, CRKL, CRIP1, BAK1, TLR3, RPLP2, PLAU, NFKBIE, MAPRE3, DLX2, DLEC1, CLU, CEBPE, CASP9, TLX2, SLA, PTGER4, POLD4, ITGA6, ITGA3, ICOSLG, ERBB2, CXCR3, BAG3, TRIP6, IL4R, ERBB3, CCL3, UNC84B, TLR5, TCN2, STX1A, STOML2, SPI1, SERPINE1, S100A10, PHB2, PDLIM4, NOLC1, MED25, LTC4S, LRP1, JAK3, HYAL3, HSPA1A, HIVEP2, GCLC, FURIN, CORO1A, COL7A1, COL11A2, CD9, CD27, AMBP, KNG1, VWF, TPM3, TLN1, SDCBP2, RPS6KA1, PIK3R1, IGL@, GALNT6,

EHD1, CDC123, ANGPT2, XYLT1, VILL, SORBS3, NOD2, NGEF, HCLS1, EPB41L1, CTRL, CRTC2, BMP1, BCL2, VAMP8, TNFRSF6B, SLC19A2, SH2B3, RND1, RAPGEF3, PSMB9, PLEKHG6, MBNL1, LAMP1, L1CAM, ITGB3, HDAC3, FEV, CCND2, BCL6, AXL, AGRN, ZFP36, TNFRSF8, SOCS3, RAC2, ICOS, TSLP, TPM1, TNFRSF14, TIMP1, TGFB3, TAP1, SRF, P2RX7, ORAI1, NTRK3, MAP3K1, MAG, LTBP4, LTBP2, LRDD, LOXL1, IRF2, IKBKG, GYPC, GTPBP1, FADD, EPHA2, DLGAP1, CTDSP1, CD37, CCR4, CANX, BCL9L, ACTN1, RALGDS, JAK2, DGKZ, CCL21, CCL17, BTK, ARHGEF1, ZC3H12A, TNK2, TGFB2, SERPINH1, SCRIB, PTPN7, PSMA5, PARVB, PARD6A, MPRIP, MAL, LY96, LPXN, ITGAE, HYAL2, HYAL1, HIC1, DUSP28, DBNL, CD274, CCR6, ARHGAP1, AQP2, ABCB6, WNT1, TNPO1, SWAP-70, SSH1, SLC9A5, RUNX2, RIPK3, RIN1, PNPLA2, PDCD1, LHX1, INS, HSPB6, HSPA1B, FUT7, CYFIP2, CTSZ, COL9A3, CD300A, ABCB9, UNK, ULBP1, TUBGCP2, TNFSF13B, TGFB1I1, SLC9A1, PTGER3, PNKD, PDLIM2, PDGFA, NTF3, NOD1, MYL6, MIP, MGMT, LTB4R2, GUK1, DKFZp779C0757, CXCL6, CTNNA1, CRIP2, CNN1, CAST, C14orf49, BPI, BCAN, ARHGDIA, ADAM15, TUBB2C, TUBA4A, TP53, TFAP2A, RAB3GAP1, NR2C2, KCNIP2, GZMA, GRLF1, BHLHB2, BCL3, ARHGEF7, AQP3, ADIPOQ, ZC3H12D, ZBP1, VWA1, VDR, TNFRSF25, THY1, TACC3, SMPD2, SMAD7, SLC19A1, SEMA4D, RUNX3, PVRL1, POMGNT1, PLXNA1, OSGEP, NCR3, MMP13, MCAM, JUND, GRB10, GABARAP, EPB49, ENTPD2, EMILIN1, DPAGT1, DHCR7, DES, DCTN3, CREBBP, CPA6, COL17A1, CLCN3, CD3D, CALCA, C1QBP, APOBEC3A, AGER, ADCY7, ACD, NPHS1, MTTP, MST1, MME, MGAT1, MAZ, KCNN4, KAT5, ITGA2B, IL27, CREB3, BGLAP, ATXN1, ARID3B, ACTN4, ZMYND10, VDAC1, UNC13D, TPP1, TNXB, TNFRSF10B, THPO, TAPBP, SSFA2, SORL1, SCGB1A1, S100A12, RPL17, PTPN22, PTH1R, PSMC5, PSMC2, PAX6, PAX5, NOS1, NNAT, NFATC2, NFATC1, NCK1, MC1R, MBTPS1, MAPK12, MAP3K5, MAP3K12, MAP1A, MALT1, MAF, LAMC1, ITGB4, HSP90AA1, HSH2D, HIF1A, GALNS, FKBP4, EDN2, DMPK, DAPP1, CXCR6, CIITA, CD3G, BMP2, APOE, APOA1, ADA, VCAN, TUBB, TM7SF2, SPHK2, SDC4, RREB1, PTPRN, PPP2CA, PARVA, NINJ1, MYO1B, MGEA5, MAPK11, LGALS9, KSR1, KRT14, H2AFX, GFI1, FHL3, DYNC1H1, DUSP14, CORO1B, CLDN1, CDC42SE1, CCL27, ARSA, APOM, AP2M1, VIPR1, UTRN, TTYH2, TRPV3, TNNI2, TIPARP, SEPT1, S100P, RTN2, RHOD, RB1CC1, RASSF1, PTPN1, PLXNB1, PIGR, PEPD, PCYT1A, NR4A2, NOXO1, NCKIPSD, MYH9, MYB, MUC17, MRC2, MPZ, MAP3K11, MAP2K7, KRT19, KRT18, KCNA2, IRF9, IQGAP2, IL2RG, IL19, IGSF8, IGFBP7, HMMR, FLNB, ENO2, EMD, ELMO1, DBN1, CYBA, CSF3R, COL2A1, CIB1, CD72, CARD11, CAPN10, CAMK2G, C13orf15, ARHGEF12, APOB, ACP5, ACLY, ACCN2, ZEB2, XBP1, TNR, TBXA2R, TAP2, SH2D3C, SH2D3A, SERPINF1, SDC3, RTN4RL2, RHOB, RASA1, RAB7A, PPP1R1B, POLR2A, PLA2G15, PIM1, PARD3, NTN1, MYO1A, LDLR, IRS1, IGF2, IGF1R, HAVCR2, GTF3A, GPC1, GLI1, FGL2, ECSIT, DNM2, COL18A1, CDSN, CD160, C1R, BMP4, AGPAT1, ADRBK1, ADD3, WDR26, TUBB2A, TSTA3, TSPO, TPX2, TNFSF15, TNFSF14, TMSB4X, TH, TBX5, STRA13, SSH2, SPATA2, SH2D2A, SERPINA1, SEC24D, RTEL1, RPS2, RAP2B, PSME2, PSEN1, PRKCD, PRIM2, PRF1, PPP3CA, PPP2R4, PIP5K1C, PIGW, PELP1, PAK6, P4HTM, NR4A1, NOTCH1, MT2A, MRPL28, MPP5, MAPT, LMAN2, KRT31, KLF6, KCNJ5, IVL, ISG15, IRF4, HSPA8, HLA-DQB1, HAS3, GRM2, GPT, GORASP2, GNAS, GMDS, G6PD, FTH1, FRAP1, FOSB, FBXO8, FAIM3, EPHA1, ELMO3, ELL, EFEMP2, DOK2, DLX4, DEF6, DCTN2, COL12A1, CGN, CDK9, CDK5R2, CDH11, CD97, CD52, CD180, CCRL2, ATN1, ARVCF, ARHGAP4, AFAP1, ADAMTS4, ACCN3, ZFPM1, VKORC1, TUBB3, TRAF5, TNFRSF10D, TJP2, TCF19, SYN1, SPINT1, SMTN, SLC29A3, SLC20A1, SERPING1, SEMA3F, RLF, RGS3, RALA, PPP1R12A, PPIB, PDE1B, NPR1, NPFF, NAT13, MMP19, MICA, MAPRE2, MAP4K1, LRP2, LAMA5, INPP5D, HDAC1, HCST, FOXO3, ETV4, ERCC1, EIF3I, EEF1A1, ECM1, DVL2, CUX1, CTDSP2, CADM4, CACNB3, BTLA, p63RhoGEF, VNN2, USF1, TRIP10, TRAF3IP2, TPM4, TNIK, TMEM8, TLN2, TCEA2, TAOK2, SVIL, SPOCK1, SNX1, SMURF1, SLC9A3, SLC4A2, SLC34A1, SKAP1, SH2B2, RPLP0, RHOH, RGS14, RFX1, RBPJ, RAB27A, PYCARD, PSMD1, PSD, PRPH, PRKCB, PPP1R14A, PPIH, POU3F1, POLR2H, PLXNC1, PCSK7, PCBD1, PARVG, NME1, NLE1, NFE2L2, MYST1, MX1, MMP28, MICAL1, MGC111011, MGAT5, MGAT3, MERTK, MDFIC, MATN4, MADD, LY9, LY6D, LIMS1, LEPREL2, IL21R, IL12A, HIP1R, HAX1, GZMM, GTPBP3, GSTP1, GSK3B, GRB7, GNB2L1, GCNT1, FXYD5, FLOT2, FGF8, ETF1, ERAP2, EPHB4, EFNA4, EEF1A2, EDAR, DOK1, DNM1, DIAPH3, DHX58, DDX5, CTF1, CSRP1, CSE1L, CREM, CHRM1, CHMP2B, CERK, CDKN2D, CDKN1C, CD96, CD84, CD248, CASP6, BAP1, ARPC1B, ARID2, ARHGEF11, ARFIP2, AQP4, AOC3, ANTXR2, ANLN, ANGPTL4, ANG, ALOX5AP, AKR1B1, ADAR, ADAM28, ACSL6, ACSL5, ACHE, ABLIM1, VWA2, VCP, VAC14, UBE2K, TUSC2, TRPC4AP, TREX1, TRAIP, TRAF4, TNN,

TMSB10, TMBIM4, TIAL1, TANK, TAF8, STRN3, SOX9, SLC7A5, SLC7A1, SIGIRR, SEMA7A, SEC61A1, SDHB, S100A11, S100A1, RND2, RNASEL, RAP2A, RAB25, PSME3, PRSS16, PRKCE, POLG, PODN, PLXNB2, PLEKHO1, PIN1, PICK1, PDIA2, PCDH1, PAOX, PAFAH1B1, P2RY11, P2RX1, NUDT2, NRXN2, NDOR1, NAGA, MYO1F, MYO1E, MYH10, MYBPC3, MTMR11, MPV17, MFAP2, MDGA1, MATN3, MAP4K2, MAP2K6, LY6G5B, LRRC4, LRMP, LOXL3, LIMA1, KCNH2, IVNS1ABP, ITPKB, IL20, IDUA, ICAM4, HPDL, HNRNPD, HLA-DMB, HIVEP3, HHIP, HBB, GRK6, GRIA2, GPX4, FOXP1, FLOT1, FLAD1, FKRP, FBXL15, EXTL2, ESPN, EFEMP1, DVL1, DLL1, DAAM1, CSDA, CRY1, COMP, CNP, CHRNB2, CDK4, CD99L2, CD53, CCDC88A, CASP7, CAPN3, CAPN2, BDKRB2, BCAR3, ATP2B4, ARC, APBB1, ANK1, AMH, ADAMTS8, ADAMTS12, ACP2, ACAP1, ZXDA, ZNF384, ZBTB16, WNT11, WARS, USO1, UROD, UNC84A, ULBP3, UCN, UBE2E3, TTYH3, TSC22D3, TRIM69, TRIM21, TNNI3, TNNC2, TNIP2, TNFSF12-TNFSF13, TNFSF12, TNFRSF11A, TMEM11, TINAGL1, SYNE2, SYF2, STK11, ST3GAL1, SREBF1, SPINT2, SPG11, SOST, SNCG, SMS, SLC9A3R2, SLC4A3, SLC2A12, SLC25A1, SLC16A1, SLC11A2, SKP1, SFRS2, SFRP4, SCP2, SCN5A, SART3, RTN4R, RPS6KB2, RPS6KA5, ROBLD3, RIPK4, RGS16, RGS12, REPS1, REM2, RAMP2, RAMP1, RAB40B, PTTG1, PTPN23, PTGIR, PTGFRN, PTGDR, PTCH1, PRPF31, PROZ, PRKAB1, PRCP, PPAP2C, POU2F2, PLEKHA1, PLD2, PKP1, PIGQ, PIAS4, PHGDH, PGP, PEG3, PDZK1, PDYN, PDCD6IP, PARK7, PAK3, PADI4, OPA1, OBSCN, NTF4, NRTN, NPPC, NP, NME3, NDEL1, NCOR2, NBPF3, MYBPH, MYBBP1A, MRO, MRLC3, MMRN2, MLL, MID2, MGC39830, MGC138368, MFN2, MFI2, METAP1, MED15, MCPH1, MBNL2, MAX, MAP3K7IP1, MAP2K4, MAP1S, MAN2A2, LY6E, LIMK2, LEPRE1, LEF1, KIAA1967, KCNJ11, KANK1, INTS6, IL25, IFITM3, ICAM5, HSPB9, HOXA10, HMOX2, HM13, HIVEP1, HEPACAM, HARS, GSR, GPSN2, GPR124, GPAA1, GMFG, GLG1, GIT2, GGPS1, GFM1, GAST, GAS8, GAS7, GALC, FYB, FUT8, FST, FANCG, EXOSC1, EXOC7, EXOC3, EXOC2, ESR2, ERBB2IP, EOMES, EIF4E, EIF2S1, EGR2, EFNA2, DSCR3, DPYSL3, DNAH1, DLAT, DEAF1, DAD1, CYFIP1, CTBP1, CRLF1, CPEB1, CNTFR, CMKLR1, CLIC4, CHPF, CHIC2, CHDH, CEBPD, CEBPA, CDT1, CDKN2C, CDK6, CDH15, CDC14A, CCL18, CCHCR1, CASK, CAPNS1, CA9, C5orf13, C1orf38, C1QL1, BTRC, BLMH, BCAP31, BAT1, ARTN, ARF5, APLNR, APEX1, AMD1, AKAP1, AIRE, AGPAT2, ADRB2, ADD1, ADAM33, ACTR1A, ACTC1, ACSS2, ZNF398, ZMYND11, YWHAQ, WTIP, WNT5B, WNT3A, WHSC1, WDR68, WARS2, VEZT, VDAC2, VAPA, UTS2R, USP14, USF2, UCP2, UBQLN1, UBA7, TWF1, TTBK1, TSPAN10, TSC1, TRPV2, TRERF1, TPT1, TPP2, TOR1B, TNNT1, TMOD4, TIFA, TGM4, TCOF1, TCEB2, SUFU, STX6, STRA8, SSR1, SSH3, SPRY2, SPAG7, SP140, SNRPG, SLC7A14, SLC35C1, SLC22A7, SLC22A11, SIGLEC12, SGMS1, SGK1, SFRS17A, SFRP5, SETD4, SERPINB1, SERBP1, SEMA4A, SEC62, SAR1A, S100A13, RYK, RSF1, RPS6KA2, RPS14, RPL36A, ROBO3, RNASEK, RGS2, RGL4, RASL12, RASGRP1, RAD52, RAD18, RAB43, RAB1A, PTP4A3, PTK6, PTBP1, PSMB7, PSMA7, PSENEN, PRKCI, PRDX1, PPARGC1B, PLXDC1, PLTP, PLEKHO2, PLEK2, PIP5K1A, PIGC, PHOX2B, PHF12, PDZK1IP1, PDZD3, PDX1, PCSK6, PCDH12, OTP, NUMBL, NSMAF, NSD1, NRD1, NPHP1, NPC1L1, NLK, NLGN3, NKX6-2, NGRN, NFKBIL1, NF1, NCAM2, NAV2, NARS, MYL3, MYH6, MYCL1, MX2, MVD, MRPS12, MPHOSPH6, MMEL1, MMACHC, MEN1, MCHR2, MBOAT7, MAN1A1, LY6G5C, LRRN1, LRP4, LRIG1, LPHN2, LOR, LONP1, LMNB2, LGALS4, LGALS3BP, LAT2, KRT85, KRT16, KLF10, KIRREL2, KEAP1, KCNK2, KCNJ4, KCNA1, IRAK1BP1, INSC, INHA, IL1F6, IL17RB, IGFBP6, IFITM1, IFIT2, IER3, HSPB7, HOXA13, HISPPD1, HBS1L, HBD, GSTM2, GSC, GRIN2D, GOLGA2, GLMN, GLIPR1, GCS1, GCLM, GBA, GABBR1, FUBP1, FOXF1, FLVCR2, FLJ21865, FIP1L1, FGD3, FGD2, FBXW7, FANCE, EZH2, EXOSC3, EVI1, ERCC4, ERCC2, ELF4, EIF2S2, EHF, EBF1, DYRK1A, DYNC1I2, DUSP11, DNM1L, DISC1, DICER1, DFNB31, DCLRE1C, DAXX, CYP27B1, CYP11A1, CUL5, CRYAA, CRTC1, CRLF2, CPZ, CPOX, CNPY3, CNBP, CLC, CHEK2, CDR2L, CDC42EP1, CDC37, CDC2L5, CD109, CARD8, C1orf9, BRAF, BMX, BLZF1, BIN2, BCS1L, BCL2A1, BATF, ATF4, ARID4B, ARHGEF19, ARHGAP10, AQP1, APBB2, ANTXR1, AKTIP, AKT3, AGPAT9, AGPAT4, ADRBK2, ABLIM2, ABCD1, AATF, AANAT

Corresponding total number of TFBS:
(For each gene listed above, the total number of TFBS for any of the selected TFs, multiplied by the number of selected Gene ontology terms containing that gene)
2096, 810, 700, 672, 546, 525, 507, 504, 500, 460, 432, 400, 364, 361, 344, 336, 330, 320, 320, 290, 270, 266, 261, 252, 250, 248, 234, 220, 217, 216, 216, 215, 210, 208, 208, 208, 204, 198, 195, 192, 189, 188, 185, 182, 180, 171, 171, 170, 170, 169, 165, 165, 165, 162, 162, 162, 160, 156, 156, 156, 154, 153, 152,

150, 144, 144, 144, 144, 144, 144, 143, 143, 143, 140, 132, 130, 130, 128, 126, 126, 124, 120, 120, 117,
117, 115, 112, 112, 111, 110, 110, 108, 108, 105, 105, 104, 103, 100, 100, 100, 99, 98, 96, 96, 96, 96, 96,
96, 94, 94, 93, 91, 90, 90, 90, 90, 84, 80, 80, 80, 80, 80, 80, 78, 78, 78, 78, 78, 76, 75, 75, 75, 72, 72, 70,
70, 70, 70, 70, 68, 68, 68, 66, 66, 66, 66, 66, 65, 65, 64, 63, 63, 62, 60, 60, 60, 60, 60, 60, 60, 60, 60, 57,
57, 56, 56, 56, 56, 56, 56, 56, 56, 55, 55, 55, 54, 54, 54, 54, 52, 52, 51, 50, 50, 49, 49, 48, 48, 48, 48, 48,
48, 48, 48, 48, 46, 46, 46, 45, 45, 45, 45, 45, 45, 45, 45, 44, 44, 44, 42, 42, 42, 42, 42, 42, 42, 41, 40, 40,
40, 40, 40, 40, 40, 40, 40, 39, 39, 39, 39, 39, 39, 39, 38, 38, 38, 38, 38, 38, 38, 36, 36, 36, 36, 36, 36, 36,
36, 36, 36, 36, 36, 36, 36, 36, 35, 34, 34, 34, 33, 33, 33, 33, 33, 33, 33, 33, 33, 33, 32, 32, 32, 32, 32, 32,
30, 30, 30, 30, 30, 30, 30, 30, 30, 30, 30, 28, 28, 28, 28, 28, 28, 28, 28, 28, 28, 27, 27, 27, 27, 27, 27, 27,
27, 27, 27, 26, 26, 26, 26, 26, 26, 26, 26, 26, 26, 25, 25, 25, 25, 24, 24, 24, 24, 24, 24, 24, 24, 24, 24, 24,
24, 24, 24, 24, 24, 24, 24, 24, 24, 24, 24, 24, 24, 24, 24, 23, 22, 22, 22, 22, 22, 22, 22, 22, 22, 22, 22, 21,
21, 21, 21, 21, 21, 21, 21, 21, 21, 21, 20, 20, 20, 20, 20, 20, 20, 20, 20, 20, 20, 20, 20, 20, 20, 20, 20, 20,
19, 19, 19, 19, 19, 18, 18, 18, 18, 18, 18, 18, 18, 18, 18, 18, 18, 18, 18, 18, 18, 18, 18, 18, 18, 18, 18, 18,
18, 18, 18, 18, 18, 18, 17, 17, 17, 17, 17, 17, 17, 16, 16, 16, 16, 16, 16, 16, 16, 16, 16, 16, 16, 16, 16, 16,
16, 16, 16, 16, 16, 16, 16, 16, 16, 15, 15, 15, 15, 15, 15, 15, 15, 15, 15, 15, 15, 15, 15, 15, 15, 15, 15, 15,
15, 14, 14, 14, 14, 14, 14, 14, 14, 14, 14, 14, 14, 14, 14, 14, 14, 14, 14, 14, 14, 14, 14, 14, 14, 14, 14, 14,
14, 13, 13, 13, 13, 13, 13, 13, 13, 13, 13, 13, 13, 13, 13, 12, 12, 12, 12, 12, 12, 12, 12, 12, 12, 12, 12, 12,
12, 12, 12, 12, 12, 12, 12, 12, 12, 12, 12, 12, 12, 12, 12, 12, 12, 12, 12, 12, 12, 12, 12, 12, 12, 12, 12, 11,
11, 11, 11, 11, 11, 11, 11, 11, 11, 11, 11, 11, 11, 11, 10, 10, 10, 10, 10, 10, 10, 10, 10, 10, 10, 10, 10, 10,
10, 10, 10, 10, 10, 10, 10, 10, 10, 10, 10, 10, 10, 10, 10, 10, 10, 10, 10, 10, 10, 10, 10, 10, 10, 10, 10, 10,
10, 10, 10, 10, 10, 10, 10, 9, 9, 9, 9, 9, 9, 9, 9, 9, 9, 9, 9, 9, 9, 9, 9, 9, 9, 9, 9, 9, 9, 9, 9, 9, 9, 9, 9, 8, 8, 8, 8,
8, 8, 8, 8, 8, 8, 8, 8, 8, 8, 8, 8, 8, 8, 8, 8, 8, 8, 8, 8, 8, 8, 8, 8, 8, 8, 8, 8, 8, 8, 8, 8, 8, 8, 8, 8, 8, 8, 8, 8, 8, 8,
8, 8, 8, 8, 8, 8, 8, 8, 8, 8, 7, 7, 7, 7, 7, 7, 7, 7, 7, 7, 7, 7, 7, 7, 7, 7, 7, 7, 7, 7, 7, 7, 7, 7, 7, 7, 7, 7, 7, 7, 7, 7,
7, 7, 7, 7, 7, 7, 7, 6, 6, 6, 6, 6, 6, 6, 6, 6, 6, 6, 6, 6, 6, 6, 6, 6, 6, 6, 6, 6, 6, 6, 6, 6, 6, 6, 6, 6, 6, 6, 6, 6, 6, 6,
6, 6, 6, 6, 6, 6, 6, 6, 6, 6, 6, 6, 6, 6, 6, 6, 6, 6, 6, 6, 6, 6, 6, 6, 6, 6, 6, 6, 6, 6, 6, 6, 6, 6, 6, 6, 6, 6, 6, 6, 6, 6,
6, 6, 6, 6, 5, 5, 5, 5, 5, 5, 5, 5, 5, 5, 5, 5, 5, 5, 5, 5, 5, 5, 5, 5, 5, 5, 5, 5, 5, 5, 5, 5, 5, 5, 5, 5, 5, 5, 5, 5, 5, 5,
5, 5, 5, 5, 5, 5, 4, 4, 4, 4, 4, 4, 4, 4, 4, 4, 4, 4, 4, 4, 4, 4, 4, 4, 4, 4, 4, 4, 4, 4, 4, 4, 4, 4, 4, 4, 4, 4, 4, 4, 4, 4,
4, 4, 4, 4, 4, 4, 4, 4, 4, 4, 4, 4, 4, 4, 4, 4, 4, 4, 4, 4, 4, 4, 4, 4, 4, 4, 4, 4, 4, 4, 4, 4, 4, 4, 4, 4, 4, 4, 4, 4, 4, 4,
4, 4, 4, 4, 4, 4, 4, 4, 4, 4, 4, 4, 4, 4, 4, 4, 4, 4, 4, 4, 4, 4, 4, 4, 4, 4, 4, 4, 4, 4, 4, 4, 4, 4, 4, 4, 3, 3, 3, 3, 3, 3,
3, 3, 3, 3, 3, 3, 3, 3, 3, 3, 3, 3, 3, 3, 3, 3, 3, 3, 3, 3, 3, 3, 3, 3, 3, 3, 3, 3, 3, 3, 3, 3, 3, 3, 3, 3, 3, 3, 3, 3, 3, 3,
3, 3, 3, 3, 3, 3, 3, 3, 3, 3, 3, 3, 3, 3, 3, 3, 3, 3, 3, 3, 3, 3, 3, 3, 3, 3, 3, 3, 3, 3, 3, 3, 3, 3, 3, 3, 3, 3, 3, 3, 3, 3,
3, 3, 3, 3, 3, 3, 3, 3, 3, 3, 3, 3, 3, 3, 3, 3, 3, 3, 3, 3, 3, 3, 3, 2, 2, 2, 2, 2, 2, 2, 2, 2, 2, 2, 2, 2, 2, 2, 2, 2, 2, 2,
2, 2, 2, 2, 2, 2, 2, 2, 2, 2, 2, 2, 2, 2, 2, 2, 2, 2, 2, 2, 2, 2, 2, 2, 2, 2, 2, 2, 2, 2, 2, 2, 2, 2, 2, 2, 2, 2, 2, 2, 2, 2,
2, 2, 2, 2, 2, 2, 2, 2, 2, 2, 2, 2, 2, 2, 2, 2, 2, 2, 2, 2, 2, 2, 2, 2, 2, 2, 2, 2, 2, 2, 2, 2, 2, 2, 2, 2, 2, 2, 2, 2, 2, 2,
2, 2, 2, 2, 2, 2, 2, 2, 2, 2, 2, 2, 2, 2, 2, 2, 2, 2, 2, 2, 2, 2, 2, 2, 2, 2, 2, 2, 2, 2, 2, 2, 2, 2, 2, 2, 2, 2, 2, 2, 2, 2,
2, 2, 2, 2, 2, 2, 2, 2, 2, 2, 2, 2, 2, 2, 2, 2, 2, 2, 2, 2, 2, 2, 2, 2, 2, 2, 2, 2, 2, 2, 2, 2, 2, 2, 2, 2, 2, 2, 2, 2, 2, 2,
2, 2, 2, 2, 2, 2, 2, 2, 2, 2, 2, 2, 2, 2, 2, 2, 2, 2, 2, 2, 2, 1, 1, 1, 1, 1, 1, 1, 1, 1, 1, 1, 1, 1, 1, 1, 1, 1, 1, 1, 1, 1,
1, 1, 1, 1, 1, 1, 1, 1, 1, 1, 1, 1, 1, 1, 1, 1, 1, 1, 1, 1, 1, 1, 1, 1, 1, 1, 1, 1, 1, 1, 1, 1, 1, 1, 1, 1, 1, 1, 1, 1, 1, 1,
1, 1, 1, 1, 1, 1, 1, 1, 1, 1, 1, 1, 1, 1, 1, 1, 1, 1, 1, 1, 1, 1, 1, 1, 1, 1, 1, 1, 1, 1, 1, 1, 1, 1, 1, 1, 1, 1, 1, 1, 1, 1,
1, 1, 1, 1, 1, 1, 1, 1, 1, 1, 1, 1, 1, 1, 1, 1, 1, 1, 1, 1, 1, 1, 1, 1, 1, 1, 1, 1, 1, 1, 1, 1, 1, 1, 1, 1, 1, 1, 1, 1, 1, 1,
1, 1, 1, 1, 1, 1, 1, 1, 1, 1, 1, 1, 1, 1, 1, 1, 1, 1, 1, 1, 1, 1, 1, 1, 1, 1, 1, 1, 1, 1, 1, 1, 1, 1, 1, 1, 1, 1, 1, 1, 1, 1,
1, 1, 1, 1, 1, 1, 1, 1, 1, 1, 1, 1, 1, 1, 1, 1, 1, 1, 1, 1, 1, 1, 1, 1, 1, 1, 1, 1, 1, 1, 1, 1, 1, 1, 1, 1, 1, 1, 1, 1, 1, 1,
1, 1, 1, 1, 1, 1, 1, 1, 1, 1, 1, 1, 1, 1, 1, 1, 1, 1, 1, 1, 1, 1, 1, 1, 1, 1, 1, 1, 1, 1, 1, 1, 1, 1, 1, 1, 1, 1, 1, 1, 1, 1,
1, 1, 1, 1, 1

Corresponding number of selected Gene ontology terms each gene is involved with:
(In the same order as above)
131, 54, 50, 84, 42, 35, 39, 42, 50, 92, 36, 40, 28, 19, 43, 48, 22, 64, 64, 29, 45, 19, 29, 84, 25, 62, 18,
110, 31, 18, 18, 43, 15, 26, 16, 13, 102, 22, 15, 16, 27, 47, 37, 13, 12, 19, 19, 10, 34, 13, 33, 15, 33, 27,
27, 9, 20, 12, 78, 12, 11, 9, 38, 10, 9, 24, 9, 18, 16, 16, 13, 11, 11, 70, 12, 10, 26, 8, 9, 9, 31, 12, 8, 13, 9,
23, 8, 14, 37, 11, 22, 9, 12, 105, 21, 13, 103, 20, 10, 20, 9, 7, 8, 24, 12, 12, 6, 12, 94, 47, 93, 7, 15, 6, 15,
15, 7, 8, 8, 8, 8, 20, 5, 39, 13, 6, 26, 6, 38, 25, 15, 15, 6, 4, 7, 10, 7, 7, 7, 34, 17, 4, 22, 6, 11, 22, 6, 5, 5,
64, 7, 9, 31, 4, 10, 15, 20, 6, 4, 20, 60, 30, 3, 19, 7, 4, 14, 8, 7, 28, 7, 14, 5, 11, 5, 9, 18, 9, 54, 13, 13, 3,
10, 5, 7, 7, 6, 6, 3, 6, 24, 4, 6, 16, 8, 23, 23, 23, 9, 5, 5, 5, 5, 45, 5, 15, 44, 22, 4, 3, 7, 6, 3, 7, 6, 3, 41, 4, 4,
8, 10, 2, 40, 8, 4, 10, 3, 3, 3, 13, 13, 3, 3, 19, 19, 19, 38, 19, 2, 2, 9, 3, 9, 18, 3, 9, 12, 4, 6, 3, 18, 6, 18, 9,
12, 7, 17, 17, 17, 3, 33, 11, 3, 33, 3, 3, 3, 3, 3, 16, 16, 32, 2, 8, 16, 3, 6, 15, 15, 3, 5, 3, 15, 5, 15, 3, 7, 2, 2,
7, 4, 28, 28, 4, 2, 4, 27, 3, 27, 3, 3, 3, 3, 9, 3, 9, 2, 2, 13, 2, 13, 13, 13, 13, 26, 2, 5, 5, 5, 25, 2, 24, 8, 4, 2,

12, 24, 3, 8, 3, 12, 3, 8, 6, 12, 3, 8, 4, 3, 4, 6, 4, 8, 24, 24, 8, 23, 22, 11, 2, 2, 2, 22, 11, 2, 2, 11, 11, 3, 3, 3, 21, 21, 3, 3, 7, 3, 3, 21, 4, 5, 20, 1, 4, 5, 10, 20, 4, 20, 10, 20, 2, 4, 4, 4, 5, 10, 1, 19, 19, 19, 19, 9, 9, 6, 18, 6, 9, 9, 6, 2, 6, 9, 9, 3, 3, 1, 6, 6, 6, 2, 3, 9, 3, 3, 3, 3, 18, 9, 1, 6, 1, 17, 1, 17, 17, 17, 1, 1, 1, 16, 8, 4, 1, 2, 8, 4, 4, 2, 16, 2, 4, 4, 4, 1, 1, 8, 16, 16, 8, 4, 8, 1, 15, 5, 5, 1, 5, 3, 1, 5, 5, 5, 5, 3, 3, 5, 5, 3, 5, 5, 3, 2, 7, 7, 14, 7, 7, 7, 2, 2, 7, 14, 14, 1, 14, 2, 2, 7, 1, 14, 7, 1, 2, 7, 2, 14, 7, 7, 7, 1, 1, 13, 13, 13, 13, 1, 13, 13, 1, 1, 13, 1, 13, 1, 4, 3, 1, 12, 6, 1, 6, 6, 3, 3, 1, 6, 4, 3, 6, 12, 12, 3, 2, 6, 4, 3, 2, 4, 1, 2, 12, 3, 6, 4, 12, 2, 3, 12, 6, 12, 4, 2, 3, 11, 11, 11, 11, 1, 1, 1, 11, 11, 11, 1, 11, 1, 1, 11, 1, 5, 2, 2, 5, 10, 10, 5, 10, 2, 5, 10, 10, 5, 5, 1, 2, 5, 5, 10, 1, 10, 10, 10, 1, 10, 2, 5, 1, 5, 10, 10, 5, 10, 10, 5, 10, 10, 5, 2, 1, 2, 5, 10, 2, 10, 5, 5, 2, 9, 1, 3, 3, 9, 1, 3, 1, 9, 1, 9, 9, 3, 3, 1, 9, 1, 1, 1, 3, 1, 3, 9, 1, 3, 9, 3, 1, 8, 8, 1, 2, 1, 4, 2, 1, 2, 8, 2, 1, 2, 4, 8, 4, 1, 2, 1, 2, 1, 8, 2, 8, 4, 4, 2, 8, 8, 8, 4, 4, 8, 4, 4, 4, 8, 8, 4, 2, 8, 8, 4, 8, 4, 8, 8, 8, 1, 2, 2, 4, 8, 2, 4, 1, 1, 7, 7, 7, 7, 1, 1, 7, 7, 1, 7, 7, 7, 1, 1, 1, 1, 7, 7, 7, 7, 7, 7, 7, 7, 7, 7, 1, 7, 1, 7, 7, 7, 7, 7, 7, 1, 1, 1, 3, 1, 2, 2, 6, 6, 1, 6, 3, 6, 1, 2, 2, 1, 6, 1, 2, 1, 3, 3, 3, 1, 6, 3, 6, 6, 3, 1, 2, 1, 1, 1, 3, 1, 2, 1, 2, 2, 2, 1, 3, 6, 3, 6, 6, 6, 6, 3, 2, 6, 1, 2, 2, 2, 6, 2, 6, 1, 6, 3, 1, 3, 1, 3, 1, 1, 3, 6, 2, 2, 6, 6, 3, 6, 3, 1, 2, 3, 3, 6, 2, 1, 1, 1, 5, 5, 5, 5, 5, 1, 5, 1, 1, 5, 5, 1, 1, 5, 5, 5, 1, 5, 1, 1, 5, 5, 5, 5, 5, 5, 5, 5, 5, 1, 5, 1, 1, 1, 5, 1, 5, 1, 1, 1, 5, 2, 4, 1, 2, 4, 2, 2, 1, 2, 1, 4, 4, 4, 1, 4, 4, 1, 1, 2, 4, 2, 1, 1, 4, 1, 4, 4, 1, 1, 4, 4, 2, 2, 2, 1, 1, 1, 2, 4, 4, 2, 4, 4, 4, 4, 2, 1, 4, 4, 4, 2, 4, 2, 4, 1, 4, 2, 1, 4, 4, 4, 1, 2, 1, 2, 2, 4, 4, 2, 1, 1, 1, 1, 2, 1, 2, 4, 4, 4, 2, 2, 2, 4, 2, 4, 1, 4, 2, 2, 4, 4, 2, 4, 2, 4, 4, 4, 4, 4, 2, 4, 4, 4, 1, 4, 2, 2, 4, 2, 4, 1, 1, 2, 2, 3, 1, 3, 1, 1, 1, 1, 3, 3, 3, 3, 3, 1, 3, 3, 1, 3, 3, 3, 3, 1, 1, 3, 1, 3, 3, 1, 3, 3, 3, 3, 1, 3, 3, 3, 1, 1, 1, 1, 1, 1, 3, 1, 3, 1, 1, 3, 1, 1, 3, 3, 3, 1, 1, 3, 1, 3, 1, 3, 1, 1, 1, 3, 3, 1, 3, 1, 3, 1, 3, 3, 3, 3, 1, 3, 3, 1, 3, 1, 3, 3, 1, 3, 1, 3, 3, 3, 1, 1, 3, 3, 1, 3, 1, 1, 1, 3, 3, 3, 3, 3, 3, 3, 3, 1, 3, 3, 1, 1, 3, 3, 1, 1, 2, 1, 1, 2, 2, 2, 1, 2, 1, 2, 1, 1, 2, 1, 2, 2, 1, 2, 1, 1, 2, 1, 1, 2, 2, 1, 1, 1, 1, 1, 2, 1, 2, 2, 2, 1, 1, 2, 2, 1, 1, 1, 2, 1, 2, 2, 1, 2, 2, 2, 1, 1, 2, 1, 2, 2, 1, 1, 2, 2, 2, 2, 2, 1, 1, 2, 2, 1, 2, 1, 2, 2, 1, 1, 2, 2, 1, 2, 2, 2, 1, 2, 1, 1, 1, 2, 2, 1, 1, 1, 2, 1, 2, 2, 2, 1, 1, 1, 1, 2, 2, 2, 2, 2, 2, 2, 1, 1, 1, 2, 2, 1, 1, 2, 2, 1, 2, 1, 1, 2, 2, 2, 2, 2, 1, 1, 1, 1, 2, 2, 2, 2, 1, 1, 1, 1, 1, 1, 2, 1, 1, 2, 2, 1, 1, 2, 2, 1, 1, 2, 2, 2, 1, 2, 1, 1, 2, 1, 2, 1, 1, 1, 2, 1, 2, 2, 1, 2, 1, 2, 2, 2, 1, 2, 2, 2, 2, 1, 2, 1, 1, 1, 2, 2, 1, 1, 1, 2, 1, 2, 2, 1, 2, 2, 1, 2, 2, 1, 2, 2, 2, 2, 1, 2, 2, 2, 2, 1

Corresponding number of selected TFs each gene is involved with:
(In the same order as above)
16, 15, 14, 8, 13, 15, 13, 12, 10, 5, 12, 10, 13, 19, 8, 7, 15, 5, 5, 10, 6, 14, 9, 3, 10, 4, 13, 2, 7, 12, 12, 5, 14, 8, 13, 16, 2, 9, 13, 12, 7, 4, 5, 14, 15, 9, 9, 17, 5, 13, 5, 11, 5, 6, 6, 18, 8, 13, 2, 13, 14, 17, 4, 15, 16, 6, 16, 8, 9, 9, 11, 13, 13, 2, 11, 13, 5, 16, 14, 14, 4, 10, 15, 9, 13, 5, 14, 8, 3, 10, 5, 12, 9, 1, 5, 8, 1, 5, 10, 5, 11, 14, 12, 4, 8, 8, 16, 8, 1, 2, 1, 13, 6, 15, 6, 6, 12, 10, 10, 10, 10, 4, 16, 2, 6, 13, 3, 13, 2, 3, 5, 5, 12, 18, 10, 7, 10, 10, 10, 2, 4, 17, 3, 11, 6, 3, 11, 13, 13, 1, 9, 7, 2, 15, 6, 4, 3, 10, 15, 3, 1, 2, 19, 3, 8, 14, 4, 7, 8, 2, 8, 4, 11, 5, 11, 6, 3, 6, 1, 4, 4, 17, 5, 10, 7, 7, 8, 8, 16, 8, 2, 12, 8, 3, 6, 2, 2, 2, 5, 9, 9, 9, 9, 1, 9, 3, 1, 2, 11, 14, 6, 7, 14, 6, 7, 14, 1, 10, 10, 5, 4, 20, 1, 5, 10, 4, 13, 13, 13, 3, 3, 13, 13, 2, 2, 2, 1, 2, 19, 19, 4, 12, 4, 2, 12, 4, 3, 9, 6, 12, 2, 6, 2, 4, 3, 5, 2, 2, 2, 11, 1, 3, 11, 1, 11, 11, 11, 11, 11, 2, 2, 1, 16, 4, 2, 10, 5, 2, 2, 10, 6, 10, 2, 6, 2, 10, 4, 14, 14, 4, 7, 1, 1, 7, 14, 7, 1, 9, 1, 9, 9, 9, 9, 3, 9, 3, 13, 13, 2, 13, 2, 2, 2, 2, 1, 13, 5, 5, 5, 1, 12, 1, 3, 6, 12, 2, 1, 8, 3, 8, 2, 8, 3, 4, 2, 8, 3, 6, 8, 6, 4, 6, 3, 1, 1, 3, 1, 1, 2, 11, 11, 11, 1, 2, 11, 11, 2, 2, 7, 7, 7, 1, 1, 7, 7, 3, 7, 7, 1, 5, 4, 1, 20, 5, 4, 2, 1, 5, 1, 2, 1, 10, 5, 5, 5, 4, 2, 19, 1, 1, 1, 1, 2, 2, 3, 1, 3, 2, 2, 3, 9, 3, 2, 2, 6, 6, 18, 3, 3, 3, 9, 6, 2, 6, 6, 6, 6, 1, 2, 18, 3, 17, 1, 17, 1, 1, 1, 17, 16, 16, 1, 2, 4, 16, 8, 2, 4, 4, 8, 1, 8, 4, 4, 4, 16, 16, 2, 1, 1, 2, 4, 2, 15, 1, 3, 3, 15, 3, 5, 15, 3, 3, 3, 3, 5, 5, 3, 3, 5, 3, 3, 5, 7, 2, 2, 1, 2, 2, 2, 7, 7, 2, 1, 1, 14, 1, 7, 7, 2, 14, 1, 2, 14, 7, 2, 7, 1, 2, 2, 2, 13, 13, 1, 1, 1, 1, 13, 1, 1, 13, 13, 1, 13, 1, 12, 3, 4, 12, 1, 2, 12, 2, 2, 4, 4, 12, 2, 3, 4, 2, 1, 1, 4, 6, 2, 3, 4, 6, 3, 12, 6, 1, 4, 2, 3, 1, 6, 4, 1, 2, 1, 3, 6, 4, 1, 1, 1, 1, 11, 11, 11, 1, 1, 1, 11, 1, 11, 11, 1, 10, 2, 5, 5, 2, 1, 1, 2, 1, 5, 2, 1, 1, 2, 2, 10, 5, 2, 2, 1, 10, 1, 1, 1, 10, 1, 5, 2, 10, 2, 1, 1, 2, 1, 1, 2, 1, 1, 2, 5, 10, 5, 2, 1, 5, 1, 2, 2, 5, 1, 9, 3, 3, 1, 9, 3, 9, 1, 9, 1, 1, 3, 3, 9, 1, 9, 9, 9, 3, 9, 3, 1, 9, 3, 1, 3, 9, 1, 1, 8, 4, 8, 2, 4, 8, 4, 1, 4, 8, 4, 2, 1, 2, 8, 4, 8, 4, 8, 1, 4, 1, 2, 2, 4, 1, 1, 1, 2, 2, 1, 2, 2, 2, 1, 1, 2, 4, 1, 1, 2, 1, 2, 1, 1, 1, 8, 4, 4, 2, 1, 4, 2, 8, 7, 1, 1, 1, 1, 1, 7, 7, 1, 1, 7, 1, 1, 1, 7, 7, 7, 7, 1, 1, 1, 1, 1, 1, 1, 1, 1, 1, 7, 1, 7, 1, 1, 1, 1, 1, 1, 7, 7, 7, 2, 6, 3, 3, 1, 1, 6, 1, 2, 1, 6, 3, 3, 6, 1, 6, 3, 6, 2, 2, 2, 6, 1, 2, 1, 1, 2, 6, 3, 6, 6, 6, 2, 6, 3, 6, 3, 3, 3, 6, 2, 1, 2, 1, 1, 1, 1, 2, 3, 1, 6, 3, 3, 3, 1, 3, 1, 6, 1, 2, 6, 2, 6, 2, 6, 6, 2, 1, 3, 3, 1, 1, 2, 1, 2, 6, 3, 2, 2, 1, 3, 5, 5, 5, 1, 1, 1, 1, 1, 5, 1, 5, 5, 1, 1, 5, 5, 1, 1, 1, 5, 1, 5, 5, 1, 1, 1, 1, 1, 1, 1, 1, 1, 5, 1, 5, 5, 5, 1, 5, 1, 5, 5, 5, 1, 2, 1, 4, 2, 1, 2, 2, 4, 2, 4, 1, 1, 1,

4, 1, 1, 4, 4, 2, 1, 2, 4, 4, 1, 4, 1, 1, 4, 4, 1, 1, 2, 2, 2, 4, 4, 4, 2, 1, 1, 2, 1, 1, 1, 1, 2, 4, 1, 1, 1, 2, 1, 2, 1, 4, 1, 2, 4, 1, 1, 1, 4, 2, 4, 2, 2, 1, 1, 2, 4, 4, 4, 4, 2, 4, 2, 1, 1, 1, 2, 2, 2, 1, 2, 1, 4, 1, 2, 2, 1, 1, 2, 1, 2, 1, 1, 1, 1, 1, 2, 1, 1, 1, 4, 1, 2, 2, 1, 2, 1, 4, 4, 2, 2, 1, 3, 1, 3, 3, 3, 3, 1, 1, 1, 1, 1, 3, 1, 1, 3, 1, 1, 1, 1, 3, 3, 1, 3, 1, 1, 3, 1, 1, 1, 1, 3, 1, 1, 1, 3, 3, 3, 3, 3, 3, 1, 3, 1, 3, 3, 1, 3, 3, 1, 1, 1, 3, 3, 1, 3, 1, 3, 1, 3, 3, 3, 1, 1, 3, 1, 3, 1, 3, 1, 1, 1, 1, 3, 1, 1, 3, 1, 3, 1, 1, 3, 1, 3, 1, 1, 1, 3, 3, 1, 1, 3, 1, 3, 3, 3, 1, 1, 1, 1, 1, 1, 1, 1, 3, 1, 1, 3, 3, 1, 1, 3, 3, 1, 2, 2, 1, 1, 1, 2, 1, 2, 1, 2, 2, 1, 2, 1, 1, 2, 1, 2, 2, 1, 2, 2, 1, 1, 2, 2, 2, 2, 2, 1, 2, 1, 1, 1, 2, 2, 1, 1, 2, 2, 2, 1, 2, 1, 1, 2, 1, 1, 1, 2, 2, 1, 2, 1, 1, 2, 2, 1, 1, 1, 1, 1, 2, 2, 1, 1, 2, 1, 2, 1, 1, 2, 2, 1, 1, 2, 1, 1, 1, 2, 1, 2, 2, 2, 1, 1, 2, 2, 2, 1, 2, 1, 1, 1, 2, 2, 2, 2, 1, 1, 1, 1, 1, 1, 2, 2, 2, 1, 1, 2, 2, 1, 1, 2, 1, 2, 2, 1, 1, 1, 1, 1, 2, 2, 2, 2, 1, 1, 1, 1, 2, 2, 2, 2, 2, 2, 1, 2, 2, 1, 1, 2, 2, 1, 1, 2, 2, 1, 1, 1, 2, 1, 2, 2, 1, 2, 1, 2, 2, 2, 1, 2, 1, 1, 2, 1, 2, 1, 1, 1, 2, 1, 1, 1, 1, 2, 1, 2, 2, 2, 1, 1, 2, 2, 2, 1, 2, 1, 1, 2, 1, 1, 2, 1, 1, 2, 1, 1, 1, 1, 2, 1, 1, 1, 1, 2, 1

Gene ontology terms ranked according to mean of effect sizes in cluster:
immature T cell proliferation in the thymus | cytokine biosynthetic process | regulation of tumor necrosis factor production | cytoskeleton | tumor necrosis factor production | macrophage activation | granuloma formation | immunoglobulin secretion | interleukin-1 receptor antagonist activity | response to tumor necrosis factor | endothelial cell activation | interleukin-1 production | interleukin-1 beta production | interleukin-12 production | specific granule | actin cytoskeleton | tumor necrosis factor receptor activity | bone resorption | T cell proliferation | tumor necrosis factor receptor binding | antibody-dependent cellular cytotoxicity | U-plasminogen activator receptor activity | cell surface | leukotriene biosynthetic process | actin filament polymerization | B cell activation | lymphocyte activation | cell motility | T-helper 1 type immune response | cell adhesion | filamentous actin | interleukin-6 receptor activity | granulocyte macrophage colony-stimulating factor biosynthetic process | immune response | granulocyte macrophage colony-stimulating factor production | inflammatory response | mucosal immune response | monocyte differentiation | negative regulation of tumor necrosis factor production | innate immune response | leukocyte migration | leukocyte activation | chemotaxis | response to lipopolysaccharide | prostanoid biosynthetic process | protein secretion by the type III secretion system | lipoxygenase pathway | proteinaceous extracellular matrix | middle lamella-containing extracellular matrix | extracellular matrix | osteoclast differentiation | interleukin-6 production | interleukin-8 production | cytokine production | monocyte activation | cytokine activity | acute inflammatory response | interleukin-10 production | phagocytosis | interferon-gamma production | mRNA transcription | lipopolysaccharide binding | fibrinogen binding | cell migration | type IV hypersensitivity | interleukin-1 receptor activity | piecemeal microautophagy of nucleus | establishment of cell polarity | interleukin-4 production | complement activation | eosinophil activation | response to host immune response | hypersensitivity | cell fraction | chemokine production | B cell proliferation | cytokine secretion | immunoglobulin production | actin filament binding | interleukin-4 receptor activity | positive regulation of NF-kappaB transcription factor activity | negative regulation of inflammatory response | positive regulation of tumor necrosis factor production | adaptive immune response | neutrophil degranulation | neutrophil apoptosis | localization of cell | antigen processing and presentation | coagulation | fibrinogen complex | respiratory burst | interleukin-2 receptor activity | arachidonic acid metabolic process | mast cell degranulation | MHC class II protein binding | leukocyte adhesion | sensitization | cell chemotaxis | MHC class II biosynthetic process | interleukin-5 production | natural killer cell receptor activity | neutrophil activation | hemostasis | receptor complex | actin binding | adaptive immune response based on somatic recombination of immune receptors built from immunoglobulin superfamily domains | lymphocyte proliferation | actin filament depolymerization | leading edge | cyclooxygenase pathway | basement membrane | collagen catabolic process | interleukin-2 production | positive chemotaxis | fibrinolysis | natural killer cell mediated cytotoxicity | naringenin-chalcone synthase activity | hyaluronic acid binding | membrane attack complex | opsonization | nerve growth factor receptor activity | nerve growth factor receptor binding | cytolysis | IkappaB kinase complex | NF-kappaB binding | neutrophil chemotaxis | mast cell activation | membrane raft | prostaglandin biosynthetic process | pathogenesis | cell maturation | evasion or tolerance of immune response of other organism during symbiotic interaction | tolerance induction | actin filament | luteolysis |

integrin binding | crotonyl-CoA reductase activity | diapedesis | chronic inflammatory response | actomyosin | MHC class I biosynthetic process | MHC class I protein binding | tryptophan catabolic process | platelet activation | monocyte chemotaxis | icosanoid metabolic process | defense response to virus | leukocyte chemotaxis

Corresponding mean of effect sizes of each Gene ontology term:
(In the same order as above)
2.19, 1.82, 1.61, 1.61, 1.58, 1.53, 1.52, 1.52, 1.46, 1.43, 1.41, 1.40, 1.36, 1.36, 1.33, 1.32, 1.31, 1.28, 1.26, 1.25, 1.25, 1.25, 1.23, 1.22, 1.21, 1.18, 1.17, 1.17, 1.16, 1.15, 1.13, 1.12, 1.12, 1.11, 1.11, 1.10, 1.10, 1.10, 1.07, 1.07, 1.07, 1.05, 1.05, 1.04, 1.04, 1.03, 1.02, 1.00, 1.00, 1.00, 0.98, 0.98, 0.97, 0.97, 0.96, 0.94, 0.94, 0.94, 0.93, 0.92, 0.92, 0.92, 0.91, 0.89, 0.89, 0.88, 0.87, 0.87, 0.87, 0.86, 0.86, 0.85, 0.84, 0.84, 0.84, 0.84, 0.83, 0.81, 0.81, 0.80, 0.79, 0.79, 0.79, 0.78, 0.77, 0.76, 0.76, 0.76, 0.75, 0.74, 0.74, 0.73, 0.72, 0.69, 0.69, 0.66, 0.66, 0.65, 0.65, 0.65, 0.63, 0.63, 0.62, 0.62, 0.61, 0.61, 0.61, 0.60, 0.60, 0.57, 0.55, 0.55, 0.54, 0.52, 0.51, 0.51, 0.51, 0.50, 0.47, 0.45, 0.44, 0.44, 0.43, 0.43, 0.41, 0.40, 0.40, 0.39, 0.39, 0.36, 0.36, 0.34, 0.33, 0.32, 0.32, 0.28, 0.28, 0.27, 0.25, 0.25, 0.24, 0.24, 0.15, 0.14, 0.09, 0.00, -0.03, -0.48

Corresponding total number of genes of each Gene ontology term:
(In the same order as above)
42, 108, 86, 612, 209, 178, 77, 51, 70, 185, 106, 31, 21, 150, 62, 605, 213, 192, 188, 214, 109, 130, 1.5K, 50, 300, 116, 232, 478, 59, 937, 439, 104, 109, 530, 107, 425, 52, 102, 61, 246, 145, 108, 371, 226, 51, 69, 43, 805, 805, 805, 144, 165, 144, 455, 117, 42, 68, 166, 304, 106, 130, 61, 84, 754, 131, 128, 200, 152, 123, 148, 71, 37, 234, 112, 155, 88, 243, 155, 62, 87, 550, 40, 61, 148, 52, 117, 38, 315, 184, 235, 195, 189, 88, 90, 178, 158, 254, 94, 181, 57, 77, 147, 110, 423, 142, 131, 158, 56, 195, 42, 289, 71, 182, 87, 96, 33, 50, 37, 134, 67, 100, 112, 179, 107, 146, 86, 91, 191, 105, 1.1K, 224, 141, 109, 177, 69, 81, 52, 47, 22, 118, 195, 197, 41, 220, 52, 24, 110, 41

Corresponding number of genes of each Gene ontology term with at least one TFBS (of selected TFs):
(In the same order as above)
23, 47, 37, 242, 84, 71, 26, 26, 24, 73, 44, 14, 10, 64, 20, 253, 92, 59, 72, 92, 35, 55, 496, 17, 120, 49, 102, 208, 25, 318, 172, 42, 41, 172, 40, 141, 19, 37, 25, 75, 54, 31, 108, 92, 20, 34, 14, 256, 256, 256, 60, 67, 52, 160, 35, 19, 19, 67, 92, 42, 63, 20, 36, 289, 48, 46, 60, 60, 56, 41, 21, 18, 76, 37, 55, 41, 91, 54, 29, 38, 231, 15, 24, 52, 14, 47, 21, 115, 53, 70, 58, 71, 23, 29, 61, 57, 93, 33, 61, 26, 21, 40, 30, 150, 47, 42, 57, 27, 79, 13, 89, 19, 83, 29, 29, 10, 18, 12, 36, 16, 38, 46, 60, 47, 61, 25, 36, 66, 31, 371, 88, 46, 41, 69, 20, 34, 19, 11, 8, 46, 73, 73, 16, 72, 15, 7, 43, 9

Corresponding total number of TFBS (of selected TFs) for each Gene ontology term (in all genes):
(In the same order as above)
137, 247, 197, 1056, 380, 337, 131, 117, 149, 391, 185, 59, 51, 281, 108, 1079, 421, 308, 337, 421, 148, 208, 1840, 105, 584, 216, 472, 863, 105, 1302, 757, 181, 183, 760, 181, 599, 88, 165, 127, 341, 225, 148, 456, 420, 88, 153, 76, 1029, 1029, 1029, 318, 292, 232, 711, 156, 92, 96, 265, 411, 150, 279, 83, 159, 1108, 231, 199, 239, 283, 211, 146, 96, 64, 301, 179, 211, 167, 383, 202, 152, 175, 971, 78, 115, 183, 69, 214, 90, 507, 197, 284, 290, 331, 110, 106, 278, 207, 356, 148, 278, 109, 101, 178, 124, 603, 253, 140, 236, 115, 353, 65, 305, 80, 359, 116, 116, 54, 68, 56, 130, 56, 157, 178, 219, 199, 253, 113, 145, 308, 137, 1409, 353, 193, 160, 277, 72, 118, 77, 64, 36, 179, 313, 313, 74, 269, 56, 25, 166, 27

Corresponding number of selected TFs each Gene ontology term is involved with:
(In the same order as above)
20, 20, 20, 20, 20, 20, 20, 20, 20, 20, 20, 19, 19, 20, 20, 20, 20, 20, 20, 20, 20, 20, 20, 20, 20, 20, 20, 20, 20, 20, 20, 20, 20, 20, 20, 20, 20, 20, 20, 20, 20, 20, 20, 20, 20, 20, 20, 20, 20, 20, 20, 20, 20, 20, 20, 20, 20, 20, 20, 20, 20, 20, 20, 20, 20, 20, 20, 20, 20, 20, 20, 20, 20, 20, 20, 20, 20, 20, 20, 20, 20, 20, 20, 20, 20, 19, 20, 20, 20, 20, 20, 20, 20, 20, 20, 20, 20, 20, 20, 20, 20, 20, 20, 20, 20, 20, 20, 20, 20, 20, 20, 20, 20, 20, 20, 20, 20, 20, 20, 20, 20, 20, 20, 20, 20, 19, 20, 20, 20, 20, 20, 20, 20, 20, 20, 20, 20, 19, 20, 20, 20, 20, 20, 20, 20, 20, 16

TFs ranked according to mean of effect sizes in cluster:

V$PEBP_Q6, V$AP1_C, V$AP1_Q6_01, V$AP1_Q6, V$NFE2_01, V$AP1_Q4_01, V$AP1_Q2_01, V$AP1_01, VMAF_Q6_01, VAML_Q6, V$NRF2_Q4, V$AP1_Q2, V$AP1_Q4, V$DR3_Q4, V$AP1FJ_Q2, V$FOXP3_Q4, VPAX_Q6, VBLIMP1_Q6, V$CACCCBINDINGFACTOR_Q6, V$BACH2_01

Corresponding mean of effect sizes of each TF:
(In the same order as above)
1.35, 1.22, 1.18, 1.07, 1.07, 1.04, 1.03, 1.01, 0.95, 0.89, 0.74, 0.73, 0.73, 0.69, 0.61, 0.60, 0.52, 0.48, 0.35, 0.33

Corresponding total number of TFBS for each TF (genome-wide):
(In the same order as above)
692, 720, 726, 721, 729, 719, 714, 721, 700, 680, 683, 692, 712, 670, 710, 689, 683, 679, 684, 686

Corresponding total number of TFBS for each TF (in all genes in selected Gene ontology terms):
(In the same order as above)
2283, 2426, 2543, 2401, 2320, 2395, 2186, 2283, 2180, 1936, 2012, 2189, 2294, 1771, 2107, 1814, 1933, 1617, 1646, 1844

Corresponding number of genes (of selected Gene ontology terms) each TF is involved with:
(In the same order as above)
276, 311, 325, 307, 278, 319, 300, 299, 297, 271, 290, 283, 293, 229, 286, 256, 278, 264, 260, 272

Corresponding number of selected Gene ontology terms each TF is involved with:
(In the same order as above)
148, 147, 148, 148, 148, 148, 147, 148, 148, 148, 148, 148, 148, 147, 148, 148, 148, 148, 146, 144

Cluster for columns 895 to 922, rows 154 to 174

Gene ontology terms

oligodendrocyte differentiation | asymmetric cell division | leaf development | methylated-DNA-[protein]-cysteine S-methyltransferase activity | viral capsid | Gram-negative-bacterium-type cell wall biogenesis | cell wall biogenesis | cell growth | kinase activity | phosphorylation | glycosaminoglycan biosynthetic process | isoprenoid biosynthetic process | protein-glutamine gamma-glutamyltransferase activity | binding | methionine biosynthetic process | oocyte growth | oocyte growth in germarium-derived egg chamber | phospholipid metabolic process | polysaccharide biosynthetic process | phosphatidylcholine biosynthetic process | phospholipid biosynthetic process | Rho guanyl-nucleotide exchange factor activity | guanyl-nucleotide exchange factor activity | GTPase activator activity | GTP catabolic process | GTPase activity | lipid modification | regulator of G-protein signaling activity

TFs

V$SMAD_Q6 | V$AP2REP_01 | V$HNF4_Q6_03 | V$HNF4_Q6_02 | V$T3R_Q6 | V$PAX8_B | V$PAX8_01 | V$USF2_Q6 | V$ZIC2_01 | V$TTF1_Q6 | V$VDR_Q6 | V$LRF_Q2 | V$LBP1_Q6 | V$AP4_Q6_01 | V$AP4_Q6 | V$AP4_Q5 | V$LFA1_Q6 | V$P53_DECAMER_Q2 | V$TAL1_Q6 | V$HEB_Q6 | V$TBX5_02

Information

All related TFs:
(List of all TFs that are related to any of the PWMs)
AP-2rep, AP-4, DeltaNp63alpha, FBI-1, HNF-4, HNF-4alpha, HNF-4alpha1, HNF-4alpha2, HNF-

4alpha3, HNF-4alpha4, HTF4, HTF4gamma, LBP-1, LF-A1, LRF, Nkx2-1, OCZF, Pax-8, RAR-alpha, RAR-alpha1, RAR-alpha:RXR-alpha, RAR-alpha:RXR-gamma, RAR-beta, RAR-beta2, RAR-gamma, RXR-alpha, RXR-beta, RXR-beta2, RXR-gamma, Smad1, Smad1.1, Smad2, Smad2-L, Smad3, Smad3:Smad4, Smad4, T3R-alpha, T3R-alpha1, T3R-alpha2, T3R-beta, T3R-beta1, T3R-beta2, TBX5, Tal-1, Tal-1alpha, USF1:USF2, USF2, USF2a, VDR, ZIC2, Zic2, p53, p53-isoform-1, p63alpha, p63gamma, p73alpha, p73beta

Ranked gene list:
(All genes of the selected Gene ontology terms with hits of any of the selected TFs, ranked according to the total number of TFBS)
HRAS, SLC2A4RG, AKT1, ARHGEF2, SSSCA1, CNTNAP1, TNK2, CTSD, CDK5, TUSC2, SLC25A1, RGS19, PTPN6, GIT1, ISYNA1, RALGDS, PLCG1, NOTCH1, ADRBK1, UBTF, RARA, MDK, FASN, CFL1, GRK6, UBE2I, CHKB, TNF, SH3BP1, SCRIB, PER1, JUNB, GALE, DGAT1, CSK, CISH, TRADD, RIN1, MAP3K11, ARHGEF1, CDKN1C, E2F4, SOCS3, RGS14, PISD, SEMA3B, RORC, RASGRP2, PNPLA2, PLXNA1, PDGFB, NR4A1, MKNK2, MEF2D, GFER, SQSTM1, RPS6KB2, PTBP1, PRKCD, PARD6A, MRPL28, MGC111011, LTB, KIAA0664, ITGB2, GPT, CLCF1, CCND2, ACTB, TNFRSF12A, IRF1, DVL1, SOCS1, EGR1, PPP1R9B, MAPK12, LCK, GUK1, SMAD7, PTP4A3, CORO1A, CD248, VEGFA, RPS6KA1, RAB1B, PKN1, FOXO3, CAMK2G, BCL2L11, ARHGDIA, OSM, EFNA1, CDK4, TSC2, SH3BP2, SH2B3, RHOC, NPDC1, NOXO1, MAP3K12, MAP2K3, LBX1, GAPDH, FLJ32987, FLII, DUSP2, BCL9L, ADAM8, TACC3, SHMT2, S100A4, PLK3, PFN1, MXI1, DNMT3A, DLG4, AKT1S1, ZAP70, GLI1, TYMP, RASSF1, RARG, OGFR, NFKBIE, NFATC1, MYO1C, SH2B2, PTK6, GNAS, DPAGT1, TGFB1, NPPC, BRD2, TMEM115, PSD4, MAPK11, JAG2, SLC9A3R1, NFIC, NECAB3, MPG, MMP11, MAP3K14, MAFA, TPP1, SYNGAP1, POLR2A, MAP2K7, IRS1, HMGA1, EMD, CD82, ARF1, ADM, p63RhoGEF, PLCD1, HSD17B8, EFNA2, DDR1, CYBA, COL11A2, NUTF2, MXD3, TPI1, RAB24, PSD, CDKN2C, PURA, PNKP, NFKB2, MLL2, INPP5D, DGKA, DDIT3, DCTN3, CPT1A, CDK9, CDC34, CCND1, CALR, C21orf33, AGRN, U2AF2, PIGW, FGFR3, BHLHB2, APEH, ZFPM1, ZFP36, ZBTB7B, VEGFB, UCN2, TNFRSF4, TMC6, THRA, TCF7, SLC25A22, SKI, SGMS1, SEPT9, RAC3, PTPN7, PTMA, PLEC1, OPRL1, NOC2L, NELF, MNT, MAZ, MAPKAPK3, MAF1, LSP1, KAT2A, JUND, ITGA5, INS, ID3, HES5, HDAC10, GNAI2, FUT7, FLNA, FGF8, DGKZ, CRIP2, CRIP1, CKB, CIRBP, CIC, CDC20, CD151, CBX4, C16orf53, ANAPC2, AHNAK, ADORA2A, ABHD14B, ZMYND10, STAT5A, S100A6, RPS15, RASSF2, PDE4A, PCP2, MAFG, LDB1, HOXA7, HIC1, HDAC7, GFI1, FBRS, E2F2, DKFZp779C0757, CDC25B, RAB34, PRDX2, MYL5, MXD4, MGAT1, LTBP4, KCNH2, HOXA9, GATA2, CYB561D2, CACNB3, ACY1, ACTG1, ZYX, WBP7, TLR9, SYTL1, RPS2, PPP1R1B, PGP, NR4A2, MC1R, LEF1, ENTPD2, DLL1, C19orf26, AXIN1, ARHGAP4, TFEB, SLC12A7, SF3A2, RXRB, PLEKHG2, MCAM, IL17C, GABARAP, CTBP1, BBC3, TNFSF13, SLC39A7, SH2D3C, POLD4, PIK3R2, LTA, LASP1, HOXA10, GPR132, GPI, FHL3, FGF9, FBXL15, DBNL, CDK6, SH3BGRL3, RUNX3, MST1, GYLTL1B, GBF1, FOSB, DUSP1, CTDSP1, CAMKK1, BAK1, REPIN1, RAP1GAP, RAD9A, PFKL, PAX6, NR3C2, NKX2-2, MAFK, GALT, DDX41, CRTC2, CRLF1, ARRB2, CDT1, BRMS1, B3GALT6, ARFGAP2, VASP, VAMP2, SOX8, SHC1, RASA3, PYGM, POU2F2, PDLIM7, PCNA, NOL3, MAPK15, LPAR2, IRF7, ILK, IFITM1, ID2, HES1, GPSM1, GALK1, EPB49, CTRL, CDKN1B, CDK5R1, ACHE, ACAA1, RING1, PSENEN, NPFF, HNRNPL, GRN, CLCN2, VIPR1, UBE2M, TNFRSF1B, SLC9A3R2, SAMD4A, RPS6KA4, RHOG, PNPLA6, PIN1, NTRK1, NDOR1, NAT6, MAPK13, LAT, KLF13, IKBKE, GSK3A, GARNL4, CDKN1A, ARAP1, TRAF4, TNFRSF25, SIN3A, PTGER1, MAL, GPC1, ESRRA, DUSP6, ARFGAP1, ACAP1, ZC3H12D, TSHZ1, TNFRSF1A, SMAD2, RGS11, RGS10, RANBP1, PRKCB, PHF1, PHB2, NGFR, MKNK1, KCNH4, ITPR3, ING1, GALNS, FAM62A, DLX2, CTF1, CD7, CD4, VAV1, STOML2, SLBP, RAB3A, PTGDS, OPRS1, LPIN1, HMX2, GRIN2D, CUX1, CDC25C, BAD, TRAF2, TNFRSF14, TM7SF2, SOS1, SLC2A1, SLC29A1, S100A3, RRAS, PTH1R, PSME2, PNMT, NTHL1, NSD1, NR3C1, NPM1, NDRG1, MYL6, MYD88, MCM2, MARCKSL1, MAP2K2, LIF, LGALS1, LEPREL2, KLF6, KHK, KCNA2, IL11, IDUA, GYPC, GRM2, GRB7, FERMT3, EPHB3, ELOVL1, EIF6, EEF2, CYC1, CXCR4, CTCF, CNP, CDC37, CD81, CACNA1G, ATF4, ANXA9, ALDOA, TLX2, TGM1, SLC12A4, SCARF2, RGS16, NLGN2, LMAN2, ISG15, IGF2, HCST, EPOR, DDX5, CSNK2B, CCNL2, ADRM1, ZFP36L1, WBP2, VEZF1, TYROBP, TRIM28, TNFRSF13C, STMN1, STK11, SKP1, ROBLD3, RNF4, PTCH1, PSTPIP1, PRMT1, PRKD2, PPARA, PLA2G6, PKM2, NCBP2, NADK, MIF4GD, MBD1, MAP4K2, MAD1L1, IL2RB, IKZF1, IGF1R, IDI1, HOXB4, HK1, HGS, GRIN2C,

GPR3, GLTSCR2, GADD45G, FXYD1, ETS1, EMG1, ELMO3, EIF5A, E2F1, DOHH, DDIT4, CSF1, CLEC11A, CERK, CDKN2D, CD79B, CD6, CD44, CCR7, CCND3, CASP9, BRSK2, BCL2L1, BACH2, ANXA6, ANP32A, ADAM15, ACVR1, TFDP1, TCF3, TAF8, STAT6, SREBF2, SREBF1, SPHK1, SEMA4D, RGL2, PTPRA, PSME1, PRAP1, PPP1R7, PMEPA1, PFKFB3, PEA15, PDK2, PAX5, PAOX, NOS3, NFE2L1, MLL5, MARK2, JARID2, ITGB7, ICAM4, HRH3, HDAC4, GIT2, GADD45B, FURIN, FSTL3, FRS3, FBXW7, ERF, DOK2, DNM2, DKFZp434P0672, DDB1, CTDSP2, CGB, CENPB, CD79A, CD63, CCDC88B, BLVRB, BID, ARHGAP9, ZBTB16, WDR1, VDAC1, VAMP8, USF2, USF1, ULK1, TPM1, TOB2, TFAP2A, TCEB2, STARD3, SORBS3, SNCG, SH2D2A, SCN5A, RRM1, RNH1, RAD23A, PPP3CA, PHF12, PFDN5, PER2, PDP2, PDCD6, NRGN, NR5A1, NR2F1, NISCH, NFATC2, NAGK, NACC1, MUC1, MT2A, MIF, MIB2, MBD3, MAP4K1, MADCAM1, LTB4R2, LMNA, KLF10, ITPKA, IL32, IL10RA, HGFAC, HDAC5, HBA2, GRIN1, GRASP, GMEB2, GEMIN4, FOSL2, FARSA, EPB41, EGR4, EFEMP2, EDC4, ECE1, DYRK1B, DLX4, DIABLO, DAK, CTSA, CSRP1, CPEB1, COASY, CEBPE, BRD4, ARNTL, APBA2, AP2M1, AMD1, AHRR, ADRA2B, TSPO, TRRAP, TMSB10, TGFB3, TEAD3, TAPBP, TAP1, SURF2, SNF1LK, SLC38A3, SF3B2, S100A13, RPS19, PVRL1, PTRH1, PPP4C, PPP1R16A, PPIF, POU3F3, PIAS4, PCOLCE, PCID2, PBX2, P2RX7, OAZ1, NUP62, NR2F6, NR1H2, NQO1, NOTCH4, NCOA4, NCAN, MZF1, MYBPC3, MTF1, MSL2, MGMT, MGAT3, MFI2, MCRS1, MCAT, MARK4, MAP1A, LOXL1, KLF3, ITPKB, ITPK1, ITGA7, ITGA6, ISCU, IRF5, IPO13, INSRR, HDGF, GZMM, GATAD2A, FZR1, FYB, F7, EIF4ENIF1, EGR3, EGR2, DYRK2, DPYSL2, DHRS11, CYB5A, CST6, CRABP2, CNTFR, CHRNB1, CAMKK2, C6orf25, C1orf38, BRMS1L, BRF1, BRD1, BCLAF1, B4GALT7, B3GAT3, ATRIP, ARRB1, AP2B1, ANXA11, AGTRAP, ACSS2, ACSL5, ABCG1

Corresponding total number of TFBS:
(For each gene listed above, the total number of TFBS for any of the selected TFs, multiplied by the number of selected Gene ontology terms containing that gene)
252, 240, 231, 228, 168, 162, 147, 133, 133, 126, 126, 126, 126, 126, 120, 114, 114, 114, 114, 105, 105, 105, 105, 105, 95, 85, 85, 84, 84, 84, 84, 84, 84, 84, 84, 84, 80, 80, 80, 78, 70, 68, 64, 64, 64, 63, 63, 63, 63, 63, 63, 63, 63, 63, 63, 60, 60, 60, 60, 60, 60, 60, 60, 60, 60, 60, 60, 60, 60, 57, 57, 57, 56, 56, 54, 54, 54, 54, 51, 51, 50, 49, 48, 48, 48, 48, 48, 48, 48, 48, 45, 45, 44, 42, 42, 42, 42, 42, 42, 42, 42, 42, 42, 42, 42, 42, 42, 42, 40, 40, 40, 40, 40, 40, 40, 40, 40, 39, 39, 38, 38, 38, 38, 38, 38, 38, 36, 36, 36, 36, 35, 35, 35, 34, 34, 34, 34, 32, 32, 32, 32, 32, 32, 32, 30, 30, 30, 30, 30, 30, 30, 30, 30, 30, 28, 28, 28, 28, 28, 28, 28, 27, 27, 26, 26, 26, 25, 24, 24, 24, 24, 24, 24, 24, 24, 24, 24, 24, 24, 24, 24, 24, 22, 22, 22, 22, 22, 21, 21, 21, 21, 21, 21, 21, 21, 21, 21, 21, 21, 21, 21, 21, 21, 21, 21, 21, 21, 21, 21, 21, 21, 21, 21, 21, 21, 21, 21, 21, 21, 21, 21, 21, 21, 21, 21, 21, 21, 21, 21, 21, 21, 21, 21, 21, 21, 21, 21, 20, 20, 20, 20, 20, 20, 20, 20, 20, 20, 20, 20, 20, 20, 20, 20, 20, 19, 19, 19, 19, 19, 19, 19, 19, 19, 19, 19, 19, 19, 18, 18, 18, 18, 18, 18, 18, 18, 18, 18, 18, 18, 18, 18, 18, 17, 17, 17, 17, 17, 17, 17, 17, 17, 17, 16, 16, 16, 16, 16, 16, 16, 16, 16, 16, 16, 16, 16, 16, 16, 15, 15, 15, 15, 15, 15, 15, 15, 15, 15, 14, 14, 14, 14, 14, 14, 14, 14, 14, 14, 14, 14, 14, 13, 13, 13, 13, 12, 12, 12, 12, 12, 12, 12, 12, 12, 12, 12, 12, 12, 12, 12, 12, 12, 12, 12, 12, 12, 12, 12, 12, 12, 11, 11, 11, 11, 11, 11, 10, 10, 10, 10, 10, 10, 10, 10, 10, 10, 10, 10, 10, 10, 10, 10, 10, 10, 10, 10, 9, 9, 9, 9, 9, 9, 9, 9, 9, 9, 8, 8, 8, 8, 8, 8, 8, 8, 8, 8, 8, 8, 8, 8, 8, 8, 8, 8, 8, 8, 8, 7, 7, 7, 7, 7, 7, 7, 7, 7, 7, 7, 7, 6, 6, 6, 6, 6, 6, 6, 6, 6, 6, 6, 6, 6, 6, 6, 6, 6, 6, 6, 6, 6, 6, 6, 6, 6, 6, 6, 6, 6, 6, 6, 6, 6, 6, 6, 6, 6, 6, 6, 6, 6, 6, 6, 6, 6, 6, 6, 5, 5, 5, 5, 5, 5, 5, 5, 5, 5, 5, 5, 5, 5, 5, 4, 4, 4, 4, 4, 4, 4, 4, 4, 4, 4, 4, 4, 4, 4, 4, 4, 4, 4, 4, 4, 4, 4, 4, 4, 4, 4, 4, 4, 4, 4, 4, 4, 4, 4, 4, 4, 4, 4, 4, 4, 4, 4, 4, 4, 4, 4, 4, 4, 4, 4, 4, 4, 4, 4, 4, 4, 4, 4, 4, 3, 3, 3, 3, 3, 3, 3, 3, 3, 3, 3, 3, 3, 3, 3, 3, 3, 3, 3, 3, 3, 3, 3, 3, 3, 3, 3, 3, 3, 3, 3, 3, 3, 3, 3, 3, 3, 3, 3, 3, 3, 3, 3, 3, 3, 3, 3, 3, 2, 2, 2, 2, 2, 2, 2, 2, 2, 2, 2, 2, 2, 2, 2, 2, 2, 2, 2, 2, 2, 2, 2, 2, 2, 2, 2, 2, 2, 2, 2, 2, 2, 2, 2, 2, 2, 2, 2, 2, 2, 2, 2, 2, 2, 2, 2, 2, 2, 2, 2, 2, 2, 2, 2, 2, 2, 2, 2, 2, 2, 2, 2, 2, 2, 2, 2, 2, 2, 2, 2, 2, 2, 2, 2, 1, 1, 1, 1, 1, 1, 1, 1, 1, 1, 1, 1, 1, 1, 1, 1, 1, 1, 1, 1, 1, 1, 1, 1, 1, 1, 1, 1, 1, 1, 1, 1, 1, 1, 1, 1, 1, 1, 1, 1, 1, 1, 1, 1, 1, 1, 1, 1, 1, 1, 1, 1, 1, 1, 1, 1, 1, 1, 1, 1, 1, 1, 1, 1, 1, 1, 1, 1, 1, 1, 1, 1, 1, 1, 1, 1, 1, 1, 1, 1, 1, 1, 1, 1, 1, 1, 1

Corresponding number of selected Gene ontology terms each gene is involved with:
(In the same order as above)
12, 12, 11, 12, 8, 9, 7, 7, 7, 6, 9, 6, 6, 6, 6, 6, 6, 6, 6, 5, 5, 5, 5, 5, 5, 5, 5, 4, 4, 4, 4, 4, 4, 4, 4, 4, 4, 4, 4, 6, 5, 4, 4, 4, 4, 3, 3, 3, 3, 3, 3, 3, 3, 3, 3, 4, 3, 5, 3, 5, 5, 3, 3, 3, 5, 3, 3, 4, 3, 3, 3, 3, 4, 4, 3, 3, 6, 3, 3, 3, 5, 7, 4, 4, 3, 6, 6, 3, 4, 6, 3, 3, 4, 7, 2, 2, 3, 2, 2, 2, 2, 3, 3, 2, 2, 2, 2, 2, 2, 2, 2, 2, 2, 5, 2, 5, 4, 3, 3, 2, 2, 2, 2, 2, 2, 2, 4, 3, 2, 3, 5, 5, 7, 2, 2, 2, 2, 2, 2, 2, 2, 2, 4, 2, 5, 2, 2, 3, 5, 2, 2, 3, 6, 2, 2, 2, 2, 2, 4, 2, 4, 3, 3, 2, 2, 2, 5, 2, 2, 2, 2, 3, 2, 4, 3, 3, 3, 2, 4, 3, 2, 3, 2, 2, 2, 2, 2, 1, 1, 1, 1, 3, 1, 1, 1, 1, 1, 1, 3, 1, 1, 1, 1, 1, 1, 1, 1,

1, 1, 1, 1, 3, 3, 3, 7, 3, 1, 1, 1, 1, 1, 1, 1, 1, 1, 1, 1, 1, 3, 1, 1, 1, 1, 1, 1, 1, 1, 4, 1, 1, 1, 2, 1, 1, 1, 1, 1, 1, 1, 1, 4, 1, 4, 1, 1, 1, 1, 1, 1, 1, 1, 1, 1, 1, 1, 1, 2, 1, 1, 1, 1, 1, 1, 2, 1, 2, 2, 1, 1, 6, 9, 1, 1, 1, 1, 1, 1, 1, 1, 1, 1, 8, 1, 1, 2, 8, 1, 2, 1, 2, 4, 2, 2, 2, 2, 4, 1, 3, 5, 1, 3, 3, 3, 1, 1, 3, 1, 7, 2, 1, 1, 2, 1, 1, 1, 1, 2, 2, 2, 1, 1, 1, 1, 3, 1, 1, 6, 1, 4, 1, 4, 4, 4, 3, 3, 4, 6, 2, 3, 4, 2, 2, 1, 1, 6, 4, 1, 6, 1, 1, 1, 1, 1, 1, 2, 1, 2, 2, 5, 1, 5, 2, 5, 5, 2, 1, 2, 2, 1, 1, 2, 1, 10, 1, 1, 1, 1, 3, 1, 3, 1, 3, 3, 1, 4, 1, 4, 4, 4, 4, 4, 4, 2, 2, 4, 4, 4, 2, 4, 2, 1, 1, 2, 2, 4, 7, 1, 1, 7, 1, 1, 1, 1, 1, 7, 7, 1, 3, 1, 1, 6, 6, 2, 1, 3, 2, 1, 1, 1, 1, 3, 6, 2, 1, 3, 3, 3, 6, 3, 3, 2, 2, 1, 3, 2, 2, 2, 2, 3, 1, 1, 1, 3, 6, 2, 3, 2, 3, 3, 2, 1, 2, 1, 1, 1, 5, 1, 1, 5, 1, 1, 1, 5, 1, 5, 5, 5, 1, 1, 4, 1, 1, 2, 1, 1, 4, 4, 2, 4, 1, 1, 1, 2, 1, 4, 4, 2, 2, 2, 1, 1, 2, 4, 4, 2, 4, 1, 1, 2, 1, 1, 2, 4, 2, 2, 4, 1, 1, 1, 4, 1, 2, 4, 4, 2, 4, 1, 2, 4, 1, 4, 4, 1, 4, 1, 4, 1, 2, 4, 3, 3, 3, 3, 3, 3, 3, 3, 1, 3, 1, 3, 3, 1, 1, 3, 3, 1, 1, 3, 1, 1, 3, 1, 1, 1, 1, 3, 3, 3, 3, 1, 3, 3, 1, 1, 3, 1, 3, 1, 1, 1, 1, 1, 1, 1, 1, 1, 2, 2, 2, 1, 1, 1, 2, 2, 1, 2, 1, 1, 2, 2, 2, 2, 2, 1, 1, 2, 1, 1, 1, 1, 1, 2, 2, 1, 1, 2, 2, 1, 1, 2, 2, 2, 1, 2, 1, 1, 1, 2, 1, 2, 2, 1, 2, 1, 2, 2, 1, 2, 2, 1, 2, 2, 1, 1, 1, 2, 1, 2, 2, 2, 1, 2, 1, 1, 2, 1, 2, 1, 1, 1, 1, 1, 1, 1, 1, 1, 1, 1, 1, 1, 1, 1, 1, 1, 1, 1, 1, 1, 1, 1, 1, 1, 1, 1, 1, 1, 1, 1, 1, 1, 1, 1, 1, 1, 1, 1, 1, 1, 1, 1, 1, 1, 1, 1, 1, 1, 1, 1, 1, 1, 1, 1, 1, 1, 1, 1, 1, 1, 1, 1, 1, 1, 1, 1, 1, 1, 1, 1, 1, 1, 1, 1, 1, 1, 1, 1, 1, 1, 1, 1, 1, 1, 1, 1, 1, 1, 1, 1, 1, 1, 1

Corresponding number of selected TFs each gene is involved with:
(In the same order as above)
21, 20, 21, 19, 21, 18, 21, 19, 19, 21, 14, 21, 21, 21, 20, 19, 19, 19, 19, 21, 21, 21, 21, 21, 19, 17, 17, 21, 21, 21, 21, 21, 21, 21, 21, 21, 20, 20, 20, 13, 14, 17, 16, 16, 16, 21, 21, 21, 21, 21, 21, 21, 21, 21, 21, 15, 20, 12, 20, 12, 12, 20, 20, 20, 12, 20, 20, 15, 20, 19, 19, 19, 14, 14, 18, 18, 9, 18, 17, 17, 10, 7, 12, 12, 16, 8, 8, 16, 12, 8, 15, 15, 11, 6, 21, 21, 14, 21, 21, 21, 21, 14, 14, 21, 21, 21, 21, 21, 20, 20, 20, 20, 20, 8, 20, 8, 10, 13, 13, 19, 19, 19, 19, 19, 19, 19, 9, 12, 18, 12, 7, 7, 5, 17, 17, 17, 17, 16, 16, 16, 16, 16, 8, 16, 6, 15, 15, 10, 6, 15, 15, 10, 5, 15, 14, 14, 14, 14, 7, 14, 7, 9, 9, 13, 13, 13, 5, 12, 12, 12, 12, 8, 12, 6, 8, 8, 8, 12, 6, 8, 12, 8, 11, 11, 11, 11, 11, 21, 21, 21, 21, 7, 21, 21, 21, 21, 21, 21, 7, 21, 21, 21, 21, 21, 21, 21, 21, 21, 21, 21, 21, 21, 7, 7, 7, 3, 7, 21, 21, 21, 21, 21, 21, 21, 21, 21, 21, 21, 21, 7, 21, 21, 21, 21, 21, 21, 21, 20, 5, 20, 20, 20, 10, 20, 20, 20, 20, 20, 20, 20, 20, 5, 20, 5, 19, 19, 19, 19, 19, 19, 19, 19, 19, 19, 19, 19, 19, 9, 18, 18, 18, 18, 18, 18, 9, 18, 9, 9, 18, 18, 3, 2, 17, 17, 17, 17, 17, 17, 17, 17, 17, 17, 2, 16, 16, 8, 2, 16, 8, 16, 8, 4, 8, 8, 8, 8, 4, 15, 5, 3, 15, 5, 5, 5, 15, 15, 5, 14, 2, 7, 14, 14, 7, 14, 14, 14, 14, 7, 7, 7, 13, 13, 13, 13, 4, 12, 12, 2, 12, 3, 12, 3, 3, 3, 4, 4, 3, 2, 6, 4, 3, 6, 6, 12, 12, 2, 3, 12, 2, 11, 11, 11, 11, 11, 11, 5, 10, 5, 5, 2, 10, 2, 5, 2, 2, 5, 10, 5, 5, 10, 10, 5, 10, 1, 10, 9, 9, 9, 3, 9, 3, 9, 3, 3, 9, 2, 8, 2, 2, 2, 2, 2, 2, 4, 4, 2, 2, 2, 4, 2, 4, 8, 8, 4, 4, 2, 1, 7, 7, 1, 7, 7, 7, 7, 7, 1, 1, 7, 2, 6, 6, 1, 1, 3, 6, 2, 3, 6, 6, 6, 6, 2, 1, 3, 6, 2, 2, 2, 1, 2, 2, 3, 3, 6, 2, 3, 3, 3, 3, 2, 6, 6, 6, 2, 1, 3, 2, 3, 2, 2, 3, 6, 3, 6, 6, 5, 1, 5, 5, 1, 5, 5, 5, 1, 5, 1, 1, 1, 5, 5, 1, 4, 4, 2, 4, 4, 1, 1, 2, 1, 4, 4, 4, 2, 4, 1, 1, 2, 2, 2, 4, 4, 2, 1, 1, 2, 1, 4, 4, 2, 4, 4, 2, 1, 2, 2, 1, 4, 4, 4, 1, 4, 2, 1, 1, 2, 1, 4, 2, 1, 4, 1, 1, 4, 1, 4, 1, 4, 2, 1, 1, 1, 1, 1, 1, 1, 1, 1, 3, 1, 3, 1, 1, 3, 3, 1, 1, 3, 3, 1, 3, 3, 1, 3, 3, 3, 3, 1, 1, 1, 1, 3, 1, 1, 3, 3, 1, 3, 1, 3, 3, 3, 3, 3, 3, 3, 3, 1, 1, 1, 2, 2, 2, 1, 1, 2, 1, 2, 2, 1, 1, 1, 1, 1, 2, 2, 1, 2, 2, 2, 2, 2, 1, 1, 2, 2, 1, 1, 2, 2, 1, 1, 1, 2, 1, 2, 2, 2, 1, 2, 1, 1, 2, 1, 2, 1, 1, 2, 1, 1, 2, 1, 1, 2, 2, 2, 1, 2, 1, 1, 1, 2, 1, 2, 2, 1, 2, 1, 2, 2, 2, 2, 1, 1, 1, 1, 1, 1, 1, 1, 1, 1, 1, 1, 1, 1, 1, 1, 1, 1, 1, 1, 1, 1, 1, 1, 1, 1, 1, 1, 1, 1, 1, 1, 1, 1, 1, 1, 1, 1, 1, 1, 1, 1, 1, 1, 1, 1, 1, 1, 1, 1, 1, 1, 1, 1, 1, 1, 1, 1, 1, 1, 1, 1, 1, 1, 1, 1, 1, 1, 1, 1, 1, 1, 1, 1, 1, 1, 1, 1, 1, 1, 1, 1, 1, 1, 1, 1, 1, 1, 1, 1

Gene ontology terms ranked according to mean of effect sizes in cluster:
leaf development | asymmetric cell division | regulator of G-protein signaling activity | phosphatidylcholine biosynthetic process | oligodendrocyte differentiation | cell wall biogenesis | polysaccharide biosynthetic process | lipid modification | GTP catabolic process | GTPase activity | cell growth | binding | GTPase activator activity | Gram-negative-bacterium-type cell wall biogenesis | oocyte growth in germarium-derived egg chamber | oocyte growth | phospholipid biosynthetic process | kinase activity | viral capsid | guanyl-nucleotide exchange factor activity | methylated-DNA-[protein]-cysteine S-methyltransferase activity | methionine biosynthetic process | glycosaminoglycan biosynthetic process | isoprenoid biosynthetic process | phospholipid metabolic process | protein-glutamine gamma-glutamyltransferase activity | phosphorylation | Rho guanyl-nucleotide exchange factor activity

Corresponding mean of effect sizes of each Gene ontology term:
(In the same order as above)
1.87, 1.84, 1.37, 1.36, 1.32, 1.23, 1.21, 1.20, 1.19, 1.11, 1.04, 1.02, 1.01, 0.95, 0.92, 0.92, 0.90, 0.83, 0.77, 0.75, 0.74, 0.66, 0.61, 0.47, 0.45, 0.44, 0.42, 0.41

Corresponding total number of genes of each Gene ontology term:
(In the same order as above)
26, 46, 102, 45, 75, 72, 41, 31, 178, 240, 1.3K, 3.2K, 146, 62, 61, 61, 88, 860, 60, 195, 174, 23, 37, 35, 44, 39, 2.4K, 365

Corresponding number of genes of each Gene ontology term with at least one TFBS (of selected TFs):
(In the same order as above)
8, 12, 20, 11, 17, 13, 7, 5, 37, 47, 254, 502, 34, 11, 11, 11, 20, 175, 13, 38, 31, 3, 9, 4, 5, 7, 411, 62

Corresponding total number of TFBS (of selected TFs) for each Gene ontology term (in all genes):
(In the same order as above)
101, 174, 227, 116, 208, 173, 93, 87, 316, 450, 2131, 4073, 326, 134, 111, 111, 165, 1437, 147, 387, 305, 43, 53, 60, 58, 61, 3306, 582

Corresponding number of selected TFs each Gene ontology term is involved with:
(In the same order as above)
21, 21, 21, 21, 21, 21, 21, 21, 21, 21, 21, 21, 21, 21, 21, 21, 21, 21, 21, 21, 21, 21, 21, 21, 21, 21, 21, 21

TFs ranked according to mean of effect sizes in cluster:
V$LFA1_Q6, V$AP4_Q6_01, V$HNF4_Q6_02, V$LBP1_Q6, VHEB_Q6, VAP4_Q6, V$SMAD_Q6, V$TTF1_Q6, V$TAL1_Q6, V$HNF4_Q6_03, V$AP4_Q5, V$VDR_Q6, V$AP2REP_01, V$PAX8_01, V$USF2_Q6, V$TBX5_02, V$ZIC2_01, V$T3R_Q6, V$PAX8_B, V$LRF_Q2, V$P53_DECAMER_Q2

Corresponding mean of effect sizes of each TF:
(In the same order as above)
1.27, 1.17, 1.16, 1.14, 1.13, 1.11, 1.10, 1.05, 1.05, 1.03, 1.00, 1.00, 0.95, 0.89, 0.88, 0.85, 0.82, 0.82, 0.81, 0.64, 0.38

Corresponding total number of TFBS for each TF (genome-wide):
(In the same order as above)
711, 699, 699, 691, 681, 692, 729, 714, 686, 701, 702, 692, 700, 728, 703, 704, 716, 698, 712, 719, 709

Corresponding total number of TFBS for each TF (in all genes in selected Gene ontology terms):
(In the same order as above)
723, 729, 768, 735, 713, 699, 753, 717, 730, 735, 698, 710, 769, 782, 755, 722, 751, 720, 734, 727, 765

Corresponding number of genes (of selected Gene ontology terms) each TF is involved with:
(In the same order as above)
304, 303, 315, 301, 293, 294, 307, 305, 299, 306, 294, 302, 313, 314, 305, 291, 301, 297, 307, 296, 320

Corresponding number of selected Gene ontology terms each TF is involved with:
(In the same order as above)
28, 28, 28, 28, 28, 28, 28, 28, 28, 28, 28, 28, 28, 28, 28, 28, 28, 28, 28, 28, 28

Cluster for columns 1 to 6, rows 176 to 186

Gene ontology terms

peptide transport | Hedgehog signaling complex | hemopoiesis | cell differentiation | T cell differentiation | cell development

TFs

V$MYB_Q5_01 | P$GBP_Q6 | V$MYB_Q3 | V$TFIIA_Q6 | V$RFX_Q6 | V$TEF1_Q6 | V$STAT6_02 | V$STAT3_02 | V$NCX_01 | V$PXRRXR_02 | V$PXRRXR_01

Information

All related TFs:
(List of all TFs that are related to any of the PWMs)
B-Myb, NCX, Ncx, RFX1, RFX1:RFX2, RFX1:RFX3, RFX2, RFX3, RFX4, RFX5, RFX5:RFXAP:RFXANK, RFXANK, RFXAP, STAT3, STAT6, TEF-1, TFIIA, TFIIA-alpha/beta, TFIIA-gamma, c-Myb, c-Myb-isoform1

Ranked gene list:
(All genes of the selected Gene ontology terms with hits of any of the selected TFs, ranked according to the total number of TFBS)
GFI1, CXCR4, CDKN1B, FASN, CD4, SSSCA1, SH2B3, RUNX3, TNF, LBX1, LEF1, GATA2, NOTCH1, TCF7, PTPN6, NFATC1, IRF1, HOXA9, GATA3, FLI1, CISH, CCND2, AKT1, ZFPM1, SHC1, HOXA10, FLII, CCR7, LYL1, ITGA5, EGR1, SOCS1, HOXB4, RARA, PAX6, OSM, NR4A2, DLL1, CSK, CD68, BCL6, BCL11B, ATXN1, ID2, SMAD7, NKX2-2, LCK, FLJ32987, BCL2L1, SOCS3, GLI1, DLL4, VEGFA, TNFRSF4, MLL5, HES5, DNMT3A, STAT5A, RUNX1, ETS1, POU2F2, LMO4, ITGB2, IKZF1, ID3, HOXB3, HES1, ZBTB7B, WNT10B, TCIRG1, STK16, RORC, RARG, PTMA, PSMB8, PLEKHG2, NR4A1, MAP3K12, LTB, LFNG, LDB1, JUNB, HOXC9, HOXA7, HOXA11, CRIP2, CRIP1, CFL1, CDK5, CDH23, ACTB, ABCB9, TNFSF13, TNFRSF18, STRA13, RPS6KA1, RING1, NFATC3, MLL, EOMES, CDK4, BMI1, BHLHB2, ARHGEF1, TGFB1, TAPBP, SMAD3, RPS6KB2, PSMB10, PAX5, MDK, LTA, IRS1, HOXB6, BCL2L11, ZAP70, NR3C2, LAG3, JUND, IL11RA, E2F4, CDKN2C, TAP1, PITX3, MAL, HSP90AB1, HOXC6, HOXC5, HOXC4, BAK1, TLR9, SATB1, NOTCH4, NGFR, JUN, ING1, HRAS, GGNBP2, FGF9, FAM65B, CD79A, CD3G, CCND3, TNFRSF6B, FOSL2, CXXC1, CD44, CALR, UNK, SOCS2, PSMB9, PCGF2, PARD6A, NFIA, MZF1, MCAM, LMNA, FOXJ1, ENO2, EBF1, DUSP6, DAXX, CDK6, C2orf28, BCL11A, ASB2, ZNF385A, VIM, TBX21, TAP2, SETD1A, PSMC5, PBX2, NDRG1, INPP5D, HAND2, CSF1, CD3D, C21orf33, VDR, TSPAN32, SP1, PRDM1, POU4F1, PIM1, ONECUT1, NEUROG2, MYD88, MAFG, LEPREL2, KLF2, IRF4, IL23A, IL17C, IGF1R, HIST1H4I, HBA2, GADD45G, FYN, CORO1A, CD81, CCND1, BACH2, ANXA6, AMH, ALCAM, AKTIP, UBE2I, TRIM28, TRAF3, TFAP2A, TAF8, SOX4, SERPINH1, SDCBP2, RASGRP1, PKNOX1, PCNA, MUC1, MMP14, MAFA, LHX9, LGALS1, KLF7, HOXA1, HIPK3, GSK3B, FIT1, FGR, EDC4, E2F2, CUX1, CIAPIN1, CHRNE, CDK2, CBX5, BTG2

Corresponding total number of TFBS:
(For each gene listed above, the total number of TFBS for any of the selected TFs, multiplied by the number of selected Gene ontology terms containing that gene)
55, 55, 50, 45, 45, 44, 44, 44, 40, 40, 36, 36, 35, 33, 33, 33, 33, 33, 33, 33, 33, 33, 33, 30, 30, 30, 30, 30, 27, 27, 27, 24, 24, 22, 22, 22, 22, 22, 22, 22, 22, 22, 22, 21, 20, 20, 20, 20, 20, 18, 18, 18, 16, 16, 16, 16, 16, 15, 15, 15, 14, 12, 12, 12, 12, 12, 12, 11, 11, 11, 11, 11, 11, 11, 11, 11, 11, 11, 11, 11, 11, 11, 11, 11, 11, 11, 11, 11, 11, 11, 11, 11, 11, 10, 10, 10, 10, 10, 10, 10, 10, 10, 10, 10, 9, 9, 9, 9, 9, 9, 9, 9, 9, 9, 9, 8, 8, 8, 8, 8, 8, 8, 7, 7, 7, 7, 7, 7, 7, 7, 6, 6, 6, 6, 6, 6, 6, 6, 6, 6, 6, 6, 5, 5, 5, 5, 5, 4, 4, 4, 4, 4, 4, 4, 4, 4, 4, 4, 4, 4, 4, 4, 4, 4, 4, 3, 3, 3, 3, 3, 3, 3, 3, 3, 3, 3, 3, 3, 3, 2, 2, 2, 2, 2, 2, 2, 2, 2, 2, 2, 2, 2, 2, 2, 2, 2, 2, 2, 2, 2, 2, 2, 2, 2, 2, 2, 2, 2, 2, 1, 1, 1, 1, 1, 1, 1, 1, 1, 1, 1, 1, 1, 1, 1, 1, 1, 1, 1, 1, 1, 1, 1, 1, 1, 1, 1, 1

Corresponding number of selected Gene ontology terms each gene is involved with:
(In the same order as above)
5, 5, 5, 5, 5, 4, 4, 4, 4, 5, 4, 4, 5, 3, 3, 3, 3, 3, 3, 3, 3, 3, 3, 3, 3, 3, 3, 3, 3, 3, 3, 3, 2, 2, 2, 2, 2, 2, 2, 2, 2, 2, 3, 2, 2, 5, 2, 5, 2, 2, 3, 4, 2, 2, 2, 2, 5, 5, 3, 2, 2, 4, 4, 2, 3, 4, 1, 1, 1, 1, 1, 1, 1, 1, 1, 1, 1, 1, 1, 1, 1, 1, 1, 1, 1, 1, 1, 1, 1, 1, 1, 1, 5, 1, 1, 1, 1, 1, 1, 1, 1, 2, 1, 1, 3, 1, 3, 1, 1, 3, 1, 1, 1, 1, 1, 2, 1, 1, 2, 1, 1, 2, 1, 1, 1, 1, 1, 1, 1, 1, 1, 3, 3, 3, 3, 1, 1, 1, 1, 1, 2, 2, 2, 1, 1, 1, 5, 1, 1, 2, 1, 1, 1, 1, 1, 1, 1, 1, 1, 4, 1, 1, 1, 2, 1, 1, 1, 1,

3, 1, 1, 1, 1, 1, 1, 1, 3, 1, 3, 1, 1, 2, 2, 1, 2, 1, 1, 2, 1, 1, 2, 2, 2, 2, 1, 1, 2, 2, 2, 2, 2, 2, 1, 2, 2, 1, 1, 1, 1, 1, 1, 1, 1, 1, 1, 1, 1, 1, 1, 1, 1, 1, 1, 1, 1, 1, 1, 1, 1, 1, 1, 1, 1, 1, 1, 1, 1

Corresponding number of selected TFs each gene is involved with:
(In the same order as above)
11, 11, 10, 9, 9, 11, 11, 11, 10, 8, 9, 9, 7, 11, 11, 11, 11, 11, 11, 11, 11, 11, 11, 10, 10, 10, 10, 10, 9, 9, 9, 8, 8, 11, 11, 11, 11, 11, 11, 11, 11, 11, 11, 7, 10, 10, 4, 10, 4, 9, 9, 6, 4, 8, 8, 8, 8, 3, 3, 5, 7, 6, 3, 3, 6, 4, 3, 11, 11, 11, 11, 11, 11, 11, 11, 11, 11, 11, 11, 11, 11, 11, 11, 11, 11, 11, 11, 11, 11, 11, 11, 2, 10, 10, 10, 10, 10, 10, 10, 10, 5, 10, 10, 3, 9, 3, 9, 9, 3, 9, 9, 9, 9, 9, 4, 8, 8, 4, 8, 8, 4, 7, 7, 7, 7, 7, 7, 7, 7, 6, 2, 2, 2, 2, 6, 6, 6, 6, 6, 3, 3, 3, 5, 5, 5, 1, 5, 4, 2, 4, 4, 4, 4, 4, 4, 4, 4, 4, 1, 4, 4, 4, 2, 4, 4, 3, 3, 1, 3, 3, 3, 3, 3, 3, 3, 1, 3, 1, 2, 2, 1, 1, 2, 1, 2, 2, 1, 2, 2, 1, 1, 1, 1, 2, 2, 1, 1, 1, 1, 1, 2, 1, 1, 2, 2, 1, 1, 1, 1, 1, 1, 1, 1, 1, 1, 1, 1, 1, 1, 1, 1, 1, 1, 1, 1, 1, 1, 1, 1, 1, 1, 1, 1

Gene ontology terms ranked according to mean of effect sizes in cluster:
hemopoiesis | cell development | Hedgehog signaling complex | peptide transport | cell differentiation | T cell differentiation

Corresponding mean of effect sizes of each Gene ontology term:
(In the same order as above)
2.24, 2.03, 1.98, 1.89, 1.88, 1.46

Corresponding total number of genes of each Gene ontology term:
(In the same order as above)
343, 507, 230, 32, 871, 172

Corresponding number of genes of each Gene ontology term with at least one TFBS (of selected TFs):
(In the same order as above)
80, 100, 49, 9, 159, 40

Corresponding total number of TFBS (of selected TFs) for each Gene ontology term (in all genes):
(In the same order as above)
497, 622, 319, 69, 932, 251

Corresponding number of selected TFs each Gene ontology term is involved with:
(In the same order as above)
11, 11, 11, 11, 11, 11

TFs ranked according to mean of effect sizes in cluster:
V$PXRRXR_01, V$PXRRXR_02, V$STAT3_02, V$STAT6_02, V$TEF1_Q6, V$MYB_Q5_01, VMYB_Q3, VNCX_01, PGBP_Q6, VRFX_Q6, V$TFIIA_Q6

Corresponding mean of effect sizes of each TF:
(In the same order as above)
2.43, 2.32, 2.11, 2.09, 2.04, 1.88, 1.79, 1.73, 1.61, 1.56, 1.46

Corresponding total number of TFBS for each TF (genome-wide):
(In the same order as above)
723, 727, 718, 737, 722, 693, 691, 701, 699, 713, 691

Corresponding total number of TFBS for each TF (in all genes in selected Gene ontology terms):
(In the same order as above)
267, 261, 263, 279, 261, 237, 237, 223, 223, 218, 221

Corresponding number of genes (of selected Gene ontology terms) each TF is involved with:
(In the same order as above)

137, 133, 133, 145, 134, 122, 116, 117, 110, 115, 130

Corresponding number of selected Gene ontology terms each TF is involved with:
(In the same order as above)
6, 6, 6, 6, 6, 6, 6, 6, 6, 6, 6

Cluster for columns 457 to 539, rows 307 to 312

Gene ontology terms

chemotaxis | leukocyte migration | cell chemotaxis | positive chemotaxis | B cell activation | immunoglobulin production | B cell proliferation | immunoglobulin secretion | interferon-gamma production | T-helper 1 type immune response | response to host immune response | adaptive immune response | adaptive immune response based on somatic recombination of immune receptors built from immunoglobulin superfamily domains | chemokine production | interleukin-10 production | interleukin-12 production | cell maturation | MHC class I biosynthetic process | MHC class I protein binding | MHC class II biosynthetic process | MHC class II protein binding | antigen processing and presentation | immune response | type IV hypersensitivity | lymphocyte proliferation | tolerance induction | lymphocyte activation | interleukin-2 production | interleukin-2 receptor activity | T cell proliferation | interleukin-4 production | cytokine production | cytokine secretion | pathogenesis | interleukin-4 receptor activity | interleukin-5 production | evasion or tolerance of immune response of other organism during symbiotic interaction | tryptophan catabolic process | defense response to virus | innate immune response | tumor necrosis factor receptor activity | tumor necrosis factor receptor binding | positive regulation of NF-kappaB transcription factor activity | response to tumor necrosis factor | IkappaB kinase complex | NF-kappaB binding | interleukin-6 receptor activity | interleukin-1 beta production | interleukin-1 production | cytolysis | natural killer cell mediated cytotoxicity | natural killer cell receptor activity | naringenin-chalcone synthase activity | immature T cell proliferation in the thymus | eosinophil activation | leukocyte activation | monocyte activation | mucosal immune response | granulocyte macrophage colony-stimulating factor biosynthetic process | granulocyte macrophage colony-stimulating factor production | interleukin-1 receptor activity | interleukin-6 production | tumor necrosis factor production | macrophage activation | inflammatory response | response to lipopolysaccharide | negative regulation of inflammatory response | regulation of tumor necrosis factor production | negative regulation of tumor necrosis factor production | positive regulation of tumor necrosis factor production | interleukin-1 receptor antagonist activity | acute inflammatory response | granuloma formation | lipopolysaccharide binding | cytokine biosynthetic process | interleukin-8 production | mRNA transcription | chronic inflammatory response | monocyte differentiation | hypersensitivity | hyaluronic acid binding | cytokine activity | rosetting

TFs

V$NFKB_Q6_01 | V$NFKAPPAB_01 | V$NFKAPPAB65_01 | V$CREL_01 | V$NFKB_Q6 | V$NFKB_C

Information

All related TFs:
(List of all TFs that are related to any of the PWMs)
NF-TNF, NF-kappaB, NF-kappaB(-like), NF-kappaB2, RelA-p65, c-Rel, p100, p105, p50, p52

Ranked gene list:
(All genes of the selected Gene ontology terms with hits of any of the selected TFs, ranked according to the total number of TFBS)
TNF, ICAM1, CD4, LTA, IRF6, CD40, CSF1, NFKBIA, CD69, REL, STAT1, CXCL10, NOD2, LTB, AKT1, IL2RA, TNFRSF4, STAT6, IRF1, RELA, EDC4, CD58, TNFRSF18, CD83, NFKB1, TNFRSF9,

TNFSF13, TGFB1, RELB, IL17C, FLT3LG, ITGAM, CD86, CD5, PER1, DPP4, CD70, TRAF2, NFATC2, CD27, PTPN6, PSMB8, MAPK14, IRF8, CREB1, IL6ST, IL1RN, IL11, GPR132, FAS, CXCR5, TRAF3, PTMA, MYD88, PDLIM7, ISYNA1, TRADD, TNFRSF6B, TNFAIP3, NFKBIB, MAP2K3, IL27, IKBKE, B2M, NFATC1, IL23A, ERCC8, EPHB2, PDCD1, PDCD1LG2, NFKB2, LAG3, JUNB, IRF5, HLA-A, CXCL1, IRF3, IL16, EGR1, CXCL5, MYB, MIF, IL7R, STAT3, PTGS1, PDE4A, PAX5, LCK, IFIH1, TNFRSF1B, TNIP1, PSMB9, LMNA, IRF2, CFLAR, BIRC3, VEGFA, SOCS3, KLRK1, TRIM63, TRAF1, TAPBP, TAP1, FGR, CISH, MAP3K8, GADD45B, CYLD, CCND1, AGER, JAK1, HLA-B, CXCL6, CCL21, RPS6KA1, ROBLD3, PLEKHF1, PLAU, PARP1, NR3C1, NFKBIE, NCAPG2, MAP4K4, LGALS3, IKBKB, ICOSLG, HIVEP2, DLX2, CXCL2, CX3CL1, TNFRSF25, ZAP70, TNFRSF14, TNFRSF11B, TMPO, STXBP3, PRG2, NDUFA2, JAK3, HSP90B1, DUSP2, SLC19A1, PSME3, PSME2, MPRIP, IL19, IL12B, HLA-DMB, GATA3, CCR10, TYROBP, TSHR, TOLLIP, SMPD1, SLC25A22, RXRB, RAB27A, PTGER4, NBPF1, IL32, HLA-C, EIF4G2, EDAR, CXCL16, COL11A2, CDKN2C, CD300A, MUC1, ICAM2, ZC3H12A, TRAF4, TMEM201, TAP2, SSSCA1, SEMA3B, RNASEK, PTGDS, PLCG1, PIBF1, PGF, PACS1, NGFR, NFKBIZ, MAZ, MAP3K14, MAP3K11, MAL, HSPA1A, HIVEP1, HCST, GABARAP, FLNA, EHD1, CYSLTR1, CXCL3, CSK, CREBBP, CRADD, CDC37, CD55, CCL25, CANX, CAMK2G, BCL3, ABCB9, ZFP36, SP1, SMPD2, SHH, RUNX3, RTEL1, PRDX2, MAP3K1, IL27RA, FYN, ERAP1, CCL1, ACTB, WDR26, TUBGCP2, TSPAN4, TSC22D3, TRPV3, TNFRSF12A, TIFA, TCIRG1, STAG1, SPATA2, SPAG1, SIRT1, SEPSECS, S1PR2, PTGER1, ORAI1, NSFL1C, NR4A2, NOTCH1, NANP, MR1, LSP1, KSR1, JUND, ITGA5, ILK, HSP90AA1, HIF1A, HAMP, GSTP1, ESAM, EGR2, DCTN3, CTSS, CRY2, CRTC2, CORO7, CFL1, BCL6, ART3, AHR, VIM, VDAC1, UBD, TUSC2, TSC2, STMN1, SH2D3C, S1PR4, RUNX1, RIPK3, RHOF, RAC2, PVRL1, PTPN7, PLP1, PLAUR, PAWR, NTRK1, NR4A1, NOTCH4, MLLT10, LRDD, KLF6, IRS1, IL4R, HFE, FOXP1, FOSB, FLJ32987, ETV6, EPB41, DCXR, DAXX, COL6A1, CDKN2A, CDK4, CCND3, CCND2, CASP6, BRAF, BDNF, ATXN1, ATN1, AIRE, AFF3, ADRBK2, ZMYND10, ZFPM1, XRCC1, WHSC1, UNC13D, UBE2L6, TNIP3, TCEA2, STRA13, SMAD7, SFRS2, SERPINH1, SERPINA1, SEC62, RTN2, REV3L, RASSF5, RASSF1, RARA, PTGES, PSMP, PPP3CA, PPARGC1B, POLDIP3, PNKD, PIM1, PIK3CD, PAK4, P21, OTP, NXT1, NTRK3, NRAS, NFYA, NFKBID, MEN1, LRP2, KIAA1524, INHBB, GSK3B, GPR172B, GDNF, GBA, GATA4, GALNT6, FDXR, EOMES, EFNA1, ECE1, EBAG9, DUSP28, DNAH1, DIABLO, DHX58, DAPP1, DAD1, CYP27B1, CXCR7, CKLF, CKAP4, CIR, CHI3L2, CDK5R2, CACNB3, BTRC, ANKRA2, ALCAM, ADA, ZBTB16, VCAN, USF2, USF1, UNC5A, TRIM69, TCEB2, TBP, TBC1D1, SRF, SREBF1, SP2, SNCG, SNCB, SLC36A2, SLC25A18, SKAP1, SERPINB9, SERPINA4, SEMA4A, S100A6, RPS6KA3, RET, PTHLH, PLTP, PDGFRA, PDGFB, PDE4DIP, PARK7, PAOX, PAFAH1B1, OGFR, MVD, MSN, MAPK12, MAPK11, MAG, LY6E, LSM2, ITM2B, IFIT3, IER3, HDC, H2AFX, GNB2L1, FUT8, FOSL1, FHL3, FGF8, FGF1, FBL, ERN1, ERCC2, ELF3, EIF4E, DRD4, DMPK, DISC1, CYBA, CDK9, CDC2L5, CD3D, CD37, CARD8, C11orf2, BCL9, BATF, AMH, ALOX5AP, ALG1, AGPAT1

Corresponding total number of TFBS:
(For each gene listed above, the total number of TFBS for any of the selected TFs, multiplied by the number of selected Gene ontology terms containing that gene)
492, 372, 280, 258, 219, 186, 174, 150, 144, 138, 136, 128, 126, 120, 120, 112, 105, 102, 95, 93, 84, 72, 70, 70, 66, 64, 63, 60, 60, 60, 60, 57, 56, 54, 50, 48, 48, 45, 45, 42, 40, 40, 40, 40, 40, 39, 39, 39, 39, 39, 36, 35, 35, 34, 33, 31, 30, 30, 30, 30, 30, 30, 30, 30, 28, 28, 26, 26, 25, 24, 24, 24, 24, 24, 24, 24, 22, 22, 22, 22, 21, 21, 21, 20, 20, 20, 20, 20, 20, 19, 18, 18, 18, 18, 18, 18, 17, 17, 17, 16, 16, 16, 16, 16, 16, 15, 15, 15, 15, 15, 14, 14, 14, 13, 12, 12, 12, 12, 12, 12, 12, 12, 12, 12, 12, 12, 12, 12, 12, 11, 10, 10, 10, 10, 10, 10, 10, 10, 10, 10, 10, 10, 10, 10, 10, 9, 9, 9, 9, 9, 9, 9, 9, 9, 8, 8, 8, 8, 8, 8, 8, 8, 8, 8, 8, 8, 8, 8, 8, 8, 7, 7, 6, 6, 6, 6, 6, 6, 6, 6, 6, 6, 6, 6, 6, 6, 6, 6, 6, 6, 6, 6, 6, 6, 6, 6, 6, 6, 6, 6, 6, 6, 6, 6, 6, 5, 5, 5, 5, 5, 5, 5, 5, 5, 5, 5, 5, 5, 4, 4, 4, 4, 4, 4, 4, 4, 4, 4, 4, 4, 4, 4, 4, 4, 4, 4, 4, 4, 4, 4, 4, 4, 4, 4, 4, 4, 4, 4, 4, 4, 4, 4, 4, 4, 4, 4, 4, 4, 3, 3, 3, 3, 3, 3, 3, 3, 3, 3, 3, 3, 3, 3, 3, 3, 3, 3, 3, 3, 3, 3, 3, 3, 3, 3, 3, 3, 3, 3, 3, 3, 3, 3, 3, 3, 3, 3, 3, 3, 3, 3, 3, 3, 3, 3, 3, 2, 2, 2, 2, 2, 2, 2, 2, 2, 2, 2, 2, 2, 2, 2, 2, 2, 2, 2, 2, 2, 2, 2, 2, 2, 2, 2, 2, 2, 2, 2, 2, 2, 2, 2, 2, 2, 2, 2, 2, 2, 2, 2, 2, 2, 2, 2, 2, 2, 2, 2, 2, 2, 2, 2, 2, 2, 2, 2, 2, 2, 2, 2, 2, 2, 2, 2, 2, 2, 2, 1, 1, 1, 1, 1, 1, 1, 1, 1, 1, 1, 1, 1, 1, 1, 1, 1, 1, 1, 1, 1, 1, 1, 1, 1, 1, 1, 1, 1, 1, 1, 1, 1, 1, 1, 1, 1, 1, 1, 1, 1, 1, 1, 1, 1, 1, 1, 1, 1, 1, 1, 1, 1, 1, 1, 1, 1, 1, 1, 1, 1, 1, 1, 1, 1, 1, 1

Corresponding number of selected Gene ontology terms each gene is involved with:

(In the same order as above)
82, 62, 70, 43, 73, 62, 29, 25, 48, 23, 34, 32, 21, 20, 20, 56, 21, 17, 19, 31, 21, 24, 14, 35, 11, 16, 63, 12, 15, 15, 12, 57, 56, 18, 10, 12, 12, 9, 9, 21, 10, 8, 40, 10, 8, 13, 39, 13, 13, 39, 9, 7, 7, 34, 11, 31, 6, 5, 6, 6, 6, 10, 5, 5, 7, 14, 26, 26, 5, 12, 4, 6, 4, 4, 12, 12, 11, 11, 11, 11, 7, 21, 7, 20, 5, 5, 4, 20, 4, 19, 3, 9, 6, 6, 6, 6, 17, 17, 17, 8, 4, 4, 8, 4, 8, 5, 3, 3, 5, 3, 7, 7, 7, 13, 2, 2, 2, 6, 4, 6, 2, 12, 6, 4, 12, 12, 4, 3, 12, 12, 11, 5, 5, 10, 5, 2, 10, 10, 10, 5, 2, 3, 3, 3, 3, 3, 9, 3, 3, 3, 4, 8, 8, 4, 2, 2, 2, 8, 4, 2, 8, 4, 4, 4, 4, 2, 4, 7, 7, 1, 3, 2, 6, 3, 1, 1, 6, 3, 3, 2, 2, 2, 2, 1, 6, 1, 2, 6, 2, 3, 3, 1, 2, 6, 6, 3, 6, 3, 1, 3, 3, 6, 1, 1, 3, 1, 5, 5, 1, 1, 1, 1, 5, 5, 5, 5, 5, 5, 1, 4, 1, 2, 1, 4, 1, 2, 1, 2, 2, 1, 2, 1, 2, 2, 4, 2, 2, 4, 4, 1, 1, 1, 4, 1, 4, 4, 4, 1, 4, 1, 2, 4, 1, 1, 2, 2, 4, 1, 4, 1, 3, 3, 1, 1, 1, 1, 3, 3, 3, 1, 1, 3, 1, 3, 3, 3, 3, 1, 1, 1, 1, 1, 3, 3, 3, 3, 1, 3, 1, 1, 3, 1, 1, 3, 1, 3, 3, 3, 1, 1, 1, 1, 1, 1, 1, 1, 1, 1, 1, 2, 1, 2, 1, 1, 2, 1, 1, 2, 1, 2, 1, 2, 1, 2, 2, 2, 1, 1, 2, 1, 1, 1, 1, 1, 1, 1, 2, 1, 2, 1, 1, 1, 2, 1, 1, 1, 2, 1, 1, 2, 1, 1, 1, 1, 2, 1, 1, 1, 2, 1, 2, 1, 2, 1, 1, 2, 1, 2, 1, 2, 2, 2, 1, 1, 1, 1, 1, 1, 1, 1, 1, 1, 1, 1, 1, 1, 1, 1, 1, 1, 1, 1, 1, 1, 1, 1, 1, 1, 1, 1, 1, 1, 1, 1, 1, 1, 1, 1, 1, 1, 1, 1, 1, 1, 1, 1, 1, 1, 1, 1, 1, 1, 1, 1, 1, 1, 1, 1, 1, 1, 1, 1, 1, 1, 1, 1, 1, 1, 1, 1, 1, 1

Corresponding number of selected TFs each gene is involved with:
(In the same order as above)
6, 6, 4, 6, 3, 3, 6, 6, 3, 6, 4, 4, 6, 6, 6, 2, 5, 6, 5, 3, 4, 3, 5, 2, 6, 4, 1, 5, 4, 4, 5, 1, 1, 3, 5, 4, 4, 5, 5, 2, 4, 5, 1, 4, 5, 3, 1, 3, 3, 1, 4, 5, 5, 1, 3, 1, 5, 6, 5, 5, 5, 3, 6, 6, 4, 2, 1, 1, 5, 2, 6, 4, 6, 6, 2, 2, 2, 2, 2, 2, 3, 1, 3, 1, 4, 4, 5, 1, 5, 1, 6, 2, 3, 3, 3, 3, 1, 1, 1, 2, 4, 4, 2, 4, 2, 3, 5, 5, 3, 5, 2, 2, 2, 1, 6, 6, 6, 2, 3, 2, 6, 1, 2, 3, 1, 1, 3, 4, 1, 1, 1, 2, 2, 1, 2, 5, 1, 1, 1, 2, 5, 3, 3, 3, 3, 3, 1, 3, 3, 3, 2, 1, 1, 2, 4, 4, 4, 1, 2, 4, 1, 2, 2, 2, 2, 4, 2, 1, 1, 6, 2, 3, 1, 2, 6, 6, 1, 2, 2, 3, 3, 3, 3, 6, 1, 6, 3, 1, 3, 2, 2, 6, 3, 1, 1, 2, 1, 2, 6, 2, 2, 1, 6, 6, 2, 5, 1, 1, 5, 5, 5, 5, 1, 1, 1, 1, 1, 1, 4, 1, 4, 2, 4, 1, 4, 2, 4, 2, 2, 4, 2, 4, 2, 2, 1, 2, 2, 1, 1, 4, 4, 4, 1, 4, 1, 1, 1, 4, 1, 4, 2, 1, 4, 4, 2, 2, 1, 4, 1, 3, 1, 1, 3, 3, 3, 3, 1, 1, 1, 3, 3, 1, 3, 1, 1, 1, 1, 3, 3, 3, 3, 3, 1, 1, 1, 1, 3, 1, 3, 3, 1, 3, 3, 1, 3, 1, 1, 1, 3, 3, 3, 3, 3, 3, 3, 2, 2, 2, 2, 1, 2, 1, 2, 2, 1, 2, 2, 1, 2, 1, 2, 1, 2, 1, 1, 1, 2, 2, 1, 2, 2, 2, 2, 2, 2, 2, 1, 2, 1, 2, 2, 2, 1, 2, 2, 2, 1, 2, 2, 1, 2, 2, 2, 2, 1, 2, 2, 2, 1, 2, 1, 2, 1, 2, 2, 1, 2, 1, 2, 1, 1, 1, 2, 1, 1, 1, 1, 1, 1, 1, 1, 1, 1, 1, 1, 1, 1, 1, 1, 1, 1, 1, 1, 1, 1, 1, 1, 1, 1, 1, 1, 1, 1, 1, 1, 1, 1, 1, 1, 1, 1, 1, 1, 1, 1, 1, 1, 1, 1, 1, 1, 1, 1, 1, 1, 1, 1, 1, 1, 1, 1, 1, 1, 1, 1, 1, 1, 1, 1, 1, 1, 1, 1, 1

Gene ontology terms ranked according to mean of effect sizes in cluster:
IkappaB kinase complex | response to tumor necrosis factor | tumor necrosis factor receptor activity | tumor necrosis factor receptor binding | immune response | interleukin-12 production | NF-kappaB binding | chemokine production | positive regulation of NF-kappaB transcription factor activity | response to lipopolysaccharide | MHC class I protein binding | MHC class I biosynthetic process | cytokine secretion | interleukin-8 production | granulocyte macrophage colony-stimulating factor production | interleukin-6 production | T-helper 1 type immune response | granulocyte macrophage colony-stimulating factor biosynthetic process | cytokine production | innate immune response | inflammatory response | granuloma formation | adaptive immune response based on somatic recombination of immune receptors built from immunoglobulin superfamily domains | regulation of tumor necrosis factor production | interleukin-10 production | response to host immune response | mucosal immune response | T cell proliferation | chronic inflammatory response | evasion or tolerance of immune response of other organism during symbiotic interaction | macrophage activation | adaptive immune response | interferon-gamma production | naringenin-chalcone synthase activity | monocyte activation | interleukin-4 production | interleukin-2 receptor activity | positive chemotaxis | interleukin-6 receptor activity | interleukin-2 production | B cell activation | negative regulation of tumor necrosis factor production | antigen processing and presentation | cytokine biosynthetic process | lymphocyte proliferation | defense response to virus | tumor necrosis factor production | interleukin-1 receptor antagonist activity | B cell proliferation | mRNA transcription | interleukin-5 production | leukocyte activation | immunoglobulin production | acute inflammatory response | immature T cell proliferation in the thymus | cell maturation | positive regulation of tumor necrosis factor production | interleukin-4 receptor activity | type IV hypersensitivity | MHC class II biosynthetic process | negative regulation of inflammatory response | cytokine activity | MHC class II protein binding | cytolysis | pathogenesis | rosetting | immunoglobulin secretion | lymphocyte activation | tryptophan catabolic process | tolerance induction | leukocyte migration | interleukin-1 receptor activity | interleukin-1 beta production | hypersensitivity | natural killer cell receptor activity | monocyte differentiation | chemotaxis | eosinophil activation | hyaluronic acid binding | lipopolysaccharide binding | interleukin-1 production | cell chemotaxis | natural killer cell mediated cytotoxicity

Corresponding mean of effect sizes of each Gene ontology term:
(In the same order as above)
4.04, 3.96, 3.84, 3.77, 3.59, 3.58, 3.54, 3.50, 3.41, 3.31, 3.18, 3.09, 3.06, 3.00, 2.98, 2.95, 2.94, 2.88, 2.74, 2.71, 2.68, 2.67, 2.67, 2.59, 2.56, 2.45, 2.43, 2.37, 2.31, 2.21, 2.20, 2.20, 2.14, 2.13, 2.12, 2.11, 2.09, 2.06, 2.06, 2.03, 2.03, 2.02, 2.02, 1.98, 1.95, 1.89, 1.81, 1.80, 1.78, 1.72, 1.68, 1.68, 1.64, 1.64, 1.62, 1.60, 1.60, 1.56, 1.53, 1.53, 1.47, 1.47, 1.42, 1.42, 1.41, 1.35, 1.34, 1.25, 1.22, 1.21, 1.18, 1.16, 1.07, 0.98, 0.97, 0.96, 0.91, 0.77, 0.59, 0.58, 0.48, 0.46, 0.43

Corresponding total number of genes of each Gene ontology term:
(In the same order as above)
107, 185, 213, 214, 530, 150, 146, 155, 550, 226, 197, 195, 243, 144, 107, 165, 59, 109, 455, 246, 425, 77, 131, 86, 166, 37, 52, 188, 22, 141, 178, 148, 106, 50, 117, 123, 189, 87, 104, 182, 116, 61, 315, 108, 158, 110, 209, 70, 88, 130, 57, 108, 155, 68, 42, 224, 61, 87, 131, 181, 40, 42, 178, 179, 1.1K, 63, 51, 232, 41, 109, 145, 128, 21, 234, 77, 102, 371, 71, 37, 61, 31, 94, 33

Corresponding number of genes of each Gene ontology term with at least one TFBS (of selected TFs):
(In the same order as above)
34, 53, 61, 61, 110, 41, 46, 41, 131, 56, 56, 55, 52, 35, 26, 42, 17, 25, 94, 51, 82, 17, 28, 25, 42, 10, 11, 42, 6, 34, 38, 29, 21, 10, 23, 26, 43, 21, 24, 46, 27, 17, 67, 27, 32, 30, 40, 15, 20, 33, 13, 18, 28, 14, 11, 48, 17, 24, 30, 38, 12, 9, 37, 32, 192, 12, 11, 50, 10, 21, 26, 23, 4, 31, 14, 17, 56, 9, 6, 12, 4, 17, 5

Corresponding total number of TFBS (of selected TFs) for each Gene ontology term (in all genes):
(In the same order as above)
119, 168, 180, 180, 295, 122, 134, 105, 377, 175, 156, 154, 162, 102, 80, 123, 49, 79, 261, 137, 218, 51, 69, 71, 107, 32, 38, 120, 25, 88, 111, 75, 60, 35, 61, 82, 125, 53, 66, 138, 76, 48, 176, 74, 93, 80, 115, 47, 62, 96, 44, 51, 72, 35, 34, 131, 43, 62, 78, 98, 29, 32, 95, 81, 458, 37, 32, 138, 33, 60, 67, 62, 13, 90, 33, 46, 128, 27, 17, 21, 11, 40, 15

Corresponding number of selected TFs each Gene ontology term is involved with:
(In the same order as above)
6, 6, 6, 6, 6, 6, 6, 6, 6, 6, 6, 6, 6, 6, 6, 6, 6, 6, 6, 6, 6, 6, 6, 6, 6, 6, 6, 6, 6, 6, 6, 6, 6, 6, 6, 6, 6, 6, 6, 6, 6, 6, 6, 6, 6, 6, 6, 6, 6, 6, 6, 6, 6, 6, 6, 6, 6, 6, 6, 6, 6, 6, 6, 6, 6, 6, 6, 6, 6, 6, 6, 6, 6, 6, 6, 6, 6, 6, 6, 6, 6, 6, 6

TFs ranked according to mean of effect sizes in cluster:
V$NFKAPPAB_01, V$NFKB_C, V$NFKAPPAB65_01, V$NFKB_Q6_01, V$NFKB_Q6, V$CREL_01

Corresponding mean of effect sizes of each TF:
(In the same order as above)
2.36, 2.30, 2.18, 2.15, 1.96, 1.43

Corresponding total number of TFBS for each TF (genome-wide):
(In the same order as above)
702, 685, 716, 713, 689, 713

Corresponding total number of TFBS for each TF (in all genes in selected Gene ontology terms):
(In the same order as above)
1420, 1308, 1346, 1313, 1283, 1193

Corresponding number of genes (of selected Gene ontology terms) each TF is involved with:
(In the same order as above)
177, 165, 189, 175, 182, 178

Corresponding number of selected Gene ontology terms each TF is involved with:
(In the same order as above)

83, 83, 83, 83, 83, 83

Cluster for columns 3 to 11, rows 83 to 111

Gene ontology terms

hemopoiesis | cell differentiation | T cell differentiation | cell development | cell activation | T cell activation | T cell receptor complex | immunological synapse | integrin activation

TFs

V$GATA1_05 | V$FAC1_01 | V$TITF1_Q3 | B$CRP_C | V$GATA_Q6 | V$GATA6_01 | V$TCF4_Q5 | V$LEF1_Q2 | V$BRCA_01 | V$AREB6_04 | V$SOX10_Q6 | V$DBP_Q6 | V$CEBP_01 | V$AP3_Q6 | V$STAT6_01 | V$STAT5A_03 | V$STAT4_01 | V$STAT5A_04 | V$STAT1_03 | V$CEBP_Q3 | V$PAX2_02 | V$HMGIY_Q6 | V$HOXA4_Q2 | V$TST1_01 | V$NFAT_Q6 | V$NFAT_Q4_01 | V$CEBP_Q2_01 | V$CEBPA_01 | V$CEBP_Q2

Information

All related TFs:
(List of all TFs that are related to any of the PWMs)
ANF-2, AP-3, BRCA1, BRCA1:USF2, C/EBP, C/EBPalpha, C/EBPalpha(p20), C/EBPalpha(p30), C/EBPbeta, C/EBPbeta(LAP), C/EBPbeta(p20), C/EBPbeta(p34), C/EBPbeta(p35), C/EBPdelta, C/EBPepsilon, C/EBPgamma, DBP, FAC1, GATA-1, GATA-1A, GATA-2, GATA-3, GATA-4, GATA-5, GATA-5A, GATA-5B, GATA-6, GATA-6A, GATA-6B, HMG, HMG-Y, HMGI-C, HOXA4, LEF-1, LEF-1S, NF-AT, NF-AT1, NF-AT1C, NF-AT2, NF-AT3, NF-AT4, Nkx2-1, POU3F1, Pax-2, Pax-2.1, Pax-2.2, Pax-2a, Pax-2b, STAT1, STAT1alpha, STAT4, STAT5A, STAT6, Sox10, TCF-1, TCF-1(P), TCF-3, TCF-4, ZEB

Ranked gene list:
(All genes of the selected Gene ontology terms with hits of any of the selected TFs, ranked according to the total number of TFBS)
CXCR4, CCR7, PTPN6, FYN, CD4, CD44, ETS1, CISH, GFI1, CSK, TNF, TCF7, LEF1, SHC1, SATB1, ITGA5, FYB, CDKN1B, BCL2L1, RUNX1, IL7R, IKZF1, STAT1, PLCG1, CTLA4, BCL11B, SH2B3, GATA3, CD3G, SSSCA1, LTB, SKAP1, NR3C2, IRF1, CD68, LCP1, BCL2, CCND2, BCL6, SOCS1, PRDM1, PER1, ID2, CCND3, AKT1, LTA, PTK2B, JUNB, LAG3, CD247, ANXA6, NFATC1, LBX1, RUNX3, PIK3R1, MYB, EGR1, ZBTB7B, HOXA10, FLI1, ID3, STAT5B, STAT5A, NR4A2, LMO4, ZAP70, PAX6, NFKBIA, LCK, CD3D, ARL4C, MAF, HOXA9, HES1, BCL2L11, RASSF5, NFATC3, HOXB4, RARA, BHLHB2, PAX5, HOXB3, GIMAP5, EBF1, NKX2-2, STAT6, ICOS, DUSP2, DLL1, RBPJ, EGR2, STIM1, PTPN7, IL16, STAT3, SMAD3, RASGRP1, ITGB2, PIK3CG, LCP2, IL2RA, GATA2, VEGFA, IL23A, CSF1, TBX21, MYC, JUN, GLI1, CFLAR, SMAD7, MLL5, MEF2D, HOXC9, HOXA11, ATXN1, RARG, MLL, LDB1, FAM65B, EOMES, DGKA, CXCR5, CDK4, CD69, CD27, ARHGEF2, PTMA, PITX3, HOXA7, CDKN2C, ALCAM, TNFRSF1A, RORC, MAP3K12, MAP3K1, IRF2, HIST1H4I, CDK6, CD226, PIK3CD, MYO1G, GRB2, ARL6IP5, WIPF1, UNK, TNFAIP8, TAP1, NFIA, LYL1, KLF7, CFL1, CD2, CBX5, IRS1, ILK, BACH2, VIM, SOS1, OSM, ETV6, DOK2, CTNNB1, CRIP1, CD82, BMI1, TCIRG1, TAPBP, SOCS2, MAPK14, ITGB1, HOXC4, FAS, SLAMF1, RING1, PXN, PSMB9, LHX9, HOXC6, HOXC5, HAND2, CREB1, CD63, STK16, NEUROG2, ITPR1, HNRNPL, DUSP6, ZFPM1, S1PR1, PSTPIP1, POU2F2, ITK, POU4F1, PLEKHG2, JARID2, ITGB7, UBC, TFAP2A, POU3F2, NEDD9, NDRG1, IL11RA, FLJ32987, CDK5, CD53, CD47, TXK, STK17B, SOCS3, SILV, ITGA6, HOXB6, CDK2, TAF8, PROX1, MAP4K4, KLF2, IL6, GSK3B, CD79A, CADM1, BCL11A, BATF, PPP1R9B, POLD4, MAL, HOXA1, GRAP2, VDR, TRAF3, TLR9, SOX2, SEMA4D, PDLIM5, PCNA, MYD88, KCNN4, KCNA3, IL21R, CSF2, CD5, CD14, CALM2, CALM1, AHSA1, NR2F2, LFNG, HIST4H4, GGNBP2, DAXX, XAB2, TNFRSF4,

STIM2, SMAD5, RAF1, PTPN11, NR4A1, HMBS, CAP1, TNFSF13, FST, FOS, FLII, FGF9, ENO2, CDH23, CALR, BAK1, ZEB1, WNK1, TNFRSF18, SELPLG, SCARB2, RPL17, RERE, PIM1, PAG1, MZF1, MEIS2, IL2, IKZF3, HES5, HCST, E2F4, E2F2, DAD1, CD28, CBLB, ASB2, ACVR1, SH3BP2, RASGRP2, RAG1, PCGF2, IL10, IGF1R, DEF6, DAPP1, CYP26B1, CD7, CD40, ACTB, TPT1, TFRC, RUNX1T1, RHOA, RBM39, PSMC5, PRKCE, PIK3CA, PIK3C3, JUND, ITGA1, IRF6, INPP5D, IL2RB, CDKN1A, C2orf28, TAL1, SRF, SOX4, RAPGEF1, PEA15, PDGFRA, OTX2, NOTCH1, ISYNA1, HIPK3, GUK1, GTF3A, ESR1, ACVR2A, WASF2, UBASH3B, TYROBP, TRIM28, TNFSF8, TNFSF13B, TNFSF10, TNFRSF1B, STRA13, SP1, RFX3, RBM45, PTPN4, PML, PLEKHA2, PDCD1, NKX6-1, NFE2, MBP, LMO2, LMNA, IRF4, ICAM3, GDF9, FOXO1, ETV5, ELF1, EDC4, DR1, CXXC1, CD83, CD3E, CCR5, BMP4, AKTIP, VAV3, TRAF1, TPM3, TBC1D10C, SIPA1, SETD1A, RGS1, RAP1B, PTPN22, PRKCB, PDE4A, PBX2, NR0B2, NOTCH4, MTHFR, MET, MCAM, JMJD1A, JAK2, IRF8, IL6ST, IGF1, HRAS, HMHA1, HLA-C, GADD45A, FGF2, EPO, EP300, EGF, CRK, CIB1, CCBP2, APBB1IP, ADA, ZNF385A, ZBTB16, WNT7B, UNC13D, TSPAN32, TGFB2, STMN1, SOX5, SMAD4, RRBP1, RRAS, RB1, PRH1, PPARA, POU5F1, POU4F2, POU2F1, POU2AF1, POLR2B, PKN1, PIK3R2, PHLDA1, PDHX, NXT1, NGRN, NFKB1, MUC1, MR1, MEIS1, MAP4K1, MALT1, MAFK, LGALS1, LAT, JAK1, ISL1, ILF3, HLA-B, HBP1, H3F3A, GLIPR1, GDNF, GATA6, FERMT3, EPHB6, DNMT3A, DKFZp434P0672, DHH, DCTN2, CTCF, CDC2L5, CD74, CD37, CD200R1, CD180, CCR6, CASP8, CALM3, ASCL1, ANXA1, XPC, VTN, UBE2I, TTF2, TRAF2, TP53, TCF7L2, TBP, SOX13, SOCS7, SOCS5, SLC2A3, SLC2A1, SLC20A1, SIX3, SIM1, SFTPC, ROBLD3, RAB27A, PUM1, PSMA7, PPIB, PPA1, POU3F1, PDE5A, PAX2, PARP1, OTX1, NR5A2, NR1H2, NOS2, NOD2, NDRG2, MN1, MDK, LYST, LY75, LRRC8A, ITPR3, ITGA3, HSPD1, HNRNPU, GPD2, GIMAP4, GADD45B, FKBP1A, FGR, EED, DDX25, CRTAM, CHRNE, CEBPB, CDR2, CCND1, CASP1, CANX, C1R, BTG2, BDNF, ALAS1

Corresponding total number of TFBS:
(For each gene listed above, the total number of TFBS for any of the selected TFs, multiplied by the number of selected Gene ontology terms containing that gene)
261, 216, 196, 196, 184, 175, 174, 168, 162, 162, 150, 145, 145, 144, 140, 140, 140, 140, 140, 135, 130, 125, 120, 120, 119, 116, 115, 112, 110, 108, 104, 100, 100, 100, 100, 92, 91, 87, 87, 85, 81, 81, 81, 80, 77, 75, 72, 69, 68, 68, 68, 66, 64, 60, 60, 60, 60, 58, 58, 58, 57, 56, 56, 56, 56, 54, 54, 54, 54, 54, 54, 52, 52, 52, 51, 50, 50, 50, 48, 48, 45, 44, 44, 44, 42, 40, 40, 40, 40, 39, 39, 38, 38, 38, 36, 36, 36, 36, 35, 35, 35, 33, 32, 32, 32, 30, 30, 30, 30, 30, 29, 29, 29, 29, 29, 29, 28, 28, 28, 28, 28, 28, 28, 28, 28, 28, 27, 27, 27, 27, 27, 26, 26, 26, 26, 26, 26, 26, 26, 25, 25, 25, 25, 24, 24, 24, 24, 24, 24, 24, 24, 24, 24, 23, 23, 23, 22, 22, 22, 22, 22, 22, 22, 22, 22, 21, 21, 21, 21, 21, 21, 21, 20, 20, 20, 20, 20, 20, 20, 20, 20, 20, 19, 19, 19, 19, 19, 18, 18, 18, 18, 18, 17, 17, 17, 17, 16, 16, 16, 16, 16, 16, 16, 16, 16, 15, 15, 15, 15, 15, 15, 15, 14, 14, 14, 14, 14, 14, 14, 14, 14, 14, 13, 13, 13, 13, 13, 12, 12, 12, 12, 12, 12, 12, 12, 12, 12, 12, 12, 12, 12, 12, 12, 11, 11, 11, 11, 11, 10, 10, 10, 10, 10, 10, 10, 10, 10, 9, 9, 9, 9, 9, 9, 9, 9, 9, 8, 8, 8, 8, 8, 8, 8, 8, 8, 8, 8, 8, 8, 8, 8, 8, 8, 8, 8, 8, 8, 8, 8, 8, 8, 8, 8, 8, 8, 7, 7, 7, 7, 7, 7, 7, 7, 7, 7, 7, 7, 6, 6, 6, 6, 6, 6, 6, 6, 6, 6, 6, 6, 6, 6, 6, 5, 5, 5, 5, 5, 5, 5, 5, 5, 5, 5, 5, 5, 5, 4, 4, 4, 4, 4, 4, 4, 4, 4, 4, 4, 4, 4, 4, 4, 4, 4, 4, 4, 4, 4, 4, 4, 4, 4, 4, 4, 4, 4, 4, 4, 4, 4, 4, 4, 4, 3, 3, 3, 3, 3, 3, 3, 3, 3, 3, 3, 3, 3, 3, 3, 3, 3, 3, 3, 3, 3, 3, 3, 3, 3, 3, 3, 3, 3, 3, 3, 3, 3, 2, 2, 2, 2, 2, 2, 2, 2, 2, 2, 2, 2, 2, 2, 2, 2, 2, 2, 2, 2, 2, 2, 2, 2, 2, 2, 2, 2, 2, 2, 2, 2, 2, 2, 2, 2, 2, 2, 2, 2, 2, 2, 2, 2, 2, 2, 2, 2, 2, 2, 2, 2, 2, 2, 2, 2, 2, 2, 2, 1, 1, 1, 1, 1, 1, 1, 1, 1, 1, 1, 1, 1, 1, 1, 1, 1, 1, 1, 1, 1, 1, 1, 1, 1, 1, 1, 1, 1, 1, 1, 1, 1, 1, 1, 1, 1, 1, 1, 1, 1, 1, 1, 1, 1, 1, 1, 1, 1, 1, 1, 1, 1, 1, 1, 1, 1, 1

Corresponding number of selected Gene ontology terms each gene is involved with:
(In the same order as above)
9, 8, 7, 7, 8, 7, 6, 6, 6, 6, 6, 5, 5, 6, 5, 7, 5, 5, 7, 5, 5, 5, 6, 5, 7, 4, 5, 4, 5, 4, 4, 4, 4, 4, 4, 7, 3, 3, 5, 3, 3, 3, 4, 7, 3, 6, 3, 4, 4, 4, 6, 4, 4, 3, 6, 5, 2, 2, 2, 3, 7, 7, 2, 2, 6, 2, 3, 9, 3, 3, 4, 2, 4, 3, 2, 2, 2, 2, 2, 3, 2, 2, 4, 2, 4, 5, 2, 2, 3, 3, 2, 2, 2, 4, 3, 4, 9, 7, 7, 7, 3, 4, 4, 4, 6, 6, 5, 2, 3, 1, 1, 1, 1, 1, 1, 1, 1, 1, 1, 1, 1, 1, 4, 1, 7, 7, 1, 1, 1, 1, 1, 3, 2, 1, 1, 2, 2, 1, 1, 2, 5, 1, 5, 1, 2, 1, 1, 1, 1, 3, 1, 1, 8, 1, 1, 1, 1, 1, 2, 2, 1, 1, 2, 1, 2, 1, 3, 1, 3, 7, 7, 1, 7, 5, 1, 2, 1, 1, 1, 1, 1, 5, 2, 1, 1, 1, 1, 1, 3, 1, 2, 3, 6, 1, 1, 1, 1, 4, 1, 2, 1, 1, 1, 1, 1, 2, 2, 5, 3, 3, 1, 5, 1, 1, 1, 2, 1, 2, 7, 1, 2, 2, 1, 1, 1, 1, 1, 1, 1, 1, 1, 3, 2, 3, 4, 1, 3, 2, 4, 2, 6, 6, 6, 2, 2, 6, 1, 1, 1, 1, 1, 1, 5, 1, 2, 5, 5, 2, 2, 1, 9, 1, 3, 3, 1, 1, 1, 1, 1, 1, 4, 4, 1, 2, 4, 1, 2, 1, 1, 1, 8, 2, 2, 2, 1, 1, 1, 8, 1, 1, 1, 1, 1, 7, 1, 7, 1, 1, 1, 1, 7, 7, 1, 1, 6, 2, 3, 1, 1, 1, 6, 6, 2, 3, 6, 2, 6, 3, 1, 5, 1, 1, 1, 1, 1, 1, 5, 1, 1, 1, 1, 1, 1, 4, 4, 2, 1, 2, 4, 2, 2, 2, 2, 1, 1, 2, 2, 1, 1, 2, 2, 4, 4, 1, 2, 2, 2, 2, 1, 1, 4, 2, 1, 4, 2, 4, 4, 1, 3, 1, 1, 1, 1, 1, 1, 1, 1, 3, 1, 1, 1, 3, 3, 1, 3, 1, 1, 3, 3, 3, 1, 3, 1, 3, 1, 3, 3, 3, 3, 3, 3, 3, 1, 3, 1, 2, 1, 1, 1, 2, 1, 1, 1, 1, 2, 1, 2, 2, 2, 2, 1,

2, 1, 1, 2, 2, 2, 1, 1, 1, 2, 2, 1, 2, 2, 2, 2, 2, 1, 2, 1, 1, 1, 2, 1, 1, 1, 1, 1, 2, 2, 2, 1, 1, 1, 1, 2, 1, 1, 1, 2, 1, 2, 1, 1, 1, 1, 1, 1, 1, 1, 1, 1, 1, 1, 1, 1, 1, 1, 1, 1, 1, 1, 1, 1, 1, 1, 1, 1, 1, 1, 1, 1, 1, 1, 1, 1, 1, 1, 1, 1, 1, 1, 1, 1, 1, 1, 1, 1, 1, 1, 1, 1, 1, 1, 1, 1, 1, 1, 1, 1, 1, 1, 1

Corresponding number of selected TFs each gene is involved with:
(In the same order as above)
29, 27, 28, 28, 23, 25, 29, 28, 27, 27, 25, 29, 29, 24, 28, 20, 28, 28, 20, 27, 26, 25, 20, 24, 17, 29, 23, 28, 22, 27, 26, 25, 25, 25, 25, 23, 13, 29, 29, 17, 27, 27, 27, 20, 11, 25, 12, 23, 17, 17, 17, 11, 16, 15, 20, 10, 12, 29, 29, 29, 19, 8, 8, 28, 28, 9, 27, 18, 6, 18, 18, 13, 26, 13, 17, 25, 25, 25, 24, 24, 15, 22, 22, 11, 21, 10, 8, 20, 20, 13, 13, 19, 19, 19, 9, 12, 9, 4, 5, 5, 5, 11, 8, 8, 8, 5, 5, 6, 15, 10, 29, 29, 29, 29, 29, 29, 28, 28, 28, 28, 28, 28, 7, 28, 4, 4, 28, 27, 27, 27, 27, 9, 13, 26, 26, 13, 13, 26, 26, 13, 5, 25, 5, 25, 12, 24, 24, 24, 24, 8, 24, 24, 3, 24, 23, 23, 23, 22, 11, 11, 22, 22, 11, 22, 11, 22, 7, 21, 7, 3, 3, 21, 3, 4, 20, 10, 20, 20, 20, 20, 20, 4, 10, 19, 19, 19, 19, 19, 6, 18, 9, 6, 3, 17, 17, 17, 17, 4, 16, 8, 16, 16, 16, 16, 16, 8, 8, 3, 5, 5, 15, 3, 15, 15, 14, 7, 14, 7, 2, 14, 7, 7, 14, 14, 13, 13, 13, 13, 13, 12, 12, 4, 6, 4, 3, 12, 4, 6, 3, 6, 2, 2, 2, 6, 6, 2, 11, 11, 11, 11, 11, 10, 2, 10, 5, 2, 2, 5, 5, 10, 1, 9, 3, 3, 9, 9, 9, 9, 9, 8, 2, 2, 8, 4, 2, 8, 4, 8, 8, 8, 1, 4, 4, 4, 8, 8, 8, 1, 8, 8, 8, 7, 7, 1, 7, 1, 7, 7, 7, 7, 1, 1, 7, 6, 1, 3, 2, 6, 6, 6, 1, 1, 3, 2, 1, 3, 1, 2, 6, 1, 5, 5, 5, 5, 5, 5, 1, 5, 5, 5, 5, 5, 5, 1, 1, 2, 4, 2, 1, 2, 2, 2, 2, 4, 4, 2, 2, 4, 4, 2, 2, 1, 1, 4, 2, 2, 2, 2, 4, 4, 1, 2, 4, 1, 2, 1, 1, 4, 1, 3, 3, 3, 3, 3, 3, 3, 1, 3, 3, 3, 1, 1, 3, 1, 3, 3, 1, 1, 1, 3, 1, 3, 1, 3, 1, 1, 1, 1, 1, 1, 1, 3, 1, 2, 1, 2, 2, 2, 1, 2, 2, 2, 2, 1, 2, 1, 1, 1, 1, 2, 1, 2, 2, 1, 1, 1, 2, 2, 2, 1, 1, 2, 1, 1, 1, 1, 1, 2, 1, 2, 2, 2, 1, 2, 2, 2, 2, 2, 1, 1, 1, 2, 2, 2, 2, 1, 2, 2, 2, 1, 2, 1, 2, 1, 1, 1, 1, 1, 1, 1, 1, 1, 1, 1, 1, 1, 1, 1, 1, 1, 1, 1, 1, 1, 1, 1, 1, 1, 1, 1, 1, 1, 1, 1, 1, 1, 1, 1, 1, 1, 1, 1, 1, 1, 1, 1, 1, 1, 1, 1, 1, 1, 1, 1, 1, 1, 1, 1, 1, 1, 1, 1

Gene ontology terms ranked according to mean of effect sizes in cluster:
cell differentiation | T cell receptor complex | cell development | hemopoiesis | T cell activation | cell activation | T cell differentiation | immunological synapse | integrin activation

Corresponding mean of effect sizes of each Gene ontology term:
(In the same order as above)
2.59, 2.32, 2.29, 2.13, 2.04, 1.98, 1.96, 1.26, 0.96

Corresponding total number of genes of each Gene ontology term:
(In the same order as above)
871, 478, 507, 343, 287, 596, 172, 112, 155

Corresponding number of genes of each Gene ontology term with at least one TFBS (of selected TFs):
(In the same order as above)
291, 175, 189, 132, 111, 200, 73, 47, 53

Corresponding total number of TFBS (of selected TFs) for each Gene ontology term (in all genes):
(In the same order as above)
2943, 1710, 1896, 1447, 1118, 1877, 806, 463, 478

Corresponding number of selected TFs each Gene ontology term is involved with:
(In the same order as above)
29, 29, 29, 29, 29, 29, 29, 29, 29

TFs ranked according to mean of effect sizes in cluster:
V$NFAT_Q6, V$NFAT_Q4_01, V$HMGIY_Q6, V$DBP_Q6, V$CEBP_Q2_01, V$STAT6_01, V$TCF4_Q5, V$CEBP_01, V$PAX2_02, V$AP3_Q6, V$STAT5A_03, V$AREB6_04, V$CEBP_Q3, V$HOXA4_Q2, V$CEBPA_01, V$STAT5A_04, V$LEF1_Q2, V$SOX10_Q6, V$STAT1_03, V$STAT4_01, V$CEBP_Q2, V$BRCA_01, V$GATA_Q6, B$CRP_C, V$TITF1_Q3, V$TST1_01, V$GATA1_05, V$FAC1_01, V$GATA6_01

Corresponding mean of effect sizes of each TF:
(In the same order as above)

2.71, 2.71, 2.44, 2.41, 2.38, 2.33, 2.25, 2.23, 2.23, 2.17, 2.13, 2.12, 2.12, 2.08, 2.08, 2.07, 2.04, 2.00, 1.97, 1.95, 1.93, 1.54, 1.49, 1.25, 1.25, 1.24, 1.18, 1.14, 1.06

Corresponding total number of TFBS for each TF (genome-wide):
(In the same order as above)
719, 736, 753, 714, 716, 732, 716, 740, 741, 741, 749, 740, 744, 719, 711, 740, 723, 729, 751, 743, 718, 730, 703, 716, 707, 730, 715, 722, 722

Corresponding total number of TFBS for each TF (in all genes in selected Gene ontology terms):
(In the same order as above)
505, 498, 480, 444, 444, 471, 473, 459, 480, 428, 460, 442, 476, 437, 437, 428, 470, 429, 440, 462, 428, 397, 399, 376, 378, 406, 421, 377, 393

Corresponding number of genes (of selected Gene ontology terms) each TF is involved with:
(In the same order as above)
183, 194, 179, 168, 173, 181, 183, 174, 185, 171, 180, 170, 178, 166, 176, 171, 178, 167, 174, 181, 165, 160, 144, 147, 147, 168, 154, 151, 156

Corresponding number of selected Gene ontology terms each TF is involved with:
(In the same order as above)
9, 9, 9, 9, 9, 9, 9, 9, 9, 9, 9, 9, 9, 9, 9, 9, 9, 9, 9, 9, 9, 9, 9, 9, 9, 9, 9, 9, 9

Cluster for columns 1296 to 1311, rows 97 to 108

Gene ontology terms

gene expression | DBD domain binding | DNA binding | chordate embryonic development | embryonic development | limb development | central nervous system development | central nervous system neuron development | gastrulation | somitogenesis | anatomical structure morphogenesis | organ development | neurogenesis | regionalization | cell fate determination | segmentation

TFs

V$STAT6_01 | V$STAT5A_03 | V$STAT4_01 | V$STAT5A_04 | V$STAT1_03 | V$CEBP_Q3 | V$PAX2_02 | V$HMGIY_Q6 | V$HOXA4_Q2 | V$TST1_01 | V$NFAT_Q6 | V$NFAT_Q4_01

Information

All related TFs:
(List of all TFs that are related to any of the PWMs)
ANF-2, C/EBP, C/EBPalpha, C/EBPalpha(p20), C/EBPalpha(p30), C/EBPbeta, C/EBPbeta(LAP), C/EBPbeta(p20), C/EBPbeta(p34), C/EBPbeta(p35), C/EBPdelta, C/EBPepsilon, C/EBPgamma, HMG, HMG-Y, HMGI-C, HOXA4, NF-AT, NF-AT1, NF-AT1C, NF-AT2, NF-AT3, NF-AT4, POU3F1, Pax-2, Pax-2.1, Pax-2.2, Pax-2a, Pax-2b, STAT1, STAT1alpha, STAT4, STAT5A, STAT6

Ranked gene list:
(All genes of the selected Gene ontology terms with hits of any of the selected TFs, ranked according to the total number of TFBS)
LBX1, PAX6, LEF1, WNT1, HAND2, DLL1, RUNX1, FGF8, CTNNB1, NR2F1, HOXA9, HES1, ID2, DLX2, NR4A2, HOXA7, CXCR4, LHX1, TFAP2A, HOXD13, HMX3, VIM, RARG, HOXB7, HOXA11, HOXA10, ETS1, EOMES, EGR2, CDC25C, HOXA1, NKX2-2, ID3, GLI1, EPHA4, RARA, PRDM1, POU4F1, FLI1, DLX1, RBPJ, NEUROG2, CD44, PTCH1, IL23A, HOXA13, RUNX3, NR3C1, JARID2, MEIS2, TCF7, SOX2, SMAD7, NFIA, LMO4, HOXC8, HOXC6, HOXB5, HOXB4,

HOXB3, HMX2, GATA3, CDKN1B, CCND2, BRD2, HOXA5, EVX2, TLE3, SSSCA1, SMARCA2, LDB1, HSPA8, HOXB6, HOXB2, CITED2, BHLHB2, TGFBR2, NR2F2, NFATC1, JUN, CDK5, ZFP36L2, ZEB2, TIPARP, THRA, TGIF1, NFATC3, MXI1, MEF2D, LFNG, KLF6, IRF1, HOXB8, HIST1H4I, HES7, GSK3B, GFI1, EGR1, DAAM1, CSK, CDK4, CDK2, PROX1, GDF11, STAT1, SIN3A, SHC1, RXRB, NR3C2, MBNL1, MAP3K14, LHX9, ETV6, BMI1, STAT3, NFYA, NFE2L1, MYB, HOXA2, ETV3, COL11A2, BCL2, ZIC2, WNT10B, TAF8, SMAD3, FOXD3, ETV1, ANXA6, AKT1, JUNB, PDLIM5, PAX3, MAF, HOXB9, FGF9, ZBTB7B, USF1, TNF, SIX1, SATB1, RREB1, PPP1R1B, NR4A3, NFKBIA, MYC, MLL, MBNL2, LTB, LTA, IRX5, IRF2, IKZF1, HOXC9, HOXC5, HOXC4, HEY2, H3F3B, FST, FOXP1, FOXG1, EBF1, CISH, BUB3, ARID1A, ARHGEF2, ZHX2, UBE2B, OTX2, OSR1, NAB2, KLF7, FOXJ2, FOSL2, CHD3, CFL1, CDKN2C, CCR7, BCL2L1, BACH2, ZIC1, ZEB1, VEGFA, PITX2, FLJ32987, E2F3, SSBP3, SOX4, PURA, NFKBIE, MBD1, MAP2K3, KLF9, ILK, HSPA1B, HOXD11, HMGA1, HIVEP1, GATA2, DUSP6, DDX5, CREB1, CDC42SE1, ACVR1, VDR, SOS1, SKP1, MNT, MIER1, MAZ, MARK2, LCP1, HOXA3, HNRNPA1, HIST4H4, CBX5, ARNTL, ADNP, SOCS1, S100A10, PIK3R1, PCNA, NEDD9, HOXD8, BCL2L11, BATF, STAT5A, SPRY1, PSMC5, NFYC, NDRG1, UBTF, TNFRSF1A, PAX5, MAP3K1, GDNF, DDIT3, CYP27B1, CDKN1A, ADAMTS6, ZFP36L1, ZFP36, VEGFB, TUSC2, TRERF1, TNFAIP3, TAP1, SRF, SHOX2, SFMBT2, S100A6, RORC, RGMB, RERE, PTMA, PTGER4, PSMB8, PSENEN, POU3F2, PITX3, PHF20, PER1, PDCD4, PAX2, OSM, NR1D1, NNAT, NIN, MZF1, MYL6, MLL2, MGAT1, MAPKAPK3, MAP4K4, MAP3K12, KLF12, JMJD3, IRS1, HOXD12, HNRNPC, HIVEP2, HDAC7, FYN, FOXN3, EVL, DUSP1, DLX6, DKFZp779C0757, CYP26B1, CYFIP2, CUGBP2, CHST11, CDK6, CAP1, BTG1, BCL6, BCL11B, ATXN1, ARID5B, ALDOA, AGER, TNIP1, RING1, RASSF1, PSMB9, PHF12, MVP, IL7R, HOXC10, GBF1, DCTN3, CRTC2, CD4, BCL9L, ATP1A1, AGPAT1, TRIB2, TNFAIP8, SLCO3A1, SGK1, SATB2, PSMP, PPOX, POLR2A, PBXIP1, OTP, OSR2, MYNN, MTA2, MLLT11, LRRFIP1, ITGB1, HOXA6, HNRNPL, FMNL1, EFNB2, DVL2, DBP, CRIP1, CKS1B, CD68, CBX4, CASP2, BMF, ANP32A, ANG, ADM, ADD3, TTF2, TAP2, TAGLN2, SUFU, SLC39A1, SLC38A2, SILV, SHMT2, S100A4, LAPTM5, ITPR1, IKBKE, IGF1R, GSK3A, GPS2, ESR1, ENO3, EFNA1, DYRK2, DYRK1A, DUSP5, CCND3, CBX3, BCOR, BCL11A, ARHGDIB, ADIPOR1, WDR1, WBP7, UNCX, TPP1, SOCS3, SLC4A2, S1PR1, RPL19, RHOC, RAB13, POU2F1, PAX9, NP, MGAT5, MAX, LASP1, IL16, HYAL2, HOXD10, HOXA4, GTF3A, GIT1, GABPB1, ENC1, ELK3, EGLN2, CYTIP, CUL1, CTLA4, CTDSP2, CLCN3, CD3D, BAK1, ARID1B, APOA2, WIPF1, VAMP8, UBC, TRIM3, TRAF3, SOCS2, SNAPC5, SMAD4, RPS18, REPIN1, POU3F3, OTX1, LRDD, HSP90B1, GAD1, EFNA3, DAXX, CSF1, CIRBP, BAT1, ARHGAP5, ZC3H12D, ZC3H12A, UGP2, TP53, TLE4, TGFB3, TCF7L2, SNRK, SLC20A1, RTN4, RPS6KA1, REV3L, RB1, PRKCE, PEA15, NR4A1, NF1, MEIS1, MAPRE2, MAL, LCK, KAT2B, H2AFZ, H2AFJ, GGPS1, GABBR1, FUS, FOXO1, FOXN2, FHOD1, FHL3, FBRS, ETV5, EP300, EHMT2, DPH1, DAD1, COL2A1, CEBPE, CDC42EP3, C2orf28, ABI3, ABCF3, ZNF384, TPM3, TG, TBPL1, POLD4, PLEC1, PDGFRA, PDE4B, PBX2, NR2E1, MGA, LPIN1, HIST1H1E, HIST1H1A, HIF1AN, HCLS1, GAPDH, EMP3, ELF5, EIF5A, DCTD, CCND1, CAMK2G, CACNB3, ALCAM, ADAMTS4, ACVR2A, ABCD2, ZAP70, UBL3, TWIST1, TUBB, TRADD, TPT1, TBX15, TBL1XR1, SYTL1, STAT6, SIM1, SEMA3B, SART3, QKI, PXN, PTGER2, PIK3CG, PIK3CA, PCGF2, NRGN, MYLIP, MYD88, MT2A, MSI2, MAPK14, KLF2, JAK1, IL6R, IL2RA, IER2, HSPA1A, HOXD3, HOXC11, HNRNPH1, GRB2, GRAP2, FOS, ERBB2IP, ENO2, ELF1, E2F4, E2F2, DUSP10, DLG4, DHH, CXCR5, CREB3, CD79A, CD63, CADM1, ATP1B1, APEX1, ADORA2A, XAB2, WNK1, UCP2, TXNIP, STAT5B, SP4, SOX13, SMEK2, SLC25A22, PTK2B, PPARA, POU2AF1, PML, PHB2, PELP1, PDCD10, PCP2, PCDH17, PARP1, ORC4L, NUMB, NFAT5, MAT2B, LGALS1, LCP2, KRIT1, ITGA6, IRF8, ING1, IKZF3, IDI1, HSP90AB1, HNRNPK, HECA, HDGF, GIT2, FOXP4, FOXO3, FOSB, ERGIC2, EIF4A1, EDAR, DR1, CFLAR, CD82, CD40, CCNL1, CAMK2D, BHLHB3, BCLAF1, ATF2, ZYX, ZNF274, ZIC4, ZFP161, YWHAZ, VGF, TSC22D1, TRAF1, TOB1, TGFBR3, TBK1, TAF12, STK38L, SSRP1, SSBP1, SRI, SLC26A8, SIL1, SETD1A, SESN1, SERPINI1, SEPT7, SEPHS1, RGS10, RAF1, PRG4, PPP3R1, POFUT1, PLAGL2, PHIP, PEG10, PDGFB, PCSK7, OTUD4, NR1H2, NOS2, NFIL3, NBPF1, MLC-B, MED21, MCAM, MAPKAPK2, MAP3K5, LIG4, KLF5, KLF13, JARID1B, IVNS1ABP, ISYNA1, IMPDH2, IL2, IGF2BP3, HSPE1, HSPD1, HS2ST1, HNRNPD, HIPK3, HIC1, GNB2L1, FRAS1, FBXW11, FAM110A, EIF4A2, EFNA4, DEDD, CXCR7, CHIC2, CD69, CD47, CD27, CALR, CALM2, CALM1, BTAF1, BRCA2, BACH1, ATF4, AQP3, AHSA1, ABHD2, ABCG1, ABCF2, ZNF260, YES1, WWP1, WASF1, USO1, UEVLD, TOX, TNFSF8, TNFRSF1B, TMSB4X, TGFBRAP1, TAX1BP1, ST6GAL1, SSR1, SRCAP, SPTBN1, SPRY4, SNRPA,

SIPA1, SFRS2IP, SEP15, SELPLG, RPS9, RPL4, RPL37, RPL28, ROCK1, RNF24, RGS16, RBM12, PUM1, PTTG1, PTGES3, PRMT1, PRKCD, PRCP, PPARGC1A, PNKD, PKNOX2, PHLDB1, PFKFB2, PER2, PARP14, OPTN, NUP37, NRN1, NCOA4, NAB1, MRPS6, MIA, MAPK13, MAP3K8, MAP2K6, LTBP2, LRBA, LMNA, LLGL2, LDLR, KREMEN2, KRAS, ITPR2, ITGA1, IMP3, IL10RA, IGF1, IFNGR1, IFITM2, IFITM1, IFIH1, HYAL3, HS6ST1, HNRNPU, HMGN3, HCRT, H3F3A, GSX1, GPR183, GLIPR1, FUBP1, FBL, FAM102A, EXOSC7, ESRRA, EPB41, EEF1A1, ECE1, DUSP4, DOCK10, DNAJC14, DDX42, DDX1, DDIT4, DBF4, CST6, CSRP1, CSNK1A1, CRIP2, CPT1A, CPNE1, CNTFR, CLDN12, CHD6, CEP57, CDC25B, CD53, CD200R1, CCR6, CBX1, CAD, C7orf23, BRAF, BBC3, BANF1, ATP5G2, ATP5B, ASXL1, ARMCX2, ANXA1, ADIPOR2, ACSL5

Corresponding total number of TFBS:
(For each gene listed above, the total number of TFBS for any of the selected TFs, multiplied by the number of selected Gene ontology terms containing that gene)
176, 132, 132, 121, 110, 110, 108, 108, 104, 99, 96, 90, 88, 88, 84, 84, 84, 80, 77, 77, 77, 72, 72, 72, 72, 72, 72, 72, 72, 72, 70, 66, 66, 66, 64, 63, 60, 60, 60, 60, 56, 55, 55, 54, 54, 54, 50, 50, 50, 49, 48, 48, 48, 48, 48, 48, 48, 48, 48, 48, 48, 48, 48, 48, 48, 45, 45, 44, 44, 44, 44, 44, 44, 44, 44, 44, 42, 42, 40, 40, 40, 36, 36, 36, 36, 36, 36, 36, 36, 36, 36, 36, 36, 36, 36, 36, 36, 36, 36, 36, 36, 36, 35, 35, 33, 33, 33, 33, 33, 33, 33, 33, 33, 33, 30, 30, 30, 30, 30, 30, 30, 30, 28, 28, 28, 28, 28, 28, 28, 28, 27, 26, 26, 25, 25, 25, 24, 24, 24, 24, 24, 24, 24, 24, 24, 24, 24, 24, 24, 24, 24, 24, 24, 24, 24, 24, 24, 24, 24, 24, 24, 24, 24, 24, 24, 24, 22, 22, 22, 22, 22, 22, 22, 22, 22, 22, 22, 22, 22, 22, 21, 21, 21, 21, 21, 21, 20, 20, 20, 20, 20, 20, 20, 20, 20, 20, 20, 20, 20, 20, 20, 20, 20, 20, 18, 18, 18, 18, 18, 18, 18, 18, 18, 18, 18, 18, 18, 16, 16, 16, 16, 16, 16, 16, 16, 15, 15, 15, 15, 15, 14, 14, 14, 14, 14, 14, 14, 14, 12, 12, 12, 12, 12, 12, 12, 12, 12, 12, 12, 12, 12, 12, 12, 12, 12, 12, 12, 12, 12, 12, 12, 12, 12, 12, 12, 12, 12, 12, 12, 12, 12, 12, 12, 12, 12, 12, 12, 12, 12, 12, 12, 12, 12, 12, 12, 12, 12, 12, 12, 12, 12, 12, 12, 12, 11, 11, 11, 11, 11, 11, 11, 11, 11, 11, 11, 11, 11, 11, 11, 10, 10, 10, 10, 10, 10, 10, 10, 10, 10, 10, 10, 10, 10, 10, 10, 10, 10, 10, 10, 10, 10, 10, 10, 10, 10, 10, 10, 10, 10, 9, 9, 9, 9, 9, 9, 9, 9, 9, 9, 9, 9, 9, 9, 9, 9, 9, 9, 9, 9, 9, 9, 9, 9, 9, 9, 9, 8, 8, 8, 8, 8, 8, 8, 8, 8, 8, 8, 8, 8, 8, 8, 8, 8, 8, 8, 8, 8, 8, 8, 8, 8, 8, 8, 8, 8, 8, 8, 7, 7, 7, 7, 7, 7, 7, 7, 7, 7, 7, 7, 7, 7, 7, 7, 7, 7, 7, 7, 7, 6, 6, 6, 6, 6, 6, 6, 6, 6, 6, 6, 6, 6, 6, 6, 6, 6, 6, 6, 6, 6, 6, 6, 6, 6, 6, 6, 6, 6, 6, 6, 6, 6, 6, 6, 6, 6, 6, 6, 6, 6, 6, 6, 5, 5, 5, 5, 5, 5, 5, 5, 5, 5, 5, 5, 5, 5, 5, 5, 5, 5, 5, 5, 5, 5, 5, 5, 5, 5, 5, 4, 4, 4, 4, 4, 4, 4, 4, 4, 4, 4, 4, 4, 4, 4, 4, 4, 4, 4, 4, 4, 4, 4, 4, 4, 4, 4, 4, 4, 4, 4, 4, 4, 4, 4, 4, 4, 4, 4, 4, 4, 4, 4, 4, 4, 4, 4, 4, 4, 4, 4, 4, 4, 4, 4, 4, 4, 4, 4, 3, 3, 3, 3, 3, 3, 3, 3, 3, 3, 3, 3, 3, 3, 3, 3, 3, 3, 3, 3, 3, 3, 3, 3, 3, 3, 3, 3, 3, 3, 3, 3, 3, 3, 3, 3, 3, 3, 3, 3, 3, 3, 3, 3, 3, 3, 3, 3, 3, 3, 3, 2, 2, 2, 2, 2, 2, 2, 2, 2, 2, 2, 2, 2, 2, 2, 2, 2, 2, 2, 2, 2, 2, 2, 2, 2, 2, 2, 2, 2, 2, 2, 2, 2, 2, 2, 2, 2, 2, 2, 2, 2, 2, 2, 2, 2, 2, 2, 2, 2, 2, 2, 2, 2, 2, 2, 2, 2, 2, 2, 2, 2, 2, 2, 2, 2, 2, 2, 2, 2, 2, 2, 2, 2, 2, 2, 2, 2, 2, 2, 2, 2, 2, 2, 2, 2, 1

Corresponding number of selected Gene ontology terms each gene is involved with:
(In the same order as above)
16, 11, 11, 11, 10, 10, 9, 12, 13, 9, 8, 10, 8, 8, 7, 7, 7, 8, 7, 7, 7, 6, 6, 6, 6, 6, 6, 6, 8, 6, 7, 6, 6, 11, 8, 9, 5, 6, 5, 6, 8, 5, 5, 6, 9, 6, 5, 5, 5, 7, 4, 12, 4, 4, 4, 4, 4, 4, 4, 4, 4, 4, 4, 4, 4, 5, 5, 4, 4, 4, 4, 4, 4, 4, 4, 4, 6, 7, 5, 10, 5, 3, 3, 4, 3, 3, 3, 3, 3, 9, 3, 3, 4, 3, 4, 4, 3, 4, 4, 3, 3, 4, 7, 5, 3, 3, 3, 3, 3, 3, 3, 3, 3, 3, 6, 3, 3, 6, 5, 3, 3, 6, 4, 4, 4, 7, 4, 7, 4, 7, 3, 13, 13, 5, 5, 5, 2, 3, 2, 6, 2, 2, 2, 2, 2, 8, 2, 2, 2, 2, 4, 3, 2, 2, 2, 2, 6, 2, 6, 2, 6, 3, 2, 2, 2, 2, 2, 2, 11, 2, 2, 2, 2, 2, 2, 2, 2, 2, 2, 2, 7, 3, 7, 7, 3, 3, 2, 5, 2, 2, 4, 2, 2, 2, 2, 4, 4, 2, 4, 2, 2, 5, 2, 5, 3, 6, 3, 2, 9, 2, 2, 2, 3, 2, 3, 2, 2, 3, 2, 2, 2, 8, 2, 2, 2, 2, 3, 3, 3, 3, 3, 2, 2, 2, 2, 7, 2, 2, 7, 2, 1, 1, 3, 1, 1, 1, 1, 6, 1, 1, 1, 1, 1, 2, 1, 1, 1, 2, 3, 1, 3, 1, 1, 12, 4, 1, 1, 1, 3, 1, 1, 1, 1, 3, 1, 1, 1, 1, 3, 4, 1, 1, 1, 1, 2, 1, 4, 2, 4, 1, 1, 1, 1, 2, 1, 1, 1, 1, 1, 2, 1, 1, 1, 1, 1, 1, 1, 1, 1, 1, 1, 1, 1, 1, 1, 1, 1, 1, 1, 2, 1, 1, 1, 1, 2, 1, 1, 1, 1, 5, 1, 1, 1, 5, 2, 2, 1, 1, 1, 1, 2, 1, 1, 1, 1, 1, 9, 1, 1, 3, 1, 1, 1, 1, 1, 1, 1, 1, 3, 1, 1, 3, 1, 3, 1, 3, 1, 1, 1, 1, 1, 1, 3, 2, 2, 4, 2, 2, 1, 1, 1, 1, 2, 4, 8, 1, 1, 2, 1, 1, 2, 4, 2, 8, 1, 2, 1, 1, 2, 1, 2, 1, 1, 1, 1, 2, 2, 2, 1, 1, 7, 1, 1, 1, 1, 7, 1, 1, 1, 7, 1, 1, 1, 1, 1, 1, 1, 1, 1, 1, 1, 1, 6, 6, 3, 6, 1, 6, 1, 3, 2, 6, 1, 3, 3, 3, 6, 1, 1, 2, 3, 2, 1, 1, 1, 3, 3, 2, 1, 1, 1, 3, 6, 3, 2, 2, 6, 3, 1, 2, 3, 1, 1, 5, 1, 5, 1, 1, 5, 1, 5, 5, 1, 1, 1, 1, 1, 1, 1, 1, 5, 1, 1, 5, 1, 1, 1, 1, 5, 1, 1, 1, 4, 1, 2, 1, 2, 1, 4, 2, 4, 4, 1, 1, 4, 1, 4, 4, 1, 1, 2, 2, 2, 1, 2, 2, 2, 2, 1, 1, 2, 4, 2, 1, 2, 1, 4, 1, 1, 2, 2, 2, 1, 1, 4, 2, 2, 1, 1, 1, 1, 2, 1, 1, 3, 1, 1, 3, 3, 3, 1, 1, 1, 3, 3, 3, 1, 3, 1, 1, 3, 3, 1, 1, 3, 1, 3, 1, 3, 3, 3, 1, 1, 1, 1, 3, 1, 3, 1, 1, 3, 3, 3, 1, 3, 3, 1, 1, 3, 1, 1, 3, 1, 3, 1, 2, 1, 1, 1, 2, 1, 2, 1, 1, 2, 2, 2, 2, 2, 1, 1, 1, 1, 1, 1, 2, 1, 1, 2, 1, 1, 1, 1, 2, 1, 1, 1, 1, 2, 2, 2, 2, 1, 2, 1, 1, 2, 2, 2, 2, 1, 1, 2, 1, 2, 1, 2, 2, 2, 1, 1, 1, 1, 2,

2, 1, 1, 1, 2, 1, 2, 1, 1, 1, 1, 1, 1, 2, 2, 2, 2, 1, 2, 1

Corresponding number of selected TFs each gene is involved with:
(In the same order as above)
11, 12, 12, 11, 11, 11, 12, 9, 8, 11, 12, 9, 11, 11, 12, 12, 12, 10, 11, 11, 11, 12, 12, 12, 12, 12, 12, 12, 9, 12, 10, 11, 11, 6, 8, 7, 12, 10, 12, 10, 7, 11, 11, 9, 6, 9, 10, 10, 10, 7, 12, 4, 12, 12, 12, 12, 12, 12, 12, 12, 12, 12, 12, 12, 12, 9, 9, 11, 11, 11, 11, 11, 11, 11, 11, 11, 7, 6, 8, 4, 8, 12, 12, 9, 12, 12, 12, 12, 12, 4, 12, 12, 9, 12, 9, 9, 12, 9, 9, 12, 12, 9, 5, 7, 11, 11, 11, 11, 11, 11, 11, 11, 11, 11, 5, 10, 10, 5, 6, 10, 10, 5, 7, 7, 7, 4, 7, 4, 7, 4, 9, 2, 2, 5, 5, 5, 12, 8, 12, 4, 12, 12, 12, 12, 12, 3, 12, 12, 12, 12, 6, 8, 12, 12, 12, 12, 4, 12, 4, 12, 4, 8, 12, 12, 12, 12, 11, 11, 2, 11, 11, 11, 11, 11, 11, 11, 11, 11, 11, 11, 3, 7, 3, 3, 7, 7, 10, 4, 10, 10, 5, 10, 10, 10, 10, 5, 5, 10, 5, 10, 10, 4, 10, 4, 6, 3, 6, 9, 2, 9, 9, 9, 6, 9, 6, 9, 9, 6, 8, 8, 8, 2, 8, 8, 8, 8, 5, 5, 5, 5, 5, 7, 7, 7, 7, 2, 7, 7, 2, 7, 12, 12, 4, 12, 12, 12, 12, 2, 12, 12, 12, 12, 12, 6, 12, 12, 12, 6, 4, 12, 4, 12, 12, 1, 3, 12, 12, 12, 4, 12, 12, 12, 12, 4, 12, 12, 12, 12, 4, 3, 12, 12, 12, 12, 6, 12, 3, 6, 3, 12, 12, 12, 12, 6, 12, 12, 12, 12, 12, 6, 12, 11, 11, 11, 11, 11, 11, 11, 11, 11, 11, 11, 11, 11, 11, 10, 10, 10, 10, 10, 5, 10, 10, 10, 10, 5, 10, 10, 10, 10, 2, 10, 10, 10, 2, 5, 5, 10, 10, 10, 10, 5, 10, 10, 10, 10, 10, 1, 9, 9, 3, 9, 9, 9, 9, 9, 9, 9, 9, 3, 9, 9, 3, 9, 3, 9, 3, 9, 9, 9, 9, 9, 9, 3, 4, 4, 2, 4, 4, 8, 8, 8, 8, 4, 2, 1, 8, 8, 4, 8, 8, 4, 2, 4, 1, 8, 4, 8, 8, 4, 8, 4, 8, 8, 8, 8, 4, 4, 4, 7, 7, 1, 7, 7, 7, 7, 1, 7, 7, 7, 1, 7, 7, 7, 7, 7, 7, 7, 7, 7, 6, 6, 6, 1, 1, 2, 1, 6, 1, 6, 2, 3, 1, 6, 2, 2, 2, 1, 6, 6, 3, 2, 3, 6, 6, 6, 2, 2, 3, 6, 6, 6, 2, 1, 2, 3, 3, 1, 2, 6, 3, 2, 6, 5, 1, 5, 1, 5, 5, 1, 5, 1, 1, 5, 5, 5, 5, 5, 5, 5, 5, 1, 5, 5, 1, 5, 5, 5, 5, 1, 5, 4, 4, 1, 4, 2, 4, 2, 4, 1, 2, 1, 1, 4, 4, 1, 4, 1, 1, 4, 4, 2, 2, 2, 4, 2, 2, 2, 2, 4, 4, 2, 1, 2, 4, 2, 4, 1, 4, 4, 2, 2, 2, 4, 4, 1, 2, 2, 4, 4, 4, 4, 2, 4, 3, 1, 3, 3, 1, 1, 1, 3, 3, 3, 1, 1, 1, 3, 1, 3, 3, 1, 1, 3, 3, 1, 3, 1, 3, 1, 1, 1, 3, 3, 3, 3, 1, 3, 1, 3, 3, 1, 1, 1, 3, 1, 1, 3, 3, 1, 3, 3, 1, 3, 1, 2, 1, 2, 2, 2, 1, 2, 1, 2, 2, 1, 1, 1, 1, 1, 2, 2, 2, 2, 2, 2, 1, 2, 2, 1, 2, 2, 2, 2, 1, 2, 2, 2, 2, 1, 1, 1, 1, 2, 1, 2, 2, 1, 1, 1, 1, 2, 2, 1, 2, 1, 2, 1, 1, 1, 2, 2, 2, 2, 1, 1, 2, 2, 2, 1, 2, 1, 2, 2, 2, 2, 2, 2, 1, 1, 1, 1, 2, 1, 2, 2, 2, 1

Gene ontology terms ranked according to mean of effect sizes in cluster:
organ development | DNA binding | gene expression | embryonic development | limb development | segmentation | chordate embryonic development | regionalization | anatomical structure morphogenesis | cell fate determination | neurogenesis | DBD domain binding | central nervous system development | central nervous system neuron development | gastrulation | somitogenesis

Corresponding mean of effect sizes of each Gene ontology term:
(In the same order as above)
3.05, 2.74, 2.56, 2.51, 2.50, 2.32, 2.29, 2.22, 2.13, 2.06, 1.83, 1.63, 1.46, 1.44, 1.32, 0.97

Corresponding total number of genes of each Gene ontology term:
(In the same order as above)
339, 1.2K, 3.8K, 1.2K, 129, 109, 671, 62, 583, 145, 409, 411, 110, 37, 293, 116

Corresponding number of genes of each Gene ontology term with at least one TFBS (of selected TFs):
(In the same order as above)
89, 281, 679, 250, 30, 35, 146, 25, 120, 42, 94, 111, 24, 9, 60, 26

Corresponding total number of TFBS (of selected TFs) for each Gene ontology term (in all genes):
(In the same order as above)
577, 1627, 3825, 1536, 212, 255, 930, 172, 749, 292, 579, 597, 179, 77, 376, 165

Corresponding number of selected TFs each Gene ontology term is involved with:
(In the same order as above)
12, 12, 12, 12, 12, 12, 12, 12, 12, 12, 12, 12, 12, 12, 12, 12

TFs ranked according to mean of effect sizes in cluster:
V$HMGIY_Q6, V$PAX2_02, V$STAT1_03, V$STAT6_01, V$TST1_01, V$STAT5A_03, V$STAT4_01, V$STAT5A_04, V$HOXA4_Q2, V$NFAT_Q4_01, V$NFAT_Q6, V$CEBP_Q3

Corresponding mean of effect sizes of each TF:
(In the same order as above)
2.82, 2.65, 2.55, 2.44, 2.39, 2.18, 2.01, 1.96, 1.62, 1.61, 1.58, 0.96

Corresponding total number of TFBS for each TF (genome-wide):
(In the same order as above)
753, 741, 751, 732, 730, 749, 743, 740, 719, 736, 719, 744

Corresponding total number of TFBS for each TF (in all genes in selected Gene ontology terms):
(In the same order as above)
1092, 1096, 1023, 1051, 1031, 1020, 1026, 940, 949, 981, 1011, 928

Corresponding number of genes (of selected Gene ontology terms) each TF is involved with:
(In the same order as above)
388, 387, 368, 390, 370, 380, 384, 359, 368, 371, 373, 348

Corresponding number of selected Gene ontology terms each TF is involved with:
(In the same order as above)
16, 16, 16, 16, 16, 16, 16, 16, 16, 16, 16, 16

Cluster for columns 2 to 4, rows 267 to 277

Gene ontology terms

Hedgehog signaling complex | hemopoiesis | cell differentiation

TFs

V$ETF_Q6 | V$E2F_Q2 | V$E2F1_Q6 | V$E2F1_Q3 | V$AP2GAMMA_01 | V$AP2ALPHA_01 | V$AP2_Q6_01 | V$AP2_Q6 | V$WT1_Q6 | V$EGR_Q6 | V$MAZ_Q6

Information

All related TFs:
(List of all TFs that are related to any of the PWMs)
AP-2, AP-2alpha, AP-2alphaA, AP-2alphaB, AP-2beta, AP-2gamma, DP-1, E2F, E2F+E4, E2F-1, E2F-3a, E2F-4, ETF, Egr-1, Egr-2, Egr-3, MAZ, WT1, WT1-del2

Ranked gene list:
(All genes of the selected Gene ontology terms with hits of any of the selected TFs, ranked according to the total number of TFBS)
SSSCA1, NOTCH1, GATA2, FASN, HOXA9, GFI1, LBX1, ZFPM1, SH2B3, AKT1, HOXA10, SOCS3, SMAD7, ID2, HES1, CCND1, BMI1, PDGFA, HBA2, FLII, IL11, TGFB1, TCF3, SOCS2, RBPJ, PTPN6, MAFK, IRF1, EGR1, ATXN1, VEGFA, RUNX3, JUND, CREBBP, CDKN1B, TCF7, STRA13, PTMA, PLEKHG2, PAX6, NKX2-2, NFATC1, MDK, MAPK12, MAFG, JUNB, JARID2, IRS1, IGF1R, HRAS, HOXC9, GATA3, DNMT3A, CRIP2, CRIP1, CFL1, CDKN1C, CDK5, PSMA7, NR3C2, MAFA, LEF1, HES5, FGF9, DLL1, CISH, CDKN2C, CD68, WNT10B, TNFRSF4, SOS1, NR4A2, LDB1, FLJ32987, CYP26B1, ACTB, UBE2I, RING1, RARA, PAX5, LYL1, HOXA7, FLI1, CCND2,

CBFA2T3, SP3, RORC, PHF13, MLL, ING1, FOSL2, E2F4, DLL4, BCL6, YY1, WNT7B, TCIRG1, SOCS1, SHC1, RARG, PRKCE, POU3F2, PAFAH1B1, NGFR, MYB, MLL5, MAP3K12, LMO4, HMGB1, GGNBP2, FOXO1, EGFL7, CUL4A, BACH2, TRIM28, TRAF3, SETD1A, RRBP1, RPS6KB2, LAG3, HOXB6, CDK6, RUNX2, NOL3, LHX3, ITGA5, IL17C, IGF2, IDUA, HSP90AB1, HHEX, FSTL3, CREB1, CORO1A, CLEC11A, CDK4, CD81, ZBTB16, WNT4, UBL4A, TNFSF13, STAT5A, SRC, PRKCZ, PKNOX1, PCNA, PCGF2, PARD6A, MCAM, IRF2, ID3, HOXB4, HOXA11, HES6, EZH2, CDKN1A, CDK5R1, ARHGEF1, AMH, ZNF385A, TSPAN32, TNFRSF13C, TFRC, TFAP2A, TCF12, STMN1, SMAD3, SLC20A1, SDC3, S1PR2, RHOA, PPARA, PIM1, OSM, NDRG2, MYST3, MUC1, LEPREL2, IRF6, IRF4, IKZF1, HOXC6, HOXC5, HOXC4, HMBS, H3F3A, GLI1, GADD45B, GAB2, EP300, EOMES, E2F2, CRABP2, CEBPB, CDKN2D, CBFB, C2orf28, C21orf33, BHLHB2, ACVR1, VIM, TMEM201, SMARCA4, SMAD2, SFTPC, ROBLD3, RBL1, PRKCA, PDGFRA, PBX2, PAX2, OTX1, ONECUT1, ODC1, NFIA, NEUROG2, MAPK7, LHX9, LEPR, IL17D, IL11RA, HOXA1, HIST1H4I, FOS, FBXW7, ENO2, CDK2, CCNY

Corresponding total number of TFBS:
(For each gene listed above, the total number of TFBS for any of the selected TFs, multiplied by the number of selected Gene ontology terms containing that gene)
33, 33, 33, 33, 30, 30, 27, 22, 22, 22, 21, 20, 20, 20, 20, 20, 20, 16, 16, 16, 15, 14, 14, 14, 14, 14, 14, 14, 14, 14, 12, 12, 12, 12, 12, 11, 11, 11, 11, 11, 11, 11, 11, 11, 11, 11, 11, 11, 11, 11, 11, 11, 11, 11, 11, 11, 11, 10, 10, 10, 10, 10, 10, 10, 10, 10, 10, 9, 9, 9, 9, 9, 9, 9, 9, 8, 8, 8, 8, 8, 8, 8, 8, 8, 7, 7, 7, 7, 7, 7, 7, 7, 7, 6, 6, 6, 6, 6, 6, 6, 6, 6, 6, 6, 6, 6, 6, 6, 6, 6, 6, 6, 5, 5, 5, 5, 5, 5, 5, 5, 4, 4, 4, 4, 4, 4, 4, 4, 4, 4, 4, 4, 4, 4, 4, 3, 3, 3, 3, 3, 3, 3, 3, 3, 3, 3, 3, 3, 3, 3, 3, 3, 3, 3, 3, 3, 3, 3, 2, 2, 2, 2, 2, 2, 2, 2, 2, 2, 2, 2, 2, 2, 2, 2, 2, 2, 2, 2, 2, 2, 2, 2, 2, 2, 2, 2, 2, 2, 2, 2, 2, 2, 2, 2, 2, 2, 2, 2, 1, 1, 1, 1, 1, 1, 1, 1, 1, 1, 1, 1, 1, 1, 1, 1, 1, 1, 1, 1, 1, 1, 1, 1, 1, 1, 1

Corresponding number of selected Gene ontology terms each gene is involved with:
(In the same order as above)
3, 3, 3, 3, 3, 3, 3, 2, 2, 2, 3, 2, 2, 2, 2, 2, 2, 2, 2, 2, 3, 2, 2, 2, 2, 2, 2, 2, 2, 2, 3, 2, 2, 2, 3, 1, 1, 1, 1, 1, 1, 1, 1, 1, 1, 1, 1, 1, 1, 1, 1, 1, 1, 1, 1, 1, 1, 1, 2, 1, 1, 2, 1, 1, 1, 2, 2, 2, 1, 1, 3, 1, 1, 1, 1, 1, 1, 1, 2, 2, 1, 1, 2, 1, 2, 1, 1, 1, 1, 1, 1, 1, 1, 1, 1, 1, 2, 2, 1, 1, 1, 2, 2, 2, 2, 1, 2, 1, 1, 1, 1, 1, 1, 1, 1, 1, 1, 1, 1, 1, 1, 1, 2, 2, 1, 2, 1, 1, 1, 1, 1, 1, 2, 2, 2, 1, 1, 1, 1, 1, 3, 3, 3, 1, 1, 1, 1, 1, 1, 1, 1, 3, 1, 1, 1, 3, 1, 1, 1, 1, 1, 1, 2, 1, 2, 1, 2, 1, 2, 1, 2, 2, 1, 2, 1, 1, 1, 1, 2, 1, 2, 1, 1, 1, 2, 1, 1, 1, 1, 2, 1, 1, 1, 2, 2, 1, 2, 2, 1, 1, 1, 1, 1, 1, 1, 1, 1, 1, 1, 1, 1, 1, 1, 1, 1, 1, 1, 1, 1, 1, 1, 1, 1, 1, 1, 1, 1, 1, 1, 1

Corresponding number of selected TFs each gene is involved with:
(In the same order as above)
11, 11, 11, 11, 10, 10, 9, 11, 11, 11, 7, 10, 10, 10, 10, 10, 10, 8, 8, 8, 5, 7, 7, 7, 7, 7, 7, 7, 7, 7, 4, 6, 6, 6, 4, 11, 11, 11, 11, 11, 11, 11, 11, 11, 11, 11, 11, 11, 11, 11, 11, 11, 11, 11, 11, 11, 11, 5, 10, 10, 5, 10, 10, 10, 5, 5, 5, 9, 9, 3, 9, 9, 9, 9, 9, 8, 8, 4, 4, 8, 8, 4, 8, 4, 7, 7, 7, 7, 7, 7, 7, 7, 7, 6, 6, 6, 3, 3, 6, 6, 6, 3, 3, 3, 3, 6, 3, 6, 6, 6, 6, 6, 6, 5, 5, 5, 5, 5, 5, 5, 5, 2, 2, 4, 2, 4, 4, 4, 4, 4, 4, 2, 2, 2, 4, 4, 3, 3, 3, 1, 1, 1, 3, 3, 3, 3, 3, 3, 3, 3, 1, 3, 3, 3, 1, 3, 3, 3, 2, 2, 2, 1, 2, 1, 2, 1, 2, 1, 2, 1, 1, 2, 1, 2, 2, 2, 2, 1, 2, 1, 2, 2, 2, 1, 2, 2, 2, 2, 1, 2, 2, 2, 1, 1, 2, 1, 1, 2, 2, 1, 1, 1, 1, 1, 1, 1, 1, 1, 1, 1, 1, 1, 1, 1, 1, 1, 1, 1, 1, 1, 1, 1, 1, 1, 1, 1, 1, 1, 1

Gene ontology terms ranked according to mean of effect sizes in cluster:
cell differentiation | hemopoiesis | Hedgehog signaling complex

Corresponding mean of effect sizes of each Gene ontology term:
(In the same order as above)
2.14, 1.58, 1.40

Corresponding total number of genes of each Gene ontology term:
(In the same order as above)
871, 343, 230

Corresponding number of genes of each Gene ontology term with at least one TFBS (of selected TFs):

(In the same order as above)
175, 94, 54

Corresponding total number of TFBS (of selected TFs) for each Gene ontology term (in all genes):
(In the same order as above)
919, 431, 281

Corresponding number of selected TFs each Gene ontology term is involved with:
(In the same order as above)
11, 11, 11

TFs ranked according to mean of effect sizes in cluster:
VETF_Q6, VAP2ALPHA_01, V$E2F1_Q6, V$E2F1_Q3, V$AP2GAMMA_01, V$E2F_Q2, VMAZ_Q6, VAP2_Q6, V$WT1_Q6, V$EGR_Q6, V$AP2_Q6_01

Corresponding mean of effect sizes of each TF:
(In the same order as above)
2.23, 2.23, 2.14, 1.89, 1.85, 1.83, 1.82, 1.62, 1.51, 0.87, 0.78

Corresponding total number of TFBS for each TF (genome-wide):
(In the same order as above)
699, 686, 691, 709, 695, 714, 734, 688, 729, 695, 690

Corresponding total number of TFBS for each TF (in all genes in selected Gene ontology terms):
(In the same order as above)
157, 148, 162, 146, 143, 156, 148, 149, 156, 134, 132

Corresponding number of genes (of selected Gene ontology terms) each TF is involved with:
(In the same order as above)
107, 102, 112, 103, 98, 110, 104, 105, 107, 97, 97

Corresponding number of selected Gene ontology terms each TF is involved with:
(In the same order as above)
3, 3, 3, 3, 3, 3, 3, 3, 3, 3, 3

Cluster for columns 138 to 140, rows 241 to 243

Gene ontology terms

hemoglobin biosynthetic process | heme biosynthetic process | heme metabolic process

TFs

V$LMO2COM_02 | V$GATA1_04 | V$GATA1_02

Information

All related TFs:
(List of all TFs that are related to any of the PWMs)
GATA-1, GATA-1A, Lmo2

Ranked gene list:
(All genes of the selected Gene ontology terms with hits of any of the selected TFs, ranked according to

the total number of TFBS)
HMBS, PPOX, HFE, ZFPM1, DCTN3, BLVRA, ALAD, AHSA1, UROD, RAB8A, NFE2, HBA2, GATA1, MYB, KLF1, HMOX2, GCLC, EPOR, DLX3

Corresponding total number of TFBS:
(For each gene listed above, the total number of TFBS for any of the selected TFs, multiplied by the number of selected Gene ontology terms containing that gene)
9, 6, 6, 3, 3, 3, 3, 3, 2, 2, 2, 2, 2, 1, 1, 1, 1, 1, 1

Corresponding number of selected Gene ontology terms each gene is involved with:
(In the same order as above)
3, 2, 2, 1, 1, 1, 3, 1, 2, 2, 2, 2, 2, 1, 1, 1, 1, 1, 1

Corresponding number of selected TFs each gene is involved with:
(In the same order as above)
3, 3, 3, 3, 3, 3, 1, 3, 1, 1, 1, 1, 1, 1, 1, 1, 1, 1, 1

Gene ontology terms ranked according to mean of effect sizes in cluster:
hemoglobin biosynthetic process | heme metabolic process | heme biosynthetic process

Corresponding mean of effect sizes of each Gene ontology term:
(In the same order as above)
4.56, 3.87, 2.07

Corresponding total number of genes of each Gene ontology term:
(In the same order as above)
37, 22, 73

Corresponding number of genes of each Gene ontology term with at least one TFBS (of selected TFs):
(In the same order as above)
12, 7, 11

Corresponding total number of TFBS (of selected TFs) for each Gene ontology term (in all genes):
(In the same order as above)
20, 13, 19

Corresponding number of selected TFs each Gene ontology term is involved with:
(In the same order as above)
3, 3, 3

TFs ranked according to mean of effect sizes in cluster:
V$LMO2COM_02, V$GATA1_04, V$GATA1_02

Corresponding mean of effect sizes of each TF:
(In the same order as above)
3.74, 3.39, 3.37

Corresponding total number of TFBS for each TF (genome-wide):
(In the same order as above)
687, 698, 694

Corresponding total number of TFBS for each TF (in all genes in selected Gene ontology terms):
(In the same order as above)
18, 18, 16

Corresponding number of genes (of selected Gene ontology terms) each TF is involved with:
(In the same order as above)
12, 11, 10

Corresponding number of selected Gene ontology terms each TF is involved with:
(In the same order as above)
3, 3, 3

Cluster for columns 349 to 368, rows 47 to 49

Gene ontology terms

cell envelope | outer membrane | porin activity | abscission | ethylene biosynthetic process | merozoite dense granule | melanosome | organelle | oligosaccharide biosynthetic process | checkpoint clamp complex | transpiration | vacuolar membrane | vacuole | multivesicular body | autophagic vacuole | autophagy | Golgi membrane | Golgi stack | Golgi apparatus | trans-Golgi network

TFs

V$SREBP1_01 | V$ARNT_02 | V$XBP1_01

Information

All related TFs:
(List of all TFs that are related to any of the PWMs)
Arnt, SREBP-1, SREBP-1a, SREBP-1b, SREBP-1c, XBP-1

Ranked gene list:
(All genes of the selected Gene ontology terms with hits of any of the selected TFs, ranked according to the total number of TFBS)
LAMP1, RAB5A, CALR, RPS27A, RAB7A, GABARAP, CTSA, GAA, BECN1, BAX, CD63, VPS26A, STX6, RRAGB, PSAP, MGAT1, IGF2R, ATP6V0C, ARFGEF2, APEX1, TMEM201, SFRS5, GOLGA1, GEMIN4, GALT, VPS11, UNC13D, TOM1, TNFSF13, SNX2, PDCD6IP, MTHFR, MDM4, MCOLN1, ING1, HSPD1, HPS1, GOLGA3, GAPDH, GALNS, CHMP4A, ATG3, ALG1, ABCB6, VDAC1, RPN1, RAB4A, PRIM2, MYD88, KDELR1, HPS5, HPS3, GGA2, GCS1, DHDDS, CTRL, COPG, CAV1, CASP3, ACTA1, VPS16, VLDLR, UVRAG, UBE4B, TSC2, TSC1, TRPV1, TPT1, TFAP2A, TBC1D20, SUB1, SLC7A1, SLC24A5, SDF4, S100A6, RPLP2, RNASEH2C, RGS20, RERE, RER1, RABAC1, RAB21, QTRT1, PRDX2, PNKD, PLDN, PLA2G1B, PLA2G15, PAICS, P76, OSBP, NR2F1, NPTN, NAPA, NAGPA, MGAT3, MARCH8, LONP1, INVS, INSIG1, GCC1, FADD, EXOC3, ERF, ENOPH1, EEF1A1, DSCR3, DNASE1, CYTH3, CUX1, CPT1A, COPB2, CNO, CHAF1A, CD164, CCL18, CASP9, BNIP3L, ATXN3, ATP6V1H, ATP6V1A, ATP6V0D1, AP1GBP1, AIDA, AGPAT1, ACOX1, VPS52, USP14, TP53INP1, TOM1L2, TMED10, TM9SF2, TIMM50, TIMM13, STX18, STMN1, SRRM2, SLC30A3, SHCBP1, SCAP, S100B, RPS9, RHEB, RANBP2, RAD17, RABEP1, RAB12, PRKAR1A, PLEKHA8, PIM1, PHF20, PEX7, PEX3, PCDH8, MYOC, MON2, MAPK1, LSS, LOXL1, INSIG2, HSPE1, HRAS, HMGB1, HLF, HADHB, GPR3, GPR132, GLTSCR2, GBA, FTMT, FTCD, FOXM1, FLVCR2, FBL, FAM168B, DOLK, CTDSP2, CORO7, CLN3, CLCN5, CERK, CASP7, C18orf8, BTG3, B4GALT7, B3GALT4, ATP5A1, ATG9B, ARFIP2, ARAP1, ANK1, ADAM7

Corresponding total number of TFBS:
(For each gene listed above, the total number of TFBS for any of the selected TFs, multiplied by the number of selected Gene ontology terms containing that gene)
30, 14, 10, 9, 9, 9, 9, 8, 8, 8, 7, 6, 6, 6, 6, 6, 6, 6, 6, 5, 5, 5, 5, 5, 4, 4, 4, 4, 4, 4, 4, 4, 4, 4, 4, 4, 4, 4, 4, 4, 4, 4, 4, 3, 3, 3, 3, 3, 3, 3, 3, 3, 3, 3, 3, 3, 3, 3, 3, 2, 2, 2, 2, 2, 2, 2, 2, 2, 2, 2, 2, 2, 2, 2, 2, 2, 2, 2, 2, 2, 2, 2,

2, 2, 2, 2, 2, 2, 2, 2, 2, 2, 2, 2, 2, 2, 2, 2, 2, 2, 2, 2, 2, 2, 2, 2, 2, 2, 2, 2, 2, 2, 2, 2, 2, 2, 2, 2, 2, 2, 2, 2, 1, 1, 1, 1, 1, 1, 1, 1, 1, 1, 1, 1, 1, 1, 1, 1, 1, 1, 1, 1, 1, 1, 1, 1, 1, 1, 1, 1, 1, 1, 1, 1, 1, 1, 1, 1, 1, 1, 1, 1, 1, 1, 1, 1, 1, 1, 1, 1, 1, 1, 1, 1, 1, 1, 1, 1, 1, 1, 1, 1, 1, 1, 1, 1, 1, 1, 1, 1

Corresponding number of selected Gene ontology terms each gene is involved with:
(In the same order as above)
10, 7, 5, 9, 9, 3, 9, 4, 4, 4, 7, 3, 3, 2, 3, 3, 3, 3, 2, 3, 5, 5, 5, 5, 5, 2, 2, 2, 4, 2, 4, 2, 2, 2, 4, 4, 4, 4, 2, 2, 2, 2, 2, 2, 3, 3, 3, 3, 3, 3, 1, 3, 3, 3, 1, 1, 3, 3, 3, 3, 2, 2, 2, 1, 1, 1, 2, 2, 2, 1, 1, 2, 2, 1, 2, 2, 1, 1, 2, 2, 2, 1, 1, 1, 2, 2, 2, 1, 2, 1, 2, 2, 1, 1, 2, 2, 1, 2, 1, 1, 2, 1, 2, 1, 2, 2, 1, 2, 1, 2, 1, 2, 2, 1, 1, 2, 1, 1, 1, 1, 2, 2, 1, 1, 1, 1, 1, 1, 1, 1, 1, 1, 1, 1, 1, 1, 1, 1, 1, 1, 1, 1, 1, 1, 1, 1, 1, 1, 1, 1, 1, 1, 1, 1, 1, 1, 1, 1, 1, 1, 1, 1, 1, 1, 1, 1, 1, 1, 1, 1, 1, 1, 1, 1, 1, 1, 1, 1, 1, 1, 1, 1, 1, 1, 1, 1, 1, 1, 1, 1, 1, 1, 1, 1, 1

Corresponding number of selected TFs each gene is involved with:
(In the same order as above)
3, 2, 2, 1, 1, 3, 1, 2, 2, 2, 1, 2, 2, 3, 2, 2, 2, 2, 3, 2, 1, 1, 1, 1, 1, 2, 2, 2, 1, 2, 1, 2, 2, 2, 1, 1, 1, 1, 2, 2, 2, 2, 2, 2, 1, 1, 1, 1, 1, 1, 3, 1, 1, 1, 3, 3, 1, 1, 1, 1, 1, 1, 2, 2, 2, 1, 1, 1, 2, 2, 1, 1, 2, 1, 1, 2, 2, 1, 1, 1, 2, 2, 2, 1, 1, 1, 2, 1, 2, 1, 1, 2, 2, 1, 1, 2, 1, 2, 2, 1, 2, 1, 2, 1, 1, 2, 1, 2, 1, 2, 1, 1, 2, 2, 1, 2, 2, 2, 2, 1, 1, 2, 2, 2, 2, 1, 1, 1, 1, 1, 1, 1, 1, 1, 1, 1, 1, 1, 1, 1, 1, 1, 1, 1, 1, 1, 1, 1, 1, 1, 1, 1, 1, 1, 1, 1, 1, 1, 1, 1, 1, 1, 1, 1, 1, 1, 1, 1, 1, 1, 1, 1, 1, 1, 1, 1, 1, 1, 1, 1, 1, 1, 1, 1, 1, 1, 1, 1, 1, 1, 1, 1, 1, 1, 1

Gene ontology terms ranked according to mean of effect sizes in cluster:
outer membrane | cell envelope | autophagy | melanosome | trans-Golgi network | vacuolar membrane | autophagic vacuole | multivesicular body | vacuole | Golgi apparatus | porin activity | organelle | oligosaccharide biosynthetic process | merozoite dense granule | transpiration | checkpoint clamp complex | ethylene biosynthetic process | Golgi membrane | Golgi stack | abscission

Corresponding mean of effect sizes of each Gene ontology term:
(In the same order as above)
2.73, 2.46, 2.27, 2.18, 2.16, 2.05, 2.04, 1.78, 1.77, 1.73, 1.60, 1.56, 1.54, 1.36, 1.22, 0.72, 0.53, 0.39, 0.16, -0.41

Corresponding total number of genes of each Gene ontology term:
(In the same order as above)
259, 91, 195, 56, 300, 72, 63, 67, 208, 482, 77, 255, 25, 40, 23, 42, 25, 93, 100, 32

Corresponding number of genes of each Gene ontology term with at least one TFBS (of selected TFs):
(In the same order as above)
35, 13, 34, 11, 42, 16, 12, 13, 33, 66, 12, 39, 6, 6, 3, 6, 3, 13, 13, 2

Corresponding total number of TFBS (of selected TFs) for each Gene ontology term (in all genes):
(In the same order as above)
51, 17, 54, 16, 60, 24, 21, 18, 46, 88, 15, 48, 8, 7, 5, 7, 5, 14, 16, 2

Corresponding number of selected TFs each Gene ontology term is involved with:
(In the same order as above)
3, 3, 3, 3, 3, 3, 3, 3, 3, 3, 3, 3, 3, 3, 3, 3, 3, 3, 3, 2

TFs ranked according to mean of effect sizes in cluster:
V$ARNT_02, V$SREBP1_01, V$XBP1_01

Corresponding mean of effect sizes of each TF:
(In the same order as above)
1.69, 1.69, 1.09

Corresponding total number of TFBS for each TF (genome-wide):

(In the same order as above)
625, 629, 639

Corresponding total number of TFBS for each TF (in all genes in selected Gene ontology terms):
(In the same order as above)
184, 178, 160

Corresponding number of genes (of selected Gene ontology terms) each TF is involved with:
(In the same order as above)
91, 92, 81

Corresponding number of selected Gene ontology terms each TF is involved with:
(In the same order as above)
20, 19, 20

Cluster for columns 1132 to 1135, rows 423 to 426

Gene ontology terms

transcription factor activity | termination of RNA polymerase III transcription | transcription initiation | acetyltransferase activity

TFs

V$E2F_Q6_01 | V$E2F_Q4_01 | V$E2F_Q3_01 | V$E2F1_Q4_01

Information

All related TFs:
(List of all TFs that are related to any of the PWMs)
DP-1, E2F, E2F+E4, E2F-1, E2F-1:DP-1, E2F-3a, E2F-4, E2F-7

Ranked gene list:
(All genes of the selected Gene ontology terms with hits of any of the selected TFs, ranked according to the total number of TFBS)
ZFP36L1, JUN, RBL1, PELP1, JUND, HIST1H4I, GTF2A1, ETV4, CDKN1A, ZFP36L2, GTF2F1, UBTF, TP73, TP53, TCF3, SMC3, SLC20A1, RPS15, RPS10, RPL8, PCNA, NFATC1, NASP, NARG1, MYB, MDM2, MAZ, MARCKSL1, MAF1, LRBA, IRF1, HOXA9, HIST4H4, FASN, E2F3, E2F1, DRAP1, CUX1, CREBZF, CNOT1, CKB, CBX4, CAD, ATP5B, AKT1, TRRAP, TFAP2A, TCF7, SP1, SHMT2, PURA, PPP1R8, POLRMT, NOL3, LBX1, KLF13, JUNB, FOSB, CTDP1, CLPX, ATF3, TRIM26, TEAD1, TAF4, SUPT3H, PCSK4, MORF4L1, ISCU, DHFR, COBRA1, CDK8, ACTB, ZEB1, SMARCA4, NFIC, ABHD14B

Corresponding total number of TFBS:
(For each gene listed above, the total number of TFBS for any of the selected TFs, multiplied by the number of selected Gene ontology terms containing that gene)
12, 12, 8, 8, 8, 8, 8, 8, 8, 6, 6, 4, 4, 4, 4, 4, 4, 4, 4, 4, 4, 4, 4, 4, 4, 4, 4, 4, 4, 4, 4, 4, 4, 4, 4, 4, 4, 4, 4, 4, 4, 4, 4, 4, 3, 3, 3, 3, 3, 3, 3, 3, 3, 3, 3, 3, 3, 3, 3, 3, 2, 2, 2, 2, 2, 2, 2, 2, 2, 2, 2, 2, 1, 1, 1, 1

Corresponding number of selected Gene ontology terms each gene is involved with:
(In the same order as above)
3, 3, 2, 2, 2, 2, 2, 2, 2, 2, 2, 1, 1, 2, 2, 1, 1, 1, 1, 1, 1, 1, 1, 1, 1, 1, 1, 1, 1, 1, 1, 1, 2, 1, 1, 2, 1, 1, 1, 1, 1, 1, 1, 1, 1, 1, 1, 1, 3, 1, 1, 1, 1, 1, 1, 1, 1, 1, 1, 1, 1, 1, 2, 1, 1, 1, 1, 1, 1, 1, 1, 1, 1, 1, 1, 1

Corresponding number of selected TFs each gene is involved with:
(In the same order as above)
4, 4, 4, 4, 4, 4, 4, 4, 4, 3, 3, 4, 4, 2, 2, 4, 4, 4, 4, 4, 4, 4, 4, 4, 4, 4, 4, 4, 4, 4, 4, 2, 4, 4, 2, 4, 4, 4, 4, 4, 4, 4, 4, 4, 3, 3, 3, 1, 3, 3, 3, 3, 3, 3, 3, 3, 3, 3, 3, 2, 1, 2, 2, 2, 2, 2, 2, 2, 2, 2, 1, 1, 1, 1

Gene ontology terms ranked according to mean of effect sizes in cluster:
transcription initiation | termination of RNA polymerase III transcription | transcription factor activity | acetyltransferase activity

Corresponding mean of effect sizes of each Gene ontology term:
(In the same order as above)
2.63, 2.41, 2.29, 1.48

Corresponding total number of genes of each Gene ontology term:
(In the same order as above)
407, 52, 83, 112

Corresponding number of genes of each Gene ontology term with at least one TFBS (of selected TFs):
(In the same order as above)
56, 8, 18, 14

Corresponding total number of TFBS (of selected TFs) for each Gene ontology term (in all genes):
(In the same order as above)
173, 29, 57, 43

Corresponding number of selected TFs each Gene ontology term is involved with:
(In the same order as above)
4, 4, 4, 4

TFs ranked according to mean of effect sizes in cluster:
V$E2F1_Q4_01, V$E2F_Q3_01, V$E2F_Q4_01, V$E2F_Q6_01

Corresponding mean of effect sizes of each TF:
(In the same order as above)
2.52, 2.47, 2.35, 1.47

Corresponding total number of TFBS for each TF (genome-wide):
(In the same order as above)
681, 690, 667, 686

Corresponding total number of TFBS for each TF (in all genes in selected Gene ontology terms):
(In the same order as above)
81, 81, 74, 66

Corresponding number of genes (of selected Gene ontology terms) each TF is involved with:
(In the same order as above)
65, 65, 60, 51

Corresponding number of selected Gene ontology terms each TF is involved with:
(In the same order as above)
4, 4, 4, 4

Cluster for columns 681 to 684, rows 9 to 17

Gene ontology terms

peptidase activity | protein digestion | endopeptidase activity | secretory granule

TFs

V$CREB_Q4_01 | V$CREB_Q2_01 | V$CREB_Q4 | V$CREBP1_Q2 | V$CREB_Q2 | V$CREB_01 | V$CREBP1CJUN_01 | V$CREBATF_Q6 | V$ATF_B

Information

All related TFs:
(List of all TFs that are related to any of the PWMs)
120-kDa, 47-kDa, ATF, ATF-1, ATF-2, ATF-4, ATF-a, ATF-like, ATF/CREB, ATF2, ATF2-isoform2, ATF3, ATF4, ATF5, ATF6, ATFa-isoform1, CRE-BP1, CRE-BP2, CREB, CREBbeta, CREMalpha, CREMbeta, CREMgamma, CREMtau, CREMtau1, CREMtau2, CREMtaualpha, c-Jun, deltaCREB

Ranked gene list:
(All genes of the selected Gene ontology terms with hits of any of the selected TFs, ranked according to the total number of TFBS)
TPP1, CYCS, MUC1, VAMP2, THOP1, LAP3, UBC, STXBP3, RCE1, PNRC1, FN1, CNDP2, CHGB, BRAF, CALCA, SH2D3C, CDC42, YME1L1, SLC18A2, RTN2, PSENEN, CTSL1, CASP9, SST, SNAP25, SCG2, RAB3A, PNPLA4, VGF, PCOLCE, IKBKAP, CGB, APPBP2, PVRL2, OSGEP, CHGA, CALM2, UNC13D, STX2, SH2D3A, PARK7, ICA1

Corresponding total number of TFBS:
(For each gene listed above, the total number of TFBS for any of the selected TFs, multiplied by the number of selected Gene ontology terms containing that gene)
18, 18, 16, 12, 12, 12, 9, 9, 9, 9, 9, 9, 9, 9, 8, 7, 7, 6, 6, 6, 6, 6, 6, 4, 4, 4, 4, 4, 3, 3, 3, 3, 3, 2, 2, 2, 2, 1, 1, 1, 1, 1

Corresponding number of selected Gene ontology terms each gene is involved with:
(In the same order as above)
2, 2, 2, 2, 2, 2, 1, 1, 1, 1, 1, 1, 1, 1, 2, 1, 1, 1, 1, 1, 1, 2, 1, 2, 2, 1, 1, 1, 1, 1, 1, 1, 1, 1, 1, 1, 1, 1, 1, 1, 1, 1

Corresponding number of selected TFs each gene is involved with:
(In the same order as above)
9, 9, 8, 6, 6, 6, 9, 9, 9, 9, 9, 9, 9, 9, 4, 7, 7, 6, 6, 6, 6, 3, 6, 2, 2, 4, 4, 4, 3, 3, 3, 3, 3, 2, 2, 2, 2, 1, 1, 1, 1, 1

Gene ontology terms ranked according to mean of effect sizes in cluster:
endopeptidase activity | secretory granule | peptidase activity | protein digestion

Corresponding mean of effect sizes of each Gene ontology term:
(In the same order as above)
1.99, 1.82, 1.65, 1.06

Corresponding total number of genes of each Gene ontology term:
(In the same order as above)
56, 117, 246, 26

Corresponding number of genes of each Gene ontology term with at least one TFBS (of selected TFs):
(In the same order as above)
10, 19, 21, 2

Corresponding total number of TFBS (of selected TFs) for each Gene ontology term (in all genes):
(In the same order as above)
49, 78, 127, 12

Corresponding number of selected TFs each Gene ontology term is involved with:
(In the same order as above)
9, 9, 9, 9

TFs ranked according to mean of effect sizes in cluster:
VATF_B, VCREB_Q4_01, V$CREBP1_Q2, V$CREBP1CJUN_01, V$CREB_Q4, V$CREB_01, V$CREB_Q2_01, V$CREBATF_Q6, V$CREB_Q2

Corresponding mean of effect sizes of each TF:
(In the same order as above)
2.57, 2.02, 1.99, 1.64, 1.55, 1.54, 1.34, 1.22, 0.80

Corresponding total number of TFBS for each TF (genome-wide):
(In the same order as above)
665, 667, 679, 652, 671, 659, 679, 671, 676

Corresponding total number of TFBS for each TF (in all genes in selected Gene ontology terms):
(In the same order as above)
37, 34, 33, 28, 29, 27, 26, 27, 25

Corresponding number of genes (of selected Gene ontology terms) each TF is involved with:
(In the same order as above)
29, 28, 26, 21, 22, 21, 21, 22, 21

Corresponding number of selected Gene ontology terms each TF is involved with:
(In the same order as above)
4, 4, 4, 4, 4, 4, 4, 4, 4

Cluster for columns 1053 to 1071, rows 161 to 167

Gene ontology terms

tectum | axon guidance | nervous system development | synaptic cleft | terminal button | cognition | toxin binding | choline O-acetyltransferase activity | neurotransmitter biosynthetic process | basal lamina | sarcoplasm | response to ATP | response to histamine | serotonin receptor activity | interchromatin granule | acetylcholinesterase activity | choline transport | angiotensin-converting enzyme inhibitor activity | saliva secretion

TFs

V$USF2_Q6 | V$ZIC2_01 | V$TTF1_Q6 | V$VDR_Q6 | V$LRF_Q2 | V$LBP1_Q6 | V$AP4_Q6_01

Information

All related TFs:
(List of all TFs that are related to any of the PWMs)
AP-4, FBI-1, LBP-1, LRF, Nkx2-1, OCZF, USF1:USF2, USF2, USF2a, VDR, ZIC2, Zic2

Ranked gene list:
(All genes of the selected Gene ontology terms with hits of any of the selected TFs, ranked according to the total number of TFBS)
FGFR3, ACHE, AGRN, AKT1, THRA, SEMA3B, PPP1R9B, PLXNA1, PER1, GPT, FLJ32987, EFNA3, CDK5, SLC25A1, PAX6, DPAGT1, LBX1, VEGFA, PNPLA6, ENTPD2, EFNA2, WNT1, SLC4A2, SLC2A4RG, SCRIB, RARA, PPP1R1B, PLEC1, NTN3, NOTCH1, NOC2L, NKX2-2, MDK, GNAI2, GFI1, FGF8, DVL1, ACTG1, WBP7, S100A6, PTDSS2, PNMT, PGP, PDE4A, MGAT1, KCNH2, EGR1, ARHGEF2, TNFSF13, SH2B2, PSD, P2RY11, JUND, DBP, CDT1, CDH15, PARD6A, NGFR, EPHB3, EPB49, EFNA1, AQP3, VASP, NR3C2, NDOR1, LAMA5, HES1, GPR132, FBXL15, ENO3, DDR1, POU2F2, NFIX, MICAL1, LIF, HRH3, HBA2, GPC1, GALR3, GALNS, FERMT3, DLG4, BRD1, TFAP2A, SEMA7A, SEMA3G, PYGM, PAX5, NOTCH4, MUC1, MAP1A, KCNA2, INS, IGF2, GRM2, GRIN1, FXYD1, AQP5, AMD1

Corresponding total number of TFBS:
(For each gene listed above, the total number of TFBS for any of the selected TFs, multiplied by the number of selected Gene ontology terms containing that gene)
98, 91, 25, 21, 14, 14, 14, 14, 14, 14, 14, 14, 14, 12, 12, 10, 9, 8, 8, 8, 8, 7, 7, 7, 7, 7, 7, 7, 7, 7, 7, 7, 7, 7, 7, 7, 7, 6, 6, 6, 6, 6, 6, 6, 6, 6, 5, 5, 5, 5, 5, 5, 5, 5, 4, 4, 4, 4, 4, 4, 3, 3, 3, 3, 3, 3, 3, 3, 3, 2, 2, 2, 2, 2, 2, 2, 2, 2, 2, 2, 2, 1, 1, 1, 1, 1, 1, 1, 1, 1, 1, 1, 1, 1, 1, 1

Corresponding number of selected Gene ontology terms each gene is involved with:
(In the same order as above)
14, 13, 5, 3, 2, 2, 2, 2, 2, 2, 2, 2, 2, 2, 3, 2, 3, 2, 2, 2, 2, 1, 1, 1, 1, 1, 1, 1, 1, 1, 1, 1, 1, 1, 1, 1, 1, 1, 1, 1, 1, 2, 1, 1, 1, 1, 1, 1, 5, 1, 1, 1, 1, 1, 1, 1, 1, 4, 2, 1, 1, 1, 1, 1, 1, 1, 1, 1, 1, 1, 1, 1, 1, 2, 2, 2, 1, 1, 1, 2, 1, 1, 2, 1, 1, 1, 1, 1, 1, 1, 1, 1, 1, 1, 1, 1, 1, 1

Corresponding number of selected TFs each gene is involved with:
(In the same order as above)
7, 7, 5, 7, 7, 7, 7, 7, 7, 7, 7, 7, 7, 6, 4, 5, 3, 4, 4, 4, 4, 7, 7, 7, 7, 7, 7, 7, 7, 7, 7, 7, 7, 7, 7, 7, 7, 6, 6, 6, 3, 6, 6, 6, 6, 6, 6, 1, 5, 5, 5, 5, 5, 5, 5, 4, 1, 2, 4, 4, 4, 3, 3, 3, 3, 3, 3, 3, 3, 3, 2, 2, 1, 1, 1, 2, 2, 2, 1, 2, 2, 1, 1, 1, 1, 1, 1, 1, 1, 1, 1, 1, 1, 1, 1, 1

Gene ontology terms ranked according to mean of effect sizes in cluster:
saliva secretion | acetylcholinesterase activity | neurotransmitter biosynthetic process | choline transport | angiotensin-converting enzyme inhibitor activity | toxin binding | terminal button | synaptic cleft | interchromatin granule | sarcoplasm | choline O-acetyltransferase activity | cognition | tectum | serotonin receptor activity | axon guidance | response to ATP | nervous system development | basal lamina | response to histamine

Corresponding mean of effect sizes of each Gene ontology term:
(In the same order as above)
2.40, 2.08, 1.89, 1.72, 1.63, 1.52, 1.51, 1.49, 1.46, 1.40, 1.26, 1.19, 1.16, 1.13, 1.05, 1.03, 1.02, 0.91, 0.85

Corresponding total number of genes of each Gene ontology term:
(In the same order as above)
27, 27, 27, 26, 63, 33, 21, 66, 35, 24, 37, 69, 116, 49, 187, 47, 185, 112, 37

Corresponding number of genes of each Gene ontology term with at least one TFBS (of selected TFs):
(In the same order as above)
7, 6, 5, 4, 11, 5, 2, 8, 5, 5, 6, 7, 15, 6, 26, 4, 26, 11, 4

Corresponding total number of TFBS (of selected TFs) for each Gene ontology term (in all genes):
(In the same order as above)
38, 29, 27, 22, 49, 22, 14, 38, 27, 23, 20, 37, 70, 27, 103, 22, 112, 50, 16

Corresponding number of selected TFs each Gene ontology term is involved with:
(In the same order as above)
7, 7, 7, 7, 7, 7, 7, 7, 7, 7, 7, 7, 7, 7, 7, 7, 7, 7, 7

TFs ranked according to mean of effect sizes in cluster:
VVDR_Q6, VLRF_Q2, V$AP4_Q6_01, V$LBP1_Q6, V$TTF1_Q6, V$ZIC2_01, V$USF2_Q6

Corresponding mean of effect sizes of each TF:
(In the same order as above)
1.60, 1.52, 1.51, 1.46, 1.35, 1.26, 1.13

Corresponding total number of TFBS for each TF (genome-wide):
(In the same order as above)
692, 719, 699, 691, 714, 716, 703

Corresponding total number of TFBS for each TF (in all genes in selected Gene ontology terms):
(In the same order as above)
109, 115, 109, 109, 99, 103, 102

Corresponding number of genes (of selected Gene ontology terms) each TF is involved with:
(In the same order as above)
58, 65, 62, 61, 57, 57, 56

Corresponding number of selected Gene ontology terms each TF is involved with:
(In the same order as above)
19, 19, 19, 19, 19, 19, 19

Cluster for columns 801 to 813, rows 267 to 282

Gene ontology terms

insulin-like growth factor binding protein complex | insulin-like growth factor receptor binding | insulin-like growth factor binding | insulin-like growth factor II binding | insulin-like growth factor I binding | insulin-like growth factor receptor activity | growth factor binding | regulation of growth | glucose transport | glucose homeostasis | insulin binding | insulin receptor binding | response to wounding

TFs

V$ETF_Q6 | V$E2F_Q2 | V$E2F1_Q6 | V$E2F1_Q3 | V$AP2GAMMA_01 | V$AP2ALPHA_01 | V$AP2_Q6_01 | V$AP2_Q6 | V$WT1_Q6 | V$EGR_Q6 | V$MAZ_Q6 | V$MAZR_01 | V$E2F1_Q3_01 | V$HES1_Q2 | V$ACAAT_B | V$AP2_Q3

Information

All related TFs:
(List of all TFs that are related to any of the PWMs)
AP-2, AP-2alpha, AP-2alphaA, AP-2alphaB, AP-2beta, AP-2gamma, DP-1, E2F, E2F+E4, E2F-1, E2F-3a, E2F-4, ETF, Egr-1, Egr-2, Egr-3, HES-1, MAZ, MAZR, WT1, WT1-del2

Ranked gene list:
(All genes of the selected Gene ontology terms with hits of any of the selected TFs, ranked according to the total number of TFBS)
IGF1R, AKT1, IRS1, CCND1, LBX1, IRS2, TGFB1, IGF2, FOXO3, FOXO1, VEGFA, ACTB, HRAS,

HOXA10, RARG, PER1, TNFSF13, SOCS3, MDK, IRF1, CTSD, PAX6, PDGFB, TFAP2A, GNAI2, UBTF, MYO1C, OGFR, HOXC6, FASN, BLVRB, SSSCA1, NKX2-2, MAPK11, MAFA, CUX1, RTN4RL1, GPC1, GFI1, SHC1, PIK3R2, HMHA1, FLJ32987, WNT10B, HBA2, PTPN6, PRKCZ, CISH, SH2B2, PPP1R13B, SP3, SOCS2, RASSF1, NUTF2, NR3C1, HMGB1, CORO7, CAMK2G, TNF, GAPDH, EGR1, BRD1, TRIP10, STMN1, SOS1, RPS6KB2, MAPK1, LRP5, HOXA11, GRB7, CDKN1A, BAX, SRC, SOCS1, RARA, PTP4A1, MPG, MAP2K3, IGF2BP3, CDKN1B, CDC20, ST3GAL4, SLC20A1, PTPRU, KLK1, HBA1, GOT2, ATF3, AKT3, SLC2A1, PPP3CA, PIK3CA, LEPROT, KCNJ4, HSP90AA1, GAB2, CREB1, CALM1, VAMP2, STAT5B, SMAD2, SLC38A2, SDC3, RHOA, PRKCA, PPARD, PPARA, PIGP, PDPK1, PAOX, NUCB2, NOL3, NEDD4, LEPR, GPI, ELAVL1, CYP11A1, CRTC2, CNBP, CASP9, AKT2, ADIPOR1, ADA, TFRC, STX6, STX16, STAT5A, SCARB1, RHOQ, RFTN1, RAPGEF1, RAB40B, PTK2, PPM2C, PCK2, ONECUT1, ODC1, NR0B2, MTHFR, MAPK7, MACF1, LRBA, LPIN1, KCNJ11, IHH, IDE, HK1, HDAC3, G6PC3, FOS, EP300, CAPN10, CALM3, CALM2, BIK, ARF6, APPL1

Corresponding total number of TFBS:
(For each gene listed above, the total number of TFBS for any of the selected TFs, multiplied by the number of selected Gene ontology terms containing that gene)
192, 176, 140, 84, 66, 66, 60, 60, 56, 54, 48, 48, 45, 40, 36, 36, 33, 28, 28, 27, 27, 24, 22, 20, 20, 16, 16, 15, 15, 15, 15, 14, 14, 14, 14, 14, 13, 13, 13, 12, 12, 12, 12, 11, 11, 10, 10, 10, 9, 9, 8, 8, 8, 8, 8, 8, 8, 8, 7, 7, 7, 7, 6, 6, 6, 6, 6, 6, 6, 6, 6, 6, 5, 5, 5, 5, 5, 5, 5, 5, 5, 4, 4, 4, 4, 4, 4, 4, 4, 3, 3, 3, 3, 3, 3, 3, 3, 3, 2, 2, 2, 2, 2, 2, 2, 2, 2, 2, 2, 2, 2, 2, 2, 2, 2, 2, 2, 2, 2, 2, 1, 1, 1, 1, 1, 1, 1, 1, 1, 1, 1, 1, 1, 1, 1, 1, 1, 1, 1, 1, 1, 1, 1, 1, 1, 1, 1, 1, 1, 1, 1, 1, 1

Corresponding number of selected Gene ontology terms each gene is involved with:
(In the same order as above)
12, 11, 10, 6, 6, 6, 6, 12, 4, 6, 8, 4, 3, 5, 4, 4, 11, 2, 2, 3, 3, 2, 2, 5, 2, 1, 1, 1, 5, 1, 5, 1, 1, 1, 1, 1, 1, 1, 1, 3, 3, 1, 1, 1, 1, 1, 2, 2, 1, 3, 1, 1, 1, 1, 1, 1, 1, 1, 7, 1, 1, 1, 1, 3, 2, 1, 6, 2, 2, 3, 6, 6, 5, 1, 1, 5, 1, 1, 5, 1, 1, 4, 1, 1, 4, 4, 2, 1, 1, 3, 1, 3, 3, 1, 1, 1, 1, 1, 1, 2, 1, 1, 1, 1, 2, 1, 2, 1, 1, 1, 2, 1, 2, 2, 1, 2, 2, 1, 1, 1, 2, 1, 2, 1, 1, 1, 1, 1, 1, 1, 1, 1, 1, 1, 1, 1, 1, 1, 1, 1, 1, 1, 1, 1, 1, 1, 1, 1, 1, 1, 1, 1, 1

Corresponding number of selected TFs each gene is involved with:
(In the same order as above)
16, 16, 14, 14, 11, 11, 10, 5, 14, 9, 6, 12, 15, 8, 9, 9, 3, 14, 14, 9, 9, 12, 11, 4, 10, 16, 16, 15, 3, 15, 3, 14, 14, 14, 14, 14, 13, 13, 13, 4, 4, 12, 12, 11, 11, 10, 5, 5, 9, 3, 8, 8, 8, 8, 8, 8, 8, 8, 1, 7, 7, 7, 6, 2, 3, 6, 1, 3, 3, 2, 1, 1, 1, 5, 5, 1, 5, 5, 1, 5, 5, 1, 4, 4, 1, 1, 2, 4, 4, 1, 3, 1, 1, 3, 3, 3, 3, 3, 2, 1, 2, 2, 2, 2, 1, 2, 1, 2, 2, 2, 1, 2, 1, 1, 2, 1, 1, 2, 2, 2, 1, 2, 1, 1, 1, 1, 1, 1, 1, 1, 1, 1, 1, 1, 1, 1, 1, 1, 1, 1, 1, 1, 1, 1, 1, 1, 1, 1, 1, 1, 1, 1, 1, 1, 1

Gene ontology terms ranked according to mean of effect sizes in cluster:
insulin-like growth factor receptor activity | insulin-like growth factor receptor binding | insulin-like growth factor I binding | insulin binding | insulin-like growth factor binding | insulin receptor binding | insulin-like growth factor II binding | growth factor binding | insulin-like growth factor binding protein complex | glucose transport | glucose homeostasis | response to wounding | regulation of growth

Corresponding mean of effect sizes of each Gene ontology term:
(In the same order as above)
1.53, 1.44, 1.33, 1.26, 1.25, 1.16, 1.12, 1.11, 0.96, 0.94, 0.87, 0.80, 0.79

Corresponding total number of genes of each Gene ontology term:
(In the same order as above)
71, 173, 132, 33, 149, 20, 132, 93, 88, 209, 197, 26, 107

Corresponding number of genes of each Gene ontology term with at least one TFBS (of selected TFs):
(In the same order as above)
19, 40, 27, 11, 33, 10, 26, 20, 19, 56, 48, 7, 37

Corresponding total number of TFBS (of selected TFs) for each Gene ontology term (in all genes):
(In the same order as above)
141, 247, 182, 66, 196, 62, 183, 134, 126, 270, 251, 52, 185

Corresponding number of selected TFs each Gene ontology term is involved with:
(In the same order as above)
16, 16, 16, 16, 16, 16, 16, 16, 16, 16, 16, 16, 16

TFs ranked according to mean of effect sizes in cluster:
VEGR_Q6, VWT1_Q6, V$MAZR_01, V$ACAAT_B, V$AP2GAMMA_01, V$AP2ALPHA_01, V$E2F1_Q3, V$AP2_Q6_01, V$E2F1_Q6, V$ETF_Q6, VMAZ_Q6, VHES1_Q2, V$AP2_Q6, V$E2F_Q2, V$AP2_Q3, V$E2F1_Q3_01

Corresponding mean of effect sizes of each TF:
(In the same order as above)
1.83, 1.65, 1.56, 1.28, 1.23, 1.20, 1.19, 1.06, 1.05, 1.02, 0.99, 0.99, 0.94, 0.88, 0.84, 0.22

Corresponding total number of TFBS for each TF (genome-wide):
(In the same order as above)
695, 729, 705, 692, 695, 686, 709, 690, 691, 699, 734, 690, 688, 714, 683, 680

Corresponding total number of TFBS for each TF (in all genes in selected Gene ontology terms):
(In the same order as above)
155, 160, 133, 148, 128, 126, 130, 126, 133, 127, 124, 134, 129, 129, 113, 100

Corresponding number of genes (of selected Gene ontology terms) each TF is involved with:
(In the same order as above)
52, 56, 43, 54, 50, 50, 52, 44, 52, 50, 48, 53, 50, 56, 46, 35

Corresponding number of selected Gene ontology terms each TF is involved with:
(In the same order as above)
13, 13, 13, 13, 13, 13, 13, 13, 13, 13, 13, 13, 13, 13, 13, 13

Cluster for columns 228 to 245, rows 262 to 265

Gene ontology terms

low-density lipoprotein particle | cholesterol absorption | cholesterol esterification | low-density lipoprotein receptor activity | cholesterol homeostasis | cholesterol metabolic process | cholesterol efflux | cholesterol transport | reverse cholesterol transport | lipoprotein metabolic process | high-density lipoprotein particle | phosphatidylcholine-sterol O-acyltransferase activity | lipid homeostasis | lipid transport | lipoprotein lipase activity | acyltransferase activity | chylomicron | lipoprotein biosynthetic process

TFs

V$BLIMP1_Q6 | V$CACCCBINDINGFACTOR_Q6 | V$FXR_Q3 | V$FXR_IR1_Q6

Information

All related TFs:
(List of all TFs that are related to any of the PWMs)

Blimp-1, CACCC-binding, FOR1, FOR2, FXR, FXR-alpha, FXR:RXR-alpha

Ranked gene list:
(All genes of the selected Gene ontology terms with hits of any of the selected TFs, ranked according to the total number of TFBS)
PLTP, APOA1, TNFSF13, NLRP3, APOB, CETP, NR1H3, APOC3, INS, APOA2, SCARB1, NR0B2, RXRB, DHCR7, MT1B, APOM, SCAP, PPARD, PER1, APP, ABCA2, MGAT1, HDLBP, SLC25A22, PON2, MUC17, LEP, KRT81, GOT2, GNAI2, FASN, CYP11A1, COG2, SNX17, PPAT, PNPLA6, MLXIPL, LRP2, FABP6, DGAT1, CD68, ANXA2, ANGPTL4, TRERF1, TNF, TFAP2A, SLC27A1, SLC16A1, SKIV2L, SFTPC, SCARF1, PITPNA, PCTP, PCSK7, OSM, MST1, MGLL, LTB, LRPAP1, LDLRAP1, HBA2, GALNS, FOXA2, FGF21, FABP3, ENPP7, DAB2, CPT1A, COL4A3BP, COASY, CDT1, ADIPOR2, ACTB

Corresponding total number of TFBS:
(For each gene listed above, the total number of TFBS for any of the selected TFs, multiplied by the number of selected Gene ontology terms containing that gene)
48, 36, 20, 18, 18, 17, 16, 16, 15, 13, 12, 12, 8, 8, 7, 7, 6, 6, 6, 6, 5, 4, 4, 3, 3, 3, 3, 3, 3, 3, 3, 3, 3, 2, 2, 2, 2, 2, 2, 2, 2, 2, 2, 1, 1, 1, 1, 1, 1, 1, 1, 1, 1, 1, 1, 1, 1, 1, 1, 1, 1, 1, 1, 1, 1, 1, 1, 1, 1, 1, 1, 1, 1

Corresponding number of selected Gene ontology terms each gene is involved with:
(In the same order as above)
16, 18, 10, 9, 18, 17, 8, 16, 15, 13, 12, 6, 2, 4, 7, 7, 6, 6, 2, 6, 5, 2, 4, 1, 3, 1, 3, 3, 3, 1, 3, 3, 3, 1, 1, 1, 2, 2, 1, 2, 1, 1, 2, 1, 1, 1, 1, 1, 1, 1, 1, 1, 1, 1, 1, 1, 1, 1, 1, 1, 1, 1, 1, 1, 1, 1, 1, 1, 1, 1, 1, 1, 1

Corresponding number of selected TFs each gene is involved with:
(In the same order as above)
3, 2, 2, 2, 1, 1, 2, 1, 1, 1, 1, 2, 4, 2, 1, 1, 1, 1, 3, 1, 1, 2, 1, 3, 1, 3, 1, 1, 1, 3, 1, 1, 1, 2, 2, 2, 1, 1, 2, 1, 2, 2, 1, 1, 1, 1, 1, 1, 1, 1, 1, 1, 1, 1, 1, 1, 1, 1, 1, 1, 1, 1, 1, 1, 1, 1, 1, 1, 1, 1, 1, 1, 1

Gene ontology terms ranked according to mean of effect sizes in cluster:
cholesterol efflux | cholesterol absorption | reverse cholesterol transport | high-density lipoprotein particle | cholesterol transport | low-density lipoprotein particle | phosphatidylcholine-sterol O-acyltransferase activity | lipoprotein lipase activity | cholesterol homeostasis | lipoprotein metabolic process | lipid transport | cholesterol metabolic process | low-density lipoprotein receptor activity | chylomicron | lipid homeostasis | cholesterol esterification | lipoprotein biosynthetic process | acyltransferase activity

Corresponding mean of effect sizes of each Gene ontology term:
(In the same order as above)
2.37, 2.19, 2.09, 2.04, 1.82, 1.75, 1.69, 1.64, 1.60, 1.59, 1.31, 1.26, 1.19, 1.16, 1.07, 0.95, 0.93, 0.72

Corresponding total number of genes of each Gene ontology term:
(In the same order as above)
93, 65, 76, 35, 96, 41, 34, 53, 110, 91, 85, 102, 112, 56, 123, 53, 25, 44

Corresponding number of genes of each Gene ontology term with at least one TFBS (of selected TFs):
(In the same order as above)
23, 12, 17, 11, 21, 9, 7, 15, 20, 18, 17, 20, 26, 13, 26, 9, 6, 7

Corresponding total number of TFBS (of selected TFs) for each Gene ontology term (in all genes):
(In the same order as above)
35, 19, 26, 15, 31, 13, 11, 23, 31, 23, 23, 27, 31, 16, 33, 12, 7, 12

Corresponding number of selected TFs each Gene ontology term is involved with:
(In the same order as above)
4, 4, 4, 4, 4, 4, 4, 4, 4, 4, 4, 4, 4, 4, 4, 4, 3, 4

TFs ranked according to mean of effect sizes in cluster:
V$CACCCBINDINGFACTOR_Q6, V$BLIMP1_Q6, VFXR_IR1_Q6, VFXR_Q3

Corresponding mean of effect sizes of each TF:
(In the same order as above)
2.46, 1.55, 1.11, 0.95

Corresponding total number of TFBS for each TF (genome-wide):
(In the same order as above)
684, 679, 659, 666

Corresponding total number of TFBS for each TF (in all genes in selected Gene ontology terms):
(In the same order as above)
122, 96, 89, 81

Corresponding number of genes (of selected Gene ontology terms) each TF is involved with:
(In the same order as above)
29, 21, 25, 24

Corresponding number of selected Gene ontology terms each TF is involved with:
(In the same order as above)
18, 18, 18, 17

Cluster for columns 385 to 390, rows 50 to 113

Gene ontology terms

T cell differentiation in the thymus | B cell differentiation | lymphocyte differentiation | tissue development | cell recognition | thyroid-stimulating hormone receptor activity

TFs

V$TBP_Q6 | V$NKX62_Q2 | V$OTX_Q1 | V$PBX1_01 | V$SRY_01 | V$HMGIY_Q3 | V$OCT1_06 | V$TBP_01 | V$TEF_Q6 | V$FOXJ2_02 | V$AFP1_Q6 | V$OCT1_03 | V$SRY_02 | V$FOXO1_01 | V$FOXO3A_Q1 | V$FOXO4_01 | V$IRF1_Q6 | V$CEBPGAMMA_Q6 | V$CDC5_01 | V$BRN2_01 | V$CDX_Q5 | V$POU1F1_Q6 | V$OCT1_04 | V$TATA_C | V$FREAC7_01 | V$PIT1_Q6 | V$IPF1_Q4 | V$OCT1_Q6 | V$OCT1_05 | V$OCT_Q6 | V$OCT1_Q5_01 | V$OCT1_B | V$OCT_C | V$GATA1_05 | V$FAC1_01 | V$TITF1_Q3 | B$CRP_C | V$GATA_Q6 | V$GATA6_01 | V$TCF4_Q5 | V$LEF1_Q2 | V$BRCA_01 | V$AREB6_04 | V$SOX10_Q6 | V$DBP_Q6 | V$CEBP_01 | V$AP3_Q6 | V$STAT6_01 | V$STAT5A_03 | V$STAT4_01 | V$STAT5A_04 | V$STAT1_03 | V$CEBP_Q3 | V$PAX2_02 | V$HMGIY_Q6 | V$HOXA4_Q2 | V$TST1_01 | V$NFAT_Q6 | V$NFAT_Q4_01 | V$CEBP_Q2_01 | V$CEBPA_01 | V$CEBP_Q2 | V$CEBPB_01 | V$GATA4_Q3

Information

All related TFs:
(List of all TFs that are related to any of the PWMs)
AFP1, ANF-2, AP-3, BRCA1, BRCA1:USF2, Brn1, C/EBP, C/EBPalpha, C/EBPalpha(p20), C/EBPalpha(p30), C/EBPbeta, C/EBPbeta(LAP), C/EBPbeta(p20), C/EBPbeta(p34), C/EBPbeta(p35), C/EBPdelta, C/EBPepsilon, C/EBPgamma, CDC5L, CDX2, Cdx-1, Cdx-2, Cdx-3, DBP, FAC1, FOXJ2, FOXL1, FOXO1, FOXO4, GATA-1, GATA-1A, GATA-2, GATA-3, GATA-4, GATA-5, GATA-5A, GATA-5B, GATA-6, GATA-6A, GATA-6B, HMG, HMG-Y, HMGI-C, HOXA4, IPF1, IRF-1, LEF-1,

LEF-1S, NF-AT, NF-AT1, NF-AT1C, NF-AT2, NF-AT3, NF-AT4, Nkx2-1, Nkx6-2, OCA-B, Oct-1, Oct-10, Oct-2, Oct-2.1, Oct-2.2, Oct-2.5, Oct-9, Oct-R, Octa-factor, Otx1, Otx2, POU2F1, POU2F1a, POU2F1b, POU2F1c, POU2F2, POU2F2B, POU2F3, POU3F1, POU3F2, POU4F1, POU4F1(l), POU5F1, POU5F1A, POU5F1B, POU5F1C, Pax-2, Pax-2.1, Pax-2.2, Pax-2a, Pax-2b, Pbx1a, Pit-1, Pit-1-xbb3, Pit-1A, Pit-1B, SRY, STAT1, STAT1alpha, STAT4, STAT5A, STAT6, Sox10, TBP, TCF-1, TCF-1(P), TCF-3, TCF-4, TEF, TEF-xbb1, TFIID, ZEB, oct-B2, oct-B3, octamer-binding

Ranked gene list:
(All genes of the selected Gene ontology terms with hits of any of the selected TFs, ranked according to the total number of TFBS)
CD44, BCL6, IKZF1, PRDM1, CD4, NR3C2, BCL2, CTLA4, PTPRC, RERE, ETS1, GNAS, TG, CXCR4, RUNX1, LEF1, CD68, ID2, BACH2, EBF1, MYB, FOXP1, NP, FLI1, VIM, TCF7, ARL4C, PAX5, CD2, MAML2, GFI1, CTNNB1, CCR7, CD226, TNF, CD69, CD247, PTMA, GRK5, CD96, CADM1, TAP1, LPIN1, IL16, PCNA, MYC, BCL2L1, TAPBP, SILV, IL2RA, TNFSF13, ITGA5, STAT5B, IKZF3, ID3, WNT10B, STAT3, LCK, TAP2, RUNX3, POU2F1, ATM, LBX1, UBC, ITGB1, ITGA4, CD27, SLC3A2, PRH1, DR1, KRAS, FAS, SELL, POU2F2, IL2, TLR1, REL, ITGA6, ENO2, STAT6, ITGB2, CNTNAP1, TNC, STAT5A, STAT4, SSFA2, PRDM2, IL6R, CXCR5, CD5, XBP1, LBR, FOS, RBMS1, CD79A, BMP2, TSHR, TCF4, TBX21, RBM45, RAG2, RAG1, LYN, IL6, IL10, EVI1, CSNK1A1, CDR2, CD40, ABL2, TNFSF13B, PRLR, CKAP4, TAOK3, MYCN, IRF4, HCST, GZMA, EDC4, ALPK1, TP63, TH1L, SH3BP5, NCR3, MAG, LY75, KIRREL3, IL2RB, GPD2, EEF2, CD7, CD52, CD28, C1R, ATP6V1H, TNFRSF25, TMPO, TFRC, TAL1, STRN3, SNRPA, RTEL1, RPL22, RNH1, POU2AF1, POLA1, NOTCH1, MSC, MPG, MMP11, MBP, LSAMP, KLRA1, IRF6, ID1, HSPD1, HRAS, HLA-C, HLA-B, GPR160, FLNB, ERBB2, DST, CGA, CDX2, CDH4, CD79B, CD40LG, CD37, BRWD2, BLNK, BCL10, ADA, VTN, ULBP2, UBE2D1, TSLP, TP53, SPIB, SLC4A3, SEPN1, RPS27A, RHO, PRKCDBP, PPARG, PAX8, OLIG2, NUDT6, NRCAM, NCAN, MYOG, MUC1, KRT31, ITIH4, IL3, FGF2, FGB, EWSR1, ENTPD1, EGF, DSG3, DLEU7, CNTNAP2, CLTA, CEACAM5, CD86, CALCA, C6, BCR, ADK

Corresponding total number of TFBS:
(For each gene listed above, the total number of TFBS for any of the selected TFs, multiplied by the number of selected Gene ontology terms containing that gene)
282, 186, 177, 120, 104, 98, 96, 90, 88, 62, 62, 61, 60, 60, 59, 59, 56, 54, 53, 52, 50, 50, 48, 46, 44, 44, 43, 40, 40, 37, 37, 37, 36, 35, 34, 33, 32, 31, 31, 30, 30, 29, 29, 28, 27, 27, 27, 26, 26, 26, 25, 25, 24, 24, 24, 22, 22, 22, 20, 20, 20, 20, 18, 17, 17, 15, 15, 14, 14, 14, 13, 13, 12, 12, 12, 11, 11, 11, 11, 10, 10, 10, 9, 9, 9, 9, 9, 9, 9, 9, 8, 8, 8, 7, 7, 7, 6, 6, 6, 6, 6, 6, 6, 6, 6, 6, 6, 6, 6, 5, 5, 5, 4, 4, 4, 4, 4, 4, 4, 3, 3, 3, 3, 3, 3, 3, 3, 3, 3, 3, 3, 3, 3, 3, 2, 2, 2, 2, 2, 2, 2, 2, 2, 2, 2, 2, 2, 2, 2, 2, 2, 2, 2, 2, 2, 2, 2, 2, 2, 2, 2, 2, 2, 2, 2, 2, 2, 2, 2, 2, 2, 1, 1, 1, 1, 1, 1, 1, 1, 1, 1, 1, 1, 1, 1, 1, 1, 1, 1, 1, 1, 1, 1, 1, 1, 1, 1, 1, 1, 1, 1, 1, 1, 1, 1, 1, 1

Corresponding number of selected Gene ontology terms each gene is involved with:
(In the same order as above)
6, 3, 3, 2, 4, 2, 3, 2, 4, 2, 1, 1, 3, 1, 1, 1, 2, 1, 1, 2, 2, 1, 2, 1, 1, 1, 1, 2, 4, 1, 1, 1, 1, 1, 1, 1, 1, 1, 1, 1, 1, 1, 1, 1, 1, 3, 1, 1, 1, 2, 5, 1, 1, 1, 1, 1, 1, 2, 1, 1, 1, 1, 1, 1, 1, 3, 3, 2, 1, 1, 1, 1, 2, 2, 4, 1, 1, 1, 1, 1, 1, 2, 1, 3, 1, 1, 1, 1, 1, 1, 3, 2, 2, 1, 1, 1, 1, 1, 1, 1, 1, 3, 3, 1, 2, 2, 1, 1, 1, 3, 2, 1, 1, 1, 1, 1, 2, 1, 1, 2, 1, 1, 3, 1, 1, 1, 1, 1, 3, 1, 1, 3, 1, 3, 1, 1, 1, 2, 1, 1, 1, 1, 1, 1, 1, 1, 1, 1, 1, 1, 1, 1, 1, 1, 1, 2, 1, 1, 1, 1, 1, 2, 1, 2, 1, 1, 1, 1, 1, 2, 2, 1, 2, 1, 2, 1, 1, 1, 1, 1, 1, 1, 1, 1, 1, 1, 1, 1, 1, 1, 1, 1, 1, 1, 1, 1, 1, 1, 1, 1, 1, 1, 1, 1, 1, 1, 1, 1, 1, 1, 1, 1, 1, 1, 1, 1

Corresponding number of selected TFs each gene is involved with:
(In the same order as above)
47, 62, 59, 60, 26, 49, 32, 45, 22, 31, 62, 61, 20, 60, 59, 59, 28, 54, 53, 26, 25, 50, 24, 46, 44, 44, 43, 20, 10, 37, 37, 37, 36, 35, 34, 33, 32, 31, 31, 30, 30, 29, 29, 28, 27, 9, 27, 26, 26, 13, 5, 25, 24, 24, 24, 22, 22, 11, 20, 20, 20, 20, 18, 17, 17, 5, 5, 7, 14, 14, 13, 13, 6, 6, 3, 11, 11, 11, 11, 10, 5, 10, 3, 9, 9, 9, 9, 9, 9, 3, 4, 4, 8, 7, 7, 7, 6, 6, 6, 6, 2, 2, 6, 3, 3, 6, 6, 6, 2, 3, 5, 5, 5, 4, 4, 2, 4, 4, 2, 4, 3, 1, 3, 3, 3, 3, 3, 1, 3, 3, 1, 3, 1, 3, 3, 2, 1, 2, 2, 2, 2, 2, 2, 2, 2, 2, 2, 2, 2, 2, 2, 2, 1, 2, 2, 2, 2, 2, 1, 2, 1, 2, 2, 2, 2, 2, 1, 1, 2, 1, 2, 1, 1, 1, 1, 1, 1, 1, 1, 1, 1, 1, 1, 1, 1, 1, 1, 1, 1, 1, 1, 1, 1, 1, 1, 1, 1, 1, 1, 1, 1, 1, 1, 1, 1, 1, 1, 1, 1

Gene ontology terms ranked according to mean of effect sizes in cluster:
B cell differentiation | T cell differentiation in the thymus | thyroid-stimulating hormone receptor activity | lymphocyte differentiation | cell recognition | tissue development

Corresponding mean of effect sizes of each Gene ontology term:
(In the same order as above)
1.44, 1.23, 1.23, 1.18, 0.96, 0.78

Corresponding total number of genes of each Gene ontology term:
(In the same order as above)
132, 67, 39, 80, 152, 134

Corresponding number of genes of each Gene ontology term with at least one TFBS (of selected TFs):
(In the same order as above)
67, 39, 25, 49, 65, 48

Corresponding total number of TFBS (of selected TFs) for each Gene ontology term (in all genes):
(In the same order as above)
911, 663, 394, 655, 669, 570

Corresponding number of selected TFs each Gene ontology term is involved with:
(In the same order as above)
64, 64, 64, 64, 64, 64

TFs ranked according to mean of effect sizes in cluster:
V$OCT1_06, V$OCT1_05, V$LEF1_Q2, V$OCT1_Q5_01, V$NFAT_Q6, V$OCT_Q6, V$IRF1_Q6, V$HMGIY_Q6, V$SOX10_Q6, V$AFP1_Q6, VOTX_Q1, VOCT1_B, V$GATA4_Q3, V$STAT6_01, V$TCF4_Q5, V$DBP_Q6, V$CEBP_Q2_01, V$POU1F1_Q6, V$HMGIY_Q3, V$CEBP_Q3, V$NKX62_Q2, V$HOXA4_Q2, VCDX_Q5, VCEBP_Q2, VTBP_Q6, VBRN2_01, V$IPF1_Q4, V$PAX2_02, V$NFAT_Q4_01, V$PBX1_01, V$CEBP_01, V$GATA_Q6, V$OCT1_04, V$FOXJ2_02, V$CEBPA_01, V$SRY_01, V$OCT1_Q6, V$STAT5A_03, V$STAT4_01, V$STAT1_03, V$TST1_01, V$AREB6_04, VSRY_02, VCEBPGAMMA_Q6, BCRP_C, VOCT1_03, V$GATA6_01, V$GATA1_05, V$CEBPB_01, V$OCT_C, VTBP_01, VPIT1_Q6, V$FOXO3A_Q1, V$TEF_Q6, V$AP3_Q6, V$STAT5A_04, V$FAC1_01, V$TATA_C, V$FOXO1_01, V$TITF1_Q3, V$FOXO4_01, V$CDC5_01, V$FREAC7_01, V$BRCA_01

Corresponding mean of effect sizes of each TF:
(In the same order as above)
2.17, 2.13, 1.97, 1.91, 1.84, 1.78, 1.68, 1.66, 1.64, 1.58, 1.56, 1.56, 1.55, 1.54, 1.52, 1.52, 1.50, 1.49, 1.48, 1.44, 1.42, 1.41, 1.41, 1.40, 1.39, 1.38, 1.38, 1.35, 1.34, 1.33, 1.29, 1.23, 1.18, 1.17, 1.15, 1.14, 1.13, 1.10, 1.09, 1.01, 1.00, 0.97, 0.97, 0.92, 0.90, 0.86, 0.80, 0.73, 0.67, 0.65, 0.64, 0.60, 0.60, 0.54, 0.54, 0.53, 0.52, 0.47, 0.37, 0.23, 0.20, 0.15, -0.01, -0.05

Corresponding total number of TFBS for each TF (genome-wide):
(In the same order as above)
717, 672, 723, 698, 719, 687, 697, 753, 729, 682, 701, 665, 713, 732, 716, 714, 716, 694, 716, 744, 698, 719, 707, 718, 723, 659, 704, 741, 736, 704, 740, 703, 662, 680, 711, 743, 679, 749, 743, 751, 730, 740, 693, 688, 716, 704, 722, 715, 703, 654, 685, 672, 689, 684, 741, 740, 722, 712, 680, 707, 696, 669, 669, 730

Corresponding total number of TFBS for each TF (in all genes in selected Gene ontology terms):
(In the same order as above)
77, 77, 80, 71, 79, 71, 71, 73, 70, 62, 64, 65, 66, 70, 72, 67, 66, 66, 67, 71, 60, 66, 57, 66, 64, 57, 57, 69, 68, 62, 67, 63, 57, 57, 62, 65, 63, 63, 64, 61, 61, 60, 57, 52, 56, 53, 57, 60, 50, 48, 47, 49, 49, 44, 51, 51, 51, 49, 44, 46, 51, 42, 39, 42

Corresponding number of genes (of selected Gene ontology terms) each TF is involved with:
(In the same order as above)
48, 51, 52, 49, 51, 49, 47, 50, 45, 41, 43, 43, 43, 46, 46, 43, 45, 44, 42, 48, 40, 46, 36, 43, 43, 37, 34, 47, 46, 41, 45, 43, 42, 39, 43, 44, 45, 43, 43, 41, 42, 43, 40, 37, 36, 37, 39, 40, 34, 35, 32, 33, 34, 32, 36, 38, 37, 34, 32, 33, 38, 29, 30, 35

Corresponding number of selected Gene ontology terms each TF is involved with:
(In the same order as above)
6, 6, 6, 6, 6, 6, 6, 6, 6, 6, 6, 6, 6, 6, 6, 6, 6, 6, 6, 6, 6, 6, 6, 6, 6, 6, 6, 6, 6, 6, 6, 6, 6, 6, 6, 6, 6, 6, 6, 6, 6, 6, 6, 6, 6, 6, 6, 6, 6, 6, 6, 6, 6, 6, 6, 6, 6, 6, 6, 6, 6, 6, 6, 6

Cluster for columns 391 to 538, rows 244 to 263

Gene ontology terms

actin binding | actomyosin | cell motility | establishment of cell polarity | leading edge | cytoskeleton | filamentous actin | actin cytoskeleton | actin filament polymerization | actin filament binding | actin filament | actin filament depolymerization | fibrinolysis | fibrinogen complex | coagulation | hemostasis | fibrinogen binding | platelet activation | protein secretion by the type III secretion system | collagen catabolic process | bone resorption | osteoclast differentiation | sensitization | nerve growth factor receptor activity | nerve growth factor receptor binding | mast cell activation | mast cell degranulation | leukotriene biosynthetic process | icosanoid metabolic process | arachidonic acid metabolic process | cyclooxygenase pathway | lipoxygenase pathway | prostaglandin biosynthetic process | prostanoid biosynthetic process | specific granule | phagocytosis | respiratory burst | piecemeal microautophagy of nucleus | neutrophil degranulation | neutrophil activation | neutrophil chemotaxis | cell surface | membrane raft | receptor complex | cell adhesion | cell migration | basement membrane | proteinaceous extracellular matrix | extracellular matrix | middle lamella-containing extracellular matrix | diapedesis | integrin binding | U-plasminogen activator receptor activity | endothelial cell activation | luteolysis | cell fraction | leukocyte adhesion | membrane attack complex | antibody-dependent cellular cytotoxicity | complement activation | opsonization | neutrophil apoptosis | leukocyte chemotaxis | crotonyl-CoA reductase activity | monocyte chemotaxis | localization of cell | chemotaxis | leukocyte migration | cell chemotaxis | positive chemotaxis | B cell activation | immunoglobulin production | B cell proliferation | immunoglobulin secretion | interferon-gamma production | T-helper 1 type immune response | response to host immune response | adaptive immune response | adaptive immune response based on somatic recombination of immune receptors built from immunoglobulin superfamily domains | chemokine production | interleukin-10 production | interleukin-12 production | cell maturation | MHC class I biosynthetic process | MHC class I protein binding | MHC class II biosynthetic process | MHC class II protein binding | antigen processing and presentation | immune response | type IV hypersensitivity | lymphocyte proliferation | tolerance induction | lymphocyte activation | interleukin-2 production | interleukin-2 receptor activity | T cell proliferation | interleukin-4 production | cytokine production | cytokine secretion | pathogenesis | interleukin-4 receptor activity | interleukin-5 production | evasion or tolerance of immune response of other organism during symbiotic interaction | tryptophan catabolic process | defense response to virus | innate immune response | tumor necrosis factor receptor activity | tumor necrosis factor receptor binding | positive regulation of NF-kappaB transcription factor activity | response to tumor necrosis factor | IkappaB kinase complex | NF-kappaB binding | interleukin-6 receptor activity | interleukin-1 beta production | interleukin-1 production | cytolysis | natural killer cell mediated cytotoxicity | natural killer cell receptor activity | naringenin-chalcone synthase activity | immature T cell proliferation in the thymus | eosinophil activation | leukocyte activation | monocyte activation | mucosal immune response | granulocyte macrophage colony-stimulating factor biosynthetic process | granulocyte macrophage colony-stimulating factor production | interleukin-1 receptor activity | interleukin-6 production | tumor necrosis factor production | macrophage activation | inflammatory response | response to lipopolysaccharide | negative regulation of inflammatory response | regulation of tumor necrosis factor production | negative regulation of tumor necrosis factor production | positive regulation of tumor necrosis factor production | interleukin-1 receptor antagonist activity | acute inflammatory response | granuloma formation |

lipopolysaccharide binding | cytokine biosynthetic process | interleukin-8 production | mRNA transcription | chronic inflammatory response | monocyte differentiation | hypersensitivity | hyaluronic acid binding | cytokine activity

TFs

V$FOXP3_Q4 | V$DR3_Q4 | V$PEBP_Q6 | V$AML_Q6 | V$PAX_Q6 | V$NRF2_Q4 | V$MAF_Q6_01 | V$AP1_Q6_01 | V$AP1_Q4_01 | V$AP1_Q6 | V$AP1_C | V$AP1_01 | V$AP1_Q2 | V$AP1FJ_Q2 | V$AP1_Q4 | V$AP1_Q2_01 | V$BACH2_01 | V$NFE2_01 | V$BLIMP1_Q6 | V$CACCCBINDINGFACTOR_Q6

Information

All related TFs:
(List of all TFs that are related to any of the PWMs)
AML1, AML1a, AML1b, AML1c, AML2, AML3, AML3-isoform1, AML3-isoform2, AP-1, Bach1, Bach1:MafK, Bach2, Blimp-1, CACCC-binding, CAR, CAR2:RXR-alpha, CAR:RXR-alpha, FOXP3, FosB, Fra-1, Fra-2, JunB, JunB:Fra-1, JunB:Fra-2, JunD, JunD:Fra-2, JunD:deltaFosB, LCR-F1, MAF, Maf, MafB, MafF, MafG, MafG:MafG, MafK, NF-E2, Nrf1, Nrf1:MafG, Nrf1:MafK, Nrf2, Nrf2:MafG, Nrf2:MafK, Nrf3, Nrf3:MafK, PEBP2, PEBP2alpha, PEBP2alphaA1, PEBP2alphaA2, PEBP2alphaB1, PEBP2alphaB2, PEBP2beta, PEBP2beta1, PEBP2beta2, PEBP2beta3, PXR-1, PXR-1:RXR-alpha, PXR-1A, PXR-1A:RXR-alpha, PXR-1A:RXR-beta, PXR-2, PXR-2:RXR-alpha, Pax-1, Pax-2, Pax-2a, Pax-3, Pax-4a, Pax-4c, Pax-5, Pax-6, Pax-8, Pax6-1, RUNX2-isoform2, RUNX3, RXR-alpha, RXR-beta, Runx3, SXR:RXR-alpha, VDR, YAP1, c-Fos, c-Jun, c-Jun:FosB, c-Jun:JunD, c-Jun:c-Fos, c-Maf, deltaFosB, v-Maf

Ranked gene list:
(All genes of the selected Gene ontology terms with hits of any of the selected TFs, ranked according to the total number of TFBS)
TNF, AKT1, LTA, CSF2, ISYNA1, LTB, TNFRSF1A, CSF1, CD44, ITGB2, CCR7, RELA, CD68, PTPN6, IL2RB, NOS2, IRF1, IL3, IL2RA, LCK, FASN, CDKN1A, PSAP, CD4, TNFRSF4, ISG20, VIM, IL6, TLR9, VASP, SHC1, LBR, SQSTM1, MTHFR, IL23A, CTSD, TNFSF13, VAV1, HGS, TNFRSF18, CD63, ITGAX, MMP9, CSK, PLCG1, MARK2, IL11, MAP2K3, HSPA9, ZYX, RHOA, GPR132, CXCR4, TNFRSF1B, PXN, DUSP2, ICAM2, OSM, MAPK14, LMNA, IL6R, CISH, NFKBIA, SLC2A4RG, SSSCA1, PER1, JUNB, ITGA5, ILK, CALM1, FLNA, DLG4, ARHGEF2, HLA-E, FLNC, TG, PTK2B, PFN1, PSMB8, GAPDH, STAT3, SOCS1, S100A4, PDLIM7, EPB41, TBX21, RASSF5, PSMP, VEGFA, SFTPC, PRAP1, ZAP70, CXCR5, IRF6, CAPG, TRAF2, IL10, STAT6, CD7, ACTB, LASP1, PTMA, TSPAN4, NR3C2, HMOX1, GPI, CCR10, ANXA2, IL2, CD2, ICAM1, DDR1, UBC, S1PR4, FGR, CALR, MAP3K14, PRDX2, PRDM1, ITGA7, IL7R, EZR, CNTNAP1, STAT1, NTRK1, NLRP3, MIF, ACTG1, PIK3CA, VCL, MAP2K1, CD55, SEMA3B, RARA, TNFAIP3, SLC3A2, PDGFB, MYO1C, CFL1, VTN, STAT5B, GIT1, PLAUR, PDE4A, NUP43, MAPK3, EFNA1, RHOC, PTPRCAP, SLC35A1, ITGB7, CD151, ITIH4, TCIRG1, SPHK1, NEU1, LIF, LGALS1, FHOD1, CTNNB1, CD40LG, ANXA6, SH3BP2, EGR1, TRAF3, PPP1R9B, PKN1, LSP1, LAG3, F3, DUSP1, CLEC11A, TNFRSF12A, S1PR1, CD82, PSMD9, IL17C, HBEGF, EPHB2, MAPKAPK2, GLTSCR2, SLC25A22, SOD1, GPR56, WDR1, FOSL1, NBPF1, MDK, FLII, FBRS, EDC4, CIC, BUD31, BCL2L1, ARF1, SCARB1, MYC, HCK, SLC9A3R1, SLC2A1, S1PR2, ROCK2, IKBKE, CD276, CAP1, ARL4C, WNK1, FOS, CD79A, VASN, TBK1, IL10RA, GATA3, ETS1, CD247, ADM, PLAT, RUNX1, RTN4, RAPGEF1, RAB5A, PLEC1, PIK3CG, HLA-B, ENO1, CFLAR, STK16, S100A6, PSTPIP1, IRAK1, IL16, FLI1, CLCF1, TNFRSF9, S100A9, PIK3R2, MYD88, ETV5, AHNAK, ABHD14B, TRADD, TNIP1, TNFRSF13C, SOS1, SFN, RHOG, PDGFRB, NFKB2, HRAS, GADD45B, FYN, FERMT3, CD81, BAX, ALCAM, TGFBR2, STAT5A, LCP2, CRK, VEGFB, UBASH3B, SELPLG, RASGRP2, MBP, LPAR2, GALT, FLJ32987, ECE1, ATF3, PTGDS, PLA2G6, LYN, CORO7, CDK5, CALM3, RAC3, PSMB10, NOS3, MUC1, LTBP3, LAMA3, GYLTL1B, FLT3LG, COL6A1, BCR, ARAP3, SLC25A10, RXRB, PLEKHF1, MMP11, LMLN, FOXP3, CTLA4, CRKL, CRIP1, BAK1, TLR3,

RPLP2, PLAU, NFKBIE, MAPRE3, DLX2, DLEC1, CLU, CEBPE, CASP9, TLX2, SLA, PTGER4, POLD4, ITGA6, ITGA3, ICOSLG, ERBB2, CXCR3, BAG3, TRIP6, IL4R, ERBB3, CCL3, UNC84B, TLR5, TCN2, STX1A, STOML2, SPI1, SERPINE1, S100A10, PHB2, PDLIM4, NOLC1, MED25, LTC4S, LRP1, JAK3, HYAL3, HSPA1A, HIVEP2, GCLC, FURIN, CORO1A, COL7A1, COL11A2, CD9, CD27, AMBP, KNG1, VWF, TPM3, TLN1, SDCBP2, RPS6KA1, PIK3R1, IGL@, GALNT6, EHD1, CDC123, ANGPT2, XYLT1, VILL, SORBS3, NOD2, NGEF, HCLS1, EPB41L1, CTRL, CRTC2, BMP1, BCL2, VAMP8, TNFRSF6B, SLC19A2, SH2B3, RND1, RAPGEF3, PSMB9, PLEKHG6, MBNL1, LAMP1, L1CAM, ITGB3, HDAC3, FEV, CCND2, BCL6, AXL, AGRN, ZFP36, TNFRSF8, SOCS3, RAC2, ICOS, TSLP, TPM1, TNFRSF14, TIMP1, TGFB3, TAP1, SRF, P2RX7, ORAI1, NTRK3, MAP3K1, MAG, LTBP4, LTBP2, LRDD, LOXL1, IRF2, IKBKG, GYPC, GTPBP1, FADD, EPHA2, DLGAP1, CTDSP1, CD37, CCR4, CANX, BCL9L, ACTN1, RALGDS, JAK2, DGKZ, CCL21, CCL17, BTK, ARHGEF1, ZC3H12A, TNK2, TGFB2, SERPINH1, SCRIB, PTPN7, PSMA5, PARVB, PARD6A, MPRIP, MAL, LY96, LPXN, ITGAE, HYAL2, HYAL1, HIC1, DUSP28, DBNL, CD274, CCR6, ARHGAP1, AQP2, ABCB6, WNT1, TNPO1, SWAP-70, SSH1, SLC9A5, RUNX2, RIPK3, RIN1, PNPLA2, PDCD1, LHX1, INS, HSPB6, HSPA1B, FUT7, CYFIP2, CTSZ, COL9A3, CD300A, ABCB9, UNK, ULBP1, TUBGCP2, TNFSF13B, TGFB1I1, SLC9A1, PTGER3, PNKD, PDLIM2, PDGFA, NTF3, NOD1, MYL6, MIP, MGMT, LTB4R2, GUK1, DKFZp779C0757, CXCL6, CTNNA1, CRIP2, CNN1, CAST, C14orf49, BPI, BCAN, ARHGDIA, ADAM15, TUBB2C, TUBA4A, TP53, TFAP2A, RAB3GAP1, NR2C2, KCNIP2, GZMA, GRLF1, BHLHB2, BCL3, ARHGEF7, AQP3, ADIPOQ, ZC3H12D, ZBP1, VWA1, VDR, TNFRSF25, THY1, TACC3, SMPD2, SMAD7, SLC19A1, SEMA4D, RUNX3, PVRL1, POMGNT1, PLXNA1, OSGEP, NCR3, MMP13, MCAM, JUND, GRB10, GABARAP, EPB49, ENTPD2, EMILIN1, DPAGT1, DHCR7, DES, DCTN3, CREBBP, CPA6, COL17A1, CLCN3, CD3D, CALCA, C1QBP, APOBEC3A, AGER, ADCY7, ACD, NPHS1, MTTP, MST1, MME, MGAT1, MAZ, KCNN4, KAT5, ITGA2B, IL27, CREB3, BGLAP, ATXN1, ARID3B, ACTN4, ZMYND10, VDAC1, UNC13D, TPP1, TNXB, TNFRSF10B, THPO, TAPBP, SSFA2, SORL1, SCGB1A1, S100A12, RPL17, PTPN22, PTH1R, PSMC5, PSMC2, PAX6, PAX5, NOS1, NNAT, NFATC2, NFATC1, NCK1, MC1R, MBTPS1, MAPK12, MAP3K5, MAP3K12, MAP1A, MALT1, MAF, LAMC1, ITGB4, HSP90AA1, HSH2D, HIF1A, GALNS, FKBP4, EDN2, DMPK, DAPP1, CXCR6, CIITA, CD3G, BMP2, APOE, APOA1, ADA, VCAN, TUBB, TM7SF2, SPHK2, SDC4, RREB1, PTPRN, PPP2CA, PARVA, NINJ1, MYO1B, MGEA5, MAPK11, LGALS9, KSR1, KRT14, H2AFX, GFI1, FHL3, DYNC1H1, DUSP14, CORO1B, CLDN1, CDC42SE1, CCL27, ARSA, APOM, AP2M1, VIPR1, UTRN, TTYH2, TRPV3, TNNI2, TIPARP, SEPT1, S100P, RTN2, RHOD, RB1CC1, RASSF1, PTPN1, PLXNB1, PIGR, PEPD, PCYT1A, NR4A2, NOXO1, NCKIPSD, MYH9, MYB, MUC17, MRC2, MPZ, MAP3K11, MAP2K7, KRT19, KRT18, KCNA2, IRF9, IQGAP2, IL2RG, IL19, IGSF8, IGFBP7, HMMR, FLNB, ENO2, EMD, ELMO1, DBN1, CYBA, CSF3R, COL2A1, CIB1, CD72, CARD11, CAPN10, CAMK2G, C13orf15, ARHGEF12, APOB, ACP5, ACLY, ACCN2, ZEB2, XBP1, TNR, TBXA2R, TAP2, SH2D3C, SH2D3A, SERPINF1, SDC3, RTN4RL2, RHOB, RASA1, RAB7A, PPP1R1B, POLR2A, PLA2G15, PIM1, PARD3, NTN1, MYO1A, LDLR, IRS1, IGF2, IGF1R, HAVCR2, GTF3A, GPC1, GLI1, FGL2, ECSIT, DNM2, COL18A1, CDSN, CD160, C1R, BMP4, AGPAT1, ADRBK1, ADD3, WDR26, TUBB2A, TSTA3, TSPO, TPX2, TNFSF15, TNFSF14, TMSB4X, TH, TBX5, STRA13, SSH2, SPATA2, SH2D2A, SERPINA1, SEC24D, RTEL1, RPS2, RAP2B, PSME2, PSEN1, PRKCD, PRIM2, PRF1, PPP3CA, PPP2R4, PIP5K1C, PIGW, PELP1, PAK6, P4HTM, NR4A1, NOTCH1, MT2A, MRPL28, MPP5, MAPT, LMAN2, KRT31, KLF6, KCNJ5, IVL, ISG15, IRF4, HSPA8, HLA-DQB1, HAS3, GRM2, GPT, GORASP2, GNAS, GMDS, G6PD, FTH1, FRAP1, FOSB, FBXO8, FAIM3, EPHA1, ELMO3, ELL, EFEMP2, DOK2, DLX4, DEF6, DCTN2, COL12A1, CGN, CDK9, CDK5R2, CDH11, CD97, CD52, CD180, CCRL2, ATN1, ARVCF, ARHGAP4, AFAP1, ADAMTS4, ACCN3, ZFPM1, VKORC1, TUBB3, TRAF5, TNFRSF10D, TJP2, TCF19, SYN1, SPINT1, SMTN, SLC29A3, SLC20A1, SERPING1, SEMA3F, RLF, RGS3, RALA, PPP1R12A, PPIB, PDE1B, NPR1, NPFF, NAT13, MMP19, MICA, MAPRE2, MAP4K1, LRP2, LAMA5, INPP5D, HDAC1, HCST, FOXO3, ETV4, ERCC1, EIF3I, EEF1A1, ECM1, DVL2, CUX1, CTDSP2, CADM4, CACNB3, BTLA, p63RhoGEF, VNN2, USF1, TRIP10, TRAF3IP2, TPM4, TNIK, TMEM8, TLN2, TCEA2, TAOK2, SVIL, SPOCK1, SNX1, SMURF1, SLC9A3, SLC4A2, SLC34A1, SKAP1, SH2B2, RPLP0, RHOH, RGS14, RFX1, RBPJ, RAB27A, PYCARD, PSMD1, PSD, PRPH, PRKCB, PPP1R14A, PPIH, POU3F1, POLR2H, PLXNC1, PCSK7, PCBD1, PARVG, NME1, NLE1, NFE2L2, MYST1, MX1, MMP28, MICAL1, MGC111011, MGAT5, MGAT3, MERTK, MDFIC, MATN4, MADD, LY9, LY6D, LIMS1, LEPREL2, IL21R, IL12A, HIP1R, HAX1, GZMM, GTPBP3, GSTP1, GSK3B, GRB7, GNB2L1,

GCNT1, FXYD5, FLOT2, FGF8, ETF1, ERAP2, EPHB4, EFNA4, EEF1A2, EDAR, DOK1, DNM1, DIAPH3, DHX58, DDX5, CTF1, CSRP1, CSE1L, CREM, CHRM1, CHMP2B, CERK, CDKN2D, CDKN1C, CD96, CD84, CD248, CASP6, BAP1, ARPC1B, ARID2, ARHGEF11, ARFIP2, AQP4, AOC3, ANTXR2, ANLN, ANGPTL4, ANG, ALOX5AP, AKR1B1, ADAR, ADAM28, ACSL6, ACSL5, ACHE, ABLIM1, VWA2, VCP, VAC14, UBE2K, TUSC2, TRPC4AP, TREX1, TRAIP, TRAF4, TNN, TMSB10, TMBIM4, TIAL1, TANK, TAF8, STRN3, SOX9, SLC7A5, SLC7A1, SIGIRR, SEMA7A, SEC61A1, SDHB, S100A11, S100A1, RND2, RNASEL, RAP2A, RAB25, PSME3, PRSS16, PRKCE, POLG, PODN, PLXNB2, PLEKHO1, PIN1, PICK1, PDIA2, PCDH1, PAOX, PAFAH1B1, P2RY11, P2RX1, NUDT2, NRXN2, NDOR1, NAGA, MYO1F, MYO1E, MYH10, MYBPC3, MTMR11, MPV17, MFAP2, MDGA1, MATN3, MAP4K2, MAP2K6, LY6G5B, LRRC4, LRMP, LOXL3, LIMA1, KCNH2, IVNS1ABP, ITPKB, IL20, IDUA, ICAM4, HPDL, HNRNPD, HLA-DMB, HIVEP3, HHIP, HBB, GRK6, GRIA2, GPX4, FOXP1, FLOT1, FLAD1, FKRP, FBXL15, EXTL2, ESPN, EFEMP1, DVL1, DLL1, DAAM1, CSDA, CRY1, COMP, CNP, CHRNB2, CDK4, CD99L2, CD53, CCDC88A, CASP7, CAPN3, CAPN2, BDKRB2, BCAR3, ATP2B4, ARC, APBB1, ANK1, AMH, ADAMTS8, ADAMTS12, ACP2, ACAP1, ZXDA, ZNF384, ZBTB16, WNT11, WARS, USO1, UROD, UNC84A, ULBP3, UCN, UBE2E3, TTYH3, TSC22D3, TRIM69, TRIM21, TNNI3, TNNC2, TNIP2, TNFSF12-TNFSF13, TNFSF12, TNFRSF11A, TMEM11, TINAGL1, SYNE2, SYF2, STK11, ST3GAL1, SREBF1, SPINT2, SPG11, SOST, SNCG, SMS, SLC9A3R2, SLC4A3, SLC2A12, SLC25A1, SLC16A1, SLC11A2, SKP1, SFRS2, SFRP4, SCP2, SCN5A, SART3, RTN4R, RPS6KB2, RPS6KA5, ROBLD3, RIPK4, RGS16, RGS12, REPS1, REM2, RAMP2, RAMP1, RAB40B, PTTG1, PTPN23, PTGIR, PTGFRN, PTGDR, PTCH1, PRPF31, PROZ, PRKAB1, PRCP, PPAP2C, POU2F2, PLEKHA1, PLD2, PKP1, PIGQ, PIAS4, PHGDH, PGP, PEG3, PDZK1, PDYN, PDCD6IP, PARK7, PAK3, PADI4, OPA1, OBSCN, NTF4, NRTN, NPPC, NP, NME3, NDEL1, NCOR2, NBPF3, MYBPH, MYBBP1A, MRO, MRLC3, MMRN2, MLL, MID2, MGC39830, MGC138368, MFN2, MFI2, METAP1, MED15, MCPH1, MBNL2, MAX, MAP3K7IP1, MAP2K4, MAP1S, MAN2A2, LY6E, LIMK2, LEPRE1, LEF1, KIAA1967, KCNJ11, KANK1, INTS6, IL25, IFITM3, ICAM5, HSPB9, HOXA10, HMOX2, HM13, HIVEP1, HEPACAM, HARS, GSR, GPSN2, GPR124, GPAA1, GMFG, GLG1, GIT2, GGPS1, GFM1, GAST, GAS8, GAS7, GALC, FYB, FUT8, FST, FANCG, EXOSC1, EXOC7, EXOC3, EXOC2, ESR2, ERBB2IP, EOMES, EIF4E, EIF2S1, EGR2, EFNA2, DSCR3, DPYSL3, DNAH1, DLAT, DEAF1, DAD1, CYFIP1, CTBP1, CRLF1, CPEB1, CNTFR, CMKLR1, CLIC4, CHPF, CHIC2, CHDH, CEBPD, CEBPA, CDT1, CDKN2C, CDK6, CDH15, CDC14A, CCL18, CCHCR1, CASK, CAPNS1, CA9, C5orf13, C1orf38, C1QL1, BTRC, BLMH, BCAP31, BAT1, ARTN, ARF5, APLNR, APEX1, AMD1, AKAP1, AIRE, AGPAT2, ADRB2, ADD1, ADAM33, ACTR1A, ACTC1, ACSS2, ZNF398, ZMYND11, YWHAQ, WTIP, WNT5B, WNT3A, WHSC1, WDR68, WARS2, VEZT, VDAC2, VAPA, UTS2R, USP14, USF2, UCP2, UBQLN1, UBA7, TWF1, TTBK1, TSPAN10, TSC1, TRPV2, TRERF1, TPT1, TPP2, TOR1B, TNNT1, TMOD4, TIFA, TGM4, TCOF1, TCEB2, SUFU, STX6, STRA8, SSR1, SSH3, SPRY2, SPAG7, SP140, SNRPG, SLC7A14, SLC35C1, SLC22A7, SLC22A11, SIGLEC12, SGMS1, SGK1, SFRS17A, SFRP5, SETD4, SERPINB1, SERBP1, SEMA4A, SEC62, SAR1A, S100A13, RYK, RSF1, RPS6KA2, RPS14, RPL36A, ROBO3, RNASEK, RGS2, RGL4, RASL12, RASGRP1, RAD52, RAD18, RAB43, RAB1A, PTP4A3, PTK6, PTBP1, PSMB7, PSMA7, PSENEN, PRKCI, PRDX1, PPARGC1B, PLXDC1, PLTP, PLEKHO2, PLEK2, PIP5K1A, PIGC, PHOX2B, PHF12, PDZK1IP1, PDZD3, PDX1, PCSK6, PCDH12, OTP, NUMBL, NSMAF, NSD1, NRD1, NPHP1, NPC1L1, NLK, NLGN3, NKX6-2, NGRN, NFKBIL1, NF1, NCAM2, NAV2, NARS, MYL3, MYH6, MYCL1, MX2, MVD, MRPS12, MPHOSPH6, MMEL1, MMACHC, MEN1, MCHR2, MBOAT7, MAN1A1, LY6G5C, LRRN1, LRP4, LRIG1, LPHN2, LOR, LONP1, LMNB2, LGALS4, LGALS3BP, LAT2, KRT85, KRT16, KLF10, KIRREL2, KEAP1, KCNK2, KCNJ4, KCNA1, IRAK1BP1, INSC, INHA, IL1F6, IL17RB, IGFBP6, IFITM1, IFIT2, IER3, HSPB7, HOXA13, HISPPD1, HBS1L, HBD, GSTM2, GSC, GRIN2D, GOLGA2, GLMN, GLIPR1, GCS1, GCLM, GBA, GABBR1, FUBP1, FOXF1, FLVCR2, FLJ21865, FIP1L1, FGD3, FGD2, FBXW7, FANCE, EZH2, EXOSC3, EVI1, ERCC4, ERCC2, ELF4, EIF2S2, EHF, EBF1, DYRK1A, DYNC1I2, DUSP11, DNM1L, DISC1, DICER1, DFNB31, DCLRE1C, DAXX, CYP27B1, CYP11A1, CUL5, CRYAA, CRTC1, CRLF2, CPZ, CPOX, CNPY3, CNBP, CLC, CHEK2, CDR2L, CDC42EP1, CDC37, CDC2L5, CD109, CARD8, C1orf9, BRAF, BMX, BLZF1, BIN2, BCS1L, BCL2A1, BATF, ATF4, ARID4B, ARHGEF19, ARHGAP10, AQP1, APBB2, ANTXR1, AKTIP, AKT3, AGPAT9, AGPAT4, ADRBK2, ABLIM2, ABCD1, AATF, AANAT

Corresponding total number of TFBS:

(For each gene listed above, the total number of TFBS for any of the selected TFs, multiplied by the number of selected Gene ontology terms containing that gene)
2096, 810, 700, 672, 546, 525, 507, 504, 500, 460, 432, 400, 364, 361, 344, 336, 330, 320, 320, 290, 270, 266, 261, 252, 250, 248, 234, 220, 217, 216, 216, 215, 210, 208, 208, 208, 204, 198, 195, 192, 189, 188, 185, 182, 180, 171, 171, 170, 170, 169, 165, 165, 165, 162, 162, 162, 160, 156, 156, 156, 154, 153, 152, 150, 144, 144, 144, 144, 144, 144, 143, 143, 143, 140, 132, 130, 130, 128, 126, 126, 124, 120, 120, 117, 117, 115, 112, 112, 111, 110, 110, 108, 108, 105, 105, 104, 103, 100, 100, 100, 99, 98, 96, 96, 96, 96, 96, 96, 94, 94, 93, 91, 90, 90, 90, 90, 84, 80, 80, 80, 80, 80, 80, 78, 78, 78, 78, 78, 76, 75, 75, 75, 72, 72, 70, 70, 70, 70, 70, 68, 68, 68, 66, 66, 66, 66, 66, 65, 65, 64, 63, 63, 62, 60, 60, 60, 60, 60, 60, 60, 60, 60, 57, 57, 56, 56, 56, 56, 56, 56, 56, 56, 55, 55, 55, 54, 54, 54, 54, 52, 52, 51, 50, 50, 49, 49, 48, 48, 48, 48, 48, 48, 48, 48, 48, 46, 46, 46, 45, 45, 45, 45, 45, 45, 45, 45, 44, 44, 44, 42, 42, 42, 42, 42, 42, 42, 41, 40, 40, 40, 40, 40, 40, 40, 40, 40, 39, 39, 39, 39, 39, 39, 39, 38, 38, 38, 38, 38, 38, 38, 36, 36, 36, 36, 36, 36, 36, 36, 36, 36, 36, 36, 36, 36, 36, 35, 34, 34, 34, 33, 33, 33, 33, 33, 33, 33, 33, 33, 33, 32, 32, 32, 32, 32, 32, 30, 30, 30, 30, 30, 30, 30, 30, 30, 30, 30, 28, 28, 28, 28, 28, 28, 28, 28, 28, 28, 27, 27, 27, 27, 27, 27, 27, 27, 27, 27, 26, 26, 26, 26, 26, 26, 26, 26, 26, 25, 25, 25, 25, 24, 24, 24, 24, 24, 24, 24, 24, 24, 24, 24, 24, 24, 24, 24, 24, 24, 24, 24, 24, 24, 24, 24, 24, 24, 24, 24, 23, 22, 22, 22, 22, 22, 22, 22, 22, 22, 22, 22, 21, 21, 21, 21, 21, 21, 21, 21, 21, 21, 21, 20, 20, 20, 20, 20, 20, 20, 20, 20, 20, 20, 20, 20, 20, 20, 20, 20, 20, 19, 19, 19, 19, 19, 18, 18, 18, 18, 18, 18, 18, 18, 18, 18, 18, 18, 18, 18, 18, 18, 18, 18, 18, 18, 18, 18, 18, 18, 18, 18, 18, 18, 18, 18, 17, 17, 17, 17, 17, 17, 17, 16, 16, 16, 16, 16, 16, 16, 16, 16, 16, 16, 16, 16, 16, 16, 16, 16, 16, 16, 16, 16, 16, 16, 16, 15, 15, 15, 15, 15, 15, 15, 15, 15, 15, 15, 15, 15, 15, 15, 15, 15, 15, 15, 15, 14, 14, 14, 14, 14, 14, 14, 14, 14, 14, 14, 14, 14, 14, 14, 14, 14, 14, 14, 14, 14, 14, 14, 14, 14, 14, 14, 14, 13, 13, 13, 13, 13, 13, 13, 13, 13, 13, 13, 13, 13, 12, 12, 12, 12, 12, 12, 12, 12, 12, 12, 12, 12, 12, 12, 12, 12, 12, 12, 12, 12, 12, 12, 12, 12, 12, 12, 12, 12, 12, 12, 12, 12, 12, 12, 12, 12, 12, 12, 12, 12, 12, 11, 11, 11, 11, 11, 11, 11, 11, 11, 11, 11, 11, 11, 11, 11, 10, 10, 10, 10, 10, 10, 10, 10, 10, 10, 10, 10, 10, 10, 10, 10, 10, 10, 10, 10, 10, 10, 10, 10, 10, 10, 10, 10, 10, 10, 10, 10, 10, 10, 10, 10, 10, 10, 10, 10, 10, 10, 10, 10, 10, 10, 10, 10, 10, 9, 9, 9, 9, 9, 9, 9, 9, 9, 9, 9, 9, 9, 9, 9, 9, 9, 9, 9, 9, 9, 9, 9, 9, 9, 9, 9, 9, 8, 8, 8, 8, 8, 8, 8, 8, 8, 8, 8, 8, 8, 8, 8, 8, 8, 8, 8, 8, 8, 8, 8, 8, 8, 8, 8, 8, 8, 8, 8, 8, 8, 8, 8, 8, 8, 8, 8, 8, 8, 8, 8, 8, 8, 8, 8, 8, 8, 8, 8, 8, 8, 8, 8, 8, 7, 7, 7, 7, 7, 7, 7, 7, 7, 7, 7, 7, 7, 7, 7, 7, 7, 7, 7, 7, 7, 7, 7, 7, 7, 7, 7, 7, 7, 7, 7, 7, 7, 7, 7, 7, 7, 7, 7, 6, 6, 6, 6, 6, 6, 6, 6, 6, 6, 6, 6, 6, 6, 6, 6, 6, 6, 6, 6, 6, 6, 6, 6, 6, 6, 6, 6, 6, 6, 6, 6, 6, 6, 6, 6, 6, 6, 6, 6, 6, 6, 6, 6, 6, 6, 6, 6, 6, 6, 6, 6, 6, 6, 6, 6, 6, 6, 6, 6, 6, 6, 6, 6, 6, 6, 6, 6, 6, 6, 6, 6, 6, 6, 6, 6, 6, 6, 6, 6, 6, 5, 5, 5, 5, 5, 5, 5, 5, 5, 5, 5, 5, 5, 5, 5, 5, 5, 5, 5, 5, 5, 5, 5, 5, 5, 5, 5, 5, 5, 5, 5, 5, 5, 5, 5, 5, 5, 5, 5, 5, 5, 5, 5, 5, 4, 3, 2, 1

Corresponding number of selected Gene ontology terms each gene is involved with:
(In the same order as above)
131, 54, 50, 84, 42, 35, 39, 42, 50, 92, 36, 40, 28, 19, 43, 48, 22, 64, 64, 29, 45, 19, 29, 84, 25, 62, 18, 110, 31, 18, 18, 43, 15, 26, 16, 13, 102, 22, 15, 16, 27, 47, 37, 13, 12, 19, 19, 10, 34, 13, 33, 15, 33, 27, 27, 9, 20, 12, 78, 12, 11, 9, 38, 10, 9, 24, 9, 18, 16, 16, 13, 11, 11, 70, 12, 10, 26, 8, 9, 9, 31, 12, 8, 13, 9, 23, 8, 14, 37, 11, 22, 9, 12, 105, 21, 13, 103, 20, 10, 20, 9, 7, 8, 24, 12, 12, 6, 12, 94, 47, 93, 7, 15, 6, 15, 15, 7, 8, 8, 8, 8, 20, 5, 39, 13, 6, 26, 6, 38, 25, 15, 15, 6, 4, 7, 10, 7, 7, 7, 34, 17, 4, 22, 6, 11, 22, 6, 5, 5,

64, 7, 9, 31, 4, 10, 15, 20, 6, 4, 20, 60, 30, 3, 19, 7, 4, 14, 8, 7, 28, 7, 14, 5, 11, 5, 9, 18, 9, 54, 13, 13, 3, 10, 5, 7, 7, 6, 6, 3, 6, 24, 4, 6, 16, 8, 23, 23, 23, 9, 5, 5, 5, 5, 45, 5, 15, 44, 22, 4, 3, 7, 6, 3, 7, 6, 3, 41, 4, 4, 8, 10, 2, 40, 8, 4, 10, 3, 3, 3, 13, 13, 3, 3, 19, 19, 19, 38, 19, 2, 2, 9, 3, 9, 18, 3, 9, 12, 4, 6, 3, 18, 6, 18, 9, 12, 7, 17, 17, 17, 3, 33, 11, 3, 33, 3, 3, 3, 3, 3, 16, 16, 32, 2, 8, 16, 3, 6, 15, 15, 3, 5, 3, 15, 5, 15, 3, 7, 2, 2, 7, 4, 28, 28, 4, 2, 4, 27, 3, 27, 3, 3, 3, 3, 9, 3, 9, 2, 2, 13, 2, 13, 13, 13, 13, 26, 2, 5, 5, 5, 25, 2, 24, 8, 4, 2, 12, 24, 3, 8, 3, 12, 3, 8, 6, 12, 3, 8, 4, 3, 4, 6, 4, 8, 24, 24, 8, 23, 22, 11, 2, 2, 2, 22, 11, 2, 2, 11, 11, 3, 3, 3, 21, 21, 3, 3, 7, 3, 3, 21, 4, 5, 20, 1, 4, 5, 10, 20, 4, 20, 10, 20, 2, 4, 4, 4, 5, 10, 1, 19, 19, 19, 19, 9, 9, 6, 18, 6, 9, 9, 6, 2, 6, 9, 9, 3, 3, 1, 6, 6, 6, 2, 3, 9, 3, 3, 3, 3, 18, 9, 1, 6, 1, 17, 1, 17, 17, 17, 1, 1, 1, 16, 8, 4, 1, 2, 8, 4, 4, 2, 16, 2, 4, 4, 4, 1, 1, 8, 16, 16, 8, 4, 8, 1, 15, 5, 5, 1, 5, 3, 1, 5, 5, 5, 5, 3, 3, 5, 5, 3, 5, 5, 3, 2, 7, 7, 14, 7, 7, 7, 2, 2, 7, 14, 14, 1, 14, 2, 2, 7, 1, 14, 7, 1, 2, 7, 2, 14, 7, 7, 7, 1, 1, 13, 13, 13, 13, 1, 13, 13, 1, 1, 13, 1, 13, 1, 4, 3, 1, 12, 6, 1, 6, 6, 3, 3, 1, 6, 4, 3, 6, 12, 12, 3, 2, 6, 4, 3, 2, 4, 1, 2, 12, 3, 6, 4, 12, 2, 3, 12, 6, 12, 4, 2, 3, 11, 11, 11, 11, 1, 1, 1, 11, 11, 11, 1, 11, 1, 1, 11, 1, 5, 2, 2, 5, 10, 10, 5, 10, 2, 5, 10, 10, 5, 5, 1, 2, 5, 5, 10, 1, 10, 10, 10, 1, 10, 2, 5, 1, 5, 10, 10, 5, 10, 10, 5, 10, 10, 5, 2, 1, 2, 5, 10, 2, 10, 5, 5, 2, 9, 1, 3, 3, 9, 1, 3, 1, 9, 1, 9, 9, 3, 3, 1, 9, 1, 1, 1, 3, 1, 3, 9, 1, 3, 9, 3, 1, 8, 8, 1, 2, 1, 4, 2, 1, 2, 8, 2, 1, 2, 4, 8, 4, 1, 2, 1, 2, 1, 8, 2, 8, 4, 4, 2, 8, 8, 8, 4, 4, 8, 4, 4, 4, 8, 8, 4, 2, 8, 8, 4, 8, 4, 8, 8, 8, 1, 2, 2, 4, 8, 2, 4, 1, 1, 7, 7, 7, 7, 1, 1, 7, 7, 1, 7, 7, 7, 1, 1, 1, 1, 7, 7, 7, 7, 7, 7, 7, 7, 7, 7, 1, 7, 1, 7, 7, 7, 7, 7, 7, 1, 1, 1, 3, 1, 2, 2, 6, 6, 1, 6, 3, 6, 1, 2, 2, 1, 6, 1, 2, 1, 3, 3, 3, 1, 6, 3, 6, 6, 3, 1, 2, 1, 1, 1, 3, 1, 2, 1, 2, 2, 2, 1, 3, 6, 3, 6, 6, 6, 6, 3, 2, 6, 1, 2, 2, 2, 6, 2, 6, 1, 6, 3, 1, 3, 1, 3, 1, 1, 3, 6, 2, 2, 6, 6, 3, 6, 3, 1, 2, 3, 3, 6, 2, 1, 1, 1, 5, 5, 5, 5, 5, 1, 5, 1, 1, 5, 5, 1, 1, 5, 5, 5, 1, 5, 1, 1, 5, 5, 5, 5, 5, 5, 5, 5, 5, 1, 5, 1, 1, 1, 5, 1, 5, 1, 1, 1, 5, 2, 4, 1, 2, 4, 2, 2, 1, 2, 1, 4, 4, 4, 1, 4, 4, 1, 1, 2, 4, 2, 1, 1, 4, 1, 4, 4, 1, 1, 4, 4, 2, 2, 2, 1, 1, 1, 2, 4, 4, 2, 4, 4, 4, 4, 2, 1, 4, 4, 4, 2, 4, 2, 4, 1, 4, 2, 1, 4, 4, 4, 1, 2, 1, 2, 2, 4, 4, 2, 1, 1, 1, 1, 2, 1, 2, 4, 4, 4, 2, 2, 2, 4, 2, 4, 1, 4, 2, 2, 4, 4, 2, 4, 2, 4, 4, 4, 4, 4, 2, 4, 4, 4, 1, 4, 2, 2, 4, 2, 4, 1, 1, 2, 2, 3, 1, 3, 1, 1, 1, 1, 3, 3, 3, 3, 3, 1, 3, 3, 1, 3, 3, 3, 3, 1, 1, 3, 1, 3, 3, 1, 3, 3, 3, 3, 1, 3, 3, 3, 1, 1, 1, 1, 1, 1, 3, 1, 3, 1, 1, 3, 1, 1, 3, 3, 3, 1, 1, 3, 1, 3, 1, 3, 1, 1, 1, 3, 3, 1, 3, 1, 3, 1, 3, 3, 3, 3, 1, 3, 3, 1, 3, 1, 3, 3, 1, 3, 1, 3, 3, 3, 1, 1, 3, 3, 1, 3, 1, 1, 1, 3, 3, 3, 3, 3, 3, 3, 3, 1, 3, 3, 1, 1, 3, 3, 1, 1, 2, 1, 1, 2, 2, 2, 1, 2, 1, 2, 1, 1, 2, 1, 2, 2, 1, 2, 1, 1, 2, 1, 1, 2, 2, 1, 1, 1, 1, 1, 2, 1, 2, 2, 2, 1, 1, 2, 2, 1, 1, 1, 2, 1, 2, 2, 1, 2, 2, 2, 1, 1, 2, 1, 2, 2, 1, 1, 2, 2, 2, 2, 2, 1, 1, 2, 2, 1, 2, 1, 2, 2, 1, 1, 2, 2, 1, 2, 2, 2, 1, 2, 1, 1, 1, 2, 2, 1, 1, 1, 2, 1, 2, 2, 2, 1, 1, 1, 1, 2, 2, 2, 2, 2, 2, 2, 2, 1, 1, 1, 2, 2, 1, 1, 2, 2, 1, 2, 1, 1, 2, 2, 2, 2, 2, 1, 1, 1, 1, 2, 2, 2, 2, 1, 1, 1, 1, 1, 1, 2, 1, 1, 2, 2, 1, 1, 2, 2, 1, 1, 2, 2, 2, 1, 2, 1, 1, 2, 1, 2, 1, 1, 1, 2, 1, 2, 2, 1, 2, 1, 2, 2, 2, 1, 2, 2, 2, 2, 1, 2, 1, 1, 1, 2, 2, 1, 1, 1, 2, 1, 2, 2, 1, 2, 2, 1, 2, 2, 1, 2, 2, 2, 2, 1, 2, 2, 2, 2, 2, 1

Corresponding number of selected TFs each gene is involved with:
(In the same order as above)
16, 15, 14, 8, 13, 15, 13, 12, 10, 5, 12, 10, 13, 19, 8, 7, 15, 5, 5, 10, 6, 14, 9, 3, 10, 4, 13, 2, 7, 12, 12, 5, 14, 8, 13, 16, 2, 9, 13, 12, 7, 4, 5, 14, 15, 9, 9, 17, 5, 13, 5, 11, 5, 6, 6, 18, 8, 13, 2, 13, 14, 17, 4, 15, 16, 6, 16, 8, 9, 9, 11, 13, 13, 2, 11, 13, 5, 16, 14, 14, 4, 10, 15, 9, 13, 5, 14, 8, 3, 10, 5, 12, 9, 1, 5, 8, 1, 5, 10, 5, 11, 14, 12, 4, 8, 8, 16, 8, 1, 2, 1, 13, 6, 15, 6, 6, 12, 10, 10, 10, 10, 4, 16, 2, 6, 13, 3, 13, 2, 3, 5, 5, 12, 18, 10, 7, 10, 10, 10, 2, 4, 17, 3, 11, 6, 3, 11, 13, 13, 1, 9, 7, 2, 15, 6, 4, 3, 10, 15, 3, 1, 2, 19, 3, 8, 14, 4, 7, 8, 2, 8, 4, 11, 5, 11, 6, 3, 6, 1, 4, 4, 17, 5, 10, 7, 7, 8, 8, 16, 8, 2, 12, 8, 3, 6, 2, 2, 2, 5, 9, 9, 9, 9, 1, 9, 3, 1, 2, 11, 14, 6, 7, 14, 6, 7, 14, 1, 10, 10, 5, 4, 20, 1, 5, 10, 4, 13, 13, 13, 3, 3, 13, 13, 2, 2, 2, 1, 2, 19, 19, 4, 12, 4, 2, 12, 4, 3, 9, 6, 12, 2, 6, 2, 4, 3, 5, 2, 2, 2, 11, 1, 3, 11, 1, 11, 11, 11, 11, 11, 2, 2, 1, 16, 4, 2, 10, 5, 2, 2, 10, 6, 10, 2, 6, 2, 10, 4, 14, 14, 4, 7, 1, 1, 7, 14, 7, 1, 9, 1, 9, 9, 9, 9, 3, 9, 3, 13, 13, 2, 13, 2, 2, 2, 2, 1, 13, 5, 5, 5, 1, 12, 1, 3, 6, 12, 2, 1, 8, 3, 8, 2, 8, 3, 4, 2, 8, 3, 6, 8, 6, 4, 6, 3, 1, 1, 3, 1, 1, 2, 11, 11, 11, 1, 2, 11, 11, 2, 2, 7, 7, 7, 1, 1, 7, 7, 3, 7, 7, 1, 5, 4, 1, 20, 5, 4, 2, 1, 5, 1, 2, 1, 10, 5, 5, 5, 4, 2, 19, 1, 1, 1, 1, 2, 2, 3, 1, 3, 2, 2, 3, 9, 3, 2, 2, 6, 6, 18, 3, 3, 3, 9, 6, 2, 6, 6, 6, 6, 1, 2, 18, 3, 17, 1, 17, 1, 1, 1, 17, 16, 16, 1, 2, 4, 16, 8, 2, 4, 4, 8, 1, 8, 4, 4, 4, 16, 16, 2, 1, 1, 2, 4, 2, 15, 1, 3, 3, 15, 3, 5, 15, 3, 3, 3, 3, 5, 5, 3, 3, 5, 3, 3, 5, 7, 2, 2, 1, 2, 2, 2, 7, 7, 2, 1, 1, 14, 1, 7, 7, 2, 14, 1, 2, 14, 7, 2, 7, 1, 2, 2, 2, 13, 13, 1, 1, 1, 1, 13, 1, 1, 13, 13, 1, 13, 1, 12, 3, 4, 12, 1, 2, 12, 2, 2, 4, 4, 12, 2, 3, 4, 2, 1, 1, 4, 6, 2, 3, 4, 6, 3, 12, 6, 1, 4, 2, 3, 1, 6, 4, 1, 2, 1, 3, 6, 4, 1, 1, 1, 1, 11, 11, 11, 1, 1, 1, 11, 1, 11, 11, 1, 10, 2, 5, 5, 2, 1, 1, 2, 1, 5, 2, 1, 1, 2, 2, 10, 5, 2, 2, 1, 10, 1, 1, 1, 10, 1, 5, 2, 10, 2, 1, 1, 2, 1, 1, 2, 1, 1, 2, 5, 10, 5, 2, 1, 5, 1, 2, 2, 5, 1, 9, 3, 3, 1, 9, 3, 9, 1, 9, 1, 1, 3, 3, 9, 1, 9, 9, 9, 3, 9, 3, 1, 9, 3, 1, 3, 9, 1, 1, 8, 4, 8, 2, 4, 8, 4, 1, 4, 8, 4, 2, 1, 2, 8, 4, 8, 4, 8, 1, 4,

1, 2, 2, 4, 1, 1, 1, 2, 2, 1, 2, 2, 2, 1, 1, 2, 4, 1, 1, 2, 1, 2, 1, 1, 1, 8, 4, 4, 2, 1, 4, 2, 8, 7, 1, 1, 1, 1, 7, 7, 1, 1, 7, 1, 1, 1, 7, 7, 7, 7, 1, 1, 1, 1, 1, 1, 1, 1, 1, 1, 7, 1, 7, 1, 1, 1, 1, 1, 1, 7, 7, 7, 2, 6, 3, 3, 1, 1, 6, 1, 2, 1, 6, 3, 3, 6, 1, 6, 3, 6, 2, 2, 2, 6, 1, 2, 1, 1, 2, 6, 3, 6, 6, 6, 2, 6, 3, 6, 3, 3, 3, 6, 2, 1, 2, 1, 1, 1, 1, 2, 3, 1, 6, 3, 3, 3, 1, 3, 1, 6, 1, 2, 6, 2, 6, 2, 6, 6, 2, 1, 3, 3, 1, 1, 2, 1, 2, 6, 3, 2, 2, 1, 3, 5, 5, 5, 1, 1, 1, 1, 1, 5, 1, 5, 5, 1, 1, 5, 5, 1, 1, 1, 5, 1, 5, 5, 1, 1, 1, 1, 1, 1, 1, 1, 5, 1, 5, 5, 5, 1, 5, 1, 5, 5, 5, 1, 2, 1, 4, 2, 1, 2, 2, 4, 2, 4, 1, 1, 1, 4, 1, 1, 4, 4, 2, 1, 2, 4, 4, 1, 4, 1, 1, 4, 4, 1, 1, 2, 2, 2, 4, 4, 4, 2, 1, 1, 2, 1, 1, 1, 1, 2, 4, 1, 1, 1, 2, 1, 2, 1, 4, 1, 2, 4, 1, 1, 1, 4, 2, 4, 2, 2, 1, 1, 2, 4, 4, 4, 4, 2, 4, 2, 1, 1, 1, 2, 2, 2, 1, 2, 1, 4, 1, 2, 2, 1, 1, 2, 1, 2, 1, 1, 1, 1, 1, 2, 1, 1, 1, 4, 1, 2, 2, 1, 2, 1, 4, 4, 2, 2, 1, 3, 1, 3, 3, 3, 3, 1, 1, 1, 1, 1, 3, 1, 1, 3, 1, 1, 1, 1, 3, 3, 1, 3, 1, 1, 3, 1, 1, 1, 1, 3, 1, 1, 1, 3, 3, 3, 3, 3, 3, 1, 3, 1, 3, 3, 1, 3, 3, 1, 1, 1, 3, 3, 1, 3, 1, 3, 1, 3, 3, 3, 1, 1, 3, 1, 3, 1, 3, 1, 1, 1, 1, 3, 1, 1, 3, 1, 3, 1, 1, 3, 1, 3, 1, 1, 1, 3, 3, 1, 1, 3, 1, 3, 3, 3, 1, 1, 1, 1, 1, 1, 1, 1, 3, 1, 1, 3, 3, 1, 1, 3, 3, 1, 2, 2, 1, 1, 1, 2, 1, 2, 1, 2, 2, 1, 2, 1, 1, 2, 1, 2, 2, 1, 2, 2, 1, 1, 2, 2, 2, 2, 2, 1, 2, 1, 1, 1, 2, 2, 1, 1, 2, 2, 2, 1, 2, 1, 1, 2, 1, 1, 1, 2, 2, 1, 2, 1, 1, 2, 2, 1, 1, 1, 1, 1, 2, 2, 1, 1, 2, 1, 2, 1, 1, 2, 2, 1, 1, 2, 1, 1, 1, 2, 1, 2, 2, 2, 1, 1, 2, 2, 2, 1, 2, 1, 1, 1, 2, 2, 2, 2, 1, 1, 1, 1, 1, 1, 2, 2, 2, 1, 1, 2, 2, 1, 1, 2, 1, 2, 2, 1, 1, 1, 1, 1, 2, 2, 2, 2, 1, 1, 1, 1, 2, 2, 2, 2, 2, 2, 1, 2, 2, 1, 1, 2, 2, 1, 1, 2, 2, 1, 1, 1, 2, 1, 2, 2, 1, 2, 1, 2, 2, 2, 1, 2, 1, 1, 2, 1, 2, 1, 1, 1, 2, 1, 1, 1, 1, 2, 1, 2, 2, 2, 1, 1, 2, 2, 2, 1, 2, 1, 1, 2, 1, 1, 2, 1, 1, 2, 1, 1, 1, 1, 2, 1, 1, 1, 1, 2, 1

Gene ontology terms ranked according to mean of effect sizes in cluster:
immature T cell proliferation in the thymus | cytokine biosynthetic process | regulation of tumor necrosis factor production | cytoskeleton | tumor necrosis factor production | macrophage activation | granuloma formation | immunoglobulin secretion | interleukin-1 receptor antagonist activity | response to tumor necrosis factor | endothelial cell activation | interleukin-1 production | interleukin-1 beta production | interleukin-12 production | specific granule | actin cytoskeleton | tumor necrosis factor receptor activity | bone resorption | T cell proliferation | tumor necrosis factor receptor binding | antibody-dependent cellular cytotoxicity | U-plasminogen activator receptor activity | cell surface | leukotriene biosynthetic process | actin filament polymerization | B cell activation | lymphocyte activation | cell motility | T-helper 1 type immune response | cell adhesion | filamentous actin | interleukin-6 receptor activity | granulocyte macrophage colony-stimulating factor biosynthetic process | immune response | granulocyte macrophage colony-stimulating factor production | inflammatory response | mucosal immune response | monocyte differentiation | negative regulation of tumor necrosis factor production | innate immune response | leukocyte migration | leukocyte activation | chemotaxis | response to lipopolysaccharide | prostanoid biosynthetic process | protein secretion by the type III secretion system | lipoxygenase pathway | proteinaceous extracellular matrix | middle lamella-containing extracellular matrix | extracellular matrix | osteoclast differentiation | interleukin-6 production | interleukin-8 production | cytokine production | monocyte activation | cytokine activity | acute inflammatory response | interleukin-10 production | phagocytosis | interferon-gamma production | mRNA transcription | lipopolysaccharide binding | fibrinogen binding | cell migration | type IV hypersensitivity | interleukin-1 receptor activity | piecemeal microautophagy of nucleus | establishment of cell polarity | interleukin-4 production | complement activation | eosinophil activation | response to host immune response | hypersensitivity | cell fraction | chemokine production | B cell proliferation | cytokine secretion | immunoglobulin production | actin filament binding | interleukin-4 receptor activity | positive regulation of NF-kappaB transcription factor activity | negative regulation of inflammatory response | positive regulation of tumor necrosis factor production | adaptive immune response | neutrophil degranulation | neutrophil apoptosis | localization of cell | antigen processing and presentation | coagulation | fibrinogen complex | respiratory burst | interleukin-2 receptor activity | arachidonic acid metabolic process | mast cell degranulation | MHC class II protein binding | leukocyte adhesion | sensitization | cell chemotaxis | MHC class II biosynthetic process | interleukin-5 production | natural killer cell receptor activity | neutrophil activation | hemostasis | receptor complex | actin binding | adaptive immune response based on somatic recombination of immune receptors built from immunoglobulin superfamily domains | lymphocyte proliferation | actin filament depolymerization | leading edge | cyclooxygenase pathway | basement membrane | collagen catabolic process | interleukin-2 production | positive chemotaxis | fibrinolysis | natural killer cell mediated

cytotoxicity | naringenin-chalcone synthase activity | hyaluronic acid binding | membrane attack complex | opsonization | nerve growth factor receptor activity | nerve growth factor receptor binding | cytolysis | IkappaB kinase complex | NF-kappaB binding | neutrophil chemotaxis | mast cell activation | membrane raft | prostaglandin biosynthetic process | pathogenesis | cell maturation | evasion or tolerance of immune response of other organism during symbiotic interaction | tolerance induction | actin filament | luteolysis | integrin binding | crotonyl-CoA reductase activity | diapedesis | chronic inflammatory response | actomyosin | MHC class I biosynthetic process | MHC class I protein binding | tryptophan catabolic process | platelet activation | monocyte chemotaxis | icosanoid metabolic process | defense response to virus | leukocyte chemotaxis

Corresponding mean of effect sizes of each Gene ontology term:
(In the same order as above)
2.19, 1.82, 1.61, 1.61, 1.58, 1.53, 1.52, 1.52, 1.46, 1.43, 1.41, 1.40, 1.36, 1.36, 1.33, 1.32, 1.31, 1.28, 1.26, 1.25, 1.25, 1.25, 1.23, 1.22, 1.21, 1.18, 1.17, 1.17, 1.16, 1.15, 1.13, 1.12, 1.12, 1.11, 1.11, 1.10, 1.10, 1.10, 1.07, 1.07, 1.07, 1.05, 1.05, 1.04, 1.04, 1.03, 1.02, 1.00, 1.00, 1.00, 0.98, 0.98, 0.97, 0.97, 0.96, 0.94, 0.94, 0.94, 0.93, 0.92, 0.92, 0.92, 0.91, 0.89, 0.89, 0.88, 0.87, 0.87, 0.87, 0.86, 0.86, 0.85, 0.84, 0.84, 0.84, 0.84, 0.83, 0.81, 0.81, 0.80, 0.79, 0.79, 0.79, 0.78, 0.77, 0.76, 0.76, 0.76, 0.75, 0.74, 0.74, 0.73, 0.72, 0.69, 0.69, 0.66, 0.66, 0.65, 0.65, 0.65, 0.63, 0.63, 0.62, 0.62, 0.61, 0.61, 0.61, 0.60, 0.60, 0.57, 0.55, 0.55, 0.54, 0.52, 0.51, 0.51, 0.51, 0.50, 0.47, 0.45, 0.44, 0.44, 0.43, 0.43, 0.41, 0.40, 0.40, 0.39, 0.39, 0.36, 0.36, 0.34, 0.33, 0.32, 0.32, 0.28, 0.28, 0.27, 0.25, 0.25, 0.24, 0.24, 0.15, 0.14, 0.09, 0.00, -0.03, -0.48

Corresponding total number of genes of each Gene ontology term:
(In the same order as above)
42, 108, 86, 612, 209, 178, 77, 51, 70, 185, 106, 31, 21, 150, 62, 605, 213, 192, 188, 214, 109, 130, 1.5K, 50, 300, 116, 232, 478, 59, 937, 439, 104, 109, 530, 107, 425, 52, 102, 61, 246, 145, 108, 371, 226, 51, 69, 43, 805, 805, 805, 144, 165, 144, 455, 117, 42, 68, 166, 304, 106, 130, 61, 84, 754, 131, 128, 200, 152, 123, 148, 71, 37, 234, 112, 155, 88, 243, 155, 62, 87, 550, 40, 61, 148, 52, 117, 38, 315, 184, 235, 195, 189, 88, 90, 178, 158, 254, 94, 181, 57, 77, 147, 110, 423, 142, 131, 158, 56, 195, 42, 289, 71, 182, 87, 96, 33, 50, 37, 134, 67, 100, 112, 179, 107, 146, 86, 91, 191, 105, 1.1K, 224, 141, 109, 177, 69, 81, 52, 47, 22, 118, 195, 197, 41, 220, 52, 24, 110, 41

Corresponding number of genes of each Gene ontology term with at least one TFBS (of selected TFs):
(In the same order as above)
23, 47, 37, 242, 84, 71, 26, 26, 24, 73, 44, 14, 10, 64, 20, 253, 92, 59, 72, 92, 35, 55, 496, 17, 120, 49, 102, 208, 25, 318, 172, 42, 41, 172, 40, 141, 19, 37, 25, 75, 54, 31, 108, 92, 20, 34, 14, 256, 256, 256, 60, 67, 52, 160, 35, 19, 19, 67, 92, 42, 63, 20, 36, 289, 48, 46, 60, 60, 56, 41, 21, 18, 76, 37, 55, 41, 91, 54, 29, 38, 231, 15, 24, 52, 14, 47, 21, 115, 53, 70, 58, 71, 23, 29, 61, 57, 93, 33, 61, 26, 21, 40, 30, 150, 47, 42, 57, 27, 79, 13, 89, 19, 83, 29, 29, 10, 18, 12, 36, 16, 38, 46, 60, 47, 61, 25, 36, 66, 31, 371, 88, 46, 41, 69, 20, 34, 19, 11, 8, 46, 73, 73, 16, 72, 15, 7, 43, 9

Corresponding total number of TFBS (of selected TFs) for each Gene ontology term (in all genes):
(In the same order as above)
137, 247, 197, 1056, 380, 337, 131, 117, 149, 391, 185, 59, 51, 281, 108, 1079, 421, 308, 337, 421, 148, 208, 1840, 105, 584, 216, 472, 863, 105, 1302, 757, 181, 183, 760, 181, 599, 88, 165, 127, 341, 225, 148, 456, 420, 88, 153, 76, 1029, 1029, 1029, 318, 292, 232, 711, 156, 92, 96, 265, 411, 150, 279, 83, 159, 1108, 231, 199, 239, 283, 211, 146, 96, 64, 301, 179, 211, 167, 383, 202, 152, 175, 971, 78, 115, 183, 69, 214, 90, 507, 197, 284, 290, 331, 110, 106, 278, 207, 356, 148, 278, 109, 101, 178, 124, 603, 253, 140, 236, 115, 353, 65, 305, 80, 359, 116, 116, 54, 68, 56, 130, 56, 157, 178, 219, 199, 253, 113, 145, 308, 137, 1409, 353, 193, 160, 277, 72, 118, 77, 64, 36, 179, 313, 313, 74, 269, 56, 25, 166, 27

Corresponding number of selected TFs each Gene ontology term is involved with:
(In the same order as above)
20, 20, 20, 20, 20, 20, 20, 20, 20, 20, 20, 19, 19, 20, 20, 20, 20, 20, 20, 20, 20, 20, 20, 20, 20, 20, 20, 20, 20, 20, 20, 20, 20, 20, 20, 20, 20, 20, 20, 20, 20, 20, 20, 20, 20, 20, 20, 20, 20, 20, 20, 20, 20, 20, 20, 20, 20, 20, 20, 20, 20, 20, 20, 20, 20, 20, 20, 20, 20, 20, 20, 20, 20, 20, 20, 20, 20, 20, 20, 20, 20, 20,

19, 20, 20, 20, 20, 20, 20, 20, 20, 20, 20, 20, 20, 20, 20, 20, 20, 20, 20, 20, 20, 20, 20, 20, 20, 20, 20, 20, 20, 20, 20, 20, 20, 20, 20, 20, 20, 20, 20, 20, 20, 20, 20, 19, 20, 20, 20, 20, 20, 20, 20, 20, 20, 20, 20, 19, 20, 20, 20, 20, 20, 20, 20, 20, 16

TFs ranked according to mean of effect sizes in cluster:
V$PEBP_Q6, V$AP1_C, V$AP1_Q6_01, V$AP1_Q6, V$NFE2_01, V$AP1_Q4_01, V$AP1_Q2_01, V$AP1_01, VMAF_Q6_01, VAML_Q6, V$NRF2_Q4, V$AP1_Q2, V$AP1_Q4, V$DR3_Q4, V$AP1FJ_Q2, V$FOXP3_Q4, VPAX_Q6, VBLIMP1_Q6, V$CACCCBINDINGFACTOR_Q6, V$BACH2_01

Corresponding mean of effect sizes of each TF:
(In the same order as above)
1.35, 1.22, 1.18, 1.07, 1.07, 1.04, 1.03, 1.01, 0.95, 0.89, 0.74, 0.73, 0.73, 0.69, 0.61, 0.60, 0.52, 0.48, 0.35, 0.33

Corresponding total number of TFBS for each TF (genome-wide):
(In the same order as above)
692, 720, 726, 721, 729, 719, 714, 721, 700, 680, 683, 692, 712, 670, 710, 689, 683, 679, 684, 686

Corresponding total number of TFBS for each TF (in all genes in selected Gene ontology terms):
(In the same order as above)
2283, 2426, 2543, 2401, 2320, 2395, 2186, 2283, 2180, 1936, 2012, 2189, 2294, 1771, 2107, 1814, 1933, 1617, 1646, 1844

Corresponding number of genes (of selected Gene ontology terms) each TF is involved with:
(In the same order as above)
276, 311, 325, 307, 278, 319, 300, 299, 297, 271, 290, 283, 293, 229, 286, 256, 278, 264, 260, 272

Corresponding number of selected Gene ontology terms each TF is involved with:
(In the same order as above)
148, 147, 148, 148, 148, 148, 147, 148, 148, 148, 148, 148, 148, 147, 148, 148, 148, 148, 146, 144

Cluster for columns 172 to 185, rows 199 to 203

Gene ontology terms

isochorismate pyruvate lyase activity | axon | dendrite | synapse | synaptogenesis | exocytosis | synaptic transmission | postsynaptic membrane | receptor clustering | postsynaptic density | N-methyl-D-aspartate selective glutamate receptor activity | dendritic spine | receptor activity | synaptic transmission, glutamatergic

TFs

V$TFIII_Q6 | V$MZF1_01 | V$VDR_Q3 | V$MZF1_02 | V$SMAD_Q6_01

Information

All related TFs:
(List of all TFs that are related to any of the PWMs)
MZF1B-C, Smad1, Smad1.1, Smad10, Smad2, Smad2-L, Smad3, Smad3:Smad4, Smad4, Smad4alpha, Smad4beta, Smad4delta3, Smad4delta4-6, Smad4delta4-7, Smad4delta5-6, Smad4delta6, Smad5, Smad6, Smad7, Smad8, TFII-I, VDR

Ranked gene list:
(All genes of the selected Gene ontology terms with hits of any of the selected TFs, ranked according to the total number of TFBS)
DLG4, PPP1R9B, CDK5, MGAT1, AKT1, PSD, GRIN2D, ACHE, FBXL15, SYNGAP1, AGRN, CAMK2G, ARHGEF2, SLC17A7, SHANK3, VAMP2, PNMT, PER1, GRM2, TNFSF13, RHBDL1, NDOR1, GRIN2C, PPP1R1B, GIT1, EFNA3, ITGB2, VGF, NRGN, NLGN2, GUK1, GABARAP, EGR1, EFNA2, BRD2, TPM3, SV2A, SLC2A4RG, SHANK1, FASN, STXBP2, SLC25A22, SEMA3B, RARA, OPRL1, NELF, JUNB, ISYNA1, COMP, CACNA1G, p63RhoGEF, TNFRSF1A, TFAP2A, SYT7, SLC22A18, SEPT5, RTN4RL2, REPIN1, MUC1, LBX1, KCNC3, GRIK5, GABBR1, ENO2, DVL1, DKFZp434P0672, CTSD, CNTNAP1, CAMKK1, BAIAP3, B3GAT1, ACCN2, TAS1R3, SYT8, SLC9A3R1, SLC12A5, RTN4RL1, RNASEK, PLXNA1, PGP, PARD6A, NGFR, MAPRE3, MAP1A, LASP1, KCNC4, IL23A, ACD, ACCN4, ZIC2, ZAP70, UNC13D, SYTL1, SQSTM1, SEMA3F, SCAMP2, RAB25, PTPRU, PTPRF, PLCG1, PAX6, NR3C2, MEA1, MC1R, LPHN1, KIAA1684, JUN, HS6ST1, FLJ32987, FHL3, ERBB3, ENTPD2, DNM2, DAGLA, CTBP2, CPLX1, CHRNE, CDKN1C, CD81, CD63, CD44, ADM2, ADM, ACTB, VIM, UBE4B, SYNJ2, STX6, SRRM2, SLC9A3R2, SLC12A2, SEMA7A, SCRIB, RAB37, PTGIR, PSMP, PNPLA6, PELP1, OPRS1, NRTN, NCOR2, MPZ, MAP3K11, MAP2K3, LCK, KLF7, KIF5A, KCNJ11, IGF1R, ICAM5, HRH3, GRN, GRIN3B, GRASP, GAD1, FOSB, EPHB3, EPB41, DPYSL3, CTNNBIP1, CTDSPL, CSAD, CORO1A, CHPT1, CAPN10, ATP6V0D1, ARL6IP5, ARID4B, ARF1, APEH, AGRP, ABR

Corresponding total number of TFBS:
(For each gene listed above, the total number of TFBS for any of the selected TFs, multiplied by the number of selected Gene ontology terms containing that gene)
52, 40, 40, 35, 35, 32, 30, 28, 27, 24, 24, 20, 20, 18, 18, 15, 15, 15, 13, 12, 12, 12, 12, 10, 10, 10, 9, 8, 8, 8, 8, 8, 8, 8, 8, 7, 6, 6, 6, 6, 5, 5, 5, 5, 5, 5, 5, 5, 5, 5, 4, 4, 4, 4, 4, 4, 4, 4, 4, 4, 4, 4, 4, 4, 4, 4, 4, 4, 4, 4, 4, 3, 3, 3, 3, 3, 3, 3, 3, 3, 3, 3, 3, 3, 3, 3, 3, 3, 2, 2, 2, 2, 2, 2, 2, 2, 2, 2, 2, 2, 2, 2, 2, 2, 2, 2, 2, 2, 2, 2, 2, 2, 2, 2, 2, 2, 2, 2, 2, 2, 2, 2, 1, 1, 1, 1, 1, 1, 1, 1, 1, 1, 1, 1, 1, 1, 1, 1, 1, 1, 1, 1, 1, 1, 1, 1, 1, 1, 1, 1, 1, 1, 1, 1, 1, 1, 1, 1, 1, 1, 1, 1, 1, 1, 1, 1, 1, 1, 1, 1

Corresponding number of selected Gene ontology terms each gene is involved with:
(In the same order as above)
13, 8, 8, 7, 7, 8, 6, 7, 9, 6, 6, 5, 4, 9, 6, 5, 3, 3, 13, 6, 3, 12, 6, 2, 2, 2, 3, 2, 4, 2, 8, 2, 2, 2, 2, 7, 6, 2, 2, 2, 1, 1, 1, 1, 1, 1, 1, 1, 5, 1, 1, 2, 2, 1, 1, 1, 1, 2, 1, 1, 1, 4, 1, 2, 1, 1, 1, 1, 1, 1, 2, 1, 1, 1, 1, 3, 1, 1, 1, 1, 1, 3, 3, 3, 1, 1, 3, 1, 1, 1, 1, 1, 1, 1, 2, 1, 1, 2, 2, 1, 1, 1, 1, 1, 2, 1, 2, 1, 1, 1, 2, 2, 2, 2, 2, 2, 2, 1, 2, 1, 2, 1, 1, 1, 1, 1, 1, 1, 1, 1, 1, 1, 1, 1, 1, 1, 1, 1, 1, 1, 1, 1, 1, 1, 1, 1, 1, 1, 1, 1, 1, 1, 1, 1, 1, 1, 1, 1, 1, 1, 1, 1, 1, 1, 1, 1, 1, 1, 1, 1, 1, 1

Corresponding number of selected TFs each gene is involved with:
(In the same order as above)
4, 5, 5, 5, 5, 4, 5, 4, 3, 4, 4, 4, 5, 2, 3, 3, 5, 5, 1, 2, 4, 1, 2, 5, 5, 5, 3, 4, 2, 4, 1, 4, 4, 4, 4, 1, 1, 3, 3, 3, 5, 5, 5, 5, 5, 5, 5, 5, 1, 5, 4, 2, 2, 4, 4, 4, 4, 2, 4, 4, 4, 1, 4, 2, 4, 4, 4, 4, 4, 4, 2, 4, 3, 3, 3, 1, 3, 3, 3, 3, 3, 1, 1, 1, 3, 3, 1, 3, 3, 2, 2, 2, 2, 2, 1, 2, 2, 1, 1, 2, 2, 2, 2, 2, 1, 2, 1, 2, 2, 2, 1, 1, 1, 1, 1, 1, 1, 2, 1, 2, 1, 2, 2, 2, 1, 1, 1, 1, 1, 1, 1, 1, 1, 1, 1, 1, 1, 1, 1, 1, 1, 1, 1, 1, 1, 1, 1, 1, 1, 1, 1, 1, 1, 1, 1, 1, 1, 1, 1, 1, 1, 1, 1, 1, 1, 1, 1, 1, 1, 1, 1, 1

Gene ontology terms ranked according to mean of effect sizes in cluster:
synaptic transmission | synaptogenesis | N-methyl-D-aspartate selective glutamate receptor activity | postsynaptic membrane | axon | isochorismate pyruvate lyase activity | postsynaptic density | dendritic spine | dendrite | synapse | synaptic transmission, glutamatergic | exocytosis | receptor clustering | receptor activity

Corresponding mean of effect sizes of each Gene ontology term:
(In the same order as above)
2.52, 1.93, 1.51, 1.42, 1.41, 1.40, 1.37, 1.25, 1.23, 1.21, 1.14, 0.74, 0.71, 0.60

Corresponding total number of genes of each Gene ontology term:

(In the same order as above)
251, 200, 208, 62, 382, 69, 162, 91, 82, 318, 38, 381, 79, 187

Corresponding number of genes of each Gene ontology term with at least one TFBS (of selected TFs):
(In the same order as above)
39, 35, 33, 13, 52, 13, 27, 18, 14, 41, 6, 47, 14, 28

Corresponding total number of TFBS (of selected TFs) for each Gene ontology term (in all genes):
(In the same order as above)
113, 82, 97, 35, 134, 28, 67, 54, 37, 105, 18, 123, 33, 64

Corresponding number of selected TFs each Gene ontology term is involved with:
(In the same order as above)
5, 5, 5, 5, 5, 5, 5, 5, 5, 5, 5, 5, 5, 5

TFs ranked according to mean of effect sizes in cluster:
V$MZF1_01, V$VDR_Q3, V$SMAD_Q6_01, V$TFIII_Q6, V$MZF1_02

Corresponding mean of effect sizes of each TF:
(In the same order as above)
1.58, 1.46, 1.32, 1.13, 1.10

Corresponding total number of TFBS for each TF (genome-wide):
(In the same order as above)
729, 720, 699, 726, 747

Corresponding total number of TFBS for each TF (in all genes in selected Gene ontology terms):
(In the same order as above)
211, 192, 191, 196, 200

Corresponding number of genes (of selected Gene ontology terms) each TF is involved with:
(In the same order as above)
84, 80, 82, 82, 79

Corresponding number of selected Gene ontology terms each TF is involved with:
(In the same order as above)
14, 14, 14, 14, 14

Cluster for columns 1225 to 1251, rows 384 to 387

Gene ontology terms

blood coagulation | high molecular weight kininogen binding | generation of a signal involved in cell-cell signaling | phospholipid binding | ribonuclease activity | carbohydrate binding | pore complex biogenesis | gamma-glutamyltransferase activity | amino acid catabolic process | amino acid metabolic process | arginine biosynthetic process | urea cycle | fibronectin binding | insemination | parturition | progesterone secretion | receptor binding | receptor biosynthetic process | cortisol biosynthetic process | drinking behavior | cation transport | amiloride-sensitive sodium channel activity | sodium ion transport | water homeostasis | water transport | quorum sensing | transcription, RNA-dependent

TFs

V$HNF1_Q6_01 | V$HNF1_Q6 | V$HNF1_01 | V$HNF1_C

Information

All related TFs:
(List of all TFs that are related to any of the PWMs)
HNF-1alpha, HNF-1alpha-A, HNF-1alpha-B, HNF-1alpha-C, HNF-1beta, HNF-1beta-A, HNF-1beta-B, HNF-1beta-C

Ranked gene list:
(All genes of the selected Gene ontology terms with hits of any of the selected TFs, ranked according to the total number of TFBS)
RERE, ALB, ABL2, NR3C1, MBNL1, IGFBP1, ATM, SLC35A1, FN1, TRA@, AGT, SLC5A1, PRLR, HSPD1, FGA, DPYD, BCL2, AQP5, AQP2, RUNX2, PTGER4, LRPPRC, IFNG, GLB1, CLCN5, CAP1, AQP7, SLC12A1, PROC, PLB1, PLA2G4A, ESR1, UGT1A6, TGFBR2, TBXAS1, SS18, RUNX1, RIPK4, PIK3R1, PGR, NR3C2, MRPL39, LAD1, HSPE1, GRM3, G6PC, FGFR4, ETS1, CYP2E1, CXCR4, CSH1, CP, C1orf116, APOH, ANXA11, ANTXR2, WWP1, WNK1, VIM, UGT1A1, TRIM5, SRC, SERPINA4, PTPRC, PPIG, PLXNA2, PDE4DIP, OTC, KCNJ1, IGFBP3, GNRHR, FBXW11, EGR1, DR1, CSH2, COMMD3, BTD, ANPEP, AKR1C3, AFM, TRDN, TGFBR3, TGFA, TBK1, SGK1, SERPINA10, SERPINA1, SELL, SCAMP2, PVRL1, NRBP1, NR1I2, MSH3, MGP, LBR, GUCA2B, GRK5, GFM1, FLJ32987, FCGRT, F5, F2RL1, DHRS2, CTNNB1, CSF1, CS, CNTF, CD44, BAX, ATP5E, ATP2B1, ASL, ARID4B, ANXA1, ANGPT1, A2M, UHMK1, TRAF3, TNFSF18, TLR3, TLR10, THBS1, TG, SSFA2, SNRPB, SMAD3, SLCO3A1, SLCO1B1, SLC25A13, SLC25A12, SLC22A5, SLC22A2, SLC20A1, SLC1A4, SERPINH1, SERPINC1, SDHA, RPL7, RARS, PTTG1, PTK2B, POU2F1, PLA2G7, PLA2G6, PAG1, ODZ1, NR2F2, NQO1, NARS, MYC, MCF2, MAN2A1, MAN1A1, LNPEP, LCT, JAK2, JAK1, ITGB1, ITGA5, IGFBP4, HOXB2, HIF1A, HGFAC, GZMB, GSR, GCDH, FGL2, FAM126A, FABP12, ETV1, ERN1, ERBB2, DUT, DNAJB9, DMD, DDX5, CYSLTR1, CPNE1, CPB2, CDA, CCND3, CCL8, CASP3, BMP4, B2M, ARHGAP24, ANXA2, ALCAM, AHR, AFP, ADH5, ACY1, ACVR2A

Corresponding total number of TFBS:
(For each gene listed above, the total number of TFBS for any of the selected TFs, multiplied by the number of selected Gene ontology terms containing that gene)
48, 27, 26, 21, 12, 12, 12, 10, 10, 9, 9, 8, 8, 8, 8, 8, 8, 8, 8, 6, 6, 6, 6, 6, 6, 6, 6, 5, 5, 5, 5, 5, 4, 4, 4, 4, 4, 4, 4, 4, 4, 4, 4, 4, 4, 4, 4, 4, 4, 4, 4, 4, 4, 4, 4, 4, 3, 3, 3, 3, 3, 3, 3, 3, 3, 3, 3, 3, 3, 3, 3, 3, 3, 3, 3, 3, 3, 3, 3, 3, 2, 2, 2, 2, 2, 2, 2, 2, 2, 2, 2, 2, 2, 2, 2, 2, 2, 2, 2, 2, 2, 2, 2, 2, 2, 2, 2, 2, 2, 2, 2, 2, 2, 2, 2, 2, 1, 1, 1, 1, 1, 1, 1, 1, 1, 1, 1, 1, 1, 1, 1, 1, 1, 1, 1, 1, 1, 1, 1, 1, 1, 1, 1, 1, 1, 1, 1, 1, 1, 1, 1, 1, 1, 1, 1, 1, 1, 1, 1, 1, 1, 1, 1, 1, 1, 1, 1, 1, 1, 1, 1, 1, 1, 1, 1, 1, 1, 1, 1, 1, 1, 1, 1, 1, 1, 1, 1

Corresponding number of selected Gene ontology terms each gene is involved with:
(In the same order as above)
12, 9, 13, 7, 3, 3, 6, 10, 5, 3, 9, 2, 4, 2, 2, 4, 2, 4, 4, 3, 3, 2, 2, 2, 2, 2, 2, 5, 5, 5, 5, 5, 1, 1, 1, 2, 1, 1, 1, 4, 4, 1, 1, 1, 2, 2, 1, 1, 2, 2, 4, 2, 1, 2, 1, 2, 1, 3, 1, 1, 1, 3, 1, 1, 1, 1, 1, 3, 3, 3, 3, 1, 1, 1, 3, 1, 1, 3, 1, 1, 1, 2, 2, 1, 2, 1, 1, 1, 1, 1, 1, 2, 2, 2, 1, 2, 1, 2, 2, 2, 2, 2, 1, 1, 2, 1, 1, 1, 2, 1, 1, 2, 2, 1, 1, 2, 1, 1, 1, 1, 1, 1, 1, 1, 1, 1, 1, 1, 1, 1, 1, 1, 1, 1, 1, 1, 1, 1, 1, 1, 1, 1, 1, 1, 1, 1, 1, 1, 1, 1, 1, 1, 1, 1, 1, 1, 1, 1, 1, 1, 1, 1, 1, 1, 1, 1, 1, 1, 1, 1, 1, 1, 1, 1, 1, 1, 1, 1, 1, 1, 1, 1, 1, 1, 1, 1, 1

Corresponding number of selected TFs each gene is involved with:
(In the same order as above)
4, 3, 2, 3, 4, 4, 2, 1, 2, 3, 1, 4, 2, 4, 4, 2, 4, 2, 2, 2, 2, 3, 3, 3, 3, 3, 3, 1, 1, 1, 1, 1, 4, 4, 4, 2, 4, 4, 4, 1, 1, 4, 4, 4, 2, 2, 4, 4, 2, 2, 1, 2, 4, 2, 4, 2, 3, 1, 3, 3, 3, 1, 3, 3, 3, 3, 3, 1, 1, 1, 1, 3, 3, 3, 1, 3, 3, 1, 3, 3, 2, 1, 1, 2, 1, 2, 2, 2, 2, 2, 2, 1, 1, 1, 2, 1, 2, 1, 1, 1, 1, 1, 2, 2, 1, 2, 2, 2, 1, 2, 2, 1, 1, 2, 2, 1, 1, 1, 1, 1, 1, 1, 1, 1, 1, 1, 1, 1, 1, 1, 1, 1, 1, 1, 1, 1, 1, 1, 1, 1, 1, 1, 1, 1, 1, 1, 1, 1, 1, 1, 1, 1, 1, 1, 1, 1, 1, 1, 1, 1, 1, 1, 1, 1, 1, 1, 1, 1, 1, 1, 1, 1, 1, 1, 1, 1, 1, 1, 1, 1, 1, 1, 1, 1, 1, 1, 1, 1, 1, 1, 1, 1

Gene ontology terms ranked according to mean of effect sizes in cluster:
high molecular weight kininogen binding | blood coagulation | water transport | pore complex biogenesis | parturition | gamma-glutamyltransferase activity | amino acid metabolic process | drinking behavior |

amino acid catabolic process | progesterone secretion | sodium ion transport | insemination | urea cycle | generation of a signal involved in cell-cell signaling | quorum sensing | transcription, RNA-dependent | amiloride-sensitive sodium channel activity | carbohydrate binding | ribonuclease activity | water homeostasis | phospholipid binding | arginine biosynthetic process | receptor binding | cation transport | cortisol biosynthetic process | receptor biosynthetic process | fibronectin binding

Corresponding mean of effect sizes of each Gene ontology term:
(In the same order as above)
4.58, 3.70, 2.82, 2.60, 2.40, 2.26, 2.26, 2.26, 2.13, 2.10, 2.09, 2.04, 1.99, 1.93, 1.89, 1.73, 1.65, 1.64, 1.46, 1.38, 1.33, 1.05, 0.74, 0.60, 0.41, 0.32, -0.01

Corresponding total number of genes of each Gene ontology term:
(In the same order as above)
48, 118, 63, 45, 102, 23, 50, 27, 30, 48, 54, 33, 48, 22, 34, 582, 70, 59, 23, 50, 90, 30, 417, 28, 25, 48, 30

Corresponding number of genes of each Gene ontology term with at least one TFBS (of selected TFs):
(In the same order as above)
11, 24, 13, 10, 18, 5, 10, 5, 6, 9, 13, 4, 9, 5, 6, 83, 16, 8, 4, 9, 19, 5, 47, 5, 2, 7, 5

Corresponding total number of TFBS (of selected TFs) for each Gene ontology term (in all genes):
(In the same order as above)
27, 40, 27, 22, 30, 8, 19, 10, 13, 15, 22, 14, 19, 10, 14, 147, 30, 18, 8, 14, 30, 9, 77, 6, 4, 11, 6

Corresponding number of selected TFs each Gene ontology term is involved with:
(In the same order as above)
4, 4, 4, 4, 4, 4, 4, 4, 4, 4, 4, 4, 4, 4, 4, 4, 4, 4, 4, 4, 4, 4, 4, 4, 3, 4, 4

TFs ranked according to mean of effect sizes in cluster:
V$HNF1_C, V$HNF1_Q6, V$HNF1_Q6_01, V$HNF1_01

Corresponding mean of effect sizes of each TF:
(In the same order as above)
2.41, 1.93, 1.68, 1.30

Corresponding total number of TFBS for each TF (genome-wide):
(In the same order as above)
651, 675, 679, 646

Corresponding total number of TFBS for each TF (in all genes in selected Gene ontology terms):
(In the same order as above)
180, 170, 164, 136

Corresponding number of genes (of selected Gene ontology terms) each TF is involved with:
(In the same order as above)
83, 88, 91, 77

Corresponding number of selected Gene ontology terms each TF is involved with:
(In the same order as above)
27, 27, 27, 26

Cluster for columns 354 to 364, rows 33 to 39

Gene ontology terms

merozoite dense granule | melanosome | organelle | oligosaccharide biosynthetic process | checkpoint clamp complex | transpiration | vacuolar membrane | vacuole | multivesicular body | autophagic vacuole | autophagy

TFs

V$USF_01 | V$MAX_01 | V$USF_Q6 | V$USF_02 | V$USF_Q6_01 | V$MYCMAX_03 | V$ARNT_01

Information

All related TFs:
(List of all TFs that are related to any of the PWMs)
Arnt, Max, Max1, USF, USF-1, USF1, USF1:USF2, USF1a, USF1b, USF2, USF2a, USF2b, c-Myc

Ranked gene list:
(All genes of the selected Gene ontology terms with hits of any of the selected TFs, ranked according to the total number of TFBS)
LAMP1, CD63, BAX, ATP6V0C, RAB5A, CTSA, CALR, APEX1, SQSTM1, MCOLN1, GABARAP, MTHFR, MAPRE2, GAA, CTSD, VPS16, VPS11, S100A6, PPA1, KIF2A, FIS1, MFSD10, ANXA6, QTRT1, GALNS, CTDSP2, SGTA, RNASEH2C, NOD2, HPS5, C18orf8, ATXN3, ATP6V1A, ATP6V0E1, ALG1, AKT1, USP14, SUMF1, RAB24, PHF20, MARCH8, IGF2R, GPSM1, CRTC2, PRKAR2B, PDIA2, NCL, MYD88, MUC1, MC1R, GAPDH, GALT, ATP6V1H, SYTL1, SLC31A2, RGS19, PLA2G1B, MYO1C, MAP1LC3A, HPS1, FADD, EEF2K, CLN3, CASP9, BNIP3L, ATG5, ARFRP1, VPS8, VPS36, UVRAG, USP3, TSC2, TCIRG1, RCC1, PROC, PISD, PEX10, MLPH, MARCKSL1, LSS, HSPA8, HSP90AA1, GGA2, FGFR3, CRTC1, CHMP4A, CHMP2A, BNIP3, BAK1, ATG4B, ATG3, ARVCF, ARF1, ACD, VAC14, UGT8, UCHL1, TOM1L2, TOM1, TICAM1, TBX1, SOS1, SLC25A1, RTN4, RABAC1, RAB4A, PEX7, PDCD6, PAWR, PARVA, OXSM, NKX2-3, NGLY1, MPG, MAPK1, LONP1, HSP90B1, HMGB1, HMBS, GGT1, GADD45B, FOXO3, FLVCR2, EXOC3, CDKN2D, ATIC, APC, ALG12, ACOX1

Corresponding total number of TFBS:
(For each gene listed above, the total number of TFBS for any of the selected TFs, multiplied by the number of selected Gene ontology terms containing that gene)
49, 35, 21, 21, 20, 18, 18, 18, 14, 14, 14, 12, 12, 12, 12, 10, 10, 10, 10, 10, 10, 8, 8, 7, 7, 7, 6, 6, 6, 6, 6, 6, 6, 6, 6, 6, 5, 5, 5, 5, 5, 5, 5, 5, 4, 4, 4, 4, 4, 4, 4, 4, 4, 3, 3, 3, 3, 3, 3, 3, 3, 3, 3, 3, 3, 3, 3, 3, 2, 2, 2, 2, 2, 2, 2, 2, 2, 2, 2, 2, 2, 2, 2, 2, 2, 2, 2, 2, 2, 2, 2, 2, 2, 2, 1, 1, 1, 1, 1, 1, 1, 1, 1, 1, 1, 1, 1, 1, 1, 1, 1, 1, 1, 1, 1, 1, 1, 1, 1, 1, 1, 1, 1, 1, 1, 1, 1

Corresponding number of selected Gene ontology terms each gene is involved with:
(In the same order as above)
7, 5, 3, 3, 5, 6, 3, 3, 2, 2, 2, 2, 2, 2, 4, 2, 2, 2, 2, 2, 2, 2, 2, 1, 1, 1, 1, 1, 1, 1, 1, 1, 2, 2, 2, 2, 1, 1, 1, 1, 1, 1, 1, 1, 1, 1, 1, 1, 2, 1, 1, 1, 1, 1, 1, 1, 1, 1, 3, 3, 1, 1, 1, 1, 1, 3, 1, 1, 2, 2, 2, 1, 2, 2, 1, 1, 1, 2, 1, 1, 1, 2, 1, 1, 2, 2, 1, 2, 2, 2, 2, 2, 2, 1, 1, 1, 1, 1, 1, 1, 1, 1, 1, 1, 1, 1, 1, 1, 1, 1, 1, 1, 1, 1, 1, 1, 1, 1, 1, 1, 1, 1, 1, 1, 1, 1, 1

Corresponding number of selected TFs each gene is involved with:
(In the same order as above)
7, 7, 7, 7, 4, 3, 6, 6, 7, 7, 7, 6, 6, 6, 3, 5, 5, 5, 5, 5, 5, 4, 4, 7, 7, 7, 6, 6, 6, 6, 6, 6, 3, 3, 3, 3, 5, 5, 5, 5, 5, 5, 5, 5, 4, 4, 4, 4, 2, 4, 4, 4, 4, 3, 3, 3, 3, 3, 1, 1, 3, 3, 3, 3, 3, 1, 3, 2, 1, 1, 1, 2, 1, 1, 2, 2, 2, 1, 2, 2, 2, 1, 2, 2, 1, 1, 2, 1, 1, 1, 1, 1, 1, 2, 1, 1, 1, 1, 1, 1, 1, 1, 1, 1, 1, 1, 1, 1, 1, 1, 1, 1, 1, 1, 1, 1, 1, 1, 1, 1, 1, 1, 1, 1, 1, 1, 1

Gene ontology terms ranked according to mean of effect sizes in cluster:
autophagy | vacuole | vacuolar membrane | melanosome | organelle | merozoite dense granule | multivesicular body | autophagic vacuole | transpiration | checkpoint clamp complex | oligosaccharide biosynthetic process

Corresponding mean of effect sizes of each Gene ontology term:
(In the same order as above)
3.14, 2.42, 2.18, 2.12, 1.64, 1.63, 1.60, 1.55, 1.35, 1.30, 1.06

Corresponding total number of genes of each Gene ontology term:
(In the same order as above)
195, 208, 72, 56, 255, 40, 67, 63, 23, 42, 25

Corresponding number of genes of each Gene ontology term with at least one TFBS (of selected TFs):
(In the same order as above)
49, 40, 17, 9, 33, 5, 13, 14, 3, 10, 6

Corresponding total number of TFBS (of selected TFs) for each Gene ontology term (in all genes):
(In the same order as above)
153, 128, 61, 38, 117, 19, 42, 45, 13, 22, 16

Corresponding number of selected TFs each Gene ontology term is involved with:
(In the same order as above)
7, 7, 7, 7, 7, 7, 7, 7, 6, 7, 6

TFs ranked according to mean of effect sizes in cluster:
V$MYCMAX_03, V$USF_02, VUSF_01, VUSF_Q6_01, VUSF_Q6, VARNT_01, V$MAX_01

Corresponding mean of effect sizes of each TF:
(In the same order as above)
2.51, 2.06, 2.05, 1.69, 1.69, 1.44, 1.26

Corresponding total number of TFBS for each TF (genome-wide):
(In the same order as above)
650, 649, 647, 664, 672, 655, 647

Corresponding total number of TFBS for each TF (in all genes in selected Gene ontology terms):
(In the same order as above)
110, 101, 103, 87, 92, 82, 79

Corresponding number of genes (of selected Gene ontology terms) each TF is involved with:
(In the same order as above)
64, 58, 59, 53, 49, 48, 49

Corresponding number of selected Gene ontology terms each TF is involved with:
(In the same order as above)
11, 11, 11, 11, 11, 10, 10

Cluster for columns 514 to 536, rows 348 to 352

Gene ontology terms

mucosal immune response | granulocyte macrophage colony-stimulating factor biosynthetic process | granulocyte macrophage colony-stimulating factor production | interleukin-1 receptor activity |

interleukin-6 production | tumor necrosis factor production | macrophage activation | inflammatory response | response to lipopolysaccharide | negative regulation of inflammatory response | regulation of tumor necrosis factor production | negative regulation of tumor necrosis factor production | positive regulation of tumor necrosis factor production | interleukin-1 receptor antagonist activity | acute inflammatory response | granuloma formation | lipopolysaccharide binding | cytokine biosynthetic process | interleukin-8 production | mRNA transcription | chronic inflammatory response | monocyte differentiation | hypersensitivity

TFs

V$STAT5B_01 | V$STAT5A_01 | V$STAT_01 | V$STAT3_01 | V$STAT1_01

Information

All related TFs:
(List of all TFs that are related to any of the PWMs)
STAT1alpha, STAT1beta, STAT2, STAT3, STAT4, STAT5A, STAT5B, STAT6

Ranked gene list:
(All genes of the selected Gene ontology terms with hits of any of the selected TFs, ranked according to the total number of TFBS)
ICAM1, CCL2, IL10, JUN, IL2RA, TNFRSF1A, CD14, SOCS3, EGR1, RELA, IRF1, TNF, FAS, CXCL10, LTA, IRF8, STAT3, AKT1, PTGER4, CD40LG, CCR1, S100A9, JUNB, CISH, IL2RB, CD69, TLR5, NOD2, NOD1, HSPD1, GPR132, TNFRSF1B, CEL, SOCS1, SLC7A2, PRKCDBP, PIK3CG, OSM, NOS3, NOS1, NDUFA2, IGL@, ICAM2, FOS, IL6ST, IL11, H2AFX, FANCM, DPEP1, CD46, THPO, TBCA, TAF8, STAT5A, RUNX1, RNASE2, RAB27A, NR3C1, MUC1, MLANA, LMNA, ERBB2, CHUK, ZFP36, TNFSF11, TNFRSF6B, TCEA2, SCARB1, MAP3K8, LIG4, FKBP4, F2RL1, CCR7, VIPR2, VIP, TOP1, TCEAL1, SYNJ2BP, STAT5B, SOD2, SHMT1, SELPLG, REV3L, RBM47, PTGES, PIGR, NSFL1C, NEDD9, MMP2, MMP12, MEFV, MAP4K4, MAP2K4, ITIH4, ING1, IL23A, IFNGR1, HPD, GLTSCR2, GJB6, GABARAP, FOXP1, CXCR4, CXCL11, CLEC11A, CDKN1A, CDK5R1, CCR6, BRCA2, ATF3, ANTXR1, ACAA2, VWF, TWIST1, TPPP, TIPARP, SFTPB, SERPING1, RPS14, PROC, PRAP1, POU4F1, PIK3R2, NFKB2, NDUFAB1, MSMB, MRE11A, MCHR2, MARK2, MAP3K14, LTC4S, KCNA3, IRF2, IL19, HSP90AA1, HNRNPC, HMGB1, HLF, GPI, GAPDH, GALNT6, DDX5, CTF1, CREBBP, CORO7, CD63, CD19, ATP2C1, ARG1, ADM

Corresponding total number of TFBS:
(For each gene listed above, the total number of TFBS for any of the selected TFs, multiplied by the number of selected Gene ontology terms containing that gene)
105, 84, 69, 60, 44, 36, 36, 35, 28, 26, 25, 23, 18, 16, 15, 15, 14, 14, 12, 12, 12, 10, 10, 10, 9, 9, 8, 8, 8, 8, 8, 7, 7, 6, 6, 6, 6, 6, 6, 6, 6, 6, 6, 5, 5, 5, 5, 5, 5, 4, 4, 4, 4, 4, 4, 4, 4, 4, 4, 4, 4, 4, 3, 3, 3, 3, 3, 3, 3, 3, 3, 3, 2, 2, 2, 2, 2, 2, 2, 2, 2, 2, 2, 2, 2, 2, 2, 2, 2, 2, 2, 2, 2, 2, 2, 2, 2, 2, 2, 2, 2, 2, 2, 2, 2, 2, 2, 2, 2, 2, 2, 1, 1, 1, 1, 1, 1, 1, 1, 1, 1, 1, 1, 1, 1, 1, 1, 1, 1, 1, 1, 1, 1, 1, 1, 1, 1, 1, 1, 1, 1, 1, 1, 1, 1, 1, 1, 1, 1

Corresponding number of selected Gene ontology terms each gene is involved with:
(In the same order as above)
21, 21, 23, 15, 11, 12, 18, 7, 7, 13, 5, 23, 6, 8, 15, 5, 7, 7, 4, 12, 3, 5, 2, 2, 9, 9, 8, 8, 4, 4, 2, 7, 7, 3, 2, 3, 6, 3, 2, 3, 6, 3, 3, 6, 5, 5, 1, 1, 5, 1, 2, 4, 2, 2, 2, 2, 1, 4, 1, 2, 1, 2, 2, 1, 3, 1, 1, 3, 3, 1, 1, 3, 3, 1, 1, 1, 2, 1, 2, 1, 1, 1, 1, 1, 2, 1, 2, 2, 2, 2, 1, 1, 1, 2, 2, 2, 1, 2, 1, 2, 2, 1, 2, 1, 2, 2, 2, 1, 1, 2, 1, 1, 1, 1, 1, 1, 1, 1, 1, 1, 1, 1, 1, 1, 1, 1, 1, 1, 1, 1, 1, 1, 1, 1, 1, 1, 1, 1, 1, 1, 1, 1, 1, 1, 1, 1, 1, 1

Corresponding number of selected TFs each gene is involved with:
(In the same order as above)
5, 4, 3, 4, 4, 3, 2, 5, 4, 2, 5, 1, 3, 2, 1, 3, 2, 2, 3, 1, 4, 2, 5, 5, 1, 1, 1, 1, 2, 2, 4, 1, 1, 2, 3, 2, 1, 2, 3, 2, 1, 2, 2, 1, 1, 1, 5, 5, 1, 5, 2, 1, 2, 2, 2, 2, 4, 1, 4, 2, 4, 2, 2, 3, 1, 3, 3, 1, 1, 3, 3, 1, 1, 2, 2, 2, 1, 2, 1, 2, 2, 2, 2, 2,

1, 2, 1, 1, 1, 1, 2, 2, 2, 1, 1, 1, 2, 1, 2, 1, 1, 2, 1, 2, 1, 1, 1, 2, 2, 1, 2, 2, 1, 1, 1, 1, 1, 1, 1, 1, 1, 1, 1, 1, 1, 1, 1, 1, 1, 1, 1, 1, 1, 1, 1, 1, 1, 1, 1, 1, 1, 1, 1, 1, 1, 1, 1, 1, 1

Gene ontology terms ranked according to mean of effect sizes in cluster:
granulocyte macrophage colony-stimulating factor production | granulocyte macrophage colony-stimulating factor biosynthetic process | positive regulation of tumor necrosis factor production | chronic inflammatory response | macrophage activation | hypersensitivity | negative regulation of tumor necrosis factor production | mRNA transcription | granuloma formation | response to lipopolysaccharide | inflammatory response | regulation of tumor necrosis factor production | acute inflammatory response | mucosal immune response | interleukin-1 receptor antagonist activity | interleukin-1 receptor activity | interleukin-6 production | negative regulation of inflammatory response | lipopolysaccharide binding | tumor necrosis factor production | interleukin-8 production | cytokine biosynthetic process | monocyte differentiation

Corresponding mean of effect sizes of each Gene ontology term:
(In the same order as above)
1.75, 1.72, 1.69, 1.66, 1.65, 1.61, 1.38, 1.28, 1.25, 1.22, 1.18, 1.16, 1.15, 1.14, 1.12, 0.93, 0.92, 0.86, 0.84, 0.81, 0.36, 0.29, 0.28

Corresponding total number of genes of each Gene ontology term:
(In the same order as above)
107, 109, 61, 22, 178, 234, 61, 130, 77, 226, 425, 86, 68, 52, 70, 128, 165, 40, 61, 209, 144, 108, 102

Corresponding number of genes of each Gene ontology term with at least one TFBS (of selected TFs):
(In the same order as above)
22, 22, 13, 6, 34, 39, 12, 31, 13, 46, 70, 15, 9, 10, 12, 21, 33, 7, 10, 37, 20, 15, 13

Corresponding total number of TFBS (of selected TFs) for each Gene ontology term (in all genes):
(In the same order as above)
48, 48, 34, 16, 77, 79, 31, 67, 27, 96, 136, 39, 23, 20, 30, 45, 64, 18, 18, 72, 43, 36, 28

Corresponding number of selected TFs each Gene ontology term is involved with:
(In the same order as above)
5, 5, 5, 5, 5, 5, 5, 5, 5, 5, 5, 5, 5, 5, 5, 5, 5, 5, 5, 5, 5, 5, 5

TFs ranked according to mean of effect sizes in cluster:
V$STAT3_01, V$STAT5B_01, V$STAT1_01, V$STAT_01, V$STAT5A_01

Corresponding mean of effect sizes of each TF:
(In the same order as above)
1.38, 1.34, 1.16, 0.94, 0.88

Corresponding total number of TFBS for each TF (genome-wide):
(In the same order as above)
609, 642, 647, 645, 678

Corresponding total number of TFBS for each TF (in all genes in selected Gene ontology terms):
(In the same order as above)
205, 237, 212, 223, 218

Corresponding number of genes (of selected Gene ontology terms) each TF is involved with:
(In the same order as above)
51, 59, 56, 52, 58

Corresponding number of selected Gene ontology terms each TF is involved with:

(In the same order as above)
23, 23, 23, 23, 23

Cluster for columns 454 to 531, rows 192 to 198

Gene ontology terms

crotonyl-CoA reductase activity | monocyte chemotaxis | localization of cell | chemotaxis | leukocyte migration | cell chemotaxis | positive chemotaxis | B cell activation | immunoglobulin production | B cell proliferation | immunoglobulin secretion | interferon-gamma production | T-helper 1 type immune response | response to host immune response | adaptive immune response | adaptive immune response based on somatic recombination of immune receptors built from immunoglobulin superfamily domains | chemokine production | interleukin-10 production | interleukin-12 production | cell maturation | MHC class I biosynthetic process | MHC class I protein binding | MHC class II biosynthetic process | MHC class II protein binding | antigen processing and presentation | immune response | type IV hypersensitivity | lymphocyte proliferation | tolerance induction | lymphocyte activation | interleukin-2 production | interleukin-2 receptor activity | T cell proliferation | interleukin-4 production | cytokine production | cytokine secretion | pathogenesis | interleukin-4 receptor activity | interleukin-5 production | evasion or tolerance of immune response of other organism during symbiotic interaction | tryptophan catabolic process | defense response to virus | innate immune response | tumor necrosis factor receptor activity | tumor necrosis factor receptor binding | positive regulation of NF-kappaB transcription factor activity | response to tumor necrosis factor | IkappaB kinase complex | NF-kappaB binding | interleukin-6 receptor activity | interleukin-1 beta production | interleukin-1 production | cytolysis | natural killer cell mediated cytotoxicity | natural killer cell receptor activity | naringenin-chalcone synthase activity | immature T cell proliferation in the thymus | eosinophil activation | leukocyte activation | monocyte activation | mucosal immune response | granulocyte macrophage colony-stimulating factor biosynthetic process | granulocyte macrophage colony-stimulating factor production | interleukin-1 receptor activity | interleukin-6 production | tumor necrosis factor production | macrophage activation | inflammatory response | response to lipopolysaccharide | negative regulation of inflammatory response | regulation of tumor necrosis factor production | negative regulation of tumor necrosis factor production | positive regulation of tumor necrosis factor production | interleukin-1 receptor antagonist activity | acute inflammatory response | granuloma formation | lipopolysaccharide binding | cytokine biosynthetic process

TFs

V$HNF4_Q6 | V$AR_Q6 | V$PR_Q2 | V$GR_Q6_01 | V$AML1_Q6 | V$AML1_01 | V$OSF2_Q6

Information

All related TFs:
(List of all TFs that are related to any of the PWMs)
AML1, AML1a, AML3, AML3-isoform2, AR, COUP, COUP-TF1, COUP-TF2, GR, GR-alpha, GR-beta, HNF-4, HNF-4alpha, HNF-4alpha1, HNF-4alpha2, HNF-4alpha3, HNF-4alpha4, HNF-4alpha7, HNF-4gamma, PR, PR-alpha, PR-beta, RUNX2-isoform2

Ranked gene list:
(All genes of the selected Gene ontology terms with hits of any of the selected TFs, ranked according to the total number of TFBS)
TNF, CD4, ITGB2, LTA, CCR7, TLR9, FASN, CSF2, LTB, TNFRSF4, IRF1, ISYNA1, VEGFA, AKT1, IL2RA, CXCR4, MYD88, STAT1, TNFRSF18, TNFRSF1A, LCK, SOCS1, PTPN6, PER1, STAT6, CD27, IL17C, PSMB8, CISH, CD44, GPR132, TG, PSMB10, MAP3K14, EDC4, STAT5A, TNFRSF1B, PTMA, NFATC1, MAP2K3, SOCS3, BCL2, CDK5R1, TAP2, LAG3, IKBKE, SILV, PTGER4, DUSP1, BCL6, ACTB, STAT5B, CSF1, CD68, FOXP3, CDKN1B, PLCG1, JUNB, IL7R, IL6R, FLJ32987,

CTRL, CSK, CD7, CD63, TNFRSF6B, SELPLG, PDCD1, OSM, IL16, EGR1, CCR10, CAPG, BCR, GATA3, CD5, APOM, ABCB9, TRAF1, TAP1, PTK2B, PRDM1, CTSD, ZAP70, TRAF6, SSSCA1, SIGIRR, S1PR4, PDE4A, ITGA5, ING1, FYN, TCIRG1, STK16, RPS6KA1, RARA, POU2F2, PNPLA2, PHB2, NFKBIE, IL23A, EHD1, DUSP2, CIC, CFL1, VIPR1, UBC, TAPBP, SMAD7, RXRB, RUNX1, RTEL1, RASSF5, PXN, NLRP3, LIF, HIVEP2, GABARAP, COL11A2, CCND2, CALR, BAK1, AGER, ADM, TNFRSF25, FOS, SLA, PIK3R1, NEDD9, DCTN3, CXCR5, CORO7, TUBGCP2, TNIP1, TNFRSF13C, SHC1, PKN1, UNC13D, TRAF5, SQSTM1, PTGER2, MAL, MADD, INPP5D, FGR, DECR1, CD300A, ZMYND10, ZFP36, ZC3H12A, TUSC2, SOS1, SLC25A22, S100A6, S100A4, RASSF1, PTPN7, PRDX2, MUC1, MAZ, LSP1, LRDD, ITGB7, GAPDH, ETS1, DUSP28, CDK5, ARVCF, TNFAIP3, SKP1, RNASEK, RIPK3, PSME1, PRAP1, NR4A1, MARK2, MAPKAPK2, LTB4R, LEF1, LCP2, ICAM3, HLA-DPB1, HLA-DMB, GUK1, GFI1, GALT, GALNT6, FHL3, FGF8, EOMES, DHX58, CFLAR, CDK4, CD74, CD226, CAMK2G, AMH, TSPAN4, TRADD, TPP1, TNFRSF14, TIRAP, STRA13, SFTPC, SEMA4D, RPLP2, PTGDS, PDGFB, LMNA, HSP90B1, CD79B, CAPN10, ATXN1, ZFPM1, TNFRSF12A, SKAP1, RUNX3, PRF1, PLEKHF1, PAX5, P2RY11, NOS3, MST1, MMP11, LY6E, LTB4R2, IL10RA, HRAS, GRK6, GNAS, CD47, CD247, ACSL6, VIM, VDAC1, USF1, UROD, TRAIP, TNFSF14, TIPARP, PSME2, PSENEN, PML, PIN1, NGFR, MRPL28, MC1R, MAP3K11, MAG, KLF6, KCNN4, IRS1, ILK, HSPA1B, FUT7, FOXO3, EFNA1, DLX2, DEAF1, DCXR, CRTC2, CD3D, CCND3, CAMKV, BCL9, ATN1, ANGPT2, ACD, VAC14, TRERF1, SSFA2, SPON2, SPHK2, SEMA3B, PSMP, POLD4, PLXNC1, PCSK7, NR4A2, NOTCH1, NOL3, MTX1, MT2A, MLL, MBNL2, LTC4S, ILF3, HIC1, GORASP2, FOXP1, FLNA, FLII, ETV6, DPCR1, DGCR2, DDR1, CDKN2C, CD79A, CD6, CCL27, CACNB3, BHLHB2, BCL2L11, AGPAT1, UCP2, TXNIP, TCEB2, TAC3, SUFU, ROBLD3, PTCH1, PNKD, NOTCH4, NHP2L1, MAPK12, MAPK11, LMAN2, LEPREL2, HLA-DPA1, HIVEP3, GNB2L1, EXOSC1, EFNA2, ECE1, DIABLO, DHCR7, DAPP1, CNTFR, CEACAM1, CDKN1C, CDC37, BATF, APBB1, AIRE

Corresponding total number of TFBS:
(For each gene listed above, the total number of TFBS for any of the selected TFs, multiplied by the number of selected Gene ontology terms containing that gene)
532, 476, 350, 287, 196, 182, 180, 174, 133, 126, 119, 116, 112, 108, 106, 102, 99, 93, 84, 81, 80, 77, 70, 70, 64, 63, 60, 56, 56, 56, 48, 45, 42, 42, 40, 39, 38, 36, 35, 35, 34, 33, 32, 30, 30, 30, 28, 28, 28, 28, 28, 26, 26, 26, 24, 24, 21, 21, 21, 21, 21, 21, 21, 21, 21, 20, 20, 20, 20, 20, 20, 20, 20, 20, 18, 18, 18, 18, 16, 16, 16, 16, 16, 15, 15, 15, 15, 15, 15, 15, 15, 15, 14, 14, 14, 14, 14, 14, 14, 14, 14, 14, 14, 14, 12, 12, 12, 12, 12, 12, 12, 12, 12, 12, 12, 12, 12, 12, 12, 12, 12, 12, 11, 11, 10, 10, 10, 10, 10, 10, 9, 9, 9, 9, 9, 8, 8, 8, 8, 8, 8, 8, 8, 8, 8, 7, 7, 7, 7, 7, 7, 7, 7, 7, 7, 7, 7, 7, 7, 7, 7, 7, 7, 7, 7, 7, 6, 6, 6, 6, 6, 6, 6, 6, 6, 6, 6, 6, 6, 6, 6, 6, 6, 6, 6, 6, 6, 6, 6, 6, 6, 6, 6, 6, 6, 5, 5, 5, 5, 5, 5, 5, 5, 5, 5, 5, 5, 5, 5, 5, 5, 4, 4, 4, 4, 4, 4, 4, 4, 4, 4, 4, 4, 4, 4, 4, 4, 4, 4, 4, 4, 4, 3, 3, 3, 3, 3, 3, 3, 3, 3, 3, 3, 3, 3, 3, 3, 3, 3, 3, 3, 3, 3, 3, 3, 3, 3, 3, 3, 3, 3, 3, 3, 3, 3, 3, 3, 3, 2, 2, 2, 2, 2, 2, 2, 2, 2, 2, 2, 2, 2, 2, 2, 2, 2, 2, 2, 2, 2, 2, 2, 2, 2, 2, 2, 2, 2, 2, 2, 2, 2, 2, 2, 2, 1, 1, 1, 1, 1, 1, 1, 1, 1, 1, 1, 1, 1, 1, 1, 1, 1, 1, 1, 1, 1, 1, 1, 1, 1, 1, 1, 1

Corresponding number of selected Gene ontology terms each gene is involved with:
(In the same order as above)
76, 68, 50, 41, 28, 26, 30, 58, 19, 21, 17, 29, 16, 18, 53, 17, 33, 31, 14, 27, 20, 11, 10, 10, 16, 21, 15, 8, 8, 28, 12, 9, 6, 6, 20, 13, 19, 6, 7, 5, 17, 11, 16, 6, 6, 5, 14, 7, 7, 4, 4, 13, 26, 13, 24, 4, 3, 3, 7, 7, 3, 3, 3, 7, 7, 5, 5, 5, 4, 10, 10, 4, 10, 10, 3, 18, 3, 3, 4, 8, 4, 8, 4, 5, 15, 3, 3, 3, 5, 5, 5, 5, 2, 2, 2, 2, 2, 2, 7, 2, 14, 2, 2, 2, 2, 6, 12, 4, 2, 2, 3, 2, 2, 3, 4, 12, 4, 2, 4, 3, 6, 2, 3, 2, 11, 11, 2, 5, 5, 2, 10, 2, 3, 3, 9, 3, 3, 2, 4, 8, 8, 2, 2, 2, 4, 8, 4, 1, 1, 1, 1, 7, 1, 1, 1, 1, 1, 1, 1, 7, 1, 1, 1, 1, 1, 1, 1, 1, 1, 6, 1, 1, 3, 3, 6, 1, 3, 6, 3, 1, 6, 6, 6, 3, 1, 1, 2, 2, 1, 1, 1, 2, 6, 1, 6, 3, 1, 1, 1, 5, 1, 5, 5, 1, 5, 1, 1, 5, 1, 5, 5, 5, 1, 1, 1, 4, 1, 1, 2, 2, 4, 1, 4, 2, 1, 1, 1, 4, 1, 1, 1, 4, 4, 1, 1, 3, 1, 1, 3, 1, 3, 3, 1, 3, 1, 3, 1, 1, 1, 1, 1, 1, 3, 1, 3, 1, 1, 1, 3, 1, 3, 1, 1, 3, 1, 1, 1, 3, 1, 2, 1, 2, 1, 2, 1, 2, 1, 1, 1, 2, 2, 2, 1, 1, 1, 1, 1, 2, 1, 2, 2, 1, 1, 1, 1, 2, 1, 2, 2, 1, 2, 1, 1, 1, 1, 1, 1, 1, 1, 1, 1, 1, 1, 1, 1, 1, 1, 1, 1, 1, 1, 1, 1, 1, 1, 1, 1, 1, 1, 1, 1, 1, 1

Corresponding number of selected TFs each gene is involved with:
(In the same order as above)
7, 7, 7, 7, 7, 7, 6, 3, 7, 6, 7, 4, 7, 6, 2, 6, 3, 3, 6, 3, 4, 7, 7, 7, 4, 3, 4, 7, 7, 2, 4, 5, 7, 7, 2, 3, 2, 6, 5, 7, 2, 3, 2, 5, 5, 6, 2, 4, 4, 7, 7, 2, 1, 2, 1, 6, 7, 7, 3, 3, 7, 7, 7, 3, 3, 4, 4, 4, 5, 2, 2, 5, 2, 2, 6, 1, 6, 6, 4, 2, 4, 2, 4, 3, 1, 5, 5, 5, 3, 3, 3, 3, 7, 7, 7, 7, 7, 7, 2, 7, 1, 7, 7, 7, 7, 2, 1, 3, 6, 6, 4, 6, 6, 4, 3, 1, 3, 6, 3, 4, 2, 6, 4, 6, 1, 1,

5, 2, 2, 5, 1, 5, 3, 3, 1, 3, 3, 4, 2, 1, 1, 4, 4, 4, 2, 1, 2, 7, 7, 7, 7, 1, 7, 7, 7, 7, 7, 7, 1, 7, 7, 7, 7, 7, 7, 7, 7, 7, 1, 6, 6, 2, 2, 1, 6, 2, 1, 2, 6, 1, 1, 1, 2, 6, 6, 3, 3, 6, 6, 6, 3, 1, 6, 1, 2, 6, 6, 5, 1, 5, 1, 1, 5, 1, 5, 5, 1, 5, 1, 1, 1, 5, 5, 4, 1, 4, 4, 2, 2, 1, 4, 1, 2, 4, 4, 4, 1, 4, 4, 4, 1, 1, 4, 3, 1, 3, 3, 1, 3, 1, 1, 3, 1, 3, 1, 3, 3, 3, 3, 3, 3, 1, 3, 1, 3, 3, 3, 1, 3, 1, 3, 3, 1, 3, 3, 3, 1, 3, 1, 2, 1, 2, 1, 2, 1, 2, 2, 2, 1, 1, 1, 2, 2, 2, 2, 2, 1, 2, 1, 1, 2, 2, 2, 2, 1, 2, 1, 1, 2, 1, 2, 2, 2, 2, 1, 1, 1, 1, 1, 1, 1, 1, 1, 1, 1, 1, 1, 1, 1, 1, 1, 1, 1, 1, 1, 1, 1, 1, 1, 1, 1, 1, 1, 1

Gene ontology terms ranked according to mean of effect sizes in cluster:
cell maturation | acute inflammatory response | adaptive immune response | immature T cell proliferation in the thymus | response to host immune response | cell chemotaxis | chemokine production | positive chemotaxis | T-helper 1 type immune response | cytokine production | cytokine secretion | interleukin-4 production | MHC class II biosynthetic process | MHC class II protein binding | tryptophan catabolic process | immune response | antigen processing and presentation | leukocyte migration | T cell proliferation | granulocyte macrophage colony-stimulating factor production | innate immune response | immunoglobulin production | interleukin-2 receptor activity | granulocyte macrophage colony-stimulating factor biosynthetic process | granuloma formation | adaptive immune response based on somatic recombination of immune receptors built from immunoglobulin superfamily domains | type IV hypersensitivity | lymphocyte activation | macrophage activation | interleukin-4 receptor activity | interleukin-12 production | response to lipopolysaccharide | localization of cell | positive regulation of tumor necrosis factor production | positive regulation of NF-kappaB transcription factor activity | interleukin-5 production | mucosal immune response | leukocyte activation | monocyte activation | natural killer cell receptor activity | regulation of tumor necrosis factor production | interleukin-6 production | naringenin-chalcone synthase activity | eosinophil activation | interleukin-1 receptor antagonist activity | cytokine biosynthetic process | interleukin-10 production | monocyte chemotaxis | crotonyl-CoA reductase activity | interleukin-1 receptor activity | B cell activation | response to tumor necrosis factor | cytolysis | evasion or tolerance of immune response of other organism during symbiotic interaction | inflammatory response | natural killer cell mediated cytotoxicity | interleukin-2 production | lymphocyte proliferation | B cell proliferation | negative regulation of inflammatory response | tolerance induction | interferon-gamma production | negative regulation of tumor necrosis factor production | MHC class I biosynthetic process | MHC class I protein binding | lipopolysaccharide binding | tumor necrosis factor production | pathogenesis | tumor necrosis factor receptor binding | tumor necrosis factor receptor activity | chemotaxis | defense response to virus | interleukin-1 production | NF-kappaB binding | immunoglobulin secretion | interleukin-1 beta production | interleukin-6 receptor activity | IkappaB kinase complex

Corresponding mean of effect sizes of each Gene ontology term:
(In the same order as above)
1.93, 1.92, 1.81, 1.75, 1.75, 1.74, 1.74, 1.73, 1.71, 1.64, 1.55, 1.54, 1.46, 1.46, 1.44, 1.44, 1.41, 1.40, 1.39, 1.31, 1.31, 1.30, 1.29, 1.28, 1.28, 1.26, 1.25, 1.24, 1.24, 1.18, 1.18, 1.18, 1.18, 1.17, 1.17, 1.10, 1.08, 1.06, 1.05, 1.03, 1.02, 1.01, 1.00, 0.99, 0.98, 0.97, 0.97, 0.97, 0.97, 0.96, 0.96, 0.93, 0.93, 0.91, 0.90, 0.85, 0.82, 0.82, 0.81, 0.81, 0.76, 0.75, 0.68, 0.67, 0.67, 0.67, 0.67, 0.67, 0.66, 0.60, 0.59, 0.49, 0.47, 0.38, 0.35, 0.33, 0.24, -0.07

Corresponding total number of genes of each Gene ontology term:
(In the same order as above)
224, 68, 148, 42, 37, 94, 155, 87, 59, 455, 243, 123, 181, 178, 41, 530, 315, 145, 188, 107, 246, 155, 189, 109, 77, 131, 131, 232, 178, 87, 150, 226, 38, 61, 550, 57, 52, 108, 117, 77, 86, 165, 50, 71, 70, 108, 166, 52, 52, 128, 116, 185, 179, 141, 425, 33, 182, 158, 88, 40, 109, 106, 61, 195, 197, 61, 209, 1.1K, 214, 213, 371, 110, 31, 146, 51, 21, 104, 107

Corresponding number of genes of each Gene ontology term with at least one TFBS (of selected TFs):
(In the same order as above)
42, 8, 23, 9, 7, 15, 22, 14, 11, 72, 40, 22, 30, 29, 9, 77, 55, 20, 32, 18, 27, 22, 35, 18, 11, 17, 21, 43, 25, 18, 22, 40, 11, 10, 97, 9, 7, 11, 12, 7, 14, 26, 7, 10, 11, 18, 24, 5, 7, 20, 22, 31, 21, 21, 56, 4, 35, 24, 16, 5, 15, 14, 9, 30, 30, 7, 25, 152, 41, 40, 45, 16, 3, 21, 8, 2, 17, 18

Corresponding total number of TFBS (of selected TFs) for each Gene ontology term (in all genes):

(In the same order as above)
165, 45, 82, 42, 31, 69, 90, 58, 43, 269, 151, 86, 115, 114, 42, 274, 195, 84, 119, 66, 123, 79, 128, 66, 42, 59, 87, 164, 109, 67, 93, 148, 36, 45, 349, 44, 30, 51, 55, 40, 58, 101, 29, 35, 44, 69, 92, 29, 35, 70, 70, 121, 86, 78, 199, 22, 131, 86, 57, 27, 63, 49, 38, 119, 119, 26, 108, 503, 128, 125, 145, 67, 13, 86, 26, 10, 47, 58

Corresponding number of selected TFs each Gene ontology term is involved with:
(In the same order as above)
7, 7, 7, 7, 7, 7, 7, 7, 7, 7, 7, 7, 7, 7, 7, 7, 7, 7, 7, 7, 7, 7, 7, 7, 7, 7, 7, 7, 7, 7, 7, 7, 7, 7, 7, 7, 7, 7, 7, 7, 7, 7, 7, 7, 7, 7, 7, 7, 7, 7, 7, 7, 7, 7, 7, 7, 7, 7, 7, 7, 7, 7, 7, 7, 7, 7, 7, 7, 7, 7, 7, 7, 7, 7, 7, 7, 7, 7

TFs ranked according to mean of effect sizes in cluster:
V$AML1_Q6, V$AML1_01, V$HNF4_Q6, V$GR_Q6_01, VPR_Q2, VAR_Q6, V$OSF2_Q6

Corresponding mean of effect sizes of each TF:
(In the same order as above)
1.49, 1.38, 1.23, 0.97, 0.85, 0.85, 0.78

Corresponding total number of TFBS for each TF (genome-wide):
(In the same order as above)
718, 710, 710, 720, 698, 699, 710

Corresponding total number of TFBS for each TF (in all genes in selected Gene ontology terms):
(In the same order as above)
1134, 1119, 992, 1021, 984, 966, 908

Corresponding number of genes (of selected Gene ontology terms) each TF is involved with:
(In the same order as above)
161, 168, 152, 177, 175, 159, 146

Corresponding number of selected Gene ontology terms each TF is involved with:
(In the same order as above)
78, 78, 78, 78, 78, 78, 78

Cluster for columns 1161 to 1204, rows 97 to 108

Gene ontology terms

lung development | ossification | osteoblast differentiation | cartilage development | chondrocyte differentiation | skeletal development | gut development | neuron development | odontogenesis | transdifferentiation | kidney development | mesoderm formation | heart development | skeletal muscle development | gliogenesis | stem cell differentiation | epithelial to mesenchymal transition | fibroblast growth factor receptor activity | fibroblast growth factor receptor binding | neural tube closure | compound eye development | camera-type eye development | eye development | neural tube formation | system development | cell-cell signaling | cell fate specification | lens development in camera-type eye | neuron migration | N-acetyltransferase activity | developmental process | cell | growth | provirus | induction | apoptosis | cell proliferation | response to osmotic stress | luciferin monooxygenase activity | transcription factor complex | decidualization | liver development | myoblast differentiation | regulation of cell proliferation

TFs

V$STAT6_01 | V$STAT5A_03 | V$STAT4_01 | V$STAT5A_04 | V$STAT1_03 | V$CEBP_Q3 | V$PAX2_02 | V$HMGIY_Q6 | V$HOXA4_Q2 | V$TST1_01 | V$NFAT_Q6 | V$NFAT_Q4_01

Information

All related TFs:
(List of all TFs that are related to any of the PWMs)
ANF-2, C/EBP, C/EBPalpha, C/EBPalpha(p20), C/EBPalpha(p30), C/EBPbeta, C/EBPbeta(LAP), C/EBPbeta(p20), C/EBPbeta(p34), C/EBPbeta(p35), C/EBPdelta, C/EBPepsilon, C/EBPgamma, HMG, HMG-Y, HMGI-C, HOXA4, NF-AT, NF-AT1, NF-AT1C, NF-AT2, NF-AT3, NF-AT4, POU3F1, Pax-2, Pax-2.1, Pax-2.2, Pax-2a, Pax-2b, STAT1, STAT1alpha, STAT4, STAT5A, STAT6

Ranked gene list:
(All genes of the selected Gene ontology terms with hits of any of the selected TFs, ranked according to the total number of TFBS)
LBX1, CTNNB1, RUNX1, FGF8, PAX6, CD44, LEF1, VIM, CDKN1B, HES1, STAT1, SSSCA1, SMAD7, CXCR4, GLI1, RUNX3, ID2, RARG, PTGER4, ETS1, TGFBR2, ID3, TNF, NFKBIA, CDC25C, RARA, PTCH1, MAP2K3, TFAP2A, SHC1, PITX3, NR4A2, IRS1, HIST1H4I, FYN, CDK6, CDK4, STAT3, WNT1, BCL2L1, MEF2D, KLF6, JUN, IRF1, IL23A, GFI1, CSK, CISH, CDK2, CCND3, CCND2, NR3C1, ILK, DUSP6, COL11A2, NKX2-2, DLX2, DLL1, CFL1, CDKN2C, SMAD3, NFATC1, JUNB, EGR1, VEGFA, SOX2, PTPN6, PTMA, PROX1, PRDM1, PER1, NFATC3, MAF, LTB, LTA, ITGA5, DUSP1, BTG1, ACVR1, WNT10B, TNFRSF1A, RBPJ, PIK3R1, AKT1, NEUROG2, HSPA8, HAND2, CITED2, CD4, BHLHB2, S100A4, NR2F2, GSK3B, STAT5A, PLCG1, DDX5, CD68, ADM, VDR, PDCD4, MXI1, GATA3, FST, CDK5, BRD2, BCL2L11, ARID2, PSMP, MYB, MARK2, EGR2, BCL2, RASSF1, NR3C2, NR2F1, CCR7, TAF8, MYC, MAP3K1, HIST4H4, GGPS1, FLJ32987, E2F3, DDIT3, CSF1, SOCS1, SGK1, S1PR1, RHOC, NFYA, JARID2, CTLA4, CREB1, ARL4C, IGF1R, TUSC2, TNFAIP3, TIPARP, RASSF5, MAPK14, MAP3K12, HOXB7, HOXB4, HOXA10, GNAS, FOXP1, FBRS, BCL6, PAX5, FGF9, ANXA6, ALCAM, SOS1, SMARCA2, PSMB9, MBNL1, IL7R, HOXD13, FOSL2, DCTN3, SIX1, PTGER2, NEDD9, IRF2, EPHA4, ZC3H12D, PDLIM5, NFKBIE, LHX1, HSPA1B, ETV3, ESR1, CKS1B, CDKN1A, ANG, TRAF3, SOCS2, MEIS2, H2AFX, FOXD3, DAXX, CYP27B1, SILV, PITX2, OSM, HOXA5, BARHL2, SPRY1, HMGA1, GAPDH, CASP2, ZFP36L2, ZFP36L1, ZFP36, WIBG, USF1, UNK, TRERF1, TRAF5, TGIF1, TCF7, TAP1, SOCS3, SHOX2, SATB1, S100A6, S100A10, RREB1, RHOF, RERE, PSMB8, PRKCE, NR4A3, NNAT, NIN, MLL2, MLL, MGAT5, LMO4, IL2RA, IKZF1, HOXA9, HOXA11, HDAC7, GIMAP5, EOMES, ENO2, DPAGT1, CUGBP2, CLIC1, CLCN3, BUB3, BCL11B, ATP2B4, ARL6IP5, ARHGEF2, TGFB3, SRF, SH2B3, RNASEK, PPP2CA, PAX3, OSR1, NAB2, MAP3K14, LDB1, HOXB2, CYB561D2, CBX8, BMI1, BACH2, ZEB1, TTF2, PTK2B, LCK, KSR1, HSP90B1, SLA, SATB2, PURA, POU4F1, PCNA, KLF9, GRAP2, GDNF, EIF5A, DLX1, BMF, BAK1, ANP32A, ADAMTS4, TXNIP, SLC39A1, SLC38A2, RTN4, OTX2, MNT, MAZ, JAK1, ITPR1, IKBKE, HOXA2, GRB2, GABARAP, EFNA1, DUSP5, C2orf28, BCL11A, ALDOA, AHSA1, STK16, SLC4A2, SART3, NARG1, MAP4K4, IL16, GIT1, FOXO1, FOXG1, EP300, EBF1, CD3D, CADM1, ZIC1, TG, PSMC5, PRDX2, POLD4, PDE4B, NDRG1, ING1, GATA2, DVL2, CYP26B1, ZNRD1, ZNF292, ZIC2, WIPF1, UBTF, TRIM3, PAX2, KAT2B, GDF11, CACYBP, TP53, SMAD4, CCND1, ZMYND10, ZEB2, VEGFB, TSHZ1, TPT1, THRA, TGFBR3, STAT6, SKP1, RORC, RASSF2, PPP1R1B, PIK3IP1, PIK3CA, PFN1, PDGFB, NR4A1, MT2A, MGAT1, MAPRE2, MAPKAPK3, LBH, KLF3, ITGB1, ICOS, HOXC8, HOXC6, HOXC4, HOXB5, HOXB3, HOXA7, HNRNPC, HIVEP2, H3F3B, FOS, FLI1, ETV1, E2F4, E2F2, DIAPH1, CUL1, CFLAR, CD69, CALM2, BAT3, ATXN1, ARID1B, ARID1A, AHNAK, UBC, TUSC4, TNIP1, SLC38A1, RHOG, RFTN1, PPP1R10, PIK3CG, PDE6D, MYO1G, MVP, MLLT10, KLF7, HOXB6, FYB, FOXJ2, ETV6, CRTC2, CDKL3, CD3G, C16orf53, AGPAT1, TOB2, TNFAIP8, TAPBP, STOML2, STAT5B, SLC35B2, SKAP1, SERTAD2, RPS6KA1, POU2F1, PLK3, PLEC1, NFYC, MYD88, HOXD11, HOXB9, HOXA1, HNRNPL, HNRNPH3, HIVEP1, GALT, CRIP1, CDC42SE1, CD247, CAMK2G, ADD3, VEZF1, TAP2, TAOK2, TAGLN2, SHMT2, RB1, RAG1AP1, RAF1, PKM2, PHB2, NIPBL, MAT2B, LCP1, LAG3, HSP90AB1, HNRNPA2B1, HECA, GNAI2, ENO3, DYRK2, DUSP2, DLX6, CD82, AXUD1, ARHGEF1, ARHGDIB, ADIPOR1, WNK1, WDR1, WBP7, TPP1, TMC6, TCF7L2, STIM1, SOX4, SLC20A1, PPARA, PIK3CD, PHF20, NP, NF1, NAT13, MZF1, MGAT4A, MCAM, MAX, MAPKAPK2, MAP2K6, MAML2, LRIG1, IL6R, IGF1, HYAL2, HSPA1A, HEY2, GTF3A, GIN1, FUS, FBXW11, ETV5, ENC1, CYTIP, CYLD, CTDSP2, CD47, CD27, CALM1, APEX1, VAMP8, TPM3, TMEM115, SLC9A3R1, SGMS1, REPIN1, PTPN7, PDGFRA, NOS2, MIA, MDGA1, LRDD, LGALS1, ISYNA1, ICK, GAD1, FOSB, EMILIN1, CIDEB, CD40, ARFIP2,

ACVR2A, VAMP2, UGP2, UCP2, TXNDC12, TWIST1, TRADD, TOB1, STK17B, RNF31, PXN, PSME2, PML, PEA15, MEIS1, MAL, LTBP4, LCP2, KRAS, KLF2, KCNA3, ITGB7, IL2, GNB2L1, GIT2, GGNBP2, FHL3, EVL, EIF4A2, EFNB2, DKFZp779C0757, DGKZ, DAD1, CXCR5, CNTNAP1, CCR10, CAP1, CALR, B3GNT2, ATF4, ARID4B, AKTIP, ADNP, ZNF384, UROD, TNFRSF1B, RGL2, PTTG1, PRKCD, PPP1R9B, PIM1, PELP1, PCF11, PARP1, OSR2, NFAT5, NCOA4, MKNK1, MGA, MBD1, MAP3K5, HSPD1, HDGF, HCLS1, GLG1, FOXO3, ESRRA, EMP3, DCTD, DBP, COL2A1, CD5, C17orf81, ANXA1, AKT1S1, ABCD2, ZAP70, VASP, TUBB, TRAF1, TNFSF8, SLAMF1, SH3BGRL3, SELPLG, RNF41, QKI, PRG4, PPM1B, POU3F2, PHIP, PHF1, OBFC2B, NDE1, MLLT3, MLC-B, MDC1, MAPK13, MADD, LFNG, LDLR, KLF5, ITGA6, HOXA4, GLRX, GCS1, GALE, FGF11, FAIM3, ERBB2IP, EIF4G2, EGLN2, DUSP10, CDC25B, CD79A, CD63, CCR6, BRAF, ATF2, AQP3, ADORA2A, ACTN1, ABI3, ABCG1, AATF, WWP1, VGF, TBPL1, SUFU, STAG1, ST8SIA1, SLC5A6, SLC25A22, SLC12A6, SEPT7, SEMA3B, REV3L, PTGES3, PRMT5, PPP1R16B, PNKD, PDCD10, PCP2, NRM, NFIL3, NBPF1, MEA1, MAP3K8, LRBA, LMNA, IRF8, IL11RA, IFNGR1, HSPE1, HNRNPK, FRS3, FRAG1, FBXW7, FBL, EDAR, DDIT4, CNTFR, CD226, CAD, BECN1, BCLAF1, BANF1, ACAA1, ZYX, VCP, TMSB4X, TLX2, TLE4, TCIRG1, TBX15, TBK1, TAX1BP1, SSBP1, SPSB2, SPRY4, SP4, SLC26A8, SERPINI1, ROCK1, RGS16, REV1, RBBP6, RASGRP1, RAD51L1, PTPN22, PRCP, PPP3R1, POFUT1, PLAGL2, PEG10, PCSK7, PAX9, P2RX3, OTX1, OSGEP, NR1H2, NOG, NLRP3, NAB1, MTP18, MAP3K11, MAP1A, JMJD2C, JARID1B, IVNS1ABP, ITGA7, ITGA1, INTS6, IMPDH2, IMP3, IL10RA, IFITM1, IFIH1, IDH1, HS2ST1, HNRNPU, HNRNPD, HLA-B, HIST1H2AE, HIPK3, HIPK1, HAX1, GSX1, FXYD5, FUBP1, ELF5, ELAVL4, ECE1, DUT, DR1, DNAJC14, CXCR7, CST6, CSRP1, CRIP2, CLCN2, CLCF1, CEBPE, CD53, CD200R1, BPGM, BBC3, AIM1, ACTR3, ACSL5, ABCF2, ZIK1, ZBTB6, YES1, WDR26, USO1, UNC13D, TYROBP, TUBGCP2, TTC9, TPMT, TOPBP1, TMEM102, TGFBRAP1, ST6GAL1, SSR1, SRP54, SPRED2, SOX13, SLC23A1, SIM1, SIAH2, SENP1, SACM1L, RCHY1, RBKS, RAB6A, RAB27A, PVRL3, PRPF4B, POU2AF1, POMGNT1, PLEKHG5, PFDN5, PER2, OVCA2, OPTN, NXPH4, NUP37, NR2E1, NMT1, NCKAP1L, MTMR11, MRO, MAGI3, LTBP2, LIG4, KIF2C, KCTD10, KCNN4, ITPR2, HSF4, HS6ST1, HLA-DMA, HLA-C, HIST1H2AM, HCST, H3F3A, GYPC, FGF14, EXOSC7, ERGIC2, EPB41L2, EPB41, EMP1, ELL2, EIF5B, EEF2, EEF1A1, DYNLRB1, DUSP4, DIDO1, DHH, DEDD2, DEDD, DDX1, CTSC, CSNK1A1, CLDN12, CHSY1, CHIC2, CEP57, CCDC72, C7orf23, C1orf103, C10orf4, BRCA2, BID, BACH1, ARMCX2, APPL1, ANKHD1, ADIPOR2, AAMP

Corresponding total number of TFBS:
(For each gene listed above, the total number of TFBS for any of the selected TFs, multiplied by the number of selected Gene ontology terms containing that gene)
374, 264, 204, 198, 192, 176, 168, 156, 132, 126, 121, 121, 120, 120, 114, 110, 110, 108, 108, 108, 105, 99, 96, 96, 96, 91, 90, 90, 88, 88, 84, 84, 84, 84, 84, 84, 84, 80, 77, 77, 72, 72, 72, 72, 72, 72, 72, 72, 72, 72, 72, 70, 70, 70, 70, 66, 66, 66, 66, 66, 64, 64, 63, 63, 60, 60, 60, 60, 60, 60, 60, 60, 60, 60, 60, 60, 60, 60, 60, 56, 56, 56, 56, 56, 55, 55, 55, 55, 55, 55, 54, 54, 54, 50, 50, 50, 50, 50, 48, 48, 48, 48, 48, 48, 48, 48, 48, 45, 45, 45, 45, 45, 44, 44, 44, 44, 42, 42, 42, 42, 42, 42, 42, 42, 40, 40, 40, 40, 40, 40, 40, 40, 40, 39, 36, 36, 36, 36, 36, 36, 36, 36, 36, 36, 36, 36, 36, 35, 35, 35, 35, 33, 33, 33, 33, 33, 33, 33, 33, 32, 32, 32, 32, 32, 30, 30, 30, 30, 30, 30, 30, 30, 30, 30, 28, 28, 28, 28, 28, 28, 28, 27, 27, 27, 27, 27, 25, 25, 25, 25, 24, 24, 24, 24, 24, 24, 24, 24, 24, 24, 24, 24, 24, 24, 24, 24, 24, 24, 24, 24, 24, 24, 24, 24, 24, 24, 24, 24, 24, 24, 24, 24, 24, 24, 24, 24, 24, 24, 24, 24, 24, 24, 24, 24, 24, 22, 22, 22, 22, 22, 22, 22, 22, 22, 22, 22, 22, 22, 22, 22, 21, 21, 21, 21, 21, 21, 20, 20, 20, 20, 20, 20, 20, 20, 20, 20, 20, 20, 20, 20, 18, 18, 18, 18, 18, 18, 18, 18, 18, 18, 18, 18, 18, 18, 18, 18, 18, 18, 18, 16, 16, 16, 16, 16, 16, 16, 16, 16, 16, 16, 16, 16, 15, 15, 15, 15, 15, 15, 15, 15, 15, 15, 15, 14, 14, 14, 14, 14, 14, 14, 14, 14, 14, 13, 13, 13, 12, 12, 12, 12, 12, 12, 12, 12, 12, 12, 12, 12, 12, 12, 12, 12, 12, 12, 12, 12, 12, 12, 12, 12, 12, 12, 12, 12, 12, 12, 12, 12, 12, 12, 12, 12, 12, 12, 12, 12, 12, 12, 12, 12, 12, 12, 12, 12, 12, 12, 12, 12, 12, 11, 11, 11, 11, 11, 11, 11, 11, 11, 11, 11, 11, 11, 11, 11, 11, 11, 11, 11, 11, 11, 11, 10, 10, 10, 10, 10, 10, 10, 10, 10, 10, 10, 10, 10, 10, 10, 10, 10, 10, 10, 10, 10, 10, 10, 10, 10, 10, 9, 9, 9, 9, 9, 9, 9, 9, 9, 9, 9, 9, 9, 9, 9, 9, 9, 9, 9, 9, 9, 9, 9, 9, 9, 9, 9, 8, 8, 8, 8, 8, 8, 8, 8, 8, 8, 8, 8, 8, 8, 8, 8, 8, 8, 8, 8, 8, 8, 8, 8, 8, 8, 8, 8, 8, 8, 8, 8, 8, 8, 8, 8, 8, 8, 8, 8, 8, 7, 7, 7, 7, 7, 7, 7, 7, 7, 7, 7, 7, 7, 7, 7, 7, 7, 7, 7, 7, 7, 7, 6, 6, 6, 6, 6, 6, 6, 6, 6, 6, 6, 6, 6, 6, 6, 6, 6, 6, 6, 6, 6, 6, 6, 6, 6, 6, 6, 6, 6, 6, 6, 6, 6, 6, 6, 6, 6, 6, 6, 6, 6, 6, 5, 5, 5, 5, 5, 5, 5, 5, 5, 5, 5, 5, 5, 5, 5, 5, 5, 5, 5, 5, 5, 5, 5, 5, 5, 5, 5, 5, 5, 5, 5, 5, 5, 4, 4, 4, 4, 4, 4, 4, 4, 4, 4, 4, 4, 4, 4, 4, 4, 4, 4, 4, 4, 4, 4, 4, 4, 4, 4, 4, 4, 4, 4, 4, 4, 4, 4, 4, 4, 4, 4, 4, 4, 4, 4, 4, 4, 4, 4, 4, 4, 3, 3, 3, 3, 3, 3, 3, 3, 3, 3, 3, 3, 3, 3, 3, 3, 3, 3, 3, 3, 3, 3, 3,

3, 3, 3, 3, 3, 3, 3, 3, 3, 3, 3, 3, 3, 3, 3, 3, 3, 3, 3, 3, 2, 2, 2, 2, 2, 2, 2, 2, 2, 2, 2, 2, 2, 2, 2, 2, 2, 2, 2, 2, 2, 2, 2, 2, 2, 2, 2, 2, 2, 2, 2, 2, 2, 2, 2, 2, 2, 2, 2, 2, 2, 2, 2, 2, 2, 2, 2, 2, 2, 2, 2, 2, 2, 2, 2, 2, 2, 2, 2, 2, 2, 2, 2, 2, 2, 2, 2, 2, 2, 2, 2, 2, 2, 2, 2, 2, 2, 2, 2, 2, 2, 2, 2, 2, 1, 1, 1, 1, 1, 1, 1, 1, 1, 1, 1, 1, 1, 1, 1, 1, 1, 1, 1, 1, 1, 1, 1, 1, 1, 1, 1, 1, 1, 1, 1, 1, 1, 1, 1, 1, 1, 1, 1, 1, 1, 1, 1, 1, 1, 1, 1, 1, 1, 1, 1, 1, 1, 1, 1, 1, 1, 1, 1, 1, 1, 1, 1, 1, 1, 1, 1, 1, 1, 1, 1, 1, 1, 1, 1, 1, 1, 1, 1, 1, 1, 1, 1, 1, 1, 1, 1, 1

Corresponding number of selected Gene ontology terms each gene is involved with:
(In the same order as above)
34, 33, 17, 22, 16, 16, 14, 13, 11, 14, 11, 11, 10, 10, 19, 11, 10, 9, 9, 9, 15, 9, 8, 8, 8, 13, 10, 9, 8, 8, 7, 7, 7, 7, 7, 7, 7, 16, 7, 7, 6, 6, 18, 6, 12, 6, 6, 6, 8, 8, 6, 7, 7, 7, 7, 6, 6, 6, 6, 6, 16, 8, 7, 7, 20, 15, 5, 5, 12, 5, 5, 5, 12, 5, 5, 5, 5, 5, 15, 8, 8, 8, 7, 14, 5, 5, 5, 5, 5, 5, 6, 9, 6, 10, 5, 5, 5, 5, 8, 4, 4, 4, 12, 6, 4, 6, 4, 9, 9, 5, 5, 9, 4, 4, 4, 4, 6, 14, 6, 7, 7, 6, 6, 6, 6, 5, 4, 5, 5, 4, 4, 5, 10, 5, 13, 3, 3, 4, 3, 18, 3, 3, 3, 3, 3, 3, 6, 3, 5, 7, 5, 7, 11, 3, 3, 3, 3, 3, 3, 3, 8, 8, 4, 4, 4, 5, 15, 3, 3, 3, 3, 10, 3, 15, 3, 4, 4, 4, 4, 4, 4, 4, 3, 9, 9, 3, 3, 5, 5, 5, 5, 2, 2, 2, 2, 3, 2, 2, 3, 2, 2, 2, 6, 2, 2, 2, 3, 2, 2, 4, 2, 4, 2, 2, 2, 2, 2, 3, 2, 6, 2, 2, 2, 2, 2, 2, 6, 2, 2, 2, 3, 2, 2, 2, 2, 2, 11, 11, 2, 2, 2, 11, 2, 2, 2, 2, 2, 2, 2, 2, 2, 3, 21, 7, 7, 7, 3, 4, 2, 2, 2, 10, 2, 5, 10, 4, 2, 2, 5, 2, 4, 6, 2, 2, 3, 9, 2, 2, 9, 2, 2, 3, 9, 2, 6, 2, 6, 2, 3, 18, 2, 2, 4, 2, 4, 2, 2, 8, 4, 16, 2, 2, 4, 5, 3, 3, 3, 3, 3, 3, 3, 5, 3, 3, 5, 2, 2, 2, 2, 2, 2, 14, 7, 2, 2, 13, 13, 13, 1, 1, 3, 1, 3, 1, 6, 6, 2, 1, 1, 1, 1, 12, 1, 6, 6, 6, 1, 2, 1, 1, 1, 6, 3, 1, 1, 1, 1, 1, 1, 4, 1, 1, 12, 1, 3, 6, 6, 6, 3, 4, 6, 6, 2, 1, 3, 1, 1, 11, 1, 1, 1, 1, 1, 1, 11, 1, 1, 1, 1, 1, 1, 1, 1, 1, 1, 1, 1, 1, 1, 1, 1, 1, 1, 10, 1, 1, 1, 5, 5, 2, 2, 2, 5, 2, 2, 1, 1, 1, 1, 2, 1, 1, 2, 2, 1, 1, 1, 3, 1, 1, 9, 1, 9, 1, 3, 1, 3, 1, 1, 3, 1, 3, 1, 1, 1, 1, 3, 3, 1, 1, 1, 3, 8, 2, 2, 2, 1, 8, 1, 2, 8, 8, 4, 2, 1, 4, 1, 2, 1, 4, 2, 4, 8, 1, 2, 4, 8, 2, 4, 2, 8, 4, 4, 8, 4, 1, 1, 4, 1, 4, 4, 4, 4, 1, 7, 1, 1, 1, 1, 1, 7, 7, 7, 1, 1, 7, 7, 1, 1, 7, 1, 1, 7, 1, 7, 1, 1, 2, 3, 6, 3, 3, 2, 1, 6, 1, 6, 3, 6, 1, 2, 2, 6, 3, 3, 1, 6, 3, 2, 1, 1, 1, 3, 3, 1, 1, 2, 3, 2, 2, 1, 3, 1, 6, 1, 3, 1, 1, 5, 5, 1, 5, 5, 1, 5, 5, 1, 5, 1, 5, 5, 1, 1, 1, 5, 5, 5, 1, 5, 5, 5, 1, 1, 1, 5, 5, 1, 5, 1, 1, 1, 2, 1, 4, 4, 2, 1, 4, 1, 1, 2, 2, 1, 4, 1, 1, 1, 1, 2, 2, 4, 2, 1, 4, 4, 4, 1, 4, 1, 1, 1, 1, 1, 1, 1, 1, 4, 1, 1, 4, 4, 4, 2, 1, 1, 2, 2, 2, 3, 3, 3, 1, 1, 1, 1, 1, 1, 3, 3, 1, 3, 1, 1, 3, 1, 1, 1, 3, 3, 1, 3, 3, 3, 3, 1, 3, 3, 3, 3, 3, 3, 3, 3, 3, 3, 1, 3, 3, 1, 3, 1, 1, 2, 2, 2, 2, 1, 1, 2, 2, 2, 1, 2, 2, 1, 1, 2, 2, 2, 2, 2, 1, 2, 2, 1, 1, 1, 1, 1, 2, 2, 2, 1, 2, 2, 2, 2, 1, 2, 1, 2, 1, 1, 1, 2, 1, 1, 2, 2, 2, 2, 1, 2, 2, 1, 1, 1, 1, 2, 2, 2, 1, 2, 2, 1, 2, 2, 2, 2, 1, 2, 2, 2, 2, 2, 1, 2, 2, 1, 2, 2, 2, 2, 1, 1, 1, 1, 1, 1, 1, 1, 1, 1, 1, 1, 1, 1, 1, 1, 1, 1, 1, 1, 1, 1, 1, 1, 1, 1, 1, 1, 1, 1, 1, 1, 1, 1, 1, 1, 1, 1, 1, 1, 1, 1, 1, 1, 1, 1, 1, 1, 1, 1, 1, 1, 1, 1, 1, 1, 1, 1, 1, 1, 1, 1, 1, 1, 1, 1, 1, 1, 1, 1, 1, 1, 1, 1, 1, 1, 1, 1, 1, 1, 1, 1, 1, 1, 1, 1, 1, 1, 1, 1, 1, 1, 1

Corresponding number of selected TFs each gene is involved with:
(In the same order as above)
11, 8, 12, 9, 12, 11, 12, 12, 12, 9, 11, 11, 12, 12, 6, 10, 11, 12, 12, 12, 7, 11, 12, 12, 12, 7, 9, 10, 11, 11, 12, 12, 12, 12, 12, 12, 12, 5, 11, 11, 12, 12, 4, 12, 6, 12, 12, 12, 9, 9, 12, 10, 10, 10, 10, 11, 11, 11, 11, 11, 4, 8, 9, 9, 3, 4, 12, 12, 5, 12, 12, 12, 5, 12, 12, 12, 12, 12, 4, 7, 7, 7, 8, 4, 11, 11, 11, 11, 11, 11, 9, 6, 9, 5, 10, 10, 10, 10, 6, 12, 12, 12, 4, 8, 12, 8, 12, 5, 5, 9, 9, 5, 11, 11, 11, 11, 7, 3, 7, 6, 6, 7, 7, 7, 7, 8, 10, 8, 8, 10, 10, 8, 4, 8, 3, 12, 12, 9, 12, 2, 12, 12, 12, 12, 12, 12, 6, 12, 7, 5, 7, 5, 3, 11, 11, 11, 11, 11, 11, 11, 4, 4, 8, 8, 8, 6, 2, 10, 10, 10, 10, 3, 10, 2, 10, 7, 7, 7, 7, 7, 7, 7, 9, 3, 3, 9, 9, 5, 5, 5, 5, 12, 12, 12, 12, 8, 12, 12, 8, 12, 12, 12, 4, 12, 12, 12, 8, 12, 12, 6, 12, 6, 12, 12, 12, 12, 12, 8, 12, 4, 12, 12, 12, 12, 12, 12, 4, 12, 12, 12, 8, 12, 12, 12, 12, 12, 2, 2, 11, 11, 11, 2, 11, 11, 11, 11, 11, 11, 11, 11, 11, 7, 1, 3, 3, 3, 7, 5, 10, 10, 10, 2, 10, 4, 2, 5, 10, 10, 4, 10, 5, 3, 9, 9, 6, 2, 9, 9, 2, 9, 9, 6, 2, 9, 3, 9, 3, 9, 6, 1, 8, 8, 4, 8, 4, 8, 8, 2, 4, 1, 8, 8, 4, 3, 5, 5, 5, 5, 5, 5, 3, 5, 5, 3, 7, 7, 7, 7, 7, 7, 1, 2, 7, 7, 1, 1, 1, 12, 12, 4, 12, 4, 12, 2, 2, 6, 12, 12, 12, 12, 1, 12, 2, 2, 2, 12, 6, 12, 12, 12, 2, 4, 12, 12, 12, 12, 12, 12, 3, 12, 12, 1, 12, 4, 2, 2, 2, 4, 3, 2, 2, 6, 12, 4, 12, 12, 1, 11, 11, 11, 11, 11, 11, 1, 11, 11, 11, 11, 11, 11, 11, 11, 11, 11, 11, 11, 11, 11, 10, 10, 10, 10, 1, 10, 10, 10, 2, 2, 5, 5, 5, 2, 5, 5, 10, 10, 10, 10, 5, 10, 10, 5, 5, 10, 9, 9, 3, 9, 9, 1, 9, 1, 9, 3, 9, 3, 9, 9, 3, 9, 3, 9, 9, 9, 9, 3, 3, 9, 9, 9, 3, 1, 4, 4, 4, 8, 1, 8, 4, 1, 1, 2, 4, 8, 2, 8, 4, 8, 2, 4, 2, 1, 8, 4, 2, 1, 4, 2, 4, 1, 2, 2, 1, 2, 8, 8, 2, 8, 2, 2, 2, 2, 7, 1, 7, 7, 7, 7, 7, 1, 1, 1, 7, 7, 1, 1, 7, 7, 1, 7, 7, 1, 7, 1, 6, 6, 3, 2, 1, 2, 2, 3, 6, 1, 6, 1, 2, 1, 6, 3, 3, 1, 2, 2, 6, 1, 2, 3, 6, 6, 6, 2, 2, 6, 6, 3, 2, 3, 3, 6, 2, 6, 1, 6, 2, 6, 5, 1, 1, 5, 1, 1, 5, 1, 1, 5, 1, 5, 1, 1, 5, 5, 5, 1, 1, 1, 5, 1, 1, 1, 5, 5, 5, 1, 1, 5, 1, 5, 5, 4, 2, 4, 1, 1, 2, 4, 1, 4, 4, 2, 2, 4, 1, 4, 4, 4, 4, 2, 2, 1, 2, 4, 1, 1, 1, 4, 1, 4, 4, 4, 4, 4, 4, 4, 4, 1, 4, 4, 1, 1, 1, 2, 4, 4, 2, 2, 2, 1, 1, 1, 3, 3, 3, 3, 3, 3, 1, 1, 3, 1, 3, 3, 1, 3, 3, 3, 1, 1, 3, 1, 1, 1, 1, 3, 1, 1, 1, 1, 1, 1, 1, 1, 1, 1, 3, 1, 1, 3, 1, 3, 2, 1, 1, 1, 1, 2, 2, 1, 1, 1, 2, 1, 1, 2, 2, 1, 1, 1, 1, 1, 2, 1, 1, 2, 2, 2, 2, 2, 1, 1, 1, 2, 1, 1, 1, 1, 2, 1, 2, 1, 2, 2, 2, 1, 2, 2, 1, 1, 1, 1, 2, 1, 1, 2, 2, 2, 2, 1, 1, 1, 2, 1, 1, 2, 1, 1, 1, 1, 2, 1, 1, 1, 1, 1, 2, 1, 1, 2, 1, 1, 1, 1, 2, 1, 1, 1, 1, 1, 1, 1, 1, 1, 1, 1, 1, 1, 1, 1, 1, 1, 1, 1, 1, 1, 1, 1, 1, 1, 1, 1, 1, 1, 1, 1, 1, 1, 1, 1, 1, 1, 1, 1, 1, 1, 1, 1, 1, 1, 1, 1, 1, 1, 1, 1, 1, 1, 1, 1, 1, 1, 1, 1, 1, 1, 1, 1, 1, 1, 1, 1, 1, 1, 1, 1, 1, 1, 1, 1, 1, 1, 1, 1, 1, 1, 1, 1, 1, 1, 1, 1, 1, 1, 1, 1, 1

Gene ontology terms ranked according to mean of effect sizes in cluster:
neural tube formation | provirus | neuron development | cell fate specification | kidney development | gut development | regulation of cell proliferation | transcription factor complex | cartilage development | apoptosis | cell proliferation | luciferin monooxygenase activity | osteoblast differentiation | mesoderm formation | developmental process | lung development | odontogenesis | myoblast differentiation | chondrocyte differentiation | heart development | decidualization | transdifferentiation | N-acetyltransferase activity | gliogenesis | liver development | stem cell differentiation | neural tube closure | system development | ossification | eye development | camera-type eye development | compound eye development | fibroblast growth factor receptor binding | skeletal muscle development | fibroblast growth factor receptor activity | cell-cell signaling | skeletal development | induction | lens development in camera-type eye | epithelial to mesenchymal transition | growth | cell | response to osmotic stress | neuron migration

Corresponding mean of effect sizes of each Gene ontology term:
(In the same order as above)
1.84, 1.78, 1.55, 1.49, 1.46, 1.45, 1.42, 1.33, 1.31, 1.31, 1.30, 1.28, 1.24, 1.22, 1.16, 1.16, 1.13, 1.12, 1.10, 1.01, 0.96, 0.88, 0.84, 0.83, 0.81, 0.76, 0.75, 0.73, 0.70, 0.67, 0.67, 0.66, 0.62, 0.62, 0.58, 0.58, 0.57, 0.49, 0.45, 0.41, 0.37, 0.23, 0.21, -0.13

Corresponding total number of genes of each Gene ontology term:
(In the same order as above)
79, 135, 69, 74, 139, 54, 165, 117, 199, 1.9K, 1.5K, 289, 239, 66, 26, 159, 122, 112, 135, 144, 117, 204, 19, 65, 100, 93, 75, 21, 283, 140, 140, 141, 182, 64, 193, 26, 133, 2.1K, 80, 229, 2.2K, 4.1K, 46, 187

Corresponding number of genes of each Gene ontology term with at least one TFBS (of selected TFs):
(In the same order as above)
25, 41, 21, 19, 29, 9, 49, 41, 40, 360, 288, 73, 61, 19, 6, 34, 24, 31, 31, 28, 21, 43, 3, 13, 21, 17, 21, 6, 50, 25, 25, 25, 47, 13, 49, 7, 21, 364, 15, 53, 372, 637, 12, 22

Corresponding total number of TFBS (of selected TFs) for each Gene ontology term (in all genes):
(In the same order as above)
170, 241, 136, 147, 175, 81, 270, 212, 240, 2033, 1624, 359, 320, 101, 52, 201, 158, 177, 176, 198, 134, 245, 24, 88, 112, 115, 103, 41, 253, 151, 151, 151, 229, 85, 223, 40, 142, 1954, 93, 272, 1984, 3486, 50, 167

Corresponding number of selected TFs each Gene ontology term is involved with:
(In the same order as above)
12, 12, 12, 12, 12, 12, 12, 12, 12, 12, 12, 12, 12, 12, 12, 12, 12, 12, 12, 12, 12, 12, 12, 12, 12, 12, 12, 12, 12, 12, 12, 12, 12, 12, 12, 12, 12, 12, 12, 12, 12, 12, 12, 12

TFs ranked according to mean of effect sizes in cluster:
V$PAX2_02, V$TST1_01, V$HMGIY_Q6, V$STAT1_03, V$NFAT_Q6, V$STAT6_01, V$STAT5A_03, V$NFAT_Q4_01, V$HOXA4_Q2, V$STAT4_01, V$STAT5A_04, V$CEBP_Q3

Corresponding mean of effect sizes of each TF:
(In the same order as above)
1.27, 1.16, 1.15, 1.03, 1.02, 1.02, 0.99, 0.83, 0.79, 0.72, 0.61, 0.58

Corresponding total number of TFBS for each TF (genome-wide):
(In the same order as above)
741, 730, 753, 751, 719, 732, 749, 736, 719, 743, 740, 744

Corresponding total number of TFBS for each TF (in all genes in selected Gene ontology terms):
(In the same order as above)
1549, 1452, 1488, 1424, 1524, 1459, 1462, 1444, 1361, 1440, 1322, 1439

Corresponding number of genes (of selected Gene ontology terms) each TF is involved with:
(In the same order as above)
392, 378, 387, 386, 390, 387, 399, 392, 370, 391, 380, 374

Corresponding number of selected Gene ontology terms each TF is involved with:
(In the same order as above)
44, 44, 44, 44, 44, 44, 44, 44, 44, 44, 44, 44

Cluster for columns 856 to 873, rows 251 to 258

Gene ontology terms

response to hypoxia | activation of protein kinase C activity | protein kinase C activity | soluble NSF attachment protein activity | arginase activity | nitric oxide biosynthetic process | D-nopaline dehydrogenase activity | nitric-oxide synthase activity | nitric-oxide synthase regulator activity | nascent polypeptide-associated complex | response to reactive oxygen species | heme catabolic process | superoxide release | myosin light chain kinase activity | stress fiber formation | smooth muscle contraction | regulation of vascular smooth muscle contraction | vascular smooth muscle contraction

TFs

V$AP1_Q6_01 | V$AP1_Q4_01 | V$AP1_Q6 | V$AP1_C | V$AP1_01 | V$AP1_Q2 | V$AP1FJ_Q2 | V$AP1_Q4

Information

All related TFs:
(List of all TFs that are related to any of the PWMs)
AP-1, FosB, Fra-1, Fra-2, JunB, JunB:Fra-1, JunB:Fra-2, JunD, JunD:Fra-2, JunD:deltaFosB, YAP1, c-Fos, c-Jun, c-Jun:FosB, c-Jun:JunD, c-Jun:c-Fos, deltaFosB

Ranked gene list:
(All genes of the selected Gene ontology terms with hits of any of the selected TFs, ranked according to the total number of TFBS)
TNF, MTHFR, AKT1, ISYNA1, CALM1, CDKN1A, NOS2, HMOX1, DDAH2, RHOA, ILK, VASP, PKM2, NLRP3, VEGFA, CALM3, PTK2B, MARK2, SSSCA1, LMNA, JUNB, DGKZ, ARHGEF2, ADM, SLC2A1, RELA, TNFRSF1A, NQO1, CD44, DGKA, FBRS, VCL, CSF2, ZYX, VDR, SQSTM1, SPSB2, SLC2A4RG, SIAH2, SHC1, SFN, S100A6, RHOC, RALGDS, PXN, PRKCD, PFN1, NFKBIA, LTB, IRF1, HMOX2, HGS, H2AFX, GAPDH, FHOD1, EFNA3, EDN2, DKFZp779C0757, DDR1, CNTNAP1, BHLHB2, BAG3, BACH1, ARHGEF1, ACY1, MYL6, LDHA, LCK, CSF1, BUD31, SOD1, PSMD9, PPP1R14A, PLCG1, PKN1, PELP1, NUP43, IL10, HSPA9, FLJ32987, CNP, ZC3H12D, UBC, SPINT1, S1PR2, RPS6KA1, RND1, NDRG1, MAD2L1BP, KSR1, IL2RB, GCLC, FOS, EGLN2, CNN1, STX1A, SNAPC2, RGS3, PRAP1, MPRIP, MAPK3, MAP2K1, JAG2, HSPB6, EGR1, DCTN3, CASP9, AMT, VAMP8, SEC61A1, PICK1, PER1, NFE2L2, NACC1, MAPK13, JUND, COL11A2, BCL2L1, BAX, ADA, UROD, SDC4, RAB5A, PTPN1, PRX, PLAU, PLA2G6, PHB2, MXI1, MAPKAPK2, KLF6, KCNJ11, ITGA3, GSTP1, GSR, GLTSCR2, FASN, CYBA, CLCN3, CA9, ADRBK1, TIPARP, TINAGL1, STRA13, SND1, SLC35A2, S1PR1, RND2, RGS2, PRKAB1, PPP1R15A, PAOX, OGG1, OBSCN, MRLC3, MAPK12, MAPK11, MAP3K1, LBR, IVL, HIF1AN, FECH, DYNC1H1, DDIT4, ARF1, ADRA1A

Corresponding total number of TFBS:
(For each gene listed above, the total number of TFBS for any of the selected TFs, multiplied by the

number of selected Gene ontology terms containing that gene)
96, 91, 91, 78, 70, 48, 42, 30, 30, 28, 25, 24, 24, 24, 21, 20, 18, 18, 16, 16, 16, 16, 16, 16, 15, 15, 14, 14, 14, 12, 10, 9, 9, 8, 8, 8, 8, 8, 8, 8, 8, 8, 8, 8, 8, 8, 8, 8, 8, 8, 8, 8, 8, 8, 8, 8, 8, 8, 8, 8, 8, 8, 8, 8, 8, 8, 7, 7, 7, 7, 7, 6, 6, 6, 6, 6, 6, 6, 6, 6, 6, 6, 5, 5, 5, 5, 5, 5, 5, 5, 5, 5, 5, 5, 5, 5, 4, 4, 4, 4, 4, 4, 4, 4, 4, 4, 4, 4, 4, 3, 3, 3, 3, 3, 3, 3, 3, 3, 3, 3, 3, 2, 2, 2, 2, 2, 2, 2, 2, 2, 2, 2, 2, 2, 2, 2, 2, 2, 2, 2, 2, 1, 1, 1, 1, 1, 1, 1, 1, 1, 1, 1, 1, 1, 1, 1, 1, 1, 1, 1, 1, 1, 1, 1, 1

Corresponding number of selected Gene ontology terms each gene is involved with:
(In the same order as above)
12, 13, 13, 13, 10, 6, 14, 6, 5, 7, 5, 3, 3, 3, 7, 10, 6, 3, 2, 2, 2, 2, 2, 2, 3, 3, 2, 7, 2, 2, 2, 3, 3, 1, 1, 1, 1, 1, 1, 1, 1, 1, 1, 1, 4, 2, 1, 2, 1, 1, 4, 1, 2, 1, 1, 1, 2, 1, 1, 1, 1, 1, 1, 1, 1, 1, 1, 1, 1, 1, 2, 2, 6, 1, 2, 2, 2, 6, 2, 1, 2, 1, 1, 1, 1, 1, 1, 1, 1, 1, 1, 1, 5, 1, 1, 1, 1, 1, 1, 1, 2, 1, 1, 2, 2, 1, 2, 1, 1, 1, 3, 3, 3, 1, 1, 1, 3, 1, 3, 1, 1, 2, 1, 1, 2, 2, 2, 1, 1, 1, 1, 1, 1, 1, 2, 1, 2, 2, 1, 1, 1, 1, 1, 1, 1, 1, 1, 1, 1, 1, 1, 1, 1, 1, 1, 1, 1, 1, 1, 1, 1, 1, 1, 1

Corresponding number of selected TFs each gene is involved with:
(In the same order as above)
8, 7, 7, 6, 7, 8, 3, 5, 6, 4, 5, 8, 8, 8, 3, 2, 3, 6, 8, 8, 8, 8, 8, 8, 5, 5, 7, 2, 7, 6, 5, 3, 3, 8, 8, 8, 8, 8, 8, 8, 8, 8, 8, 8, 2, 4, 8, 4, 8, 8, 2, 8, 4, 8, 8, 8, 4, 8, 8, 8, 8, 8, 8, 8, 8, 7, 7, 7, 7, 7, 3, 3, 1, 6, 3, 3, 3, 1, 3, 6, 3, 5, 5, 5, 5, 5, 5, 5, 5, 5, 5, 5, 1, 5, 5, 4, 4, 4, 4, 4, 2, 4, 4, 2, 2, 4, 2, 4, 3, 3, 1, 1, 1, 3, 3, 3, 1, 3, 1, 3, 2, 1, 2, 2, 1, 1, 1, 2, 2, 2, 2, 2, 2, 2, 1, 2, 1, 1, 2, 2, 2, 1, 1, 1, 1, 1, 1, 1, 1, 1, 1, 1, 1, 1, 1, 1, 1, 1, 1, 1, 1, 1, 1

Gene ontology terms ranked according to mean of effect sizes in cluster:
stress fiber formation | nascent polypeptide-associated complex | protein kinase C activity | regulation of vascular smooth muscle contraction | myosin light chain kinase activity | nitric oxide biosynthetic process | response to hypoxia | vascular smooth muscle contraction | nitric-oxide synthase regulator activity | smooth muscle contraction | activation of protein kinase C activity | response to reactive oxygen species | heme catabolic process | soluble NSF attachment protein activity | nitric-oxide synthase activity | arginase activity | D-nopaline dehydrogenase activity | superoxide release

Corresponding mean of effect sizes of each Gene ontology term:
(In the same order as above)
1.95, 1.70, 1.55, 1.47, 1.44, 1.35, 1.27, 1.25, 1.24, 1.21, 1.13, 1.06, 1.05, 0.88, 0.87, 0.76, 0.75, 0.47

Corresponding total number of genes of each Gene ontology term:
(In the same order as above)
165, 136, 157, 51, 130, 101, 124, 27, 43, 105, 302, 115, 57, 119, 56, 44, 77, 48

Corresponding number of genes of each Gene ontology term with at least one TFBS (of selected TFs):
(In the same order as above)
34, 33, 29, 10, 24, 13, 32, 8, 10, 15, 54, 26, 14, 23, 11, 7, 14, 6

Corresponding total number of TFBS (of selected TFs) for each Gene ontology term (in all genes):
(In the same order as above)
170, 122, 140, 47, 117, 74, 123, 32, 49, 62, 229, 89, 49, 95, 50, 32, 63, 25

Corresponding number of selected TFs each Gene ontology term is involved with:
(In the same order as above)
8, 8, 8, 8, 8, 8, 8, 8, 8, 8, 8, 8, 8, 8, 8, 8, 8, 8

TFs ranked according to mean of effect sizes in cluster:
V$AP1_Q4, V$AP1_Q6_01, V$AP1_Q2, V$AP1FJ_Q2, V$AP1_01, V$AP1_Q4_01, V$AP1_Q6, V$AP1_C

Corresponding mean of effect sizes of each TF:
(In the same order as above)

1.47, 1.35, 1.33, 1.30, 1.24, 1.17, 0.85, 0.81

Corresponding total number of TFBS for each TF (genome-wide):
(In the same order as above)
712, 726, 692, 710, 721, 719, 721, 720

Corresponding total number of TFBS for each TF (in all genes in selected Gene ontology terms):
(In the same order as above)
213, 212, 203, 196, 193, 196, 181, 174

Corresponding number of genes (of selected Gene ontology terms) each TF is involved with:
(In the same order as above)
87, 91, 82, 82, 92, 89, 82, 84

Corresponding number of selected Gene ontology terms each TF is involved with:
(In the same order as above)
18, 18, 18, 18, 18, 18, 18, 18

Cluster for columns 17 to 56, rows 322 to 322

Gene ontology terms

small nuclear ribonucleoprotein complex | spliceosome | spliceosome assembly | mRNA processing | heterogeneous nuclear ribonucleoprotein complex | RNA binding | ribonucleoprotein complex | nuclear transport | RNA metabolic process | helicase activity | spindle | spindle assembly | kinetochore | anaphase | chromosome segregation | sister chromatid cohesion | telophase | meiosis | prophase | origin recognition complex | cell cycle arrest | cell cycle | regulation of cell cycle | cell division | mitosis | interphase | metaphase | cell cycle checkpoint | cellular response to DNA damage stimulus | DNA damage checkpoint | response to DNA damage stimulus | nuclear matrix | chromatin assembly | nucleosome assembly | heterochromatin | chromatin remodeling | nucleosome | spermatid development | macronucleus | male pronucleus

TFs

V$NKX3A_01

Information

All related TFs:
(List of all TFs that are related to any of the PWMs)
Nkx3-1

Ranked gene list:
(All genes of the selected Gene ontology terms with hits of any of the selected TFs, ranked according to the total number of TFBS)
HIST1H4I, HIST1H4C, HIST2H2AA3, CDC2, PTTG1, CHEK2, CHEK1, BUB3, CENPC1, CENPE, CBX5, HNRPDL, CDK2, AURKA, MDM2, MCM2, TOP1, SKP1, SEPT7, DDX5, CDC27, TPX2, SMC4, SMARCA5, MAPRE2, HIST1H3E, HIST1H3C, H2AFZ, FBXW7, DBF4, CTNNB1, BCL2, SMARCA2, SKP2, SFRS11, RACGAP1, HIST1H1T, H3F3B, ERBB2IP, CLIP1, CKS1B, ATM, TP73, SLU7, PAFAH1B1, NFYA, LIG4, HNRNPA1, UBE2B, SSB, SNRPN, ORC1L, NPAT, NET1, HIST2H3C, UBE2K, TP63, STAG2, RUNX2, RUNX1, RPS6KA3, RAF1, PRPF4B, PIK3CA, HNRNPH1, FILIP1L, ETS1, CHD1, CDKN2C, CDK6, BMI1, BCL6, ANAPC10, TLK1, STRN3, SMC6, PIK3C3, NFKB1, NEK1, NEDD9, LSM4, LATS1, IL2, HNRNPU, HNRNPD, ENOPH1,

CAMK4, TXNIP, TGFBR2, SMAD2, SAMD4A, REL, PTPRC, PRH1, PIK3R1, NOVA1, ING3, IKZF3, ID2, HNRNPR, HMGB2, HIF1A, HBP1, ELAVL4, DUT, DCLRE1A, CREM, CD69, CD44, BTG1, AHR, ZNF638, XRCC4, VCPIP1, UBE3A, TNFSF10, SUPT3H, SP3, SNRPG, SHC1, SERTAD2, PXMP3, PURA, PSMC2, PPP2CA, ORC4L, NIPBL, NFYC, MLL5, MBNL1, IFNG, HNRNPH3, HIST2H3A, FYN, FOXG1, EIF2S1, EHMT2, EFTUD2, DICER1, DHX15, DCP2, COLEC12, CHORDC1, CEP57, BIRC2, ARID4A, ADNP, ZWINT, ZNRD1, ZNF238, ZFX, ZFP36L2, YTHDC1, XPO7, TGFB2, TES, STK38L, SRPK2, SLC38A2, SILV, SETDB2, SERBP1, RREB1, RPL15, RBM45, PRKCH, PRKAR1A, PRDM2, PRDM1, POLR2B, PHIP, PHC3, PDE3A, PCF11, PAWR, NT5C3, NF1, NEUROG2, NDUFB6, NAT13, NARG1, MAPKSP1, MAP3K4, LSM6, KPNA4, KLRK1, KIAA0419, ITPR1, HLTF, HIST1H3H, HIST1H3G, HIST1H2BF, HIST1H2BB, HIST1H1D, HIST1H1B, HIPK3, HDAC7, GSK3B, GOLGA1, GBP2, GABPA, FIP1L1, ETV1, ENO1, EID1, EGR2, EED, DYRK1A, DNAJB9, DMTF1, DHX32, CYP51A1, CSTF3, CSTF1, CLDN1, CLCN3, CIT, CHD2, CEP170, CBFB, CA2, BTD, BCL9, ATP6V1A, ATF7IP, ARSG, ARID2, ANAPC4, AFF1, ACO1, AATF, AASDHPPT

Corresponding total number of TFBS:
(For each gene listed above, the total number of TFBS for any of the selected TFs, multiplied by the number of selected Gene ontology terms containing that gene)
29, 29, 26, 24, 18, 18, 18, 18, 17, 16, 16, 15, 15, 15, 13, 12, 11, 11, 11, 10, 10, 9, 9, 9, 9, 9, 9, 9, 9, 9, 9, 9, 8, 8, 8, 8, 8, 8, 8, 8, 8, 8, 7, 7, 7, 7, 7, 7, 6, 6, 6, 6, 6, 6, 5, 5, 5, 5, 5, 5, 5, 5, 5, 5, 5, 5, 5, 5, 5, 5, 5, 5, 4, 4, 4, 4, 4, 4, 4, 4, 4, 4, 4, 4, 4, 4, 3, 3, 3, 3, 3, 3, 3, 3, 3, 3, 3, 3, 3, 3, 3, 3, 3, 3, 3, 3, 3, 3, 3, 2, 2, 2, 2, 2, 2, 2, 2, 2, 2, 2, 2, 2, 2, 2, 2, 2, 2, 2, 2, 2, 2, 2, 2, 2, 2, 2, 2, 2, 2, 2, 1, 1, 1, 1, 1, 1, 1, 1, 1, 1, 1, 1, 1, 1, 1, 1, 1, 1, 1, 1, 1, 1, 1, 1, 1, 1, 1, 1, 1, 1, 1, 1, 1, 1, 1, 1, 1, 1, 1, 1, 1, 1, 1, 1, 1, 1, 1, 1, 1, 1, 1, 1, 1, 1, 1, 1, 1, 1, 1, 1, 1, 1, 1, 1, 1, 1, 1, 1, 1, 1, 1, 1, 1, 1, 1, 1, 1, 1, 1, 1, 1, 1, 1

Corresponding number of selected Gene ontology terms each gene is involved with:
(In the same order as above)
29, 29, 26, 24, 18, 18, 18, 18, 17, 16, 16, 15, 15, 15, 13, 12, 11, 11, 11, 10, 10, 9, 9, 9, 9, 9, 9, 9, 9, 9, 9, 9, 8, 8, 8, 8, 8, 8, 8, 8, 8, 8, 7, 7, 7, 7, 7, 7, 6, 6, 6, 6, 6, 6, 5, 5, 5, 5, 5, 5, 5, 5, 5, 5, 5, 5, 5, 5, 5, 5, 5, 5, 4, 4, 4, 4, 4, 4, 4, 4, 4, 4, 4, 4, 4, 4, 3, 3, 3, 3, 3, 3, 3, 3, 3, 3, 3, 3, 3, 3, 3, 3, 3, 3, 3, 3, 3, 3, 3, 2, 2, 2, 2, 2, 2, 2, 2, 2, 2, 2, 2, 2, 2, 2, 2, 2, 2, 2, 2, 2, 2, 2, 2, 2, 2, 2, 2, 2, 2, 2, 1, 1, 1, 1, 1, 1, 1, 1, 1, 1, 1, 1, 1, 1, 1, 1, 1, 1, 1, 1, 1, 1, 1, 1, 1, 1, 1, 1, 1, 1, 1, 1, 1, 1, 1, 1, 1, 1, 1, 1, 1, 1, 1, 1, 1, 1, 1, 1, 1, 1, 1, 1, 1, 1, 1, 1, 1, 1, 1, 1, 1, 1, 1, 1, 1, 1, 1, 1, 1, 1, 1, 1, 1, 1, 1, 1, 1, 1, 1, 1, 1, 1, 1

Corresponding number of selected TFs each gene is involved with:
(In the same order as above)
1, 1

Gene ontology terms ranked according to mean of effect sizes in cluster:
chromatin assembly | anaphase | heterochromatin | nucleosome assembly | metaphase | kinetochore | mRNA processing | chromosome segregation | small nuclear ribonucleoprotein complex | regulation of cell cycle | mitosis | origin recognition complex | cellular response to DNA damage stimulus | macronucleus | cell cycle checkpoint | prophase | interphase | telophase | male pronucleus | nucleosome | sister chromatid cohesion | spindle | nuclear transport | spermatid development | cell cycle | nuclear matrix | meiosis | chromatin remodeling | heterogeneous nuclear ribonucleoprotein complex | spliceosome | RNA binding | cell division | helicase activity | spindle assembly | RNA metabolic process | cell cycle arrest | DNA damage checkpoint | ribonucleoprotein complex | response to DNA damage stimulus | spliceosome assembly

Corresponding mean of effect sizes of each Gene ontology term:
(In the same order as above)
5.14, 4.86, 4.61, 4.52, 4.39, 4.10, 4.00, 3.98, 3.90, 3.52, 3.50, 3.37, 3.12, 3.05, 2.82, 2.79, 2.78, 2.77, 2.36,

2.30, 2.24, 2.23, 2.16, 2.09, 1.88, 1.75, 1.63, 1.48, 1.44, 1.34, 1.33, 1.31, 1.24, 1.24, 1.20, 1.07, 0.88, 0.29, 0.15, 0.09

Corresponding total number of genes of each Gene ontology term:
(In the same order as above)
101, 263, 196, 83, 295, 151, 109, 278, 197, 648, 635, 64, 111, 31, 171, 212, 382, 128, 21, 190, 93, 302, 185, 119, 1.3K, 197, 369, 410, 240, 119, 448, 451, 90, 161, 98, 628, 168, 339, 303, 73

Corresponding number of genes of each Gene ontology term with at least one TFBS (of selected TFs):
(In the same order as above)
21, 32, 29, 16, 36, 20, 14, 31, 23, 72, 61, 11, 15, 6, 21, 21, 37, 15, 5, 21, 10, 22, 17, 10, 102, 20, 26, 38, 21, 10, 31, 38, 7, 12, 7, 54, 13, 20, 21, 4

Corresponding total number of TFBS (of selected TFs) for each Gene ontology term (in all genes):
(In the same order as above)
21, 32, 29, 16, 36, 20, 14, 31, 23, 72, 61, 11, 15, 6, 21, 21, 37, 15, 5, 21, 10, 22, 17, 10, 102, 20, 26, 38, 21, 10, 31, 38, 7, 12, 7, 54, 13, 20, 21, 4

Corresponding number of selected TFs each Gene ontology term is involved with:
(In the same order as above)
1, 1, 1, 1, 1, 1, 1, 1, 1, 1, 1, 1, 1, 1, 1, 1, 1, 1, 1, 1, 1, 1, 1, 1, 1, 1, 1, 1, 1, 1, 1, 1, 1, 1, 1, 1, 1, 1, 1, 1

TFs ranked according to mean of effect sizes in cluster:
V$NKX3A_01

Corresponding mean of effect sizes of each TF:
(In the same order as above)
2.47

Corresponding total number of TFBS for each TF (genome-wide):
(In the same order as above)
674

Corresponding total number of TFBS for each TF (in all genes in selected Gene ontology terms):
(In the same order as above)
990

Corresponding number of genes (of selected Gene ontology terms) each TF is involved with:
(In the same order as above)
232

Corresponding number of selected Gene ontology terms each TF is involved with:
(In the same order as above)
40

Cluster for columns 1294 to 1295, rows 154 to 170

Gene ontology terms

signal transduction | Notch signaling pathway

TFs

V$SMAD_Q6 | V$AP2REP_01 | V$HNF4_Q6_03 | V$HNF4_Q6_02 | V$T3R_Q6 | V$PAX8_B | V$PAX8_01 | V$USF2_Q6 | V$ZIC2_01 | V$TTF1_Q6 | V$VDR_Q6 | V$LRF_Q2 | V$LBP1_Q6 | V$AP4_Q6_01 | V$AP4_Q6 | V$AP4_Q5 | V$LFA1_Q6

Information

All related TFs:
(List of all TFs that are related to any of the PWMs)
AP-2rep, AP-4, FBI-1, HNF-4, HNF-4alpha, HNF-4alpha1, HNF-4alpha2, HNF-4alpha3, HNF-4alpha4, LBP-1, LF-A1, LRF, Nkx2-1, OCZF, Pax-8, RAR-alpha, RAR-alpha1, RAR-alpha:RXR-alpha, RAR-alpha:RXR-gamma, RAR-beta, RAR-beta2, RAR-gamma, RXR-alpha, RXR-beta, RXR-beta2, RXR-gamma, Smad1, Smad1.1, Smad2, Smad2-L, Smad3, Smad3:Smad4, Smad4, T3R-alpha, T3R-alpha1, T3R-alpha2, T3R-beta, T3R-beta1, T3R-beta2, USF1:USF2, USF2, USF2a, VDR, ZIC2, Zic2

Ranked gene list:
(All genes of the selected Gene ontology terms with hits of any of the selected TFs, ranked according to the total number of TFBS)
LFNG, HES5, NRARP, NOTCH1, MFNG, TLE3, JAG2, HES7, HES3, DLL1, LHX1, NKX2-2, VEGFA, PTBP1, DLL4, C21orf33, ZFPM1, ZFP36, WNT10B, WNT1, VEGFB, TUSC2, TSPAN4, TSPAN32, TNK2, TNFRSF6B, TNFRSF4, TNFRSF18, TNFRSF12A, TNF, TCF7, TBC1D10C, STRA13, SSSCA1, SLC2A4RG, SKI, SIPA1, SH3BP2, SH3BP1, SH2B3, SEMA3B, SCRIB, SBF1, RPS6KB2, RORC, RGS19, RASSF2, RASGRP2, RARG, RARA, RAC3, PTPN7, PTPN6, PNPLA2, PLXNA1, PLCB3, PFN1, PER1, PDGFB, OVOL1, OPRL1, NR4A1, NPHP4, NPDC1, NFATC1, MGC111011, MEF2D, MDK, MAZ, MAPKAPK3, MAP3K12, MAP3K11, MAP2K3, MAF1, LTBP4, LTB, LSP1, KREMEN2, JUNB, ISYNA1, HRAS, GPX4, GNAI2, GIT1, GFI1, GATA3, FLNA, FLJ32987, FLII, FGFRL1, FGF8, FBRS, FASN, EFNA4, EFNA3, DUSP7, DUSP2, DGKZ, DGAT1, CXXC5, CSK, CISH, CFL1, CDK5, CD151, CBX4, BCL9L, ANAPC2, AKT1, AHNAK, ADORA2A, ABHD14B, TRADD, TAS1R3, TAGLN2, SMAD7, SIGIRR, S100A4, RIN1, PRKCD, PLK3, NFKBIE, MXD4, MCAM, KISS1R, IRF1, HDAC7, GATA2, DVL1, CNTNAP1, CLCF1, C19orf26, BCL11B, ARHGEF2, AMH, ACTB, WBP7, UBE2I, RXRB, RASSF1, RALGDS, PPP1R9B, PPP1R1B, PLCG1, P2RY11, MC1R, MAPK12, MAPK11, IL17C, HS6ST1, GUK1, GRK6, GNAS, EHD1, E2F4, CTBP1, BBC3, APC2, ADRBK1, TLR9, SOCS3, PTP4A3, NXPH4, LLGL2, HOXA10, FOXC1, CCND2, BCL6, ADM, WNT10A, SYNGAP1, SOCS1, SLC9A3R1, SLC12A7, SBNO2, LTA, GPS2, EMD, EGR1, CAMK2G, p63RhoGEF, TBX2, STMN3, SQSTM1, RGS14, PYGO2, PSD, PAX6, PARD6A, OSM, GAPDH, EFNA2, EFNA1, DVL2, CCND1, ARVCF, ZAP70, S1PR4, RING1, RHOC, NFKB2, LBX1, HOXA11, FLI1, CYBA, ARHGEF1, WNT11, VAMP2, RFNG, PTK6, PSENEN, POU2F2, GLI1, FHOD1, FGFR3, DGKQ, DGKA, CDKN1C, BHLHB2, BCL2L11, ATP2A3, AGAP2, SH2B2, RPS6KA1, PDE4A, MXD3, MRPL28, LAG3, ITGB2, FGF22, CDK4, CDC34, AQP3, AKT1S1, AGER, TNFRSF25, NXPH3, NR4A2, MAP2K7, LEF1, LASP1, GARNL4, FBXL15, CORO1A, CDK9, ARHGDIA, ZYX, UCN2, TENC1, RPS6KA4, PTGDS, PKN1, NR3C2, MAL, LCK, IKBKE, FOXO3, FGF9, ESRRA, DLX2, DLG4, DCTN3, CRTC2, COL11A2, CD82, CALY, CALR, BMF, BAD, ARRB2, ADCY7, TRAF4, TNIP1, TGFB1, PDCD1, MAP3K14, LTBP3, JUND, ITGA5, IRS1, INPP5D, IL11RA, ID3, HES1, EOMES, DHX58, DBNL, CRLF1, CDC20, ZIC2, SLC9A3R2, S100A3, RAD9A, NR4A3, NKD2, NDOR1, MXI1, KLF2, HCST, GPSM1, GPR132, GGNBP2, ENTPD2, DDR1, DDIT3, CDKN2C, CD248, ASB2, AGRN, VIPR1, VGF, VASP, TSC2, TRIP10, TNFRSF1B, TNFRSF14, TM7SF2, TLX2, STAT5A, SEMA3G, RUNX3, PTCH1, PRKACA, NPPC, NFKBID, NAB2, MGC4238, LEFTY1, IFITM1, ID2, HOXB4, HCRT, GSK3A, FOSB, FERMT3, EPHB3, DUSP1, CDK6, CD79B, CCR7, BRD2, BAK1, ARF1, ANP32A, UCP2, TRIM28, TPP1, TNFRSF13C, SLC12A4, RGL2, PTH1R, PMEPA1, PHB2, PAX5, NPW, NEUROG2, MTA2, MST1, MAPK15, MAPK13, LPAR2, LAT, LAMA5, KLF6, ITPR3, IL11, ICAM4, GPI, GPC1, FSTL3, ERF, ELMO3, E2F2, DHCR7, CTF1, CMIP, CDK5R1, CDC25B, CD81, CD7, WBP2, TYROBP, TNFSF13, TNFRSF1A, SNX20, SMAD2, SLC29A1, SLC17A7, SAMD4A, RRAS, RGS11, RGS10, RAP1GAP, PRMT1, PIN1, PIK3R2, PDLIM7, PDCD6, PCNA, NR3C1, NOTCH4, NOL3, NISCH, NFATC3, MUC1, MTA1, MICAL1, MGRN1,

LTB4R2, LNX2, LIMD1, KIAA0319L, KCNH4, ISG15, IRF7, INS, ING1, IDUA, HGFAC, HES6, GALR3, FOXJ1, FLOT2, DLX4, DAAM1, CXCR4, CNP, CERK, CDKN1B, CDC37, CD79A, CD4, CARD9, CAMK1D, BID, AXIN1, ARC, ADCY4, ZHX2, ZFP36L1, ZBTB16, WNT7B, WNT7A, WNT4, VAV1, TRRAP, TRIM3, TRERF1, TRAP1, TRAF2, TGM1, TGIF2, TFDP1, TFAP2A, TCF3, TAOK3, STMN1, STK25, STAT6, STARD3, SREBF1, SRA1, SPHK1, SORL1, SORBS3, SNF1LK, SKP1, SHC1, SH3RF3, S100A13, ROBLD3, RHOG, RAD23A, PVRL1, PTPRA, PTGER1, PTCHD2, PSMB9, PRRG2, PRKCB, PPP3CA, PPARA, PLEKHO2, NUDT1, NRGN, NR2F6, NR2F1, NPM1, NOG, NKX3-2, NGFR, NDRG1, MYD88, MT2A, MIF, MARCKSL1, MAP4K2, MAP3K6, MAP2K2, MAD1L1, LMNA, LIF, LGALS1, KLF10, KCNA2, IRF5, IL32, IL21R, IL10RA, IKZF1, IGF2, IGF1R, HOXC6, HOXC4, HIVEP1, HES4, HDAC5, GRM2, GRIN1, GRB7, GRASP, GLTSCR2, GIT2, GEMIN4, GADD45B, FRS3, FOSL2, FBXW7, EPB41, EGR4, EGR2, ECE1, DUSP6, DTX1, DPYSL2, DOK2, DIABLO, CUX1, CRABP2, CPLX1, CPEB1, CLEC11A, CDKN2D, CDC25C, CD63, CD3G, CAMKK2, ATF4, ARRB1, ARNTL, AQP5, AGTRAP, ACTN1, ACAA1, ABI3

Corresponding total number of TFBS:
(For each gene listed above, the total number of TFBS for any of the selected TFs, multiplied by the number of selected Gene ontology terms containing that gene)
34, 34, 32, 32, 32, 28, 28, 28, 28, 28, 26, 24, 20, 20, 20, 20, 17, 17, 17, 17, 17, 17, 17, 17, 17, 17, 17, 17,
17, 17, 17, 17, 17, 17, 17, 17, 17, 17, 17, 17, 17, 17, 17, 17, 17, 17, 17, 17, 17, 17, 17, 17, 17, 17, 17, 17,
17, 17, 17, 17, 17, 17, 17, 17, 17, 17, 17, 17, 17, 17, 17, 17, 17, 17, 17, 17, 17, 17, 17, 17, 17, 17, 17, 17,
17, 17, 17, 17, 17, 17, 17, 17, 17, 17, 17, 17, 17, 17, 17, 17, 17, 17, 17, 17, 17, 17, 17, 17, 17, 17, 17, 17,
16, 16, 16, 16, 16, 16, 16, 16, 16, 16, 16, 16, 16, 16, 16, 16, 16, 16, 16, 16, 16, 16, 16, 16, 15, 15, 15, 15,
15, 15, 15, 15, 15, 15, 15, 15, 15, 15, 15, 15, 15, 15, 15, 15, 15, 15, 15, 14, 14, 14, 14, 14, 14, 14, 14, 14,
14, 13, 13, 13, 13, 13, 13, 13, 13, 13, 13, 13, 12, 12, 12, 12, 12, 12, 12, 12, 12, 12, 12, 12, 12, 12, 12, 12,
11, 11, 11, 11, 11, 11, 11, 11, 11, 11, 10, 10, 10, 10, 10, 10, 10, 10, 10, 10, 10, 10, 10, 10, 10, 10, 9, 9, 9, 9,
9, 9, 9, 9, 9, 9, 9, 9, 9, 8, 8, 8, 8, 8, 8, 8, 8, 8, 8, 8, 7, 7, 7, 7, 7, 7, 7, 7, 7, 7, 7, 7, 7, 7, 7, 7, 7, 7, 7, 7, 7, 7,
7, 7, 7, 6, 6, 6, 6, 6, 6, 6, 6, 6, 6, 6, 6, 6, 6, 6, 6, 6, 6, 5, 5, 5, 5, 5, 5, 5, 5, 5, 5, 5, 5, 5, 5, 5, 5, 5, 5, 5, 5, 4,
4, 4, 4, 4, 4, 4, 4, 4, 4, 4, 4, 4, 4, 4, 4, 4, 4, 4, 4, 4, 4, 4, 4, 4, 4, 4, 4, 4, 4, 4, 4, 4, 4, 4, 3, 3, 3, 3, 3, 3, 3, 3,
3, 3, 3, 3, 3, 3, 3, 3, 3, 3, 3, 3, 3, 3, 3, 3, 3, 3, 3, 3, 3, 3, 3, 3, 3, 3, 3, 3, 2, 2, 2, 2, 2, 2, 2, 2, 2, 2, 2, 2, 2, 2,
2, 2, 2, 2, 2, 2, 2, 2, 2, 2, 2, 2, 2, 2, 2, 2, 2, 2, 2, 2, 2, 2, 2, 2, 2, 2, 2, 2, 2, 2, 2, 2, 2, 2, 2, 2, 2, 2, 2, 2, 2, 2,
2, 2, 1, 1, 1, 1, 1, 1, 1, 1, 1, 1, 1, 1, 1, 1, 1, 1, 1, 1, 1, 1, 1, 1, 1, 1, 1, 1, 1, 1, 1, 1, 1, 1, 1, 1, 1, 1, 1, 1, 1, 1,
1, 1, 1, 1, 1, 1, 1, 1, 1, 1, 1, 1, 1, 1, 1, 1, 1, 1, 1, 1, 1, 1, 1, 1, 1, 1, 1, 1, 1, 1, 1, 1, 1, 1, 1, 1, 1, 1, 1, 1, 1, 1,
1, 1, 1, 1, 1, 1, 1, 1, 1, 1, 1, 1, 1, 1, 1, 1, 1, 1, 1, 1, 1, 1, 1, 1, 1, 1, 1, 1, 1, 1, 1, 1, 1, 1, 1

Corresponding number of selected Gene ontology terms each gene is involved with:
(In the same order as above)
2, 2, 2, 2, 2, 2, 2, 2, 2, 2, 2, 2, 2, 2, 2, 2, 1, 1, 1, 1, 1, 1, 1, 1, 1, 1, 1, 1, 1, 1, 1, 1, 1, 1, 1, 1, 1, 1, 1, 1, 1, 1,
1, 1, 1, 1, 1, 1, 1, 1, 1, 1, 1, 1, 1, 1, 1, 1, 1, 1, 1, 1, 1, 1, 1, 1, 1, 1, 1, 1, 1, 1, 1, 1, 1, 1, 1, 1, 1, 1, 1, 1, 1, 1,
1, 1, 1, 1, 1, 1, 1, 1, 1, 1, 1, 1, 1, 1, 1, 1, 1, 1, 1, 1, 1, 1, 1, 1, 1, 1, 1, 1, 1, 1, 1, 1, 1, 1, 1, 1, 1, 1, 1, 1, 1, 1,
1, 1, 1, 1, 1, 1, 1, 1, 1, 1, 1, 1, 1, 1, 1, 1, 1, 1, 1, 1, 1, 1, 1, 1, 1, 1, 1, 1, 1, 1, 1, 1, 1, 1, 1, 1, 1, 1, 1, 2, 1, 1,
1, 1, 1, 1, 1, 1, 1, 1, 1, 1, 1, 1, 1, 1, 1, 1, 1, 1, 1, 1, 1, 1, 1, 1, 1, 1, 2, 1, 1, 1, 1, 1, 1, 1, 1, 1, 1, 1, 1, 1, 2, 1,
1, 1, 1, 1, 1, 1, 1, 1, 1, 1, 1, 1, 1, 1, 1, 1, 1, 1, 1, 1, 1, 1, 1, 1, 1, 1, 1, 1, 1, 1, 1, 1, 1, 1, 1, 1, 1, 1, 1, 1, 1, 1,
1, 1, 1, 1, 1, 1, 1, 1, 1, 1, 1, 1, 1, 1, 1, 1, 1, 1, 1, 1, 1, 1, 1, 1, 1, 1, 1, 1, 1, 1, 1, 2, 1, 1, 1, 1, 1, 1, 1, 1, 1, 1,
1, 1, 1, 1, 1, 1, 1, 1, 1, 1, 1, 1, 1, 1, 1, 1, 1, 1, 1, 1, 1, 1, 1, 1, 1, 1, 1, 1, 1, 1, 1, 1, 1, 1, 1, 1, 1, 1, 1, 1, 1, 1,
1, 1, 1, 1, 1, 1, 1, 1, 1, 1, 1, 1, 1, 1, 1, 1, 1, 1, 1, 1, 1, 1, 1, 1, 1, 1, 1, 1, 1, 1, 1, 1, 1, 1, 1, 1, 1, 1, 1, 1, 1, 1,
1, 1, 1, 1, 2, 1, 1, 1, 1, 1, 1, 1, 1, 1, 1, 1, 1, 1, 1, 1, 1, 1, 2, 1, 1, 1, 1, 1, 1, 1, 1, 1, 1, 1, 1, 1, 1, 1, 1, 1, 1, 2,
1, 1, 1, 1, 1, 1, 1, 1, 1, 1, 1, 1, 1, 1, 1, 1, 1, 1, 1, 1, 1, 1, 1, 1, 1, 1, 1, 1, 1, 1, 1, 1, 1, 1, 1, 1, 1, 1, 1, 1, 1, 1,
1, 1, 1, 1, 1, 1, 1, 1, 1, 1, 1, 1, 1, 1, 1, 1, 1, 1, 1, 1, 1, 1, 1, 1, 1, 1, 1, 1, 1, 1, 1, 1, 1, 1, 1, 1, 1, 1, 1, 1, 1, 1,
1, 1, 1, 1, 1, 1, 1, 1, 1, 1, 1, 1, 1, 1, 1, 1, 1, 1, 1, 1, 1, 1, 1, 1, 1, 1, 1, 1, 1, 1, 1, 1, 1, 1, 1, 1, 1, 1, 1, 1, 1, 1,
1, 1, 1, 1, 1, 1, 1, 1, 1

Corresponding number of selected TFs each gene is involved with:
(In the same order as above)
17, 17, 16, 16, 16, 14, 14, 14, 14, 14, 13, 12, 10, 10, 10, 10, 17, 17, 17, 17, 17, 17, 17, 17, 17, 17, 17, 17,
17, 17, 17, 17, 17, 17, 17, 17, 17, 17, 17, 17, 17, 17, 17, 17, 17, 17, 17, 17, 17, 17, 17, 17, 17, 17, 17, 17,
17, 17, 17, 17, 17, 17, 17, 17, 17, 17, 17, 17, 17, 17, 17, 17, 17, 17, 17, 17, 17, 17, 17, 17, 17, 17, 17, 17,

17, 17, 17, 17, 17, 17, 17, 17, 17, 17, 17, 17, 17, 17, 17, 17, 17, 17, 17, 17, 17, 17, 17, 17, 17, 17, 17, 17, 16, 16, 16, 16, 16, 16, 16, 16, 16, 16, 16, 16, 16, 16, 16, 16, 16, 16, 16, 16, 16, 16, 16, 16, 15, 15, 15, 15, 15, 15, 15, 15, 15, 15, 15, 15, 15, 15, 15, 15, 15, 15, 15, 15, 15, 15, 15, 14, 14, 14, 14, 14, 14, 7, 14, 14, 14, 13, 13, 13, 13, 13, 13, 13, 13, 13, 13, 13, 12, 12, 12, 12, 12, 12, 12, 12, 12, 12, 12, 12, 12, 12, 6, 12, 11, 11, 11, 11, 11, 11, 11, 11, 11, 11, 10, 10, 5, 10, 10, 10, 10, 10, 10, 10, 10, 10, 10, 10, 10, 10, 9, 9, 9, 9, 9, 9, 9, 9, 9, 9, 9, 9, 9, 8, 8, 8, 8, 8, 8, 8, 8, 8, 8, 8, 7, 7, 7, 7, 7, 7, 7, 7, 7, 7, 7, 7, 7, 7, 7, 7, 7, 7, 7, 7, 7, 7, 7, 7, 7, 6, 6, 6, 6, 6, 6, 6, 6, 6, 6, 6, 6, 3, 6, 6, 6, 6, 6, 5, 5, 5, 5, 5, 5, 5, 5, 5, 5, 5, 5, 5, 5, 5, 5, 5, 5, 5, 5, 4, 4, 4, 4, 4, 4, 4, 4, 4, 4, 4, 4, 4, 4, 4, 4, 4, 4, 4, 4, 4, 4, 4, 4, 4, 4, 4, 4, 4, 4, 4, 4, 4, 4, 4, 3, 3, 3, 3, 3, 3, 3, 3, 3, 3, 3, 3, 3, 3, 3, 3, 3, 3, 3, 3, 3, 3, 3, 3, 3, 3, 3, 3, 3, 3, 3, 3, 3, 3, 3, 3, 2, 2, 1, 2, 2, 2, 2, 2, 2, 2, 2, 2, 2, 2, 2, 2, 2, 2, 2, 2, 2, 1, 2, 2, 2, 2, 2, 2, 2, 2, 2, 2, 2, 2, 2, 2, 2, 2, 2, 2, 1, 2, 2, 2, 2, 2, 2, 2, 2, 2, 2, 2, 2, 2, 2, 2, 2, 2, 2, 1

Gene ontology terms ranked according to mean of effect sizes in cluster:
Notch signaling pathway | signal transduction

Corresponding mean of effect sizes of each Gene ontology term:
(In the same order as above)
2.49, 1.92

Corresponding total number of genes of each Gene ontology term:
(In the same order as above)
103, 3.9K

Corresponding number of genes of each Gene ontology term with at least one TFBS (of selected TFs):
(In the same order as above)
28, 550

Corresponding total number of TFBS (of selected TFs) for each Gene ontology term (in all genes):
(In the same order as above)
284, 4325

Corresponding number of selected TFs each Gene ontology term is involved with:
(In the same order as above)
17, 17

TFs ranked according to mean of effect sizes in cluster:
V$AP4_Q6_01, V$LRF_Q2, V$LBP1_Q6, V$AP4_Q5, V$USF2_Q6, V$VDR_Q6, V$SMAD_Q6, V$AP4_Q6, V$AP2REP_01, V$PAX8_01, V$HNF4_Q6_03, V$LFA1_Q6, V$ZIC2_01, V$PAX8_B, V$T3R_Q6, V$TTF1_Q6, V$HNF4_Q6_02

Corresponding mean of effect sizes of each TF:
(In the same order as above)
3.01, 3.00, 2.86, 2.75, 2.71, 2.63, 2.33, 2.20, 2.17, 2.09, 1.98, 1.94, 1.74, 1.63, 1.60, 1.51, 1.36

Corresponding total number of TFBS for each TF (genome-wide):
(In the same order as above)
699, 719, 691, 702, 703, 692, 729, 692, 700, 728, 701, 711, 716, 712, 698, 714, 699

Corresponding total number of TFBS for each TF (in all genes in selected Gene ontology terms):
(In the same order as above)
278, 289, 275, 266, 279, 261, 278, 258, 280, 285, 267, 263, 275, 268, 265, 254, 268

Corresponding number of genes (of selected Gene ontology terms) each TF is involved with:

(In the same order as above)
262, 273, 258, 251, 258, 245, 265, 245, 265, 268, 255, 253, 261, 254, 254, 243, 258

Corresponding number of selected Gene ontology terms each TF is involved with:
(In the same order as above)
2, 2, 2, 2, 2, 2, 2, 2, 2, 2, 2, 2, 2, 2, 2, 2, 2

Cluster for columns 5 to 11, rows 193 to 198

Gene ontology terms

T cell differentiation | cell development | cell activation | T cell activation | T cell receptor complex | immunological synapse | integrin activation

TFs

V$AR_Q6 | V$PR_Q2 | V$GR_Q6_01 | V$AML1_Q6 | V$AML1_01 | V$OSF2_Q6

Information

All related TFs:
(List of all TFs that are related to any of the PWMs)
AML1, AML1a, AML3, AML3-isoform2, AR, GR, GR-alpha, GR-beta, PR, PR-alpha, PR-beta, RUNX2-isoform2

Ranked gene list:
(All genes of the selected Gene ontology terms with hits of any of the selected TFs, ranked according to the total number of TFBS)
ITGB2, CCR7, CSK, CD4, CXCR4, PTPN6, PLCG1, AKT1, NFATC1, FASN, TNF, TCF7, LTB, ETS1, CISH, BCL11B, LCK, TNFRSF4, TNFRSF18, SH2B3, PTK2B, GFI1, FYB, ZAP70, SOCS1, PER1, LTA, LEF1, FYN, CDKN1B, SKAP1, IL17C, SEMA4D, LYL1, LAG3, GATA3, CD7, CD3G, CD27, BCL2, ZBTB7B, VEGFA, TLR9, TCIRG1, STAT1, SHC1, RUNX1, RASSF5, PTPN7, POU2F2, LCP1, JUNB, IRF1, IL7R, IL2RA, DUSP2, BCL6, STAT5B, STAT5A, NR4A1, NFATC3, ITGA5, CD82, CD6, CD44, STAT6, IKZF1, CD3D, TSPAN4, SSSCA1, SATB1, LY6E, GIMAP5, EDC4, CSF2, LCP2, TNFRSF1A, TAL1, STK16, SH3BP2, RUNX3, PSTPIP1, PIK3R1, PAX6, MYO1G, MEF2D, LFNG, KCNN4, ITGB7, IL21R, HRAS, EGR1, DLL1, CDK5R1, CD63, CCND2, BCR, BCL2L11, ARHGEF2, SLC2A4RG, PPP1R9B, HMHA1, HES5, GUK1, FOXP3, DEF6, CD5, AMH, TRAF1, TBC1D10C, STRA13, STIM1, SIPA1, SELPLG, PXN, PRDM1, PDCD1, MYD88, LBX1, LAT, ITGA6, IL16, HNRNPL, FLI1, DGKA, CDH23, CD247, CD226, ZFPM1, VAV3, UNC13D, UBC, TNFSF14, TNFRSF6B, TAPBP, PKN1, PDE4A, NOTCH1, MAL, ISYNA1, INPP5D, ILK, IL23A, IFITM3, GPR132, GORASP2, GLI1, CXCR5, C21orf33, ZEB1, XAB2, TNFRSF1B, TNFRSF13C, TAP1, SOCS3, SILV, RASGRP2, NOTCH4, NOL3, NKX2-2, NGFR, NEDD9, MAT2A, ID3, ICAM3, FOS, FGR, EGR3, EBF1, CSF1, CFLAR, CD79B, CD68, CD47, CD300A, CCND3, BHLHB2, AIRE, XPC, SSFA2, PROX1, POU4F1, POU3F2, POLD4, PAX5, NR4A2, MUC1, ILF3, ID2, GATA2, FLII, FERMT3, DOK2, DNMT3A, DIABLO, DECR1, DCXR, DCTN2, DAPP1, CDH15, CD74, CBLB, CAP1, CALR, BATF

Corresponding total number of TFBS:
(For each gene listed above, the total number of TFBS for any of the selected TFs, multiplied by the number of selected Gene ontology terms containing that gene)
42, 42, 36, 36, 35, 30, 30, 30, 25, 25, 24, 24, 24, 24, 24, 24, 21, 20, 20, 20, 20, 20, 20, 18, 18, 18, 18, 18, 18, 18, 16, 16, 15, 15, 15, 15, 15, 15, 15, 15, 12, 12, 12, 12, 12, 12, 12, 12, 12, 12, 12, 12, 12, 12, 12, 12, 10, 10, 10, 10, 10, 10, 10, 10, 9, 9, 9, 8, 8, 8, 8, 8, 8, 8, 7, 6, 6, 6, 6, 6, 6, 6, 6, 6, 6, 6, 6, 6, 6, 6, 6, 6,

6, 6, 6, 6, 6, 5, 5, 5, 5, 5, 5, 5, 5, 5, 4, 4, 4, 4, 4, 4, 4, 4, 4, 4, 4, 4, 4, 4, 4, 4, 4, 4, 4, 3, 3, 3, 3, 3, 3, 3, 3, 3, 3, 3, 3, 3, 3, 3, 3, 3, 3, 3, 3, 3, 2, 2, 2, 2, 2, 2, 2, 2, 2, 2, 2, 2, 2, 2, 2, 2, 2, 2, 2, 2, 2, 2, 2, 2, 2, 2, 2, 1, 1, 1, 1, 1, 1, 1, 1, 1, 1, 1, 1, 1, 1, 1, 1, 1, 1, 1, 1, 1, 1, 1, 1, 1, 1

Corresponding number of selected Gene ontology terms each gene is involved with:
(In the same order as above)
7, 7, 6, 6, 7, 5, 5, 6, 5, 5, 4, 4, 4, 4, 4, 4, 7, 4, 4, 4, 5, 4, 5, 6, 3, 3, 3, 3, 6, 3, 4, 4, 3, 3, 3, 3, 5, 5, 5, 5, 2, 2, 2, 2, 4, 4, 3, 2, 2, 2, 3, 2, 2, 4, 6, 2, 2, 5, 5, 2, 2, 5, 2, 5, 5, 3, 3, 3, 2, 2, 4, 2, 2, 4, 4, 7, 2, 3, 1, 1, 2, 2, 3, 1, 1, 1, 1, 2, 1, 2, 2, 3, 1, 3, 2, 2, 3, 3, 1, 1, 1, 1, 1, 1, 5, 1, 5, 1, 1, 1, 1, 2, 1, 1, 1, 2, 1, 2, 2, 2, 4, 2, 1, 1, 1, 1, 4, 2, 1, 3, 1, 3, 1, 1, 1, 1, 1, 3, 1, 1, 1, 1, 3, 1, 1, 3, 1, 3, 1, 1, 1, 2, 2, 1, 1, 1, 1, 2, 2, 1, 2, 1, 1, 2, 2, 2, 1, 2, 2, 2, 2, 2, 2, 2, 1, 2, 1, 2, 1, 1, 1, 1, 1, 1, 1, 1, 1, 1, 1, 1, 1, 1, 1, 1, 1, 1, 1, 1, 1, 1, 1, 1, 1

Corresponding number of selected TFs each gene is involved with:
(In the same order as above)
6, 6, 6, 6, 5, 6, 6, 5, 5, 5, 6, 6, 6, 6, 6, 6, 3, 5, 5, 5, 4, 5, 4, 3, 6, 6, 6, 6, 3, 6, 4, 4, 5, 5, 5, 5, 3, 3, 3, 3, 6, 6, 6, 6, 3, 3, 4, 6, 6, 6, 4, 6, 6, 3, 2, 6, 6, 2, 2, 5, 5, 2, 5, 2, 2, 3, 3, 3, 4, 4, 2, 4, 4, 2, 2, 1, 3, 2, 6, 6, 3, 3, 2, 6, 6, 6, 6, 3, 6, 3, 3, 2, 6, 2, 3, 3, 2, 2, 6, 5, 5, 5, 5, 5, 1, 5, 1, 5, 4, 4, 4, 2, 4, 4, 4, 2, 4, 2, 2, 2, 1, 2, 4, 4, 4, 4, 1, 2, 3, 1, 3, 1, 3, 3, 3, 3, 3, 1, 3, 3, 3, 3, 1, 3, 3, 1, 3, 1, 3, 2, 2, 1, 1, 2, 2, 2, 2, 1, 1, 2, 1, 2, 2, 1, 1, 1, 2, 1, 1, 1, 1, 1, 1, 1, 2, 1, 2, 1, 1, 1, 1, 1, 1, 1, 1, 1, 1, 1, 1, 1, 1, 1, 1, 1, 1, 1, 1, 1, 1, 1, 1, 1, 1

Gene ontology terms ranked according to mean of effect sizes in cluster:
T cell receptor complex | cell activation | immunological synapse | T cell activation | integrin activation | cell development | T cell differentiation

Corresponding mean of effect sizes of each Gene ontology term:
(In the same order as above)
2.59, 2.35, 2.34, 2.33, 2.19, 2.18, 2.10

Corresponding total number of genes of each Gene ontology term:
(In the same order as above)
478, 596, 112, 287, 155, 507, 172

Corresponding number of genes of each Gene ontology term with at least one TFBS (of selected TFs):
(In the same order as above)
97, 118, 30, 61, 30, 111, 48

Corresponding total number of TFBS (of selected TFs) for each Gene ontology term (in all genes):
(In the same order as above)
338, 374, 110, 223, 117, 359, 158

Corresponding number of selected TFs each Gene ontology term is involved with:
(In the same order as above)
6, 6, 6, 6, 6, 6, 6

TFs ranked according to mean of effect sizes in cluster:
VGR_Q6_01, VAR_Q6, VPR_Q2, VAML1_Q6, V$AML1_01, V$OSF2_Q6

Corresponding mean of effect sizes of each TF:
(In the same order as above)
2.97, 2.64, 2.41, 2.06, 1.93, 1.79

Corresponding total number of TFBS for each TF (genome-wide):
(In the same order as above)
720, 699, 698, 718, 710, 710

Corresponding total number of TFBS for each TF (in all genes in selected Gene ontology terms):
(In the same order as above)
304, 287, 288, 279, 273, 248

Corresponding number of genes (of selected Gene ontology terms) each TF is involved with:
(In the same order as above)
115, 107, 110, 114, 107, 94

Corresponding number of selected Gene ontology terms each TF is involved with:
(In the same order as above)
7, 7, 7, 7, 7, 7

Cluster for columns 801 to 805, rows 373 to 383

Gene ontology terms

insulin-like growth factor binding protein complex | insulin-like growth factor receptor binding | insulin-like growth factor binding | insulin-like growth factor II binding | insulin-like growth factor I binding

TFs

V$P53_01 | V$CHX10_01 | V$POU3F2_01 | V$MEF2_04 | V$SRF_Q6 | V$SRF_C | V$SRF_Q5_01 | V$SRF_Q4 | V$SRF_Q5_02 | V$SRF_01 | V$TATA_01

Information

All related TFs:
(List of all TFs that are related to any of the PWMs)
Chx10, MEF-2A, POU3F2, SRF, SRF-I, SRF-L, SRF-M, SRF-S, TBP, aMEF-2, p53, p53-isoform-1

Ranked gene list:
(All genes of the selected Gene ontology terms with hits of any of the selected TFs, ranked according to the total number of TFBS)
LBX1, FHL2, CYR61, ACTB, PTP4A1, HOXC6, CTGF, GHRL, BLVRB, IGF1R, FN1, DCN, AKT1, PDLIM5, THBS1, RARG, MMP2, TNF, SBDS, LEP, IGFBP3, IGF1, CLU, VTN, PSMP, PRH1, NDC80, FOXO3, VEGFA, STAT5B, SCN5A, S100P, PTRH1, PRL, PAEP, LHCGR, LATS1, IRS1, IGFBP7, IGFBP5, IGFBP2, IGF2, GH1, FOXO1, ESR1, CDKN1A, CCND1, BAX, TTR, SPP1, SERPINE1, RETN, PRH2, MMP3, HOXA10, GCG, ERBB2, CSH2, BEST1, STMN1, SCN2A, PIK3R1, IRF1, DUOXA1, CEACAM1, BHMT, BCL2, ANXA6, SSSCA1, KIAA1804, HRAS, HOXA11, HIF1A, HBA1, GRB2, GRB10, GAB1, FCGR1A, DMD, CYP11A1, CADM1, SMAD2, SHC1, SDCBP, RASSF1, PTPRF, PTEN, PSMD9, PIK3R2, PDGFRB, MYH6, LEPROT, ITGB5, IL17F, GRB7, GADD45A, EPHB2, CDH13, CDH1, CDC20, CD44, CASP3, CASP10, ATP8A2

Corresponding total number of TFBS:
(For each gene listed above, the total number of TFBS for any of the selected TFs, multiplied by the number of selected Gene ontology terms containing that gene)
35, 35, 28, 24, 20, 20, 20, 16, 16, 15, 15, 15, 15, 14, 12, 12, 12, 10, 10, 10, 10, 10, 10, 8, 8, 8, 8, 8, 5, 5, 5, 5, 5, 5, 5, 5, 5, 5, 5, 5, 5, 5, 5, 5, 5, 5, 5, 4, 4, 4, 4, 4, 4, 4, 4, 4, 4, 4, 3, 3, 3, 3, 3, 3, 3, 3, 3, 2, 2, 2, 2, 2, 2, 2, 2, 2, 2, 2, 2, 2, 1, 1, 1, 1, 1, 1, 1, 1, 1, 1, 1, 1, 1, 1, 1, 1, 1, 1, 1, 1, 1, 1

Corresponding number of selected Gene ontology terms each gene is involved with:
(In the same order as above)
5, 5, 4, 3, 5, 5, 5, 4, 4, 5, 5, 5, 5, 2, 3, 4, 4, 5, 5, 5, 5, 5, 5, 2, 4, 4, 4, 4, 5, 1, 5, 5, 5, 5, 5, 5, 5, 5, 5, 5, 5, 5,

5, 5, 5, 5, 5, 5, 4, 4, 4, 4, 4, 4, 4, 4, 4, 4, 4, 3, 1, 1, 3, 3, 1, 3, 3, 1, 1, 2, 1, 1, 2, 2, 1, 1, 1, 2, 1, 2, 1, 1, 1, 1, 1, 1, 1, 1, 1, 1, 1, 1, 1, 1, 1, 1, 1, 1, 1, 1, 1, 1, 1

Corresponding number of selected TFs each gene is involved with:
(In the same order as above)
7, 7, 7, 8, 4, 4, 4, 4, 4, 3, 3, 3, 3, 7, 4, 3, 3, 2, 2, 2, 2, 2, 2, 4, 2, 2, 2, 2, 1, 5, 1, 1, 1, 1, 1, 1, 1, 1, 1, 1, 1, 1, 1, 1, 1, 1, 1, 1, 1, 1, 1, 1, 1, 1, 1, 1, 1, 1, 1, 3, 3, 1, 1, 3, 1, 1, 3, 2, 1, 2, 2, 1, 1, 2, 2, 2, 1, 2, 1, 2, 1, 1, 1, 1, 1, 1, 1, 1, 1, 1, 1, 1, 1, 1, 1, 1, 1, 1, 1, 1, 1, 1

Gene ontology terms ranked according to mean of effect sizes in cluster:
insulin-like growth factor binding | insulin-like growth factor II binding | insulin-like growth factor binding protein complex | insulin-like growth factor I binding | insulin-like growth factor receptor binding

Corresponding mean of effect sizes of each Gene ontology term:
(In the same order as above)
1.52, 1.18, 1.14, 1.09, 0.75

Corresponding total number of genes of each Gene ontology term:
(In the same order as above)
149, 132, 88, 132, 173

Corresponding number of genes of each Gene ontology term with at least one TFBS (of selected TFs):
(In the same order as above)
69, 64, 46, 64, 78

Corresponding total number of TFBS (of selected TFs) for each Gene ontology term (in all genes):
(In the same order as above)
148, 132, 93, 122, 149

Corresponding number of selected TFs each Gene ontology term is involved with:
(In the same order as above)
11, 11, 11, 11, 11

TFs ranked according to mean of effect sizes in cluster:
VSRF_Q5_02, VTATA_01, V$MEF2_04, V$SRF_C, VSRF_Q4, VPOU3F2_01, V$P53_01, V$SRF_Q6, VSRF_Q5_01, VSRF_01, V$CHX10_01

Corresponding mean of effect sizes of each TF:
(In the same order as above)
2.63, 2.09, 2.04, 1.58, 1.02, 1.00, 0.88, 0.66, 0.45, 0.36, -0.19

Corresponding total number of TFBS for each TF (genome-wide):
(In the same order as above)
683, 736, 621, 687, 664, 630, 615, 703, 693, 659, 669

Corresponding total number of TFBS for each TF (in all genes in selected Gene ontology terms):
(In the same order as above)
81, 86, 69, 68, 58, 51, 48, 50, 51, 48, 34

Corresponding number of genes (of selected Gene ontology terms) each TF is involved with:
(In the same order as above)
23, 22, 21, 19, 18, 20, 16, 16, 18, 14, 11

Corresponding number of selected Gene ontology terms each TF is involved with:
(In the same order as above)

5, 5, 5, 5, 5, 5, 5, 5, 5, 5, 5

Cluster for columns 624 to 627, rows 9 to 22

Gene ontology terms

microtubule organizing center | centrosome | microtubule nucleation | microtubule binding

TFs

V$CREB_Q4_01 | V$CREB_Q2_01 | V$CREB_Q4 | V$CREBP1_Q2 | V$CREB_Q2 | V$CREB_01 | V$CREBP1CJUN_01 | V$CREBATF_Q6 | V$ATF_B | V$CREB_02 | V$ATF_01 | V$ATF3_Q6 | V$ATF1_Q6 | V$CREBP1_01

Information

All related TFs:
(List of all TFs that are related to any of the PWMs)
120-kDa, 47-kDa, ATF, ATF-1, ATF-2, ATF-4, ATF-a, ATF-like, ATF/CREB, ATF2, ATF2-isoform2, ATF3, ATF4, ATF5, ATF6, ATFa-isoform1, CRE-BP1, CRE-BP2, CREB, CREBbeta, CREMalpha, CREMbeta, CREMgamma, CREMtau, CREMtau1, CREMtau2, CREMtaualpha, c-Jun, deltaCREB

Ranked gene list:
(All genes of the selected Gene ontology terms with hits of any of the selected TFs, ranked according to the total number of TFBS)
MAPRE2, TUBGCP2, PARD6A, UBC, MXI1, NEDD1, CENPE, CDC42, NDC80, PAK1, PAFAH1B1, NEK1, GABARAPL1, KIF1A, IKBKAP, ID1, CDC14B, CALM2, UBQLN1, SSSCA1, CEP57, CCDC41, TMEM201, RAD51C, PRC1, TUBGCP6, NF1, IFT20, CNTROB, TACC3, RASSF1, XRCC2, SQSTM1, PARK2, MOAP1, MAP1B, CCNA2, CALM1, AURKA, PLK4, NDEL1, BCAS2, RAB6A, NXF1, EPB41, BRCA1, TRIP10, RAD51L1, MCM2, CDKN1A, CDK5RAP2, CDK2, UBE2E2, TUBGCP5, SKP1, SGOL1, SGMS1, NUMA1, NEK9, MAPT, HSP90AA1, FGFR1, FBL, AURKB, ZBTB16, TP53, SSNA1, SLC25A23, RARA, PTTG1, PIK3CG, NPM1, MAP2K1, GSK3B, GSK3A, EDC4, E2F3, CROCC, CETN3, ATF4, APC, ANAPC2

Corresponding total number of TFBS:
(For each gene listed above, the total number of TFBS for any of the selected TFs, multiplied by the number of selected Gene ontology terms containing that gene)
48, 36, 26, 24, 24, 22, 22, 20, 16, 14, 14, 14, 14, 12, 12, 12, 12, 12, 11, 10, 10, 10, 9, 9, 9, 8, 8, 8, 8, 7, 7, 6, 6, 6, 6, 6, 6, 6, 6, 5, 5, 5, 4, 4, 4, 4, 3, 3, 3, 3, 3, 3, 2, 2, 2, 2, 2, 2, 2, 2, 2, 2, 2, 2, 1, 1, 1, 1, 1, 1, 1, 1, 1, 1, 1, 1, 1, 1, 1, 1, 1

Corresponding number of selected Gene ontology terms each gene is involved with:
(In the same order as above)
4, 3, 2, 2, 3, 2, 2, 2, 2, 1, 1, 1, 1, 1, 3, 1, 1, 3, 1, 1, 1, 1, 3, 1, 1, 1, 1, 1, 1, 1, 1, 1, 2, 1, 2, 3, 1, 3, 3, 1, 1, 1, 1, 1, 1, 2, 1, 1, 1, 1, 1, 1, 1, 2, 1, 1, 1, 2, 1, 2, 1, 1, 1, 1, 1, 1, 1, 1, 1, 1, 1, 1, 1, 1, 1, 1, 1, 1, 1, 1, 1, 1

Corresponding number of selected TFs each gene is involved with:
(In the same order as above)
12, 12, 13, 12, 8, 11, 11, 10, 8, 14, 14, 14, 14, 12, 4, 12, 12, 4, 11, 10, 10, 10, 3, 9, 9, 8, 8, 8, 8, 7, 7, 6, 3, 6, 3, 2, 6, 2, 2, 5, 5, 5, 4, 4, 4, 2, 3, 3, 3, 3, 3, 3, 2, 1, 2, 2, 2, 1, 2, 1, 2, 2, 2, 2, 1, 1, 1, 1, 1, 1, 1, 1, 1, 1, 1, 1, 1, 1, 1, 1, 1, 1

Gene ontology terms ranked according to mean of effect sizes in cluster:

centrosome | microtubule organizing center | microtubule nucleation | microtubule binding

Corresponding mean of effect sizes of each Gene ontology term:
(In the same order as above)
2.83, 1.78, 1.63, 1.59

Corresponding total number of genes of each Gene ontology term:
(In the same order as above)
243, 46, 42, 69

Corresponding number of genes of each Gene ontology term with at least one TFBS (of selected TFs):
(In the same order as above)
68, 11, 12, 22

Corresponding total number of TFBS (of selected TFs) for each Gene ontology term (in all genes):
(In the same order as above)
349, 84, 66, 108

Corresponding number of selected TFs each Gene ontology term is involved with:
(In the same order as above)
14, 14, 14, 14

TFs ranked according to mean of effect sizes in cluster:
V$CREB_01, V$CREBP1_01, V$CREBATF_Q6, V$CREBP1CJUN_01, V$ATF3_Q6, V$ATF1_Q6, V$CREB_Q4, V$CREBP1_Q2, VATF_B, VCREB_Q4_01, VATF_01, VCREB_Q2, V$CREB_Q2_01, V$CREB_02

Corresponding mean of effect sizes of each TF:
(In the same order as above)
3.04, 2.78, 2.60, 2.51, 2.37, 2.10, 1.93, 1.86, 1.75, 1.65, 1.45, 1.42, 1.23, 0.75

Corresponding total number of TFBS for each TF (genome-wide):
(In the same order as above)
659, 662, 671, 652, 663, 671, 671, 679, 665, 667, 660, 676, 679, 656

Corresponding total number of TFBS for each TF (in all genes in selected Gene ontology terms):
(In the same order as above)
53, 54, 49, 47, 48, 45, 41, 40, 42, 39, 39, 39, 35, 36

Corresponding number of genes (of selected Gene ontology terms) each TF is involved with:
(In the same order as above)
36, 38, 34, 31, 31, 31, 29, 28, 29, 28, 28, 27, 25, 26

Corresponding number of selected Gene ontology terms each TF is involved with:
(In the same order as above)
4, 4, 4, 4, 4, 4, 4, 4, 4, 4, 4, 4, 4, 4
